# Integrated analyses of miRNA-mRNA expression profiles of ovaries reveal the crucial interaction networks that regulate the prolificacy of goats in the follicular phase

**DOI:** 10.1186/s12864-021-08156-2

**Published:** 2021-11-11

**Authors:** Yufang Liu, Zuyang Zhou, Xiaoyun He, Lin Tao, Yanting Jiang, Rong Lan, Qionghua Hong, Mingxing Chu

**Affiliations:** 1 Key Laboratory of Animal Genetics, Breeding and Reproduction of Ministry of Agriculture and Rural Affairs, Institute of Animal Science, Chinese Academy of Agricultural Sciences, No. 2 Yuanmingyuan West Rd, Beijing, 100193 China; 2 College of Life Sciences and Food Engineering, Hebei University of Engineering, Handan, 056021 China; 3 Yunnan Animal Science and Veterinary Institute, Kunming, 650224 China

**Keywords:** Prolific goats, Follicular phase, Ovarian tissues, miRNA-mRNA pairs

## Abstract

**Background:**

Litter size is an important index of mammalian prolificacy and is determined by the ovulation rate. The ovary is a crucial organ for mammalian reproduction and is associated with follicular development, maturation and ovulation. However, prolificacy is influenced by multiple factors, and its molecular regulation in the follicular phase remains unclear.

**Methods:**

Ten female goats with no significant differences in age and weight were randomly selected and divided into either the high-yielding group (*n* = 5, HF) or the low-yielding group (*n* = 5, LF). Ovarian tissues were collected from goats in the follicular phase and used to construct mRNA and miRNA sequencing libraries to analyze transcriptomic variation between high- and low-yield Yunshang black goats. Furthermore, integrated analysis of the differentially expressed (DE) miRNA-mRNA pairs was performed based on their correlation. The STRING database was used to construct a PPI network of the DEGs. RT–qPCR was used to validate the results of the predicted miRNA-mRNA pairs. Luciferase analysis and CCK-8 assay were used to detect the function of the miRNA-mRNA pairs and the proliferation of goat granulosa cells (GCs).

**Results:**

A total of 43,779 known transcripts, 23,067 novel transcripts, 424 known miRNAs and 656 novel miRNAs were identified by RNA-seq in the ovaries from both groups. Through correlation analysis of the miRNA and mRNA expression profiles, 263 negatively correlated miRNA-mRNA pairs were identified in the LF vs. HF comparison. Annotation analysis of the DE miRNA-mRNA pairs identified targets related to biological processes such as “estrogen receptor binding (GO:0030331)”, “oogenesis (GO:0048477)”, “ovulation cycle process (GO:0022602)” and “ovarian follicle development (GO:0001541)”. Subsequently, five KEGG pathways (oocyte meiosis, progesterone-mediated oocyte maturation, GnRH signaling pathway, Notch signaling pathway and TGF-β signaling pathway) were identified in the interaction network related to follicular development, and a PPI network was also constructed. In the network, we found that *CDK12, FAM91A1, PGS1, SERTM1, SPAG5, SYNE1, TMEM14A, WNT4,* and *CAMK2G* were the key nodes, all of which were targets of the DE miRNAs. The PPI analysis showed that there was a clear interaction among the *CAMK2G, SERTM1, TMEM14A, CDK12, SYNE1* and *WNT4* genes. In addition, dual luciferase reporter and CCK-8 assays confirmed that miR-1271-3p suppressed the proliferation of GCs by inhibiting the expression of *TXLNA*.

**Conclusions:**

These results increase the understanding of the molecular mechanisms underlying goat prolificacy. These results also provide a basis for studying interactions between genes and miRNAs, as well as the functions of the pathways in ovarian tissues involved in goat prolificacy in the follicular phase.

**Supplementary Information:**

The online version contains supplementary material available at 10.1186/s12864-021-08156-2.

## Background

In mammals, litter size is a crucial economic trait because it has a notable impact on profitability in the breeding industry. Ovulation rate and embryo survival are directly linked to mammalian litter size [1]. Most goat breeds produce litter sizes of two kids, which limits production in the goat industry, including the production of meat, milk and fur. Ovaries directly mediate ovulation, which has a significant impact on the fecundity of mammals. Traditional breeding methods require too much time to improve litter sizes and have shown low heritability. Many molecular technologies have been used to analyze the mechanisms underlying reproduction in goats. Molecular mechanisms occurring in the ovarian tissues in the follicular phase may contribute to observing differences in litter size [2]. Many genes, transcription factors, ncRNAs and signaling pathways regulate the complex and precise process of reproduction [3]. These factors have been used to construct a network of many interactions in vivo that regulate the ovulation rate of goats.

Interactions between many genes are involved in ovulation rate and litter size. Several studies have shown that *bone morphogenetic protein receptor type 1B (BMPR1B)*, also known as *FecB*, plays a major role in sheep fertility. Q249R mutations within *FecB* are highly associated with ovulation rates in many sheep breeds around the world [4]. Another crucial gene, *growth differentiation Factor 9 (GDF9),* also plays a critical role in early folliculogenesis [5]. In addition, growth hormones and members of the insulin-like growth factor (IGF) system (*IGF-I* and *IGF-II*) may regulate follicular development and atresia [6, 7], and *FER1L4* and *SRD5A2* were found to be associated with high fecundity in goats [8]. Second, many interactions between miRNAs and mRNAs influence biological processes in the goat ovary. miRNAs are endogenous noncoding RNAs that regulate gene expression at the posttranscriptional level by binding to the 3′-UTR of target mRNAs. Multiple miRNAs act on folliculogenesis by regulating transcription factors and genes associated with it. For example, miR-26b promoted apoptosis in porcine granulosa cells (GCs) during follicular atresia by targeting the ataxia-temporal (ATM) gene and increasing the number of DNA breaks [9]. These interactions play important regulatory roles in the regulation of biological processes involved in ovulation rates and fertility. For example, miR-133b represses the expression of its target genes *forkhead Box L2* (*FOXL2*) and *transgelin 2* (*TAGLN2*) in GCs, thereby regulating oocyte growth and maturation [10].

However, the regulation of ovulation rates in goats is a highly complex process involving the interaction of multiple miRNAs and mRNAs within intricate signaling pathways rather than a single network of interactions. miR-141 affects the MAPK signaling pathway by regulating *DAPK1* expression, which leads to the development of polycystic ovary syndrome (PCOS) [11]. Moreover, both miR-206 and miR-1 expression were found to be downregulated in the large follicles of a variety of goats, which might be associated with oocyte maturation and ovulation [12]. In addition, *mitogen-activated protein kinase kinase kinase 3 (MAP 3 K3)* interacts with extracellular signal-regulated kinase (ERK) in the p38 MAPK pathway to regulate litter size traits in Berkshire pigs [13]. miR-122 has been shown to increase *LHR* mRNA levels by modulating *LRBP* expression through *SREBP* activation, a phenomenon that is essential for regulating key reproductive processes, such as ovulation and CL function [14]. These studies suggest that reproductive traits are regulated by multiple interaction networks of genes, miRNAs and signaling pathways. It is therefore important to identify the network of interactions between genes, miRNAs and signaling pathways in the regulation of ovulation rate and prolificacy. There is currently a lack of understanding of these networks in goats.

Goats are important agricultural animals, and as with other animals, genetic factors are the main players in ovulation and litter size. For example, goats have a kidding rate heritability of 0.11–0.14, and the ovulation number of the estrus cycle is 2–6 [15]. Therefore, elucidating the mechanisms by which genes, miRNAs and pathways interact in goat reproduction, particularly in relation to the ovarian ovulation rate, is of great significance for improving genetics and productive performance in goats. Several reports have described gene regulation, miRNA identification and pathway regulation in goat prolificacy. For instance, for litter size, the expression of miRNAs associated with ovulation, such as miR-122, miR-99a, miR-141, miR-493 and miR-200a, and the expression of mRNAs, such as *CD36, TNFAIP6, CYP11A1, SERPINA5* and *PTGFR,* have been identified and comparatively analyzed in the ovaries of prolific and nonprolific goats [16–19]. Moreover, *CYP11A1* plays a key role in the regulation of steroid-producing pathways in granulosa cells [20]. However, these studies have been limited to the role of genes, miRNAs or pathways alone in goat prolificacy, and there is a lack of understanding of the interactions of these factors in goat high yield traits. Furthermore, early studies have focused on goat estrus and entire reproductive cycle periods, but an understanding of the changes in the transcriptomes in goat ovaries in the follicular phase remains lacking.

The Yunshang black goat is an excellent breed due to its fecundity and meat production, and it is native to Yunnan Province, China. This breed has many favorable characteristics, especially its tender, tasty meat and excellent fecundity. Yunshang black goats are suitable for cultivation and reproduction. Many studies have been conducted on the molecular mechanisms related to resource conservation, strain selection, industrial production and trait development of this local breed. Therefore, Yunshang black goats might serve as a representative model to study mammalian prolificacy regulation. To reveal the crucial molecular mechanisms and molecular networks underlying prolificacy in this breed, in the present study, we identified the miRNA and mRNA expression profiles in follicular phase ovaries from goats with large and small litter sizes. Important miRNA-mRNA negative correlation interaction networks and pathways associated with prolific traits were identified. Our aim was to help understand the molecular mechanisms involved in the formation of prolific traits in the follicular phase ovaries of goats and to provide a basis for further investigation of the interactions among miRNAs, mRNAs and signaling pathways associated with prolificacy. Therefore, the Yunshang black goat might provide a typical model for studying prolific traits in mammals. Our study also provides basic data for breed trait mining and resource conservation.

## Results

### cDNA library sequencing and transcriptome profiles of the ovary

**Fig. 1:**
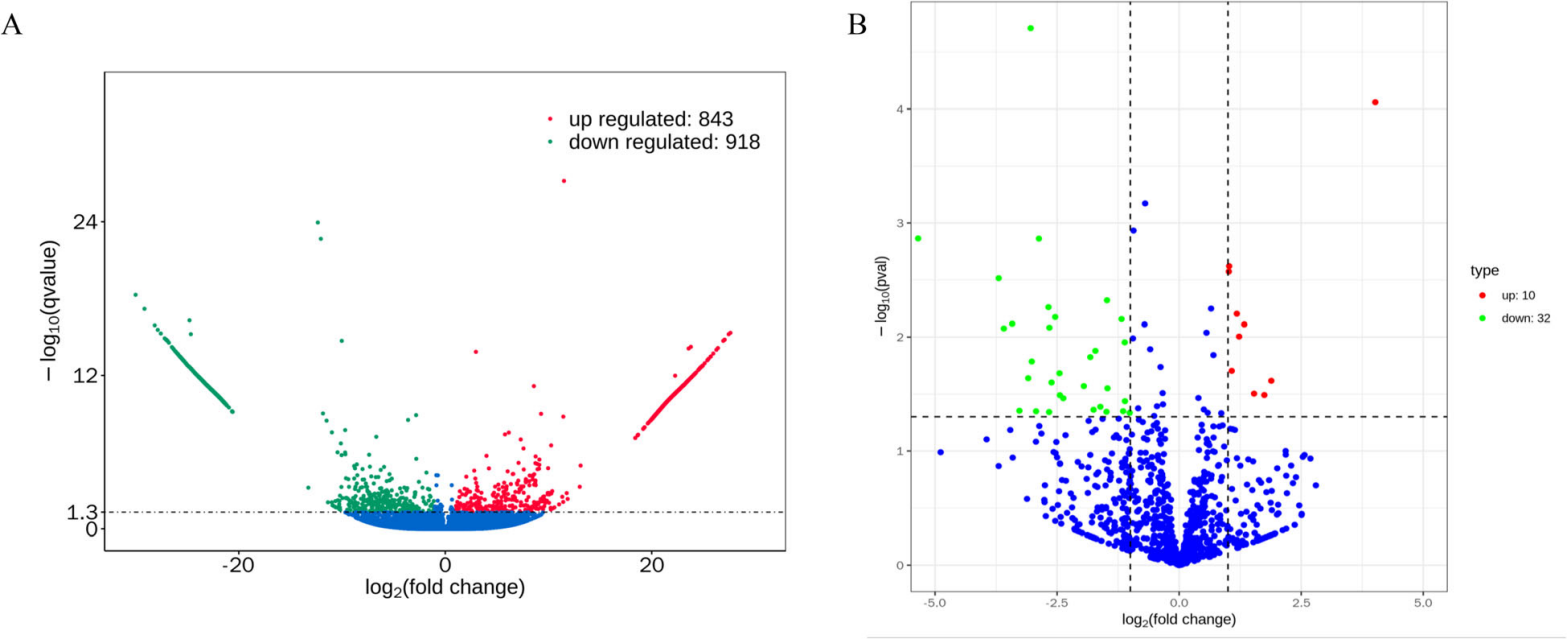
Differentially expressed genes (DEGs) and miRNAs (DEMs) in goat ovaries. **A** Volcano diagram of DEGs in the comparison. **B** Volcano diagram of DEMs in the comparison

RNA-seq was performed on 10 cDNA libraries using the Illumina Novaseq platform. The AMPure XP system (Beckman Coulter, Beverly, MA, United States) was used to purify library fragments, preferentially selecting cDNA fragments of 150 bp in length. Greater than 50 million raw reads were generated from each library. After filtering out the low-quality reads, the clean reads ranged from 15.1 to 24.6 G, with more than 92.9% of the clean reads mapped to the genome (additional files, Table S1). Although the GC content of the clean data ranged from 45.8 to 53.3%, the quality scores of the clean reads were higher than 98.1 and 92.3% for Q20 and Q30, respectively, indicating that the reliability and quality of the sequencing data were adequate for further analysis. Finally, a total of 43,779 known transcripts and 23,067 novel transcripts were identified in the data (shown in additional files, Table S2). To investigate the key mRNAs involved in the formation of reproductive traits in goats, we analyzed the differentially expressed genes (DEGs) in the ovaries of the LF and HF groups of Yunshang black goats. The number of up- and downregulated genes between the HF and LF groups is shown in Fig. 1A, and a list of the DEGs identified in the comparison is given in additional file Table S3. The log2 (fold change) values ranged from − 30 to 27.62186.

### Sequencing of small RNA library and miRNA transcriptome profiling analysis in the ovary

The ovarian tissues collected from goats in the LF and HF comparison were also used to construct small RNA libraries. A total of 1.084 to 1.416 G clean reads were generated after filtering the low-quality reads, and more than 93.95% of these reads were mapped to the genome (additional files, Table S4). The Q20 and Q30 quality scores were greater than 99.12 and 96.24%, respectively, and the GC content of the clean data ranged from 48.44 to 49.93%. These results showed that the samples were reliable and of high quality. Finally, 424 known miRNAs and 656 novel miRNAs were identified (additional files, Table S5). To investigate the key miRNAs involved in prolificacy, we analyzed the DEMs in the comparison. The number of up- and downregulated miRNAs in the HF vs. LF comparison is shown in Fig. 1B.

### Correlation analysis of differentially expressed miRNAs and mRNAs involved in prolificacy

**Fig. 2:**
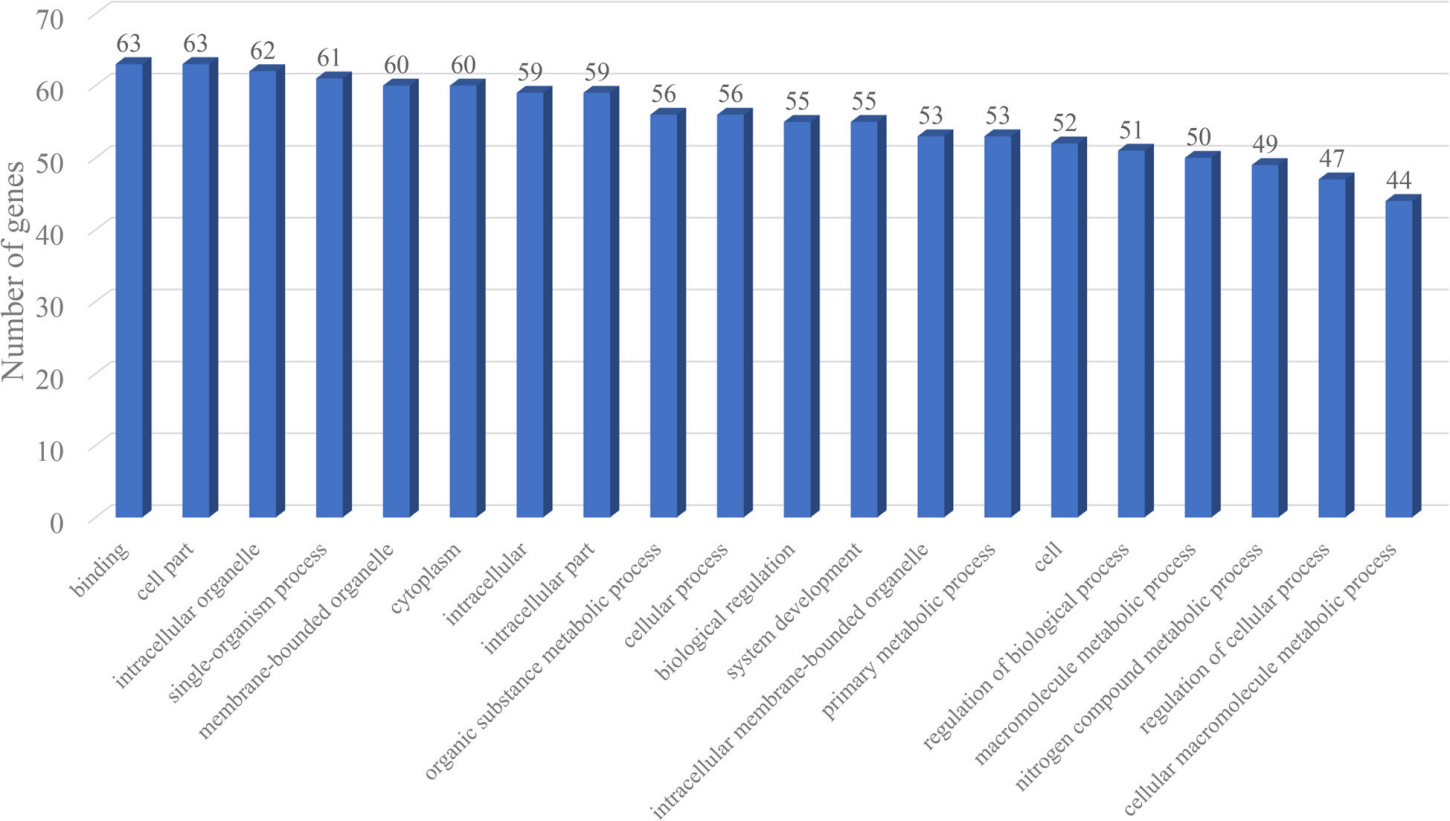
The top 20 GO terms enriched in the comparison

We integrated the miRNA and mRNA data from Yunshang black goat ovaries, predicted the potential target genes of the DEMs, completed the correlation analysis of the DEM-mRNA pairs, and screened DEM-mRNA pairs with negatively correlated expression. A total of 263 miRNA-mRNA pairs were predicted in the LF vs. HF comparison (Table 1, Pages 23–25). Among these pairs, 115 miRNA-mRNA pairs were found to have negatively correlated expression (*q* value < 0.1). To gain insights into the functions of the miRNA-mRNA pairs with negatively correlated expression between the groups with different litter sizes, we performed GO enrichment analysis to reveal the enriched biological process terms (corrected *P* values < 0.05). The associations were mainly with cellular components and the binding of molecular functions (Fig. 2). The GO terms related to reproductive traits are also shown in Table 2, and these terms include reproductive process, ovulation cycle, estrogen receptor binding and ovarian follicle development.

**Table 1:** DE mRNA-miRNA pairs in the ovaries of Yunshang black goats

miRNA	target_mRNA	target_gene
chi-miR-106a-5p	XM_018041407.1; XR_001918311.1; XM_018064479.1; XM_018057889.1; XM_018053373.1	IDE; LOC102191038; MINK1; PTPRT; SYNE1
chi-miR-1271-3p	XM_018054838.1; XM_005686081.3; XM_013969748.2; XM_018066975.1; XM_018048727.1; XM_018040274.1; XM_018053425.1; XM_018044841.1; XM_018064209.1; XR_001918311.1; XM_018041660.1; XM_018053556.1; XM_018059006.1; XM_018039504.1; XM_018043955.1; XM_018061012.1; XM_018057639.1	ADRA2B; AREL1; ARRB1; ATG7; CDC42EP1; CLEC16A; FNDC1; GNB1L; LOC102179130; LOC102191038; MAP 7D1; MYO9A; NCAPD3; PIAS2; STARD8; THOC5; TXLNA
chi-miR-129-3p	XM_018049845.1; XM_018063764.1; XM_005681729.3; XM_018058010.1; XM_013962525.2; XM_018064112.1; XM_018053317.1	ADD1; EXOC7; JCHAIN; S100PBP; SLC16A1; SPC25; STXBP5
chi-miR-133a-5p	XM_018064437.1; XR_001917960.1; XM_018056398.1; XM_005690449.3; XM_013963206.2; XM_018043375.1; XM_005699249.3; XM_018039076.1; XM_018052928.1	ATP2A3; C1H3orf70; CLYBL; CR2; LOC100860959; LOC102176672; RPS24; SYNGAP1; UBE3D
chi-miR-150	XM_005689442.3; XM_018044583.1	C15H11orf57; CASK
chi-miR-197-5p	XM_013974705.2; XM_013964246.2; XM_005686081.3; XM_018040526.1; XM_018048707.1; XM_005685676.3; XM_018059948.1; XM_018066137.1; XM_005698368.3; XM_018060910.1; XM_005697014.3; XM_018041659.1; XM_018040051.1; XM_018066046.1; XM_005695796.3; XM_018056069.1; XM_005688536.3; XM_018060998.1; XM_018039563.1; XM_013963935.1; XM_018043108.1; XM_018059808.1; XR_001918292.1; XM_005687506.3; XM_005694147.3; XM_018064309.1; XM_018043955.1; XM_018054388.1; XM_018053944.1; XM_018057639.1; XM_018061433.1; XM_018059762.1; XM_018057873.1; XM_018061466.1	ABI3BP; ANO2; AREL1; ARHGAP17; BCL2L13; CA12; CFAP74; CLK3; CUEDC2; DERL3; DSG3; EBF3; EME2; EXOC3L4; FLNB; KCNT1; MANBAL; MTMR3; MYO5B; NACA; PICALM; POU2AF1; PPARGC1B; SERTM1; SLC16A5; SPAG5; STARD8; STARD9; TTLL5; TXLNA; ULK1; USP2; ZHX3; ZNF605
chi-miR-20b	XM_018042438.1	RBP1
chi-miR-22-5p	XM_018065844.1; XM_018049669.1; XM_018059762.1	LIMS2; TFDP2; USP28
chi-miR-2290	XR_001917611.1; XM_018065632.1; XM_018064479.1; XM_018055379.1	CC2D1B; EGFLAM; MINK1; WNT4
chi-miR-2332	XM_018058543.1; XM_018066965.1	FAM91A1; MKRN2OS
chi-miR-324-3p	XM_018040427.1; XM_018067206.1; XM_013964246.2; XM_018040526.1; XM_018040189.1; XM_018051909.1; XM_018044583.1; XM_018061310.1; XM_005686853.3; XM_018059700.1; XM_018055747.1; XM_018059522.1; XM_018060910.1; XM_013972218.2; XM_018057810.1; XM_018056058.1; XM_018048123.1; XM_005701565.3; XM_018065660.1; XM_018062272.1; XM_018066878.1; XM_018064290.1; XM_018049887.1; XM_005690341.3; XM_018058938.1; XR_001919614.1; XM_018058118.1; XM_018063080.1; XM_018064421.1; XM_018051474.1; XM_018042980.1; XM_018049665.1; XM_018039202.1; XM_018055379.1; XM_018057873.1; XM_018054539.1	ABCA3; ALS2CL; ANO2; ARHGAP17; ATXN2L; CAD; CASK; CLIP1; CLIP4; CXCR5; DENND1A; DENND5A; DERL3; DHRS7B; DLGAP4; GBGT1; ITGA7; LOC102174648; LPCAT1; LRP3; LRRFIP2; LYRM9; MFSD7; MS4A1; OBSL1; PGS1; PHC2; PPP6R1; RAP1GAP2; SLC25A42; SYVN1; TFDP2; TMEM14A; WNT4; ZHX3; ZNF219
chi-miR-671-5p	XM_005675149.3; XM_005682894.3; XM_005683975.3; XM_013962885.2; XM_005686284.2; XM_018066965.1; XM_018038476.1; XM_018058490.1; XM_005701709.3; XM_018066993.1; XM_018051145.1	ADIPOQ; AZU1; DMTN; FAAH; LYG2; MKRN2OS; SCN10A; SP140; TCF19; TRAK1; ZNF557
chi-miR-877-3p	XM_018038615.1; XM_018067206.1; XM_018064437.1; XM_018042365.1; XM_018061265.1; XM_013974717.2; XM_018060910.1; XM_018064555.1; XM_018050130.1; XM_018043465.1; XM_018054948.1; XM_018064688.1; XM_018057830.1; XM_018045584.1; XM_018048271.1; XR_001919058.1; NM_001285620.1; XM_018050077.1; XM_018048718.1; XM_005690341.3; XM_005687901.3; XM_018064421.1; XM_018055004.1; XM_013962525.2; XM_018056156.1; XM_018063111.1; XM_018047914.1; XM_018040289.1	ABHD16A; ALS2CL; ATP2A3; CAMK2G; CAMKK2; CD19; DERL3; ELP5; G3BP1; INCENP; LOC102168673; LOC102186431; LOC108633201; LOC108635459; LOC108636023; LOC108637413; MAP 34-B; MAT2B; MICAL3; MS4A1; PARD3; RAP1GAP2; RTKN; SLC16A1; TMEM8C; TNNT1; YAF2; ZNF48
novel_1007	XM_018040882.1	GTF2IRD1
novel_310	XM_018040526.1; XM_018059648.1; XM_018042405.1; XM_018051199.1; XM_018064421.1	ARHGAP17; ARHGEF12; KCNMA1; PALM3; RAP1GAP
novel_438	XM_018066196.1; XM_018042503.1; XM_018067206.1; XM_018048138.1; XM_018046320.1; XM_013969748.2; XM_018040189.1; XM_018038690.1; XM_018038657.1; XM_005699439.3; XM_018048727.1; XR_001919744.1; XM_018038867.1; XM_018043863.1; XM_005683975.3; XR_001918836.1; XM_018065632.1; XM_018052116.1; XM_005695796.3; XM_018061064.1; XM_018048123.1; XM_018042405.1; XM_018059422.1; XM_018043129.1; XM_018065660.1; XM_018054954.1; XM_018064479.1; XM_018060966.1; XM_018064342.1; XM_018043433.1; XM_018050582.1; XM_018061395.1; XM_018057706.1; XM_018044560.1; XM_018040897.1; XM_018038847.1; XM_018063080.1; XM_018052538.1; XR_001919368.1; XM_018057252.1; XM_005675055.2; XM_018064900.1; XM_018063718.1; XM_005694147.3; XM_018062143.1; XM_013962917.2; XM_018039076.1; XM_018057826.1; XM_013976203.2; XM_018038907.1; XM_018048633.1; XM_018050486.1; XM_018051734.1; XM_018040289.1; XM_018064916.1	ADAMTSL3; AIFM2; ALS2CL; ANKS1B; ARHGEF11; ARRB1; ATXN2L; BEND6; CAPN11; CCDC81; CDC42EP1; CDK12; DDR1; DLG3; DMTN; DNMT3A; EGFLAM; FAM214B; FLNB; HPS4; ITGA7; KCNMA1; LOC102178093; LOC102180829; LPCAT1; MAP 4; MINK1; MORC2; MYO18A; MYRF; N4BP3; NCOR2; NOL4L; PARS2; PKMYT1; PPP1R10; PPP6R1; PRMT2; PXN; RIN2; RUBCN; SAMD14; SEPT9; SLC16A5; SLC9A5; ST3GAL3; SYNGAP1; TLDC2; TMEM164; TNXB; TULP3; WDR18; WDR4; ZNF48; ZNF652
novel_535	XM_018040734.1; XM_005690924.3; XM_018064170.1; XM_018049845.1; XM_013964246.2; XM_018043902.1; XM_018040526.1; XM_018061343.1; XM_018064437.1; XM_018040189.1; XM_005682894.3; XM_018051909.1; XM_018040274.1; XM_018040051.1; XM_018059451.1; XM_018039067.1; XM_018044491.1; XM_018043071.1; XM_018044841.1; XM_018049805.1; XR_001919624.1; XM_005696018.3; XM_018062272.1; XM_013972345.2; XM_018058938.1; XM_018052180.1; XM_018040897.1; XM_005699158.3; XM_018064421.1; XM_005694147.3; XM_018051474.1; XM_018040224.1; XM_018066557.1; XM_018041207.1; XM_018061012.1; XM_018050841.1; XM_018056156.1; XM_013971985.2; XM_018066993.1	ABCC1; ABL2; ACACA; ADD1; ANO2; AP1S2; ARHGAP17; ARL6IP4; ATP2A3; ATXN2L; AZU1; CAD; CLEC16A; EME2; FAM160A2; FARS2; FBXW5; GAB2; GNB1L; HGFAC; JMJD6; LOC102173049; LRP3; METTL23; OBSL1; PAX5; PKMYT1; PSAP; RAP1GAP; SLC16A5; SLC25A42; SMURF1; STRA6; TCF7L2; THOC5; TJP3; TMEM8C; TNFRSF13B; TRAK1
novel_772	XM_018053542.1; XM_013972218.2; XM_018064916.1	BMP4; DHRS7B; ZNF652
novel_929	XM_018053425.1	FNDC1

**Table 2:** GO terms related to reproduction traits

GO ID	GO_term	Gene name	***P Value***
GO:0022414	reproductive process	PSAP; BMP4; WNT4; PHC2	0.3325582
GO:0061458	reproductive system development	PSAP; BMP4; WNT4	0.01212244
GO:0048608	reproductive structure development	PSAP; BMP4; WNT4	0.01135883
GO:0046879	hormone secretion	CAMK2G; ARRB1; SLC16A1	0.209929
GO:0044703	multiorganism reproductive process	MAP 34-B; BMP4; WNT4	0.7181564
GO:0032504	multicellular organism reproduction	BMP4; WNT4; PHC2	0.5974827
GO:0030072	peptide hormone secretion	SLC16A1; ARRB1; CAMK2G	0.1901019
GO:0019953	sexual reproduction	BMP4; WNT4; PHC2	0.5027936
GO:0003006	developmental process involved in reproduction	PSAP; BMP4; WNT4	0.0233747
GO:2000242	negative regulation of reproductive process	WNT4; BMP4	0.01002786
GO:2000241	regulation of reproductive process	BMP4; WNT4	0.03072193
GO:0022412	cellular process involved in reproduction in multicellular organism	WNT4; BMP4	0.3218491
GO:0009755	hormone-mediated signaling pathway	BMP4; PRMT2	0.2756622
GO:0060135	maternal process involved in female pregnancy	WNT4	0.7846507
GO:0048599	oocyte development	WNT4	0.3290652
GO:0048477	oogenesis	WNT4	0.3216878
GO:0042698	ovulation cycle	BMP4	0.6052335
GO:0030331	estrogen receptor binding	PRMT2	0.315868
GO:0022602	ovulation cycle process	BMP4	0.5585276
GO:0009994	oocyte differentiation	WNT4	0.3685613
GO:0001541	ovarian follicle development	BMP4	0.7456533

### miRNA-mRNA interaction network related to reproduction

**Fig. 3:**
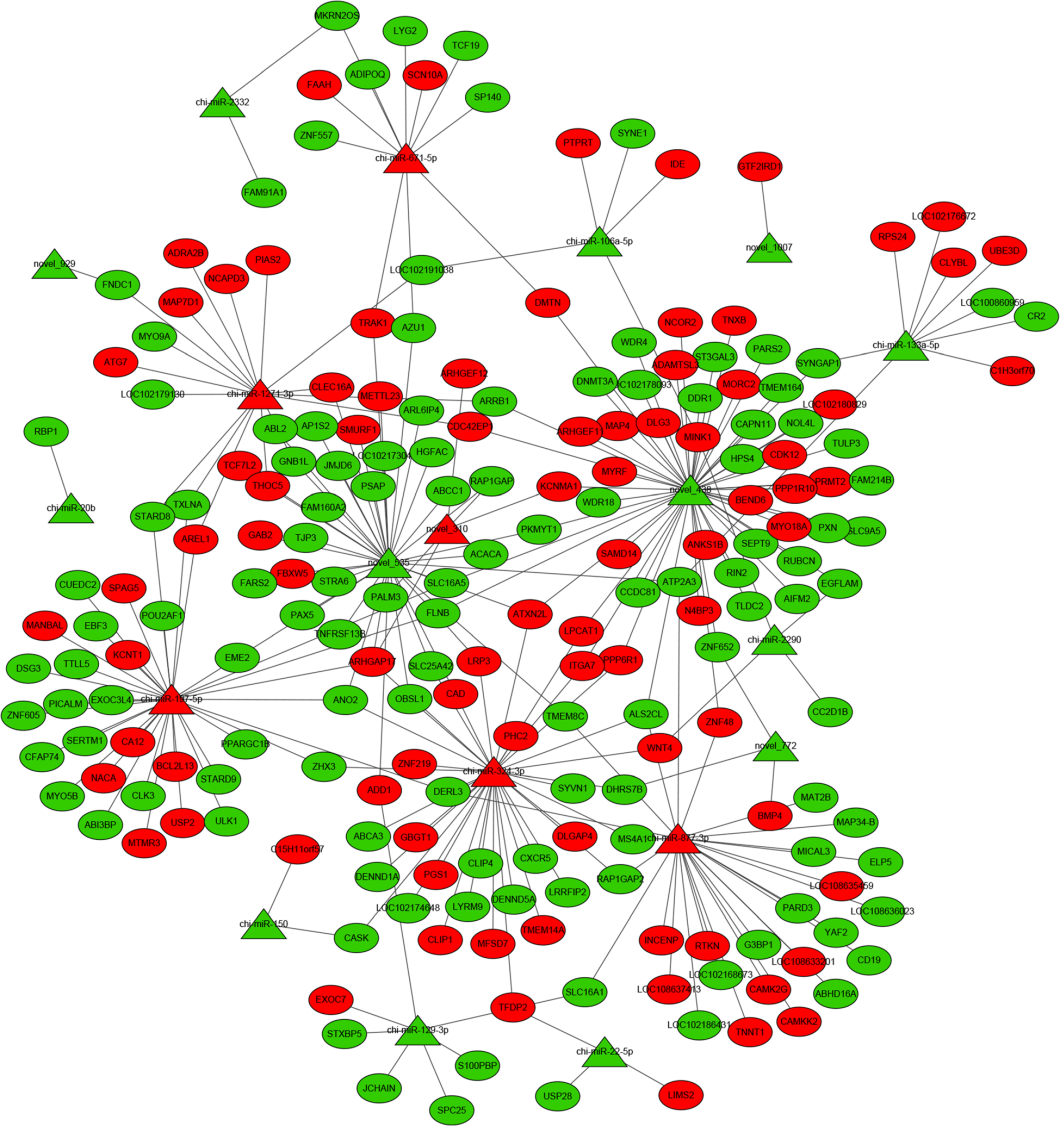
The DE miRNA-mRNA interaction network in the comparison. Red color: upregulated; Green color: downregulated

To identify key molecular players in the prolificacy of goat ovaries, we focused on some DEG-DEM interactions associated with the ovaries of goats with different litter sizes based on the annotations of the negatively correlated miRNA-mRNA pairs at the expression level (Fig. 3 and Table 1, Pages 23–25). For example, novel_438 was a core gene in the network (Fig. 3), and a total of 55 miRNA-mRNA pairs were identified whose targets were closely related to biological processes such as hormone secretion, hormone-mediated signaling pathways and protein binding. The core gene of the *SLC16A5* network was targeted by 3 miRNAs, including novel_438, novel_535 and chi-miR-197-5p. Moreover, the network revealed chi-miR-324-3p as an important node, and its targets were associated with oocyte development. The results indicated that *WNT4* is targeted by 2 miRNAs, namely, chi-miR-324-3p and chi-miR-2290.

### miRNA pathways related to reproduction

To further understand how DEMs work with DEGs in pathways regulating goat prolificacy, we performed KEGG pathway analysis of the miRNA-mRNA pairs with correlated expression. The KEGG terms enriched in pathway categories for the comparison are shown in Table 3 (Pages 25–27). The two top pathways were autophagy-animal (ko04140) and Hippo signaling pathways (ko04390). Interestingly, we found that five signaling pathways, including oocyte meiosis (ko04114), progesterone-mediated oocyte maturation (ko04914), the GnRH signaling pathway (ko04912), the Notch signaling pathway (ko04330) and the TGF-beta signaling pathway (ko04350), were directly and undirectedly associated with the reproductive process. Eight genes, including *CAMK2G, NCOR2, PKMYT1, PKMYT1, SMURF1, BMP4, WNT4* and *TCF7L2,* were involved in the five pathways and might be important candidate genes for further study.

**Table 3:** The KEGG pathways enriched in the comparison

Gene name	KEGG Pathway	Pathway ID
ABCA3	ABC transporters	ko02010
ABCC1	Sphingolipid signaling pathway; Vitamin digestion and absorption; ABC transporters	ko04071; ko04977; ko02010
ABL2	ErbB signaling pathway; Ras signaling pathway;	ko04012; ko04014;
ACACA	Aflatoxin biosynthesis; AMPK signaling pathway; Fatty acid biosynthesis; Fatty acid metabolism; Glucagon signaling pathway; Insulin signaling pathway; Propanoate metabolism; Pyruvate metabolism;	ko00254; ko04152; ko00061; ko01212; ko04922; ko04910; ko00640; ko00620
ADIPOQ	Adipocytokine signaling pathway; AMPK signaling pathway; Longevity regulating pathway – mammal; PPAR signaling pathway;	ko04920; ko04152; ko04211; ko03320;
ADRA2B	cGMP - PKG signaling pathway; Neuroactive ligand-receptor interaction;	ko04022; ko04080;
ANKS1B	Aldosterone synthesis and secretion;	ko04925;
ANO2	Olfactory transduction;	ko04740;
AP1S2	Lysosome;	ko04142;
ARHGEF11	Vascular smooth muscle contraction	ko04270
ARHGEF12	Axon guidance; Platelet activation; Regulation of actin cytoskeleton; Vascular smooth muscle contraction;	ko04360; ko04611; ko04810; ko04270
ARRB1	Chemokine signaling pathway; Endocytosis; Hedgehog signaling pathway; MAPK signaling pathway; Olfactory transduction;	ko04062; ko04144; ko04340; ko04010; ko04740;
ATG7	Autophagy – animal; Autophagy - other eukaryotes; Autophagy – yeast; Ferroptosis;	ko04140; ko04136; ko04138; ko04216
ATP2A3	Calcium signaling pathway; cGMP - PKG signaling pathway; Pancreatic secretion;	ko04020; ko04022; ko04972;
BCL2L13	Mitophagy – animal;	ko04137;
BMP4	Hedgehog signaling pathway – fly; Hippo signaling pathway; MAPK signaling pathway – fly; Signaling pathways regulating pluripotency of stem cells; TGF-beta signaling pathway; Thyroid hormone signaling pathway;	ko04341; ko04390; ko04013; ko04550; ko04350; ko04919;
CA12	Nitrogen metabolism;	ko00910;
CAMK2G	Adrenergic signaling in cardiomyocytes; Axon guidance; Calcium signaling pathway; Cholinergic synapse; Circadian entrainment; Dopaminergic synapse; ErbB signaling pathway; Gastric acid secretion; Glucagon signaling pathway; GnRH signaling pathway; HIF-1 signaling pathway; Inflammatory mediator regulation of TRP channels; Insulin secretion; Long-term potentiation; Melanogenesis; Necroptosis; Neurotrophin signaling pathway; Olfactory transduction; Oocyte meiosis; Oxytocin signaling pathway; Phototransduction – fly; Wnt signaling pathway	ko04261; ko04360; ko04020; ko04725; ko04713; ko04728; ko04012; ko04971; ko04922; ko04912; ko04066; ko04750; ko04911; ko04720; ko04916; ko04217; ko04722; ko04740; ko04114; ko04921; ko04745; ko04310
CAMKK2	Adipocytokine signaling pathway; AMPK signaling pathway; Autophagy – animal; Longevity regulating pathway – mammal; Oxytocin signaling pathway;	ko04920; ko04152; ko04140; ko04211; ko04921;
CD19	B cell receptor signaling pathway; Hematopoietic cell lineage; PI3K-Akt signaling pathway;	ko04662; ko04640; ko04151;
CLIP1	mTOR signaling pathway;	ko04150;
CR2	B cell receptor signaling pathway; Complement and coagulation cascades; Hematopoietic cell lineage;	ko04662; ko04610; ko04640;
CXCR5	Chemokine signaling pathway; Cytokine-cytokine receptor interaction;	ko04062; ko04060;
DLG3	Hippo signaling pathway; Tight junction;	ko04390; ko04530;
DNMT3A	Cysteine and methionine metabolism;	ko00270;
EXOC7	Insulin signaling pathway;	ko04910;
FAAH	Retrograde endocannabinoid signaling;	ko04723;
FARS2	Aminoacyl-tRNA biosynthesis	ko00970;
FLNB	Focal adhesion; MAPK signaling pathway;	ko04510; ko04010;
GAB2	Fc epsilon RI signaling pathway; Fc gamma R-mediated phagocytosis; Osteoclast differentiation; Phospholipase D signaling pathway; Ras signaling pathway; Sphingolipid signaling pathway;	ko04664; ko04666; ko04380; ko04072; ko04014; ko04071;
GBGT1	Glycosphingolipid biosynthesis - globo and isoglobo series;	ko00603;
GTF2IRD1	Basal transcription factors; cGMP - PKG signaling pathway;	ko03022; ko04022;
ITGA7	ECM-receptor interaction; Focal adhesion; PI3K-Akt signaling pathway; Regulation of actin cytoskeleton;	ko04512; ko04510; ko04151; ko04810;
KCNMA1	cGMP - PKG signaling pathway; Insulin secretion; Pancreatic secretion; Renin secretion; Salivary secretion; Vascular smooth muscle contraction	ko04022; ko04911; ko04972; ko04924; ko04970; ko04270
LPCAT1	Ether lipid metabolism; Glycerophospholipid metabolism;	ko00565; ko00564;
MAP 34-B	NOD-like receptor signaling pathway; Salivary secretion;	ko04621; ko04970;
MAP 4	MAPK signaling pathway;	ko04010;
MAT2B	Biosynthesis of amino acids; Cysteine and methionine metabolism;	ko01230; ko00270;
MS4A1	Hematopoietic cell lineage;	ko04640;
MTMR3	Autophagy – animal; Inositol phosphate metabolism; Phosphatidylinositol signaling system;	ko04140; ko00562; ko04070;
NCOR2	Notch signaling pathway;	ko04330;
PARS2	Aminoacyl-tRNA biosynthesis	ko00970;
PARD3	Axon guidance; Chemokine signaling pathway; Endocytosis; Hippo signaling pathway; Hippo signaling pathway -fly; Neuroactive ligand-receptor interaction; Rap1 signaling pathway; Tight junction;	ko04360; ko04062; ko04144; ko04390; ko04391; ko04080; ko04015; ko04530
PKMYT1	Cell cycle; Oocyte meiosis; Progesterone-mediated oocyte maturation;	ko04110; ko04114; ko04914;
PGS1	Glycerophospholipid metabolism;	ko00564;
PIAS2	Jak-STAT signaling pathway; Ubiquitin mediated proteolysis;	ko04630; ko04120;
PSAP	Lysosome;	ko04142;
PXN	Chemokine signaling pathway; Focal adhesion; Leukocyte transendothelial migration; Regulation of actin cytoskeleton; VEGF signaling pathway;	ko04062; ko04510; ko04670; ko04810; ko04370
RPS24	Ribosome;	ko03010;
SMURF1	Adherens junction; Axon guidance; cAMP signaling pathway; Endocytosis; Hedgehog signaling pathway; Hedgehog signaling pathway – fly; TGF-beta signaling pathway; Ubiquitin mediated proteolysis	ko04520; ko04360; ko04024; ko04144; ko04340; ko04341; ko04350; ko04120
ST3GAL3	Glycosaminoglycan biosynthesis - keratan sulfate; Glycosphingolipid biosynthesis - lacto and neolacto series; Mannose type O-glycan biosynthesis; Other types of O-glycan biosynthesis; Various types of N-glycan biosynthesis;	ko00533; ko00601; ko00515; ko00514; ko00513;
SYNGAP1	Ras signaling pathway;	ko04014;
SYVN1	Protein processing in endoplasmic reticulum; Ubiquitin mediated proteolysis;	ko04141; ko04120;
TCF7L2	Adherens junction; Hippo signaling pathway; Melanogenesis; Wnt signaling pathway	ko04520; ko04390; ko04916; ko04310
TFDP2	Cell cycle;	ko04110;
THOC5	RNA transport;	ko03013;
TJP3	Tight junction	ko04530
TNFRSF13B	Cytokine-cytokine receptor interaction; Intestinal immune network for IgA production;	ko04060; ko04672;
TNXB	ECM-receptor interaction; Focal adhesion; PI3K-Akt signaling pathway;	ko04512; ko04510; ko04151;
ULK1	Autophagy – animal; Autophagy - other eukaryotes; Autophagy – yeast; Longevity regulating pathway – worm; Mitophagy – yeast; mTOR signaling pathway;	ko04140; ko04136; ko04138; ko04212; ko04139; ko04150;
USP2	IL-17 signaling pathway;	ko04657;
WNT4	Hippo signaling pathway; Melanogenesis; mTOR signaling pathway; Signaling pathways regulating pluripotency of stem cells; Thyroid hormone signaling pathway; Wnt signaling pathway;	ko04390; ko04916; ko04150; ko04550; ko04919; ko04310;

### PPI network related to prolificacy trait

**Fig. 4:**
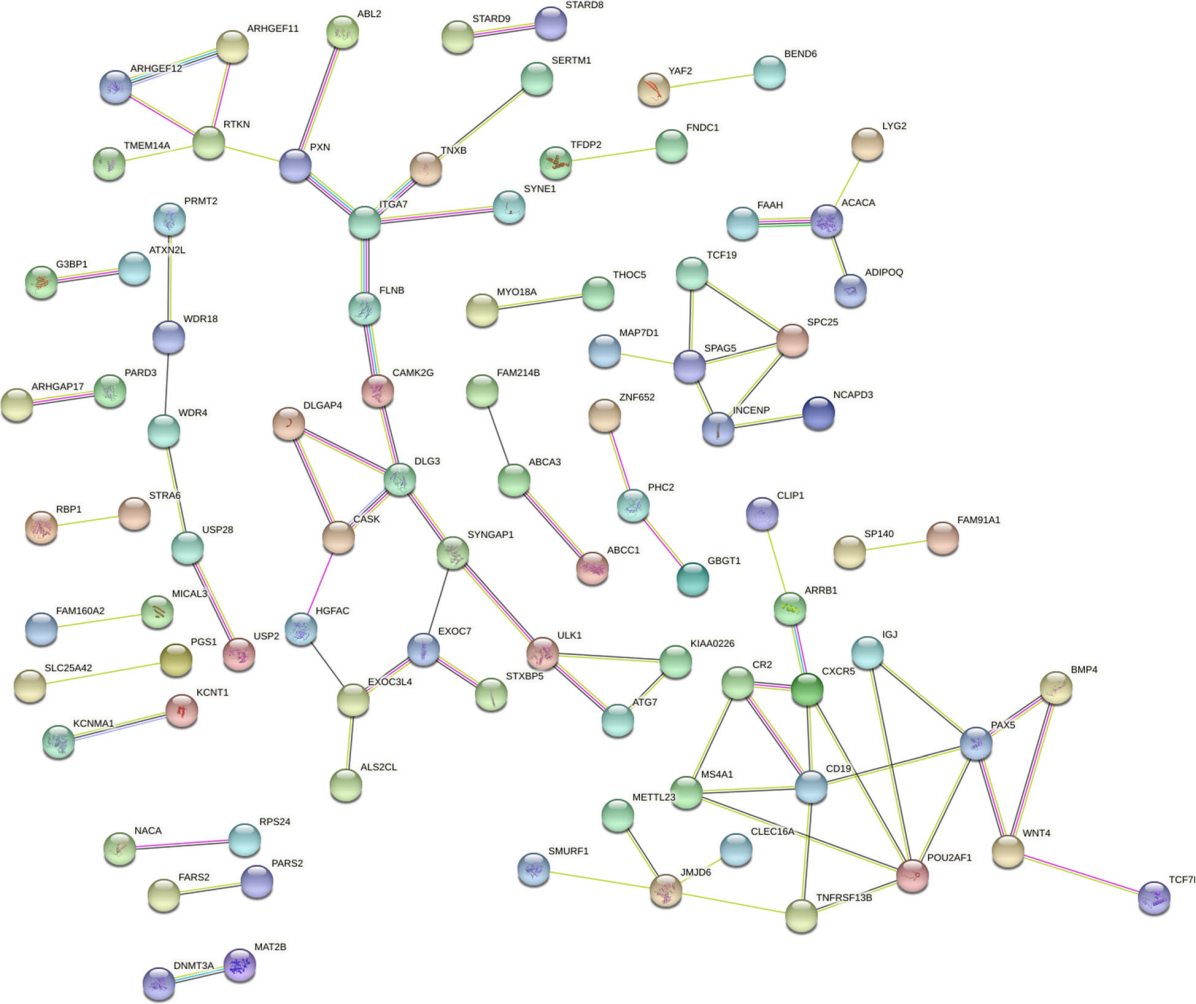
The protein–protein interaction (PPI) network of differentially expressed proteins in the comparison

The PPI network was constructed using the extracted target gene list from the STRING database in Cytoscape (Fig. 4). The PPI network of the DEGs in the LF vs. HF comparison contained 98 protein-protein nodes. *CDK12, FAM91A1, PGS1, SERTM1, SPAG5, SYNE1, TMEM14A, WNT4, CAMK2G* and other genes were the key core nodes of the networks, and all of them were targets of DE miRNAs.

### Verification of miRNA and mRNA expression profiles with RT–qPCR

**Fig. 5:**
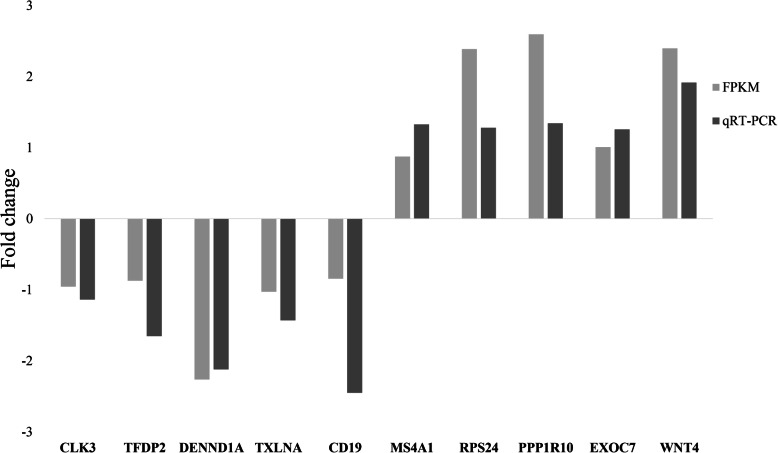
Real-time quantitative PCR (RT–qPCR) validation of differentially expressed mRNAs

**Fig. 6:**
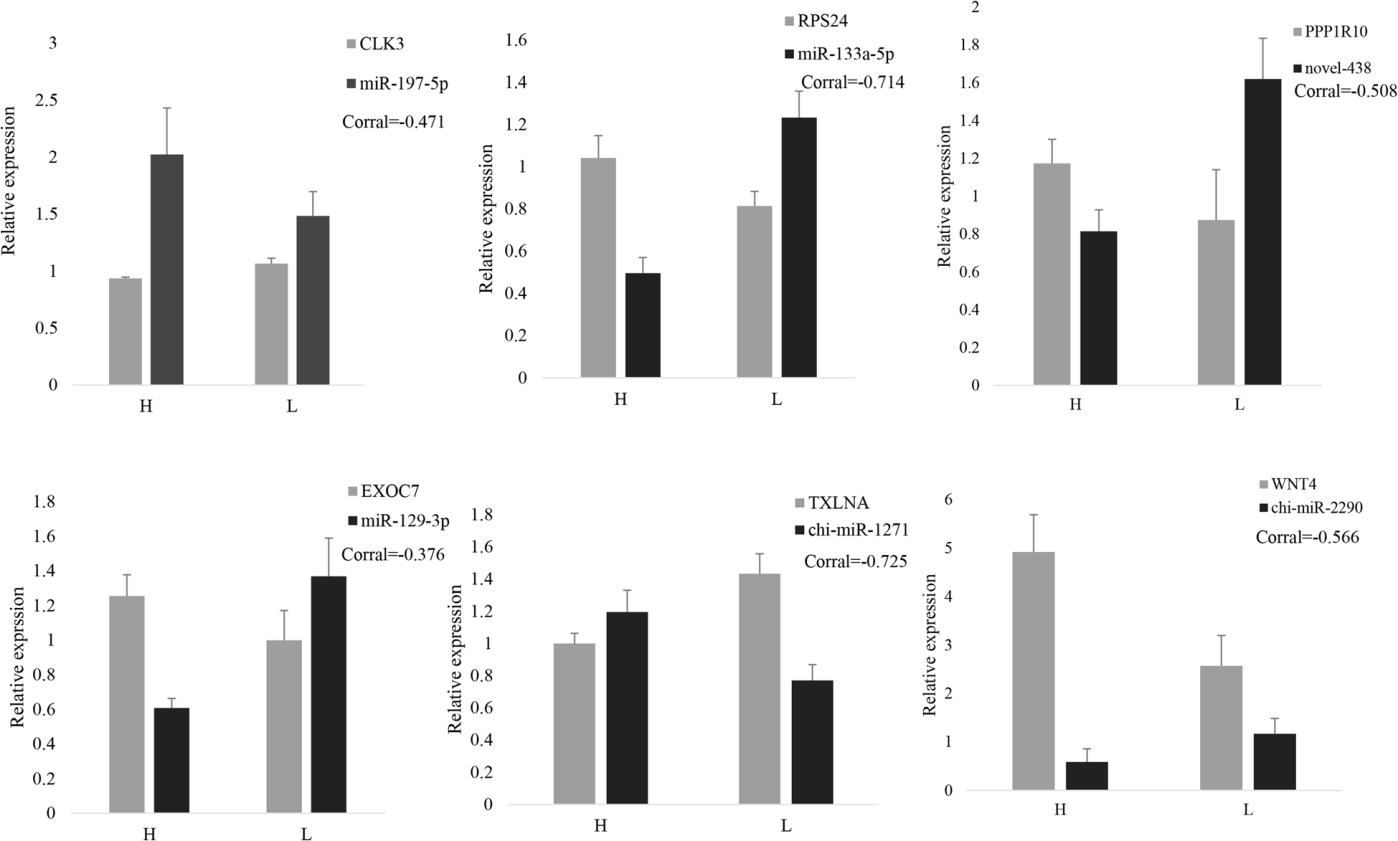
Real-time quantitative PCR validation of the differentially expressed miRNAs and their corresponding target mRNAs

The frequently occurring genes in the miRNA-mRNA pairs, including *CLK3, RPS24, TFDP2, PPP1R10, EXOC7, DENND1A, TXLNA, MS4A1, CD19* and *WNT4,* were selected for RT–qPCR validation. The gene expression levels were determined and compared with the RNA-seq data, which showed similar patterns (Fig. 5), suggesting that the RNA-seq data were credible. Furthermore, seven miRNA-mRNA connections were negatively correlated, including miR-197-5p-*CLK3*, miR-133a-5p-*RPS24*, novel_438-*PPP1R10*, miR-129-3p-*EXOC7*, miR-1271-3p-*TXLNA* and miR-2290-*WNT4*, indicating that they have potential interactivity (Fig. 6).

### The effects of predicted miRNA-mRNA pairs on GC proliferation

**Fig. 7:**
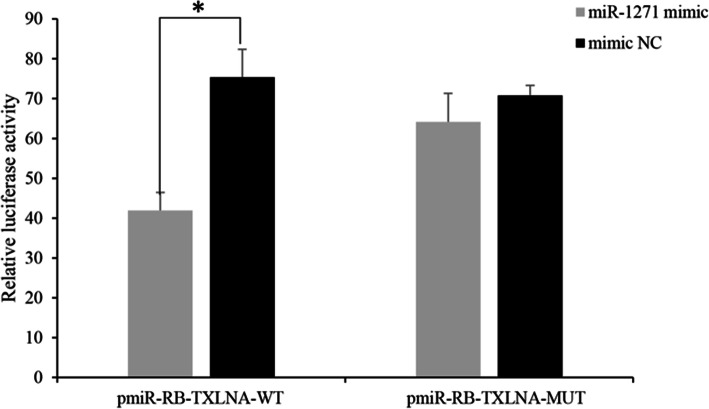
Verification of the predicted miRNA-mRNA pairs using a luciferase assay. *P < 0.05

**Fig. 8:**
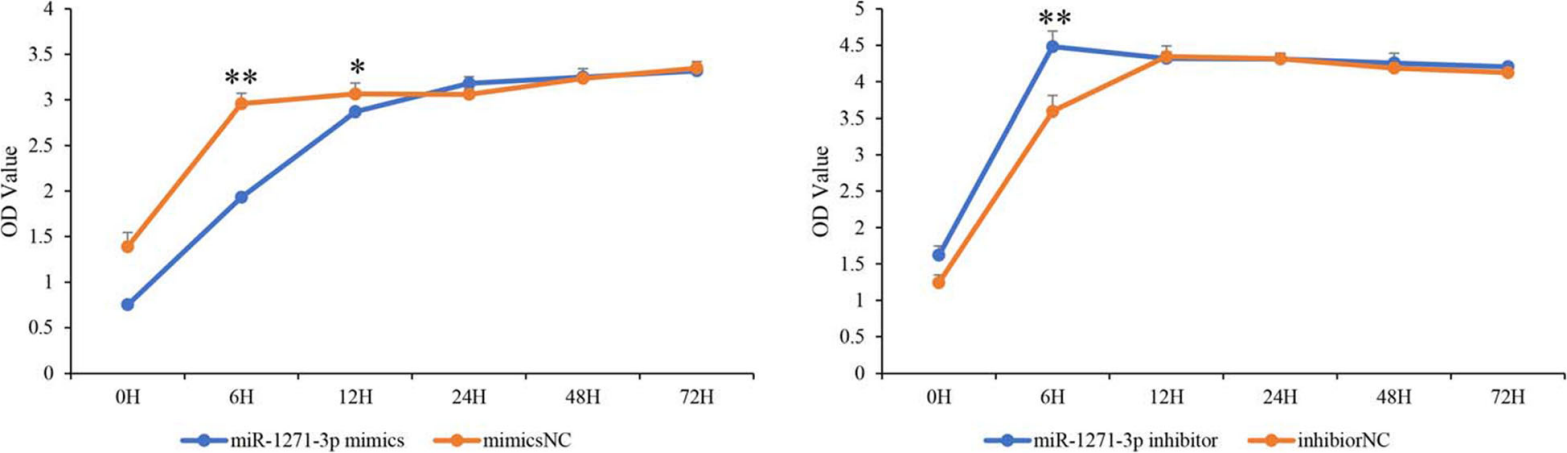
Overexpression and inhibition of miR-1271 in GCs stimulated cell proliferation, as determined by the CCK-8 method. *P < 0.05; **P < 0.01

To illustrate the functions of the predicted miRNA-mRNA pairs, two pairs were selected for dual luciferase reporter assays and CCK-8 assays. The dual luciferase reporter assay results showed that miR-1271 could bind to the 3’UTR of the *TXLNA* gene (Fig. 7). To further assess the functional role of miR-1271, we performed miRNA overexpression and repression assays. Goat GCs were used to optimize the dose of miR-1271 in the overexpression and repression experiments. We found that overexpression of miR-1271 significantly reduced the rapid cell proliferation rate and that inhibiting miR-1271 expression significantly increased cell proliferation, as measured by CCK-8 assay (Fig. 8).

## Discussion

Precise regulation of hormones, genes, noncoding RNAs and other factors, including their involvement in spatiotemporal expression, signal cascades, transcriptional regulation and feedback mechanisms, affects the prolificacy trait [21]. In recent years, many studies have been conducted on gene identification and functional research, miRNA identification and pathway regulation for prolificacy in goats. However, these studies have focused on estrus and entire reproductive cycle periods [19, 22], and there is still a lack of understanding of the molecular mechanisms associated with prolificacy in the follicular phase of goat ovaries. Therefore, it is important to identify these molecular mechanisms. In this study, RNA-seq was performed to assess the follicular phase ovaries of two groups (high- and low-yielding) of Yunshang black goats, and then miRNA and mRNA profiles related to prolificacy were constructed. In total, 43,779 known transcripts, 23,067 novel transcripts, 424 known miRNAs and 656 novel miRNAs were identified in the goat ovarian samples (additional files, Table S2). These data provide a valuable resource for analyzing the molecular mechanisms of prolificacy and transcriptional regulation in goat ovaries.

The Yunshang black goat is a local breed used for meat production in China. With its tender flesh, unique flavor and high fecundity, this breed has important development and utilization value. Therefore, it is crucial to understand the molecular mechanisms underlying prolificacy in Yunshang black goats for their development and utilization. Accompanied by the increase in FSH production, the follicular phase includes follicle recruitment and dominant follicle development. During this period, multiple follicles develop and ovulate [23, 24]. At present, there is a lack of understanding of the molecular mechanisms underlying the prolificacy of Yunshang black goat ovaries in the follicular phase. Differentially expressed genes and miRNAs may play important regulatory roles in prolificacy. In this study, 206 DEGs and 19 DEMs were identified as being differentially expressed in this comparison (Table 1). Thirteen genes, including *FAM91A1*, *DENND1A*, *SYNE1*, *TJP3*, *ST3GAL3*, *EGFLAM*, *SERTM1*, *WNT4*, *CDK12*, *TMEM164*, *TMEM14A*, *SPAG5*, *BMP4* and *PGS1*, were directly associated with fertility. Moreover, we screened DE miRNA-mRNA pairs in Yunshang black goat ovaries in the follicular phase and then performed functional enrichment analysis. The KEGG pathway enrichment results showed that in the LF vs. HF comparison, the genes were mostly enriched in pathways associated with fertility. These results indicate that the follicular phase is an important stage for the prolificacy of Yunshang black goat ovaries, which is consistent with findings from previous studies in goats, bovines and humans [17, 25, 26]. Therefore, the miRNA and mRNA expression profiles obtained in this study reflect the molecular regulation of the follicular phase of Yunshang black goat ovaries.

Prolificacy involves many biological processes. To gain insight into how miRNAs and their target genes regulate prolificacy, we constructed interaction networks between miRNAs and mRNAs related to prolificacy (Fig. 3). Among these interaction networks, *WNT4* was predicted to be a target of miRNAs, such as chi-miR-2290 and chi-miR-324-3p. This gene is also involved in biological processes including reproductive processes, oocyte development, oogenesis and oocyte differentiation. *WNT4* plays a regulatory role in ovarian follicular development and female fertility [27]. miR-324-3p has also been suggested to regulate ovarian cancer [28]. miR-2290 is a novel miRNA that was first reported in virus-infected bovines [29]. In our study, miR-2290 was found to be an important regulator of the *WNT4* gene in the Wnt signaling pathway. In addition, among the networks, the most complex miRNA-mRNA interaction network was based on miR-324-3p, novel_438 and novel_535 (Fig. 3). miR-1271 targeted *TXLNA*, and high *TXLNA* expression could be a favorable biomarker and therapeutic target for pancreatic adenocarcinoma patients [30]. In our study, *TXLNA* expression was significantly lower in the high-yielding goats than in the low-yielding goats, and the dual luciferase reporter assay showed that its expression was regulated by miR-1271. The CCK-8 assay showed that overexpression of miR-1271 could inhibit the proliferation of GCs and that inhibition of expression promoted the proliferation of GCs. We deduced that miR-1271-*TXLNA* was a potential regulatory pathway of goat prolificacy.

Furthermore, the network is related to ovarian follicular development and oogenesis. The interaction network consists of eight core genes, including *ARHGAP17, ALS2CL, ANO2, ATP2A3, ATXN2L, DERL3, MINK1* and *SLC16A5*, and 9 miRNAs, including miR-197-5p and miR-133a-5p. Some studies have confirmed that *ARHGAP17* is a member of the Rho family that is associated with follicular growth and granulosa cell maturation [31]. *SLC16A5* belongs to solute carrier proteins, which are included in the top 100 most highly expressed genes in opossum during pregnancy [32]. *MINK1* is a member of the germinal center kinases and is known to regulate cytoskeletal organization and oncogene-induced cell senescence, which is essential for cytokinesis [33]. *ATP2A3*, a member of the SERCA family, is an intracellular calcium pump and translocates cytosolic Ca^2+^ ions to the sarcoplasmic reticulum lumen. Its upregulated status in the uterus suggests that *ATP2A3* helps in oviposition [34]. In addition, miR-133a-5p is a target of the circular RNA scinderin (circSCIN), which affects estrogen secretion [35]. These findings suggest that these miRNA-mRNA interaction networks may play key roles in the regulation of prolificacy in follicular phase ovaries in Yunshang black goats, thus providing new insights for further understanding the regulatory mechanisms of goat prolificacy.

Follicle development in the follicular phase is the main factor affecting the ovulation rate of mammals [36, 37]. Therefore, the pathways involved in adhesion, growth, migration and differentiation of follicles might be critical for goat reproduction. In our study, the GnRH signaling pathway, oocyte meiosis, TGF-beta signaling pathway, Notch signaling pathway and Wnt signaling pathway were enriched in the follicular phase ovaries of goats with different litter sizes (Table 3, Pages 27–29). Similar results have been reported in previous studies of other species [38, 39]. In fact, the GnRH signaling pathway has an integral role in the process of ovulation, such as initiating pituitary secretion of luteinizing hormone (LH) and follicle-stimulating hormone [40]. At the end of the follicular phase, estradiol switches from suppressing release to inducing a sustained surge of release, which induces ovulation [41]. Oocyte meiosis is considered a specialized form of cell division required for the formation of haploid gametes, and the mutation of meiosis-specific cohesion components in female mammals results in an increased frequency of oocyte aneuploidy and premature ovarian failure [42–44]. In addition, the reinitiation of oocyte meiosis is known to play a key role in the follicle phase [45], thus implicating an enrichment of pathways related to follicle development and ovulation rate. Therefore, the GnRH signaling pathway, oocyte meiosis, TGF-beta signaling pathway, Notch signaling pathway and Wnt signaling pathway may be key to further exploring the molecular mechanisms underlying the regulation of goat prolificacy.

Moreover, we also demonstrated interactions among genes related to the prolificacy of Yunshang black goats in the follicular phase, such as the PPI network of *CAMK2G* and *PXN* (Fig. 4). *CAMK2G* and *PXN* are regulatory factors related to the dynamics of focal adhesion assembly and disassembly [46, 47]. Moreover, *CAMK2G* is linked to the oocyte meiosis and GnRH signaling pathways (Table 3), which are involved in ovulation and high yielding [48, 49]. Among the PPI networks of *SYNE1, CDK12*, and *PGS1* (Fig. 4), the ovarian and other cancer-related gene *SYNE1* is located 19 kb downstream of *ESR1*, and it partially contributes to ovarian cancer [50]. In our data, these three interacting genes were differentially expressed in the follicular phase ovaries of Yunshang black goats with different litter sizes, and the same expression trend was observed in ovarian carcinoma [51, 52]. Moreover, *BMP4, SMURF1, TCF7L2* and *WNT4* belong to the TGF-beta signaling pathway and GnRH signaling pathway, and we identified an interaction between *BMP4* and *SMURF1* and between *WNT4* and *TCF7L2* (Fig. 4). Studies have shown that *BMP4* is synthesized by vaginal tissues, and its expression is negatively regulated by estrogen [53, 54]. Thus, the interactions among genes in these pathways affect the ovulation rate, and the interactions of miRNAs and mRNAs in these pathways may provide new insights into the regulation of prolificacy in goats. The above results indicate a role of complex intergene interactions in the prolificacy of Yunshang black goat ovaries.

## Conclusions

In summary, we analyzed the miRNA and transcriptome profiles in follicular phase ovaries from different litters of Yunshang black goats and constructed an interaction network of proteins, miRNAs, mRNAs and signaling pathways involved in the prolificacy trait. These results provide a valuable resource for further research into the molecular mechanism underling the prolificacy in the follicular phase of goat ovary and new insights into the interactions among various factors related to the development and regulation of goat oocytes. Further studies should validate the functions of these key interaction networks and their components at the cellular level in goat prolificacy.

## Materials and methods

### Ethics statement

All the experimental procedures described in the present study were approved by the Science Research Department (in charge of animal welfare issues) of the Institute of Animal Sciences, Chinese Academy of Agricultural Sciences (IAS-CAAS) (Beijing, China). Ethical approval was provided by the animal ethics committee of IASCAAS (No. IAS2019–63).

### Sample collection, RNA extraction and quality analysis

Female native domestic goats, known as Yunshang black goats, were used in this study. Ten goats with no significant differences in age, weight, or height were selected and divided into either the high-yielding group (*n* = 5, average litter size 3.00 ± 0.38, HF group) or the low-yielding group (*n* = 5, litter size 1.32 ± 0.19, LF group) (*P* < 0.05) according to their litter size records. Additionally, all the goats were fed under the same conditions, with free access to water, on a goat farm in Yunnan Province. Ovarian tissues were collected from goats in the follicular phase, frozen immediately in liquid nitrogen and stored at − 80 °C until RNA extraction.

Total RNA for RNA sequencing (RNA-seq) was isolated from 10 ovarian tissue samples (mixed powders of the entire ovary) with the TRIzol reagent (Invitrogen, Carlsbad, CA, United States) according to the manufacturer’s protocol and previous study [55]. The NanoDrop 2000 spectrophotometer (Thermo Scientific, Wilmington, DE, United States) was used to evaluated on the purity and concentration of the RNA samples, and standard denaturing agarose gel electrophoresis was used to monitor degradation and contamination. The Agilent Bioanalyzer 2100 system (Agilent Technologies, Palo Alto, CA, United States) with an RNA Nano 6000 Assay Kit was used to assess the integrity of total RNA.

### mRNA and miRNA library preparation and sequencing

A total of 10 cDNA libraries were constructed from the ovarian tissues of Yunshang black goats from both groups (LF and HF). Three micrograms of RNA from each sample were used as input material for cDNA library and miRNA library construction. First, rRNA was removed with an Epicenter Ribo-ZeroTM rRNA Removal Kit (Epicenter, Madison, WI, United States), and rRNA-free residues were cleaned by ethanol precipitation. Second, following the manufacturer’s recommendations, rRNA-depleted RNA was used to prepare sequencing libraries using the NEBNext® UltraTM Directional RNA Library Prep Kit and Illumina® (NEB, Ipswich, MA, United States). Finally, the products were purified by the AMPure XP system, and an Agilent Bioanalyzer 2100 system was used to assess the library quality. The Illumina Novaseq platform was used to sequence libraries, and 150 bp paired-end reads were generated.

Raw data in fastq format were first processed by in-house scripts. After the removal of reads containing adapters, reads containing poly-N, and low-quality reads from the raw data, the Illumina sequencing raw reads were obtained, among which the number of bases with a quality value Q ≤ 20 was > 50%. The Q20, Q30, and GC contents of the clean data were calculated. Based on the clean data, all high-quality downstream areas were analyzed. Reference genome and gene model annotation files were downloaded directly from the genome website. Bowtie v2.2.3 was used to build the reference genome index, and TopHat v2.0.12 was used to align paired-end clean reads to the reference genome. Both known and novel transcripts from the TopHat alignment results were constructed and identified by the Cufflinks v2.1.1 Reference Annotation Based Transcript (RABT) assembly method. The fragments per kilobase per million reads (FPKM) [56] for each gene were calculated based on the length of the gene and read counts mapped to the gene.

Clean high-quality reads with lengths of 18–35 nt were used in the subsequent analyses. The small RNA tags were mapped to reference sequences in Bowtie21, which were used to search for known miRNAs. miRBase22.0 was used as a reference, and the potential miRNAs and secondary structures were obtained with the modified software mirdeep2 and srna-tools-cli. To remove tags originating from protein-coding genes, repeat sequences, rRNAs, tRNAs, snRNAs, and snoRNAs, small RNA tags were mapped to the Repeat Masker or Rfam databases or data from the specified species itself [57]. The novel miRNAs were predicted by the characteristics of the miRNA precursor hairpin structures. The novel miRNAs were predicted with the software miREvo and mirdeep2 by exploring the secondary structures, Dicer cleavage sites and minimum free energy of the small RNA tags unannotated in the former steps [57, 58].

### Analysis of DE mRNA and miRNA

The expression levels of mRNAs in both libraries constructed from ovaries were estimated based on the FPKM values of the Illumina sequencing data. The fold change of the expression of each mRNA between the HF and LF groups was calculated according to the comparison. Differential expression analysis of both groups was performed with the DESeq R package (1.8.3). Genes with an adjusted *P* < 0.05 and |FC| > 1 identified by DESeq were considered to be differentially expressed. The DEGseq R package was used to analyze the DEMs according to the normalized transcripts per kilobase per million reads (TPM) values. The *q*-valued were adjusted by the *P values*. A *q*-value < 0.01 and |FC| > 1 were set as the thresholds for significant DEMs by default.

### Gene ontology and Kyoto encyclopedia of genes and genomes analyses

The DEGs targeted by miRNAs were annotated and classified by Gene Ontology (GO2) with the GOSeq (Release2.12) software and Kyoto Encyclopedia of Genes and Genomes (KEGG) pathway analysis with the KOBAS (v2.0) software to visualize the data [59–61]. Only GO terms or KEGG pathways with corrected *P* values (*t*-test) < 0.05 were considered to indicate significant enrichment.

### Counter expression miRNA-mRNA analysis

The miRNA and transcriptome profiles of both groups were constructed using ovarian samples from Yunshang black goats. To analyze the interactions between miRNAs and mRNAs, miRNA target genes were predicted by miRanda with psRobot_tar in psRobot [62, 63]. Then, miRNA-mRNA interactions were calculated according to miRNA and transcriptome expression profile data, and negatively correlated miRNA-mRNA pairs were identified using Pearson correlation analysis. Finally, all these analyses were performed using “GeneSymbol” as a unique identifier for all genes/transcripts involved.

### Verification of miRNA and mRNA expression profiles with RT–qPCR

For the RT–qPCR analysis, reverse transcription was performed using a PrimeScript™ RT reagent kit (TaKaRa) and miRcute Plus miRNA First-Strand cDNA Kit (TIANGEN, Beijing, China) for mRNA and miRNA according to the manufacturers’ instructions and previous study [55], respectively. RT–qPCR was performed by a RocheLight Cycler®480 II system (Roche Applied Science, Mannheim, Germany) with SYBR Green qPCR Mix Kit (TaKaRa, Dalian, China) and miRcute Plus miRNA qPCR Kit (TIANGEN, Beijing, China) for mRNAs and miRNAs, respectively. RT–qPCR analysis of mRNA and miRNA expression was conducted by the following procedure: for mRNA, initial denaturation at 95 °C for 5 min, followed by 40 cycles of denaturation at 95 °C for 5 s and annealing at 60 °C for 30 s; for miRNAs, initial denaturation at 95 °C for 15 min, followed by 40 cycles of denaturation at 94 °C for 20 s and annealing at 60 °C for 34 s. The data were analyzed with the 2^*-∆∆Ct*^ method. The goat *PRL19* gene and U6 were used as reference genes for the normalization of the target gene data. The sequences of the RT–qPCR primers are listed in Supplementary Table S6.

### Protein–protein interaction (PPI) network analysis of DEGs

Known and predicted protein–protein interaction analyses of the DEGs were performed using the STRING database (https://string-db.org/). Because the goat database was not provided in STRING, the highly homologous species sheep was used as an alternative to analyze the correlation of DEGs. PPI analysis of DEGs was based on the STRING database4 (Organism: *Ovis aries*). The networks were constructed by extracting the target gene list from the database. Otherwise, the target gene sequences were aligned to the selected reference protein sequences by Blastx (v2.2.28), and then the networks were constructed according to the known interactions of the selected reference species.

### Validation the function of miRNA-mRNA pairs

#### Vector construction

The 3′ UTR of *TXLNA* containing the predicted target site, including wild type (WT) and mutant type (MUT), was cloned into the pmiR-RB-Report vector (isolated using Xho I and Not I (Takara, Dalian, China)). The recombinant vectors were named pmiR-RB-TXLNA-WT and pmiR-RB-TXLNA-MUT. The insert sequences, including the wild-type/mutant sequences, miRNA mimic and mimic-NC, were synthesized by RiboBio company (Guangzhou, China) (the information is shown in Supplemental Fig. S1).

#### Cell culture, transfection and dual luciferase assays

293 T cells, a human renal epithelial cell line, were used to validate the miRNA targets. The cells were seeded into 24-well plates. Cotransfection with 200 ng mRNA-3’UTR-WT target or mRNA-3’UTR-MUT target and 10 μL of miRNA mimic or mimic-NC was performed with Lipofectamine 2000 (Invitrogen, USA). Then, the luciferase activities were measured using the Dual Luciferase Reporter Assay System (Promega, WI, USA) at 48 h post-transfection. The assays were performed in triplicate.

#### Cell counting Kit-8 (CCK-8) assay in granulosa cells (GCs)

Goat GCs were obtained from our laboratory. GCs were inoculated into 96-well plates with approximately 100 μL of cell suspension per well in 3 replicates. The cells were incubated for 2–4 h at 37 °C in an incubator, and after cell appositioning, subsequent experiments were conducted. The cell proliferation assay was performed with the CCK-8 method (Beyotime, Beijing, China). Goat GCs were cultured in 96-well plates and assessed by adding 10 μL CCK-8 per well following the protocol. After the addition of the CCK-8 solution, the absorbance at 450 nm was measured at 0 h, 6 h, 12 h, 24 h, 48 h and 72 h to assess cell proliferation.

### Statistical analysis

The GraphPad Prism (version 5.0) software (San Diego, CA, United States) was used to statistical analyses of the RT–qPCR results and graphs. The statistical significance of the data was tested by performing paired *t*-tests [64]. The results are presented as the means ± SEMs of three replicates, and statistical significance was represented by *P* values (*P < 0.05; **P < 0.01).

## Supplementary Information

**Additional file 1: Table S1.** The information of RNA-seq data.

**Additional file 2: Table S2.** All of the DE mRNAs in the comparison.

**Additional file 3: Table S3.** The information of DE mRNAs in the comparison.

**Additional file 4: Table S4.** The information of miRNA sequencing data.

**Additional file 5: Table S5.** All of the expression miRNAs in the comparison.

**Additional file 6: Table S6.** Primer sequences used for qPCR in this study.

**Additional file 7: Fig. S1.** The sequence of miRNA-mRNA pair for Dual Luciferase Reporter Assay.

## Data Availability

All data generated and analyzed during this study are included in this published article and its supplementary information files. The raw data for this study can be found in BioProject ID PRJNA731513 on NCBI. The URL is https://www.ncbi.nlm.nih.gov/sra/PRJNA731513, accessed date: 23 May 2021.

## Abbreviations

GO: Gene Ontology
KEGG: Kyoto Encyclopedia of Genes and Genomes
PPI: Protein–protein interactions
DE: Differentially express
GC: Granulose cellular
PCOS: Polycystic ovary syndrome
BMPR1B: Bone morphogenetic protein receptor type 1B
GDF9: Growth differentiation Factor 9
IGF: Insulin-like growth factor
ATM: Ataxia telangiectasia mutated
FOXL2: Forkhead Box L2
TAGLN2: Transgelin 2
MAP 3 K3: Mitogen-activated protein kinase kinase kinase 3
ERK: Extracellular signal-regulated kinase
DEGs: Differentially expressed genes
DEMs: Differentially expressed miRNAs
LH: Luteinizing hormone
RABT: Reference Annotation Based Transcript
FPKM: Fragments per kilobase per million reads
FC: Fold change
TPM: Transcripts per kilobase per million reads
