# Supplementary material for: Integrated analyses of miRNA-mRNA expression profiles of ovaries reveal the crucial interaction networks that regulate the prolificacy of goats in the follicular phase
Source: BMC Genomics. 2021 Nov 11;22:812. doi: 10.1186/s12864-021-08156-2 (PMC8582148; doi:10.1186/s12864-021-08156-2)
Supplement: Supplementary file 1 — Additional file 1: Table S1. The information of RNA-seq data. [file 12864_2021_8156_MOESM1_ESM.pdf]

Table S1 The information of RNA-seq data

Sample name	Raw reads	Raw bases	Clean reads	Clean bases	Error rate(%)	Q20(%)	Q30(%)	GC content(%)	Total mapped
HF-1	110059570	16.5G	109570172	16.4G	0.03	98.01	94.18	51.65	105954528 (96.7%)
HF-2	149168582	22.4G	148187868	22.2G	0.03	97.71	93.59	48.89	142902849 (96.43%)
HF-3	165256564	24.8G	164219488	24.6G	0.03	97.61	93.34	48.84	158462267 (96.49%)
HF-4	100001136	15G	99578072	14.9G	0.02	98.27	94.85	52.72	96294943 (96.7%)
HF-5	100800604	15.1G	100348242	15.1G	0.03	97.96	94.05	49.66	96908787 (96.57%)
LF-1	102585116	15.4G	102208602	15.3G	0.02	98.53	95	46.21	99264769 (97.12%)
LF-2	129258642	19.4G	128736444	19.3G	0.02	98.24	94.7	47.61	119602467 (92.9%)
LF-3	103312740	15.5G	102912346	15.4G	0.02	98.58	95.26	50.49	100075443 (97.24%)
LF-4	125008336	18.8G	124494838	18.7G	0.02	98.42	95.18	50.9	120693801 (96.95%)
LF-5	104121182	15.6G	103708734	15.6G	0.02	98.44	94.93	50.16	100662136 (97.06%)
