# Supplementary material for: Integrated analyses of miRNA-mRNA expression profiles of ovaries reveal the crucial interaction networks that regulate the prolificacy of goats in the follicular phase
Source: BMC Genomics. 2021 Nov 11;22:812. doi: 10.1186/s12864-021-08156-2 (PMC8582148; doi:10.1186/s12864-021-08156-2)
Supplement: Supplementary file 3 — Additional file 3: Table S3. The information of DE mRNAs in the comparison. [file 12864_2021_8156_MOESM3_ESM.pdf]

Table S3 The information of DE mRNAs in the comparison

transcript_id	gene_id	gene_name	feature_id	LF_FPKM	HF_FPKM	log2(foldchange)	pvalue	qvalue
Novel_023053	XLOC_235835	.	transcript	0	0.307403	19.88530359	5.49E-11	3.38E-09
Novel_023047	XLOC_235834	.	transcript	0	0.14309	20.88216528	5.46E-12	3.91E-10
Novel_023003	XLOC_235042	.	transcript	0	0.129207	22.3754033	1.46E-13	2.08E-11
Novel_022972	XLOC_234403	.	transcript	0	6.094138	13.10945925	1.90E-07	1.14E-05
Novel_022961	XLOC_234278	.	transcript	0	0.177229	20.69014275	8.58E-12	5.95E-10
Novel_022911	XLOC_233558	.	transcript	0	0.149964	23.77747655	3.96E-15	1.56E-12
Novel_022884	XLOC_233097	.	transcript	6.954974	0	-13.2645695	1.08E-05	0.000611
Novel_022871	XLOC_232979	.	transcript	1.934904	0	-24.49253828	5.86E-16	3.89E-13
Novel_022835	XLOC_232793	.	transcript	0	0.059379	19.88530359	5.49E-11	3.38E-09
Novel_022784	XLOC_232244	.	transcript	0.258217	0	-24.14106835	1.51E-15	8.07E-13
Novel_022755	XLOC_231835	.	transcript	0.320342	0	-23.53503325	7.56E-15	2.47E-12
Novel_022732	XLOC_231642	.	transcript	0.000709	0.170582	6.942953304	4.70E-06	0.000271
Novel_022681	XLOC_231217	.	transcript	0	0.17791	21.89680462	4.74E-13	4.97E-11
Novel_022670	XLOC_231155	.	transcript	0.101976	0	-8.195352867	0.000582	0.025568
Novel_022627	XLOC_230759	.	transcript	0.081333	0	-6.825638832	0.000158	0.007838
Novel_022587	XLOC_230535	.	transcript	1.421109	5.718053	1.955129449	4.22E-05	0.002264
Novel_022586	XLOC_230535	.	transcript	0	2.855964	26.92653459	5.70E-19	1.97E-15
Novel_022560	XLOC_230320	.	transcript	0	0.160525	21.38883936	1.63E-12	1.35E-10
Novel_022507	XLOC_230122	.	transcript	0.464027	0	-24.29024755	1.01E-15	6.00E-13
Novel_022483	XLOC_230037	.	transcript	0.183034	0	-25.36242636	5.28E-17	6.71E-14
Novel_022421	XLOC_229446	.	transcript	0.008486	1.364406	7.096623373	1.16E-07	6.99E-06
Novel_022392	XLOC_229211	.	transcript	0	0.091248	20.60383762	1.05E-11	7.11E-10
Novel_022360	XLOC_228980	.	transcript	0.151077	0	-22.09842173	2.97E-13	3.47E-11
Novel_022348	XLOC_228954	.	transcript	0	0.266034	21.93409821	4.34E-13	4.61E-11
Novel_022346	XLOC_228940	.	transcript	0.169167	0.258775	0.577293912	0.000458	0.020636
Novel_022320	XLOC_228719	.	transcript	0.279935	0	-22.66213943	7.10E-14	1.26E-11
Novel_022299	XLOC_228653	.	transcript	0	0.228859	20.83098338	6.16E-12	4.37E-10
Novel_022290	XLOC_228652	.	transcript	0	0.381471	21.3679908	1.72E-12	1.41E-10
Novel_022283	XLOC_228648	.	transcript	0.061587	0	-22.00421936	3.75E-13	4.10E-11
Novel_022278	XLOC_228621	.	transcript	0	0.148857	6.614693979	6.40E-05	0.003382
Novel_022272	XLOC_228587	.	transcript	0.298208	0.005577	-5.549008594	0.000339	0.01588
Novel_022248	XLOC_228415	.	transcript	0	0.081489	22.09276698	2.94E-13	3.45E-11
Novel_022185	XLOC_227828	.	transcript	0	0.042011	20.0094184	4.15E-11	2.58E-09
Novel_022176	XLOC_227824	.	transcript	0.164773	0	-21.15738604	2.95E-12	2.23E-10
Novel_022170	XLOC_227769	.	transcript	0	0.037383	20.13322413	3.12E-11	1.98E-09
Novel_022066	XLOC_226650	.	transcript	0	0.019912	21.29716036	2.04E-12	1.62E-10

Novel_022050	XLOC_226478	.	transcript	0	0.389306	23.78993176	1.94E-18	5.78E-15
Novel_022014	XLOC_225713	.	transcript	0.037969	0	-21.78261445	6.48E-13	6.26E-11
Novel_021958	XLOC_225056	.	transcript	0.051886	0	-21.40284138	1.64E-12	1.35E-10
Novel_021950	XLOC_225017	.	transcript	0.095793	0	-21.98020946	3.98E-13	4.31E-11
Novel_021930	XLOC_224601	.	transcript	0	0.264002	6.086813783	0.00102	0.042133
Novel_021927	XLOC_224575	.	transcript	0.023383	0.251044	3.240816307	0.000378	0.017495
Novel_021910	XLOC_224388	.	transcript	0.112271	0.008057	-3.734919038	0.000337	0.01581
Novel_021900	XLOC_224383	.	transcript	0.06416	0	-24.61886402	4.15E-16	3.04E-13
Novel_021887	XLOC_224286	.	transcript	0.047214	0	-8.795837271	0.000281	0.013393
Novel_021865	XLOC_223252	.	transcript	0.097175	0.279057	1.455575714	0.000212	0.010324
Novel_021796	XLOC_221490	.	transcript	0.020723	0	-20.64363503	9.54E-12	6.54E-10
Novel_021780	XLOC_221422	.	transcript	0	0.127669	20.97869984	4.35E-12	3.18E-10
Novel_021716	XLOC_220942	.	transcript	0	0.13464	23.98803227	2.27E-15	1.05E-12
Novel_021709	XLOC_220874	.	transcript	0.346979	0	-24.58352986	4.57E-16	3.21E-13
Novel_021688	XLOC_220673	.	transcript	0.089371	0	-21.35441181	1.85E-12	1.49E-10
Novel_021661	XLOC_220212	.	transcript	0.028326	0.26815	3.072074882	0.000234	0.011333
Novel_021646	XLOC_219978	.	transcript	0	0.103805	7.817453663	0.001196	0.048614
Novel_021643	XLOC_219950	.	transcript	0	0.352631	22.48967989	1.09E-13	1.68E-11
Novel_021596	XLOC_218806	.	transcript	0	0.164661	22.65763034	7.16E-14	1.27E-11
Novel_021595	XLOC_218776	.	transcript	0.105181	0	-22.69455726	6.55E-14	1.19E-11
Novel_021590	XLOC_218703	.	transcript	0.190554	0	-21.32317892	1.99E-12	1.59E-10
Novel_021540	XLOC_217972	.	transcript	0.001103	0.147809	6.57069743	0.000538	0.023869
Novel_021537	XLOC_217885	.	transcript	0	0.172983	20.99507951	4.18E-12	3.08E-10
Novel_021529	XLOC_217779	.	transcript	0.07737	0	-22.57565363	8.95E-14	1.48E-11
Novel_021516	XLOC_217732	.	transcript	0.086377	0	-21.74566367	7.10E-13	6.75E-11
Novel_021505	XLOC_217696	.	transcript	0	0.065396	21.48899171	1.28E-12	1.09E-10
Novel_021498	XLOC_217690	.	transcript	0	0.169542	20.72178649	7.96E-12	5.55E-10
Novel_021490	XLOC_217619	.	transcript	0.173134	0	-23.17169531	1.95E-14	5.14E-12
Novel_021435	XLOC_217478	.	transcript	0	0.048413	19.97878667	4.45E-11	2.76E-09
Novel_021431	XLOC_217472	.	transcript	0.265898	0	-23.78995762	3.86E-15	1.55E-12
Novel_021358	XLOC_216919	.	transcript	0	0.150917	21.5140817	1.21E-12	1.04E-10
Novel_021356	XLOC_216892	.	transcript	0.278957	69.09613	7.889793292	0.000119	0.006038
Novel_021324	XLOC_216379	.	transcript	0	0.07709	18.62179437	8.16E-10	4.96E-08
Novel_021307	XLOC_216223	.	transcript	0.10286	0	-21.74471299	7.12E-13	6.75E-11
Novel_021298	XLOC_216210	.	transcript	0.652521	0	-23.7166134	4.69E-15	1.75E-12
Novel_021261	XLOC_215940	.	transcript	0	0.02946	20.70563032	8.27E-12	5.75E-10
Novel_021230	XLOC_215813	.	transcript	0.581945	2.455703	2.071114958	0.000726	0.031165
Novel_021209	XLOC_215605	.	transcript	0.056935	0	-21.62084	9.64E-13	8.70E-11

Novel_021204	XLOC_215581	.	transcript	0.001986	0.166751	5.706625728	4.33E-06	0.000249
Novel_021189	XLOC_215527	.	transcript	0.665145	0	-22.90113023	3.91E-14	8.12E-12
Novel_021153	XLOC_215345	.	transcript	0	0.052307	20.02608322	3.99E-11	2.48E-09
Novel_021154	XLOC_215345	.	transcript	0	0.090778	21.0126772	4.01E-12	2.97E-10
Novel_021137	XLOC_215323	.	transcript	0	6.189204	27.62186427	7.01E-20	4.66E-16
Novel_021122	XLOC_215287	.	transcript	0	0.098149	22.92652763	3.61E-14	7.73E-12
Novel_021095	XLOC_215167	.	transcript	0.11181	0	-8.101895028	0.000724	0.031076
Novel_021023	XLOC_215083	.	transcript	0	0.237227	22.62351888	7.80E-14	1.33E-11
Novel_020980	XLOC_214513	.	transcript	0.118623	0	-5.674172313	0.000144	0.007212
Novel_020939	XLOC_213338	.	transcript	0	0.089599	20.38552664	1.74E-11	1.15E-09
Novel_020934	XLOC_213236	.	transcript	0	0.041491	21.94660927	4.21E-13	4.50E-11
Novel_020923	XLOC_213114	.	transcript	0.046111	0	-23.04193217	2.72E-14	6.40E-12
Novel_020818	XLOC_211788	.	transcript	0.107847	0	-22.20267045	2.29E-13	2.90E-11
Novel_020761	XLOC_211245	.	transcript	0.03881	0.000588	-5.759019756	0.00058	0.025469
Novel_020749	XLOC_211146	.	transcript	0.206841	0	-22.89322013	3.99E-14	8.25E-12
Novel_020731	XLOC_210850	.	transcript	0.038399	0.23108	2.501727085	0.000316	0.014894
Novel_020595	XLOC_208555	.	transcript	0	0.060487	20.14497434	3.04E-11	1.93E-09
Novel_020590	XLOC_208538	.	transcript	0.294547	0	-24.79586702	2.55E-16	2.16E-13
Novel_020499	XLOC_207240	.	transcript	0	0.069557	19.30768224	1.85E-10	1.13E-08
Novel_020465	XLOC_206604	.	transcript	0	0.330823	8.737165313	0.000383	0.017668
Novel_020460	XLOC_206594	.	transcript	0.021223	0	-21.42982929	1.54E-12	1.28E-10
Novel_020446	XLOC_206325	.	transcript	0	0.042246	20.47399141	1.40E-11	9.28E-10
Novel_020402	XLOC_205566	.	transcript	0	0.194255	20.51885639	1.28E-11	8.51E-10
Novel_020371	XLOC_205186	.	transcript	0	0.101983	21.09807008	3.28E-12	2.47E-10
Novel_020368	XLOC_205097	.	transcript	0.270457	0	-22.63583061	7.69E-14	1.32E-11
Novel_020295	XLOC_203882	.	transcript	0.193856	0	-24.12535759	1.58E-15	8.37E-13
Novel_020277	XLOC_203739	.	transcript	1.323125	0	-23.82670142	3.50E-15	1.45E-12
Novel_020267	XLOC_203681	.	transcript	0.25396	0.019949	-3.659035008	0.000286	0.013627
Novel_020194	XLOC_202860	.	transcript	0	0.150932	22.50367114	1.05E-13	1.64E-11
Novel_020166	XLOC_202613	.	transcript	0.057786	0	-21.60127593	1.01E-12	9.02E-11
Novel_020122	XLOC_201963	.	transcript	0.059153	0	-22.14306274	2.65E-13	3.22E-11
Novel_020102	XLOC_201781	.	transcript	0.065031	0	-7.903364494	0.001164	0.047427
Novel_020079	XLOC_201708	.	transcript	0.052463	0	-21.74243585	7.16E-13	6.76E-11
Novel_020028	XLOC_201296	.	transcript	1.694654	0	-24.42249499	7.08E-16	4.57E-13
Novel_019936	XLOC_200021	.	transcript	0.216982	1.90E-05	-7.978964918	0.000522	0.023225
Novel_019928	XLOC_199956	.	transcript	0.129479	0	-23.67090093	5.29E-15	1.93E-12
Novel_019906	XLOC_199902	.	transcript	0.149061	0	-7.142698333	3.99E-05	0.002151
Novel_019836	XLOC_199308	.	transcript	0	0.102372	22.25117307	1.98E-13	2.61E-11

Novel_019783	XLOC_198847	.	transcript	0.144456	0	-21.55416235	1.14E-12	9.86E-11
Novel_019771	XLOC_198745	.	transcript	0	0.115324	20.68126768	8.76E-12	6.05E-10
Novel_019761	XLOC_198636	.	transcript	0.117049	0	-22.11100372	2.87E-13	3.42E-11
Novel_019738	XLOC_198395	.	transcript	0.254267	0	-22.23032754	2.12E-13	2.74E-11
Novel_019740	XLOC_198395	.	transcript	0	0.319398	20.32985427	1.89E-11	1.24E-09
Novel_019696	XLOC_198325	.	transcript	0.007157	0.269524	4.723132861	2.81E-05	0.001532
Novel_019661	XLOC_198193	.	transcript	0	0.040079	21.00467869	4.09E-12	3.02E-10
Novel_019615	XLOC_197829	.	transcript	0.097424	0	-7.546780551	1.84E-05	0.001023
Novel_019559	XLOC_197508	.	transcript	0	0.193934	9.115498542	6.37E-08	3.83E-06
Novel_019542	XLOC_197301	.	transcript	0	0.281643	22.00567035	3.64E-13	4.02E-11
Novel_019517	XLOC_197279	.	transcript	0.209345	0.000244	-8.923382139	7.55E-05	0.003942
Novel_019522	XLOC_197279	.	transcript	0	0.232195	20.55894054	1.17E-11	7.82E-10
Novel_019526	XLOC_197279	.	transcript	0	0.446967	21.91586433	4.49E-13	4.74E-11
Novel_019501	XLOC_197242	.	transcript	0.105507	0	-21.62005021	9.66E-13	8.70E-11
Novel_019490	XLOC_197239	.	transcript	0.170067	0	-21.55416235	1.14E-12	9.86E-11
Novel_019424	XLOC_196675	.	transcript	0.466626	0	-23.07510083	2.46E-14	5.92E-12
Novel_019332	XLOC_195720	.	transcript	0.062611	0	-20.64363503	9.54E-12	6.54E-10
Novel_019304	XLOC_195413	.	transcript	0.057446	0	-22.11124254	2.87E-13	3.42E-11
Novel_019270	XLOC_195149	.	transcript	0.049925	0	-22.52203322	1.03E-13	1.61E-11
Novel_019271	XLOC_195149	.	transcript	0	0.101865	22.37905879	1.44E-13	2.07E-11
Novel_019264	XLOC_195119	.	transcript	0	0.177754	22.82269454	4.70E-14	9.21E-12
Novel_019259	XLOC_194989	.	transcript	0	0.616586	8.905925752	1.41E-07	8.45E-06
Novel_019246	XLOC_194968	.	transcript	0	0.030523	19.21345211	2.30E-10	1.41E-08
Novel_019194	XLOC_194146	.	transcript	0.218413	0	-8.566867042	0.000362	0.016781
Novel_019176	XLOC_193928	.	transcript	0.080117	0	-22.08664807	3.05E-13	3.55E-11
Novel_019177	XLOC_193928	.	transcript	0.00242	0.20767	6.155201036	4.87E-10	2.97E-08
Novel_019158	XLOC_193629	.	transcript	0	0.028293	21.32432188	1.91E-12	1.53E-10
Novel_019145	XLOC_193571	.	transcript	0.099145	0	-22.72052042	6.20E-14	1.14E-11
Novel_019146	XLOC_193571	.	transcript	0	0.027472	20.05380525	3.75E-11	2.34E-09
Novel_019049	XLOC_192507	.	transcript	0	0.290484	22.2224207	2.13E-13	2.75E-11
Novel_019038	XLOC_192463	.	transcript	0	0.539678	23.62677036	5.89E-15	2.08E-12
Novel_019022	XLOC_192401	.	transcript	0.033286	0	-22.11224546	2.84E-13	3.40E-11
Novel_018992	XLOC_192062	.	transcript	0.091663	0.002487	-5.187845924	1.78E-05	0.000989
Novel_018987	XLOC_192010	.	transcript	0	0.059978	21.01292304	4.01E-12	2.97E-10
Novel_018980	XLOC_191943	.	transcript	0.052059	0	-22.05121544	3.34E-13	3.79E-11
Novel_018946	XLOC_191476	.	transcript	0	0.026791	6.931583564	0.000132	0.006683
Novel_018938	XLOC_191439	.	transcript	0.212674	0	-25.97929552	9.13E-18	1.88E-14
Novel_018937	XLOC_191439	.	transcript	0.147492	0	-25.27015371	6.84E-17	8.09E-14

Novel_018914	XLOC_191270	.	transcript	0	0.15239	20.63861079	9.68E-12	6.62E-10
Novel_018904	XLOC_191169	.	transcript	0.054804	0	-21.4785782	1.36E-12	1.16E-10
Novel_018902	XLOC_191169	.	transcript	0	0.06054	20.53555813	1.23E-11	8.22E-10
Novel_018893	XLOC_191154	.	transcript	0	0.169724	7.302977245	0.00033	0.015526
Novel_018840	XLOC_190751	.	transcript	0.042034	0	-21.29699898	2.12E-12	1.67E-10
Novel_018809	XLOC_190598	.	transcript	0.081949	0	-21.68027171	8.34E-13	7.69E-11
Novel_018798	XLOC_190507	.	transcript	0.046389	0	-20.65638042	9.33E-12	6.42E-10
Novel_018797	XLOC_190507	.	transcript	0.262271	0	-9.115142918	0.000156	0.007775
Novel_018775	XLOC_190316	.	transcript	0.167825	0	-22.62457183	7.91E-14	1.34E-11
Novel_018774	XLOC_190316	.	transcript	0.077904	0	-20.62213642	1.02E-11	6.92E-10
Novel_018772	XLOC_190305	.	transcript	0.704796	0	-24.41056785	7.30E-16	4.64E-13
Novel_018752	XLOC_190240	.	transcript	0	0.115177	21.30175441	2.01E-12	1.61E-10
Novel_018714	XLOC_189836	.	transcript	0	0.035301	20.30749451	2.08E-11	1.35E-09
Novel_018705	XLOC_189729	.	transcript	0.036599	0	-21.82059253	5.90E-13	5.87E-11
Novel_018692	XLOC_189689	.	transcript	0	0.106384	22.19930533	2.25E-13	2.86E-11
Novel_018675	XLOC_189564	.	transcript	0	0.066058	20.89908715	5.25E-12	3.76E-10
Novel_018671	XLOC_189551	.	transcript	0	0.102229	6.44587721	0.000105	0.005393
Novel_018655	XLOC_189404	.	transcript	0	0.171684	22.47933988	1.12E-13	1.71E-11
Novel_018618	XLOC_188839	.	transcript	0.529151	0	-24.08983418	1.73E-15	8.76E-13
Novel_018511	XLOC_188048	.	transcript	0	0.167161	21.76293764	6.60E-13	6.36E-11
Novel_018476	XLOC_187849	.	transcript	0.023325	0	-22.92434902	3.69E-14	7.80E-12
Novel_018466	XLOC_187552	.	transcript	0	0.091128	21.29148684	2.06E-12	1.63E-10
Novel_018461	XLOC_187496	.	transcript	0	0.092749	8.618006198	9.21E-07	5.43E-05
Novel_018428	XLOC_187443	.	transcript	0.226197	0.725225	1.653407276	3.74E-05	0.002024
Novel_018402	XLOC_187377	.	transcript	0.025893	0	-22.10579611	2.91E-13	3.45E-11
Novel_018406	XLOC_187377	.	transcript	0.000675	0.150208	7.597816364	0.000322	0.01519
Novel_018404	XLOC_187377	.	transcript	0	0.036667	21.58123972	1.03E-12	9.14E-11
Novel_018383	XLOC_187255	.	transcript	0.033571	0	-23.29421457	1.42E-14	3.97E-12
Novel_018371	XLOC_187255	.	transcript	0.021727	0	-23.01248269	2.94E-14	6.56E-12
Novel_018359	XLOC_187255	.	transcript	0.025458	0	-22.68913148	6.72E-14	1.21E-11
Novel_018378	XLOC_187255	.	transcript	0	0.054145	9.109483206	1.64E-07	9.85E-06
Novel_018381	XLOC_187255	.	transcript	0	0.092179	9.921647888	3.74E-05	0.002023
Novel_018345	XLOC_187255	.	transcript	0	0.005223	19.9875996	4.36E-11	2.71E-09
Novel_018339	XLOC_187255	.	transcript	0	0.004377	20.68662192	8.65E-12	5.98E-10
Novel_018355	XLOC_187255	.	transcript	0	0.01897	21.29716036	2.04E-12	1.62E-10
Novel_018385	XLOC_187255	.	transcript	0	0.039457	22.20671955	2.22E-13	2.82E-11
Novel_018372	XLOC_187255	.	transcript	0	0.022619	22.26408154	1.92E-13	2.53E-11
Novel_018354	XLOC_187255	.	transcript	0	0.039777	22.38696317	1.40E-13	2.04E-11

Novel_018380	XLOC_187255	.	transcript	0	0.039222	22.91931073	3.68E-14	7.80E-12
Novel_018363	XLOC_187255	.	transcript	0	0.048364	23.15355123	2.02E-14	5.21E-12
Novel_018330	XLOC_187255	.	transcript	0	0.057737	23.2187637	1.70E-14	4.62E-12
Novel_018310	XLOC_187029	.	transcript	0.076588	0	-22.54274044	9.74E-14	1.57E-11
Novel_018301	XLOC_187020	.	transcript	0	0.14323	22.24929823	1.99E-13	2.62E-11
Novel_018249	XLOC_186657	.	transcript	0.043847	0	-24.09126182	1.73E-15	8.76E-13
Novel_018267	XLOC_186657	.	transcript	0.033239	0	-22.49640213	1.09E-13	1.67E-11
Novel_018250	XLOC_186657	.	transcript	0	0.014671	21.67741468	8.12E-13	7.52E-11
Novel_018212	XLOC_185849	.	transcript	0.105041	0	-21.82376466	5.86E-13	5.84E-11
Novel_018195	XLOC_185612	.	transcript	0	0.079974	20.09400811	3.42E-11	2.15E-09
Novel_018132	XLOC_185124	.	transcript	0	0.173698	22.03516384	3.39E-13	3.83E-11
Novel_018049	XLOC_184641	.	transcript	0	0.336247	21.38542556	1.60E-12	1.33E-10
Novel_018045	XLOC_184617	.	transcript	0	0.066921	20.74266489	7.58E-12	5.29E-10
Novel_017950	XLOC_183504	.	transcript	0	0.142194	21.95499207	4.13E-13	4.43E-11
Novel_017926	XLOC_183318	.	transcript	0	0.072497	21.76293764	6.60E-13	6.36E-11
Novel_017915	XLOC_183310	.	transcript	0	0.040417	20.12153158	3.21E-11	2.03E-09
Novel_017916	XLOC_183310	.	transcript	0	0.095681	21.14133367	2.95E-12	2.23E-10
Novel_017867	XLOC_182705	.	transcript	0.072805	0	-22.02813841	3.53E-13	3.97E-11
Novel_017820	XLOC_182359	.	transcript	2.697424	0	-23.13942519	2.09E-14	5.35E-12
Novel_017819	XLOC_182358	.	transcript	4.823175	0	-24.93969865	1.72E-16	1.72E-13
Novel_017796	XLOC_182242	.	transcript	0.137539	0	-23.01610328	2.91E-14	6.56E-12
Novel_017751	XLOC_182010	.	transcript	0.435133	0	-10.57972885	0.000473	0.021249
Novel_017731	XLOC_181529	.	transcript	0	0.038836	20.64356897	9.57E-12	6.55E-10
Novel_017718	XLOC_180698	.	transcript	0.312053	0	-24.82335948	2.36E-16	2.03E-13
Novel_017712	XLOC_180698	.	transcript	0	0.030431	21.96489874	4.03E-13	4.35E-11
Novel_017717	XLOC_180698	.	transcript	0	0.067186	22.30754983	1.72E-13	2.35E-11
Novel_017615	XLOC_180320	.	transcript	0.073016	0	-6.202076968	0.000827	0.034966
Novel_017608	XLOC_180198	.	transcript	0.152842	0	-22.887957	4.05E-14	8.34E-12
Novel_017607	XLOC_180198	.	transcript	0.124085	0	-22.61317268	8.15E-14	1.36E-11
Novel_017602	XLOC_180189	.	transcript	0	0.064896	22.16140204	2.48E-13	3.09E-11
Novel_017580	XLOC_179630	.	transcript	7.367697	0.585264	-3.753714754	0.000768	0.032782
Novel_017523	XLOC_179391	.	transcript	0.34252	0.027024	-3.603110426	4.96E-11	3.06E-09
Novel_017519	XLOC_179388	.	transcript	0	0.179202	21.90755982	4.64E-13	4.87E-11
Novel_017512	XLOC_179355	.	transcript	0.081419	0	-22.72150464	6.19E-14	1.14E-11
Novel_017508	XLOC_179355	.	transcript	0.33537	0	-22.64157225	7.58E-14	1.32E-11
Novel_017504	XLOC_179355	.	transcript	0.18669	0	-21.79364918	6.31E-13	6.14E-11
Novel_017462	XLOC_178816	.	transcript	0.127835	0	-22.92669309	3.65E-14	7.77E-12
Novel_017451	XLOC_178485	.	transcript	0	0.059772	20.08195542	3.51E-11	2.21E-09

Novel_017401	XLOC_177829	.	transcript	0.043419	0	-21.27093062	2.21E-12	1.73E-10
Novel_017352	XLOC_177443	.	transcript	0	0.117423	22.06830137	3.10E-13	3.60E-11
Novel_017329	XLOC_177261	.	transcript	0.149002	0	-23.56931361	6.84E-15	2.32E-12
Novel_017331	XLOC_177261	.	transcript	0	0.404754	9.844647632	0.000126	0.006395
Novel_017299	XLOC_176698	.	transcript	0	0.366797	23.30237104	1.37E-14	3.85E-12
Novel_017298	XLOC_176651	.	transcript	0.099694	0	-22.5186415	1.03E-13	1.62E-11
Novel_017296	XLOC_176651	.	transcript	0	0.163944	22.53168909	9.83E-14	1.58E-11
Novel_017281	XLOC_176568	.	transcript	1.348038	0	-23.45859079	9.24E-15	2.87E-12
Novel_017276	XLOC_176531	.	transcript	11.61116	0.153353	-6.380836578	5.00E-05	0.002672
Novel_017270	XLOC_176518	.	transcript	57.62935	0	-30	3.64E-23	5.24E-19
Novel_017273	XLOC_176518	.	transcript	3.222033	0.09468	-5.142077059	0.000107	0.005471
Novel_017239	XLOC_176233	.	transcript	0.031253	0	-23.78835194	3.88E-15	1.55E-12
Novel_017233	XLOC_176233	.	transcript	0.041836	0	-22.72894609	6.07E-14	1.13E-11
Novel_017234	XLOC_176233	.	transcript	0.158214	0	-10.43926686	1.72E-08	1.04E-06
Novel_017220	XLOC_176115	.	transcript	0.158286	0	-21.8058633	6.12E-13	6.02E-11
Novel_017207	XLOC_175986	.	transcript	0	0.107948	21.80394741	5.97E-13	5.91E-11
Novel_017201	XLOC_175918	.	transcript	0.00301	0.120258	5.140917713	6.12E-06	0.000351
Novel_017176	XLOC_175807	.	transcript	0.005456	0.133889	4.637663436	0.000343	0.015999
Novel_017084	XLOC_174915	.	transcript	0.060401	0	-22.29854725	1.79E-13	2.42E-11
Novel_017086	XLOC_174915	.	transcript	0	0.190647	9.325297845	0.000144	0.007186
Novel_017081	XLOC_174915	.	transcript	0	0.05366	21.06159815	3.57E-12	2.68E-10
Novel_016981	XLOC_174168	.	transcript	0.056709	0	-22.37812327	1.47E-13	2.09E-11
Novel_016975	XLOC_174069	.	transcript	0.017507	0.206567	3.280635072	7.72E-05	0.004021
Novel_016964	XLOC_174062	.	transcript	0.139853	0	-24.0314637	2.03E-15	9.79E-13
Novel_016968	XLOC_174062	.	transcript	0.058629	0	-22.91366425	3.79E-14	7.95E-12
Novel_016951	XLOC_174062	.	transcript	0.062438	0	-22.73887292	5.92E-14	1.10E-11
Novel_016955	XLOC_174062	.	transcript	0.050187	0	-22.29109622	1.83E-13	2.46E-11
Novel_016952	XLOC_174062	.	transcript	0	0.237679	23.50697683	8.05E-15	2.60E-12
Novel_016936	XLOC_173839	.	transcript	0	0.11484	8.513862674	1.07E-06	6.28E-05
Novel_016940	XLOC_173839	.	transcript	0	0.059953	20.53555813	1.23E-11	8.22E-10
Novel_016899	XLOC_173458	.	transcript	0	0.111278	21.97230467	3.95E-13	4.29E-11
Novel_016733	XLOC_169973	.	transcript	0	0.02506	21.0117197	4.02E-12	2.97E-10
Novel_016728	XLOC_169961	.	transcript	0	0.389902	20.6001563	1.06E-11	7.12E-10
Novel_016715	XLOC_169782	.	transcript	0.14306	0	-22.76502964	5.54E-14	1.05E-11
Novel_016697	XLOC_169673	.	transcript	0.287833	0	-25.93637271	1.03E-17	1.98E-14
Novel_016665	XLOC_168881	.	transcript	0.242927	0	-22.7778478	5.36E-14	1.03E-11
Novel_016661	XLOC_168881	.	transcript	0	0.351246	22.30772782	1.72E-13	2.35E-11
Novel_016599	XLOC_167981	.	transcript	0.245703	1.44E-05	-10.63745665	0.000136	0.006859

Novel_016567	XLOC_167409	.	transcript	0.003053	0.195781	5.707308986	0.000655	0.028474
Novel_016467	XLOC_166701	.	transcript	0	0.136276	21.01292304	4.01E-12	2.97E-10
Novel_016456	XLOC_166634	.	transcript	0	0.049773	21.06360456	3.55E-12	2.67E-10
Novel_016435	XLOC_166523	.	transcript	0.066057	0	-22.07359764	3.15E-13	3.65E-11
Novel_016407	XLOC_166352	.	transcript	0.418174	0.008769	-5.554538653	0.000358	0.016584
Novel_016399	XLOC_166329	.	transcript	0	0.070065	20.98334394	4.30E-12	3.15E-10
Novel_016353	XLOC_166055	.	transcript	0	0.19913	21.78766751	6.22E-13	6.08E-11
Novel_016305	XLOC_165837	.	transcript	0.174348	0.003723	-5.292618697	5.04E-05	0.002694
Novel_016289	XLOC_165771	.	transcript	0.105953	0	-21.72861404	7.40E-13	6.94E-11
Novel_016286	XLOC_165767	.	transcript	0.333858	0.012185	-4.868455123	0.000271	0.012962
Novel_016207	XLOC_165133	.	transcript	0	0.214692	20.89908715	5.25E-12	3.76E-10
Novel_016206	XLOC_165108	.	transcript	0.152143	0	-7.105319196	0.000481	0.02152
Novel_016134	XLOC_164956	.	transcript	0.064215	0	-21.62084	9.64E-13	8.70E-11
Novel_016080	XLOC_164424	.	transcript	0.075903	0	-22.13885153	2.68E-13	3.25E-11
Novel_016066	XLOC_164337	.	transcript	0.052216	0	-22.45317447	1.21E-13	1.83E-11
Novel_016047	XLOC_164198	.	transcript	0	0.126327	20.10997827	3.29E-11	2.08E-09
Novel_015923	XLOC_163120	.	transcript	0	0.527009	10.04630249	3.19E-05	0.001734
Novel_015895	XLOC_162964	.	transcript	0.219504	0	-23.35627248	1.21E-14	3.48E-12
Novel_015896	XLOC_162964	.	transcript	0.090391	0	-22.19981161	2.30E-13	2.91E-11
Novel_015886	XLOC_162951	.	transcript	1.841826	0	-24.58584171	4.54E-16	3.21E-13
Novel_015887	XLOC_162951	.	transcript	0.614254	0	-23.09054096	2.40E-14	5.82E-12
Novel_015857	XLOC_162793	.	transcript	0	0.129414	21.82985147	5.61E-13	5.63E-11
Novel_015851	XLOC_162781	.	transcript	0	0.70663	23.5731177	2.93E-18	7.80E-15
Novel_015824	XLOC_162658	.	transcript	0	0.769193	24.50254685	5.67E-16	3.79E-13
Novel_015797	XLOC_162622	.	transcript	0.103863	0	-21.6536291	8.90E-13	8.15E-11
Novel_015790	XLOC_162560	.	transcript	0.018535	0	-21.9448617	4.34E-13	4.61E-11
Novel_015787	XLOC_162533	.	transcript	0	0.095083	21.17603012	2.72E-12	2.07E-10
Novel_015786	XLOC_162533	.	transcript	0	0.205767	21.85535303	5.23E-13	5.35E-11
Novel_015769	XLOC_162427	.	transcript	0.119719	0	-21.29699898	2.12E-12	1.67E-10
Novel_015763	XLOC_162374	.	transcript	0.001108	0.047982	4.826198	0.000531	0.023595
Novel_015727	XLOC_162054	.	transcript	0.178642	0	-23.77538971	4.01E-15	1.57E-12
Novel_015726	XLOC_162054	.	transcript	0	0.031898	20.435114	1.55E-11	1.02E-09
Novel_015674	XLOC_161936	.	transcript	0	0.072307	20.57089999	1.13E-11	7.62E-10
Novel_015661	XLOC_161647	.	transcript	0.07522	0	-6.652246985	0.000675	0.029226
Novel_015650	XLOC_161587	.	transcript	0.150005	0	-24.10817224	1.65E-15	8.66E-13
Novel_015604	XLOC_161328	.	transcript	0.285725	0	-9.677285316	2.47E-08	1.49E-06
Novel_015591	XLOC_161262	.	transcript	0	0.30432	22.33967655	1.59E-13	2.21E-11
Novel_015525	XLOC_160965	.	transcript	0	0.103245	21.24311166	2.32E-12	1.80E-10

Novel_015517	XLOC_160853	.	transcript	0	0.285157	23.0391801	2.71E-14	6.40E-12
Novel_015513	XLOC_160831	.	transcript	0	0.077157	22.71839697	6.14E-14	1.14E-11
Novel_015503	XLOC_160814	.	transcript	0.003034	0.150232	5.018418003	0.000537	0.023836
Novel_015491	XLOC_160767	.	transcript	0	0.037522	21.16888734	2.77E-12	2.10E-10
Novel_015434	XLOC_160386	.	transcript	0.389772	0	-21.97204018	4.06E-13	4.37E-11
Novel_015356	XLOC_159766	.	transcript	0	0.089372	20.91298051	5.08E-12	3.65E-10
Novel_015303	XLOC_159574	.	transcript	0.053668	0	-8.744049076	1.38E-05	0.000774
Novel_015270	XLOC_159479	.	transcript	0	0.025157	21.20932299	2.51E-12	1.94E-10
Novel_015230	XLOC_159287	.	transcript	0	0.163165	8.139086654	3.27E-06	0.000189
Novel_015223	XLOC_159229	.	transcript	0	0.055998	21.16451777	2.80E-12	2.12E-10
Novel_015215	XLOC_159182	.	transcript	0.624169	0	-22.366144	1.52E-13	2.14E-11
Novel_015186	XLOC_159068	.	transcript	0	0.664283	24.08807197	1.73E-15	8.76E-13
Novel_015137	XLOC_158773	.	transcript	0.074002	0	-20.62213642	1.02E-11	6.92E-10
Novel_015061	XLOC_158348	.	transcript	0	0.08282	21.6538743	8.61E-13	7.91E-11
Novel_014943	XLOC_157438	.	transcript	0	0.371165	8.424252837	0.000547	0.024215
Novel_014826	XLOC_155984	.	transcript	0.235418	0	-22.91335497	3.79E-14	7.95E-12
Novel_014733	XLOC_155397	.	transcript	0.081413	3.52E-05	-6.116216434	0.000126	0.00636
Novel_014717	XLOC_155281	.	transcript	0	0.357305	23.41374683	1.03E-14	3.11E-12
Novel_014597	XLOC_154564	.	transcript	0.064106	0.218422	1.740464022	0.000387	0.017807
Novel_014549	XLOC_153750	.	transcript	0.647732	0.006629	-6.480629342	1.53E-06	8.95E-05
Novel_014553	XLOC_153750	.	transcript	0	0.150518	22.40962478	1.34E-13	1.97E-11
Novel_014534	XLOC_153630	.	transcript	0	0.081088	21.29598344	2.04E-12	1.62E-10
Novel_014448	XLOC_152637	.	transcript	0	0.161318	23.12151066	2.19E-14	5.49E-12
Novel_014375	XLOC_152390	.	transcript	0.304054	0	-23.86141021	3.19E-15	1.37E-12
Novel_014374	XLOC_152390	.	transcript	0.406907	0	-23.78148535	3.95E-15	1.56E-12
Novel_014376	XLOC_152390	.	transcript	0.248468	0.000802	-7.232894262	0.000142	0.007115
Novel_014331	XLOC_151853	.	transcript	0.019906	0	-21.62005021	9.66E-13	8.70E-11
Novel_014313	XLOC_151393	.	transcript	0.043907	0	-22.85126063	4.44E-14	8.89E-12
Novel_014310	XLOC_151393	.	transcript	0.155303	0	-9.501632585	8.22E-05	0.004272
Novel_014306	XLOC_151274	.	transcript	0.083906	0	-22.06218876	3.25E-13	3.71E-11
Novel_014299	XLOC_151110	.	transcript	0	0.083783	9.083702495	3.36E-07	2.00E-05
Novel_014297	XLOC_151110	.	transcript	0	0.075366	22.52874055	9.87E-14	1.58E-11
Novel_014273	XLOC_150905	.	transcript	0.138031	0	-22.59357149	8.44E-14	1.40E-11
Novel_014267	XLOC_150905	.	transcript	5.72E-05	0.138008	7.806227412	0.000296	0.01407
Novel_014186	XLOC_150341	.	transcript	0.076584	4.60E-06	-9.189840733	0.000827	0.034968
Novel_014173	XLOC_150298	.	transcript	0	0.054218	22.29216766	1.79E-13	2.43E-11
Novel_014110	XLOC_149526	.	transcript	0	0.047896	22.13679436	2.63E-13	3.21E-11
Novel_014075	XLOC_149355	.	transcript	0.151231	0	-21.90875937	4.75E-13	4.97E-11

Novel_014060	XLOC_149309	.	transcript	3.90931	0.032041	-6.73099641	6.67E-05	0.003513
Novel_014041	XLOC_149036	.	transcript	0.002807	0.122633	5.104384454	4.11E-05	0.002208
Novel_014024	XLOC_148964	.	transcript	0	0.095082	21.3500727	1.77E-12	1.44E-10
Novel_014027	XLOC_148964	.	transcript	0	0.114721	21.92008611	4.49E-13	4.74E-11
Novel_013952	XLOC_147239	.	transcript	0.186061	0.013606	-3.68400676	1.18E-05	0.000663
Novel_013833	XLOC_146547	.	transcript	0.066835	0	-22.00027861	3.79E-13	4.13E-11
Novel_013822	XLOC_146503	.	transcript	0	0.227444	22.18663367	2.33E-13	2.93E-11
Novel_013797	XLOC_146253	.	transcript	0	0.164274	22.27657046	1.86E-13	2.48E-11
Novel_013768	XLOC_146114	.	transcript	9.490926	0	-25.50706984	3.51E-17	4.97E-14
Novel_013742	XLOC_145991	.	transcript	0.159455	0	-22.78837948	5.22E-14	1.01E-11
Novel_013711	XLOC_145731	.	transcript	0	0.306915	8.584042405	3.12E-14	6.86E-12
Novel_013705	XLOC_145723	.	transcript	0	0.013243	20.27395765	2.25E-11	1.45E-09
Novel_013631	XLOC_145026	.	transcript	0.149967	0	-22.86710346	4.27E-14	8.63E-12
Novel_013604	XLOC_144518	.	transcript	0.062371	0	-21.56004654	1.12E-12	9.77E-11
Novel_013594	XLOC_144498	.	transcript	0	0.383766	22.44476701	1.22E-13	1.83E-11
Novel_013577	XLOC_144387	.	transcript	0	0.198379	20.63846691	9.68E-12	6.62E-10
Novel_013530	XLOC_143970	.	transcript	0.103031	0	-23.40892706	1.05E-14	3.16E-12
Novel_013492	XLOC_143572	.	transcript	0.094611	0	-22.83501293	4.63E-14	9.17E-12
Novel_013495	XLOC_143572	.	transcript	0	0.169946	22.36612398	1.49E-13	2.10E-11
Novel_013484	XLOC_143534	.	transcript	0.018851	0	-21.29699898	2.12E-12	1.67E-10
Novel_013486	XLOC_143534	.	transcript	0	0.023245	20.61598037	1.02E-11	6.93E-10
Novel_013487	XLOC_143534	.	transcript	0	0.029708	20.94859087	4.67E-12	3.38E-10
Novel_013481	XLOC_143508	.	transcript	0	0.381496	20.63531717	9.75E-12	6.66E-10
Novel_013467	XLOC_143376	.	transcript	0.155913	0	-21.90857724	4.75E-13	4.97E-11
Novel_013432	XLOC_142857	.	transcript	0.440247	0	-24.02144464	2.09E-15	9.96E-13
Novel_013431	XLOC_142857	.	transcript	0.465871	0	-23.9073722	2.82E-15	1.25E-12
Novel_013403	XLOC_142651	.	transcript	0.468498	0.011501	-5.363622566	0.000153	0.007632
Novel_013389	XLOC_142621	.	transcript	0.164674	0	-22.70799376	6.40E-14	1.17E-11
Novel_013362	XLOC_142444	.	transcript	0.112322	0	-21.63847178	9.24E-13	8.41E-11
Novel_013358	XLOC_142401	.	transcript	0	0.174732	21.46115663	1.37E-12	1.16E-10
Novel_013278	XLOC_141550	.	transcript	0.088646	0	-22.24637733	2.05E-13	2.68E-11
Novel_013279	XLOC_141550	.	transcript	0.237817	0	-9.181680512	0.000157	0.007806
Novel_013233	XLOC_141029	.	transcript	0	0.065513	20.10327058	3.34E-11	2.11E-09
Novel_013216	XLOC_140949	.	transcript	0.341535	0	-8.342998993	0.000542	0.024014
Novel_013137	XLOC_139891	.	transcript	0	0.088065	21.01491743	3.87E-12	2.87E-10
Novel_013130	XLOC_139861	.	transcript	0.221863	0	-22.84442911	4.52E-14	8.98E-12
Novel_013107	XLOC_139098	.	transcript	0.187574	0	-22.67853451	6.90E-14	1.23E-11
Novel_013096	XLOC_139042	.	transcript	0	0.116833	20.36137695	1.76E-11	1.15E-09

Novel_013048	XLOC_138659	.	transcript	0.032429	0	-22.05022182	3.34E-13	3.79E-11
Novel_013029	XLOC_138467	.	transcript	0.953022	0.001286	-8.11861325	0.001016	0.041996
Novel_013031	XLOC_138467	.	transcript	0	2.677122	24.08325936	1.76E-15	8.82E-13
Novel_013002	XLOC_138040	.	transcript	0	0.533764	22.37392283	1.46E-13	2.08E-11
Novel_012995	XLOC_137995	.	transcript	0.057581	0	-21.42676906	1.55E-12	1.28E-10
Novel_012975	XLOC_137919	.	transcript	0	0.090252	21.07830478	3.43E-12	2.58E-10
Novel_012944	XLOC_137534	.	transcript	0.20314	0	-24.6670211	3.64E-16	2.74E-13
Novel_012945	XLOC_137534	.	transcript	0.03709	0	-21.59194623	1.03E-12	9.15E-11
Novel_012926	XLOC_137390	.	transcript	0	0.051098	19.90252721	5.29E-11	3.26E-09
Novel_012889	XLOC_137134	.	transcript	1.078452	0	-25.38190045	5.00E-17	6.44E-14
Novel_012895	XLOC_137134	.	transcript	0.735314	0	-24.92784786	1.77E-16	1.74E-13
Novel_012883	XLOC_137124	.	transcript	0	0.066792	20.9151659	5.05E-12	3.64E-10
Novel_012865	XLOC_136802	.	transcript	0	0.197609	20.56694094	1.14E-11	7.69E-10
Novel_012829	XLOC_136165	.	transcript	0	0.132298	7.881889578	0.001021	0.042133
Novel_012819	XLOC_136157	.	transcript	0.000363	0.335942	9.461449855	0.00014	0.007005
Novel_012778	XLOC_135961	.	transcript	0.468962	0	-22.11508234	2.85E-13	3.40E-11
Novel_012776	XLOC_135961	.	transcript	0.210149	0	-21.85803047	5.38E-13	5.45E-11
Novel_012725	XLOC_135550	.	transcript	0.148041	0	-22.45600873	1.21E-13	1.83E-11
Novel_012706	XLOC_135461	.	transcript	0.00244	0.24818	6.541128064	5.54E-05	0.002948
Novel_012687	XLOC_135461	.	transcript	0	0.006407	20.24160198	2.43E-11	1.56E-09
Novel_012693	XLOC_135461	.	transcript	0	0.013966	20.52133039	1.27E-11	8.48E-10
Novel_012682	XLOC_135461	.	transcript	0	0.013006	21.1785971	2.70E-12	2.07E-10
Novel_012680	XLOC_135461	.	transcript	0	0.018758	22.16488168	2.46E-13	3.07E-11
Novel_012659	XLOC_135145	.	transcript	0.057765	0	-22.53260646	9.99E-14	1.59E-11
Novel_012629	XLOC_134645	.	transcript	0.065526	0	-21.65837094	8.79E-13	8.07E-11
Novel_012591	XLOC_134365	.	transcript	0	0.049183	20.17391609	2.84E-11	1.81E-09
Novel_012588	XLOC_134326	.	transcript	0.213026	0	-21.46296773	1.42E-12	1.19E-10
Novel_012583	XLOC_134293	.	transcript	0.0769	0	-21.58668338	1.05E-12	9.27E-11
Novel_012534	XLOC_133954	.	transcript	5.40E-06	0.151137	9.023480683	0.000643	0.027988
Novel_012535	XLOC_133954	.	transcript	0	0.162077	23.01068372	2.91E-14	6.56E-12
Novel_012523	XLOC_133759	.	transcript	0.166064	0	-22.05800877	3.28E-13	3.74E-11
Novel_012485	XLOC_133249	.	transcript	0.20299	0	-24.19543061	1.31E-15	7.24E-13
Novel_012398	XLOC_132365	.	transcript	0.074792	0	-21.30458473	2.08E-12	1.65E-10
Novel_012396	XLOC_132365	.	transcript	0	0.113499	20.61217552	1.03E-11	6.98E-10
Novel_012364	XLOC_132045	.	transcript	0.020203	0	-23.58808574	6.58E-15	2.26E-12
Novel_012362	XLOC_132045	.	transcript	0.049663	0	-9.265752937	0.000388	0.01787
Novel_012322	XLOC_131875	.	transcript	0.12975	0	-21.81844846	5.93E-13	5.88E-11
Novel_012320	XLOC_131875	.	transcript	0	0.490881	22.14656454	2.57E-13	3.18E-11

Novel_012249	XLOC_130995	.	transcript	0.137318	0	-23.45404643	9.35E-15	2.88E-12
Novel_012243	XLOC_130983	.	transcript	0.269118	0	-23.25394166	1.57E-14	4.35E-12
Novel_012242	XLOC_130983	.	transcript	0.083498	0	-22.87622374	4.17E-14	8.54E-12
Novel_012172	XLOC_130251	.	transcript	0.094277	0	-21.31889336	1.93E-12	1.55E-10
Novel_012156	XLOC_129692	.	transcript	0.370252	0	-22.32688188	1.68E-13	2.30E-11
Novel_012153	XLOC_129608	.	transcript	0	0.027094	19.90252721	5.29E-11	3.26E-09
Novel_012131	XLOC_129476	.	transcript	1.05916	0	-11.84670253	1.42E-11	9.45E-10
Novel_012094	XLOC_128835	.	transcript	0	0.071386	21.05386266	3.64E-12	2.72E-10
Novel_012078	XLOC_128809	.	transcript	0	0.188241	22.38886687	1.41E-13	2.04E-11
Novel_012035	XLOC_128531	.	transcript	0	0.224019	22.11358087	2.79E-13	3.36E-11
Novel_012012	XLOC_128384	.	transcript	0.080881	0	-23.83935402	3.39E-15	1.41E-12
Novel_011999	XLOC_128266	.	transcript	0.162598	0	-6.553385666	0.000234	0.011333
Novel_011965	XLOC_127876	.	transcript	0.093523	0	-21.47716115	1.37E-12	1.16E-10
Novel_011955	XLOC_127802	.	transcript	0	0.112245	21.13404925	3.01E-12	2.27E-10
Novel_011950	XLOC_127760	.	transcript	0	0.06692	21.82419528	5.69E-13	5.68E-11
Novel_011948	XLOC_127746	.	transcript	0	0.127901	21.28577554	2.09E-12	1.65E-10
Novel_011921	XLOC_127412	.	transcript	0	0.035781	20.98469186	4.29E-12	3.15E-10
Novel_011911	XLOC_127130	.	transcript	0	0.128008	21.53813053	1.14E-12	9.89E-11
Novel_011880	XLOC_126251	.	transcript	0.109249	0	-23.4763392	8.82E-15	2.77E-12
Novel_011875	XLOC_126214	.	transcript	0.000746	0.290353	7.59933528	2.79E-05	0.001525
Novel_011866	XLOC_126145	.	transcript	0.169513	0	-22.87392056	4.19E-14	8.54E-12
Novel_011862	XLOC_126084	.	transcript	0.393041	0	-23.76711718	4.10E-15	1.59E-12
Novel_011821	XLOC_125366	.	transcript	0.075611	0	-20.65638042	9.33E-12	6.42E-10
Novel_011806	XLOC_124915	.	transcript	0.062613	0	-22.02813841	3.53E-13	3.97E-11
Novel_011790	XLOC_124827	.	transcript	0.049177	0	-21.8058633	6.12E-13	6.02E-11
Novel_011782	XLOC_124655	.	transcript	0	0.183956	22.77553501	5.31E-14	1.02E-11
Novel_011767	XLOC_124555	.	transcript	0.090825	0	-22.62081179	7.98E-14	1.34E-11
Novel_011765	XLOC_124552	.	transcript	0	0.281109	21.95788675	4.10E-13	4.41E-11
Novel_011716	XLOC_124190	.	transcript	0	0.819294	23.47239404	8.78E-15	2.77E-12
Novel_011714	XLOC_124190	.	transcript	0	0.306708	23.63168678	5.82E-15	2.07E-12
Novel_011715	XLOC_124190	.	transcript	0	0.761559	24.51640396	5.46E-16	3.69E-13
Novel_011713	XLOC_124188	.	transcript	0.122163	0	-22.29660616	1.81E-13	2.44E-11
Novel_011628	XLOC_123540	.	transcript	0.263328	0	-23.31277542	1.35E-14	3.82E-12
Novel_011618	XLOC_123504	.	transcript	0.151694	0	-7.612999167	0.000137	0.006892
Novel_011594	XLOC_123371	.	transcript	0.155897	0	-9.359521065	0.000264	0.012671
Novel_011519	XLOC_122491	.	transcript	0	0.054251	20.31667047	2.05E-11	1.33E-09
Novel_011514	XLOC_122483	.	transcript	0	0.074115	21.07803055	3.44E-12	2.58E-10
Novel_011511	XLOC_122483	.	transcript	0	0.182311	22.12072259	2.73E-13	3.30E-11

Novel_011512	XLOC_122483	.	transcript	0	0.213251	22.33623104	1.60E-13	2.23E-11
Novel_011501	XLOC_122317	.	transcript	0.102109	0	-24.23621963	1.17E-15	6.66E-13
Novel_011499	XLOC_122317	.	transcript	0	0.296889	26.41420391	2.58E-18	7.20E-15
Novel_011478	XLOC_121929	.	transcript	0.189034	0	-21.79987446	6.21E-13	6.08E-11
Novel_011464	XLOC_121756	.	transcript	0	0.260672	23.08266934	2.42E-14	5.85E-12
Novel_011432	XLOC_121516	.	transcript	0.032492	0	-21.74566367	7.10E-13	6.75E-11
Novel_011436	XLOC_121516	.	transcript	0.10291	0	-20.59191736	1.05E-11	7.11E-10
Novel_011435	XLOC_121516	.	transcript	0.357316	0.033621	-3.416186154	0.000138	0.006916
Novel_011392	XLOC_121101	.	transcript	0	0.070735	20.95982557	4.55E-12	3.31E-10
Novel_011306	XLOC_120400	.	transcript	0	0.140896	22.00980214	3.61E-13	4.01E-11
Novel_011290	XLOC_120231	.	transcript	0	0.24206	9.479516999	0.000122	0.006168
Novel_011244	XLOC_119776	.	transcript	0	0.085339	21.05103258	3.66E-12	2.74E-10
Novel_011205	XLOC_119558	.	transcript	0	0.044104	20.39096272	1.72E-11	1.13E-09
Novel_011129	XLOC_119171	.	transcript	0.457775	0.065822	-2.857488677	0.001043	0.042897
Novel_011115	XLOC_118954	.	transcript	0.193953	0	-7.808395223	0.001076	0.04412
Novel_011117	XLOC_118954	.	transcript	0	0.115471	21.33730188	1.85E-12	1.50E-10
Novel_011052	XLOC_118031	.	transcript	0.202417	0	-22.36352431	1.53E-13	2.15E-11
Novel_011020	XLOC_117711	.	transcript	0	0.206299	22.5206179	1.01E-13	1.59E-11
Novel_010984	XLOC_117096	.	transcript	0.133665	0	-21.84584887	5.54E-13	5.57E-11
Novel_010938	XLOC_116366	.	transcript	0.131106	0	-8.566656315	4.96E-06	0.000285
Novel_010898	XLOC_115794	.	transcript	0	0.068162	20.84155734	6.01E-12	4.27E-10
Novel_010868	XLOC_115503	.	transcript	0	2.569116	23.74492752	4.31E-15	1.65E-12
Novel_010849	XLOC_115280	.	transcript	0	0.035259	19.16928696	2.50E-10	1.53E-08
Novel_010830	XLOC_115080	.	transcript	0.036234	0	-21.42982929	1.54E-12	1.28E-10
Novel_010800	XLOC_114576	.	transcript	0	0.260073	11.42295147	0.00016	0.007964
Novel_010789	XLOC_114447	.	transcript	0	0.052076	20.27659058	2.22E-11	1.43E-09
Novel_010776	XLOC_114386	.	transcript	0	0.030005	20.8697733	5.62E-12	4.00E-10
Novel_010729	XLOC_113738	.	transcript	0.065807	0	-22.39051295	1.42E-13	2.06E-11
Novel_010696	XLOC_113333	.	transcript	0.03152	0	-21.55805372	1.12E-12	9.80E-11
Novel_010658	XLOC_112982	.	transcript	0.079788	0	-21.59194623	1.03E-12	9.15E-11
Novel_010653	XLOC_112889	.	transcript	0	0.185243	21.17603012	2.72E-12	2.07E-10
Novel_010611	XLOC_112608	.	transcript	0.173501	0	-23.43989752	9.70E-15	2.97E-12
Novel_010561	XLOC_111559	.	transcript	0	0.452927	8.975855268	0.000183	0.009011
Novel_010557	XLOC_111558	.	transcript	0	0.08033	20.99593746	4.17E-12	3.07E-10
Novel_010550	XLOC_111304	.	transcript	0	0.087286	21.34601584	1.81E-12	1.48E-10
Novel_010495	XLOC_110685	.	transcript	0	0.087399	8.608279992	0.00044	0.019921
Novel_010502	XLOC_110685	.	transcript	0	0.157949	9.50571607	0.000113	0.005789
Novel_010494	XLOC_110685	.	transcript	0	0.148641	22.80449459	4.92E-14	9.56E-12

Novel_010485	XLOC_110662	.	transcript	0.265327	0	-22.11100372	2.87E-13	3.42E-11
Novel_010484	XLOC_110653	.	transcript	0	0.157874	10.25449681	4.93E-09	2.99E-07
Novel_010481	XLOC_110651	.	transcript	0.181501	0	-21.75727108	6.90E-13	6.61E-11
Novel_010424	XLOC_110470	.	transcript	0	0.094582	21.50898696	1.22E-12	1.05E-10
Novel_010413	XLOC_110440	.	transcript	0	0.325375	7.692896259	0.001217	0.049368
Novel_010307	XLOC_109594	.	transcript	0.046318	0	-21.83739202	5.66E-13	5.67E-11
Novel_010271	XLOC_109174	.	transcript	0.057564	0	-23.10446154	2.32E-14	5.71E-12
Novel_010272	XLOC_109174	.	transcript	0.056094	0	-22.94701589	3.47E-14	7.48E-12
Novel_010253	XLOC_108980	.	transcript	0.103336	0	-22.19525392	2.33E-13	2.93E-11
Novel_010258	XLOC_108980	.	transcript	0.006757	0.148089	3.952440026	0.000404	0.018493
Novel_010218	XLOC_108316	.	transcript	0.097703	0	-22.53260646	9.99E-14	1.59E-11
Novel_010182	XLOC_107861	.	transcript	8.356334	0	-25.7156198	1.94E-17	3.34E-14
Novel_010187	XLOC_107861	.	transcript	1.360773	0	-24.2783599	1.05E-15	6.11E-13
Novel_010193	XLOC_107861	.	transcript	0.188533	0	-21.47716115	1.37E-12	1.16E-10
Novel_010197	XLOC_107861	.	transcript	0.000564	2.533369	10.23094112	0.000724	0.031076
Novel_010171	XLOC_107861	.	transcript	0	0.663192	20.28031749	2.11E-11	1.37E-09
Novel_010180	XLOC_107861	.	transcript	0	1.689749	22.34784611	1.55E-13	2.17E-11
Novel_010181	XLOC_107861	.	transcript	0	2.593209	24.17731835	1.37E-15	7.47E-13
Novel_010152	XLOC_107405	.	transcript	0.176983	0	-23.13151468	2.16E-14	5.42E-12
Novel_010139	XLOC_107188	.	transcript	0.285224	0	-22.17098827	2.44E-13	3.05E-11
Novel_010123	XLOC_106609	.	transcript	0.167408	0	-23.41247384	1.04E-14	3.15E-12
Novel_010122	XLOC_106609	.	transcript	0.21894	0	-23.37533817	1.15E-14	3.38E-12
Novel_010124	XLOC_106609	.	transcript	0.150169	0	-22.64445401	7.43E-14	1.30E-11
Novel_010009	XLOC_105734	.	transcript	0	0.092185	23.2617737	1.52E-14	4.23E-12
Novel_009953	XLOC_105562	.	transcript	0.111954	0.000305	-7.907599572	4.98E-05	0.002665
Novel_009919	XLOC_105407	.	transcript	0	0.060056	22.28843963	1.81E-13	2.44E-11
Novel_009908	XLOC_105364	.	transcript	0	0.039775	21.41820157	1.52E-12	1.27E-10
Novel_009911	XLOC_105364	.	transcript	0	0.039995	23.02077555	2.84E-14	6.51E-12
Novel_009870	XLOC_105253	.	transcript	0	0.324124	22.15022527	2.53E-13	3.15E-11
Novel_009854	XLOC_105177	.	transcript	0.183119	0	-23.01374178	2.93E-14	6.56E-12
Novel_009845	XLOC_105143	.	transcript	0.0295	0	-21.61620533	9.75E-13	8.76E-11
Novel_009815	XLOC_104784	.	transcript	0	0.128613	22.92552079	3.62E-14	7.73E-12
Novel_009807	XLOC_104724	.	transcript	0	0.194101	8.044411796	0.000878	0.036851
Novel_009768	XLOC_103970	.	transcript	0.38128	0	-23.97885652	2.34E-15	1.07E-12
Novel_009757	XLOC_103893	.	transcript	0.139928	0	-22.53237206	9.96E-14	1.59E-11
Novel_009737	XLOC_103728	.	transcript	0.070139	0	-21.30458473	2.08E-12	1.65E-10
Novel_009694	XLOC_103093	.	transcript	0.1993	0	-23.03263658	2.79E-14	6.46E-12
Novel_009685	XLOC_103037	.	transcript	14.61029	0	-27.04319258	4.03E-19	1.59E-15

Novel_009686	XLOC_103037	.	transcript	137.9042	1.120301	-7.068760941	0.000326	0.015334
Novel_009681	XLOC_103037	.	transcript	168.7331	3.554334	-5.767834625	0.000995	0.041203
Novel_009665	XLOC_102902	.	transcript	0.252855	0	-21.89138272	4.95E-13	5.13E-11
Novel_009655	XLOC_102898	.	transcript	0	0.126757	22.2434309	2.01E-13	2.63E-11
Novel_009657	XLOC_102898	.	transcript	0	0.245437	23.60660445	6.21E-15	2.16E-12
Novel_009645	XLOC_102850	.	transcript	0.169427	0	-22.72035896	6.09E-14	1.13E-11
Novel_009640	XLOC_102368	.	transcript	0	1.016496	21.85243811	5.26E-13	5.35E-11
Novel_009620	XLOC_102302	.	transcript	0.031737	0	-21.87505187	5.16E-13	5.29E-11
Novel_009598	XLOC_101788	.	transcript	0.038601	0	-22.01663194	3.63E-13	4.02E-11
Novel_009597	XLOC_101788	.	transcript	0.008124	0.47988	5.785740522	6.76E-10	4.12E-08
Novel_009540	XLOC_101242	.	transcript	0	0.072205	21.43896792	1.45E-12	1.21E-10
Novel_009309	XLOC_098823	.	transcript	0.024581	0	-21.93198926	4.48E-13	4.74E-11
Novel_009267	XLOC_098491	.	transcript	0.176402	0	-23.36528066	1.18E-14	3.42E-12
Novel_009249	XLOC_098302	.	transcript	0.392066	0	-23.85839475	3.22E-15	1.37E-12
Novel_009247	XLOC_098302	.	transcript	0.277792	0	-23.40086769	1.07E-14	3.21E-12
Novel_009250	XLOC_098302	.	transcript	0.186718	0	-22.86428698	4.30E-14	8.68E-12
Novel_009253	XLOC_098302	.	transcript	0.103778	0	-22.49646487	1.09E-13	1.68E-11
Novel_009221	XLOC_097822	.	transcript	0	0.2452	8.160185732	0.000557	0.024583
Novel_009190	XLOC_097494	.	transcript	0	0.2884	21.47288121	1.33E-12	1.13E-10
Novel_009166	XLOC_097124	.	transcript	0.024086	0	-22.22297997	2.18E-13	2.79E-11
Novel_009172	XLOC_097124	.	transcript	0	0.010924	20.26381283	2.31E-11	1.49E-09
Novel_009153	XLOC_097016	.	transcript	0.05845	0	-21.53956803	1.18E-12	1.02E-10
Novel_009145	XLOC_096956	.	transcript	0.277502	0	-21.85176916	5.47E-13	5.52E-11
Novel_009110	XLOC_096577	.	transcript	0.144409	0	-23.41903754	1.02E-14	3.11E-12
Novel_009013	XLOC_095197	.	transcript	0	0.099891	22.52432129	1.00E-13	1.59E-11
Novel_008965	XLOC_095031	.	transcript	0.1622	0	-8.546130174	0.00105	0.043166
Novel_008935	XLOC_094971	.	transcript	1.59051	0	-25.88404164	1.20E-17	2.20E-14
Novel_008928	XLOC_094971	.	transcript	2.611162	0	-25.56094853	3.01E-17	4.49E-14
Novel_008953	XLOC_094971	.	transcript	0.12498	0	-23.7549823	4.24E-15	1.63E-12
Novel_008946	XLOC_094971	.	transcript	0.129702	0	-22.38130839	1.46E-13	2.08E-11
Novel_008957	XLOC_094971	.	transcript	0.191108	0	-21.84252978	5.59E-13	5.62E-11
Novel_008951	XLOC_094971	.	transcript	0.163575	0	-21.78394899	6.46E-13	6.26E-11
Novel_008948	XLOC_094971	.	transcript	0.647312	0	-10.67842306	0.000418	0.019051
Novel_008938	XLOC_094971	.	transcript	0	0.179784	20.03666091	3.90E-11	2.42E-09
Novel_008931	XLOC_094971	.	transcript	0	0.401117	21.70215005	7.66E-13	7.15E-11
Novel_008942	XLOC_094971	.	transcript	0	0.069868	22.66096973	7.07E-14	1.26E-11
Novel_008944	XLOC_094971	.	transcript	0	0.071941	23.10304676	2.30E-14	5.69E-12
Novel_008949	XLOC_094971	.	transcript	0	0.281691	24.51573113	5.47E-16	3.69E-13

Novel_008821	XLOC_093955	.		transcript	0.073826	0	-21.96576406	4.12E-13	4.43E-11
Novel_008759	XLOC_093114	.		transcript	0	0.282635	8.403941511	0.000409	0.018682
Novel_008745	XLOC_093011	.		transcript	0.038879	0	-21.3913526	1.69E-12	1.39E-10
Novel_008736	XLOC_093001	.		transcript	0	0.178346	20.96488162	4.49E-12	3.27E-10
Novel_008733	XLOC_092982	.		transcript	0	0.041177	21.29163865	2.06E-12	1.63E-10
Novel_008723	XLOC_092942	.		transcript	0	0.150148	21.77950233	6.34E-13	6.17E-11
Novel_008700	XLOC_092839	.		transcript	0.137965	0	-23.30654984	1.37E-14	3.85E-12
Novel_008672	XLOC_092307	.		transcript	0	0.21474	21.77690722	6.37E-13	6.19E-11
Novel_008659	XLOC_092183	.		transcript	0	0.056982	21.87986551	4.96E-13	5.13E-11
Novel_008649	XLOC_092156	.		transcript	0.20606	0	-22.75346256	5.70E-14	1.08E-11
Novel_008612	XLOC_091972	.		transcript	0.005602	0.208599	5.096433617	0.000176	0.008649
Novel_008593	XLOC_091765	.		transcript	0.261573	0	-23.49573727	8.38E-15	2.68E-12
Novel_008592	XLOC_091765	.		transcript	0.069063	0	-21.71392804	7.68E-13	7.15E-11
Novel_008525	XLOC_091309	.		transcript	0	0.158962	22.4630401	1.17E-13	1.77E-11
Novel_008501	XLOC_091135	.		transcript	0	0.030279	20.25185713	2.38E-11	1.53E-09
Novel_008454	XLOC_090510	.		transcript	0.000217	0.012028	5.065044099	0.000849	0.035735
Novel_008453	XLOC_090510	.		transcript	0	0.013364	21.0457649	3.71E-12	2.77E-10
Novel_008428	XLOC_090438	.		transcript	0.095361	0	-22.52937966	9.91E-14	1.59E-11
Novel_008400	XLOC_090296	.		transcript	1.86E-05	0.422373	10.05087446	0.00019	0.009312
Novel_008354	XLOC_089876	.		transcript	0.489323	0.001823	-7.914265509	0.000148	0.007386
Novel_008352	XLOC_089876	.		transcript	0	0.185673	22.35546945	1.51E-13	2.13E-11
Novel_008342	XLOC_089514	.		transcript	0	0.485412	22.71034784	6.26E-14	1.15E-11
Novel_008333	XLOC_089420	.		transcript	0.142413	0	-23.06111952	2.59E-14	6.19E-12
Novel_008321	XLOC_089333	.		transcript	0	0.214268	22.73946744	5.82E-14	1.10E-11
Novel_008298	XLOC_089085	.		transcript	0.373666	0	-24.81512334	2.42E-16	2.07E-13
Novel_008286	XLOC_089008	.		transcript	0.047484	1.00E-05	-7.300507308	6.41E-05	0.003384
Novel_008273	XLOC_088803	.		transcript	0.205956	0	-22.63279005	7.75E-14	1.33E-11
Novel_008172	XLOC_086552	.		transcript	0.181477	0	-9.431747355	8.71E-05	0.004521
Novel_008170	XLOC_086552	.		transcript	0.072415	0.00407	-3.896145803	0.000171	0.008449
Novel_008166	XLOC_086552	.		transcript	0	0.137953	21.97500899	3.93E-13	4.27E-11
Novel_008142	XLOC_086045	.		transcript	0.095307	0.25483	1.34579841	0.000495	0.022132
Novel_008109	XLOC_085829	.		transcript	0	0.048533	22.44981726	1.20E-13	1.82E-11
Novel_008088	XLOC_085697	.		transcript	0.278787	0	-8.202123807	1.09E-05	0.000614
Novel_008083	XLOC_085640	.		transcript	0.251385	0	-25.42721333	4.40E-17	5.97E-14
Novel_008075	XLOC_085616	.		transcript	0.078159	0	-23.19593037	1.82E-14	4.89E-12
Novel_008059	XLOC_085237	.		transcript	0	0.223258	21.36717569	1.72E-12	1.41E-10
Novel_007986	XLOC_084491	.		transcript	0	0.073299	21.27072819	2.17E-12	1.70E-10
Novel_007984	XLOC_084481	.		transcript	0.225685	0	-22.39806958	1.40E-13	2.04E-11

Novel_007966	XLOC_083735	.	transcript	0	0.125539	8.633325095	0.000295	0.014015
Novel_007896	XLOC_082470	.	transcript	0	0.088384	21.73157905	7.13E-13	6.75E-11
Novel_007882	XLOC_082357	.	transcript	0.11789	0.014048	-3.024264743	0.000466	0.020976
Novel_007853	XLOC_081807	.	transcript	0	0.04354	20.97204055	4.42E-12	3.22E-10
Novel_007828	XLOC_081603	.	transcript	0	0.07836	21.90694605	4.61E-13	4.85E-11
Novel_007819	XLOC_081568	.	transcript	0	0.075732	20.6001563	1.06E-11	7.12E-10
Novel_007788	XLOC_081199	.	transcript	0.394754	0	-23.13406752	2.14E-14	5.41E-12
Novel_007706	XLOC_080591	.	transcript	0.151817	0	-8.839122454	0.00023	0.011145
Novel_007653	XLOC_080205	.	transcript	0	0.41437	20.32113164	2.02E-11	1.32E-09
Novel_007636	XLOC_080004	.	transcript	0	0.090671	22.37990524	1.44E-13	2.07E-11
Novel_007624	XLOC_079860	.	transcript	0.036501	0	-21.45490579	1.45E-12	1.21E-10
Novel_007609	XLOC_079492	.	transcript	0.162305	0	-9.287725619	0.000119	0.006065
Novel_007604	XLOC_079082	.	transcript	0	0.078448	22.97402173	3.20E-14	6.99E-12
Novel_007574	XLOC_078511	.	transcript	0	0.108513	22.23435444	2.07E-13	2.69E-11
Novel_007518	XLOC_077877	.	transcript	0.792788	0.007663	-6.356840808	0.000161	0.00797
Novel_007513	XLOC_077857	.	transcript	0	0.07427	20.30749451	2.08E-11	1.35E-09
Novel_007512	XLOC_077857	.	transcript	0	0.365413	22.73641728	5.86E-14	1.10E-11
Novel_007494	XLOC_077640	.	transcript	0.121431	0	-22.96144821	3.35E-14	7.29E-12
Novel_007436	XLOC_077238	.	transcript	0.195382	0	-23.56797604	6.93E-15	2.33E-12
Novel_007435	XLOC_077238	.	transcript	0.227991	0	-23.18830378	1.85E-14	4.95E-12
Novel_007426	XLOC_077133	.	transcript	0.082603	0	-23.06154157	2.59E-14	6.19E-12
Novel_007315	XLOC_075795	.	transcript	0	0.082576	6.235856502	0.000351	0.016325
Novel_007312	XLOC_075754	.	transcript	0	10.62231	24.75040111	2.88E-16	2.32E-13
Novel_007310	XLOC_075754	.	transcript	0	66.95437	27.45338921	1.17E-19	5.95E-16
Novel_007267	XLOC_075198	.	transcript	0.062885	0	-21.34827649	1.87E-12	1.51E-10
Novel_007254	XLOC_075050	.	transcript	0.757631	0.18141	-2.150814858	6.93E-05	0.003644
Novel_007229	XLOC_074801	.	transcript	0.048388	0	-22.35625291	1.55E-13	2.17E-11
Novel_007223	XLOC_074693	.	transcript	0.12091	2.52E-05	-10.27030951	0.00025	0.012027
Novel_007207	XLOC_074347	.	transcript	0	0.131071	20.7910991	6.60E-12	4.65E-10
Novel_007196	XLOC_074218	.	transcript	0.001244	0.080387	5.374802854	0.000767	0.032748
Novel_007198	XLOC_074218	.	transcript	0	0.115696	21.27775201	2.13E-12	1.67E-10
Novel_007189	XLOC_074169	.	transcript	0	0.098494	20.02608322	3.99E-11	2.48E-09
Novel_007157	XLOC_073472	.	transcript	0.087932	0	-22.00421936	3.75E-13	4.10E-11
Novel_007112	XLOC_072711	.	transcript	0	0.212588	20.87404528	5.57E-12	3.97E-10
Novel_007058	XLOC_072258	.	transcript	0.334344	0.003303	-6.782879187	0.000148	0.007405
Novel_007025	XLOC_072130	.	transcript	0.105592	0.00093	-6.511057242	0.001085	0.044426
Novel_007027	XLOC_072130	.	transcript	1.393347	8.438037	2.639687067	0.001159	0.04724
Novel_007017	XLOC_072117	.	transcript	0	4.119832	26.26431321	4.00E-18	9.87E-15

Novel_006987	XLOC_072070	.	transcript	0.485626	0	-24.16815167	1.40E-15	7.57E-13
Novel_006982	XLOC_072013	.	transcript	0.214085	0	-22.38969334	1.43E-13	2.07E-11
Novel_006970	XLOC_071855	.	transcript	0	0.064605	20.76873209	7.13E-12	4.99E-10
Novel_006936	XLOC_071731	.	transcript	0	0.053043	21.05103258	3.66E-12	2.74E-10
Novel_006906	XLOC_071656	.	transcript	0	0.09294	21.76100218	6.63E-13	6.38E-11
Novel_006891	XLOC_071544	.	transcript	0.100781	0	-22.56023463	9.31E-14	1.52E-11
Novel_006835	XLOC_071363	.	transcript	0.144205	0	-21.97935451	3.99E-13	4.31E-11
Novel_006819	XLOC_071309	.	transcript	0.328835	0	-22.85141488	4.44E-14	8.89E-12
Novel_006818	XLOC_071302	.	transcript	0	0.095329	22.0589601	3.19E-13	3.67E-11
Novel_006778	XLOC_070620	.	transcript	0	0.147188	7.785375373	3.40E-06	0.000197
Novel_006766	XLOC_070511	.	transcript	0.292574	0	-23.0310398	2.80E-14	6.47E-12
Novel_006744	XLOC_070040	.	transcript	0	0.062095	20.435114	1.55E-11	1.02E-09
Novel_006719	XLOC_069941	.	transcript	0.194413	0	-23.04146719	2.73E-14	6.40E-12
Novel_006655	XLOC_069583	.	transcript	0.618148	0	-23.80018684	3.76E-15	1.52E-12
Novel_006651	XLOC_069441	.	transcript	0	0.048023	20.05380525	3.75E-11	2.34E-09
Novel_006642	XLOC_069402	.	transcript	0.128623	0	-24.74558498	2.93E-16	2.32E-13
Novel_006621	XLOC_069325	.	transcript	0.220325	0	-23.55329549	7.21E-15	2.38E-12
Novel_006598	XLOC_068912	.	transcript	0.224721	0	-9.032701429	0.00019	0.009324
Novel_006588	XLOC_068890	.	transcript	0	0.011208	5.186017196	1.45E-05	0.000809
Novel_006583	XLOC_068850	.	transcript	1.309325	0	-27.1847446	2.63E-19	1.26E-15
Novel_006493	XLOC_066592	.	transcript	0	0.096615	21.86232297	5.18E-13	5.30E-11
Novel_006495	XLOC_066592	.	transcript	0	0.088856	22.04739148	3.29E-13	3.74E-11
Novel_006484	XLOC_066185	.	transcript	0.079261	0	-23.5329253	7.60E-15	2.48E-12
Novel_006481	XLOC_066185	.	transcript	0.078122	0	-8.39976636	1.94E-05	0.001074
Novel_006478	XLOC_066156	.	transcript	0.230761	0.616019	1.361894388	6.30E-05	0.003333
Novel_006450	XLOC_066003	.	transcript	0.234774	0	-8.871654055	0.0002	0.009783
Novel_006446	XLOC_066003	.	transcript	0	0.051181	20.83209308	6.15E-12	4.36E-10
Novel_006385	XLOC_065664	.	transcript	0	0.16867	20.83277676	6.14E-12	4.36E-10
Novel_006384	XLOC_065664	.	transcript	0	0.243083	21.43896792	1.45E-12	1.21E-10
Novel_006324	XLOC_065384	.	transcript	0.031218	0	-21.60616845	1.00E-12	8.92E-11
Novel_006301	XLOC_065300	.	transcript	0.545009	0	-10.0274775	5.61E-19	1.97E-15
Novel_006277	XLOC_065224	.	transcript	0	0.113759	22.62898055	7.70E-14	1.32E-11
Novel_006259	XLOC_065167	.	transcript	0	0.994093	24.83603507	2.28E-16	2.02E-13
Novel_006239	XLOC_065104	.	transcript	0	0.231296	21.91934821	4.50E-13	4.74E-11
Novel_006167	XLOC_064752	.	transcript	0.130394	0	-21.56942554	1.09E-12	9.60E-11
Novel_006161	XLOC_064660	.	transcript	0	0.348194	9.090707606	0.000175	0.008617
Novel_006162	XLOC_064660	.	transcript	0	0.147498	21.72717415	7.21E-13	6.80E-11
Novel_006156	XLOC_064443	.	transcript	0	0.061663	21.1228402	3.09E-12	2.33E-10

Novel_006144	XLOC_064064	.	transcript	0.120124	0	-21.86532585	5.28E-13	5.37E-11
Novel_006135	XLOC_064009	.	transcript	0.318597	0	-22.76478564	5.54E-14	1.05E-11
Novel_006084	XLOC_063664	.	transcript	0	0.100748	20.8373419	6.07E-12	4.31E-10
Novel_006058	XLOC_063470	.	transcript	0	0.146046	8.370061285	0.000434	0.019654
Novel_006059	XLOC_063470	.	transcript	0	0.034139	20.05380525	3.75E-11	2.34E-09
Novel_006006	XLOC_062892	.	transcript	0.06572	0	-21.88958244	4.98E-13	5.15E-11
Novel_005986	XLOC_062723	.	transcript	0	0.057522	20.54957812	1.19E-11	7.97E-10
Novel_005980	XLOC_062680	.	transcript	0.076718	0	-22.24334319	2.07E-13	2.69E-11
Novel_005951	XLOC_062486	.	transcript	0.712019	0	-23.66287365	5.39E-15	1.95E-12
Novel_005946	XLOC_062466	.	transcript	0.157728	0	-23.32090733	1.32E-14	3.74E-12
Novel_005913	XLOC_061972	.	transcript	0.226164	4.92E-05	-10.18045518	0.000769	0.032795
Novel_005854	XLOC_061351	.	transcript	0	0.161765	20.5338615	1.24E-11	8.24E-10
Novel_005841	XLOC_060948	.	transcript	0.099396	0	-21.74364395	7.13E-13	6.75E-11
Novel_005830	XLOC_060882	.	transcript	0	0.238979	9.326811492	8.72E-05	0.004524
Novel_005765	XLOC_060615	.	transcript	0	0.249099	6.757129113	7.10E-05	0.003729
Novel_005744	XLOC_060235	.	transcript	0	0.027644	20.17391609	2.84E-11	1.81E-09
Novel_005710	XLOC_059944	.	transcript	0	0.146499	22.94500059	3.45E-14	7.45E-12
Novel_005690	XLOC_059537	.	transcript	0.255359	0	-8.828487772	2.04E-07	1.22E-05
Novel_005670	XLOC_059304	.	transcript	0	0.140941	22.09898561	2.89E-13	3.43E-11
Novel_005632	XLOC_059086	.	transcript	0	0.251443	8.998195968	0.000204	0.009961
Novel_005629	XLOC_059086	.	transcript	0	0.054201	19.26680722	2.02E-10	1.24E-08
Novel_005628	XLOC_059078	.	transcript	0	0.713861	7.970300001	0.000821	0.034755
Novel_005617	XLOC_058888	.	transcript	0	0.047587	21.17711015	2.70E-12	2.07E-10
Novel_005607	XLOC_058724	.	transcript	0	0.058893	20.45185691	1.49E-11	9.89E-10
Novel_005578	XLOC_058130	.	transcript	0.170426	0	-21.74918584	7.04E-13	6.71E-11
Novel_005576	XLOC_058130	.	transcript	0	0.160501	6.800675506	0.000472	0.021212
Novel_005577	XLOC_058130	.	transcript	0	0.268896	21.24329181	2.32E-12	1.80E-10
Novel_005515	XLOC_057472	.	transcript	0.135607	0	-22.47476596	1.16E-13	1.76E-11
Novel_005504	XLOC_057324	.	transcript	0.058583	0	-23.17117327	1.95E-14	5.14E-12
Novel_005424	XLOC_056856	.	transcript	0	0.039773	21.88915127	4.85E-13	5.05E-11
Novel_005420	XLOC_056829	.	transcript	0	0.393258	22.50502806	1.05E-13	1.63E-11
Novel_005336	XLOC_056106	.	transcript	0.03688	0	-22.09842173	2.97E-13	3.47E-11
Novel_005320	XLOC_056082	.	transcript	0.072592	0	-22.13151446	2.73E-13	3.30E-11
Novel_005276	XLOC_055340	.	transcript	0.083509	0	-7.397413951	3.58E-05	0.001939
Novel_005277	XLOC_055340	.	transcript	0.000526	0.233215	7.603399639	3.89E-07	2.31E-05
Novel_005273	XLOC_055340	.	transcript	0	0.077872	21.03195629	3.83E-12	2.85E-10
Novel_005257	XLOC_055181	.	transcript	0.033693	0	-20.62213642	1.02E-11	6.92E-10
Novel_005211	XLOC_054449	.	transcript	0	0.061471	21.89399349	4.79E-13	5.00E-11

Novel_005192	XLOC_054131	.	transcript	0	0.067925	20.21392846	2.59E-11	1.66E-09
Novel_005154	XLOC_053537	.	transcript	0.196829	0	-22.09842173	2.97E-13	3.47E-11
Novel_005148	XLOC_053522	.	transcript	0.243298	0	-22.08672965	3.05E-13	3.55E-11
Novel_005120	XLOC_053330	.	transcript	0.14391	0	-8.860476412	0.000293	0.013945
Novel_005118	XLOC_053330	.	transcript	0.126677	0	-8.665877888	0.000829	0.034991
Novel_005034	XLOC_052826	.	transcript	0	0.251875	23.48705694	8.49E-15	2.71E-12
Novel_005023	XLOC_052778	.	transcript	0	0.073707	20.88975953	5.36E-12	3.84E-10
Novel_004974	XLOC_052485	.	transcript	0.106732	0	-22.28489384	1.86E-13	2.48E-11
Novel_004961	XLOC_052336	.	transcript	0	0.155051	20.63846691	9.68E-12	6.62E-10
Novel_004951	XLOC_052286	.	transcript	0.022836	0.799071	5.039568297	0.00029	0.013777
Novel_004952	XLOC_052286	.	transcript	0	0.024048	20.47369755	1.42E-11	9.43E-10
Novel_004956	XLOC_052286	.	transcript	0	0.063798	21.88837379	4.86E-13	5.05E-11
Novel_004921	XLOC_051739	.	transcript	0	0.071055	21.28577554	2.09E-12	1.65E-10
Novel_004918	XLOC_051738	.	transcript	0	0.040496	21.40419851	1.56E-12	1.29E-10
Novel_004875	XLOC_051245	.	transcript	1.728257	0	-11.36635055	0.000173	0.00852
Novel_004783	XLOC_050645	.	transcript	0	0.278396	22.27657046	1.86E-13	2.48E-11
Novel_004779	XLOC_050497	.	transcript	0.106848	0	-23.46276417	9.14E-15	2.85E-12
Novel_004741	XLOC_049838	.	transcript	0	0.051655	21.08969727	3.34E-12	2.51E-10
Novel_004732	XLOC_049798	.	transcript	0	0.105299	19.61585629	9.34E-11	5.73E-09
Novel_004730	XLOC_049771	.	transcript	0.143683	0.001765	-6.466981618	0.000278	0.013273
Novel_004669	XLOC_049608	.	transcript	0.068085	0	-21.44086638	1.49E-12	1.25E-10
Novel_004573	XLOC_047659	.	transcript	0.034579	0	-22.0976792	2.97E-13	3.48E-11
Novel_004515	XLOC_047338	.	transcript	0.148084	0	-22.00908792	3.70E-13	4.07E-11
Novel_004483	XLOC_047156	.	transcript	0.145527	0	-21.87760587	5.13E-13	5.26E-11
Novel_004447	XLOC_046985	.	transcript	0.033114	0	-6.077438279	0.000399	0.018268
Novel_004452	XLOC_046985	.	transcript	0	0.190475	23.56483945	6.93E-15	2.33E-12
Novel_004418	XLOC_046809	.	transcript	0.329818	0	-23.74196612	4.38E-15	1.67E-12
Novel_004414	XLOC_046809	.	transcript	0.001689	0.140962	4.912489579	0.001231	0.049826
Novel_004419	XLOC_046809	.	transcript	0	0.559333	23.4806731	8.64E-15	2.73E-12
Novel_004381	XLOC_046723	.	transcript	0	0.027357	20.21392846	2.59E-11	1.66E-09
Novel_004379	XLOC_046697	.	transcript	0.140907	0	-21.63847178	9.24E-13	8.41E-11
Novel_004336	XLOC_046297	.	transcript	0.119044	0	-21.6536291	8.90E-13	8.15E-11
Novel_004299	XLOC_045877	.	transcript	0.003827	0.157789	5.104167644	0.000526	0.023386
Novel_004280	XLOC_045820	.	transcript	0.143376	0	-22.4995765	1.09E-13	1.67E-11
Novel_004266	XLOC_045756	.	transcript	0.042614	0	-22.15940348	2.55E-13	3.15E-11
Novel_004258	XLOC_045743	.	transcript	0.321279	0.003362	-6.505671549	3.22E-05	0.001746
Novel_004210	XLOC_045354	.	transcript	0.180734	0	-7.054886133	0.000253	0.012181
Novel_004194	XLOC_045044	.	transcript	0.165292	0	-22.30228021	1.78E-13	2.42E-11

Novel_004156	XLOC_044866	.	transcript	0.058589	0	-21.34211722	1.90E-12	1.53E-10
Novel_004148	XLOC_044816	.	transcript	0.246009	0	-23.46474563	9.09E-15	2.85E-12
Novel_004147	XLOC_044816	.	transcript	0.06862	0	-21.75727108	6.90E-13	6.61E-11
Novel_004086	XLOC_044185	.	transcript	0.123557	4.40E-06	-10.39754894	0.000115	0.005871
Novel_004082	XLOC_044185	.	transcript	0.377446	5.26E-05	-10.1994189	4.81E-05	0.002576
Novel_003961	XLOC_043355	.	transcript	0.049887	0	-21.57381065	1.08E-12	9.54E-11
Novel_003898	XLOC_041850	.	transcript	0	0.111384	20.435114	1.55E-11	1.02E-09
Novel_003787	XLOC_040354	.	transcript	0.075546	0	-21.94782694	4.31E-13	4.59E-11
Novel_003772	XLOC_039795	.	transcript	0	0.148249	20.79813197	6.66E-12	4.68E-10
Novel_003756	XLOC_039767	.	transcript	0.541803	0.049407	-3.458202432	0.000386	0.017796
Novel_003725	XLOC_039498	.	transcript	0.119399	0	-21.51578744	1.25E-12	1.06E-10
Novel_003732	XLOC_039498	.	transcript	0	0.082395	18.40339003	1.29E-09	7.84E-08
Novel_003674	XLOC_038985	.	transcript	0.077948	0.175794	1.123148081	0.000397	0.018184
Novel_003658	XLOC_038870	.	transcript	0.689226	0	-22.53711471	9.88E-14	1.58E-11
Novel_003638	XLOC_038545	.	transcript	0.181353	0	-21.88751101	5.01E-13	5.15E-11
Novel_003620	XLOC_038438	.	transcript	0.225786	0.003601	-5.178164266	0.000281	0.013393
Novel_003529	XLOC_038408	.	transcript	10.01784	0.937835	-3.500161873	0.000907	0.037969
Novel_003525	XLOC_038347	.	transcript	0.10813	0	-22.55533211	9.37E-14	1.52E-11
Novel_003519	XLOC_038269	.	transcript	0.544472	0	-23.72077558	4.64E-15	1.73E-12
Novel_003488	XLOC_038050	.	transcript	0.038731	0	-21.61620533	9.75E-13	8.76E-11
Novel_003483	XLOC_037912	.	transcript	0	0.048359	20.26381283	2.31E-11	1.49E-09
Novel_003464	XLOC_037508	.	transcript	0.085637	0	-22.69455726	6.55E-14	1.19E-11
Novel_003451	XLOC_037411	.	transcript	0	0.655133	24.03974409	1.97E-15	9.63E-13
Novel_003448	XLOC_037411	.	transcript	0	1.863612	25.51404646	3.43E-17	4.94E-14
Novel_003450	XLOC_037411	.	transcript	0	2.338425	25.70920787	1.97E-17	3.34E-14
Novel_003427	XLOC_036883	.	transcript	0.199242	0	-23.10373744	2.32E-14	5.71E-12
Novel_003364	XLOC_036213	.	transcript	0.056497	0	-22.59693975	8.36E-14	1.39E-11
Novel_003358	XLOC_035935	.	transcript	0.075374	0	-22.32506403	1.68E-13	2.31E-11
Novel_003357	XLOC_035935	.	transcript	0.056517	0	-22.02009014	3.60E-13	4.01E-11
Novel_003354	XLOC_035911	.	transcript	0	0.144679	8.938547728	3.66E-07	2.17E-05
Novel_003346	XLOC_035814	.	transcript	0.542838	0	-23.19931866	1.81E-14	4.89E-12
Novel_003350	XLOC_035814	.	transcript	0	0.108072	19.93114645	4.96E-11	3.06E-09
Novel_003349	XLOC_035814	.	transcript	0	0.386657	21.55692948	1.09E-12	9.59E-11
Novel_003315	XLOC_035223	.	transcript	0	0.04707	21.36655171	1.72E-12	1.41E-10
Novel_003307	XLOC_035008	.	transcript	0.412648	0	-22.86630709	4.26E-14	8.63E-12
Novel_003237	XLOC_034101	.	transcript	0.132433	0	-23.60018564	6.37E-15	2.20E-12
Novel_003199	XLOC_033781	.	transcript	0.664244	0	-24.22880679	1.19E-15	6.73E-13
Novel_003194	XLOC_033781	.	transcript	0	0.257713	22.9968992	3.02E-14	6.70E-12

Novel_003192	XLOC_033781	.	transcript	0	0.575809	23.81491177	3.59E-15	1.47E-12
Novel_003190	XLOC_033750	.	transcript	0.094056	0	-22.31859631	1.71E-13	2.34E-11
Novel_003177	XLOC_033669	.	transcript	0	0.073733	20.5575245	1.17E-11	7.84E-10
Novel_003160	XLOC_033539	.	transcript	0.129555	0	-21.34211722	1.90E-12	1.53E-10
Novel_003124	XLOC_033383	.	transcript	0	0.065525	20.92004779	4.99E-12	3.60E-10
Novel_003119	XLOC_033361	.	transcript	0	0.264065	9.490479074	6.01E-05	0.003194
Novel_003106	XLOC_033351	.	transcript	0	0.032836	20.46250269	1.46E-11	9.65E-10
Novel_003058	XLOC_033073	.	transcript	0	0.063187	20.66696553	9.06E-12	6.25E-10
Novel_003046	XLOC_032911	.	transcript	0.11293	0	-22.3730347	1.49E-13	2.11E-11
Novel_003045	XLOC_032911	.	transcript	0	0.309699	8.823928447	0.000285	0.013558
Novel_003029	XLOC_032812	.	transcript	0	0.097442	21.60114867	9.79E-13	8.78E-11
Novel_003021	XLOC_032749	.	transcript	0.043621	0	-22.14599572	2.64E-13	3.21E-11
Novel_002995	XLOC_032383	.	transcript	0	0.104783	20.6001563	1.06E-11	7.12E-10
Novel_002975	XLOC_032212	.	transcript	0.334795	0	-23.98220324	2.32E-15	1.07E-12
Novel_002972	XLOC_032206	.	transcript	0	0.197947	6.653424026	5.34E-05	0.002846
Novel_002932	XLOC_031970	.	transcript	0.32378	0	-8.102500105	0.000768	0.032782
Novel_002905	XLOC_031742	.	transcript	0.241211	0	-22.48696571	1.12E-13	1.71E-11
Novel_002895	XLOC_031583	.	transcript	0	0.124156	21.25577178	2.25E-12	1.75E-10
Novel_002877	XLOC_031184	.	transcript	0.309921	0	-23.24420351	1.61E-14	4.43E-12
Novel_002848	XLOC_031099	.	transcript	0	0.06089	20.22715959	2.52E-11	1.61E-09
Novel_002803	XLOC_030661	.	transcript	0	0.044186	7.349111027	1.36E-05	0.00076
Novel_002794	XLOC_030543	.	transcript	0.097006	0	-21.82059253	5.90E-13	5.87E-11
Novel_002760	XLOC_029872	.	transcript	0.370266	0.000134	-7.886891541	0.000125	0.006314
Novel_002753	XLOC_029673	.	transcript	1.236277	2.830109	1.170076625	0.000161	0.00799
Novel_002732	XLOC_029535	.	transcript	0	0.122391	20.66524957	9.09E-12	6.27E-10
Novel_002725	XLOC_029503	.	transcript	0	0.050057	21.98097541	3.87E-13	4.22E-11
Novel_002666	XLOC_028700	.	transcript	0	0.115164	21.20336183	2.55E-12	1.96E-10
Novel_002609	XLOC_028416	.	transcript	0.194349	0	-22.55408308	9.46E-14	1.54E-11
Novel_002582	XLOC_028305	.	transcript	0.088818	0	-22.02009014	3.60E-13	4.01E-11
Novel_002579	XLOC_028258	.	transcript	0.070556	0	-22.02009014	3.60E-13	4.01E-11
Novel_002575	XLOC_028113	.	transcript	0	0.089505	22.2675695	1.90E-13	2.52E-11
Novel_002535	XLOC_027535	.	transcript	0.294857	0.007881	-5.179893084	2.05E-06	0.000119
Novel_002524	XLOC_027486	.	transcript	0	0.043267	19.93114645	4.96E-11	3.06E-09
Novel_002504	XLOC_027288	.	transcript	0.339187	0	-9.652643607	7.53E-05	0.003935
Novel_002486	XLOC_027231	.	transcript	0.079667	0	-22.33828255	1.63E-13	2.26E-11
Novel_002469	XLOC_027187	.	transcript	0	0.596051	23.62840106	5.86E-15	2.08E-12
Novel_002452	XLOC_027152	.	transcript	0	0.169906	21.8534665	5.29E-13	5.37E-11
Novel_002430	XLOC_026899	.	transcript	0.196271	0	-22.69359408	6.64E-14	1.20E-11

Novel_002429	XLOC_026899	.	transcript	0	0.097669	19.70844674	7.60E-11	4.66E-09
Novel_002422	XLOC_026884	.	transcript	0	4.344346	23.5135468	7.93E-15	2.56E-12
Novel_002407	XLOC_026788	.	transcript	0	0.051636	21.63824963	8.94E-13	8.17E-11
Novel_002398	XLOC_026722	.	transcript	0.551238	0	-25.69338561	2.07E-17	3.37E-14
Novel_002336	XLOC_026067	.	transcript	0.168436	0	-8.774697944	0.000339	0.01586
Novel_002328	XLOC_025705	.	transcript	0.40631	0	-27.01821836	4.34E-19	1.63E-15
Novel_002326	XLOC_025705	.	transcript	0.019499	0	-22.84452996	4.52E-14	8.98E-12
Novel_002329	XLOC_025705	.	transcript	0.13449	0.00042	-8.047688674	0.000247	0.011922
Novel_002324	XLOC_025705	.	transcript	0.007057	0.216124	4.912379572	6.47E-05	0.003411
Novel_002309	XLOC_025705	.	transcript	7.40E-06	0.120223	11.02391199	0.000258	0.012396
Novel_002305	XLOC_025705	.	transcript	0	0.096345	23.33774845	1.25E-14	3.57E-12
Novel_002302	XLOC_025705	.	transcript	0	0.07649	24.71979433	3.13E-16	2.40E-13
Novel_002318	XLOC_025705	.	transcript	0	0.207155	25.12576976	1.02E-16	1.12E-13
Novel_002287	XLOC_025509	.	transcript	0.04298	0	-22.53727339	9.86E-14	1.58E-11
Novel_002271	XLOC_025435	.	transcript	0.359874	0	-24.48294942	6.01E-16	3.96E-13
Novel_002243	XLOC_025305	.	transcript	0.149581	0	-24.84190142	2.25E-16	2.02E-13
Novel_002251	XLOC_025305	.	transcript	0	0.282341	8.740371184	2.92E-06	0.000169
Novel_002214	XLOC_024870	.	transcript	0.459894	0	-23.37363269	1.15E-14	3.38E-12
Novel_002171	XLOC_024256	.	transcript	2.950307	0	-26.48416649	2.11E-18	6.07E-15
Novel_002170	XLOC_024256	.	transcript	0	0.374697	22.65491511	7.21E-14	1.27E-11
Novel_002166	XLOC_024001	.	transcript	0	0.269222	23.12806621	2.16E-14	5.42E-12
Novel_002153	XLOC_023907	.	transcript	0.317285	0	-23.79267147	3.83E-15	1.55E-12
Novel_002085	XLOC_023328	.	transcript	0	0.292697	22.63143621	7.65E-14	1.32E-11
Novel_002013	XLOC_022674	.	transcript	0	0.051123	20.74443933	7.55E-12	5.27E-10
Novel_001996	XLOC_022605	.	transcript	0	0.065028	6.701030625	0.000107	0.005461
Novel_001937	XLOC_022058	.	transcript	0	0.034147	20.34410577	1.92E-11	1.25E-09
Novel_001903	XLOC_021809	.	transcript	0.066615	0	-22.74087431	5.89E-14	1.10E-11
Novel_001910	XLOC_021809	.	transcript	0.044229	0	-22.45600873	1.21E-13	1.83E-11
Novel_001900	XLOC_021807	.	transcript	0.232604	0	-10.04809654	2.88E-08	1.74E-06
Novel_001894	XLOC_021730	.	transcript	0	0.0449	20.79038809	6.78E-12	4.76E-10
Novel_001896	XLOC_021730	.	transcript	0	0.044103	20.82353824	6.27E-12	4.43E-10
Novel_001727	XLOC_019030	.	transcript	0	0.11053	23.25334067	1.56E-14	4.32E-12
Novel_001695	XLOC_018810	.	transcript	0	0.067197	20.41208246	1.64E-11	1.08E-09
Novel_001692	XLOC_018802	.	transcript	0	12.91422	24.86417971	2.11E-16	1.94E-13
Novel_001677	XLOC_018510	.	transcript	0	0.135433	22.4306408	1.27E-13	1.89E-11
Novel_001674	XLOC_018449	.	transcript	0.031486	0.25648	2.993696502	0.000424	0.019291
Novel_001671	XLOC_018447	.	transcript	0	0.523557	23.93493724	2.61E-15	1.17E-12
Novel_001576	XLOC_017848	.	transcript	0.040996	0	-22.55533211	9.37E-14	1.52E-11

Novel_001575	XLOC_017848	.	transcript	0.039014	0	-21.88751101	5.01E-13	5.15E-11
Novel_001557	XLOC_017756	.	transcript	0.168679	0	-21.40301864	1.64E-12	1.35E-10
Novel_001534	XLOC_017484	.	transcript	0	0.189668	8.126365334	0.000987	0.040877
Novel_001456	XLOC_016987	.	transcript	0.643772	0	-25.13197468	1.01E-16	1.12E-13
Novel_001436	XLOC_016556	.	transcript	0	0.097902	21.1946609	2.60E-12	2.00E-10
Novel_001413	XLOC_016247	.	transcript	0.083012	0	-22.708443	6.40E-14	1.17E-11
Novel_001415	XLOC_016247	.	transcript	0	0.205863	22.20655226	2.22E-13	2.82E-11
Novel_001410	XLOC_016247	.	transcript	0	0.16322	22.90976008	3.77E-14	7.95E-12
Novel_001356	XLOC_015994	.	transcript	0.077667	0	-22.02813841	3.53E-13	3.97E-11
Novel_001377	XLOC_015994	.	transcript	0.06659	0	-8.831758181	8.04E-05	0.004182
Novel_001335	XLOC_015994	.	transcript	0.026834	8.00E-07	-7.716049036	0.000739	0.031719
Novel_001359	XLOC_015994	.	transcript	2.20E-06	0.008352	8.059404503	6.23E-05	0.003302
Novel_001349	XLOC_015994	.	transcript	7.60E-06	0.085333	11.85293981	8.98E-05	0.004647
Novel_001383	XLOC_015994	.	transcript	0	0.004762	20.87395902	5.57E-12	3.97E-10
Novel_001374	XLOC_015994	.	transcript	0	0.032882	21.4102653	1.55E-12	1.29E-10
Novel_001376	XLOC_015994	.	transcript	0	0.032835	21.54466716	1.12E-12	9.79E-11
Novel_001379	XLOC_015994	.	transcript	0	0.046319	21.7100619	7.51E-13	7.03E-11
Novel_001360	XLOC_015994	.	transcript	0	0.008701	22.41703864	1.31E-13	1.94E-11
Novel_001329	XLOC_015994	.	transcript	0	0.04261	24.72250627	3.11E-16	2.40E-13
Novel_001339	XLOC_015994	.	transcript	0	0.097583	24.93037602	1.75E-16	1.74E-13
Novel_001367	XLOC_015994	.	transcript	0	0.063068	25.3843184	4.95E-17	6.44E-14
Novel_001308	XLOC_015723	.	transcript	0	0.119651	21.8242727	5.68E-13	5.68E-11
Novel_001245	XLOC_014484	.	transcript	0.126344	0	-23.77538971	4.01E-15	1.57E-12
Novel_001234	XLOC_014275	.	transcript	0	0.286521	22.81642941	4.79E-14	9.35E-12
Novel_001153	XLOC_013475	.	transcript	0.456123	0	-8.792452999	0.001078	0.044198
Novel_001145	XLOC_013424	.	transcript	0.258982	0	-22.75956747	5.62E-14	1.06E-11
Novel_001143	XLOC_013422	.	transcript	0	0.059746	20.05943405	3.70E-11	2.32E-09
Novel_001082	XLOC_012797	.	transcript	0.013185	0.402929	4.755065233	0.000163	0.008083
Novel_000977	XLOC_012099	.	transcript	0	0.11691	23.16016709	1.98E-14	5.18E-12
Novel_000968	XLOC_012019	.	transcript	0	0.264806	20.6031881	1.00E-11	6.84E-10
Novel_000956	XLOC_011838	.	transcript	0	0.09063	20.35821365	1.86E-11	1.22E-09
Novel_000957	XLOC_011838	.	transcript	0	0.367002	20.96842338	4.29E-12	3.15E-10
Novel_000948	XLOC_011657	.	transcript	0.10624	0	-21.62005021	9.66E-13	8.70E-11
Novel_000928	XLOC_011536	.	transcript	0.176493	0	-21.94562525	4.34E-13	4.61E-11
Novel_000919	XLOC_011405	.	transcript	0.030171	0	-21.88251912	5.07E-13	5.21E-11
Novel_000915	XLOC_011372	.	transcript	0	0.218155	21.55178575	1.10E-12	9.66E-11
Novel_000894	XLOC_011315	.	transcript	0	0.093008	20.90787219	5.14E-12	3.69E-10
Novel_000889	XLOC_011281	.	transcript	0	0.013363	20.03717251	3.89E-11	2.42E-09

Novel_000881	XLOC_011196	.	transcript	0	0.506357	8.278244589	0.000982	0.040707
Novel_000821	XLOC_010882	.	transcript	0.198125	0	-23.34989397	1.23E-14	3.52E-12
Novel_000818	XLOC_010882	.	transcript	0.001303	0.134987	5.794313068	0.000373	0.017248
Novel_000712	XLOC_009273	.	transcript	0.074223	0	-22.28945712	1.84E-13	2.47E-11
Novel_000658	XLOC_007997	.	transcript	0.001791	0.043047	3.931290557	0.00072	0.030967
Novel_000607	XLOC_007097	.	transcript	0	0.099871	8.147786176	6.93E-06	0.000396
Novel_000583	XLOC_006610	.	transcript	0.304431	0	-7.576374357	9.31E-06	0.000527
Novel_000524	XLOC_005657	.	transcript	0.028144	0	-22.47064157	1.17E-13	1.77E-11
Novel_000517	XLOC_005379	.	transcript	0.225415	0	-23.58180569	6.68E-15	2.29E-12
Novel_000467	XLOC_005065	.	transcript	0.114859	0.012899	-2.918274707	0.000506	0.022561
Novel_000424	XLOC_004566	.	transcript	0.259116	0	-22.37343095	1.49E-13	2.11E-11
Novel_000417	XLOC_004523	.	transcript	0	0.323459	22.26460609	1.91E-13	2.52E-11
Novel_000394	XLOC_004290	.	transcript	0.334987	0	-25.11758042	1.05E-16	1.13E-13
Novel_000351	XLOC_003925	.	transcript	0.086717	0	-22.27832868	1.89E-13	2.51E-11
Novel_000349	XLOC_003925	.	transcript	0.327352	0.092829	-1.852926376	0.000229	0.011139
Novel_000336	XLOC_003874	.	transcript	0.285202	0	-23.41067879	1.05E-14	3.15E-12
Novel_000307	XLOC_003270	.	transcript	0.104127	0	-22.35625291	1.55E-13	2.17E-11
Novel_000284	XLOC_003222	.	transcript	0	0.037953	20.74443933	7.55E-12	5.27E-10
Novel_000237	XLOC_002464	.	transcript	0.126723	0	-22.1101321	2.88E-13	3.42E-11
Novel_000225	XLOC_002419	.	transcript	0.064517	0	-22.15277366	2.59E-13	3.19E-11
Novel_000217	XLOC_002415	.	transcript	0.119696	0	-22.0531449	3.30E-13	3.76E-11
Novel_000204	XLOC_002405	.	transcript	0	0.021652	20.05380525	3.75E-11	2.34E-09
Novel_000207	XLOC_002405	.	transcript	0	0.044165	20.95299955	4.62E-12	3.36E-10
Novel_000147	XLOC_001855	.	transcript	0	1.753432	24.77970567	2.66E-16	2.19E-13
Novel_000120	XLOC_001707	.	transcript	0	0.092252	21.67741468	8.12E-13	7.52E-11
Novel_000102	XLOC_001531	.	transcript	0.122235	0	-23.10867885	2.29E-14	5.69E-12
Novel_000097	XLOC_001531	.	transcript	0.807298	0.001251	-8.46385103	0.000203	0.009919
Novel_000088	XLOC_001531	.	transcript	0	0.06725	22.71027466	6.24E-14	1.14E-11
Novel_000079	XLOC_001467	.	transcript	0.262574	0	-23.5731443	6.84E-15	2.32E-12
Novel_000073	XLOC_001263	.	transcript	0.219555	0.101595	-1.113318778	0.000592	0.025969
Novel_000033	XLOC_000428	.	transcript	0.057369	0	-22.0700679	3.18E-13	3.67E-11
Novel_000026	XLOC_000309	.	transcript	0	0.03879	20.82051972	6.31E-12	4.46E-10
Novel_000005	XLOC_000013	.	transcript	0	0.09746	20.65844754	9.24E-12	6.36E-10
XM_018041131	gene-ZSCAN20	ZSCAN20	transcript	0.72877	0	-25.88662766	1.19E-17	2.20E-14
XM_018046812	gene-ZNF783	ZNF783	transcript	0.066462	0	-21.53326404	1.19E-12	1.03E-10
XM_013976377	gene-ZNF711	ZNF711	transcript	0	0.829097	10.35881357	2.05E-05	0.001135
XM_018064916	gene-ZNF652	ZNF652	transcript	0.964819	0.14295	-2.813581687	0.000686	0.029655
XM_018045750	gene-ZNF644	ZNF644	transcript	0.427541	0.010299	-5.298218066	0.000264	0.012671

XM_018061466	gene-ZNF605	ZNF605	transcript	0.957262	0	-10.92656253	0.000306	0.014479
XM_018051145	gene-ZNF557	ZNF557	transcript	0.429581	0	-22.15940348	2.55E-13	3.15E-11
XM_018039517	gene-ZNF532	ZNF532	transcript	0.000309	0.190343	8.337732359	0.000613	0.026751
XM_013971160	gene-ZNF527	ZNF527	transcript	0.065476	0.565253	2.995062823	4.49E-06	0.000259
XM_013975149	gene-ZNF518A	ZNF518A	transcript	0.365416	1.85736	2.340136353	0.000452	0.020418
XM_013975150	gene-ZNF518A	ZNF518A	transcript	0.147514	1.316183	3.139898909	0.000557	0.024583
XM_018055495	gene-ZNF512	ZNF512	transcript	0	0.132166	21.29716036	2.04E-12	1.62E-10
XM_018050545	gene-ZNF496	ZNF496	transcript	1.929846	0.299843	-2.698702726	0.00079	0.033518
XM_018040289	gene-ZNF48	ZNF48	transcript	0.034161	0.321629	3.101387774	8.99E-06	0.000511
XM_005676869	gene-ZNF436	ZNF436	transcript	0.869275	0.033368	-4.686587911	4.08E-05	0.002193
XM_018051864	gene-ZNF395	ZNF395	transcript	0.000607	0.486456	9.476207857	0.000703	0.030285
XM_018054539	gene-ZNF219	ZNF219	transcript	0	0.189941	22.55479153	9.28E-14	1.52E-11
XM_018057873	gene-ZHX3	ZHX3	transcript	0.15047	0.000754	-6.608932389	0.000422	0.019184
XM_018049211	gene-ZGRF1	ZGRF1	transcript	0.301565	0	-24.15001519	1.48E-15	7.93E-13
XM_018057184	gene-ZEB1	ZEB1	transcript	0.01439	3.816749	7.973773373	0.000354	0.016451
XM_018047914	gene-YAF2	YAF2	transcript	2.096168	0.123856	-4.089641736	7.30E-05	0.003822
XM_018062518	gene-XRCC1	XRCC1	transcript	0.246406	0.867525	1.772776135	0.000506	0.022561
XM_018062520	gene-XRCC1	XRCC1	transcript	0.605725	2.672879	2.076388284	2.93E-05	0.001593
XM_018062519	gene-XRCC1	XRCC1	transcript	0.325811	1.464929	2.120886284	1.54E-05	0.00086
XM_018062521	gene-XRCC1	XRCC1	transcript	0.256976	1.176168	2.148576262	0.000199	0.009732
XM_018067241	gene-XIRP1	XIRP1	transcript	0	0.052675	20.99500159	4.18E-12	3.08E-10
XM_018044166	gene-XIAP	XIAP	transcript	0.270258	0	-23.88891196	2.96E-15	1.29E-12
XM_018043742	gene-XG	XG	transcript	0	0.411339	23.18448153	1.86E-14	4.97E-12
XM_018043743	gene-XG	XG	transcript	0	1.66231	24.66014777	3.68E-16	2.74E-13
XM_018055379	gene-WNT4	WNT4	transcript	0.273502	1.449893	2.398922749	2.28E-05	0.001256
XM_013976555	gene-WNK3	WNK3	transcript	0.19039	0.002231	-6.013430115	2.37E-05	0.001302
XM_018065029	gene-WIPF2	WIPF2	transcript	0.120105	0	-23.01374178	2.93E-14	6.56E-12
XM_018065396	gene-WDR45B	WDR45B	transcript	0.009912	0.144665	3.556235257	0.000542	0.024014
XM_018051734	gene-WDR4	WDR4	transcript	0.056953	0	-21.58668338	1.05E-12	9.27E-11
XM_018050486	gene-WDR18	WDR18	transcript	0.098151	0	-22.01685381	3.63E-13	4.02E-11
XM_013973964	gene-WASF2	WASF2	transcript	0	0.415268	9.478842922	6.14E-05	0.003257
XM_018045978	gene-WARS2	WARS2	transcript	1.100005	0.053459	-4.410794492	0.000825	0.034918
XM_013967055	gene-VCPKMT	VCPKMT	transcript	0.14677	0.002765	-4.528826102	0.000114	0.005823
XM_018059762	gene-USP28	USP28	transcript	0.263158	0	-23.54970785	7.28E-15	2.39E-12
XM_018059759	gene-USP28	USP28	transcript	0.104334	0	-22.41576294	1.34E-13	1.97E-11
XM_005689551	gene-USP2	USP2	transcript	0	0.498573	9.241982006	9.07E-05	0.004689
XM_018043658	gene-USP11	USP11	transcript	0	0.31761	21.8037034	5.92E-13	5.88E-11
XM_018049590	gene-USO1	USO1	transcript	5.557215	0	-27.55720159	8.54E-20	5.27E-16

XM_018061433	gene-ULK1	ULK1	transcript	0.302953	0	-23.974618	2.36E-15	1.07E-12
XM_018061434	gene-ULK1	ULK1	transcript	0	0.32403	23.04189475	2.69E-14	6.39E-12
XM_005686796	gene-UGP2	UGP2	transcript	0.146553	0	-21.66999139	8.55E-13	7.87E-11
XM_005690714	gene-UBE4B	UBE4B	transcript	0.044668	1.49951	5.089329937	0.000135	0.006827
XM_018052928	gene-UBE3D	UBE3D	transcript	0	0.94316	23.08959856	2.38E-14	5.78E-12
XM_018043652	gene-UBA1	UBA1	transcript	0	1.739481	24.90631178	1.88E-16	1.78E-13
XM_018057639	gene-TXLNA	TXLNA	transcript	1.890102	0.000967	-10.28109626	0.000635	0.027634
XM_018048633	gene-TULP3	TULP3	transcript	0.604702	0.002308	-7.136696819	9.48E-05	0.004888
XM_018053944	gene-TTLL5	TTLL5	transcript	0.157739	0	-22.95431203	3.41E-14	7.41E-12
XM_018045970	gene-TTF2	TTF2	transcript	0	0.128962	21.06719954	3.52E-12	2.65E-10
XR_001919091.	gene-TSHZ2	TSHZ2	transcript	0	0.148302	8.553853563	0.00039	0.017913
XM_018038261	gene-TSEN2	TSEN2	transcript	0.157354	1.359693	3.045502915	1.71E-05	0.000952
XM_018040227	gene-TRRAP	TRRAP	transcript	0.751369	0	-26.36587167	2.98E-18	7.80E-15
XM_018040234	gene-TRRAP	TRRAP	transcript	0.201053	0	-24.46585349	6.29E-16	4.12E-13
XM_018061500	gene-TRPC3	TRPC3	transcript	0.160043	0	-22.54991316	9.56E-14	1.55E-11
XM_005685642	gene-TRIM9	TRIM9	transcript	0.162599	0	-22.94075198	3.53E-14	7.58E-12
XM_018052688	gene-TRIM32	TRIM32	transcript	0	0.769336	22.98325019	3.11E-14	6.85E-12
XM_018061612	gene-TRIM2	TRIM2	transcript	0	0.152513	22.44627593	1.22E-13	1.83E-11
XM_018039091	gene-TRERF1	TRERF1	transcript	0	0.107876	21.3347761	1.86E-12	1.50E-10
XM_018066993	gene-TRAK1	TRAK1	transcript	0	0.064211	20.80042017	6.62E-12	4.66E-10
XM_018040186	gene-TRAF3IP1	TRAF3IP1	transcript	0.956612	0.029557	-4.870651722	0.000429	0.01943
XM_018047312	gene-TRA2A	TRA2A	transcript	0	0.30576	21.85438436	5.28E-13	5.37E-11
XM_005686368	gene-TPRKB	TPRKB	transcript	0.95832	0	-23.7379508	4.43E-15	1.68E-12
XM_005689575	gene-TPH1	TPH1	transcript	0.179969	0	-22.66389933	7.16E-14	1.27E-11
XM_013969273	gene-TPD52	TPD52	transcript	0.231163	0	-23.10017022	2.34E-14	5.71E-12
XM_018057743	gene-TP53INP2	TP53INP2	transcript	0	0.373961	22.78146488	5.22E-14	1.01E-11
XM_018057741	gene-TP53INP2	TP53INP2	transcript	0	0.351813	23.03179007	2.76E-14	6.45E-12
XM_018038907	gene-TNXB	TNXB	transcript	0	0.03631	21.18708002	2.65E-12	2.03E-10
XM_018063111	gene-TNNT1	TNNT1	transcript	0	0.692102	21.81306245	5.84E-13	5.83E-11
XM_013964598	gene-TNIK	TNIK	transcript	0.018504	1.03562	5.686262536	4.11E-05	0.002208
XM_018050942	gene-TNFSF9	TNFSF9	transcript	0.160635	0	-22.02009014	3.60E-13	4.01E-11
XM_013971985	gene-TNFRSF13E	TNFRSF13	transcript	2.259768	0	-24.77976802	5.39E-21	5.18E-17
XM_018052682	gene-TNC	TNC	transcript	4.429173	0	-27.85110032	3.47E-20	2.73E-16
XM_018054202	gene-TMOD2	TMOD2	transcript	0.067254	0.001123	-5.745194195	0.001177	0.047878
XM_018056156	gene-TMEM8C	TMEM8C	transcript	0.147296	0	-20.9630296	4.39E-12	3.21E-10
XM_005688979	gene-TMEM68	TMEM68	transcript	0.481476	0	-23.66002355	5.44E-15	1.96E-12
XM_018058063	gene-TMEM54	TMEM54	transcript	0	0.308467	7.196318565	1.37E-05	0.000769
XM_018059990	gene-TMEM183A	TMEM183	transcript	0.837555	0	-9.21404718	0.000166	0.008201

XM_013976203	gene-TMEM164	TMEM164	transcript	0.008751	0.879761	6.355109211	5.53E-06	0.000318
XM_018044337	gene-TMEM164	TMEM164	transcript	0	0.344741	23.16061778	1.98E-14	5.18E-12
XM_018039202	gene-TMEM14A	TMEM14A	transcript	0	0.256744	21.15967554	2.83E-12	2.15E-10
XM_018054885	gene-TMEM131	TMEM131	transcript	1.204046	0	-24.91702464	1.82E-16	1.77E-13
XM_018063692	gene-TMC6	TMC6	transcript	0.540125	0.016583	-5.039751346	0.000132	0.006667
XM_005681504	gene-TLR10	TLR10	transcript	4.700537	0.332154	-3.95167302	0.000798	0.03386
XM_018065314	gene-TLK2	TLK2	transcript	0	1.137199	23.8500938	3.25E-15	1.38E-12
XM_018057826	gene-TLDC2	TLDC2	transcript	0.10926	0	-21.52332958	1.22E-12	1.05E-10
XM_018050841	gene-TJP3	TJP3	transcript	0.24547	0.000308	-7.532290335	2.17E-05	0.001196
XM_018041409	gene-TIAL1	TIAL1	transcript	0	0.33868	8.788315668	0.000608	0.026601
XM_005678695	gene-THRAP3	THRAP3	transcript	5.642419	0.473843	-3.607809815	0.000545	0.024155
XM_018061012	gene-THOC5	THOC5	transcript	0.354474	3.106604	3.08064862	0.000437	0.019761
XM_018049669	gene-TFDP2	TFDP2	transcript	2.066809	0.00426	-8.745443242	3.13E-07	1.87E-05
XM_018049665	gene-TFDP2	TFDP2	transcript	0	3.860358	27.06281444	3.80E-19	1.59E-15
XM_018041207	gene-TCF7L2	TCF7L2	transcript	0.69368	0	-24.78423843	2.64E-16	2.19E-13
XM_018041239	gene-TCF7L2	TCF7L2	transcript	0	0.161245	20.20358062	2.51E-11	1.61E-09
XM_018041227	gene-TCF7L2	TCF7L2	transcript	0	0.083715	20.94305208	4.73E-12	3.42E-10
XM_018041237	gene-TCF7L2	TCF7L2	transcript	0	0.133351	21.55030445	1.11E-12	9.69E-11
XM_018041230	gene-TCF7L2	TCF7L2	transcript	0	0.418419	23.04137048	2.69E-14	6.39E-12
XM_018039406	gene-TCF4	TCF4	transcript	6.967894	0.388067	-4.230996637	2.65E-05	0.00145
XM_005701709	gene-TCF19	TCF19	transcript	0.544122	0	-8.693440339	9.02E-06	0.000512
XM_018054264	gene-TCF12	TCF12	transcript	0.079429	0.479569	2.539925861	0.000851	0.035826
XM_018039175	gene-TBC1D7	TBC1D7	transcript	0.364177	0.058522	-2.611165537	0.00096	0.039916
XM_018058533	gene-TBC1D31	TBC1D31	transcript	8.338335	5.229933	-0.71359128	0.000422	0.019184
XM_018047094	gene-TAX1BP1	TAX1BP1	transcript	0.329043	0.019467	-4.07075934	0.000313	0.014772
XM_018063484	gene-TARM1	TARM1	transcript	0.331407	0	-22.21816622	2.20E-13	2.81E-11
XM_018042438	gene-TARBP1	TARBP1	transcript	0.217901	0	-22.52324215	1.00E-13	1.59E-11
XR_001917414.	gene-TARBP1	TARBP1	transcript	0.087583	0	-22.15940348	2.55E-13	3.15E-11
XM_018042439	gene-TARBP1	TARBP1	transcript	0.066997	0	-21.60616845	1.00E-12	8.92E-11
XM_018058152	gene-TAF2	TAF2	transcript	0	0.232984	22.68102217	6.75E-14	1.22E-11
XM_018041285	gene-TACC2	TACC2	transcript	0.223169	0	-24.22055157	1.22E-15	6.86E-13
XM_018042980	gene-SYVN1	SYVN1	transcript	1.672798	0	-10.8953968	0.000318	0.014991
XM_005680681	gene-SYT10	SYT10	transcript	0.225131	2.622371	3.498386565	2.81E-05	0.001532
XM_018047458	gene-SYNJ1	SYNJ1	transcript	1.301754	0	-26.1698638	5.27E-18	1.26E-14
XM_018047361	gene-SYNJ1	SYNJ1	transcript	0	0.177015	8.733218133	1.22E-07	7.35E-06
XM_018039076	gene-SYNGAP1	SYNGAP1	transcript	0.145916	0	-23.14145838	2.11E-14	5.36E-12
XM_018053373	gene-SYNE1	SYNE1	transcript	0.09661	0	-24.74765846	2.92E-16	2.32E-13
XM_018053376	gene-SYNE1	SYNE1	transcript	0.049246	0	-23.82019304	3.56E-15	1.47E-12

XM_018053372	gene-SYNE1	SYNE1	transcript	0.02364	0	-22.83367885	4.65E-14	9.17E-12
XM_018053371	gene-SYNE1	SYNE1	transcript	0.014218	0	-22.14599572	2.64E-13	3.21E-11
XM_018043747	gene-SYN1	SYN1	transcript	0.107261	0	-7.024659949	4.70E-05	0.00252
XM_018043550	gene-SVIP	SVIP	transcript	1.724786	0.947408	-0.919721996	0.000463	0.020838
XM_018057197	gene-SVIL	SVIL	transcript	0	0.189791	22.65964419	7.12E-14	1.26E-11
XM_018044072	gene-STXBP5L	STXBP5L	transcript	0	0.070036	21.67666469	8.15E-13	7.54E-11
XM_018053317	gene-STXBP5	STXBP5	transcript	0.053117	0	-22.47064157	1.17E-13	1.77E-11
XM_018055812	gene-STXBP1	STXBP1	transcript	0.020125	0.63642	4.92372519	0.000413	0.018842
XM_018055742	gene-STRBP	STRBP	transcript	6.295764	0.13983	-5.592850495	2.16E-05	0.001193
XM_018066557	gene-STRA6	STRA6	transcript	0.113791	0	-21.71410655	7.67E-13	7.15E-11
XM_018054388	gene-STARD9	STARD9	transcript	0.170975	0	-24.5868436	4.53E-16	3.21E-13
XM_018043955	gene-STARD8	STARD8	transcript	0.152201	0	-8.033303249	0.000924	0.038622
XM_013962917	gene-ST3GAL3	ST3GAL3	transcript	0.798197	0.011988	-5.548286053	0.000441	0.019942
XM_005699129	gene-SRGN	SRGN	transcript	412.9451	58.97661	-2.824453712	1.96E-11	1.28E-09
XM_005699128	gene-SRGN	SRGN	transcript	42.55963	338.9498	2.967653001	6.60E-18	1.42E-14
XM_018040170	gene-SRCAP	SRCAP	transcript	0.000174	0.140023	7.869618478	0.000115	0.005878
XM_018055900	gene-SPTAN1	SPTAN1	transcript	5.245137	0	-28.14412033	1.40E-20	1.21E-16
XM_018062440	gene-SPOPL	SPOPL	transcript	0.088	0.004934	-3.759493335	1.84E-05	0.001023
XM_018062882	gene-SPIB	SPIB	transcript	5.05059	0.140474	-5.209194618	0.000475	0.021278
XM_018064112	gene-SPC25	SPC25	transcript	0.612096	0	-10.11483355	3.46E-09	2.10E-07
XM_018064309	gene-SPAG5	SPAG5	transcript	0	0.188922	21.95172637	4.16E-13	4.46E-11
XM_018058490	gene-SP140	SP140	transcript	0.53831	0	-23.98992987	2.27E-15	1.05E-12
XM_013973217	gene-SP140	SP140	transcript	0.57376	0	-23.78126395	3.94E-15	1.56E-12
XM_018048396	gene-SOX5	SOX5	transcript	0	0.045398	20.69014275	8.58E-12	5.95E-10
XM_018046084	gene-SNX27	SNX27	transcript	0.148498	0	-21.9722658	4.06E-13	4.37E-11
XM_018050675	gene-SNCAIP	SNCAIP	transcript	0.003917	0.301092	5.913132924	0.000945	0.039401
XM_018052939	gene-SNAP91	SNAP91	transcript	0	0.229346	22.22360395	2.12E-13	2.74E-11
XM_018054396	gene-SNAP23	SNAP23	transcript	0.005141	3.580893	8.710386622	4.22E-06	0.000244
XM_018040224	gene-SMURF1	SMURF1	transcript	0	0.068253	20.82051972	6.31E-12	4.46E-10
XM_013969151	gene-SLCO5A1	SLCO5A1	transcript	0.574228	0.135802	-2.165709313	0.000972	0.04034
XM_018062143	gene-SLC9A5	SLC9A5	transcript	0.107056	0	-22.15277366	2.59E-13	3.19E-11
XM_018066955	gene-SLC41A3	SLC41A3	transcript	0	0.193663	21.25631967	2.25E-12	1.75E-10
XM_005674922	gene-SLC35A5	SLC35A5	transcript	18.23787	6.454959	-1.547573098	1.52E-05	0.000847
XM_018066580	gene-SLC28A1	SLC28A1	transcript	0.174448	0	-22.01573633	3.64E-13	4.02E-11
XR_001918291.	gene-SLC26A2	SLC26A2	transcript	0.021281	0.168069	2.870166829	4.38E-05	0.002351
XM_018051474	gene-SLC25A42	SLC25A42	transcript	0.224174	0.005476	-5.175398145	0.001122	0.045879
XM_005687432	gene-SLC25A30	SLC25A30	transcript	6.005666	1.934584	-1.68081788	1.34E-05	0.000751
XM_018066359	gene-SLC25A29	SLC25A29	transcript	0.241827	0	-22.85015338	4.46E-14	8.89E-12

XM_018066358	gene-SLC25A29	SLC25A29	transcript	0.088056	0	-22.01573633	3.64E-13	4.02E-11
XM_018042716	gene-SLC25A22	SLC25A22	transcript	0.354022	0	-23.16276783	1.99E-14	5.19E-12
XM_005694147	gene-SLC16A5	SLC16A5	transcript	0.379251	0.006371	-5.601677548	0.00024	0.011587
XM_013972380	gene-SLC16A5	SLC16A5	transcript	0.026953	0.519899	4.104309167	0.000845	0.03561
XM_018065408	gene-SLC16A3	SLC16A3	transcript	0.547433	0.054318	-3.284974728	0.000669	0.029007
XM_013962525	gene-SLC16A1	SLC16A1	transcript	0.063592	0	-21.89128967	4.95E-13	5.13E-11
XM_013974418	gene-SLC14A1	SLC14A1	transcript	0.269493	0.087291	-1.58346929	0.000843	0.03554
XM_005685450	gene-SLC12A6	SLC12A6	transcript	1.005156	0	-11.49718373	5.63E-11	3.46E-09
XM_018050705	gene-SLC12A2	SLC12A2	transcript	0	0.357591	23.54918219	7.22E-15	2.38E-12
XM_018050860	gene-SIRT6	SIRT6	transcript	0.414643	0	-22.639895	7.61E-14	1.32E-11
XM_018065988	gene-SIN3A	SIN3A	transcript	0.000552	0.108825	5.663081127	0.001184	0.048133
XM_018062922	gene-SIGLEC10	SIGLEC10	transcript	1.324227	0	-25.27765582	6.70E-17	8.04E-14
XM_013975257	gene-SHTN1	SHTN1	transcript	0	0.297631	22.22172001	2.13E-13	2.75E-11
XM_018045696	gene-SH3GLB1	SH3GLB1	transcript	0.02114	1.222953	5.720252201	2.74E-05	0.001496
XM_018048806	gene-SGSM3	SGSM3	transcript	0	0.881352	23.24548426	1.59E-14	4.37E-12
XM_005699154	gene-SGPL1	SGPL1	transcript	0.074978	0	-21.3484107	1.84E-12	1.49E-10
XM_018045259	gene-SGIP1	SGIP1	transcript	0	0.094325	20.55457662	1.18E-11	7.89E-10
XM_018046467	gene-SFT2D2	SFT2D2	transcript	0.024814	2.529836	6.588747683	8.66E-07	5.12E-05
XM_018061422	gene-SFSWAP	SFSWAP	transcript	0	0.278591	8.297553132	0.000471	0.021199
XM_018066259	gene-SETD3	SETD3	transcript	1.404481	0.023029	-5.871634465	9.66E-05	0.004979
XM_005687506	gene-SERTM1	SERTM1	transcript	0.727845	0.064697	-3.569790008	0.000678	0.029335
XM_013974466	gene-SERPINB11	SERPINB1	transcript	0.144275	0	-21.52332958	1.22E-12	1.05E-10
XM_018063718	gene-SEPT9	SEPT9	transcript	6.599708	0.894014	-3.002648377	0.000413	0.018828
XM_013971828	gene-SEPT4	SEPT4	transcript	0	0.201745	20.6001563	1.06E-11	7.12E-10
XM_018041195	gene-SEMA4G	SEMA4G	transcript	0	0.096099	21.03819158	3.78E-12	2.82E-10
XM_018052515	gene-SEMA4D	SEMA4D	transcript	0.004001	0.966844	7.350631833	8.57E-06	0.000488
XM_018049659	gene-SEC31A	SEC31A	transcript	0.2359	0	-22.57866998	8.76E-14	1.45E-11
XM_018049651	gene-SEC31A	SEC31A	transcript	0.255307	0	-22.49826136	1.07E-13	1.65E-11
XM_018050373	gene-SEC24A	SEC24A	transcript	0.486368	0.009051	-5.534930655	2.69E-05	0.00147
XM_018059913	gene-SDF4	SDF4	transcript	0.506579	0	-8.20803513	0.001169	0.047609
XM_018048877	gene-SCUBE1	SCUBE1	transcript	0.077691	0	-22.74282555	5.86E-14	1.10E-11
XM_018038476	gene-SCN10A	SCN10A	transcript	0.009749	2.295372	7.578686131	8.65E-09	5.24E-07
XM_018048118	gene-SARNP	SARNP	transcript	14.31099	5.870597	-1.30617039	0.000259	0.012462
XM_018064900	gene-SAMD14	SAMD14	transcript	0.005602	0.832413	6.532354637	8.87E-07	5.24E-05
XM_018054556	gene-SALL2	SALL2	transcript	0.238712	0	-8.605408258	0.000338	0.015829
XM_018050900	gene-SAFB	SAFB	transcript	0.0888	2.423812	4.832952841	0.00053	0.023569
XM_018058010	gene-S100PBP	S100PBP	transcript	0.428241	0.01727	-4.379597989	1.24E-05	0.000695
XM_018046138	gene-S100A16	S100A16	transcript	0.004323	0.237289	5.227755435	0.00063	0.027451

XM_018038796	gene-RUNX2	RUNX2	transcript	0	3.735592	12.77436431	1.13E-39	9.77E-35
XM_018058204	gene-RUNX1T1	RUNX1T1	transcript	0.346629	0	-24.98910377	1.50E-16	1.56E-13
XM_005675055	gene-RUBCN	RUBCN	transcript	0.275541	0	-24.03068347	2.03E-15	9.79E-13
XM_018055004	gene-RTKN	RTKN	transcript	0	0.184772	21.88859188	4.86E-13	5.05E-11
XM_018057228	gene-RRBP1	RRBP1	transcript	0	0.151505	22.0508636	3.26E-13	3.72E-11
XM_005699249	gene-RPS24	RPS24	transcript	0	4.485366	23.88608169	2.97E-15	1.29E-12
XM_018063886	gene-RPL38	RPL38	transcript	9.802124	95.78086	3.161042865	1.96E-05	0.001084
XM_018039601	gene-RNF152	RNF152	transcript	0	0.166144	8.772966462	0.000365	0.016892
XM_018039599	gene-RNF152	RNF152	transcript	0	0.069995	21.61985126	9.35E-13	8.50E-11
XM_018066654	gene-RNF123	RNF123	transcript	0.072337	0	-21.71410655	7.67E-13	7.15E-11
XM_018066655	gene-RNF123	RNF123	transcript	0.038144	0.291188	2.805564194	4.78E-07	2.83E-05
XM_018043856	gene-RLIM	RLIM	transcript	0.236128	0	-24.16953552	1.40E-15	7.57E-13
XM_018057252	gene-RIN2	RIN2	transcript	0.530832	0.066292	-2.998709217	6.10E-05	0.003238
XM_018061414	gene-RIMBP2	RIMBP2	transcript	0.057696	0	-21.69448166	8.05E-13	7.47E-11
XR_001919233.	gene-RIC3	RIC3	transcript	0.286943	1.012251	1.802537849	0.000283	0.013504
XM_005690464	gene-RGS18	RGS18	transcript	12.6186	0.661402	-4.379800296	0.000815	0.034545
XM_018060396	gene-RERE	RERE	transcript	0.46459	0.000326	-9.326224769	9.26E-06	0.000525
XM_018052162	gene-RECK	RECK	transcript	0.218395	0.013916	-3.821279844	0.000102	0.005228
XM_005696367	gene-RCAN2	RCAN2	transcript	0.022454	0.340482	3.84696518	0.000824	0.034896
XM_018055726	gene-RBM18	RBM18	transcript	0	0.436334	22.60300252	8.22E-14	1.37E-11
XM_018057832	gene-RBL1	RBL1	transcript	0	0.119692	21.79865033	6.05E-13	5.97E-11
XM_018055110	gene-RASGRP3	RASGRP3	transcript	0.112149	0.000526	-6.371272633	0.000967	0.040151
XM_005682636	gene-RAPGEF6	RAPGEF6	transcript	0.070244	0	-22.61904888	8.03E-14	1.35E-11
XM_018064706	gene-RAPGEF4	RAPGEF4	transcript	0.07568	0	-21.80335148	6.16E-13	6.05E-11
XM_018064421	gene-RAP1GAP2	RAP1GAP	transcript	0.06392	0	-22.25094715	2.03E-13	2.65E-11
XM_018039704	gene-RAB27B	RAB27B	transcript	1.278535	0.012729	-6.730673088	1.56E-06	9.15E-05
XM_018059281	gene-QSER1	QSER1	transcript	0.001587	0.572846	7.805258968	2.24E-06	0.000131
XR_001919368.	gene-PXN	PXN	transcript	0.055905	0.000246	-5.840680823	2.07E-05	0.001145
XM_018057889	gene-PTPRT	PTPRT	transcript	0	0.081058	22.09399223	2.92E-13	3.45E-11
XM_018039447	gene-PTPRM	PTPRM	transcript	0.347766	0	-24.28634549	1.02E-15	6.02E-13
XM_018039450	gene-PTPRM	PTPRM	transcript	0.044394	0	-21.27949486	2.21E-12	1.73E-10
XM_005684283	gene-PTPN3	PTPN3	transcript	0.101438	0	-7.908859651	0.00104	0.042809
XM_005698175	gene-PTEN	PTEN	transcript	39.81594	29.7984	-0.456235864	0.000266	0.012732
XM_018052473	gene-PTCH1	PTCH1	transcript	0	0.275308	22.99635389	3.02E-14	6.70E-12
XM_005699158	gene-PSAP	PSAP	transcript	0.620411	0.000336	-7.527112458	0.000101	0.005181
XM_013965993	gene-PRUNE2	PRUNE2	transcript	0.000713	0.095218	6.650263648	6.32E-05	0.003342
XM_005683795	gene-PRUNE2	PRUNE2	transcript	0	0.032074	21.26457557	2.20E-12	1.72E-10
XM_013971791	gene-PRR11	PRR11	transcript	0.496389	0.187767	-1.427008511	5.89E-05	0.003132

XM_018052538	gene-PRMT2	PRMT2	transcript	2.934299	0	-25.59612706	2.73E-17	4.14E-14
XM_018052530	gene-PRMT2	PRMT2	transcript	0	2.924294	24.87135538	2.07E-16	1.93E-13
XM_005680143	gene-PRICKLE1	PRICKLE1	transcript	0.117976	1.048705	3.124764241	0.000692	0.029873
XM_013967810	gene-PREB	PREB	transcript	21.28099	12.27957	-0.84046122	0.001053	0.043271
XM_018060604	gene-PRDM2	PRDM2	transcript	0.252386	0	-24.20304704	1.28E-15	7.14E-13
XM_018060600	gene-PRDM2	PRDM2	transcript	0.113182	0	-22.99992003	3.03E-14	6.70E-12
XM_018066458	gene-PRC1	PRC1	transcript	0	0.438443	8.456394784	1.41E-06	8.26E-05
XM_018043015	gene-PPP6R3	PPP6R3	transcript	3.483895	0	-12.05274584	1.01E-27	2.18E-23
XM_018063080	gene-PPP6R1	PPP6R1	transcript	0	0.37965	22.96363116	3.29E-14	7.17E-12
XM_018066067	gene-PPP2R5C	PPP2R5C	transcript	0.005382	0.487553	5.885658568	1.61E-05	0.000894
XM_018052347	gene-PPP2R2A	PPP2R2A	transcript	1.778728	0.031189	-5.734225115	2.75E-05	0.001501
XM_018047405	gene-PPP1R9A	PPP1R9A	transcript	0.001673	0.327475	7.332280436	0.000172	0.008477
XM_018047401	gene-PPP1R9A	PPP1R9A	transcript	0	0.202337	9.487431323	8.99E-05	0.00465
XR_001919345.	gene-PPP1R12B	PPP1R12B	transcript	0.535444	0.000602	-7.387727967	2.48E-05	0.001363
XM_018038847	gene-PPP1R10	PPP1R10	transcript	0	3.138251	25.96078808	9.61E-18	1.93E-14
XM_013965808	gene-PPIP5K2	PPIP5K2	transcript	0.20988	0	-23.65508763	5.51E-15	1.98E-12
XM_018043244	gene-PPFIA1	PPFIA1	transcript	0.097616	0	-22.11135218	2.85E-13	3.40E-11
XM_018043247	gene-PPFIA1	PPFIA1	transcript	0.078641	0	-22.04704646	3.37E-13	3.82E-11
XR_001918292.	gene-PPARGC1B	PPARGC1	transcript	0.123544	0	-21.90875937	4.75E-13	4.97E-11
XM_018050753	gene-PP2D1	PP2D1	transcript	0.337983	0.861405	1.281794239	0.000987	0.040877
XM_018062488	gene-POU2F2	POU2F2	transcript	0.698644	0	-25.15416073	9.46E-17	1.08E-13
XM_018062486	gene-POU2F2	POU2F2	transcript	0	0.045576	20.30763474	2.09E-11	1.35E-09
XM_018046443	gene-POU2F1	POU2F1	transcript	0.345645	0.004117	-6.324010945	1.26E-05	0.000706
XM_018059808	gene-POU2AF1	POU2AF1	transcript	0.852238	0	-23.72431994	4.59E-15	1.73E-12
XM_018059807	gene-POU2AF1	POU2AF1	transcript	15.60578	0.56969	-4.909551329	0.000113	0.005781
XM_013976479	gene-PORCN	PORCN	transcript	0	0.240721	20.6819459	8.54E-12	5.93E-10
XM_018059607	gene-PLEKHA7	PLEKHA7	transcript	0.594506	0.003096	-6.916855298	0.001036	0.042669
XM_018060008	gene-PLEKHA6	PLEKHA6	transcript	0	0.080857	21.59977203	9.82E-13	8.80E-11
XM_018060009	gene-PLEKHA6	PLEKHA6	transcript	0	0.383654	23.84712512	3.29E-15	1.39E-12
XM_018048418	gene-PLEKHA5	PLEKHA5	transcript	0	0.173114	21.91934821	4.50E-13	4.74E-11
XM_005692690	gene-PLEKHA4	PLEKHA4	transcript	3.231419	6.83316	1.037162141	0.000351	0.016319
XM_018041997	gene-PLEKHA2	PLEKHA2	transcript	1.047901	0.177313	-2.558410099	3.68E-06	0.000213
XM_018062422	gene-PLD3	PLD3	transcript	0.115466	0.007507	-3.418789201	0.001004	0.041483
XM_005676914	gene-PLA2G2A	PLA2G2A	transcript	3.968569	0	-25.12857814	1.02E-16	1.12E-13
XM_018063329	gene-PKP4	PKP4	transcript	0	0.069612	20.87404528	5.57E-12	3.97E-10
XM_018040897	gene-PKMYT1	PKMYT1	transcript	0.103642	0	-20.62213642	1.02E-11	6.92E-10
XM_018053000	gene-PKIB	PKIB	transcript	0	0.303172	21.51475639	1.20E-12	1.04E-10
XM_018065380	gene-PITPNC1	PITPNC1	transcript	0	0.458077	23.02696438	2.78E-14	6.46E-12

XM_018060180	gene-PIKFYVE	PIKFYVE	transcript	0.217033	0	-24.37663276	8.02E-16	5.02E-13
XM_018065533	gene-PIK3R1	PIK3R1	transcript	20.07795	10.66871	-0.970910928	0.000614	0.026812
XM_018043108	gene-PICALM	PICALM	transcript	1.117538	0.018751	-5.794192733	0.000194	0.00952
XM_018039504	gene-PIAS2	PIAS2	transcript	0	0.472792	23.87604037	3.05E-15	1.32E-12
XR_001295467.	gene-PHTF1	PHTF1	transcript	0.230517	0	-22.52324215	1.00E-13	1.59E-11
XM_018043850	gene-PHKA1	PHKA1	transcript	0	0.188258	20.75758822	7.09E-12	4.96E-10
XM_018052891	gene-PHIP	PHIP	transcript	1.413251	0.007814	-7.375449522	2.67E-08	1.61E-06
XM_018057793	gene-PHF20	PHF20	transcript	3.243663	0.152861	-4.419254477	0.000138	0.006945
XM_018058118	gene-PHC2	PHC2	transcript	0.064891	0.704779	3.246067089	0.00109	0.044614
XM_018048525	gene-PHC1	PHC1	transcript	0	0.101288	20.95032561	4.65E-12	3.38E-10
XM_013974149	gene-PHACTR1	PHACTR1	transcript	0	0.908382	24.29999941	9.81E-16	5.85E-13
XR_001919614.	gene-PGS1	PGS1	transcript	0	0.357726	21.99643284	3.73E-13	4.08E-11
XM_018062639	gene-PGLYRP1	PGLYRP1	transcript	29.15958	0.062828	-8.773944801	0.000218	0.010608
XM_005677748	gene-PDZK1	PDZK1	transcript	0.181689	0	-22.11508234	2.85E-13	3.40E-11
XM_018040360	gene-PDPK1	PDPK1	transcript	0	0.089656	21.89602628	4.77E-13	4.98E-11
XM_018045991	gene-PDE4DIP	PDE4DIP	transcript	0.14897	0	-23.7243922	4.59E-15	1.73E-12
XM_018045987	gene-PDE4DIP	PDE4DIP	transcript	0.05243	0	-21.44978909	1.42E-12	1.19E-10
XM_013962491	gene-PDE4DIP	PDE4DIP	transcript	0.000293	1.342199	11.33331037	5.93E-05	0.003154
XM_018045995	gene-PDE4DIP	PDE4DIP	transcript	0	1.554241	25.39712204	4.77E-17	6.34E-14
XM_018066352	gene-PCSK6	PCSK6	transcript	0.306839	0.00078	-7.293715501	0.000955	0.039705
XM_005688969	gene-PCMTD1	PCMTD1	transcript	3.406739	0	-12.3463004	3.99E-29	1.15E-24
XM_018057947	gene-PCIF1	PCIF1	transcript	0.036991	0.641264	3.99395468	3.26E-08	1.97E-06
XM_018056819	gene-PCDH17	PCDH17	transcript	0	0.298558	9.920373276	2.93E-05	0.001597
XM_018043142	gene-PC	PC	transcript	0.293526	0	-23.38510766	1.12E-14	3.31E-12
XM_018066713	gene-PBRM1	PBRM1	transcript	0.488427	3.439026	2.813950885	7.21E-05	0.003779
XM_018052180	gene-PAX5	PAX5	transcript	3.310263	0	-25.42518526	4.42E-17	5.97E-14
XM_018044560	gene-PARS2	PARS2	transcript	0.466674	0.053655	-3.18054841	0.000247	0.011922
XM_018053553	gene-PARP6	PARP6	transcript	0	0.159605	21.17651843	2.72E-12	2.07E-10
XM_005685180	gene-PARP6	PARP6	transcript	0	0.277397	22.42307906	1.29E-13	1.92E-11
XM_018046870	gene-PARP12	PARP12	transcript	0.898944	0.106592	-3.174548333	0.000305	0.014476
XM_005687901	gene-PARD3	PARD3	transcript	1.456217	0	-26.29780506	3.63E-18	9.23E-15
XM_018057054	gene-PARD3	PARD3	transcript	0.109263	0	-22.7655526	5.53E-14	1.05E-11
XM_018057049	gene-PARD3	PARD3	transcript	0.053194	0	-21.34845017	1.87E-12	1.51E-10
XM_018057052	gene-PARD3	PARD3	transcript	0.048984	0	-21.2259514	2.52E-12	1.94E-10
XM_018066060	gene-PAPOLA	PAPOLA	transcript	1.231429	3.28344	1.342591536	0.000565	0.024879
XM_018066058	gene-PAPOLA	PAPOLA	transcript	3.567394	12.20668	1.723542549	0.00034	0.015896
XM_018048067	gene-PAN2	PAN2	transcript	0.094609	0.011457	-2.925072614	3.96E-05	0.002135
XM_018051199	gene-PALM3	PALM3	transcript	0.132131	0	-21.90505536	4.79E-13	5.00E-11

XM_018044119	gene-PAK3	PAK3	transcript	0	0.095077	22.03031884	3.42E-13	3.87E-11
XM_013970662	gene-P2RX7	P2RX7	transcript	0	0.128131	6.48530429	0.000316	0.014894
XM_018058343	gene-OXR1	OXR1	transcript	0.778084	0.002781	-7.897693131	0.000574	0.025253
XM_018058341	gene-OXR1	OXR1	transcript	0.003927	0.430724	6.513291781	0.001136	0.046445
XM_018047076	gene-OSBPL3	OSBPL3	transcript	0.243316	0	-9.247941531	0.000136	0.006856
XM_005691996	gene-OGFOD1	OGFOD1	transcript	2.919112	10.8683	1.871922496	1.32E-05	0.000743
XM_018049444	gene-OCIAD1	OCIAD1	transcript	0.019322	3.947862	7.285221898	1.57E-05	0.000877
XM_018058938	gene-OBSL1	OBSL1	transcript	1.944107	0	-26.90556304	6.07E-19	2.01E-15
XM_018058936	gene-OBSL1	OBSL1	transcript	0.500089	3.60E-06	-9.696864416	3.10E-10	1.89E-08
XM_018058945	gene-OBSL1	OBSL1	transcript	0.797861	0.002623	-8.052420555	9.96E-06	0.000563
XM_018058939	gene-OBSL1	OBSL1	transcript	0	1.307309	11.50144233	1.48E-32	6.38E-28
XM_013965840	gene-NUGGC	NUGGC	transcript	0.666475	0	-24.31211648	9.55E-16	5.73E-13
XM_018059611	gene-NUCB2	NUCB2	transcript	3.083472	0	-10.8250251	0.000348	0.016205
XM_018047132	gene-NT5C3A	NT5C3A	transcript	1.952338	0.014899	-6.904211195	0.000266	0.012752
XM_018055215	gene-NRXN1	NRXN1	transcript	0	0.269986	9.666604829	0.00011	0.005625
XM_018055212	gene-NRXN1	NRXN1	transcript	0	0.395454	10.26810442	3.24E-05	0.001755
XM_018042027	gene-NRG1	NRG1	transcript	0.27518	0.000313	-8.678235674	5.20E-05	0.002776
XM_018042025	gene-NRG1	NRG1	transcript	0.340258	0.00057	-8.006986053	2.45E-06	0.000142
XM_018047205	gene-NRCAM	NRCAM	transcript	0.065265	0	-22.72574402	6.12E-14	1.13E-11
XM_018047735	gene-NR4A1	NR4A1	transcript	0.124091	0	-21.83739202	5.66E-13	5.67E-11
XM_013965655	gene-NR3C1	NR3C1	transcript	0.365095	0.006994	-5.671037512	3.23E-06	0.000187
XM_005680501	gene-NR1H4	NR1H4	transcript	0	0.137579	21.10206905	3.24E-12	2.45E-10
XR_001917281.	gene-NPTX2	NPTX2	transcript	0	0.14855	21.17992375	2.70E-12	2.06E-10
XM_018066324	gene-NOVA1	NOVA1	transcript	0.221435	0	-8.264111603	0.000601	0.026327
XR_001918227.	gene-NOP14	NOP14	transcript	0	1.045186	9.961792819	3.06E-07	1.82E-05
XM_018057706	gene-NOL4L	NOL4L	transcript	0.010888	0.573896	5.448792716	6.57E-05	0.003459
XM_013970348	gene-NMNAT2	NMNAT2	transcript	0.249382	0.005647	-5.249128697	0.001001	0.041402
XM_005675808	gene-NIPA2	NIPA2	transcript	0.365619	0	-22.62091688	7.99E-14	1.34E-11
XM_018054355	gene-NIN	NIN	transcript	0	0.634823	24.94241185	1.70E-16	1.72E-13
XM_018051248	gene-NFIX	NFIX	transcript	0.065758	1.704677	4.599312141	0.000691	0.029858
XM_018051243	gene-NFIX	NFIX	transcript	0.002944	0.191167	5.971639324	9.44E-05	0.004871
XM_018051244	gene-NFIX	NFIX	transcript	0	0.398296	9.564447296	5.49E-05	0.002927
XM_018060027	gene-NFASC	NFASC	transcript	0.077036	0	-23.00155275	3.02E-14	6.70E-12
XM_018054667	gene-NEO1	NEO1	transcript	0.003187	0.267101	5.861148641	7.18E-05	0.003769
XM_018054670	gene-NEO1	NEO1	transcript	0	0.146261	22.28626439	1.82E-13	2.45E-11
XM_018054669	gene-NEO1	NEO1	transcript	0	0.317974	23.37092505	1.15E-14	3.38E-12
XM_018054674	gene-NEO1	NEO1	transcript	0	0.446439	23.80549177	3.68E-15	1.50E-12
XM_018054373	gene-NEMF	NEMF	transcript	0	1.226568	24.63517548	3.95E-16	2.92E-13

XM_018047900 gene-NELL2	NELL2	transcript	1.832838	0.419336	-2.210332658	1.07E-05	0.000603
XM_018062880 gene-NEB	NEB	transcript	0.077272	0	-24.11763362	1.61E-15	8.49E-13
XM_018062974 gene-NEB	NEB	transcript	0.053339	0	-23.66733801	5.34E-15	1.94E-12
XM_018061395 gene-NCOR2	NCOR2	transcript	0	0.038371	20.77334729	7.06E-12	4.94E-10
XM_018061396 gene-NCOR2	NCOR2	transcript	0	0.100495	22.04132955	3.33E-13	3.79E-11
XM_018061391 gene-NCOR2	NCOR2	transcript	0	0.139747	22.62861723	7.70E-14	1.32E-11
XM_018064714 gene-NCOR1	NCOR1	transcript	0.353542	2.151668	2.519277071	7.77E-05	0.004044
XM_018064703 gene-NCOR1	NCOR1	transcript	0.03985	0.357347	3.079031518	6.33E-05	0.003345
XM_018064712 gene-NCOR1	NCOR1	transcript	0.144728	2.873686	4.228690204	3.17E-07	1.89E-05
XM_018059006 gene-NCAPD3	NCAPD3	transcript	0.389247	0.021729	-4.082284393	0.000318	0.014988
XM_018059011 gene-NCAPD3	NCAPD3	transcript	0.002346	0.063371	4.605809905	4.02E-05	0.002163
XM_018056648 gene-NBEA	NBEA	transcript	1.205651	0.018028	-6.071981368	9.43E-05	0.004869
XM_018056652 gene-NBEA	NBEA	transcript	1.100933	0.074051	-3.92384076	0.001143	0.046657
XM_013963655 gene-NAV3	NAV3	transcript	0	0.265969	23.54879672	7.23E-15	2.38E-12
XM_018060868 gene-NAV1	NAV1	transcript	0.405402	0	-25.30841677	6.14E-17	7.48E-14
XM_018056081 gene-NACC2	NACC2	transcript	0.559605	0.002153	-7.862866724	2.41E-06	0.00014
XM_013963935 gene-NACA	NACA	transcript	8.147929	32.39114	1.947198695	0.000338	0.015842
XM_018056801 gene-NAA16	NAA16	transcript	0.205039	0	-23.16102908	2.00E-14	5.20E-12
XM_018050582 gene-N4BP3	N4BP3	transcript	0	0.043657	20.30749451	2.08E-11	1.35E-09
XM_018043433 gene-MYRF	MYRF	transcript	0.067467	0.353398	2.363854998	0.000651	0.028287
XM_018041294 gene-MYOF	MYOF	transcript	5.285279	0.019949	-8.017610859	3.59E-07	2.13E-05
XM_018053556 gene-MYO9A	MYO9A	transcript	0.144193	0	-24.25733706	1.11E-15	6.38E-13
XM_018053561 gene-MYO9A	MYO9A	transcript	0.118134	0	-23.60933485	6.20E-15	2.16E-12
XM_018039563 gene-MYO5B	MYO5B	transcript	0.156712	0	-23.18436116	1.89E-14	5.00E-12
XM_005676279 gene-MYO1B	MYO1B	transcript	0.004933	2.261837	8.466098986	2.35E-06	0.000137
XM_018064342 gene-MYO18A	MYO18A	transcript	0.280365	0	-9.702515225	1.78E-08	1.08E-06
XM_018064339 gene-MYO18A	MYO18A	transcript	0.021144	0.473824	4.389545744	0.000365	0.016911
XM_018064341 gene-MYO18A	MYO18A	transcript	0	0.10894	22.192592	2.29E-13	2.90E-11
XM_018064637 gene-MYH10	MYH10	transcript	0.174199	0	-22.50362164	1.05E-13	1.64E-11
XM_018051653 gene-MX1	MX1	transcript	9.185153	2.501322	-1.908348833	0.000233	0.011296
XM_018041914 gene-MTUS1	MTUS1	transcript	0	0.290295	22.67625816	6.83E-14	1.23E-11
XM_018058554 gene-MTSS1	MTSS1	transcript	0	0.044767	20.16617858	2.89E-11	1.84E-09
XM_018053388 gene-MTRF1L	MTRF1L	transcript	6.07687	4.177462	-0.594436224	0.000602	0.026363
XM_018060998 gene-MTMR3	MTMR3	transcript	0	0.044453	20.51885639	1.28E-11	8.51E-10
XM_018060994 gene-MTMR3	MTMR3	transcript	0	0.070228	21.17651843	2.72E-12	2.07E-10
XM_005686615 gene-MTIF2	MTIF2	transcript	0	0.46784	22.49731884	1.07E-13	1.65E-11
XM_018057590 gene-MTG2	MTG2	transcript	0	0.193218	7.309804933	1.70E-09	1.03E-07
XM_005691141 gene-MSMO1	MSMO1	transcript	0	1.180328	23.50281231	8.15E-15	2.62E-12

XM_005690341	gene-MS4A1	MS4A1	transcript	24.59866	0.056517	-8.73990996	0.000162	0.00803
XM_005687043	gene-MRRF	MRRF	transcript	0.004208	0.879693	6.977195727	5.90E-06	0.000338
XM_013973833	gene-MRPL14	MRPL14	transcript	0.018121	3.16598	6.792484819	2.37E-05	0.001303
XM_018047068	gene-MPP6	MPP6	transcript	0	0.429716	24.09870241	1.69E-15	8.68E-13
NM_001314186	gene-MPO	MPO	transcript	2.201223	0	-25.53706912	3.23E-17	4.72E-14
XM_018051963	gene-MPDZ	MPDZ	transcript	0.050941	0	-21.95004229	4.29E-13	4.57E-11
XM_018060966	gene-MORC2	MORC2	transcript	0	0.044993	20.18470684	2.77E-11	1.77E-09
XR_001919352.	gene-MORC2	MORC2	transcript	0	0.047645	20.71355023	8.12E-12	5.65E-10
XM_005696649	gene-MOG	MOG	transcript	0.114773	0	-22.03860101	3.44E-13	3.88E-11
XM_005689367	gene-MMP8	MMP8	transcript	0.433963	0	-22.62428318	7.90E-14	1.34E-11
XM_018057220	gene-MKX	MKX	transcript	0.022407	1.074162	5.437543287	0.000524	0.023311
XM_018066965	gene-MKRN2OS	MKRN2OS	transcript	0.659071	0.076738	-3.125158892	0.000583	0.02557
XM_018040535	gene-MKL2	MKL2	transcript	0.775979	0	-25.81247266	1.47E-17	2.65E-14
XM_018066477	gene-MIPOL1	MIPOL1	transcript	0	0.212853	21.23976749	2.33E-12	1.80E-10
XM_018064479	gene-MINK1	MINK1	transcript	0.056892	0.571724	3.328337774	0.000185	0.009067
XM_018064477	gene-MINK1	MINK1	transcript	0	0.200558	8.404727347	0.001226	0.049671
XM_018064468	gene-MINK1	MINK1	transcript	0	0.203896	22.55425003	9.29E-14	1.52E-11
XM_018064470	gene-MINK1	MINK1	transcript	0	0.216625	22.61108641	8.05E-14	1.35E-11
XM_018064475	gene-MINK1	MINK1	transcript	0	0.238111	22.66238276	7.07E-14	1.26E-11
XM_018064476	gene-MINK1	MINK1	transcript	0	0.300345	22.94722045	3.43E-14	7.42E-12
XM_018064471	gene-MINK1	MINK1	transcript	0	0.653819	24.09830904	1.69E-15	8.68E-13
XM_018045307	gene-MIER1	MIER1	transcript	2.31159	0.152508	-3.982554969	0.000195	0.009535
XM_018048718	gene-MICAL3	MICAL3	transcript	0.153733	0	-23.68000845	5.16E-15	1.90E-12
XM_018049887	gene-MFSD7	MFSD7	transcript	2.472507	6.768804	1.414962238	2.10E-05	0.001162
XM_018039806	gene-METTL4	METTL4	transcript	1.911926	1.224424	-0.690638126	0.001041	0.042842
XM_013972345	gene-METTL23	METTL23	transcript	0	0.22172	20.6001563	1.06E-11	7.12E-10
XM_013963919	gene-METTL1	METTL1	transcript	0.371696	0.137694	-1.431950333	0.001102	0.04507
XM_018055432	gene-MEIS1	MEIS1	transcript	0.148565	0.899855	2.557168032	0.00034	0.015893
XM_018040693	gene-MEIOB	MEIOB	transcript	0.184817	0.063395	-1.490687129	4.90E-05	0.002621
XM_018039401	gene-MEF2D	MEF2D	transcript	0.902367	2.30E-05	-10.62471383	0.000447	0.020185
XM_018047181	gene-MDFIC	MDFIC	transcript	4.669617	2.753601	-0.792967475	0.000921	0.038505
XM_018038857	gene-MDC1	MDC1	transcript	0	0.228849	9.007690842	0.000251	0.012087
XM_018053016	gene-MCM9	MCM9	transcript	0.413279	0.001971	-6.923085231	4.96E-06	0.000285
XM_018056429	gene-MBNL2	MBNL2	transcript	0	0.08314	20.95982557	4.55E-12	3.31E-10
XM_018050077	gene-MAT2B	MAT2B	transcript	15.85641	2.613835	-2.670853439	0.00087	0.036505
XM_018066072	gene-MARK3	MARK3	transcript	0.088821	0	-21.57438231	1.08E-12	9.53E-11
XM_005676250	gene-MARCO	MARCO	transcript	0.573053	0	-23.0171232	2.90E-14	6.56E-12
XM_018041660	gene-MAP7D1	MAP7D1	transcript	0	0.227989	22.12960993	2.68E-13	3.25E-11

XM_018053250	gene-MAP7	MAP7	transcript	1.206566	0.005403	-7.688258907	9.02E-06	0.000512
XM_018054954	gene-MAP4K4	MAP4K4	transcript	0.004812	0.559535	6.361746831	0.00106	0.043486
XM_018054952	gene-MAP4K4	MAP4K4	transcript	0.004772	0.849546	7.070248504	2.98E-06	0.000172
XM_018054946	gene-MAP4K4	MAP4K4	transcript	2.00E-07	0.135451	7.750466688	0.00075	0.032099
XM_018054931	gene-MAP4K4	MAP4K4	transcript	0	0.089896	20.15170485	2.83E-11	1.80E-09
XM_018054930	gene-MAP4K4	MAP4K4	transcript	0	0.08389	21.61145786	9.55E-13	8.65E-11
XM_018054939	gene-MAP4K4	MAP4K4	transcript	0	0.094522	21.74204987	6.95E-13	6.63E-11
XM_018054941	gene-MAP4K4	MAP4K4	transcript	0	0.122866	22.11986088	2.75E-13	3.31E-11
XM_018054943	gene-MAP4K4	MAP4K4	transcript	0	0.1275	22.14218795	2.60E-13	3.19E-11
XM_018054955	gene-MAP4K4	MAP4K4	transcript	0	0.188611	22.20388908	2.22E-13	2.82E-11
XM_018054934	gene-MAP4K4	MAP4K4	transcript	0	0.123425	22.20999189	2.20E-13	2.81E-11
XM_018054949	gene-MAP4K4	MAP4K4	transcript	0	0.153625	22.37703516	1.45E-13	2.08E-11
XM_018066783	gene-MAP4	MAP4	transcript	0	0.239122	22.40726179	1.34E-13	1.97E-11
XM_018054017	gene-MAP3K9	MAP3K9	transcript	0.370566	0.023734	-3.888123278	0.0007	0.030165
NM_001285620	gene-MAP34-B	MAP34-B	transcript	24.17699	0.054868	-8.866268863	0.000481	0.021528
NM_001285546	gene-MAP28	MAP28	transcript	39.97109	0.056176	-9.500072015	0.000766	0.032746
XM_018060151	gene-MAP2	MAP2	transcript	0.673362	0	-25.17239148	8.99E-17	1.04E-13
XM_018060100	gene-MAP2	MAP2	transcript	0	0.037713	20.908769	5.11E-12	3.67E-10
XM_005688536	gene-MANBAL	MANBAL	transcript	0.984723	0	-23.28621921	1.44E-14	4.02E-12
XM_018066104	gene-MAN2A2	MAN2A2	transcript	0	0.123202	21.2711835	2.17E-12	1.70E-10
XM_018064290	gene-LYRM9	LYRM9	transcript	1.783351	0.115222	-4.035446354	0.000596	0.026107
XM_005686284	gene-LYG2	LYG2	transcript	1.755332	0	-25.66251613	2.26E-17	3.56E-14
XM_013967445	gene-LYG2	LYG2	transcript	0.544096	0	-23.99852112	2.20E-15	1.04E-12
NM_001285548	gene-LTF	LTF	transcript	9.070083	0.128891	-6.254354916	4.20E-07	2.49E-05
XM_018053973	gene-LTBP2	LTBP2	transcript	0.11439	0	-23.45404643	9.35E-15	2.88E-12
XM_018066878	gene-LRRFIP2	LRRFIP2	transcript	1.333458	0	-25.46087216	4.00E-17	5.57E-14
XM_018040638	gene-LRRFIP1	LRRFIP1	transcript	6.266816	0.887547	-2.898299994	2.55E-05	0.001401
XM_018046792	gene-LRRC61	LRRC61	transcript	0.353574	0	-7.699873749	4.95E-06	0.000285
XM_018062272	gene-LRP3	LRP3	transcript	0	0.160156	20.99921982	4.14E-12	3.05E-10
XM_018056756	gene-LRCH1	LRCH1	transcript	0.00652	0.579271	6.182453441	9.80E-05	0.005044
XM_018065660	gene-LPCAT1	LPCAT1	transcript	0.031929	0.503479	3.829090258	0.000102	0.005228
XM_018054984	gene-LOXL3	LOXL3	transcript	0	0.07378	21.5140817	1.21E-12	1.04E-10
XM_018066534	gene-LOC108638	LOC10863	transcript	0.215065	0.003337	-6.197779582	1.17E-05	0.000661
XM_018056711	gene-LOC108637	LOC10863	transcript	0.060036	0	-7.410073805	0.000241	0.011621
XM_018048271	gene-LOC108636	LOC10863	transcript	0.258724	0	-22.4259048	1.31E-13	1.94E-11
XM_018045584	gene-LOC108635	LOC10863	transcript	0.01114	0.301736	3.877246511	0.000547	0.024215
XM_005676907	gene-LOC108633	LOC10863	transcript	4.196025	0.126913	-5.100357436	0.000405	0.018528
XM_018057830	gene-LOC108633	LOC10863	transcript	0	5.977776	10.57136633	0.000478	0.021401

XM_013965750	gene-LOC108633	LOC10863	transcript	0	0.146961	22.2838127	1.82E-13	2.45E-11
XM_018061723	gene-LOC106503	LOC10650	transcript	0	0.226477	22.32809844	1.64E-13	2.26E-11
XR_001919044.	gene-LOC102191	LOC10219	transcript	0.128248	0	-21.43601256	1.51E-12	1.26E-10
XM_018050132	gene-LOC102191	LOC10219	transcript	0	0.1902	22.39011687	1.40E-13	2.04E-11
XR_001918311.	gene-LOC102191	LOC10219	transcript	0.064518	0	-21.35441181	1.85E-12	1.49E-10
XM_018057616	gene-LOC102188	LOC10218	transcript	0.228923	0.003079	-5.929503251	2.42E-07	1.45E-05
XR_001919897.	gene-LOC102188	LOC10218	transcript	0.062076	0.009246	-2.543465399	0.000573	0.025185
XM_018064688	gene-LOC102186	LOC10218	transcript	0.167087	0	-22.50200114	1.06E-13	1.65E-11
XM_013973155	gene-LOC102185	LOC10218	transcript	0.0213	1.614721	6.071940171	3.52E-05	0.001908
XM_018053251	gene-LOC102184	LOC10218	transcript	0	0.043317	5.625268013	2.34E-05	0.001285
XM_018052845	gene-LOC102183	LOC10218	transcript	0.066198	0.66586	3.254475719	0.000105	0.005374
XM_018045797	gene-LOC102182	LOC10218	transcript	0.144301	0.806819	2.388983817	0.000426	0.019335
XM_013971738	gene-LOC102182	LOC10218	transcript	0.007793	0.239216	4.643536832	4.69E-05	0.002514
XM_018051328	gene-LOC102181	LOC10218	transcript	0	0.116853	20.28466342	2.20E-11	1.42E-09
XM_018043129	gene-LOC102180	LOC10218	transcript	0	0.106622	20.15977516	2.94E-11	1.86E-09
XM_018058942	gene-LOC102180	LOC10218	transcript	6.388503	0	-25.69852167	2.04E-17	3.37E-14
XM_018038535	gene-LOC102180	LOC10218	transcript	0	0.043471	18.68266608	7.18E-10	4.37E-08
XM_018064209	gene-LOC102179	LOC10217	transcript	0.418992	0	-23.2373206	1.64E-14	4.50E-12
XM_018064205	gene-LOC102179	LOC10217	transcript	0	0.211974	21.23721818	2.35E-12	1.82E-10
XM_018064500	gene-LOC102178	LOC10217	transcript	0.163475	0	-22.19981161	2.30E-13	2.91E-11
XM_013976808	gene-LOC102178	LOC10217	transcript	0.232083	0	-21.55416235	1.14E-12	9.86E-11
XM_018059422	gene-LOC102178	LOC10217	transcript	0.411573	0	-23.03999847	2.74E-14	6.41E-12
XM_018040761	gene-LOC102177	LOC10217	transcript	0.89438	0	-25.66121078	2.27E-17	3.56E-14
XR_001919990.	gene-LOC102177	LOC10217	transcript	0.058245	0	-21.85908257	5.36E-13	5.44E-11
XM_005695603	gene-LOC102177	LOC10217	transcript	1.578218	0.984135	-0.717922059	1.12E-06	6.57E-05
XM_018057134	gene-LOC102176	LOC10217	transcript	0.10525	0	-22.22394221	2.17E-13	2.79E-11
XM_018043375	gene-LOC102176	LOC10217	transcript	0.271892	0.708351	1.345485254	0.000303	0.01437
XM_018051572	gene-LOC102176	LOC10217	transcript	0.095277	0	-21.72359752	7.38E-13	6.94E-11
XM_018042468	gene-LOC102175	LOC10217	transcript	0.060501	0	-22.06965481	3.19E-13	3.67E-11
XM_018042467	gene-LOC102175	LOC10217	transcript	0.158858	0.00194	-6.509804864	0.00011	0.005609
XM_005691669	gene-LOC102175	LOC10217	transcript	9.527923	0.214603	-5.549006205	5.30E-05	0.002829
XM_018057015	gene-LOC102175	LOC10217	transcript	0	0.1665	22.44358498	1.23E-13	1.83E-11
XM_018065944	gene-LOC102175	LOC10217	transcript	0.41109	0	-21.85699517	5.40E-13	5.45E-11
XM_005701565	gene-LOC102174	LOC10217	transcript	1.884983	0	-24.05619941	1.90E-15	9.38E-13
XR_001917206.	gene-LOC102173	LOC10217	transcript	0.025797	0	-21.44713607	1.47E-12	1.23E-10
XR_001917203.	gene-LOC102173	LOC10217	transcript	0	0.085965	22.22932741	2.09E-13	2.72E-11
XM_005696018	gene-LOC102173	LOC10217	transcript	3.438748	0.045071	-6.334824947	3.85E-05	0.00208
XM_013973454	gene-LOC102172	LOC10217	transcript	0.001821	0.138115	5.426466826	0.000241	0.011621

XM_013968888	gene-LOC102171[illegible]	LOC10217	transcript	0.028192	0	-5.03861214	0.00013	0.006557
XM_005696010	gene-LOC102171[illegible]	LOC10217	transcript	12.73874	0.202355	-6.023830826	0.000946	0.039406
XM_018044148	gene-LOC102170[illegible]	LOC10217	transcript	0.709623	0.004996	-7.072017833	0.00081	0.034368
XM_018044149	gene-LOC102170[illegible]	LOC10217	transcript	1.513236	0.036179	-5.403354258	9.08E-07	5.36E-05
XM_018058871	gene-LOC102170[illegible]	LOC10217	transcript	1.503223	0	-23.01312652	2.93E-14	6.56E-12
XM_005691046	gene-LOC102169[illegible]	LOC10216	transcript	0.346872	0	-21.87782236	5.12E-13	5.26E-11
XM_018042111	gene-LOC102169[illegible]	LOC10216	transcript	0.030582	0.978474	5.006422404	0.000156	0.007775
XM_018038479	gene-LOC102169[illegible]	LOC10216	transcript	18.99144	0.013778	-10.50392695	0.000474	0.021251
XM_018063553	gene-LOC102168[illegible]	LOC10216	transcript	0.197719	0	-24.06704451	1.85E-15	9.17E-13
XM_018054948	gene-LOC102168[illegible]	LOC10216	transcript	3.529197	0.014562	-7.97261244	0.000673	0.029157
XM_013963206	gene-LOC100860[illegible]	LOC10086	transcript	2.786981	0	-25.17477982	8.91E-17	1.04E-13
XM_013963208	gene-LOC100860[illegible]	LOC10086	transcript	0.575768	0	-23.76086118	4.17E-15	1.61E-12
XM_005681844	gene-LIN54	LIN54	transcript	0.108824	0.005302	-3.766298526	8.37E-05	0.004347
XM_018065844	gene-LIMS2	LIMS2	transcript	0.0394	0.653613	3.890984636	0.000704	0.030333
XM_018049401	gene-LIMCH1	LIMCH1	transcript	0.032999	0	-21.52332958	1.22E-12	1.05E-10
XM_018047803	gene-LIMA1	LIMA1	transcript	0.342154	0.001797	-7.062671155	0.000462	0.020834
XM_013975418	gene-LETM2	LETM2	transcript	0	0.045771	20.24160198	2.43E-11	1.56E-09
XM_005687439	gene-LCP1	LCP1	transcript	2.411686	0.042192	-5.865161869	5.28E-05	0.002816
XM_018046682	gene-KMT2C	KMT2C	transcript	0	0.0291	21.45053304	1.41E-12	1.19E-10
XM_018046928	gene-KLHDC10	KLHDC10	transcript	0.473243	0.01104	-5.386872352	2.80E-06	0.000163
XM_005679589	gene-KLHDC10	KLHDC10	transcript	0.863903	4.684073	2.325491564	0.000429	0.01943
XM_005676429	gene-KLF7	KLF7	transcript	0.077813	0	-22.63967522	7.62E-14	1.32E-11
XM_018065575	gene-KIF2A	KIF2A	transcript	0	0.085279	20.46913502	1.44E-11	9.52E-10
XM_018065577	gene-KIF2A	KIF2A	transcript	0	0.147196	21.20143615	2.56E-12	1.97E-10
XM_018060361	gene-KIF1B	KIF1B	transcript	0.000327	0.677654	8.760552952	6.78E-06	0.000388
XM_018056934	gene-KIF16B	KIF16B	transcript	0.053471	0	-21.79662048	6.26E-13	6.12E-11
XM_018038722	gene-KIF13A	KIF13A	transcript	0	0.856508	24.72169037	3.11E-16	2.40E-13
XM_018039531	gene-KIAA1468	KIAA1468	transcript	0.584437	5.051012	3.153813602	0.0003	0.014232
XM_018057092	gene-KIAA1217	KIAA1217	transcript	0	0.145033	22.31698152	1.68E-13	2.30E-11
XM_018040846	gene-KIAA0556	KIAA0556	transcript	0.678481	0.230513	-1.598972564	0.000122	0.006209
XM_018040845	gene-KIAA0556	KIAA0556	transcript	1.417183	2.255489	0.625105555	6.82E-06	0.00039
XM_018052008	gene-KDM4C	KDM4C	transcript	1.073695	0.06854	-3.964871283	1.06E-05	0.000599
XM_018061280	gene-KDM2B	KDM2B	transcript	0.071245	0.000172	-5.405131572	0.000504	0.022481
XM_018052345	gene-KCTD9	KCTD9	transcript	11.11736	8.79103	-0.381679055	0.000743	0.031855
XM_018056069	gene-KCNT1	KCNT1	transcript	0	0.096286	21.34343883	1.82E-12	1.49E-10
XM_005687464	gene-KCNRG	KCNRG	transcript	1.584728	0.988963	-0.725935392	0.000126	0.006395
XM_018042405	gene-KCNMA1	KCNMA1	transcript	0	0.0245	20.44484769	1.52E-11	1.00E-09
XM_018042413	gene-KCNMA1	KCNMA1	transcript	0	0.134574	22.79252708	5.09E-14	9.87E-12

XM_018050952	gene-KANK3	KANK3	transcript	1.035951	2.307925	1.104777414	0.000395	0.018115
XR_001919624.	gene-JMJD6	JMJD6	transcript	0.459649	0	-23.04155676	2.73E-14	6.40E-12
XM_018063726	gene-JMJD6	JMJD6	transcript	0.001372	0.248968	5.846413759	0.000119	0.006065
XM_018042211	gene-JMJD1C	JMJD1C	transcript	0.320336	0.030366	-3.438193214	0.000326	0.015334
XM_005685922	gene-JKAMP	JKAMP	transcript	11.39589	5.169005	-1.185873902	0.000942	0.039282
XM_005681729	gene-JCHAIN	JCHAIN	transcript	414.1503	21.43715	-4.38736298	0.001157	0.047203
XR_001918100.	gene-ITPR2	ITPR2	transcript	0	0.226747	23.31718144	1.32E-14	3.74E-12
XM_013973557	gene-ITPR1	ITPR1	transcript	0	0.165148	22.90065632	3.86E-14	8.06E-12
XM_013973555	gene-ITPR1	ITPR1	transcript	0	3.234636	27.0557848	3.88E-19	1.59E-15
XM_018060232	gene-ITPKB	ITPKB	transcript	0.01164	0.361892	4.825424785	8.90E-06	0.000506
XM_018060233	gene-ITPKB	ITPKB	transcript	0	0.298935	9.279320377	1.53E-11	1.01E-09
XM_018048123	gene-ITGA7	ITGA7	transcript	0	0.370939	23.02516735	2.81E-14	6.47E-12
XM_018056365	gene-IRS2	IRS2	transcript	5.479443	8.788706	0.6513432	0.000102	0.005228
XM_018066662	gene-IP6K1	IP6K1	transcript	0.362177	0	-23.90841642	2.82E-15	1.25E-12
XM_018041933	gene-INPP5B	INPP5B	transcript	0	0.413973	22.74987889	5.67E-14	1.07E-11
XM_018054889	gene-INPP4A	INPP4A	transcript	0.193133	0	-23.0171232	2.90E-14	6.56E-12
XM_018043465	gene-INCENP	INCENP	transcript	0.000414	0.283432	7.227299247	0.000999	0.041322
XM_013967696	gene-IL1RN	IL1RN	transcript	0	0.754641	9.247282214	0.000506	0.022561
XM_018056948	gene-IL15RA	IL15RA	transcript	0	0.111082	18.40339003	1.29E-09	7.84E-08
XM_018041368	gene-IKZF5	IKZF5	transcript	0.070896	0	-21.74243585	7.16E-13	6.76E-11
XM_018063200	gene-IGLON5	IGLON5	transcript	0.322667	0.529616	0.683841306	0.000632	0.027517
XM_018046972	gene-IGF2BP2	IGF2BP2	transcript	0	0.048902	19.96221159	4.62E-11	2.86E-09
XR_001917002.	gene-IFT122	IFT122	transcript	0.007077	0.063057	3.042390865	0.000178	0.008763
XM_018041407	gene-IDE	IDE	transcript	0.026268	0.692027	4.629172957	0.00123	0.049809
XM_018061064	gene-HPS4	HPS4	transcript	0.114581	0	-22.14811542	2.62E-13	3.21E-11
XM_018049805	gene-HGFAC	HGFAC	transcript	0.20451	0	-22.91366425	3.79E-14	7.95E-12
XM_018042237	gene-HEATR1	HEATR1	transcript	0	0.639376	10.44731476	0.000556	0.024577
XM_018040455	gene-HAGHL	HAGHL	transcript	0.595714	0.010488	-5.370759526	0.000309	0.014625
XM_018040882	gene-GTF2IRD1	GTF2IRD1	transcript	0	0.235475	21.80633143	5.94E-13	5.88E-11
XM_018063297	gene-GRAMD1A	GRAMD1/	transcript	0.504526	0	-23.9013565	2.87E-15	1.26E-12
XM_018063301	gene-GRAMD1A	GRAMD1/	transcript	0.058966	0.533106	3.136885395	0.000828	0.034989
XM_005674826	gene-GPR15	GPR15	transcript	0.808989	0.06074	-3.817591153	0.001223	0.049585
XM_018042033	gene-GPM6A	GPM6A	transcript	0.95286	0.05853	-4.072191344	0.000312	0.014768
XM_018044841	gene-GNB1L	GNB1L	transcript	0.220591	0	-21.21574396	2.49E-12	1.92E-10
XM_018040572	gene-GNA12	GNA12	transcript	0.098643	0.780385	2.913252995	2.01E-06	0.000117
XM_018048022	gene-GLI1	GLI1	transcript	0	0.085959	20.79876032	6.65E-12	4.67E-10
XM_018041623	gene-GFRA1	GFRA1	transcript	0.280642	0	-24.59100078	4.48E-16	3.21E-13
XM_018065536	gene-GFM2	GFM2	transcript	0.450999	0	-23.68131368	5.15E-15	1.90E-12

XM_018058231	gene-GEM	GEM	transcript	11.23197	38.13787	1.787725734	0.000906	0.037952
XM_013969769	gene-GDPD5	GDPD5	transcript	0.000283	0.574435	8.602437253	0.000166	0.008201
XM_018056058	gene-GBGT1	GBGT1	transcript	0	0.114936	20.07603221	3.56E-11	2.24E-09
XM_018056059	gene-GBGT1	GBGT1	transcript	0	0.28842	21.63907619	8.93E-13	8.16E-11
XM_018051497	gene-GATAD2A	GATAD2A	transcript	0.999807	0	-10.98303357	4.70E-10	2.87E-08
XM_018051492	gene-GATAD2A	GATAD2A	transcript	0	0.19269	22.37703516	1.45E-13	2.08E-11
XM_013972765	gene-GAPT	GAPT	transcript	5.064763	0.258582	-4.387945432	0.000267	0.01279
XM_018043071	gene-GAB2	GAB2	transcript	8.983425	13.0475	0.50953094	0.000708	0.030481
XM_018050130	gene-G3BP1	G3BP1	transcript	1.724995	0.413499	-2.078991924	0.000146	0.007298
XM_005678212	gene-FUBP1	FUBP1	transcript	1.307594	0	-24.83565936	2.29E-16	2.02E-13
XM_005678217	gene-FUBP1	FUBP1	transcript	0.907291	0	-24.26255648	1.09E-15	6.33E-13
XM_005678213	gene-FUBP1	FUBP1	transcript	0.676844	0	-23.92929236	2.67E-15	1.19E-12
XM_005678211	gene-FUBP1	FUBP1	transcript	0.493587	0	-23.48745696	8.57E-15	2.72E-12
XM_005678218	gene-FUBP1	FUBP1	transcript	0.44793	0	-23.36728652	1.17E-14	3.41E-12
XM_005678206	gene-FUBP1	FUBP1	transcript	0.358208	0	-23.10232516	2.33E-14	5.71E-12
XM_018061820	gene-FOXF1	FOXF1	transcript	0	0.119432	20.68880499	8.60E-12	5.96E-10
XM_018051909	gene-FOCAD	FOCAD	transcript	0.206615	1.844967	3.137656767	0.000166	0.008194
XM_018053425	gene-FNDC1	FNDC1	transcript	0.419402	0	-24.78281232	2.65E-16	2.19E-13
XM_018055969	gene-FNBP1	FNBP1	transcript	2.511368	0	-26.7749622	8.95E-19	2.76E-15
XM_018046955	gene-FLNC	FLNC	transcript	0.075453	0	-22.85013506	4.46E-14	8.89E-12
XM_005695796	gene-FLNB	FLNB	transcript	0.193886	0.002585	-5.734496072	0.000104	0.005311
XM_005681620	gene-FIP1L1	FIP1L1	transcript	3.044181	0.345094	-3.161527269	0.000612	0.026744
XM_018062132	gene-FHOD1	FHOD1	transcript	0.31752	0	-8.733615053	0.000815	0.034545
XM_013966192	gene-FGD3	FGD3	transcript	0.581997	0.03269	-4.107412278	0.001219	0.049432
XM_018054134	gene-FERMT2	FERMT2	transcript	0	0.373098	22.26494779	2.23E-15	1.05E-12
XM_005677143	gene-FCRLA	FCRLA	transcript	11.51399	0.156533	-6.308689049	9.52E-05	0.004908
XM_018046527	gene-FCRL5	FCRL5	transcript	4.565252	0.070239	-6.137011256	2.30E-05	0.001264
XM_013976239	gene-FCRL4	FCRL4	transcript	0.738263	0	-23.67356789	5.25E-15	1.92E-12
XM_018060103	gene-FCMR	FCMR	transcript	6.81436	0.427116	-4.13238409	0.000691	0.029859
XM_005682408	gene-FCER2	FCER2	transcript	1.719179	0	-25.61023395	2.62E-17	4.04E-14
XM_018059931	gene-FCAMR	FCAMR	transcript	2.289611	0.002302	-10.06403877	9.49E-06	0.000537
XM_005691175	gene-FBXW7	FBXW7	transcript	0.62907	0.082098	-2.965267163	0.00092	0.038478
XM_018044491	gene-FBXW5	FBXW5	transcript	0	0.149485	20.95299955	4.62E-12	3.36E-10
XM_018039067	gene-FARS2	FARS2	transcript	0.208557	0	-22.10376808	2.93E-13	3.45E-11
XM_018058543	gene-FAM91A1	FAM91A1	transcript	0.916657	0	-24.72986636	3.06E-16	2.40E-13
XM_018058541	gene-FAM91A1	FAM91A1	transcript	0.469801	0	-23.82766327	3.49E-15	1.45E-12
XM_018045181	gene-FAM83H	FAM83H	transcript	0.101624	0	-22.62184355	7.97E-14	1.34E-11
XM_013967109	gene-FAM63B	FAM63B	transcript	6.585518	5.020377	-0.435976367	0.000954	0.039705

XM_018052116	gene-FAM214B	FAM214B	transcript	0.104131	0	-21.78877234	6.38E-13	6.20E-11
XM_018059374	gene-FAM168A	FAM168A	transcript	0	0.217644	21.78386594	6.27E-13	6.13E-11
XM_018059451	gene-FAM160A2	FAM160A2	transcript	0.16554	0	-22.62879517	7.83E-14	1.33E-11
XM_013965712	gene-FAM114A2	FAM114A2	transcript	0.129555	0	-7.127147773	0.000135	0.006826
XM_013962885	gene-FAAH	FAAH	transcript	0.02409	0.359035	3.649294061	0.001139	0.046507
XM_018065134	gene-EZH1	EZH1	transcript	0	2.133876	11.76285872	3.00E-05	0.001629
XM_018063764	gene-EXOC7	EXOC7	transcript	0	1.40426	10.07100239	3.85E-05	0.00208
XM_018066046	gene-EXOC3L4	EXOC3L4	transcript	0.132186	0.000465	-7.118664799	0.000443	0.020027
XM_013969295	gene-ESRP1	ESRP1	transcript	0.163888	0	-20.61954296	9.79E-12	6.68E-10
XM_005680706	gene-ERGIC2	ERGIC2	transcript	1.963423	1.438545	-0.485547139	0.000911	0.038145
XM_018042330	gene-ERCC6	ERCC6	transcript	0	0.073686	21.31835062	1.94E-12	1.55E-10
XM_005676494	gene-ERBB4	ERBB4	transcript	0.187234	0	-24.57024834	4.74E-16	3.27E-13
XM_018061442	gene-EP400	EP400	transcript	0.062032	0.000667	-6.369598696	0.000381	0.01759
XM_018040051	gene-EME2	EME2	transcript	0.29028	0.000464	-6.359204956	0.000474	0.021251
XM_018064555	gene-ELP5	ELP5	transcript	6.77501	3.857083	-0.85644781	6.94E-06	0.000396
NM_001287178	gene-ELOVL1	ELOVL1	transcript	3.140364	0.152102	-4.348745263	7.98E-05	0.004152
XM_018059844	gene-ELMOD1	ELMOD1	transcript	0.219562	0	-22.64787876	7.46E-14	1.30E-11
XM_018045197	gene-ELF4	ELF4	transcript	0.358139	0.025524	-3.756046463	0.000229	0.011111
XM_018055414	gene-EHBP1	EHBP1	transcript	0	2.39288	25.71978384	1.92E-17	3.34E-14
XM_018065632	gene-EGFLAM	EGFLAM	transcript	1.11943	0.038843	-4.959778943	0.000276	0.01318
XM_018039033	gene-ECI2	ECI2	transcript	0.000764	0.156865	5.672726222	0.000744	0.031873
XM_018041659	gene-EBF3	EBF3	transcript	0.05582	0	-21.59194623	1.03E-12	9.15E-11
XM_018041663	gene-EBF3	EBF3	transcript	0.04504	0	-21.34211722	1.90E-12	1.53E-10
XM_018041657	gene-EBF3	EBF3	transcript	0.217513	0.027353	-2.946652021	2.67E-05	0.001461
XM_005675012	gene-EAF2	EAF2	transcript	11.03393	0.455206	-4.706718894	0.000278	0.013273
XM_018055055	gene-DYSF	DYSF	transcript	0	0.087033	21.78871065	6.20E-13	6.08E-11
XM_005697014	gene-DSG3	DSG3	transcript	0.137605	0	-22.77191042	5.44E-14	1.04E-11
XM_018039460	gene-DSC3	DSC3	transcript	1.095902	0.025992	-5.538652583	0.000117	0.005963
XM_013969550	gene-DPAGT1	DPAGT1	transcript	0.187549	0	-22.3814837	1.46E-13	2.08E-11
XM_018056417	gene-DOCK9	DOCK9	transcript	5.16E-05	0.392447	9.587480392	0.00038	0.017574
XM_018044192	gene-DOCK11	DOCK11	transcript	0.146274	0	-23.85803801	3.22E-15	1.37E-12
XR_001918836.	gene-DNMT3A	DNMT3A	transcript	0.054059	0	-21.73253411	7.33E-13	6.91E-11
XM_013970162	gene-DNM3	DNM3	transcript	0.224314	0	-23.77267921	4.04E-15	1.57E-12
XM_005695793	gene-DNASE1L3	DNASE1L	transcript	7.517217	0.763548	-3.399103975	0.000884	0.037085
XM_018059003	gene-DNAJB2	DNAJB2	transcript	0.351728	1.731144	2.320706776	0.000456	0.020571
XM_005683975	gene-DMTN	DMTN	transcript	0	0.056419	5.946779314	8.46E-06	0.000482
XM_018057810	gene-DLGAP4	DLGAP4	transcript	0	0.762098	24.18753233	1.33E-15	7.31E-13
XM_018043863	gene-DLG3	DLG3	transcript	0.002125	0.152016	5.407588765	0.000421	0.019181

XM_018043864	gene-DLG3	DLG3	transcript	0	0.148311	20.81017651	6.26E-12	4.43E-10
XM_018058187	gene-DIS3L2	DIS3L2	transcript	0.023848	0.312159	3.470003783	7.65E-05	0.003988
XM_018056820	gene-DIAPH3	DIAPH3	transcript	0	0.373772	22.67192428	6.89E-14	1.23E-11
XM_013972218	gene-DHRS7B	DHRS7B	transcript	2.584668	0	-25.07561572	1.18E-16	1.26E-13
XM_018060910	gene-DERL3	DERL3	transcript	24.79619	1.657803	-4.016474961	0.000838	0.03535
XM_018059522	gene-DENND5A	DENND5A	transcript	2.756807	0	-26.89398654	6.29E-19	2.01E-15
XM_018059523	gene-DENND5A	DENND5A	transcript	0	0.737488	23.90444099	2.83E-15	1.25E-12
XM_018045946	gene-DENND2C	DENND2C	transcript	0.567432	0.000875	-8.822526661	2.01E-06	0.000117
XM_018055747	gene-DENND1A	DENND1A	transcript	0.120091	0	-22.64495449	7.52E-14	1.31E-11
XM_018038867	gene-DDR1	DDR1	transcript	0.104194	0	-22.14371832	2.65E-13	3.22E-11
XM_018038868	gene-DDR1	DDR1	transcript	0.213554	0	-8.284249842	0.000697	0.030066
XM_018038865	gene-DDR1	DDR1	transcript	0	0.116951	21.23976749	2.33E-12	1.80E-10
XM_018038890	gene-DAAM2	DAAM2	transcript	0.417567	0	-24.57379611	4.69E-16	3.27E-13
XM_018059700	gene-CXCR5	CXCR5	transcript	1.873054	0.081119	-4.653128999	0.000305	0.014442
XM_005681804	gene-CXCL13	CXCL13	transcript	67.57432	0.359353	-7.700446924	0.000342	0.015939
XM_005698368	gene-CUEDC2	CUEDC2	transcript	16.22743	4.681481	-1.824051577	6.81E-07	4.03E-05
XM_018047195	gene-CTTNBP2	CTTNBP2	transcript	0	0.09744	21.45501863	1.39E-12	1.18E-10
XM_005686767	gene-CTNNA2	CTNNA2	transcript	0.115722	0.634125	2.451268405	0.000161	0.007999
XM_018066284	gene-CTAGE5	CTAGE5	transcript	0.214398	0	-7.897527444	8.94E-05	0.004628
XM_018067244	gene-CSRNP1	CSRNP1	transcript	0	0.148555	21.18708002	2.65E-12	2.03E-10
XM_018065968	gene-CRTC3	CRTC3	transcript	0.361438	0.012439	-4.800957718	0.000428	0.019403
XM_018040033	gene-CRAMP1	CRAMP1	transcript	0.220076	0.000896	-7.132327015	0.000161	0.007975
XM_005690449	gene-CR2	CR2	transcript	13.12753	0	-29.13953058	6.03E-22	6.51E-18
XM_018062806	gene-CPT1C	CPT1C	transcript	0	0.070319	20.10997827	3.29E-11	2.08E-09
XM_018039276	gene-CPNE5	CPNE5	transcript	0	0.061137	20.29915542	2.13E-11	1.38E-09
XM_018041460	gene-CPEB3	CPEB3	transcript	0.325684	0	-24.64650989	1.11E-19	5.95E-16
XM_018041466	gene-CPEB3	CPEB3	transcript	0	0.23805	23.22965009	1.66E-14	4.52E-12
XM_018061086	gene-CORO1C	CORO1C	transcript	0.018004	0.819733	5.357368899	0.000674	0.0292
XM_005690548	gene-CNST	CNST	transcript	0.010565	0.252084	4.253831356	0.00035	0.016301
XM_005676856	gene-CNR2	CNR2	transcript	3.375486	0.126049	-4.832759866	0.000549	0.024255
XM_018056398	gene-CLYBL	CLYBL	transcript	0	0.342108	7.132757407	2.51E-05	0.00138
XM_018066137	gene-CLK3	CLK3	transcript	1.2291	0	-9.568477239	7.27E-05	0.003807
XM_018066136	gene-CLK3	CLK3	transcript	0.86866	0	-9.105814017	0.000151	0.007527
XM_005686853	gene-CLIP4	CLIP4	transcript	0.200521	0	-22.35025605	1.58E-13	2.21E-11
XM_018061310	gene-CLIP1	CLIP1	transcript	0.025026	0.109521	2.03610994	1.44E-06	8.45E-05
XM_005682410	gene-CLEC4G	CLEC4G	transcript	3.276789	0.155367	-4.516814623	0.000231	0.011196
XM_018040274	gene-CLEC16A	CLEC16A	transcript	0.053054	0.574554	3.415476938	0.000393	0.018057
XM_018067155	gene-CLASP2	CLASP2	transcript	0.478226	0	-24.84136659	2.25E-16	2.02E-13

XM_018067134	gene-CLASP2	CLASP2	transcript	0.000862	0.408492	8.625867961	0.000252	0.012101
XM_005692637	gene-CKM	CKM	transcript	0.803601	0	-23.56015969	7.07E-15	2.36E-12
XM_018042600	gene-CHRM3	CHRM3	transcript	0.149747	0.056175	-1.434217544	0.000358	0.016584
XM_018045908	gene-CHI3L2	CHI3L2	transcript	0.493713	0	-23.1384456	2.12E-14	5.38E-12
XM_013970400	gene-CFHR5	CFHR5	transcript	0.13386	0	-22.10376808	2.93E-13	3.45E-11
XM_018059948	gene-CFAP74	CFAP74	transcript	0.42371	0.0666	-2.767784036	0.000385	0.017731
XM_018051822	gene-CEP44	CEP44	transcript	1.913229	1.125356	-0.803345319	0.000838	0.03535
XM_018049251	gene-CENPE	CENPE	transcript	1.40608	0	-25.3149312	6.01E-17	7.42E-14
XM_018056977	gene-CELF2	CELF2	transcript	0.056654	0	-22.40755376	1.37E-13	2.01E-11
XM_018056580	gene-CDK8	CDK8	transcript	2.11344	1.05221	-1.038234698	0.000431	0.019542
XR_001919744.	gene-CDK12	CDK12	transcript	0	0.179528	8.17525912	1.16E-06	6.80E-05
XM_018061911	gene-CDK10	CDK10	transcript	0.737272	0.001708	-7.301800505	2.32E-07	1.39E-05
XM_018065030	gene-CDC6	CDC6	transcript	0.965935	0.005194	-7.109199045	0.000142	0.007115
XM_018048727	gene-CDC42EP1	CDC42EP1	transcript	0.566275	1.171977	1.017069251	0.000774	0.03297
XM_005693997	gene-CD79B	CD79B	transcript	11.74734	0.109984	-6.836873993	3.27E-05	0.00177
XM_018063189	gene-CD47	CD47	transcript	1.573317	0	-26.11538867	6.17E-18	1.37E-14
XM_018059246	gene-CD44	CD44	transcript	0.164559	0	-22.19177404	2.35E-13	2.95E-11
XM_013974717	gene-CD19	CD19	transcript	5.302965	0.016576	-8.356484789	0.001181	0.048028
XM_005697642	gene-CD19	CD19	transcript	1.80435	0.102679	-4.198663213	0.000931	0.038857
XM_018062510	gene-CD177	CD177	transcript	1.317559	0	-24.61371141	4.21E-16	3.05E-13
XM_018042346	gene-CCSER2	CCSER2	transcript	0.435399	0	-24.39219222	7.66E-16	4.83E-13
XM_018042345	gene-CCSER2	CCSER2	transcript	1.042389	0.000622	-9.388055038	2.27E-06	0.000132
XM_005693784	gene-CCR7	CCR7	transcript	10.82695	0.37496	-4.954349036	0.00014	0.007016
XM_005684094	gene-CCL19	CCL19	transcript	4.853059	0.183706	-4.803588885	0.001159	0.04724
XM_005699439	gene-CCDC81	CCDC81	transcript	0.245933	0.03067	-2.92829013	0.000847	0.035665
XM_013974512	gene-CCDC78	CCDC78	transcript	0.42702	0	-23.22241179	1.71E-14	4.63E-12
XM_018062700	gene-CCDC114	CCDC114	transcript	0.439797	0	-8.5224405	1.10E-06	6.48E-05
XM_018053730	gene-CCDC112	CCDC112	transcript	0.398148	0	-23.1873154	1.87E-14	4.97E-12
XM_018052313	gene-CCAR2	CCAR2	transcript	0.021575	1.472298	5.919419115	1.33E-05	0.000744
XR_001917611.	gene-CC2D1B	CC2D1B	transcript	0.499876	0	-9.959334775	7.62E-05	0.003977
XM_018062153	gene-CBLB	CBLB	transcript	0	0.09975	21.63907619	8.93E-13	8.16E-11
XM_018061868	gene-CBFA2T3	CBFA2T3	transcript	0.226428	0	-22.32258846	1.66E-13	2.29E-11
XM_018044583	gene-CASK	CASK	transcript	0.885417	0	-26.03140326	7.86E-18	1.66E-14
XM_005680743	gene-CASC1	CASC1	transcript	3.165471	0.000705	-10.25017451	0.000132	0.006667
XM_005685535	gene-CAPN3	CAPN3	transcript	0.230005	0	-21.99649757	3.72E-13	4.08E-11
XM_018038657	gene-CAPN11	CAPN11	transcript	0.376007	0.021826	-4.11775194	0.000384	0.017723
XM_018055384	gene-CAPG	CAPG	transcript	1.807307	11.98189	2.783363166	0.000247	0.011922
XM_018063668	gene-CANT1	CANT1	transcript	2.497154	0.017217	-7.167006829	1.36E-06	8.00E-05

XM_018063669	gene-CANT1	CANT1	transcript	0.84865	0.010254	-5.953842238	5.99E-05	0.003182
XM_018061265	gene-CAMKK2	CAMKK2	transcript	0.010776	0.495787	5.119781764	0.000511	0.022762
XM_018064432	gene-CAMKK1	CAMKK1	transcript	0.003926	0.221641	5.421634254	0.000138	0.006928
XM_018042365	gene-CAMK2G	CAMK2G	transcript	0	0.140813	21.51767258	1.20E-12	1.03E-10
XM_018049196	gene-CAMK2D	CAMK2D	transcript	0	0.053273	20.18686584	2.76E-11	1.76E-09
XM_018049194	gene-CAMK2D	CAMK2D	transcript	0	0.054577	20.21392846	2.59E-11	1.66E-09
XM_013963421	gene-CADPS2	CADPS2	transcript	0	0.063941	20.80699234	6.52E-12	4.60E-10
XR_001918827.	gene-CAD	CAD	transcript	0.001661	0.400252	7.570163428	8.49E-06	0.000483
XM_018063053	gene-CACNB4	CACNB4	transcript	0.141849	0	-23.51850921	7.90E-15	2.56E-12
XM_018052132	gene-CA9	CA9	transcript	0.004436	0.136625	4.331979851	0.001216	0.049362
XM_005685676	gene-CA12	CA12	transcript	0	0.043913	20.03717251	3.89E-11	2.42E-09
XM_018048613	gene-C5H12orf4	C5H12orf4	transcript	0.129581	0	-22.02300874	3.58E-13	4.01E-11
XM_018060116	gene-C4BPA	C4BPA	transcript	0.280524	0	-22.86341172	4.31E-14	8.68E-12
XM_018060115	gene-C4BPA	C4BPA	transcript	7.258079	0.603577	-3.684493757	0.000333	0.015636
XM_018066474	gene-C21H15orf3	C21H15orf	transcript	0.000225	0.137143	6.706677445	6.87E-05	0.003615
XR_001917960.	gene-C1H3orf70	C1H3orf70	transcript	0	0.160575	21.83588186	5.53E-13	5.56E-11
XM_005694032	gene-C19H17orf6.	C19H17orf	transcript	1.137015	0.015551	-6.116212775	0.000788	0.033465
XM_005689442	gene-C15H11orf5	C15H11orf	transcript	0.000865	0.758332	8.603808558	6.23E-06	0.000357
XM_018058448	gene-C14H8orf46	C14H8orf4	transcript	0.46505	0.038695	-3.676137451	0.000548	0.024236
XM_005674919	gene-BTLA	BTLA	transcript	6.583863	0.145043	-5.616885248	3.87E-05	0.002087
XM_018065368	gene-BPTF	BPTF	transcript	1.082868	0.516298	-1.119565081	0.000136	0.006832
XM_018065363	gene-BPTF	BPTF	transcript	0.947916	0.535116	-0.870264549	1.10E-06	6.48E-05
XM_018046912	gene-BPGM	BPGM	transcript	1.308527	0.036847	-5.247397308	0.00035	0.016305
XM_018049150	gene-BMPR1B	BMPR1B	transcript	0.300498	0.027933	-3.389909663	0.000977	0.040517
XM_018053542	gene-BMP4	BMP4	transcript	0	0.343551	7.809416024	4.03E-06	0.000233
XM_018044302	gene-BEX4	BEX4	transcript	0.276804	0.003756	-5.377624983	0.000561	0.024736
XM_018038690	gene-BEND6	BEND6	transcript	0.044487	3.286368	6.042892529	5.43E-07	3.21E-05
XM_005684604	gene-BEND3	BEND3	transcript	0.001743	0.132119	5.56220568	9.74E-06	0.000551
XM_013972729	gene-BDP1	BDP1	transcript	0.732617	0	-26.15259524	5.54E-18	1.29E-14
XM_018048707	gene-BCL2L13	BCL2L13	transcript	3.950112	2.057411	-0.992161129	0.000395	0.018105
XM_018048709	gene-BCL2L13	BCL2L13	transcript	0	0.186333	21.48571687	1.29E-12	1.10E-10
XM_018063458	gene-BAZ2B	BAZ2B	transcript	0	0.67111	11.43383615	2.63E-11	1.68E-09
NM_001285545	gene-BAC7.5	BAC7.5	transcript	67.76618	0.035661	-10.98254659	0.000173	0.008528
NM_001285577	gene-BAC5	BAC5	transcript	50.45785	0.044996	-10.21131287	0.000481	0.021527
XM_005682894	gene-AZU1	AZU1	transcript	6.537032	0.133765	-5.673493774	1.72E-05	0.000957
XM_018040189	gene-ATXN2L	ATXN2L	transcript	0	0.851115	10.01509657	2.86E-05	0.001557
XM_018064437	gene-ATP2A3	ATP2A3	transcript	0.432074	0	-23.15058797	2.03E-14	5.22E-12
XM_018054364	gene-ATL1	ATL1	transcript	0.107604	0	-21.5995871	1.02E-12	9.05E-11

XM_018066975	gene-ATG7	ATG7	transcript	0	0.072783	20.6001563	1.06E-11	7.12E-10
XM_018044805	gene-ARVCF	ARVCF	transcript	0.074291	0	-21.56942554	1.09E-12	9.60E-11
XM_018063938	gene-ARSG	ARSG	transcript	1.981216	3.310853	0.69681284	0.000773	0.032928
XM_013969748	gene-ARRB1	ARRB1	transcript	0.064952	0.000164	-6.37705441	0.000753	0.032206
XM_018045164	gene-ARMCX4	ARMCX4	transcript	0	0.297273	23.09712026	2.33E-14	5.71E-12
XM_018058256	gene-ARMC9	ARMC9	transcript	0	0.388381	22.44358498	1.23E-13	1.83E-11
XM_018061343	gene-ARL6IP4	ARL6IP4	transcript	0.372151	0.01719	-4.039011865	0.000565	0.024879
XM_005693735	gene-ARL5C	ARL5C	transcript	3.362245	0.049863	-6.093864372	0.000349	0.016275
XM_018042289	gene-ARID4B	ARID4B	transcript	0	0.403938	23.57401773	6.77E-15	2.31E-12
XM_005685912	gene-ARID4A	ARID4A	transcript	1.156303	0.189812	-2.598560554	0.000951	0.03959
XM_018053402	gene-ARID1B	ARID1B	transcript	0.046111	0	-22.09964221	2.93E-13	3.45E-11
XM_018053404	gene-ARID1B	ARID1B	transcript	0.039649	0	-21.72400567	7.37E-13	6.94E-11
XM_005700426	gene-ARHGEF6	ARHGEF6	transcript	0.000836	0.957601	8.751368272	3.66E-08	2.21E-06
XM_018059648	gene-ARHGEF12	ARHGEF1	transcript	0.917696	4.0935	2.075076524	0.000961	0.03993
XM_018046320	gene-ARHGEF11	ARHGEF1	transcript	0	0.032029	20.10997827	3.29E-11	2.08E-09
XM_018040526	gene-ARHGAP17	ARHGAP1	transcript	0	0.128932	20.93819734	4.73E-12	3.42E-10
XM_005686081	gene-AREL1	AREL1	transcript	0	0.165443	22.0940761	2.93E-13	3.45E-11
XM_018049360	gene-ARAP2	ARAP2	transcript	0.110794	0	-22.64758113	7.37E-14	1.29E-11
XM_018053748	gene-APC	APC	transcript	0.923697	0.013887	-6.04959112	0.000175	0.008622
XM_018043902	gene-AP1S2	AP1S2	transcript	10.39012	4.340783	-1.281654304	0.000337	0.0158
XM_018048158	gene-ANO4	ANO4	transcript	0.18349	2.014221	3.418591626	0.000778	0.033093
XM_013964246	gene-ANO2	ANO2	transcript	0.070463	0.004256	-3.760354474	0.000753	0.032206
XM_018048138	gene-ANKS1B	ANKS1B	transcript	0	0.187953	8.451917018	0.000417	0.018999
XM_018061886	gene-ANKRD11	ANKRD11	transcript	0	0.242242	23.44288274	9.53E-15	2.93E-12
XM_005699169	gene-ANAPC16	ANAPC16	transcript	50.28104	41.51333	-0.318126969	0.000375	0.017322
XM_018054805	gene-ANAPC1	ANAPC1	transcript	0	0.197714	22.41114772	1.33E-13	1.96E-11
XM_018067206	gene-ALS2CL	ALS2CL	transcript	0.321342	0.016912	-4.249295823	7.21E-05	0.003779
XM_018049217	gene-ALPK1	ALPK1	transcript	0	0.800555	24.02747182	2.04E-15	9.79E-13
XM_018042503	gene-AIFM2	AIFM2	transcript	3.792701	0.35088	-3.440718743	0.001047	0.043038
XM_018046942	gene-AHCYL2	AHCYL2	transcript	0.060311	0	-21.74364395	7.13E-13	6.75E-11
XM_018058735	gene-AGFG1	AGFG1	transcript	0.303224	0	-23.61775129	6.08E-15	2.14E-12
XM_018053471	gene-AFDN	AFDN	transcript	0	0.058916	20.79281065	6.64E-12	4.67E-10
XM_018053475	gene-AFDN	AFDN	transcript	0	0.063874	21.36655171	1.72E-12	1.41E-10
XM_018053474	gene-AFDN	AFDN	transcript	0	0.082233	21.71632913	7.40E-13	6.94E-11
XM_018053481	gene-AFDN	AFDN	transcript	0	0.148361	22.50069314	1.06E-13	1.65E-11
XM_018054838	gene-ADRA2B	ADRA2B	transcript	0	0.06173	20.13322413	3.12E-11	1.98E-09
XM_005675149	gene-ADIPOQ	ADIPOQ	transcript	0.323933	0	-23.02149877	2.86E-14	6.55E-12
XM_018049845	gene-ADD1	ADD1	transcript	0	0.307819	21.97138398	3.96E-13	4.30E-11

XM_018064258	gene-ADAP2	ADAP2	transcript	0	0.169006	20.93470533	4.82E-12	3.48E-10
XM_018066196	gene-ADAMTSL3	ADAMTSI	transcript	0	0.037429	20.28466342	2.20E-11	1.42E-09
XM_018065957	gene-ADAL	ADAL	transcript	0.197029	0	-21.80587972	6.12E-13	6.02E-11
XM_018064170	gene-ACACA	ACACA	transcript	0.077411	0	-22.89617814	3.96E-14	8.21E-12
XM_018056136	gene-ABO	ABO	transcript	0	0.399061	22.27465998	1.87E-13	2.49E-11
XM_005690924	gene-ABL2	ABL2	transcript	0.438832	0.008028	-5.654582411	2.85E-05	0.001551
XM_013974705	gene-ABI3BP	ABI3BP	transcript	3.456724	0	-27.0412334	4.05E-19	1.59E-15
XM_013974785	gene-ABI3BP	ABI3BP	transcript	0.781758	0	-24.90985187	1.86E-16	1.78E-13
XM_013974439	gene-ABI3BP	ABI3BP	transcript	0.167841	0	-23.14304235	2.10E-14	5.35E-12
XM_013974409	gene-ABI3BP	ABI3BP	transcript	0	0.980842	24.66153713	3.68E-16	2.74E-13
XM_018038615	gene-ABHD16A	ABHD16A	transcript	7.62971	1.127082	-2.809537379	5.59E-08	3.37E-06
XM_018040734	gene-ABCC1	ABCC1	transcript	0.146961	0	-23.19897961	1.82E-14	4.89E-12
XM_018040732	gene-ABCC1	ABCC1	transcript	0.929524	0.001203	-8.570496206	1.52E-07	9.15E-06
XM_018040427	gene-ABCA3	ABCA3	transcript	0.080342	0	-22.51796431	1.04E-13	1.62E-11
XM_018047508	gene-ABCA13	ABCA13	transcript	0.087086	0.010591	-3.142994348	0.000863	0.036231
