# Supplementary material for: Integrated analyses of miRNA-mRNA expression profiles of ovaries reveal the crucial interaction networks that regulate the prolificacy of goats in the follicular phase
Source: BMC Genomics. 2021 Nov 11;22:812. doi: 10.1186/s12864-021-08156-2 (PMC8582148; doi:10.1186/s12864-021-08156-2)
Supplement: Supplementary file 4 — Additional file 4: Table S4. The information of miRNA sequencing data. [file 12864_2021_8156_MOESM4_ESM.pdf]

Table S4 The information of miRNA sequencing data

Sample	Total_sRNA	Mapped_sRNA	Positive_Mapped_sRN	Negative_Mapped_sRNA
LF-1	21156239 (100.00%)	20648312 (97.60%)	5349311 (25.28%)	15299001 (72.31%)
LF-2	21634940 (100.00%)	21199731 (97.99%)	5532968 (25.57%)	15666763 (72.41%)
LF-3	24763700 (100.00%)	24146048 (97.51%)	7389776 (29.84%)	16756272 (67.66%)
LF-4	27098590 (100.00%)	26362258 (97.28%)	6839653 (25.24%)	19522605 (72.04%)
LF-5	27252271 (100.00%)	25604040 (93.95%)	14676904 (53.86%)	10927136 (40.10%)
HF-1	22063921 (100.00%)	21530347 (97.58%)	6553855 (29.70%)	14976492 (67.88%)
HF-2	24477098 (100.00%)	23635229 (96.56%)	8758874 (35.78%)	14876355 (60.78%)
HF-3	26553388 (100.00%)	25764633 (97.03%)	9304511 (35.04%)	16460122 (61.99%)
HF-4	25752962 (100.00%)	25227925 (97.96%)	12943356 (50.26%)	12284569 (47.70%)
HF-5	26999924 (100.00%)	26340879 (97.56%)	7185336 (26.61%)	19155543 (70.95%)
