# Supplementary material for: Integrated analyses of miRNA-mRNA expression profiles of ovaries reveal the crucial interaction networks that regulate the prolificacy of goats in the follicular phase
Source: BMC Genomics. 2021 Nov 11;22:812. doi: 10.1186/s12864-021-08156-2 (PMC8582148; doi:10.1186/s12864-021-08156-2)
Supplement: Supplementary file 5 — Additional file 5: Table S5. All of the expression miRNAs in the comparison. [file 12864_2021_8156_MOESM5_ESM.pdf]

Table S5 All of the expression miRNAs in the comparison

sRNA	LF_ova_readc	HF_ova_read	log2FoldChai	pval	padj	significant
chi-let-7a-3p	175.7096237	182.0297109	0.05295	0.83106	0.99632	FALSE
chi-let-7a-5p	128332.1217	128389.217	0.00064775	0.9984	0.99939	FALSE
chi-let-7b-3p	258.621192	250.1645606	-0.044825	0.88785	0.99639	FALSE
chi-let-7b-5p	57121.6494	58957.67662	0.045655	0.89877	0.99639	FALSE
chi-let-7c-3p	32.57293123	37.45329753	0.21592	0.65508	0.99632	FALSE
chi-let-7c-5p	74677.77381	70598.74128	-0.081029	0.87417	0.99632	FALSE
chi-let-7d-3p	437.2320511	357.5164633	-0.29104	0.078267	0.82935	FALSE
chi-let-7d-5p	2686.691537	2730.26747	0.023416	0.9275	0.99939	FALSE
chi-let-7e-3p	184.7514496	183.0854536	-0.0086297	0.97827	0.99939	FALSE
chi-let-7e-5p	15057.54472	15934.36584	0.081703	0.80898	0.99632	FALSE
chi-let-7f-3p	13.87719416	12.99936758	-0.11801	0.78732	0.99632	FALSE
chi-let-7f-5p	249086.2869	274645.4985	0.14093	0.55518	0.99632	FALSE
chi-let-7g-3p	11.63537485	13.33684745	0.2214	0.61613	0.99632	FALSE
chi-let-7g-5p	161091.6884	152397.5823	-0.080039	0.72779	0.99632	FALSE
chi-let-7i-3p	228.6198716	208.2605945	-0.13492	0.56106	0.99632	FALSE
chi-let-7i-5p	230319.9833	225307.9285	-0.031743	0.83542	0.99632	FALSE
chi-miR-1	22051.74397	22981.22716	0.059588	0.90183	0.99639	FALSE
chi-miR-100-3p	27.90710483	25.59968258	-0.10912	0.81398	0.99632	FALSE
chi-miR-100-5p	184218.1694	240223.6415	0.38297	0.39879	0.99632	FALSE
chi-miR-101-3p	147124.5506	133462.2705	-0.1406	0.62447	0.99632	FALSE
chi-miR-101-5p	63.41258268	70.70057992	0.16539	0.72792	0.99632	FALSE
chi-miR-103-3p	11555.69618	10969.21793	-0.075237	0.82888	0.99632	FALSE
chi-miR-103-5p	1.705706749	1.280626375	-0.52866	0.61726	0.99632	FALSE
chi-miR-105a	114.5684499	63.82125657	-0.83962	0.26189	0.99632	FALSE
chi-miR-105b-3p	10.22399966	6.978209284	-0.51985	0.57487	0.99632	FALSE
chi-miR-105b-5p	2.937270753	1.936415152	-0.51866	0.58932	0.99632	FALSE
chi-miR-106a-3p	1.30556971	0.204429791	-2.2961	0.3496	0.99632	FALSE
chi-miR-106a-5p	63.79962275	22.91523645	-1.4889	0.045237	0.81581	TRUE
chi-miR-106b-3p	2042.050241	1432.047211	-0.51233	0.049199	0.82935	FALSE
chi-miR-106b-5p	1698.000913	1568.528773	-0.1149	0.62388	0.99632	FALSE
chi-miR-107-3p	1530.405756	1681.176003	0.13612	0.45033	0.99632	FALSE
chi-miR-10a-3p	135.1569606	62.20974209	-1.1152	0.011108	0.47859	TRUE
chi-miR-10a-5p	44516.32713	32800.19516	-0.44061	0.22159	0.9752	FALSE
chi-miR-10b-3p	755.6381331	834.1662301	0.1436	0.6737	0.99632	FALSE
chi-miR-10b-5p	453027.0031	738531.704	0.70507	0.076225	0.82935	FALSE
chi-miR-1185-3p	6.878273128	9.628881831	0.49266	0.311	0.99632	FALSE
chi-miR-1185-5p	1.083073431	0.927686865	-0.20962	0.89092	0.99639	FALSE
chi-miR-1197-3p	13.13632624	20.97383491	0.66781	0.2485	0.98825	FALSE
chi-miR-122	16.97184696	19.03236352	0.16452	0.68052	0.99632	FALSE
chi-miR-1224	1.016714624	1.689448115	0.63066	0.60176	0.99632	FALSE
chi-miR-1248-5p	25.5793541	21.95349955	-0.22956	0.58186	0.99632	FALSE
chi-miR-124a	0.475177909	8.975981548	4.0181	8.76E-05	0.045267	TRUE
chi-miR-125a-3p	122.9770028	96.60379977	-0.34464	0.17888	0.92563	FALSE
chi-miR-125a-5p	21518.6773	24861.15991	0.20834	0.54041	0.99632	FALSE
chi-miR-125b-3p	9960.707242	9301.039626	-0.098807	0.83489	0.99632	FALSE
chi-miR-125b-5p	122653.555	139503.8786	0.18572	0.70836	0.99632	FALSE
chi-miR-126-3p	101915.1105	80547.64004	-0.33947	0.17096	0.92563	FALSE
chi-miR-126-5p	6544.324998	5269.573904	-0.31274	0.10657	0.88062	FALSE
chi-miR-1271-3p	12.11798373	11.21333459	-0.09227	0.84039	0.99632	FALSE
chi-miR-1271-5p	7561.910016	5843.934246	-0.37171	0.071644	0.82935	FALSE
chi-miR-127-3p	9275.927856	9429.801147	0.023731	0.94032	0.99939	FALSE
chi-miR-127-5p	196.1490269	213.9424253	0.12372	0.66741	0.99632	FALSE
chi-miR-128-3p	3694.413505	3142.407072	-0.23372	0.30794	0.99632	FALSE
chi-miR-128-5p	7.288633347	6.828761961	-0.069525	0.89926	0.99639	FALSE
chi-miR-129-3p	75.84520143	61.61367886	-0.29565	0.46965	0.99632	FALSE
chi-miR-129-5p	728.5240389	465.7253873	-0.64674	0.068788	0.82935	FALSE

chi-miR-1296	71.21260282	54.0301272	-0.39024	0.28579	0.99632	FALSE
chi-miR-1306-3p	11.43702819	7.106208939	-0.7302	0.12439	0.88062	FALSE
chi-miR-1306-5p	52.64772897	42.77792053	-0.30869	0.31754	0.99632	FALSE
chi-miR-1307-3p	1344.409199	849.7243172	-0.66247	0.12081	0.88062	FALSE
chi-miR-1307-5p	507.4820453	265.459921	-0.93494	0.001166	0.23635	FALSE
chi-miR-130a-3p	1240.263501	1139.678427	-0.12155	0.76938	0.99632	FALSE
chi-miR-130a-5p	38.57205181	49.07922796	0.3542	0.45379	0.99632	FALSE
chi-miR-130b-3p	43.47750743	33.90479659	-0.37731	0.3728	0.99632	FALSE
chi-miR-130b-5p	167.0132483	132.5728673	-0.33791	0.22931	0.9752	FALSE
chi-miR-133a-3p	605.5733282	627.1804295	0.051446	0.91082	0.99791	FALSE
chi-miR-133a-5p	19.95512607	14.21110216	-0.47137	0.42359	0.99632	FALSE
chi-miR-133b	22.85248305	16.11546216	-0.49214	0.45817	0.99632	FALSE
chi-miR-134	25.21761516	22.83005987	-0.14687	0.82425	0.99632	FALSE
chi-miR-1343	300.8485115	225.0184201	-0.41833	0.12014	0.88062	FALSE
chi-miR-135a	724.6755216	810.9365996	0.1628	0.76832	0.99632	FALSE
chi-miR-135b-3p	0	0.685178412	1.9662	0.52915	0.99632	FALSE
chi-miR-135b-5p	11.35770396	23.54530349	1.0771	0.019763	0.6592	TRUE
chi-miR-136-3p	1103.703908	1214.762681	0.13814	0.61993	0.99632	FALSE
chi-miR-136-5p	31.52887997	41.4538675	0.38058	0.37912	0.99632	FALSE
chi-miR-137	0.763925026	2.377759835	1.6773	0.1903	0.9318	FALSE
chi-miR-1388-3p	316.6591546	233.9937937	-0.43501	0.10888	0.88062	FALSE
chi-miR-1388-5p	909.4338019	864.8495061	-0.072694	0.77733	0.99632	FALSE
chi-miR-140-3p	52998.27041	50417.87727	-0.071994	0.72387	0.99632	FALSE
chi-miR-140-5p	3730.008988	5134.189739	0.46089	0.05877	0.82935	FALSE
chi-miR-141	33.7347699	23.3575469	-0.52674	0.3297	0.99632	FALSE
chi-miR-143-3p	6840949.516	6405638.083	-0.094853	0.78614	0.99632	FALSE
chi-miR-143-5p	3115.384669	3023.944341	-0.042812	0.89372	0.99639	FALSE
chi-miR-144-3p	4.250379393	2.391877397	-0.89705	0.2811	0.99632	FALSE
chi-miR-144-5p	9.630700071	16.90556599	0.82446	0.060887	0.82935	FALSE
chi-miR-145-3p	7738.705922	6890.648355	-0.16739	0.6088	0.99632	FALSE
chi-miR-145-5p	67495.47445	87040.24309	0.3669	0.34865	0.99632	FALSE
chi-miR-1468-3p	0	0	NA	NA	NA	NA
chi-miR-1468-5p	1693.566016	1331.157632	-0.34805	0.16005	0.92563	FALSE
chi-miR-146a	2756.063852	1368.181949	-1.0106	0.096344	0.88062	FALSE
chi-miR-146b-3p	132.8495838	38.81446463	-1.7787	0.068225	0.82935	FALSE
chi-miR-146b-5p	11891.19697	5407.746819	-1.1368	0.28421	0.99632	FALSE
chi-miR-147-3p	78.97847829	48.27705823	-0.71971	0.077153	0.82935	FALSE
chi-miR-147-5p	4.769836819	3.641185751	-0.39585	0.5544	0.99632	FALSE
chi-miR-148a-3p	2171765.608	2229817.48	0.038058	0.89881	0.99639	FALSE
chi-miR-148a-5p	4680.801694	4196.703792	-0.15733	0.51151	0.99632	FALSE
chi-miR-148b-3p	9079.354307	8121.88188	-0.16086	0.56447	0.99632	FALSE
chi-miR-148b-5p	553.5262113	494.828839	-0.16366	0.58717	0.99632	FALSE
chi-miR-150	14181.23299	2316.201822	-2.6142	0.025015	0.73901	TRUE
chi-miR-151-3p	61433.97615	51868.10549	-0.24418	0.20038	0.9318	FALSE
chi-miR-151-5p	1293.534256	1117.444093	-0.21051	0.48712	0.99632	FALSE
chi-miR-153	8.683686028	21.60765077	1.3338	0.007751	0.43488	TRUE
chi-miR-154a-3p	8.149667896	11.05991564	0.45072	0.37967	0.99632	FALSE
chi-miR-154a-5p	0	0.233901939	0.62873	0.84233	0.99632	FALSE
chi-miR-154b-3p	0.46722334	1.0909872	1.4671	0.37966	0.99632	FALSE
chi-miR-154b-5p	102.0837397	127.4863565	0.31576	0.37184	0.99632	FALSE
chi-miR-155-3p	1.986857016	0	-3.4598	0.065393	0.82935	FALSE
chi-miR-155-5p	6413.594893	3559.600191	-0.8495	0.27272	0.99632	FALSE
chi-miR-15a-3p	0.597780311	0.364679331	-0.49261	0.80328	0.99632	FALSE
chi-miR-15a-5p	304.9811765	240.7865411	-0.34377	0.37004	0.99632	FALSE
chi-miR-15b-3p	80.16384201	38.31655407	-1.0733	0.06145	0.82935	FALSE
chi-miR-15b-5p	491.5290907	305.6020722	-0.68737	0.10365	0.88062	FALSE
chi-miR-16a-3p	27.19045168	9.996473499	-1.468	0.028132	0.78617	TRUE
chi-miR-16a-5p	18233.5409	18126.14412	-0.0085537	0.9751	0.99939	FALSE

chi-miR-16b-3p	12.0734665	6.892475739	-0.84926	0.26654	0.99632	FALSE
chi-miR-16b-5p	4534.330671	4203.996807	-0.10933	0.66502	0.99632	FALSE
chi-miR-17-3p	89.0157872	88.58104918	-0.013829	0.95976	0.99939	FALSE
chi-miR-17-5p	1784.026199	2257.053578	0.33884	0.31602	0.99632	FALSE
chi-miR-1814	9.239868377	4.735047447	-0.99083	0.19488	0.9318	FALSE
chi-miR-181b-3p	11.06712656	7.938706147	-0.45331	0.38025	0.99632	FALSE
chi-miR-181b-5p	1862.863668	1585.284685	-0.2326	0.19598	0.9318	FALSE
chi-miR-181c-3p	102.7650169	91.95669741	-0.15884	0.45572	0.99632	FALSE
chi-miR-181c-5p	80.07734404	88.13732538	0.14893	0.58513	0.99632	FALSE
chi-miR-181d	237.9721886	264.5310803	0.1558	0.60946	0.99632	FALSE
chi-miR-182	275.3680409	43.71200526	-2.6572	0.008267	0.43488	TRUE
chi-miR-183	169.1723639	26.51176179	-2.6767	0.005485	0.43488	TRUE
chi-miR-1839	0.957537029	0.435280936	-1.1962	0.48993	0.99632	FALSE
chi-miR-184	107.1483668	12.61738869	-3.0919	0.022953	0.7192	TRUE
chi-miR-186-3p	1.757919075	1.526230294	-0.11703	0.92294	0.99939	FALSE
chi-miR-186-5p	12670.92005	12338.59341	-0.038372	0.8768	0.99632	FALSE
chi-miR-187	17.78299118	21.96751691	0.29741	0.6237	0.99632	FALSE
chi-miR-188-3p	1.821747465	1.580259822	-0.21894	0.8232	0.99632	FALSE
chi-miR-188-5p	34.60922685	36.66544544	0.08288	0.78726	0.99632	FALSE
chi-miR-18a-3p	7.278384217	8.090316734	0.11964	0.83977	0.99632	FALSE
chi-miR-18a-5p	112.4707721	95.95442851	-0.23597	0.63584	0.99632	FALSE
chi-miR-18b-3p	0.130556971	0.233901939	0.05166	0.98696	0.99939	FALSE
chi-miR-18b-5p	4.290344075	0.832483209	-2.4509	0.020751	0.67052	TRUE
chi-miR-190a-3p	1.595601742	0.768642246	-1.0667	0.38794	0.99632	FALSE
chi-miR-190a-5p	16.22076919	29.61837612	0.89053	0.059531	0.82935	FALSE
chi-miR-190b	40.19046402	42.23421345	0.076128	0.909	0.99778	FALSE
chi-miR-191-3p	155.0153351	87.84569555	-0.8242	0.053177	0.82935	FALSE
chi-miR-191-5p	31574.79419	22922.76422	-0.46202	0.22312	0.9752	FALSE
chi-miR-192-3p	7.854295576	8.47099877	0.13583	0.7862	0.99632	FALSE
chi-miR-192-5p	16133.50123	12390.13497	-0.38091	0.018297	0.63064	FALSE
chi-miR-193a	608.3220821	573.2408563	-0.084581	0.70599	0.99632	FALSE
chi-miR-193b-3p	610.2089035	576.6309018	-0.080563	0.71788	0.99632	FALSE
chi-miR-193b-5p	18.40275982	21.65623667	0.21629	0.57695	0.99632	FALSE
chi-miR-194	1727.190694	1638.815953	-0.075581	0.62369	0.99632	FALSE
chi-miR-195-3p	4700.616862	4765.859314	0.020006	0.95367	0.99939	FALSE
chi-miR-195-5p	476.1891524	499.346353	0.069972	0.85733	0.99632	FALSE
chi-miR-196a	419.8294993	381.8421373	-0.13679	0.80781	0.99632	FALSE
chi-miR-196b	1287.180348	1347.566038	0.066174	0.93678	0.99939	FALSE
chi-miR-197-3p	915.571644	723.7573152	-0.3384	0.031003	0.81544	FALSE
chi-miR-197-5p	0.130556971	0	-0.52541	0.86798	0.99632	FALSE
chi-miR-199a-3p	65091.21721	62140.1145	-0.06693	0.82941	0.99632	FALSE
chi-miR-199a-5p	60730.51189	79026.68342	0.37993	0.076162	0.82935	FALSE
chi-miR-199b-5p	14157.12046	20107.31177	0.50622	0.043169	0.81581	FALSE
chi-miR-19a	201.8733646	163.3101335	-0.30716	0.74593	0.99632	FALSE
chi-miR-19b-3p	4084.587578	5881.729979	0.52592	0.27319	0.99632	FALSE
chi-miR-19b-5p	2.299563759	4.27538543	0.8218	0.29549	0.99632	FALSE
chi-miR-200a	1848.378994	1166.941405	-0.66343	0.30131	0.99632	FALSE
chi-miR-200b	2721.61093	1329.656328	-1.0334	0.10476	0.88062	FALSE
chi-miR-200c	384.2648079	196.4486945	-0.96741	0.071739	0.82935	FALSE
chi-miR-202-3p	29.2586393	27.34911703	-0.093023	0.91426	0.99791	FALSE
chi-miR-202-5p	3536.713718	3950.911972	0.15981	0.87281	0.99632	FALSE
chi-miR-204-3p	19.14157521	15.14079037	-0.3312	0.56642	0.99632	FALSE
chi-miR-204-5p	2925.261165	2859.768924	-0.032504	0.94221	0.99939	FALSE
chi-miR-206	97.17662386	61.87475838	-0.64727	0.28468	0.99632	FALSE
chi-miR-208b	0	0	NA	NA	NA	NA
chi-miR-20a-3p	8.167410092	14.2120217	0.81218	0.13273	0.88062	FALSE
chi-miR-20a-5p	4755.905507	7050.799209	0.56797	0.11527	0.88062	FALSE
chi-miR-20b	92.08797411	30.2453945	-1.6143	0.04088	0.81581	TRUE

chi-miR-211	57.31333785	60.9628608	0.092189	0.77991	0.99632	FALSE
chi-miR-21-3p	45.04626828	48.43628275	0.090231	0.88247	0.99639	FALSE
chi-miR-214-3p	3096.324896	3968.425299	0.35817	0.1859	0.92563	FALSE
chi-miR-214-5p	831.6771921	1035.712109	0.31746	0.18615	0.92563	FALSE
chi-miR-215-5p	78.28105286	43.948958	-0.83957	0.04216	0.81581	FALSE
chi-miR-21-5p	1737680.302	2993344.96	0.7846	0.38259	0.99632	FALSE
chi-miR-216b	12.4397608	8.911205748	-0.51779	0.44154	0.99632	FALSE
chi-miR-217-3p	0.775387257	0	-2.0901	0.50173	0.99632	FALSE
chi-miR-217-5p	203.5741205	16.92030954	-3.5914	0.008412	0.43488	TRUE
chi-miR-218	7562.74241	7057.756487	-0.09967	0.61897	0.99632	FALSE
chi-miR-219	1.294203397	0.435280936	-1.5824	0.30973	0.99632	FALSE
chi-miR-221-3p	2254.632228	1839.950223	-0.29364	0.25353	0.99632	FALSE
chi-miR-221-5p	919.1377719	724.4717275	-0.34422	0.43706	0.99632	FALSE
chi-miR-222-3p	1926.619151	1432.584693	-0.42792	0.29212	0.99632	FALSE
chi-miR-223-3p	225.2126705	68.49285204	-1.7191	0.20218	0.93326	FALSE
chi-miR-223-5p	188.8879387	50.90365519	-1.8941	0.17587	0.92563	FALSE
chi-miR-22-3p	31198.45721	28609.02755	-0.12503	0.6212	0.99632	FALSE
chi-miR-224-3p	12.70259061	13.68611462	0.10517	0.80686	0.99632	FALSE
chi-miR-224-5p	7460.778282	6900.829952	-0.11244	0.699	0.99632	FALSE
chi-miR-22-5p	551.4018216	451.3237593	-0.28999	0.24085	0.98745	FALSE
chi-miR-2284a	0	0.214241227	0.62873	0.84233	0.99632	FALSE
chi-miR-2284d	0.205489872	0	-0.52541	0.86798	0.99632	FALSE
chi-miR-2284e	2.129138024	0.201378997	-2.9313	0.044795	0.81581	TRUE
chi-miR-2290	0	0.16024954	0.62873	0.84233	0.99632	FALSE
chi-miR-2318	63.79435888	19.01573222	-1.7528	0.043419	0.81581	TRUE
chi-miR-2331	5.648337863	10.19001282	0.79764	0.13527	0.88062	FALSE
chi-miR-2332	40.3170482	18.79619019	-1.1107	0.036478	0.81581	TRUE
chi-miR-2335	3.471318587	1.110610071	-1.6574	0.15389	0.92563	FALSE
chi-miR-23a	16840.16752	13007.21008	-0.37256	0.15217	0.92563	FALSE
chi-miR-23b-3p	16517.888	12311.71951	-0.42396	0.1704	0.92563	FALSE
chi-miR-23b-5p	3.99989222	4.45581628	0.14845	0.81414	0.99632	FALSE
chi-miR-2404	6.523980828	1.879207645	-1.8202	0.014975	0.553	TRUE
chi-miR-2411-3p	6.929354371	5.728533035	-0.31506	0.58164	0.99632	FALSE
chi-miR-2411-5p	71.04395397	51.15605282	-0.48567	0.23693	0.97891	FALSE
chi-miR-2432	5.572977273	2.810548414	-0.96151	0.14942	0.91966	FALSE
chi-miR-24-3p	34919.96086	29775.68764	-0.22991	0.24354	0.98754	FALSE
chi-miR-24-5p	229.8994219	169.2165674	-0.43851	0.11325	0.88062	FALSE
chi-miR-2483-3p	29.51252802	35.19623177	0.26495	0.68888	0.99632	FALSE
chi-miR-2483-5p	173.2321434	197.7462178	0.19353	0.73669	0.99632	FALSE
chi-miR-25-3p	21842.18612	17359.09715	-0.33144	0.038904	0.81581	FALSE
chi-miR-25-5p	15.05555654	12.90159015	-0.24731	0.61647	0.99632	FALSE
chi-miR-26a-3p	23.714822	31.58357503	0.42826	0.1926	0.9318	FALSE
chi-miR-26a-5p	1338399.96	1243952.278	-0.10558	0.74415	0.99632	FALSE
chi-miR-26b-3p	21.48632669	20.04530121	-0.11961	0.75088	0.99632	FALSE
chi-miR-26b-5p	54754.1278	47627.57442	-0.20115	0.43383	0.99632	FALSE
chi-miR-27a-3p	19831.48634	12235.36884	-0.69677	0.000673	0.23202	FALSE
chi-miR-27a-5p	276.0442074	124.8778007	-1.1473	0.044721	0.81581	TRUE
chi-miR-27b-3p	183448.5286	140694.514	-0.38281	0.19407	0.9318	FALSE
chi-miR-27b-5p	142.346965	147.896667	0.060457	0.80053	0.99632	FALSE
chi-miR-28-3p	6609.2199	6177.774542	-0.097281	0.70987	0.99632	FALSE
chi-miR-28-5p	2672.426802	2388.42924	-0.16205	0.39499	0.99632	FALSE
chi-miR-296-3p	236.9661894	180.8970031	-0.38795	0.18139	0.92563	FALSE
chi-miR-29a-3p	27757.78809	26630.64594	-0.059774	0.80365	0.99632	FALSE
chi-miR-29a-5p	114.2607672	134.1055063	0.23432	0.33301	0.99632	FALSE
chi-miR-29b-3p	1634.76453	1670.587936	0.031168	0.93054	0.99939	FALSE
chi-miR-29b-5p	43.18078533	48.75929972	0.16882	0.64416	0.99632	FALSE
chi-miR-29c-3p	0.443263714	0.640998162	0.70933	0.76666	0.99632	FALSE
chi-miR-29c-5p	660.2854949	662.9001051	0.0062384	0.98196	0.99939	FALSE

chi-miR-301a-3p	12.35479797	14.46655837	0.19266	0.78963	0.99632	FALSE
chi-miR-301a-5p	46.18118476	32.30691906	-0.51986	0.059585	0.82935	FALSE
chi-miR-301b	2.660093835	4.147112964	0.53883	0.55907	0.99632	FALSE
chi-miR-30a-3p	1749.984167	1498.034981	-0.22382	0.42128	0.99632	FALSE
chi-miR-30a-5p	110276.468	146021.5643	0.40506	0.098197	0.88062	FALSE
chi-miR-30b-3p	34.19662615	29.41647929	-0.20817	0.57049	0.99632	FALSE
chi-miR-30b-5p	4000.879574	4283.842016	0.098862	0.68908	0.99632	FALSE
chi-miR-30c-3p	96.32895069	90.58387225	-0.088193	0.75789	0.99632	FALSE
chi-miR-30c-5p	15357.96168	17934.69391	0.22381	0.34324	0.99632	FALSE
chi-miR-30d-3p	218.5743671	190.6116612	-0.19768	0.34273	0.99632	FALSE
chi-miR-30e-3p	3380.659444	2937.516529	-0.20258	0.44725	0.99632	FALSE
chi-miR-30e-5p	43031.66965	50905.08946	0.2424	0.50472	0.99632	FALSE
chi-miR-30f-3p	126.3554405	119.4213307	-0.074209	0.76834	0.99632	FALSE
chi-miR-30f-5p	1303.848383	1585.461238	0.28243	0.22795	0.9752	FALSE
chi-miR-320-3p	6192.229589	6548.682546	0.080872	0.79385	0.99632	FALSE
chi-miR-323a-3p	19.98925557	32.19344131	0.69407	0.10051	0.88062	FALSE
chi-miR-323b	18.71006148	15.74651312	-0.2499	0.56291	0.99632	FALSE
chi-miR-324-3p	6.874835434	8.45513486	0.28191	0.62379	0.99632	FALSE
chi-miR-324-5p	84.77349472	74.98077572	-0.1827	0.59632	0.99632	FALSE
chi-miR-326-3p	19.85732755	14.39721797	-0.49019	0.37472	0.99632	FALSE
chi-miR-326-5p	4.241805299	1.490694399	-1.5171	0.12066	0.88062	FALSE
chi-miR-328-3p	852.7832422	801.4405105	-0.088612	0.76962	0.99632	FALSE
chi-miR-329a-3p	1.298650304	1.22844272	-0.11471	0.91741	0.99791	FALSE
chi-miR-329a-5p	0	0	NA	NA	NA	NA
chi-miR-329b-3p	39.19688611	33.39484205	-0.23448	0.61731	0.99632	FALSE
chi-miR-330-3p	175.2949768	127.6818721	-0.46091	0.32844	0.99632	FALSE
chi-miR-330-5p	126.0354372	85.13085884	-0.5741	0.16138	0.92563	FALSE
chi-miR-331-3p	215.9111586	210.1191777	-0.035073	0.90003	0.99639	FALSE
chi-miR-331-5p	333.3415556	357.3378747	0.10176	0.65825	0.99632	FALSE
chi-miR-335-3p	666.7480869	514.019151	-0.37532	0.086675	0.85354	FALSE
chi-miR-335-5p	211.5491429	343.9207729	0.70231	0.014381	0.55074	FALSE
chi-miR-338-3p	62.97763732	42.9292113	-0.56505	0.1321	0.88062	FALSE
chi-miR-338-5p	40.28338149	26.75733374	-0.60649	0.13337	0.88062	FALSE
chi-miR-33a-3p	28.60242513	19.37491471	-0.55817	0.11317	0.88062	FALSE
chi-miR-33a-5p	15.5238948	10.24461889	-0.63194	0.1821	0.92563	FALSE
chi-miR-33b-3p	1.440049981	0.604136991	-1.3249	0.45608	0.99632	FALSE
chi-miR-33b-5p	0	0.374490767	1.2001	0.70412	0.99632	FALSE
chi-miR-340-3p	253.7144897	221.9404093	-0.19698	0.64273	0.99632	FALSE
chi-miR-340-5p	3873.424304	3184.685831	-0.28268	0.55339	0.99632	FALSE
chi-miR-342-3p	5633.212309	5163.232983	-0.12581	0.75725	0.99632	FALSE
chi-miR-342-5p	37.1724261	21.52428366	-0.80278	0.1199	0.88062	FALSE
chi-miR-3431-3p	882.2580446	806.5559589	-0.12862	0.62196	0.99632	FALSE
chi-miR-3431-5p	9227.768654	8095.600181	-0.18874	0.31461	0.99632	FALSE
chi-miR-3432-3p	36.59511399	43.7125386	0.27198	0.46028	0.99632	FALSE
chi-miR-3432-5p	5310.560758	5304.665611	-0.0014905	0.99596	0.99939	FALSE
chi-miR-345-3p	37.78245647	40.48284383	0.085257	0.75827	0.99632	FALSE
chi-miR-345-5p	10.38192155	11.50511436	0.11537	0.81708	0.99632	FALSE
chi-miR-346-5p	0	1.219918719	2.8009	0.20046	0.9318	FALSE
chi-miR-34a	342.5411837	449.9685185	0.39285	0.23277	0.97831	FALSE
chi-miR-34b-3p	167.8822404	90.78876884	-0.8867	0.22378	0.9752	FALSE
chi-miR-34b-5p	6.72599125	5.0764319	-0.4039	0.6717	0.99632	FALSE
chi-miR-34c-3p	48.84222954	21.66734355	-1.172	0.12662	0.88062	FALSE
chi-miR-34c-5p	3436.331568	1685.524918	-1.0277	0.25911	0.99632	FALSE
chi-miR-361-3p	3890.313313	2619.691824	-0.57073	0.067153	0.82935	FALSE
chi-miR-361-5p	1591.554917	1499.999654	-0.084924	0.72881	0.99632	FALSE
chi-miR-362-3p	14.00116954	14.18164845	0.05501	0.92868	0.99939	FALSE
chi-miR-362-5p	504.7973693	793.7678538	0.6537	0.005647	0.43488	FALSE
chi-miR-363-3p	302.1131733	92.17037759	-1.7159	0.013197	0.52485	TRUE

chi-miR-363-5p	3.351974096	0.844102676	-2.0755	0.12485	0.88062	FALSE
chi-miR-365-3p	270.3205131	361.0012109	0.4184	0.19787	0.9318	FALSE
chi-miR-369-3p	214.4513997	302.615428	0.49664	0.17632	0.92563	FALSE
chi-miR-369-5p	124.6003687	147.9989249	0.24654	0.50567	0.99632	FALSE
chi-miR-374a-3p	1397.976009	1801.86761	0.36614	0.18891	0.93016	FALSE
chi-miR-374a-5p	2073.851545	2874.96484	0.47118	0.066528	0.82935	FALSE
chi-miR-374b-3p	196.5793579	244.0308368	0.3103	0.24268	0.98754	FALSE
chi-miR-374b-5p	6061.382922	7967.703391	0.39446	0.034215	0.81581	FALSE
chi-miR-376a	5.263222184	4.97714036	-0.10029	0.9069	0.99759	FALSE
chi-miR-376b-3p	4.896840263	8.180635333	0.73035	0.2554	0.99632	FALSE
chi-miR-376b-5p	12.94436417	24.81742296	0.92234	0.091363	0.88062	FALSE
chi-miR-376c-3p	49.96772897	74.85166095	0.58402	0.10269	0.88062	FALSE
chi-miR-376d	3.402839403	3.18878687	-0.15174	0.83658	0.99632	FALSE
chi-miR-376e-3p	21.22265448	29.8793016	0.50404	0.22291	0.9752	FALSE
chi-miR-377	3.977779115	5.797337881	0.50827	0.56682	0.99632	FALSE
chi-miR-378-3p	15849.9521	11704.41172	-0.43749	0.063773	0.82935	FALSE
chi-miR-378-5p	127.1828468	121.9971015	-0.065442	0.76951	0.99632	FALSE
chi-miR-379-3p	69.45094573	72.95685067	0.066977	0.84029	0.99632	FALSE
chi-miR-379-5p	10098.99766	12876.62707	0.3505	0.33106	0.99632	FALSE
chi-miR-380-3p	170.680115	193.2486497	0.17992	0.52639	0.99632	FALSE
chi-miR-380-5p	5.200353386	6.869730832	0.36344	0.57993	0.99632	FALSE
chi-miR-381	166.9804279	197.8637667	0.24202	0.42044	0.99632	FALSE
chi-miR-382-3p	210.47151	300.2932028	0.51348	0.15254	0.92563	FALSE
chi-miR-382-5p	324.085662	396.7595783	0.29166	0.44645	0.99632	FALSE
chi-miR-383	244.9345066	297.8148646	0.2824	0.81011	0.99632	FALSE
chi-miR-3955-3p	1.179555564	1.765040172	0.47972	0.70603	0.99632	FALSE
chi-miR-3955-5p	61.09817745	69.71309117	0.18401	0.65606	0.99632	FALSE
chi-miR-3958-3p	1303.309602	1646.654881	0.33706	0.36021	0.99632	FALSE
chi-miR-3958-5p	0	0.448143166	1.2139	0.70085	0.99632	FALSE
chi-miR-3959-3p	15.1015325	27.68469168	0.86298	0.04655	0.81581	FALSE
chi-miR-3959-5p	392.9078881	589.4993469	0.58473	0.046126	0.81581	FALSE
chi-miR-409-3p	747.6134892	1106.581059	0.56496	0.078643	0.82935	FALSE
chi-miR-409-5p	146.6199924	186.9556209	0.34687	0.40303	0.99632	FALSE
chi-miR-410-3p	43.55760835	64.05840863	0.55502	0.1861	0.92563	FALSE
chi-miR-410-5p	0	0.374490767	1.2001	0.70412	0.99632	FALSE
chi-miR-411a-3p	293.6642655	302.1932901	0.040214	0.86226	0.99632	FALSE
chi-miR-411a-5p	13066.52858	15583.97643	0.25416	0.38611	0.99632	FALSE
chi-miR-411b-3p	1.147891121	3.072076629	1.4167	0.11857	0.88062	FALSE
chi-miR-411b-5p	83.57176819	136.2748065	0.70245	0.060768	0.82935	FALSE
chi-miR-412-3p	1.10941524	2.244501358	1.0466	0.48522	0.99632	FALSE
chi-miR-412-5p	87.07162658	109.4584813	0.32588	0.47435	0.99632	FALSE
chi-miR-421-3p	77.653717	73.77388929	-0.086059	0.78468	0.99632	FALSE
chi-miR-423-3p	6693.83889	5652.681428	-0.24395	0.31927	0.99632	FALSE
chi-miR-423-5p	5095.438719	4398.00288	-0.21252	0.48597	0.99632	FALSE
chi-miR-424-3p	584.8615975	639.246844	0.12874	0.82566	0.99632	FALSE
chi-miR-424-5p	3036.373511	4094.638127	0.43151	0.45045	0.99632	FALSE
chi-miR-425-3p	76.52607672	39.98132738	-0.94653	0.010266	0.46153	FALSE
chi-miR-425-5p	651.9010125	435.0835578	-0.58467	0.25729	0.99632	FALSE
chi-miR-429	67.81183875	27.72619503	-1.2876	0.072562	0.82935	FALSE
chi-miR-432-3p	0.130556971	0.204429791	0.05166	0.98696	0.99939	FALSE
chi-miR-432-5p	112.8425762	147.0782993	0.38104	0.31656	0.99632	FALSE
chi-miR-433	35.25402474	35.19552259	0.0015217	0.99695	0.99939	FALSE
chi-miR-449a-3p	0.854243459	0.204429791	-1.5953	0.44271	0.99632	FALSE
chi-miR-449a-5p	146.417909	17.8229507	-3.0418	1.96E-05	0.020246	TRUE
chi-miR-449b-3p	0.214063967	0	-0.52541	0.86798	0.99632	FALSE
chi-miR-449b-5p	0.996678299	0	-2.4269	0.37951	0.99632	FALSE
chi-miR-449c	0.719763187	0.418671017	-0.81465	0.65959	0.99632	FALSE
chi-miR-450-3p	23.36925019	23.54320974	0.015653	0.97856	0.99939	FALSE

chi-miR-450-5p	6332.428246	8832.024073	0.48004	0.28111	0.99632	FALSE
chi-miR-451-3p	0	0.320499081	1.1924	0.70595	0.99632	FALSE
chi-miR-451-5p	3793.50076	5409.47393	0.51196	0.16566	0.92563	FALSE
chi-miR-454-3p	128.1729987	162.4803394	0.33647	0.45933	0.99632	FALSE
chi-miR-454-5p	6.073514143	2.663217048	-1.2325	0.077054	0.82935	FALSE
chi-miR-455-3p	1978.716167	1914.626591	-0.047168	0.91292	0.99791	FALSE
chi-miR-455-5p	13631.95991	19556.64275	0.52072	0.1862	0.92563	FALSE
chi-miR-483	8.317565059	14.7575812	0.85455	0.18366	0.92563	FALSE
chi-miR-485-3p	10.00369933	6.827441126	-0.5692	0.28373	0.99632	FALSE
chi-miR-485-5p	84.12830629	113.1892756	0.42115	0.1775	0.92563	FALSE
chi-miR-487a-3p	5.524342578	6.298339053	0.13462	0.8287	0.99632	FALSE
chi-miR-487a-5p	0	0	NA	NA	NA	NA
chi-miR-487b-3p	111.4268406	129.6291119	0.21528	0.50225	0.99632	FALSE
chi-miR-487b-5p	0.854243459	0	-2.1846	0.38873	0.99632	FALSE
chi-miR-490	395.1631271	559.3786742	0.50262	0.26729	0.99632	FALSE
chi-miR-491-3p	6.927814667	9.992161839	0.52538	0.42893	0.99632	FALSE
chi-miR-491-5p	22.17583774	18.01525652	-0.2754	0.5397	0.99632	FALSE
chi-miR-493-3p	715.2790235	1182.473246	0.72506	0.1019	0.88062	FALSE
chi-miR-493-5p	125.6529112	198.9808699	0.66286	0.16359	0.92563	FALSE
chi-miR-494	297.8167853	606.039554	1.0244	0.002394	0.34503	TRUE
chi-miR-495-3p	397.9248065	488.4534203	0.29415	0.30434	0.99632	FALSE
chi-miR-496-3p	1.27494043	1.26905158	0.014068	0.99134	0.99939	FALSE
chi-miR-496-5p	0	0	NA	NA	NA	NA
chi-miR-497-5p	4229.482797	5048.825801	0.25563	0.45894	0.99632	FALSE
chi-miR-499-3p	0.942401248	0.971867116	0.067973	0.96164	0.99939	FALSE
chi-miR-499-5p	1068.558485	1043.491566	-0.033397	0.90294	0.99639	FALSE
chi-miR-500-3p	724.0634366	686.6396334	-0.075166	0.70885	0.99632	FALSE
chi-miR-500-5p	274.1513474	403.4272059	0.55977	0.009157	0.45089	FALSE
chi-miR-502a	4.881608564	6.15844712	0.29593	0.73884	0.99632	FALSE
chi-miR-502b-3p	9.529135343	8.972498411	-0.12962	0.79392	0.99632	FALSE
chi-miR-502b-5p	25.11426	26.05738244	0.066721	0.8722	0.99632	FALSE
chi-miR-504	427.4584142	283.2405762	-0.59348	0.012784	0.52485	FALSE
chi-miR-505-3p	242.8603012	207.0090545	-0.22848	0.24947	0.98834	FALSE
chi-miR-532-3p	205.286041	192.1110689	-0.092314	0.74297	0.99632	FALSE
chi-miR-532-5p	40110.17711	42873.82741	0.096154	0.73115	0.99632	FALSE
chi-miR-542-3p	5405.779446	7723.461227	0.51481	0.29032	0.99632	FALSE
chi-miR-542-5p	80.14025368	105.816186	0.40634	0.41222	0.99632	FALSE
chi-miR-543-3p	155.1856428	113.4215963	-0.45565	0.056634	0.82935	FALSE
chi-miR-543-5p	1.29007621	0.588731993	-1.1333	0.43821	0.99632	FALSE
chi-miR-544-5p	7.647622305	10.40807025	0.42044	0.45269	0.99632	FALSE
chi-miR-545-3p	2.046404386	0.940549095	-1.1556	0.31758	0.99632	FALSE
chi-miR-545-5p	0.826980058	0	-2.1356	0.40513	0.99632	FALSE
chi-miR-582-5p	16.60133934	23.00729251	0.48137	0.30092	0.99632	FALSE
chi-miR-592	220.7134547	215.2847398	-0.033044	0.93031	0.99939	FALSE
chi-miR-628-5p	21.33984853	21.96496526	0.03653	0.91579	0.99791	FALSE
chi-miR-655	52.98135599	79.8605083	0.59412	0.084243	0.84571	FALSE
chi-miR-656	4.784103322	4.983901002	0.026876	0.97098	0.99939	FALSE
chi-miR-660	2262.774375	2657.574449	0.23178	0.22991	0.9752	FALSE
chi-miR-665	3.640089638	7.761135794	1.0942	0.064272	0.82935	FALSE
chi-miR-671-3p	77.95401431	50.51153871	-0.63386	0.1374	0.88062	FALSE
chi-miR-671-5p	44.46033855	44.42167224	-0.0098744	0.98119	0.99939	FALSE
chi-miR-708-3p	4396.728123	3581.33025	-0.29592	0.38089	0.99632	FALSE
chi-miR-708-5p	775.8932283	864.4327749	0.15583	0.50963	0.99632	FALSE
chi-miR-7-3p	165.4519326	134.4292268	-0.30392	0.33369	0.99632	FALSE
chi-miR-758	58.62826126	67.23985711	0.1973	0.45104	0.99632	FALSE
chi-miR-7-5p	17695.22176	16571.46712	-0.094696	0.84581	0.99632	FALSE
chi-miR-767	122.1554241	60.0368317	-1.0191	0.15642	0.92563	FALSE
chi-miR-873-3p	0	0.408859582	1.2139	0.70085	0.99632	FALSE

chi-miR-873-5p	15.57674515	2.150573865	-2.8722	0.001372	0.23635	TRUE
chi-miR-874-3p	469.1432073	390.5848716	-0.26495	0.39901	0.99632	FALSE
chi-miR-874-5p	13.59847613	11.05616925	-0.28364	0.55097	0.99632	FALSE
chi-miR-876-3p	0.689241875	1.049857743	0.61297	0.72719	0.99632	FALSE
chi-miR-876-5p	0.344620938	0	-1.0662	0.73552	0.99632	FALSE
chi-miR-877-3p	0.443263714	0.805515988	0.97229	0.67233	0.99632	FALSE
chi-miR-877-5p	45.77278371	30.72216411	-0.59173	0.18843	0.93016	FALSE
chi-miR-92a-3p	12693.99621	10332.22524	-0.29695	0.20537	0.94381	FALSE
chi-miR-92a-5p	13.66628013	13.01645582	-0.099093	0.86482	0.99632	FALSE
chi-miR-93-3p	16.88352981	11.13847153	-0.59349	0.18088	0.92563	FALSE
chi-miR-93-5p	6986.963989	8128.448075	0.21819	0.50281	0.99632	FALSE
chi-miR-9-3p	29.14681241	59.510396	1.0175	0.00267	0.34503	TRUE
chi-miR-9-5p	2566.822249	3549.074174	0.4671	0.21577	0.97476	FALSE
chi-miR-96	25.03350488	4.344819955	-2.5393	0.006692	0.43488	TRUE
chi-miR-98-3p	2.041753592	1.262811535	-0.79259	0.43623	0.99632	FALSE
chi-miR-98-5p	3437.945257	3872.205682	0.1716	0.35772	0.99632	FALSE
chi-miR-99a-3p	846.1773201	1201.69772	0.50657	0.37352	0.99632	FALSE
chi-miR-99a-5p	1592476.035	1385092.368	-0.20129	0.73023	0.99632	FALSE
chi-miR-99b-3p	588.1264923	498.4510142	-0.23862	0.39295	0.99632	FALSE
chi-miR-99b-5p	33786.57999	32904.94979	-0.038123	0.86431	0.99632	FALSE
novel_1000	0.383716344	0.402757994	0.093353	0.97215	0.99939	FALSE
novel_1001	0.130556971	0.578920558	1.132	0.64013	0.99632	FALSE
novel_1004	0.130556971	0.204429791	0.05166	0.98696	0.99939	FALSE
novel_1006	0.253159373	0.613289372	1.2003	0.68945	0.99632	FALSE
novel_1007	18.19543717	0.233901939	-6.3504	0.036093	0.81581	TRUE
novel_1008	0.130556971	0	-0.52541	0.86798	0.99632	FALSE
novel_1009	2.476647098	2.628133235	0.065194	0.94849	0.99939	FALSE
novel_101	302.774842	273.1471777	-0.14556	0.65772	0.99632	FALSE
novel_1011	0.890974335	3.308465392	1.8422	0.1804	0.92563	FALSE
novel_1012	0.541536716	0	-1.5903	0.61278	0.99632	FALSE
novel_1013	0	0.233901939	0.62873	0.84233	0.99632	FALSE
novel_1014	0.336046843	1.249911464	1.6651	0.32071	0.99632	FALSE
novel_1015	0	0.408859582	1.2139	0.70085	0.99632	FALSE
novel_1016	0.214063967	0	-0.52541	0.86798	0.99632	FALSE
novel_1018	0	0.16024954	0.62873	0.84233	0.99632	FALSE
novel_102	116.712753	173.8373269	0.57438	0.35336	0.99632	FALSE
novel_1020	18.86135123	18.78655099	-0.025229	0.96627	0.99939	FALSE
novel_1021	0.214063967	0.364679331	0.62308	0.83951	0.99632	FALSE
novel_1022	0.652784855	0	-1.8813	0.54706	0.99632	FALSE
novel_1027	4.878527183	1.805555247	-1.4463	0.12488	0.88062	FALSE
novel_1028	0.601084086	0.569109122	-0.049606	0.98072	0.99939	FALSE
novel_1029	2.464980981	3.091040447	0.30611	0.71689	0.99632	FALSE
novel_1030	0	0.418671017	1.2139	0.70085	0.99632	FALSE
novel_1031	0	0.402757994	1.2139	0.70085	0.99632	FALSE
novel_1032	0	0.935607756	2.367	0.44527	0.99632	FALSE
novel_109	170.8617024	128.4612103	-0.4158	0.43127	0.99632	FALSE
novel_114	384.5270171	182.1583168	-1.08	0.080202	0.82935	FALSE
novel_120	211.8313068	148.4803364	-0.51729	0.14256	0.88797	FALSE
novel_123	268.3039859	201.5337688	-0.41572	0.26604	0.99632	FALSE
novel_125	147.6548612	176.0628894	0.25254	0.36488	0.99632	FALSE
novel_130	104.5572054	121.2475561	0.20664	0.70066	0.99632	FALSE
novel_132	132.8226689	126.8457309	-0.073631	0.74808	0.99632	FALSE
novel_135	0	0	NA	NA	NA	NA
novel_136	72.89431731	79.04821185	0.12013	0.86493	0.99632	FALSE
novel_144	105.9268143	50.23756444	-1.0817	0.13549	0.88062	FALSE
novel_145	45.1263709	23.32251186	-0.96305	0.24538	0.98825	FALSE
novel_146	72.435011	82.49324466	0.18543	0.87303	0.99632	FALSE
novel_148	19.75039567	44.89464261	1.1834	0.11574	0.88062	FALSE

novel_149	38.82193478	30.15498996	-0.35438	0.64022	0.99632	FALSE
novel_15	25267.35635	19835.69268	-0.34919	0.12538	0.88062	FALSE
novel_150	1.282371392	1.819717481	0.57603	0.59649	0.99632	FALSE
novel_152	111.6353547	37.81001456	-1.5685	0.05227	0.82935	FALSE
novel_154	0.130556971	0.214241227	0.05166	0.98696	0.99939	FALSE
novel_155	78.77492907	47.14150646	-0.74548	0.055695	0.82935	FALSE
novel_158	106.3494496	75.78027712	-0.4919	0.37243	0.99632	FALSE
novel_161	15.65435082	11.3855896	-0.43933	0.45043	0.99632	FALSE
novel_162	48.56052433	33.84530739	-0.5277	0.48717	0.99632	FALSE
novel_164	30.28622435	17.86654837	-0.78286	0.13967	0.88062	FALSE
novel_165	23.45839195	21.47815948	-0.11781	0.88211	0.99639	FALSE
novel_170	15.46500488	23.017464	0.56816	0.25279	0.99632	FALSE
novel_172	17.67654434	21.23898191	0.2807	0.58173	0.99632	FALSE
novel_174	54.04232632	53.17310488	-0.020412	0.96344	0.99939	FALSE
novel_175	32.90445842	25.3459743	-0.39523	0.4595	0.99632	FALSE
novel_176	1.254488466	0.748981534	-0.72296	0.58189	0.99632	FALSE
novel_180	44.51667388	52.77806026	0.24534	0.43209	0.99632	FALSE
novel_181	15.60905733	19.75628279	0.30904	0.58182	0.99632	FALSE
novel_182	63.61533559	28.21066686	-1.1797	0.006974	0.43488	TRUE
novel_187	25.98460896	39.45731231	0.61742	0.08904	0.86856	FALSE
novel_188	10.64876405	6.693568268	-0.72159	0.38593	0.99632	FALSE
novel_196	31.57245694	27.79493483	-0.2034	0.69088	0.99632	FALSE
novel_197	35.59094581	27.56884189	-0.38537	0.27177	0.99632	FALSE
novel_199	22.45040456	20.92027829	-0.081856	0.81403	0.99632	FALSE
novel_201	32.02740088	14.78080295	-1.107	0.12217	0.88062	FALSE
novel_203	11.04436798	15.54546418	0.52546	0.30728	0.99632	FALSE
novel_206	1.678215357	1.215580491	-0.43775	0.72555	0.99632	FALSE
novel_207	11.7102901	26.03833368	1.1814	0.006259	0.43488	TRUE
novel_210	0.78437699	0.844140518	0.083385	0.96055	0.99939	FALSE
novel_211	14.310958	15.18340497	0.086483	0.85481	0.99632	FALSE
novel_212	25.21611523	23.7394864	-0.11365	0.80602	0.99632	FALSE
novel_214	154.3453918	94.17008501	-0.70936	0.007753	0.43488	FALSE
novel_216	8.070513734	18.41711919	1.228	0.009892	0.46153	TRUE
novel_220	21.82106267	10.92794015	-1.0135	0.046205	0.81581	TRUE
novel_222	0.336046843	0.204429791	-0.48917	0.87684	0.99632	FALSE
novel_223	19.61362211	15.64061193	-0.35714	0.47596	0.99632	FALSE
novel_225	17.81518568	17.18190292	-0.064811	0.88558	0.99639	FALSE
novel_226	2.144677388	1.084803099	-1.103	0.38837	0.99632	FALSE
novel_227	5.008873815	5.411167262	0.11972	0.84569	0.99632	FALSE
novel_228	0.380208682	0.930737659	1.2152	0.52633	0.99632	FALSE
novel_232	15.79456093	26.14893155	0.69768	0.34616	0.99632	FALSE
novel_235	23.11915575	30.23697636	0.39378	0.64336	0.99632	FALSE
novel_238	12.96254937	17.97389079	0.50195	0.31789	0.99632	FALSE
novel_240	7.181560599	4.137163071	-0.85759	0.34035	0.99632	FALSE
novel_241	16.08760998	16.55142156	0.042777	0.91108	0.99791	FALSE
novel_242	14.10024561	5.904107787	-1.23	0.18239	0.92563	FALSE
novel_244	21.10134344	17.76415965	-0.22547	0.58602	0.99632	FALSE
novel_248	15.57694904	7.522488216	-1.0839	0.15401	0.92563	FALSE
novel_249	13.40051774	3.825910158	-1.8541	0.054379	0.82935	FALSE
novel_250	7.989299454	8.871090915	0.11578	0.83176	0.99632	FALSE
novel_251	0.253159373	0	-0.52541	0.86798	0.99632	FALSE
novel_253	7.348390207	6.498710256	-0.12186	0.88619	0.99639	FALSE
novel_254	14.62808847	6.711409677	-1.1669	0.13958	0.88062	FALSE
novel_255	0.862198028	0.844102676	-0.1045	0.94864	0.99939	FALSE
novel_256	13.34759734	18.81070226	0.50478	0.2621	0.99632	FALSE
novel_258	8.127707211	10.44170433	0.31416	0.58486	0.99632	FALSE
novel_262	5.622391779	4.375459183	-0.32274	0.59697	0.99632	FALSE
novel_263	9.423377572	4.588909729	-1.0221	0.19337	0.9318	FALSE

novel_264	6.329965236	6.56407387	0.0025909	0.99704	0.99939	FALSE
novel_265	6.016332716	8.471067285	0.51939	0.37571	0.99632	FALSE
novel_266	14.66887238	10.78462953	-0.48991	0.4113	0.99632	FALSE
novel_267	3.627132153	0.233901939	-3.6968	0.003054	0.35091	TRUE
novel_270	12.9053415	4.989458021	-1.4049	0.15528	0.92563	FALSE
novel_272	15.69173036	6.31472356	-1.3356	0.075977	0.82935	FALSE
novel_274	11.02107793	16.83262305	0.59331	0.15904	0.92563	FALSE
novel_275	9.15027368	6.954786883	-0.41178	0.4777	0.99632	FALSE
novel_276	9.898590786	6.087131609	-0.66268	0.3569	0.99632	FALSE
novel_28	7878.127365	7346.499409	-0.10067	0.71569	0.99632	FALSE
novel_282	5.475249638	2.668113714	-1.0293	0.13776	0.88062	FALSE
novel_284	3.057694859	0.613289372	-2.3274	0.072649	0.82935	FALSE
novel_285	13.72486232	8.467224512	-0.67955	0.27721	0.99632	FALSE
novel_286	0	0.16024954	0.62873	0.84233	0.99632	FALSE
novel_288	9.282042334	0.607187785	-3.9442	0.079073	0.82935	FALSE
novel_291	4.703668003	10.57494881	1.1414	0.065308	0.82935	FALSE
novel_292	2.140504336	0.405808788	-2.4406	0.12999	0.88062	FALSE
novel_294	5.352965543	2.818634332	-0.92807	0.24711	0.98825	FALSE
novel_295	1.749594732	4.829068725	1.5147	0.12346	0.88062	FALSE
novel_297	6.994071957	6.747016814	-0.030669	0.95769	0.99939	FALSE
novel_298	4.907195516	2.930828748	-0.81447	0.43929	0.99632	FALSE
novel_301	3.161869755	1.819717481	-0.87218	0.44803	0.99632	FALSE
novel_303	2.275080526	1.86939621	-0.29826	0.76712	0.99632	FALSE
novel_304	4.39177907	2.049351135	-1.139	0.23256	0.97831	FALSE
novel_305	3.529057435	1.079329891	-1.6954	0.16062	0.92563	FALSE
novel_306	4.214280092	1.982332192	-1.1649	0.39864	0.99632	FALSE
novel_307	6.694230965	5.824422314	-0.24546	0.76544	0.99632	FALSE
novel_309	3.774677877	1.154790321	-1.7132	0.064945	0.82935	FALSE
novel_31	82.62403446	109.2867127	0.39841	0.24161	0.98745	FALSE
novel_310	1.954215328	6.978761982	1.8874	0.024154	0.73457	TRUE
novel_313	3.003463672	2.647210239	-0.19628	0.80793	0.99632	FALSE
novel_315	3.564059469	3.366248141	-0.065856	0.93747	0.99939	FALSE
novel_317	1.345176673	1.806169628	0.44795	0.76164	0.99632	FALSE
novel_318	3.86138068	4.1482241	0.13878	0.85843	0.99632	FALSE
novel_319	0.808336615	0.428482453	-0.95659	0.67005	0.99632	FALSE
novel_322	1.522413845	1.536079571	-0.026087	0.98059	0.99939	FALSE
novel_324	0.974481331	1.045481673	0.085655	0.96115	0.99939	FALSE
novel_325	3.114711811	1.526268136	-1.0374	0.22854	0.9752	FALSE
novel_326	1.044455768	0.364679331	-1.43	0.51978	0.99632	FALSE
novel_327	1.104003138	0.467803878	-1.4915	0.47936	0.99632	FALSE
novel_328	3.982487829	4.041603845	0.088925	0.91424	0.99791	FALSE
novel_329	1.187510134	1.630597604	0.32893	0.80343	0.99632	FALSE
novel_330	2.13998073	1.205731214	-0.86559	0.49895	0.99632	FALSE
novel_331	2.080956966	0.361628537	-2.3992	0.17996	0.92563	FALSE
novel_332	2.325684028	3.36978169	0.47663	0.56959	0.99632	FALSE
novel_333	5.199625893	3.184880986	-0.71979	0.38131	0.99632	FALSE
novel_334	0.190104341	0.16024954	0.05166	0.98696	0.99939	FALSE
novel_335	2.386994055	2.281965639	-0.11368	0.889	0.99639	FALSE
novel_336	6.363774161	1.215580491	-2.4434	0.032334	0.81544	TRUE
novel_337	2.042992643	2.558190684	0.38715	0.66718	0.99632	FALSE
novel_338	2.663767384	1.499846781	-0.85684	0.44345	0.99632	FALSE
novel_339	2.204070925	1.970155586	-0.11018	0.90595	0.99759	FALSE
novel_340	1.104099056	0.701705817	-0.67431	0.71517	0.99632	FALSE
novel_341	0.581775254	0.480748621	-0.25033	0.9166	0.99791	FALSE
novel_343	3.486346399	3.391288275	-0.13068	0.85304	0.99632	FALSE
novel_344	3.019850553	1.046806949	-1.507	0.20096	0.9318	FALSE
novel_345	2.753038603	4.162460719	0.66648	0.40614	0.99632	FALSE
novel_346	0.130556971	0.628053419	1.221	0.65273	0.99632	FALSE

novel_347	2.778557	1.0111507	-1.4181	0.17947	0.92563	FALSE
novel_350	2.35936088	2.302831273	-0.10584	0.91877	0.99791	FALSE
novel_351	0.618232274	0.405808788	-0.56274	0.79324	0.99632	FALSE
novel_352	1.12455102	2.90334776	1.498	0.22049	0.9752	FALSE
novel_353	0.253159373	1.401592331	2.393	0.1699	0.92563	FALSE
novel_354	2.424320352	4.150145655	0.84039	0.39762	0.99632	FALSE
novel_355	1.85442942	1.110610071	-0.65646	0.58637	0.99632	FALSE
novel_356	0.380208682	1.982358762	2.2942	0.13442	0.88062	FALSE
novel_357	0.906359866	1.847984708	0.98696	0.48418	0.99632	FALSE
novel_358	2.330608677	1.0909872	-1.1192	0.38994	0.99632	FALSE
novel_361	0.763925026	0.628053419	-0.31142	0.85903	0.99632	FALSE
novel_363	0	0.16024954	0.62873	0.84233	0.99632	FALSE
novel_364	0.740215151	1.006894984	0.42757	0.80118	0.99632	FALSE
novel_367	1.55924064	0.233901939	-2.4413	0.12855	0.88062	FALSE
novel_368	1.560013997	0.214241227	-2.5314	0.10443	0.88062	FALSE
novel_369	0.652784855	1.277658094	0.82252	0.63611	0.99632	FALSE
novel_37	6857.637479	6110.955497	-0.16622	0.26813	0.99632	FALSE
novel_370	2.683695741	0.963260601	-1.4752	0.14535	0.89996	FALSE
novel_371	3.81465461	0.989681956	-1.9982	0.1366	0.88062	FALSE
novel_372	0.261113942	0.402757994	0.15063	0.96194	0.99939	FALSE
novel_373	1.436638238	1.792693017	0.37745	0.70858	0.99632	FALSE
novel_374	2.009947365	0.99828847	-1.0887	0.43478	0.99632	FALSE
novel_375	2.31009461	3.01304982	0.31446	0.74295	0.99632	FALSE
novel_377	0.380208682	1.520084034	1.8751	0.285	0.99632	FALSE
novel_379	0.775802895	1.607841426	1.0101	0.56709	0.99632	FALSE
novel_381	1.309588929	0.97491791	-0.32512	0.80553	0.99632	FALSE
novel_382	0	0	NA	NA	NA	NA
novel_384	1.314655361	2.006978916	0.72098	0.55606	0.99632	FALSE
novel_385	1.10022162	0.610238579	-0.87401	0.58575	0.99632	FALSE
novel_387	1.966916609	0.569109122	-1.674	0.20719	0.94795	FALSE
novel_388	3.674904019	0.682045105	-2.4847	0.29842	0.99632	FALSE
novel_389	0	0.204429791	0.62873	0.84233	0.99632	FALSE
novel_390	0.802650658	1.684671804	0.98774	0.45397	0.99632	FALSE
novel_391	1.032993537	0.588731993	-0.88725	0.62663	0.99632	FALSE
novel_392	0.573820685	2.549546329	2.1838	0.10776	0.88062	FALSE
novel_393	1.690570968	1.682769987	-0.02985	0.97812	0.99939	FALSE
novel_394	1.068307425	1.177501828	0.22805	0.85174	0.99632	FALSE
novel_395	1.551190152	2.237702875	0.42089	0.69608	0.99632	FALSE
novel_397	0.458649245	0.201378997	-0.5808	0.84044	0.99632	FALSE
novel_398	2.128040756	2.738759991	0.4284	0.65971	0.99632	FALSE
novel_4	2.340813356	4.42747337	0.96595	0.33306	0.99632	FALSE
novel_400	1.443973282	0.415620223	-1.8701	0.2337	0.97831	FALSE
novel_401	2.112655225	1.458088944	-0.51325	0.63849	0.99632	FALSE
novel_403	430.1008121	396.8851454	-0.11401	0.63706	0.99632	FALSE
novel_404	2.06876079	0.364679331	-2.3197	0.18263	0.92563	FALSE
novel_407	0	0	NA	NA	NA	NA
novel_408	0.522227884	1.149848983	0.93983	0.65554	0.99632	FALSE
novel_409	0.320661312	0.640998162	0.78629	0.75273	0.99632	FALSE
novel_410	2.034942155	0.883424102	-1.3338	0.26519	0.99632	FALSE
novel_411	1.059733331	0.480748621	-0.97147	0.61091	0.99632	FALSE
novel_412	0.380208682	0.682127618	0.77822	0.74294	0.99632	FALSE
novel_413	0.886527428	0.361628537	-1.0566	0.59847	0.99632	FALSE
novel_414	0.344620938	1.187313264	1.6049	0.29906	0.99632	FALSE
novel_415	0.214063967	0.201378997	0.05166	0.98696	0.99939	FALSE
novel_416	0.727717756	0.636659933	-0.32114	0.86885	0.99632	FALSE
novel_417	0	0	NA	NA	NA	NA
novel_418	0.6421919	0	-1.7782	0.57011	0.99632	FALSE
novel_420	0.672093687	1.065142386	0.56624	0.72241	0.99632	FALSE

novel_421	0.130556971	0.320499081	0.6153	0.84563	0.99632	FALSE
novel_422	0.253159373	0.608392706	1.1822	0.59759	0.99632	FALSE
novel_423	1.092540907	0	-2.5921	0.3223	0.99632	FALSE
novel_424	0	0	NA	NA	NA	NA
novel_425	1.041044024	0	-2.4607	0.22584	0.9752	FALSE
novel_426	0.190104341	0.320499081	0.6153	0.84563	0.99632	FALSE
novel_427	0.918441623	1.362967801	0.56866	0.63427	0.99632	FALSE
novel_428	0.380208682	0.480748621	0.3122	0.91809	0.99791	FALSE
novel_429	1.669869253	0.610238579	-1.4971	0.31941	0.99632	FALSE
novel_430	1.607849385	0.59858127	-1.4151	0.3509	0.99632	FALSE
novel_432	0.383716344	0	-1.0662	0.73552	0.99632	FALSE
novel_433	0.60965818	1.445734741	1.2662	0.35645	0.99632	FALSE
novel_434	0	0	NA	NA	NA	NA
novel_435	0.585698554	0.201378997	-1.1009	0.67955	0.99632	FALSE
novel_436	0.759478119	0	-1.9892	0.52341	0.99632	FALSE
novel_437	1.238483409	0.374490767	-1.6693	0.31288	0.99632	FALSE
novel_438	0.261113942	0.96322276	1.3668	0.5427	0.99632	FALSE
novel_439	0.410979745	2.34023961	2.534	0.11269	0.88062	FALSE
novel_440	0	0.418671017	1.2139	0.70085	0.99632	FALSE
novel_441	0.344620938	0	-1.0662	0.73552	0.99632	FALSE
novel_442	0.214063967	0	-0.52541	0.86798	0.99632	FALSE
novel_443	157.8797512	164.1546775	0.059145	0.77778	0.99632	FALSE
novel_444	0.190104341	0	-0.52541	0.86798	0.99632	FALSE
novel_445	0.573820685	0.233901939	-1.021	0.65601	0.99632	FALSE
novel_446	1.436126681	0.701705817	-1.1389	0.61893	0.99632	FALSE
novel_447	0.601084086	0.214241227	-1.1268	0.6678	0.99632	FALSE
novel_449	1.341253372	0.467803878	-1.6838	0.3399	0.99632	FALSE
novel_450	0.522227884	0.361628537	-0.47894	0.85245	0.99632	FALSE
novel_452	1.013826488	0.855239388	-0.1518	0.92412	0.99939	FALSE
novel_453	0.130556971	0.805515988	1.5968	0.58542	0.99632	FALSE
novel_454	0.46722334	1.631200714	2.0088	0.14201	0.88797	FALSE
novel_455	0.130556971	0.415620223	0.63686	0.84027	0.99632	FALSE
novel_456	0	0	NA	NA	NA	NA
novel_457	0.391670913	0.607187785	0.37407	0.87959	0.99639	FALSE
novel_458	0.46722334	0	-1.1604	0.71302	0.99632	FALSE
novel_459	0.618232274	0.16024954	-1.1554	0.6671	0.99632	FALSE
novel_460	0	0	NA	NA	NA	NA
novel_462	0.214063967	0.201378997	0.05166	0.98696	0.99939	FALSE
novel_463	0	0	NA	NA	NA	NA
novel_464	0.428127933	1.162793726	1.5183	0.4042	0.99632	FALSE
novel_465	0.681287306	0.521878078	-0.23456	0.90858	0.99778	FALSE
novel_466	0.594272649	0.402757994	-0.52968	0.8285	0.99632	FALSE
novel_468	0.589206216	0.566058328	0.02719	0.98749	0.99939	FALSE
novel_469	0.428127933	0	-1.1604	0.71302	0.99632	FALSE
novel_470	0.795839221	0.435280936	-1.0239	0.5687	0.99632	FALSE
novel_471	0.410979745	0.821429011	0.99938	0.61665	0.99632	FALSE
novel_472	0.261113942	0.640998162	0.82364	0.7822	0.99632	FALSE
novel_473	0.261113942	0.613289372	0.71334	0.81393	0.99632	FALSE
novel_474	1.061495988	0.80297322	-0.36314	0.82151	0.99632	FALSE
novel_475	0.594272649	0	-1.6941	0.58906	0.99632	FALSE
novel_476	0	0.16024954	0.62873	0.84233	0.99632	FALSE
novel_477	0.190104341	0	-0.52541	0.86798	0.99632	FALSE
novel_478	0.261113942	0	-1.0116	0.74866	0.99632	FALSE
novel_479	3.054403133	0.428482453	-2.9334	0.082898	0.8451	FALSE
novel_48	2022.425988	1684.907718	-0.26328	0.23763	0.97891	FALSE
novel_480	0	0.402757994	1.2139	0.70085	0.99632	FALSE
novel_481	0	0.64272368	1.8357	0.55798	0.99632	FALSE
novel_482	0	0	NA	NA	NA	NA

novel_483	0	0.773538913	2.1206	0.49596	0.99632	FALSE
novel_484	0	0.613289372	1.7776	0.57105	0.99632	FALSE
novel_485	0.834934627	0.364679331	-1.0693	0.55535	0.99632	FALSE
novel_487	0	0	NA	NA	NA	NA
novel_488	0.428127933	0.832483209	1.0072	0.61174	0.99632	FALSE
novel_490	0.344620938	0.201378997	-0.48917	0.87684	0.99632	FALSE
novel_491	0	0	NA	NA	NA	NA
novel_492	0.383716344	0	-1.0662	0.73552	0.99632	FALSE
novel_493	0.205489872	0.204429791	0.05166	0.98696	0.99939	FALSE
novel_494	0.597160785	0	-1.7382	0.57911	0.99632	FALSE
novel_495	0.380208682	0	-1.1604	0.71302	0.99632	FALSE
novel_497	1.202741832	0.723257075	-0.60939	0.73412	0.99632	FALSE
novel_499	0.190104341	0	-0.52541	0.86798	0.99632	FALSE
novel_500	0.719763187	0.16024954	-1.391	0.52687	0.99632	FALSE
novel_502	0.344620938	0	-1.0662	0.73552	0.99632	FALSE
novel_503	0	0	NA	NA	NA	NA
novel_504	1.914704283	0.783350349	-1.3035	0.28763	0.99632	FALSE
novel_507	0.570313023	1.42434851	1.3268	0.44676	0.99632	FALSE
novel_509	1.280608735	0.16024954	-2.1938	0.31305	0.99632	FALSE
novel_51	2246.064843	1786.632272	-0.33065	0.27713	0.99632	FALSE
novel_510	1.200007528	0.435280936	-1.582	0.32824	0.99632	FALSE
novel_512	0.451218283	0.521878078	0.12034	0.95563	0.99939	FALSE
novel_513	1.622199753	1.138877298	-0.54919	0.64372	0.99632	FALSE
novel_515	1.59177436	1.235241203	-0.29451	0.8108	0.99632	FALSE
novel_517	0.443263714	0	-1.1604	0.71302	0.99632	FALSE
novel_518	0	0.55440102	1.6343	0.60364	0.99632	FALSE
novel_519	0.740215151	0.408859582	-0.85712	0.64287	0.99632	FALSE
novel_520	0.913898797	0.408859582	-1.2113	0.63708	0.99632	FALSE
novel_522	0.6421919	0.817719163	0.37033	0.88351	0.99639	FALSE
novel_523	1.0637646	1.216785412	0.084106	0.95378	0.99939	FALSE
novel_524	0.344620938	0.214241227	-0.48925	0.87578	0.99632	FALSE
novel_525	0.772748871	0	-2.06	0.50814	0.99632	FALSE
novel_526	0	0.16024954	0.62873	0.84233	0.99632	FALSE
novel_527	0.391670913	0.604136991	0.38225	0.89452	0.99639	FALSE
novel_528	0	0.672233669	1.891	0.54568	0.99632	FALSE
novel_529	1.342192618	0.521878078	-1.3145	0.45698	0.99632	FALSE
novel_530	6.360766006	3.146960519	-1.0216	0.10588	0.88062	FALSE
novel_531	1.24994564	0.628053419	-1.1095	0.54265	0.99632	FALSE
novel_532	0.989866862	0.935607756	-0.14018	0.93856	0.99939	FALSE
novel_533	0.261113942	0.588731993	0.65738	0.80087	0.99632	FALSE
novel_534	0.506318746	1.475289402	1.8959	0.22422	0.9752	FALSE
novel_535	3.365630785	0.364679331	-3.0192	0.016366	0.58352	TRUE
novel_536	0	0	NA	NA	NA	NA
novel_537	166.3641194	150.9280689	-0.13744	0.64265	0.99632	FALSE
novel_539	0	0.233901939	0.62873	0.84233	0.99632	FALSE
novel_540	0.190104341	0.16024954	0.05166	0.98696	0.99939	FALSE
novel_541	0.214063967	0.408859582	0.63686	0.84027	0.99632	FALSE
novel_542	3.733813362	4.762647153	0.31941	0.71319	0.99632	FALSE
novel_543	0.380208682	0	-1.1604	0.71302	0.99632	FALSE
novel_544	0.404168308	0.201378997	-0.58206	0.84661	0.99632	FALSE
novel_546	0	0	NA	NA	NA	NA
novel_547	0.522227884	0	-1.5995	0.61066	0.99632	FALSE
novel_548	5.908354537	6.754380565	0.15272	0.80466	0.99632	FALSE
novel_549	78.1195257	45.02314385	-0.78924	0.10361	0.88062	FALSE
novel_551	0.214063967	0.701705817	1.3646	0.64327	0.99632	FALSE
novel_552	3.133355254	2.012489964	-0.67784	0.42959	0.99632	FALSE
novel_553	0.380208682	0.204429791	-0.58337	0.85331	0.99632	FALSE
novel_554	6.401140724	6.061939018	-0.12643	0.84467	0.99632	FALSE

novel_555	1.769177419	0.982883473	-0.82521	0.47023	0.99632	FALSE
novel_556	2564.760638	2100.819103	-0.28754	0.065804	0.82935	FALSE
novel_558	0.130556971	0.632912244	1.2363	0.64791	0.99632	FALSE
novel_559	2.154012791	1.016047366	-1.0591	0.46392	0.99632	FALSE
novel_561	3.937456714	7.191462702	0.87738	0.16919	0.92563	FALSE
novel_562	0.253159373	0.402757994	0.63686	0.84027	0.99632	FALSE
novel_563	0.55011081	0.320499081	-0.46528	0.83732	0.99632	FALSE
novel_564	28.22687226	14.38108473	-0.99085	0.16632	0.92563	FALSE
novel_565	0	0.233901939	0.62873	0.84233	0.99632	FALSE
novel_566	1.904111328	2.732778758	0.58749	0.63294	0.99632	FALSE
novel_567	0.336046843	0.16024954	-0.48917	0.87684	0.99632	FALSE
novel_569	0.934446679	1.110527558	0.29327	0.86642	0.99632	FALSE
novel_57	1391.504337	1919.192512	0.4641	0.34529	0.99632	FALSE
novel_570	0.652784855	0	-1.8813	0.54706	0.99632	FALSE
novel_571	0.711808618	1.051621102	0.64925	0.72747	0.99632	FALSE
novel_572	0	0	NA	NA	NA	NA
novel_573	0.581775254	0.748981534	0.26714	0.88927	0.99639	FALSE
novel_574	0	0.361628537	1.2001	0.70412	0.99632	FALSE
novel_576	1.579322829	0.71465056	-1.0607	0.44331	0.99632	FALSE
novel_577	0	0	NA	NA	NA	NA
novel_578	0.130556971	0	-0.52541	0.86798	0.99632	FALSE
novel_579	3.514075493	2.06648165	-0.72422	0.42185	0.99632	FALSE
novel_580	2.958324589	0.408859582	-2.821	0.070286	0.82935	FALSE
novel_581	36.54456699	17.23777279	-1.1006	0.11689	0.88062	FALSE
novel_583	21.83314627	21.24161713	-0.062781	0.90316	0.99639	FALSE
novel_584	4.330890284	2.93758939	-0.60244	0.39482	0.99632	FALSE
novel_587	0.130556971	0.201378997	0.05166	0.98696	0.99939	FALSE
novel_588	0.130556971	1.22844272	2.1935	0.16489	0.92563	FALSE
novel_589	0.696423087	0.204429791	-1.2802	0.61328	0.99632	FALSE
novel_59	555.6619792	484.1443676	-0.19784	0.49771	0.99632	FALSE
novel_590	0.261113942	0.16024954	-0.43457	0.89053	0.99639	FALSE
novel_591	0.458649245	0	-1.1604	0.71302	0.99632	FALSE
novel_592	0.190104341	1.699342064	2.6887	0.11656	0.88062	FALSE
novel_593	0.214063967	0	-0.52541	0.86798	0.99632	FALSE
novel_595	0.130556971	0	-0.52541	0.86798	0.99632	FALSE
novel_598	0.877583559	0.405808788	-1.1143	0.56968	0.99632	FALSE
novel_600	4.673486758	2.486357589	-0.9988	0.21588	0.97476	FALSE
novel_601	6.731029471	8.109405347	0.26626	0.65349	0.99632	FALSE
novel_603	0.190104341	1.043756155	2.004	0.36209	0.99632	FALSE
novel_604	1.611606798	3.670702572	1.2521	0.13459	0.88062	FALSE
novel_607	1.424556481	2.398638039	0.7905	0.4396	0.99632	FALSE
novel_608	225.7725926	171.1506475	-0.40342	0.080208	0.82935	FALSE
novel_609	0.558684904	0.566058328	0.027497	0.98834	0.99939	FALSE
novel_610	0.214063967	0.402757994	0.63686	0.84027	0.99632	FALSE
novel_611	0	0	NA	NA	NA	NA
novel_613	53.63836591	57.79137444	0.098587	0.79356	0.99632	FALSE
novel_615	0.854243459	0	-2.1846	0.38873	0.99632	FALSE
novel_616	0.870772122	0.796909473	-0.18405	0.89555	0.99639	FALSE
novel_617	0.60573488	0	-1.7525	0.5759	0.99632	FALSE
novel_618	2.620366854	1.787740407	-0.5092	0.55243	0.99632	FALSE
novel_619	61.39834872	14.5684034	-2.0766	0.44878	0.99632	FALSE
novel_620	1.022400582	0.364679331	-1.3007	0.50255	0.99632	FALSE
novel_621	0.190104341	1.110527558	2.0338	0.35218	0.99632	FALSE
novel_622	0.428127933	0.844140518	1.027	0.60419	0.99632	FALSE
novel_623	0.506318746	0	-1.1604	0.71302	0.99632	FALSE
novel_624	1.260424175	0	-2.7572	0.28169	0.99632	FALSE
novel_626	2.350025477	0	-3.6972	0.13561	0.88062	FALSE
novel_627	6.938713873	1.364172723	-2.373	0.034405	0.81581	TRUE

novel_628	20.35121262	0.701705817	-4.8859	0.10258	0.88062	FALSE
novel_630	0	0	NA	NA	NA	NA
novel_631	0.644830286	0	-1.8208	0.56054	0.99632	FALSE
novel_632	3.265862529	2.936346627	-0.1499	0.84981	0.99632	FALSE
novel_633	1.3344878	2.980063523	1.1578	0.27243	0.99632	FALSE
novel_634	0.938893586	0.927686865	0.015794	0.99111	0.99939	FALSE
novel_638	0.253159373	0	-0.52541	0.86798	0.99632	FALSE
novel_639	24.72089547	12.3138124	-1.0237	0.2189	0.9752	FALSE
novel_64	253.2380201	522.9115035	1.0465	0.063799	0.82935	FALSE
novel_640	0.130556971	0.214241227	0.05166	0.98696	0.99939	FALSE
novel_641	0.214063967	0	-0.52541	0.86798	0.99632	FALSE
novel_642	0	0	NA	NA	NA	NA
novel_643	1.524176502	0.204429791	-2.5002	0.11376	0.88062	FALSE
novel_644	33.25060198	23.94441981	-0.48819	0.22758	0.9752	FALSE
novel_649	0.466603814	0	-1.4415	0.64719	0.99632	FALSE
novel_65	1020.845688	745.4420271	-0.45342	0.040437	0.81581	FALSE
novel_650	0	0	NA	NA	NA	NA
novel_651	1.566683652	0	-3.1165	0.26354	0.99632	FALSE
novel_652	0.771879595	0.408859582	-0.96024	0.66921	0.99632	FALSE
novel_654	0.391670913	0	-1.3195	0.67555	0.99632	FALSE
novel_655	0.419553839	0.320499081	-0.015258	0.9956	0.99939	FALSE
novel_656	1.745325762	1.273319866	-0.54121	0.73349	0.99632	FALSE
novel_658	0	0	NA	NA	NA	NA
novel_659	2.592591896	0.214241227	-3.2722	0.044302	0.81581	TRUE
novel_660	0.514273315	0.886474896	0.71557	0.68192	0.99632	FALSE
novel_661	0	0.682127618	1.9608	0.53033	0.99632	FALSE
novel_662	0	0.204429791	0.62873	0.84233	0.99632	FALSE
novel_663	6.014706316	2.885367893	-1.126	0.24604	0.98825	FALSE
novel_664	5.195572859	2.56680847	-1.1138	0.24767	0.98825	FALSE
novel_665	0	0.563007534	1.6759	0.59414	0.99632	FALSE
novel_666	0.763925026	0.415620223	-0.88096	0.63168	0.99632	FALSE
novel_668	0.736291851	1.030811412	0.34177	0.83092	0.99632	FALSE
novel_669	0.395594213	0.467803878	0.0073383	0.99773	0.99939	FALSE
novel_670	2.809577814	4.346601417	0.66936	0.45678	0.99632	FALSE
novel_672	1.789213745	3.84814574	1.0815	0.21548	0.97476	FALSE
novel_673	0.205489872	0	-0.52541	0.86798	0.99632	FALSE
novel_674	2.833037937	1.149931496	-1.2227	0.25987	0.99632	FALSE
novel_675	1.436638238	2.175752457	0.66496	0.49739	0.99632	FALSE
novel_678	3.44745488	1.540976238	-1.1652	0.22447	0.9752	FALSE
novel_679	0.570313023	0.204429791	-1.0738	0.73226	0.99632	FALSE
novel_681	0.973446167	0.43833173	-1.3333	0.49242	0.99632	FALSE
novel_682	0	0	NA	NA	NA	NA
novel_684	0.395594213	1.164601757	1.5313	0.36057	0.99632	FALSE
novel_685	8.175224292	9.071886541	0.12539	0.81751	0.99632	FALSE
novel_686	1.194321571	0.394151479	-1.6097	0.3849	0.99632	FALSE
novel_687	2.837796699	2.83461587	-0.031877	0.97839	0.99939	FALSE
novel_688	0.205489872	0.773538913	1.5475	0.52436	0.99632	FALSE
novel_690	1.068307425	0.924636071	-0.1067	0.94082	0.99939	FALSE
novel_691	16.84205775	19.18621762	0.18104	0.66092	0.99632	FALSE
novel_693	0	1.022204898	2.5102	0.35678	0.99632	FALSE
novel_694	0	0	NA	NA	NA	NA
novel_696	1.198864396	1.179347701	0.020821	0.98638	0.99939	FALSE
novel_697	1.994703617	1.477052761	-0.50279	0.60101	0.99632	FALSE
novel_698	2.440855467	1.454379096	-0.80512	0.43119	0.99632	FALSE
novel_699	0.130556971	0.61699922	1.2065	0.65859	0.99632	FALSE
novel_7	1620.270239	2265.25875	0.48373	0.33803	0.99632	FALSE
novel_700	0.344620938	0.739170098	0.95916	0.60739	0.99632	FALSE
novel_701	0.858274727	0.320499081	-1.1398	0.61969	0.99632	FALSE

novel_702	0.46722334	0.415620223	0.012567	0.99555	0.99939	FALSE
novel_706	0.60965818	1.532369724	1.3786	0.34182	0.99632	FALSE
novel_708	2.375186154	2.782895569	0.14216	0.9	0.99639	FALSE
novel_709	0	0.467803878	1.2139	0.70085	0.99632	FALSE
novel_710	0.783341826	0	-2.1352	0.49219	0.99632	FALSE
novel_711	1.120008194	0.408859582	-1.4825	0.42767	0.99632	FALSE
novel_712	2.26299877	2.420708594	0.089766	0.92053	0.99877	FALSE
novel_713	0.214063967	0	-0.52541	0.86798	0.99632	FALSE
novel_714	0	0	NA	NA	NA	NA
novel_715	0.799762521	0.204429791	-1.5271	0.46881	0.99632	FALSE
novel_716	0.130556971	0	-0.52541	0.86798	0.99632	FALSE
novel_719	2.163258726	1.154790321	-0.87264	0.43995	0.99632	FALSE
novel_724	0	0.204429791	0.62873	0.84233	0.99632	FALSE
novel_726	0.89003509	0.361628537	-1.0498	0.60528	0.99632	FALSE
novel_727	0	0.55440102	1.6343	0.60364	0.99632	FALSE
novel_728	1.708736668	0.578920558	-1.4977	0.31097	0.99632	FALSE
novel_729	0.419553839	0.903084814	1.0978	0.58184	0.99632	FALSE
novel_73	404.0101724	598.0655074	0.56711	0.085811	0.85316	FALSE
novel_730	8.578106276	3.258032526	-1.3761	0.10521	0.88062	FALSE
novel_731	0	0.233901939	0.62873	0.84233	0.99632	FALSE
novel_732	0.570313023	0.80297322	0.46375	0.83445	0.99632	FALSE
novel_733	0.190104341	0.402757994	0.63686	0.84027	0.99632	FALSE
novel_736	0.458649245	1.390538133	1.7879	0.23012	0.9752	FALSE
novel_737	0	0.204429791	0.62873	0.84233	0.99632	FALSE
novel_738	0.641322624	0	-1.8237	0.5599	0.99632	FALSE
novel_740	0.205489872	0.640998162	1.3095	0.66596	0.99632	FALSE
novel_741	0	0	NA	NA	NA	NA
novel_743	1.706326274	5.736593682	1.7448	0.032239	0.81544	TRUE
novel_744	0.760417364	0	-2.0445	0.51145	0.99632	FALSE
novel_745	0	0.935607756	2.367	0.44527	0.99632	FALSE
novel_746	0	0.16024954	0.62873	0.84233	0.99632	FALSE
novel_747	0.712332225	0	-1.9893	0.52337	0.99632	FALSE
novel_748	0.190104341	0.204429791	0.05166	0.98696	0.99939	FALSE
novel_749	3181.431104	3956.829545	0.31501	0.17861	0.92563	FALSE
novel_750	0.190104341	1.237049234	2.208	0.23491	0.97891	FALSE
novel_752	3.340761616	0.981678551	-1.8206	0.1083	0.88062	FALSE
novel_756	1.241991071	0.214241227	-2.2192	0.23615	0.97891	FALSE
novel_757	0.808336615	0.364679331	-0.96464	0.63536	0.99632	FALSE
novel_758	5.744506723	1.689448115	-1.7875	0.4245	0.99632	FALSE
novel_759	0.767432688	0.364679331	-0.88125	0.66695	0.99632	FALSE
novel_760	0	0.408859582	1.2139	0.70085	0.99632	FALSE
novel_763	0	0	NA	NA	NA	NA
novel_766	2.291655054	1.327902091	-0.78965	0.44484	0.99632	FALSE
novel_767	1.005252393	0.856964906	-0.24708	0.89083	0.99639	FALSE
novel_769	2.061636085	0.201378997	-2.8645	0.060366	0.82935	FALSE
novel_770	0	0.320499081	1.1924	0.70595	0.99632	FALSE
novel_772	7.63590472	2.044353852	-1.9521	0.026913	0.77301	TRUE
novel_773	0	0.588731993	1.7241	0.58319	0.99632	FALSE
novel_775	0.811844277	0.361628537	-0.9484	0.60771	0.99632	FALSE
novel_778	0.419553839	0.214241227	-0.58138	0.84326	0.99632	FALSE
novel_779	1.59445861	0.829432415	-0.95454	0.43001	0.99632	FALSE
novel_78	424.2797217	417.2037445	-0.023932	0.9333	0.99939	FALSE
novel_781	1.756787993	0.837342035	-1.1629	0.49542	0.99632	FALSE
novel_783	1.037786114	1.884763366	0.889	0.43965	0.99632	FALSE
novel_784	1.032993537	0.640998162	-0.66793	0.74306	0.99632	FALSE
novel_786	12.37349699	19.09049299	0.65362	0.17517	0.92563	FALSE
novel_787	2.615989915	1.001339264	-1.4009	0.16851	0.92563	FALSE
novel_788	13.84011818	15.40366469	0.17677	0.74624	0.99632	FALSE

novel_789	0.320661312	0.214241227	-0.48917	0.87684	0.99632	FALSE
novel_79	318.6125673	268.1961347	-0.25108	0.49573	0.99632	FALSE
novel_790	0.190104341	0.16024954	0.05166	0.98696	0.99939	FALSE
novel_793	0	0	NA	NA	NA	NA
novel_794	2.837962587	8.512012314	1.5345	0.031339	0.81544	TRUE
novel_795	1.108795715	3.016194399	1.3908	0.19741	0.9318	FALSE
novel_796	5.9789485	3.109420556	-1.0105	0.18142	0.92563	FALSE
novel_797	0.419553839	0.428482453	0.0070915	0.99783	0.99939	FALSE
novel_798	0.395594213	0.607187785	0.56614	0.79287	0.99632	FALSE
novel_799	3.682800674	0.64272368	-2.5706	0.10213	0.88062	FALSE
novel_80	217.5964824	200.0732598	-0.11851	0.73998	0.99632	FALSE
novel_801	5.738860183	4.993675895	-0.25353	0.70398	0.99632	FALSE
novel_803	2.880087913	2.994714383	0.11075	0.88479	0.99639	FALSE
novel_805	0	0.701705817	1.9454	0.53369	0.99632	FALSE
novel_807	3.062237684	3.82653137	0.35929	0.68256	0.99632	FALSE
novel_808	0.130556971	0	-0.52541	0.86798	0.99632	FALSE
novel_809	1.59177436	2.587783187	0.81261	0.49473	0.99632	FALSE
novel_810	0.261113942	0	-1.0116	0.74866	0.99632	FALSE
novel_813	0.823472396	0	-2.1439	0.48179	0.99632	FALSE
novel_816	0.466603814	0	-1.4415	0.64719	0.99632	FALSE
novel_818	0	0	NA	NA	NA	NA
novel_819	0	0	NA	NA	NA	NA
novel_82	1028.550088	25.28730884	-5.3468	0.001367	0.23635	TRUE
novel_820	0.510765653	0.233901939	-0.95841	0.7245	0.99632	FALSE
novel_821	0.320661312	0.201378997	-0.48917	0.87684	0.99632	FALSE
novel_822	0.60965818	0.204429791	-1.1365	0.62097	0.99632	FALSE
novel_823	0.253159373	0.233901939	0.05166	0.98696	0.99939	FALSE
novel_824	0.205489872	0.991489987	1.8996	0.3308	0.99632	FALSE
novel_825	1.218127364	0.739170098	-0.58098	0.72334	0.99632	FALSE
novel_826	0.130556971	0.701705817	1.3676	0.65772	0.99632	FALSE
novel_828	0.834934627	1.327902091	0.62588	0.64872	0.99632	FALSE
novel_829	0.832296241	0.408859582	-0.99045	0.6631	0.99632	FALSE
novel_83	445.2559321	412.2483188	-0.10922	0.64473	0.99632	FALSE
novel_830	8.883135559	8.411482037	-0.027187	0.9623	0.99939	FALSE
novel_832	0.783341826	0	-2.1352	0.49219	0.99632	FALSE
novel_833	0	0	NA	NA	NA	NA
novel_835	1.084836089	0.850901159	-0.42612	0.75008	0.99632	FALSE
novel_837	3.40494773	4.028621261	0.30726	0.65557	0.99632	FALSE
novel_842	3.024904936	3.228660994	0.18357	0.82626	0.99632	FALSE
novel_843	2.018043717	0.984729345	-1.0854	0.32293	0.99632	FALSE
novel_845	0.214063967	0.773538913	1.5476	0.5228	0.99632	FALSE
novel_846	0.130556971	0	-0.52541	0.86798	0.99632	FALSE
novel_850	0.404168308	0.821429011	1.003	0.58131	0.99632	FALSE
novel_851	62.46405307	52.6368733	-0.25456	0.65855	0.99632	FALSE
novel_855	0	0	NA	NA	NA	NA
novel_856	0	0.524928872	1.6018	0.6111	0.99632	FALSE
novel_858	0	0.978627758	2.4598	0.30082	0.99632	FALSE
novel_860	1.42656889	0.402757994	-1.7772	0.37481	0.99632	FALSE
novel_861	1.232939234	0	-2.7313	0.37321	0.99632	FALSE
novel_862	0.506318746	1.236390181	1.5882	0.44462	0.99632	FALSE
novel_864	0.214063967	0.214241227	0.05166	0.98696	0.99939	FALSE
novel_865	2.5130082	1.917368821	-0.45327	0.63459	0.99632	FALSE
novel_867	0.823722147	0.620050014	-0.39529	0.80763	0.99632	FALSE
novel_868	0.428127933	0.402757994	0.0024934	0.99935	0.99939	FALSE
novel_870	1.24792678	0	-2.7618	0.26858	0.99632	FALSE
novel_871	0.130556971	0	-0.52541	0.86798	0.99632	FALSE
novel_872	0.410979745	0.662384392	0.65638	0.78319	0.99632	FALSE
novel_873	1.044455768	0.672233669	-0.76888	0.71483	0.99632	FALSE

novel_874	0.410979745	0.569109122	0.48899	0.84209	0.99632	FALSE
novel_875	0	0.662384392	1.873	0.54968	0.99632	FALSE
novel_876	3.576631017	4.169741958	0.12376	0.90388	0.99639	FALSE
novel_878	1.091647526	0.374490767	-1.4289	0.40146	0.99632	FALSE
novel_880	2.089423093	0.569109122	-1.8605	0.13928	0.88062	FALSE
novel_881	1.922039327	0	-3.4095	0.11439	0.88062	FALSE
novel_883	0.130556971	0	-0.52541	0.86798	0.99632	FALSE
novel_884	0.205489872	0.817719163	1.6156	0.56566	0.99632	FALSE
novel_885	0.190104341	0.233901939	0.05166	0.98696	0.99939	FALSE
novel_886	4.789885199	4.02741634	-0.17427	0.81556	0.99632	FALSE
novel_888	0.712332225	0.814668369	0.073959	0.9686	0.99939	FALSE
novel_889	0.681287306	2.801866218	2.1816	0.10004	0.88062	FALSE
novel_89	459.507101	602.8359257	0.39048	0.3385	0.99632	FALSE
novel_890	0	1.017214446	2.5071	0.36492	0.99632	FALSE
novel_891	2.302759566	1.279383612	-0.8532	0.45645	0.99632	FALSE
novel_892	0	0.233901939	0.62873	0.84233	0.99632	FALSE
novel_895	17.92414841	6.613720496	-1.4777	0.004773	0.43488	TRUE
novel_896	1.771541949	1.606763689	-0.052901	0.9699	0.99939	FALSE
novel_897	0.894481997	0.428482453	-1.1308	0.55511	0.99632	FALSE
novel_898	0.261113942	0.613289372	0.71334	0.81393	0.99632	FALSE
novel_899	1.40917095	0.521878078	-1.323	0.42243	0.99632	FALSE
novel_9	13213.91518	15921.63142	0.26897	0.27272	0.99632	FALSE
novel_900	3.949334583	7.635400357	0.9768	0.12078	0.88062	FALSE
novel_901	0	0.204429791	0.62873	0.84233	0.99632	FALSE
novel_902	0.522227884	0.788302959	0.43679	0.84308	0.99632	FALSE
novel_903	2.063444607	2.728244829	0.4208	0.68366	0.99632	FALSE
novel_904	0	0.233901939	0.62873	0.84233	0.99632	FALSE
novel_906	0.570313023	0.811617575	0.47919	0.82667	0.99632	FALSE
novel_907	0	0.817719163	2.1931	0.48074	0.99632	FALSE
novel_908	0.799762521	0.613289372	-0.38114	0.84493	0.99632	FALSE
novel_910	0.46722334	0.214241227	-0.5803	0.83809	0.99632	FALSE
novel_911	3.606430438	1.938837565	-0.85069	0.34052	0.99632	FALSE
novel_912	2.141061758	1.06819318	-1.0511	0.34374	0.99632	FALSE
novel_913	0	0	NA	NA	NA	NA
novel_914	2.763529188	4.074620815	0.49088	0.50958	0.99632	FALSE
novel_915	0.886527428	0.886557409	0.13463	0.93359	0.99939	FALSE
novel_917	0	0.480748621	1.521	0.62965	0.99632	FALSE
novel_918	0.190104341	0.364679331	0.62304	0.8431	0.99632	FALSE
novel_919	1.178936039	0.16024954	-2.1642	0.25831	0.99632	FALSE
novel_923	0	0	NA	NA	NA	NA
novel_925	0.506318746	0	-1.1604	0.71302	0.99632	FALSE
novel_929	8.752895045	0.866814183	-3.4197	0.007645	0.43488	TRUE
novel_93	112.8955472	103.0636951	-0.13012	0.90298	0.99639	FALSE
novel_930	0.712332225	0.853951953	0.060999	0.96876	0.99939	FALSE
novel_932	0	0	NA	NA	NA	NA
novel_933	0	0	NA	NA	NA	NA
novel_935	2.363200317	0.361628537	-2.5162	0.083366	0.8451	FALSE
novel_936	30.49366042	28.43030289	-0.12166	0.81735	0.99632	FALSE
novel_938	0.253159373	1.324851298	2.3309	0.19165	0.9318	FALSE
novel_939	1.514982883	3.027896379	0.84052	0.36785	0.99632	FALSE
novel_940	0.214063967	0	-0.52541	0.86798	0.99632	FALSE
novel_943	0.130556971	0.214241227	0.05166	0.98696	0.99939	FALSE
novel_944	0.410979745	0.408859582	0.0023466	0.99939	0.99939	FALSE
novel_946	0.506318746	0.201378997	-0.58337	0.85331	0.99632	FALSE
novel_947	0.581775254	0.201378997	-1.1513	0.66371	0.99632	FALSE
novel_948	18.46146351	7.940330712	-1.2385	0.052021	0.82935	FALSE
novel_949	0	0	NA	NA	NA	NA
novel_950	2.425577904	2.342058913	-0.15719	0.89422	0.99639	FALSE

novel_951	0.458649245	0.604136991	0.55582	0.81801	0.99632	FALSE
novel_952	0.60965818	0.748981534	0.34743	0.84346	0.99632	FALSE
novel_955	0.858274727	3.030439147	1.6355	0.18036	0.92563	FALSE
novel_957	4.355713583	4.170973448	-0.12469	0.88373	0.99639	FALSE
novel_958	1.227021178	0	-2.7497	0.20018	0.9318	FALSE
novel_959	1.044455768	0	-2.5381	0.41126	0.99632	FALSE
novel_96	270.9847867	172.2165961	-0.65552	0.079584	0.82935	FALSE
novel_960	2.489048575	0.405808788	-2.6637	0.045493	0.81581	TRUE
novel_961	0.727717756	0.608392706	-0.39274	0.82527	0.99632	FALSE
novel_963	0	0	NA	NA	NA	NA
novel_965	2.513519757	2.373421606	-0.0059372	0.9941	0.99939	FALSE
novel_967	0.763925026	0	-2.0425	0.44073	0.99632	FALSE
novel_969	0.391670913	0.320499081	-0.17884	0.95476	0.99939	FALSE
novel_971	1.012637491	0	-2.4152	0.43509	0.99632	FALSE
novel_972	0.458649245	0.204429791	-0.58072	0.84009	0.99632	FALSE
novel_973	4.88536038	2.710492764	-0.82045	0.32781	0.99632	FALSE
novel_974	0	0	NA	NA	NA	NA
novel_975	3.062391517	4.258837195	0.42229	0.60829	0.99632	FALSE
novel_976	0.380208682	0.320499081	-0.019731	0.99501	0.99939	FALSE
novel_977	0.506318746	0	-1.1604	0.71302	0.99632	FALSE
novel_978	8.627726076	7.664269395	-0.17212	0.77181	0.99632	FALSE
novel_979	0	0.448143166	1.2139	0.70085	0.99632	FALSE
novel_980	0	0	NA	NA	NA	NA
novel_981	0.214063967	0.669182875	1.297	0.60974	0.99632	FALSE
novel_982	22.27961324	18.13467049	-0.27254	0.56128	0.99632	FALSE
novel_983	0.475177909	0.578920558	0.17922	0.92515	0.99939	FALSE
novel_985	0.190104341	0.204429791	0.05166	0.98696	0.99939	FALSE
novel_986	0.380208682	0.953411325	1.2399	0.51647	0.99632	FALSE
novel_987	0.130556971	1.574158234	2.5685	0.10838	0.88062	FALSE
novel_988	0.214063967	0	-0.52541	0.86798	0.99632	FALSE
novel_989	0.506318746	0	-1.1604	0.71302	0.99632	FALSE
novel_990	0.783341826	0	-2.1352	0.49219	0.99632	FALSE
novel_991	0.205489872	1.056618385	2.0274	0.29657	0.99632	FALSE
novel_992	0.775802895	0	-2.07	0.506	0.99632	FALSE
novel_995	0.261113942	0	-1.0116	0.74866	0.99632	FALSE
novel_996	0	0	NA	NA	NA	NA
novel_997	0.391670913	0.43833173	-0.17137	0.94849	0.99939	FALSE
novel_999	0.190104341	0.201378997	0.05166	0.98696	0.99939	FALSE

619
620
621
622
623
624
625
626
627
628
629
630
631
632
633
634
635
636
637
638
639
640
641
642
643
644
645
646
647
648
649
650
651
652
653
654
655
656
