# Supplementary material for: Integrated analyses of miRNA-mRNA expression profiles of ovaries reveal the crucial interaction networks that regulate the prolificacy of goats in the follicular phase
Source: BMC Genomics. 2021 Nov 11;22:812. doi: 10.1186/s12864-021-08156-2 (PMC8582148; doi:10.1186/s12864-021-08156-2)
Supplement: Supplementary file 6 — Additional file 6: Table S6. Primer sequences used for qPCR in this study. [file 12864_2021_8156_MOESM6_ESM.docx]

Table S6 Primer sequences used for qPCR in this study

| Gene name | GenBank accession number | Primer Sequence 5’-3’ | Product size(bp) |
| --- | --- | --- | --- |
| CLK3 | XM_018066137.1 | F: CTCTGCCACGCCCTTAGATT | 251 |
|  |  | R: CCCAGCTCAAGGATCACCTC |  |
| RPS24 | XM_005699249.3 | F: TTCACCCTGGAAAGGCAAC | 139 |
|  |  | R: ATGCCGAAGCCAGTTGTCT |  |
| TFDP2 | XM_018049669.1 | F: CTCAGTCCAACCAAAGGTAACG | 196 |
|  |  | R: TTGCTGGTGCAGGGGTATAT |  |
| PPP1R10 | XM_018038847.1 | F: AGGCACGGAAGATGGTGAGT | 262 |
|  |  | R: TCATCGTCACTCGACTTGCTC |  |
| EXOC7 | XM_018063764.1 | F:GCAGACCAAGCCTGAGTTTG | 118 |
|  |  | R:GAAGTCCTCCAGCGCTTTG |  |
| TXLNA | XM_018057639.1 | F:ATGACCTGAACAAGAGGGTGC | 109 |
|  |  | R:CCCTGTTCCTTAGAGGCATTG |  |
| MS4A1 | XM_005690341.3 | F:TGTGCCAGGATACGGTTTGT | 129 |
|  |  | R:AGCTTTGGGTCTGGAGCATAT |  |
| CD19 | XM_013974717.2 | F:GAGAGATGTGGGTCATGGGC | 157 |
|  |  | R:AGCAGCCAATGCCATACTGAC |  |
| WNT4 | XM_018055379.1 | F:TCAAGCCGCACACGGAT | 232 |
|  |  | R:CGGCATTTGACGAAGCAG |  |
| RPL19 | XM_005693740.3 | F:ATCGCCAATGCCAACTC | 154 |
|  |  | R:CCTTTCGCTTACCTATACC |  |
