# Supplementary figures and images for: Integrated analyses of miRNA-mRNA expression profiles of ovaries reveal the crucial interaction networks that regulate the prolificacy of goats in the follicular phase
Source: BMC Genomics. 2021 Nov 11;22:812. doi: 10.1186/s12864-021-08156-2 (PMC8582148; doi:10.1186/s12864-021-08156-2)

**Fig. S1** The sequence of miRNA-mRNA pair for Dual Luciferase Reporter Assay


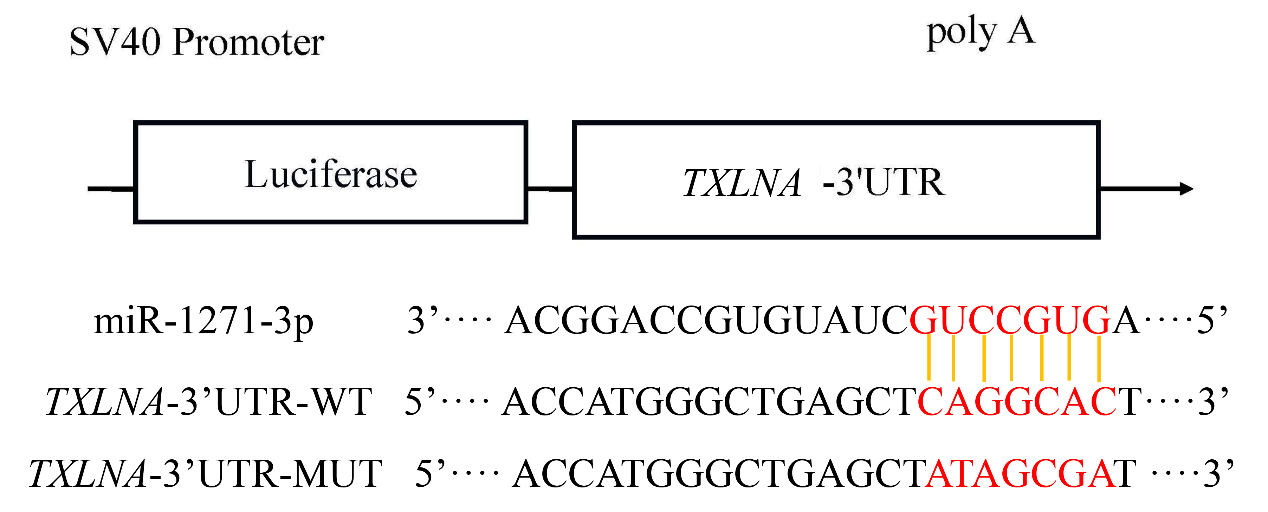

Supplement: Supplementary file 7 — Additional file 7: Fig. S1. The sequence of miRNA-mRNA pair for Dual Luciferase Reporter Assay. [file 12864_2021_8156_MOESM7_ESM.docx]
