# Supplementary material for: Integrated analyses of miRNA-mRNA expression profiles of ovaries reveal the crucial interaction networks that regulate the prolificacy of goats in the follicular phase
Source: BMC Genomics. 2021 Nov 11;22:812. doi: 10.1186/s12864-021-08156-2 (PMC8582148; doi:10.1186/s12864-021-08156-2)
Supplement: Supplementary file 2 — Additional file 2: Table S2. All of the DE mRNAs in the comparison. [file 12864_2021_8156_MOESM2_ESM.pdf]

Table S2 All of the DE mRNAs in the comparison

transcript_id	gene_id	gene_name	feature_id	LF-1 FPK	LF-2 FPK	LF-3 FPK	LF-4 FPK	LF-5 FPK	HF-1 FPK	HF-2 FPK	HF-3 FPK	HF-4 FPK
YP_004111301.1	gene-ATP6	ATP6	transcript	0.802413	0.621465	753.2938	97.24674	975.426	74.44208	0.89022	113.9919	778.214
YP_004111300.1	gene-ATP8	ATP8	transcript	5.684863	6.21269	1277.943	4.600085	1860.443	6.491105	5.672342	10.04517	1158.257
YP_004111298.1	gene-COX1	COX1	transcript	1676.16	1734.204	2297.154	1757.718	2420.13	1287.774	1591.27	1743.172	2198.547
YP_004111299.1	gene-COX2	COX2	transcript	205.3983	198.4553	1572.468	154.9789	1614.696	138.6587	163.4938	175.6672	1602.641
YP_004111302.1	gene-COX3	COX3	transcript	103.4309	99.88882	395.5022	357.7453	493.9335	379.6763	210.7185	128.8071	404.0378
YP_004111308.1	gene-CYTB	CYTB	transcript	153.15	168.8028	645.5519	228.9525	899.6445	214.9428	233.1701	217.3374	564.9015
YP_004111296.1	gene-ND1	ND1	transcript	12.57546	14.97911	146.2373	26.26168	207.8325	23.53729	27.44105	19.89194	187.1597
YP_004111297.1	gene-ND2	ND2	transcript	61.17589	85.00707	106.3312	86.09565	148.3937	93.01727	112.4573	92.64511	101.7735
YP_004111303.1	gene-ND3	ND3	transcript	295.5466	442.5107	490.3342	737.3056	605.5889	1104.047	440.7455	531.538	587.2662
YP_004111305.1	gene-ND4	ND4	transcript	72.47672	75.96249	257.1863	117.3563	434.5182	102.9454	131.3421	109.3482	225.3372
YP_004111304.1	gene-ND4L	ND4L	transcript	109.2442	149.0172	137.1066	201.8767	214.4172	180.7099	215.4482	210.1416	97.93539
YP_004111306.1	gene-ND5	ND5	transcript	28.91768	40.67178	74.77557	57.06173	121.2431	37.83704	63.59434	51.67565	85.69728
YP_004111306.1	gene-ND6	ND6	transcript	8.672583	5.743041	29.96919	11.80258	40.04024	12.28566	12.29625	10.23311	27.13326
NM_001285537.1	gene-LOC100	LOC10086	transcript	0	0	0	0	0	0	0	0	0
NM_001285538.1	gene-ITGB2	ITGB2	transcript	2.128078	9.26758	1.697672	1.281451	2.037577	1.49135	3.127244	1.037494	6.06393
NM_001285539.1	gene-PAEP	PAEP	transcript	0	0	0	0	0	0	0	0	0
NM_001285540.1	gene-TLR6	TLR6	transcript	6.495692	8.594054	3.060127	2.969497	3.930404	4.840994	5.976955	3.857054	3.161706
NM_001285544.1	gene-IL18	IL18	transcript	5.923738	32.43973	3.49338	1.989148	3.201903	3.998083	4.294907	2.965657	7.947046
NM_001285545.1	gene-BAC7.5	BAC7.5	transcript	0.492321	338.3386	0	0	0	0	0	0	0.048975
NM_001285546.1	gene-MAP28	MAP28	transcript	0.118958	199.6134	0.062882	0	0.060226	0.071763	0	0	0.209119
NM_001285548.1	gene-LTF	LTF	transcript	0.232575	42.94202	1.258958	0.108445	0.808415	0.089654	0.200667	0.105829	0.048917
NM_001285550.1	gene-SOD1	SOD1	transcript	273.6609	314.7771	322.6935	315.7375	335.7621	295.3771	272.1781	339.8494	254.5714
NM_001285551.1	gene-LYZL4	LYZL4	transcript	0	0	0	0	0	0	0	0	0
NM_001285553.1	gene-TSC2	TSC2	transcript	0	0	0	0	0	0	0	0	0
NM_001285554.1	gene-PAG-3	PAG-3	transcript	0	0	0	0	0	0	0	0	0
NM_001285555.1	gene-DCN	DCN	transcript	1201.971	36.14849	560.5521	591.1802	587.4352	503.211	749.2079	888.0246	414.2157
NM_001285557.1	gene-EPHX1	EPHX1	transcript	51.49059	15.70953	35.27981	23.2951	37.11502	22.07499	63.35641	39.1962	30.79586
NM_001285559.1	gene-OAZ1	OAZ1	transcript	91.54031	205.3452	115.9725	151.2677	115.3509	123.627	89.81339	90.63767	195.5551
NM_001285560.1	gene-PAG-6	PAG-6	transcript	0	0	0	0	0	0	0	0	0
NM_001285561.1	gene-AANAT	AANAT	transcript	0	0	0	0	0.038471	0	0	0	0
NM_001285563.1	gene-SLC39A	SLC39A3	transcript	0.307424	0.343112	0.000568	0.000162	0.453862	0.000054	0.393249	0.000968	0.000243
NM_001285565.1	gene-PAG-11	PAG-11	transcript	0	0	0	0	0	0	0	0	0
NM_001285566.1	gene-IGFBP5	IGFBP5	transcript	17.34179	0.614698	20.70268	14.3771	16.75396	14.98023	22.45708	22.30002	31.26083
NM_001285567.1	gene-HMOX1	HMOX1	transcript	12.17142	11.93115	22.50648	18.56512	19.20921	22.9433	23.30345	23.14107	49.41316
NM_001285568.1	gene-PAG12	PAG12	transcript	0	0.230247	0.123233	0	0	0	0.021794	0	0
NM_001285569.1	gene-POU5F1	POU5F1	transcript	0.197832	0.117968	0.388988	0.506333	0.309628	0.607888	0.82438	0.136894	0.175507

NM_001285570.1	gene-CSF2	CSF2	transcript	0	0	0	0	0	0	0	0	0
NM_001285571.1	gene-FKBP8	FKBP8	transcript	7.865191	4.969752	0.671947	0.532359	0.703357	0.670086	5.017084	3.466921	0.750974
NM_001285572.1	gene-TNNC1	TNNC1	transcript	0	0	0.064909	0.106141	0	0	0	0	0
NM_001285573.1	gene-LOC100	LOC10086	transcript	1.443079	0.16837	1.773642	1.358868	2.932834	1.59906	2.912717	3.33127	1.233981
NM_001285574.1	gene-TLR4	TLR4	transcript	4.75273	6.072436	1.654681	1.603462	2.32725	1.073972	3.331701	2.370878	10.94358
NM_001285575.1	gene-BMPR1	BMPR1B	transcript	0.33528	0	0.317271	0.168589	0.115813	0.285553	0.168933	0.149863	0.071497
NM_001285576.1	gene-NANOG	NANOG	transcript	0	0	0	0	0	0	0	0	0
NM_001285577.1	gene-BAC5	BAC5	transcript	0.239053	252.0502	0	0	0	0.062053	0	0	0
NM_001285578.1	gene-CD36	CD36	transcript	17.70305	10.73725	14.70769	14.80965	12.62849	19.80523	22.39253	11.45651	158.9229
NM_001285579.1	gene-INSL3	INSL3	transcript	90.15868	0.111899	111.0146	537.1673	252.6454	164.693	112.0949	267.524	13.93459
NM_001285580.1	gene-LOC100	LOC10086	transcript	0	1.809912	0	0	0	0	0	0	0.707342
NM_001285581.1	gene-INHBA	INHBA	transcript	19.57236	0	25.98369	261.0578	100.5873	40.24464	67.69877	128.2282	6.800928
NM_001285582.1	gene-SEPP1	SEPP1	transcript	153.2248	78.66871	129.3254	154.4222	156.6509	173.2998	217.2335	141.4695	405.2943
NM_001285584.1	gene-OVN	OVN	transcript	5.65518	0.025447	123.462	42.48599	48.63064	0.328232	9.120013	0.126603	2.14041
NM_001285585.1	gene-CSN1S2	CSN1S2	transcript	0	0	0	0	0	0	0	0	0
NM_001285586.1	gene-GH1	GH1	transcript	0.144301	0	25.97518	13.36179	20.77146	12.25975	4.618744	1.992433	15.82589
NM_001285587.1	gene-CSN3	CSN3	transcript	0	0	0	0	0	0	0	0	0
NM_001285588.1	gene-BMP15	BMP15	transcript	0.585347	0	0.553274	0.611074	0.843017	1.925508	0.764114	0.470254	0.286329
NM_001285589.1	gene-EIF4EB	EIF4EBP1	transcript	8.63958	3.451582	14.30859	63.36084	21.22968	21.01665	14.37036	25.06329	26.04596
NM_001285591.1	gene-MC4R	MC4R	transcript	0.016193	0	0.034238	0.020357	0.016561	0	0.024221	0.008931	0
NM_001285592.1	gene-BMPR1	BMPR1A	transcript	1.299959	0.542829	0.520038	0.162633	0.080964	0.074868	0.080724	0.136749	0.115892
NM_001285593.1	gene-GFI1B	GFI1B	transcript	0	0	0	0.021595	0	0	0	0	0
NM_001285594.1	gene-RERG	RERG	transcript	0.123014	0.132849	0.026127	0.017471	0.016081	0.018858	0.099902	0.073264	0.018856
NM_001285595.1	gene-PLIN3	PLIN3	transcript	3.479674	1.277329	4.501519	3.69067	3.34986	2.724018	6.421652	3.424238	4.768036
NM_001285596.1	gene-PLIN2	PLIN2	transcript	1.353177	0.071467	0.362166	0.310198	0.618509	0.556918	0.868862	0.644481	0.668794
NM_001285597.1	gene-GABAR	GABARAI	transcript	53.61811	103.184	62.31537	57.17662	54.84601	59.0313	44.42143	52.20532	78.70145
NM_001285598.1	gene-ASMT	ASMT	transcript	0	0	0	0	0	0	0	0	0
NM_001285599.1	gene-RERGL	RERGL	transcript	10.19437	1.343664	3.42144	3.062236	3.435703	1.016933	4.299173	2.159643	0.882364
NM_001285600.1	gene-DIMT1	DIMT1	transcript	17.22065	16.14706	13.88934	12.18363	13.47949	13.28274	11.95457	13.65151	11.3916
NM_001285601.1	gene-MSX2_	MSX2	transcript	0	0	0	0	0	0	0	0	0
NM_001285602.1	gene-MYF6	MYF6	transcript	0.085956	0.107658	0	0.05131	0.049082	0	0.090987	0.154933	0.277875
NM_001285603.1	gene-TLR2	TLR2	transcript	4.791511	20.44775	3.567111	2.81992	3.284759	2.645554	4.015099	3.523293	2.924345
NM_001285604.1	gene-CREM	CREM	transcript	1.403307	1.053665	1.593302	1.045675	1.445823	1.532095	1.453454	1.6014	1.379434
NM_001285605.1	gene-TLR1	TLR1	transcript	2.299391	12.55626	0.992427	1.077491	1.44456	1.437232	3.315967	1.492025	2.956486
NM_001285606.1	gene-INHA	INHA	transcript	94.40282	0	193.5175	1574.868	583.3143	259.9751	232.7438	670.6787	60.06852
NM_001285607.1	gene-LPL	LPL	transcript	0.105468	0.009626	4.041877	1.022257	3.324292	0.014402	0.084974	1.846851	6.734001
NM_001285608.1	gene-CDK2	CDK2	transcript	3.95442	4.824085	2.393647	0.139258	2.247035	2.237083	2.696291	3.90422	1.69899
NM_001285609.1	gene-FTH1	FTH1	transcript	505.1525	499.3174	556.6541	495.3163	881.9111	463.9387	1365.169	550.2274	3429.215

NM_001285610.1	gene-RNASE	RNASEH2	transcript	20.20532	26.92393	0.25564	0.152194	15.12573	0.282435	18.76154	25.95314	0.108921
NM_001285611.1	gene-DPF2	DPF2	transcript	2.83473	0.147292	2.544279	3.37233	0.305139	0.379966	3.901904	3.39841	0.342454
NM_001285612.1	gene-GNRHR	GNRHR	transcript	0.474408	0	0.654738	0.309359	0.842931	0.550321	0.289655	0.30198	0.413779
NM_001285613.1	gene-OGN	OGN	transcript	1.120463	0.313349	0.293079	0.186993	0.291642	0.272806	0.375895	0.432744	0.211667
NM_001285614.1	gene-MMADI	MMADHC	transcript	0.005357	0.004317	0.004089	0.003079	0.005259	0.003448	0.004202	0.002987	0.004121
NM_001285615.1	gene-MGST3	MGST3	transcript	36.2623	30.51115	67.0669	47.09542	50.91761	37.92737	41.05354	48.27261	49.56036
NM_001285616.1	gene-AMELX	AMELX	transcript	0	0	0	0.086053	0	0	0	0	0
NM_001285617.1	gene-LPO	LPO	transcript	0	0	0	0	0	0	0	0	0
NM_001285618.1	gene-BTN1A	BTN1A1	transcript	0	0	0	0	0	0	0	0	0
NM_001285619.1	gene-SCD	SCD	transcript	32.73729	4.924844	90.80895	105.4888	93.47655	62.82235	51.83403	128.051	21.31466
NM_001285620.1	gene-MAP34-	MAP34-B	transcript	0.082787	120.7277	0	0.046256	0.028224	0.099885	0	0	0.032704
NM_001285622.1	gene-PRP1	PRP1	transcript	0	0	0	0	0	0	0	0	0
NM_001285623.1	gene-LOC100	LOC10086	transcript	15.85251	63.90324	16.7586	32.83648	37.15592	31.71496	20.81521	30.2323	76.47045
NM_001285624.1	gene-XDH	XDH	transcript	0	0	0	0	0	0	0	0	0
NM_001285625.1	gene-LGALS	LGALS15	transcript	0	0.241951	0.110242	0	0.104196	0	0	0	0
NM_001285626.1	gene-GLYCA	GLYCAM	transcript	0	0	0	0	0.05209	0	0	0	0
NM_001285627.1	gene-RSPO1	RSPO1	transcript	0.156234	0	0.039542	0.082604	0.155405	0	0.049352	0.024245	0.070484
NM_001285628.1	gene-ELOVL	ELOVL5	transcript	19.08869	20.36929	22.94863	27.25007	21.95998	20.77637	19.93906	25.77039	36.06281
NM_001285629.1	gene-FASN	FASN	transcript	6.145243	6.960394	10.78759	13.85857	11.44007	9.952623	10.84028	13.65513	5.699739
NM_001285630.1	gene-AGP	AGP	transcript	0.477047	1.660145	0.230559	0.313614	0.26343	0.352402	0.337822	0.206531	0.141876
NM_001285631.1	gene-PPARG	PPARGC1	transcript	0.02736	0	0	0	0.030302	0.06147	0.062191	0.025724	0.009156
NM_001285632.1	gene-RPL21	RPL21	transcript	1704.546	1466.271	2048.775	1914.263	2065.505	2382.103	2126.989	2050.049	2276.983
NM_001285634.1	gene-CRSP-1	CRSP-1	transcript	0	0	0	0.170071	0	0	0.075754	0	0
NM_001285635.1	gene-LALBA	LALBA	transcript	0	0.054797	0	0.08874	0	0.056443	0	0.05229	0
NM_001285636.1	gene-FSHR	FSHR	transcript	10.20548	0	18.70598	28.57204	18.50683	27.18873	11.70888	31.83729	10.70608
NM_001285637.1	gene-LOC100	LOC10086	transcript	10.84804	54.43171	15.06525	5.698841	6.604391	10.26185	37.02676	4.430297	680.4884
NM_001285638.1	gene-IL12A	IL12A	transcript	0.113286	0.348282	0.165028	0.220627	0.266327	0.161093	0.172606	0.149236	0
NM_001285639.1	gene-RPS18	RPS18	transcript	1776.986	1697.294	3132.581	3285.797	2984.129	4094.121	2292.125	2130.766	3450.108
NM_001285640.1	gene-IL6	IL6	transcript	0.287041	1.210325	0.14066	0.043012	0.107761	0.064235	0.150838	0.064258	0.072062
NM_001285641.1	gene-RPS6KI	RPS6KB1	transcript	1.139819	2.796849	0.462057	1.120036	1.354165	1.46275	1.767825	1.306261	0.36816
NM_001285642.1	gene-CFLAR	CFLAR	transcript	5.691458	1.56017	1.858303	3.452119	4.941542	4.167945	36.77148	1.992987	2.547865
NM_001285643.1	gene-SDF4	SDF4	transcript	45.91385	29.47416	33.40706	0.729123	0.964719	0.951934	42.55492	45.99296	0.765069
NM_001285644.1	gene-FSHB	FSHB	transcript	0	0	0	0.0465	0.02879	0	0.002335	0.301438	0.01932
NM_001285645.1	gene-MAGEI	MAGED2	transcript	71.41094	28.68232	54.67158	55.25256	55.19159	44.17507	61.96726	78.12166	49.40169
NM_001285646.1	gene-BMP4	BMP4	transcript	10.58773	2.539188	5.535438	4.721171	6.91926	4.430262	7.299908	5.634007	4.607588
NM_001285647.1	gene-PRKAG	PRKAG3	transcript	0	0	1.315447	0.998183	0.008584	0.015965	0.002014	0.00642	1.025198
NM_001285648.1	gene-GHR	GHR	transcript	0.03076	0	0.031329	0.030823	0.014799	0.010879	0.010409	0.005792	0
NM_001285649.1	gene-HNRNP	HNRNPK	transcript	0	0	0	0	0	0	0	0	0

NM_001285650.1	gene-LGALS:	LGALS16	transcript	0.186705	0.660529	0.236864	0.44732	0.618451	0	0.313543	0.177625	0.265521
NM_001285651.1	gene-HMGA2	HMGA2	transcript	0.661788	0	1.664295	0.314604	0	0.465603	0.247398	0.711578	0.049916
NM_001285652.1	gene-RORA	RORA	transcript	19.35262	21.94967	9.996496	8.621626	10.9873	11.64033	16.7698	14.00352	8.831061
NM_001285653.1	gene-FFAR3	FFAR3	transcript	0	0.059327	0.022501	0	0	0	0.004457	0.012186	0.016353
NM_001285654.1	gene-FOXP2	FOXP2	transcript	0.199301	0.068775	0.181476	0.058923	0.086294	0.081478	0.170006	0.065526	0.119879
NM_001285655.1	gene-FFAR2	FFAR2	transcript	0.10771	0.455141	0.069913	0.116408	0.101794	0.040027	0.071292	0.133845	0.078503
NM_001285656.1	gene-RHEB	RHEB	transcript	24.94857	17.00078	21.96746	24.17352	25.4655	21.85989	27.79609	23.26309	25.29566
NM_001285657.1	gene-UBE2K	UBE2K	transcript	10.71355	19.26826	14.49604	16.83349	16.60067	15.45818	15.60775	14.75281	15.2451
NM_001285658.1	gene-PPARG	PPARG	transcript	24.8386	3.900629	35.38634	24.18559	28.60079	44.13649	24.80013	47.9854	18.90502
NM_001285659.1	gene-RAB14	RAB14	transcript	0.148978	0.122989	0.155196	0.217402	0.165906	0.174316	0.312314	0.264452	0.070019
NM_001285660.1	gene-ST6GAI	ST6GAL1	transcript	0.244893	0.176962	0.301392	0.125045	0.307828	0.323299	0.268708	0.380403	0.477987
NM_001285661.1	gene-GHITM	GHITM	transcript	0.167233	0.138163	0.185549	0.147271	0.166271	0.117181	0.11837	0.099119	0.161351
NM_001285662.1	gene-SPARC	SPARC	transcript	0.163021	0.157364	0.236512	0.188932	0.278549	0.318983	0.149147	0.099359	0.263006
NM_001285663.1	gene-CAPN1	CAPN1	transcript	1.517524	2.751515	4.030254	3.690139	2.447648	2.390253	1.689051	1.909622	1.834157
NM_001285664.1	gene-URM1	URM1	transcript	20.5044	28.20213	30.67377	36.41195	29.90725	39.90393	32.08934	26.50862	30.95211
NM_001285666.1	gene-SLC39A	SLC39A1	transcript	22.29981	25.94901	30.06562	30.83384	33.92572	26.95942	30.72187	29.91769	47.73586
NM_001285667.1	gene-SPP1	SPP1	transcript	1.187377	18.35986	6.647999	1.699376	3.657799	2.024887	5.005231	4.070419	48.34482
NM_001285668.1	gene-TNNC2	TNNC2	transcript	0.200993	0.39065	0.099644	0.137856	0.108411	0.358508	0.355067	0.170531	0.239529
NM_001285669.1	gene-PRLR	PRLR	transcript	0.000931	0.045268	0.120885	0.000397	0.184371	0	0.000213	0.106668	0.000219
NM_001285670.1	gene-KITLG	KITLG	transcript	13.01812	3.848423	17.34717	38.01018	17.71273	16.77499	11.35924	26.16239	6.670894
NM_001285671.1	gene-CD9	CD9	transcript	59.88981	73.52928	74.21771	56.47344	76.22273	58.55761	47.32876	50.24012	104.59
NM_001285672.1	gene-SOX2	SOX2	transcript	0.040353	0	0	0.001739	0.023938	0	0.068119	0.012278	0
NM_001285673.1	gene-POU1F1	POU1F1	transcript	0	0	0.189858	0	0	0	0	0	0
NM_001285674.1	gene-NPR2	NPR2	transcript	11.5061	2.823425	4.125532	9.155814	10.39291	3.201211	14.26815	5.237437	3.698121
NM_001285675.1	gene-SOD3	SOD3	transcript	6.688946	8.099548	11.00352	12.41749	14.10893	12.74886	10.39784	10.21594	9.496073
NM_001285676.1	gene-CD81	CD81	transcript	108.6245	78.04316	103.1287	138.6886	121.4641	80.72465	140.2626	112.2898	124.1
NM_001285677.2	gene-BTG1	BTG1	transcript	206.2936	320.5771	169.37	198.8647	176.4993	161.1893	212.2366	248.1459	206.8763
NM_001285678.1	gene-IGFBP4	IGFBP4	transcript	65.53579	28.40423	69.13234	62.5833	82.07111	68.89574	110.5318	63.58133	102.7136
NM_001285679.1	gene-NPPC	NPPC	transcript	0.067103	0	0.099543	0.298674	0.076756	0.025321	0.03521	0.009723	0
NM_001285681.1	gene-IL4	IL4	transcript	0	0.051793	0	0	0.095565	0	0	0	0
NM_001285682.1	gene-IFNG	IFNG	transcript	1.200479	3.088538	0.594003	0.361864	0.854336	0.647024	1.389232	0.991514	0.413245
NM_001285685.1	gene-RHOB	RHOB	transcript	13.86015	8.527325	14.39434	11.25133	16.92628	12.07474	15.7295	12.8029	16.0377
NM_001285686.1	gene-TLR9	TLR9	transcript	0.19401	1.766998	0.449105	0.167302	0.196885	0.408292	0.461028	0.314032	0.328526
NM_001285687.1	gene-CRSP-2	CRSP-2	transcript	0	0	0	0	0	0	0	0	0
NM_001285688.1	gene-ESR2	ESR2	transcript	4.785715	0.149787	2.333004	4.369019	3.684662	4.169614	4.529773	4.003207	2.300484
NM_001285689.1	gene-CHI3L1	CHI3L1	transcript	17.3064	23.14497	11.20072	9.737823	16.34997	3.830444	20.10732	8.907703	56.25335
NM_001285690.1	gene-GNS	GNS	transcript	72.58283	46.46108	38.6352	33.49911	37.9129	30.87347	59.44313	47.04374	92.6422
NM_001285691.1	gene-MANBA	MANBA	transcript	11.94467	16.20174	10.05744	11.3228	10.66699	6.799157	6.678409	11.14414	6.461602

NM_001285692.1	gene-BCNT	BCNT	transcript	74.77923	47.41421	97.1967	98.60007	101.9835	110.2398	89.27808	107.9738	93.7952
NM_001285693.1	gene-P97BCN	P97BCNT	transcript	75.27642	76.35534	72.32698	64.5976	62.06563	68.73286	64.72221	74.64085	53.88346
NM_001285694.1	gene-SLC11A	SLC11A1	transcript	0.288464	1.278241	0.319297	0.141723	0.224666	0.157597	0.231474	0.129235	4.205675
NM_001285695.1	gene-CSN1S1	CSN1S1	transcript	0	0	0	0	0	0	0	0	0
NM_001285696.1	gene-CCNT1	CCNT1	transcript	9.065202	10.72043	9.126024	9.977463	10.66706	9.835896	11.28662	7.999688	6.396352
NM_001285697.1	gene-IGF1	IGF1	transcript	2.104066	0.38151	1.065969	0.744408	1.116512	0.853522	1.777778	1.268793	0.721627
NM_001285698.1	gene-PSMA6	PSMA6	transcript	75.83143	160.3981	83.2652	83.41235	80.04475	100.897	67.68643	84.37068	98.02107
NM_001285699.1	gene-TLR5	TLR5	transcript	0.345815	0.22206	0.439979	0.227069	0.22758	0.100609	0.512084	0.356124	1.030262
NM_001285700.1	gene-IL12B	IL12B	transcript	0.04916	0.868751	0.084518	0	0.108982	0	0.079118	0.028807	0
NM_001285701.1	gene-FABP3	FABP3	transcript	1.654392	18.57374	4.936237	2.320854	4.885111	4.300963	3.404468	1.524618	4.101212
NM_001285702.1	gene-TNFSF1	TNFSF13E	transcript	0.856733	1.564892	0.621801	0	0.700875	0.423755	0.409557	0.358113	2.898269
NM_001285703.1	gene-HSP70.1	HSP70.1	transcript	69.26	30.73763	69.34573	48.8899	115.2386	43.66584	30.48046	39.39097	37.02201
NM_001285704.1	gene-IFNA	IFNA	transcript	0	0	0	0	0	0	0	0.016728	0.026547
NM_001285705.1	gene-ARG1	ARG1	transcript	0	0	0	0	0	0	0	0	0
NM_001285706.1	gene-ASL	ASL	transcript	4.555487	5.790502	1.947789	1.807011	2.630672	1.68684	3.441719	1.990818	3.497652
NM_001285707.1	gene-ABCG2	ABCG2	transcript	24.33993	1.687099	14.06433	1.818353	4.100787	3.086501	10.92148	4.43196	12.03625
NM_001285708.1	gene-GDF9	GDF9	transcript	0.349727	0	0.31012	0	0.581201	0	0.542828	0.509539	0
NM_001285709.1	gene-ANGPT	ANGPTL3	transcript	0	0	0	0	0	0	0	0	0
NM_001285710.2	gene-KISS1	KISS1	transcript	0	0.09085	0.022195	0	0.064416	0	0.142257	0.035253	0
NM_001285711.1	gene-S-LZ	S-LZ	transcript	11.17014	42.83115	13.48732	5.949119	15.85055	20.26216	16.83403	6.276605	126.1882
NM_001285712.1	gene-GPX4	GPX4	transcript	113.156	61.34681	161.1519	241.9756	169.9456	151.1903	125.2481	184.2091	214.2931
NM_001285713.1	gene-RIDA	RIDA	transcript	39.30977	86.83904	46.7217	41.8814	46.6385	50.46849	38.16916	34.59073	81.87666
NM_001285714.1	gene-KRT26	KRT26	transcript	0	0	0	0	0	0	0	0	0
NM_001285715.1	gene-NGF	NGF	transcript	29.19984	0.168206	22.50659	18.68632	22.54701	20.93904	26.53069	19.86004	12.45598
NM_001285716.1	gene-3BHSD	3BHSD	transcript	16.65942	0	18.59079	40.11087	34.26279	10.51399	14.94031	28.57983	7.802258
NM_001285717.1	gene-LOC100	LOC10086	transcript	0	0.034499	0.068519	0.036168	0.044503	0	0.129679	0.024062	0
NM_001285718.1	gene-LDH-A	LDH-A	transcript	5.623229	11.75564	7.193439	2.525971	3.552318	6.846981	3.582467	3.393649	7.358953
NM_001285719.1	gene-KRT27	KRT27	transcript	0.165676	0	0.339339	0.207672	0.265002	0.706677	0.228677	0.302909	0.267846
NM_001285720.1	gene-PAG-2	PAG-2	transcript	0	0	0	0	0	0	0	0	0
NM_001285721.1	gene-CXCL1(	CXCL10	transcript	42.86559	50.19507	44.82579	35.97469	38.38269	46.26104	66.14428	52.18338	27.23732
NM_001285722.1	gene-PAG-8	PAG-8	transcript	0	0.01207	0	0	0	0	0	0	0.06342
NM_001285723.1	gene-CXCR3	CXCR3	transcript	0.478363	4.007278	0.458986	0.274222	0.550605	0.419855	1.046407	0.294695	0.738471
NM_001285724.1	gene-KIT	KIT	transcript	1.034313	0	0.718504	0.746118	0.804458	0.493052	0.560058	0.584176	0.797051
NM_001285725.1	gene-IL17A	IL17A	transcript	0	0.164604	0.028121	0.055224	0	0	0	0	0.058567
NM_001285726.1	gene-SLAMF	SLAMF1	transcript	1.488868	40.89128	0.867302	0.822232	1.433652	0.883938	1.436031	0.682992	1.071929
NM_001285727.1	gene-TYRP1	TYRP1	transcript	0.056013	0	0.015612	0	0	0	0.042032	0.01064	0
NM_001285728.1	gene-HIF1A	HIF1A	transcript	2.955635	2.203664	3.844273	4.625484	3.575666	3.244548	3.805887	5.72733	2.455218
NM_001285729.1	gene-OTC_1	OTC	transcript	0	0	0	0	0	0	0	0	0

NM_001285730.1	gene-ADIPOI	ADIPOR1	transcript	0.53515	0.453756	0.745154	0.443997	0.385878	0.323106	0.230432	0.328479	0.344199
NM_001285731.1	gene-SDHD	SDHD	transcript	62.28691	58.63742	82.78147	62.3724	77.10058	52.93135	49.76644	34.55712	75.52935
NM_001285732.1	gene-PDIA3	PDIA3	transcript	141.8439	233.4492	188.4257	204.8875	208.9425	167.2424	166.4655	174.5963	144.4017
NM_001285733.1	gene-MYOG	MYOG	transcript	0	0	0.053583	0	0	0.047058	0	0	0
NM_001285734.1	gene-COPZ1	COPZ1	transcript	0	0	0	0	0	0	0	0	0
NM_001285735.1	gene-PRP6	PRP6	transcript	0	0	0	0	0	0	0	0	0
NM_001285736.1	gene-TNFSF1	TNFSF13	transcript	5.617244	2.665649	5.543122	2.855898	2.96826	3.418581	6.161945	1.793704	10.10474
NM_001285737.1	gene-MSTN	MSTN	transcript	0.217524	1.222663	0.060006	0.022333	0.041467	0.3249	0.189035	0.213385	0.234635
NM_001285738.1	gene-INSIG2	INSIG2	transcript	7.831761	9.003331	6.743762	7.113916	6.622069	7.57628	7.674082	5.982056	5.573092
NM_001285739.1	gene-PNPLA2	PNPLA2	transcript	2.219978	2.924143	2.627081	2.69404	2.743827	2.491481	3.133901	2.617476	3.316303
NM_001285740.1	gene-HMGCS	HMGCS2	transcript	0.032177	0	0.119121	0.013806	0.046717	0.03975	0.095021	0.102835	0.019522
NM_001285741.1	gene-SLC2A3	SLC2A3	transcript	28.29435	56.17815	17.20253	14.33207	12.87763	12.27919	17.86732	15.40155	23.85592
NM_001285742.1	gene-NCR1	NCR1	transcript	0.703168	15.31544	0.864859	0.613014	3.022012	2.988439	6.200867	1.856382	3.485816
NM_001285743.1	gene-CRISP2	CRISP2	transcript	0	0	0	0	0	0	0	0	0
NM_001285744.1	gene-CRISP1	CRISP1	transcript	0	0	0	0	0	0	0	0	0
NM_001285745.1	gene-CRISP3	CRISP3	transcript	0.866609	1.038097	0.944774	0.030427	0.035223	1.281952	0.302704	0.175452	0
NM_001285746.1	gene-LEF1	LEF1	transcript	0.242329	0.194398	0	0.152365	0	0	0.123198	0.086906	0.135915
NM_001285747.1	gene-CYP19A	CYP19A1	transcript	39.59784	0	33.50481	757.6616	207.3776	30.86581	90.25215	293.7121	9.254629
NM_001285748.1	gene-MTOR	MTOR	transcript	0.106974	3.783062	0.028372	0.05297	0.045501	0.268815	3.1998	0.874093	0.111476
NM_001285749.1	gene-RPSA	RPSA	transcript	902.5759	376.4789	540.8574	565.7999	602.712	1232.331	33.47972	61.7663	510.4898
NM_001285750.1	gene-AKT1	AKT1	transcript	0.000074	0.000023	0.000013	0.000008	0.000014	0.000015	0.00003	0.000035	0.000008
NM_001285751.1	gene-NR1H3	NR1H3	transcript	24.20302	28.37235	20.40972	17.79731	17.17452	16.90268	23.1072	22.58836	25.90681
NM_001285752.1	gene-TCAP	TCAP	transcript	3.735418	1.575368	1.763886	1.948735	2.49588	3.747153	2.67951	2.480277	3.759959
NM_001285753.1	gene-PTHLH	PTHLH	transcript	0.01682	0.231508	0.014057	0.04312	0.073703	0	0	0.555132	0
NM_001285754.1	gene-MYLPF	MYLPF	transcript	0	0	0	0.110554	0.022502	0	0.2013	0	0
NM_001285755.1	gene-SREBF1	SREBF1	transcript	8.389819	6.085626	9.240551	10.64812	10.4144	9.571482	12.26264	11.05707	5.834343
NM_001285756.1	gene-LOC100	LOC10086	transcript	0.169939	0	0.208009	0.03087	0	0.065612	0.01624	0	0.01186
NM_001285757.1	gene-TLR8	TLR8	transcript	0.29819	0.209241	0.063892	0.119585	0.056924	0.061631	0.063668	0.043996	0.068316
NM_001285758.1	gene-IL22	IL22	transcript	0.136239	0	0.031808	0.075563	0.020902	0.086213	0.15895	0.11252	0
NM_001285759.1	gene-CYM	CYM	transcript	1.937138	0	2.819323	1.880395	3.003195	9.425763	1.483708	3.403375	0.533847
NM_001285760.1	gene-SP1	SP1	transcript	2.683819	1.605894	1.486609	1.113707	1.071833	0.903773	1.17905	2.155472	0.907277
NM_001285761.1	gene-AGPAT	AGPAT1	transcript	3.709027	6.8861	3.612488	6.1651	3.788938	4.045413	3.874287	5.609572	4.742327
NM_001285762.1	gene-LOC100	LOC10086	transcript	0	0.041954	0	0	0	0	0	0	0
NM_001285763.1	gene-IGFBP1	IGFBP1	transcript	0.161086	0	0.224203	0.082342	0.209546	0.107064	0.36205	0.788111	0.553844
NM_001285764.1	gene-FST	FST	transcript	7.2271	1.478847	23.04698	61.91864	33.57849	19.767	21.1814	37.23605	5.081929
NM_001285765.1	gene-MMP14	MMP14	transcript	94.61218	19.02221	71.17563	54.78211	67.84895	52.84314	126.9989	68.64368	276.4494
NM_001285766.1	gene-KRT25	KRT25	transcript	0.86354	0	0.821373	0.188545	0.558782	1.780079	0.191905	0.892764	0.197787
NM_001285767.1	gene-KRTAP	KRTAP11-	transcript	0	0.031877	0	0	0	0	0	0	0

NM_001285768.1	gene-LOC100	LOC10086	transcript	0	0	0	0	0	0	0	0	0
NM_001285769.1	gene-ETS1	ETS1	transcript	3.824327	23.4697	3.713486	2.656087	2.811809	3.09726	4.376055	3.249822	3.260764
NM_001285770.1	gene-LOC100	LOC10086	transcript	0	0	0	0	0	0	0	0	0
NM_001285771.1	gene-KRTAP	KRTAP15-	transcript	0	0	0	0	0	0	0	0	0
NM_001285772.1	gene-SLC39A	SLC39A2	transcript	0	0	0.114908	0.048975	0	0	0.022523	0	0
NM_001285773.1	gene-LOC100	LOC10086	transcript	0	0	0	0	0	0	0	0	0
NM_001285774.1	gene-KRTAP	KRTAP3-1	transcript	0	0	0	0	0	0	0	0	0
NM_001285775.1	gene-B4GAL	B4GALT1	transcript	16.97612	23.12477	15.59551	19.00309	19.49537	14.08683	25.95026	18.43615	25.97187
NM_001286087.1	gene-RPS10	RPS10	transcript	874.9136	821.673	1200.864	1202.191	1100.978	1420.607	854.504	794.1808	1501.696
NM_001286088.1	gene-INSIG1	INSIG1	transcript	6.736271	5.403746	13.96043	18.33187	15.60534	9.850851	9.290832	11.68461	13.02887
NM_001286089.1	gene-CASP3	CASP3	transcript	0.114166	0.099428	0.201041	0.186318	0.237635	0.17448	0.096949	0.061116	0.162852
NM_001286090.1	gene-LOC102	LOC10217	transcript	0.656757	0.166546	0.162923	0	1.043352	0	1.21925	0	0
NM_001286091.1	gene-RAB7A	RAB7A	transcript	0.334626	0.477731	0.439276	2.135324	1.388757	2.764379	0.515902	1.123347	0.978264
NM_001286092.1	gene-CASR	CASR	transcript	0	0	0	0	0	0	0	0	0
NM_001286442.1	gene-TNF	TNF	transcript	0	3.803468	0	0.531706	0.310992	0.019041	0.513053	0.178187	0.547573
NM_001286443.1	gene-RHOA	RHOA	transcript	37.42882	52.719	38.95319	46.20569	52.78121	39.20202	55.92201	54.14173	41.5387
NM_001286962.1	gene-SERF2	SERF2	transcript	409.7638	717.1176	927.6151	959.3871	974.2956	1088.611	712.8496	700.0798	1292.736
NM_001286965.1	gene-RAB1A	RAB1A	transcript	32.42955	41.12334	32.66019	36.71528	33.91734	35.23839	31.33535	30.32496	47.33793
NM_001286975.1	gene-SPACA	SPACA3	transcript	0	0	0.426035	0	0.191809	0	0.041716	0	0
NM_001286976.1	gene-SOLD1	SOLD1	transcript	0	0	0	0	0	0	0	0	0
NM_001286991.1	gene-SLC39A	SLC39A13	transcript	14.12807	10.39802	15.34282	20.85024	16.48794	14.66653	13.6275	15.633	18.90547
NM_001287026.1	gene-KANSL	KANSL2	transcript	0.985236	0.420885	0.2886	0.42508	0.341003	0.209173	0.525618	0.679273	0.147769
NM_001287027.1	gene-KANSL	KANSL2	transcript	0	0	0	0	0	0	0	0	0
NM_001287029.1	gene-TMSB4	TMSB4X	transcript	139.3735	326.0949	225.2358	240.6595	293.8647	255.2832	267.684	147.5497	481.9944
NM_001287034.1	gene-VEGFA	VEGFA	transcript	0.929814	0.61913	0.242958	0.319213	0.824593	0.004243	0.829549	1.058645	0.054462
NM_001287035.1	gene-VEGFA	VEGFA	transcript	2.71411	2.678057	3.622785	3.774593	3.773615	5.113754	3.622593	3.907358	2.420725
NM_001287037.1	gene-MYF5	MYF5	transcript	0	0	0	0	0.065876	0	0.025283	0.015318	0
NM_001287041.1	gene-IGF2	IGF2	transcript	15.09921	2.392374	21.3116	13.66621	29.2388	17.84901	38.17015	22.46516	25.68112
NM_001287042.1	gene-IGF2	IGF2	transcript	0.527655	0.100914	1.540266	0.683832	0.560416	1.18646	2.922313	2.347373	1.204127
NM_001287178.1	gene-ELOVL	ELOVL1	transcript	8.866171	6.386056	0.165823	0.120197	0.163572	0.153089	0.114264	0.125389	0.210193
NM_001287231.1	gene-DDIT3	DDIT3	transcript	0.846287	1.49669	0.357716	0.063812	0.022503	0.177113	0.062872	0.044021	0.188154
NM_001287233.1	gene-RAP1B	RAP1B	transcript	21.15784	47.40101	16.77047	15.95838	16.78195	17.07331	16.94477	20.28449	21.85324
NM_001287234.1	gene-CYB5A	CYB5A	transcript	37.25929	16.86597	29.46218	41.66848	40.1075	26.88786	40.87172	33.34784	25.68925
NM_001287235.1	gene-IZUMO	IZUMO1	transcript	0.273324	0.080455	0.267676	0.148523	0.091044	0.094699	0.120492	0.144068	0.271661
NM_001287236.1	gene-NPPA	NPPA	transcript	0	0	0	0	0	0	0	0	0.067138
NM_001287555.1	gene-SST	SST	transcript	0	0	0	0	0	0	0	0	0
NM_001287558.1	gene-FABP1	FABP1	transcript	0	0	0	0	0	0	0	0	0
NM_001287560.1	gene-RAB18	RAB18	transcript	82.20992	66.97018	71.64082	63.43501	70.88341	62.00585	60.90642	57.69704	70.88994

NM_001287562.1	gene-TYR	TYR	transcript	0	0	0	0	0.016138	0	0	0	0
NM_001287564.1	gene-BMP2	BMP2	transcript	2.372305	0.573418	1.850708	1.377248	1.676796	0.918594	2.356851	2.362631	2.41307
NM_001287566.1	gene-LOC102	LOC10217	transcript	0.109777	0	0.073553	0.265116	2.335967	2.556049	1.733659	0.547058	1.120214
NM_001287567.1	gene-IL2	IL2	transcript	0	0.722283	0.075243	0.265438	0	0	0	0.256038	0.124833
NM_001287568.1	gene-MYOZ2	MYOZ2	transcript	0	0	0	0	0	0.018944	0	0	0
NM_001287569.1	gene-RAB8A	RAB8A	transcript	8.96543	20.63777	15.82851	14.25852	14.04635	15.08024	15.8706	16.39001	17.08672
NM_001287570.1	gene-LOC102	LOC10219	transcript	0	0	0	0	0	0	0	0	0
NM_001287571.1	gene-HSPA5	HSPA5	transcript	200.5134	590.6668	370.5278	287.2202	297.836	253.7693	172.0385	293.0194	209.3028
NM_001287572.1	gene-TAC3	TAC3	transcript	0.726406	0.103226	1.790033	0.907204	4.929467	0.45156	1.766806	2.646242	0.704731
NM_001287573.1	gene-ADIPOQ	ADIPOQ	transcript	0.019176	0.179775	0	0	0	0	0.046363	0.070766	0.092929
NM_001287574.1	gene-LOC102	LOC10217	transcript	60.93886	5.364942	83.4921	292.9313	134.1948	92.80317	94.80171	185.7091	75.9585
NM_001287575.1	gene-FABP2	FABP2	transcript	0	0.027649	0	0	0	0	0.01111	0.013279	0
NM_001287576.1	gene-TSHB	TSHB	transcript	0	0	0	0.194362	0	0	0.118082	0.089178	0.094662
NM_001288818.1	gene-ETV5	ETV5	transcript	1.329601	1.861571	1.119653	1.356939	1.483663	0.802918	2.344543	1.640684	0.790956
NM_001289106.1	gene-MAN2A	MAN2A1	transcript	34.1125	62.64128	32.37523	43.83227	38.30606	30.74707	35.79582	41.39591	40.3971
NM_001289948.1	gene-BTG3	BTG3	transcript	62.82391	29.62675	36.11736	46.19063	47.26995	36.67122	42.39587	51.28365	29.89221
NM_001289949.1	gene-MYOZ3	MYOZ3	transcript	0.158083	0	0.107802	0.027229	0.032044	0	0.045353	0	0
NM_001290107.1	gene-ACE2	ACE2	transcript	0.519589	0.568068	0	0.710629	0.952344	0.55562	0.314804	0	0.517161
NM_001290120.1	gene-OAZ2	OAZ2	transcript	59.41019	23.25184	88.23463	84.14883	77.05235	77.80612	63.86282	51.39443	71.79549
NM_001290529.1	gene-OAZ3	OAZ3	transcript	0	0.270478	0.267307	0	0	0.448827	0.20707	0	0
NM_001291297.1	gene-PEG10	PEG10	transcript	2.4776	0.002838	4.93234	4.636594	3.152859	2.812651	4.241885	2.19832	2.439234
NM_001291298.1	gene-PEG10	PEG10	transcript	2.55826	0.94404	4.260934	5.37069	2.881315	2.592324	3.752321	2.260319	2.121751
NM_001291299.1	gene-PEG10	PEG10	transcript	6.555377	0.532403	26.98841	26.64749	26.8511	29.45713	10.66138	13.36216	14.2378
NM_001291300.1	gene-PEG10	PEG10	transcript	7.494209	0.830971	0.09828	2.55679	2.860425	1.119532	10.39256	15.47228	0.038001
NM_001291697.1	gene-AZIN1	AZIN1	transcript	1.619093	3.674578	1.493345	3.013904	2.093458	1.949552	2.312257	2.340147	0.908555
NM_001291698.1	gene-AZIN1	AZIN1	transcript	30.88343	44.09432	33.94603	58.76653	40.33398	34.1569	31.31411	37.78505	30.72003
NM_001291699.1	gene-AZIN1	AZIN1	transcript	4.328738	6.840873	5.496311	8.712558	5.635544	7.179099	5.236264	5.292428	5.445279
NM_001291820.1	gene-FASLG	FASLG	transcript	1.59491	9.775611	1.40119	0.586869	0.985895	3.065865	1.737062	0.518226	1.78459
NM_001291973.1	gene-FGF5	FGF5	transcript	0	0	0.019635	0	0	0	0	0	0
NM_001293062.1	gene-DEFB12	DEFB126	transcript	0	0	0	0	0	0	0	0	0
NM_001293756.1	gene-AZIN2	AZIN2	transcript	0	0	0	0.097439	0	0	0	0	0
NM_001301483.1	gene-OAZ3	OAZ3	transcript	0	0	0	0	0	0	0	0	0
NM_001303466.1	gene-RBP5	RBP5	transcript	0	0	0	0	0	0	0	0	0
NM_001312693.1	gene-RARRE	RARRES2	transcript	495.6874	65.89603	504.8773	397.1257	461.8413	518.6291	353.0896	598.8862	425.7126
NM_001314142.1	gene-TGFB1	TGFB1	transcript	2.187351	6.046287	1.791087	1.191635	3.560311	2.079424	6.28679	2.715323	4.519297
NM_001314145.1	gene-LOC102	LOC10218	transcript	16.24226	0	38.65294	85.72697	88.61977	36.86182	49.82511	76.12372	6.219412
NM_001314147.1	gene-UCP2	UCP2	transcript	0.6697	0.635827	2.312029	4.748831	0.742873	1.573675	10.9342	4.402887	12.62469
NM_001314156.1	gene-ITGB1	ITGB1	transcript	4.875053	0.085611	0.150285	0.121025	0.13445	0.105995	0.075198	0.063773	0.162287

NM_001314158.1	gene-ENO2	ENO2	transcript	0.368217	0.237927	0.3418	0.237204	0.303268	0.478902	0.300774	0.180396	0.315103
NM_001314163.1	gene-CTSH	CTSH	transcript	78.75886	127.9565	68.02062	20.47001	46.97232	48.7131	135.2417	67.25668	708.0703
NM_001314169.1	gene-ACVR2	ACVR2B	transcript	0.266077	0.350622	1.294689	0.209738	0.181245	0.110458	0.086508	0.087703	0.082313
NM_001314172.1	gene-BPI	BPI	transcript	0.737427	1.935351	0.556059	0	0	0	0.751595	0	1.000456
NM_001314175.1	gene-APAF1	APAF1	transcript	6.302666	2.402173	0.391105	0.207062	0.267326	0.499267	4.259696	1.690626	0.32729
NM_001314177.1	gene-LIN28A	LIN28A	transcript	0	0	0	0.332331	0.054075	0.309165	0.131012	0.037435	0
NM_001314180.1	gene-ST3GAI	ST3GAL5	transcript	11.27076	19.42716	9.04562	11.81654	12.12184	8.948024	15.32396	14.55135	18.99862
NM_001314182.1	gene-RAB2A	RAB2A	transcript	50.47397	44.45374	49.87216	43.70255	44.20006	42.31158	45.53627	51.51122	40.74207
NM_001314184.1	gene-ITGAV	ITGAV	transcript	4.730237	1.845864	2.033209	1.772557	1.935518	1.462618	3.707754	2.885307	3.104201
NM_001314186.1	gene-MPO	MPO	transcript	0	11.00612	0	0	0	0	0	0	0
NM_001314188.1	gene-LOC102	LOC10218	transcript	244.0324	1413.749	155.0299	89.48273	121.0871	61.56706	189.7629	140.7547	514.0569
NM_001314190.1	gene-TNNT2	TNNT2	transcript	0	0	0	0	0	0	0	0	0
NM_001314194.1	gene-SLC50A	SLC50A1	transcript	1.516369	5.367969	1.458585	2.280531	1.980357	1.830475	2.391017	3.064788	3.10792
NM_001314200.1	gene-MEF2D	MEF2D	transcript	0	0	0	0	0	0	0	0	0
NM_001314202.1	gene-MAPK1	MAPK1	transcript	14.04583	13.96451	9.14975	5.962249	8.477793	8.574861	13.51901	12.22493	0.294632
NM_001314204.1	gene-MEF2C	MEF2C	transcript	12.73207	15.27967	4.680456	3.188467	4.784502	2.770014	5.731953	5.633638	3.920388
NM_001314206.1	gene-LOC102	LOC10217	transcript	3.367648	2.003752	2.613755	2.219582	2.600684	2.306244	2.695603	2.349438	3.508178
NM_001314207.1	gene-ARC	ARC	transcript	0.096018	0.013443	0.267973	0.022423	0.120612	0.053538	0.18669	0.381361	0.400155
NM_001314210.1	gene-TNNT3	TNNT3	transcript	0.458714	0.392771	1.019088	1.004907	0.640532	0.397933	1.203546	0.388422	1.009804
NM_001314212.1	gene-DLK1	DLK1	transcript	0	0	0	0	0	0	0	0	0
NM_001314213.1	gene-BCL2	BCL2	transcript	2.064155	0.039002	1.505698	1.164593	2.114672	0.05825	2.464396	1.257994	1.713764
NM_001314215.1	gene-SLC2A1	SLC2A12	transcript	4.18844	0.531009	2.988728	1.612583	3.023103	1.757445	3.758444	2.312159	1.141163
NM_001314219.1	gene-IGFBP3	IGFBP3	transcript	73.52091	94.91932	68.28405	100.1913	87.27868	43.29158	120.0859	84.03162	21.91844
NM_001314221.1	gene-CIDEA	CIDEA	transcript	4.14467	1.816183	0.601168	0.206327	0.559744	0.110245	0.240821	0.620198	0.34409
NM_001314223.1	gene-SLC2A1	SLC2A1	transcript	9.86038	1.860698	6.549024	0.125332	5.592366	3.202308	7.999805	10.38453	2.786691
NM_001314225.1	gene-EEF1D	EEF1D	transcript	339.9601	188.807	524.0003	491.4113	519.158	525.0589	404.3149	468.0601	523.0444
NM_001314227.1	gene-SLC2A4	SLC2A4	transcript	1.017163	0.169306	0.153433	0.52095	0.31738	0.268602	0.565703	0.495478	0.070709
NM_001314229.1	gene-RBP4	RBP4	transcript	0	0	0	0	0	0	0	0	0
NM_001314231.1	gene-MEF2A	MEF2A	transcript	0.385062	0	0	0.245124	0.377985	0	0.279639	0.492761	0
NM_001314233.1	gene-HSPA8	HSPA8	transcript	945.1201	1831.273	1366.457	1089.454	914.7161	1045.364	517.5485	1302.498	836.0559
NM_001314235.1	gene-FAS	FAS	transcript	0.782728	5.366687	1.321026	0.384109	0.765851	1.671641	0.920567	0.751092	0.211169
NM_001314237.1	gene-OPRK1	OPRK1	transcript	0.245339	0.01281	0.170055	0.050607	0.265914	0.049075	0	0	0.027476
NM_001314239.1	gene-TNNI3	TNNI3	transcript	13.50055	0.119397	39.57359	84.9809	53.81048	42.16204	24.99857	69.34787	16.55394
NM_001314241.1	gene-DAZL	DAZL	transcript	0.043881	0.015616	0	0.16457	0.201284	0	0.080834	0.087427	0.100518
NM_001314243.1	gene-IGFBP6	IGFBP6	transcript	289.8046	16.2681	424.8118	373.7499	413.929	274.4931	292.7973	327.5472	157.7954
NM_001314245.1	gene-CTSB	CTSB	transcript	0.309824	0.302619	0.253252	0.353797	0.427533	0.454059	0.277305	0.137051	0.755744
NM_001314247.1	gene-PRNP	PRNP	transcript	0.08348	0.104724	0.164374	0.117758	0.143421	0.175671	0.120772	0.099896	0.148857
NM_001314253.1	gene-CMPK1	CMPK1	transcript	26.70769	50.11196	34.96654	34.26821	34.1662	39.62573	24.2022	32.41694	35.49974

NM_001314255.1	gene-PTGER2	PTGER2	transcript	1.335187	3.490889	1.417855	1.969035	2.939565	1.357484	1.798718	2.442121	0.327381
NM_001314257.1	gene-ELOVL6	ELOVL6	transcript	1.755752	3.229791	6.187785	5.390611	6.45251	3.489551	2.852606	8.550227	1.121786
NM_001314259.1	gene-PITX2	PITX2	transcript	0	0.071913	0.037506	0	0	0	0	0	0
NM_001314262.1	gene-B4GALI	B4GALNT	transcript	0	0	0	0	0	0	0	0	0.039892
NM_001314264.1	gene-GPRC5I	GPRC5D	transcript	0	0.064491	0	0	0	0	0	0	0
NM_001314266.1	gene-CTSV	CTSV	transcript	86.73007	14.60551	71.58942	41.22721	65.62463	22.14726	67.41219	75.36493	48.54454
NM_001314268.1	gene-TRAM1	TRAM1	transcript	3.829633	19.99541	4.60155	4.017318	4.16581	4.090439	5.449471	6.461174	4.189744
NM_001314269.1	gene-MMP9	MMP9	transcript	0.784896	4.186886	6.448752	2.711865	1.975956	1.909384	0.883209	3.439847	5.668869
NM_001314271.1	gene-NANOG	NANOG	transcript	0.064177	0	0	0	0	0	0	0	0
NM_001314273.1	gene-ZBTB16	ZBTB16	transcript	0	0	0.15928	0	0	0	0	0.129247	0.228041
NM_001314275.1	gene-TMEM6	TMEM63A	transcript	0.46022	0.205386	0.38368	0.235537	0.129996	0.122673	0.211348	0.314494	0.210673
NM_001314278.1	gene-STAT3	STAT3	transcript	1.44615	0.302349	1.048648	0.277679	0.409375	0.438083	0.390426	0.189415	0.357601
NM_001314279.1	gene-LHCGR	LHCGR	transcript	13.13616	0	8.019125	93.62207	40.68008	13.02385	16.16752	55.49131	5.014236
NM_001314281.1	gene-LTBR	LTBR	transcript	6.395611	2.742991	7.409709	9.108596	8.222619	7.527523	10.01969	7.989703	9.163361
NM_001314283.1	gene-TSC1	TSC1	transcript	0.599416	1.111241	0.18212	1.19803	0.198252	1.438655	0.255685	0.869846	0.93719
NM_001314285.1	gene-ELF5	ELF5	transcript	0	0	0	0	0	0	0	0	0
NM_001314287.1	gene-AKR1B	AKR1B1	transcript	107.6901	34.38086	124.3175	100.4809	112.1768	121.9661	79.43472	134.9874	69.93591
NM_001314289.1	gene-IDO1	IDO1	transcript	1.187102	45.31844	0.661219	0.504753	0.637727	1.170302	1.903831	0.536175	3.562922
NM_001314291.1	gene-NOD2	NOD2	transcript	0.277869	1.583817	0.226383	0.098911	0.230117	0.117069	0.231468	0.223718	0.913793
NM_001314293.1	gene-DSG4	DSG4	transcript	0	0.010241	0	0	0	0	0	0	0
NM_001314295.1	gene-LHX2	LHX2	transcript	0.027188	0.022029	0	0.021981	0.055802	0.082174	0.014107	0	0
NM_001314297.1	gene-ARHGA	ARHGAP3	transcript	0.013275	0	0	0.176621	0.06384	0.020725	0.028791	0.068421	0.049591
NM_001314299.1	gene-IGFBP2	IGFBP2	transcript	55.71856	8.800615	134.1571	221.743	193.8522	167.2242	65.19997	140.6681	99.07159
NM_001314301.1	gene-SERPIN	SERPINA1	transcript	6.34104	0	2.062809	3.348242	1.68338	4.056145	3.054772	4.064822	2.485996
NM_001314303.1	gene-HSPA2	HSPA2	transcript	13.21634	7.023954	11.89879	10.55209	14.85536	8.417504	14.05756	14.16265	6.505083
NM_001314305.1	gene-DGAT2	DGAT2	transcript	0.386507	0.0203	0.27547	0.399086	0.509039	0.328359	0.34275	0.407322	0.134253
NM_001314307.1	gene-ADIPOI	ADIPOR2	transcript	0	0	0	0	0	0	0	0	0
NM_001314309.1	gene-TMEM1	TMEM147	transcript	76.31429	85.70618	115.9673	129.608	116.9784	104.8317	103.4081	113.8284	133.2735
NM_001314312.1	gene-MFGE8	MFGE8	transcript	0.102115	0.062668	1.930653	1.123388	0.21424	0.069538	0.206486	0.242731	0.002366
NM_001314314.1	gene-EEF1B2	EEF1B2	transcript	1188.329	769.6435	1818.574	1591.239	1776.854	2061.345	1111.446	1240.784	1896.606
NM_001314316.1	gene-ITGAL	ITGAL	transcript	1.909126	5.707397	1.572384	1.143265	1.149571	1.235936	1.755704	1.01401	1.554694
NM_001314319.1	gene-SIRT1	SIRT1	transcript	11.84897	10.43251	8.395094	9.031271	8.216406	6.007157	10.51562	13.25533	7.359196
NM_001314321.1	gene-LOC102	LOC10217	transcript	322.4404	0	619.1844	1699.068	1202.347	307.8586	308.4339	1459.087	118.3925
NM_001314323.1	gene-SLC5A1	SLC5A1	transcript	0	0.007393	0	0	0.004781	0	0.056499	0.023897	0
NM_001314325.1	gene-FOXM1	FOXM1	transcript	0.145194	1.107149	0.527138	0.498188	0.643478	0.537968	0.46678	0.669583	0.101071
NM_001314327.1	gene-NFE2L2	NFE2L2	transcript	94.55306	17.48315	61.27611	46.08333	61.36155	52.38876	73.58094	64.80516	58.1142
NM_001314329.1	gene-TNFSF1	TNFSF14	transcript	0.454158	3.265887	0.153618	0.432505	0.920396	0.757846	1.191006	0.627342	0.839063
NM_001314331.1	gene-RPLP0	RPLP0	transcript	1628.693	1562.873	1472.938	1465.745	1667.858	1496.881	1337.526	1713.448	1605.174

NM_001314333.1	gene-ODF1	ODF1	transcript	0	0.13218	0	0	0	0	0	0.018919	0
NM_001314338.1	gene-NEURO	NEUROG3	transcript	0	0	0	0	0	0	0	0	0
NM_001314340.1	gene-MAN2B	MAN2B1	transcript	63.5664	157.0571	39.11965	37.51253	40.87412	30.176	65.41963	41.21548	93.96345
NM_001314342.1	gene-ACTB	ACTB	transcript	1.430615	1.524312	1.124908	1.153852	1.157101	0.982272	1.112898	1.183311	1.076774
NM_001314344.1	gene-HSF1	HSF1	transcript	1.838748	1.801401	1.137715	0.731668	0.71027	0.785278	1.120399	1.823609	0.79958
NM_001314346.1	gene-PID1	PID1	transcript	6.83002	0.797559	6.919216	5.521673	7.226561	4.130846	11.53451	3.868078	3.82979
NM_001314348.1	gene-ITGB6	ITGB6	transcript	0	0	0	0	0	0	0	0	0
NM_001314350.1	gene-TIMP1	TIMP1	transcript	1.627839	0.772053	0.656169	0.512462	0.880779	0.597025	0.545499	0.460152	0.659552
NM_001314352.1	gene-CTSF	CTSF	transcript	37.90041	6.749058	37.04541	32.68158	39.70341	25.97247	52.46547	30.07608	54.91325
NM_001319276.1	gene-FTO	FTO	transcript	30.4145	12.06171	29.08769	23.48726	30.66006	30.22113	35.86115	30.40541	23.64543
NM_001319277.1	gene-RPRD1I	RPRD1B	transcript	13.89896	9.688259	8.295414	8.74917	9.254848	7.746161	11.00571	12.1518	6.508684
NM_001319278.1	gene-TMEM1	TMEM173	transcript	1.488164	5.515429	0.661556	0	1.257243	0.471827	2.616479	0.865147	2.89925
Novel_000001	XLOC_00001	.	transcript	0.042323	0.014482	0.178381	0.130067	0.049617	0.121328	0.150668	0.096564	0.154889
Novel_000002	XLOC_00001	.	transcript	0	0	0.214583	0.020178	0.017953	0.04846	0.369631	0	0
Novel_000003	XLOC_00001	.	transcript	0.98421	0.247357	0.593858	0.910047	0.833576	0.754772	0.590523	0.935526	0.950867
Novel_000004	XLOC_00001	.	transcript	0	0	0	0	0	0	0	0	0
Novel_000005	XLOC_00001	.	transcript	0	0	0	0	0	0	0.487302	0	0
Novel_000006	XLOC_00001	.	transcript	2.868773	4.160198	2.733676	2.565232	2.661464	2.830675	3.292042	3.431956	2.401612
Novel_000007	XLOC_00002	.	transcript	0.890475	0.696702	0.735598	0.894541	0.670491	0.432384	0.948999	1.497827	0.83767
Novel_000008	XLOC_00002	.	transcript	0.224648	0.251675	0.149553	0.333627	0.354552	0.378742	0	0.117263	0.277668
Novel_000009	XLOC_00002	.	transcript	0.238506	0.914819	0.59845	0.427527	0	0.434865	1.174091	0	1.075361
Novel_000010	XLOC_00002	.	transcript	0	0	0.688129	0.245455	0.461581	0.254007	0.23124	0.516003	0.864431
Novel_000011	XLOC_00003	.	transcript	0	0	0.026758	0.021213	0	0	0	0.014491	0.068082
Novel_000012	XLOC_00004	.	transcript	0.10466	0.349754	0.230627	0.425439	0.207891	0.195339	0.237155	0.268685	0.472374
Novel_000013	XLOC_00004	.	transcript	0	0.291022	0.095851	0.091427	0	0	0	0	0
Novel_000014	XLOC_00004	.	transcript	0	0.267421	0	0	0.014071	0	0	0	0
Novel_000015	XLOC_00004	.	transcript	0.26301	0.331175	0.533936	0.3043	0.511756	0.467491	0.453394	0.343566	0.459222
Novel_000016	XLOC_00004	.	transcript	0	0.220726	0	0	0	0	0	0	0
Novel_000017	XLOC_00011	.	transcript	0.162685	0.01991	0.218327	0.132369	0.198517	0.108302	0.175359	0.256898	0.028855
Novel_000018	XLOC_00016	.	transcript	0.216708	0.338954	0.097388	0.18816	0.134185	0.208352	0.299549	0.204775	0.277969
Novel_000019	XLOC_00018	.	transcript	1.05687	1.197663	0.939332	1.054458	0.794442	0.568738	0.954876	1.10232	0.731824
Novel_000020	XLOC_00020	.	transcript	0.999268	1.401373	0.415918	0.667938	0.647466	0.878076	0.851434	1.27808	0.845125
Novel_000021	XLOC_00025	.	transcript	0.232266	0.120752	0.121281	0.108391	0.128147	0.093419	0.18578	0.116516	0.182572
Novel_000022	XLOC_00030	.	transcript	0.019101	0.196921	0	0	0	0	0	0	0
Novel_000023	XLOC_00030	.	transcript	0.576718	0.738175	0.344762	0.624753	0.68254	0.415484	0.423083	0.67675	0.470295
Novel_000024	XLOC_00030	.	transcript	0.471794	0	0.489672	0.279797	0.463268	0.011005	0.088298	0.351424	0.138827
Novel_000025	XLOC_00030	.	transcript	0	0	0	0	0	0	0	0.030995	0.084365
Novel_000026	XLOC_00030	.	transcript	0	0	0	0	0	0	0.193952	0	0

Novel_000027	XLOC_0003C.	transcript	0	0.30991	0.001734	0	0.002792	0.364919	0.164721	0.059878	0
Novel_000028	XLOC_0003C.	transcript	0	0	0	0	0.002656	0	0	0	0
Novel_000029	XLOC_00042.	transcript	0	0	0	0	0	0	0.130395	0.071146	0
Novel_000030	XLOC_00042.	transcript	0	0.003583	0.220808	0.297974	0.138345	0.102286	0.156138	0.042784	0.001601
Novel_000031	XLOC_00042.	transcript	0	0	0	0	0	0	0	0	0
Novel_000032	XLOC_00042.	transcript	0.278036	0	0.239483	0	0.601828	0	0	0	0
Novel_000033	XLOC_00042.	transcript	0	0	0	0	0.286843	0	0	0	0
Novel_000034	XLOC_00042.	transcript	0.086363	0	0.151186	0.117797	0	0.050153	0.0237	0.007761	0
Novel_000035	XLOC_00042.	transcript	0.541499	0	0.450813	0.488145	0.771603	0.940145	0.955535	0.864645	0.435592
Novel_000036	XLOC_00042.	transcript	0.174245	0	0.023653	0.080643	0.067248	0.107235	0.149356	0.030667	0.116423
Novel_000037	XLOC_00042.	transcript	0.001844	0	0.002101	0	0.002249	0.216212	0.00111	0.00342	0
Novel_000038	XLOC_00052.	transcript	0	0	0	0	0.139662	0.228344	0.300556	0.164416	0
Novel_000039	XLOC_00052.	transcript	0	0	0	0	0	0.131716	0	0	0
Novel_000040	XLOC_00052.	transcript	0.302116	0	0	0	0.129742	0.091729	0	0.348655	0
Novel_000041	XLOC_00052.	transcript	0	0	0	0	0	0	0	0	0
Novel_000042	XLOC_00052.	transcript	0.380904	0	0	0.37967	0.111157	0.088106	0.216736	0.106634	0
Novel_000043	XLOC_00052.	transcript	0.116263	0	0.141514	0.143004	0.08037	0.077524	0.074807	0.026767	0.062779
Novel_000044	XLOC_00052.	transcript	0.312894	0.013501	0.381339	0.265144	0.306614	0.249071	0.200845	0.170096	0.107101
Novel_000045	XLOC_00063.	transcript	0	0.093513	0	0	0.117682	0.04114	0.104725	0.294339	0.08711
Novel_000046	XLOC_00063.	transcript	0	0	0	0	0	0	0	0	0
Novel_000047	XLOC_00063.	transcript	0.16319	0	0	0.22524	0	0	0	0	0
Novel_000048	XLOC_00063.	transcript	0.595002	0.845603	0.477021	0.329326	0.418393	0.378526	0	0	0.18362
Novel_000049	XLOC_00063.	transcript	0	0.029073	0.095659	0	0.100673	0	0.773271	0.220682	0.292516
Novel_000050	XLOC_00063.	transcript	0	0	0	0	0	0	0.000787	0.298071	0
Novel_000051	XLOC_00081.	transcript	0.214583	0.246499	0.240018	0.212037	0.432595	0.256478	0.568608	0.192537	0.223085
Novel_000052	XLOC_00081.	transcript	0.015041	0.050101	0	0	0	0	0	0	0
Novel_000053	XLOC_00083.	transcript	0.360502	0.224707	0.354984	0.408987	0.374131	0.255464	0.353169	0.364548	0.282538
Novel_000054	XLOC_00083.	transcript	0	0	0.007685	0.070241	0	0	0.018172	0	0
Novel_000055	XLOC_00083.	transcript	0	0	0	0	0	0	0	0.096887	0
Novel_000056	XLOC_00087.	transcript	0.294337	0.00714	0.18502	0.379857	0.349852	0.411518	0.322057	0.389489	0.158656
Novel_000057	XLOC_00087.	transcript	0	0	0	0	0	0.057083	0.013228	0	0
Novel_000058	XLOC_00087.	transcript	0	0	0.280355	0.120914	0.551346	0.108581	0.162843	0.404549	0.146687
Novel_000059	XLOC_00087.	transcript	0	0	0.529023	0.19943	0	0	0	0	0
Novel_000060	XLOC_00087.	transcript	0	0	0	0.544967	0	0.349202	0	0.202703	0
Novel_000061	XLOC_00091.	transcript	0.683534	0.948358	0.901805	0.852576	0.628024	0.958797	0.789977	0.826822	0.606713
Novel_000062	XLOC_00097.	transcript	0.172983	0.250907	0.183808	0.418258	0.411615	0.454585	0.456225	0.325709	0.564507
Novel_000063	XLOC_00105.	transcript	0.270632	0.130656	0.452375	1.024758	0.403549	0.447011	0.374496	0.768314	0.431634
Novel_000064	XLOC_00105.	transcript	0.248011	0.329705	0.398616	0.501089	0.34695	0.295366	0.358788	0.319238	0.283206

Novel_000065	XLOC_00106.	transcript	0.584477	0.715664	0.602719	0.554045	0.551134	0.552969	0.969097	0.69074	0.648874
Novel_000066	XLOC_00109.	transcript	0.670548	0.996406	1.150221	1.108335	1.582211	1.447448	1.053102	0.994945	1.057071
Novel_000067	XLOC_00112.	transcript	0.296772	0.262724	0.26623	0.873317	0.328622	0.473151	0.153979	0.188272	0.176652
Novel_000068	XLOC_00112.	transcript	0.627345	0.244773	0.449575	0.281228	0.460596	0.738236	0.629945	0.492688	0.352596
Novel_000069	XLOC_00114.	transcript	0.361502	0.237686	0.422064	0.794169	0.391229	0.860393	0.299281	0.546543	0.539566
Novel_000070	XLOC_00115.	transcript	0.353347	0.544223	0.017853	0.548702	0.425793	0.674041	0.83936	0.710459	0.163948
Novel_000071	XLOC_00115.	transcript	0.469647	0	0.752676	0	0	0	0	0	0
Novel_000072	XLOC_00115.	transcript	0.117372	0.058053	0.068014	0	0.017001	0.022705	0.124126	0.050251	0
Novel_000073	XLOC_00126.	transcript	0.261166	0.247389	0.236592	0.136894	0.215733	0.081538	0.104671	0.108088	0.091426
Novel_000074	XLOC_00132.	transcript	0.100246	0.05538	0	0.056346	0	0.094845	0.082028	0.086072	0.11703
Novel_000075	XLOC_00137.	transcript	0.077495	1.543804	0.052271	0.030576	0.048994	0.096289	0.136063	0.036542	0.064376
Novel_000076	XLOC_00145.	transcript	0	0.821393	0.01489	0	0.028809	0	0	0	0
Novel_000077	XLOC_00146.	transcript	0	0	0	0	0	0	0	0	0
Novel_000078	XLOC_00146.	transcript	0.824383	1.234422	1.009228	0	0.920153	0.524688	1.321052	1.064233	1.484919
Novel_000079	XLOC_00146.	transcript	0	0	0	1.312872	0	0	0	0	0
Novel_000080	XLOC_00151.	transcript	0.213707	0.287299	0.212297	0.182787	0.166976	0.097831	0.323788	0.251541	0.063477
Novel_000081	XLOC_00151.	transcript	1.550636	1.238517	1.197103	0.759062	1.421201	1.782062	1.423449	0.649626	0.744426
Novel_000082	XLOC_00151.	transcript	0	0	0	1.169102	0.703635	0	0	0.292056	1.043779
Novel_000083	XLOC_00152.	transcript	0.237217	0.092642	0.172438	0.070413	0.264283	0.062145	0.192511	0.215771	0.234607
Novel_000084	XLOC_00152.	transcript	0	0	0	0	0	0	0.124506	0	0
Novel_000085	XLOC_00153.	transcript	0	0	0	0.126596	0.117689	0	0.128634	0.095355	0
Novel_000086	XLOC_00153.	transcript	0	0.001723	0.001298	0.146266	0.236899	0	0.108322	0.096946	0
Novel_000087	XLOC_00153.	transcript	0	0	0	0	0	0	0	0	0.000508
Novel_000088	XLOC_00153.	transcript	0	0	0	0	0	0.000995	0.335257	0	0
Novel_000089	XLOC_00153.	transcript	0	0	0	0.000646	0	0	0.000703	0.00125	0
Novel_000090	XLOC_00153.	transcript	0.149624	0.018328	0.03835	0.001813	0	0.007263	0.26416	0.104862	0.386332
Novel_000091	XLOC_00153.	transcript	0.233767	0	0.040048	0	0.110441	0	0.182351	0.100548	0
Novel_000092	XLOC_00153.	transcript	0	0.127804	0	0.160921	0.019421	0.003592	0.001355	0	0
Novel_000093	XLOC_00153.	transcript	0.195324	0.346166	0.324928	0	0	0	0	0.102768	0
Novel_000094	XLOC_00153.	transcript	0.128757	0.006841	0.029922	0.127998	0.027922	0	0	0.092601	0
Novel_000095	XLOC_00153.	transcript	0.084267	0.027681	0.013415	0.107133	0	0.617731	2.80E-05	0.098773	0.038128
Novel_000096	XLOC_00153.	transcript	0	0	0	0.186169	0	0	0	0.040462	0
Novel_000097	XLOC_00153.	transcript	0.130348	0	1.968018	0	1.938125	0	0.006254	0	0
Novel_000098	XLOC_00153.	transcript	0	0	0.009957	0	0	0	0.006292	0.006544	0.47142
Novel_000099	XLOC_00153.	transcript	0	0.000994	0	0	0	0	0.27057	0.272663	0
Novel_000100	XLOC_00153.	transcript	0.474334	1.052561	0	0	0.062256	0.005983	0.004524	0	0.03019
Novel_000101	XLOC_00153.	transcript	0	0	0.121341	0.046886	0	0	0.33068	0	0
Novel_000102	XLOC_00153.	transcript	0	0.611175	0	0	0	0	0	0	0

Novel_000103	XLOC_00153.	transcript	0.70483	0.754667	0.242536	0.158544	0.352962	0.428284	0.605281	0.308129	0.238305
Novel_000104	XLOC_00153.	transcript	0	0	0.123189	0.044968	0	0	0	0	0
Novel_000105	XLOC_00163.	transcript	0	0	0.108517	0.043014	0	0.182493	0.326259	0	0.302291
Novel_000106	XLOC_00167.	transcript	0.264032	0	0.626995	1.793704	0.941616	0.770633	0.875139	0.773674	0.197574
Novel_000107	XLOC_00167.	transcript	0	0.260749	0	0	0.009022	0	0.019641	0.148232	0
Novel_000108	XLOC_00167.	transcript	1.16852	0.01524	1.38432	2.692507	1.096143	0.744494	1.205108	1.290022	1.675916
Novel_000109	XLOC_00167.	transcript	0	0	0	0	0	0	0	0.145708	0
Novel_000110	XLOC_00167.	transcript	0	0	0	0.277895	0.636651	0.765554	0.129962	0.487299	0
Novel_000111	XLOC_00168.	transcript	0.270703	0.370195	0.243288	0.305762	0.31364	0.315111	0.414052	0.278016	0.284931
Novel_000112	XLOC_00169.	transcript	0.090596	0.197662	0.472324	0.653187	0.273246	0.581269	0.426111	0.347705	0.701733
Novel_000113	XLOC_00169.	transcript	0.074674	0.047519	0	0	0	0	0	0	0
Novel_000114	XLOC_00170.	transcript	0	0.014922	0	0.014171	0.034666	0.169788	0.030912	0.082883	0
Novel_000115	XLOC_00170.	transcript	0	0	0	0.145243	0	0	0.054284	0	0
Novel_000116	XLOC_00170.	transcript	0.149255	0.719998	0.442542	0.134792	0.380077	0.214399	0.307909	0.343339	0.030141
Novel_000117	XLOC_00170.	transcript	0	0.008099	0	0	0.003182	0.00781	0.001924	0	0.157643
Novel_000118	XLOC_00170.	transcript	0	0	0	0.118116	0	0	0	0	0
Novel_000119	XLOC_00170.	transcript	0.314116	0.04018	0.024402	0.022992	0.025967	0.02041	0.033162	0.030189	0.017967
Novel_000120	XLOC_00170.	transcript	0	0	0	0	0	0.46126	0	0	0
Novel_000121	XLOC_00172.	transcript	0.527978	0.260152	0.518409	0.546473	0.512974	0.583368	0.378141	0.63491	0.543415
Novel_000122	XLOC_00172.	transcript	0	0	0	0	0.001104	0	0.211549	0	0
Novel_000123	XLOC_00173.	transcript	9.920002	3.557862	9.212484	11.50294	8.93407	8.362062	9.371582	10.8472	8.691916
Novel_000124	XLOC_00175.	transcript	0	0.21254	0.282833	0.452295	0.148673	0	0.36825	0.432343	0.070266
Novel_000125	XLOC_00176.	transcript	0.244064	3.203863	0.155127	0.112456	0.336466	0.214263	0.523395	0.10915	0.707125
Novel_000126	XLOC_00176.	transcript	0.476528	0.542403	0.407932	0.140475	0.264024	0.481838	0.441973	0.405324	0.144844
Novel_000127	XLOC_00176.	transcript	0	0.366064	0	0	0	0	0	0	0
Novel_000128	XLOC_00176.	transcript	0.349101	0.337107	0.17575	0.022104	0.2532	0.12926	0.401073	0.353204	0.304961
Novel_000129	XLOC_00176.	transcript	0.034149	0	0.005736	0.272013	0	0.049488	0	0	0.004039
Novel_000130	XLOC_00176.	transcript	0	0.002874	0.003861	0.002024	0.002932	0	0.18993	0.000807	0
Novel_000131	XLOC_00178.	transcript	0.605506	0.295022	0.365915	0.228939	0.375849	0.445709	0.636485	0.417441	0.229404
Novel_000132	XLOC_00179.	transcript	0.202097	0.609441	0.20223	0.234973	0.190303	0.196909	0.219173	0.185534	0.21933
Novel_000133	XLOC_00179.	transcript	0.32838	0.125126	0.194441	0.21037	0.47155	0.401949	0.230431	0.547455	0.546976
Novel_000134	XLOC_00179.	transcript	0	0	0	0	0	0	0	0	0
Novel_000135	XLOC_00179.	transcript	0	0.439835	0.380954	0.378311	0	0	0.510098	0.08822	0
Novel_000136	XLOC_00179.	transcript	0.167119	0.125438	0.187517	0.192715	0.142836	0.059237	0.19071	0.115706	0.362209
Novel_000137	XLOC_00180.	transcript	0.156551	0	0.066759	0.18219	0.04437	0.08968	0.263446	0.228135	0.173401
Novel_000138	XLOC_00182.	transcript	0.117652	0.009177	0.001995	0.164829	0	0.0686	0.193402	0.238739	0
Novel_000139	XLOC_00182.	transcript	0.125853	0.046937	0.395178	0.152108	0.19317	0.169622	0.380019	0.227023	0.220986
Novel_000140	XLOC_00183.	transcript	1.289862	0.245288	0.8074	1.228953	2.176462	2.329601	1.41617	1.237753	0.900609

Novel_000141	XLOC_00183.	transcript	0.81704	0.166347	1.409741	1.235322	1.325231	1.432282	0.994411	0.928794	0.675885
Novel_000142	XLOC_00183.	transcript	0.520228	0.011958	1.257592	1.910291	1.091984	1.092147	2.359059	1.171502	0.685861
Novel_000143	XLOC_00183.	transcript	1.801341	0.258437	1.907264	2.134007	2.529854	1.750213	1.994691	3.181882	1.642064
Novel_000144	XLOC_00184.	transcript	0	0.163346	0.39101	0.510098	0.097128	0.037254	0.018402	0.088243	0.260821
Novel_000145	XLOC_00184.	transcript	0	0	0.13793	0	0.193643	0	0.197071	0.37782	0.095126
Novel_000146	XLOC_00184.	transcript	0.153893	0.070467	0.096055	0.124703	0.108701	0.27595	0.294044	0.115372	0.151146
Novel_000147	XLOC_00185.	transcript	0	0	0	0	0	0	0	8.767158	0
Novel_000148	XLOC_00189.	transcript	0.979603	0.541818	0.718953	0.463807	0.926938	0.861249	1.110413	1.028142	0.892293
Novel_000149	XLOC_00189.	transcript	0	0.476335	0	0.227112	0	0	0	0	0
Novel_000150	XLOC_00189.	transcript	0.039373	0.237785	0.160735	0.046798	0.067375	0.112204	0.035621	0.042129	0.168639
Novel_000151	XLOC_00189.	transcript	0.004533	0	0	0.359321	0	0	0.007244	0	0.007004
Novel_000152	XLOC_00189.	transcript	0	0	0	0	0	0	0	0	0.429367
Novel_000153	XLOC_00189.	transcript	0	0	0.43169	0	0.421652	1.459453	0	0	0
Novel_000154	XLOC_00189.	transcript	0.913203	0.816656	1.296255	1.042722	1.205099	0.956642	1.091439	1.168371	0.864493
Novel_000155	XLOC_00190.	transcript	0.297082	0.400035	0.413939	0.384386	0.42712	0.489806	0.317735	0.474279	0.432027
Novel_000156	XLOC_00190.	transcript	0.112967	0.040264	0.026683	0.15083	0.116142	0.090593	0.247886	0.223597	0.069843
Novel_000157	XLOC_00190.	transcript	0.490201	0.573613	0.410419	0.552653	0.601449	0.367402	0.196343	0.521384	0.185122
Novel_000158	XLOC_00190.	transcript	0.251119	0.105931	0.309084	0.13383	0.105931	0.31923	0.607528	0.134337	0.212043
Novel_000159	XLOC_00191.	transcript	0.179906	0	0.127268	0.064998	0	0.103725	0.164445	0.087171	0
Novel_000160	XLOC_00192.	transcript	0.508303	0.466048	0.711618	0.873072	0.976354	1.211081	0.818361	0.680627	0.828433
Novel_000161	XLOC_00192.	transcript	0.468328	0.198918	0.583308	0.290283	0.380605	0.210941	0.342758	0.480111	0.330691
Novel_000162	XLOC_00192.	transcript	0.382937	0	0	0	0	0.280301	0.427786	0	0.252737
Novel_000163	XLOC_00192.	transcript	0	0.380574	0.373259	0.195018	0.322292	0.123822	0	0.38667	0
Novel_000164	XLOC_00192.	transcript	0.04154	0.109817	0	0	0.004038	0.019132	0.03511	0	0.041819
Novel_000165	XLOC_00192.	transcript	0	0.313907	0.354222	0.101485	0.107208	0.086785	0.132649	0.209023	0.070262
Novel_000166	XLOC_00192.	transcript	0.055902	0.103348	0	0.320356	0.117714	0.009386	0.011968	0.154966	0
Novel_000167	XLOC_00192.	transcript	0.430286	0.359245	0.667742	0.679552	0.833605	0.923117	0.622066	1.025946	0.263616
Novel_000168	XLOC_00192.	transcript	0	0	0	0	0	0	0	0	0
Novel_000169	XLOC_00193.	transcript	0	0.428943	0.087052	0.182686	0	0	0	0	0
Novel_000170	XLOC_00193.	transcript	0.74278	0.10858	0.512095	0.488762	0.601437	0.57201	0.669791	0.681701	0.419402
Novel_000171	XLOC_00197.	transcript	0.184565	0.447771	0.291201	0.341769	1.194017	1.113341	1.046313	1.177546	0.317579
Novel_000172	XLOC_00197.	transcript	0.631719	0.105008	0.026391	0.060572	0.046368	0.048646	0.455574	0.080975	0.070008
Novel_000173	XLOC_00197.	transcript	0.060827	0.168787	0.0642	0.632169	0.22632	0.27637	0.236819	0.196009	0.080604
Novel_000174	XLOC_00197.	transcript	0.095651	0.170179	0.062715	0	0.043696	0.248323	0.027853	0	0.119051
Novel_000175	XLOC_00197.	transcript	0.351972	0.044874	0.033517	0.06913	0.104487	0.008995	0.532488	0.183845	0.183313
Novel_000176	XLOC_00197.	transcript	0.01577	0	0	0	0	0	0	0.159687	0.066839
Novel_000177	XLOC_00197.	transcript	0	0	0.404746	0.415191	0.067074	0.208108	0.011618	0.219219	0
Novel_000178	XLOC_00197.	transcript	0.022664	0	0	0	0.358595	0.001766	0	0	0

Novel_000179	XLOC_00204.	transcript	2.11806	1.846553	0	0	0	0	0	2.974463	0.596449
Novel_000180	XLOC_00204.	transcript	0	0	2.531439	2.618244	2.37067	3.684718	2.763058	0.197691	1.41702
Novel_000181	XLOC_00207.	transcript	0.244567	0.367691	0.244924	0.174246	0.189335	0.217722	0.247117	0.266973	0.170204
Novel_000182	XLOC_00210.	transcript	0.472909	0.213741	0.519344	0.650535	0.249838	0.518234	0.007369	0.107094	0.451669
Novel_000183	XLOC_00210.	transcript	0.027196	0	0.022292	0.001178	0.000295	0.00121	0	0.08472	0.006642
Novel_000184	XLOC_00210.	transcript	0.040431	0.430124	0	0.054577	0.240231	0.014602	0.671816	0.376532	0
Novel_000185	XLOC_00210.	transcript	0	0	0	0.004559	0.000362	0.014729	0	0.090814	0
Novel_000186	XLOC_00213.	transcript	0.532004	0.806926	0.182695	0.265961	0.214989	0.508728	0.268154	0.263688	0.157424
Novel_000187	XLOC_00213.	transcript	0	0	0.164529	0	0	0	0	0	0
Novel_000188	XLOC_00213.	transcript	0.337176	2.437477	0.632089	1.146247	1.019652	0.744282	1.120124	1.449762	1.097998
Novel_000189	XLOC_00217.	transcript	0.392682	0.165413	0.45772	0.690588	0.421282	0.401423	0.419307	0.491673	0.35976
Novel_000190	XLOC_00219.	transcript	0.066696	0.049803	0.355299	0.058306	0.095867	0.094021	0	0.05076	0
Novel_000191	XLOC_00219.	transcript	0.392885	0	0.00097	0.229299	0.244614	0.087673	0	0.323844	0.245232
Novel_000192	XLOC_00219.	transcript	0	0.136629	0	0.090886	0.12884	0.325126	0.493971	0	0.186711
Novel_000193	XLOC_00219.	transcript	0	0	0.01607	0	0	0	0.034986	0.123402	0
Novel_000194	XLOC_00227.	transcript	5.451008	3.491136	5.888733	6.079311	5.069932	5.754828	5.380108	6.307585	5.0482
Novel_000195	XLOC_00232.	transcript	0.094505	0.069313	0.002939	0.036215	0	0.037786	0.092029	0.020359	0.044783
Novel_000196	XLOC_00232.	transcript	0.304667	0.359455	0.317674	0.236686	0.43034	0.228097	0.3158	0.385648	0.259288
Novel_000197	XLOC_00232.	transcript	0.258071	0.149098	0.18582	0.07904	0.230421	0.061727	0.099454	0.133609	0.07027
Novel_000198	XLOC_00236.	transcript	0.226078	0.038647	0.469285	0.325047	0.474493	0.216201	0.718884	0.519977	0.268827
Novel_000199	XLOC_00236.	transcript	0.620242	0	0.108105	0.553211	0.833355	0.758246	0.543293	0.260319	0.226418
Novel_000200	XLOC_00240.	transcript	0	0	0	0.121052	0	0	0	0	0.020398
Novel_000201	XLOC_00240.	transcript	0.897143	0.829736	1.00853	1.965673	1.380895	1.759083	1.519312	1.119395	1.764967
Novel_000202	XLOC_00240.	transcript	0	0	0	0	0.068691	0.348707	0	0.185731	0
Novel_000203	XLOC_00240.	transcript	0	0.020472	0	0.023431	0.028369	0	0.607413	0	0
Novel_000204	XLOC_00240.	transcript	0	0	0	0	0	0	0	0.108262	0
Novel_000205	XLOC_00240.	transcript	0.186412	0.12024	0.110855	0.190999	0.148461	0.116823	0	0	0.15119
Novel_000206	XLOC_00240.	transcript	0	0	0	0	0	0	0	0	0
Novel_000207	XLOC_00240.	transcript	0	0	0	0	0	0	0	0.220826	0
Novel_000208	XLOC_00240.	transcript	0.003832	0	0	0	0	0	0	0.118823	0
Novel_000209	XLOC_00240.	transcript	0	1.10E-05	0	0	0	8.00E-06	0.021795	0	0
Novel_000210	XLOC_00240.	transcript	0.072547	0.065085	0	0.020347	0.048103	0	0.038661	0	0.009888
Novel_000211	XLOC_00240.	transcript	0.056717	0.056146	0	0.013463	0.030755	0	0.039574	0	0.047995
Novel_000212	XLOC_00240.	transcript	0.123584	0.015695	0.006596	0.025824	0.034276	0.009284	0.06744	0.102195	0.009951
Novel_000213	XLOC_00240.	transcript	0.115436	0	0	0	0	0	0.10183	0	0.021782
Novel_000214	XLOC_00240.	transcript	0.04824	0	0	0	0	0	0.034939	0	0.038699
Novel_000215	XLOC_00240.	transcript	0.778794	3.28011	1.837278	2.564898	2.055037	1.919301	2.367669	2.338655	1.597821
Novel_000216	XLOC_00241.	transcript	0.716941	0.871271	0.727661	0.234967	0.744927	0.543621	0.852478	0.819871	0.364346

Novel_000217	XLOC_00241.	transcript	0	0	0	0.598482	0	0	0	0	0
Novel_000218	XLOC_00241.	transcript	1.5098	2.928969	1.412801	1.470595	1.158536	1.243538	2.292794	1.613798	2.531746
Novel_000219	XLOC_00241.	transcript	4.079622	2.665576	3.796744	4.448026	4.087142	3.712985	4.907023	5.744363	5.737828
Novel_000220	XLOC_00241.	transcript	0	0	0.8677	0	0	0	0.392196	0	0
Novel_000221	XLOC_00241.	transcript	0.964401	0.269613	0.10848	1.053781	1.127433	0.282577	1.499227	1.387817	1.169611
Novel_000222	XLOC_00241.	transcript	0.027817	0.051573	0.156043	0	0.009964	0.685493	0.033108	0	0.044277
Novel_000223	XLOC_00241.	transcript	0.223275	0	0.01847	0.227292	0	0	0.010825	0.454522	0
Novel_000224	XLOC_00241.	transcript	0.332067	0.151084	0.275136	0.081379	0.298638	0.412407	0.438762	0.160461	0.144881
Novel_000225	XLOC_00241.	transcript	0	0.322587	0	0	0	0	0	0	0
Novel_000226	XLOC_00241.	transcript	0.303525	0.130131	0.380796	0.263271	0.302938	0.277359	0.340933	0.157542	0.352656
Novel_000227	XLOC_00241.	transcript	0.064996	0	0	0.229635	0.023889	0	0.06402	0.148445	0
Novel_000228	XLOC_00242.	transcript	0.493585	0.044855	0.19565	0.252099	0.221361	0.177282	0.377497	0.220982	0.055029
Novel_000229	XLOC_00243.	transcript	0.334604	0	0	0	0	0	0.245131	0	0
Novel_000230	XLOC_00243.	transcript	0.050628	0	0	0	0	0	0	0.129906	0
Novel_000231	XLOC_00243.	transcript	0.903253	0.576962	0.808684	0.858691	0.488664	0.877138	1.001995	0.760892	1.334228
Novel_000232	XLOC_00244.	transcript	0.962537	0.106915	0.47819	0.203276	0.691543	0.455282	0.201037	0.560867	0.65074
Novel_000233	XLOC_00244.	transcript	0.008086	0.029762	0	0.17825	0.004405	0.110877	0.199587	0.005067	0.008649
Novel_000234	XLOC_00244.	transcript	0	0	0.465623	0.087536	0	0.357246	0.243226	0.268647	0
Novel_000235	XLOC_00244.	transcript	0	0	0	0	0	0	0.211801	0	0
Novel_000236	XLOC_00246.	transcript	0.896075	1.636	0.185768	1.075264	1.11718	1.036531	0.984156	0.978712	0.445375
Novel_000237	XLOC_00246.	transcript	0	0	0.633614	0	0	0	0	0	0
Novel_000238	XLOC_00250.	transcript	0.30881	0.584326	0.863587	0.732546	0.599305	0.628414	1.130649	0.683087	0.220568
Novel_000239	XLOC_00250.	transcript	1.46197	0.63365	0.184519	0.028548	0.309314	0.358979	0.550628	0.330402	0.62958
Novel_000240	XLOC_00251.	transcript	0.473014	0.156452	0.062754	0	0	0.370937	0.430109	0	0.438137
Novel_000241	XLOC_00251.	transcript	0.011105	0.098093	0.351289	0.536304	0.315925	0	0	0.204281	0
Novel_000242	XLOC_00251.	transcript	0	0	0	0.082693	0	0	0	0	0
Novel_000243	XLOC_00251.	transcript	0.084762	0.009822	0.031945	0.044705	0.143807	0.186884	0.225254	0.133283	0
Novel_000244	XLOC_00251.	transcript	0.058294	0.144104	0.233935	0.073039	0.019911	0.008203	0.100769	0.188475	0
Novel_000245	XLOC_00251.	transcript	0	0	0	0	0	0	0	0	0
Novel_000246	XLOC_00251.	transcript	0	0	0	0	0	0	0.338011	0	0
Novel_000247	XLOC_00252.	transcript	0.060069	0.441528	0.046359	0.320901	0.173223	0.552524	0.435957	0.44469	0.168006
Novel_000248	XLOC_00271.	transcript	0.139777	0	0.141353	0.143399	0	0.325998	0.112055	0.21747	0
Novel_000249	XLOC_00274.	transcript	0	0.896982	1.465668	3.520811	2.206594	1.75619	1.51724	3.326443	1.547063
Novel_000250	XLOC_00274.	transcript	1.117243	0.073471	0.294726	1.432679	0.221436	0.361033	0.65587	0.27176	0.175992
Novel_000251	XLOC_00278.	transcript	0.700861	0.014006	0.544613	0.896959	0.756698	0.692078	0.866844	0.632327	2.152956
Novel_000252	XLOC_00278.	transcript	0.490661	0.767144	0.537205	0.543721	0.45453	0.616001	0.190166	0.554882	0.538987
Novel_000253	XLOC_00278.	transcript	0	0	0	0	0.051433	0	0.382616	0.016827	0
Novel_000254	XLOC_00278.	transcript	0.397562	0.0655	0.499026	0.326389	0.574334	0.325242	0.074243	0	0.391371

Novel_000255	XLOC_00278.	transcript	0.153927	0.082714	0.176335	0.324305	0.153494	0.281624	0.722008	0.686395	0.231508
Novel_000256	XLOC_00279.	transcript	1.334769	1.241862	1.336283	0.854128	1.322323	0.43658	1.392425	0.696151	0.665296
Novel_000257	XLOC_00279.	transcript	0.593124	0	0.14276	0.486848	0	0.837341	0	0.313759	0.136053
Novel_000258	XLOC_00292.	transcript	1.07707	0.686884	0.784226	0.924789	0.887278	0.658103	0.53655	0.999185	0.615164
Novel_000259	XLOC_00294.	transcript	0.915328	1.113967	1.036174	0.837961	0.839457	1.122688	0.791182	0.824766	0.695176
Novel_000260	XLOC_00299.	transcript	0	0	0	0.214205	0	0.195166	0	0.240022	0.069231
Novel_000261	XLOC_00301.	transcript	0.4731	0.061436	0.449837	0.600079	0.391046	0.645356	0.424574	0.790829	0.24852
Novel_000262	XLOC_00301.	transcript	0.003041	0.019155	0.099358	0.001297	0.237877	0.01488	0	0.114756	1.10E-05
Novel_000263	XLOC_00301.	transcript	0.283798	0	0.506276	0.333958	0.216864	0.698339	0.485008	0.333589	0.419082
Novel_000264	XLOC_00301.	transcript	0	0.087728	0	0	0.176703	0	0	0.117166	0
Novel_000265	XLOC_00303.	transcript	0.702428	0.410579	0.889975	0.792239	0.645064	0.961175	0.640369	0.527298	1.197018
Novel_000266	XLOC_00304.	transcript	1.07243	0.889496	0.380266	0.765831	0.785441	0.728946	0.62416	1.109043	0.618483
Novel_000267	XLOC_00304.	transcript	0.107581	0.012708	0.164185	0	0.011599	0.209408	0.241241	0.188637	0.139117
Novel_000268	XLOC_00304.	transcript	0.114103	0	0.194839	0	0.230166	0.400976	0	0.274972	0
Novel_000269	XLOC_00304.	transcript	0	0	0	0	0	0	0.125075	0	0
Novel_000270	XLOC_00304.	transcript	0.005867	0	0.003059	0.234342	0.006836	0	0	0	0
Novel_000271	XLOC_00304.	transcript	0.020322	0	0	0.278768	0	0	0	0	0
Novel_000272	XLOC_00305.	transcript	0	1.021593	0.069559	0	0	0	0	0.506314	0
Novel_000273	XLOC_00305.	transcript	0	0	1.025327	0	0	0	0	0.002647	0
Novel_000274	XLOC_00306.	transcript	0.118595	1.052648	0.343617	0.426074	0.427072	0.371961	0.448285	0.444251	0.029089
Novel_000275	XLOC_00314.	transcript	0	0.025897	0.055872	0.053709	0.03044	0.040158	0.319445	0.098664	0.100068
Novel_000276	XLOC_00314.	transcript	0.570904	0.758823	0.634739	0.533086	0.486742	0.416823	0.973551	0.787916	0.579261
Novel_000277	XLOC_00315.	transcript	0.078285	0.016307	0	0.083359	0.092145	0.084904	0.188065	0.126225	0.076006
Novel_000278	XLOC_00315.	transcript	0.331221	0.159156	0.220276	0.213894	0.167537	0.253507	0.007011	0.192451	0.214028
Novel_000279	XLOC_00315.	transcript	0	0	0	0.016853	0.029042	0	0	0.063067	0
Novel_000280	XLOC_00315.	transcript	0	0	0	0	0	0	0.188143	0	0
Novel_000281	XLOC_00319.	transcript	0	0.003659	0.061402	0.036548	0.032014	0.066461	0.049193	0.007435	0.011192
Novel_000282	XLOC_00319.	transcript	0.223122	0.087467	0.202733	0.228179	0.195801	0.156838	0.403786	0.239499	0.189796
Novel_000283	XLOC_00322.	transcript	0.058599	0.246436	0.131297	0.147101	0.220997	0.230275	0	0.169978	0.07735
Novel_000284	XLOC_00322.	transcript	0	0	0	0	0	0	0.189763	0	0
Novel_000285	XLOC_00322.	transcript	0.170228	0	0.222552	0.044201	0.016209	0	0.015198	0.079258	0.085454
Novel_000286	XLOC_00322.	transcript	0.182239	0.588116	0.130411	0.123014	0.077449	0.027717	0.205441	0.128867	0.152854
Novel_000287	XLOC_00322.	transcript	0.1222	0	0.055368	0.24141	0.151706	0.192128	0.182174	0.129302	0.16551
Novel_000288	XLOC_00322.	transcript	0	0	0	0	0	0	0	0	0
Novel_000289	XLOC_00324.	transcript	0.45704	1.476019	0.704775	0.735288	0.745826	0.826173	0.943202	0.784828	1.162646
Novel_000290	XLOC_00326.	transcript	0.072738	0.254754	0.076899	0.040381	0.099248	0.116048	0.163109	0.034724	0.385296
Novel_000291	XLOC_00327.	transcript	0.105535	0.419822	0.063159	0.016473	0.010051	0.007905	0.047936	0.113637	0.011787
Novel_000292	XLOC_00327.	transcript	0.200521	0.242244	0.184947	0.118951	0.163193	0.171715	0.132989	0.15779	0.111497

Novel_000293	XLOC_00327.	transcript	0.331714	0.150532	0.111733	0.108072	0.227306	0.146519	0.461165	0.133145	0.184352
Novel_000294	XLOC_00327.	transcript	0.039001	0.058814	0.030595	0.057091	0	0	0	0.068806	0.045102
Novel_000295	XLOC_00327.	transcript	0.078697	0.134723	0.072754	0.064876	0.109244	0.059085	0	0.22566	0.052589
Novel_000296	XLOC_00327.	transcript	0	0	0	0	0	0	0	0	0
Novel_000297	XLOC_00327.	transcript	0.07854	0.139871	0.089714	0.123037	0.111358	0.104013	0.188971	0.134815	0.176419
Novel_000298	XLOC_00327.	transcript	0	0	0	0	0	0	0	0	0
Novel_000299	XLOC_00327.	transcript	0.04972	0	0.045412	0	0	0	0	0.091859	0
Novel_000300	XLOC_00327.	transcript	0.043399	0.332825	0.042849	0.100986	0	0	0.041223	0.109282	0.053319
Novel_000301	XLOC_00327.	transcript	0.032416	0.158211	0.034198	0.061312	0	0	0	0.051208	0.044436
Novel_000302	XLOC_00327.	transcript	0.053431	0.007397	0.046573	0.001755	0.022104	0.0014	0.015475	0.108182	0.000779
Novel_000303	XLOC_00327.	transcript	0.372291	0.215561	0.140439	0.179166	0.422388	0.348394	0.417479	0.133167	0.147051
Novel_000304	XLOC_00327.	transcript	0.048672	0.070495	0	0.102526	0	0.070105	0.068624	0.104944	0
Novel_000305	XLOC_00327.	transcript	0.028975	0.035178	0	0.059747	0	0.047629	0.002623	0.15075	0
Novel_000306	XLOC_00327.	transcript	0	2.4639	0	0	0	0.067291	0.106885	0.188392	0.179156
Novel_000307	XLOC_00327.	transcript	0	0.520635	0	0	0	0	0	0	0
Novel_000308	XLOC_00332.	transcript	0.064254	0	0	0	0	0	0	0	0
Novel_000309	XLOC_00332.	transcript	0	0.044792	0	0	0	0	0	0	0
Novel_000310	XLOC_00337.	transcript	0.311286	0.258337	0.245752	0.14091	0.11362	0.099289	0.13641	0.093648	0.132761
Novel_000311	XLOC_00342.	transcript	0.918714	2.014059	0.974341	1.578857	1.248124	1.344134	0.91679	0.881403	1.489057
Novel_000312	XLOC_00343.	transcript	0.083968	0.202332	0.060896	0.050133	0.075671	0.154248	0.108219	0.192527	0.231297
Novel_000313	XLOC_00343.	transcript	0.183476	0.03123	0.260297	0.23043	0.237842	0.015988	0.349093	0.192133	0
Novel_000314	XLOC_00343.	transcript	0	0	0	0	0	0	0	0	0
Novel_000315	XLOC_00343.	transcript	0	0	0	0	0	0	0	0	0
Novel_000316	XLOC_00345.	transcript	1.627245	1.216346	1.008142	0.899564	1.119228	0.837135	1.572162	1.2549	1.278662
Novel_000317	XLOC_00349.	transcript	0.014756	0.182041	0.036465	0	0.006953	0.074923	0.009593	0.014111	0.184517
Novel_000318	XLOC_00349.	transcript	0.414585	0.291975	0.195808	0.372096	0.390268	0.421456	0.417219	0.424845	0.505409
Novel_000319	XLOC_00350.	transcript	0	0	0.170383	0.066651	0	0.139412	0.072187	0.155528	0.156222
Novel_000320	XLOC_00365.	transcript	0	0	0	0	0	0	0.009	0	0
Novel_000321	XLOC_00374.	transcript	0.290357	0.613787	0.32752	0.291562	0.39039	0.57039	0.387509	0.265353	0.689363
Novel_000322	XLOC_00378.	transcript	0.47291	0.137819	0.347846	0.090859	0.177745	0.198923	0.342696	0.232856	0.214411
Novel_000323	XLOC_00382.	transcript	1.489805	0.278113	0.700849	0.602889	0.749317	0.321829	0.568845	0.189191	0.182993
Novel_000324	XLOC_00382.	transcript	0.194799	0.258313	0.306166	0.26538	0.267581	0.273941	0.675932	0.858709	0.288085
Novel_000325	XLOC_00382.	transcript	0.108107	0.008598	0	0	0	0	0	0	0
Novel_000326	XLOC_00382.	transcript	0.453411	0.165688	0.386511	0.4327	0.327952	0.339409	0.370241	0.604303	0.38554
Novel_000327	XLOC_00382.	transcript	0.120503	0.130666	0.156505	0.338322	0.296708	0	0.269398	0.207155	0.45771
Novel_000328	XLOC_00382.	transcript	0.034886	0	0.125596	0.185566	0.007328	0.037651	0.021524	0.108343	0
Novel_000329	XLOC_00382.	transcript	0	0	0	0	0	0	0	0	0
Novel_000330	XLOC_00382.	transcript	1.119445	0.431197	0.837935	0.234703	0.89477	0.852625	1.366244	0.700763	0.458054

Novel_000331	XLOC_0038[illegible]	transcript	0.236223	0.974805	0.230132	0.293005	0.436827	0.30177	0.406263	0.356553	0.256176
Novel_000332	XLOC_00387	transcript	0.294532	0.010543	0.678676	0.427186	0.833196	0.957219	0.341644	0.295508	0.551296
Novel_000333	XLOC_00387	transcript	0.287526	0.012321	0.170241	0.268375	0	0	0.682833	0.519586	0
Novel_000334	XLOC_00387	transcript	0.348556	0.019485	0.04739	0	0	0.002728	0.142358	0.157555	0.005258
Novel_000335	XLOC_00387	transcript	0.306303	0.018356	0.03004	0	0	0.013633	0.161226	0.140724	0.019812
Novel_000336	XLOC_00387	transcript	0	1.40181	0.024202	0	0	0	0	0	0
Novel_000337	XLOC_00387	transcript	0.696425	0.429156	0	0.021096	0.059568	0	0.020548	0.042397	0
Novel_000338	XLOC_00387	transcript	0.274523	0.575195	0.038433	0.025326	0.00463	0.096651	0.009047	0.010789	0.015667
Novel_000339	XLOC_00387	transcript	0.179097	0	0	0	0	0	0.129848	0.151438	0
Novel_000340	XLOC_0038[illegible]	transcript	0.221145	0.038745	0.270425	0.219408	0.183603	0.243661	0.109296	0.233708	0.083104
Novel_000341	XLOC_0039[illegible]	transcript	0.06308	0.040708	0.251183	0.04804	0.235772	0.046504	0.022232	0.034715	0.008493
Novel_000342	XLOC_0039[illegible]	transcript	0	0	0.004602	0.004263	0.012877	0	0	0	0
Novel_000343	XLOC_0039[illegible]	transcript	0.414555	0.118524	0	0	0	0.271825	0.077277	0.202313	0.214137
Novel_000344	XLOC_0039[illegible]	transcript	0	0	0	0.125187	0	0	0.343295	0.048784	0
Novel_000345	XLOC_00391	transcript	0.209815	0.282655	0.177674	0.557316	0.312572	0.265988	0.248252	0.346128	0.260674
Novel_000346	XLOC_00391	transcript	0	0	0	0.071208	0	0	0	0	0
Novel_000347	XLOC_00392	transcript	0.04825	0.040918	0	0	0	0	0	0.182606	0
Novel_000348	XLOC_00392	transcript	0.118387	0.289327	0.107704	0.134465	0.13043	0.178458	0.195374	0.009677	0.187495
Novel_000349	XLOC_00392	transcript	0.347607	0.142034	0.263685	0.587594	0.295841	0.075237	0.101911	0.180244	0.029898
Novel_000350	XLOC_00392	transcript	0.004681	0	0	0	0.023795	0	0.030448	0.263975	0.046726
Novel_000351	XLOC_00392	transcript	0	0	0.433585	0	0	0	0	0	0
Novel_000352	XLOC_00392	transcript	1.304593	1.854821	0.405328	0.605107	1.218579	1.025057	1.836519	1.247904	1.001825
Novel_000353	XLOC_00392	transcript	0	0.360193	0.146646	0.059269	0	0.341292	0.055455	0.036953	0
Novel_000354	XLOC_00392	transcript	0.165459	0.095232	0.102034	0.174478	0.220113	0.201239	0.280766	0.176762	0.165005
Novel_000355	XLOC_00393	transcript	0.219424	0	0	0	0	0	0.177361	0	0.152466
Novel_000356	XLOC_00393	transcript	0.253674	0.162956	0	0	0	0.907164	0.225698	0	0.161726
Novel_000357	XLOC_00393	transcript	0.089657	0.156553	0.228722	0.144671	0.25966	0.293775	0.111361	0.124032	0.52368
Novel_000358	XLOC_00393	transcript	0.084076	0.000978	0.406876	0.216931	0.394327	0.009084	0.164899	0.52772	0.167714
Novel_000359	XLOC_00393	transcript	0.070698	0.090069	0	0.192611	0	0	0	0	0
Novel_000360	XLOC_00393	transcript	0	0	0	0	0	0	0	0	0
Novel_000361	XLOC_00393	transcript	0.167908	0.149327	0	0	0	0	0.297646	0	0
Novel_000362	XLOC_00394	transcript	0.004436	0	0.052091	0	0.012248	0.030615	0	0.006532	0.017575
Novel_000363	XLOC_00394	transcript	0.134427	0.013539	0.185602	0.153776	0.149484	0.463375	0.173153	0.209742	0.158698
Novel_000364	XLOC_00397	transcript	1.790893	0	2.640335	3.528329	2.594223	2.19357	4.129413	3.966114	0.616855
Novel_000365	XLOC_0040[illegible]	transcript	0.083207	0	0.131981	0.098705	0.085073	0.11522	0.097971	0.055406	0.141887
Novel_000366	XLOC_0040[illegible]	transcript	0.309181	0.189518	0.314357	0.363974	0.457519	0.327363	0.4236	0.482251	0.516124
Novel_000367	XLOC_00401	transcript	0.369987	0.044403	0.154334	0.108965	0.277176	0.05744	0.164354	0.080439	0.39376
Novel_000368	XLOC_00401	transcript	0	0.013658	0.051422	0	0	0.161754	0.046186	0	0

Novel_000369	XLOC_00401	transcript	0	0	0	0.006445	0	0	0.058615	0.082314	0
Novel_000370	XLOC_00401	transcript	0	0	0.035423	0.058426	0.04883	0	0	0	0.053999
Novel_000371	XLOC_00401	transcript	0	0.375563	0.031673	0.369331	0.033135	0	0.265601	0.252167	0
Novel_000372	XLOC_00405	transcript	0.682697	0.62788	2.810384	3.504227	2.790903	3.505956	2.880129	3.260473	1.791182
Novel_000373	XLOC_00405	transcript	0.394763	0	0	0	0	0	0	0	0
Novel_000374	XLOC_00405	transcript	0	0	0	0	0	0	0	0	0
Novel_000375	XLOC_00405	transcript	0.203433	0.180002	0.132995	0.137073	0.069212	0.213545	0.260322	0.169559	0.143956
Novel_000376	XLOC_00412	transcript	0	0.035454	0.05074	0	0	0.071997	0.071789	0.031223	0
Novel_000377	XLOC_00412	transcript	0.048174	0	0	0.05314	0.072212	0.075986	0.281007	0	0
Novel_000378	XLOC_00417	transcript	0.533696	1.719378	0.781923	0.996227	0.842623	0.413447	0.456412	0.556812	0.741689
Novel_000379	XLOC_00417	transcript	0.006857	0	0.007116	0	0.00752	0	0	0.370688	0
Novel_000380	XLOC_00418	transcript	0.32158	0	0	0	0.053549	1.100132	0	0.007621	0.471565
Novel_000381	XLOC_00418	transcript	0.078509	0.527831	0.369173	0	0.449531	0.10595	0.185204	0.22173	0.256838
Novel_000382	XLOC_00418	transcript	1.011508	2.793266	1.722504	1.441976	1.332019	0.855411	1.150651	1.914829	1.800113
Novel_000383	XLOC_00418	transcript	0.06548	0.901864	0.448718	0.68311	0.045055	0	0.400164	0.03583	0
Novel_000384	XLOC_00422	transcript	1.424037	0.798176	0.973102	1.162923	1.104462	1.126368	1.442193	1.245102	0.836396
Novel_000385	XLOC_00422	transcript	0.009094	0.035226	0.008095	0.007043	0	0	0.016558	0.006455	0.005365
Novel_000386	XLOC_00422	transcript	0.443401	0.424504	0.296342	0.338288	0.341147	0.346377	0.39297	0.381362	0.253726
Novel_000387	XLOC_00428	transcript	0.206085	0.162092	0.184731	0.236683	0.231273	0.274299	0.444738	0.176423	0.165839
Novel_000388	XLOC_00428	transcript	0.106838	0.016907	0.085278	0.038234	0.10044	0.072755	0.088895	0.132925	0.178485
Novel_000389	XLOC_00428	transcript	0.321321	0.366925	0.196122	0.192256	0.099493	0.170621	0.096644	0.213938	0.203254
Novel_000390	XLOC_00428	transcript	0.093787	0	0.136283	0.176463	0.145812	0.280031	0.209698	0.295767	0.311126
Novel_000391	XLOC_00428	transcript	1.035188	1.145078	1.205187	0.996175	1.327006	1.300554	1.0464	1.250145	1.204821
Novel_000392	XLOC_00429	transcript	0	0.002791	0.011922	0	0.025623	0	0.002441	0	0
Novel_000393	XLOC_00429	transcript	0.824551	0.200539	0.210587	0.579017	0.640313	0.724219	0.746201	0.931441	0.465219
Novel_000394	XLOC_00429	transcript	0	1.200592	0.474341	0	0	0	0	0	0
Novel_000395	XLOC_00429	transcript	0.630679	0.166505	1.294994	1.190243	0.409963	0.737803	0.32889	0.28618	0.76699
Novel_000396	XLOC_00429	transcript	0.008492	0.135141	0	0.388503	0	0.25536	0.026717	0.004862	0
Novel_000397	XLOC_00429	transcript	0.088087	0.062946	0.271475	0.092416	0.086786	0.369124	0.340639	0.382986	0.151642
Novel_000398	XLOC_00429	transcript	0.262531	0.297445	0.290942	0.206804	0.214872	0.099993	0.156057	0.348609	0.185809
Novel_000399	XLOC_00430	transcript	0.283096	0.15523	0.311051	0.216964	0.247322	0.352438	0.236514	0.291956	0.340905
Novel_000400	XLOC_00433	transcript	0.310195	0.116961	0.237136	0.165389	0.245233	0.115126	0.499996	0.166533	0.086813
Novel_000401	XLOC_00434	transcript	0.210096	0	0	0	0	0.020865	0.355337	0.007079	0
Novel_000402	XLOC_00434	transcript	0.227594	0.478527	0.399819	0.283786	0.348102	0.303517	0.523963	0.561395	0.218975
Novel_000403	XLOC_00435	transcript	0.376311	0.507511	0.310245	0.15397	0.255234	0.610768	0.784105	0.419659	0.554086
Novel_000404	XLOC_00435	transcript	0.042665	0	0.271544	0.570847	0.496892	0	0.099797	0.06649	0
Novel_000405	XLOC_00435	transcript	0.052444	0.195449	0.249364	0.163273	0.286367	0.250199	0.265931	0.220331	0.163786
Novel_000406	XLOC_00436	transcript	0.172283	0.061649	0.452112	0	0.18976	0	0	0	0.204177

Novel_000407	XLOC_00437.	transcript	0.142387	0.401939	0.702601	0.57743	0.340184	0.514617	0.071072	0.839444	0.562549
Novel_000408	XLOC_00439.	transcript	0.118868	0.02828	0.277039	0.21651	0.095217	0.278683	0.102429	0	0.163536
Novel_000409	XLOC_00439.	transcript	0	0.173425	0	0.027426	0.203184	0.030882	0.003876	0.249323	0.042457
Novel_000410	XLOC_00440.	transcript	0.182837	0.182656	0.151154	0.126127	0.290693	0.19843	0.217844	0.168539	0.203525
Novel_000411	XLOC_00441.	transcript	0.203225	0.129047	0.179299	0.301153	0.247946	0.548196	0.400068	0.217705	0.243733
Novel_000412	XLOC_00441.	transcript	0.440555	0.571245	0.827223	0.591217	0.857411	0.609426	0.050694	0.457912	0.739449
Novel_000413	XLOC_00444.	transcript	0.119655	0.302265	0.061437	0.571644	0.028757	0.069973	0.147047	0.063656	0.071413
Novel_000414	XLOC_00444.	transcript	0.074254	0.214903	0.345494	0.33024	0.497091	0.117425	0.828345	0.555087	0.462761
Novel_000415	XLOC_00452.	transcript	2.190662	3.179763	1.233381	1.118772	1.280888	1.165405	2.348634	1.639574	2.256614
Novel_000416	XLOC_00452.	transcript	0.210975	0.253915	0.162514	0.204314	0.147009	0.177931	0.32619	0.257044	0.485696
Novel_000417	XLOC_00452.	transcript	0	0	0	0	0	0	0	0	0
Novel_000418	XLOC_00453.	transcript	0.363337	0.440805	0.851416	0.883489	0.923129	0.773746	0.752131	0.833441	0.680326
Novel_000419	XLOC_00453.	transcript	0.462571	0	0.048395	0.027675	0.00562	0.138787	0.021255	0.020957	0
Novel_000420	XLOC_00455.	transcript	0.456457	0.546391	0.656647	0.760073	0.703424	0.906752	0.912028	0.802032	0.636583
Novel_000421	XLOC_00456.	transcript	3.851264	2.851784	4.77841	5.050763	4.797493	5.174633	5.017872	4.984615	5.370156
Novel_000422	XLOC_00456.	transcript	0.111346	0	0.006626	0.026329	0.086833	0.08145	0.072814	0.0283	0.21167
Novel_000423	XLOC_00456.	transcript	1.041629	3.501593	0.392592	0.386306	0.621818	0.589873	1.406359	0.387643	2.157308
Novel_000424	XLOC_00456.	transcript	0	1.295581	0	0	0	0	0	0	0
Novel_000425	XLOC_00459.	transcript	0.188996	0	0.122302	0.003423	0.002537	0.143089	0.002098	0.003482	0.035984
Novel_000426	XLOC_00459.	transcript	0.543738	0	1.804174	0.777877	1.007745	2.496105	0.583998	1.026506	0.070031
Novel_000427	XLOC_00459.	transcript	0.588317	0	0.801745	0.010719	1.585629	0.786788	0.461953	0.01028	0.048
Novel_000428	XLOC_00459.	transcript	0	0.692971	0.000283	3.297912	0	0	0	0.974797	1.548596
Novel_000429	XLOC_00465.	transcript	0	0	0	0	0	0	0	0.036533	0
Novel_000430	XLOC_00465.	transcript	0	0.028272	0.110913	0.791239	0.481297	0.036461	0.070944	0.072069	0.046707
Novel_000431	XLOC_00465.	transcript	0.21734	0.032086	0.263705	0	0	0.318459	0.30841	0.30079	0.19996
Novel_000432	XLOC_00466.	transcript	0.847397	0.017357	0.002688	0	0.004338	0.748086	0.025807	0.002528	0
Novel_000433	XLOC_00466.	transcript	0	0.959268	0.273685	0	0.823471	0	0.741949	0.950255	0
Novel_000434	XLOC_00466.	transcript	0.003698	0.012216	0.174619	0.004088	0.006756	0	0.007963	0.000693	0.037274
Novel_000435	XLOC_00466.	transcript	0.002811	0.491868	0.267613	0.75717	0.009491	0.042768	0.586098	0.007632	0.585379
Novel_000436	XLOC_00466.	transcript	0.291291	0.257614	0.249338	0.258945	0.199125	0.05028	0.473817	0.221705	0.088724
Novel_000437	XLOC_00467.	transcript	0.054603	0.113137	0.115327	0.038136	0.035274	0.087621	0.115782	0.057173	0
Novel_000438	XLOC_00470.	transcript	0.116362	0.116284	0.19527	0.403916	0.170741	0.136021	0.144891	0.285763	0.242893
Novel_000439	XLOC_00470.	transcript	0	0.709674	0.064587	0.044181	0.125043	0.138882	0.093962	0.221639	0.164075
Novel_000440	XLOC_00477.	transcript	0.201931	0.114634	0.090973	0.026854	0.121024	0.032758	0.119517	0.126911	0.837375
Novel_000441	XLOC_00478.	transcript	0	0.175294	0	0	0.017197	0	0	0	0.228065
Novel_000442	XLOC_00479.	transcript	0.146268	0.092712	0.291879	0.070243	0.030227	0.291405	0.224004	0.136925	0.195203
Novel_000443	XLOC_00481.	transcript	0.51246	0.678819	0.368895	0.437728	0.110591	0.358559	0.337085	0.444532	0.332428
Novel_000444	XLOC_00481.	transcript	0	0	0.005712	0.199268	0.498782	0	0	0.275802	0.044758

Novel_000445	XLOC_00488.	transcript	0.352017	0.453768	0	0	0	0	0.100669	0.172914	0
Novel_000446	XLOC_00488.	transcript	0.340876	0.002199	0.042277	0.025641	0.10787	0.011281	0.285268	0.227051	0
Novel_000447	XLOC_00488.	transcript	0	0	0.003863	0	0	0.02303	0	0.069896	0
Novel_000448	XLOC_00488.	transcript	0	0.009877	0.346873	0.179217	0.091944	0.207526	0.03851	0.071626	0.232999
Novel_000449	XLOC_00492.	transcript	0	1.870856	1.687131	0.890766	0.006647	1.775677	0	1.811189	1.344809
Novel_000450	XLOC_00492.	transcript	2.841462	0.556512	0.473997	0.50652	0.633802	0.292723	0.624375	0.092718	0.050801
Novel_000451	XLOC_00492.	transcript	2.216862	2.223068	0.807111	0.828986	1.813692	0.655766	1.642271	1.123621	0.901432
Novel_000452	XLOC_00492.	transcript	0	0.579962	0	0.442951	1.894967	0.533446	1.608107	0.840672	0.306882
Novel_000453	XLOC_00492.	transcript	0.090115	0.095151	0	0.101449	0.038517	0.127464	0.590717	0.279201	0
Novel_000454	XLOC_00492.	transcript	0.034484	0	0.076056	0.024117	0.066221	0.01736	0.119873	0.068895	0.110835
Novel_000455	XLOC_00495.	transcript	0	0.226856	0	0	0.052969	0.001343	0.101342	0.067725	0.006119
Novel_000456	XLOC_00495.	transcript	0	0.173487	0	0.083456	0.081146	0.014311	0.236014	0.183197	0.015813
Novel_000457	XLOC_00495.	transcript	0.016993	0.141708	0	0	0	0.028453	0.3579	0.166978	0.071059
Novel_000458	XLOC_00495.	transcript	0.4193	0.171086	0.309987	0.617288	0.493476	0.29405	0	0.124023	0.240134
Novel_000459	XLOC_00497.	transcript	0.397367	0.020828	0.243385	0.174965	0	0.207776	0.381473	2.80E-05	0.08733
Novel_000460	XLOC_00497.	transcript	0	0	0.000103	0.470177	0.231819	0	0.000146	0.23688	0
Novel_000461	XLOC_00503.	transcript	0	0	0	0	0	0	0	0.04399	0
Novel_000462	XLOC_00504.	transcript	0.030889	0.119975	0.04361	0.00898	0.319655	0.013987	0.032719	0.028653	0.010075
Novel_000463	XLOC_00504.	transcript	0.58581	0.495576	0.53802	0.426102	0.236119	0.52167	0.676417	0.560844	0.644307
Novel_000464	XLOC_00506.	transcript	0	0.02586	0	0.019024	0	0	0.002944	0	0
Novel_000465	XLOC_00506.	transcript	0	0.032044	0	0	0	0	0	0	0
Novel_000466	XLOC_00506.	transcript	0.314684	0.834348	0.434412	0.210542	0.381855	0.313586	0.810273	0.521804	0.67684
Novel_000467	XLOC_00506.	transcript	0.197553	0.160439	0.022245	0.012356	0.181701	0.007999	0.023605	0.006024	0.012468
Novel_000468	XLOC_00512.	transcript	2.069996	0.468979	1.590712	1.793368	1.878188	1.665855	2.021303	1.154496	0.947872
Novel_000469	XLOC_00512.	transcript	0.264049	0.385113	0.255496	0.215425	0.235023	0.267377	0.34349	0.293889	0.179979
Novel_000470	XLOC_00514.	transcript	0.072583	0.083289	0.44068	0.614714	0.335584	0.055992	0.118572	0.113152	0.162774
Novel_000471	XLOC_00514.	transcript	0.256208	0.407055	0	0.260297	0.422899	0.39504	0.420362	0	0.358588
Novel_000472	XLOC_00514.	transcript	0.174723	0	0.087096	0	0	0	0	0.3449	0
Novel_000473	XLOC_00514.	transcript	0.784723	0.041966	0.34068	0.025117	0.033099	0.056836	0.581715	0.065933	0.003692
Novel_000474	XLOC_00514.	transcript	0.174954	0	0.097149	0	0	0	0.264316	0	0
Novel_000475	XLOC_00514.	transcript	0.797283	0.284258	0.146153	0.220381	0.382861	0.277941	0.687453	0.435981	0.125538
Novel_000476	XLOC_00514.	transcript	0.001992	0	0	0	0	0.146626	0	0.134352	0
Novel_000477	XLOC_00514.	transcript	0.196431	0.144893	0.216453	0.118163	0.12941	0.104227	0.306883	0.153534	0.183908
Novel_000478	XLOC_00514.	transcript	0.321229	0.087548	0.048153	0.109922	0.174117	0.121866	0.327853	0.144442	0
Novel_000479	XLOC_00514.	transcript	0.073582	0.017101	0.008167	0.00857	0.052409	0.019902	0.040996	0.010302	0.021613
Novel_000480	XLOC_00514.	transcript	0	0.275227	0.145806	0.092309	0.284706	0.310622	0.573518	0.532632	0.115624
Novel_000481	XLOC_00514.	transcript	0.704384	0.815467	0.372203	0.158389	0.358134	0.423903	0.662977	0.321836	0.265136
Novel_000482	XLOC_00514.	transcript	0	0.00303	0	0	0	0.37503	0	0	0

Novel_000483	XLOC_00514.	transcript	0.49688	0.377122	0.279073	0.469005	0.564275	0.285334	0.403958	0.454237	0.293932
Novel_000484	XLOC_00514.	transcript	0.114027	0.310175	0.204462	0.105534	0.163605	0.255095	0.173513	0.168363	0.281228
Novel_000485	XLOC_00515.	transcript	0.391888	0.67922	0.33751	0.203375	0.348232	0.418878	0.616744	0.319144	0.44522
Novel_000486	XLOC_00519.	transcript	0.066722	0	0.049107	0.0647	0.073693	0.047009	0.096741	0.121347	0.013033
Novel_000487	XLOC_00519.	transcript	0.024284	0	0	0.049879	0	0	0	0	0
Novel_000488	XLOC_00519.	transcript	0.120283	0	0.044698	0.051352	0.049738	0.049184	0.051304	0.059179	0.089118
Novel_000489	XLOC_00519.	transcript	0.106118	0.000423	0.058641	0.058547	0.049681	0.048399	0.063579	0.059005	0.158791
Novel_000490	XLOC_00519.	transcript	0.100555	0.08862	0.054197	0.061059	0.003737	0.013809	0.264634	0.01259	0.104723
Novel_000491	XLOC_00519.	transcript	0.022562	0.117575	0.072501	0.231926	0.002288	0	0.031092	0.003452	0
Novel_000492	XLOC_00519.	transcript	0.069217	0.028111	0.062637	0.075247	0	0.004551	0.018435	0.021853	0
Novel_000493	XLOC_00519.	transcript	0.014779	0.126628	0.103301	0	0.035287	0.097256	0.052099	0.083517	0.054782
Novel_000494	XLOC_00519.	transcript	0.05204	0	0.064313	0.000902	0.06208	0.058212	0.094007	0.07826	0
Novel_000495	XLOC_00519.	transcript	0.085659	0	0.160146	0	0.060154	0.056594	0.106206	0.112401	0.095303
Novel_000496	XLOC_00519.	transcript	0.064302	0.298417	0.049004	0.000663	0.082581	0.041906	0.095164	0.049804	0
Novel_000497	XLOC_00519.	transcript	0.114982	0.001751	0.11733	0.040157	0.073605	0.138327	0.076335	0.065711	0.14392
Novel_000498	XLOC_00519.	transcript	0.085481	0	0.050931	0	0.045955	0.05368	0.045926	0.039327	0.057661
Novel_000499	XLOC_00519.	transcript	0	0	0	0.00272	0	0	0	0	0
Novel_000500	XLOC_00519.	transcript	0.166551	0.269911	0.130913	0.115599	0.210481	0.138511	0.15759	0.115227	0.145659
Novel_000501	XLOC_00519.	transcript	0.202661	0.050434	0.019584	0.026322	0.172135	0.098496	0.137197	0.068001	0.054168
Novel_000502	XLOC_00522.	transcript	0.400841	0.448981	0.317945	0.418885	0.377653	0.388154	0.368198	0.435674	0.26335
Novel_000503	XLOC_00522.	transcript	0.007783	0	0.028057	0	0	0	0	0.004425	0
Novel_000504	XLOC_00522.	transcript	0.136183	1.032702	0.246669	0.368476	0.287789	0.657338	0.191225	0.642327	0.161972
Novel_000505	XLOC_00524.	transcript	0.171049	0.269018	0.240423	0.062916	0.189515	0.096414	0.288103	0.130519	0.165551
Novel_000506	XLOC_00525.	transcript	0	0.37661	0.431139	0.205428	0.551895	0.533264	0.622465	0.138745	0.174407
Novel_000507	XLOC_00525.	transcript	0.206153	0.234224	0.244482	0.217604	0.277567	0.210142	0.337298	0.289439	0.134643
Novel_000508	XLOC_00526.	transcript	0	0	0.137824	0	0.252842	0	0.252594	0.157811	0
Novel_000509	XLOC_00526.	transcript	0	0	0	0.09891	0	0	0	0	0
Novel_000510	XLOC_00526.	transcript	0.173396	0.365425	0	0.010378	0	0.08206	0.06676	0	0.196859
Novel_000511	XLOC_00529.	transcript	0.242818	0.231485	0.192665	0.132179	0.229005	0.2324	0.294986	0.112853	0.26031
Novel_000512	XLOC_00532.	transcript	0.197095	0.338842	0.194208	0.207461	0.273297	0.276	0.112195	0.20266	0.151691
Novel_000513	XLOC_00537.	transcript	0	0.20821	0	0.182829	0.204341	0	0	0	0.244843
Novel_000514	XLOC_00537.	transcript	0.123707	0.09455	0.342172	0.163552	0.055817	0.15355	0.485938	0.242344	0.849934
Novel_000515	XLOC_00537.	transcript	0	0	0.422839	0.867163	0.436404	0	1.455497	3.507124	0.448885
Novel_000516	XLOC_00537.	transcript	0.397041	0.16473	1.076218	0.027954	0.502735	1.103249	1.636429	0.109908	0.712539
Novel_000517	XLOC_00537.	transcript	0	0	0	1.127074	0	0	0	0	0
Novel_000518	XLOC_00537.	transcript	0.458273	0	0.370132	0.076876	1.300581	0	1.046022	0.352954	0
Novel_000519	XLOC_00540.	transcript	7.769097	5.244694	6.002945	5.949806	6.253717	5.03439	9.607873	7.110332	11.63489
Novel_000520	XLOC_00565.	transcript	0.223311	0.039171	0	0.087467	0.108028	0.108191	0	0.067009	0

Novel_000521	XLOC_00565.	transcript	0.097341	0.117752	0.133636	0.105069	0.110289	0.060386	0.240653	0.113351	0.077072
Novel_000522	XLOC_00565.	transcript	0	0	0	0.072115	0.179215	0	3.30E-05	0.116365	0
Novel_000523	XLOC_00565.	transcript	0.330636	0.098026	0.1894	0.175361	0.171389	0.270385	0.28469	0.145858	0.184828
Novel_000524	XLOC_00565.	transcript	0	0.140718	0	0	0	0	0	0	0
Novel_000525	XLOC_00565.	transcript	0.159537	0.083438	0.122942	0.166115	0.142002	0.208852	0.048547	0.109103	0
Novel_000526	XLOC_00565.	transcript	0.174061	0.104522	0.08473	0.159484	0.144635	0.156158	0.344623	0.138861	0
Novel_000527	XLOC_00565.	transcript	0.304703	0	0.12849	0.109561	0.125762	0.193084	0	0.152625	0
Novel_000528	XLOC_00565.	transcript	0.05939	0.203553	0.168088	0.241869	0.139953	0.318076	0.207351	0.243536	0.240488
Novel_000529	XLOC_00565.	transcript	0.299231	0.457175	0.257481	0.224564	0.255535	0.125105	0.42039	0.325076	0.356591
Novel_000530	XLOC_00566.	transcript	0.284139	0.159205	0.18569	0.187362	0.235027	0.229595	0.300928	0.203588	0.07295
Novel_000531	XLOC_00581.	transcript	0.330649	0.019271	0.173358	0.099985	0.120398	0.177974	0.305637	0.241219	0.064991
Novel_000532	XLOC_00581.	transcript	4.050606	0.093004	2.452161	1.831324	2.541738	2.641069	3.143446	2.180017	1.352915
Novel_000533	XLOC_00582.	transcript	2.169838	0.071911	1.334646	0.94187	1.400392	1.309141	1.912036	1.117744	0.738525
Novel_000534	XLOC_00582.	transcript	3.958593	0.140634	1.839557	1.052606	1.535583	1.718917	2.437491	1.378322	0.733137
Novel_000535	XLOC_00582.	transcript	0.765046	0.015347	0.470591	0.320082	0.361733	0.385054	0.532057	0.35989	0.225252
Novel_000536	XLOC_00582.	transcript	0.195845	0.37326	0.058212	0.093455	0.041047	0.040627	0.314759	0.093636	0.035702
Novel_000537	XLOC_00583.	transcript	0	0.266261	0	0	0.066558	0.06384	0.019786	0.053015	0.317535
Novel_000538	XLOC_00584.	transcript	0	0	0	0	0.051193	0	0	0.10492	0
Novel_000539	XLOC_00584.	transcript	0	0	0.023437	0.00718	0.067828	0.027815	0	0.057168	0.013682
Novel_000540	XLOC_00584.	transcript	0.005609	0.024451	0	0	0	0	0.021839	0.058611	0
Novel_000541	XLOC_00584.	transcript	0	0.000515	0.001095	0.083975	0	0.093412	0.00097	0.076555	0.10631
Novel_000542	XLOC_00584.	transcript	0	0	0.178152	0	0	0	0.489274	0	0
Novel_000543	XLOC_00584.	transcript	0.096067	0	0.223488	0.078632	0.113213	0.096317	0.195905	0.087767	0.091546
Novel_000544	XLOC_00584.	transcript	0.100767	0	0	0.232204	0.078675	0.063939	0	0.085358	0
Novel_000545	XLOC_00584.	transcript	0.09428	0	0	0.137966	0.086566	0.066095	0	0.188593	0
Novel_000546	XLOC_00584.	transcript	0.115706	0	0.034873	0.094816	0.109044	0.12858	0	0.12598	0.22078
Novel_000547	XLOC_00584.	transcript	0.074926	0	0	0.119957	0.052934	0.048242	0	0.142097	0
Novel_000548	XLOC_00584.	transcript	0	0	0	0	0	0	0	0	0
Novel_000549	XLOC_00584.	transcript	0.139807	0	0	0.190654	0.113421	0.166854	0	0.232771	0
Novel_000550	XLOC_00584.	transcript	0.000393	0.006839	0.001424	0.000775	0.203056	0	0.255401	0.096916	0.119677
Novel_000551	XLOC_00584.	transcript	0	0	0.082243	0	0	0	0.313685	0	0
Novel_000552	XLOC_00584.	transcript	0.281194	0	0	0.083534	0.071907	0.074991	0.132802	0.095806	0.042953
Novel_000553	XLOC_00584.	transcript	0.592124	0.25346	0.187615	0.112491	0.114495	0.179811	0.235574	0.163374	0.198797
Novel_000554	XLOC_00584.	transcript	0.032014	0.023532	0.044074	0.034016	0.088334	0.116582	0.051531	0.070954	0.04799
Novel_000555	XLOC_00584.	transcript	0	0	0.09446	0.168395	0.101851	0.003628	0.163738	0.142448	0.002148
Novel_000556	XLOC_00592.	transcript	0.37813	0	0.192675	0	0	0.016428	0.212702	0.317563	0
Novel_000557	XLOC_00592.	transcript	0.304992	0.037019	0.232254	0.701023	0.155173	0.552669	0.354644	0.277251	0.335263
Novel_000558	XLOC_00592.	transcript	0.309525	0	0.171958	0	0.374777	0	0.215791	0.246562	0

Novel_000559	XLOC_00592.	transcript	0.287915	0	0.191841	0	0.354727	0.02592	0.239221	0.250009	0
Novel_000560	XLOC_00616.	transcript	0.464298	0.397515	0.340737	0.476523	0.429693	0.435949	0.323993	0.298406	0.646665
Novel_000561	XLOC_00620.	transcript	0.812786	0.373285	0.431454	0.294966	0.459722	0.392748	0.728691	0.408559	0.235359
Novel_000562	XLOC_00620.	transcript	0.79095	0.693553	0.407464	0.396278	0.488784	0.751315	0.565851	0.503133	0.561974
Novel_000563	XLOC_00621.	transcript	0.037414	0.039923	0.028334	0.165111	0.109116	0.012934	0.089155	0.057492	0.02524
Novel_000564	XLOC_00630.	transcript	0.228608	0.129694	0.070605	0.273688	0.179189	0.20586	0.696182	0.242941	0.153064
Novel_000565	XLOC_00633.	transcript	0	0	0.191785	0.148294	0	0	0.066349	0.138921	0
Novel_000566	XLOC_00633.	transcript	0.158409	0.052856	0.008213	0.022292	0.133427	0.126834	0.213644	0.108978	0.293211
Novel_000567	XLOC_00633.	transcript	0	0	0	0	0	0	0	0	0
Novel_000568	XLOC_00634.	transcript	0	0	0	0	0	0	0	0	0
Novel_000569	XLOC_00634.	transcript	0.271452	0.334824	0.614361	0.324412	0.506436	0.840058	0.482224	0.537463	0.807287
Novel_000570	XLOC_00637.	transcript	0	0	0.176275	0	0	0	0	0.066068	0
Novel_000571	XLOC_00637.	transcript	0.33526	0.021167	0.229698	0.260078	0.291753	0.338473	0.231841	0.233264	0.106083
Novel_000572	XLOC_00641.	transcript	0	0.087562	0.25579	0	0	0	0.034237	0	0
Novel_000573	XLOC_00642.	transcript	0.051414	0.274086	0.060269	0.10596	0.128055	0	0.102921	0.134112	0
Novel_000574	XLOC_00642.	transcript	0.241837	0.428868	0.176101	0	0.228074	0	0.351652	0	0
Novel_000575	XLOC_00655.	transcript	0	0	0	0	0	0.002749	0	0	0
Novel_000576	XLOC_00655.	transcript	0.093961	0.106213	0.149271	0.054355	0.165983	0.04467	0.181662	0.140713	0.512857
Novel_000577	XLOC_00655.	transcript	0	0	0	0	0	0.010562	0	0	0
Novel_000578	XLOC_00655.	transcript	0	0	0	0.2018	0	0	0	0	0
Novel_000579	XLOC_00655.	transcript	0	0	0	0	0	0	0	0	0
Novel_000580	XLOC_00661.	transcript	0	0	0	0	0	0	0	0	0
Novel_000581	XLOC_00661.	transcript	0.098039	0	0	0	0	0	0	0	0
Novel_000582	XLOC_00661.	transcript	0.489204	0.215023	0.343829	0.465968	0.356911	0.561369	0.595314	0.608591	0.546113
Novel_000583	XLOC_00661.	transcript	0	0.633954	0.321321	0.219995	0.346886	0	0	0	0
Novel_000584	XLOC_00661.	transcript	0.20843	0.100632	0	0.009952	0.114289	0.17523	0.184833	0.30081	0.174406
Novel_000585	XLOC_00661.	transcript	0.181688	0.020812	0.432389	0.364415	0.210433	0.241048	0.173618	0.213921	0.008595
Novel_000586	XLOC_00662.	transcript	0	0.307449	0	0	0	0	0	0.820317	0
Novel_000587	XLOC_00662.	transcript	0	0.226719	0.122793	0.227865	0.177742	0	0.24268	0	0
Novel_000588	XLOC_00662.	transcript	0.565481	0.319849	0.543159	0.723347	0.557164	0.622212	0.364099	0.150283	0.411443
Novel_000589	XLOC_00662.	transcript	0.966264	0.900472	1.458358	1.114545	1.174375	1.12158	1.57445	1.806503	0.939345
Novel_000590	XLOC_00662.	transcript	0.332486	0.177007	0.178505	0.559508	0.435758	0.641808	0.178889	0.191423	0.347699
Novel_000591	XLOC_00665.	transcript	0.285087	0.559183	0.388432	0.629719	0.331423	0.74649	0.60769	0.672497	0.495824
Novel_000592	XLOC_00665.	transcript	0.113308	0.1539	0.214386	0.344016	0.12642	0.355549	0.444776	0.374574	0.296132
Novel_000593	XLOC_00665.	transcript	0.406432	0.169742	0.201386	0.094801	0.319764	0.34506	0.436564	0.172901	0.04371
Novel_000594	XLOC_00666.	transcript	0.12299	0.140945	0.254779	0.184496	0.112496	0.149674	0.135155	0.16682	0.080314
Novel_000595	XLOC_00666.	transcript	0.942654	0.012556	0.850287	0	0.497968	0	0.583768	0.399488	0.258593
Novel_000596	XLOC_00666.	transcript	0.133802	0	0.751648	0	0	0	0.213179	0.272971	0

Novel_000597	XLOC_00666.	transcript	0.245655	0	0.534058	0.178799	0	0.158775	0.221846	0.054997	0.047096
Novel_000598	XLOC_00666.	transcript	0.251179	0	0	0.310018	0	0.553336	0	0.033495	0
Novel_000599	XLOC_00666.	transcript	0.190723	0.005335	0	0.365917	0.237782	0.265934	0.190651	0.082125	0.217826
Novel_000600	XLOC_00666.	transcript	0.019094	0	0.010496	0	0.031741	0	0.167668	0	0.033482
Novel_000601	XLOC_00666.	transcript	0.242056	0.616941	0.362956	0.331599	0.388209	0.4013	0.372703	0.214395	0.263321
Novel_000602	XLOC_00672.	transcript	0.497152	0.488917	0.670221	0.420202	0.600432	0.178901	0.586914	0.569434	0.469633
Novel_000603	XLOC_00709.	transcript	0.630466	0.745373	0.536535	0.426951	0.351931	0.574016	0.721194	0.726996	0.367316
Novel_000604	XLOC_00709.	transcript	0.157378	0	0.144181	0.123068	0.333068	0.151711	0.130547	0.150888	0
Novel_000605	XLOC_00709.	transcript	0.536169	0.246543	0.136586	0.291166	0.257594	0.160399	0.182701	0.225919	0.517125
Novel_000606	XLOC_00709.	transcript	0	0	0	0	0	0	0	0	0
Novel_000607	XLOC_00709.	transcript	0	0	0	0	0	0.000229	0.210766	0.272186	0.013208
Novel_000608	XLOC_00711.	transcript	0	0	0	0	0	0	0	0	0
Novel_000609	XLOC_00711.	transcript	0	0	0	0	0	0	0	0	0
Novel_000610	XLOC_00711.	transcript	0.090119	0	0.057165	0.029155	0.032287	0.108731	0.093017	0.020639	0
Novel_000611	XLOC_00716.	transcript	0.073117	0	0.013998	0.107356	0.335701	0.510866	0.149696	0.241926	0.051599
Novel_000612	XLOC_00716.	transcript	0.3634	0.081655	0.588089	0.859456	0.67155	0.97736	0.536536	0.450675	0.200546
Novel_000613	XLOC_00716.	transcript	0	0.025398	0.06474	0.051624	0.007855	0.131895	0.01739	0.109335	0.051003
Novel_000614	XLOC_00717.	transcript	0.517455	0	0	0.085972	0.001189	0	0.127292	0	0.071876
Novel_000615	XLOC_00717.	transcript	0.098535	0.005882	0	0.105046	0	0	0.08447	0	0.004666
Novel_000616	XLOC_00717.	transcript	0.111669	0.175233	0.109046	0.100228	0.121294	0.104267	0.133462	0.081511	0.749951
Novel_000617	XLOC_00717.	transcript	0.041202	0.039644	0.032895	0	0.326776	0	0	0.339113	0
Novel_000618	XLOC_00717.	transcript	0.562643	0.324625	0.609469	0.658641	0.321789	0.889789	0.506812	0.41303	0.406388
Novel_000619	XLOC_00719.	transcript	0.069655	0.085031	0.098504	0.244408	0.45733	0.022376	0.103118	0.045248	0.17088
Novel_000620	XLOC_00719.	transcript	0.643834	0.379085	0.42703	0.357272	0.071554	0.493058	0.758983	0.465883	0.362295
Novel_000621	XLOC_00727.	transcript	0.440836	0.293222	0.289063	0.463209	0.295286	0.369153	0.287297	0.351668	0.55782
Novel_000622	XLOC_00727.	transcript	0	0.215104	0	0	0	0.055522	0	0	0
Novel_000623	XLOC_00727.	transcript	0	0	0	0	0	0	0	0	0.082054
Novel_000624	XLOC_00727.	transcript	0.03795	0.152147	0.13049	0	0.019922	0.023501	0.039331	0.011152	0.161052
Novel_000625	XLOC_00729.	transcript	0.086854	1.095146	0.093429	0.041395	0.109451	0.152713	0.224024	0.049643	0.165081
Novel_000626	XLOC_00752.	transcript	0.05506	0	0.082723	0.00809	0.05088	0.091617	0.100898	0.023769	0.170654
Novel_000627	XLOC_00767.	transcript	0.171292	0.000894	0.07671	0.040322	0.072099	0.095289	0.110636	0.039287	0.113728
Novel_000628	XLOC_00767.	transcript	0.177426	0.002141	0.118965	0.03901	0.094608	0.140275	0.092929	0.044713	0.126532
Novel_000629	XLOC_00769.	transcript	0.347746	0.003056	0.240512	0.096099	0.177517	0.142416	0.201175	0.113211	0.228635
Novel_000630	XLOC_00781.	transcript	0.169037	0.020125	0.223662	0.124736	0.102181	0.147352	0.221017	0.107396	0.13895
Novel_000631	XLOC_00782.	transcript	0	0.232825	0	0	0	0.017049	0.033577	0	0
Novel_000632	XLOC_00782.	transcript	0	0	0	0	0	0	0	0	0
Novel_000633	XLOC_00782.	transcript	0	0	0	0	0	0.005122	0.078282	0	0
Novel_000634	XLOC_00782.	transcript	0	0.179058	0.25617	0	0	0	0.00967	0	0

Novel_000635	XLOC_00784.	transcript	0.182168	0.235467	0.163839	0.05471	0.109822	0	0.146603	0.196342	0
Novel_000636	XLOC_00784.	transcript	0	0	0.155986	0	0	0.0468	0	0	0.01979
Novel_000637	XLOC_00787.	transcript	0.428867	0.804395	0.589018	0.555431	0.732587	0.620949	0.541971	0.628468	0.362405
Novel_000638	XLOC_00790.	transcript	0.044115	0.216137	0.102194	0.061769	0.039705	0.012396	0.1396	0.025426	0.220841
Novel_000639	XLOC_00791.	transcript	0.004724	0	0	0	0	0.171459	0.324101	0	0.001678
Novel_000640	XLOC_00791.	transcript	0.206754	0.005986	0.087506	0.08877	0.151358	0.0543	0.108479	0.092895	0.088779
Novel_000641	XLOC_00791.	transcript	0.685561	0	0.096741	0.245413	0.386217	0.049196	0.604834	0.334745	0.1936
Novel_000642	XLOC_00795.	transcript	0.318708	1.292877	0.279937	0.199663	0.228264	0.273942	0.288217	0.264262	0.320683
Novel_000643	XLOC_00796.	transcript	0.305504	0	0	0	0	0.491924	0	0	0
Novel_000644	XLOC_00796.	transcript	0.363896	0	0	0	0	0.740451	0	0	0
Novel_000645	XLOC_00796.	transcript	0	0.002074	0	0.475809	0	0	0	0	0.154236
Novel_000646	XLOC_00796.	transcript	0.210376	0	0	0.617847	1.942948	0.186366	0	0.229509	2.108186
Novel_000647	XLOC_00796.	transcript	0.348405	2.595404	1.787668	1.054322	0.328229	0.398057	1.469141	2.005447	0
Novel_000648	XLOC_00798.	transcript	0	0	0.081445	0.03121	0.007658	0.005836	0.000194	0.190207	0
Novel_000649	XLOC_00798.	transcript	0.105283	0.014099	0.31347	0.044897	0.328763	0.210125	0.353964	0.075315	0.23765
Novel_000650	XLOC_00798.	transcript	0.015041	0	0.398118	0.015034	0.653924	0.331966	0.360481	0.344079	0.024591
Novel_000651	XLOC_00798.	transcript	0	0	0.234935	0	0	0	0	0.27298	0.749148
Novel_000652	XLOC_00798.	transcript	0.193144	0	0.198148	0.219334	0.176701	0.159424	0.120682	0.115347	0.203775
Novel_000653	XLOC_00798.	transcript	0.451159	0	0.34563	0.431144	0.652843	0.517067	0.487707	0.610261	0.560335
Novel_000654	XLOC_00798.	transcript	0.96955	0.411041	0.756376	1.510962	0.502354	0.754379	0.964064	0.756674	1.075602
Novel_000655	XLOC_00798.	transcript	0.394937	0.174693	0.215965	0.442292	0.33408	0.156607	0.232033	0.41591	0.228676
Novel_000656	XLOC_00798.	transcript	0.161754	0	0.874395	0.100176	1.23561	0.375819	0.417327	0.441988	0
Novel_000657	XLOC_00799.	transcript	0.016555	0.056596	0	0	0	0	0.789852	0.0116	0.013258
Novel_000658	XLOC_00799.	transcript	0	0.001423	0.000165	0.000802	0.006567	0.007279	0.049866	0.010843	0.005497
Novel_000659	XLOC_00799.	transcript	0.03727	0.193729	0.008999	0.008446	0.016344	0.01149	0.17645	0.007784	0.01624
Novel_000660	XLOC_00799.	transcript	0.396422	0.017832	0.217614	0.331299	0.27087	0.326937	0	0.379695	0.335335
Novel_000661	XLOC_00799.	transcript	0	0	0	0	0	0	0.098426	0	0
Novel_000662	XLOC_00799.	transcript	0	0.013794	0	0.013178	0.014942	0	0.032011	0.000205	0.032662
Novel_000663	XLOC_00799.	transcript	0	0.017603	0.14198	0.022172	0.010497	0	0.017014	0.004719	0
Novel_000664	XLOC_00799.	transcript	0	0	0	0	0.023278	0	0	0	0
Novel_000665	XLOC_00799.	transcript	0	0.004674	0	0	0	0.038989	0	0.065203	0
Novel_000666	XLOC_00799.	transcript	0	0.002601	0.076867	0.047741	0.024209	0.009524	0.042268	0.000588	0
Novel_000667	XLOC_00803.	transcript	0.230132	0.141794	0.189774	0.139031	0.245216	0.177651	0.169764	0.213158	0.073668
Novel_000668	XLOC_00807.	transcript	0.450465	0.363913	0.233934	0.114492	0.134977	0.180589	0.45507	0.205393	0.082114
Novel_000669	XLOC_00807.	transcript	0.231945	0.459857	0.257422	0.423249	0.458512	0.311301	0.287488	0.191713	0.244042
Novel_000670	XLOC_00807.	transcript	0.114858	0	0	0	0	0.087068	0.042366	0.213179	0
Novel_000671	XLOC_00811.	transcript	0.399592	0.318705	0.296675	0.378076	0.242128	0.314133	0.259734	0.451695	0.233665
Novel_000672	XLOC_00811.	transcript	0.183012	0.512921	0.029767	0.047195	0.057594	0.156916	0.125782	0.01209	0.161281

Novel_000673	XLOC_00812.	transcript	0.020745	0.181256	0.070566	0.27097	0.216873	0.309679	0.205206	0.228394	0.350352
Novel_000674	XLOC_00812.	transcript	0.744856	1.013511	0.606374	0.614555	0.44973	0.341894	0.813163	0.57962	0.31882
Novel_000675	XLOC_0081€.	transcript	1.515898	1.302691	1.199198	1.083255	0.713265	1.135397	1.292686	1.362759	1.095803
Novel_000676	XLOC_00817.	transcript	0.381359	0.000477	0.661365	0	0	0.001809	0.236109	0.352437	0.653392
Novel_000677	XLOC_00817.	transcript	0.505418	1.051999	0.144549	0.832319	0.713926	1.037804	0.719399	0.57807	0
Novel_000678	XLOC_00817.	transcript	0.208622	0.348516	0.349161	0.404936	0.613802	0.426757	0.194338	0.136556	0.177667
Novel_000679	XLOC_00817.	transcript	0	0.005681	0.032642	0.02485	0.008307	0	0.052402	0.113321	0
Novel_000680	XLOC_00822.	transcript	0	0.045918	0.149964	0.167527	0.155438	0.096181	0.146794	0.110409	0.119723
Novel_000681	XLOC_00822.	transcript	0.194818	0.008129	0	0.002537	0.024686	0	0.058658	0.006355	0.035409
Novel_000682	XLOC_00822.	transcript	0.268356	0.635228	0.404041	0.399793	0.33901	0.323622	0.290121	0.358211	0.785164
Novel_000683	XLOC_00822.	transcript	0	0	0	0	0	0	0	0	0.011133
Novel_000684	XLOC_0082⁹.	transcript	0.166828	0.204865	0.317614	0.262605	0.21696	0.220446	0.210697	0.35302	0.297527
Novel_000685	XLOC_00837.	transcript	0.036522	0	0	0	0	0	0	0	0.410757
Novel_000686	XLOC_0085€.	transcript	0.299265	0.261578	0.088765	0.173762	0.168426	0.283511	0.266737	0.121605	0.150825
Novel_000687	XLOC_0085€.	transcript	0.608202	1.122387	0.704723	0.682768	0.311664	1.005305	0.472909	0.807145	0.540259
Novel_000688	XLOC_0085ε.	transcript	0.613773	0.701415	0.306115	0.137582	0.334266	0.272653	0.277388	0.416554	0.11337
Novel_000689	XLOC_0085ε.	transcript	0.305828	0.249706	0.440858	0.489865	0.486368	0.669844	0.323878	0.740197	0.269593
Novel_000690	XLOC_00861.	transcript	0.626022	0.075836	0.473442	0.611358	0.392684	0.645023	0.619169	0.682625	0.193552
Novel_000691	XLOC_0086ε.	transcript	0.332497	0.288072	0.602638	0.672984	0.77592	0.479732	0.814778	0.647797	0.632588
Novel_000692	XLOC_0086⁹.	transcript	0.083816	0	0.318139	0.146347	0.069508	0.070769	0	0.049988	0.103299
Novel_000693	XLOC_0086⁹.	transcript	0.144528	0.050717	0.298747	0.066415	0.217558	0.255794	0.301151	0.206103	0.141124
Novel_000694	XLOC_0086⁹.	transcript	0	0.006678	0	0.077628	0	0	0	0	0
Novel_000695	XLOC_0087C.	transcript	0.243718	0	0	0	2.840762	4.630281	0.623121	0.010533	0
Novel_000696	XLOC_0087C.	transcript	1.10831	2.23575	0	0.181387	0	0.125413	0.200955	0	0
Novel_000697	XLOC_0087C.	transcript	0.503337	0.39831	0.747082	0.5519	0	0.027775	1.42368	1.236898	0.042462
Novel_000698	XLOC_0087C.	transcript	0.062578	0.189549	0.226345	0.042013	0.564935	0.634058	0	0.026579	0.45213
Novel_000699	XLOC_00871.	transcript	0.103441	2.78795	0.090805	0.046064	0.126817	0.031035	0.099927	0.065029	0.259606
Novel_000700	XLOC_00872.	transcript	0	0	0	0	0	0.269825	0	0	0
Novel_000701	XLOC_00872.	transcript	0.282008	0	0	0.809791	0.614925	0.539427	0.254632	0	0
Novel_000702	XLOC_00872.	transcript	0	0	0.166028	0	0	0.572029	0.533941	0.20486	0.210957
Novel_000703	XLOC_00872.	transcript	0.840013	0.435088	0.474798	0.464938	0.429117	0	0.431137	0.650359	0.291542
Novel_000704	XLOC_00872.	transcript	0.405224	0.379625	0.501141	0.477205	0.56069	0.566841	0.330054	0.43024	0.667261
Novel_000705	XLOC_00872.	transcript	0	0	0	0	0	0	0.003206	0.000194	0
Novel_000706	XLOC_0088ε.	transcript	0.252598	0.051575	0.073403	0.075525	0.031823	0.049996	0.20876	0.033592	0
Novel_000707	XLOC_0090ε.	transcript	0.750565	0.103696	0.369158	0.215309	0.259304	0.284478	0.487145	0.2627	0.318452
Novel_000708	XLOC_0090ε.	transcript	0.319401	0.30737	0.33031	0.450913	0.398906	0.36656	0.44675	0.636465	0.278152
Novel_000709	XLOC_0090ε.	transcript	0.431837	0.098625	0.387851	0.341174	0.356892	0.365628	0.645871	0.351356	0.479428
Novel_000710	XLOC_00927.	transcript	0	0	0.064049	0.007479	0	0.277328	0	0	0

Novel_000711	XLOC_00927.	transcript	0.021105	0	0.696447	0.633338	0	0.666545	1.115384	0.489924	0.670257
Novel_000712	XLOC_00927.	transcript	0	0	0	0	0.371116	0	0	0	0
Novel_000713	XLOC_00927.	transcript	0	0.467016	0	0	0.556462	0.025971	0.009291	0.020746	0.016846
Novel_000714	XLOC_00927.	transcript	0.010162	0	0	0	0	0	0	1.384266	0
Novel_000715	XLOC_00927.	transcript	0.818043	0.687969	0.120767	0.088573	0	0	0	0.015473	0.317034
Novel_000716	XLOC_00927.	transcript	0.094454	0.34315	0	0	0	0	0.000845	0	0
Novel_000717	XLOC_00927.	transcript	0.058872	0.036387	0.019158	0.002933	0.083956	0.157722	0.101228	0	0.061223
Novel_000718	XLOC_00942.	transcript	0.04453	0.169309	0.058768	0.093301	0.090936	0.214905	0	0.089233	0
Novel_000719	XLOC_00943.	transcript	0.901532	1.213592	0.504893	0.192067	0.22688	0.491955	0.687995	0.326736	0.262622
Novel_000720	XLOC_00944.	transcript	0.190806	0.060684	0.021798	0.108518	0.044246	0	0.070837	0.114414	0.33726
Novel_000721	XLOC_00946.	transcript	0.26598	0.376722	0.27075	0.284438	0.457318	0.291716	0.328665	0.29123	0.413924
Novel_000722	XLOC_00946.	transcript	0.007853	0.000724	0	0.063416	0.003328	0	0.141596	0.001205	0
Novel_000723	XLOC_00946.	transcript	0.235684	2.695144	0.090435	0	0.118019	0.141919	0	0.070127	0.038354
Novel_000724	XLOC_00948.	transcript	0.485437	0.667523	0.211616	0.161497	0.198157	0.304362	0.343084	0.224303	0.193947
Novel_000725	XLOC_00950.	transcript	0	0	0	0	0	0	0	0.052573	0
Novel_000726	XLOC_00950.	transcript	0.257147	0.033708	0.114123	0.110059	0.109131	0.096761	0.085024	0.023598	0.063692
Novel_000727	XLOC_00950.	transcript	0.084903	0.012507	0.030291	0	0	0	0.020269	0.07169	0.025324
Novel_000728	XLOC_00950.	transcript	0.031381	0	0.01089	0.081968	0.044607	0	0.456279	0.012621	0
Novel_000729	XLOC_00954.	transcript	1.908904	0.079408	0.738824	1.026126	0.726267	1.257534	0.880725	0.68725	1.086979
Novel_000730	XLOC_00954.	transcript	3.578329	1.488963	4.055252	3.178421	3.570664	2.427311	5.474757	4.853605	1.978702
Novel_000731	XLOC_00960.	transcript	0.164289	0.031025	0.331797	0.165749	0.153192	0.070717	0.430359	0.160001	0.143925
Novel_000732	XLOC_00973.	transcript	0.474758	0.709204	0.525651	0.660616	0.642134	0.689978	0.67306	0.619789	0.735104
Novel_000733	XLOC_00974.	transcript	0.345768	0.39442	0.208463	0.365909	0.240196	0.235799	0.195451	0.364995	0.310558
Novel_000734	XLOC_00984.	transcript	0.088	0	0.046517	0.178938	0.047103	0.053087	0.197224	0.264349	0.104291
Novel_000735	XLOC_00988.	transcript	0.064666	0.095464	0.047764	0	0	0	0.042537	0.092014	0.040873
Novel_000736	XLOC_00988.	transcript	0.032385	0.092699	0.01053	0.010257	0.050938	0.048071	0.064877	0.024541	0.089233
Novel_000737	XLOC_00988.	transcript	0.042428	0.08482	0.037035	0.016644	0.040671	0.030116	0.034241	0.044861	0.062874
Novel_000738	XLOC_00994.	transcript	0	0	0	0.158695	0.350033	0	0	0	0.05478
Novel_000739	XLOC_00994.	transcript	0.2856	0.510524	0.600017	0.074596	0.025148	0.352344	0.454509	0.108014	0.414287
Novel_000740	XLOC_00997.	transcript	0.386068	0.640139	0.422136	0.708652	0.400786	0.446733	0.451058	0.654635	0.355297
Novel_000741	XLOC_01004.	transcript	0	0.170171	0	0.01912	0	0	0	0	0
Novel_000742	XLOC_01004.	transcript	0	0.053087	0	0.053684	0	0	0.063555	0	0
Novel_000743	XLOC_01013.	transcript	0.465633	0.826223	0.022095	0.109595	0.2326	0.309557	0.109209	0.155648	0.28628
Novel_000744	XLOC_01013.	transcript	0.683657	0.241464	0.13203	0.163659	0.373138	0.177296	0.287732	0.154947	0.264197
Novel_000745	XLOC_01013.	transcript	0.452562	0.416728	0.209404	0.101145	0.286343	0.224739	0.218764	0.163635	0.145526
Novel_000746	XLOC_01014.	transcript	0.294908	0.204958	0.13759	0.181011	0.160367	0.296456	0.15474	0.226963	0.068855
Novel_000747	XLOC_01016.	transcript	0	0	0.718621	0	0.87892	0.75405	1.602689	0	0.60444
Novel_000748	XLOC_01016.	transcript	0.936918	0.792751	0.691747	0.651145	0	0	0	1.068111	0

Novel_000749	XLOC_01021.	transcript	1.561804	8.740459	3.115114	3.030542	2.2447	2.446318	2.318893	2.986989	2.623161
Novel_000750	XLOC_01025.	transcript	0.093144	0.00512	0.17874	0.125096	0.328143	0	0.122776	0.134886	0.008888
Novel_000751	XLOC_01025.	transcript	0.326977	0.227511	0.307082	0.45243	0.253639	0.631029	0.435536	0.222962	0.444441
Novel_000752	XLOC_01025.	transcript	0.354035	0	0	0	0	0	0	0	0
Novel_000753	XLOC_01025.	transcript	0.654579	0.228287	0.770302	0.903324	0.808664	0.992961	1.363704	0.966776	0.934269
Novel_000754	XLOC_01025.	transcript	0.147404	0.130967	0.270988	0.089684	0.191102	0.220173	0.270245	0.368908	0.286977
Novel_000755	XLOC_01026.	transcript	0	0.017396	0.047714	0.011127	0	0.678508	0.020727	0	0.056705
Novel_000756	XLOC_01026.	transcript	1.943501	0.24598	2.138849	1.827091	1.935176	2.363024	1.738457	1.917358	3.491278
Novel_000757	XLOC_01027.	transcript	0	0.062549	0.248797	0	0.11777	0	0	0.100371	0.176067
Novel_000758	XLOC_01027.	transcript	0.190839	0	0.0776	0.146936	0	0.173858	0.122312	0	0
Novel_000759	XLOC_01028.	transcript	0.12247	0.421582	0.109667	0.237064	0.575958	0.442531	0.090433	0.387212	0.107865
Novel_000760	XLOC_01028.	transcript	0.469985	0.349717	0.440682	0.471712	0.016798	0.356383	0.520629	0.55402	0.299476
Novel_000761	XLOC_01029.	transcript	0.290285	0	0	0.218995	0.122971	0.220274	0.108641	0.153414	0.028709
Novel_000762	XLOC_01033.	transcript	0.085465	0	0.218288	0.101075	0	0.024886	0.177934	0.017839	0.080051
Novel_000763	XLOC_01035.	transcript	0.026757	0	0	0.176064	0	0.030549	0	0	0
Novel_000764	XLOC_01035.	transcript	0	0	0	0.340715	0	0	0	0.327643	0
Novel_000765	XLOC_01035.	transcript	0.120528	0.152298	0.685907	0.097552	0.094985	0.576223	0.482191	0.164779	0.255769
Novel_000766	XLOC_01035.	transcript	0.293771	0.207706	0.212177	0.203407	0.24012	0.412127	0.342575	0.310383	0.273116
Novel_000767	XLOC_01039.	transcript	0.176909	0	0.306902	0.059563	0.003477	0.049075	0.013118	0.055815	0.042232
Novel_000768	XLOC_01039.	transcript	0.231636	0	0.00147	0.204095	0.03543	0.058554	0.066051	0.367867	0
Novel_000769	XLOC_01039.	transcript	0.417813	0.176297	0.421615	0.42464	0.640698	0.305636	0.448655	0.124479	0.451383
Novel_000770	XLOC_01039.	transcript	0.000138	0	0.143247	0.018256	0.007722	0	0.001045	0	0.277741
Novel_000771	XLOC_01039.	transcript	0.000383	0	0	0.010634	0	0.124407	0.007886	0	0
Novel_000772	XLOC_01039.	transcript	0	0	0	0.02147	0.036869	0.009105	0	0	0
Novel_000773	XLOC_01039.	transcript	0	0	0	0	0	0	0	0	0
Novel_000774	XLOC_01039.	transcript	0	0	0	0	0	0	0.038444	0.009056	0.017059
Novel_000775	XLOC_01039.	transcript	0.004328	0	0.012746	0	0.001431	0.002589	0.000698	0.001428	0
Novel_000776	XLOC_01039.	transcript	0.372285	0	0	0.172433	0.126542	0.30437	0.166145	0.581875	0
Novel_000777	XLOC_01048.	transcript	0.574526	0.577975	0.380043	0.133546	0.249898	0.240489	0.405165	0.263425	0.388352
Novel_000778	XLOC_01053.	transcript	2.216069	0.173696	0.830292	0.165371	0.077164	0.08666	0.183883	0.876889	0.629783
Novel_000779	XLOC_01054.	transcript	0.265343	0.390337	0.286004	0.215878	0.194602	0	0.40199	0.383591	0.167358
Novel_000780	XLOC_01054.	transcript	0.048037	0.042153	0.106462	0.111626	0.271527	0	0.200925	0.159505	0.187129
Novel_000781	XLOC_01054.	transcript	0.059237	0.209669	0	0	0	0	0	0.077896	0
Novel_000782	XLOC_01054.	transcript	0	0	0	0.027244	0.004387	0.240376	0.006272	0	0.011521
Novel_000783	XLOC_01054.	transcript	0.200252	0.040434	0.040134	0.010868	0.017122	0	0	0	0.038675
Novel_000784	XLOC_01054.	transcript	0.43018	0.321211	0.098667	0.265994	0.227697	0.351546	0.440664	0.452529	0.327518
Novel_000785	XLOC_01054.	transcript	0	0	0	0	0	0	0	0	0
Novel_000786	XLOC_01057.	transcript	0.175884	0	0.308517	0.110558	0.133345	0.113057	0.217593	0.012504	0.077472

Novel_000787	XLOC_01061.	transcript	0.155588	0.193353	0.018283	0.065127	0	0.135	0.103041	0.059145	0.204954
Novel_000788	XLOC_01065.	transcript	0.067679	0.211542	0.054191	0.000552	0	0.13555	0.309268	0.120503	0.00266
Novel_000789	XLOC_01065.	transcript	0.002889	0.16886	0.091374	0.146716	0.019722	0.062015	0.097571	0.063435	0.07912
Novel_000790	XLOC_01065.	transcript	0.047627	0.0041	0.066879	0.034767	0	0.021314	0.040175	0.025608	0.0227
Novel_000791	XLOC_01065.	transcript	0.13438	0.07381	0.074072	0.104249	0.136861	0.0773	0.117718	0.058301	0.085097
Novel_000792	XLOC_01065.	transcript	0.332053	0.004704	0.019891	0.016201	0.13215	0.044635	0.092149	0.044918	0.032298
Novel_000793	XLOC_01065.	transcript	0.032892	0.009994	0.020929	0.045088	0	0.011943	0.058337	0.011246	0.046845
Novel_000794	XLOC_01065.	transcript	0.516971	0.008896	0.192576	0.205358	0.226649	0.192287	0.361944	0.175796	0.160643
Novel_000795	XLOC_01065.	transcript	0.005142	0	0.009267	0.008759	0	0.246227	0.003533	0.010914	0.00535
Novel_000796	XLOC_01071.	transcript	0.060059	0	0.041932	0.238073	0.188412	0.121191	0.078253	0.142398	0.022791
Novel_000797	XLOC_01077.	transcript	0.26315	0	0.458556	0.48464	0.39232	0.306458	0.715797	0.629056	0.236995
Novel_000798	XLOC_01077.	transcript	0.031235	0.092051	0.161335	0.029751	0.180698	0.018114	0	0.178874	0
Novel_000799	XLOC_01078.	transcript	0	0.004134	0	0	0	0	0	0	0
Novel_000800	XLOC_01078.	transcript	0.763905	0.365631	1.017346	0.425092	0.49026	0.934616	0.95255	0.225147	0
Novel_000801	XLOC_01078.	transcript	0.146577	0.128357	0	0.057812	0.136726	0.237328	0	0	0.634687
Novel_000802	XLOC_01078.	transcript	0	0	0	0	0	0	0.061333	0.501711	0
Novel_000803	XLOC_01078.	transcript	0	0	0	0	0.008964	0.025247	0	0	0
Novel_000804	XLOC_01078.	transcript	0	0	0	0	0	0	0.086232	0.014271	0
Novel_000805	XLOC_01078.	transcript	0.167542	0.219091	0.109765	0.052472	0.125546	0.068566	0.17582	0.146909	0.100775
Novel_000806	XLOC_01078.	transcript	0	0	0.371628	0.35162	0.154074	0.078439	0.334726	0.536287	0.12413
Novel_000807	XLOC_01078.	transcript	0.596711	0.438413	0.031238	0.081001	0.188008	0.392884	0.059571	0.133897	0.823507
Novel_000808	XLOC_01078.	transcript	0.20793	0.006236	0.199133	0.361588	0.439003	0.183885	0.241785	0.263249	0.246727
Novel_000809	XLOC_01078.	transcript	0	0	0	0.016267	0	0	0	0	0
Novel_000810	XLOC_01078.	transcript	0	0	0	0	0	0	0	0	0
Novel_000811	XLOC_01078.	transcript	0	0	0	0	0	0	0	0	0
Novel_000812	XLOC_01083.	transcript	0	0.073238	0	0.024645	0	0.019915	0	0	0
Novel_000813	XLOC_01083.	transcript	0.029293	0.034002	0.090732	0.042288	0.073924	0.280606	0.633194	0.029213	0.098935
Novel_000814	XLOC_01083.	transcript	0.878778	0.592758	0.67353	0.814139	0.756308	0.779318	0.745655	0.762959	0.550538
Novel_000815	XLOC_01086.	transcript	0.338643	0	0.883476	0.878211	0.464236	0.551714	0.544762	0.81363	0.479331
Novel_000816	XLOC_01086.	transcript	0.865709	0.150506	0.58782	0.798918	0.859167	1.464161	0.783567	0.630954	0.530092
Novel_000817	XLOC_01086.	transcript	0.008658	0.233041	0	0.007421	0.245574	0.005503	0.009293	0.00448	0.142512
Novel_000818	XLOC_01088.	transcript	0.004492	0	0	0	0.002023	0.221396	0.142741	0.306201	0
Novel_000819	XLOC_01088.	transcript	0	0	0	0	0.405914	0.768336	0	0	0
Novel_000820	XLOC_01088.	transcript	0	0	0	0	0.317695	0.325989	0	0	0
Novel_000821	XLOC_01088.	transcript	0	0	0	0.990584	3.90E-05	0	0	0	0
Novel_000822	XLOC_01088.	transcript	0	0	0.019773	0.477446	0	0.474269	0	0.985111	0.046212
Novel_000823	XLOC_01088.	transcript	0.63127	0	0	0.46876	0	0	0	0.445433	0
Novel_000824	XLOC_01088.	transcript	0.044729	0	0.020982	0	0.080842	0.798439	0.050185	0.012969	0.009187

Novel_000825	XLOC_01088.	transcript	0.467512	0.317803	1.701798	0.688295	0.77606	0.56124	1.121113	0.500446	1.332562
Novel_000826	XLOC_01089.	transcript	0.064559	0.105229	0.099517	0.078886	0.077063	0.093885	0.177354	0.089404	0.104855
Novel_000827	XLOC_01090.	transcript	0.007048	0	0	0	0	0	0.204418	0.106263	0
Novel_000828	XLOC_01090.	transcript	0.237613	0.490623	0.07165	0.237449	0.259558	0.198838	0.148394	0.269911	0.146631
Novel_000829	XLOC_01090.	transcript	0.043326	0	0.141621	0	0.061597	0.121329	0.020258	0	0
Novel_000830	XLOC_01090.	transcript	2.954403	2.025208	1.802991	2.184128	2.011854	2.045853	2.048714	2.413757	2.078533
Novel_000831	XLOC_01092.	transcript	0.003265	0	0.025907	0	0	0	0	0.001012	0
Novel_000832	XLOC_01092.	transcript	0	0	0.019257	0	0	0	0	0.393007	0
Novel_000833	XLOC_01092.	transcript	0	0	0	0	0	0	0	0	0
Novel_000834	XLOC_01092.	transcript	0.349437	0.245115	0.619376	0.472006	0.575573	0.404848	1.2169	0.784495	0.605525
Novel_000835	XLOC_01092.	transcript	0.267964	0.197795	0.195368	0.110093	0.182259	0.106738	0.151075	0.177879	0.230187
Novel_000836	XLOC_01092.	transcript	0	0.018981	0	0	0	0	0.290575	0.184398	0.023971
Novel_000837	XLOC_01093.	transcript	0.065634	0.002799	0.157795	0.611888	0.172864	0.049851	0.278202	0.247775	0.027947
Novel_000838	XLOC_01093.	transcript	0.13626	0.245163	0	0.3796	0.406828	0.672159	0.082254	0	0.435627
Novel_000839	XLOC_01093.	transcript	0	0	0	0	0	0	0	0	0
Novel_000840	XLOC_01093.	transcript	3.819931	1.328798	4.319479	3.86384	2.445307	4.442503	3.525752	3.34021	4.38417
Novel_000841	XLOC_01095.	transcript	0.438891	0.348164	0.287597	0.151768	0.285655	0.322633	0.445673	0.220142	0.165886
Novel_000842	XLOC_01097.	transcript	0.008152	0	0	0.509531	0.005708	0.002626	0.633272	0	0
Novel_000843	XLOC_01097.	transcript	1.099118	0.314281	0.711017	0.223285	1.410341	0.704169	0.355421	1.240203	0.992808
Novel_000844	XLOC_01100.	transcript	0.176276	0.037226	0.070303	0.061644	0.058074	0.037944	0.054226	0.088263	0.104498
Novel_000845	XLOC_01100.	transcript	0.053897	1.384613	0.222261	0.167237	0	0.226915	0.386695	0.108804	0.710801
Novel_000846	XLOC_01100.	transcript	0	0.180456	0	0.08925	0	0	0	0	0.556388
Novel_000847	XLOC_01100.	transcript	0.056718	0.153239	0.22239	0.342032	0.065429	0.126118	0.027375	0.025709	0.529811
Novel_000848	XLOC_01100.	transcript	0	0	0.365102	0	0	0	0.359025	0	0
Novel_000849	XLOC_01100.	transcript	1.154724	0.297513	0	0.330488	0.020323	0	0.890474	0	0.269049
Novel_000850	XLOC_01100.	transcript	0.003527	0.422817	0.017201	2.50E-05	1.284933	0.669227	0.382975	1.224707	0
Novel_000851	XLOC_01103.	transcript	0.1763	0.615149	0.18118	0	0.192314	0.064818	0.146202	0.061518	0.018631
Novel_000852	XLOC_01104.	transcript	0.114955	0.373038	0.041972	0.097468	0.086711	0.092887	0.209166	0.126624	0.101155
Novel_000853	XLOC_01105.	transcript	0.096446	0.173897	0.078131	0.28691	0.031906	0.085172	0.092156	0.093781	0
Novel_000854	XLOC_01105.	transcript	0	0	0.159358	0	0	0.00932	0	0.429946	0
Novel_000855	XLOC_01105.	transcript	0.298812	0.138248	0.135402	0.339607	0.130056	0.069505	0.378573	0	0.037971
Novel_000856	XLOC_01105.	transcript	0	0	0	0	0	0	0	0	0
Novel_000857	XLOC_01105.	transcript	0	0.008453	0.049918	0.058236	0.028529	0.027325	0.150398	0.004307	0.146351
Novel_000858	XLOC_01107.	transcript	0.107085	0.307682	0.068086	0.178403	0.211272	0.143456	0.21458	0.164306	0.166128
Novel_000859	XLOC_01107.	transcript	0.003074	0.215571	0.134709	0.000958	0.079674	0.001581	0.33497	0	0.000641
Novel_000860	XLOC_01107.	transcript	1.045941	0.92185	1.372487	1.036139	1.257244	0.91127	1.170436	0.974424	1.315155
Novel_000861	XLOC_01107.	transcript	0	0.214414	0.817361	0.288206	0.705166	0.43101	0.308328	0.511359	0.436279
Novel_000862	XLOC_01107.	transcript	0.213883	0.068532	0.265969	0.161995	0.108475	0.359791	0.188722	0.294971	0.072795

Novel_000863	XLOC_01113.	transcript	0.456266	0.896218	0.517113	0.58477	0.541211	0.526163	0.55543	0.686975	0.416772
Novel_000864	XLOC_01114.	transcript	0	0	0	0	0	0	0.008904	0	0.027091
Novel_000865	XLOC_01114.	transcript	0.652827	0.496593	1.077061	0.912702	1.09445	1.058064	1.34142	1.232858	1.771943
Novel_000866	XLOC_01114.	transcript	0	0	0	0	0	0	0	0	0
Novel_000867	XLOC_01114.	transcript	1.805253	1.747802	2.441681	2.788924	2.980407	2.354956	3.710718	2.258682	1.556428
Novel_000868	XLOC_01115.	transcript	0.691042	1.355947	1.218693	1.182717	1.424036	0.939774	1.469343	1.397766	1.396224
Novel_000869	XLOC_01115.	transcript	0	0	0	0	0	0	0	0	0
Novel_000870	XLOC_01115.	transcript	0	0	0	0	0	0	0	0	0
Novel_000871	XLOC_01115.	transcript	0	0.140781	0.026199	0	0.012735	0.024396	0	0	0.010581
Novel_000872	XLOC_01115.	transcript	0.034768	0.003118	0.229776	0.177962	0.237429	0.186647	0.42072	0.230015	0.077293
Novel_000873	XLOC_01115.	transcript	0.224964	0.176282	0	0	0	0	0.060696	0	0.02097
Novel_000874	XLOC_01116.	transcript	0	0.073113	0	0.03868	0.419531	0	0.106645	0.241833	0.066971
Novel_000875	XLOC_01116.	transcript	0.205946	0.317656	0.266112	0.366172	0.07476	0.357442	0.319481	0.174816	0.276417
Novel_000876	XLOC_01116.	transcript	2.536787	2.691885	3.297339	4.231248	3.184078	4.151547	3.375882	3.569706	4.651874
Novel_000877	XLOC_01119.	transcript	0.149464	0.334649	0.941099	0.584087	0.658267	0.543888	0.606936	0.661296	1.245162
Novel_000878	XLOC_01119.	transcript	2.017656	2.451434	0	0.166075	1.216745	0.228925	0.021111	0.175962	0.001954
Novel_000879	XLOC_01119.	transcript	0.565791	0.452696	0	0	0	0.022705	0	0.509038	0
Novel_000880	XLOC_01119.	transcript	0.160722	0.059953	0	0	1.674601	2.135704	0	0	1.294803
Novel_000881	XLOC_01119.	transcript	0	0	0	0	0	0.134646	0	1.775537	0.621602
Novel_000882	XLOC_01119.	transcript	7.00E-06	0.001333	0.832742	0	0.561429	0.004138	0	0.463362	4.00E-06
Novel_000883	XLOC_01119.	transcript	0.541102	0.634822	0	1.456993	0.341959	0.29757	1.09674	0.493908	0.595913
Novel_000884	XLOC_01120.	transcript	0	0	0	0	0	0	0.488581	0.354303	0
Novel_000885	XLOC_01121.	transcript	0	0.266944	0.173627	0.085604	0.028461	0.201642	0.194225	0.044685	0.243307
Novel_000886	XLOC_01121.	transcript	0.038911	0.257645	0.020569	0.031579	0.17909	0.070265	0.079301	0.094572	0.206978
Novel_000887	XLOC_01125.	transcript	0.016341	0.192214	0.044461	0.053051	0.113852	0.205631	0.431787	0.1355	0.060617
Novel_000888	XLOC_01125.	transcript	0	0	0.344641	0.430596	0	0	0.167865	0.267697	0
Novel_000889	XLOC_01128.	transcript	0	0	0	0	0	0	0	0.066815	0
Novel_000890	XLOC_01128.	transcript	0.304029	0.367631	0.40872	0.120703	0.391912	0.499059	0.512767	0.312114	0.378224
Novel_000891	XLOC_01128.	transcript	0.270528	0	0	0.179475	0.136527	0	0.118811	0.09411	0.12053
Novel_000892	XLOC_01128.	transcript	0.004606	0	0	0	0	0	0	0	0
Novel_000893	XLOC_01129.	transcript	0.034966	0	0.09257	0.014677	0.179111	0	0.129127	0.149495	0.278321
Novel_000894	XLOC_01131.	transcript	0	0	0	0	0	0	0.46504	0	0
Novel_000895	XLOC_01131.	transcript	0.615355	0	0	0	2.85276	0	0.282443	0	3.70E-05
Novel_000896	XLOC_01131.	transcript	0.606141	0	3.501632	1.183563	0.743891	0	0.285464	2.091777	0.702384
Novel_000897	XLOC_01131.	transcript	2.252875	0	1.376545	2.535111	0.87348	2.833363	3.288294	2.145426	4.966739
Novel_000898	XLOC_01131.	transcript	0.408932	0	0	0	0.80008	0	0.634109	0	0
Novel_000899	XLOC_01131.	transcript	0.955955	0	0	0	0.78513	0	0.321244	0	0
Novel_000900	XLOC_01131.	transcript	2.58534	1.06193	1.757631	2.067203	1.055512	3.142831	2.457164	2.673576	3.085174

Novel_000901	XLOC_01132.	transcript	0.104026	0.018379	0.045607	0.04656	0.112044	0.158394	0.106753	0.120301	0.210249
Novel_000902	XLOC_01133.	transcript	10.5432	0	0.026847	0	14.54559	0	0.034138	0	0
Novel_000903	XLOC_01133.	transcript	2.118566	0	0	0.057465	3.332343	0	0.114596	3.958305	0
Novel_000904	XLOC_01133.	transcript	1.803432	0	0	0	0	0.024519	0.022659	0.032091	0
Novel_000905	XLOC_01133.	transcript	0	0	18.07835	0	0.057798	0	23.42948	9.543017	0
Novel_000906	XLOC_01133.	transcript	1.258434	1.780631	0	18.83574	4.8171	18.79613	0	5.556182	17.58639
Novel_000907	XLOC_01135.	transcript	0.079379	0.035369	0.260964	0.055349	4.927037	0.247055	0.193671	0.198397	0.161462
Novel_000908	XLOC_01135.	transcript	19.49495	2.152456	17.56047	16.48144	15.82805	20.86623	22.0077	19.14278	14.87943
Novel_000909	XLOC_01136.	transcript	0.241395	0.395151	0.344483	0.291811	0.341352	0.27752	0.498041	0.320791	0.259884
Novel_000910	XLOC_01136.	transcript	0.646494	0.200444	0.394923	0.370498	0.532923	0.395169	0.472219	0.544215	0.380641
Novel_000911	XLOC_01136.	transcript	0.001062	0	0	0	0	0	0	0.000998	0
Novel_000912	XLOC_01137.	transcript	0	0	0	0	0	0.108956	0	0	0
Novel_000913	XLOC_01137.	transcript	0	0	0	0	0	0	0	0.159509	0.253952
Novel_000914	XLOC_01137.	transcript	0.068027	0	0.012383	0.020188	0.016751	0.136052	0.015	0.069788	0
Novel_000915	XLOC_01137.	transcript	0	0	0	0	0	0	0	1.090774	0
Novel_000916	XLOC_01137.	transcript	0	0	0	0	0	0	0.164973	0	0
Novel_000917	XLOC_01137.	transcript	1.329443	0.66978	2.391422	2.154978	2.139461	2.058232	1.529534	0.641738	1.806905
Novel_000918	XLOC_01140.	transcript	0.296338	1.852486	0.196864	0.121635	0.046788	0.038868	0.394424	0.125734	0.389947
Novel_000919	XLOC_01140.	transcript	0	0	0	0	0.150855	0	0	0	0
Novel_000920	XLOC_01141.	transcript	0.240389	1.422689	0.114664	0.09556	0.10623	0.220247	0.296064	0.089909	0.206842
Novel_000921	XLOC_01142.	transcript	0.28589	0	0.194876	0.113106	0.168083	0.329259	0.430833	0.157435	0.338519
Novel_000922	XLOC_01151.	transcript	0	0.16762	0.383661	0.125522	0.135057	0.367509	0.409892	0.274683	0.223774
Novel_000923	XLOC_01151.	transcript	4.390379	2.36016	5.129043	3.930899	4.291037	5.566674	5.101188	5.082929	5.235452
Novel_000924	XLOC_01151.	transcript	0	0.278932	0	0.000218	0.014439	0.119229	0	0	0
Novel_000925	XLOC_01153.	transcript	0.100457	0.585811	0.039083	0.142734	0.134274	0.152495	0.311185	0.109522	0.146835
Novel_000926	XLOC_01153.	transcript	0.01347	0.084593	0.029911	0.021657	0.238361	0	0.303519	1.50E-05	0.40518
Novel_000927	XLOC_01153.	transcript	1.243231	0.277756	0.937714	0.703501	2.159508	1.358935	1.714708	1.429877	1.411972
Novel_000928	XLOC_01153.	transcript	0	0.882467	0	0	0	0	0	0	0
Novel_000929	XLOC_01153.	transcript	0	0	0.001279	0	0	0	0	0	0
Novel_000930	XLOC_01153.	transcript	1.133145	0.780721	1.120145	0.565534	0	0.764115	0.395889	0.583434	0.249716
Novel_000931	XLOC_01153.	transcript	0.170601	0.336696	0	0.224885	0.231456	0.245726	0.291743	0.321161	0
Novel_000932	XLOC_01153.	transcript	0.032634	0	0	0	0.066811	0	0.009978	0.058983	0
Novel_000933	XLOC_01153.	transcript	0.082095	0.163027	0.155268	0.196362	0.154593	0.193364	0.297539	0.118804	0.065847
Novel_000934	XLOC_01156.	transcript	0.119915	0.315417	0.33268	0.374959	0.07865	0.713303	0.106925	0.113794	0.428611
Novel_000935	XLOC_01156.	transcript	0	0.000152	0.207449	0.108486	0.21652	0.125805	0.256458	0.277602	0.267905
Novel_000936	XLOC_01156.	transcript	0.348893	0	0.216066	0	0.277502	0.113444	0.272423	0.292996	0
Novel_000937	XLOC_01156.	transcript	0.32474	0.657458	0.219405	0.608289	0.280463	0.63327	0.258261	0.271427	0.783063
Novel_000938	XLOC_01156.	transcript	0.070311	0.040773	0.007131	0.009198	0.012975	0.335146	0.016705	0.004997	0.016546

Novel_000939	XLOC_01156.	transcript	0.447527	0.14432	0.289255	0.344182	0.300098	0.198507	0.304465	0.310343	0.046933
Novel_000940	XLOC_01156.	transcript	0.566223	0.108499	0.367651	0.017319	0.360685	0	0.332137	0.329725	0.02487
Novel_000941	XLOC_01156.	transcript	0	0	0	0	0	0	0	0	0
Novel_000942	XLOC_01156.	transcript	0.27173	0.055414	0.216288	0	0.224091	0.062555	0.259649	0.23904	0.075849
Novel_000943	XLOC_01157.	transcript	0.058416	0.039646	0	0	0.041778	0.09154	0.223591	0.444578	0.111252
Novel_000944	XLOC_01162.	transcript	0	0	0	0.355935	0	0	0.432479	0.496209	0.728342
Novel_000945	XLOC_01162.	transcript	0.370894	0.199031	0.528464	0.100607	0.445092	0.648589	0.027599	0	0.073481
Novel_000946	XLOC_01165.	transcript	0	0	0	0	0	0.009019	0	0	0.018352
Novel_000947	XLOC_01165.	transcript	0.19593	0.285274	0.156341	0.114381	0.174901	0.079361	0.259013	0.26768	0.141642
Novel_000948	XLOC_01165.	transcript	0	0.531202	0	0	0	0	0	0	0
Novel_000949	XLOC_01165.	transcript	0	0	0.349422	0	0	0	0	0	0
Novel_000950	XLOC_01165.	transcript	0.723272	0.333306	0.339295	0.693405	0.500991	0.742157	0.855391	0.9416	0.685018
Novel_000951	XLOC_01167.	transcript	0	0.011359	0.357178	0.149762	0	0.135087	0.142002	0.048798	0.197453
Novel_000952	XLOC_01167.	transcript	0.239594	0.333552	0.085125	0.123655	0.322861	0.239638	0.231103	0.317279	0.17594
Novel_000953	XLOC_01172.	transcript	0.448213	0.119976	0.525424	0.749055	0.43367	0.672787	0.80645	0.681014	1.001188
Novel_000954	XLOC_01183.	transcript	0	0	0.066062	0.104259	0.088594	0.154901	0.062103	0.266796	0.282901
Novel_000955	XLOC_01183.	transcript	0.372333	0.572454	0.489294	0.911041	0.455773	1.063612	0.869556	0.195296	0.66893
Novel_000956	XLOC_01183.	transcript	0	0	0	0	0	0	0	0.453148	0
Novel_000957	XLOC_01183.	transcript	0	0	0	0	0	0	0.989245	0.845766	0
Novel_000958	XLOC_01183.	transcript	1.307923	0.93867	1.173606	0.956273	0.974216	1.182771	0.917211	0.657251	0.930407
Novel_000959	XLOC_01183.	transcript	0.214319	0.242625	0.183867	0.357454	0.205994	0.272778	0.051618	0.069862	0.154755
Novel_000960	XLOC_01193.	transcript	0.059009	0.11075	0.044183	0.139992	0.082448	0	0.369481	0.140446	0
Novel_000961	XLOC_01194.	transcript	0.122661	0	0.303994	0.125162	0.174309	0.344516	0.192414	0.22807	0.128672
Novel_000962	XLOC_01196.	transcript	0	0.080002	0	0	0.124579	0	0.397241	0.16611	0.109276
Novel_000963	XLOC_01196.	transcript	0.16473	0.074054	0.28213	0.248794	0.01484	0.217144	0.035472	0.134968	0.010711
Novel_000964	XLOC_01196.	transcript	0.110221	0	0	0.139068	0.238374	0.062647	0.017605	0.055948	0
Novel_000965	XLOC_01196.	transcript	0.166697	0.040814	0.149957	0.064249	0.258686	0.19098	0.269689	0.220819	0.263833
Novel_000966	XLOC_01198.	transcript	0.212711	0.733739	0.512068	0.376334	0.62639	0.494016	0.812213	0.484556	0.37248
Novel_000967	XLOC_01200.	transcript	0.25992	0.392504	0.284495	0.159848	0.53208	0.525458	0.350825	0.648506	0.419508
Novel_000968	XLOC_01201.	transcript	0	0	0	0	0	1.242606	0	0.081423	0
Novel_000969	XLOC_01201.	transcript	0.890191	1.745532	0.753956	1.01166	0.976332	0.078694	0.831023	0.925235	0.71805
Novel_000970	XLOC_01204.	transcript	0.634727	0.255184	0.757565	1.11904	0.980955	1.027872	1.025494	1.06254	0.953671
Novel_000971	XLOC_01204.	transcript	0.107273	0	0.170115	0.067429	0.165085	0.179389	0.229586	0.038079	0
Novel_000972	XLOC_01208.	transcript	0.141972	0.417264	0.473786	0.592434	0.259464	0.304286	0.350753	0.264795	0.214486
Novel_000973	XLOC_01208.	transcript	0.110556	0.170311	0.322392	0.191141	0.241717	0.23586	0.381779	0.321983	0.385452
Novel_000974	XLOC_01209.	transcript	0.690176	1.304569	0.914244	1.065218	0.949085	1.079556	1.469143	0.531354	0.877656
Novel_000975	XLOC_01209.	transcript	0	0	0	0	0	0	0	0	0
Novel_000976	XLOC_01209.	transcript	0	0	0.031213	0	0	0	0	0	0

Novel_000977	XLOC_01209.	transcript	0	0	0	0	0	0	0	0.584548	0
Novel_000978	XLOC_01209.	transcript	0.033582	0	0	0	0	0	0	0	0
Novel_000979	XLOC_01210.	transcript	0.41253	0.361207	0.827228	1.211248	0.95258	1.146097	0.847549	0.861779	0.617693
Novel_000980	XLOC_01210.	transcript	0.225647	0.220861	0.48404	0.648384	0.261902	0.676401	0.797311	0.338168	0.918748
Novel_000981	XLOC_01210.	transcript	0.265156	0.183753	0.273909	0.160033	0.149068	0.215703	0.276667	0.182332	0.115075
Novel_000982	XLOC_01214.	transcript	0	0.152776	0.013308	0.036299	0.086512	0.020531	0.647181	0.375633	0
Novel_000983	XLOC_01214.	transcript	0.2772	0.146352	0.347156	0.422514	0.162001	0.195249	0.089652	0.142146	0.473567
Novel_000984	XLOC_01214.	transcript	0	0.023775	0	0.028468	0	0	0	0.082886	0
Novel_000985	XLOC_01215.	transcript	0	0.435969	0	0	0	0	0	0	0
Novel_000986	XLOC_01220.	transcript	0.159127	0.065191	0.215375	0.212854	0.1939	0.030834	0.455133	0.319752	0.206693
Novel_000987	XLOC_01220.	transcript	1.239811	0.535167	1.694242	1.630314	1.354321	1.53558	1.553237	1.336337	1.174114
Novel_000988	XLOC_01221.	transcript	0.045732	0	0.045762	0.013377	0.022136	0	0	0.064029	0
Novel_000989	XLOC_01221.	transcript	0	0	0.232898	0	0	0.151504	0	0	0
Novel_000990	XLOC_01221.	transcript	1.485256	1.519564	1.556661	1.474405	1.24557	1.540495	1.932466	1.92685	1.585637
Novel_000991	XLOC_01221.	transcript	0.144235	0.148587	0	0.356435	0.497204	0	0.366056	0.245342	0.193842
Novel_000992	XLOC_01221.	transcript	0.615383	0.559812	0.387928	0.668409	0.131329	0.38061	0.52075	0.005558	0.543979
Novel_000993	XLOC_01221.	transcript	0.081967	0.035872	0.023292	0.017819	0.217295	0.05389	0.034999	0.194627	0.034729
Novel_000994	XLOC_01221.	transcript	0.006001	0	0	0.076258	0.245491	0.00996	0	0.322574	0.025955
Novel_000995	XLOC_01221.	transcript	0	0	0	0	0.167728	0	0.40541	0.38441	0
Novel_000996	XLOC_01221.	transcript	0	0	0.154183	0	0	0	0	0	0
Novel_000997	XLOC_01222.	transcript	0.049153	0	0.049275	0.19384	0.308194	0.012529	0.546648	0.280858	0.122084
Novel_000998	XLOC_01222.	transcript	0.365446	0.135379	1.657547	6.887352	3.100044	1.838654	1.792593	2.345963	0.929681
Novel_000999	XLOC_01222.	transcript	0.019751	0.025791	0.028058	0.229489	0.051052	0.031094	0.0707	0.22867	0.023926
Novel_001000	XLOC_01222.	transcript	0.161079	0.161976	0.177753	0.262457	0.516985	0.337261	0.290062	0.284499	0.160357
Novel_001001	XLOC_01222.	transcript	0.646931	0.212919	0.341268	1.499262	1.112032	0.931907	1.174728	0.456728	0.751661
Novel_001002	XLOC_01224.	transcript	0	0	0.202401	0	0	0	0	0	0.021486
Novel_001003	XLOC_01224.	transcript	0.044329	0.354899	0	0	0.016422	0.047539	0.064262	0.26618	0
Novel_001004	XLOC_01224.	transcript	0.436685	0.301422	0.586491	0.593621	0.71629	0.510862	0.536115	0.392336	0.399046
Novel_001005	XLOC_01224.	transcript	0.201415	0	0.000766	0	0.022053	1.247104	0.000834	0.000317	1.3669
Novel_001006	XLOC_01224.	transcript	2.47561	0.598594	4.174253	4.950693	4.444436	3.194039	4.61503	4.507275	1.890478
Novel_001007	XLOC_01224.	transcript	0	0.339544	0	0	0	0	0	0	0
Novel_001008	XLOC_01226.	transcript	124.9101	152.9022	155.3039	186.9171	167.669	175.5764	155.9721	145.1872	231.8969
Novel_001009	XLOC_01226.	transcript	0.458754	0.691253	0.382176	0.265594	0.481013	0.395407	0.841021	0.35018	0.35449
Novel_001010	XLOC_01226.	transcript	0.118742	0	0.019518	0.107559	0.174731	0.059379	0.459793	0.066904	0.023139
Novel_001011	XLOC_01235.	transcript	0.353678	0.284673	0.305389	0.372692	0.343997	0.438716	0.610011	0.447696	0.326998
Novel_001012	XLOC_01237.	transcript	0.287377	0.097282	0.442482	0.632265	0.322567	0.677364	0.247174	0.665594	0.230743
Novel_001013	XLOC_01238.	transcript	0	0.02943	0.0544	0.139767	0.135028	0	0.310055	0.222809	0.050027
Novel_001014	XLOC_01238.	transcript	0.19593	0.11865	0.292333	0.170262	0.007694	0.220521	0.282587	0.072916	0.298024

Novel_001015	XLOC_01238.	transcript	0.194961	0	0	0.067199	0.163983	0	0	0.087405	0
Novel_001016	XLOC_01240.	transcript	0.756029	0.772088	1.027382	0.672697	0.932037	1.099507	1.110695	0.586495	0.618316
Novel_001017	XLOC_01240.	transcript	0.043288	0.031248	0.056933	0.034874	0.051563	0.072292	0.077302	0.054384	0.067077
Novel_001018	XLOC_01240.	transcript	0.628745	0.261317	0.205946	0.134005	0.997525	0.13641	0.159797	0.125887	0.234444
Novel_001019	XLOC_01240.	transcript	0	0	0.877672	0.016344	0.039837	0.924195	1.065185	0.921346	0.784495
Novel_001020	XLOC_01241.	transcript	0.831187	0.24848	1.156907	1.80495	1.243543	1.369616	1.303521	1.85322	1.03915
Novel_001021	XLOC_01241.	transcript	0	0.734797	0	0	0	0	0	0.012049	0.012492
Novel_001022	XLOC_01241.	transcript	0.11876	0.042601	0.140652	0.126849	0.148766	0.154393	0.257035	0.269778	0.158836
Novel_001023	XLOC_01241.	transcript	0.102665	0	0.245304	0.048197	0.233123	0.025938	0.205033	0.122429	0.12498
Novel_001024	XLOC_01241.	transcript	0	0.213423	0.291424	0	0	0	0	0	0
Novel_001025	XLOC_01241.	transcript	0.201444	0.119461	0.100003	0.113314	0.119225	0.083876	0.118169	0.220645	0.117337
Novel_001026	XLOC_01241.	transcript	0.108835	0	0.052854	0.030944	0.064944	0.02978	0.136497	0.203674	0.097702
Novel_001027	XLOC_01241.	transcript	0.149834	0.241555	0.209004	0.1321	0.344213	0.093448	0.376096	0.163534	0.145506
Novel_001028	XLOC_01241.	transcript	0.029793	0	0.013382	0.031439	0.003847	0.033374	0.01194	0.062492	0.006207
Novel_001029	XLOC_01241.	transcript	0.122828	0.260386	0.166299	0.186431	0.160427	0.230614	0.123721	0.184421	0.157952
Novel_001030	XLOC_01241.	transcript	0.102955	0	0.398136	0.483535	0.217478	1.185762	0.121493	0.094505	0.126467
Novel_001031	XLOC_01241.	transcript	0.072924	0	0.151337	0.080219	0	0	0.026642	0.043432	0
Novel_001032	XLOC_01241.	transcript	0.030963	0.011161	0.013624	0.014178	0.020768	0.058262	0.005768	0.002577	0.046167
Novel_001033	XLOC_01241.	transcript	0.130911	0.153895	0.138158	0.296086	0.062788	0.175182	0.172477	0.171857	0.179798
Novel_001034	XLOC_01242.	transcript	0.099364	0.004758	0.141649	0.160145	0.112618	0.152649	0.198062	0.123367	0.08756
Novel_001035	XLOC_01242.	transcript	0.551079	0	0.111282	0.133347	0.023831	0.049061	0.077142	0.018746	0.514239
Novel_001036	XLOC_01242.	transcript	0.225333	0	0.024545	0.038394	0.189113	0.040292	0.397958	0.241612	0.016125
Novel_001037	XLOC_01242.	transcript	0.026254	0	0.67033	0.177799	0.390987	0.303046	0.360757	0.15212	0.057324
Novel_001038	XLOC_01242.	transcript	0	0	0	0.266183	0.267287	0.3641	0	0.314869	0
Novel_001039	XLOC_01245.	transcript	0.853814	0.407272	0.361319	0.274548	0.648767	0.534956	0.77942	0.399535	0.453696
Novel_001040	XLOC_01246.	transcript	0.122468	0.793762	0.036646	0.044295	0.092477	0.070363	0.176576	0.030771	0.174805
Novel_001041	XLOC_01251.	transcript	0.00168	0	0.009212	0.190844	0	0.139196	0.187343	0	0.295458
Novel_001042	XLOC_01253.	transcript	0.207254	0	0	0.047074	0.195293	0.098761	0.065895	0.049857	0
Novel_001043	XLOC_01253.	transcript	0	0	0.245073	0	0	0	0.10234	0	0
Novel_001044	XLOC_01253.	transcript	0.142528	0.027482	0.059908	0.1107	0	0	0.142306	0.095368	0
Novel_001045	XLOC_01255.	transcript	0.172039	0.387521	0.162071	0.051379	0.135476	0.082139	0.194651	0.115459	0.334487
Novel_001046	XLOC_01258.	transcript	0.368251	0.825426	0.432228	0.43634	0.383446	0.400729	0.462345	0.31157	0.360654
Novel_001047	XLOC_01258.	transcript	0	0	0	0	0	0	0.017993	0.235203	0
Novel_001048	XLOC_01258.	transcript	0.222325	0.091919	0.159849	0.142578	0.181036	0	0.084861	0.139676	0.10601
Novel_001049	XLOC_01259.	transcript	0.138851	0.08657	0.14938	0.142762	0.2159	0.103436	0.253242	0.177684	0.26702
Novel_001050	XLOC_01259.	transcript	0	0.029772	0	0.010726	0.021654	0	0.038029	0	0
Novel_001051	XLOC_01260.	transcript	0.27438	0.154384	0.253405	0.258994	0.298783	0.132334	0.466202	0.249574	0.318696
Novel_001052	XLOC_01261.	transcript	0.159652	0.10684	0.205661	0.204542	0.218644	0	0.253794	0.06206	0.199357

Novel_001053	XLOC_01261.	transcript	0	0	0	0	0	0	0	0	0
Novel_001054	XLOC_01261.	transcript	0	0	0.049569	0.006403	0	0.255216	0	0.214323	0
Novel_001055	XLOC_01262.	transcript	0.00351	0.460343	0.012979	0	0.037665	0	0	0.007476	0.014528
Novel_001056	XLOC_01262.	transcript	0.113714	0.828657	0.115633	0.082567	0.101352	0.179241	0.370837	0.147682	0.575895
Novel_001057	XLOC_01264.	transcript	0.805994	0.804246	0.914575	0.638126	0.745263	0.912284	0.777428	0.966998	0.849351
Novel_001058	XLOC_01266.	transcript	0.138351	0.284248	0.018161	0.018045	0.03402	0.08315	0.196683	0.034474	0.599355
Novel_001059	XLOC_01268.	transcript	0.507034	0.697015	0.897291	0.702751	0.804945	1.002317	0.776158	0.80591	0.823128
Novel_001060	XLOC_01270.	transcript	0.472797	0.015606	0.437273	0.041312	0.0147	0.062343	1.016433	0.008877	0.431211
Novel_001061	XLOC_01270.	transcript	0	0.360014	0	0.617238	0.636079	0.736003	0.003143	0.74183	0.010073
Novel_001062	XLOC_01270.	transcript	0	0	0	0.258704	0	0.58181	0.106976	0.200284	0
Novel_001063	XLOC_01270.	transcript	0.06875	0.090657	0.176189	0.064259	0.351449	0.069445	0.04509	0.062198	0.11338
Novel_001064	XLOC_01276.	transcript	0.398942	0.458434	0.616581	0.687733	0.928144	0.735698	1.042397	0.868708	1.003216
Novel_001065	XLOC_01276.	transcript	0.000873	0	0	0	0.196962	0.077639	0.163997	0	0
Novel_001066	XLOC_01276.	transcript	0.048089	0	0.074778	0.164803	0.087485	0.077563	0.081076	0.076937	0
Novel_001067	XLOC_01276.	transcript	0.053571	0	0.100688	0.339359	0.141276	0.047796	0.091075	0.066933	0.34622
Novel_001068	XLOC_01276.	transcript	0	0	0	0.018931	0	0	0	0	0
Novel_001069	XLOC_01276.	transcript	0.069793	0	0.032455	0	0	0.064355	0.042913	0.085529	0.246708
Novel_001070	XLOC_01276.	transcript	0.09774	0	0.040154	0	0	0.055914	0.077199	0.063785	0.239652
Novel_001071	XLOC_01276.	transcript	0.023322	0.240171	0.073798	0.049536	0.030969	0.091879	0.054878	0.092458	0
Novel_001072	XLOC_01276.	transcript	0.087376	0	0	0	0	0	0	0	0
Novel_001073	XLOC_01276.	transcript	0.069823	0	0.05602	0	0	1.049609	0.085177	0.028144	0
Novel_001074	XLOC_01276.	transcript	1.316319	0	0	0.890533	0.805834	0	1.608952	0.153911	0
Novel_001075	XLOC_01276.	transcript	0.038245	0.831764	0.023777	0	0	0.007009	0.062958	0.138305	0
Novel_001076	XLOC_01276.	transcript	0	0	0.648518	0	0	0	0	1.170048	0
Novel_001077	XLOC_01276.	transcript	0	0.221145	0	0.158903	0	0.103718	0	0.078256	0.002836
Novel_001078	XLOC_01277.	transcript	1.240447	1.386718	1.361262	1.440577	0.893817	1.805462	1.377747	1.332278	0.80628
Novel_001079	XLOC_01277.	transcript	0	0	0	0	0	0	0	0	0
Novel_001080	XLOC_01277.	transcript	0.168128	0.394758	0.203816	0.169546	0.113683	0.127868	0.324937	0.175016	0.145693
Novel_001081	XLOC_01279.	transcript	0	0.424575	0	0	0	0.579115	0.000257	1.722206	0.077636
Novel_001082	XLOC_01279.	transcript	0.008422	0.048566	0.000318	0.004349	0.00427	1.206235	0.018039	0.720114	0.065386
Novel_001083	XLOC_01279.	transcript	0	0.015921	0	0	0	0	0.003708	0	0
Novel_001084	XLOC_01279.	transcript	1.280357	0	1.392268	0.867219	1.541456	0.791497	1.406193	0.694203	0.960944
Novel_001085	XLOC_01279.	transcript	0.730092	0	4.008173	1.886408	1.126748	0.902548	1.361199	0.670362	2.095877
Novel_001086	XLOC_01279.	transcript	1.178147	0	1.168245	0.758212	1.215291	0.72595	1.119579	0.629155	0.866659
Novel_001087	XLOC_01279.	transcript	1.871385	0	1.289601	2.680726	2.22133	2.473558	1.779305	0.814817	1.049124
Novel_001088	XLOC_01280.	transcript	0	0	0	0	0	0	0	0.019678	0
Novel_001089	XLOC_01281.	transcript	1.634879	4.32319	4.160306	8.098815	5.627692	5.33731	4.51176	4.898894	5.453185
Novel_001090	XLOC_01281.	transcript	0.149763	0.003764	0.07067	0.164414	0.204567	0.290165	0.187122	0.437711	0.096219

Novel_001091	XLOC_01281.	transcript	0.113912	0	0.17539	0.097466	0.283619	0.339827	0.081248	0.149026	0.066327
Novel_001092	XLOC_01286.	transcript	0.222263	0.562721	0.117125	0.027533	0.333804	0.367613	0.332057	0.153661	0.188293
Novel_001093	XLOC_01286.	transcript	0.333205	0.291517	0.048051	0.396668	0.094745	0	0.210154	0.108868	0.051116
Novel_001094	XLOC_01286.	transcript	0	0.475056	0.037065	0	0.193958	0.273863	0.000678	0.000584	0.039066
Novel_001095	XLOC_01286.	transcript	0	0.386579	0.44619	0	0.075579	0	0.38615	0.466313	0.427114
Novel_001096	XLOC_01288.	transcript	0.267715	1.799832	0.976904	1.376566	0.950021	1.035383	0.828219	1.26084	1.163623
Novel_001097	XLOC_01288.	transcript	0.443702	0.333799	0	0	0	0	0	0	0
Novel_001098	XLOC_01291.	transcript	65.50441	62.10025	54.38779	28.73948	47.23866	33.74424	38.72612	55.43092	38.33719
Novel_001099	XLOC_01291.	transcript	0.301841	0.361496	0.356506	0.477899	0.169375	0.400616	0.58573	0.36963	0.004939
Novel_001100	XLOC_01292.	transcript	0.129509	0.120683	0.240678	0.160075	0.126194	0.070444	0.136481	0.159854	0.121898
Novel_001101	XLOC_01292.	transcript	0	0	0.056579	0.047954	0.061059	0	0.023348	0.042677	0
Novel_001102	XLOC_01292.	transcript	0	0	0	0	0	0	0	0	0
Novel_001103	XLOC_01293.	transcript	0.190499	0.279236	0.278343	0.098252	0.23116	0.211279	0.268762	0.197584	0.236996
Novel_001104	XLOC_01296.	transcript	0	0.146517	0.064577	0.029764	0.0412	0.032266	0.028865	0.090455	0.045802
Novel_001105	XLOC_01296.	transcript	0.133232	0.34633	0.192745	0	0.087808	0.049261	0.11879	0.102306	0.240937
Novel_001106	XLOC_01296.	transcript	0.077697	0.021292	0.000103	0.001858	0.047452	0.086115	0	0.002282	0
Novel_001107	XLOC_01298.	transcript	0.059241	0.66415	0.079917	0.046013	0.082121	0.069855	0.118484	0.114711	0.05089
Novel_001108	XLOC_01298.	transcript	0.094922	0.050459	0.226294	0.201196	0.198437	0.28887	0.368291	0.163881	0.155843
Novel_001109	XLOC_01298.	transcript	0.140952	0.01533	0.106573	0.076233	0.067789	0.324328	0	0.153405	0
Novel_001110	XLOC_01298.	transcript	0.023965	0	0.003585	0.026787	0.035368	0.065593	0.382992	0.016102	0.271421
Novel_001111	XLOC_01298.	transcript	0	0	0	0	0	0	0	0	0
Novel_001112	XLOC_01301.	transcript	4.4475	4.149459	5.248571	6.322896	6.042147	6.768549	6.351793	5.863856	6.461851
Novel_001113	XLOC_01301.	transcript	2.036395	3.100635	2.761376	2.539071	2.567511	3.032517	2.671407	2.080474	3.254469
Novel_001114	XLOC_01301.	transcript	0	0	0	0	0	0	0	0	0
Novel_001115	XLOC_01303.	transcript	0.228583	0.670085	0.131986	0.074873	0.171586	0.239335	0.444465	0.181539	0.103761
Novel_001116	XLOC_01310.	transcript	1.744525	1.430332	1.188343	1.598393	1.029538	0.950636	0.970162	1.04081	0.430986
Novel_001117	XLOC_01310.	transcript	0	0	0	0	0	0	0.26519	0.053358	0
Novel_001118	XLOC_01310.	transcript	0	1.372492	0	0	0	0.165101	0.710176	0.954569	1.28815
Novel_001119	XLOC_01312.	transcript	0.20225	0.239867	0.148427	0.242973	0.205631	0.20024	0.297913	0.275213	0.259971
Novel_001120	XLOC_01312.	transcript	0	0	0.006359	0	0	0	0	0.137069	0
Novel_001121	XLOC_01320.	transcript	0	0	0	0.063797	0	0	0.018863	0.022921	0.095832
Novel_001122	XLOC_01333.	transcript	0.03648	0.138634	0.067719	0.091595	0.088911	0.044162	0.154529	0.041791	0.382632
Novel_001123	XLOC_01333.	transcript	0	0	0	0	0	0	0	0	0
Novel_001124	XLOC_01334.	transcript	0.478594	0.651722	0.647395	0.944274	0.718379	0.815217	0.855599	0.790752	0.510619
Novel_001125	XLOC_01340.	transcript	0	0	0	0	0.105033	0	0	0.080487	0
Novel_001126	XLOC_01340.	transcript	0.251897	0.004219	0.333398	0.642923	0.322495	0.511573	0.509772	0.353148	0.225718
Novel_001127	XLOC_01340.	transcript	0.121952	0.146498	0.247406	0.977999	0.379074	0.385161	0.480377	0.484405	0.227965
Novel_001128	XLOC_01341.	transcript	0.171266	0	0.334935	0.640695	0.603964	0.459552	0.161008	0.264428	0.161466

Novel_001129	XLOC_01341.	transcript	0.173513	0.028133	0.138403	0.178916	0.135523	0.180356	0.282238	0.10937	0.085452
Novel_001130	XLOC_01342.	transcript	0.205063	0.667625	0.405729	0.42774	0.051077	0.133705	0.430716	0.366596	0.337679
Novel_001131	XLOC_01342.	transcript	0	0.076519	0	0.02533	0.087609	0.329447	0	0	0.018522
Novel_001132	XLOC_01342.	transcript	0	0	0	0.016825	0	0	0	0	0
Novel_001133	XLOC_01342.	transcript	0.137463	0.322885	0.170372	0.345271	0.403368	0	0.256012	0.282592	0.238995
Novel_001134	XLOC_01342.	transcript	0.170117	0.124143	0.164161	0.077667	0.143935	0.112829	0.27568	0.171843	0.094563
Novel_001135	XLOC_01342.	transcript	0	0	0	0.095942	0	0	0.05107	0	0
Novel_001136	XLOC_01342.	transcript	0.060305	0	0	0.007893	0.019823	0.009905	0.015786	0.015962	0.088734
Novel_001137	XLOC_01342.	transcript	0.080444	0.178732	0.119363	0.078606	0.121457	0.12117	0.101904	0.051378	0.082182
Novel_001138	XLOC_01342.	transcript	0	0	0.069091	0	0	0	0	0	0
Novel_001139	XLOC_01342.	transcript	0	0	0	0.059902	0	0.039078	0	0.052007	0
Novel_001140	XLOC_01342.	transcript	0	0	0.002974	0.002181	0.006133	0	0	0	0
Novel_001141	XLOC_01342.	transcript	0.23325	0.031108	0.121154	0.251567	0.123161	0.040013	0.316403	0.255498	0.350571
Novel_001142	XLOC_01342.	transcript	0	0	0	0.014301	0.004286	0	0	0	0
Novel_001143	XLOC_01342.	transcript	0	0	0	0	0	0	0.298732	0	0
Novel_001144	XLOC_01342.	transcript	3.483361	1.228298	2.422011	3.555261	1.618583	1.235821	4.134576	3.481205	1.537391
Novel_001145	XLOC_01342.	transcript	0	0	0	0	1.29491	0	0	0	0
Novel_001146	XLOC_01343.	transcript	0.094401	0.098519	0.100211	0.083447	0.11783	0	0	0.06847	0
Novel_001147	XLOC_01343.	transcript	0.134167	0.111829	0.077423	0.131124	0.148462	0	0	0.1652	0.005292
Novel_001148	XLOC_01343.	transcript	0.170301	0.298841	0.583918	0.447745	0.161872	0.643565	0.232001	0.135362	0.362641
Novel_001149	XLOC_01343.	transcript	0.096981	0.139714	0.49605	0.397476	0.148665	0	0	0.100994	0
Novel_001150	XLOC_01343.	transcript	0.094775	0.079566	0.083705	0.08744	0.138646	0.01212	0.022148	0.075551	0.014879
Novel_001151	XLOC_01343.	transcript	0	0	0	0	0.159427	0.00023	0.002174	0	0.008457
Novel_001152	XLOC_01344.	transcript	0.14139	1.035486	0.016896	0.316006	0.144315	0.423147	0.565378	0.191711	0.000616
Novel_001153	XLOC_01347.	transcript	0.009918	0.176028	0	2.094671	0	0	0	0	0
Novel_001154	XLOC_01347.	transcript	0.711968	0	0.64928	0	0.70295	0.694894	1.245497	0.694719	4.205391
Novel_001155	XLOC_01348.	transcript	0.123396	0.30707	0.21492	0.197269	0.203678	0.24829	0.299994	0.204179	0.257602
Novel_001156	XLOC_01349.	transcript	0.885149	1.235652	1.119325	0.811098	1.217521	1.008142	1.423079	1.023227	1.034651
Novel_001157	XLOC_01349.	transcript	0.044798	0.06822	0.047171	3.00E-05	0.035835	0.052077	0.063602	0.063957	0.046877
Novel_001158	XLOC_01349.	transcript	0	0.002356	0.015981	0	0.019055	0.01794	0	0	0
Novel_001159	XLOC_01349.	transcript	0.020567	0	0	0.363065	0.004733	0.003188	0.014663	0.009656	0.025248
Novel_001160	XLOC_01349.	transcript	0.058531	0.039175	0.078208	0.058065	0.084876	0.074597	0.096213	0.050076	0.047507
Novel_001161	XLOC_01350.	transcript	0.083584	0.017893	0.145376	0.074685	0.091933	0.051485	0.064177	0.014957	0
Novel_001162	XLOC_01350.	transcript	0.001969	0	0	0.001155	0	0.001663	0.001031	0.001202	0.041969
Novel_001163	XLOC_01350.	transcript	0.13555	0.000133	0.042385	0.031523	0.070483	0.065839	0.120343	0.103264	0.123123
Novel_001164	XLOC_01350.	transcript	0.582824	0	1.21029	0.761713	0.659711	0.785031	1.251858	0.462291	0.611686
Novel_001165	XLOC_01350.	transcript	0	0.042422	0.000322	0.000467	0.000355	0.000373	0	0.000181	0.001185
Novel_001166	XLOC_01350.	transcript	0.136099	0.029822	0.064043	0.039493	0.145624	0.389172	0.249138	0.02865	0.168799

Novel_001167	XLOC_01350.	transcript	0.281171	0.190071	0.141229	0.399713	0.340087	0.291039	0.256335	0.240979	0.177374
Novel_001168	XLOC_01350.	transcript	0	0	0	0	0	0	0	0	0
Novel_001169	XLOC_01350.	transcript	0.002753	0	0.002492	0	0	0	0	0.002712	0
Novel_001170	XLOC_01351.	transcript	0.924687	3.931023	0.822414	1.383286	1.578829	1.460838	1.23403	1.452666	2.090844
Novel_001171	XLOC_01358.	transcript	7.660527	1.363965	23.82001	41.81033	34.38895	33.75643	12.45281	26.23646	19.34213
Novel_001172	XLOC_01359.	transcript	0.430712	0.477624	0.569698	0.622923	0.61382	0.455168	0.675305	0.888712	0.464235
Novel_001173	XLOC_01359.	transcript	0.394363	0.189197	0.315031	0.4285	0.508429	0.376938	0.497134	0.572132	0.435735
Novel_001174	XLOC_01359.	transcript	0	0	0	0	0	0	0	0	0
Novel_001175	XLOC_01359.	transcript	0	0.31345	0	0	0	0	0	0	0
Novel_001176	XLOC_01363.	transcript	0.383565	0.628608	0.764178	0.565518	0.640253	0.670236	0.861627	0.722083	0.471824
Novel_001177	XLOC_01363.	transcript	0	0	0.056122	0	0.027897	0.12478	0.048807	0.218121	0.128492
Novel_001178	XLOC_01363.	transcript	0.219809	0	0.007932	0	0.003564	0	0	0	0
Novel_001179	XLOC_01371.	transcript	0.172701	0.153704	0.265321	0.149785	0.178912	0.220008	0.360751	0.024766	0.190633
Novel_001180	XLOC_01371.	transcript	0	0.009921	0	0.025927	0.018122	0	0	0.114734	0
Novel_001181	XLOC_01371.	transcript	0	0	0.014284	0	0	0	0	0.173577	0
Novel_001182	XLOC_01373.	transcript	0	0.611143	0.055475	0.01469	0.062748	0	0	0.093448	0.016559
Novel_001183	XLOC_01373.	transcript	0.44086	0.386203	0.386365	0.304657	0.243932	0.253528	0.35712	0.387032	0.22698
Novel_001184	XLOC_01373.	transcript	0	0	0	0.00468	0	0.001509	0	0	0.012996
Novel_001185	XLOC_01373.	transcript	0.028624	0.04485	0	0	0.014054	0	0.019497	0.012064	0
Novel_001186	XLOC_01375.	transcript	0.329153	0.450728	0.373444	0.365854	0.35922	0.341649	0.333114	0.352884	0.386322
Novel_001187	XLOC_01384.	transcript	0.014583	0.157666	0	0.368413	0.12765	0.413601	0.11298	0.015483	0.172725
Novel_001188	XLOC_01384.	transcript	0.488199	0	0	0	0.091012	0.001071	0.007999	0	0
Novel_001189	XLOC_01384.	transcript	0	0	0	0.267941	0	0	0	0.047493	0
Novel_001190	XLOC_01384.	transcript	0.663391	0.081353	0.801525	0.206323	0.664621	0.505739	1.197694	0.913141	0.682519
Novel_001191	XLOC_01384.	transcript	0	0	0	0	0	0.407686	0	0	0
Novel_001192	XLOC_01387.	transcript	0.207966	0	0.001487	0.437136	0.012556	0.34574	0	0.229033	0.44093
Novel_001193	XLOC_01387.	transcript	0.29746	0.266477	0.36846	0.172069	0.483406	0.157399	0.77437	0.326229	0.008778
Novel_001194	XLOC_01388.	transcript	0	0	0	0.014403	0	0	0	0	0.005082
Novel_001195	XLOC_01388.	transcript	0.32595	0.123061	0.158583	0.19239	0.184901	0.209651	0.278321	0.257379	0.334224
Novel_001196	XLOC_01388.	transcript	0.006598	0	0	0	0	0.000639	0	0.015705	0
Novel_001197	XLOC_01388.	transcript	0.047525	0.047977	0.098697	0.039637	0.048608	0.049149	0.122066	0.03957	0.030468
Novel_001198	XLOC_01388.	transcript	0.25007	0	0	0	0	0	0	0.00349	0
Novel_001199	XLOC_01388.	transcript	0	0.295663	0.252861	0.187621	0.278258	0.266453	0.355205	0.297089	0.174268
Novel_001200	XLOC_01388.	transcript	0.322497	0.253533	0.432379	0.491225	0.422991	0.469438	0.620313	0.487513	0.005499
Novel_001201	XLOC_01388.	transcript	0	0	0	0.234037	0	0	0	0	0.692749
Novel_001202	XLOC_01389.	transcript	0.292996	0.417139	0.066838	0.07198	0.073072	0.239266	0.213473	0.192158	0.142276
Novel_001203	XLOC_01390.	transcript	0.94695	0.45181	0.551627	0.419065	0.639216	0	0.910975	0.476431	0.263869
Novel_001204	XLOC_01390.	transcript	0.018447	0.026934	0.065871	0.012772	0.034474	0.027172	0.166933	0.026718	0.106759

Novel_001205	XLOC_01390.	transcript	0.080796	0.033481	0.026409	0.033801	0.030989	0	0.04532	0.069235	0.059846
Novel_001206	XLOC_01390.	transcript	0.251653	0.029505	0	0.319497	0.302194	0	0	0.072665	0
Novel_001207	XLOC_01395.	transcript	0.198907	1.667578	0.373099	0.205431	0.118443	0.257115	0.173468	0.054165	0.510591
Novel_001208	XLOC_01395.	transcript	0	0	0	0	0	0	0	0	0
Novel_001209	XLOC_01395.	transcript	0.470738	0	0.016983	0.123932	0.47114	0	0.516332	0.455126	0
Novel_001210	XLOC_01396.	transcript	0.503555	0.436728	0.403671	0.337886	0.258445	0.302966	0.527731	0.445643	0.481756
Novel_001211	XLOC_01396.	transcript	0	0.00164	0	0	0	0	0	0	0
Novel_001212	XLOC_01396.	transcript	0.257018	0.530571	0.16545	0.202759	0.198326	0.208532	0.17966	0.121689	0
Novel_001213	XLOC_01397.	transcript	0	0.244713	0	0	0.117559	0.124851	0.217203	0	0.111762
Novel_001214	XLOC_01397.	transcript	0.963107	1.659264	1.179086	1.254405	1.243578	1.783368	1.285653	1.155451	1.721837
Novel_001215	XLOC_01397.	transcript	0	0	0	0.391788	0	0	0	0.406698	0
Novel_001216	XLOC_01398.	transcript	1.26417	0.010049	0.003954	1.250644	1.398689	0.09482	0.854796	0.005616	1.46959
Novel_001217	XLOC_01398.	transcript	0.128413	0.128586	0.577344	0.081707	0.003252	0.765089	0.587974	0.448429	0.114454
Novel_001218	XLOC_01398.	transcript	0.042213	0.076341	0.037362	0.050485	0.099668	0	0.02536	0.574292	0
Novel_001219	XLOC_01399.	transcript	0.110285	0	0.2883	0.046238	0.250736	0.135385	0.112338	0.19762	0.17781
Novel_001220	XLOC_01399.	transcript	0.141985	0	0.45668	0.133222	0.259334	0.398848	0.225977	0.194861	0.725395
Novel_001221	XLOC_01399.	transcript	0.09726	0.475735	0.50325	0.19583	0.091058	0.557733	0.117961	0.133487	0.875355
Novel_001222	XLOC_01399.	transcript	0	0	0	0	0	0	0	0	0
Novel_001223	XLOC_01399.	transcript	0.163765	0	0	0	0.050216	0	1.424858	0.076788	0
Novel_001224	XLOC_01399.	transcript	0	0	0	0.934388	0	0.003494	0	0.007902	0
Novel_001225	XLOC_01406.	transcript	0.067789	0.193349	0.108078	0.118906	0.116092	0	0.068857	0.16377	0.072741
Novel_001226	XLOC_01406.	transcript	0.256789	0.299976	0.271481	0.099072	0.173245	0.293801	0.346231	0.318927	0.223923
Novel_001227	XLOC_01407.	transcript	1.672346	2.15235	0.820808	0.729152	0.808109	0.708089	1.386331	0.933577	0.669386
Novel_001228	XLOC_01407.	transcript	0.021766	0	0	0.025769	0	0.038221	0	0.051435	0
Novel_001229	XLOC_01412.	transcript	0.465859	0.597969	0.532014	0.489669	0.305729	0.725188	0.351816	0.531814	0.429558
Novel_001230	XLOC_01425.	transcript	0.146585	0.478449	0.010066	0.342524	0.262267	0.045079	0.234673	0.193533	0.01749
Novel_001231	XLOC_01427.	transcript	0.056583	0.02291	0.0425	0.153279	0.127343	1.096935	0.200779	0	0.145204
Novel_001232	XLOC_01427.	transcript	0.686471	0.203653	0.684123	1.1807	0.873809	0.791812	0.873922	0.332552	0.329435
Novel_001233	XLOC_01427.	transcript	0.523585	0.045596	0.709025	0.075508	0.060154	0.342736	0.304989	0.111838	0.83755
Novel_001234	XLOC_01427.	transcript	0	0	0	0	0	0	0	1.432603	0
Novel_001235	XLOC_01427.	transcript	0.862293	1.579235	0.984912	0.90417	1.229918	1.309842	1.188514	0.902872	1.320938
Novel_001236	XLOC_01427.	transcript	0.325274	0.334706	0.093523	0.323096	0.197791	0.270087	0.714079	0.280915	0.476704
Novel_001237	XLOC_01427.	transcript	0.131785	0.171646	1.489287	1.030467	0.971359	1.477537	0.185467	0.155159	0.679644
Novel_001238	XLOC_01436.	transcript	1.083228	1.307223	4.567809	4.057767	1.52111	4.51336	3.905589	0.936774	3.749715
Novel_001239	XLOC_01436.	transcript	0.476954	0	0.00322	0	1.800844	0	0.00685	0	0.975646
Novel_001240	XLOC_01440.	transcript	0.618571	0.988405	0.907306	0.843759	0.750596	0.938346	0.840632	0.633006	0.52222
Novel_001241	XLOC_01443.	transcript	0.177439	0.017391	0.304615	0.218643	0.309138	0.291246	0.458896	0.266601	0.20826
Novel_001242	XLOC_01443.	transcript	0.102257	0	0.145413	0.140925	0.242084	0.151114	0.259133	0.202771	0.123195

Novel_001243	XLOC_01445.	transcript	0	0	0	0.240136	0	0.120129	0	0	0
Novel_001244	XLOC_01448.	transcript	0.015093	0	0	0.001895	0	0.002009	0	0	0
Novel_001245	XLOC_01448.	transcript	0	0	0	0.63172	0	0	0	0	0
Novel_001246	XLOC_01448.	transcript	0	0	0	0	0	0	0	0	0
Novel_001247	XLOC_01448.	transcript	0.493345	0	0.858212	0.540033	1.7417	1.100528	0.886867	1.432838	0.779234
Novel_001248	XLOC_01448.	transcript	0.000176	0.016316	0.223231	0.671173	0.354267	0.304669	0.383004	0.681862	0
Novel_001249	XLOC_01449.	transcript	0.234555	0.281748	0.166516	0.082847	0	0.081262	0.198095	0.202577	0.219652
Novel_001250	XLOC_01449.	transcript	0.420216	0.283142	0.710515	0.015388	0.921681	0.276226	0.234961	0.238041	0.235753
Novel_001251	XLOC_01449.	transcript	0.394229	0.311744	0.530583	1.312625	0.183882	0.475958	1.04382	0.643102	1.203938
Novel_001252	XLOC_01449.	transcript	0.706923	0.224764	0.47979	0.662427	0.72519	0.446437	0.749597	0.423052	0.555287
Novel_001253	XLOC_01450.	transcript	0	0.011437	0.000191	0	0	0	0	0	0
Novel_001254	XLOC_01450.	transcript	0.338399	0.429217	0.291144	0.107676	0.298439	0.447906	0.046879	0.462129	0.296627
Novel_001255	XLOC_01450.	transcript	0	0	0.038103	0.416562	0	0	0.2958	0	0
Novel_001256	XLOC_01451.	transcript	0.144476	0.160016	0.157874	0.184437	0.058541	0.177477	0.135356	0.112516	0.132329
Novel_001257	XLOC_01451.	transcript	0.253294	0	0.133781	0.211933	0.159756	0.222838	0.165677	0.670366	0
Novel_001258	XLOC_01451.	transcript	0.709006	0.06626	0.976227	0.799581	0.602805	0.964491	0.930581	0.622158	0.52439
Novel_001259	XLOC_01453.	transcript	0.088317	0.150276	0.097776	0.314336	0.165611	0.16655	0.293508	0.23117	0.126603
Novel_001260	XLOC_01461.	transcript	0	0	0.282039	0.138718	0.162664	0.207245	0.329381	0.085106	0.06727
Novel_001261	XLOC_01462.	transcript	0.077079	0.12489	0.0748	0.211022	0.092334	0.047689	0.066907	0.16192	0.267031
Novel_001262	XLOC_01462.	transcript	0.581897	0.485953	0.760858	0.332314	0.44331	0.017636	0.307058	0.639197	0.4813
Novel_001263	XLOC_01462.	transcript	0	0	0	0.130569	0	0.445691	0.560875	0	0
Novel_001264	XLOC_01463.	transcript	0.057079	3.84255	0.050974	0	0.03671	0.041862	0.133412	0.040963	0
Novel_001265	XLOC_01463.	transcript	0.507886	1.004426	0.595449	0.714108	0.793309	0.588372	0.920019	0.946808	0.576479
Novel_001266	XLOC_01463.	transcript	0	0	0	0	0	0	0	0	0
Novel_001267	XLOC_01466.	transcript	0.200158	0	0.365044	0	0	0.333504	0.355292	0.144809	0.117931
Novel_001268	XLOC_01466.	transcript	0.112699	0	0.030497	0.273766	0.095303	0	0.065906	0.011882	0
Novel_001269	XLOC_01467.	transcript	1.266739	3.188626	0.890818	0.87202	1.099743	1.256813	1.730098	1.086724	1.171002
Novel_001270	XLOC_01472.	transcript	0.18521	0.213081	0.2339	0.04419	0.214047	0.180066	0.232216	0.135077	0.165124
Novel_001271	XLOC_01473.	transcript	0.577431	2.364261	1.085444	0.748164	0.681518	0.944057	0.722038	0.847601	0.39193
Novel_001272	XLOC_01477.	transcript	1.046965	8.093522	0.646714	0.336713	0.475522	0.912423	0.824799	0.708912	1.35351
Novel_001273	XLOC_01501.	transcript	0.214186	0.089869	0.184052	0.194466	0.138069	0.192245	0.265776	0.200059	0.037973
Novel_001274	XLOC_01501.	transcript	0.053119	0.231295	0.098441	0.207956	0.054329	0.064197	0.08874	0.120536	0.070507
Novel_001275	XLOC_01502.	transcript	1.000259	0.327267	0.74805	0.973528	1.14625	0.145577	0.920019	0.664649	0.651099
Novel_001276	XLOC_01502.	transcript	0	0.005704	0	0.002776	0	0.247018	0.434275	0	0
Novel_001277	XLOC_01502.	transcript	0.581909	0.388219	0.350208	0.501451	0.331593	0.101157	0.224106	0.58977	0.249716
Novel_001278	XLOC_01502.	transcript	0.072022	0	0	0	0.005796	0.534829	0.201443	0.043549	0.37514
Novel_001279	XLOC_01502.	transcript	0.098317	0.011896	0.252996	0	0.021591	0.020887	0.046102	0.120649	0.047798
Novel_001280	XLOC_01504.	transcript	0.139297	0	0.019856	0.00787	0.037825	0.035407	0.043603	0.144307	0.074949

Novel_001281	XLOC_01531.	transcript	0.377359	0.477156	0.343616	0.239615	0.291229	0.424869	0.351124	0.447665	0.271839
Novel_001282	XLOC_01531.	transcript	3.361272	2.532639	1.522792	1.297556	1.466931	1.491594	2.428486	1.476971	0.853868
Novel_001283	XLOC_01537.	transcript	0.272205	1.164317	0.491146	0.164678	0.167534	0.220954	0.297394	0.267206	0.089002
Novel_001284	XLOC_01545.	transcript	0.446814	0.174773	0.808594	0.363073	0.505008	0.809918	0.508072	0.688067	0.964104
Novel_001285	XLOC_01547.	transcript	0.28916	0.380029	0	0.184421	0.152788	0.260691	0.189333	0	0.255405
Novel_001286	XLOC_01547.	transcript	0	0	0.152882	0	0	0	0	0.176054	0
Novel_001287	XLOC_01547.	transcript	0.095822	0.771308	0.089575	0.420082	0.129321	0.103638	0	0.487271	0.10959
Novel_001288	XLOC_01548.	transcript	0.325648	0.39243	0.190116	0.095298	0.094547	0.107094	0.199652	0.252673	0.207264
Novel_001289	XLOC_01548.	transcript	0.175857	0.613857	0.168768	0.049545	0.091271	0.167105	0.197995	0.091492	0.143222
Novel_001290	XLOC_01548.	transcript	0.015482	0.314326	0.054561	0.016955	0.040749	0.111998	0.077195	0.079229	0.02782
Novel_001291	XLOC_01548.	transcript	0.024482	0.149505	0.051162	0.030778	0.01252	0.043225	0.137234	0.031165	0
Novel_001292	XLOC_01548.	transcript	0.022882	0.028692	0	0	0.054595	1.510207	0.031813	0	0
Novel_001293	XLOC_01548.	transcript	0.023514	0.001403	0	0	0.037475	0.951408	0.00683	0	0.024084
Novel_001294	XLOC_01548.	transcript	0.03404	0.389803	0	0	0.021381	0	0.051895	0	0
Novel_001295	XLOC_01548.	transcript	0.014121	0.708338	0.027763	0	0	0	0	0.012353	0
Novel_001296	XLOC_01548.	transcript	0	0	0.021169	0	0	0	0.010858	0	0
Novel_001297	XLOC_01548.	transcript	0	0.030013	0	0	0.043909	0	0.009583	0	0
Novel_001298	XLOC_01548.	transcript	0	0.002369	0	0	0	0	0	0	0
Novel_001299	XLOC_01548.	transcript	0.614425	0.887456	0.815653	0.764109	0.693367	0	0.740734	0.943653	0.654017
Novel_001300	XLOC_01549.	transcript	0	0	0	0.070275	0.015158	0.021797	0	0.013064	0
Novel_001301	XLOC_01549.	transcript	0	0	0.315059	0.022406	0	0.487295	0	0.033667	0
Novel_001302	XLOC_01549.	transcript	0	0	0.049794	0.009707	0.008687	0.032693	0	0	0
Novel_001303	XLOC_01549.	transcript	0.555101	0.708637	0	0.447332	0.560023	0	0.570472	0.419544	0.399808
Novel_001304	XLOC_01559.	transcript	0.098772	0	0.019646	0.033492	0.159742	0	0.304872	0.164507	0.02368
Novel_001305	XLOC_01559.	transcript	0.376746	0.117411	0.167982	0.092213	0.224218	0.223691	0.314947	0.162309	0.263909
Novel_001306	XLOC_01572.	transcript	0.516534	0.080506	0.235034	0.738403	0.327206	0	0.902642	0.592101	1.115658
Novel_001307	XLOC_01572.	transcript	0.478931	0.377384	1.078001	1.095972	1.427122	1.445179	1.162153	1.266359	1.100747
Novel_001308	XLOC_01572.	transcript	0	0	0	0	0	0	0.598257	0	0
Novel_001309	XLOC_01572.	transcript	1.928139	0	0	0	0	0.075348	0	0	0
Novel_001310	XLOC_01573.	transcript	0.892773	0.102434	0.368493	0.363968	0.428222	0.41378	0.935737	0.687247	0.384011
Novel_001311	XLOC_01575.	transcript	0.540388	0.554323	0.099417	0.141852	0.301965	0.183245	0.904059	0.235416	2.433717
Novel_001312	XLOC_01575.	transcript	0	0	0	0	0	0	0	0	0
Novel_001313	XLOC_01578.	transcript	3.230335	1.138963	0	0	1.308345	0	0	2.146875	1.27669
Novel_001314	XLOC_01578.	transcript	0	0	0	0.364013	0.434031	1.212968	0.00338	0.114637	0.239625
Novel_001315	XLOC_01578.	transcript	0	1.873544	2.811695	1.045194	0.853341	0.463602	0.406876	0.382734	0.356603
Novel_001316	XLOC_01583.	transcript	0.173649	0.480929	0.045896	0.090475	0.17435	0.09164	0.157955	0.133288	0.120819
Novel_001317	XLOC_01588.	transcript	0	0	0	0	0	0	0	0	0
Novel_001318	XLOC_01588.	transcript	0.078074	0	0.181865	0.092183	0.098781	0.223588	0.160881	0.178996	0.087154

Novel_001319	XLOC_01590.	transcript	0.121083	0	0.064435	0.371535	0.058641	0.276578	0.239004	0.289225	0.176663
Novel_001320	XLOC_01590.	transcript	0.00553	0.033328	0.010635	0.013243	0.082569	0.020569	0.058875	0.049757	0.090644
Novel_001321	XLOC_01597.	transcript	0	0.01919	0	0	0	0	0.003056	0	0.268805
Novel_001322	XLOC_01597.	transcript	0.033483	0.011499	0.146022	0.038072	0.011621	0.036892	0	0	0.059085
Novel_001323	XLOC_01597.	transcript	0.354812	0.28926	0.327778	0.423838	0.379904	0.347818	0.57836	0.483029	0.093307
Novel_001324	XLOC_01599.	transcript	0	0	0	0	0.067201	0	0	0.117001	0.000279
Novel_001325	XLOC_01599.	transcript	0.042328	0	0.04901	0.031214	0	0	0	0.022491	0
Novel_001326	XLOC_01599.	transcript	0	0	0.042611	0.013593	0.013899	0	0.01399	0.000328	5.00E-04
Novel_001327	XLOC_01599.	transcript	0.036433	0	0.04373	0.03094	0	0	0	0.011056	0
Novel_001328	XLOC_01599.	transcript	0.017634	0	0.000279	0.007836	0.024034	0	0.001216	0.00649	0
Novel_001329	XLOC_01599.	transcript	0	0	0	0	0	0	0	0.213052	0
Novel_001330	XLOC_01599.	transcript	0.001254	0	0	0	0.291751	0	0	0.001776	0.089626
Novel_001331	XLOC_01599.	transcript	0.003984	0	0	5.00E-04	0	0	0.000785	8.90E-05	0
Novel_001332	XLOC_01599.	transcript	0.001887	0.001246	0.010697	0.002499	0.015444	0.394241	0.002835	0.000834	5.00E-05
Novel_001333	XLOC_01599.	transcript	0.007272	0	0	0	0.002597	0	0.01482	0	0
Novel_001334	XLOC_01599.	transcript	0	0	0.029099	0	0.000266	0	0.015746	1.50E-05	0
Novel_001335	XLOC_01599.	transcript	0	0	0.011464	0.004702	0.118006	4.00E-06	0	0	0
Novel_001336	XLOC_01599.	transcript	0	0	0.001161	0	0.143573	0	0	0.000848	0.000739
Novel_001337	XLOC_01599.	transcript	0.09064	0	0.453534	0.041732	1.167401	0	0.389062	1.008087	0
Novel_001338	XLOC_01599.	transcript	0	0	0.004685	0	0	0.003323	0.711917	0.115759	0
Novel_001339	XLOC_01599.	transcript	0	0	0	0	0	0	0.357654	0.130261	0
Novel_001340	XLOC_01599.	transcript	0.113384	0	0	0	0	5.00E-06	0	0	0.000419
Novel_001341	XLOC_01599.	transcript	0.087042	0	1.20E-05	0	0	1.70E-05	0	0	0.000755
Novel_001342	XLOC_01599.	transcript	0.029891	0	0.088118	0.017453	0	0	0.255194	0.070006	0
Novel_001343	XLOC_01599.	transcript	0.001964	0	0.001181	0.001781	0.128392	0.004377	0.133944	0	0
Novel_001344	XLOC_01599.	transcript	0.236447	0	0	0	3.40E-05	0	0.640758	0.000978	0.310826
Novel_001345	XLOC_01599.	transcript	0.500906	0	0	0	0.003098	0	0.448853	0	0
Novel_001346	XLOC_01599.	transcript	0	0	5.10E-05	0	0	0	0.03464	0	0
Novel_001347	XLOC_01599.	transcript	0.216038	0	0.012178	0.034983	0.002296	0.020325	0.002072	0.001903	0.006916
Novel_001348	XLOC_01599.	transcript	0	0	3.50E-05	0	0	0	0	4.50E-05	0
Novel_001349	XLOC_01599.	transcript	0	0	3.80E-05	0	0	0.311661	0	0.000125	0.114877
Novel_001350	XLOC_01599.	transcript	0.017514	0.000298	0.13549	0.218888	0.024982	1.10E-05	0.002292	0.360312	0
Novel_001351	XLOC_01599.	transcript	0	0	0.009447	0	0	0.000895	0	0.003779	0
Novel_001352	XLOC_01599.	transcript	0	0	0.01748	0.225383	0	0.009555	6.10E-05	0.003305	0
Novel_001353	XLOC_01599.	transcript	0.333438	0	0.063224	0.226491	0.0025	0.271289	0.012025	0	0
Novel_001354	XLOC_01599.	transcript	4.70E-05	0	0.000187	0	0.002715	0	0	0	0.000229
Novel_001355	XLOC_01599.	transcript	0	0	0	0	0.000577	0.000326	0	0.106794	6.00E-06
Novel_001356	XLOC_01599.	transcript	0	0	0.388336	0	0	0	0	0	0

Novel_001357	XLOC_0159…	transcript	0.515536	0	0	0.146846	0	0	0.096872	0	0
Novel_001358	XLOC_0159…	transcript	0.064843	0	0.076171	0.030931	0.000161	0.02463	0.052781	0.014707	4.70E-05
Novel_001359	XLOC_0159…	transcript	0	0	0	0	1.10E-05	0.023786	0.016994	0	0.000969
Novel_001360	XLOC_0159…	transcript	0	0	0	0	0	0.043504	0	0	0
Novel_001361	XLOC_0159…	transcript	0.058274	0	0.023573	0.029409	0	0.007191	0.014976	0.032185	0
Novel_001362	XLOC_0159…	transcript	0.155094	0	0.028527	0.074419	0	0.007291	0.021599	0.096546	0
Novel_001363	XLOC_0159…	transcript	0	0	0.167126	0.043062	0	0.017579	0.061923	0.000711	0
Novel_001364	XLOC_0159…	transcript	0	0	0.256078	0.15351	0	0.014241	0.302773	0	0.000402
Novel_001365	XLOC_0159…	transcript	0.022062	0	0.024941	0.005411	0	0.003161	0	0.11939	0
Novel_001366	XLOC_0159…	transcript	0.011122	0	0.145443	0.061978	0	0.000904	0.007382	0.000813	0.004054
Novel_001367	XLOC_0159…	transcript	0	0	0	0	0	0.107918	0.20742	0	0
Novel_001368	XLOC_0159…	transcript	0.045587	0	0	0	0	0.08327	0	0	0
Novel_001369	XLOC_0159…	transcript	0.933399	0	0.418707	0.27709	0.378024	0.362051	0.544844	0.095913	0.20864
Novel_001370	XLOC_0159…	transcript	0.12994	0	0.100342	0.044085	0	0.049152	0	0.062035	0.013635
Novel_001371	XLOC_0159…	transcript	0.036177	0	0.024875	0.040904	0.05408	0.033129	0	0.018306	0
Novel_001372	XLOC_0159…	transcript	0	0	0	0	0	0	0	0	0
Novel_001373	XLOC_0159…	transcript	0.071619	0	0.06637	0.081193	0	0.093299	0	0.061561	0
Novel_001374	XLOC_0159…	transcript	0	0	0	0	0	0.164405	0	7.00E-06	0
Novel_001375	XLOC_0159…	transcript	0.090385	0	0	0	0	0.026308	0	0	0
Novel_001376	XLOC_0159…	transcript	0	0	0	0	0	0	0.164173	0	0
Novel_001377	XLOC_0159…	transcript	0.015347	0	0	0.079284	0.238321	0	0	0	0
Novel_001378	XLOC_0159…	transcript	0.047143	0	0.19917	0.104066	0	0.178106	0	0.085558	0.001688
Novel_001379	XLOC_0159…	transcript	0	0	0	0	0	0.231595	0	0	0
Novel_001380	XLOC_0159…	transcript	0.012634	0	0.004156	0	0	0.008567	0	3.00E-06	0
Novel_001381	XLOC_0159…	transcript	0.009575	0	0.001969	0.003877	0.018608	0.003328	0.000626	0.002776	0.157116
Novel_001382	XLOC_0159…	transcript	0.579614	0	0.107263	0.091058	0.188268	0.064259	0.265934	0.107366	0.096343
Novel_001383	XLOC_0159…	transcript	0	0	0	0	0	0	0	0.023809	0
Novel_001384	XLOC_0159…	transcript	0.312645	7.10E-05	0.183771	0.047176	0.137328	0.173332	0.266375	0.198188	0.325434
Novel_001385	XLOC_0159…	transcript	0	0	0	0.049599	6.50E-05	0.000129	0	0	0
Novel_001386	XLOC_0159…	transcript	0.472474	0	0	0.036798	0	0	0.272374	0	0
Novel_001387	XLOC_0159…	transcript	2.096589	0	0.21713	0.112038	0.566363	0.194951	0.58159	0.461916	0.37859
Novel_001388	XLOC_0159…	transcript	0.000484	0	0.155385	0.001566	0.000899	0.001107	0.001483	0	0
Novel_001389	XLOC_0159…	transcript	0.231696	0	0.120612	0.08912	0.522944	0.061751	0.135404	0.128792	0.083772
Novel_001390	XLOC_0159…	transcript	0	0	0	0	0	0	0	0	0
Novel_001391	XLOC_0159…	transcript	1.621612	0	0.422197	2.00E-06	0.658834	1.153728	0.96646	0	0.150929
Novel_001392	XLOC_0159…	transcript	0	0	0.103885	0	0	0.192111	0	0.084558	0.200343
Novel_001393	XLOC_0159…	transcript	0.132897	0	0.062546	0	0	0.120967	0	0.038228	0
Novel_001394	XLOC_0159…	transcript	0.016772	0	0.010662	0	0.145103	0	0	0	0

Novel_001395	XLOC_0159.	transcript	0.049455	0	0	0	0	0.005164	0.136002	0.171058	0
Novel_001396	XLOC_0159.	transcript	0	0	0	0	0	0	0	0	0
Novel_001397	XLOC_0159.	transcript	0.002166	0	0.275168	0.000766	0.50761	0.003654	0.00165	1.276485	0.007034
Novel_001398	XLOC_0159.	transcript	1.692996	0	0.641312	0.536872	0.754918	0.833525	1.071017	0.771236	0.492562
Novel_001399	XLOC_0159.	transcript	1.040839	0	0.390841	0.351064	0.436628	0.440104	0.693651	0.379446	0.281707
Novel_001400	XLOC_0160.	transcript	0.758962	0	0.190247	0.189477	0.339924	0.402459	0.368846	0.261797	0.154722
Novel_001401	XLOC_0160.	transcript	0.157561	0.303639	0.229873	0.22968	0.296602	0.366922	0.16253	0.217573	0.10793
Novel_001402	XLOC_0161.	transcript	0.15689	0.393806	0.232567	0.365115	0.333714	0.197235	0.347299	0.264758	0.181741
Novel_001403	XLOC_0161.	transcript	0.088678	0.051197	0.057428	0.028496	0.15273	0.142034	0.155541	0.036297	0.077134
Novel_001404	XLOC_0161.	transcript	0.016208	0.020476	0.270242	0	0.009372	0.007332	0.131818	0.019608	0.021473
Novel_001405	XLOC_0161.	transcript	0.259878	0.123294	0	0.246898	0.011713	0.153613	0.152672	0.143951	0.035782
Novel_001406	XLOC_0161.	transcript	0.089885	0.223246	0.247773	0.040319	0.254568	0	0.15889	0.088577	0.092951
Novel_001407	XLOC_0161.	transcript	0.754138	0.64862	0.516383	0.491866	0.480565	0.647788	0.703501	0.451731	0.388446
Novel_001408	XLOC_0162.	transcript	0.146464	0.212476	0.378048	0	0.184022	0	0.006487	0.113265	0.364309
Novel_001409	XLOC_0162.	transcript	0.349044	0.518419	0.812044	0.925343	0.646525	0.548575	0.007639	0.026208	0.61502
Novel_001410	XLOC_0162.	transcript	0	0	0	0	0	0.81453	0	0	0.001569
Novel_001411	XLOC_0162.	transcript	0.193091	0.605449	0.528174	0.24471	0.580761	0.320866	0.757999	1.12362	0.482101
Novel_001412	XLOC_0162.	transcript	0.150596	0.088625	0.141033	0	0.115983	0	0.272613	0.245405	0.202027
Novel_001413	XLOC_0162.	transcript	0.415058	0	0	0	0	0	0	0	0
Novel_001414	XLOC_0162.	transcript	0	0.024248	0	0	0	0	0	0	0
Novel_001415	XLOC_0162.	transcript	0	0	0	0	0	0	1.029317	0	0
Novel_001416	XLOC_0162.	transcript	0.150108	0.88797	0.038031	0.02196	0.062141	0.046597	0.161438	0.02871	0.16723
Novel_001417	XLOC_0163.	transcript	0.038318	0.039538	0.339977	0	0	0.438241	0.368717	0.067081	0.084175
Novel_001418	XLOC_0163.	transcript	0.810078	0.829854	0.058344	0.488664	0.445058	0.06102	0.283201	0.647754	0.481297
Novel_001419	XLOC_0163.	transcript	0.088746	0.66964	0.069542	0	0	0	0.163796	0.521693	0
Novel_001420	XLOC_0163.	transcript	0.458351	0.020494	0.374107	0.514212	0.316238	0.207194	0.335972	0.028551	0.225864
Novel_001421	XLOC_0163.	transcript	0	0.032705	0	0	0	0.521485	0.017262	0.00012	0.292404
Novel_001422	XLOC_0163.	transcript	0	0	0.283397	0	0	0.111618	0.000907	0.00079	0.107226
Novel_001423	XLOC_0163.	transcript	0.825192	0	0.335118	0.29806	0.887616	0.857565	0.293638	0.402422	0.274252
Novel_001424	XLOC_0163.	transcript	1.00E-06	0.013119	0	0	0	0	0	0	0
Novel_001425	XLOC_0163.	transcript	0.229224	0.016931	0.057132	0.071237	0	0.704413	0.213514	0.419433	0.092756
Novel_001426	XLOC_0163.	transcript	0.000959	0.009751	0.026958	0.175843	0.066188	0.000995	0.508484	0.094064	0
Novel_001427	XLOC_0163.	transcript	0.228993	0	0.076563	0	0.118552	0.186459	0.055246	0.339746	0.213169
Novel_001428	XLOC_0163.	transcript	0.283651	0.04083	0.32574	0.115055	0.303896	0.348188	0.396546	0.240432	0.099626
Novel_001429	XLOC_0163.	transcript	0.378004	0	0	0	0	0	0.000257	0	0
Novel_001430	XLOC_0164.	transcript	0.513445	0.174586	0.404364	0.463573	0.29198	0.363468	0.386461	0.651864	0.274288
Novel_001431	XLOC_0164.	transcript	0.050593	0	0.141739	0.121726	0.240561	0.107363	0.079255	0.102343	0.026789
Novel_001432	XLOC_0164.	transcript	0.15547	0	0	0.118573	0.050435	0.064656	0	0	0

Novel_001433	XLOC_01653.	transcript	0.049975	0.209343	0.251189	0.436625	0.372224	0.468378	0.19018	0.319323	0.125469
Novel_001434	XLOC_01655.	transcript	0.016542	0.364464	0	0	0.006767	0	0.038984	0.011365	0.007842
Novel_001435	XLOC_01655.	transcript	0.010509	0.369165	0	0.010208	0	0	0	0	0
Novel_001436	XLOC_01655.	transcript	0	0	0	0	0	0	0	0	0.489511
Novel_001437	XLOC_01658.	transcript	0.077161	0.026293	0.014939	0.017818	0.00659	0	0.094489	0.358561	0.033471
Novel_001438	XLOC_01658.	transcript	0.285847	0.527425	0.41557	0.518531	0.410028	0.484806	0.495655	0.255581	0.350249
Novel_001439	XLOC_01668.	transcript	0.186795	2.227704	0.07733	0.057189	0.044775	0.05434	0.221458	0.038894	0.141581
Novel_001440	XLOC_01677.	transcript	0	0	0	0.572126	0.163732	0.098517	0.23657	0	0.071361
Novel_001441	XLOC_01677.	transcript	0.09244	0	0.049951	0.00521	0.010145	0.060251	0.335486	0.032451	0
Novel_001442	XLOC_01677.	transcript	0	0	0.019871	0	0.161813	0	0	0	0
Novel_001443	XLOC_01677.	transcript	1.190283	0	0.261979	0.313488	0.300924	0	0.434797	0.323396	0
Novel_001444	XLOC_01677.	transcript	1.01221	0	0.486132	0.466777	0.762588	0.273274	0.92647	0.362166	0.330103
Novel_001445	XLOC_01677.	transcript	0.036693	0.178426	0.097815	0.051307	0.080343	0.501071	0.783881	0	0.210145
Novel_001446	XLOC_01677.	transcript	0	0	0.835178	0.285754	0.138252	0.81791	0.890805	0.929244	0.406848
Novel_001447	XLOC_01677.	transcript	0	0	0.109513	0.287511	0.250593	0	0.268212	0	0
Novel_001448	XLOC_01678.	transcript	2.122097	0.413004	2.961075	3.91268	3.059881	3.729477	3.067488	2.736371	2.639327
Novel_001449	XLOC_01678.	transcript	0	0	0	0	0	0	0	0	0
Novel_001450	XLOC_01694.	transcript	0.509471	0.184876	0.095706	0.028265	0.390341	0.087189	0.374505	0.357486	0.039072
Novel_001451	XLOC_01694.	transcript	0	0	0.043836	0.534025	0	0.221404	0.028251	0.016584	0.556742
Novel_001452	XLOC_01694.	transcript	0.040155	0.023991	0.298959	0.050957	0.01141	0.27458	0.026428	0	0.016148
Novel_001453	XLOC_01698.	transcript	1.649397	0	2.713513	2.110171	1.301864	2.009516	2.635522	2.982071	0
Novel_001454	XLOC_01698.	transcript	0.497018	0.610919	0.002506	0	0	0.014558	0.015871	0.014754	0.968896
Novel_001455	XLOC_01698.	transcript	0.507765	0	2.041996	2.215389	0.91588	1.414376	1.651945	0.862845	2.534256
Novel_001456	XLOC_01698.	transcript	0.899442	0	0	0	2.319418	0	0	0	0
Novel_001457	XLOC_01703.	transcript	0.000807	0	0.009045	0.001542	0.832578	0.015436	0.044367	0.004871	0.008108
Novel_001458	XLOC_01703.	transcript	0.977417	0.814767	0.988333	1.051379	0.349639	1.103936	1.932202	1.13206	1.381906
Novel_001459	XLOC_01703.	transcript	0	0	0	0	0	0	0	0	0.356909
Novel_001460	XLOC_01704.	transcript	0	0.031353	0	0	0	0	0	0	0
Novel_001461	XLOC_01704.	transcript	0	0.145382	0	0.207484	0	0	0.915918	0.461088	0.096638
Novel_001462	XLOC_01704.	transcript	0	0	0.104956	0	0	0.189434	0	0	0
Novel_001463	XLOC_01704.	transcript	0.006158	0	0.038667	0	0.320375	0.136423	0	0	0.000251
Novel_001464	XLOC_01704.	transcript	0.043241	0.080749	0	0	0	0	0	0.007741	0.153
Novel_001465	XLOC_01704.	transcript	0	0.077226	0.187736	0	0	0.163647	0	0	0
Novel_001466	XLOC_01704.	transcript	0	0.091349	0	0.09506	0.037214	0	0.036212	0	0.108752
Novel_001467	XLOC_01704.	transcript	0	0.008584	0	0	0	0	0	0	0
Novel_001468	XLOC_01704.	transcript	0	0.08486	0	0	0	0	0	0	0.126641
Novel_001469	XLOC_01704.	transcript	0	0	0	0	0	0	0	0	0
Novel_001470	XLOC_01704.	transcript	0.20782	0.209625	0.038183	0.024124	0.044395	0.072099	0.012788	0.006058	0

Novel_001471	XLOC_01704.	transcript	0.17974	0.152163	0	0	0	0	0	0	0.14042
Novel_001472	XLOC_01704.	transcript	0.109804	0.129114	0	0	0	0	0.025169	0.028411	0.120321
Novel_001473	XLOC_01704.	transcript	0.204324	0.112448	0	0.257176	0	0	0	0	0.075886
Novel_001474	XLOC_01704.	transcript	0.348493	0.239569	0	0	0	0	0	0	0.120444
Novel_001475	XLOC_01704.	transcript	0.319018	0.194641	0	0	0	0	0	0	0.095226
Novel_001476	XLOC_01704.	transcript	0	0	0.016547	0.000214	0	0.062116	0.437185	0.229145	0
Novel_001477	XLOC_01704.	transcript	0	0	0.020452	0	0	0.073302	0	0	0
Novel_001478	XLOC_01704.	transcript	0.000182	0	0.011327	0	0	0.060845	0	0	0.00554
Novel_001479	XLOC_01704.	transcript	0.240638	0.110539	0.202662	0	0	0.246222	0	0	0.19184
Novel_001480	XLOC_01704.	transcript	0.329425	0.309124	1.053161	0	0	0.688773	0	0	0.19739
Novel_001481	XLOC_01704.	transcript	0.116256	0.080609	0.171837	0	0	0.186555	0.015239	0	0.166004
Novel_001482	XLOC_01704.	transcript	0.229253	0.354022	0	0	0	0.187344	0	0.035624	0.150861
Novel_001483	XLOC_01704.	transcript	0.201313	0.108143	0.261663	0	0	0.131532	0	0	0.102977
Novel_001484	XLOC_01704.	transcript	1.122988	0.312676	0.263083	0.081446	0.342667	0.340647	0.694808	0.353019	0.32984
Novel_001485	XLOC_01704.	transcript	0.083791	0.133301	0.157616	0.337775	0.04459	0.158911	0.205821	0.203511	0
Novel_001486	XLOC_01704.	transcript	0.246301	0.24282	0.117512	0.142011	0.152598	0.177293	0.242638	0.079054	0
Novel_001487	XLOC_01715.	transcript	0.172247	0.393717	0.217268	0.316564	0.375603	0.339109	0.281064	0.180929	0
Novel_001488	XLOC_01716.	transcript	0.033927	0.017547	0.045519	0.073355	0.0026	0.031402	0.110015	0.170123	0.02693
Novel_001489	XLOC_01716.	transcript	0.424631	0.488991	1.154271	0.502261	0.864275	0.835603	0.749646	0.899804	0.95198
Novel_001490	XLOC_01716.	transcript	0	0	0	0	0.162759	0.244145	0.53985	0	0
Novel_001491	XLOC_01716.	transcript	0	0	0.287364	1.285285	0.348019	0	0	0.58119	0
Novel_001492	XLOC_01716.	transcript	0.56224	0.549413	0	0	0	0	0	0	0
Novel_001493	XLOC_01716.	transcript	0.230451	0.033029	0.409348	0.257419	0.327856	0.064915	0.008119	0.129008	0.047388
Novel_001494	XLOC_01716.	transcript	0	0	0	0	0	0.180266	0	0.254392	0
Novel_001495	XLOC_01717.	transcript	0	0.012959	0	0	0	0	0.214522	0.044961	0
Novel_001496	XLOC_01717.	transcript	0.522508	0.322343	0.503114	0.453559	0.493355	0.586897	0.752719	0.499493	0.302536
Novel_001497	XLOC_01717.	transcript	0.506926	0.493387	0.556466	0.378606	0.473819	0.454987	0.482503	0.545033	0.507742
Novel_001498	XLOC_01717.	transcript	0	0	0	0	0	0	0.219323	0.266193	0
Novel_001499	XLOC_01719.	transcript	0.323065	0.330755	0.004636	0.634478	0.662353	0	0.723732	0.340904	0.650124
Novel_001500	XLOC_01719.	transcript	0.309411	0.200936	0.052791	0	0	0.612423	0	0.190301	0
Novel_001501	XLOC_01719.	transcript	0.482276	0	0.284151	0.012329	0	0	0.036548	0.463149	0
Novel_001502	XLOC_01719.	transcript	0	0.981501	0.687114	0	0	0	0	0	0
Novel_001503	XLOC_01720.	transcript	0.01507	0	0	0	0.048876	0.001569	0	0.097491	0.008324
Novel_001504	XLOC_01720.	transcript	0.285044	0.403121	0.378104	0.488355	0.322364	0.523644	0.297078	0.345695	0.477487
Novel_001505	XLOC_01720.	transcript	0.158374	0.421102	0.108975	0.097364	0.061386	0.082526	0.366662	0.049449	0.337812
Novel_001506	XLOC_01720.	transcript	0	0	0	0	0	0	0	0	0
Novel_001507	XLOC_01720.	transcript	0	0	0	0	0	0	0	0	0
Novel_001508	XLOC_01721.	transcript	0.182827	0.374329	0.302048	0.181756	0.351373	0.205196	0.455012	0.37135	0.135916

Novel_001509	XLOC_01722.	transcript	0.243541	0.324469	0.106137	0.252601	0.371368	0.218141	0.297329	0.264195	0.117093
Novel_001510	XLOC_01727.	transcript	0.275814	0	0	0.168058	0	0	0.158849	0	0
Novel_001511	XLOC_01727.	transcript	0.270743	0	0	0.236832	0.008212	0.004502	0.24462	0.002078	0
Novel_001512	XLOC_01727.	transcript	0	0.133806	0.182417	0.143143	0.32469	0.039397	0.160322	0.201108	0.282715
Novel_001513	XLOC_01727.	transcript	0	0	0.411483	0.385053	0.39126	0	0.236645	0.25113	0.535936
Novel_001514	XLOC_01727.	transcript	0.272385	0.13592	0	0.022462	0	0.359212	0.117691	0.11542	0.233116
Novel_001515	XLOC_01727.	transcript	0.016485	0.364691	0	0	0	0.115884	0.288169	0.11764	0.200133
Novel_001516	XLOC_01727.	transcript	0.143392	0.219488	0.555518	0.011798	0.658021	0.25172	0.365274	0.13521	0.01543
Novel_001517	XLOC_01727.	transcript	0.138799	0.169131	0	0.04619	0.08863	0.198669	0.104971	0.136454	0.248214
Novel_001518	XLOC_01728.	transcript	0.019218	0.497041	0.110424	0.144728	0.082257	0.249041	0.201457	0.231343	0.237439
Novel_001519	XLOC_01729.	transcript	0.226444	0.404636	0.470875	0.858593	0.350162	0.308907	0.345559	0.717398	0.50419
Novel_001520	XLOC_01734.	transcript	0.161152	0.529337	0.279122	0.297513	0.206594	0.260975	0.358449	0.126906	0.26398
Novel_001521	XLOC_01734.	transcript	0.611577	2.01699	0.78904	0.841322	0.486728	0.833967	0.914145	0.802918	1.179092
Novel_001522	XLOC_01734.	transcript	0	0	0.067073	0	0	0	0	0	0
Novel_001523	XLOC_01734.	transcript	0.048186	0.195473	1.411798	0.199171	0.78386	1.311461	0.685934	0.541776	1.388888
Novel_001524	XLOC_01734.	transcript	0.888635	0	0	1.064094	0.47053	0.495768	0.954283	0.54892	0
Novel_001525	XLOC_01735.	transcript	0.027815	0.333723	0.114952	0.114912	0.111209	0.100002	0.081156	0.228853	0
Novel_001526	XLOC_01735.	transcript	0.306471	0	0	0	0	0	0	0	0
Novel_001527	XLOC_01735.	transcript	0	0.047659	0.045675	0.649554	0.142258	0.015993	0.191521	0.14437	0
Novel_001528	XLOC_01737.	transcript	0.755115	0.391649	0.800957	1.451455	1.007126	0.576254	0.977959	1.021002	0.973891
Novel_001529	XLOC_01737.	transcript	0	0.00397	0.226067	0	0	0	0	0	0.132309
Novel_001530	XLOC_01737.	transcript	0	0	0	0	0	0	0	0	0
Novel_001531	XLOC_01737.	transcript	0.143883	0	0.052003	0.257633	0.337742	0.26733	0.363272	0.209552	0
Novel_001532	XLOC_01747.	transcript	0	0.040852	0	0.51791	0	0.665164	0	0.011183	0
Novel_001533	XLOC_01747.	transcript	5.263656	3.542654	6.106369	7.178277	7.39631	7.487755	5.092499	8.395615	6.514689
Novel_001534	XLOC_01748.	transcript	0	0	0	0	0	0	0.303405	0.036234	0
Novel_001535	XLOC_01748.	transcript	0.349475	0	0	1.969695	1.555983	0.621828	1.136209	0	0.405598
Novel_001536	XLOC_01748.	transcript	0.807349	0.444229	6.864761	5.697099	3.581512	8.473819	4.137521	4.077429	7.8236
Novel_001537	XLOC_01756.	transcript	0.105351	0.099033	0.059058	0	0	0	0.089068	0.067017	0.39663
Novel_001538	XLOC_01758.	transcript	1.549556	0.34475	0.926377	0.658652	1.470999	0.770383	1.107247	1.583688	1.040528
Novel_001539	XLOC_01758.	transcript	0.009536	0	0	0.015884	0.015816	0	0	0	0.002256
Novel_001540	XLOC_01758.	transcript	0	0	1.836295	0.547112	0.444934	1.591906	0.514025	0.89512	0.418123
Novel_001541	XLOC_01758.	transcript	0	0	0	0.002378	0	0	0	0	0
Novel_001542	XLOC_01763.	transcript	0	3.464268	0	0	1.025288	0.067212	0.060456	0	0.216479
Novel_001543	XLOC_01765.	transcript	1.167101	0.806681	1.571317	1.593575	1.725949	1.766431	2.067483	2.115054	1.98639
Novel_001544	XLOC_01768.	transcript	136.8467	180.9836	249.5214	255.9671	243.0896	328.017	215.8044	188.6353	286.0403
Novel_001545	XLOC_01770.	transcript	0.32922	0.755513	0.336	0.461059	0.388493	0.421719	0.740195	0.439487	0.394307
Novel_001546	XLOC_01771.	transcript	0.37374	0	0.530283	0.663349	1.00543	0.415238	0.278796	0.490195	0.412097

Novel_001547	XLOC_01771.	transcript	0	0.022064	0	0	0	0	0	0.404702	0
Novel_001548	XLOC_01774.	transcript	0	0	0.292561	0.121737	0.057762	0	0.021066	0.022504	0
Novel_001549	XLOC_01774.	transcript	0.247931	0.246818	0.032433	0.852617	0.389437	0.598138	0.427545	0.9591	0.263882
Novel_001550	XLOC_01774.	transcript	0	0.072266	0.036873	0.087109	0.178361	0	0.052169	0.29481	0
Novel_001551	XLOC_0177[illegible].	transcript	0.610833	0.018637	0.388642	0.213369	0.373316	0.269452	0.846367	0.209248	0.073225
Novel_001552	XLOC_0177[illegible].	transcript	0.355046	0.57441	0.804736	0	0.414619	0	0.011701	0.167997	0
Novel_001553	XLOC_0177[illegible].	transcript	0.526996	0.637811	0	0.160798	0.408497	0.885746	0	0.117228	0.478631
Novel_001554	XLOC_0177[illegible].	transcript	0.176312	0.320884	0.065349	0	0.431847	0.303008	1.181474	0.622612	0.221204
Novel_001555	XLOC_0177[illegible].	transcript	0	0	0.255245	0.880361	0	0	0	0.485221	0
Novel_001556	XLOC_0177[illegible].	transcript	0	0	0	0	0	0	0	0.006736	0
Novel_001557	XLOC_0177[illegible].	transcript	0	0	0	0	0.843396	0	0	0	0
Novel_001558	XLOC_0177[illegible].	transcript	2.576998	0.810037	2.00333	1.994976	1.791241	2.096236	2.577651	2.473169	2.256217
Novel_001559	XLOC_0177[illegible].	transcript	1.199967	1.180913	2.038686	2.597883	2.176562	3.460763	1.789094	2.165502	3.324245
Novel_001560	XLOC_0177[illegible].	transcript	1.592118	1.491935	1.569669	1.588547	1.490189	1.410397	1.938649	1.783729	1.776987
Novel_001561	XLOC_0177[illegible].	transcript	0.364518	0.295536	0.489796	0.464574	0.629287	0.452867	0.875594	0.640916	0.404735
Novel_001562	XLOC_0178[illegible].	transcript	0	0	0.248985	0.722468	0.312247	0.164108	0	0.606656	0.84797
Novel_001563	XLOC_0178[illegible].	transcript	0.358407	0.210334	0.297139	0.271274	0.571559	0.372375	1.210031	0.324876	0.247266
Novel_001564	XLOC_0178[illegible].	transcript	0	0	0	0	0	0	0	0	0
Novel_001565	XLOC_01782.	transcript	0.245158	0.293552	0.281769	0.372467	0.298458	0.312492	0.322712	0.326984	0.350086
Novel_001566	XLOC_0178[illegible].	transcript	1.597403	1.218138	2.401929	2.654361	1.655553	2.632483	2.447334	2.652885	2.056974
Novel_001567	XLOC_0178[illegible].	transcript	0.394826	0.53948	0.193249	0.206539	0.224374	0.298023	0.146779	0.342379	0.100997
Novel_001568	XLOC_01784.	transcript	0.161496	0	0.093921	0	0	0.135587	0.020248	0.100086	0
Novel_001569	XLOC_01784.	transcript	0.007587	0.854995	0.095601	0.588329	0	0	0	0.080969	0.473628
Novel_001570	XLOC_01784.	transcript	0	0	0.305216	0	0	0	0.224922	0.074683	0
Novel_001571	XLOC_01784.	transcript	0	0.083332	0.097098	0.09932	0	0.613592	0.096924	0.077006	0.098601
Novel_001572	XLOC_01784.	transcript	0.594905	0.055464	0.061036	0.18995	0.265138	0.33952	0.078904	0.048066	0.071546
Novel_001573	XLOC_01784.	transcript	0	0	0	0	0	0	0	0	0
Novel_001574	XLOC_01784.	transcript	0.2946	0.496802	0.260802	0.576082	1.053414	0.642212	0.486966	0.551591	0.404954
Novel_001575	XLOC_01784.	transcript	0	0	0.195072	0	0	0	0	0	0
Novel_001576	XLOC_01784.	transcript	0	0	0.204981	0	0	0	0	0	0
Novel_001577	XLOC_01784.	transcript	0.705357	2.045248	2.520435	2.142011	2.272889	2.849645	1.518385	1.886302	1.234887
Novel_001578	XLOC_01784.	transcript	0.298451	0	0.211146	0	0.518444	0	0.453411	0.460637	0
Novel_001579	XLOC_0178[illegible].	transcript	0	0.380157	0.134698	0.065519	0	0	0.395062	0.05606	0.409691
Novel_001580	XLOC_0178[illegible].	transcript	0.249394	2.279975	0	0.120264	0.319851	0.188055	0.143828	0.131638	0.486377
Novel_001581	XLOC_01787.	transcript	0.003281	0.00408	0.012192	0.018817	0.043365	0.154571	0.20566	0.013066	0.08406
Novel_001582	XLOC_01787.	transcript	0.002549	0.117263	1.30E-05	3.40E-05	3.40E-05	0	0.245281	0	0
Novel_001583	XLOC_01787.	transcript	0	0.513874	0.066093	0.263454	0	0.248037	0.219504	0.276599	0.00751
Novel_001584	XLOC_01787.	transcript	0.35632	0	0.390558	0.156537	0.485735	0.062906	0.003446	0.070768	0.437951

Novel_001585	XLOC_01787.	transcript	0	0	0	0	0	0	0	0.048123	0
Novel_001586	XLOC_01788.	transcript	0.184801	0.927234	0.238828	0.157485	0.279521	0.211747	0.301886	0.244466	0.453716
Novel_001587	XLOC_01789.	transcript	0.106197	0.045908	0.158342	0.168275	0.296461	0.199422	0.337891	0.242533	0.201
Novel_001588	XLOC_01790.	transcript	0	0	0	0	0	0	0	0	0
Novel_001589	XLOC_01790.	transcript	0.490751	0.452337	0.732034	0.682982	0.476349	0.353298	0.494081	0.308641	0.814328
Novel_001590	XLOC_01790.	transcript	0.021519	0.328506	0	0.190504	0.318544	0.252485	0.298542	0.27835	0.262185
Novel_001591	XLOC_01790.	transcript	0.399928	0.231994	0.593422	0.543387	0.322746	0.417492	0.466185	0.358269	0.247648
Novel_001592	XLOC_01791.	transcript	0.064555	0.824516	0.030721	0.032154	0.135875	0.050746	0.127622	0.05114	0.060658
Novel_001593	XLOC_01791.	transcript	0.013414	0.115927	4.80E-05	0	0	0	0.060336	0	0.02781
Novel_001594	XLOC_01791.	transcript	1.074647	0.70404	0.964506	0.657586	0.909766	0.818576	1.961894	1.132192	0.738958
Novel_001595	XLOC_01793.	transcript	0.312258	0.103438	0.188614	0.016813	0.063612	0.203236	0.218651	0.112724	0.112168
Novel_001596	XLOC_01793.	transcript	0.260232	0.368557	0.18984	0.382576	0.244927	0.282939	0.5026	0.333128	0.352236
Novel_001597	XLOC_01794.	transcript	0.494921	0.276758	0.452729	0.474486	0.353051	0.637729	0.563321	0.426371	0.617061
Novel_001598	XLOC_01794.	transcript	0.020808	0.225215	0.010802	0.015759	0	0.015588	0.04896	0.021726	0.06548
Novel_001599	XLOC_01794.	transcript	0.112609	0.479021	0.058103	0.08531	0.102512	0.080184	0.105555	0.090151	0.179561
Novel_001600	XLOC_01794.	transcript	0	0.348134	0	0	0	0	0	0	0
Novel_001601	XLOC_01794.	transcript	0.145588	0.2207	0.293689	0.16053	0.484914	0.242674	0.378863	0.408019	0.128965
Novel_001602	XLOC_01796.	transcript	0.016925	0.014125	0	0	0	0.086678	0.126595	0.139817	0
Novel_001603	XLOC_01796.	transcript	0.093061	0.076395	0.190047	0.110884	0.242675	0.061403	0.032242	0.038962	0.17351
Novel_001604	XLOC_01797.	transcript	0.125378	0.163029	0	0.16297	0	0.260602	0.004098	0	0.065754
Novel_001605	XLOC_01797.	transcript	0	0	0.092355	0.040124	0.117399	0	0.282227	0.415876	0
Novel_001606	XLOC_01797.	transcript	0	0	0	0	0	0	0.002876	0	0.221624
Novel_001607	XLOC_01798.	transcript	0.013585	0.181855	0.131664	0.055913	0.096239	0.029923	0.075433	0.008056	0.029426
Novel_001608	XLOC_01798.	transcript	0	0	0.053382	0.026787	0.328141	0.290258	0.211973	0	0.285409
Novel_001609	XLOC_01798.	transcript	0.072265	0.022051	0	0	0	0	0	0.298976	0
Novel_001610	XLOC_01798.	transcript	0	0	0.01004	0	0	0	0	0	0
Novel_001611	XLOC_01798.	transcript	0.561911	1.197574	0.844207	0.736188	0.909936	1.020142	1.124358	1.0632	0.539482
Novel_001612	XLOC_01798.	transcript	0.209273	0.292175	0.188705	0.148713	0.165758	0.148014	0.265392	0.245875	0.129351
Novel_001613	XLOC_01799.	transcript	0.432932	0.657269	0.457853	0.461539	0.413371	0.507496	0.889226	0.488761	0.513329
Novel_001614	XLOC_01799.	transcript	0.328919	0.976818	0.244039	0.366233	0.411638	0.355063	0.700839	0.429688	0.48249
Novel_001615	XLOC_01800.	transcript	0	0	0	0	0.082289	0	0	0.047574	0
Novel_001616	XLOC_01809.	transcript	0	0	0	0	0	0	0	0	0.596291
Novel_001617	XLOC_01809.	transcript	1.008603	0.343935	0.795394	1.077294	1.283451	0.863339	1.699073	1.675064	0.696047
Novel_001618	XLOC_01809.	transcript	0.457112	0.366091	0.495716	0.212796	0.433097	0.271216	0.68954	0.415931	0.244661
Novel_001619	XLOC_01812.	transcript	0	0.272726	0.151173	0.044806	0.353723	0.057441	0.0236	0.16679	0.031626
Novel_001620	XLOC_01813.	transcript	0.143474	0.127668	0.306507	0	0.142586	0	0.234589	0.001973	0.018171
Novel_001621	XLOC_01813.	transcript	0	0	0	0.228245	0	0.191723	0	0.443993	0
Novel_001622	XLOC_01815.	transcript	0.538664	0.760687	0.921378	0.996955	0.622596	0.896768	1.015972	0.471605	0.51259

Novel_001623	XLOC_01815.	transcript	0	0	0.054969	0.036523	0.532502	0.240146	0.274432	0.88716	0.414344
Novel_001624	XLOC_01816.	transcript	0	0	0.370817	0.05892	0.489319	0	0.228221	0.373014	0
Novel_001625	XLOC_01816.	transcript	0.350199	0	0	0	0	0	0	0.087457	0
Novel_001626	XLOC_01816.	transcript	0	0.412857	0.315199	0.181101	0	0	0	0	0
Novel_001627	XLOC_01816.	transcript	0.013707	0	0.003496	0.328498	0.259704	0.586378	0.317593	0.259282	0.382011
Novel_001628	XLOC_01816.	transcript	0	0.901752	0.441476	0.660078	0.510566	0.570318	0.732313	0.694704	0
Novel_001629	XLOC_01816.	transcript	0.285013	0	0	0	0	0	0	0	0.21398
Novel_001630	XLOC_01816.	transcript	0	0	0.008033	0.02401	0	0.02103	0.041499	0	0.39808
Novel_001631	XLOC_01816.	transcript	0	0	0	0	0	0	0	0	0
Novel_001632	XLOC_01816.	transcript	0.824717	0.362807	0.508487	0.685946	0.28118	0.893162	0.941149	0.950648	0.286363
Novel_001633	XLOC_01816.	transcript	0	0	0.218491	0	0.469143	0	0	0	0
Novel_001634	XLOC_01816.	transcript	0.009775	0.982099	0.024325	0.130416	0.172739	0.042926	0.263212	0.041433	0.008989
Novel_001635	XLOC_01816.	transcript	0.228837	3.093644	0.226492	0.220774	0.24365	0.295149	0.481394	0.122103	0.481908
Novel_001636	XLOC_01817.	transcript	0.114382	0.094546	0.130835	0.077305	0.069881	0.027601	0.171034	0.170752	0.081334
Novel_001637	XLOC_01818.	transcript	0.044739	0	0.301127	0.137767	0.230742	0.168969	0.281843	0.159539	0.193526
Novel_001638	XLOC_01818.	transcript	0	0	0	0.014614	0	0.104096	0	0.06784	0.129265
Novel_001639	XLOC_01819.	transcript	0.272518	0.374551	0.235025	0.137908	0.344586	0.2301	0.325851	0.273042	0.364873
Novel_001640	XLOC_01821.	transcript	0.018702	0.23157	0.197719	0.094098	0.114768	0.045129	0.036564	0	0.066492
Novel_001641	XLOC_01821.	transcript	0.058726	0.058126	0.086937	0.014775	0.060104	0.056674	0.149342	0.148019	0.102351
Novel_001642	XLOC_01822.	transcript	0.220505	0.118368	0.325736	0.371216	0.340737	0.221994	0.516598	0.362904	0.320596
Novel_001643	XLOC_01822.	transcript	0	0	0	0	0	0	0	0	0
Novel_001644	XLOC_01829.	transcript	0.061231	0.172496	0.227434	0.149284	0.170171	0.133418	0.132586	0.056936	0.087371
Novel_001645	XLOC_01830.	transcript	0.153119	0.088209	0	0.065742	0.097521	0.187699	0.172066	0	0
Novel_001646	XLOC_01830.	transcript	0.011432	0.012096	0.20529	0	0.057074	0	0	0.276988	0.355455
Novel_001647	XLOC_01831.	transcript	2.952299	3.945142	4.384439	4.669951	5.77692	5.455791	5.924009	4.720275	4.65281
Novel_001648	XLOC_01836.	transcript	0.052421	0.1548	0.135281	0.347204	0.255033	0.196716	0.368542	0.225806	0.13783
Novel_001649	XLOC_01836.	transcript	0	0	0	0	0	0	0	0	1.087116
Novel_001650	XLOC_01836.	transcript	0.099731	0	0	0	0	0	0	0	0
Novel_001651	XLOC_01836.	transcript	0	0	0	0	0	0	0	0	0
Novel_001652	XLOC_01836.	transcript	0	0	0	0	0	0	0	0	0
Novel_001653	XLOC_01836.	transcript	0	0	0	0	0	0	0	0	0
Novel_001654	XLOC_01836.	transcript	0	0	0	0	0	0	0	0	0
Novel_001655	XLOC_01837.	transcript	0.078296	0.045934	0.114797	0.082421	0.085354	0.079428	0.044797	0.026205	0.151992
Novel_001656	XLOC_01837.	transcript	0.01942	0	0	0	0.016754	0	0	0.074391	0
Novel_001657	XLOC_01837.	transcript	0	0	0.138645	0.170427	0.206725	0.081383	0.317875	0.046179	0
Novel_001658	XLOC_01837.	transcript	0	0	0	0.240251	0.266379	0	0	0.362186	0
Novel_001659	XLOC_01837.	transcript	0	0.270105	0.51821	0.267267	0.320338	0.983975	0.632649	0.824235	0
Novel_001660	XLOC_01837.	transcript	0.202365	0.705795	0	0.118287	0.133254	0	1.140719	0.295535	0

Novel_001661	XLOC_01837.	transcript	0.170553	0.178169	0.000832	0	0	0.070906	0	0.141625	0
Novel_001662	XLOC_01837.	transcript	0.376442	0	0	0	0	0.002459	0	0	0
Novel_001663	XLOC_01837.	transcript	0.216886	0.173044	0.27491	0.328416	0	0.001317	0.110193	0.190114	0.004976
Novel_001664	XLOC_01837.	transcript	0.16155	0.21878	0	0.121783	0	0.014705	0.197108	0.185988	0.433931
Novel_001665	XLOC_01837.	transcript	0.219229	0.221134	0.375818	0.299031	0.443518	0.095201	0.198758	0.339271	0.582281
Novel_001666	XLOC_01837.	transcript	0.257186	0.172269	0.337448	0.587975	0.31086	0.006237	0.109506	0.277452	0
Novel_001667	XLOC_01837.	transcript	0.269499	0.170663	0.104904	0.182935	0.416177	0.132121	0.144858	0.202562	0.024936
Novel_001668	XLOC_01837.	transcript	0.267403	0.20519	0.244154	0.221054	0.402322	0.179928	0.150741	0.291871	0.619501
Novel_001669	XLOC_01840.	transcript	0.228645	0.010573	0.157075	0.201537	0.505938	0.15412	0.46193	0.131421	0.307811
Novel_001670	XLOC_01840.	transcript	0	0	0	0	0	0	0	0	0
Novel_001671	XLOC_01844.	transcript	0	0	0	0	0	0	0	1.247893	0
Novel_001672	XLOC_01844.	transcript	0.662408	0.011853	1.459651	1.886693	1.161431	1.430061	0.996625	0.964141	0.809316
Novel_001673	XLOC_01844.	transcript	0.44149	0	0.447073	0.66491	0.568284	0	0.537949	0.136517	0.320434
Novel_001674	XLOC_01844.	transcript	0.074306	0.018656	0.027611	0.025355	0.011504	0.584786	0.159565	0.391676	0.13855
Novel_001675	XLOC_01851.	transcript	0.421798	1.517932	0.329438	0.005615	0.230415	0	0.406204	0.003808	0.336554
Novel_001676	XLOC_01851.	transcript	0.161402	0.282053	0.135471	0.341884	0.01836	0.427491	0.1049	0.039878	0.022342
Novel_001677	XLOC_01851.	transcript	0	0	0	0	0	0.102606	0	0.574558	0
Novel_001678	XLOC_01851.	transcript	0.137701	0.009015	0.139694	0	0.132573	0.001589	0.207806	0.0289	0.064643
Novel_001679	XLOC_01853.	transcript	0.032464	0.006693	1.250529	0	0	0.588159	0.030776	0	1.185491
Novel_001680	XLOC_01853.	transcript	0.207605	0.017074	0.154564	0.054859	0.076724	0.052826	0.155184	0.088887	0.140603
Novel_001681	XLOC_01855.	transcript	0.492271	0.404684	1.265612	11.487	4.836091	5.892346	4.293591	1.636313	5.223938
Novel_001682	XLOC_01855.	transcript	2.028626	0.881885	3.640596	28.35639	11.79204	5.221472	4.931914	14.95807	0.27128
Novel_001683	XLOC_01856.	transcript	2.938104	1.027773	3.18706	3.070021	3.023142	4.248024	3.405475	3.344165	5.056358
Novel_001684	XLOC_01858.	transcript	0	0	0.025334	0.016376	0.038724	0	0.011768	0	0
Novel_001685	XLOC_01858.	transcript	0.01418	0.074476	0.012261	0.043267	0.035661	0.046188	0.032526	0.038382	0.006872
Novel_001686	XLOC_01858.	transcript	0	0	0	0.017231	0	0	0	0	0
Novel_001687	XLOC_01858.	transcript	0	0	0	0.002862	0	0	0	0	0
Novel_001688	XLOC_01861.	transcript	0.526358	0.645972	0.607561	0.615925	0.605269	0.623705	0.483763	0.685031	0.484222
Novel_001689	XLOC_01862.	transcript	0.097869	0	0.030581	0	0	0	0.048797	0.018554	0.719643
Novel_001690	XLOC_01863.	transcript	0.011142	0.01796	0.036035	0.031246	0.022361	0.029917	0.03277	0.015746	0.001233
Novel_001691	XLOC_01863.	transcript	0.394001	0.079457	0.221247	0.301341	0.176972	0.097099	0.21914	0.206621	0.061018
Novel_001692	XLOC_01880.	transcript	0	0	0	0	0	0	0	64.57109	0
Novel_001693	XLOC_01880.	transcript	0.050814	0.02261	0.118251	0.035989	0.085207	0.102273	0.104086	0.043794	0.17107
Novel_001694	XLOC_01881.	transcript	0.356777	0.20126	0.549899	0.403874	0	0.481585	0.393312	0.21579	0.47967
Novel_001695	XLOC_01881.	transcript	0	0	0	0	0	0	0.335986	0	0
Novel_001696	XLOC_01881.	transcript	0.006686	0.203632	0.004174	0.120748	0.460172	0.072386	0	0.369099	0.011209
Novel_001697	XLOC_01881.	transcript	0	0.36883	0.092306	0	0.029278	0	0.175682	0.081277	0.479512
Novel_001698	XLOC_01881.	transcript	0.388271	0	0	0	0	0	0	0	0.025699

Novel_001699	XLOC_01881.	transcript	0	0	0.264433	1.301622	0.590391	0.863721	0.907054	0.736464	0
Novel_001700	XLOC_01881.	transcript	0.032767	0	0.404447	0.164684	0.163065	0.786743	0.493764	0.51877	0.153327
Novel_001701	XLOC_01884.	transcript	0.043639	0	0.140363	0.152367	0.139164	0.227909	0.32448	0	0
Novel_001702	XLOC_01884.	transcript	0.14134	0.042998	0.140164	0.079311	0.222641	0.045849	0.066589	0.307455	0.4214
Novel_001703	XLOC_01885.	transcript	0.083539	0.072966	0.183256	0.15987	0.224112	0.12664	0.317732	0.223413	0.134711
Novel_001704	XLOC_01886.	transcript	0.078028	0.088967	0.031194	0.070853	0.089725	0.064473	0.116164	0.059606	0.040083
Novel_001705	XLOC_01887.	transcript	0	0	0	0	0.085225	0	0.010764	0	0.037041
Novel_001706	XLOC_01887.	transcript	0.458902	0	0.435283	0.394701	0.140645	0.462622	0.128839	0.318948	0.097012
Novel_001707	XLOC_01887.	transcript	0.555276	0	0	0	0	0.001704	0.014123	0	0.001539
Novel_001708	XLOC_01887.	transcript	0	0	0.937115	1.209457	1.154086	0.885989	1.63111	1.101558	0.81141
Novel_001709	XLOC_01887.	transcript	0.004328	0	0	0	0.003641	0	0.00622	0.035215	0.173946
Novel_001710	XLOC_01887.	transcript	0.096826	0.317347	0.003732	0	0.002079	0	0.014822	0.014402	0
Novel_001711	XLOC_01887.	transcript	0.444967	0	0	0	0	0.009792	0.602388	0	0
Novel_001712	XLOC_01887.	transcript	0	0	0.007109	0.010525	0.012567	0	0	0	0.015421
Novel_001713	XLOC_01894.	transcript	0	0.101025	0.198016	0.735712	0.46097	0.459567	3.940142	1.443776	0.005028
Novel_001714	XLOC_01894.	transcript	0.720743	0.192642	1.452247	2.054703	1.903955	1.984174	4.269307	8.763323	2.480035
Novel_001715	XLOC_01894.	transcript	0	0	0	1.579744	0	0.03015	0	0	0.028995
Novel_001716	XLOC_01903.	transcript	0	0	0	0.008342	0.010493	0	0.260645	0	0
Novel_001717	XLOC_01903.	transcript	0	0	0	0	0	0	0	0	0
Novel_001718	XLOC_01903.	transcript	0.972852	0	0.846192	1.403378	1.043691	0.206887	1.264929	1.224039	4.551218
Novel_001719	XLOC_01903.	transcript	0	0	0	0	0	0	0	0	0
Novel_001720	XLOC_01903.	transcript	0.236929	0	0.166833	0.217579	0.169448	0	0.17513	0.167639	0.690622
Novel_001721	XLOC_01903.	transcript	0.237059	1.063676	0.157748	0.177195	0.168403	0	0.179664	0.166929	0.456132
Novel_001722	XLOC_01903.	transcript	0.207749	0	0.141953	0.166287	0.156977	0	0.171425	0.188691	0.462103
Novel_001723	XLOC_01903.	transcript	0.300934	0.003361	0.191889	0.161639	0.133062	0.164104	0.149522	0.181716	0.502084
Novel_001724	XLOC_01903.	transcript	0.247644	0	0.160501	0.173512	0.124204	0	0.141352	0.197789	0.471106
Novel_001725	XLOC_01903.	transcript	0.283844	1.1816	0.185376	0.16183	0.18812	0.17384	0.214462	0.159039	0.489845
Novel_001726	XLOC_01903.	transcript	0.238262	0.040404	0.165839	0.174212	0.15843	0.758299	0.205711	0.198908	0.508404
Novel_001727	XLOC_01903.	transcript	0	0	0	0	0	0	0	0	0.552652
Novel_001728	XLOC_01909.	transcript	0.040544	0	0.208919	0.22584	0.232347	0.19887	0.29981	0.110915	0.230391
Novel_001729	XLOC_01909.	transcript	0.326962	0.481121	0.011428	0.009238	0.050776	0.13781	0.061067	0.303699	0.011634
Novel_001730	XLOC_01911.	transcript	0.100351	0.089006	0.185406	0	0.236944	0	0.060412	0.079794	0.119382
Novel_001731	XLOC_01911.	transcript	0	0	0.015067	0	0	0	0.002796	0.001957	0.018153
Novel_001732	XLOC_01911.	transcript	0.131507	0.443088	0.026225	0.11255	0.050191	0.261019	0.098024	0.124133	0.05337
Novel_001733	XLOC_01911.	transcript	0	0.329292	0	0.030118	0	0.027324	0.022502	0	0.039289
Novel_001734	XLOC_01912.	transcript	0.213514	0.020504	0.272887	0.262651	0.130566	0.227022	0.264938	0.212298	0.141126
Novel_001735	XLOC_01912.	transcript	0	0	0	0	0	0	0	0	0
Novel_001736	XLOC_01912.	transcript	0.430802	0.149032	0.299684	0.231097	0.233905	0.259648	0.282686	0.335851	0.257742

Novel_001737	XLOC_01912.	transcript	0.206568	0	0.193969	0.161865	0	0.093569	0.442873	0.035488	0.141343
Novel_001738	XLOC_01912.	transcript	0.423754	0.15061	0.123147	0.109638	0.28683	0.165039	0.176005	0.157591	0.066933
Novel_001739	XLOC_01912.	transcript	0	0	0	0.01085	0.000285	0	0.023413	0.016746	0
Novel_001740	XLOC_01917.	transcript	4.129666	0.793057	4.990462	8.778077	5.712062	6.771617	3.899906	7.167454	5.023001
Novel_001741	XLOC_01917.	transcript	0.009888	2.036701	0.049932	0.016565	0.028563	0.021858	0.067332	0.029237	0.021701
Novel_001742	XLOC_01917.	transcript	0.152964	0.085971	0.078355	0.202326	0.098312	0.144328	0.095762	0.153534	0.132383
Novel_001743	XLOC_01924.	transcript	0.187578	0.100896	0.291327	0.343167	0.444271	0.495211	0.200592	0.439928	0.462743
Novel_001744	XLOC_01924.	transcript	0.215978	0.098542	0.1593	0	0.078882	0	0.402377	0.098908	0
Novel_001745	XLOC_01927.	transcript	0.199176	0	0	0.006983	0	0	0	0	0
Novel_001746	XLOC_01927.	transcript	0.363386	0.57427	0.427066	0.528634	0.416413	0.541451	0.476684	0.655457	0.337795
Novel_001747	XLOC_01927.	transcript	0	0.004962	0.01742	0.231504	0.519062	0.007385	0.015465	0.497205	0.324118
Novel_001748	XLOC_01927.	transcript	0.007943	0.00467	0.023687	0	0	0.008579	0.016452	0	0
Novel_001749	XLOC_01927.	transcript	0.887911	0.632191	0.621796	0.263973	0.142595	0.643508	0.94122	0.204271	0.114888
Novel_001750	XLOC_01936.	transcript	0.497964	0	0	0	0	0	0	0.466417	0
Novel_001751	XLOC_01936.	transcript	0.03957	0	0	0	0	0.004433	0.005573	0	0
Novel_001752	XLOC_01936.	transcript	0.109569	0.226748	0.362131	0.402815	0.449859	0.636961	0.538143	0.794144	0.294741
Novel_001753	XLOC_01940.	transcript	0.67156	0.736441	0.161923	0.724077	0.724796	0.310426	0.834006	0.173906	0.058208
Novel_001754	XLOC_01940.	transcript	0.725297	0.890406	2.075629	1.663587	0.939575	1.363337	0.853229	2.221596	1.497675
Novel_001755	XLOC_01942.	transcript	0.565065	0.022429	0	0.382823	0	0	0	0	0.29829
Novel_001756	XLOC_01942.	transcript	0.00785	0.258099	0.337365	0.035387	0.392689	0.169021	0.544664	0.494708	0.027807
Novel_001757	XLOC_01942.	transcript	0	0	0.014196	0.094926	0.089615	0	0	0	0
Novel_001758	XLOC_01942.	transcript	0.02811	0.077137	0.01691	0	0.000736	0.120748	0.044728	0.079856	0.02254
Novel_001759	XLOC_01942.	transcript	0	0	0.018295	0	0	0.038305	0	0	0
Novel_001760	XLOC_01942.	transcript	0	0	0.089786	0	0.075706	0	0.017533	0	0
Novel_001761	XLOC_01944.	transcript	0.381004	0.266218	0.480758	0.367251	0.20333	0.549566	0.350532	0.286436	0.381855
Novel_001762	XLOC_01945.	transcript	0.134038	0.354936	0.18061	0.133713	0.232436	0.224377	0.202096	0.204208	0.148142
Novel_001763	XLOC_01945.	transcript	0.066094	0.014512	0.064057	0.058371	0.06119	0	0.043191	0.256407	0.148474
Novel_001764	XLOC_01945.	transcript	0.695947	0.498574	0.998201	0.276142	0.618951	0.538673	0.594143	0.398092	0.689428
Novel_001765	XLOC_01945.	transcript	0.553308	0.534254	0.397922	0.27458	0.304126	0.506525	0.411391	0.344052	0.359673
Novel_001766	XLOC_01945.	transcript	0.311471	0.657745	0.505461	0.351188	0.337157	0.574027	0.448465	0.175234	0.342654
Novel_001767	XLOC_01945.	transcript	1.699202	1.096476	0	0.541995	0.355008	0.680329	0.828866	0.440605	0
Novel_001768	XLOC_01945.	transcript	2.61555	1.461031	1.309189	1.092538	1.206806	1.888543	1.539141	1.908609	1.457101
Novel_001769	XLOC_01950.	transcript	0.07629	0.292833	0.11925	0.062527	0.078426	0.146963	0.176365	0.053029	0.178811
Novel_001770	XLOC_01950.	transcript	0.023084	0.101753	0.012235	0	0.02487	0.110031	0.016611	0.043577	0.057798
Novel_001771	XLOC_01950.	transcript	0	0.051875	0	0	0	0	0	0	0
Novel_001772	XLOC_01950.	transcript	0.09886	0.089642	0.225136	0	0	0.285735	0.0936	0.164771	0
Novel_001773	XLOC_01950.	transcript	0	0.049915	0	0.229306	0.211384	0	0.093475	0.118579	0.467026
Novel_001774	XLOC_01950.	transcript	0.079494	0	0	0	0	0	0	0	0

Novel_001775	XLOC_01953.	transcript	0.635863	0.113142	0.384727	0.053746	0.183927	0.183646	0.39371	0.022496	0.381877
Novel_001776	XLOC_01953.	transcript	0	0	0.240792	0.244519	0.145348	0.1972	0.134334	0.451337	0
Novel_001777	XLOC_01953.	transcript	0	0	0	0.213429	0.239023	0	0.189471	0.062607	0
Novel_001778	XLOC_01954.	transcript	5.840969	5.078762	3.481795	3.527602	3.402015	3.499538	4.6244	4.416214	2.788673
Novel_001779	XLOC_01954.	transcript	0.022432	3.561816	0.012447	0.016233	0	0.015547	0.367718	0.016226	0.053136
Novel_001780	XLOC_01954.	transcript	0.438466	0.699121	0.213786	0.261368	0.496443	0.610019	0.499609	0.48927	0.474501
Novel_001781	XLOC_01954.	transcript	0	0.150274	0	0	0.022598	0.127858	0	0.031495	0
Novel_001782	XLOC_01954.	transcript	0	0.017905	0.018245	0.122318	0.017651	0.074405	0.192704	0.106017	0.040906
Novel_001783	XLOC_01956.	transcript	0.076558	0.138524	0.078253	0.085741	0.065415	0.098288	0.075206	0.091683	0.069668
Novel_001784	XLOC_01956.	transcript	0.510523	4.958061	0.58918	0.215984	0.394334	0.575406	0.603783	0.387074	0.550008
Novel_001785	XLOC_01959.	transcript	0	0	0	2.119884	0.031803	0	0	0	0
Novel_001786	XLOC_01959.	transcript	6.247875	0	0	1.032614	0	0	0	3.412784	0
Novel_001787	XLOC_01959.	transcript	0.113722	0.678189	2.318081	0.035584	2.454772	2.30525	3.252012	0.045918	1.13007
Novel_001788	XLOC_01959.	transcript	0	0	0.236283	0.293055	0	0.074259	0	0.118239	0
Novel_001789	XLOC_01959.	transcript	0	0	0	0	0	0	0	0	0
Novel_001790	XLOC_01967.	transcript	0.023001	0.092996	0.029356	0.329582	0.408643	0.031495	0.394681	0.18919	0.570592
Novel_001791	XLOC_01967.	transcript	1.093125	2.045962	0.748289	0.475806	0.562019	0.370387	0.365941	1.078572	0.875853
Novel_001792	XLOC_01967.	transcript	0	0	0	0.051372	0.084008	0.692998	0.089804	0	0
Novel_001793	XLOC_01973.	transcript	0.178003	0.328097	0.016793	0.078808	0.079767	0.153313	0.055099	0.071463	0.094214
Novel_001794	XLOC_01974.	transcript	0.646241	0.931338	1.213326	1.069734	1.104204	0.888154	0.78119	1.067189	0.661771
Novel_001795	XLOC_01974.	transcript	0.410455	0.441435	0.535924	0.729376	0.533751	0.493307	0.624579	0.702317	0.390344
Novel_001796	XLOC_01974.	transcript	0.013387	0	0.027628	0.1012	0.02952	0	0.100812	0.047567	0.024264
Novel_001797	XLOC_01975.	transcript	0	0	0	0	0	0	0	0.286376	0
Novel_001798	XLOC_01975.	transcript	0	0	0	0	0	0	0	0	0.409723
Novel_001799	XLOC_01975.	transcript	0	0	0	0	0	0	0	0	0
Novel_001800	XLOC_01975.	transcript	0.319513	0.016064	0.071309	0.052794	0.079328	0.029192	0.490196	0.031542	0.239901
Novel_001801	XLOC_01990.	transcript	0	0	0	0	0	0	0	0	0
Novel_001802	XLOC_01992.	transcript	0.140702	0.043591	0.409844	0.34839	0.358138	0.352667	0.360441	0.296571	0.394768
Novel_001803	XLOC_02003.	transcript	0	0	0	0	0	0	0	0	0
Novel_001804	XLOC_02003.	transcript	0	0	0	0	0.250017	0	0	0.148934	0.222819
Novel_001805	XLOC_02003.	transcript	0	0	0	0	0	0	0	0	0
Novel_001806	XLOC_02003.	transcript	0.375561	0.062193	0.420495	0.393587	0.136139	0.437305	0.338466	0.288333	0.10338
Novel_001807	XLOC_02003.	transcript	0	0	0	0	0	0	0	0	0
Novel_001808	XLOC_02003.	transcript	0	0	0	0	0	0	0	0	0
Novel_001809	XLOC_02003.	transcript	0	0	0	0	0	0	0	0	0.013147
Novel_001810	XLOC_02003.	transcript	0	0	0	0	0	0	0	0	0
Novel_001811	XLOC_02012.	transcript	0.046536	0.004505	0.026573	0.023117	0.016737	0.036669	0.029583	0.055403	0
Novel_001812	XLOC_02012.	transcript	0	0	0	0	0.003095	0	0	0	0

Novel_001813	XLOC_02012.	transcript	0	0	0.016757	0.04352	0.014458	0	0.006434	0.038361	0
Novel_001814	XLOC_02012.	transcript	0.046228	0	0.173876	0.070452	0.137007	0.096447	0.35649	0.089201	0
Novel_001815	XLOC_02012.	transcript	0	0	0	0.039609	0	0	0.011073	0	0
Novel_001816	XLOC_02012.	transcript	0.449683	0	0.025652	0	0.209257	0.112688	0	0.199021	0.043525
Novel_001817	XLOC_02012.	transcript	0.125279	0.517578	0.141197	0.304428	0.25658	0.238256	0.270289	0.123451	0.390071
Novel_001818	XLOC_02013.	transcript	1.283573	0.950944	0.765253	0.849022	0.921895	0.826308	0.92458	1.081703	0.762373
Novel_001819	XLOC_02013.	transcript	0.098494	0.206874	0.081127	0.148027	0.090785	0.251221	0.060748	0.12885	0.404696
Novel_001820	XLOC_02013.	transcript	0	0.032184	0	0.007927	0.067614	0.058901	0.270831	0.106651	0
Novel_001821	XLOC_02014.	transcript	0.164825	0.294066	0.212679	0.012212	0.155111	0.216992	0.34562	0.491874	0.262125
Novel_001822	XLOC_02014.	transcript	0	0	0	0.537145	0	0	0	0	0
Novel_001823	XLOC_02015.	transcript	0.140453	0	0.120282	0	0.064754	0	0.183393	0.109195	0.02532
Novel_001824	XLOC_02023.	transcript	0.448839	0.069172	0	0.208789	0.247198	0.344372	0.469496	0.237172	0.359475
Novel_001825	XLOC_02031.	transcript	0.332924	0.151287	0	0	0	0.300843	0.446478	0.084996	0
Novel_001826	XLOC_02031.	transcript	0.16965	0.225283	0	0.023405	0	0.150224	0.144552	0.09417	0
Novel_001827	XLOC_02031.	transcript	0.082471	1.70E-05	0.182444	0.220941	0.345661	0.002195	0.00522	0.206845	0.11312
Novel_001828	XLOC_02031.	transcript	0.093606	0.002126	0.182865	0.053785	0.105722	2.30E-05	0.009549	0.101075	0.078815
Novel_001829	XLOC_02036.	transcript	0.121324	0.706455	0.019619	0.028877	0.042268	0.034348	0.066014	0.0163	0.23603
Novel_001830	XLOC_02057.	transcript	1.871961	0.428203	1.068529	0.692921	1.614883	1.504901	1.746486	1.550158	1.073822
Novel_001831	XLOC_02057.	transcript	0.744344	0.139557	0.33863	0.153052	0.325928	0.440747	0.671802	0.503476	0.279514
Novel_001832	XLOC_02062.	transcript	0	0.009372	0.019667	0.002962	0	0	0.02146	0	0.108986
Novel_001833	XLOC_02062.	transcript	0.175011	0.107599	0.050781	0.253448	0.196564	0.153635	0.311255	0.243925	0.059817
Novel_001834	XLOC_02062.	transcript	0	0	0	0	0	0	0	0	0
Novel_001835	XLOC_02062.	transcript	0.151468	0.236797	0.128909	0.068129	0.11418	0.024519	0.226462	0.098638	0.187353
Novel_001836	XLOC_02062.	transcript	0	0.594036	0.320126	0.677097	1.282934	1.241436	1.471048	0.811821	0
Novel_001837	XLOC_02062.	transcript	1.526207	1.260804	0.449545	0.826455	0.220195	0.423155	1.05467	1.643795	0.742791
Novel_001838	XLOC_02066.	transcript	13.80217	14.21696	10.59709	0	0.622602	23.34745	18.18374	20.94461	15.81429
Novel_001839	XLOC_02066.	transcript	0	0	0	9.750235	0.591527	0.104612	0	0.02803	0.084145
Novel_001840	XLOC_02066.	transcript	0	0	0	0	0	0	0	0	0
Novel_001841	XLOC_02073.	transcript	0	0	0	0	0	0	0.087649	0.247323	0
Novel_001842	XLOC_02074.	transcript	0.045879	0.028518	0.145511	1.955674	0.562309	0.359803	0.034312	0.407983	0.067693
Novel_001843	XLOC_02074.	transcript	0.026354	0.138184	0.139031	0.148489	0.162558	0.088186	0.088993	0.148222	0.133579
Novel_001844	XLOC_02074.	transcript	0	0.011843	0	0	0	0	0.080032	0	0.049026
Novel_001845	XLOC_02074.	transcript	0.016032	0.026476	0.045115	0.01089	0	0.044529	0	0.046434	0.005598
Novel_001846	XLOC_02074.	transcript	0.044498	0.099376	0.086402	0.019355	0.062781	0.116864	0	0.130741	0.009283
Novel_001847	XLOC_02074.	transcript	0.013268	0.012862	0.022399	0.003677	0	0.014772	0	0.011028	0.021814
Novel_001848	XLOC_02074.	transcript	0.210464	0	0.068895	0.199389	0.10457	0.066607	0.157233	0.143241	0.033569
Novel_001849	XLOC_02074.	transcript	0.166713	0.31872	0	0	0	0	0.103973	0.007055	0
Novel_001850	XLOC_02084.	transcript	0.144696	0	0.077875	0.055409	0.258847	0.06442	0.08514	0.061344	0.063489

Novel_001851	XLOC_02089.	transcript	0.325638	0.276406	0.504279	0.344148	0.451718	0.320346	0.567131	0.485966	0.534513
Novel_001852	XLOC_02098.	transcript	0	0.016108	0.348263	0.718005	0.080699	0.112652	0	0.057488	0.03671
Novel_001853	XLOC_02098.	transcript	0.694815	0.205872	0.180777	0.17113	0.486995	0.448109	0.883567	0.796764	0.389095
Novel_001854	XLOC_02098.	transcript	0	0	0	0	0	0	0	0	0
Novel_001855	XLOC_02098.	transcript	0.045616	0	0.179266	0.0794	0.094408	0.150913	0.210616	0.043762	0.007892
Novel_001856	XLOC_02105.	transcript	0	0	0.018286	0.034831	0.033202	0.007401	0	0	0
Novel_001857	XLOC_02105.	transcript	0.281667	0.180488	0.531104	0.498372	0.457066	0.530298	0.574943	0.249337	0.336739
Novel_001858	XLOC_02105.	transcript	0.195587	0	0	0	0	0	0.110393	0.305979	0
Novel_001859	XLOC_02106.	transcript	0.514483	0.126384	0.209531	0.153358	0.264406	0.27045	0.58101	0.330639	0.158636
Novel_001860	XLOC_02112.	transcript	0.494466	0.914648	0.734275	1.065701	0.666797	1.00878	0.701971	0.864393	0.970723
Novel_001861	XLOC_02130.	transcript	0.488583	0	0.220001	0.098943	0.540052	0	0.347119	0.494705	0.024026
Novel_001862	XLOC_02138.	transcript	0.130603	0.557249	0.157401	0.114032	0.196372	0.241327	0.068644	0.19311	0.315689
Novel_001863	XLOC_02140.	transcript	0.311732	0.062687	0.027948	0.009309	0.019057	0.001682	0.100505	0.10194	0.269597
Novel_001864	XLOC_02140.	transcript	0.475687	0.408938	0.450623	0.195288	0.614592	0.357066	0.507362	0.504609	0.403787
Novel_001865	XLOC_02140.	transcript	0	0	0	0.002147	0	0	0	0	0.454023
Novel_001866	XLOC_02140.	transcript	0	0	0	0	0.535715	0	0	0.36705	0.016175
Novel_001867	XLOC_02140.	transcript	0.423857	0.437415	0.251303	0.290189	0.14167	0.279727	0.646153	0.107918	0
Novel_001868	XLOC_02141.	transcript	0	0	0	0	0	0	0	0	0
Novel_001869	XLOC_02141.	transcript	0.404401	0.344219	0.7827	0.345057	0.409017	0.235081	0.322313	0.024721	0.290903
Novel_001870	XLOC_02141.	transcript	0	0.052741	0	0	0	0	0.103332	0.349889	0
Novel_001871	XLOC_02141.	transcript	0.015572	0	0	0	0	0	0	0	0
Novel_001872	XLOC_02141.	transcript	0	0	0.011039	0.010812	0.030611	0.024059	0	0	0.032081
Novel_001873	XLOC_02142.	transcript	0.205415	0.356269	0.421342	0.771249	0.799063	0.536448	0.497551	0.392111	0.435904
Novel_001874	XLOC_02146.	transcript	0	0	0	0	0	0	0	0	0
Novel_001875	XLOC_02146.	transcript	1.337946	0.556075	1.350119	1.064896	1.465496	1.687962	1.713577	1.438284	1.43318
Novel_001876	XLOC_02148.	transcript	0.551044	0.604978	0.369234	0.321264	0.463247	0.434244	0.396298	0.319927	0.08613
Novel_001877	XLOC_02149.	transcript	0.085691	0.245761	0.400676	0.332987	0.146072	0.233646	0.118369	0.14787	0.229501
Novel_001878	XLOC_02152.	transcript	0.01605	0.780759	0	0.005649	0	0	0.009775	0.063488	0.034684
Novel_001879	XLOC_02152.	transcript	0.975557	0.315933	2.68513	1.738634	1.24673	3.67014	1.235729	4.252	0.757175
Novel_001880	XLOC_02152.	transcript	1.571492	0	3.422838	3.580784	3.314234	1.889848	2.492175	1.199833	1.306911
Novel_001881	XLOC_02153.	transcript	0.010184	0.006724	0.007375	0.045275	0.058841	0	0	0.110424	0.026267
Novel_001882	XLOC_02153.	transcript	0.019632	0.436447	0.18791	0	0.051733	0.020762	0.194134	0.143427	0.179469
Novel_001883	XLOC_02155.	transcript	0.138076	0	0	0.331551	0.093674	0.182204	0.060276	0.45752	0.224109
Novel_001884	XLOC_02155.	transcript	0.127796	0	0.104022	0.193266	0.333455	0.138425	0.088579	0.151656	0.05848
Novel_001885	XLOC_02155.	transcript	0	0.042791	0.209242	0.25035	0.258679	0.141407	0.252315	0.471667	0.120829
Novel_001886	XLOC_02155.	transcript	0	0.108065	0.251147	0.230428	0.003221	0.237279	0.437185	0.288783	0.419013
Novel_001887	XLOC_02155.	transcript	0.763893	0.103294	0.411082	0.292418	0.633013	0.378571	0.445334	0.527382	0.301726
Novel_001888	XLOC_02155.	transcript	0.120883	2.291166	0.093447	0.110969	0.239449	0.151387	0.252811	0.262776	0.051896

Novel_001889	XLOC_02170.	transcript	1.062078	0	1.516771	1.023304	0	0	0	0.864532	0
Novel_001890	XLOC_02171.	transcript	0.111334	0	0.131687	0	0.183569	0.019042	0.259843	0.090896	0.150534
Novel_001891	XLOC_02171.	transcript	0.042681	0	0	0	0.034659	0.010895	0.024467	0	0
Novel_001892	XLOC_02171.	transcript	0.015666	0	0	0	0	0	0	0	0
Novel_001893	XLOC_02171.	transcript	0	0	0	0	0	0	0	0	0
Novel_001894	XLOC_02173.	transcript	0	0	0	0	0	0	0	0.224499	0
Novel_001895	XLOC_02173.	transcript	0	0	0	0	0	0	0.101702	0.00233	0
Novel_001896	XLOC_02173.	transcript	0	0	0	0	0	0	0	0.220516	0
Novel_001897	XLOC_02173.	transcript	0.424727	0.044396	0.51461	0.183591	0.363794	0.303376	0.156025	0.080409	0.159486
Novel_001898	XLOC_02180.	transcript	0.769425	0.0882	0.087563	0.472665	0.117409	0	0.307719	0	0
Novel_001899	XLOC_02180.	transcript	0.009752	0.068056	0	0.008324	0	0.752365	0.028962	0.28171	0.527307
Novel_001900	XLOC_02180.	transcript	0	0.05855	0.418863	0.1651	0.520508	0	0	0	0
Novel_001901	XLOC_02180.	transcript	0.551717	0.360975	0.42537	0.337594	0.401899	0.484915	0.518771	0.411611	0.268089
Novel_001902	XLOC_02180.	transcript	0	0	0	0	0	0	0	0	0
Novel_001903	XLOC_02180.	transcript	0	0.333075	0	0	0	0	0	0	0
Novel_001904	XLOC_02180.	transcript	0	0.14201	0	0.354662	0	0	0	0.36233	0
Novel_001905	XLOC_02180.	transcript	0.041313	0	0	0	0	0	0	0.176082	0
Novel_001906	XLOC_02180.	transcript	0.343993	0.049577	0	0.004082	0.3521	0	0	0	0.220658
Novel_001907	XLOC_02180.	transcript	0.17967	0.556819	0	0.165075	0.336765	0.314293	0.288737	0.098261	0.071563
Novel_001908	XLOC_02180.	transcript	0.329751	0	0	0.000781	0	0	0	8.50E-05	0
Novel_001909	XLOC_02180.	transcript	0	0	0	0	0	0.091876	0	0	0.213204
Novel_001910	XLOC_02180.	transcript	0	0.221143	0	0	0	0	0	0	0
Novel_001911	XLOC_02180.	transcript	0.027312	0.080283	0.410567	0.003423	0.052218	0.116916	0.093667	0.290367	0.07265
Novel_001912	XLOC_02180.	transcript	0	0.086172	0	0.21083	0	0.016421	0.211691	0	0
Novel_001913	XLOC_02180.	transcript	0	0	0	0	0.026995	0	0	0	0
Novel_001914	XLOC_02180.	transcript	0	0.118566	0	0	0	0	0	0.048596	0.005434
Novel_001915	XLOC_02184.	transcript	0	1.209428	0	0.017509	0.003943	0	0	0	0
Novel_001916	XLOC_02184.	transcript	0	0	0.095629	0.966182	0.616698	0.48124	0	0.948588	0
Novel_001917	XLOC_02184.	transcript	0.726856	0.593701	0.275263	0	0	0.409037	0.39793	0	0.693926
Novel_001918	XLOC_02184.	transcript	0	0	0	0	0	0	0	0	0.025896
Novel_001919	XLOC_02184.	transcript	0.517596	0	0	0	0	0	0.436839	0	0.220386
Novel_001920	XLOC_02184.	transcript	0	0	0	0	0	0	0	0	0
Novel_001921	XLOC_02184.	transcript	0	0.030144	0	0	0.12507	0	0.20867	0.001217	0
Novel_001922	XLOC_02184.	transcript	0.112741	0.153345	0.154357	0.144619	0.169397	0.272026	0	0.447822	0.1953
Novel_001923	XLOC_02189.	transcript	0.037427	0	0.236178	0	0	0	0.850767	0.14005	0
Novel_001924	XLOC_02189.	transcript	0.003529	0	0	0	0	0	0	0.172321	0
Novel_001925	XLOC_02189.	transcript	0.011535	0.016043	0.369559	0.626397	0.400621	0.590574	0.563237	0.228966	0.302679
Novel_001926	XLOC_02189.	transcript	0.206287	0.506845	0.121064	0.166152	0.281397	0	0.191965	0.176127	0.199225

Novel_001927	XLOC_02196.	transcript	0.016505	0	0.073014	0	0	0	0.261566	0	0.306471
Novel_001928	XLOC_02196.	transcript	0	0.282491	0.074406	0.022309	0.221543	0	0.172637	0.054175	0
Novel_001929	XLOC_02196.	transcript	0.552481	0.067814	0.287556	0.411991	0.123905	0.328826	0.244804	0.416839	0.127067
Novel_001930	XLOC_02198.	transcript	0.164374	0.450159	0.472137	0.165439	0.426406	0.236956	0.287441	0.349359	0.369721
Novel_001931	XLOC_02198.	transcript	0.610596	0.639407	0.676662	0.965072	0.694173	0.437917	0.5557	0.789912	0.707477
Novel_001932	XLOC_02198.	transcript	0.142585	0.119562	0.401059	0.6024	0.370966	0.355004	0.301205	0.350916	0.201945
Novel_001933	XLOC_02200.	transcript	0.203935	0	0.053917	0.091346	0.074003	0.3734	0.090236	0.071672	0.053556
Novel_001934	XLOC_02201.	transcript	0.107179	0.034455	0.1343	0.099955	0.145018	0.19216	0.286796	0.093278	0.134305
Novel_001935	XLOC_02202.	transcript	0.383767	0.71427	0.434369	0.24741	0.382079	0.311193	0.414112	0.418587	0.539702
Novel_001936	XLOC_02202.	transcript	0	0	0	0	0	0	0	0	0
Novel_001937	XLOC_02205.	transcript	0	0	0	0	0	0	0	0.170734	0
Novel_001938	XLOC_02205.	transcript	0.076789	0.003902	0.089221	0.54445	0.320197	0.101897	0.108584	0	0.027617
Novel_001939	XLOC_02210.	transcript	0.060449	0	0	0.155324	0.166435	0.148742	4.20E-05	0.02909	0
Novel_001940	XLOC_02210.	transcript	0.251846	0	0	0	0.08149	0.098253	0.068409	0.039326	0
Novel_001941	XLOC_02210.	transcript	0.175447	0.106265	0.158989	0.03811	0	0.125688	0.199687	0.164005	0.105033
Novel_001942	XLOC_02210.	transcript	0.528078	0.710351	0.651412	0.578957	0.645829	0.611359	0.562559	0.66448	0.674168
Novel_001943	XLOC_02210.	transcript	0.334914	0.328751	0.331571	0.243443	0.357121	0.258114	0.490328	0.526463	0.284737
Novel_001944	XLOC_02212.	transcript	0	0	0.00375	0	0.133379	0	0	0.916789	0
Novel_001945	XLOC_02212.	transcript	0.00463	0.021552	0	0	0	0.003661	0	0	0.712801
Novel_001946	XLOC_02212.	transcript	0.031969	0	0	0	0	0	0	0	0.566496
Novel_001947	XLOC_02212.	transcript	0.169842	0.065955	0.138439	0.15192	0.315598	0.066748	0.270655	0	0
Novel_001948	XLOC_02212.	transcript	0.328049	0.140174	0.666158	0.563561	0.311124	0.525881	0.441027	0.603891	0.258514
Novel_001949	XLOC_02212.	transcript	0.643321	0.152466	0.600591	1.535987	0.871322	0.671085	0.857254	1.378129	0.457674
Novel_001950	XLOC_02215.	transcript	0.203747	0	0	0.038464	0.480351	0	0.026795	0.04671	0
Novel_001951	XLOC_02215.	transcript	0.019477	0.060404	0.322485	0.109329	0.039355	0.275672	0.276669	0.197577	0.203468
Novel_001952	XLOC_02217.	transcript	6.30E-05	5.10E-05	17.66089	1.30E-05	38.33116	0	44.43498	3.60E-05	8.60E-05
Novel_001953	XLOC_02217.	transcript	19.24495	11.16335	34.96925	55.27491	3.34744	20.18159	0.396346	37.23815	34.04068
Novel_001954	XLOC_02217.	transcript	12.74926	0	0	0	1.977932	14.43707	1.818676	0	27.29842
Novel_001955	XLOC_02217.	transcript	0	0	0	0	0	0	0	0	0
Novel_001956	XLOC_02217.	transcript	0	0	0	0	0	0	0	0.001805	0
Novel_001957	XLOC_02219.	transcript	0	0.081919	0	0.008598	0	0	0	0	0.200797
Novel_001958	XLOC_02219.	transcript	0.839861	0.131351	0.441887	0.447587	0.526587	0.693779	0.813672	0.500429	0.3729
Novel_001959	XLOC_02220.	transcript	0.627634	0.330122	0.384802	0.244296	0.239348	0.191057	0.594276	0.50043	0.248034
Novel_001960	XLOC_02220.	transcript	0.15107	0.176449	0.268921	0.23749	0.196943	0.321954	0.22704	0.212465	0.266492
Novel_001961	XLOC_02220.	transcript	0.128647	0	0.001774	0.018599	0.118135	0.021174	0.267576	0.125318	0.064665
Novel_001962	XLOC_02220.	transcript	0.251703	0.051076	0.218012	0.128552	0.240609	0.101257	0.526532	0.192644	0.328245
Novel_001963	XLOC_02220.	transcript	0.288808	0.053363	0.280955	0.167564	0.316555	0.324584	0.595324	0.125036	0.448123
Novel_001964	XLOC_02221.	transcript	0.688911	0.874117	0.298677	0.500909	0.442346	0.557617	0.442898	0.684258	0.727279

Novel_001965	XLOC_02221.	transcript	0.081552	0.242151	0.620799	0.413969	0.38525	0.486143	0.26588	0.630542	0.413702
Novel_001966	XLOC_02223.	transcript	0.069808	0.011014	0.100988	0.002631	0.048973	0.059068	0	0.057911	0.005866
Novel_001967	XLOC_02223.	transcript	0.083212	0.123373	0	0.027267	0	0.000282	0.005795	0	0.038594
Novel_001968	XLOC_02223.	transcript	0.068538	0.612748	0	0.101259	0.14785	0.122517	0.237849	0.133279	0.165078
Novel_001969	XLOC_02227.	transcript	0.319035	0.375136	0.174681	0.196773	0.346005	0	0.154937	0.396957	0.365786
Novel_001970	XLOC_02227.	transcript	0.727078	0.36641	0.489456	0.317779	0.614727	0.578837	0.999448	0.611558	0.275474
Novel_001971	XLOC_02230.	transcript	0.028813	0.334322	0.030256	0	0	0	0.1073	0	0.068294
Novel_001972	XLOC_02230.	transcript	0	0	0	0	0	0	0.309802	0.043479	0
Novel_001973	XLOC_02230.	transcript	0.130661	0.009657	0.167241	0.097415	0.234979	0.043814	0.189366	0.199879	0.069001
Novel_001974	XLOC_02233.	transcript	0.103433	0.001383	0	0	0	0.068167	0	0.03835	0
Novel_001975	XLOC_02233.	transcript	0	0.470022	0	0	0	0	0	0	0
Novel_001976	XLOC_02233.	transcript	0	0	0.000567	0	0.002294	0.039466	0	0	0
Novel_001977	XLOC_02233.	transcript	0.325312	0.449573	0.364538	0.301481	0.308027	0.255348	0.388923	0.343506	0.297392
Novel_001978	XLOC_02234.	transcript	0	0	0.151132	0.03967	0	0	0	0.192432	0
Novel_001979	XLOC_02236.	transcript	6.116133	4.701716	3.840156	5.501525	4.882778	10.00071	5.081538	5.108235	7.024374
Novel_001980	XLOC_02240.	transcript	0	0	0	0	0	0.183952	0	0	0.010589
Novel_001981	XLOC_02240.	transcript	0.300657	0.379299	0.258914	0.25442	0.219664	0.138583	0.184708	0.161576	0.209023
Novel_001982	XLOC_02240.	transcript	2.689686	1.311518	2.38964	2.15629	2.13597	2.403805	3.106759	2.686317	1.99802
Novel_001983	XLOC_02241.	transcript	0	0.097681	0.012682	0	0	0	0	0	0.003185
Novel_001984	XLOC_02241.	transcript	0.013285	0.016709	0	0.493192	0	0	0	0	0.00761
Novel_001985	XLOC_02241.	transcript	0.46266	0.350179	0.389895	0.018965	0.445944	0.474191	0.550951	0.445593	0.387739
Novel_001986	XLOC_02241.	transcript	0.201907	0.363827	0.388404	0.339029	0.359235	0.49908	0.401568	0.368621	0.385895
Novel_001987	XLOC_02241.	transcript	0.174754	0	0.03695	0.058585	0.026691	0.106204	0.138887	0	0.571416
Novel_001988	XLOC_02258.	transcript	0	0	0	0.231768	0.253064	0	0	0	0
Novel_001989	XLOC_02258.	transcript	0	0	0.815398	0.40137	0.478046	0	0	0	0.399895
Novel_001990	XLOC_02258.	transcript	0	0	0	0	0	0	0	0	0
Novel_001991	XLOC_02258.	transcript	0.541305	0.641753	0.699558	0.597868	0.550901	0.618828	0.769295	0.409188	0.183924
Novel_001992	XLOC_02258.	transcript	0	0	0	0	0	0	0	0	0
Novel_001993	XLOC_02259.	transcript	0.338402	0.811331	0.62825	1.160808	0.66933	0.605159	0.879163	0.618324	0.450558
Novel_001994	XLOC_02260.	transcript	0.246747	0.069389	0	0	0	0	0.127337	0	0
Novel_001995	XLOC_02260.	transcript	0.119621	0.163394	0.400215	0.187522	0.3298	0.26333	0.187464	0.196197	0.176946
Novel_001996	XLOC_02260.	transcript	0	0	0	0	0	0.019925	0.150155	0.092041	0
Novel_001997	XLOC_02260.	transcript	0.748995	2.528278	0.21075	3.464681	0.760961	1.735727	0.529151	0.39644	0.319366
Novel_001998	XLOC_02260.	transcript	0.778652	0	0.411165	2.47679	0.588002	0	0.364127	0	0
Novel_001999	XLOC_02261.	transcript	0.298253	0.261383	0.488591	0.417395	0.561256	0.510531	0.655251	0.350867	0.387845
Novel_002000	XLOC_02261.	transcript	0.050237	0	0.263913	0.27813	0.290035	0.174283	0.358819	0.173946	0.187515
Novel_002001	XLOC_02261.	transcript	0	0	0.011964	0.059028	0.057603	0	0	0.000112	0
Novel_002002	XLOC_02261.	transcript	0.000566	0.358011	0.107203	0	0.08426	0.000535	0.001005	0.000822	0.010791

Novel_002003	XLOC_02261.	transcript	0.002638	0	0.001193	0.020342	0.08229	0.021093	0.047118	0.005893	0.051388
Novel_002004	XLOC_02261.	transcript	0	0	0	8.00E-06	0	0.039983	0.063658	0.028369	0
Novel_002005	XLOC_02261.	transcript	0.061998	0	0	0	0	0	0	0	0.082582
Novel_002006	XLOC_02261.	transcript	0.29714	0.022879	0.053154	0.120611	0.239396	0.252512	0.583999	0.226634	0.031694
Novel_002007	XLOC_02262.	transcript	0.129689	0.014501	0	0.111574	0.183489	0.071826	0.482296	0.033665	0.097423
Novel_002008	XLOC_02262.	transcript	0	0	0.139164	0	0	0	0	0.093203	0.024028
Novel_002009	XLOC_02262.	transcript	0	0	0	0	0	0	0	0	0
Novel_002010	XLOC_02264.	transcript	0.159312	0.024438	0.074412	0.000487	0.083299	0.057065	0.072891	0.011769	0.751062
Novel_002011	XLOC_02267.	transcript	0	0.143568	0	0	0	0	0.115421	0.151556	0
Novel_002012	XLOC_02267.	transcript	0.294652	0.107883	0.208977	0.238165	0.422727	0.129017	0.089324	0.069724	0.057164
Novel_002013	XLOC_02267.	transcript	0	0	0	0	0	0	0.255615	0	0
Novel_002014	XLOC_02267.	transcript	0	0	0.132163	0.100052	0.131572	0	0	0.135426	0.105695
Novel_002015	XLOC_02267.	transcript	0	0.268149	0	0	0	0	0.077857	0	0
Novel_002016	XLOC_02267.	transcript	0.353734	0.057298	0	0	0	0.468264	0.058846	0.277083	0
Novel_002017	XLOC_02277.	transcript	0	0	0	0.253059	0	0.296987	0	0	0
Novel_002018	XLOC_02277.	transcript	0	0	0	0	0	0	0	0	0
Novel_002019	XLOC_0228C.	transcript	0.013998	0	0.235606	0.201089	0.13556	0.291138	0.028529	0.128385	0.223494
Novel_002020	XLOC_0228C.	transcript	0	0	0.314472	0.380269	0	0.302589	0.120394	0.138699	0.030946
Novel_002021	XLOC_0228C.	transcript	0	0.23855	0	0.003734	0.535328	0.002275	0.570437	0	0.203374
Novel_002022	XLOC_0228C.	transcript	0.283306	0	0	0	0	0	0	0.288404	0.002562
Novel_002023	XLOC_0228C.	transcript	0	0	0.228499	0	0	0.143272	0	0	0
Novel_002024	XLOC_0228C.	transcript	0.119667	0.283233	0.25156	0.062071	0.000569	0.117092	0.17602	0.088682	0.086579
Novel_002025	XLOC_0228C.	transcript	0.369431	0.231798	0	0.493044	0.168428	0.083725	0.112554	0.226936	0.646065
Novel_002026	XLOC_0228C.	transcript	0.256362	0.137699	0	0.337704	0.431869	0.14692	0.11614	0.220423	0.176363
Novel_002027	XLOC_0228C.	transcript	0.000716	0.09247	0	0.041096	0.194302	0.195277	0.000274	0.127388	0.249833
Novel_002028	XLOC_0228C.	transcript	0.192384	0.246214	0.195623	0.132773	0.283645	0.315834	0.46274	0.278554	0.224453
Novel_002029	XLOC_02282.	transcript	0.053044	0.352803	0.346456	0.115892	0.090581	0.194275	0.205964	0.06011	0.111208
Novel_002030	XLOC_02282.	transcript	0	0.001649	0.013822	0	0.056402	0	0	0	0
Novel_002031	XLOC_02282.	transcript	0	0.219326	0.018704	0	0	0	0.01847	0.023451	0
Novel_002032	XLOC_02282.	transcript	0	0	0.036512	0	0	0.08984	0.098475	0	0.395499
Novel_002033	XLOC_02282.	transcript	0	0	0	0	0	0	0.087508	0.297202	0.304261
Novel_002034	XLOC_02282.	transcript	0.654412	1.366699	0.24542	0.305664	0.506601	0.391312	0.749306	0.492233	0.779467
Novel_002035	XLOC_02282.	transcript	0	0	0.373819	0.246396	0.459804	0	0.424059	0.483016	0.39487
Novel_002036	XLOC_02282.	transcript	0	0	0	0.132719	0.166	0	0.218769	0.138055	0
Novel_002037	XLOC_02294.	transcript	0	0	0	0	0	0	0	0	0
Novel_002038	XLOC_02294.	transcript	0.222991	0.102494	0.203503	0.030954	0.096667	0.238201	0.464888	0.375012	0.284388
Novel_002039	XLOC_02294.	transcript	0.025502	0.023752	0.195729	0.32942	0.309148	0.03934	0.042003	0.022597	0.100641
Novel_002040	XLOC_02294.	transcript	0	0	0.00205	0	0.001908	0	0.028646	0.043895	0

Novel_002041	XLOC_02296.	transcript	0.131802	0.047534	0.292008	0.417274	0.322931	0.303895	0.315906	0.336615	0.128503
Novel_002042	XLOC_02301.	transcript	0.48335	1.221524	0.749592	0.691336	0.659371	0.475283	0.821505	0.663973	0.482207
Novel_002043	XLOC_02301.	transcript	0.201303	0.049624	0.125897	0.132851	0.025583	0.32129	0.312907	0.111805	0.144188
Novel_002044	XLOC_02301.	transcript	0.728459	0.546927	0.389459	0.53511	0.410009	0.298	0.496833	0.635433	0.306634
Novel_002045	XLOC_02301.	transcript	0.074406	0.245149	0.212169	0.001758	0.274079	0.475831	0.420757	0.078311	0.095851
Novel_002046	XLOC_02301.	transcript	0	0	0	0.013619	0	0	0.030834	0.00534	0
Novel_002047	XLOC_02301.	transcript	0.067445	2.263306	0.06746	0.11758	0.11015	0.178501	0.269286	0.190885	0.336849
Novel_002048	XLOC_02302.	transcript	0.114945	0.32136	0.176439	0.232451	0.199694	0.194747	0.180477	0.250256	0.174156
Novel_002049	XLOC_02302.	transcript	0.750125	1.025108	0.582166	0.616177	0.657274	0.800942	1.031484	0.895529	0.324374
Novel_002050	XLOC_02302.	transcript	0.209266	0.131873	0.420422	0.242658	0.215058	0.283273	0.131165	0.328705	0.041905
Novel_002051	XLOC_02306.	transcript	0.156522	0	0.211539	0.206846	0.302732	0.136417	0.373808	0.196158	0.168622
Novel_002052	XLOC_02307.	transcript	0.121661	0.345031	0.051656	0.079556	0.083154	0.155971	0.10604	0.057578	0.059641
Novel_002053	XLOC_02307.	transcript	0.133809	0.175067	0.046887	0.044028	0.037962	0.027754	0.08676	0.072738	0.015569
Novel_002054	XLOC_02307.	transcript	0.34731	0.232151	0.045629	0.342998	0.113694	0.361734	0.083598	0.290902	0.009886
Novel_002055	XLOC_02307.	transcript	1.482942	2.573349	2.604963	2.189411	2.55285	2.34153	2.7787	2.861451	1.854359
Novel_002056	XLOC_02308.	transcript	0.408249	0	0.022333	0	0	0	0.72545	0	0
Novel_002057	XLOC_02308.	transcript	0.08395	0.364274	0.518578	0.728842	0.519989	0.605578	0	0.786966	0.579911
Novel_002058	XLOC_02308.	transcript	1.147872	2.069189	1.62834	3.243503	2.449895	2.819403	2.237952	3.192426	2.199277
Novel_002059	XLOC_02309.	transcript	0.325457	0.07569	0.294481	0.213557	0.336725	0.236568	0.522457	0.260403	0.271866
Novel_002060	XLOC_02311.	transcript	0.166875	0.097553	0.208888	0.192035	0.150024	0.194329	0.200142	0.183959	0.179164
Novel_002061	XLOC_02312.	transcript	0.823754	0.620538	1.160445	1.209221	1.089172	1.226711	0.668625	1.435926	1.147954
Novel_002062	XLOC_02312.	transcript	0.480749	0.019029	1.165762	0.585003	0.284591	1.844609	0.48865	0.621732	1.506401
Novel_002063	XLOC_02312.	transcript	0.568614	0	1.471968	3.878239	2.950232	2.470796	1.726209	2.030426	0.364006
Novel_002064	XLOC_02312.	transcript	0.170096	0.6384	0.163179	0.354913	0.610366	0.746511	0.551201	0.319671	0.543209
Novel_002065	XLOC_02313.	transcript	0.480746	0.679618	0.537915	0.832025	0.585038	0.650925	1.012099	0.873382	0.767166
Novel_002066	XLOC_02313.	transcript	0.111551	0.495403	0	0	0	0	0	0	0
Novel_002067	XLOC_02314.	transcript	0.096066	0.694484	0	0.014061	0.038495	0.011156	0.01722	0.012212	0.05218
Novel_002068	XLOC_02315.	transcript	0.292305	0	0.141434	0.128452	0.20871	0.03489	0.573962	0.262351	0.197854
Novel_002069	XLOC_02320.	transcript	0.179693	0	0	0.070932	0.074937	0.022692	0.29065	0.137088	0.022289
Novel_002070	XLOC_02325.	transcript	0.494889	0.516523	0.522226	0.440467	0.540802	0.580739	0.807027	0.549771	0.470343
Novel_002071	XLOC_02326.	transcript	0.263296	0	0.281386	0.110589	0.326073	0.146069	0.289089	0.152319	0.491492
Novel_002072	XLOC_02327.	transcript	0.61245	1.14485	0.881256	1.300877	0.937721	1.355133	0.809185	1.056388	1.049424
Novel_002073	XLOC_02327.	transcript	0	0.043697	0.107126	0.173466	0.196607	0.07179	0.095535	0.235848	0.341667
Novel_002074	XLOC_02327.	transcript	0.136201	0.286055	0.317723	0.098218	0.210866	0.251261	0.544246	0.432253	0.215838
Novel_002075	XLOC_02327.	transcript	0.406993	0.533324	0.48888	0.220198	0.313885	0.316714	0.552617	0.409355	0.386264
Novel_002076	XLOC_02327.	transcript	0.44789	0.316372	0.131949	0.222188	0.165644	0.187426	0.379969	0.289206	0.09727
Novel_002077	XLOC_02327.	transcript	0	0.249627	0.174733	0.036838	0.263668	0	0.132726	0.017652	0
Novel_002078	XLOC_02327.	transcript	0.207097	0.506576	0.02344	0.117612	0.115939	0	0.16407	0.175289	0.091197

Novel_002079	XLOC_02327.	transcript	0	0.167396	0.09258	0	0.183973	0	0.095477	0.070088	0.271398
Novel_002080	XLOC_02327.	transcript	0.214418	0.035689	0.011444	0.092538	0.071351	0.032524	0.006875	0.026408	0.014817
Novel_002081	XLOC_02327.	transcript	0	0	0	0.036434	0.013788	0	0.118537	0.001955	0.160917
Novel_002082	XLOC_02327.	transcript	1.232539	1.000251	0.989534	1.13126	0.825101	1.137209	1.537254	1.431007	1.145593
Novel_002083	XLOC_02327.	transcript	0.224802	0.050847	0.459993	0.304227	0.405448	0.420712	0.470825	0.438864	0.302449
Novel_002084	XLOC_02332.	transcript	0.11381	1.494333	1.145338	1.445131	1.95435	1.488263	2.027745	3.593737	1.409235
Novel_002085	XLOC_02332.	transcript	0	0	0	0	0	1.463487	0	0	0
Novel_002086	XLOC_02332.	transcript	0.076113	0.101691	0.135279	0.178935	0.099234	0.149467	0.131417	0.228836	0.208316
Novel_002087	XLOC_02334.	transcript	0.495826	0.352116	0.34561	0.258055	0.17313	0.569331	0.275036	0.341721	0.437512
Novel_002088	XLOC_02334.	transcript	0.006273	0.341986	0.069745	0.32348	0.505669	0.052621	0.252587	0.342578	0.023448
Novel_002089	XLOC_02334.	transcript	0.340052	0.344535	0.557446	0.38474	0.413843	0.612884	0.386098	0.865343	0.172684
Novel_002090	XLOC_02336.	transcript	0	0.027584	0.202289	0.111685	0.152329	0.438617	0.057664	0.185481	0.091375
Novel_002091	XLOC_02338.	transcript	0.035843	0.094755	0	0.127761	0.115895	0.160626	0.043418	0.109915	0
Novel_002092	XLOC_02340.	transcript	0	0	0.170036	0.026816	0.030766	0.04491	0.122759	0.02383	0
Novel_002093	XLOC_02340.	transcript	0.029379	0	0	0	0	0	0.10175	0.008941	0
Novel_002094	XLOC_02340.	transcript	0.481219	0.101439	0.202423	0.351999	0.475156	0.079695	0.577231	0.070354	0.304889
Novel_002095	XLOC_02340.	transcript	0	0	0	0	0	0	0	0.327824	0.025742
Novel_002096	XLOC_02340.	transcript	0.041677	0.102567	0.169508	0.064507	0.138907	0.241277	0.187483	0.182651	0.133526
Novel_002097	XLOC_02343.	transcript	0	0.007935	0.028725	0	0	0	0.014513	0.030512	0
Novel_002098	XLOC_02343.	transcript	0.21674	0.316409	0.40681	0.403862	0.546386	0.462431	0.568539	0.245572	0.391425
Novel_002099	XLOC_02343.	transcript	0.64077	0	0.140965	0.078143	0	0.134037	0.181207	0.354492	0.240391
Novel_002100	XLOC_02344.	transcript	0.060375	0	0.27755	0.673692	0.206403	0.375041	0.297017	0.111775	0.064129
Novel_002101	XLOC_02345.	transcript	1.164005	0.800868	1.563478	1.865348	1.604593	1.189816	1.273778	1.464065	1.421705
Novel_002102	XLOC_02353.	transcript	0.592935	0.577473	0.839629	0.190678	0.69056	0.128805	0.451494	0.161456	0.224737
Novel_002103	XLOC_02353.	transcript	0	0	0	0	0	0	0	0	0
Novel_002104	XLOC_02358.	transcript	0.36277	1.016704	0.542827	0.384006	0.665249	0.534882	1.005522	0.780602	0.556581
Novel_002105	XLOC_02359.	transcript	0.321158	0.340051	0.190753	0.243929	0.150267	0.287847	0.295592	0.230203	0
Novel_002106	XLOC_02359.	transcript	0.013377	0.153373	0.042426	0.522198	0.353885	0.016139	0.103567	0.111118	0.063412
Novel_002107	XLOC_02359.	transcript	0.25516	0.512565	0.51802	0.15962	0.207321	0.395041	0.418222	0.343458	0.365346
Novel_002108	XLOC_02359.	transcript	0.02582	0	0	0	0	0	0	0	0
Novel_002109	XLOC_02359.	transcript	0.431783	0.087412	0.39832	0.176199	0.345046	0.021246	0.585012	0.282852	0.142244
Novel_002110	XLOC_02359.	transcript	0.205207	0.005489	0.07213	0.068798	0.027606	0.157942	0.219051	0.119464	0.023567
Novel_002111	XLOC_02359.	transcript	0.063413	0	0.135128	0.012769	0.176378	0	0.198126	0.099433	0.01674
Novel_002112	XLOC_02360.	transcript	0.100392	0.05929	0.015076	0.09629	0.065363	0.171961	0.174689	0.115859	0.144128
Novel_002113	XLOC_02362.	transcript	0.11618	0.091365	0.166132	0.101651	0.057493	0.030734	0.20404	0.096486	0.072231
Novel_002114	XLOC_02362.	transcript	0.098911	0.746529	0.323554	0.075675	0.353763	0	0.158974	0.090509	0.087916
Novel_002115	XLOC_02362.	transcript	1.248135	0.227715	0.840962	0.45864	0.863534	0.703129	0.641112	0.81667	1.069679
Novel_002116	XLOC_02362.	transcript	0	1.622754	0	0.185055	0	0	0.66807	0.39801	0

Novel_002117	XLOC_02362.	transcript	0.230395	0.06833	0.06973	0.165652	0.03373	0.019895	0.122869	0.132045	0.038679
Novel_002118	XLOC_02363.	transcript	0.091329	0.113681	0.0286	0.076607	0.157709	0.054613	0.044392	0.130664	0.106971
Novel_002119	XLOC_02364.	transcript	0.633217	0.318533	0.979141	1.639457	0.963948	0.811287	0.838759	1.193551	0.847942
Novel_002120	XLOC_02365.	transcript	0.186421	0.459278	0.283565	0.235525	0.259846	0.202489	0.391999	0.302201	0.273525
Novel_002121	XLOC_02367.	transcript	0.057392	0.025887	0.077076	0.017025	0.122095	0	0.075427	0.035847	0.027966
Novel_002122	XLOC_02368.	transcript	0.007543	0	0	0	0	0	0	0	0
Novel_002123	XLOC_02371.	transcript	0.025183	0.037336	0.05301	0.302186	0.121703	0.070895	0.19606	0.128067	0.039726
Novel_002124	XLOC_02374.	transcript	0.062448	0.054461	0.257201	0.583939	0.03759	0.250554	0.096095	0.091041	0.127066
Novel_002125	XLOC_02374.	transcript	0.568413	0.011929	1.342208	0.589375	0	0.090688	0.088584	0.908233	1.415102
Novel_002126	XLOC_02374.	transcript	0.323489	0	0	0	0	0.46405	0	0	0
Novel_002127	XLOC_02376.	transcript	0.877249	0.795283	0.699546	1.132807	0.792151	0.824531	1.115865	1.32899	0.783109
Novel_002128	XLOC_02379.	transcript	0.74092	0.968825	0.521107	0.796836	0.691489	0.846769	0.98005	0.751879	1.101667
Novel_002129	XLOC_02379.	transcript	0.224805	0.076682	0.402613	0.060037	0.239688	0.111631	0.245253	0.245767	0.07609
Novel_002130	XLOC_02379.	transcript	0.33287	0.091574	0.155959	0.088369	0.118589	0.19061	0.385714	0.27679	0.187187
Novel_002131	XLOC_02383.	transcript	0.341768	0.613639	0.341162	0.427124	0.308368	0.312051	0.433799	0.461949	0.310886
Novel_002132	XLOC_02383.	transcript	0.147168	0.304722	0.213248	0.083482	0.026307	0.335311	0.29648	0.022072	0
Novel_002133	XLOC_02383.	transcript	0	0	0	0	0.040453	0	0	0	0
Novel_002134	XLOC_02383.	transcript	0	0	0	0	0.113754	0	0	0.121823	0.077239
Novel_002135	XLOC_02383.	transcript	0.250328	0.212235	0.138809	0.003538	0.182621	0.24913	0.376305	0.175061	0.070199
Novel_002136	XLOC_02383.	transcript	0.421295	0.314397	0.705145	0.996038	0.559449	0.915572	0.648378	0.726854	0.64164
Novel_002137	XLOC_02385.	transcript	0.010773	0.005248	0	0.010349	0	0	0	0.176908	0.022244
Novel_002138	XLOC_02385.	transcript	0.034386	0.394574	0.011511	0.022066	0.011364	0.028807	0.664529	0.246079	0.630529
Novel_002139	XLOC_02385.	transcript	0.0092	0.005046	0.015114	0.018809	0	0.005342	0.001249	0.212962	0
Novel_002140	XLOC_02385.	transcript	0.527694	0.464856	0.531985	0.536333	0.52187	0.459053	0.068781	0.05748	0.024762
Novel_002141	XLOC_02385.	transcript	0.099524	0.109724	0	0	0	0	0	0	0
Novel_002142	XLOC_02385.	transcript	0.642893	0.463346	0.548709	0.354917	0.481721	0.420087	0.823532	0.646346	0.319425
Novel_002143	XLOC_02385.	transcript	0.019885	0	0	0.296368	0.039093	0	0.026857	0	0
Novel_002144	XLOC_02387.	transcript	8.501994	4.308614	8.310248	7.388543	8.342616	9.243981	7.77255	7.381492	12.05404
Novel_002145	XLOC_02390.	transcript	0.587486	0.511887	0.775628	0.821902	0.929357	0.818371	0.864354	0.915352	0.843802
Novel_002146	XLOC_02390.	transcript	0.058875	0.002669	0	0.483658	0	0	0.104889	0.323889	0
Novel_002147	XLOC_02390.	transcript	0.078429	0	0.040308	0.670879	0.147033	0.042711	0.042056	0.154286	0.003812
Novel_002148	XLOC_02390.	transcript	0	0.004435	0.042549	0.378877	0.220061	0	0.094728	0.186536	0
Novel_002149	XLOC_02390.	transcript	0.097567	0.16147	0.401108	0.351801	0.045767	0.288995	0.06097	0.10849	0.104685
Novel_002150	XLOC_02390.	transcript	0	0.01539	0	0.337232	0.026351	0	0.041626	0.0892	0
Novel_002151	XLOC_02390.	transcript	0	0	0	3.060734	0.232842	0	0.366173	1.658831	0
Novel_002152	XLOC_02390.	transcript	0	0	0	0.027734	0	0	0.012333	0.019065	0
Novel_002153	XLOC_02390.	transcript	0	0	0	0	1.586424	0	0	0	0
Novel_002154	XLOC_02393.	transcript	0.442253	0.128694	0.470649	0.557394	0.557583	0.410451	0.447288	0.459964	0.339198

Novel_002155	XLOC_02394.	transcript	0.231398	0.257343	0.590345	0.048417	0.484877	0.033243	0.695292	0.067433	0.246236
Novel_002156	XLOC_02394.	transcript	0.00234	0.16688	0.000656	0.396176	0.22854	0.561503	0.01246	0.462443	0.01927
Novel_002157	XLOC_02397.	transcript	0.056759	0	0.054813	0.20173	0.091327	0.188409	0.13087	0.013871	0.020267
Novel_002158	XLOC_02399.	transcript	5.06553	1.098288	13.1714	18.73304	16.38627	15.16555	9.732874	7.270899	3.824415
Novel_002159	XLOC_02399.	transcript	0	0	0	0	0	0	0	0	0
Novel_002160	XLOC_02399.	transcript	0	0	0	7.887705	0	0	0	10.55319	2.062308
Novel_002161	XLOC_02399.	transcript	0	0	0	0	0	0	0	0	0
Novel_002162	XLOC_02400.	transcript	0	0	0	0.007404	0	0	0.00327	0	0
Novel_002163	XLOC_02400.	transcript	0	0	0	0	0	0	0	0	0
Novel_002164	XLOC_02400.	transcript	0.0761	0.347143	0.114636	0.036823	0.07612	0.126268	0.042901	0.124568	0.116789
Novel_002165	XLOC_02400.	transcript	0.87057	0.863675	0.715675	0.729903	0.73843	0.747458	0.507912	0.846843	0.603357
Novel_002166	XLOC_02400.	transcript	0	0	0	0	0	0	1.346112	0	0
Novel_002167	XLOC_02425.	transcript	0.849747	0.10599	1.270195	1.615359	0.173662	0.413118	2.04417	0.540062	0.360253
Novel_002168	XLOC_02425.	transcript	0.055915	1.681438	6.482153	13.35349	12.34038	9.056449	12.32114	12.2143	12.21462
Novel_002169	XLOC_02425.	transcript	0	0	0	0	0	0	0.002841	0	0
Novel_002170	XLOC_02425.	transcript	0	0	0	0	0	0	0	1.873484	0
Novel_002171	XLOC_02425.	transcript	14.75153	0	0	0	0	0	0	0	0
Novel_002172	XLOC_02429.	transcript	0.008246	0.243209	0	0.261272	0.186642	0.016085	0.353079	0.077811	0
Novel_002173	XLOC_02430.	transcript	0.273102	0.014921	0.248547	0.229682	0.044719	0.126563	0	0.1113	0
Novel_002174	XLOC_02430.	transcript	0.072644	0.001123	0.003556	0.009065	0	0.005376	0.181133	0.217752	0.254806
Novel_002175	XLOC_02433.	transcript	0.093263	0.033127	0	0	0.068134	0.064299	0	0.250433	0
Novel_002176	XLOC_02433.	transcript	0	0	0.084912	0	0	0	0.141654	0	0.112073
Novel_002177	XLOC_02440.	transcript	0.128488	0.064545	0.041392	0.016567	0	0.119091	0.029418	0.238492	0.298005
Novel_002178	XLOC_02440.	transcript	0	0.026738	0.109769	0	0.148459	0	0.251626	0.029061	0
Novel_002179	XLOC_02447.	transcript	0.285885	0.613283	0.273008	0.149473	0.208259	0.006277	0.255331	0.249745	0.235119
Novel_002180	XLOC_02447.	transcript	0.258794	0.347733	0.200159	0.111245	0.09184	0.062766	0.130599	0.130918	0.153536
Novel_002181	XLOC_02452.	transcript	0.144569	0.113958	0.410368	0.125895	0.325025	0.275763	0.651439	0.23023	0.234926
Novel_002182	XLOC_02463.	transcript	0.138133	2.20149	0.08299	0.024797	0.070576	0.023878	0.231731	0.030051	0.070559
Novel_002183	XLOC_02464.	transcript	0.966768	0.220005	0.780657	0.666958	0.595031	1.130676	0.980548	0.253022	0.258995
Novel_002184	XLOC_02464.	transcript	0.880823	0.741939	1.358826	0.748285	1.282175	0.650596	1.040291	1.008285	0.881187
Novel_002185	XLOC_02464.	transcript	0.776228	0.758656	0.815514	0.638925	0.594491	0.869949	0.594906	1.035101	0.829529
Novel_002186	XLOC_02464.	transcript	0.478098	1.989404	0.797283	1.429305	1.667239	0.795389	1.116121	1.492446	1.865796
Novel_002187	XLOC_02466.	transcript	0.902883	0.323171	0.723326	0.881299	0.963436	0.692128	0.811801	0.846	1.077772
Novel_002188	XLOC_02466.	transcript	0	0	0	0	0	0	0.429	0	0
Novel_002189	XLOC_02467.	transcript	1.06373	0.273988	0.938427	0.894873	0.818211	0.855765	1.204052	0.796635	0.819618
Novel_002190	XLOC_02475.	transcript	0.593293	0.70619	0.575402	0.65045	0.640601	0.718464	0.767861	0.81089	0.420873
Novel_002191	XLOC_02475.	transcript	0	0	0.039492	0	0.114575	0.015023	0.027937	0	0
Novel_002192	XLOC_02475.	transcript	0	0	0	0	0	0	0	0	0

Novel_002193	XLOC_02475.	transcript	0	0.020222	0	0	0	0	0	0	0.02272
Novel_002194	XLOC_02475.	transcript	0	0.034179	0	0	0	0	0	0.01561	0
Novel_002195	XLOC_02475.	transcript	0.07611	0	0.069392	0.030027	0.047498	0.027957	0.02807	0.040445	0.144935
Novel_002196	XLOC_02475.	transcript	0.027137	0.059749	0	0	0.022529	0	0	0.085104	0
Novel_002197	XLOC_02475.	transcript	0.067573	0	0.058677	0	0.009513	0.033659	0.108342	0.008471	0.008097
Novel_002198	XLOC_02475.	transcript	0.10014	0.018253	0.056059	0.062316	0.029506	0.032044	0.055341	0.041742	0.01171
Novel_002199	XLOC_02475.	transcript	0.164192	0.104669	0.058318	0.124569	0.101861	0.194393	0.32558	0.060794	0.035282
Novel_002200	XLOC_02481.	transcript	0	0	0.000281	0	0	0	0.209985	0	0
Novel_002201	XLOC_02481.	transcript	0.117404	0.101547	0.396887	0.120434	0.107213	0.394178	0	0.105991	0.155184
Novel_002202	XLOC_02486.	transcript	0.175614	0.014017	0.000822	0.027753	0.121805	0.00729	0.015678	0	0
Novel_002203	XLOC_02486.	transcript	0.266074	0.268322	0.369032	0.097318	0.263384	0.380646	0.009987	0.114174	0.372412
Novel_002204	XLOC_02486.	transcript	0.021115	0.141808	0	0.137636	0.003839	0.095447	0.39261	0.333511	0
Novel_002205	XLOC_02486.	transcript	0	0	0	0.179687	0	0	0	0	0
Novel_002206	XLOC_02486.	transcript	0	0	0	0	0	0	0	0	0
Novel_002207	XLOC_02486.	transcript	0.073987	0.115411	0.478188	0.296004	0.270255	0.422145	0.32399	0.300669	0.316059
Novel_002208	XLOC_02486.	transcript	0.549571	0.316826	0	0.106621	0	0	0	0	0
Novel_002209	XLOC_02487.	transcript	2.363064	1.33311	0.629988	1.195679	0.783295	1.871786	1.019698	1.053474	0.29128
Novel_002210	XLOC_02487.	transcript	0.270093	0.687638	0.218861	0.36834	0.268439	0.216049	0.234014	0.471279	0.093198
Novel_002211	XLOC_02487.	transcript	0.297666	0.073198	0.114313	0.381617	0.190214	0.152867	0.149352	0.154892	0.099473
Novel_002212	XLOC_02487.	transcript	0.284675	0.145461	0.092573	0.183758	0.096031	0.081578	0.137634	0.336008	0.23038
Novel_002213	XLOC_02487.	transcript	0.112596	3.171042	0.139984	0.257291	0.166649	0.36028	0.538355	0.269771	0.01782
Novel_002214	XLOC_02487.	transcript	0	0	0	0	2.299472	0	0	0	0
Novel_002215	XLOC_02491.	transcript	0.080669	0.078861	0.19796	0.088746	0.294271	0.225148	0.101918	0.034556	0.067605
Novel_002216	XLOC_02491.	transcript	0	0	0	0	0	0	0.041769	0	0
Novel_002217	XLOC_02496.	transcript	0.115389	0.036083	0	0.205547	0	0.030371	0.202442	0.093788	0.072303
Novel_002218	XLOC_02496.	transcript	0	0	0.326787	0	0.126858	0	0.034701	0	0
Novel_002219	XLOC_02506.	transcript	0.267289	0.130399	0.174082	0.315435	0.242973	0.216874	0.202593	0.3561	0.126319
Novel_002220	XLOC_02506.	transcript	0	0	0	0	0	0	0.020487	0	0
Novel_002221	XLOC_02508.	transcript	0.095974	0.048332	0.19898	0.149242	0.162817	0.069619	0.098805	0.208399	0.191124
Novel_002222	XLOC_02508.	transcript	0	0.142061	0	0	0	0	0	0	0.131158
Novel_002223	XLOC_02508.	transcript	0.038952	0.062368	0.044487	0.094401	0.068518	0.194011	0.118417	0.147374	0.238116
Novel_002224	XLOC_02510.	transcript	0	0	0.029423	0	0	0.321824	0	0	0.179138
Novel_002225	XLOC_02510.	transcript	0.192271	0.123701	0.228158	0.22766	0.15798	0	0.325292	0.210822	0
Novel_002226	XLOC_02510.	transcript	0	0	0	0	0	0	0	0	0
Novel_002227	XLOC_02515.	transcript	0.101936	0.032844	0.118433	0.178407	0.143235	0.145678	0.150145	0.304257	0.308325
Novel_002228	XLOC_02515.	transcript	0.212833	0	0.095665	0.05547	0.132331	0.129174	0.153611	0.074144	0.234122
Novel_002229	XLOC_02516.	transcript	0	0.056529	0.318141	0.379558	0.291082	0.517075	0.041283	0.250372	0.335627
Novel_002230	XLOC_02516.	transcript	0.216134	0	0.225521	0.007283	0.108158	0.060857	0.321019	0.173524	0.119284

Novel_002231	XLOC_02530.	transcript	0.493763	0.702968	0.583431	0.223166	0.708053	0.794235	0.813044	0.72732	0.547729
Novel_002232	XLOC_02530.	transcript	0	0.111704	0.007296	0	0.002685	0	0.012031	0	0.003906
Novel_002233	XLOC_02530.	transcript	0	0	0	0	0	0	0	0	0
Novel_002234	XLOC_02530.	transcript	0.01603	0.078603	0.034359	0	0.009658	0.005119	0.070525	0.015865	0.057677
Novel_002235	XLOC_02530.	transcript	0	0	0.014055	5.80E-05	0	0	0.000556	0	0.024336
Novel_002236	XLOC_02530.	transcript	0.144258	0.425465	0.077307	0.058035	0.069696	0.075346	0.146711	0.117376	0.299606
Novel_002237	XLOC_02530.	transcript	0	0	0	0	0	0	0	0	0
Novel_002238	XLOC_02530.	transcript	0.448902	0	0.00023	0.152684	0	0.116479	0	0	0
Novel_002239	XLOC_02530.	transcript	0	0.002042	0	0	0.000159	5.00E-04	0.007341	0.001032	0.010271
Novel_002240	XLOC_02530.	transcript	0	0	0	0	0	0	0	0	0
Novel_002241	XLOC_02530.	transcript	0	0	0.15875	0	0.347334	0	0.248431	0	0.212765
Novel_002242	XLOC_02530.	transcript	0	0	0.004126	0	0.232633	0	0	0	0.016016
Novel_002243	XLOC_02530.	transcript	0	0	0.56656	0	0.181346	0	0	0	0
Novel_002244	XLOC_02530.	transcript	0.114912	0.017682	0.115823	0.394982	0.24454	0.478604	0.539168	0.16385	0.018627
Novel_002245	XLOC_02530.	transcript	0.00113	0.000777	0.001712	0.001597	0.206228	0	0.001207	0.389055	0.007413
Novel_002246	XLOC_02530.	transcript	0.003176	0	0	0	0.193525	0	0	0	0
Novel_002247	XLOC_02530.	transcript	0.045315	0	0.234353	0	0.136539	0	0	0	0
Novel_002248	XLOC_02530.	transcript	0.319306	0.100556	0	0	0	0.275065	0.417046	0.184527	0
Novel_002249	XLOC_02530.	transcript	1.221156	0.132268	0.071213	0	0	0	0.609749	0.462753	0
Novel_002250	XLOC_02530.	transcript	0	0	0	0	0	0	0	0.17035	0
Novel_002251	XLOC_02530.	transcript	0	0	0	0	0	0.687813	0.273137	0	0.058606
Novel_002252	XLOC_02530.	transcript	0	0	0	0	0	0.409439	0.171665	0	0
Novel_002253	XLOC_02530.	transcript	0.253313	0.232067	0.121372	0	0.083622	0.245513	0.39664	0.084237	0.041807
Novel_002254	XLOC_02530.	transcript	0	0	0	0	0.04342	0	0.319821	0	0.141846
Novel_002255	XLOC_02530.	transcript	4.508197	4.973992	0.118177	0	0	3.487397	8.197409	2.036612	2.218226
Novel_002256	XLOC_02530.	transcript	0.391754	0.91144	0.085628	0	0.170004	0.159342	1.161904	0.030216	0.066259
Novel_002257	XLOC_02531.	transcript	0.815649	0	0	0	0.012246	0	0.25786	0.093222	0.04214
Novel_002258	XLOC_02531.	transcript	0.724576	0.039324	0	0	0	0.981505	1.45086	0.635862	0
Novel_002259	XLOC_02539.	transcript	0.378878	0.331607	0	0.31656	0.251883	0	0.285222	0.249105	0.094156
Novel_002260	XLOC_02539.	transcript	0.000287	0.002202	0.025571	0.058569	0.058968	0.404944	0.027007	0.002477	0.060298
Novel_002261	XLOC_02539.	transcript	0.455475	0.287165	0.158384	0.07485	0.243812	0.345652	0.307172	0.389828	0.561644
Novel_002262	XLOC_02539.	transcript	0.537131	0.523132	0.622423	0.219161	0.27852	0.38477	0.508503	0.507476	0
Novel_002263	XLOC_02540.	transcript	0.192953	0	0.212514	0.074304	0	0.170876	0.145354	0.162381	0.058987
Novel_002264	XLOC_02540.	transcript	0	0	0	0.029224	0.188267	0	0	0.045249	0
Novel_002265	XLOC_02540.	transcript	0	0	0	0	0.065622	0	0	0	0
Novel_002266	XLOC_02540.	transcript	0.203029	0	0.143542	0.110816	0.090948	0.148319	0.132043	0.168447	0.015953
Novel_002267	XLOC_02543.	transcript	0	0	1.852559	0	0	0.683152	0	0.802327	0.004585
Novel_002268	XLOC_02543.	transcript	0	0.002084	0	0.003679	0	0.158379	0	0	0

Novel_002269	XLOC_0254[illegible]	transcript	1.669854	0.377197	0	0.297893	1.354001	0	0.428542	0	0.984217
Novel_002270	XLOC_0254[illegible]	transcript	0.426601	0	0.149308	0	0	0	0.192178	0.125078	0.66518
Novel_002271	XLOC_0254[illegible]	transcript	0	0	0	1.799368	0	0	0	0	0
Novel_002272	XLOC_0254[illegible]	transcript	1.910727	0	0.922965	1.280714	1.686004	2.601507	3.842289	2.621577	0.914675
Novel_002273	XLOC_0254[illegible]	transcript	0.0824	0.122431	0	0	0	0.030628	0.00688	0.004773	0.012491
Novel_002274	XLOC_0254[illegible]	transcript	0.043481	0.747462	0.055187	0.029134	0.053508	0.042081	0.063042	0.04716	0.062484
Novel_002275	XLOC_0254[illegible]	transcript	0	0	0	0	0	0	0	0.338989	0
Novel_002276	XLOC_0254[illegible]	transcript	0.057652	0	0.053758	0	0	0	0	0	0.010649
Novel_002277	XLOC_0254[illegible]	transcript	0.389856	0.045237	0	0.006787	0	0	0.167604	0.23056	0
Novel_002278	XLOC_0254[illegible]	transcript	0.082133	0.011467	0.150964	0.157132	0.007419	0	0.050763	0.361738	0
Novel_002279	XLOC_0254[illegible]	transcript	0	0	0	0.326503	0.000589	0	0	0	0.19022
Novel_002280	XLOC_0254[illegible]	transcript	0.487838	0.192083	0.471253	0.280692	0.605789	0.844495	0.780858	0.606989	0.365641
Novel_002281	XLOC_0254[illegible]	transcript	0	0	0	0.160766	0	0	0	0	0
Novel_002282	XLOC_0255[illegible]	transcript	0.464889	0.937676	0.558358	0.62966	0.616139	0.291066	0.222444	0.711107	0.458679
Novel_002283	XLOC_0255[illegible]	transcript	0.012689	0.004132	0	0	0.025956	0.041103	0.647689	0.005494	0.009782
Novel_002284	XLOC_0255[illegible]	transcript	0	0.010202	0	0.279755	0	0	0	0.362948	0
Novel_002285	XLOC_0255[illegible]	transcript	0	0.404456	0.439672	0.295652	0.037066	0.411619	0.380029	0.473991	0.219396
Novel_002286	XLOC_0255[illegible]	transcript	0	0	0	0	0.003015	0	0.035404	0	0
Novel_002287	XLOC_0255[illegible]	transcript	0	0	0	0	0.214902	0	0	0	0
Novel_002288	XLOC_0255[illegible]	transcript	0.655603	0.117175	0.161599	0.016123	0.014009	0.179028	0.311345	0.097874	0.120414
Novel_002289	XLOC_0255[illegible]	transcript	0	0	0	0.394975	0.603016	0	0	0.217243	0
Novel_002290	XLOC_0255[illegible]	transcript	0.364047	0.270502	0.459398	0.336065	0.471847	0.277017	0.431813	0.404779	0.582638
Novel_002291	XLOC_0255[illegible]	transcript	0.028852	0.276011	0.033267	0.16294	0.103055	0.091724	0.283509	0.224068	0.17497
Novel_002292	XLOC_0255[illegible]	transcript	0	0	0	0	0	0	0	0	0
Novel_002293	XLOC_0255[illegible]	transcript	0	0.517601	0	0.197587	0	0	0	0.349572	0
Novel_002294	XLOC_0255[illegible]	transcript	0	0	0	0.316753	0	0	0	0	0
Novel_002295	XLOC_0255[illegible]	transcript	3.344481	2.01084	4.085931	7.171531	3.952981	4.115134	3.205817	3.567179	1.559338
Novel_002296	XLOC_0257[illegible]	transcript	0	0	0	0	0	0	0	0	0.003902
Novel_002297	XLOC_0257[illegible]	transcript	0.581432	0	0.066584	0.097661	0.106922	0.062641	0.637486	0.156019	0.140059
Novel_002298	XLOC_0257[illegible]	transcript	0	0.008968	0.002199	0	0.163462	0	0	0	0.008336
Novel_002299	XLOC_0257[illegible]	transcript	0	0	0	2.138208	0	0.141004	0	1.00E-06	0.052624
Novel_002300	XLOC_0257[illegible]	transcript	0	0	0	0	0	0	0	0	0.016645
Novel_002301	XLOC_0257[illegible]	transcript	0	0	0	0	0	0	0.000256	0	0
Novel_002302	XLOC_0257[illegible]	transcript	0	0	0	0	0	0.084883	0	0	0.297566
Novel_002303	XLOC_0257[illegible]	transcript	0	0	0	0.344284	0	0.010693	0	0	0
Novel_002304	XLOC_0257[illegible]	transcript	0.003844	4.00E-06	0	0	0.003375	0	0	0	0
Novel_002305	XLOC_0257[illegible]	transcript	0	0	0	0	0	0	0.477551	0	0.004176
Novel_002306	XLOC_0257[illegible]	transcript	0	0.010137	0	0	0	0.380284	0	0	0

Novel_002307	XLOC_02570.	transcript	0	0	0	0	9.00E-04	0.00141	0	0.017037	0.003404
Novel_002308	XLOC_02570.	transcript	0.872465	0	0	0	0	0.347996	0.223728	0	0
Novel_002309	XLOC_02570.	transcript	0	0	0	0	3.70E-05	0.08846	0.50526	0	0.007395
Novel_002310	XLOC_02570.	transcript	0.41081	0.00468	0.451923	0.171909	0.049486	0.55892	0.346961	0.195972	0.904892
Novel_002311	XLOC_02570.	transcript	0.367335	0	0.165496	0.359368	1.28679	0.743685	0.704259	0.151927	0.015741
Novel_002312	XLOC_02570.	transcript	0	0	0	0	0	0	0	0	0.006976
Novel_002313	XLOC_02570.	transcript	0	0.097065	0.847545	0.359119	0	0	0	0.000298	0.32351
Novel_002314	XLOC_02570.	transcript	2.130702	0.074644	0.434583	0.250201	1.021374	0	0	0	0.072411
Novel_002315	XLOC_02570.	transcript	0.319255	0	0.021367	0	0.140182	0.108516	0.236831	0.070423	0
Novel_002316	XLOC_02570.	transcript	0.002272	0.000763	0	0.298563	0	0	0	0	0.009242
Novel_002317	XLOC_02570.	transcript	0.003648	0	0	0	0	0	0	0	0.007387
Novel_002318	XLOC_02570.	transcript	0	0	0	0	0	0	0	0	5.00E-06
Novel_002319	XLOC_02570.	transcript	0	0	0	0.006542	0	0	0	0	0.073763
Novel_002320	XLOC_02570.	transcript	0	0	0	0	0	0	0	0	0.005566
Novel_002321	XLOC_02570.	transcript	0	0	0.483317	0.00031	0	2.105754	0	0	0.010739
Novel_002322	XLOC_02570.	transcript	0	0	0.196447	3.00E-05	0.086292	0	0	0.156842	0
Novel_002323	XLOC_02570.	transcript	0.259176	4.80E-05	0.192276	0.012291	0.113837	0.335449	0.173633	0.159567	0.01435
Novel_002324	XLOC_02570.	transcript	0.010783	0.007418	0.010321	0.00322	0.003542	0.877698	0.005183	0.003266	0.190396
Novel_002325	XLOC_02570.	transcript	0	0.017602	2.074354	0	0.555619	0	0	3.116488	0.008797
Novel_002326	XLOC_02570.	transcript	0	0	0	0	0.097493	0	0	0	0
Novel_002327	XLOC_02570.	transcript	0	0	0.018928	0.018477	2.872355	0	0.01655	0	0.08707
Novel_002328	XLOC_02570.	transcript	0	0	0	0	2.03155	0	0	0	0
Novel_002329	XLOC_02570.	transcript	0	0.003735	5.00E-06	0.668711	0	0	1.00E-06	0.002099	0
Novel_002330	XLOC_02577.	transcript	0.471493	0.209911	0.408635	0.181352	0.555598	0.291472	0.141636	0.282684	0.388149
Novel_002331	XLOC_02577.	transcript	0.008383	0.258148	0.229565	0.490746	0.279461	0.47271	0.432731	0.356127	0.138701
Novel_002332	XLOC_02601.	transcript	0.232825	0.974146	0.26611	0.139844	0.258335	0.166132	0.247948	0.226853	0.34339
Novel_002333	XLOC_02601.	transcript	0	0	0	0	0	0	0	0	0.685042
Novel_002334	XLOC_02606.	transcript	0.42653	0.350787	0.111289	0.138686	0.272617	0.670311	0.09851	0.453378	0.356881
Novel_002335	XLOC_02606.	transcript	0.042004	0.070256	0.115625	0.361068	0.155919	0.119498	0.482639	0.292895	0.473753
Novel_002336	XLOC_02606.	transcript	0	0	0.564159	0.157113	0.120907	0	0	0	0
Novel_002337	XLOC_02606.	transcript	0	0	0	0	0.112381	0	0	0	0
Novel_002338	XLOC_02606.	transcript	0.477003	0.545498	0.621689	0.592292	0.464291	0.35839	0.418687	0.459525	0.636977
Novel_002339	XLOC_02606.	transcript	0.273329	0.568431	0.134965	0.040961	0.194041	0.193417	0.14351	0.208457	0.120771
Novel_002340	XLOC_02606.	transcript	0	0	0	0	0	0	0	0	0
Novel_002341	XLOC_02607.	transcript	0.287349	0.125723	0.661624	0.610713	0.606516	0.662032	0.576545	0.739164	0.333026
Novel_002342	XLOC_02612.	transcript	0.565841	0.957395	0.430605	0.311982	0.39515	0.473714	0.505763	0.362264	0.356469
Novel_002343	XLOC_02613.	transcript	0.238507	0.006483	0	0.405146	0.560804	0	0.38782	0.407564	0.280948
Novel_002344	XLOC_02615.	transcript	0.154816	1.926672	0.164189	0.086564	0.123304	0.107455	0.20914	0.062742	0.166113

Novel_002345	XLOC_02617.	transcript	0.321342	0.584833	0.327266	0.248733	0.201605	0.162057	0.140497	0.322375	0.281935
Novel_002346	XLOC_02618.	transcript	0.200267	0.806331	0.096862	0.131495	0.084905	0.053586	0.205515	0.117183	0.097022
Novel_002347	XLOC_02619.	transcript	0.250413	0	4.456695	1.80157	1.749596	0	0.558665	0	0.07811
Novel_002348	XLOC_02619.	transcript	0.118237	0.347577	0.339334	0.230153	0.219271	0.264006	0.168853	0.34448	0.108469
Novel_002349	XLOC_02622.	transcript	0.351237	0.095386	0.238297	0.12608	0.179705	0.21199	0.206495	0.18395	0.10223
Novel_002350	XLOC_02628.	transcript	0	0	0	0	0	0	0	0	0
Novel_002351	XLOC_02628.	transcript	0.304389	0.207315	0.197907	0.385437	0.427787	0.510339	0.517655	0.029926	0.306044
Novel_002352	XLOC_02628.	transcript	0	0	0.270371	0	0	0	0	0.000114	0
Novel_002353	XLOC_02628.	transcript	0	0.069755	0	0.002579	0.044372	0	0	0.192669	0.037269
Novel_002354	XLOC_02634.	transcript	0.238317	0.113048	0.300687	0.242538	0.654546	0.484046	0.119354	0.138851	0.154477
Novel_002355	XLOC_02635.	transcript	0	0	0	0.40559	0	0	0	0	0
Novel_002356	XLOC_02635.	transcript	336.2302	230.6771	356.8264	251.1667	359.8771	272.4821	193.6612	244.166	274.3806
Novel_002357	XLOC_02635.	transcript	0.044263	0.110422	0.093903	0.041564	0	0.051831	0.033214	0	0.052632
Novel_002358	XLOC_02636.	transcript	0.119484	0.008412	0.317648	0.562353	0.383836	0.485375	0.371115	0.251206	0.219725
Novel_002359	XLOC_02636.	transcript	0.374478	0	0.908186	1.359639	0.746274	0.614137	0.113413	0.635676	0.291643
Novel_002360	XLOC_02636.	transcript	0.020345	0	0.14158	0.263417	0.200613	0.063469	0.101553	0.388102	0.059869
Novel_002361	XLOC_02636.	transcript	0.00638	0	0.035331	0.152396	0.14444	0.042865	0.218137	0.074334	0.104868
Novel_002362	XLOC_02636.	transcript	0	0	0	0	0	0	0	0.045157	0
Novel_002363	XLOC_02636.	transcript	0.054687	0	0.020666	0.4028	0.012624	0.086177	0.108663	0.261582	0.033825
Novel_002364	XLOC_02636.	transcript	0	0	0.066495	0.975409	0.163254	0	0	0.497241	0.069052
Novel_002365	XLOC_02636.	transcript	0.145736	0	0.242704	0.200769	0.269986	0.423964	0.055261	0.00776	0
Novel_002366	XLOC_02636.	transcript	0.02895	0	0.014154	0.095453	0.045443	0.268918	0.006372	0.026684	0
Novel_002367	XLOC_02636.	transcript	0.005333	0	0.004693	0.183477	0.008931	0.068224	0.110547	0.056661	0
Novel_002368	XLOC_02636.	transcript	0	0	0	0	0	0	0.08187	0.141115	0
Novel_002369	XLOC_02638.	transcript	0	0	0	0	0.268072	0.355261	0.612059	0.372155	0
Novel_002370	XLOC_02638.	transcript	0.480252	0.469401	0.416831	0.408024	0.146127	0.092021	0.001282	0.083956	0.306874
Novel_002371	XLOC_02638.	transcript	0.098835	0	0	0	0	0.023776	0	0	0
Novel_002372	XLOC_02638.	transcript	0.388514	0.382802	0.310082	0.511951	0.321211	0.316772	0.333437	0.358015	0.557989
Novel_002373	XLOC_02638.	transcript	1.36353	0.953523	0.002294	0.009871	1.331561	2.492239	0.004677	1.280812	2.135637
Novel_002374	XLOC_02638.	transcript	3.777682	1.620978	2.540921	4.303984	4.127606	3.246099	2.981102	4.508963	4.039459
Novel_002375	XLOC_02638.	transcript	0.02351	1.047751	1.86427	1.740245	0	0.003463	1.525095	0	0
Novel_002376	XLOC_02638.	transcript	0	0.002412	0	0	0	0	0.002132	0	0.40898
Novel_002377	XLOC_02638.	transcript	0.833394	0.705974	0.895577	3.090339	1.429233	1.137598	1.061518	1.322915	0.460879
Novel_002378	XLOC_02640.	transcript	0.192294	0.040748	0.118395	0.205265	0.181511	0.124857	0.201144	0.164647	0.016656
Novel_002379	XLOC_02649.	transcript	2.173039	0.05513	2.871675	2.210003	2.553353	2.67848	3.265227	2.066203	1.818104
Novel_002380	XLOC_02649.	transcript	0.453889	0	0.141682	0.008806	0.687323	0.306972	1.120148	0.606135	0.021316
Novel_002381	XLOC_02649.	transcript	0.154895	0	0	0.009128	0	0	0	0	0.021301
Novel_002382	XLOC_02649.	transcript	0	0	0.46923	0.191841	0	0	0	0	0

Novel_002383	XLOC_02649.	transcript	0.231079	0.019728	0.270417	0	0.409699	0.143562	0.282696	0.059504	0.10363
Novel_002384	XLOC_02649.	transcript	0	0	0	0.190675	0	0	0	0	0
Novel_002385	XLOC_02656.	transcript	0.257083	0.083332	0.200221	0.473764	0.146031	0.28791	0.329058	0.298173	0.374547
Novel_002386	XLOC_02656.	transcript	0	0	0	0	0	0	0.265396	0	0
Novel_002387	XLOC_02656.	transcript	0	0	0	0	0	0	0.283272	0.001069	0
Novel_002388	XLOC_02656.	transcript	0.3822	0	0	0	0	0	0.116861	0.015294	0
Novel_002389	XLOC_02656.	transcript	0.016723	0.121097	0.6425	0.467964	0.512429	0.742563	0.166159	0.662732	0.495438
Novel_002390	XLOC_02659.	transcript	0.282385	0.020821	0.254958	0.233101	0.089833	0.099751	0.407001	0.232141	0.221042
Novel_002391	XLOC_02665.	transcript	0.051774	0.152378	0.111975	0.124006	0.097267	0.073225	0.047834	0.137125	0.193263
Novel_002392	XLOC_02668.	transcript	0.439338	0.321747	0.419992	0.255294	0.096726	0.392759	0.367911	0.275736	0.238605
Novel_002393	XLOC_02668.	transcript	0	0	0	0.117516	0	0	0	0	0.279789
Novel_002394	XLOC_02670.	transcript	0.300399	0.145692	0.427203	0.45	0.442204	0.441948	0.383821	0.480438	0.235401
Novel_002395	XLOC_02672.	transcript	3.157412	0.084115	0.208546	0.419932	0	0.52738	4.689581	2.929124	0.938241
Novel_002396	XLOC_02672.	transcript	0.112636	1.143877	2.517334	2.535397	0.408638	2.581128	0.128964	0.129722	2.472151
Novel_002397	XLOC_02672.	transcript	0	0	0	0	0	0	0	0	0
Novel_002398	XLOC_02672.	transcript	0	0	0	0	2.756191	0	0	0	0
Novel_002399	XLOC_02673.	transcript	0	0	0.917864	0.639747	0	0.126698	0.006777	0.064098	0.150028
Novel_002400	XLOC_02675.	transcript	0.204677	0.06825	0.331633	0.229153	0.205117	0.355397	0.348915	0.327377	0.300246
Novel_002401	XLOC_02675.	transcript	0.112418	0.081736	0.205186	0	0.038787	0.128146	0.063725	0.192299	0.044745
Novel_002402	XLOC_02675.	transcript	0.036144	0.155731	0.111638	0.090827	0.037029	0.065522	0.054154	0.062186	0.25744
Novel_002403	XLOC_02677.	transcript	0	0.059402	0.038378	0.029112	0.000477	0	0.009885	0.452055	0
Novel_002404	XLOC_02677.	transcript	0.15359	0.084405	0.05311	0.294738	0.105465	0.122656	0.243158	0.165167	0.206751
Novel_002405	XLOC_02678.	transcript	0	4.50E-05	0	0	0	0	0	0	0
Novel_002406	XLOC_02678.	transcript	0.457966	0.202234	0.17249	0.333301	0.306488	0.206852	0.327719	0.323395	0.292029
Novel_002407	XLOC_02678.	transcript	0	0	0	0	0	0	0	0.245934	0.012248
Novel_002408	XLOC_02679.	transcript	0.042162	0	0.089149	0	0.121798	0	0.066284	0.13479	0
Novel_002409	XLOC_02680.	transcript	0.118806	0.142149	0.172839	0.033279	0.004834	0.01031	0.06404	0.184299	0.275092
Novel_002410	XLOC_02680.	transcript	0.340944	0.039146	0.046619	0.072669	0.037515	0.142895	0.118818	0.027043	0.011195
Novel_002411	XLOC_02683.	transcript	3.623729	30.99893	6.623399	6.543309	6.316351	13.20663	5.475974	7.158835	5.620703
Novel_002412	XLOC_02685.	transcript	0.269429	0	0.007619	0.007751	0	0.360973	0.30201	0.034928	0
Novel_002413	XLOC_02685.	transcript	0	0.240779	0.277188	0.384327	0.227644	0	0.054133	0.389032	0.325598
Novel_002414	XLOC_02687.	transcript	0.073445	0.333497	0.096206	0.473223	0.095554	0.132428	0.225264	0.112636	0.296866
Novel_002415	XLOC_02687.	transcript	0	0	0.405439	0.419729	0.35829	0.592406	0.211367	0.120952	0.55877
Novel_002416	XLOC_02687.	transcript	0	0	0.087396	0.186838	0.214828	0.186046	0.112886	0.079999	0.187326
Novel_002417	XLOC_02688.	transcript	1.144633	0	0	2.710493	2.70E-05	0	0	3.545158	0
Novel_002418	XLOC_02688.	transcript	0	3.243555	0	0	0	8.699828	0	3.607364	1.059244
Novel_002419	XLOC_02688.	transcript	0	0.007083	0	0	0	0	0	0	13.93332
Novel_002420	XLOC_02688.	transcript	4.067522	3.508329	0	0.024188	0	0	0.879448	0	0.011676

Novel_002421	XLOC_02688.	transcript	0	0.854184	0	0.067132	0.380678	4.956208	5.974972	0	0.052489
Novel_002422	XLOC_02688.	transcript	0	0	0	0	0	21.72173	0	0	0
Novel_002423	XLOC_02688.	transcript	0	0	0	0	0	0	0	0	0
Novel_002424	XLOC_02688.	transcript	9.346796	4.157953	20.32827	0.006613	0.001696	0	0.896936	0	0
Novel_002425	XLOC_02688.	transcript	0	2.552043	0.963205	1.910417	0.019724	0	0	0.082755	1.087227
Novel_002426	XLOC_02688.	transcript	1.593848	1.288767	1.042074	0.171182	1.903563	1.63506	1.000114	1.237283	1.207712
Novel_002427	XLOC_02689.	transcript	0.201824	0.073393	0.249119	0.189543	0.262661	0.423793	0.831216	0.801793	0.048024
Novel_002428	XLOC_02689.	transcript	5.457606	2.909887	3.221693	3.447953	2.545918	2.931148	7.802312	2.581776	20.69396
Novel_002429	XLOC_02689.	transcript	0	0	0	0	0	0	0	0	0.488347
Novel_002430	XLOC_02689.	transcript	0.981355	0	0	0	0	0	0	0	0
Novel_002431	XLOC_02689.	transcript	0	0	0	0	0	0	0	0	0
Novel_002432	XLOC_02689.	transcript	0.052876	0.633554	0.62649	0.566552	0.710041	0.523289	0.879066	0.852074	0.090202
Novel_002433	XLOC_02690.	transcript	2.547557	2.568969	3.550629	5.054191	4.191081	3.297088	3.583009	4.025721	4.832553
Novel_002434	XLOC_02690.	transcript	0.095328	0.063951	0.229531	0.45418	0.267382	0.237389	0.359591	0.341769	0.26788
Novel_002435	XLOC_02691.	transcript	0.118735	0.280908	0.216541	0.018643	0.118787	0.290209	0.2565	0.080478	0.149711
Novel_002436	XLOC_02691.	transcript	0.499166	0.811867	0.451001	0.621974	0.552813	0.609344	0.511476	0.457311	0.24033
Novel_002437	XLOC_02692.	transcript	2.281713	0.511168	3.15194	2.315953	3.375885	2.41388	3.249305	2.229573	1.678378
Novel_002438	XLOC_02697.	transcript	0.201559	0.16594	0.07837	0.134446	0.179792	0.212759	0.161778	0.206362	0.123735
Novel_002439	XLOC_02697.	transcript	0.097892	0	0	0.000258	0	0	0	0	0
Novel_002440	XLOC_02698.	transcript	0.107813	0.036053	0.076474	0.11286	0.097834	0.110726	0.866679	0.990519	0.00829
Novel_002441	XLOC_02704.	transcript	0.456063	0.659566	0.81432	0.670734	0.906217	0.664863	0.655219	0.461256	0.461125
Novel_002442	XLOC_02705.	transcript	0.059057	0.208509	0.577045	0.30836	0.136778	0.435994	0.4274	0.190742	0.139202
Novel_002443	XLOC_02711.	transcript	0.429256	43.74393	1.081124	0	0.943317	3.939694	0.300459	0.658261	22.71036
Novel_002444	XLOC_02712.	transcript	0.138075	0.309145	0.907495	0.367304	0.553324	0.378601	0.055942	0.017181	0
Novel_002445	XLOC_02712.	transcript	0.178863	0.044296	0.606578	0.596067	0.214576	0.180965	0.283741	0.048773	0.353013
Novel_002446	XLOC_02714.	transcript	0.016675	0.01288	0	0.003145	0.003458	0	0.001426	0.000898	0
Novel_002447	XLOC_02714.	transcript	0.120479	0.170384	0.212571	0.275519	0.140185	0.132276	0.441411	0.196649	0.286645
Novel_002448	XLOC_02714.	transcript	0.11295	0.269564	0.033464	0	0.104408	0.183058	0.020168	0.048216	0.026705
Novel_002449	XLOC_02714.	transcript	0	0	0	0.005305	0.005456	0	0	0	0
Novel_002450	XLOC_02715.	transcript	0.086455	0.132709	0.263261	0.408031	0.225048	0.229092	0.212057	0.27586	0.737659
Novel_002451	XLOC_02715.	transcript	0.896545	0.817962	1.578039	1.522278	1.582997	1.525149	1.381923	1.254252	2.796357
Novel_002452	XLOC_02715.	transcript	0	0	0	0	0	0	0.849528	0	0
Novel_002453	XLOC_02715.	transcript	0.085665	0.275607	0	0.068043	0	0.399645	0.104308	0	0
Novel_002454	XLOC_02717.	transcript	0.045037	0	0.059167	0.020084	0.067017	0.172857	0.049579	0.310627	0.077408
Novel_002455	XLOC_02717.	transcript	0.434073	0.347324	0.35846	0.331263	0.34083	0.164846	0.324345	0	0.453235
Novel_002456	XLOC_02717.	transcript	0.008814	0	0.021087	0	0	0	0.41542	0	0
Novel_002457	XLOC_02717.	transcript	0	0.022523	0	0.181903	0.266658	0.041126	0.008127	0.127192	0
Novel_002458	XLOC_02718.	transcript	0	0.195777	0.166574	0.32379	0.085802	0	0	0.107588	0.082907

Novel_002459	XLOC_02718.	transcript	0	0.16237	0	0	0	0	0	0	0
Novel_002460	XLOC_02718.	transcript	0.317299	0.205419	0.017879	0.136524	0.150403	0.045321	0.290385	0.053651	0
Novel_002461	XLOC_02718.	transcript	0.188933	0.312614	0.085288	0.065795	0.114704	0.100823	0.142419	0.18621	0.159492
Novel_002462	XLOC_02718.	transcript	0.161502	0.064238	0.184742	0.221087	0.153526	0.184658	0.149705	0.132691	0.181362
Novel_002463	XLOC_02718.	transcript	0	0	0	0	0	0	0.283686	0	0
Novel_002464	XLOC_02718.	transcript	0	0	0	0	0	0	0	0	0
Novel_002465	XLOC_02718.	transcript	0	0	0	0	0	0	0	0	0
Novel_002466	XLOC_02718.	transcript	0	0	0	0	2.339916	0	0	4.705175	0
Novel_002467	XLOC_02718.	transcript	1.09781	0.725175	0.970808	4.409799	1.259735	0.096608	3.358455	0	0.699455
Novel_002468	XLOC_02718.	transcript	0	1.421214	1.817909	0.205395	0.013714	0.358774	0	0	1.987398
Novel_002469	XLOC_02718.	transcript	0	0	0	0	0	1.585937	1.39432	0	0
Novel_002470	XLOC_02718.	transcript	0.163956	0.06897	0.157398	0.255316	0.188636	0.206814	0.223809	0.15813	0.000361
Novel_002471	XLOC_02718.	transcript	0.296601	0	0.164626	0.277959	0.223899	0.212694	0.33751	0.199508	0.239519
Novel_002472	XLOC_02718.	transcript	0	0	0.314788	0	0.270171	0	0.342567	0	0.361487
Novel_002473	XLOC_02718.	transcript	0.189137	0.047881	0.168539	0.19914	0.242106	0.177888	0.221344	0.153188	0.146163
Novel_002474	XLOC_02718.	transcript	0.029675	0.045664	0.029691	0.150415	0.032364	0.063837	0.038576	0.002665	0.054257
Novel_002475	XLOC_02718.	transcript	1.113761	0.692947	0.952401	0.652571	1.100603	0.777185	1.504363	1.505619	0.80143
Novel_002476	XLOC_02720.	transcript	1.642487	1.989546	1.276822	1.993264	1.480041	1.46306	1.17199	1.825259	1.392276
Novel_002477	XLOC_02720.	transcript	0.175043	0.070066	0.297729	0.335718	0.21829	0.150459	0.047245	0.000471	0.149753
Novel_002478	XLOC_02720.	transcript	0	0	0	0.031225	0.109083	0	0.362857	0.435806	0.024361
Novel_002479	XLOC_02720.	transcript	0.397994	0	0.075855	0	0.236466	0.398504	0.075103	0.17395	0.112183
Novel_002480	XLOC_02720.	transcript	0.034022	0.492364	0.350375	0.57935	0	0.065405	0.371918	0.436911	0.414592
Novel_002481	XLOC_02722.	transcript	0.483777	0.469545	0.417074	0.544302	0.374294	0.397236	0.442895	0.605877	0.590032
Novel_002482	XLOC_02722.	transcript	0	0.135239	0.380831	0.189263	0	0.266465	0.04569	0.129868	0
Novel_002483	XLOC_02722.	transcript	0.194595	0	0	0	0.255443	0	0.458569	0	0.068307
Novel_002484	XLOC_02722.	transcript	0.205129	0.566688	0	0.24816	0.210801	0	0	0.410232	0.394532
Novel_002485	XLOC_02723.	transcript	0	0.203673	0	0.002746	0	0.025417	0	0	0.446368
Novel_002486	XLOC_02723.	transcript	0.398334	0	0	0	0	0	0	0	0
Novel_002487	XLOC_02723.	transcript	0.001001	0.003858	0	0.002805	0.004621	0	0.027613	0.000349	0.040352
Novel_002488	XLOC_02723.	transcript	0.003637	1.80E-05	0	2.00E-05	0	0	0	0.200674	0
Novel_002489	XLOC_02723.	transcript	0.217719	0.757101	0.244194	0.380844	0.340841	0.22454	0.788495	0.237837	0
Novel_002490	XLOC_02723.	transcript	0.028047	0.185102	0.244537	0.01819	0.106723	0.22323	0.060092	0.006684	0.103097
Novel_002491	XLOC_02723.	transcript	0.00221	1.488383	0.4319	0	0.324353	0	0.322204	0.003379	0
Novel_002492	XLOC_02723.	transcript	4.342892	5.97868	4.335419	5.641334	4.108773	5.356286	5.122158	7.184337	4.833436
Novel_002493	XLOC_02723.	transcript	0.468477	0.30418	0.385549	0.50275	0.857641	0.253321	0.706688	0.895319	0.487976
Novel_002494	XLOC_02723.	transcript	0	0	0.233969	0.146325	0	0.243567	0.144314	0	0
Novel_002495	XLOC_02723.	transcript	0.216892	0.387227	0.193728	0.15206	0.400155	0.497098	0.607574	0.225283	0.51998
Novel_002496	XLOC_02723.	transcript	0.137842	0.082718	0.275016	0.144227	0.148774	0.248521	0.269598	0.316356	0.459224

Novel_002497	XLOC_02725.	transcript	1.237585	1.413754	1.190023	1.638382	1.276577	1.505682	1.543695	1.397278	1.322586
Novel_002498	XLOC_02727.	transcript	0.089259	0.103201	0.109443	0.30318	0.21133	0.213968	0.254446	0.187732	0.110023
Novel_002499	XLOC_02727.	transcript	0	0.068369	0.023293	0	0.14399	0.194052	0.049959	0.059246	0
Novel_002500	XLOC_02727.	transcript	0.367284	0.403538	0.429714	0.313967	0.428179	0.058324	0.174857	0.006836	0.018653
Novel_002501	XLOC_02727.	transcript	0	0.650832	0	0	0.35658	0.557241	0.452896	0.65804	0.589215
Novel_002502	XLOC_02727.	transcript	0	0.019883	0	0.000486	0.002488	0.002304	0.010307	0.044021	0
Novel_002503	XLOC_02728.	transcript	1.19161	0.062703	1.285575	0	0.639917	0.271853	1.30646	0.635743	1.863272
Novel_002504	XLOC_02728.	transcript	0.399256	0	0.264962	1.031719	0	0	0	0	0
Novel_002505	XLOC_02728.	transcript	0	0	0	0	0.955338	0.989488	0.481627	0.893807	0
Novel_002506	XLOC_02729.	transcript	0.321364	0.017764	0.53863	0.473945	0.54429	0.426612	0.424692	1.267476	3.433036
Novel_002507	XLOC_02730.	transcript	1.563903	1.965616	2.930025	2.786951	2.729939	2.383663	2.088839	3.32797	1.801631
Novel_002508	XLOC_02731.	transcript	0	0	0	0.205762	0.080325	0.261436	0.016263	0.214176	0.070072
Novel_002509	XLOC_02731.	transcript	0.300037	0.113071	0.376905	0.334951	0.244367	0.393117	0.018668	0.402185	0.346815
Novel_002510	XLOC_02731.	transcript	0.00557	0	0.013684	0.009876	0	0.012909	0.551775	0.008488	0.042806
Novel_002511	XLOC_02731.	transcript	0	0.038953	0.008435	0	0	0	0	0	0
Novel_002512	XLOC_02731.	transcript	0.178274	0.039448	0.219851	0.162392	0.603256	0.311869	0.076076	0.065945	0.416822
Novel_002513	XLOC_02732.	transcript	0.170363	0.030242	0.179171	0.163638	0.177728	0.141896	0.333618	0.254335	0.183978
Novel_002514	XLOC_02732.	transcript	0	0.141061	0	0	0	0	0	0	0
Novel_002515	XLOC_02732.	transcript	0	0.214611	0.051765	0.028442	0.022086	0.063013	0.038948	0.031254	0.011679
Novel_002516	XLOC_02735.	transcript	0.00598	0.595845	0	0	0	0	0.016783	0	0
Novel_002517	XLOC_02744.	transcript	0	0	0.09595	0.132787	0.065939	0.106766	0.106762	0	0.01947
Novel_002518	XLOC_02745.	transcript	0	0.028533	0.100069	0.056396	0.152671	0	0.050325	0	0.159492
Novel_002519	XLOC_02747.	transcript	0	0	0	0	0	0	0.003013	0.009944	0
Novel_002520	XLOC_02747.	transcript	0	0	0.098558	0	0	0.140747	0	0.238981	0
Novel_002521	XLOC_02747.	transcript	0.212857	0.18226	0.457973	0.695194	0.584199	0.396245	0.907412	0.531142	0.485932
Novel_002522	XLOC_02747.	transcript	0	0	0.135816	0	0	0	0	0	0
Novel_002523	XLOC_02747.	transcript	0.333581	0.043886	0.188891	0	0.033268	0.051362	0.031245	0.033206	0.168224
Novel_002524	XLOC_02748.	transcript	0	0	0	0	0	0	0	0.216333	0
Novel_002525	XLOC_02748.	transcript	0.323108	0.372234	0.439397	0.407601	0.474177	0.284635	0.546217	0.369444	0.359924
Novel_002526	XLOC_02748.	transcript	0	0	0	0	0	0.01387	0	0	0
Novel_002527	XLOC_02748.	transcript	0.073963	0.075104	0.001326	0.014979	0.024359	0	0.046557	0.014658	0.041373
Novel_002528	XLOC_02753.	transcript	0.218301	0.222106	0.418197	0.330247	0.231679	0.218375	0.29736	0.243695	0.134541
Novel_002529	XLOC_02753.	transcript	0	0.00695	0.114256	0.168939	0	0.152283	0.137125	0	0
Novel_002530	XLOC_02753.	transcript	0	0	0	0.081611	0.057986	0	0	0	0
Novel_002531	XLOC_02753.	transcript	0	0.279304	0	0.031605	0	0	0.179606	0.215256	0
Novel_002532	XLOC_02753.	transcript	0.082629	1.379782	0.090618	0.088834	0.110376	0.341692	0.561798	0.078114	0.660558
Novel_002533	XLOC_02753.	transcript	0	0.480418	0	0	0	0.017817	0.09267	0	0
Novel_002534	XLOC_02753.	transcript	0.618542	0.210693	0.441185	0.713784	0.013973	1.000209	0.220732	0.566468	1.004523

Novel_002535	XLOC_02753.	transcript	0.294421	0.440142	0.282391	0.11254	0.344792	0	0	0.012231	0.027176
Novel_002536	XLOC_02753.	transcript	0	0	0.137363	0	0.028846	0	0	0	0.033424
Novel_002537	XLOC_02753.	transcript	0	0.084546	0	0	0	0.074413	0	0	0
Novel_002538	XLOC_02753.	transcript	1.232762	1.189021	1.557545	1.264754	1.397342	1.164968	1.503636	1.471539	1.30606
Novel_002539	XLOC_02754.	transcript	0.126077	0.325445	0.170832	0.171	0.223004	0.15988	0.12737	0.147498	0.010234
Novel_002540	XLOC_02754.	transcript	0	0	0.049766	0.109313	0.116213	0.055097	0.332468	0.111523	0.038474
Novel_002541	XLOC_02754.	transcript	0.320311	0.11334	0.222526	0.543791	0.555673	0.421878	0.783825	0.30281	0.238921
Novel_002542	XLOC_02755.	transcript	0.390021	0.242358	0.446228	0.370389	0.471342	0.352372	0.373365	0.224833	0.249714
Novel_002543	XLOC_02756.	transcript	0.179778	0	0.172148	0.14666	0.073957	0.043658	0.054125	0.05169	0.064325
Novel_002544	XLOC_02756.	transcript	0.67494	0.403302	0.422236	0.269556	0.452465	0.498942	0.490131	0.445932	0.336668
Novel_002545	XLOC_02756.	transcript	0.079971	0.06628	0.084547	0	0.286277	0	0.131156	0.091575	0.047388
Novel_002546	XLOC_02759.	transcript	0.024967	0.025549	0	0.078282	0.021359	0.014498	0.180847	0.02443	0.034188
Novel_002547	XLOC_02759.	transcript	0	0	0	0	0	0	0.104054	7.50E-05	0.017236
Novel_002548	XLOC_02759.	transcript	0.094375	0.073927	0.14684	0.087531	0.095898	0.070334	0.118881	0.183862	0.153848
Novel_002549	XLOC_02766.	transcript	32.26992	0.345393	8.990017	1.018556	0	2.562595	10.40233	1.886167	5.124248
Novel_002550	XLOC_02766.	transcript	0	0	0	0	8.533452	0	0	0	0
Novel_002551	XLOC_02768.	transcript	0	0	0	0	0	0	0	0	0
Novel_002552	XLOC_02769.	transcript	2.028758	1.504533	1.809847	2.176471	1.856313	1.732224	2.158098	2.079947	2.458667
Novel_002553	XLOC_02770.	transcript	0.12171	0	0.121822	0.186615	0.124483	0.220271	0.228528	0.296354	0.433607
Novel_002554	XLOC_02772.	transcript	0.25681	0.096148	0.201441	0.157792	0.326049	0.026466	0.279031	0.149704	0.184805
Novel_002555	XLOC_02772.	transcript	0	0.121453	0.008318	0	0	0.113196	0	0.130121	0
Novel_002556	XLOC_02773.	transcript	0.132186	0.005766	0.242	0.0304	0.14319	0.063141	0.097269	0.083154	0.111999
Novel_002557	XLOC_02783.	transcript	2.10292	3.10717	3.787467	3.683186	2.899466	3.593735	3.305146	3.861984	2.684445
Novel_002558	XLOC_02783.	transcript	0	0	1.91273	4.379173	0	0	2.954238	0	0
Novel_002559	XLOC_02783.	transcript	1.233984	4.702826	2.769621	0.434736	2.320014	1.03591	0.884997	5.270352	1.254839
Novel_002560	XLOC_02783.	transcript	3.206293	2.275873	1.231879	2.52387	4.121184	4.438171	3.774446	2.98802	4.561481
Novel_002561	XLOC_02787.	transcript	0	0	0.157108	0.058001	0.028087	0.02592	0.061245	0.011284	0.114452
Novel_002562	XLOC_02787.	transcript	0.29866	0.044395	0.245642	0.109636	0.236282	0.346317	0.201921	0.181448	0.057804
Novel_002563	XLOC_02792.	transcript	0.796592	0.705024	0.828619	0.922182	0.821082	0.95537	1.072307	0.922331	0.741893
Novel_002564	XLOC_02796.	transcript	0.328168	0.182866	0.485125	0.146977	0.4133	0.418528	0.258399	0.268498	0.06913
Novel_002565	XLOC_02796.	transcript	0	0.109432	0.029982	0	0.012529	0.022303	0	0	0
Novel_002566	XLOC_02796.	transcript	0.013896	0	0	0	0	0	0	0	0.076592
Novel_002567	XLOC_02796.	transcript	0	0	0	0.438289	0	0	0.585468	0.482936	0.604238
Novel_002568	XLOC_02797.	transcript	0.096833	0	0.09321	0	0	0.043305	0.270225	0.151111	0.042537
Novel_002569	XLOC_02807.	transcript	0.076003	0	0.241058	0	0.242894	0.046088	0.369764	0.043515	0.021549
Novel_002570	XLOC_02807.	transcript	0.399635	0.140458	0	0.273196	0.084701	0.234555	0.287879	0.255923	0.176035
Novel_002571	XLOC_02807.	transcript	0.220155	0.074988	0	0.105893	0.157355	0	0.079355	0.163567	0.005192
Novel_002572	XLOC_02808.	transcript	0.128327	0.432197	0.200077	0.282899	0.241506	0.253378	0.229198	0.192786	0.236736

Novel_002573	XLOC_02811.	transcript	4.188939	0.024019	3.183968	3.323153	2.777642	4.228736	1.701461	0.917898	4.14454
Novel_002574	XLOC_02811.	transcript	0.157733	0	0.410397	0.113624	0.039754	0.132275	0.949503	0.039259	0
Novel_002575	XLOC_02811.	transcript	0	0	0	0	0	0.447524	0	0	0
Novel_002576	XLOC_02811.	transcript	0.28035	0.126	0.311102	0.426363	1.010264	0	2.620654	4.369516	0.449209
Novel_002577	XLOC_02811.	transcript	0.008017	0.629308	0.828729	2.944361	1.069141	0.070125	0.619833	0.46668	3.055372
Novel_002578	XLOC_02824.	transcript	0.539915	5.480543	0.842313	0.563759	0.551297	0.547701	1.112184	0.783943	2.712385
Novel_002579	XLOC_02825.	transcript	0	0.352778	0	0	0	0	0	0	0
Novel_002580	XLOC_02825.	transcript	0.035538	0.532002	0.025559	0.036534	0.03391	0.065095	0.140375	0.020774	0.012417
Novel_002581	XLOC_02830.	transcript	0.547742	2.80E-05	0	0.292616	0.256195	0.014822	0	0	0.27332
Novel_002582	XLOC_02830.	transcript	0	0.44409	0	0	0	0	0	0	0
Novel_002583	XLOC_02830.	transcript	0	0	0.336695	0	0	0.275997	0.312809	0.32518	0
Novel_002584	XLOC_02832.	transcript	0.179941	0.374254	0.072481	0.051563	0.107239	0.03424	0.214146	0.023236	0.092125
Novel_002585	XLOC_02832.	transcript	0	0.019761	0.019696	0	0.093794	0.045032	0.067738	0	0
Novel_002586	XLOC_02837.	transcript	0	0	0	0.193674	0.025308	0	0	0	0
Novel_002587	XLOC_02837.	transcript	0.173777	0.176951	0.134	0	0.074639	0	0.188998	0.226586	0.068153
Novel_002588	XLOC_02837.	transcript	0.198244	0.209698	0.227626	0.076913	0.193716	0.261115	0.284231	0.125687	0.148061
Novel_002589	XLOC_02837.	transcript	0	0	0	0	0	0	0	0	0
Novel_002590	XLOC_02837.	transcript	0.014424	0.077042	0.360839	0.008421	0.005293	0.002331	0.18064	0.000957	0.018906
Novel_002591	XLOC_02837.	transcript	0.282925	0.186234	0.175141	0.385714	0.3307	0.386997	0.419828	0.43505	0.274008
Novel_002592	XLOC_02840.	transcript	0.360103	0.513895	0.297431	0.303884	0.374892	0.375164	0.696196	0.400587	0.311186
Novel_002593	XLOC_02840.	transcript	0.429988	0.460383	0	0.023926	0	0.063195	0.020655	0.224562	0.005754
Novel_002594	XLOC_02840.	transcript	0	0	0.32667	0.010716	0.087974	0.036404	0.487139	0	0
Novel_002595	XLOC_02840.	transcript	0	0	0.005275	0.480691	0.397755	0.329192	0.157441	0.246874	0.353622
Novel_002596	XLOC_02840.	transcript	0.246398	2.00E-05	0.505084	0.166361	0.331169	0.363643	0.003253	0	0.256407
Novel_002597	XLOC_02840.	transcript	0	0	0	0.056098	0.008744	0	0.605933	0.040439	0
Novel_002598	XLOC_02840.	transcript	0	0	0	0	0.014296	0	0.445232	0	0
Novel_002599	XLOC_02840.	transcript	0.255239	0.220588	0	0.245445	0	0.021546	0	0	0.014277
Novel_002600	XLOC_02840.	transcript	0	0.024882	0.280775	0	0.334703	0.16442	0.070753	0.308403	0.163069
Novel_002601	XLOC_02841.	transcript	0.071464	0.072501	0.137303	0.048987	0.2101	0.045964	0.114603	0.063399	0.297332
Novel_002602	XLOC_02841.	transcript	0.736559	0.750394	0.676229	0.880965	1.044871	1.164978	1.059379	0.753944	1.072415
Novel_002603	XLOC_02841.	transcript	0	0	0	0.065454	0	0	0.196442	0	0.017744
Novel_002604	XLOC_02841.	transcript	0	0.002933	0.003488	0.002326	0.029716	0.00325	0.012137	0.054756	0.261781
Novel_002605	XLOC_02841.	transcript	0	0	0	0	0.150737	0.068949	0.279631	0	0.051965
Novel_002606	XLOC_02841.	transcript	0.372128	0.33573	0.444929	0.069309	0	0.637347	0.192813	0.588924	0.202562
Novel_002607	XLOC_02841.	transcript	0.061036	0	0	0	0.080939	0.104273	0.132422	0	0
Novel_002608	XLOC_02841.	transcript	0	0	0	0	1.458878	0	0	0	0.748462
Novel_002609	XLOC_02841.	transcript	0	0	0	0.971746	0	0	0	0	0
Novel_002610	XLOC_02841.	transcript	0.4822	0.360103	0.693094	0	0	0	0.513861	0.777039	0

Novel_002611	XLOC_02841.	transcript	0.616148	0.56796	0.069707	0.441578	0.019686	0.309137	0.058799	0.384923	0.288878
Novel_002612	XLOC_02841.	transcript	0.753814	0	0	0.001622	0.586363	0.763608	0	0.28037	0
Novel_002613	XLOC_02841.	transcript	0.127175	0.096598	0.064499	0	0.796622	0.101424	0.099879	0.371223	0.099875
Novel_002614	XLOC_02841.	transcript	0	1.074265	0.058093	0.975649	0	0.021032	0.445779	0.436393	0.801693
Novel_002615	XLOC_0284[illegible].	transcript	0.284661	0.62481	0.863006	1.229423	0.746041	0.515706	0.542673	0.828583	0.668655
Novel_002616	XLOC_02851.	transcript	0.042377	0.157678	0	0	0.414187	0	0.385299	0.55479	0
Novel_002617	XLOC_02851.	transcript	0.867064	1.81975	1.588178	1.392707	0.760946	1.336698	0.947412	0.776393	0.98436
Novel_002618	XLOC_02852.	transcript	0.011631	0	0.509502	0.088623	0.032247	0.57569	0	0.189374	0.129328
Novel_002619	XLOC_02852.	transcript	0.18992	1.542695	0.346039	0.639601	0.040157	0.000584	0.035496	0.005834	0.002964
Novel_002620	XLOC_02852.	transcript	0	0	0	0	0	0.004284	0	0	0
Novel_002621	XLOC_02852.	transcript	0	5.50E-05	0.169165	0.005908	0	0.833346	0	0.444841	0.936289
Novel_002622	XLOC_02852.	transcript	0	0	0	0	0	0	0	0	0
Novel_002623	XLOC_02852.	transcript	0.938003	0.707442	0.015178	0.328468	0	0	1.335552	0.359856	0.001332
Novel_002624	XLOC_02852.	transcript	0	0	0	0	0.898191	0	0.111716	0	0
Novel_002625	XLOC_02852.	transcript	0.724881	0	0.150815	0	0.018523	0	0.645396	0.694431	0.020975
Novel_002626	XLOC_02852.	transcript	0.139642	0.053344	0.589231	0.799092	0.539805	0.624407	0.520915	0.517041	0.468464
Novel_002627	XLOC_02852.	transcript	0	0	0.289117	0.158476	0.008153	0	0	0.081709	0
Novel_002628	XLOC_0285[illegible].	transcript	0.561207	0.81272	0.945758	0.977207	0.881942	0.703964	0.556802	0.826405	0.877059
Novel_002629	XLOC_0285[illegible].	transcript	0.163636	0	0.248468	0.330026	0.478697	0.362661	0.369222	0.546131	0.129727
Novel_002630	XLOC_0285[illegible].	transcript	0.27096	0.812476	0.87878	0.791388	0.62755	1.112398	0.388407	0.878661	0.861903
Novel_002631	XLOC_0285[illegible].	transcript	0	0	0	0	0	0	0	0	0
Novel_002632	XLOC_0285[illegible].	transcript	0.125584	0.954735	1.232928	1.067594	0.846105	0.978066	0.180446	1.054995	0.845739
Novel_002633	XLOC_0285[illegible].	transcript	0	0.995177	0.233316	0.004984	0.410916	0.062613	0.02713	0.647955	0.007048
Novel_002634	XLOC_0285[illegible].	transcript	1.902018	0.728602	0.433908	1.092141	0.342698	1.333169	2.039019	0.94087	0.780106
Novel_002635	XLOC_02857.	transcript	0.389329	0.336977	0.337002	0.343819	0.373369	0.542484	0.429388	0.50769	0.321984
Novel_002636	XLOC_02857.	transcript	0.221731	0.672717	0.06498	0.127788	0.109247	0.106467	0.251325	0.150128	0.070472
Novel_002637	XLOC_02857.	transcript	0.014155	0.208828	0.146312	0.133529	0.152994	0.152908	0.087151	0.099661	0.198012
Novel_002638	XLOC_02857.	transcript	0	0	0	0	0	0	0	0	0
Novel_002639	XLOC_02857.	transcript	0.246609	0.977018	0.494816	0.526482	0.403164	0.501617	0.569324	0.430979	0.576692
Novel_002640	XLOC_02857.	transcript	0	0.859852	0.011041	0	0.020286	0	0	0	0
Novel_002641	XLOC_02857.	transcript	0	0.004399	0	0	0	0	0	0	0.007885
Novel_002642	XLOC_02857.	transcript	0.101399	0	0.207479	0.022895	0.121877	0.075572	0.139651	0.055839	0
Novel_002643	XLOC_02857.	transcript	0	0	0	0.004095	0	0	0	0.002446	0
Novel_002644	XLOC_02857.	transcript	0	0	0	0.684783	0	0.171908	0	0	0.021983
Novel_002645	XLOC_02857.	transcript	0.158603	0.146185	0.153359	0.051195	0.425224	0.058643	0.200703	0.129972	0.292608
Novel_002646	XLOC_02857.	transcript	0	0	0	0	0	0.299908	0.024632	0.504434	0
Novel_002647	XLOC_0285[illegible].	transcript	1.011467	0.845609	0.919677	0.952849	1.022173	1.023742	1.372751	1.122885	1.300664
Novel_002648	XLOC_0286[illegible].	transcript	0.580692	0.23428	0.727128	0.831715	1.020037	0.820493	0.687067	1.480293	0.773839

Novel_002649	XLOC_02862.	transcript	0.661886	0.638022	0.345944	0.186651	0.376257	0.318462	0.480785	0.365528	0.361996
Novel_002650	XLOC_02863.	transcript	0.405465	0	0.939521	2.009212	0.828951	1.133409	0.508357	1.798983	0.465341
Novel_002651	XLOC_02863.	transcript	0	0.14284	0.012877	0.088508	0	0.005682	0.238903	0.173032	0.310831
Novel_002652	XLOC_02863.	transcript	0.324699	0.742262	0.411816	0.10228	0.231064	0.472663	0.526966	0.367222	0.202869
Novel_002653	XLOC_02863.	transcript	0.075608	0.154067	0.090494	0.101379	0.178364	0.00163	0.013997	0.0995	0.192909
Novel_002654	XLOC_02863.	transcript	0.034124	0.001146	0.156004	0.12072	0.1991	0.129704	0.202604	0.266364	0.229803
Novel_002655	XLOC_02863.	transcript	22.53421	35.68642	42.10429	39.47515	41.41507	41.3275	35.41844	53.51671	70.8804
Novel_002656	XLOC_02868.	transcript	0.524761	0.748161	0.575621	0.633952	0.565186	0.499035	0.837693	0.520209	0.40357
Novel_002657	XLOC_02868.	transcript	0	0	0	0	0	0	0.030694	0	0
Novel_002658	XLOC_02868.	transcript	0.450458	0.194249	0.355943	0.233601	0.354459	0.40381	0.51737	0.380927	0.277657
Novel_002659	XLOC_02868.	transcript	0.171717	0.153271	0.183005	0.268961	0.299483	0.343528	0.317329	0.300876	0.236642
Novel_002660	XLOC_02868.	transcript	0	0	0	0.004254	0.006868	0.00032	0.053109	0	0.005854
Novel_002661	XLOC_02868.	transcript	0.118589	0.102204	0.119953	0.289309	0.237997	0.093016	0.252808	0.1918	0.194704
Novel_002662	XLOC_02869.	transcript	0.751236	0.121032	0.963759	1.634099	1.24048	1.04327	1.548376	1.307315	1.253468
Novel_002663	XLOC_02870.	transcript	0.034452	0.322539	0.127645	0	0.446968	0.422694	0.564642	0.271765	0
Novel_002664	XLOC_02870.	transcript	0.661015	0	0.217315	0	0	0	0	0.246692	0
Novel_002665	XLOC_02870.	transcript	0	0.489988	0	0.667757	0.275519	0.400423	0.579109	0.3561	0.704968
Novel_002666	XLOC_02870.	transcript	0	0	0	0	0	0	0	0.000576	0
Novel_002667	XLOC_02870.	transcript	0.204464	0	0.127354	0.108856	0.289271	0	0.252393	0.1523	0.142621
Novel_002668	XLOC_02872.	transcript	4.768648	5.535874	4.4188	4.888244	4.235897	3.806664	4.828834	5.969966	3.543058
Novel_002669	XLOC_02873.	transcript	0.20237	0.823627	0.364242	0.065188	0.220878	0.391478	0.101825	0.275515	0.173086
Novel_002670	XLOC_02873.	transcript	0	0.078174	0.078704	0.290505	0	0	0	0.235379	0
Novel_002671	XLOC_02874.	transcript	1.138214	1.037415	1.092339	1.481937	0.857802	1.320672	1.052261	1.417901	1.116195
Novel_002672	XLOC_02874.	transcript	3.041636	3.800665	4.818892	4.615997	4.20498	5.430722	2.931537	4.2852	4.630734
Novel_002673	XLOC_02878.	transcript	1.064768	1.775037	0.548205	1.426198	1.258536	1.043496	0.696892	0.588939	2.831439
Novel_002674	XLOC_02879.	transcript	0.272809	0.436573	0.311743	0.290622	0.192324	0.342701	0.348756	0.384731	0.270181
Novel_002675	XLOC_02879.	transcript	0.21623	0.053772	0	0.096314	0.088154	0.530556	0.194423	0.064539	0.123998
Novel_002676	XLOC_02879.	transcript	0.03991	0.025798	0	0	0	0.00541	0	0	0.044949
Novel_002677	XLOC_02879.	transcript	0	0	0	0.112197	0.219786	0	0.085838	0.067832	0
Novel_002678	XLOC_02879.	transcript	0	0	0.204641	0	0	0	0.213057	0.204318	0
Novel_002679	XLOC_02881.	transcript	0	0.002753	0	0	0	0	0	0	0
Novel_002680	XLOC_02881.	transcript	0.006052	0	0	0.375482	0.404395	0	0	0.07765	0
Novel_002681	XLOC_02881.	transcript	0	0	0	0	0	0	0	0	0
Novel_002682	XLOC_02881.	transcript	0	0.461823	0.309025	0.307542	0.468632	0.656859	0.67192	0.798375	0.770628
Novel_002683	XLOC_02881.	transcript	0	0	0.136002	0	0.180533	0	0	0	0
Novel_002684	XLOC_02881.	transcript	0.403875	0	0.156679	0	0	0	0.352967	0	0
Novel_002685	XLOC_02881.	transcript	0.530147	0.152731	0.08881	0.258941	0.105692	0.177612	0.186615	0.250161	0.31779
Novel_002686	XLOC_02881.	transcript	0.025599	0	0.095211	0.200131	0.162774	0.120285	0.072276	0.129099	0.008013

Novel_002687	XLOC_02883	transcript	0.220949	0	0	0.035801	0	0	0.284498	0.088025	0.130926
Novel_002688	XLOC_02885	transcript	0.464826	1.623492	0.983951	1.142331	0.948197	1.475136	0.915095	1.208822	1.448676
Novel_002689	XLOC_02888	transcript	0	0	0.000109	0	0	0	0.111681	0.083791	0
Novel_002690	XLOC_02888	transcript	0.314111	0	0.105835	0.092866	0.139707	0.066739	0.293251	0.145621	0.101655
Novel_002691	XLOC_02893	transcript	0	0	0	0	0	0	0	0	0
Novel_002692	XLOC_02893	transcript	2.116523	0.858913	1.341624	1.171362	1.462307	1.115439	2.028157	1.27807	1.151121
Novel_002693	XLOC_02900	transcript	0.459839	0	0	0	0.087402	3.30E-05	0	0	0
Novel_002694	XLOC_02900	transcript	0.467827	0.778523	0.472903	0.471675	0.435132	0.613331	0.913059	0.667404	0.448206
Novel_002695	XLOC_02900	transcript	0	0.404561	0.332792	0	0	0	0	0.109603	0
Novel_002696	XLOC_02901	transcript	0	0	0.149023	0.195983	0	0	0.010068	0	0.002411
Novel_002697	XLOC_02901	transcript	1.300875	0.575605	1.358319	1.066387	1.240721	0.720655	0.635597	1.034282	0.838335
Novel_002698	XLOC_02901	transcript	0	0	0	0.103223	0	0	0	0	0.140593
Novel_002699	XLOC_02901	transcript	0	0	0	0.208955	0	0	0.603994	0	0
Novel_002700	XLOC_02902	transcript	0	0	0	0	0	0.168297	0.164037	0	0
Novel_002701	XLOC_02902	transcript	0.17704	0	0.186362	0.199513	0.008226	0.170655	0.168955	0.175114	0
Novel_002702	XLOC_02902	transcript	0.123914	0.251639	0.19199	0.007312	0.040936	0.042103	0.150489	0.134041	0.198776
Novel_002703	XLOC_02902	transcript	0.169019	0	0.18816	0.225051	0	0.187242	0.233076	0.196505	0
Novel_002704	XLOC_02902	transcript	0.020949	0.02796	0	0.001977	0.332308	0.013092	0.279445	0.005248	0.025188
Novel_002705	XLOC_02902	transcript	0.804442	0.795138	0.348738	0.3405	0.2134	0.56815	0.150257	0.368121	0.400927
Novel_002706	XLOC_02902	transcript	0.378363	0.187152	0.136122	0.176436	0.176053	0.228005	0.233486	0.178435	0.308497
Novel_002707	XLOC_02902	transcript	0.167224	0.174203	0.123429	0.140612	0.25878	0.179972	0.176459	0.171404	0.190411
Novel_002708	XLOC_02903	transcript	0.534276	0.377533	0.690818	0.784167	0.637328	1.190357	0.502976	0.872432	0.747148
Novel_002709	XLOC_02904	transcript	0.209437	0.065821	0.23221	0.185067	0.240656	0.204132	0.064564	0.077095	0.301458
Novel_002710	XLOC_02904	transcript	0.415619	0.051734	0.50242	0.615653	0.331829	0.518832	0.515492	0.562043	0.316811
Novel_002711	XLOC_02905	transcript	0	0.256925	0	0	0.006257	0.256503	0.141672	0.029682	0.202841
Novel_002712	XLOC_02905	transcript	0.94479	0.223447	0.68118	0.676889	0.597756	0.625623	0.698097	0.691865	0.481357
Novel_002713	XLOC_02906	transcript	0.693325	1.196605	0.400779	0.53866	0.592641	0.206488	1.029413	0.739447	1.582662
Novel_002714	XLOC_02907	transcript	0	0	0.060892	0	0.089025	0.388481	0	0.00203	0
Novel_002715	XLOC_02907	transcript	0	0	0	0.288787	0	0.002196	0	0.001632	0
Novel_002716	XLOC_02907	transcript	0.738435	1.062211	0.716674	0.702791	0.396883	0.407163	0.730648	1.153105	0.792802
Novel_002717	XLOC_02912	transcript	0.179391	0.044186	0.087524	0.089115	0.097689	0.112666	0.147326	0.034589	0.061743
Novel_002718	XLOC_02924	transcript	0.038462	0.079692	0.046691	0.011296	0.047635	0.042313	0.143448	0.013799	0.045467
Novel_002719	XLOC_02924	transcript	0.342082	0.111527	0.475242	0.341861	0.341681	0.732873	0.529228	0.315607	0.377354
Novel_002720	XLOC_02924	transcript	0	0	0	0	0	0	0	0.19948	0
Novel_002721	XLOC_02942	transcript	0.177419	0.175043	0.299673	0.421556	0.210785	0.394236	0.450657	0.208929	0.136528
Novel_002722	XLOC_02950	transcript	0.03404	0.026843	0.025135	0.102984	0.209746	0	0.27574	0.183666	0.140052
Novel_002723	XLOC_02950	transcript	0.157145	0	0.087644	0.105052	0.019808	0.078742	0.149716	0.077262	0.03025
Novel_002724	XLOC_02950	transcript	0	0	0.090157	0	0	0	0	0	0.003147

Novel_002725	XLOC_02950.	transcript	0	0	0	0	0	0	0.064367	0	0
Novel_002726	XLOC_02950.	transcript	0.373134	0.135464	0.173952	0.251076	0.300582	0.307541	0.258011	0.23272	0.242642
Novel_002727	XLOC_02950.	transcript	0.015224	0.015409	0.072993	0.094746	0.245901	0.048375	0.07141	0.072435	0.002226
Novel_002728	XLOC_02953.	transcript	0	0.058044	0.09387	0.487333	0.198863	0.34127	0.377634	0.237645	0.017326
Novel_002729	XLOC_02953.	transcript	0.144068	0	0.120295	0.001023	0.164497	0.201313	0.119998	0	0.287007
Novel_002730	XLOC_02953.	transcript	0	1.427234	0.044964	1.143977	0	0.024568	0	0	0
Novel_002731	XLOC_02953.	transcript	0.278444	0.063512	0.006594	0.015683	0.03309	0	0.343669	0.010285	0.474553
Novel_002732	XLOC_02953.	transcript	0	0	0	0	0	0	0	0.611953	0
Novel_002733	XLOC_02954.	transcript	1.784864	1.826386	1.233271	0.897971	1.497697	1.368392	1.516546	1.863293	0.990234
Novel_002734	XLOC_02957.	transcript	0.814419	0.558444	1.071702	1.578715	1.052115	1.085547	1.116294	1.828493	1.073119
Novel_002735	XLOC_02958.	transcript	0.178719	0	0.002864	1.127381	0.405652	1.096734	0	0	1.081636
Novel_002736	XLOC_02958.	transcript	0.660696	0	0.364815	0.256544	0.461359	0.109188	1.287517	0.912131	0.19882
Novel_002737	XLOC_02958.	transcript	0	0.048112	0.231998	0.021496	0	0.002495	0.007362	0.014713	0
Novel_002738	XLOC_02959.	transcript	0.650509	0.045262	0.481646	0.421419	0.472888	0.461864	0.801811	0.656549	0.316247
Novel_002739	XLOC_02959.	transcript	0	0	0	0	0	0	0	0	0
Novel_002740	XLOC_02959.	transcript	0.356166	0.182151	0.388142	0.777174	0.427552	0.370224	0.655271	0.717398	0.213428
Novel_002741	XLOC_02960.	transcript	0.539024	0.102222	0.307267	0.236989	0.331445	0.351184	0.762986	0.372209	0.30547
Novel_002742	XLOC_02961.	transcript	0.242005	0.065966	0.500359	0.265215	0.239566	0.391399	0.58558	0.39825	0.12858
Novel_002743	XLOC_02962.	transcript	0.101415	0.456373	0.092191	0	0.035688	0.147592	0.231856	0.030016	0.041421
Novel_002744	XLOC_02962.	transcript	0.043448	0	0	0.030949	0.075537	0.103958	0.319396	0	0.029175
Novel_002745	XLOC_02962.	transcript	0.210347	1.5507	0.137151	0.265113	0.172369	0.247288	0.376381	0.247606	0.339069
Novel_002746	XLOC_02963.	transcript	0.002759	0	0.046365	0.017296	0.015212	0.151652	0.210867	0.109685	0.024827
Novel_002747	XLOC_02963.	transcript	0.118677	0.026698	0.038366	0.078989	0.02133	0.063414	0.02925	0.103346	0.052683
Novel_002748	XLOC_02963.	transcript	0	0	0	0.030409	0.03711	0.043777	0.13568	0.114725	0
Novel_002749	XLOC_02964.	transcript	1.102977	0.763724	0.712725	0.494285	0.376235	1.061098	0.776393	0.73695	0.310337
Novel_002750	XLOC_02964.	transcript	0.195734	0.595224	0.537574	0.405276	0.386	0.078367	0.548242	0.292098	0.697896
Novel_002751	XLOC_02966.	transcript	0	0	0	0	0	0	0	0.07399	0
Novel_002752	XLOC_02966.	transcript	0.012718	0.707216	0.118066	0	0.056873	0	0.041657	0	0
Novel_002753	XLOC_02967.	transcript	1.712922	0.845476	1.585792	0.920137	1.117058	2.739061	3.081605	1.962565	4.042319
Novel_002754	XLOC_02968.	transcript	0.015194	0	0.173911	0.162416	0	0	0.238011	0.075108	0
Novel_002755	XLOC_02972.	transcript	0.146235	0.220909	0.004148	0.007315	0.002355	0	0.08278	0.653688	0.002945
Novel_002756	XLOC_02972.	transcript	0.061004	0.251954	0.042924	0.051128	0.062394	0.061254	0.092254	0.058019	0.048198
Novel_002757	XLOC_02986.	transcript	0.174752	0.090888	0.195693	0.102352	0.121013	0.078955	0.128449	0.100694	0.074663
Novel_002758	XLOC_02987.	transcript	0.024061	0	0.038401	0	0	0.013537	0.027166	0	0.010331
Novel_002759	XLOC_02987.	transcript	0	0	0.275093	0	0	0	0	0	0
Novel_002760	XLOC_02987.	transcript	0.007684	1.069531	0	0.770522	0.003593	0.000672	0	0	0
Novel_002761	XLOC_02987.	transcript	0.530718	0	0.94536	0	0.7544	0.853645	0.741665	0.777699	0.920531
Novel_002762	XLOC_02988.	transcript	0.583204	0.056145	0.426873	0.198129	0.350155	0.642317	0.477555	0.310821	0.377591

Novel_002763	XLOC_02992.	transcript	0.846697	0.179103	0.189928	0.195531	0.209564	0.066994	0.266294	0.128089	0.137037
Novel_002764	XLOC_02992.	transcript	0.324447	0.156147	0.533731	0.274873	0.255596	0.323778	0.33915	0.527492	0.33324
Novel_002765	XLOC_02992.	transcript	0.00618	0	0	0.123692	0.08187	0.30859	0.120752	0.187247	0
Novel_002766	XLOC_02992.	transcript	0.552739	0.232309	0.551121	0.380159	0.57087	0.534569	0.677084	0.509008	0.336677
Novel_002767	XLOC_02995.	transcript	0	0	0	0	0	0	0.000618	0	0
Novel_002768	XLOC_02995.	transcript	0.223431	0	0.122868	0	0	0	0.370205	0.079029	0
Novel_002769	XLOC_02995.	transcript	0.877133	0	0.843209	0.718282	0.609363	0.700816	0.732854	0.728581	1.983712
Novel_002770	XLOC_02995.	transcript	0.161922	0	0.044383	0.195617	0.011073	0.006447	0.040926	0.035903	0.023215
Novel_002771	XLOC_02995.	transcript	0.032395	0.100108	0.013792	0	0.053594	0.058462	0.044296	0.062273	0.081881
Novel_002772	XLOC_02999.	transcript	0.042628	0	0	0	0	0	0	0	0
Novel_002773	XLOC_03015.	transcript	0.182239	0.078288	0.320787	0.107714	0	0.419531	0.108048	0.026568	0.207132
Novel_002774	XLOC_03015.	transcript	0.233926	0	0.315102	0.180496	0.493824	0.323126	0.354799	0.207541	0.158876
Novel_002775	XLOC_03015.	transcript	0.157683	0.167081	0.000394	0.122548	0.210033	0.001204	0.284812	0.303143	0.017863
Novel_002776	XLOC_03015.	transcript	0	0	0	0.020738	0.003667	0.058815	0.041962	0.75984	0
Novel_002777	XLOC_03015.	transcript	0.730291	0	0	0.180254	0.14973	0	0.235157	0	0
Novel_002778	XLOC_03015.	transcript	0.216113	0.289174	0.278339	0.521638	0.203263	0.268526	0.417487	0.290443	0.292017
Novel_002779	XLOC_03022.	transcript	0.526564	0.441849	0.467889	0.312891	0.37139	0.395061	0.423294	0.45655	0.3181
Novel_002780	XLOC_03034.	transcript	0	0	0.02309	0	0.022412	0	0.032777	0	0
Novel_002781	XLOC_03039.	transcript	0.151842	0.05395	0.123863	0.102702	0.038471	0.134382	0.117234	0.100585	0.136453
Novel_002782	XLOC_03039.	transcript	0	0	0.03409	0.003577	0.05723	0	0	0.00544	0.006052
Novel_002783	XLOC_03039.	transcript	0.029506	0.015505	0	0.036785	0.143943	0.045967	0.174884	0.010421	0.138459
Novel_002784	XLOC_03039.	transcript	0.358234	0.113211	0.106398	0.354862	0.165914	0.405913	0.865705	0.244449	0.372504
Novel_002785	XLOC_03039.	transcript	0.290536	0.166066	0.140299	0.413192	0.167973	0.565142	0.348247	0.174791	0.461809
Novel_002786	XLOC_03039.	transcript	0.558172	0.248639	0.535493	0.282363	0.398304	0.376821	0.469024	0.383761	0.240387
Novel_002787	XLOC_03045.	transcript	1.801828	1.833534	2.011237	1.676239	2.063567	1.910264	2.6672	2.043044	2.319582
Novel_002788	XLOC_03050.	transcript	0.059947	0.485629	0.033124	0	0	0.089763	0	0.536254	0.029056
Novel_002789	XLOC_03050.	transcript	0.597688	0.07914	0.894102	0.528292	0.728767	0.744548	0.885853	0.319907	0.394613
Novel_002790	XLOC_03054.	transcript	0.169181	0.449978	0.426478	0.629098	0.491129	0.540188	0.274518	0.417865	0.278725
Novel_002791	XLOC_03054.	transcript	0.108136	0.336893	0	0	0	0	0.207977	0.349808	0.114973
Novel_002792	XLOC_03054.	transcript	0.096331	0.320377	0	0	0.154028	0.323193	0	0.007623	0
Novel_002793	XLOC_03054.	transcript	0	0.096082	0	0.444848	0	0	0	0	0
Novel_002794	XLOC_03054.	transcript	0	0	0.48503	0	0	0	0	0	0
Novel_002795	XLOC_03054.	transcript	0.000687	0.120624	0	0	0.36402	0.745437	0	0	0
Novel_002796	XLOC_03054.	transcript	0.915036	0.273614	0.044684	0.544948	0.322195	0.067845	0.531482	0.256533	0.120522
Novel_002797	XLOC_03054.	transcript	0.120759	0.074393	0	0.200815	0	0.417328	0	0.631052	0
Novel_002798	XLOC_03054.	transcript	0.143882	0.137838	0.860515	0.052735	0.256802	0	0.272605	0.049754	0.468212
Novel_002799	XLOC_03057.	transcript	0.322215	0.307847	0.314345	0.356949	0.373038	0.371457	0.411606	0.547109	0.193209
Novel_002800	XLOC_03061.	transcript	0.003771	0	0	0.000673	0	0	0	0	0

Novel_002801	XLOC_03061.	transcript	0.332199	0.026166	0.2007	0.073104	0.135209	0.104662	0.017547	0.099973	0.176706
Novel_002802	XLOC_03064.	transcript	0.163254	0.240066	0.161682	0.186775	0.242758	0.267901	0.236941	0.147364	0.134755
Novel_002803	XLOC_03066.	transcript	0	0	0	0	0	0.000266	0.054495	0.052538	3.00E-06
Novel_002804	XLOC_03066.	transcript	0	0	0.059135	0	0.000462	0	0	0.040599	0
Novel_002805	XLOC_03066.	transcript	0.063843	0.016705	0	0.025411	0.024688	0.022909	0.006918	0.04144	0.006445
Novel_002806	XLOC_03066.	transcript	1.047862	1.407399	1.334097	1.580403	0.941584	1.231583	0.851364	1.712644	1.244286
Novel_002807	XLOC_03067.	transcript	0.182632	0.049682	0.107612	0.138485	0.186423	0.102078	0.238612	0.250809	0.087147
Novel_002808	XLOC_03069.	transcript	0.240686	0.15785	0.171129	0.072599	0.018686	0.194938	0.026369	0.004878	0.201429
Novel_002809	XLOC_03069.	transcript	0.029696	0.043541	0.022746	0.289371	0.246254	0.035357	0.248932	0.268807	0.062577
Novel_002810	XLOC_03076.	transcript	0.267804	0.228047	0.287053	1.120128	0.440232	0.643957	0.388009	0.233498	0.207444
Novel_002811	XLOC_03076.	transcript	0	0.064568	0	0.560307	0.168978	0	0	0.061791	0
Novel_002812	XLOC_03076.	transcript	0.064276	0	0	0	0	0	0	0.044592	0
Novel_002813	XLOC_03078.	transcript	0.226453	0.044694	0.148816	0.031006	0	0.561878	0.429069	0.215195	0.064916
Novel_002814	XLOC_03078.	transcript	0.238857	0.011516	0.007752	0	0	0.300367	0.105583	0.238491	0
Novel_002815	XLOC_03078.	transcript	0.251513	0.229406	0.034143	0.043792	0.290828	0.014083	0.088294	0	0.18331
Novel_002816	XLOC_03078.	transcript	0	0	0.392374	0.120395	0.03304	0	0.147295	0.227119	0.234971
Novel_002817	XLOC_03088.	transcript	0.552871	0.346667	0.624263	0.993732	0.530555	0.501425	0.600443	1.066376	0.275984
Novel_002818	XLOC_03089.	transcript	0	0	0	0	0	0	0	0	0
Novel_002819	XLOC_03089.	transcript	0	0	0	0	0	0	0	0.004647	0
Novel_002820	XLOC_03089.	transcript	0.154878	0.099739	0	0.320137	0.234685	0	0.2627	0.111151	0
Novel_002821	XLOC_03089.	transcript	0	0.457762	0.203303	0.064996	0	0.411123	0	0.106086	0.250983
Novel_002822	XLOC_03090.	transcript	0.888071	0.170866	0.768717	0.88576	1.000452	1.200224	1.163745	0.905596	0.475812
Novel_002823	XLOC_03090.	transcript	0.022385	0	0.232814	0.169009	0.022082	0.193696	0.100745	0.184376	0.109067
Novel_002824	XLOC_03091.	transcript	0	0.379753	0.497098	0.926424	0.799856	0	0.465554	0.946699	0.517248
Novel_002825	XLOC_03091.	transcript	0	0	0	0	0	0	0.476791	0	0
Novel_002826	XLOC_03091.	transcript	0.096701	0.022565	0	0.096999	0.17362	0.459656	0.027898	0.121309	0.033027
Novel_002827	XLOC_03091.	transcript	0	0	0.294245	0	0	0	0	0.111282	0
Novel_002828	XLOC_03091.	transcript	1.15658	0	0.542375	0.345153	0.407301	2.620141	0.426027	0.366522	0.267369
Novel_002829	XLOC_03093.	transcript	0.130496	0.086995	0.194902	0.125113	0.181245	0.112569	0.085982	0.12037	0
Novel_002830	XLOC_03093.	transcript	0	0.137287	0.10986	0	0	0	0.168234	0.093552	0.211376
Novel_002831	XLOC_03095.	transcript	0.64723	0.813711	0.40373	0.368995	0.504011	0.378639	0.773687	0.45951	0.432324
Novel_002832	XLOC_03098.	transcript	0.579438	0.488892	0.164461	0.045587	0	0	0.059588	0.252236	0.078253
Novel_002833	XLOC_03098.	transcript	0.106648	0.041943	0.026569	0.049001	0.021042	0	0.072757	0.048789	0.086196
Novel_002834	XLOC_03098.	transcript	0.059324	0.042451	0.161228	0.086988	0.052689	0.173698	0.143792	0.032031	0
Novel_002835	XLOC_03098.	transcript	0.100086	0.108718	0.087431	0.219987	0.076941	0.166921	0.126548	0.095557	0.071314
Novel_002836	XLOC_03098.	transcript	0	0	0	0	0	0	0	0	0
Novel_002837	XLOC_03098.	transcript	0	0	0	0	0	0	0	0	0
Novel_002838	XLOC_03098.	transcript	0.035845	0.110626	0.002938	0	0	0	0	0.038912	0

Novel_002839	XLOC_03098.	transcript	0.008733	0.004898	0	0	0	0	0	0.008463	0
Novel_002840	XLOC_03098.	transcript	0.004971	0	0	0	0	0.005084	0	0	0
Novel_002841	XLOC_03098.	transcript	0	0.025276	0.2874	0	0.272006	0	0.196358	0	0.257498
Novel_002842	XLOC_03108.	transcript	0.016072	0	0.053187	0.002524	0	0	0	0	0.004571
Novel_002843	XLOC_03108.	transcript	0.48077	0.377674	0.381701	0.487377	0.406521	0.437295	0.671351	0.383135	0.43519
Novel_002844	XLOC_03109.	transcript	0	0	0	0.096639	0	0.074446	0	0	0
Novel_002845	XLOC_03109.	transcript	0.39696	1.292343	0.753808	0.27756	0.403214	0.192717	0.632009	0.665889	0.220698
Novel_002846	XLOC_03109.	transcript	0.36948	1.613788	0.252957	0.981599	0.712545	1.25114	0.685916	0.548995	1.118325
Novel_002847	XLOC_03109.	transcript	0.28723	0.199472	0.282236	0.436521	0.337658	0.279768	0.408723	0.333793	0.348224
Novel_002848	XLOC_03109.	transcript	0	0	0	0	0	0	0	0.304451	0
Novel_002849	XLOC_03110.	transcript	0.464229	0.554161	0.441035	0.567785	0.770426	0.462971	0.531444	0.51924	0.547341
Novel_002850	XLOC_03110.	transcript	0.025471	0.244553	0.07175	0.009732	0.008684	0.010005	0.05957	0.021699	0.06248
Novel_002851	XLOC_03110.	transcript	0.620641	2.646136	0.938007	0.964435	1.18714	1.097779	1.739901	1.167653	1.526045
Novel_002852	XLOC_03111.	transcript	0.038039	0.133576	0.185671	0.312228	0.278284	0.14327	0.126117	0.247595	0.136401
Novel_002853	XLOC_03111.	transcript	0	0	0	0.06905	0	0	0.111665	0	0
Novel_002854	XLOC_03111.	transcript	0	0	0	0	0	0	0	0.034643	0
Novel_002855	XLOC_03113.	transcript	0	0.513676	0.379556	1.036122	0.904849	0.264776	0.875423	0.937548	0
Novel_002856	XLOC_03113.	transcript	0.792296	0	0.262134	0	0.017905	0.346917	0.520375	0	0.465682
Novel_002857	XLOC_03113.	transcript	0.921584	0	0.756562	0.569169	0.447906	0.738101	0.986015	0.462851	0
Novel_002858	XLOC_03113.	transcript	0.986943	0	1.125537	1.290011	0.718364	0.371688	1.564144	0.733684	0
Novel_002859	XLOC_03113.	transcript	0.649594	0	2.214299	0	0	0	0.410448	0	0
Novel_002860	XLOC_03113.	transcript	0.552487	0.016464	0.734348	0.570784	0.59201	1.010425	0.600709	2.764288	0.479735
Novel_002861	XLOC_03113.	transcript	0	0.067581	0.086509	0.03864	0	0.295313	0.06052	0	0.268571
Novel_002862	XLOC_03113.	transcript	0	0.079934	0.016932	0	0.062827	0.689636	0	0	0
Novel_002863	XLOC_03113.	transcript	0	0.006778	0	0	0	0	0	0	0
Novel_002864	XLOC_03113.	transcript	1.577078	0	0.002021	0	0	0	0.522485	0	0
Novel_002865	XLOC_03113.	transcript	0.31733	0	0.003272	0	0	0	0.282212	0	0
Novel_002866	XLOC_03113.	transcript	0.592686	0	1.521474	0.211803	0.207158	0.125851	1.365143	0.112876	4.60E-05
Novel_002867	XLOC_03113.	transcript	2.00161	0.343078	3.690034	1.123206	4.304497	2.7215	2.246763	1.738176	3.218846
Novel_002868	XLOC_03114.	transcript	0.338464	0.007108	0.177157	0.126664	0.43923	0.398865	0.395946	0.289315	0.089405
Novel_002869	XLOC_03114.	transcript	0.116444	0.820359	0.042666	0.067647	0.096312	0.064923	0.086591	0.130885	0.21897
Novel_002870	XLOC_03115.	transcript	0.092283	0.40595	0.234731	0.144298	0.218882	0.180773	0.419616	0.144522	0.289855
Novel_002871	XLOC_03116.	transcript	0	0.445234	0.002746	0.008294	0	0	4.10E-05	0.005259	0
Novel_002872	XLOC_03116.	transcript	0.788207	0.377734	0.8085	0.881257	0.810136	0.894679	0.977558	1.009737	0.833331
Novel_002873	XLOC_03116.	transcript	0.326418	0.009199	0	0.032997	0.885948	0.030693	0.020965	0.003338	0
Novel_002874	XLOC_03116.	transcript	0.408145	0.970607	0.646253	1.094053	0.053099	0.8924	0.973835	0.82999	0.606237
Novel_002875	XLOC_03116.	transcript	0	0	0.328972	0	0	0	0	0	0
Novel_002876	XLOC_03118.	transcript	0.064728	0	0.250014	0	0	0.244317	0	0	0

Novel_002877	XLOC_03118.	transcript	0.376815	1.172789	0	0	0	0	0	0	0
Novel_002878	XLOC_03120.	transcript	0.129411	0	0.135104	0.199036	0.287357	0.663958	0.097001	0.209032	0.025633
Novel_002879	XLOC_03121.	transcript	0.042794	0.577068	0.039603	0.007254	0.045115	0.02289	0.045549	0.032442	0.011233
Novel_002880	XLOC_03122.	transcript	0.689647	6.267199	0.34463	0.274031	0.355629	0.43797	0.952312	0.307937	2.652362
Novel_002881	XLOC_03139.	transcript	0	0	0	0	0	0	0	0.232643	0
Novel_002882	XLOC_03139.	transcript	0.238109	0.035643	0.28395	0.277836	0.20297	0.059371	0.250377	0.279753	0.371807
Novel_002883	XLOC_03142.	transcript	0.352902	0.323369	0.148269	0.128229	0.269501	0.149327	0.415405	0.400672	0.237347
Novel_002884	XLOC_03142.	transcript	0.397985	0.521827	0.71075	0	0	0.542393	0.14298	0.734789	0.843944
Novel_002885	XLOC_03142.	transcript	0.056402	0.095919	0.001026	0.571911	0.666619	0.004839	0.879573	0.019713	0.00465
Novel_002886	XLOC_03144.	transcript	0.594041	0.257471	0.125933	0.051368	0.376504	0.210539	0.772432	0.079371	0.381449
Novel_002887	XLOC_03144.	transcript	0	0.626915	0	0	0.087699	0.017489	0.021945	0.333658	0
Novel_002888	XLOC_03144.	transcript	0	0.191431	0.463695	0.154674	0	0	0.194141	0.095817	0.124791
Novel_002889	XLOC_03144.	transcript	0	0	0	0	0	0	0	0	0
Novel_002890	XLOC_03144.	transcript	0.040427	0.096044	0	0.171876	0	0.115761	0	0	0
Novel_002891	XLOC_03144.	transcript	0	0	0	0.000609	0	0	0	0.202964	0
Novel_002892	XLOC_03155.	transcript	0.403389	0.036603	0.187196	0.295714	0.193917	0.318929	0.111568	0.166183	0
Novel_002893	XLOC_03155.	transcript	0	0.150861	0.241198	0.489696	0.411773	0.248592	0.698092	0.511612	0.341382
Novel_002894	XLOC_03158.	transcript	4.828329	3.809962	5.594837	7.953311	5.911478	5.99616	6.303795	7.247302	4.606265
Novel_002895	XLOC_03158.	transcript	0	0	0	0	0	0	0	0	0
Novel_002896	XLOC_03164.	transcript	0	0	0	0	0	0	0	0	0
Novel_002897	XLOC_03164.	transcript	0.972515	0.151006	1.608892	1.696352	1.413434	1.555543	1.400103	1.93376	1.201977
Novel_002898	XLOC_03164.	transcript	0	0.316689	0.031163	0	0	0.035564	0.109933	0.050629	0.174665
Novel_002899	XLOC_03165.	transcript	0.070062	0.383919	0.079978	0.091943	0.163722	0.187284	0.214447	0.106042	0.131362
Novel_002900	XLOC_03165.	transcript	0.295027	0.505607	0.598608	0.723757	0.351804	0.450586	0.324044	0.45784	0.144817
Novel_002901	XLOC_03165.	transcript	0.149775	0	0.172059	0.200305	0.111684	0.118217	0.165715	0.034903	0.027657
Novel_002902	XLOC_03165.	transcript	0.014165	0	0	0	0.406404	0.509492	0	0.029287	0
Novel_002903	XLOC_03165.	transcript	0	0.961357	0	0	0	0	0.243365	0	0.468422
Novel_002904	XLOC_03166.	transcript	0.275748	4.017648	0.356085	0.275689	0.301954	0.327232	0.324321	0.240572	0.242137
Novel_002905	XLOC_03174.	transcript	0	0	0	1.206053	0	0	0	0	0
Novel_002906	XLOC_03174.	transcript	0.614478	1.384045	1.237296	0.920265	0.81337	1.219004	0.796172	1.194293	0.448989
Novel_002907	XLOC_03177.	transcript	0.024362	0	0.017098	0	0.067018	0	0.035779	0	0
Novel_002908	XLOC_03177.	transcript	0.074018	0	0.043074	0.020054	0.04571	0.0628	0.034834	0.442413	0
Novel_002909	XLOC_03177.	transcript	0.907253	1.655861	1.033805	0.908676	1.161087	0.954737	1.419653	0.26024	0.750224
Novel_002910	XLOC_03177.	transcript	0	0.00067	0.2308	0.007563	0	0	0.014736	0.822875	0
Novel_002911	XLOC_03182.	transcript	0.226399	0.077268	0.255645	0.307543	0.253464	0.17919	0.462398	0.465794	0.281537
Novel_002912	XLOC_03182.	transcript	0	0	0	0	0.042974	0	0	0	0
Novel_002913	XLOC_03188.	transcript	0	0	0	0.245593	0	0	0	0	0
Novel_002914	XLOC_03188.	transcript	0.079546	0.360301	0	0.01798	0	0.343749	0.17476	0.316606	0.073213

Novel_002915	XLOC_03190.	transcript	0.102583	0.047859	0.091028	0.055834	0.037168	0.168692	0.207572	0.078672	0.141736
Novel_002916	XLOC_03190.	transcript	0	0	0	0.005158	0.05736	0	0	0	0
Novel_002917	XLOC_03190.	transcript	0.384206	0.242185	0.041544	0.007211	0.25605	0.001074	0.073351	0.009233	0.000606
Novel_002918	XLOC_03190.	transcript	0	0.067583	0	0	0	0	0	0	0.078804
Novel_002919	XLOC_03190.	transcript	0.091335	0	0	0	0	0	0	0	0
Novel_002920	XLOC_03190.	transcript	0.392215	0	0	0	0	0	0	0	0.002156
Novel_002921	XLOC_03190.	transcript	0.18644	0	0	0	0	0	0	0	0
Novel_002922	XLOC_03190.	transcript	0.23956	0	0	0	0	0	0	0	0.204217
Novel_002923	XLOC_03190.	transcript	0	0.078674	0.259237	0	0.230293	0	0	0	0.10016
Novel_002924	XLOC_03190.	transcript	0.071616	0.073835	0.078155	0	0.182134	0	0	0	0.230952
Novel_002925	XLOC_03190.	transcript	0.07676	0.046034	0.231988	0.312354	0	0.263155	0	0.040161	0
Novel_002926	XLOC_03190.	transcript	0	0.051241	0	0	0	0	0	0.148817	0
Novel_002927	XLOC_03190.	transcript	0	0	0	0	0	0	0	0.076556	0
Novel_002928	XLOC_03193.	transcript	0.263125	0.384355	0.286291	0.148152	0.282012	0.276183	0.169426	0.388351	0.192206
Novel_002929	XLOC_03194.	transcript	0.079589	0.12016	0.124169	0.098799	0.15272	0.124166	0.313606	0.27293	0.06009
Novel_002930	XLOC_03195.	transcript	0.58525	0.564185	0.407428	0.426093	0.389558	0.455187	0.585343	0.444522	0.5575
Novel_002931	XLOC_03195.	transcript	0	0	0	0	0.012029	0	0	0	0
Novel_002932	XLOC_03197.	transcript	0.68004	0	0.513878	0	0.424983	0	0	0	0
Novel_002933	XLOC_03197.	transcript	0	0	0.153022	0	0	0	0.050489	0	0
Novel_002934	XLOC_03197.	transcript	0.299012	0.163182	0.165265	0.218603	0.193233	0.600763	0	0.257098	0.374131
Novel_002935	XLOC_03197.	transcript	0	0.128242	0.178089	0.086712	0	0.49568	0.055812	0.176795	0
Novel_002936	XLOC_03197.	transcript	0.254028	0.12723	0.174255	0.153477	0.215924	0.15912	0	0.17797	0.358467
Novel_002937	XLOC_03197.	transcript	0	0.111815	0.132663	0.383173	0	0.003525	0.189073	0.72613	0
Novel_002938	XLOC_03197.	transcript	0.000116	0.144119	0.133626	0.183026	6.30E-05	0.005097	0.213076	0.204506	0
Novel_002939	XLOC_03197.	transcript	0.344969	0	0.175989	0	0.246169	0	1.007638	0	0
Novel_002940	XLOC_03197.	transcript	0.328418	0.371184	0.351264	0.2869	0.298162	0.46103	0.518022	0.332217	0.342376
Novel_002941	XLOC_03197.	transcript	0.223927	0	0	0	0.1606	0.087508	0.217102	0.101384	0
Novel_002942	XLOC_03197.	transcript	0.302493	0.225921	0.29596	0.340868	0.319746	0.330843	0.367213	0.299819	0.210342
Novel_002943	XLOC_03197.	transcript	0.486901	0.392086	0	0	0	0	0	0	0
Novel_002944	XLOC_03197.	transcript	0	0.405939	0.485857	0.24781	0.594384	0.538166	0.741461	0	0.444635
Novel_002945	XLOC_03197.	transcript	0	0	0.036204	0.165714	0.003634	0	0.001441	0.400631	0
Novel_002946	XLOC_03198.	transcript	0	0.205884	0	0.022001	0.018605	0.060272	0.073533	0.010482	0
Novel_002947	XLOC_03198.	transcript	0	0	0	0.026998	0.032562	0	0	0.036887	0.076029
Novel_002948	XLOC_03198.	transcript	0.076191	0.101736	0.098909	0.150734	0.120812	0.218381	0.310343	0.004862	0
Novel_002949	XLOC_03198.	transcript	0.034382	0.370412	0	0	0.040869	0.068929	0	0.252153	0.131513
Novel_002950	XLOC_03199.	transcript	0	0.592806	0	0.385461	0.448775	0.521606	0.575901	0.50996	0.320739
Novel_002951	XLOC_03199.	transcript	0.014231	0	0.38392	0	0	0	0.004993	0.002725	0
Novel_002952	XLOC_03199.	transcript	0.657601	0	0.095961	0.012523	0.041177	0.036535	0.033943	0.014588	0.058651

Novel_002953	XLOC_03199.	transcript	0.233281	0.397312	0.193315	0.148571	0.360051	0.190557	0.220486	0.158269	0.172385
Novel_002954	XLOC_03201.	transcript	0.236752	0.424523	0.372761	0.361097	0.319581	0.370883	0.390046	0.431616	0.305004
Novel_002955	XLOC_03203.	transcript	0.361659	0.017152	0.30317	0.313876	0.459811	0.294758	0.627667	0.432329	0.226647
Novel_002956	XLOC_03203.	transcript	0	0	0.057932	0	0.059005	0.213674	0.071681	0.054769	0
Novel_002957	XLOC_03203.	transcript	0.029137	0	0.034979	0	0.026657	0.014287	0.017585	0.086938	0
Novel_002958	XLOC_03203.	transcript	0	0	0	0	0	0	0	0	0
Novel_002959	XLOC_03203.	transcript	0.426063	0.012369	0.146411	0.003602	0.055919	0.389036	0.236812	0.355158	0.080302
Novel_002960	XLOC_03203.	transcript	0.00512	0.410322	0.399255	0.578893	0.512308	0.052234	0.297117	0.064745	0.543215
Novel_002961	XLOC_03217.	transcript	0.186469	0.000437	0.000811	0.178596	0.481404	0.013247	0.214337	0.146786	0.000533
Novel_002962	XLOC_03218.	transcript	0	0.204805	0	0.136828	0.024529	0	0.012522	0.228634	0
Novel_002963	XLOC_03218.	transcript	0.22725	0	0.2148	0.03956	0.428572	0.11883	0.379652	0	0.107579
Novel_002964	XLOC_03218.	transcript	0.399815	0.356777	0.366826	0.575901	0.489153	0.423509	0.730502	0.640162	0.485554
Novel_002965	XLOC_03218.	transcript	0.157387	0.004264	0.088752	0.021875	0.162485	0.081719	0.033212	0	0.189793
Novel_002966	XLOC_03218.	transcript	0	0	0.334455	0	0.1728	0	0	0.521125	0
Novel_002967	XLOC_03218.	transcript	0.032102	0.291665	0.170008	0.478495	0.282992	0.421371	0.63516	0.255863	0.307276
Novel_002968	XLOC_03220.	transcript	0	0.160866	0.068068	0	0	0	0	0	0.057746
Novel_002969	XLOC_03220.	transcript	0.170344	0	0	0.019224	0.061891	0.298852	0.018337	0	0
Novel_002970	XLOC_03220.	transcript	0	0.193996	0.269582	0.269462	0.314842	0.105412	0.426327	0.392783	0.204411
Novel_002971	XLOC_03220.	transcript	0	0	0.02146	0	0	0	0	0	0
Novel_002972	XLOC_03220.	transcript	0	0	0	0	0	0	0.210339	0.404806	0.183703
Novel_002973	XLOC_03221.	transcript	0.466693	1.14767	0.519252	0.770995	0.532048	0.597788	0.570803	0.61752	0.425206
Novel_002974	XLOC_03221.	transcript	0.006364	0.80175	0.01226	0.116609	0.232735	0.169493	0.518466	0.205813	0.017448
Novel_002975	XLOC_03221.	transcript	0	1.673977	0	0	0	0	0	0	0
Novel_002976	XLOC_03225.	transcript	0.188393	0.431993	0.230605	0.412309	0.363534	0.116421	0.427198	0.156403	0.353478
Novel_002977	XLOC_03226.	transcript	0.155354	0	0.005071	0.075922	0.076034	0	0	0	0
Novel_002978	XLOC_03226.	transcript	0.02216	0	0.402637	0.054997	0.071719	0.116097	0.21484	0.094254	0.070792
Novel_002979	XLOC_03226.	transcript	0	0	0.153814	0.10244	0.113426	0	0	0.223233	0
Novel_002980	XLOC_03226.	transcript	0.175385	0.004593	0.16178	0.067459	0.151224	0.102296	0.256957	0.137669	0.029224
Novel_002981	XLOC_03227.	transcript	0.018178	0	0	0.040867	0	0	0	0	0
Novel_002982	XLOC_03227.	transcript	0.041463	0.015314	0.104769	0.036545	0.208013	0.077818	0.215903	0.081435	0.165571
Novel_002983	XLOC_03227.	transcript	0.101757	0	0.055938	0.034995	0	0.04189	0.12641	0.039051	0.094282
Novel_002984	XLOC_03227.	transcript	0.255908	0	0.146827	0.065031	0.243494	0.14024	0.095087	0.032947	0.025748
Novel_002985	XLOC_03227.	transcript	0.07431	0.02934	0.0765	0.026319	0	0.04015	0.077604	0.024681	0.052936
Novel_002986	XLOC_03227.	transcript	0.218688	0.408086	0.084438	0.19335	0.130916	0.103887	0.175675	0.340792	0.112079
Novel_002987	XLOC_03229.	transcript	0.524532	0	0	0	0.776237	0.580735	0	0.213484	0.323914
Novel_002988	XLOC_03231.	transcript	0.864148	1.226691	0.453369	2.195726	2.885554	2.496548	0.857739	1.210942	3.103089
Novel_002989	XLOC_03231.	transcript	0	0	4.00E-06	0	0	0	0	0	0
Novel_002990	XLOC_03231.	transcript	4.950716	1.14092	5.857301	3.582312	5.155168	2.392978	6.759376	6.259962	0

Novel_002991	XLOC_03232.	transcript	0.061386	2.194708	0.130337	0.112338	0.079978	0.158401	0.214353	0.173699	0.484622
Novel_002992	XLOC_03232.	transcript	0.190929	0.049384	0.052432	0	0	0.172881	0.051099	0.034115	0.070145
Novel_002993	XLOC_03233.	transcript	0.25082	0.033239	0.448661	1.822436	0.655494	0.521742	0.183929	0.498808	0.111166
Novel_002994	XLOC_03233.	transcript	0	0	0	0.078028	0	0.165495	0	0.24115	0
Novel_002995	XLOC_03238.	transcript	0	0	0	0	0	0	0	0	0
Novel_002996	XLOC_03238.	transcript	0.740334	0.699864	0.703617	0.847932	0.629005	0.353646	0.7312	0.756625	0.499365
Novel_002997	XLOC_03238.	transcript	0.024636	0.040836	0.375656	2.340595	0.932558	0.50011	0.606403	0.925993	0.122043
Novel_002998	XLOC_03239.	transcript	0.260874	0.094668	0.250049	0.279585	0.352582	0.201147	0.399136	0.301838	0.267511
Novel_002999	XLOC_03241.	transcript	0.098248	0.912511	0.021421	0.013746	0.033521	0.05943	0.160994	0.109688	0.275516
Novel_003000	XLOC_03245.	transcript	0.835761	0.083716	0.063451	0.620892	0.733529	0	0.426862	0.587002	0
Novel_003001	XLOC_03245.	transcript	0	0.041862	1.000888	0.926539	0.512056	0.859796	0.792948	0.670627	0.725252
Novel_003002	XLOC_03245.	transcript	0	0	0	0	0	0	0	0	0
Novel_003003	XLOC_03246.	transcript	0	0.753188	0	0	0.246134	0	0.060063	0	0
Novel_003004	XLOC_03252.	transcript	0.019628	0.407439	0.061906	0.01645	0.080299	0.094488	0.102462	0	0.173606
Novel_003005	XLOC_03253.	transcript	0	0.194379	0	0.013916	0.033965	0	0.01791	0.037758	0
Novel_003006	XLOC_03253.	transcript	0.123176	0.793027	0	0.102884	0	0.074366	0	0	0
Novel_003007	XLOC_03256.	transcript	0.307517	0.192656	0.192719	0.238571	0.250275	0.24567	0.236869	0.519769	0.376559
Novel_003008	XLOC_03260.	transcript	0.66673	0.004641	0.680235	1.020778	0.833662	0.677566	0.850715	1.047297	0.394369
Novel_003009	XLOC_03262.	transcript	0.100988	0.127926	0.02137	0.047888	0.141205	0.056585	0.202723	0.118445	0.126113
Novel_003010	XLOC_03262.	transcript	0	0.468061	0.136574	0	0	0	0.038125	0.010571	0
Novel_003011	XLOC_03268.	transcript	0	0	0	0	0	0	0	0	1.28004
Novel_003012	XLOC_03268.	transcript	0.182934	0.457869	0.453542	0.539259	0.366428	0.309948	0.934571	0.58935	0.306443
Novel_003013	XLOC_03269.	transcript	7.562133	4.485594	7.863806	9.355324	6.606603	8.216729	9.147262	8.936501	8.119742
Novel_003014	XLOC_03271.	transcript	0.193512	0.283587	0.312797	0.312095	0.020326	0.490711	0.053748	0.107353	0.071784
Novel_003015	XLOC_03271.	transcript	0	0.539088	0	0	0	0	0	0	0
Novel_003016	XLOC_03271.	transcript	0	0.750473	0	0	0	0	0	0	0
Novel_003017	XLOC_03274.	transcript	0.288631	0.981683	0.485298	0.445364	0.395846	0.422206	0.530834	0.546017	0.618533
Novel_003018	XLOC_03274.	transcript	0.039798	0	0.216636	0.144093	0.009354	0.016314	0.179655	0.388144	0
Novel_003019	XLOC_03274.	transcript	0	0.808552	0	0.110671	0.279867	0	0.125901	0.153001	0.022112
Novel_003020	XLOC_03274.	transcript	0.300769	0.002938	0.189899	0.127272	0.15255	0.401207	0.197055	0	0.342021
Novel_003021	XLOC_03274.	transcript	0	0.218103	0	0	0	0	0	0	0
Novel_003022	XLOC_03274.	transcript	0	0	0	0.005028	0.118217	0.262651	0	0.085573	0
Novel_003023	XLOC_03274.	transcript	0.15267	0	0.046638	0.392618	0.272431	0.071139	0.24143	0.146145	0.097137
Novel_003024	XLOC_03274.	transcript	0.113522	0.004263	0.111014	0.052604	0	0.064895	0.091737	0.109145	0.201465
Novel_003025	XLOC_03274.	transcript	0.168132	0	0.047778	0	0	0	0.232001	0	0.009456
Novel_003026	XLOC_03279.	transcript	0.623744	0.877464	0.391385	0.283947	0.550444	0.35923	0.423907	0.407508	0.226647
Novel_003027	XLOC_03281.	transcript	0	0	0	0	0	0	0	0	0
Novel_003028	XLOC_03281.	transcript	0	0	0	0	0	0	0	0	0

Novel_003029	XLOC_03281.	transcript	0	0	0	0	0	0	0	0.487212	0
Novel_003030	XLOC_03281.	transcript	0.246791	0.08363	0.306308	0.462147	0.573179	0.379992	0.631913	0.134241	0.16832
Novel_003031	XLOC_03281.	transcript	0.568337	0	0.336574	0.302891	0.176242	0.268861	0.364867	0.188118	0.224238
Novel_003032	XLOC_03281.	transcript	0	0	0	0	0	0	0	0	0.515686
Novel_003033	XLOC_03281.	transcript	0.016288	0	0	0	0.043268	0.037826	0	0	0.160925
Novel_003034	XLOC_03283.	transcript	1.238834	3.195192	1.665736	1.724997	0.976737	2.461993	1.729082	2.602582	1.915278
Novel_003035	XLOC_03284.	transcript	0.473759	0.42341	0.367963	0.233965	0.388548	0.455291	0.445376	0.269807	0.259036
Novel_003036	XLOC_03285.	transcript	0.01313	0.00383	0	0	0	0	0	0.239575	0
Novel_003037	XLOC_03288.	transcript	0.038334	0	0.087856	0	0	0	0.02114	0.088085	0
Novel_003038	XLOC_03289.	transcript	0	0	0	0	0	0.058369	0.319601	0.326136	0.171714
Novel_003039	XLOC_03290.	transcript	0.002438	0	0	0	0.042568	0	0.004807	0	0
Novel_003040	XLOC_03290.	transcript	0.228839	0.391428	0.210841	0.422109	0.153084	0.379264	0.499404	0.305526	0.208605
Novel_003041	XLOC_03291.	transcript	0.258395	3.219231	0.607943	0.337308	0.397846	0.504998	0.672615	0.32448	0.794928
Novel_003042	XLOC_03291.	transcript	0.431524	0.019284	0.032373	0.153755	0.470105	0.007793	0.137771	0.290897	0.028372
Novel_003043	XLOC_03291.	transcript	0	0	0	0	0.210854	0	0	0	0
Novel_003044	XLOC_03291.	transcript	0	0	0	0	0	0	0	0	0
Novel_003045	XLOC_03291.	transcript	0	0	0	0	0	1.054506	0.216754	0.277234	0
Novel_003046	XLOC_03291.	transcript	0	0	0	0.564652	0	0	0	0	0
Novel_003047	XLOC_03291.	transcript	0.944789	0.415906	1.175644	0.136195	0.26816	0.045816	0.252432	0.128775	1.266365
Novel_003048	XLOC_03291.	transcript	0	0	0	0	0	0	0	0	0
Novel_003049	XLOC_03303.	transcript	0.121707	0	0.148895	0.118039	0.120879	0.106203	0.319505	0.104021	0.250504
Novel_003050	XLOC_03304.	transcript	0.092276	0.07423	0.153707	0.028184	0.262026	0.191158	0.151532	0.141669	0.141529
Novel_003051	XLOC_03304.	transcript	0	0	0	0	0	0	0	0	0
Novel_003052	XLOC_03304.	transcript	0	0	0	0.264736	0.040148	0	0.137385	0.097374	0
Novel_003053	XLOC_03305.	transcript	0	0	0	0	0.085711	0	0	0	0
Novel_003054	XLOC_03306.	transcript	1.259545	0.734151	2.673153	1.982428	2.381082	2.9557	3.441239	2.600054	3.007413
Novel_003055	XLOC_03306.	transcript	0	0	0	0	0	0	0	0	0
Novel_003056	XLOC_03306.	transcript	0	0	0	0	0	0	0	0	0
Novel_003057	XLOC_03306.	transcript	0.448057	1.012326	1.311481	1.164111	0.820602	1.418667	0.602405	0.50452	0.475377
Novel_003058	XLOC_03307.	transcript	0	0	0	0	0	0	0.31592	1.60E-05	0
Novel_003059	XLOC_03307.	transcript	0	0	0.063771	1.298378	0	0	0	0	1.042958
Novel_003060	XLOC_03307.	transcript	0	0.266331	0.308775	0	0	0.09484	0.421105	0.166139	0
Novel_003061	XLOC_03307.	transcript	0.604737	0.393753	0.62232	0	0.216717	0.965882	0.21081	0.560615	0
Novel_003062	XLOC_03307.	transcript	0	0.251219	0	0.079231	0.62473	0	0	0.189698	0.085054
Novel_003063	XLOC_03307.	transcript	0	0	0	0.015347	0	0.01283	0	0	0.007651
Novel_003064	XLOC_03307.	transcript	0	0	0	0	0	0	0	0	0
Novel_003065	XLOC_03308.	transcript	0.161294	0.516302	0.380519	0.707857	0.551958	0.719513	0.678	0.632726	0.488992
Novel_003066	XLOC_03308.	transcript	0.246861	0.017584	0.08775	0.283789	0.193143	0.254231	0	0.176073	0

Novel_003067	XLOC_03312.	transcript	0.003087	0.011551	0.000251	0.002737	0.006446	0.010949	0.022787	0.009312	0.000942
Novel_003068	XLOC_03312.	transcript	0	0.422002	0.466944	0.439423	0.119552	0.108152	0.60403	0.398531	0.502854
Novel_003069	XLOC_03312.	transcript	0.33893	0.220343	0.1635	0.16491	0.28418	0.421818	0.276964	0.255168	0.145443
Novel_003070	XLOC_03312.	transcript	0.003396	0.044084	0.039217	0.013928	0.074948	0.050159	0.015424	0.043659	0.033712
Novel_003071	XLOC_03312.	transcript	0	0	0	0.005866	0.024676	0	0	0	0.017843
Novel_003072	XLOC_03315.	transcript	0.188876	0.212933	1.216815	1.349609	0.885865	0.650967	0.730255	0.746419	0.945538
Novel_003073	XLOC_03321.	transcript	0	0.004763	0.175715	0.030864	0.139661	0.270744	0.081733	0.029255	0.021858
Novel_003074	XLOC_03326.	transcript	0	0	0.120601	0	0.110072	0.065715	0.063381	0.163282	0.063083
Novel_003075	XLOC_03326.	transcript	0.751011	0.681636	1.045037	1.54653	0.972304	0.999347	1.05238	1.142122	0.937038
Novel_003076	XLOC_03328.	transcript	0.050177	0.015192	0.096115	0.022998	0.077531	0.075793	0.262757	0.148521	0.331867
Novel_003077	XLOC_03328.	transcript	0.168977	0	0.162559	0.069779	0.394625	0.117529	0.185794	0.157672	0.155306
Novel_003078	XLOC_03328.	transcript	0	0	0	0	0	0	0	0	0
Novel_003079	XLOC_03328.	transcript	0.59053	0.433981	0.499032	0.327079	0	0.265225	0.535661	0.92864	0.366943
Novel_003080	XLOC_03328.	transcript	0.095049	0	0.141065	1.14569	0.167755	0.034166	0.097502	0.016851	1.170186
Novel_003081	XLOC_03328.	transcript	0.502271	2.072033	0.574792	0	0.744495	0.931038	0.837955	0.570645	0
Novel_003082	XLOC_03328.	transcript	0.043187	0.198199	0.248163	0.049398	0.128756	0.259106	0.1223	0.291082	0.120513
Novel_003083	XLOC_03328.	transcript	0.167494	0.246491	0.17021	0.221895	0.061385	0	0.290855	0	0.146677
Novel_003084	XLOC_03328.	transcript	0	0.203852	0.046114	0.04748	0.090287	0.172213	0.130476	0.194155	0.015515
Novel_003085	XLOC_03329.	transcript	0.407952	0.30341	0.945913	0	0.584607	1.109826	0.063818	0.839025	0.550812
Novel_003086	XLOC_03329.	transcript	0.436714	0.008337	0.020208	0.891077	0.580264	0.056062	1.083324	0.162426	0.612596
Novel_003087	XLOC_03329.	transcript	0.097802	3.514834	0.111948	0.08857	0.124528	0.280096	0.360718	0.012037	0.300208
Novel_003088	XLOC_03329.	transcript	0.071807	1.407201	0.060571	0	0.126178	0.214841	0.321603	0.095966	0.397625
Novel_003089	XLOC_03330.	transcript	0	0.004923	0	0	0	0	0	0.181129	0
Novel_003090	XLOC_03330.	transcript	0.102	0.316511	0.197113	0.375291	0.290401	0.454824	0.230734	0.39318	0.204729
Novel_003091	XLOC_03331.	transcript	0	1.115772	0	0.168931	0	0	0.037687	0	0.0637
Novel_003092	XLOC_03331.	transcript	0.219533	2.55174	0.231871	0.07801	0.06446	0.129775	0.305827	0.243868	0.148013
Novel_003093	XLOC_03332.	transcript	0.403557	8.076556	0.363524	0.637919	0.685598	0.740218	0.516381	0.413516	1.603262
Novel_003094	XLOC_03335.	transcript	0.036081	0.008983	0.114228	0	0	0.071883	0.168988	0.111105	0.057783
Novel_003095	XLOC_03335.	transcript	0	0	0	0	0	0	0	0.029779	0
Novel_003096	XLOC_03335.	transcript	0	0	0	0	0	0	0	0	0
Novel_003097	XLOC_03335.	transcript	0.063647	0.090734	0.021064	0	0.116973	0.281753	0	0.002024	0.404106
Novel_003098	XLOC_03335.	transcript	0.196892	0	0.108179	0.382219	0.22588	0.203145	0.222064	0.29287	0.369473
Novel_003099	XLOC_03335.	transcript	0	0	0.254761	0.329202	0	0	0	0	0
Novel_003100	XLOC_03335.	transcript	0	0	0.15731	0.095171	0.423876	0.368375	0.346791	0.004425	0
Novel_003101	XLOC_03335.	transcript	0.041682	0	0	0	0	0	0.147462	0.000512	0
Novel_003102	XLOC_03335.	transcript	0.176739	0	0.191485	0	0	0	0	0.055958	0
Novel_003103	XLOC_03335.	transcript	0.022352	0	0.197296	0.078056	0.082805	0	0.11092	0	0.019131
Novel_003104	XLOC_03335.	transcript	0	0	0	0	0	0	0.09789	0	0

Novel_003105	XLOC_03335.	transcript	0	0.03436	0	0	0	0.127231	0	0.281597	0.086307
Novel_003106	XLOC_03335.	transcript	0	0	0	0	0	0	0	0.164181	0
Novel_003107	XLOC_03335.	transcript	1.00E-06	0.017024	0	0	0	0	0	0	0
Novel_003108	XLOC_03335.	transcript	0.234639	0	0	0.51916	0.378563	0	0.49731	0	0
Novel_003109	XLOC_03335.	transcript	0	0	0.242135	0	0.003213	0.049379	0.002663	0.040023	0.0173
Novel_003110	XLOC_03335.	transcript	0.379846	0.252572	0.698386	0.442196	0.370831	0.17392	0.451477	0.89192	0.793655
Novel_003111	XLOC_03335.	transcript	0	0	0	0.578482	0.186025	0	0.058812	0.087548	0
Novel_003112	XLOC_03335.	transcript	0	0	1.471394	0.002921	0.407215	1.339333	0.002237	0.193776	0.665655
Novel_003113	XLOC_03335.	transcript	0	0	0	0.28408	0	0	0	0.28327	0
Novel_003114	XLOC_03335.	transcript	0	0	0	0.159142	0.322105	0	0.479046	0.47503	0
Novel_003115	XLOC_03336.	transcript	3.073607	0.633587	3.998199	2.261894	3.677319	3.684504	3.827805	3.209101	2.059829
Novel_003116	XLOC_03336.	transcript	0	0	0	0	0	0	0	0	0
Novel_003117	XLOC_03336.	transcript	0.252444	0.044773	0.310889	0.327007	0.497936	0.354147	0.41359	0.323914	0.354522
Novel_003118	XLOC_03336.	transcript	0.262675	0.182274	0.402188	0.433563	0.379589	0.486279	0.101641	0.164205	0.543647
Novel_003119	XLOC_03336.	transcript	0	0	0	0	0	0	0.38093	0.506406	0
Novel_003120	XLOC_03336.	transcript	0	0	0	0.106294	0.052278	0.043615	0.114824	0.052547	0.256135
Novel_003121	XLOC_03336.	transcript	0	0.091176	0	0.050846	0	0.11787	0	0.130623	0
Novel_003122	XLOC_03336.	transcript	0.155332	0.013142	0.252493	0.086425	0.154911	0	0.016007	0.173586	0.035726
Novel_003123	XLOC_03337.	transcript	0	0	0.084536	0	0	0	0	0.088385	0.689695
Novel_003124	XLOC_03338.	transcript	0	0	0	0	0	0	0	0.327625	0
Novel_003125	XLOC_03338.	transcript	0.684457	0.211876	1.198649	0.609046	0.488034	1.026298	0.773617	0.854061	0.35529
Novel_003126	XLOC_03339.	transcript	0	0.218424	0.072706	0	0.039234	0.027474	0.017144	0.032812	0.027167
Novel_003127	XLOC_03340.	transcript	0	0	0	0	0	0	0.042314	0	0
Novel_003128	XLOC_03341.	transcript	0.311962	0.407614	0.076663	0.572958	0	0.475281	0.064193	0.444497	0.134001
Novel_003129	XLOC_03341.	transcript	0.360518	0.665697	0.791816	0.548642	0.819064	0.499723	1.093452	0.505472	0.555974
Novel_003130	XLOC_03349.	transcript	0.354572	0.238842	0.406961	0.205225	0.1293	0.526521	0.397724	0.192286	0.153305
Novel_003131	XLOC_03349.	transcript	0.028485	0.310553	0.008362	0.188199	0.651202	0	0.120725	0.371915	0.221191
Novel_003132	XLOC_03349.	transcript	0.247498	0.389388	0.207425	0.027262	0.281337	0.212179	0.224428	0.127352	0.189416
Novel_003133	XLOC_03349.	transcript	0.129433	0.104928	0	0.038941	0.052315	0	0.148847	0.103188	0
Novel_003134	XLOC_03349.	transcript	0.095671	0.018692	0	0	0	0	0.025809	0	0
Novel_003135	XLOC_03349.	transcript	0	0.169079	0	0.089344	0	0	0.10355	0	0
Novel_003136	XLOC_03349.	transcript	0.528631	0.022813	0.113648	0.070298	0.206633	0.191644	0.060421	0.040662	0.026438
Novel_003137	XLOC_03349.	transcript	0	0	0	0	0	0	0	0	0
Novel_003138	XLOC_03349.	transcript	0.168797	0.317161	0.038259	0.166407	0.030942	0.061885	0.103728	0.08068	0.182932
Novel_003139	XLOC_03349.	transcript	0.703389	0.212928	0.854052	0.685571	0.726206	0.837948	1.412458	1.338209	1.164549
Novel_003140	XLOC_03353.	transcript	0.055295	0.2307	0.325083	0.148153	0.169125	0.450224	0.248007	0.194718	0.181925
Novel_003141	XLOC_03353.	transcript	0.270107	2.074538	0.007019	0.046886	0.057256	0.566594	0.215418	0.104121	0.070747
Novel_003142	XLOC_03353.	transcript	0.144242	1.736876	0.041908	0	0.035862	0	0.095138	0	0.066121

Novel_003143	XLOC_03353.	transcript	0.023785	0.584284	0	0	0	0	0.034957	0.037326	0.06896
Novel_003144	XLOC_03353.	transcript	0	0.780234	0.184885	0	0	0	0.049838	0	0.192835
Novel_003145	XLOC_03353.	transcript	0	1.196241	0.024192	0.027853	0.014773	0.000553	0.111696	0	0
Novel_003146	XLOC_03353.	transcript	0	0.434668	0	0	0	0	0	0	0
Novel_003147	XLOC_03353.	transcript	0	0.197432	0	0	0	0.05987	0	0	0.308657
Novel_003148	XLOC_03353.	transcript	0	0.403335	0	0	0	0.4656	0.008198	0	0
Novel_003149	XLOC_03353.	transcript	0	0.210453	0	0.217061	0	0	0	0	0.158731
Novel_003150	XLOC_03353.	transcript	0	0.201471	0	0.063329	0	0	0	0	0
Novel_003151	XLOC_03353.	transcript	0	0.254422	0	0	0	0	0	0	0
Novel_003152	XLOC_03353.	transcript	0	0.184892	0	0	0	0.017034	0	0	0
Novel_003153	XLOC_03353.	transcript	0.009985	0.198701	0	0.002748	0	0	0.02754	0	0.020234
Novel_003154	XLOC_03353.	transcript	0.01653	0.838547	0.076279	0.025493	0.058928	0.071427	0.238136	0.085491	0.029353
Novel_003155	XLOC_03353.	transcript	0.028251	0.488718	0.089602	0.047355	0	0	0	0	0.066962
Novel_003156	XLOC_03353.	transcript	0.137101	1.030904	0	0.009336	0.206528	0	0.133563	0.072414	0.06923
Novel_003157	XLOC_03353.	transcript	0.203585	0.607512	0.127031	0	0.30762	0.267561	0.393784	0.002689	0.156233
Novel_003158	XLOC_03353.	transcript	0	1.523448	0.324584	0	0	0	0.206979	0.079469	0.342168
Novel_003159	XLOC_03353.	transcript	0	2.305989	0	0	0	0.022083	0	0	0
Novel_003160	XLOC_03353.	transcript	0	0.647773	0	0	0	0	0	0	0
Novel_003161	XLOC_03353.	transcript	0.026543	1.393436	0.024276	0.008744	0.022395	0.094889	0.049602	0.015095	0.025356
Novel_003162	XLOC_03354.	transcript	0.038974	0.437177	0	0	0	0.14647	0.012664	0	0.019934
Novel_003163	XLOC_03355.	transcript	0	0.456856	0	0	0	0	0.08756	0.054939	0
Novel_003164	XLOC_03359.	transcript	0.040705	0.021284	0.522447	0.66789	0.223799	0.13214	0.051194	0.139207	0.093415
Novel_003165	XLOC_03360.	transcript	0.416573	0.262054	0.180164	0.172909	0.312145	0.508231	0.351653	0.514408	0.250115
Novel_003166	XLOC_03360.	transcript	0.069653	0.126886	0.263428	0.011675	0.099736	0.425389	0.139035	0.206442	0.099057
Novel_003167	XLOC_03360.	transcript	0	0	0	0.33228	0	0	0.199413	0	0
Novel_003168	XLOC_03360.	transcript	0	0	0	0.22192	0.082689	0.119304	0.093379	0.234214	0.220867
Novel_003169	XLOC_03364.	transcript	0.136277	0.474043	0.228865	0.049758	0.025182	0.139058	0.364594	0.192404	1.270884
Novel_003170	XLOC_03364.	transcript	0	0	0	0	0	0	0	0	0.101342
Novel_003171	XLOC_03365.	transcript	0.051164	0	0	0.106218	0	0	0	0.141619	0.456647
Novel_003172	XLOC_03365.	transcript	0.018453	0.107057	0.09761	0	0.015138	0	0.082806	0	0.942602
Novel_003173	XLOC_03366.	transcript	0.460011	0	0	0	0	0	0.000138	0	0
Novel_003174	XLOC_03366.	transcript	0.205321	0.762988	0.645345	0.558495	0.39507	1.142685	0.446061	0.370071	0.483542
Novel_003175	XLOC_03366.	transcript	0.015071	0.046905	0	0	0	0.007249	0.088898	0.034126	0.012762
Novel_003176	XLOC_03366.	transcript	0.129236	0.122161	0.028395	0.12277	0.04326	0.127493	0.074507	0.112889	0.057394
Novel_003177	XLOC_03366.	transcript	0	0	0	0	0	0	0	0	0
Novel_003178	XLOC_03366.	transcript	0	0.04104	0	0	0	0	0.041429	0.003299	0.009785
Novel_003179	XLOC_03366.	transcript	0.024317	0.738338	0.537569	0.42577	0.583626	0.568687	0.727059	0.557662	0.459344
Novel_003180	XLOC_03366.	transcript	0	0	0	0	0	0	0	0	0

Novel_003181	XLOC_03366.	transcript	0.462054	0.002024	0	0	0	0	0	0	0
Novel_003182	XLOC_03366.	transcript	0.301124	0	0.039398	0	0	0	0	0.051856	0
Novel_003183	XLOC_03366.	transcript	0.105362	0.567939	0.17268	0.02532	0.084373	0.082676	0.152472	0.27318	0.152049
Novel_003184	XLOC_03366.	transcript	0	0.386069	0	0.080329	0.123228	0.125725	0.252543	0	0.111286
Novel_003185	XLOC_03367.	transcript	0.089465	0	0.102069	0	0	0	0	0	0
Novel_003186	XLOC_03368.	transcript	0	0	0	0	0	0.000354	0	0	0
Novel_003187	XLOC_03368.	transcript	0.408348	0.531697	0.611364	0.447308	0.620834	0.600982	0.329275	0.343531	0.621455
Novel_003188	XLOC_03368.	transcript	0.119213	0	0.027156	0.038779	0	0	0.447742	0.302552	0
Novel_003189	XLOC_03373.	transcript	0.212496	0	0.197316	0.180281	0.153935	0.103146	0.32118	0.14836	0.044079
Novel_003190	XLOC_03375.	transcript	0	0.47028	0	0	0	0	0	0	0
Novel_003191	XLOC_03375.	transcript	0.479197	0.730186	0.571928	0.61953	0.51996	0.605476	0.542869	0.82451	0.3353
Novel_003192	XLOC_03378.	transcript	0	0	0	0	0	0	0	0	2.879044
Novel_003193	XLOC_03378.	transcript	0.199446	0	0	0	0	0.978712	0	0	0.057783
Novel_003194	XLOC_03378.	transcript	0	0	0	0	0	0	0	0	1.288567
Novel_003195	XLOC_03378.	transcript	3.598908	1.58279	0.007881	1.926814	3.602812	1.074439	4.267304	0.006682	0.252652
Novel_003196	XLOC_03378.	transcript	2.226749	0.002766	4.577236	0.0363	0.213481	0.964297	0.444643	4.769221	0.068282
Novel_003197	XLOC_03378.	transcript	0	0	0	0	2.919792	2.876561	1.66504	0	0
Novel_003198	XLOC_03378.	transcript	0.146139	0.062244	0.602097	0.187064	0.388163	0	0.595863	0.774104	2.242982
Novel_003199	XLOC_03378.	transcript	0	0	0	3.321222	0	0	0	0	0
Novel_003200	XLOC_03379.	transcript	0.072791	0.390386	0.114774	0.064829	0.052484	0.079929	0.054421	0.115313	0.127544
Novel_003201	XLOC_03379.	transcript	0	0.001508	0	0.18451	0	0	0	0.068021	0.156975
Novel_003202	XLOC_03379.	transcript	0.104387	0.279757	0.03498	0	0.030789	0.149376	0.11637	0.015833	0
Novel_003203	XLOC_03382.	transcript	1.35378	1.321188	1.279112	1.312508	1.418893	1.603649	1.317461	1.416065	1.378026
Novel_003204	XLOC_03391.	transcript	0.03159	0.029555	0.44794	0.229603	0.058213	0.259481	0.267353	0.26459	0
Novel_003205	XLOC_03391.	transcript	0.018628	0	0.532347	0.360318	0.238954	0.337528	0.524902	0.531283	0.269228
Novel_003206	XLOC_03391.	transcript	0.564986	0.368501	0	0	0.34351	0	0	0	0.008762
Novel_003207	XLOC_03391.	transcript	4.702363	1.836762	2.743169	2.728441	3.784965	3.432687	5.567205	2.648811	2.736885
Novel_003208	XLOC_03393.	transcript	0.231338	0.322734	0.248944	0.309024	0.400033	0.383159	0.355552	0.481191	0.338012
Novel_003209	XLOC_03396.	transcript	0.719648	0.716042	0.716872	0.502722	0.592706	0.569937	0.748733	0.528713	0.247467
Novel_003210	XLOC_03396.	transcript	0.018374	0	0	0	0.129118	0	0	0.165087	0.304679
Novel_003211	XLOC_03396.	transcript	0	0.034018	0.098362	0	0.03172	0.052385	0.05449	0.109534	0
Novel_003212	XLOC_03397.	transcript	0.786405	0.046908	0.093321	0.955874	0.921706	0.956319	0.838543	0.75345	0.254944
Novel_003213	XLOC_03397.	transcript	0.176229	0.006456	0.897516	0	0	0	0	0.307725	0.418622
Novel_003214	XLOC_03397.	transcript	0.169131	0.108432	0.461814	0.563755	0.49982	0.738498	0.156256	0.312948	0
Novel_003215	XLOC_03398.	transcript	0.168105	0.433541	0.103799	0.083664	0.119672	0.123172	0.34753	0.081218	0.42871
Novel_003216	XLOC_03398.	transcript	0	0.555919	0	0	0	0	0	0	0.263175
Novel_003217	XLOC_03399.	transcript	0.392491	0.073639	0.769189	0.563148	0.472885	0.435186	0.972961	0.738504	0.388112
Novel_003218	XLOC_03400.	transcript	0	0.065438	0.034998	0.017332	0.036513	0	0.126291	0.039746	0.038445

Novel_003219	XLOC_03403.	transcript	0.480837	0.606598	0.863344	0.856739	0.865736	0.948092	1.214066	0.935776	0.693929
Novel_003220	XLOC_03403.	transcript	0	0	0	0	0	0	0	0	0.00477
Novel_003221	XLOC_03410.	transcript	0.000209	0	0	0	0.004599	0.186198	0	0.371294	0
Novel_003222	XLOC_03410.	transcript	0.000209	0	0	0.315476	0	4.00E-06	0	0	0
Novel_003223	XLOC_03410.	transcript	0.046909	0.100211	0.004587	2.40E-05	0.042577	1.30E-05	0.715306	0	0.125758
Novel_003224	XLOC_03410.	transcript	0.00622	0	0	0.013402	0.041575	0	0.013459	0	0
Novel_003225	XLOC_03410.	transcript	0.040519	0.317605	0	0	0.016106	0.14619	0	0.02773	0
Novel_003226	XLOC_03410.	transcript	0.021259	0	0	0.002819	0	0	0.089526	0.045947	0
Novel_003227	XLOC_03410.	transcript	0.147876	0.041873	0	0.001541	9.50E-05	0.163182	0.000296	0	0
Novel_003228	XLOC_03410.	transcript	0.139728	0.050157	0	0.0948	0.060438	0	0	0.028949	0
Novel_003229	XLOC_03410.	transcript	0.004354	0.022628	0.01625	0.006341	0.242391	0.429975	0.002212	0.003094	0.50685
Novel_003230	XLOC_03410.	transcript	0	0	0.332207	0.062741	0	0	0.014446	0	0
Novel_003231	XLOC_03410.	transcript	0	0.000544	0	0	0	0	0	0.175913	0
Novel_003232	XLOC_03410.	transcript	0	0.020509	0.48597	0.012237	0	0.043522	0.033838	0.00861	0.00139
Novel_003233	XLOC_03410.	transcript	0.040534	0	0	0	0.008325	0	0	0.000144	0
Novel_003234	XLOC_03410.	transcript	0.49382	0.260484	0.375694	0.396718	0.354548	0.72403	0.43119	0.119582	0.075248
Novel_003235	XLOC_03410.	transcript	0.032455	0.100049	0.035796	0.112244	0.05778	0.040896	0.105931	0	0.040171
Novel_003236	XLOC_03410.	transcript	0.409348	0	0.558712	0	0.485382	0.205844	0	0	0.241642
Novel_003237	XLOC_03410.	transcript	0	0.662166	0	0	0	0	0	0	0
Novel_003238	XLOC_03410.	transcript	1.426716	0.616437	0	0.830365	0.90121	0.736908	1.875011	1.238251	0.409546
Novel_003239	XLOC_03413.	transcript	0.242028	0	0.076942	0.036381	0.098743	0.156162	0.22775	0.274044	0.103496
Novel_003240	XLOC_03414.	transcript	0.17533	0	0	0.167064	0	0.018148	0.011475	0	0
Novel_003241	XLOC_03414.	transcript	0.380422	0	0	0.013111	0	0	0	0	0.047607
Novel_003242	XLOC_03414.	transcript	0	0.030281	0.045628	0	0.004326	0.007237	0.006659	0.039534	0.106256
Novel_003243	XLOC_03414.	transcript	0	0	0	0	0	0.07055	0.099296	0	0
Novel_003244	XLOC_03414.	transcript	0	0.009178	0	0	0	0.025286	0.02022	0.114341	0
Novel_003245	XLOC_03414.	transcript	0.005111	0	0	0	0.081522	0.011439	0.01454	0	0
Novel_003246	XLOC_03414.	transcript	0.134998	0.011602	0.617171	0.312331	0.387434	0.829864	0.600891	0.537642	0.324162
Novel_003247	XLOC_03414.	transcript	0.520196	0.477032	0.391437	0.685469	0.272841	0.358207	0.27457	0.450732	0.303033
Novel_003248	XLOC_03417.	transcript	0.267203	0.543519	0.679058	0.522141	0.344591	1.325354	0.464237	0.514073	0.151435
Novel_003249	XLOC_03418.	transcript	0	1.034784	0	0	0	0	0	0	0.610069
Novel_003250	XLOC_03418.	transcript	0	0.17914	0.016714	0.040614	0.044272	0	0	0.114306	0.07235
Novel_003251	XLOC_03418.	transcript	0	0.167664	0.023286	0	0	0	0.035553	0	0
Novel_003252	XLOC_03418.	transcript	0	0	0	0	0	0	0	0	0
Novel_003253	XLOC_03418.	transcript	0	0	0	0	0.001327	0	0	0	0
Novel_003254	XLOC_03418.	transcript	0.200297	0.012832	0.157221	0.071429	0.068937	0.013653	0.250325	0.074267	0
Novel_003255	XLOC_03418.	transcript	0	0.007565	0	0	0	0	0	0.293193	0
Novel_003256	XLOC_03418.	transcript	0	0.384905	0	0	0	0.011808	0.004538	0.002802	0

Novel_003257	XLOC_03418.	transcript	0	0	0	0	0.070658	0	0	0	0.06993
Novel_003258	XLOC_03418.	transcript	0	0.018598	0.060737	0.157657	0.09504	0.254989	0.047731	0	0.106398
Novel_003259	XLOC_03420.	transcript	0	0	0	0	0.40907	0.007706	0.041965	0	0
Novel_003260	XLOC_03420.	transcript	0.732525	1.057306	1.118053	1.255083	0.687018	1.35673	1.112952	1.660354	1.127417
Novel_003261	XLOC_03431.	transcript	0.828202	0	1.195107	6.646283	4.835585	0.957838	1.404067	2.380285	0.572955
Novel_003262	XLOC_03431.	transcript	0.015324	0.003874	0.255961	3.886855	1.704442	1.283878	2.950223	1.233268	0.005522
Novel_003263	XLOC_03433.	transcript	0.323615	0.03819	0.132821	0.198746	0.19034	0.23191	0.428551	0.234373	0.216902
Novel_003264	XLOC_03433.	transcript	0.150265	0	0	0.096322	0.153446	0	0.125242	0.283267	0.099084
Novel_003265	XLOC_03442.	transcript	0	0.045544	0	0	0	0	0	0	0.047269
Novel_003266	XLOC_03442.	transcript	0.191426	0.106435	0.031238	0.519105	0.735888	0.012901	0.784436	1.242819	0.165677
Novel_003267	XLOC_03442.	transcript	1.663284	0.456436	0.82223	0.015048	0	0.629166	0.235934	0.009326	0.371074
Novel_003268	XLOC_03443.	transcript	0	0	0.116606	0	0	0	0	0	0
Novel_003269	XLOC_03444.	transcript	2.734566	3.238801	3.649174	2.922549	3.086147	2.533096	3.043686	3.089963	3.696842
Novel_003270	XLOC_03445.	transcript	0.220108	0.230763	0.052957	0.550008	0.38079	0.227412	0.232515	0.304577	0.370356
Novel_003271	XLOC_03445.	transcript	9.736465	30.06077	20.02691	25.04958	20.25692	28.90372	15.69396	16.39291	22.70447
Novel_003272	XLOC_03446.	transcript	0.023628	1.292842	0.010937	0.038729	0.044216	0.07372	0.203851	0.083199	0.208114
Novel_003273	XLOC_03446.	transcript	0	0	0.577489	0.103163	0.066896	0	0	0.201445	0.599611
Novel_003274	XLOC_03447.	transcript	0.814635	0.634378	0	0	0.089016	0.515972	0.738149	0.07081	0.020226
Novel_003275	XLOC_03447.	transcript	0	0.980081	1.158938	1.527244	0.822946	0.617311	0.821887	1.525994	0.848747
Novel_003276	XLOC_03447.	transcript	0.028036	0.003353	0	0	0	0	0.048684	0.002965	0
Novel_003277	XLOC_03447.	transcript	0.164337	1.333829	0.419987	0.081594	0.077943	0.254032	0.248588	0.048976	0.083204
Novel_003278	XLOC_03447.	transcript	0.021111	0.257354	0.104382	0.134655	0.191477	0.265164	0.097255	0.042573	0.038754
Novel_003279	XLOC_03447.	transcript	0	0.317211	0	0.087016	0.071638	0.102486	0	0.009411	0
Novel_003280	XLOC_03447.	transcript	0	0	0	0.073135	0	0	0	0	0
Novel_003281	XLOC_03449.	transcript	0.055132	0	0.090152	0.047885	0.116483	0.101158	0.117706	0.065043	0
Novel_003282	XLOC_03449.	transcript	6.301756	7.595963	5.684344	9.144169	6.207111	4.079543	9.152866	6.851672	1.898142
Novel_003283	XLOC_03457.	transcript	0.407428	0	0.013755	1.002632	0.30787	0.042385	0.437512	0.085076	0.241542
Novel_003284	XLOC_03457.	transcript	0.196669	0.052976	0	0	0	0	0	0	0.434745
Novel_003285	XLOC_03457.	transcript	0	0.013964	0.21382	0.002417	0	0	0	0	0.001703
Novel_003286	XLOC_03457.	transcript	0	0.000969	0	0.00242	0.349203	0	0.244323	0.330277	0
Novel_003287	XLOC_03457.	transcript	0.004192	0.343671	0.491194	0.010956	0.009055	0.76998	0.004217	0.006503	0.036961
Novel_003288	XLOC_03457.	transcript	0.420954	0	0	0.833934	0.722456	0.092086	0.964002	0.286022	0.362151
Novel_003289	XLOC_03458.	transcript	0.189324	0.002671	0.004039	0.151379	0.203293	0.153507	0.335595	0.100947	0.052481
Novel_003290	XLOC_03458.	transcript	0.479213	0.239133	0.210617	0.223577	0.22391	0.424046	0.254871	0.253864	0.214071
Novel_003291	XLOC_03459.	transcript	0.193204	0.0396	0.261763	0.200737	0.200169	0.142708	0.097234	0.143685	0.075839
Novel_003292	XLOC_03469.	transcript	0.005026	0	0.000432	0.030583	0.09708	0.130522	0.047233	0.026119	0.077885
Novel_003293	XLOC_03469.	transcript	0.041742	0.005206	0.047293	0.105731	0.053941	0.469531	0	0	0
Novel_003294	XLOC_03469.	transcript	0.788994	0.294388	0.570159	0.718598	0.699689	1.183755	0.92	0.797883	0.684591

Novel_003295	XLOC_03472.	transcript	0.25573	0.617859	0.166355	0.285611	0.212855	0.208118	0.427661	0.151342	0.259367
Novel_003296	XLOC_03472.	transcript	0	0	0.026718	0.020758	0.168723	0.066368	0	0	0
Novel_003297	XLOC_03472.	transcript	0.192978	0.732126	0	0	0.01878	0	0.006503	0.228931	0
Novel_003298	XLOC_03473.	transcript	0.122906	0.00214	0.153868	0.156943	0.15956	0.35635	0.1706	0.25016	0.123196
Novel_003299	XLOC_03476.	transcript	0.09151	0.002273	0.13984	0.085077	0.096528	0.135839	0.0933	0.201414	0.037486
Novel_003300	XLOC_03489.	transcript	0	0	0	0.015841	0.331379	0	0	0.141121	0
Novel_003301	XLOC_03492.	transcript	0	0	0	0	0	0.148841	0.036668	0.107708	0
Novel_003302	XLOC_03496.	transcript	0.081669	0.159634	0.303508	0.154782	0.299918	0.138526	0.529602	0.097086	0.00863
Novel_003303	XLOC_03497.	transcript	0	0	0.328579	0	0	0	0.012475	0	0
Novel_003304	XLOC_03498.	transcript	0.189755	0.034507	0.224194	0.472901	0.028389	0.199931	0.155001	0.30073	0.098605
Novel_003305	XLOC_03500.	transcript	0	0.207217	0	0	0.265526	0.247439	0.502265	0	0
Novel_003306	XLOC_03500.	transcript	1.751556	0.840406	2.10981	0.328297	1.335483	1.561763	1.543025	2.301816	1.985641
Novel_003307	XLOC_03500.	transcript	0	0	0	2.063239	0	0	0	0	0
Novel_003308	XLOC_03500.	transcript	0.688072	0	0	0	0	0	0	0	0
Novel_003309	XLOC_03506.	transcript	0.273747	0.168488	0.319461	0.312337	0.290353	0.305367	0.325871	0.447342	0.125849
Novel_003310	XLOC_03506.	transcript	0	0	0	0	0	0	0	0	0.572784
Novel_003311	XLOC_03511.	transcript	0.059654	0.535131	0.29962	0.149451	0.108401	0.352194	0.136044	0.121507	0.109246
Novel_003312	XLOC_03513.	transcript	0.342096	0.376561	0.197691	0.106914	0.184893	0.029291	0.459272	0.244454	0.337386
Novel_003313	XLOC_03514.	transcript	0	0.236399	0.087888	0.003251	0	0.055723	0.076128	0.047332	0
Novel_003314	XLOC_03522.	transcript	0	0	0.442476	0	0	0.11054	0	0.242432	0
Novel_003315	XLOC_03522.	transcript	0	0	0	0	0	0	0	0.235348	0
Novel_003316	XLOC_03522.	transcript	0.594534	0.656335	0	0.375128	0.39386	0.444738	0.576042	0	0.330336
Novel_003317	XLOC_03523.	transcript	0	0.047501	0	0.118155	0.051101	0.141864	0.175265	0.063047	0.059495
Novel_003318	XLOC_03523.	transcript	0.467419	0	0.247558	0.006073	0.073573	0.131461	0.231077	0.07275	0.044842
Novel_003319	XLOC_03523.	transcript	0.002724	0.032309	0.005244	0.064977	0.09606	0.080043	0.092735	0.122385	0
Novel_003320	XLOC_03523.	transcript	0.006693	0.526641	0	0.259385	0.417186	0.952682	0.626253	0.297402	0
Novel_003321	XLOC_03523.	transcript	0.013228	0.712602	0.29256	0.135157	0.084134	9.80E-05	0.132106	0.217764	0.281874
Novel_003322	XLOC_03523.	transcript	0.367489	0	0	0	0	0	0	0	0
Novel_003323	XLOC_03523.	transcript	1.405316	0.882385	0.799827	0.572686	1.094836	0.830912	0.665793	0.119282	0.756576
Novel_003324	XLOC_03523.	transcript	0	0	0	0	0	0.402145	0	0	0
Novel_003325	XLOC_03523.	transcript	0	0	0	0.593173	0	0.343606	0.567771	0.179811	0
Novel_003326	XLOC_03529.	transcript	0.233689	0.18089	0.677049	0.913005	0.589074	0.522129	0.605457	0.516045	0.488428
Novel_003327	XLOC_03538.	transcript	0.374601	1.209935	0.251182	0.267445	0.39985	0.344832	0.44125	0.368257	0.271484
Novel_003328	XLOC_03538.	transcript	0.073696	0.559899	0.235719	0.022813	0.133271	0.136642	0.209237	0.323843	0.384334
Novel_003329	XLOC_03546.	transcript	0	0.027813	0.209747	0.074018	0.072712	0.04654	0.136118	0.066034	0.274247
Novel_003330	XLOC_03546.	transcript	0.510412	0.226738	0.40968	0.331683	0.457156	0.546733	0.768013	0.47068	0.045765
Novel_003331	XLOC_03549.	transcript	0.257167	0.267459	0	0.645505	0	0.537902	0	0.392565	0.442985
Novel_003332	XLOC_03549.	transcript	0	0	0.165185	0	0.469039	0.338941	0.368516	0.414297	0

Novel_003333	XLOC_03549.	transcript	0	0	0.436743	0	0	0.00143	0	0	0
Novel_003334	XLOC_03549.	transcript	0	0	0.192744	0.00645	0	0	0	0	0.194289
Novel_003335	XLOC_03549.	transcript	0	0.067261	0.185659	0	0	0	0.387418	0.001802	0
Novel_003336	XLOC_03572.	transcript	1.344785	1.045407	0.577647	0.476277	0.504667	0.444102	0.768658	0.584793	0.371433
Novel_003337	XLOC_03574.	transcript	0.039317	0.021702	0.068203	0.035179	0.095678	0	0.12358	0.030749	0.539175
Novel_003338	XLOC_03576.	transcript	0.188313	0	0.077663	0.072584	0.065126	0	0.100661	0.041291	0.08211
Novel_003339	XLOC_03579.	transcript	0.313262	0	0.138924	0.226617	0.216399	0.20455	0.489676	0.319888	0.096958
Novel_003340	XLOC_03580.	transcript	0	0.96393	0.342808	0.171448	0.134479	0	0.089996	0.034448	0.253084
Novel_003341	XLOC_03580.	transcript	0.314369	1.803912	0.185855	0.054486	0.09571	0.067668	0.629554	0.198311	0.15925
Novel_003342	XLOC_03581.	transcript	0.269333	1.243706	0	0.22418	0.162728	0.070469	0.620188	0.022812	0
Novel_003343	XLOC_03581.	transcript	0	0.537118	0	0.020984	0.045141	0.06367	0.202218	0.094255	0.034948
Novel_003344	XLOC_03581.	transcript	0	3.166629	0.246103	0	0	0.843025	0	0	0
Novel_003345	XLOC_03581.	transcript	0	0.756574	0.202443	0	0	0	0	0	0
Novel_003346	XLOC_03581.	transcript	0	2.714192	0	0	0	0	0	0	0
Novel_003347	XLOC_03581.	transcript	0	0.830076	0	0	0	0	0.07381	0	0.047364
Novel_003348	XLOC_03581.	transcript	0.770076	0	0	0	0	0	0	0	0
Novel_003349	XLOC_03581.	transcript	0	0	0	0	0	0	1.933285	0	0
Novel_003350	XLOC_03581.	transcript	0	0	0	0	0	0	0	0.540362	0
Novel_003351	XLOC_03581.	transcript	0	0.018938	0.023943	0.815203	0.972036	0.006568	0.285205	0.018267	0.897765
Novel_003352	XLOC_03581.	transcript	0.023284	0	0.006553	0	0	0	0	0	0
Novel_003353	XLOC_03591.	transcript	0.248566	0.396518	0.221506	0.129139	0.191692	0	0.276729	0.131544	0
Novel_003354	XLOC_03591.	transcript	0	0	0	0	0	0.221969	0	0.100706	0.168228
Novel_003355	XLOC_03592.	transcript	1.41363	1.691627	0.841478	0.697798	0.728739	1.124709	1.000156	0.635108	0.733401
Novel_003356	XLOC_03593.	transcript	0	0.07967	0	0	0	0.030121	0	0	0
Novel_003357	XLOC_03593.	transcript	0	0.282586	0	0	0	0	0	0	0
Novel_003358	XLOC_03593.	transcript	0	0.376869	0	0	0	0	0	0	0
Novel_003359	XLOC_03593.	transcript	0	0.340237	0.010134	0.04328	0.090011	0.018746	0.095429	0.044759	0.016954
Novel_003360	XLOC_03616.	transcript	1.354453	1.653866	1.812234	1.921324	1.71287	2.086029	1.358163	1.90393	1.646747
Novel_003361	XLOC_03618.	transcript	0.20639	0.02547	0.134923	0.105873	0.1306	0.10588	0.191418	0.106924	0.048339
Novel_003362	XLOC_03619.	transcript	0.684323	1.459238	0.657397	0.533207	0.662884	0.537301	1.032942	0.751538	0.79341
Novel_003363	XLOC_03619.	transcript	0	0.751109	0.016455	0.024089	0.047757	0.031436	0.074286	0.12714	0.125853
Novel_003364	XLOC_03621.	transcript	0	0	0	0	0.282483	0	0	0	0
Novel_003365	XLOC_03621.	transcript	0.545015	0.074507	0.322425	0.200484	0	0.342128	0.5428	0.374105	0.191499
Novel_003366	XLOC_03621.	transcript	0	0	0.032606	0	0.001987	0	0	0.014285	0
Novel_003367	XLOC_03634.	transcript	0.550839	0.047468	0.382211	0.901671	0.541878	0.481186	0.621501	0.713231	0.262382
Novel_003368	XLOC_03635.	transcript	0.644156	0.019338	0.26057	0.182335	0.412173	0.164406	0.435682	0.187201	0.235756
Novel_003369	XLOC_03635.	transcript	0.438471	0.071221	0.076369	0.088294	0.219455	0.068576	0.817799	0.184387	0.268765
Novel_003370	XLOC_03635.	transcript	0.402581	0.035844	0.395169	0.293816	0.549401	0.273748	0.329925	0.022281	0.223137

Novel_003371	XLOC_03635.	transcript	0.217979	0.031671	0.131803	0.136611	0.151893	0.015417	0.164489	0.327193	0.08356
Novel_003372	XLOC_03635.	transcript	0.848276	0	0.047651	0.178835	0.111088	0.003645	0.175986	0.117304	0.248401
Novel_003373	XLOC_03635.	transcript	0.337499	0	0.003264	0.043713	0.013843	0	0.011689	0.007304	0.010761
Novel_003374	XLOC_03635.	transcript	0.162674	0	0	0	0.08119	0.222548	0.206948	0	0.068067
Novel_003375	XLOC_03635.	transcript	0.476234	0	0.482527	0	0.101988	0	0.105141	0.01925	0
Novel_003376	XLOC_03635.	transcript	1.103824	0	0.008559	0.001492	0.00559	0	0.436127	0.40732	0
Novel_003377	XLOC_03635.	transcript	0.522217	0.010284	0.193906	0.051316	0.311746	0.126521	0.359715	0.160795	0.223279
Novel_003378	XLOC_03635.	transcript	0.948914	0.032734	0.27318	0.158663	0.205193	0.204198	0.431028	0.291903	0.173469
Novel_003379	XLOC_03635.	transcript	0.324949	0.017696	0.032788	0.040725	0.071666	0	0.24881	0.068465	0.016162
Novel_003380	XLOC_03635.	transcript	0.07454	0	0	0	0	0	0	0	0.000918
Novel_003381	XLOC_03635.	transcript	0.073252	0	0.001682	0.058438	0.161066	0	0.090344	0.001163	0
Novel_003382	XLOC_03635.	transcript	0.114002	0	0.014157	0.041275	0.307031	0.008626	0.078362	0.056748	0.004624
Novel_003383	XLOC_03635.	transcript	0.450569	0	0.187789	0	0	0	0	0.000326	0
Novel_003384	XLOC_03635.	transcript	0.606221	0	0.217457	0.388056	0.200223	0.212788	0.404927	0.216103	0
Novel_003385	XLOC_03635.	transcript	0.757981	0.010238	0.325138	0.254433	0.395391	0.158402	0.562552	0.311003	0.33832
Novel_003386	XLOC_03636.	transcript	0.768075	0.022306	0.221564	0.299757	0.34933	0.239198	0.565568	0.311479	0.202495
Novel_003387	XLOC_03644.	transcript	0.319292	0.036128	0.268329	0.224955	0.353928	0.21196	0.349786	0.171137	0.274419
Novel_003388	XLOC_03644.	transcript	0	0	0	0	0	0	0	0.009834	0.001411
Novel_003389	XLOC_03644.	transcript	0.342616	0.016724	0.053189	0.012661	0.014292	0	0.025656	0.335957	0.059878
Novel_003390	XLOC_03644.	transcript	0	0	0	0	0.00464	0.028977	0.00472	0	0.008421
Novel_003391	XLOC_03644.	transcript	0.13943	0	0	0.153902	0.017208	0	0.358765	0.09911	0
Novel_003392	XLOC_03645.	transcript	0.121817	0.366635	0.069351	0.041418	0	0.297826	0.05544	0.13968	0.117134
Novel_003393	XLOC_03645.	transcript	0.11619	0.341271	0.017034	0.057058	0.12081	0.14289	0.076375	0.058891	0
Novel_003394	XLOC_03646.	transcript	0.035531	0.004195	0.079403	0.041315	0.064517	0	0.034279	0.058512	0
Novel_003395	XLOC_03646.	transcript	0.254221	0.288018	0.216206	0.181545	0.274816	0.173274	0.329646	0.131749	0.100371
Novel_003396	XLOC_03649.	transcript	0.13421	0.013846	0.174964	0.197007	0.096197	0.189025	0.051984	0.134517	0.11355
Novel_003397	XLOC_03651.	transcript	0.031056	0.40779	0.625886	0.24247	0.27892	0.337863	0.072653	0.053583	0
Novel_003398	XLOC_03651.	transcript	0	0.352715	0.016579	0.005722	0.003822	0	0.013097	0	0
Novel_003399	XLOC_03651.	transcript	0.051277	0.01057	0.017248	0.000977	0	0.028745	0.011286	0.010866	0.010994
Novel_003400	XLOC_03651.	transcript	1.22268	0.172625	0.553976	0.366631	0.449781	0.435062	0.882247	0.86395	0.50823
Novel_003401	XLOC_03651.	transcript	0.267327	0	0.374144	0	0	0	0.002857	0	0
Novel_003402	XLOC_03653.	transcript	0.063889	0.013243	0	0	0.079441	0.043281	0	0.116585	0.042608
Novel_003403	XLOC_03653.	transcript	0	0	0.142318	0.345793	0	0	0.056392	0	0
Novel_003404	XLOC_03654.	transcript	0.253547	0	0	0.13421	0	0	0.109027	0.192109	0.28467
Novel_003405	XLOC_03654.	transcript	0.005932	0.150404	0.336896	0	0.179435	0.212049	0.081722	0.070807	0
Novel_003406	XLOC_03655.	transcript	2.232071	1.861577	0.827618	0.42873	1.757992	0.393688	2.874756	0.981586	7.734953
Novel_003407	XLOC_03655.	transcript	0.009911	0.050182	0.093031	0.326503	0.090196	0	0.119411	0.322369	0.037208
Novel_003408	XLOC_03655.	transcript	0.395645	0.434112	0.208181	0.153836	0	0	0.624932	0.395769	0.711897

Novel_003409	XLOC_03655.	transcript	0	0	0	0	0.317311	0.813124	0	0	0
Novel_003410	XLOC_03655.	transcript	0	0	0.551742	0.202422	0.566424	0	0.428144	0.11669	0.445836
Novel_003411	XLOC_03655.	transcript	0.413087	0.058339	0.023803	0.121564	0.042478	0.174504	0.095833	0.325016	0.105144
Novel_003412	XLOC_03655.	transcript	0	0.477597	0	0.272035	0	0.362183	0.354453	0.157409	0
Novel_003413	XLOC_03655.	transcript	0.660663	0.855302	0.311478	0.310398	0.682007	0.339291	0.493963	0.410905	0.297113
Novel_003414	XLOC_03658.	transcript	0.287289	0.151954	0.282184	0.161737	0.441198	0.285193	0.335891	0.286711	0.229566
Novel_003415	XLOC_03672.	transcript	0.610816	0.847638	0.428954	0.342184	0.526129	0.66544	0.727426	0.346688	0.423592
Novel_003416	XLOC_03672.	transcript	0	0	0.285541	0	0.130785	0	0.083346	0.284507	0.034998
Novel_003417	XLOC_03673.	transcript	0.432188	0.000335	0.10344	0.111549	0	0.33116	0.733627	0.092421	0.133344
Novel_003418	XLOC_03673.	transcript	0.698669	5.11269	0.304351	0.205807	0.342746	0.733084	1.446624	0.189041	0.264442
Novel_003419	XLOC_03673.	transcript	0.13087	4.069548	0.065024	0.116204	0.178567	0.393284	0.660962	0.050788	0.118441
Novel_003420	XLOC_03675.	transcript	0.256893	0.057318	0.344665	0.403564	0.386464	0.384296	0.100898	0.368408	0.29902
Novel_003421	XLOC_03675.	transcript	0	0	0	0	0	0	0.489293	0.00116	0.019006
Novel_003422	XLOC_03685.	transcript	0.168731	0.06739	0.088984	0.077019	0.041596	0.122644	0.094412	0.103427	0.028512
Novel_003423	XLOC_03685.	transcript	0	0.205185	4.00E-06	0	0	0	0	0	0.004201
Novel_003424	XLOC_03688.	transcript	0.036205	0	0.613554	0.014998	0.673713	0	0.857643	0.167304	0.706478
Novel_003425	XLOC_03688.	transcript	0	0	0	0	0	0	0.111548	0	0
Novel_003426	XLOC_03688.	transcript	0	0	0	0	0	0	0.01406	0	0
Novel_003427	XLOC_03688.	transcript	0	0.996209	0	0	0	0	0	0	0
Novel_003428	XLOC_03688.	transcript	0.756001	0.379839	0	0.470529	0	0.558335	0.004266	0.709023	0
Novel_003429	XLOC_03697.	transcript	0.444352	0.127164	0.435025	0.412986	0.460367	0.562705	0.7159	0.401107	0.347333
Novel_003430	XLOC_03710.	transcript	0.246496	0.037092	0.317795	0.126435	0.190723	0.060747	0.694175	0.226943	1.603852
Novel_003431	XLOC_03711.	transcript	0.742583	0.127332	0.410446	0.701278	0.511595	0.633587	0.788747	0.597334	0.661065
Novel_003432	XLOC_03721.	transcript	0.010259	0.217937	0	0.002278	0.114792	1.00E-06	0	0.008764	0.001999
Novel_003433	XLOC_03721.	transcript	0.091988	0.045855	0.214677	0.140255	0	0.121514	0.097853	0.115538	0.059524
Novel_003434	XLOC_03721.	transcript	0	0	0	0	0	0.004525	0	0	0
Novel_003435	XLOC_03721.	transcript	0	0	0	0	0.054349	0.001996	0.033065	0.00058	0.000822
Novel_003436	XLOC_03721.	transcript	0.194196	0.598988	0.19644	0.298001	0.227253	0.195055	0.429027	0.280764	0.157578
Novel_003437	XLOC_03721.	transcript	0.326757	0.454803	0.115796	0.084242	0.089816	0.251962	0.326885	0.101542	0.291614
Novel_003438	XLOC_03721.	transcript	0.408981	0.931507	0.523139	0.423097	0.550636	0.553898	0.524365	0.413669	0.602182
Novel_003439	XLOC_03723.	transcript	0.387681	0.486822	0.554969	0.620402	0.429085	0.496297	0.467466	0.617516	0.470425
Novel_003440	XLOC_03723.	transcript	0.151197	0.058485	0.094665	0.081744	0.103204	0.123906	0.114978	0.1297	0.082599
Novel_003441	XLOC_03729.	transcript	0	0.023315	0.118965	0	0.028773	0.452294	0.096108	0.081642	0.085309
Novel_003442	XLOC_03736.	transcript	8.666528	0	28.94975	0	22.94759	2.97304	24.50234	33.03559	0
Novel_003443	XLOC_03736.	transcript	14.38612	29.3968	3.789575	31.94664	1.100259	24.43529	1.035503	0.374366	29.93994
Novel_003444	XLOC_03736.	transcript	0	0	0	0	0	0	0	0	0
Novel_003445	XLOC_03738.	transcript	0.598113	0.06734	1.334226	1.475016	1.229769	1.431931	0.962616	0.510183	0.632481
Novel_003446	XLOC_03738.	transcript	0	0.009652	0	0	0	0	0	0	0.071258

Novel_003447	XLOC_03739.	transcript	0.005386	0.016973	0.052195	0.014106	0.060355	0	0.112383	0.009636	0.01124
Novel_003448	XLOC_03741.	transcript	0	0	0	0	0	0	9.31806	0	0
Novel_003449	XLOC_03741.	transcript	13.691	4.627255	26.92329	32.57544	25.65496	18.00611	7.256265	23.44266	27.23205
Novel_003450	XLOC_03741.	transcript	0	0	0	0	0	0	11.69213	0	0
Novel_003451	XLOC_03741.	transcript	0	0	0	0	0	0	3.275667	0	0
Novel_003452	XLOC_03741.	transcript	0	0	0	0	0	0	0	0	0
Novel_003453	XLOC_03742.	transcript	0.350188	0.012489	0.40294	0.214848	0.73923	0.548706	0.39662	0.174371	0.343369
Novel_003454	XLOC_03745.	transcript	0.357596	0.282211	0.426387	0.428802	0.448718	0.354891	0.538247	0.600769	0.370165
Novel_003455	XLOC_03746.	transcript	0.691893	3.793525	0.150406	0.058127	0.266937	0.20015	0.676613	0.185424	0.798195
Novel_003456	XLOC_03747.	transcript	0.275543	0.243343	0.068846	0.029794	0.016059	0.551007	0.030318	0.08693	0.05677
Novel_003457	XLOC_03749.	transcript	0.339004	0.539349	0.320946	0.085265	0.393182	0.402281	0.349238	0.384981	0.18238
Novel_003458	XLOC_03749.	transcript	0.384095	0.476999	0.501963	0.394795	0.516192	0.429894	0.561972	0.459468	0.386705
Novel_003459	XLOC_03749.	transcript	0.028779	0	0	0.13825	0.102097	0	0	0.018364	0.136759
Novel_003460	XLOC_03750.	transcript	0.949431	1.247129	0.724227	0.844833	0.746967	1.122974	1.102009	0.762367	0.801547
Novel_003461	XLOC_03750.	transcript	0.7593	0.02829	0.013735	0.19137	0.095202	0.485212	0.065527	0.52748	0.262562
Novel_003462	XLOC_03750.	transcript	0	0.826829	0.577854	0.354018	0.064807	0.064584	0.749131	0.193869	0.080797
Novel_003463	XLOC_03750.	transcript	0	0	0.002112	0	0	0	0	0	0
Novel_003464	XLOC_03750.	transcript	0	0	0	0	0.428183	0	0	0	0
Novel_003465	XLOC_03751.	transcript	0.451165	0.099481	0.183955	0.142292	0.231795	0.219918	0.339334	0.244676	0.11935
Novel_003466	XLOC_03767.	transcript	1.120445	0.697491	1.684297	1.91337	2.30278	2.271055	2.455865	1.4874	3.730205
Novel_003467	XLOC_03770.	transcript	0.097047	0.154045	0.08681	0.211484	0.095736	0.053235	0.065003	0.091239	0.041745
Novel_003468	XLOC_03770.	transcript	0.385828	0	0.560162	0.438195	0.664875	0.544196	0.888153	0.669427	0.455005
Novel_003469	XLOC_03774.	transcript	0.030243	0.03843	0.048749	0.064047	0.087948	0.188447	0.040805	0.022123	0.271463
Novel_003470	XLOC_03774.	transcript	0	0	0	0	0	0	0	0	0
Novel_003471	XLOC_03775.	transcript	0.942545	0.929282	0.277264	0.477792	0.6778	0.060048	1.656722	0.743399	0.45464
Novel_003472	XLOC_03775.	transcript	0	0	0.170063	0.38017	0	0.538093	0	0	0.305912
Novel_003473	XLOC_03788.	transcript	1.250411	0.033206	0.853752	0.372658	0.809603	0.869164	1.016176	0.618578	0.418009
Novel_003474	XLOC_03790.	transcript	0.47128	0	0	0	0.071593	0.158596	0.338179	0.248481	0.202431
Novel_003475	XLOC_03790.	transcript	0.012566	0	0	0	0	0.010782	0	0	0
Novel_003476	XLOC_03790.	transcript	0.385001	0.592313	0.563162	0.711472	0.9252	0.723531	0.632832	0.670882	0.28693
Novel_003477	XLOC_03790.	transcript	0.006937	0	0.158929	0.004288	0.002046	0	0	0.007942	0
Novel_003478	XLOC_03791.	transcript	0.080379	0.42884	0.266298	0.205548	0.097208	0.207198	0.174478	0.255657	0.229221
Novel_003479	XLOC_03791.	transcript	0.09026	0.351746	0.052834	0.096112	0.068279	0.021003	0.037376	0.085838	0.044024
Novel_003480	XLOC_03791.	transcript	0	0	0	0	0	0	0.202486	0.144681	0
Novel_003481	XLOC_03791.	transcript	0	0	0	0.126034	0	0	0	0	0
Novel_003482	XLOC_03791.	transcript	0.190457	0	0.280299	0.247601	0.398703	0.553398	0.074623	0	0.268425
Novel_003483	XLOC_03791.	transcript	0	0	0	0	0	0	0	0.241794	0
Novel_003484	XLOC_03791.	transcript	0	0	0.157802	0	0	0	0.050568	0.116482	0

Novel_003485	XLOC_03796.	transcript	0.307454	0.089908	0.283296	0.373582	0.261687	0.420837	0.39653	0.090506	0.401408
Novel_003486	XLOC_03796.	transcript	0	0.37601	0	0	0	0	0	0	0
Novel_003487	XLOC_03796.	transcript	0	0.051104	0	0	0	0	0	0.270145	0
Novel_003488	XLOC_03805.	transcript	0	0	0	0.193653	0	0	0	0	0
Novel_003489	XLOC_03805.	transcript	0.049024	0	0	0	0	0	0.008217	0.006244	0
Novel_003490	XLOC_03805.	transcript	0	0	0	0.116462	0.218735	0	0.353819	0.296062	0.000286
Novel_003491	XLOC_03805.	transcript	0.222728	0.552221	0.316016	0.001225	0.01635	0.284071	0	0	0.272932
Novel_003492	XLOC_03805.	transcript	0	0.05299	0.449677	0.031657	0.107543	0.040067	0.330616	0.13033	0.039356
Novel_003493	XLOC_03808.	transcript	0.356247	0.117764	0.349453	0.303741	0.518697	0.135584	0.598994	0.057374	0.262646
Novel_003494	XLOC_03808.	transcript	0	0	0	0	0.022232	0	0.000394	0	0
Novel_003495	XLOC_03808.	transcript	0	1.088921	0.036233	0.477235	0.007727	0.011639	0.116607	0.943013	0.039995
Novel_003496	XLOC_03809.	transcript	0.454739	0.484559	0.377244	0.353815	0.409882	0.390786	0.701229	0.52174	0.325619
Novel_003497	XLOC_03809.	transcript	0.212455	0.097156	0.136769	0.241739	0.20371	0.095006	0.040443	0.118479	0.367853
Novel_003498	XLOC_03809.	transcript	0	0	0	0	0	0	0	0.029725	0.205788
Novel_003499	XLOC_03809.	transcript	0.289151	0.149933	0.194448	0.036736	0	0.259521	0.340799	0.218199	0
Novel_003500	XLOC_03810.	transcript	0.530578	0.279567	0.490788	0.448263	0.406817	0.215524	0.582964	0.509974	0.374167
Novel_003501	XLOC_03810.	transcript	0.90455	0	1.081237	0.612992	0.937197	1.195943	0.703466	0.698605	1.722257
Novel_003502	XLOC_03810.	transcript	0.244101	0.162199	0.417097	0.094189	0.371502	0.155761	1.084651	0.499056	0.146987
Novel_003503	XLOC_03812.	transcript	4.73104	2.594425	0.956774	1.277685	0.88366	1.261088	1.354359	0.971237	1.061477
Novel_003504	XLOC_03812.	transcript	2.083733	12.20089	4.840388	4.446313	4.852932	4.548804	5.923567	5.15159	5.7864
Novel_003505	XLOC_03812.	transcript	0.385286	0.175843	0.076182	0.292635	0.28035	0.067791	0.229948	0.134923	0.036021
Novel_003506	XLOC_03812.	transcript	0.104932	0.59741	0.320436	0.294017	0.212446	0.367045	0.421265	0.509876	0.368875
Novel_003507	XLOC_03813.	transcript	0	0	0	0.583958	0	1.041183	0.456327	0.104906	0
Novel_003508	XLOC_03814.	transcript	0.015543	0	0.036721	0	0	0.462248	0.361975	0.149618	0.22946
Novel_003509	XLOC_03814.	transcript	0	0	0.1561	0.406974	0	0	0.059853	0	0
Novel_003510	XLOC_03814.	transcript	0.375646	0.044158	0.193624	0.369849	0.472394	0.29527	0	0.100601	0.123574
Novel_003511	XLOC_03814.	transcript	0.500211	0	1.598773	1.250206	1.279305	0.67789	1.045239	1.189289	0.344498
Novel_003512	XLOC_03815.	transcript	0.321524	0.741848	0.023092	0.036973	0.081809	0.115457	0	0.028861	0.133946
Novel_003513	XLOC_03817.	transcript	0.257275	2.042974	0.200033	0.019425	0.120001	0.161158	0.227993	0.140887	0.201056
Novel_003514	XLOC_03824.	transcript	0.11728	0.206158	0	0.245014	0.177077	0.154434	0.368048	0.110241	0.164116
Novel_003515	XLOC_03824.	transcript	0.109713	0	0.215154	8.60E-05	0	0.077853	0.070034	0.15285	0
Novel_003516	XLOC_03824.	transcript	0.328017	0.275144	0.380683	0.118593	0.443796	0.308061	0.695442	0.493004	0.269959
Novel_003517	XLOC_03824.	transcript	0	0	0	0.624693	0.090004	0	0	0.112347	0
Novel_003518	XLOC_03826.	transcript	0.434758	0.415835	0.570663	0.342656	0.442191	0.625344	0.450806	0.458683	0.341441
Novel_003519	XLOC_03826.	transcript	0	2.722362	0	0	0	0	0	0	0
Novel_003520	XLOC_03826.	transcript	2.909293	5.080983	3.82997	4.168489	4.044804	3.968774	3.613048	3.567899	3.153079
Novel_003521	XLOC_03834.	transcript	0.557205	0.06916	0.782459	1.437795	1.33187	1.623322	1.500331	0.948097	0.981015
Novel_003522	XLOC_03834.	transcript	0	0.139366	0	0.002474	0	0	0.174976	0.87508	0.014614

Novel_003523	XLOC_03834.	transcript	0.980528	0.01181	0	0.213889	0.38211	0.276522	0.30972	0.001223	0.278134
Novel_003524	XLOC_03834.	transcript	0	0	0	0	0	0	0	0	0
Novel_003525	XLOC_03834.	transcript	0	0	0.540648	0	0	0	0	0	0
Novel_003526	XLOC_03838.	transcript	0.238972	0.240504	0.134288	0.195729	0.126779	0.161075	0.209346	0.189562	0.180613
Novel_003527	XLOC_03840.	transcript	1.634609	0.578618	0.253612	0.451732	2.390152	1.010647	1.258749	2.165996	1.147342
Novel_003528	XLOC_03840.	transcript	0.105512	2.654015	0.123444	0.196826	0	0.089876	0.926123	0.019665	0.414226
Novel_003529	XLOC_03840.	transcript	2.687875	44.23501	1.478237	0.596354	1.091702	2.196215	0.34948	0.873575	0.487258
Novel_003530	XLOC_03840.	transcript	0.316043	10.80021	0.530361	0.086556	0.290452	0.136008	1.193069	0.915354	1.28449
Novel_003531	XLOC_03840.	transcript	0.21236	6.199199	0.730544	0.294379	0.204406	0.798718	0.936565	0.226387	1.371722
Novel_003532	XLOC_03840.	transcript	0	0	0	0.108854	0	0	0	0	0.407007
Novel_003533	XLOC_03840.	transcript	0.051376	1.864196	0.139725	0	0.122675	0.403771	0.176982	0.051571	0
Novel_003534	XLOC_03840.	transcript	0	0	0	0	0	0	0	0	0
Novel_003535	XLOC_03840.	transcript	0.06267	0.423886	0	0.229098	0.568876	0.205036	0.371494	0.902623	0.270611
Novel_003536	XLOC_03840.	transcript	0.116848	0.61377	0.472548	0.247863	0.697203	0.282748	0.190689	0.594216	0.343756
Novel_003537	XLOC_03840.	transcript	0	0	0	0	0.025773	0	0	0	0
Novel_003538	XLOC_03840.	transcript	0	4.945946	0	0.270268	0.449901	1.347648	0.401044	0	0
Novel_003539	XLOC_03840.	transcript	0	5.594848	0	0	0.431592	0	0.433437	0	0
Novel_003540	XLOC_03840.	transcript	0.138849	1.352635	0	0.147241	0.134891	0.209968	0	0	0
Novel_003541	XLOC_03840.	transcript	0	3.033308	0	0.27766	0.361066	0.278636	0.279175	0	0
Novel_003542	XLOC_03840.	transcript	0	6.5276	0.271755	0	0	0.347441	1.078778	0.353533	0.370192
Novel_003543	XLOC_03840.	transcript	0.228906	1.708026	0	0.194784	0	0.301171	0	0	0
Novel_003544	XLOC_03840.	transcript	0.103614	0.54886	0.152986	0.332913	0.92522	0	0.256209	0.277998	0.284024
Novel_003545	XLOC_03840.	transcript	0.110657	0.987108	0.032599	0.048776	0.02886	0.085061	0.235763	0.181448	0.634249
Novel_003546	XLOC_03840.	transcript	0	0	0	0	0	0	0	0	0
Novel_003547	XLOC_03840.	transcript	0	0	0	0	0	0	0.510989	0	0
Novel_003548	XLOC_03840.	transcript	0	3.363548	0	0	0.01219	0.318895	0	0	0
Novel_003549	XLOC_03840.	transcript	0.201262	2.943679	0.025947	0.034028	0.107651	0.315341	0.243654	0	2.154052
Novel_003550	XLOC_03840.	transcript	0.142662	7.381468	0.160023	0.172294	0.07003	0.31266	0.793154	0.180897	0.993493
Novel_003551	XLOC_03840.	transcript	0.001844	5.744861	0	0.243724	0.179447	0	0.304024	0	0
Novel_003552	XLOC_03840.	transcript	0.004986	0.723355	0	0	0	0.323456	0	0	0
Novel_003553	XLOC_03840.	transcript	0.177597	4.977132	0.183141	0.054203	0.017382	0.332231	0.522802	0.096301	0.380413
Novel_003554	XLOC_03840.	transcript	0	0	0	0	0	0.003339	0.744974	0	0
Novel_003555	XLOC_03840.	transcript	0	0.894657	0	0	0.001431	0	0.007796	0	0
Novel_003556	XLOC_03840.	transcript	0	0.947853	0	0	0.427038	0.416843	0	0	0
Novel_003557	XLOC_03840.	transcript	0	0	0	0	0	0	0	0	0
Novel_003558	XLOC_03840.	transcript	0	0.433665	0.182257	0	0.311395	0.528576	0	0	0
Novel_003559	XLOC_03840.	transcript	0	0	0	0.143774	0	0	0	0	1.131443
Novel_003560	XLOC_03840.	transcript	0	0.244831	0	0.205871	0	0	2.295269	0	0

Novel_003561	XLOC_0384(.	transcript	0	0.776524	0	0	0.116007	0.393138	0	0	0
Novel_003562	XLOC_0384(.	transcript	0	1.198599	0.302513	0	0.107575	0.229641	0.301152	0.142932	0
Novel_003563	XLOC_0384(.	transcript	0	1.55624	0	0	0.180518	0.420444	0	0	0
Novel_003564	XLOC_0384(.	transcript	0	0	0	0	0	0	0	0	0
Novel_003565	XLOC_0384(.	transcript	0	0.393156	0.010187	0	0	0.017807	0	0	0.49722
Novel_003566	XLOC_0384(.	transcript	0	0.581844	0.619665	0	0.102542	0.235445	0.247665	0.109564	0.457233
Novel_003567	XLOC_0384(.	transcript	0.076586	2.579348	0	0.255631	0	0	0.180725	0	0
Novel_003568	XLOC_0384(.	transcript	0	0	0	0	0.095058	0	0.319171	0.101664	0.048645
Novel_003569	XLOC_0384(.	transcript	0	0	0	0.17827	0	0	0	0	0.416112
Novel_003570	XLOC_0384(.	transcript	0.838573	13.87234	0.588083	0.358015	0.528104	1.964345	2.088045	0.581644	2.864818
Novel_003571	XLOC_0384(.	transcript	0.06312	0	0	0	0	0	0.147391	0	0
Novel_003572	XLOC_0384(.	transcript	0.451733	4.554367	0.127269	0	0.122382	0.214145	0	0	0
Novel_003573	XLOC_0384(.	transcript	0	0.877951	0	0	0	0	0.2128	0	1.067746
Novel_003574	XLOC_0384(.	transcript	0	0.611303	0.055624	0.040259	0.064221	0.114807	0.18009	0.121687	0.361876
Novel_003575	XLOC_0384(.	transcript	0.021077	1.169348	0	0.152526	0.092364	0	0.079231	0.091011	0.027015
Novel_003576	XLOC_0384(.	transcript	0.06196	0.569186	0.105797	0.077009	0.065312	0.140968	0	0	0
Novel_003577	XLOC_0384(.	transcript	0	0.858349	0	0	0	0	0	0	0
Novel_003578	XLOC_0384(.	transcript	0.180609	0.789966	0.228865	0.050524	0.077071	0.250044	0.000462	1.40E-05	0.00022
Novel_003579	XLOC_0384(.	transcript	0	0	0.238913	0	0.096522	0.406555	0.233282	0	0
Novel_003580	XLOC_0384(.	transcript	0.10135	0	0.183251	0	0.083327	0.222608	0	0	0
Novel_003581	XLOC_0384(.	transcript	0	0	0.323303	0	0.079913	0.255167	0.219738	0.082947	0.482574
Novel_003582	XLOC_0384(.	transcript	0	0.916007	0	0.289939	0	1.954377	0.436509	0	2.532859
Novel_003583	XLOC_0384(.	transcript	0	0.929986	0.356061	0	0	0	0.419396	0	0.131156
Novel_003584	XLOC_0384(.	transcript	0.063377	1.264016	0.246304	0.208462	0	0.174108	0.680089	0	0.202027
Novel_003585	XLOC_0384(.	transcript	0	1.67059	0.100103	0.098366	0	0	1.009252	0	0.148493
Novel_003586	XLOC_0384(.	transcript	0	0	0	0.069132	0.094161	0	0.09128	0.146582	0.115226
Novel_003587	XLOC_0384(.	transcript	0	0	0	0.052275	0.12387	0	0.834853	0.311481	0.065433
Novel_003588	XLOC_0384(.	transcript	0.269246	5.192293	0.328379	0.090828	0	0.202553	0.511294	0	0.325189
Novel_003589	XLOC_0384(.	transcript	0.001038	0	0	0	0	0	0	0	0
Novel_003590	XLOC_0384(.	transcript	0	0	0	0	0	0	0	0	0.174764
Novel_003591	XLOC_0384(.	transcript	0	0	0	0	0.596977	0	0.366498	0.169792	0
Novel_003592	XLOC_0384(.	transcript	0	2.882078	0.358258	0	0	0.143887	0.295236	0	0.248261
Novel_003593	XLOC_0384(.	transcript	0	0	0	0	0.206251	0	0.059946	0.266157	0.211031
Novel_003594	XLOC_0384(.	transcript	0.130561	0.658539	0.130278	0	0.070315	0.150632	0.115778	0.028911	0.063006
Novel_003595	XLOC_0384(.	transcript	0.527945	0.972658	0	0.022277	0	0.175409	0	0	0
Novel_003596	XLOC_0384(.	transcript	0	0	0	0.028134	0.081149	0	0.067177	0.293268	0.118063
Novel_003597	XLOC_0384(.	transcript	0.871779	0	0	0	0	0.282589	0	0	0
Novel_003598	XLOC_0384(.	transcript	0	4.583808	0.268127	0	0.198218	0.256572	0	0	0

Novel_003599	XLOC_03840.	transcript	0.280413	4.06301	0.272325	0.192147	0.192417	0.493715	0.64776	0.020605	0.174057
Novel_003600	XLOC_03840.	transcript	0.241496	0	0.215471	0	0.126986	0.204513	0.027647	0.36017	0.868221
Novel_003601	XLOC_03840.	transcript	0.157255	3.847159	0.112078	0.069957	0.091877	0.115734	0.036101	0.143871	0.426982
Novel_003602	XLOC_03840.	transcript	0.143232	0.28061	0.198118	0	0.133359	0.179272	0.059481	0.107288	0.466206
Novel_003603	XLOC_03840.	transcript	0.095393	0.296782	0.100086	0	0.088833	0.169779	0.082814	0.094914	0
Novel_003604	XLOC_03841.	transcript	0	3.57901	0	0.527854	0	0.135159	0.584976	0.339363	0.265521
Novel_003605	XLOC_03841.	transcript	0	0.24404	0	0	0	0	0	0	0
Novel_003606	XLOC_03841.	transcript	0.245428	4.175783	0	0.189044	0	0.271017	0.301175	0.200273	0.542389
Novel_003607	XLOC_03841.	transcript	0.1302	1.39579	0	0.128842	0.22137	0	0.295274	0	0.076841
Novel_003608	XLOC_03841.	transcript	0	7.207885	0.229576	0.122772	0.248243	0.032191	0.109982	0.084196	0.07523
Novel_003609	XLOC_03841.	transcript	0.311026	0.956895	0	0.25388	0.057839	0.204006	3.380513	0.1103	0.037812
Novel_003610	XLOC_03842.	transcript	0.044058	0.546753	0	0	0.180249	0.027812	0	0	0
Novel_003611	XLOC_03842.	transcript	0.452332	2.88846	0.122886	0	0.208633	0.198455	0.139829	0.212679	0
Novel_003612	XLOC_03842.	transcript	0.0104	2.725964	0.251375	0	0	0	0.025446	0	0.032103
Novel_003613	XLOC_03842.	transcript	0.529223	12.61395	0.137977	0.402631	0.207732	0.475613	0.526774	0.320897	1.043789
Novel_003614	XLOC_03842.	transcript	0.318761	28.28308	0.419961	0.144154	0.492101	1.69384	0.892569	0.130662	0.741007
Novel_003615	XLOC_03842.	transcript	0.01953	0.586373	0	0.034161	0.039801	0.804981	0	0	0.031861
Novel_003616	XLOC_03842.	transcript	0	17.0939	0	0.232631	0.365003	0.716033	0.472712	0	0.563917
Novel_003617	XLOC_03843.	transcript	0.22612	1.600504	0	0.046475	0.273854	0.023925	0.301916	0.052915	0
Novel_003618	XLOC_03843.	transcript	0	0.412605	0	0	0	0	0.079484	0	0
Novel_003619	XLOC_03843.	transcript	0.083591	0.636866	0	0.062536	0.122473	0	0.290147	0.097187	0.828988
Novel_003620	XLOC_03843.	transcript	0.054239	0.743959	0.05792	0.135613	0.137199	0	0	0	0
Novel_003621	XLOC_03844.	transcript	0	0.003638	0	0	0	0	0	0	0
Novel_003622	XLOC_03844.	transcript	0	0.4081	0	0	0	0.271245	0	0	0
Novel_003623	XLOC_03844.	transcript	0.038105	0.829228	0.033608	0.00289	0	0.071284	0.001355	0.074007	0.136844
Novel_003624	XLOC_03844.	transcript	0.559068	1.538483	0.335826	0.460572	0.092826	0	0.662592	0.155889	0.161339
Novel_003625	XLOC_03844.	transcript	0	0.272163	0	0.042042	0	0.060523	0.30013	0.057441	0
Novel_003626	XLOC_03845.	transcript	0.054425	4.015494	0.077486	0.022807	0.38112	0.126109	0.136223	0.137731	1.04383
Novel_003627	XLOC_03845.	transcript	0	4.020484	0	0.066323	0.091973	0	0.040352	0	0.1492
Novel_003628	XLOC_03845.	transcript	0.119106	1.657362	0.02563	0.019314	0	0.124821	0.074113	0.042108	0.087161
Novel_003629	XLOC_03846.	transcript	0.459451	4.642061	0	0.104144	0	0.301572	0.119756	0.067561	0.347135
Novel_003630	XLOC_03846.	transcript	0.265896	3.907827	0.142915	0.202096	0.279843	0.073223	0.249412	0	0.630962
Novel_003631	XLOC_03846.	transcript	0	1.094469	0.218077	0.071893	0.084214	0	0.059379	0.048621	0.612003
Novel_003632	XLOC_03850.	transcript	0.046875	0.390968	0.364801	0.18638	0.313625	0.410911	0.350622	0.228821	0.20425
Novel_003633	XLOC_03853.	transcript	0.282521	0.329845	0.282564	0.155764	0.258209	0.417343	0.372101	0.360062	0.275279
Novel_003634	XLOC_03853.	transcript	0.216048	0.254679	0.236692	0.208273	1.307338	0.172554	0.581028	0.29752	0.187925
Novel_003635	XLOC_03853.	transcript	0.267504	0	0	0.138219	0	0	0.916057	0.268623	0.179111
Novel_003636	XLOC_03853.	transcript	1.910338	0.014996	2.060869	1.47089	1.05306	1.551311	0.94188	1.673399	1.260726

Novel_003637	XLOC_03854.	transcript	0	0	0	0.600652	0.61496	0	0	0	0
Novel_003638	XLOC_03854.	transcript	0	0	0.906763	0	0	0	0	0	0
Novel_003639	XLOC_03854.	transcript	0.145041	0.258308	0.386145	1.191849	0.460852	0.750025	0.702749	0.347822	0.477556
Novel_003640	XLOC_03854.	transcript	0	0	0	0	0	0	0.010571	0	0
Novel_003641	XLOC_03854.	transcript	0	0	0	0.009929	0.028569	0.041568	0	0	0
Novel_003642	XLOC_03854.	transcript	0.973998	1.344085	0.918229	0.943012	1.050258	1.203634	1.27269	1.361491	1.426753
Novel_003643	XLOC_03855.	transcript	0.311539	0.8839	0.248467	0.137773	0.199408	0.291928	0.451732	0.309774	0.279636
Novel_003644	XLOC_03855.	transcript	0.067325	0.266143	0	0.125721	0	0	0.061566	0.092248	0.212651
Novel_003645	XLOC_03855.	transcript	0.244704	1.013018	0.299472	0.275323	0.231512	0.257306	0.351363	0.272357	0.311552
Novel_003646	XLOC_03856.	transcript	0.20773	0.006688	0.307614	0.307304	0.382008	0.390267	0.796535	0.489158	0.776814
Novel_003647	XLOC_03856.	transcript	0	0	0	0	0.018878	0	0	0.036187	0
Novel_003648	XLOC_03856.	transcript	0.013505	0.088442	0.021832	0.010141	0	0.022579	0.040212	0	0
Novel_003649	XLOC_03858.	transcript	1.006973	1.878156	0.939587	0.737756	0.761775	0	0.262811	0.949696	0
Novel_003650	XLOC_03858.	transcript	0	0.011599	0.102428	0.056188	0.081068	0.083263	0.045989	0.085244	0.045672
Novel_003651	XLOC_03858.	transcript	0.208546	0.037321	0.194883	0.194549	0.249964	0.687559	0.75825	0.209066	0.616791
Novel_003652	XLOC_03858.	transcript	0.019211	0	0	0.081759	0	0	0.009805	0.005738	0
Novel_003653	XLOC_03858.	transcript	0.043795	0.019798	0.239473	0.149934	0.121891	0.10568	0.169109	0.195978	0.160394
Novel_003654	XLOC_03858.	transcript	0	0	0	0.080584	0.031705	0	0.363674	0.227528	0
Novel_003655	XLOC_03858.	transcript	0	0	0	0	0.010613	0.001825	0	0.153021	0.010047
Novel_003656	XLOC_03858.	transcript	0.249698	0.080512	0.248797	0.116048	0.435139	0.306555	0.072745	0.207688	0.187591
Novel_003657	XLOC_03877.	transcript	0.902138	1.771695	1.111581	1.4947	1.174012	1.114114	1.326447	1.249005	0.981555
Novel_003658	XLOC_03887.	transcript	0	3.300143	0.145989	0	0	0	0	0	0
Novel_003659	XLOC_03887.	transcript	0	0.217985	2.09194	0.017109	0.094923	0	0.084029	0.068291	0.116981
Novel_003660	XLOC_03887.	transcript	0.031031	0	0.014694	0.042561	0.045848	0.002241	0	0.001285	0
Novel_003661	XLOC_03887.	transcript	0	0.083067	0.001159	0	0	0.012444	0	0	0
Novel_003662	XLOC_03887.	transcript	0.497007	0	0.453067	0.546104	0.348862	0.68165	0.818748	0.708635	0.934942
Novel_003663	XLOC_03887.	transcript	0.050897	0.180613	0.00752	0	0	0.049202	0.021935	0.022385	0
Novel_003664	XLOC_03888.	transcript	0.006006	0	0	0	0.003778	0	0	0	0
Novel_003665	XLOC_03888.	transcript	0.537502	0.871215	0.635058	0.53319	0.472595	0.419909	1.08977	0.56279	0.686276
Novel_003666	XLOC_03888.	transcript	0.955077	0.710173	0.495395	0.453003	0.732781	0.648572	1.117353	0.825735	0.742439
Novel_003667	XLOC_03889.	transcript	0.222459	0.09695	0.103259	0.147017	0.108119	0.117731	0.124945	0.160785	0.158328
Novel_003668	XLOC_03889.	transcript	0.029732	0	0.097235	0.153497	0.010966	0.050528	0.022338	0	0.070566
Novel_003669	XLOC_03889.	transcript	0.058722	0.058346	0	0.098852	0.168744	0	0.103494	0.241789	0.053733
Novel_003670	XLOC_03889.	transcript	0	0	0.024585	0.059559	0	0.132	0.162842	0.090356	0.072978
Novel_003671	XLOC_03891.	transcript	0.511873	0.140223	0.401254	0.171518	0.131605	0.209595	0.787946	0.415382	0.257693
Novel_003672	XLOC_03898.	transcript	0	0.200419	0	0.199018	0	0	0.417611	0.263956	0
Novel_003673	XLOC_03898.	transcript	0.238821	0.039516	0.281974	0.055467	0.287824	0.230155	0.312836	0.162284	0.479255
Novel_003674	XLOC_03898.	transcript	0.055958	0.052116	0.06629	0.100868	0.114508	0.155248	0.228528	0.173013	0.187195

Novel_003675	XLOC_03898.	transcript	0	0	0	0	0	0	0	0	0
Novel_003676	XLOC_03903.	transcript	0.491466	0	0.395637	0.265708	0.394151	0.168704	0.431667	0.681345	0
Novel_003677	XLOC_03904.	transcript	0	0.00102	0	0	0.398134	0	0.059258	0.001409	0
Novel_003678	XLOC_03904.	transcript	0	0.93795	0	0.007716	0.017921	1.303658	0.351472	0	0.003899
Novel_003679	XLOC_03904.	transcript	0.011533	0.511991	0	0.173525	0.746581	0.924531	0.838262	0.215658	0.710515
Novel_003680	XLOC_03904.	transcript	0.522614	0.215287	0.692049	0.236987	0.51268	0.574848	0.945337	0.532014	0.516969
Novel_003681	XLOC_03910.	transcript	0.261987	0.104387	0.282079	0.069726	0.262954	0.209879	0.154043	0.365704	0.05378
Novel_003682	XLOC_03910.	transcript	0.437492	0.018469	0.254058	0	0.427908	0	0.511472	0.163924	0.152215
Novel_003683	XLOC_03910.	transcript	0	0	0	0	0	0.108526	0	0	0
Novel_003684	XLOC_03911.	transcript	0	0	0.184202	0	0	0.085283	0	0.021191	0
Novel_003685	XLOC_03911.	transcript	0.230314	0.313579	0.283345	0.222957	0.607123	0.358629	0.388442	0.331538	0.610846
Novel_003686	XLOC_03911.	transcript	1.073523	1.904903	1.603804	2.029967	1.19619	1.114595	1.659949	1.305066	1.72783
Novel_003687	XLOC_03911.	transcript	0	0	0	0	0	0	0	0	0
Novel_003688	XLOC_03912.	transcript	0.445194	0.820261	0.258629	0.4349	0.535507	0.363177	0.327503	0.427411	0.411924
Novel_003689	XLOC_03916.	transcript	0	0.260426	0.089377	0	0.215747	0	0	0.141311	0.21801
Novel_003690	XLOC_03916.	transcript	0	0.219649	0.069239	0.021358	0	0	0.265266	0.153184	0
Novel_003691	XLOC_03916.	transcript	0	0	0	0	0	0	0	0	0
Novel_003692	XLOC_03916.	transcript	0.21857	0.102823	0.221191	0.262844	0.174735	0.271371	0.232432	0.20551	0.02144
Novel_003693	XLOC_03916.	transcript	0	0.032072	0.068797	0.289812	0.040583	0.023365	0.151456	0.027476	0.228883
Novel_003694	XLOC_03916.	transcript	0	0	0	0.290281	0.090223	0	0	0.199437	0
Novel_003695	XLOC_03917.	transcript	0.015183	0	0	0	0	0	0	0.009217	0
Novel_003696	XLOC_03917.	transcript	0.142922	0.161714	0.205747	0.239156	0.234321	0.4557	0.176498	0.295089	0.31616
Novel_003697	XLOC_03922.	transcript	0.369606	0.515545	0.185395	0.296017	0.310444	0.287653	0.332218	0.326885	0.18818
Novel_003698	XLOC_03923.	transcript	0.201741	0.044597	0.099412	0.058302	0.150706	0.016231	0.211782	0.171475	0
Novel_003699	XLOC_03939.	transcript	0.004735	0	0.034856	0	0.01355	0.03019	0.033483	0	0.031171
Novel_003700	XLOC_03939.	transcript	0.194832	0.591501	0.228544	0.275893	0.149882	0.154622	0.20829	0.198859	0.165915
Novel_003701	XLOC_03943.	transcript	0.488887	0.198565	0.447644	0.414095	0.476613	0.575909	0.492714	0.435887	0.382008
Novel_003702	XLOC_03944.	transcript	0.145522	0.137321	0.323799	0.372052	0.337695	0.434983	0.466603	0.476895	0.239021
Novel_003703	XLOC_03945.	transcript	0.117348	0.200609	0.094398	0.044024	0.176005	0	0.179732	0.149583	0.039787
Novel_003704	XLOC_03945.	transcript	0.398064	0.509768	0.238354	0.607604	0.259164	0.55963	0.343483	0.457893	0.185782
Novel_003705	XLOC_03947.	transcript	0.064874	0.06465	0.212813	0.152509	0.146179	0.136581	0.118299	0.070391	0.260182
Novel_003706	XLOC_03949.	transcript	0.015162	0.05008	0	0	0	0	0	0.189961	0
Novel_003707	XLOC_03949.	transcript	0	0.094852	0.049827	0.007895	0.015378	0	0.011372	0.005884	0.005254
Novel_003708	XLOC_03949.	transcript	0.254394	0	0	0	0	0	0	0	0
Novel_003709	XLOC_03949.	transcript	0	0	0	0	0	0	0	0	0
Novel_003710	XLOC_03949.	transcript	0	0	0	0	0.430127	0	0.463009	0	0.28998
Novel_003711	XLOC_03949.	transcript	0	0	0.007564	0	0	0.212654	0	0	0
Novel_003712	XLOC_03949.	transcript	0	0	0.44161	0	0	0	0	0	0

Novel_003713	XLOC_03949.	transcript	0.025733	1.570259	0	0	0	0	0.599361	0.009967	0.010745
Novel_003714	XLOC_03949.	transcript	0.231454	0.714661	0	0	0	0	0.336277	0	0
Novel_003715	XLOC_03949.	transcript	0.000561	0.694926	3.20E-05	0.292067	0.090645	0.060185	0.02559	0.296328	0.172779
Novel_003716	XLOC_03949.	transcript	0.56606	1.505894	0.470932	0.028356	0.342703	0.556156	0.356556	0.526326	0.728001
Novel_003717	XLOC_03949.	transcript	0.059609	0.012526	0	0	0.01475	0.082588	0	0	0
Novel_003718	XLOC_03949.	transcript	0	0	0	0	0	0	0	0	0
Novel_003719	XLOC_03949.	transcript	0	0	0	0	0	0	0	0.221533	0
Novel_003720	XLOC_03949.	transcript	0	0	0.520983	0	0.14498	0	0.352756	0	0
Novel_003721	XLOC_03949.	transcript	0	0.000317	0	0.388826	0	0	0.001624	0	0
Novel_003722	XLOC_03949.	transcript	0	0	0	0	0	0	0	0	0
Novel_003723	XLOC_03949.	transcript	0	0	0	0	0	0	0	0	0
Novel_003724	XLOC_03949.	transcript	0	0	0	0	0	0	0	0	0
Novel_003725	XLOC_03949.	transcript	0	0.596993	0	0	0	0	0	0	0
Novel_003726	XLOC_03949.	transcript	0	1.236118	0.03619	0.018877	0	0	0	0	0
Novel_003727	XLOC_03949.	transcript	0	0	0	0	0.318801	0	0.525974	0	0
Novel_003728	XLOC_03949.	transcript	0	0	0	0	0	0	0.180001	0	0.075926
Novel_003729	XLOC_03949.	transcript	0.054248	0	0	0	0	0.092629	0.220404	0.253669	0
Novel_003730	XLOC_03949.	transcript	0	0	0	0	0	0	0	0	0
Novel_003731	XLOC_03949.	transcript	0.716285	0	0	0	0	0	0	0	0
Novel_003732	XLOC_03949.	transcript	0	0	0	0	0	0	0	0.411974	0
Novel_003733	XLOC_03949.	transcript	0	0	0	0	0.442883	0	0	0.021995	0
Novel_003734	XLOC_03949.	transcript	0	0	0	0	0	0	0	0	0
Novel_003735	XLOC_03949.	transcript	0.013703	0.110247	0.015143	0	0	0.27393	0.041076	0.013215	0.142826
Novel_003736	XLOC_03949.	transcript	0.014074	0.452899	0	0.039187	0	0.06085	0.019364	0	0.055041
Novel_003737	XLOC_03949.	transcript	0.187651	3.443309	0.250052	0.229873	0.143379	0.714291	0.797199	0.257253	0.221035
Novel_003738	XLOC_03949.	transcript	0	0.453191	0	0	0	0	0	0.205772	0
Novel_003739	XLOC_03952.	transcript	0	0.213805	0	0	0.240894	0	0	0.006835	0.07036
Novel_003740	XLOC_03952.	transcript	0	0.461012	0	0.009585	0	0.016372	0.011069	0	0
Novel_003741	XLOC_03952.	transcript	0.056879	0.194788	0.201255	0.062171	0.027719	0	0.149795	0.002967	9.40E-05
Novel_003742	XLOC_03952.	transcript	0	0	0	0	0	0.023543	0	0.068562	0.22797
Novel_003743	XLOC_03954.	transcript	0.452655	0.266289	0.824462	0.754991	0.671749	0.726252	0.548423	0.532395	0.456008
Novel_003744	XLOC_03955.	transcript	0.285289	0.275334	0.366388	0.347807	0.327413	0.372704	0.433523	0.347413	0.241179
Novel_003745	XLOC_03957.	transcript	0.851416	0.331055	0.382054	0.704044	0.326832	1.444906	1.61243	0.228739	0.935876
Novel_003746	XLOC_03957.	transcript	0.091663	0.008092	0.03834	0.01169	0.037082	0.073979	0.041664	0.025436	0.00313
Novel_003747	XLOC_03957.	transcript	0.253229	0.183744	0.586081	0.271703	0.416609	0.000214	0	0.176375	0.011539
Novel_003748	XLOC_03957.	transcript	0.239657	0.921748	0.564681	0.255306	1.049257	0.001351	0.009522	1.013086	0.004969
Novel_003749	XLOC_03957.	transcript	0.141553	0.044548	0.140978	0.080553	0.232737	0.190704	0.143995	0.141231	0.160134
Novel_003750	XLOC_03957.	transcript	0.777338	0.110087	0.23973	0.174281	0.175666	0.176722	0.317847	0.237631	0.056573

Novel_003751	XLOC_03957.	transcript	0.946442	0.960959	0.710567	0.783494	0.522627	0.80239	1.260884	0.919484	0.59113
Novel_003752	XLOC_03971.	transcript	0	0.651862	0	0.236182	0.631194	0.183189	0.056729	0	1.343697
Novel_003753	XLOC_03971.	transcript	0.069902	0.138919	0.239632	0.235684	0.002324	0	0	0.104724	0
Novel_003754	XLOC_03971.	transcript	0	0	0	0	0	0	0.060537	0.107713	0.119909
Novel_003755	XLOC_03972.	transcript	0.690463	0.223909	0.975349	0.938107	0.793164	0.970632	0.711595	0.912131	1.570053
Novel_003756	XLOC_03976.	transcript	0.898788	0.614216	0.037512	0.010066	1.148435	0.065453	0.085567	0.030684	0.051413
Novel_003757	XLOC_03976.	transcript	0	1.003769	1.213938	1.830085	0	1.151559	2.011345	1.794346	1.190831
Novel_003758	XLOC_03976.	transcript	0.260822	0.739526	0.334881	0.464201	0.543374	0.342327	0.531556	0.521172	0.455728
Novel_003759	XLOC_03977.	transcript	0.356527	0.46594	0.468992	1.035511	1.104534	0.756914	0	0.325661	0.047378
Novel_003760	XLOC_03977.	transcript	6.195891	20.2703	18.35305	18.06397	16.57691	17.60141	13.50186	21.68231	15.11587
Novel_003761	XLOC_03977.	transcript	0	0.361142	0	0	0	0	0.362247	0	0
Novel_003762	XLOC_03977.	transcript	0	0	0	0	0	0	0	0	0
Novel_003763	XLOC_03977.	transcript	0.003339	0	0.008021	0	0.004896	0	0.006256	0.004023	0.004052
Novel_003764	XLOC_03977.	transcript	0	0	0	0.028272	0	0	0	0.397177	0
Novel_003765	XLOC_03977.	transcript	0	0.022442	0.007602	0.119233	0.017411	0.015795	0.036607	0	0
Novel_003766	XLOC_03977.	transcript	0	0.085562	0	0	0.285222	0	0.156575	0.175615	0.241087
Novel_003767	XLOC_03977.	transcript	0.306395	0.125779	0.271664	0.188335	0.257362	0.239784	0.220571	0.104546	0.170432
Novel_003768	XLOC_03977.	transcript	0.53125	0.255536	0.420129	0.660368	0.341375	0.808209	0	0	0.624843
Novel_003769	XLOC_03978.	transcript	0	0	0	0	0.662784	0	0	0	0
Novel_003770	XLOC_03978.	transcript	0.031739	0	0	0.579381	0	0	0	0	0
Novel_003771	XLOC_03978.	transcript	0.409569	1.019239	1.031892	0.945701	0.627158	1.077564	1.168457	1.223127	1.420972
Novel_003772	XLOC_03979.	transcript	0	0	0	0	0	0	0	0	0
Novel_003773	XLOC_03979.	transcript	0.584568	0.037189	1.601853	0.646835	0.398643	1.149663	1.746379	0.754273	0.381625
Novel_003774	XLOC_03992.	transcript	0	0.370721	0	0	0	0	0	0	0
Novel_003775	XLOC_03997.	transcript	0	0	0.04778	0.04962	0.058317	0	0.404928	0.036144	0
Novel_003776	XLOC_03999.	transcript	0.745273	1.554307	0.961508	1.07944	0.967859	1.276571	1.096084	1.030867	1.176982
Novel_003777	XLOC_04014.	transcript	3.128232	1.447098	0.694198	1.852156	0.682056	2.302621	0.995955	1.139825	1.88694
Novel_003778	XLOC_04014.	transcript	0.015548	0.89031	0.01804	0	0	0	0.025523	0	0
Novel_003779	XLOC_04014.	transcript	0.039465	0.997292	0	0.087488	0	0	0	0	0.140311
Novel_003780	XLOC_04021.	transcript	0.47606	0.722535	0	2.274926	1.52621	3.79485	0.807144	1.752646	0
Novel_003781	XLOC_04021.	transcript	0	0.133236	0.346416	0	0.002348	0.067307	0.189787	0.073966	0.598605
Novel_003782	XLOC_04025.	transcript	0.77855	0.843384	0.540124	0.61835	0.488212	0.666808	0.743973	0.275595	0.546791
Novel_003783	XLOC_04028.	transcript	0.282574	0.075268	0.186048	0.153785	0.288715	0.189183	0.383575	0.287193	0.281797
Novel_003784	XLOC_04028.	transcript	0.569668	0	0.276411	0	0.27459	0.279452	0	0.509183	0.369378
Novel_003785	XLOC_04028.	transcript	0.063756	0.013278	0.175909	0.515974	0.062909	0.023518	0.458955	0.273746	0.036302
Novel_003786	XLOC_04035.	transcript	0.074148	0	0.157829	0	0	0	0	0	0.223062
Novel_003787	XLOC_04035.	transcript	0.377731	0	0	0	0	0	0	0	0
Novel_003788	XLOC_04035.	transcript	0.173896	1.154158	0.219955	0.470258	0.68615	0.884119	0.329665	0.4266	0.486227

Novel_003789	XLOC_04035.	transcript	0.015403	0.986508	0.134238	0.007818	0.00562	0	0.016437	0.144752	0
Novel_003790	XLOC_04037.	transcript	0	0.492306	0.008677	0.390199	0.374073	0.169561	0.466649	0.274486	0.258226
Novel_003791	XLOC_04037.	transcript	0	0	0	0	0	0.167628	0	0	0
Novel_003792	XLOC_04037.	transcript	0.34352	0	0.319384	0	0	0	0	0	0
Novel_003793	XLOC_04037.	transcript	0.191914	0.76203	0.089768	0.436706	0.269335	0.253262	0.246093	0.263967	0.272775
Novel_003794	XLOC_04037.	transcript	0.288498	0	0.015311	0.090837	0	0.042771	0	0.073417	0
Novel_003795	XLOC_04039.	transcript	0	0	0	0	0.228786	0	0.250842	0	0
Novel_003796	XLOC_04039.	transcript	1.332912	1.45004	0.660988	1.172557	0.789318	0.706474	0.851209	0.749059	0.536042
Novel_003797	XLOC_04045.	transcript	0.453199	0.525363	0.515794	0.298516	0.329848	0.493615	0.488548	0.429198	0.426443
Novel_003798	XLOC_04047.	transcript	1.258869	0.257277	1.717959	2.514335	1.749823	2.109307	3.023565	2.819315	1.232655
Novel_003799	XLOC_04054.	transcript	0	0.532646	0.005538	0.523726	0.020534	9.30E-05	0.033155	0.007946	0
Novel_003800	XLOC_04054.	transcript	1.956299	0.173185	1.497794	0.344145	0.513616	1.573851	0.46713	1.168219	0.392366
Novel_003801	XLOC_04054.	transcript	0	0.624301	0	0	0	0	0	0	0
Novel_003802	XLOC_04054.	transcript	0	0.640768	0	1.0531	0	0.446794	1.759843	0.719833	0.465506
Novel_003803	XLOC_04054.	transcript	0.320013	0.548464	0.316918	0.649744	1.678233	0.445994	0	0.796997	0.898473
Novel_003804	XLOC_04055.	transcript	0.071878	0.270296	0.295736	0.186627	0.220252	0.258548	0.150661	0.303464	0.098181
Novel_003805	XLOC_04058.	transcript	0.264639	1.2162	0.322629	0.333956	0.558153	0.354586	0.354092	0.447275	0.961904
Novel_003806	XLOC_04060.	transcript	0.509917	0.721993	0.339654	0.32893	0.364462	0.460543	0.521706	0.384147	0.297725
Novel_003807	XLOC_04060.	transcript	0.475857	0.740358	0.301841	0.314951	0.372938	0.497106	0.586687	0.323712	0.273176
Novel_003808	XLOC_04061.	transcript	0.971263	1.033187	0.990586	1.187488	1.367357	0.9313	1.115164	1.153536	0.917179
Novel_003809	XLOC_04063.	transcript	0	0	0.175625	0.002817	0.147841	0.346559	0	0	0
Novel_003810	XLOC_04063.	transcript	0.005908	0.021232	0.055773	0.002558	0.064643	0.015754	0	0	0
Novel_003811	XLOC_04063.	transcript	0.308068	0.090784	0.180521	0.215963	0.202926	0.084832	0.409167	0.423039	0.246099
Novel_003812	XLOC_04063.	transcript	0.00846	0	0.011519	0.001745	0	0	0.18573	0	0
Novel_003813	XLOC_04063.	transcript	0.027967	0	0	0	0	0.111817	0.010243	0.095802	0
Novel_003814	XLOC_04063.	transcript	0.103037	0	0.104527	0.028999	0.046433	0.015387	0.093519	0.147276	0.049547
Novel_003815	XLOC_04068.	transcript	0.164577	0.028548	0.1049	0.129966	0.084293	0.038114	0.189944	0.090157	0.048985
Novel_003816	XLOC_04080.	transcript	0.672701	1.825103	0.631955	0.361133	0.552794	0.509194	0.539943	0.534594	0.255302
Novel_003817	XLOC_04085.	transcript	0.01174	0	0.000868	0.005158	0.517421	0.022055	0	0	0
Novel_003818	XLOC_04085.	transcript	0.55707	0.061837	0.674996	0.668641	0.210474	0.905458	0.478116	0.513787	0.328087
Novel_003819	XLOC_04086.	transcript	1.738428	0.379443	1.471582	1.796536	1.286539	2.012541	1.309359	0.854263	0.541857
Novel_003820	XLOC_04086.	transcript	0	0	0.021408	0.053899	0.482151	0	0.018142	0.185045	0
Novel_003821	XLOC_04086.	transcript	0.223244	0	0.30673	0.003217	0.3655	0.102951	0.187972	0.634552	0.027227
Novel_003822	XLOC_04087.	transcript	1.031833	1.333564	0.794905	1.116178	1.101592	1.183959	0.686155	1.266868	1.336361
Novel_003823	XLOC_04088.	transcript	0.028555	0.010196	0.024666	0.147441	0	0.20221	0.145125	0.028657	0.117997
Novel_003824	XLOC_04088.	transcript	0.340864	0.380292	0.272089	0.290442	0.199734	0	0.128629	0.40772	0.009384
Novel_003825	XLOC_04089.	transcript	0.050505	0	0	0	0.051656	0	0.018886	0	0
Novel_003826	XLOC_04089.	transcript	0	0	0.013502	0	0.013063	0	0.076382	0.007312	0

Novel_003827	XLOC_04093.	transcript	0.145174	0.007968	0.05097	0.009095	0	0.115369	0.090667	0.026029	0.565952
Novel_003828	XLOC_04093.	transcript	0.985632	0.097498	1.151664	1.762774	1.091211	1.466448	1.797311	1.069383	1.817869
Novel_003829	XLOC_04093.	transcript	1.042153	0.080931	0.65575	0.401479	0.668039	0.498997	0.365272	0.683104	0.514362
Novel_003830	XLOC_04095.	transcript	0.227785	0	0.173382	0.624236	0.135684	0.545885	0.745946	0.800638	3.135507
Novel_003831	XLOC_04095.	transcript	0.543945	0	0.22276	0.775075	0.051806	0.139634	0.402266	0.149953	0.385874
Novel_003832	XLOC_04095.	transcript	0	0	0.225338	0.398637	0	0.325471	0	0.320347	0.650557
Novel_003833	XLOC_04095.	transcript	0	0	0.29997	0.372224	1.31552	0.632592	0.408383	0	0
Novel_003834	XLOC_04095.	transcript	0.959264	0.053231	0	2.072021	0	0	0	0.126784	0.558067
Novel_003835	XLOC_04099.	transcript	0.590348	0.35952	0.638583	0.429464	0.600717	0.489359	0.768015	0.530779	0.702808
Novel_003836	XLOC_04104.	transcript	0	0.109232	0.134696	0	0	0	0	0	0
Novel_003837	XLOC_04104.	transcript	0	0.110736	0	0	0	0	0	0	0
Novel_003838	XLOC_04104.	transcript	0.132551	0	0	0	0.151973	0.343966	0.138891	0	0.274875
Novel_003839	XLOC_04104.	transcript	0	1.216743	0	0	0	0	0	0.098943	0
Novel_003840	XLOC_04104.	transcript	0	0.101225	0.114192	0.124637	0	0	0	0.052784	0
Novel_003841	XLOC_04104.	transcript	0	0	0	0	0	0	0	0.39377	0
Novel_003842	XLOC_04104.	transcript	0.197319	0.242361	0.038612	0.074359	0.117406	0.152232	0.332452	0.155056	0.223866
Novel_003843	XLOC_04104.	transcript	0.054534	0.22268	0.188482	0.107807	0.113416	0.167136	0.235848	0.221809	0.095475
Novel_003844	XLOC_04105.	transcript	0	0	0	0.185145	0	0.275347	0.28634	0.224246	0.202964
Novel_003845	XLOC_04105.	transcript	0	0	0	0.83451	0.779629	0.306592	0.631283	0.623955	0.291886
Novel_003846	XLOC_04105.	transcript	0.378591	0.090308	0.187674	0	0.718568	0.321172	0	0	0
Novel_003847	XLOC_04105.	transcript	1.254011	0.275093	0.645374	0.161408	0.080573	0.341686	0.41509	0.294455	0.382542
Novel_003848	XLOC_04105.	transcript	0.000612	0.646084	0.717368	0.194711	0.015097	0.250931	0.788257	1.171592	0.740818
Novel_003849	XLOC_04106.	transcript	0.197591	0.031024	0.11022	0.165634	0.086456	0.03593	0.245919	0.170625	0.236465
Novel_003850	XLOC_04107.	transcript	0.1908	0.043354	0.144176	0.214758	0.166168	0.099374	0.218828	0.248179	0.175013
Novel_003851	XLOC_04107.	transcript	0	0	0	0	0	0	0	0	0
Novel_003852	XLOC_04107.	transcript	1.016456	0.25566	0.110005	0.073869	0.087234	0.186288	0.86325	0.518727	0.587116
Novel_003853	XLOC_04107.	transcript	0.979601	0.468008	0.605729	0.552515	0.717505	0.72765	1.142562	0.817791	0.650232
Novel_003854	XLOC_04109.	transcript	0.418867	2.950892	0.293351	0.209842	0.31973	0.26262	0.474161	0.25933	0.265016
Novel_003855	XLOC_04111.	transcript	0	0	0	0.161853	0	0	0	0	0
Novel_003856	XLOC_04111.	transcript	0.045911	0.071233	0	0	0.047334	0.10499	0.068713	0.087165	0.109346
Novel_003857	XLOC_04113.	transcript	0.257934	0.420598	0.263791	0.373238	0.323496	0.224231	0.448518	0.461377	0.214237
Novel_003858	XLOC_04114.	transcript	0.394534	0.538733	0.694136	0.666282	0.844997	0.721628	0.6353	0.449643	0.403502
Novel_003859	XLOC_04114.	transcript	0.132145	0	0.333592	0.210213	0.261845	0.922824	0.4889	0.336368	0.103173
Novel_003860	XLOC_04128.	transcript	0	0.017116	0.08739	0.094652	0.133877	0.359484	0.219485	0.280693	0.018146
Novel_003861	XLOC_04128.	transcript	0.241461	0.227886	0.069448	0.131078	0.131163	0.179094	0.046392	0	0.331924
Novel_003862	XLOC_04130.	transcript	0.009632	0.038165	0	0.075274	0.013317	0	0.014408	0	0.022831
Novel_003863	XLOC_04134.	transcript	0	0.182268	0.071003	0	0	0	0.17185	0.200005	0
Novel_003864	XLOC_04134.	transcript	0.354585	0.168746	0.019396	0.094838	0.270907	0.39446	0.151469	0.138366	0.041367

Novel_003865	XLOC_04134.	transcript	0	0	0	0.464747	0.420451	0	0.10553	0	0.848092
Novel_003866	XLOC_04134.	transcript	0	0.414337	0.788437	0	0	0	0	0	0
Novel_003867	XLOC_04134.	transcript	0	0.113203	0	0	0	0.214056	0.280631	0	0
Novel_003868	XLOC_04137.	transcript	0.176211	0.605102	0.236648	0.019368	0.016784	0.009958	0.032875	0.267313	2.50E-05
Novel_003869	XLOC_04137.	transcript	0.286462	0.309837	0.325807	0.247168	0.264569	0.388064	0.223477	0.321303	0.412866
Novel_003870	XLOC_04140.	transcript	0	0.224523	0.186798	0	0	0	0.160691	0	0
Novel_003871	XLOC_04140.	transcript	0.257439	0.256826	0	0.159508	0	0	0	0.170412	0.050055
Novel_003872	XLOC_04140.	transcript	0	0	0	0	0.208595	0.080105	0.098822	0	0.082836
Novel_003873	XLOC_04143.	transcript	0.118506	0.742619	0.029213	0.013864	0.016882	0.019959	0.08885	0.037938	0.013099
Novel_003874	XLOC_04147.	transcript	0.83222	1.05134	1.071468	1.010564	0.687758	1.108642	0.859724	1.054586	0.814942
Novel_003875	XLOC_04148.	transcript	1.266408	0.247018	1.523938	1.295573	1.379355	1.239126	1.595568	2.055124	1.759115
Novel_003876	XLOC_04149.	transcript	0.000451	0	0	0	0	0	0.001938	0.371444	0
Novel_003877	XLOC_04149.	transcript	0	0	0	0	0	0	0.292876	0.199374	0
Novel_003878	XLOC_04149.	transcript	0.56897	0.312588	0.437516	0.543488	0.487682	0.596873	0.39914	0.18336	0.722512
Novel_003879	XLOC_04149.	transcript	0	0	0	0	0	0	0	0	0
Novel_003880	XLOC_04168.	transcript	0.165301	0	0	0	0	0	0	0.023661	0
Novel_003881	XLOC_04169.	transcript	0	0	0	0.376417	0	0.356893	0	0	0.222742
Novel_003882	XLOC_04169.	transcript	0.176755	0.090557	0.187315	0	0.395707	0	0.376891	0.231285	0
Novel_003883	XLOC_04171.	transcript	0.128318	1.015838	0.091741	0.201898	0.098609	0.038753	0.12007	0.146844	0.057099
Novel_003884	XLOC_04172.	transcript	0.497807	0	0	0	0	0	0.046423	0.023277	0
Novel_003885	XLOC_04172.	transcript	0.088404	0.010663	0.200945	0.115695	0	0.155577	0.293918	0.27043	0
Novel_003886	XLOC_04172.	transcript	0	0	0.061838	0.004201	0.174746	0	0	0	0
Novel_003887	XLOC_04172.	transcript	0.219639	0.113502	0.112086	0.071218	0.160833	0.060304	0.09841	0.055643	0.26094
Novel_003888	XLOC_04180.	transcript	0	0	0	0	0	0	0	0	0
Novel_003889	XLOC_04180.	transcript	0.357652	0	0.665334	0	0	0	0	0	0
Novel_003890	XLOC_04180.	transcript	0	0	0	0	0	0	0	0	0
Novel_003891	XLOC_04180.	transcript	0.31762	0	0.026341	0.001967	0	0	0	0	0
Novel_003892	XLOC_04180.	transcript	0.153407	0.121477	0.07722	0.07026	0.029962	0.030092	0.105876	0.083311	0.01733
Novel_003893	XLOC_04182.	transcript	0.04948	0.011405	0.048583	0.167521	0.004621	0.017806	0.046738	0.046165	0.019638
Novel_003894	XLOC_04182.	transcript	1.250654	1.622238	1.636491	2.793347	1.901074	2.643203	1.357787	2.541298	1.662384
Novel_003895	XLOC_04185.	transcript	0	0.090134	0	0.115905	0	0.298173	0	0	0.055247
Novel_003896	XLOC_04185.	transcript	0	0.039461	0	0.14261	0	0.75726	0	0	0.134981
Novel_003897	XLOC_04185.	transcript	0.029129	0.116607	0	0	0.047622	0	0	0.005442	0
Novel_003898	XLOC_04185.	transcript	0	0	0	0	0	0	0	0.556922	0
Novel_003899	XLOC_04185.	transcript	0.343298	0.265574	0.415122	0.588843	0.419844	0.498604	0.477452	0.135113	0.353671
Novel_003900	XLOC_04185.	transcript	0.154959	0.073175	0.117509	0.159527	0	0.007015	0.272181	0.132791	0.188824
Novel_003901	XLOC_04185.	transcript	0	0.077594	0.128887	0.099268	0.118243	0.228427	0.091759	0.238644	0.117045
Novel_003902	XLOC_04185.	transcript	0.087248	0.06268	0.11714	0.141268	0	0.00162	0	0.145791	0.169459

Novel_003903	XLOC_0419C.	transcript	1.290387	1.620444	1.178302	1.038265	1.051422	0.856988	1.168897	1.103062	1.106227
Novel_003904	XLOC_0419E.	transcript	1.508927	1.579993	2.183694	2.845962	1.426869	2.52938	1.624673	2.554896	2.127632
Novel_003905	XLOC_0419E.	transcript	0	0	0	0	0	1.176854	0	0	0
Novel_003906	XLOC_0419E.	transcript	0.060577	0.088622	0.133753	0.135197	0.177691	0.111501	0.170363	0.187543	0.16529
Novel_003907	XLOC_04197.	transcript	0	0	0.179168	0	0	0.383386	0	0	0.391723
Novel_003908	XLOC_04197.	transcript	1.034595	0.312792	0.841411	0.954533	0.889689	1.102544	1.398073	0.799532	1.037076
Novel_003909	XLOC_0420З.	transcript	2.773965	0.441204	3.833199	2.922401	3.731058	2.564038	3.329394	2.547594	3.483217
Novel_003910	XLOC_0420E.	transcript	0.085904	0.086618	0	0.102931	0	0	0	0.049989	0
Novel_003911	XLOC_0420E.	transcript	0.058119	0.652274	0.132477	0	0.126973	0.109477	0.146499	0.030698	0.233562
Novel_003912	XLOC_0420€.	transcript	0.127705	0.037576	0.264203	0.142696	0.27091	0.064798	0.262912	0.18199	0.187892
Novel_003913	XLOC_0420€.	transcript	0.06304	0.460865	0.271804	0.282535	0.140242	0.339336	0.296941	0.526929	0.166612
Novel_003914	XLOC_0420ε.	transcript	0.239974	0.710577	0.605371	0.345892	0.613168	0.018614	0.326528	0.577791	0.265684
Novel_003915	XLOC_0420ε.	transcript	0.473622	0	0	0	0	0	0	0	0
Novel_003916	XLOC_0420ε.	transcript	0.39356	0.480748	0.271262	0.466562	0.378025	0.848027	0.568227	0.235732	0.363046
Novel_003917	XLOC_0420ς.	transcript	0.067362	0.110603	0	0	0	0.018168	0	0	0.009218
Novel_003918	XLOC_0420ς.	transcript	0.12769	0.176689	0.124794	0.118646	0.076619	0.136898	0.114229	0.114679	0.134242
Novel_003919	XLOC_0420ς.	transcript	0.300503	0.310434	0.265373	0.214541	0.13717	0.23686	0.346274	0.201621	0.232136
Novel_003920	XLOC_0420ς.	transcript	0.731976	0.599559	0.945256	0.375461	0.663077	0.428232	0.62057	0.6384	0.631904
Novel_003921	XLOC_04211.	transcript	0.415013	0.895682	2.313871	3.250305	2.549461	1.556094	0.75458	1.721834	1.489316
Novel_003922	XLOC_04211.	transcript	0.078991	0.130789	0.027837	0.12027	0.121905	0.174727	0.236308	0.045176	0.123259
Novel_003923	XLOC_0421ς.	transcript	0.05281	0.14184	0.09185	0.063548	0.007799	0	0.014258	0	0.009037
Novel_003924	XLOC_04221.	transcript	0.016828	1.592166	0	0.01681	0	0	0	0	0.009311
Novel_003925	XLOC_04221.	transcript	0.22299	0.275082	0.1538	0.136933	0.113835	0.002945	0.255705	0.000779	0.011718
Novel_003926	XLOC_04221.	transcript	0	0.447877	0	0	0.03362	0.137331	0.138543	0.075832	0.210947
Novel_003927	XLOC_04221.	transcript	0	1.856441	0	0	0.012478	0	0.037786	0.072188	0
Novel_003928	XLOC_04221.	transcript	0.016859	1.15807	0.011566	0.018479	0.006088	0.086193	0	0.034227	0.092313
Novel_003929	XLOC_04221.	transcript	0	0.129286	0	0	0.012609	0	0	0	0.155993
Novel_003930	XLOC_04221.	transcript	0	0.928327	0	0.008997	0	0	0	0	0.000518
Novel_003931	XLOC_0422ς.	transcript	0.382098	0.045246	0.279609	0.419531	0.418344	0.31642	0.181145	0.410696	0.268884
Novel_003932	XLOC_0424Ε.	transcript	0	0	0.276553	0.33662	0.145686	0.320933	0.561092	0.435307	0.052469
Novel_003933	XLOC_04251.	transcript	2.001972	2.190146	1.642274	2.576669	2.716582	2.914103	2.502765	2.773756	1.912652
Novel_003934	XLOC_04251.	transcript	0.13789	0.215256	1.743365	0.117798	0.183527	0.181043	0.030383	0.159407	0.0798
Novel_003935	XLOC_04251.	transcript	0.083758	1.527841	0.580626	0.163421	1.900971	0.539603	0.269613	0.431244	1.758056
Novel_003936	XLOC_04251.	transcript	1.804271	0	0.517884	1.668235	0	1.432069	0.54827	0.873887	0
Novel_003937	XLOC_0425Ձ.	transcript	0.474984	0.422227	0.182151	0	0.335688	0.247679	0.31283	0.267315	0.152051
Novel_003938	XLOC_0425Ձ.	transcript	0	0.270825	0	0.315999	0.113937	0.292518	0	0.124732	0.284389
Novel_003939	XLOC_0425Ձ.	transcript	0	0	0.283053	0.005056	0	0	0.13935	0.087865	0
Novel_003940	XLOC_0425Ձ.	transcript	0.005775	0.091708	0.042942	0.124276	0	0.263185	0.089328	0.143252	0.065364

Novel_003941	XLOC_04285.	transcript	0.024113	0	0	0	0	0	0.018191	0	0
Novel_003942	XLOC_04285.	transcript	0.131807	0.200877	0.240813	0.498026	0.631118	0.366265	0.388975	0.004044	0.426759
Novel_003943	XLOC_04285.	transcript	0.373408	0.043691	0.222877	0.164457	0.218498	0.169287	0.32883	0.754095	0.134298
Novel_003944	XLOC_04285.	transcript	0.452272	0.053385	0.218856	0.049407	0.283496	0.17006	0.26414	0.01947	0.203694
Novel_003945	XLOC_04285.	transcript	0.271802	0.059042	0.507331	0.611266	0.534816	0.46415	0.623328	0.414718	0.480503
Novel_003946	XLOC_04285.	transcript	0	0	0	0	0	0	0	0	0
Novel_003947	XLOC_04287.	transcript	0.014212	0.396485	0	0	0	0	0.404992	0.2775	0.2219
Novel_003948	XLOC_04287.	transcript	0.081706	0.435067	0	0.057772	0.090652	0	0.081249	0	0.141832
Novel_003949	XLOC_04287.	transcript	1.062149	0.335541	0.506003	0.704806	0.906148	0.681343	0.699221	0.748003	0.780221
Novel_003950	XLOC_04287.	transcript	0.97911	0.457708	0.865086	0.718297	0.567629	0.989845	0.611386	0.749142	0.186975
Novel_003951	XLOC_04287.	transcript	0	0	0.004382	0.001553	0	0.006796	0	0	0.004857
Novel_003952	XLOC_04300.	transcript	0	0	0	0	0	0.114019	0	0.36569	0
Novel_003953	XLOC_04300.	transcript	0.008603	0.056095	0	0.019383	0.020004	0	0	0.252746	0
Novel_003954	XLOC_04300.	transcript	0.278741	0	0	0	0	0.051171	0.23565	0.149408	0
Novel_003955	XLOC_04300.	transcript	0.09926	0.044817	0.262687	0.160278	0.253557	0.218261	0.030047	0.068436	0.022699
Novel_003956	XLOC_04300.	transcript	0.281317	0	0.1245	0.214694	0.073524	0.223534	0.17669	0.189217	0
Novel_003957	XLOC_04308.	transcript	0.372748	0.016411	0.228575	0.269721	0.291841	0.373636	0.310147	0.250244	0.172611
Novel_003958	XLOC_04308.	transcript	0	0	0	0	0	0	0	0.015232	0
Novel_003959	XLOC_04308.	transcript	0.133962	0.015771	0.056889	0.142576	0.107329	0.291474	0.101781	0.162151	0.075753
Novel_003960	XLOC_04335.	transcript	0.216131	0.113216	0.412171	0.672321	0.394673	0.355386	0.392927	0.639905	0.305328
Novel_003961	XLOC_04335.	transcript	0	0	0	0.249437	0	0	0	0	0
Novel_003962	XLOC_04335.	transcript	0	0	0	0	0	0.082054	0	0	0
Novel_003963	XLOC_04339.	transcript	0	0.601054	0	0.533658	0.238936	0	0.052576	0.333204	0.370019
Novel_003964	XLOC_04339.	transcript	0.508443	0.335361	0.512849	0.062857	0.324659	0.606837	0.617966	0.146739	0.506271
Novel_003965	XLOC_04339.	transcript	0.189775	0.143589	0.142305	0.083825	0.048642	0.184391	0.368261	0.067435	0.06799
Novel_003966	XLOC_04339.	transcript	0.357287	0.103157	0.378959	0.194223	0.49756	0.317454	0.470421	0.181734	0.271581
Novel_003967	XLOC_04339.	transcript	0.510031	0.459203	0.532419	0.452257	0.676214	0.896075	0.567597	0.387002	0.287493
Novel_003968	XLOC_04339.	transcript	0.320743	0.051626	0.454957	0.284656	0.071771	0.098663	0.669919	0.518581	0.460248
Novel_003969	XLOC_04346.	transcript	17.41556	0.679436	9.725397	15.95682	21.31748	15.79547	15.49295	14.6302	15.68227
Novel_003970	XLOC_04346.	transcript	9.181938	0.534934	4.791759	5.67811	2.385512	6.539009	6.423762	7.573878	2.307216
Novel_003971	XLOC_04348.	transcript	2.663147	5.12577	2.875104	3.564444	2.660881	3.20948	4.409827	4.510679	4.020468
Novel_003972	XLOC_04348.	transcript	0.17827	0.154777	0.112451	0.116804	0.125902	0.199098	0.180998	0.088659	0.075828
Novel_003973	XLOC_04355.	transcript	0.379462	0.656673	0.434845	0.406899	0.489452	0.424728	0.455949	0.525086	0.408154
Novel_003974	XLOC_04355.	transcript	0	0.084744	0	0.055487	0.120658	0	0.064201	0.052892	0
Novel_003975	XLOC_04357.	transcript	0.324848	0.162421	0.476299	0.173293	0.204351	0.1882	0.322964	0.277042	0.128137
Novel_003976	XLOC_04364.	transcript	0.224236	0.312799	0.316437	0.355212	0.455788	0.301258	0.211386	0.431762	0.25815
Novel_003977	XLOC_04364.	transcript	0	0.027538	0	0	0	0	0	0	0.193648
Novel_003978	XLOC_04364.	transcript	0	0	0	0	0.001275	0	0.153151	0	0

Novel_003979	XLOC_04365.	transcript	0.511701	0.39311	0.430322	0.259494	0.419045	0.369463	0.427809	0.384335	0.667508
Novel_003980	XLOC_04365.	transcript	0	0	0	0.130621	0	0.198103	0.225934	0.416616	0
Novel_003981	XLOC_04367.	transcript	0.387578	0.302485	0.288146	0.276156	0.248478	0.416682	0.5175	0.038125	0.333139
Novel_003982	XLOC_04367.	transcript	0	0	0	0.060557	0	0	0	0.423401	0
Novel_003983	XLOC_04367.	transcript	0	0	0	0	0	0	0	0	0
Novel_003984	XLOC_04367.	transcript	0.023826	0.006112	0.016271	0.0312	0.03903	0.025718	0.017774	0.023415	0.018338
Novel_003985	XLOC_04367.	transcript	0.246188	0	0.270274	0.167761	0	0	0.126018	0.174152	0.242788
Novel_003986	XLOC_04367.	transcript	1.395084	0.308807	0.692927	0.55128	0.41496	0.737109	1.271399	0.728929	0.276308
Novel_003987	XLOC_04367.	transcript	0.514652	0.09151	0.129771	0.063446	0.066781	0.072312	0.071294	0.371671	0
Novel_003988	XLOC_04367.	transcript	0.173721	0	0	0	0	0	0.003916	0	0
Novel_003989	XLOC_04367.	transcript	0.151823	0	0.02669	0.437719	0	0.001327	0.01822	0.004346	0.003944
Novel_003990	XLOC_04367.	transcript	0.052134	0	0	0	0	0	0.005947	0	0
Novel_003991	XLOC_04367.	transcript	0.02459	0.138077	0.083121	0.10094	0.072185	0.226328	0.044306	0.029321	0.039364
Novel_003992	XLOC_04367.	transcript	0	0.07632	0.231175	0	0.55214	0.503815	0.184753	0.171128	0.202351
Novel_003993	XLOC_04367.	transcript	0.001234	0.001268	0.618239	0	0	0	0.001079	0.002398	0.312251
Novel_003994	XLOC_04367.	transcript	0.075176	0	0	0	0	0	0.254749	0	0.035842
Novel_003995	XLOC_04367.	transcript	0.770069	0.456035	0.314948	0.367772	0.437657	0.393064	1.426969	0.22122	0.486064
Novel_003996	XLOC_04367.	transcript	0	0	0	0	0	0	0	0	0.002561
Novel_003997	XLOC_04367.	transcript	2.715774	1.095256	1.388981	1.900765	1.889512	1.504817	1.99502	2.083773	0.739551
Novel_003998	XLOC_04367.	transcript	2.504247	2.991439	1.282624	2.92573	3.009371	1.968468	1.579464	1.767681	2.38528
Novel_003999	XLOC_04367.	transcript	1.058795	0.833679	0.680297	0.515297	0.823812	0.728129	1.123591	0.623831	0.586013
Novel_004000	XLOC_04369.	transcript	0.289285	2.406126	0.090338	0.152026	0.095412	0.166655	0.326128	0.206583	0.896137
Novel_004001	XLOC_04371.	transcript	1.698848	0.032964	2.173147	0.758214	1.414181	0.638589	1.837592	2.298498	1.519118
Novel_004002	XLOC_04371.	transcript	7.236851	3.896043	7.805482	6.5806	7.075815	8.400235	6.364577	8.229696	6.723898
Novel_004003	XLOC_04371.	transcript	0.925725	0.363511	1.751696	3.194071	1.695087	2.109312	1.894639	1.031859	1.859507
Novel_004004	XLOC_04375.	transcript	0.433227	0.044642	0.231962	0	0.843163	0.185104	0.191392	0.140851	0
Novel_004005	XLOC_04375.	transcript	0.321397	0	0.049007	0.519991	0	0.213461	0.246434	0.15225	0
Novel_004006	XLOC_04376.	transcript	0	0	0.460311	0	0	0.473228	0.270717	0.224192	0
Novel_004007	XLOC_04380.	transcript	0.220555	0.330043	0.113319	0.084696	0.092064	0.105278	0.211905	0.098508	0.156858
Novel_004008	XLOC_04380.	transcript	0	0	0	0.115651	0.053044	0	0	0	0.142622
Novel_004009	XLOC_04380.	transcript	0	0	0.007607	0	0.035895	0.070241	0.010301	0	0
Novel_004010	XLOC_04380.	transcript	0	0	0.155355	0	0	0.104102	0.316577	0.150045	0
Novel_004011	XLOC_04380.	transcript	0.181758	0.069788	0.072534	0.060867	0.156313	0.19497	0.192812	0.091261	0.049302
Novel_004012	XLOC_04381.	transcript	0.006367	0	0	0.461938	0.001014	0.002086	0	0	0.00128
Novel_004013	XLOC_04381.	transcript	0.186636	0.203448	0.169521	0.11444	0.227611	0.164547	0.227926	0.120949	0
Novel_004014	XLOC_04381.	transcript	0.014101	1.174818	0.637098	0.877868	0.437139	1.233838	1.111344	1.280058	0.895583
Novel_004015	XLOC_04383.	transcript	0	0.831056	0.340847	0.393513	0.331426	0.286185	0.53055	0.476037	0.636382
Novel_004016	XLOC_04384.	transcript	0.367653	0.082369	0.357141	0.236598	0.273218	0.366694	0.914423	0.314616	0.383805

Novel_004017	XLOC_04385.	transcript	0.222726	0.037129	1.022272	0.138984	0.902115	0.371062	0.795516	0.813518	0.411024
Novel_004018	XLOC_04389.	transcript	0.019433	0	0.02398	0	0.067765	0.060257	0	0.212162	0
Novel_004019	XLOC_04389.	transcript	0.048849	0	0.062316	0.00113	0.026031	0	0.026229	0.006706	0
Novel_004020	XLOC_04389.	transcript	0.090526	0	0.193917	0.001081	0.126423	0.189044	0.160373	0.019172	0.098962
Novel_004021	XLOC_04389.	transcript	0.013077	0	0	0	0	0.002525	0.020723	0.008428	0
Novel_004022	XLOC_04389.	transcript	0	0	0.000107	0	0.000287	0.000286	0.106522	0.014826	0
Novel_004023	XLOC_04389.	transcript	0.00601	0	0	0.205311	0	0	0.003708	0.012528	0
Novel_004024	XLOC_04389.	transcript	0.034906	0	0.054791	0.069403	0	0	0	0.200987	0
Novel_004025	XLOC_04389.	transcript	0.059445	0	0	0	0.07595	0.548168	0	0	0
Novel_004026	XLOC_04389.	transcript	0.449508	0	0.159895	0.205369	0.078699	0	0.076562	0.06253	0.057105
Novel_004027	XLOC_04389.	transcript	0.023988	0	0.002844	0.106957	0.344285	0.027138	0.402907	0.154548	0.009064
Novel_004028	XLOC_04389.	transcript	0.01089	0	0	0.008404	0	0.03116	0	0	0
Novel_004029	XLOC_04389.	transcript	4.20E-05	0	0.069171	0	2.00E-05	0.00113	1.30E-05	0.000245	0.075868
Novel_004030	XLOC_04390.	transcript	0	0	0	0.139071	0	0.19233	0	0.026635	0
Novel_004031	XLOC_04390.	transcript	0.061682	0	0	0	0.045107	0.055816	0.269721	0.116514	0.018275
Novel_004032	XLOC_04390.	transcript	0.60075	0.827934	0.712032	0.945493	0.879639	0.711042	0.656937	0.826068	1.007035
Novel_004033	XLOC_04391.	transcript	0.052024	0	0.073604	0.054723	0.081633	0	0.081625	0.056485	0
Novel_004034	XLOC_04391.	transcript	0.049124	0.042822	0.059198	0.042139	0.087185	0.057299	0.076587	0.062994	0.12504
Novel_004035	XLOC_04391.	transcript	0.069506	0	0.075733	0.035957	0.097124	0.005946	0.126768	0.081361	0
Novel_004036	XLOC_04391.	transcript	0.031239	0	0.069818	0.040444	0.071905	0.00309	0.129166	0.084118	0.071813
Novel_004037	XLOC_04391.	transcript	0.052628	0	0.036811	0.039568	0.061874	0.078499	0.058888	0.028782	0
Novel_004038	XLOC_04391.	transcript	0.124667	0	0.064538	0.039633	0.075542	0.039433	0.075004	0.041522	0.137453
Novel_004039	XLOC_04391.	transcript	0.06683	0.01215	0.062301	0.032965	0.104668	0.013068	0.110838	0.075584	0.039341
Novel_004040	XLOC_04391.	transcript	0	0	0	0	0	0	0	0	0
Novel_004041	XLOC_04391.	transcript	0.0441	0	0.060495	0.037384	0.078078	0	0.079715	0.026975	0
Novel_004042	XLOC_04391.	transcript	0.039166	0	0.040955	0.045509	0.083407	0	0.080053	0.039199	0
Novel_004043	XLOC_04391.	transcript	0.055323	0	0.047333	0.048213	0.218172	0	0.079542	0.062039	0
Novel_004044	XLOC_04391.	transcript	0.074047	0	0.029668	0.043823	0.099384	0	0.060087	0.026752	0
Novel_004045	XLOC_04391.	transcript	0.050372	0	0.060322	0.047513	0.139917	0	0.08292	0.078215	0
Novel_004046	XLOC_04391.	transcript	0.040794	0	0.045666	0.040232	0.060586	0.064545	0.076563	0.028492	0.125501
Novel_004047	XLOC_04391.	transcript	0.091745	0	0.069446	0.046492	0.076857	0.172323	0.072566	0.059256	0
Novel_004048	XLOC_04391.	transcript	0.060908	0	0.05284	0.041966	0.089349	0.111912	0.073444	0.065043	0
Novel_004049	XLOC_04391.	transcript	0.03189	0	0.046952	0.039517	0.051975	0	0.135636	0.097472	0
Novel_004050	XLOC_04391.	transcript	0.065654	0	0.065127	0.047587	0.0803	0	0.07767	0.061169	0
Novel_004051	XLOC_04392.	transcript	1.220196	0.536601	2.625275	2.333481	2.418735	2.844223	2.803602	1.715308	3.375095
Novel_004052	XLOC_04392.	transcript	0	0	0	0	0	0	0	0	0
Novel_004053	XLOC_04393.	transcript	0.108391	0.204023	0.014531	0.164978	0	0	0.176027	0	0.079465
Novel_004054	XLOC_04393.	transcript	0.043314	0.017949	0.045792	0	0	0	0.032394	0.024716	0

Novel_004055	XLOC_04394	transcript	0	0.075809	0.030691	0.025611	0.030192	0	0	0	0
Novel_004056	XLOC_0439(	transcript	2.754976	0.217453	1.175525	0.666598	1.180995	1.275328	1.97924	1.308657	1.044222
Novel_004057	XLOC_0439{	transcript	0.879395	0.807467	0.852958	0.633945	0.871773	0.637708	1.194654	0.872929	1.449596
Novel_004058	XLOC_0439{	transcript	0.47302	0.737219	0.36984	0.474739	0.774618	0.925679	0.318714	0.333704	0.565952
Novel_004059	XLOC_0439{	transcript	0.010983	0.013807	0.061933	0	0.035312	0.028592	0.180808	0.157874	0
Novel_004060	XLOC_0439(	transcript	0.374146	0.98289	0.289898	0.367612	0.475849	0.407401	0.579283	0.402423	0.392327
Novel_004061	XLOC_0439(	transcript	0	0	0	0	0	0.007012	0	0	0
Novel_004062	XLOC_0439(	transcript	0	0.313351	0	0	0	0	0.021362	0.385865	0
Novel_004063	XLOC_0439(	transcript	0.60983	0.225076	0.485772	0.582389	0.131881	0.477326	0.521589	0.621956	0.390187
Novel_004064	XLOC_0439(	transcript	0	0.69629	0.036173	0	0.567391	0	0	0	0
Novel_004065	XLOC_0439(	transcript	0.113921	0.044893	0.045163	0.01384	0.019628	0.372186	0.032181	0.024723	0.001376
Novel_004066	XLOC_0440(	transcript	0	0	0.253214	0.037032	0.067498	0	0.076573	0	0.116702
Novel_004067	XLOC_0440(	transcript	0	0.07876	0.09486	0	0.142624	0.106068	0.183366	0	0.168934
Novel_004068	XLOC_04402	transcript	1.788068	2.717959	3.502558	3.303384	2.336602	1.921243	2.544586	2.008945	3.665433
Novel_004069	XLOC_04402	transcript	1.393858	1.86585	0.137415	1.736885	1.476996	1.187573	1.665232	0	0.837033
Novel_004070	XLOC_0440:	transcript	0.696005	0.467471	0.638615	0.654574	0.740424	0.718152	0.709501	0.804664	0.534543
Novel_004071	XLOC_0440:	transcript	0	0.162956	0	0.254817	0	0	0	0	0.141046
Novel_004072	XLOC_04404	transcript	0	1.147672	0	0.437475	0.187447	0.274783	0.381367	0.189205	0.015455
Novel_004073	XLOC_04404	transcript	0.696772	1.419626	0.989661	0.09472	0.17063	0	0.149346	0	0.481329
Novel_004074	XLOC_04404	transcript	0	0.00862	0	0.005932	0.052113	0.690256	0.030992	1.333145	0
Novel_004075	XLOC_04404	transcript	0	0.006004	0	0	0	0.33709	0	0	0
Novel_004076	XLOC_04404	transcript	0	0	0.792587	0.885067	0	0.89608	0.217507	0.814722	0.477252
Novel_004077	XLOC_04404	transcript	0.960671	0.025684	0.624948	0.027685	0.541075	0	0.332013	0.900804	0.009419
Novel_004078	XLOC_04411	transcript	0	0	0.002708	0.581632	0	0.004473	0.008589	0.370457	0.006865
Novel_004079	XLOC_04411	transcript	0.155257	0.120797	0.331566	0.148412	0.292981	0.278577	0.296354	0.126093	0.202526
Novel_004080	XLOC_04412	transcript	0.177518	0.279172	0.638295	0.11805	0.417148	0.137285	0.354902	0.227276	0.205259
Novel_004081	XLOC_0441(	transcript	0.152634	0.068779	0.185174	0.274682	0.37932	0.144403	0.277568	0.181836	0.270601
Novel_004082	XLOC_0441{	transcript	6.60E-05	0	0.000126	4.60E-05	1.886992	0.000137	4.30E-05	8.30E-05	0
Novel_004083	XLOC_0441{	transcript	2.456537	1.545567	1.808206	1.35095	0.000243	1.766915	1.945702	1.513955	1.032213
Novel_004084	XLOC_0441{	transcript	0	0	0	0	0	0	0	0	0
Novel_004085	XLOC_0441{	transcript	0	0	0	0	0	0	0	0	0
Novel_004086	XLOC_0441{	transcript	7.70E-05	0.617699	0	0	9.00E-06	0	2.20E-05	0	0
Novel_004087	XLOC_04421	transcript	0.534928	0.276668	0.340345	0.336021	0.373095	0.576274	0.863832	0.639331	0.516725
Novel_004088	XLOC_04421	transcript	0	0	0.195146	0	0	0.083372	0	0	0
Novel_004089	XLOC_04421	transcript	0	0	0	0	0	0	0	0	0
Novel_004090	XLOC_04422	transcript	0.030667	0.092231	0.121988	0.154429	0.062941	0.0185	0.126568	0.079247	0.07281
Novel_004091	XLOC_0442(	transcript	0.508712	0	0.360401	0.205473	0.196456	0.295058	0.538883	0.25079	0.204278
Novel_004092	XLOC_0442(	transcript	0	0	0.547574	0	0	0	0	0	0

Novel_004093	XLOC_04429.	transcript	0.673274	0	0	0.702177	0.391799	0.249837	0.521517	0.128472	0.177623
Novel_004094	XLOC_04430.	transcript	0.306306	0.69458	0.187187	0.216197	0.229664	0.224452	0.311475	0.269711	0.263105
Novel_004095	XLOC_04430.	transcript	0.008665	0.017949	0.009271	0.008874	0.007152	0	0.00251	0	0.011226
Novel_004096	XLOC_04430.	transcript	0	0.002776	0	0	0	0	0	0.041658	0
Novel_004097	XLOC_04430.	transcript	0	0	0	0	0	0	0	0	0.057823
Novel_004098	XLOC_04432.	transcript	0.142575	0.21788	0.325811	0.109871	0.243067	0.077287	0.204643	0.225524	0.263827
Novel_004099	XLOC_04433.	transcript	0	0	0	0.137432	0	0.08205	0.00383	0.182718	0
Novel_004100	XLOC_04433.	transcript	0.672115	1.102087	0.697438	0.516482	0.547611	0.603208	0.772494	0.720452	0.655329
Novel_004101	XLOC_04434.	transcript	0	0.337247	0	0	0	0.031157	0.189433	0.144048	0
Novel_004102	XLOC_04435.	transcript	0.699334	0.504798	0.826296	0.756852	0.508977	0.621553	0.861431	0.643539	0.619526
Novel_004103	XLOC_04435.	transcript	0.098018	0.234702	0.010228	0	0.19316	0.186004	0	0.110199	0.118224
Novel_004104	XLOC_04435.	transcript	0	0	0	0.000333	0	0.000228	0.000408	0.006904	0
Novel_004105	XLOC_04436.	transcript	0	0.030983	0	0.779614	3.292776	2.561138	1.070615	0.41685	0.127305
Novel_004106	XLOC_04437.	transcript	0.143097	0.582649	0.143106	0.098333	0.120001	0.109997	0.155965	0.03732	1.310245
Novel_004107	XLOC_04437.	transcript	0	0	0	0	0.016428	0	0	0	0
Novel_004108	XLOC_04437.	transcript	0.518085	0.229508	0.424064	0.316414	0.655496	0.331355	0.365096	0.548229	0.344791
Novel_004109	XLOC_04437.	transcript	0.093327	0.231872	0.169862	0.27071	0.099483	0.288936	0.455011	0.209183	0
Novel_004110	XLOC_04437.	transcript	0	0.051201	0.099309	0	0.010883	0.109056	0.007564	0.076719	0
Novel_004111	XLOC_04437.	transcript	0	0.054988	0.11589	0	0	0.112115	0	0.083957	0.284867
Novel_004112	XLOC_04437.	transcript	0.033692	0.497615	0.013702	0.171483	0.09678	0.031813	0.010939	0.003272	0.03396
Novel_004113	XLOC_04438.	transcript	0.860565	1.130512	1.084748	0.955026	1.108253	0.864771	1.303547	0.978463	0.688542
Novel_004114	XLOC_04439.	transcript	0	0.224847	0.007857	0	0.306935	0.44357	0	0	0
Novel_004115	XLOC_04439.	transcript	0.037849	0.003633	0.001635	0.001499	0.109705	0.005133	0.227201	0.313195	0.370446
Novel_004116	XLOC_04439.	transcript	1.049038	0.637909	0.992754	0.851062	0.740553	0.814222	1.326974	1.100054	0.615485
Novel_004117	XLOC_04439.	transcript	0.888936	0.164857	0.622758	0.605626	0.544541	0.843913	0.66133	0.904531	0.32315
Novel_004118	XLOC_04439.	transcript	0	0.494194	0	0	0	0	0	0	0.106575
Novel_004119	XLOC_04439.	transcript	0	1.227813	0.074385	0	0	0	0.061607	0	0
Novel_004120	XLOC_04440.	transcript	2.633462	5.567026	1.75572	2.428741	2.589107	2.611443	3.101388	2.976363	2.343468
Novel_004121	XLOC_04440.	transcript	0.303302	0.827392	0.411606	0.309539	0.360636	0.269082	0.22123	0.244054	0.129532
Novel_004122	XLOC_04444.	transcript	1.011811	1.66092	1.401048	0.915362	1.088502	1.117939	1.439712	1.152617	0.83035
Novel_004123	XLOC_04447.	transcript	0.80547	0.921381	1.099078	1.210184	1.150021	1.138618	0.929055	1.052128	0.779105
Novel_004124	XLOC_04450.	transcript	0.140183	0.029761	0.132896	0.128702	0.143678	0.195265	0.130529	0.111209	0.153944
Novel_004125	XLOC_04450.	transcript	0.147922	0.019644	0.174145	0.164284	0.169047	0.168762	0.111887	0.097772	0.120945
Novel_004126	XLOC_04450.	transcript	0.150581	0.014267	0.159785	0.090366	0.147433	0.159382	0.136991	0.077846	0.117304
Novel_004127	XLOC_04457.	transcript	0.100759	0.460886	0.362843	0.006354	0.205521	0.093434	0.20655	0.02857	0.034118
Novel_004128	XLOC_04458.	transcript	0.089178	0.11222	0.329353	0.479837	0.159618	0.188293	0.212092	0.263293	0
Novel_004129	XLOC_04458.	transcript	0.285536	0.389429	0.198139	0.259395	0.270849	0.15797	0.416241	0.39632	0.278601
Novel_004130	XLOC_04462.	transcript	0	0	0.054096	0	0	0	0.020257	0	0.199314

Novel_004131	XLOC_04462.	transcript	0.009807	0	0.003019	0.002575	0.012243	0	0	0.003567	0
Novel_004132	XLOC_04462.	transcript	0	0	0.00067	0	0	0.022962	0	0.012603	0
Novel_004133	XLOC_04462.	transcript	0.127258	0.006453	0.233508	0.103925	0.013716	0.167224	0.141507	0.139371	0.013821
Novel_004134	XLOC_04462.	transcript	0.024209	0.005922	0.013029	0.058513	0.180532	0.018821	0.014328	0.024505	0.005848
Novel_004135	XLOC_04462.	transcript	0.001069	0	0	0.122469	0.0416	0.00027	0.060873	0.000378	0
Novel_004136	XLOC_04462.	transcript	0.093444	0	0.115275	0.089081	0.079588	0.100804	0.072661	0.181152	0.10708
Novel_004137	XLOC_04469.	transcript	0.074448	0	0.085134	0.051427	0.181416	0.126818	0.094928	0.027719	0.188318
Novel_004138	XLOC_04475.	transcript	0	0	0	0	0	0	0	0	0
Novel_004139	XLOC_04475.	transcript	1.090273	1.672428	0.686085	1.315689	1.078678	1.804317	1.059493	1.667765	1.047321
Novel_004140	XLOC_04480.	transcript	0.181022	0.266115	0.143534	0.189519	0.023143	0	0.213118	0.090515	0.142703
Novel_004141	XLOC_04480.	transcript	0.131504	0.187315	0.138763	0.078725	0.152241	0	0.090317	0.112011	0.222418
Novel_004142	XLOC_04480.	transcript	0	0	0	0	0	0	0.174656	0	0
Novel_004143	XLOC_04481.	transcript	0.124563	0.192754	0.178483	0.114582	0.161289	0.080555	0.170792	0.211062	0.24981
Novel_004144	XLOC_04481.	transcript	0	0	0	0	0	0	0	0	0
Novel_004145	XLOC_04481.	transcript	0.84282	0	0	0	0.97385	0.17492	0	0	0
Novel_004146	XLOC_04481.	transcript	0.000795	1.310184	0.020325	1.060578	0	1.222497	1.03381	1.475948	0.8701
Novel_004147	XLOC_04481.	transcript	0	0	0	0.343098	0	0	0	0	0
Novel_004148	XLOC_04481.	transcript	0	0	1.230045	0	0	0	0	0	0
Novel_004149	XLOC_04481.	transcript	0	0.143624	0	0	0	0	0.258788	0.19819	0.087313
Novel_004150	XLOC_04483.	transcript	0.190414	0.075597	0.251648	0.293333	0.332387	0.259275	0.507698	0.440194	0.269057
Novel_004151	XLOC_04483.	transcript	0.399807	0.155963	0.509804	0.54169	0.553829	0.846409	0.293382	0.497816	0.43058
Novel_004152	XLOC_04486.	transcript	0.377814	0.724154	0.43292	0.506592	0.332149	0.637561	0.524172	0.23609	0.338514
Novel_004153	XLOC_04486.	transcript	0	0	0	0	0	0	0	0	0
Novel_004154	XLOC_04486.	transcript	0.23287	0	0	0.02767	0.201722	0	0.296143	0.538184	0
Novel_004155	XLOC_04486.	transcript	0	0	0	0	0	0.005699	0	0	0
Novel_004156	XLOC_04486.	transcript	0	0.292943	0	0	0	0	0	0	0
Novel_004157	XLOC_04486.	transcript	0	0	0	0	0.002803	0.005132	0.008399	0	0
Novel_004158	XLOC_04486.	transcript	0.326676	0.977554	0.684506	0.799926	0.526386	0.932588	0.459029	0.236124	0.507731
Novel_004159	XLOC_04486.	transcript	0	0	0	0	0	0.011953	0	0	0
Novel_004160	XLOC_04486.	transcript	0.748365	0	0	0	0	0	0.759631	0.731822	0
Novel_004161	XLOC_04490.	transcript	0.085411	0.009147	0.182823	0.217266	0.137023	0.161506	0.18579	0.165808	0.019358
Novel_004162	XLOC_04495.	transcript	0	0	0.278035	0.116115	0	0	3.40E-05	0	0
Novel_004163	XLOC_04495.	transcript	0.068677	0.140683	0.036303	0.116097	0.141783	0.413886	0.069722	0.147517	0.074172
Novel_004164	XLOC_04495.	transcript	0.159581	0.07158	0.364402	0.354245	0.309783	0.224717	0.297196	0.411168	0.416948
Novel_004165	XLOC_04496.	transcript	2.275521	3.566781	2.012746	1.720938	1.879015	2.053481	2.480141	2.813646	2.471029
Novel_004166	XLOC_04496.	transcript	0.733535	1.17871	1.044363	0.645823	0.908095	0.555741	1.412045	0.469345	0.358144
Novel_004167	XLOC_04496.	transcript	0.441316	0.088316	0.576322	0.510543	0.507733	0.620493	0.558315	0.418573	0.456961
Novel_004168	XLOC_04496.	transcript	0.003137	0	0	0	0.018606	0	0	0	0.008136

Novel_004169	XLOC_04496	transcript	0.518565	0.302856	0.151483	0.314249	0.25373	0.264938	0.323664	0.311935	0.28305
Novel_004170	XLOC_04496	transcript	0.005529	0	0	0	0	0	0.022139	0.013121	0.008723
Novel_004171	XLOC_04496	transcript	0.622528	0	0.613153	0.375299	0.741793	0.534789	0.689134	0.689489	0.307331
Novel_004172	XLOC_04496	transcript	0.07873	0.023466	0.221117	0.120826	0.010654	0.031285	0.207264	0.077446	0.144892
Novel_004173	XLOC_04496	transcript	0.130919	0.003489	0.26074	0.190287	0.39176	0.161666	0.193784	0.150453	0.119796
Novel_004174	XLOC_04496	transcript	0.363703	0.38977	0.261411	0.312205	0.247949	0.268003	0.397655	0.188698	0.271951
Novel_004175	XLOC_04496	transcript	0.035073	0	0.205847	0.138041	0.122769	0.175241	0.166144	0.110065	0.141148
Novel_004176	XLOC_04497	transcript	0.701418	1.579762	0.67081	0.977019	0.632534	0.693761	0.813086	1.139872	0.654253
Novel_004177	XLOC_04498	transcript	6.180651	1.244385	0.269035	0.416897	0.597045	4.663245	4.29967	1.790607	10.27032
Novel_004178	XLOC_04498	transcript	0.187817	0	5.872644	4.415091	6.219917	0.009589	2.130933	1.387037	0.177381
Novel_004179	XLOC_04498	transcript	0.00142	0.046713	0.000789	1.66474	0.000761	0.978621	4.353717	4.386471	0
Novel_004180	XLOC_04498	transcript	0	0	0.244737	0.393482	0.441072	0.402007	2.417176	0.626578	0
Novel_004181	XLOC_04498	transcript	0.293674	0.234028	0.143603	0.439572	0.711385	0.146285	0.121246	0.578241	0.140995
Novel_004182	XLOC_04499	transcript	0	0	0	0	0	0	0	0	0
Novel_004183	XLOC_04500	transcript	0	0	0	0	0	0	0	0	0
Novel_004184	XLOC_04501	transcript	0.787003	0.073477	0.264038	0	0.47729	0.105316	0.322934	0.534838	0
Novel_004185	XLOC_04501	transcript	1.291047	0.590238	1.569554	2.095594	1.892957	2.063038	2.424783	2.760291	2.631937
Novel_004186	XLOC_04503	transcript	0.185129	0.378875	0.414247	0.384236	0.34758	0.393036	0.433644	0.513861	0.573282
Novel_004187	XLOC_04503	transcript	0.226592	0.106974	0.222763	0.168796	0.156801	0.055147	0.22248	0.118444	0.04899
Novel_004188	XLOC_04504	transcript	0.306413	0.101354	0.193533	0.192362	0.134674	0.192479	0.177862	0.107801	0.027865
Novel_004189	XLOC_04504	transcript	0	0.270582	0.165205	0.279576	0.321075	0.0989	0.312183	0.413306	0.2918
Novel_004190	XLOC_04504	transcript	0	8.50E-05	0	0	0.00325	0	0.004569	0.003231	0.001534
Novel_004191	XLOC_04504	transcript	0	0	0	0	0	0	0.26141	0.40427	0
Novel_004192	XLOC_04504	transcript	0.764405	0	0.29056	0.762516	0.447611	0.439199	0.467664	0.282529	0
Novel_004193	XLOC_04504	transcript	0.423622	0	0.312798	0.334566	0	0.29005	0.541191	0.724659	0.31964
Novel_004194	XLOC_04504	transcript	0	0.826458	0	0	0	0	0	0	0
Novel_004195	XLOC_04504	transcript	0.10561	0.514974	0	0.249407	0	0.258864	0.077126	0.068176	0.0371
Novel_004196	XLOC_04504	transcript	0	0	2.90E-05	0	0.07782	0.164684	0.007004	0	0
Novel_004197	XLOC_04504	transcript	0	0.23956	0	0.178338	0.173609	0.007773	0	0.310435	0
Novel_004198	XLOC_04504	transcript	0.404213	0	0.541332	0.196982	0.086394	0.145777	0.764114	0.179097	0.483425
Novel_004199	XLOC_04505	transcript	0.512614	0.045826	0.526004	0.288125	0.487912	0.336628	0.464042	0.39633	0.297556
Novel_004200	XLOC_04505	transcript	0	0	0	0.016538	0	0	0	0	0
Novel_004201	XLOC_04507	transcript	9.894086	10.91776	15.07839	23.81711	16.20029	20.29053	11.1039	10.77679	13.8667
Novel_004202	XLOC_04511	transcript	0.266923	0	0.42224	0.19688	0.308218	0.445602	0.181146	0.139459	0
Novel_004203	XLOC_04528	transcript	0.390182	0.207873	0.379407	1.222569	0.875418	0.723576	0.524503	0.484246	0.598122
Novel_004204	XLOC_04529	transcript	0.131108	0.021135	0	0.068121	0	0	0	0	0.039789
Novel_004205	XLOC_04529	transcript	0.421508	0.503149	0.314808	0.393588	0.335847	0.485239	0.413751	0.459718	0.551055
Novel_004206	XLOC_04533	transcript	0.536817	0.474229	0.546173	0.709317	0.478474	0.577367	0.458421	0.427228	0.562339

Novel_004207	XLOC_04533.	transcript	0	0.073431	0	0	0	0	0	0.000793	0
Novel_004208	XLOC_04535.	transcript	0.892628	1.173467	0.479412	0.65097	0.434298	0.463132	1.101278	0.973655	1.532579
Novel_004209	XLOC_04535.	transcript	0	0	0.465874	0	0.247508	0	0	0	0
Novel_004210	XLOC_04535.	transcript	0.015718	0.004883	0.123327	0.005204	0.75454	0	0	0	0
Novel_004211	XLOC_04538.	transcript	0.376774	0.825724	0.530262	0.357755	0.330043	0.482224	0.423023	0.467623	0.236201
Novel_004212	XLOC_04540.	transcript	0.117697	0.045109	0.066088	0.05474	0.023529	0.107872	0.012866	0.419273	0.215512
Novel_004213	XLOC_04540.	transcript	0.086805	0	0.085432	0.071735	0.712319	0.119006	0.340547	0.211937	0
Novel_004214	XLOC_04543.	transcript	0.09599	0.308659	0.187513	0.162454	0.245551	0.045736	0.191812	0.346606	0.290266
Novel_004215	XLOC_04543.	transcript	0.149791	0.103981	0.057586	0.0245	0.096008	0.088283	0.126758	0.085606	0
Novel_004216	XLOC_04543.	transcript	0.508129	0.247683	0.287983	0.601908	0.322276	0.228936	0.87808	0.57387	0.408015
Novel_004217	XLOC_04549.	transcript	0.122387	0.478894	0.129266	0.117101	0.161351	0.142718	0.157401	0.071467	0.376991
Novel_004218	XLOC_04549.	transcript	0	0.052009	0	0	0	0	0	0.028414	0.017886
Novel_004219	XLOC_04550.	transcript	0.294656	0.032496	0.190335	0.221258	0.470093	0.519767	0.790633	0.415604	0.35145
Novel_004220	XLOC_04550.	transcript	0.972999	0.764885	1.272572	1.001677	0.819865	0.914201	1.143923	0.986892	1.114877
Novel_004221	XLOC_04550.	transcript	0.70552	1.120874	0.275557	0.358423	0.501829	0.587397	1.263867	0.620574	0.594658
Novel_004222	XLOC_04550.	transcript	0	0	0	0	0.024042	0	0	0	0
Novel_004223	XLOC_04557.	transcript	0.177127	0.052683	0.293524	0.185726	0.321231	0.214071	0.262626	0.37705	0.309629
Novel_004224	XLOC_04557.	transcript	0	0	0.175589	0	0	0	0	0	0
Novel_004225	XLOC_04558.	transcript	1.044958	2.35973	1.946505	2.513384	2.524862	2.195102	1.783893	2.50162	1.77703
Novel_004226	XLOC_04559.	transcript	0.585148	0.030784	0.408704	2.412078	0.848493	0.658396	1.043583	0.727251	0.118096
Novel_004227	XLOC_04559.	transcript	0	0	0	0	0	0	0.288089	0	0
Novel_004228	XLOC_04562.	transcript	0.668893	0.608636	0.466765	0.637847	0.566444	0.891537	0.649226	0.539773	0.768013
Novel_004229	XLOC_04563.	transcript	0	0	0.583668	2.860934	1.214342	0.557179	0.819365	1.504376	0
Novel_004230	XLOC_04563.	transcript	0.014448	0	0	0	0	0	0	0	0
Novel_004231	XLOC_04563.	transcript	0.349455	0.162582	0	0	0	0	0	0	0.681429
Novel_004232	XLOC_04563.	transcript	0	0	0	0.001377	0	0	0	0.128892	0
Novel_004233	XLOC_04570.	transcript	0.027725	0	0.073301	0.117148	0.188445	0.129457	0.165101	0.073392	0.080193
Novel_004234	XLOC_04570.	transcript	0.005136	0	0.079009	0.065539	0.155506	0.088804	0.143329	0.071342	0.062688
Novel_004235	XLOC_04570.	transcript	0	0	0.082002	0.05601	0	0	0	0.060979	0.065721
Novel_004236	XLOC_04570.	transcript	0.01002	0	0.074124	0.064017	0	0	0	0.063239	0.128716
Novel_004237	XLOC_04570.	transcript	0.080439	0.004621	0.072316	0.06106	0.12031	0.131549	0.170582	0.160309	0
Novel_004238	XLOC_04570.	transcript	0	0.002522	0.06988	0	0.106788	0	0.077717	0.071361	0.003137
Novel_004239	XLOC_04570.	transcript	0	0	0	0	0	0	0	0.065531	0
Novel_004240	XLOC_04570.	transcript	0	0	0.131281	0.103569	0	0	0	0.115126	0.195866
Novel_004241	XLOC_04570.	transcript	0.07046	0	0.1278	0.08632	0.207109	0.210816	0.308027	0.133732	0.088714
Novel_004242	XLOC_04570.	transcript	0.112174	0	0.118516	0.097733	0	0	0	0.106117	0.099325
Novel_004243	XLOC_04570.	transcript	0.447241	0.076189	0.16947	0.100038	0.157535	0.383278	0.27085	0.174427	0
Novel_004244	XLOC_04570.	transcript	0.037627	0	0.093033	0.051299	0.064951	0	0.102951	0.065886	0.083904

Novel_004245	XLOC_04570.	transcript	0.239261	0	0.13558	0.103476	0	0	0	0.135687	0
Novel_004246	XLOC_04570.	transcript	0	0.007796	0.13736	0.14776	0.282327	0.220687	0.421328	0.161113	0.120567
Novel_004247	XLOC_04571.	transcript	0	0.04189	0	0	0	0	0	0.09159	0
Novel_004248	XLOC_04571.	transcript	0	0.163198	0	0	0	0	0	0	0
Novel_004249	XLOC_04571.	transcript	0	0	0.124246	0	0	0	0	0	0
Novel_004250	XLOC_04571.	transcript	0	0	0	0	0	0	0	0	0.024431
Novel_004251	XLOC_04571.	transcript	0.036384	0	0.011605	0.015223	0	0	0.015573	0.005596	0
Novel_004252	XLOC_04571.	transcript	0.271013	0	0.259856	0	0	0	0.175999	0	0.048146
Novel_004253	XLOC_04571.	transcript	0	0.000356	0	0	0	0	0.108139	0	0.197771
Novel_004254	XLOC_04571.	transcript	0.158768	0	0	0.234269	0	0	0	0	0
Novel_004255	XLOC_04571.	transcript	0.109327	0	0.020448	0.066924	0.129733	0.090746	0.010874	0.069647	0.004493
Novel_004256	XLOC_04573.	transcript	0.022575	0	0.074661	0	0.058969	0	0.061461	0.0026	0
Novel_004257	XLOC_04574.	transcript	5.513321	12.31878	5.621789	5.201162	7.164024	6.195297	10.72669	8.194721	6.611642
Novel_004258	XLOC_04574.	transcript	0.634152	0.631452	0.33737	0	0.003422	0.001451	0.00926	0.006101	0
Novel_004259	XLOC_04575.	transcript	0.755445	0.4401	0.552312	0.279982	0.401303	0.483063	0.781467	0.002055	0.455102
Novel_004260	XLOC_04575.	transcript	0.008563	0	0	0.062541	0.019018	0	0.026608	0	0
Novel_004261	XLOC_04575.	transcript	0	6.50E-05	0	0	0	0	0	0.097853	0
Novel_004262	XLOC_04575.	transcript	0	0	0	0	0	0	0	0.004876	0
Novel_004263	XLOC_04575.	transcript	0	0	0	0.076568	0	0	0	0.056306	0
Novel_004264	XLOC_04575.	transcript	0	0	0	0	0.115693	0.163007	0.062855	0.054584	0
Novel_004265	XLOC_04575.	transcript	0	0	0	0	0	0	0.005491	0	0
Novel_004266	XLOC_04575.	transcript	0.213072	0	0	0	0	0	0	0	0
Novel_004267	XLOC_04575.	transcript	0	0.167435	0	0	0	0	0	0	0.121909
Novel_004268	XLOC_04576.	transcript	0.071	0	0	1.353496	0	0	0	0	0
Novel_004269	XLOC_04576.	transcript	0.693968	0	1.591983	0.653775	1.558551	1.432926	1.465292	1.349643	1.545214
Novel_004270	XLOC_04579.	transcript	0.474897	0.160061	0.399132	0.24177	0.29625	0.299235	0.73409	0.177508	0.129449
Novel_004271	XLOC_04580.	transcript	0.202814	1.225978	0.343318	0.132394	0.291821	0.273469	0	0.339929	0.468732
Novel_004272	XLOC_04580.	transcript	0.079172	1.397464	0.01339	0	0	0	0.613928	0	0
Novel_004273	XLOC_04580.	transcript	0	0.016482	0	0	0	0	0.000801	0	0
Novel_004274	XLOC_04580.	transcript	0	0	0	0	0	0	0	0	0
Novel_004275	XLOC_04580.	transcript	0.557514	0	0.006756	0	0.435555	0	0	0	0
Novel_004276	XLOC_04580.	transcript	0	0	0.036925	0	0.070232	0.005146	0	0.398556	0
Novel_004277	XLOC_04580.	transcript	0.055314	0.116591	0.40806	0.396142	0.148278	0.359019	0.657183	0.281653	0.301379
Novel_004278	XLOC_04581.	transcript	0.263219	6.238293	0.244509	0.196597	0.226652	0.252527	0.492477	0.232652	0.373338
Novel_004279	XLOC_04581.	transcript	0.041778	0.269548	0	0.07446	0	0	0	0	0.05446
Novel_004280	XLOC_04582.	transcript	0	0.716882	0	0	0	0	0	0	0
Novel_004281	XLOC_04582.	transcript	0.007422	0.020788	0	0	0	0	0	0.007569	0.012067
Novel_004282	XLOC_04582.	transcript	0	0	0	0	0	0	0	0	0

Novel_004283	XLOC_04582.	transcript	0	0	0	0	0	0	0	0	0
Novel_004284	XLOC_04582.	transcript	0.050075	0.020373	0.041578	0.052619	0.034144	0.050347	0.045102	0.078811	0.049454
Novel_004285	XLOC_04582.	transcript	0.013972	0.783144	0.156758	0.169556	0.178814	0.067428	0.093074	0.152422	0.092356
Novel_004286	XLOC_04582.	transcript	0	0	0	0	0	0	0	0	0
Novel_004287	XLOC_04582.	transcript	0	1.198877	0.078054	0	0	0	0.186922	0.011007	0.16647
Novel_004288	XLOC_04582.	transcript	0.330919	0.196624	0.426753	0.420012	0.335318	0.365494	0.456231	0.323378	0.353062
Novel_004289	XLOC_04585.	transcript	0.38364	0.188906	0.100181	0.155799	0.286033	0.220372	0.364437	0.36441	0.274688
Novel_004290	XLOC_04585.	transcript	0.111988	0.049487	0.076166	0.018674	0.076984	0.035736	0.146291	0.078667	0.009226
Novel_004291	XLOC_04585.	transcript	0.130827	0	0.015317	0	0.044306	0.154664	0.086684	0.0579	0.01717
Novel_004292	XLOC_04587.	transcript	0.027527	0.129933	0.013443	0.159299	0.289358	0	0.080783	0.076252	0
Novel_004293	XLOC_04587.	transcript	0.010279	0.453777	0.134154	0.699662	0.114536	0.016793	0.235346	0.156549	0.000538
Novel_004294	XLOC_04587.	transcript	0.713903	0	0.46091	0.051925	0.483991	0.220261	0.00971	0.682761	0.355337
Novel_004295	XLOC_04587.	transcript	0.86802	0.241359	0.562035	0.286917	0.454288	0.579971	0.679435	1.046197	0.258027
Novel_004296	XLOC_04587.	transcript	0.102793	0.447698	0.102032	0.00153	0.350283	0.451953	0	0.320889	0.003659
Novel_004297	XLOC_04587.	transcript	0.1982	0	0.177082	0.467882	0.136785	0.127418	0.330453	0.095172	0
Novel_004298	XLOC_04587.	transcript	0.058678	0.174033	0.000462	0.090501	0.00045	0.002607	0.00206	0.089495	0.000425
Novel_004299	XLOC_04587.	transcript	0.004822	0	0.002185	0.01012	0.002007	0.01497	0.001273	0.001499	0.327357
Novel_004300	XLOC_04587.	transcript	0.232334	0	0.184141	0.142321	0.131631	0.162645	0.144891	0.08123	0
Novel_004301	XLOC_04598.	transcript	0.527312	0.26475	0.428003	0	0.265471	0.443209	0	0	0.418299
Novel_004302	XLOC_04598.	transcript	0	0.256955	0	0.590381	0	0.049863	0.279299	0.258204	0.106265
Novel_004303	XLOC_04599.	transcript	0.256436	0.054675	0.365543	0.725309	0.478709	0.386734	0.652459	0.319064	0.524037
Novel_004304	XLOC_04599.	transcript	0	0.114708	0.015126	0.048069	0	0.041972	0	0.142015	0.041529
Novel_004305	XLOC_04599.	transcript	0	0.273075	0.110587	0.198631	0	0.608475	0.039848	0.056909	0
Novel_004306	XLOC_04600.	transcript	0.053036	0.032054	0	0.018435	0.070384	0.048449	0.317019	0.310782	0.111792
Novel_004307	XLOC_04600.	transcript	0.526472	0.196521	0.387499	0.352213	0.440279	0.299007	0.287979	0.281231	0.369068
Novel_004308	XLOC_04601.	transcript	0.296944	0.446121	0.289112	0.288045	0.360661	0.475622	0.386142	0.509385	0.717607
Novel_004309	XLOC_04602.	transcript	3.85018	1.392749	1.881187	4.307897	3.109045	3.403195	3.955815	4.213152	2.937985
Novel_004310	XLOC_04605.	transcript	1.452477	0.275997	1.222079	1.237958	1.219016	1.625873	1.481964	1.389727	0.955108
Novel_004311	XLOC_04605.	transcript	0	0	0	0	0	0	0	0	0
Novel_004312	XLOC_04606.	transcript	0	0.01458	0.086617	0.269417	0.412004	0.164024	0.178626	0.175009	0
Novel_004313	XLOC_04606.	transcript	0.11871	0	0	0	0	0	0.131337	0	0.093323
Novel_004314	XLOC_04606.	transcript	0	0	0	0	0.126415	0	0	0.442954	0.127505
Novel_004315	XLOC_04606.	transcript	0.536633	0.22862	0.512385	0.20118	0.184061	0.453636	0.212964	0.287459	0.289159
Novel_004316	XLOC_04606.	transcript	0.372785	0	0	0	0	0	0.329064	0.194397	0
Novel_004317	XLOC_04606.	transcript	0	0.026246	0.274363	0	0.246141	0	0.15255	0.277988	0.209644
Novel_004318	XLOC_04606.	transcript	0.122155	0.041566	0.020101	0.443286	0	0.211289	0	0.197458	0.327471
Novel_004319	XLOC_04606.	transcript	0	0	0	0.108473	0	0	0	0	0
Novel_004320	XLOC_04606.	transcript	0.036142	0.016779	0.169189	0.019254	0.054363	0.020758	0.029614	0.033306	0.029415

Novel_004321	XLOC_04606.	transcript	0.223803	0.093551	0.337743	0.250962	0.359465	0.399815	0.226985	0.133846	0.043056
Novel_004322	XLOC_04606.	transcript	0.190261	0	0	0	0.05989	0	0.697428	0	0
Novel_004323	XLOC_04608.	transcript	0.42448	0.03271	0.11755	0.100744	0.202501	0.29603	0.318741	0.207666	0.188447
Novel_004324	XLOC_04609.	transcript	0.253828	0.045455	0.197785	0.232854	0.258063	0.299165	0.413916	0.276709	0.170975
Novel_004325	XLOC_04617.	transcript	0.440211	0.556382	0.517127	0.583172	0.462172	0.588082	0.48462	0.641575	0.434773
Novel_004326	XLOC_04619.	transcript	0.22863	0.770855	1.048747	1.148446	1.13179	0.979298	1.277544	0.823534	1.186527
Novel_004327	XLOC_04621.	transcript	0.104974	0.100512	0.234789	0.186416	0.345987	0.165811	0.166878	0.264924	0.175499
Novel_004328	XLOC_04622.	transcript	0	0	0	0	0	0	0	0	0
Novel_004329	XLOC_04622.	transcript	0.536799	0.305506	0.349378	0.386823	0.033607	0.243023	0.484682	0.051816	0.360078
Novel_004330	XLOC_04622.	transcript	0	0	0	0	0.376952	0	0	0.263884	0
Novel_004331	XLOC_04622.	transcript	0	0	0	0	0	0	0	0	0
Novel_004332	XLOC_04623.	transcript	0.220238	0.578037	0	0	0.335259	0.049527	0.749284	0.360855	0.003723
Novel_004333	XLOC_04623.	transcript	0	0.015674	0.233917	0.001808	0.197491	0	0	0.198535	0
Novel_004334	XLOC_04623.	transcript	0.350126	0.259815	0.610438	0.624575	0.402323	0.610578	0.356563	0.334627	0.544488
Novel_004335	XLOC_04624.	transcript	1.030098	0.112846	0.801573	0.56951	0.736623	0.667958	1.09496	0.983054	0.822045
Novel_004336	XLOC_04629.	transcript	0.595221	0	0	0	0	0	0	0	0
Novel_004337	XLOC_04629.	transcript	0.028438	0.687011	0.453277	0	0.417732	0	0.643277	0.294004	0
Novel_004338	XLOC_04629.	transcript	7.883082	1.000226	0.959536	5.087121	1.53356	6.583924	1.207282	1.293123	4.118933
Novel_004339	XLOC_04636.	transcript	0.525568	1.226701	1.109784	0.72303	0.912477	0.741856	1.029246	1.200704	0.963378
Novel_004340	XLOC_04642.	transcript	0.463036	0.510759	0.295482	0.328983	0.395745	0.299921	0.476654	0.31418	0.377807
Novel_004341	XLOC_04642.	transcript	0	0.232211	0.056392	0	0	0.056271	0	0.104284	0
Novel_004342	XLOC_04646.	transcript	0.355969	0.309258	0.784872	0.465327	0.287734	0.454557	0.358781	0.778589	0.366299
Novel_004343	XLOC_04647.	transcript	0.011038	0	0	0	0.029732	0.030171	0	0	0
Novel_004344	XLOC_04647.	transcript	0	0	0	0	0.012002	0	0	0	0
Novel_004345	XLOC_04647.	transcript	0.144974	0.101071	0.181344	0.396348	0.197011	0	0	0.141422	0.137474
Novel_004346	XLOC_04647.	transcript	0.078645	0	0.205121	0.207291	0	0.161064	0.251204	0.124216	0
Novel_004347	XLOC_04647.	transcript	0	0	0	0	0	0	0	0	0
Novel_004348	XLOC_04648.	transcript	0.225263	0.127891	0.177463	0.244146	0.191212	0.277157	0.270143	0.243807	0.346124
Novel_004349	XLOC_04648.	transcript	0.02204	0.087029	0	0	0	0	0	0	0
Novel_004350	XLOC_04648.	transcript	0	0	0.02252	0.162907	0.101458	0.153179	0.054412	0.086759	0.193431
Novel_004351	XLOC_04648.	transcript	0	0	0.022128	0	0	0	0	0	0
Novel_004352	XLOC_04649.	transcript	0.097453	0.187567	0.360902	0.122549	0.384353	0.408148	0.107382	0.230662	0.184193
Novel_004353	XLOC_04649.	transcript	0.487817	0.37533	0.547334	0.316801	0.49538	0.395885	0.539316	0.40954	0.419796
Novel_004354	XLOC_04650.	transcript	0.372202	0.693123	0.576435	0.173217	0.053136	0.606212	0.650112	0.284876	0.442024
Novel_004355	XLOC_04650.	transcript	0	0	0.018991	0	0	0	0.356304	0	0
Novel_004356	XLOC_04653.	transcript	0.207444	0.292457	0.304585	0.290063	0.249196	0.430209	0.391149	0.450635	0.41136
Novel_004357	XLOC_04658.	transcript	0.008155	0.043813	0.018089	0.064871	0.294523	0.183096	0	0	0
Novel_004358	XLOC_04658.	transcript	0.786033	0.455054	0.625013	0.111133	0.214913	0.385015	0.430098	0.180905	0.196861

Novel_004359	XLOC_04659.	transcript	0	0	0	2.267017	0	0	0.596958	0	0
Novel_004360	XLOC_04659.	transcript	2.929282	1.477427	2.580619	2.300639	2.309983	5.718219	3.040711	3.114558	7.406585
Novel_004361	XLOC_04660.	transcript	0.013869	0.083533	0.177203	0.206699	0.139968	0.144114	0.241366	0.180528	0.534816
Novel_004362	XLOC_04666.	transcript	2.24982	4.472681	2.521716	0.018958	3.103064	1.929103	1.300217	1.81805	0.594585
Novel_004363	XLOC_04666.	transcript	0.525925	2.979678	0.809993	0.002981	1.615785	0.117192	0.288694	0.004754	0.058728
Novel_004364	XLOC_04666.	transcript	0.099305	0.237366	0.72037	0.093258	0.72779	0.250681	0.168222	0.102829	0.094746
Novel_004365	XLOC_04666.	transcript	0.11577	0.227248	0.024412	0	0.174205	0.145053	0.560926	0	0
Novel_004366	XLOC_04666.	transcript	0.01296	0.434264	0	0.030657	0.500517	0.254921	0	0.052646	0
Novel_004367	XLOC_04666.	transcript	0	0	0	0	0	0	0	0.247738	0
Novel_004368	XLOC_04666.	transcript	0	0.193072	0	0.005838	0.43835	0.116637	0.013142	0	0.019405
Novel_004369	XLOC_04666.	transcript	0.948989	0.064433	0	0.108684	0.195484	0.082332	0	1.052709	0
Novel_004370	XLOC_04666.	transcript	0	8.452939	0	1.034647	0.160679	1.196026	2.39297	0	1.273372
Novel_004371	XLOC_04666.	transcript	0	0.014196	0.083413	0	0	0	0.030515	0.131228	0.638333
Novel_004372	XLOC_04666.	transcript	0	0.488532	0.026279	0.105991	0	0	0	0.068651	1.201587
Novel_004373	XLOC_04667.	transcript	0.536227	0.434988	0.717074	0.781188	0.500414	0.401774	0.95362	0.794929	0.667294
Novel_004374	XLOC_04667.	transcript	0	0	0	0	0.250626	0.185638	0	0	0
Novel_004375	XLOC_04667.	transcript	0.091129	0.22791	0.00748	0.19598	0.007956	0.308694	0	0.009668	0.432331
Novel_004376	XLOC_04667.	transcript	0.245676	0.001766	0.28262	0.05839	0.181912	0	0.383982	0.258015	0
Novel_004377	XLOC_04667.	transcript	0	0.002101	0	0	0.26279	0	0	0	0.003551
Novel_004378	XLOC_04668.	transcript	0.045753	0.075221	0.110307	0.068992	0.031442	0.027042	0.042785	0.076677	0.176999
Novel_004379	XLOC_04669.	transcript	0	0.704537	0	0	0	0	0	0	0
Novel_004380	XLOC_04672.	transcript	0	0.737738	0.048053	0	0.03845	0.017152	0	0.014052	0.014372
Novel_004381	XLOC_04672.	transcript	0	0	0	0	0	0	0.136786	0	0
Novel_004382	XLOC_04672.	transcript	0.016243	1.871065	0.061651	0.051893	0.05186	0.201606	0.283923	0.048019	0.116157
Novel_004383	XLOC_04672.	transcript	0.304029	0	0	0	0	0	0.061441	0	0
Novel_004384	XLOC_04672.	transcript	0	0.473513	0	0	0	0.067275	0.012253	0	0
Novel_004385	XLOC_04672.	transcript	0.208796	0.778113	0.036923	0.007917	0.031788	0.246809	0.086746	0.033603	0.194216
Novel_004386	XLOC_04672.	transcript	0.00347	0.32576	0	0	0.011048	0	0.07788	0	0.285341
Novel_004387	XLOC_04674.	transcript	0	0.053989	0	0	0	0	0	0	0
Novel_004388	XLOC_04674.	transcript	0	0.482986	0	0	0	0.153386	0	0	0
Novel_004389	XLOC_04674.	transcript	0	0.563338	0	0	0	0.052191	0.010748	0	0
Novel_004390	XLOC_04674.	transcript	0	0.26585	0	0	0	0	0.014833	0	0
Novel_004391	XLOC_04675.	transcript	0	0.543376	0.119691	0	0.096346	0.262177	0.100737	0	0.332306
Novel_004392	XLOC_04678.	transcript	0	0	0.08638	0	0.08953	0	0.148991	6.00E-05	0.00481
Novel_004393	XLOC_04678.	transcript	0	0.107239	0	1.252683	0	0	0.315999	0.008322	0.022602
Novel_004394	XLOC_04678.	transcript	0.202375	0.119927	0.108251	0	0.108868	0.311775	0.102082	0.144658	0.112337
Novel_004395	XLOC_04678.	transcript	0	0	0.108291	0	0.123564	0	0.126711	0	0.116729
Novel_004396	XLOC_04678.	transcript	0.120779	0.073845	0.12593	0.078737	0.085096	0.140535	0.081352	0.140496	0.109275

Novel_004397	XLOC_04678.	transcript	0.137962	0.089067	0.093261	0.213878	0.080869	0.16063	0.123988	0.160952	0.150862
Novel_004398	XLOC_04678.	transcript	0.184843	0.10252	0.092905	0.0273	0.092426	0.2055	0.136123	0.279584	0.116836
Novel_004399	XLOC_04679.	transcript	0.894722	0.541229	0.35052	0.002178	0.397053	0	0.652662	0.659443	1.666413
Novel_004400	XLOC_04679.	transcript	1.597273	1.647652	3.265785	2.649201	2.33064	3.085926	5.554351	1.402423	4.675445
Novel_004401	XLOC_04679.	transcript	1.178747	0.219692	0.696816	1.087528	0.901481	1.22099	1.233458	1.349621	3.214132
Novel_004402	XLOC_04679.	transcript	0	0.002481	0.880551	0	0	0	0.713682	0	0
Novel_004403	XLOC_04679.	transcript	0.016245	0.012571	0.758541	0.027833	0.029342	0.019971	0.674638	0.073468	0
Novel_004404	XLOC_04679.	transcript	0.003067	0	0.013898	1.00E-06	2.80E-05	1.00E-04	0.163713	0.008984	0.031767
Novel_004405	XLOC_04679.	transcript	0	0	0	0	9.00E-06	0	0	0	0
Novel_004406	XLOC_04679.	transcript	0.055937	0.012547	0	0.199931	0.111979	0.19654	0.504336	0.126758	0.067664
Novel_004407	XLOC_04679.	transcript	1.219936	0.770908	0	1.556303	1.34387	2.16162	0.090872	1.229846	2.051031
Novel_004408	XLOC_04680.	transcript	0.396819	0.116018	0.371199	0.205337	0.387287	0.336506	0.537064	0.487076	0.645873
Novel_004409	XLOC_04680.	transcript	0.021365	0	0.361323	0.060217	0.302866	0	0.088043	0.03579	0.05064
Novel_004410	XLOC_04680.	transcript	0.776506	0.788816	1.000963	1.19941	1.157735	0.467163	1.183374	1.043716	0.780498
Novel_004411	XLOC_04680.	transcript	0	0	0.162545	0	0	0.353537	0	0.377694	0
Novel_004412	XLOC_04680.	transcript	0.048239	0.024198	0.154854	0	0.075378	0.014609	0.080654	0.108694	0
Novel_004413	XLOC_04680.	transcript	0.115922	0.156428	0	0.06903	0.025559	0	0.367419	0.0704	0
Novel_004414	XLOC_04680.	transcript	0	0	6.20E-05	0.003204	0.005177	0.000627	0.312702	0.000739	0.016754
Novel_004415	XLOC_04680.	transcript	0	0	0	0	0	0	0	0	0
Novel_004416	XLOC_04680.	transcript	0.776734	0	0.867979	0.125637	1.151918	0.157825	0.138177	1.279627	1.103484
Novel_004417	XLOC_04680.	transcript	0	0	0.342971	0	0.012923	0	0	0	0
Novel_004418	XLOC_04680.	transcript	0	0	0	1.649092	0	0	0	0	0
Novel_004419	XLOC_04680.	transcript	0	0	0	0	0	1.536148	1.260516	0	0
Novel_004420	XLOC_04681.	transcript	0.134686	1.819403	0.032605	0.059565	0.010515	0.080085	0.165278	0.091722	0.085284
Novel_004421	XLOC_04681.	transcript	0.787839	0.729978	1.404029	1.343798	1.564419	1.247836	1.159611	0.093208	0.581105
Novel_004422	XLOC_04681.	transcript	0	0.461884	0	0	0	0	0	0.964888	0.530222
Novel_004423	XLOC_04681.	transcript	0	0	0	0	0	0	0	0	0
Novel_004424	XLOC_04681.	transcript	0.967636	0.074855	0.446837	0.437364	0.49914	0.333371	0.37072	0.312181	0.501297
Novel_004425	XLOC_04683.	transcript	0	0.102265	0.287518	0	0	0	0.0605	0	0
Novel_004426	XLOC_04683.	transcript	0.081512	1.048234	0	0.00729	0.122127	0.113807	19.92437	0	0
Novel_004427	XLOC_04683.	transcript	0.349944	0.008662	0.051613	0.140861	0.08102	0.028323	0.046892	0.070359	0
Novel_004428	XLOC_04683.	transcript	0.005216	0.091097	0.047658	0.037007	0.367141	0.068525	0.091061	0.098235	0
Novel_004429	XLOC_04683.	transcript	0	0.145162	0.046278	0.177133	0	0.925664	0.220692	0.367811	0.212025
Novel_004430	XLOC_04683.	transcript	0.187292	0.60566	0.308572	0	0.073432	0.036091	0.047161	2.047853	0
Novel_004431	XLOC_04683.	transcript	0.055759	0.073523	0	0.231283	0.009367	0	0.015067	0.080305	0
Novel_004432	XLOC_04684.	transcript	0.037425	4.410701	0.092321	0.031087	0.100287	0.051262	0.204014	0.073568	0.088607
Novel_004433	XLOC_04684.	transcript	0.370261	0.1897	0.50669	0.662947	0.711965	0.494574	0.748895	0.706021	0.704318
Novel_004434	XLOC_04685.	transcript	3.318727	5.734662	5.356961	3.334345	4.071318	4.545893	3.58528	4.10721	7.148081

Novel_004435	XLOC_04685	transcript	0.13617	0.079861	0.129739	0.106939	0.294316	0.094512	0.134741	0.098698	0.287665
Novel_004436	XLOC_04687	transcript	0.120864	0.057637	0.117848	0.030207	0.121086	0.129746	0.164319	0.129099	0.098911
Novel_004437	XLOC_04687	transcript	0.642495	0.324212	0.72037	1.129719	1.121338	0.894315	0.868836	0.873526	0.668013
Novel_004438	XLOC_04687	transcript	0	0.50074	0	0	0	0	0	0.35658	0
Novel_004439	XLOC_04695	transcript	0.122232	0.015814	0.24846	0.337407	0.123561	0.319087	0.027452	0.207185	0
Novel_004440	XLOC_04695	transcript	0	0	0	0.009305	0.123747	0	0	0.201699	0
Novel_004441	XLOC_04695	transcript	0.015861	0	0	0.264337	0.078345	0	0.093877	0.081767	0.010871
Novel_004442	XLOC_04697	transcript	0.733254	0.232611	0.98509	1.377062	1.071472	1.07719	0.866175	0.904884	1.038197
Novel_004443	XLOC_04698	transcript	0.039985	0	0.059282	0.067426	0.056547	0.0534	0.013571	0	0.021292
Novel_004444	XLOC_04698	transcript	0.133459	0.046446	0.147151	0.122182	0.157381	0.184481	0.321199	0.166129	0.144673
Novel_004445	XLOC_04698	transcript	0	0	0	0	0	0	0	0.023635	0
Novel_004446	XLOC_04698	transcript	0	0	0	0	0.03321	0	0	0	0
Novel_004447	XLOC_04698	transcript	0.016085	0.076668	0.044162	0.028654	0	0	0	0	0
Novel_004448	XLOC_04698	transcript	0	0.063644	0	0.000573	0	0	0.19367	0.084422	0
Novel_004449	XLOC_04698	transcript	0	0	0.489176	0	0.033993	0	0	0.612804	0
Novel_004450	XLOC_04698	transcript	0.451914	0.155805	0.154895	0.345062	0.318637	0.432669	0.282747	0.224967	0.084354
Novel_004451	XLOC_04698	transcript	0	0.572991	0.009869	0.198512	0.0092	0	0	0	0
Novel_004452	XLOC_04698	transcript	0	0	0	0	0	0.082483	0.86989	0	0
Novel_004453	XLOC_04698	transcript	0.941689	0	0.569412	0	1.393592	0.853551	0	0.231152	0
Novel_004454	XLOC_04698	transcript	0	0.807311	0	0	0	0	0.616747	0.317757	0
Novel_004455	XLOC_04698	transcript	0	0.004315	0	0	0	0	0	0	1.375704
Novel_004456	XLOC_04699	transcript	0	0	0	0	0	0	0	0	0
Novel_004457	XLOC_04699	transcript	0.523053	0.868453	0.460906	0.51953	0.568472	0.583653	0.699266	0.528833	0.491413
Novel_004458	XLOC_04699	transcript	0	0	0	0	0	0	0	0	0
Novel_004459	XLOC_04702	transcript	0.675364	3.982283	1.508119	1.21158	1.445054	1.487468	1.516329	1.530411	1.236182
Novel_004460	XLOC_04706	transcript	0.34234	0.412774	1.237594	1.028511	0.881204	0.785145	1.53795	0.980775	0.762147
Novel_004461	XLOC_04706	transcript	0.275929	0.145373	0.385739	0.620228	0.208039	0.483903	0	0.448631	0
Novel_004462	XLOC_04706	transcript	0	0	0	0	0	0	0	0	0
Novel_004463	XLOC_04706	transcript	0.027826	1.210193	0.058837	0.0227	0	0.13783	0.110715	0.105473	0.263271
Novel_004464	XLOC_04707	transcript	0.06922	0.013402	0.102361	0.014451	0.07864	0.229906	0.232899	0.136247	0.006531
Novel_004465	XLOC_04707	transcript	0	0	0	0	0	0	0	0	0
Novel_004466	XLOC_04708	transcript	0	0.002272	0	0	0	0.028298	0	0	0.000591
Novel_004467	XLOC_04708	transcript	0.531108	0.009247	0.005945	0	0.71725	0	1.035501	0	0.436595
Novel_004468	XLOC_04708	transcript	0.008325	0	0	0	0.0345	0	0.014517	0	0
Novel_004469	XLOC_04708	transcript	0.03485	0	0.751073	0.537603	0.126231	0	0.143337	0.278019	0.031812
Novel_004470	XLOC_04708	transcript	0.000379	0.135006	0	0.001327	0.009732	0.81045	0.00138	0.004446	0
Novel_004471	XLOC_04708	transcript	0	0	0	0	0	0.012761	0.004714	0.556084	0
Novel_004472	XLOC_04708	transcript	0.013726	0	0.712221	0.464005	0.000517	0.051565	0.000137	0.077853	1.00881

Novel_004473	XLOC_04711.	transcript	0.395303	0.639475	0.426875	0.432486	0.43464	0.369336	0.339421	0.582918	0.577791
Novel_004474	XLOC_04711.	transcript	0.013143	0	0.146831	0.142442	0.204966	0.302674	0.255309	0.119773	0
Novel_004475	XLOC_04715.	transcript	0	0	0	0	0	0	0	0	0
Novel_004476	XLOC_04715.	transcript	0	0	0.373419	0.252957	0.079531	0	0.748485	0.553369	0
Novel_004477	XLOC_04715.	transcript	0	0.540332	0	0.073605	0.031025	0.064364	0.203123	0	0
Novel_004478	XLOC_04715.	transcript	0.579271	0.034807	0	0	0.088454	0	0.019922	0.009404	0.247958
Novel_004479	XLOC_04715.	transcript	0.131852	0.236443	0.18969	0.290889	0.327365	0.123797	0.47536	0.315711	0.220239
Novel_004480	XLOC_04715.	transcript	0	0	0	0	0	0	0	0	0
Novel_004481	XLOC_04715.	transcript	0.075482	0.09395	0.124991	0.091037	0.410798	0.058783	0.632576	0.109762	0.076685
Novel_004482	XLOC_04715.	transcript	1.323305	0.306013	1.341483	1.188382	0.023385	1.193271	0.068957	1.615326	1.271594
Novel_004483	XLOC_04715.	transcript	0	0	0	0	0.727633	0	0	0	0
Novel_004484	XLOC_04715.	transcript	0	0.179761	0	0	0	0	0.895569	0.15337	0
Novel_004485	XLOC_04717.	transcript	0.097832	0.032578	0	0	0.241165	0.073356	0.656348	0	0.14435
Novel_004486	XLOC_04717.	transcript	0	0.200369	0.418934	0.187985	0	0	0	0.242554	0
Novel_004487	XLOC_04717.	transcript	0.372661	0.240437	0	0.541656	0.216254	0.434997	0.237714	0.219754	0.355549
Novel_004488	XLOC_04717.	transcript	0.12749	0.348019	0.347073	0.28336	0.370034	0.226692	0.230707	0.223575	0.27166
Novel_004489	XLOC_04717.	transcript	0	0	0	0	0.17948	0	0.119519	0.131051	0.152318
Novel_004490	XLOC_04717.	transcript	0.335771	0.340207	0.614069	0.636965	0.30078	0.617952	0.806892	0.320835	0.286892
Novel_004491	XLOC_04718.	transcript	0.791748	0.719169	1.022299	0.431754	0.89552	0.796283	1.200297	0.756691	0.88966
Novel_004492	XLOC_04718.	transcript	0	0	0	0.532442	0	0	0	0	0
Novel_004493	XLOC_04719.	transcript	0.007666	0.40496	0	0	0.019451	0	0.006834	0.000669	0
Novel_004494	XLOC_04719.	transcript	0.358096	0.201608	0.106257	0.175278	0.226121	0.136355	0.463054	0.298954	0.16342
Novel_004495	XLOC_04721.	transcript	103.0518	113.7628	177.1408	166.9549	139.5546	182.4359	124.5298	162.9146	144.808
Novel_004496	XLOC_04723.	transcript	0.93945	1.335948	1.238243	1.32763	1.187489	1.472282	1.48055	1.026646	1.022618
Novel_004497	XLOC_04723.	transcript	0	0	0	0	0	0	0	0	0
Novel_004498	XLOC_04726.	transcript	0.090717	0.317708	0.375751	0.379174	0.765616	0.399967	0.610327	0.321223	0.446747
Novel_004499	XLOC_04726.	transcript	0.013502	0.257893	0.114739	0.0851	0.04344	0.25055	0.15405	0.037303	0.106913
Novel_004500	XLOC_04726.	transcript	0.179769	0.034521	0.132549	0.243665	0.192277	0.342372	0.27729	0.071783	0.098727
Novel_004501	XLOC_04729.	transcript	0.341167	0.382184	0.570106	0.519114	0.776645	0.493581	0.934761	0.713981	0.519302
Novel_004502	XLOC_04730.	transcript	0	0.019489	0	0	0	0	0	0	0
Novel_004503	XLOC_04730.	transcript	0.144144	0.186027	0.206743	0.383343	0.443269	0.350958	0.206945	0.096905	0.273405
Novel_004504	XLOC_04730.	transcript	0	0	0	0	0	0	0	0.087165	0
Novel_004505	XLOC_04730.	transcript	0.284285	0.072337	0.599829	0.692542	0.482173	0.720232	0.551208	0.544752	0.367257
Novel_004506	XLOC_04730.	transcript	1.499341	1.427423	1.576644	1.565594	1.54924	1.738722	1.786325	1.970152	1.325371
Novel_004507	XLOC_04731.	transcript	0.321828	1.226612	0.119888	0.358672	0.134099	0.224397	0.504962	0.254483	0.078087
Novel_004508	XLOC_04731.	transcript	0.038048	0.853728	0.100789	0.015263	0.117666	0.100205	0.076722	0.01564	0.010719
Novel_004509	XLOC_04732.	transcript	0.115653	0.546149	0.171178	0.077544	0.047315	0	0.103622	0.105903	0.027321
Novel_004510	XLOC_04732.	transcript	0	0.886739	0	0	0	0	0	0	0

Novel_004511	XLOC_04732.	transcript	0	1.36565	0	0	0	0	0	0	0.290895
Novel_004512	XLOC_04732.	transcript	0.335158	0.839185	0.782809	0.408226	0.619864	0.616781	0.574831	0.505709	0.354889
Novel_004513	XLOC_04733.	transcript	1.287116	0.959271	0.748615	1.071917	0.666077	0.981582	0.309796	0.868394	0.291088
Novel_004514	XLOC_04733.	transcript	0.040151	0.240467	0.072432	0.14916	0.236928	0.021286	1.068228	0.014855	0.795071
Novel_004515	XLOC_04733.	transcript	0	0	0	0.740422	0	0	0	0	0
Novel_004516	XLOC_04733.	transcript	0	0	0	0.003269	0	0	0.005094	0	0
Novel_004517	XLOC_04733.	transcript	0	0	0	0	0	0.036567	0.033931	0.012604	0
Novel_004518	XLOC_04733.	transcript	0.086503	0.062954	0.012508	0	0.140226	0.258557	0.509644	0.158974	0.213203
Novel_004519	XLOC_04733.	transcript	0	0	0.564468	0.234489	0.271707	0.427663	0	1.184485	0
Novel_004520	XLOC_04733.	transcript	0	0.008398	0.328251	0.099427	0.160423	0.132811	0	0.086266	0
Novel_004521	XLOC_04733.	transcript	0.579239	0.074579	0.020539	0	0	0	0.197934	0.037551	0.190592
Novel_004522	XLOC_04733.	transcript	0.058142	0.049795	0.0086	0	0.085252	0.013203	0.285339	0.078398	0.026534
Novel_004523	XLOC_04733.	transcript	0	0	0.115601	0	0.010002	0.186926	0	0.006523	0
Novel_004524	XLOC_04733.	transcript	0.112808	0	0.160399	0.026915	0.15996	0.165709	0.701843	0.051488	0.215742
Novel_004525	XLOC_04733.	transcript	0.043154	0	0.016248	0	0.085073	0.023369	0.098662	0.034127	0
Novel_004526	XLOC_04733.	transcript	0.00741	0.083495	0.050519	0.054462	0.09225	0.457031	0.443451	0.399986	0.07848
Novel_004527	XLOC_04733.	transcript	0.455142	0.945589	0.390094	0.440584	0.250709	0.025243	0.318137	0.000324	0.32818
Novel_004528	XLOC_04733.	transcript	0	0	0	0	0.241878	0	0	0.180153	0
Novel_004529	XLOC_04734.	transcript	0.139802	0.274283	0.122999	0.043974	0.132174	0.187752	0.230907	0.168458	0.127867
Novel_004530	XLOC_04735.	transcript	3.306925	0.747995	0.883348	2.936921	2.319648	2.598663	1.569484	1.267273	0.548734
Novel_004531	XLOC_04735.	transcript	0.06166	0.029082	0.49438	0	0	0.349805	1.089148	0	0.876105
Novel_004532	XLOC_04735.	transcript	0.001389	0.616874	0.049627	0.306456	0.178011	0.005585	0.317987	1.222985	0.075058
Novel_004533	XLOC_04735.	transcript	0.170132	0	0.75595	0.22961	0.228965	0.395653	0.740433	0.251979	0.14632
Novel_004534	XLOC_04735.	transcript	0	0.372429	0	0	0	0	0	0	1.228066
Novel_004535	XLOC_04735.	transcript	0.076134	0.157551	0.049412	3.037269	1.324323	0.127229	0.569851	0.871822	0.060302
Novel_004536	XLOC_04735.	transcript	0	0	0.01008	0	0.618684	0.028425	0.460653	0.250377	0
Novel_004537	XLOC_04735.	transcript	0	0	0	0	0	0	0.007659	0.074963	0.019169
Novel_004538	XLOC_04735.	transcript	6.403577	0	8.266838	0.004632	7.208922	10.03746	3.308168	4.48661	0.011344
Novel_004539	XLOC_04735.	transcript	0	0	0	0.182079	0.030952	0.199549	0.445176	0.382963	0.009024
Novel_004540	XLOC_04735.	transcript	0.462073	0	0.16548	0.998492	0.064464	0.067243	0.43505	0.359355	0
Novel_004541	XLOC_04735.	transcript	0.011898	0	0.943602	4.114596	1.113052	0.407352	0.406887	1.037962	0.265261
Novel_004542	XLOC_04735.	transcript	0.006686	0.067506	0.058964	0.981959	0.305819	0.013024	0.579117	0.003932	0
Novel_004543	XLOC_04735.	transcript	0	0	0	1.810461	0.006079	0.018175	0	0.088446	0.321613
Novel_004544	XLOC_04735.	transcript	0	0	0.059411	0.590742	0.052106	0.046691	0.018235	0.054704	0.028538
Novel_004545	XLOC_04735.	transcript	0	0	0	0	0	0	0	0	0
Novel_004546	XLOC_04735.	transcript	0	0	0.833393	1.654365	0	0.809079	0	0.590257	0
Novel_004547	XLOC_04735.	transcript	0	0	0.383104	0.885199	0.524541	0.397132	0	0.553077	0
Novel_004548	XLOC_04735.	transcript	0.105934	0	0	0	0	0	0.020326	0	0

Novel_004549	XLOC_04736.	transcript	0.102049	0.0209	0.342406	0.586888	0.35578	0.810325	0.253528	0.562418	0.282555
Novel_004550	XLOC_04737.	transcript	2.075913	2.238271	1.987553	3.437108	2.13511	2.418732	1.727389	2.987627	1.891943
Novel_004551	XLOC_04737.	transcript	0.30737	0	0.525125	0.198772	0.273719	0.298442	0.19308	0.467647	0
Novel_004552	XLOC_04738.	transcript	0	0.138326	0.021681	0	0	0.234348	0	0.318586	0.018509
Novel_004553	XLOC_04738.	transcript	1.252359	1.844175	0.949448	1.044813	1.299754	0.855694	1.478858	1.087863	1.076018
Novel_004554	XLOC_04738.	transcript	0.15586	0	0	0.146171	0	0	0	0.094857	0.072022
Novel_004555	XLOC_04738.	transcript	0	0	0.310523	0	0.03834	0.122647	0	6.80E-05	0.078888
Novel_004556	XLOC_04738.	transcript	0.254953	0	4.40E-05	0	0.002948	0.020992	0	0	0
Novel_004557	XLOC_04738.	transcript	0.687567	0.653844	0.722311	1.041194	0.578694	0.727968	0.82476	0.784573	0.546924
Novel_004558	XLOC_04739.	transcript	0	0	0	0.066555	0	0	0	0	0
Novel_004559	XLOC_04741.	transcript	0.079651	0.098095	0.154743	0.103067	0.040746	0.092378	0.064845	0.119116	0.147801
Novel_004560	XLOC_04742.	transcript	0.390533	0.146753	0.576052	0.566668	0.74972	0.346918	0.634575	0.704289	0.585908
Novel_004561	XLOC_04743.	transcript	0.224135	0	0.212766	0.171048	0.174059	0.143034	0.523134	0.34196	0.076467
Novel_004562	XLOC_04743.	transcript	0	0	0	0	0.134295	0	0.02094	0	0.01806
Novel_004563	XLOC_04750.	transcript	3.377775	0.633724	3.966443	2.930514	3.589805	2.971293	4.440788	3.273693	2.008654
Novel_004564	XLOC_04753.	transcript	0.135366	0.05372	0	0	0	0	0.249791	0.126987	0.068639
Novel_004565	XLOC_04754.	transcript	0.099807	0.054791	0.090823	0.118344	0.149571	0.098254	0.274714	0.063929	0.032963
Novel_004566	XLOC_04754.	transcript	0.331386	0.398603	1.06251	0.663742	1.270651	0.326439	0.847024	0.686601	0.600174
Novel_004567	XLOC_04756.	transcript	0.093637	0.011013	0	0.016817	0	0.047988	0.099977	0.107036	0.113678
Novel_004568	XLOC_04756.	transcript	0.269977	0.159797	0.319554	0.243428	0.19837	0.081282	0.303011	0.299442	0
Novel_004569	XLOC_04764.	transcript	0.060932	0.28446	0.190594	0.456644	0.495155	0.066915	0.83893	0.371365	0.585562
Novel_004570	XLOC_04764.	transcript	0.723287	0.320423	0.179949	0.065008	0.036936	0.252608	0.208937	0.4215	0.005269
Novel_004571	XLOC_04764.	transcript	0	0.38257	0.511095	0.563053	0.405081	0.379573	0.482021	0.614041	0
Novel_004572	XLOC_04765.	transcript	0.151448	0.275315	0.201645	0.09357	0.032247	0.275484	0.21282	0.220479	0.1271
Novel_004573	XLOC_04765.	transcript	0	0	0	0.172893	0	0	0	0	0
Novel_004574	XLOC_04765.	transcript	0	0	0	0	0	0	0	0	0
Novel_004575	XLOC_04765.	transcript	0.084691	0.339883	0.104612	0.021921	0.267416	0.146796	0.123918	0.022229	0.123869
Novel_004576	XLOC_04769.	transcript	0.283641	0.048047	0.557387	0	0	0	0.637348	0.125722	0
Novel_004577	XLOC_04769.	transcript	0	0	0	0	0.062497	0	0	0	0
Novel_004578	XLOC_04769.	transcript	0.122683	0.035626	0.013246	0.177396	0	0.096491	0.046611	0.050907	0.048366
Novel_004579	XLOC_04769.	transcript	0.159431	0.584728	0.247542	0.329239	0.557411	0.422832	0.324519	0.443436	0.524177
Novel_004580	XLOC_04769.	transcript	0.127484	0.141683	0	0.066043	0.031217	0.247302	0	0.087224	0.170763
Novel_004581	XLOC_04769.	transcript	0.044511	0.092313	0.00066	0.027228	0.047616	0.000242	0.148906	0.056057	0.182878
Novel_004582	XLOC_04769.	transcript	0	0.081306	0.039049	0.035226	0.016136	0.182337	0.048262	0.041609	0.129937
Novel_004583	XLOC_04769.	transcript	0.500098	0	0.66282	0.416506	1.00E-04	0	0.834394	0.077505	0
Novel_004584	XLOC_04769.	transcript	0	0	0	0	0.590935	0	0	0.172671	0
Novel_004585	XLOC_04769.	transcript	0.532801	0.299055	1.665168	1.640008	1.101547	1.581318	3.387034	2.285948	0.927643
Novel_004586	XLOC_04769.	transcript	0	0	0	0	0	0	0	0	0

Novel_004587	XLOC_04769.	transcript	1.032418	0.926842	0	0.431302	0.283602	0	0.14461	0	0.592831
Novel_004588	XLOC_04770.	transcript	0.701191	2.005696	0.840528	1.108176	0.813865	1.106765	0.997982	1.019552	1.4364
Novel_004589	XLOC_04771.	transcript	0	0.207893	0.372275	0.262983	0	0.00987	0.064829	0.130083	0.27254
Novel_004590	XLOC_04771.	transcript	0	0.02241	0	0	0.250335	0	0	0	0
Novel_004591	XLOC_04771.	transcript	0.512714	0	0.208879	0.212924	0.254727	0.466506	0.335336	0.094395	0.229469
Novel_004592	XLOC_04771.	transcript	0	0	0	0	1.530084	1.197214	2.291183	1.639679	0
Novel_004593	XLOC_04771.	transcript	1.179163	0.771277	1.537515	0.912753	0	0	0.017206	0	1.19388
Novel_004594	XLOC_04771.	transcript	0.644964	0.985248	1.063744	0.690411	0.620694	1.28927	0.993063	1.111439	1.140776
Novel_004595	XLOC_04771.	transcript	0	0	0.022674	0.975698	0.629576	0	0.032826	0	0
Novel_004596	XLOC_04771.	transcript	0.033065	0.259892	0.014236	0.106106	0.003219	0.148778	0	0.126099	0.08708
Novel_004597	XLOC_04776.	transcript	0	0.058978	0	0.27071	0	0.390085	0	0.010223	0
Novel_004598	XLOC_04776.	transcript	0.563104	0.396364	0.598184	0.214736	0.542935	0.020201	0.925606	0.562556	0.536873
Novel_004599	XLOC_04776.	transcript	0	0	0	0	0	0	0.27615	0	0
Novel_004600	XLOC_04776.	transcript	0.094485	0.294155	0.190255	0.260581	0.160444	0.216081	0.133696	0.261013	0.342957
Novel_004601	XLOC_04776.	transcript	0.650361	0.890497	1.077237	1.018631	0.494691	0.81111	0.870815	0.822782	0.696244
Novel_004602	XLOC_04776.	transcript	0	0.000211	0	0.168699	0	0.219231	0	0.003531	0
Novel_004603	XLOC_04776.	transcript	0.237961	0.126627	0.121934	0.025849	0.183968	0	0.363161	0.244162	0.136132
Novel_004604	XLOC_04776.	transcript	0	0.022265	0.017561	0.08855	0	0.051515	0	0	0
Novel_004605	XLOC_04800.	transcript	0	0	0	0	0	0	0	0	0
Novel_004606	XLOC_04800.	transcript	0.14384	0.011555	0.04422	0.070072	0.113572	0.016822	0.208861	0.184017	0.185488
Novel_004607	XLOC_04806.	transcript	0.023769	0.381055	0.44127	0.30298	0.349532	0.413786	0.58548	0.54563	0.303903
Novel_004608	XLOC_04806.	transcript	0.50109	0.113083	0.337351	0.219264	0.131438	0.260179	0.220601	0.058223	0.335033
Novel_004609	XLOC_04807.	transcript	0	0	0	0	0	0	0	0	0
Novel_004610	XLOC_04807.	transcript	0	0	0	0.024839	0	0	0.106872	0.062414	0.124605
Novel_004611	XLOC_04807.	transcript	0.173877	0.174506	0.046399	0.035682	0.053139	0.07353	0.099381	0.054081	0.077363
Novel_004612	XLOC_04807.	transcript	0	0.058768	0	0.035011	0	0	0.14566	0.079673	0.147203
Novel_004613	XLOC_04813.	transcript	0.645193	0.242588	0.704006	1.193608	0.550055	0.77426	0.627165	1.036179	0.594441
Novel_004614	XLOC_04813.	transcript	0.003556	0	0.004097	0	0.003249	0.003849	0.407795	0	0
Novel_004615	XLOC_04819.	transcript	0.156007	0	0.130896	0.16421	0	0.154354	0.064589	0.28668	0
Novel_004616	XLOC_04853.	transcript	0.027784	0	0	0	0.004815	0	0.021553	0.000499	0
Novel_004617	XLOC_04853.	transcript	0.282874	0.217923	0.473573	0.348064	0.361177	0.463551	0.327408	0.552501	0.367545
Novel_004618	XLOC_04870.	transcript	0.233883	0	0	0	0	1.295316	0	0	0
Novel_004619	XLOC_04888.	transcript	0.044866	0.075493	0.043195	0.020284	0	0.07971	0	0	0.042492
Novel_004620	XLOC_04888.	transcript	0	0.02394	0.051652	0	0	0.374385	0.028539	0	0
Novel_004621	XLOC_04888.	transcript	0.315302	0	0	0	0	0	0	0	0
Novel_004622	XLOC_04888.	transcript	0	0.107485	0	0	0	0	0.048824	0	0
Novel_004623	XLOC_04904.	transcript	0.685431	0.364198	0.141715	1.02421	1.213047	1.205776	0.02533	0.77105	0.633511
Novel_004624	XLOC_04904.	transcript	2.080328	0	1.012465	0.265761	0	0	2.060442	0	0

Novel_004625	XLOC_04911.	transcript	0.138263	0.908132	0.496642	0.380637	0.467273	0.229136	0.337698	0.418563	0.470062
Novel_004626	XLOC_04911.	transcript	0.518304	0.310136	0.303786	0.156112	0.307408	0.514456	0.406003	0.489234	0.270103
Novel_004627	XLOC_04911.	transcript	0	0	0	0	0	0	0.012254	0.594456	0
Novel_004628	XLOC_04911.	transcript	0.422634	0.026432	0.091984	0.160582	0.179613	0.077153	0.375913	0.132948	0.266016
Novel_004629	XLOC_04913.	transcript	0.14321	0	0.094257	0.039333	0.115012	0.143265	0.440598	0.077313	0.106687
Novel_004630	XLOC_04913.	transcript	0.02817	0.122039	0.261951	0.503764	0.235473	0.408034	0.433005	0.101331	0.314339
Novel_004631	XLOC_04924.	transcript	0.133869	0.198502	0.072544	0.014728	0.033378	0.112657	0.205674	0.054288	0.043546
Novel_004632	XLOC_04929.	transcript	0.110928	0.166615	0.08786	0.032133	0.134058	0.104717	0.232062	0.086282	0.055256
Novel_004633	XLOC_04930.	transcript	0	0.082578	0.249633	0	0.32736	0	0.000292	0	0
Novel_004634	XLOC_04930.	transcript	0.651169	0.101853	0	0.186792	0	0.270096	0.636625	0.198581	0
Novel_004635	XLOC_04930.	transcript	0.194154	0.092248	0	0.337898	0	0.296357	0.265594	0.224826	0
Novel_004636	XLOC_04932.	transcript	0.349313	0.716359	0.37867	0	0.2921	0.399427	0.389767	0.460108	0.598188
Novel_004637	XLOC_04932.	transcript	0	0	0	0.404806	0.193482	0	0	0	0
Novel_004638	XLOC_04932.	transcript	0	0.247187	0	0.005196	0.108243	0	0.194715	0	0.001881
Novel_004639	XLOC_04932.	transcript	0	0	0	0	0	0	0	0	0
Novel_004640	XLOC_04933.	transcript	0	0.453448	0.207452	0	0.206879	0.204197	0.085595	0.001621	0
Novel_004641	XLOC_04933.	transcript	0.227607	0.284156	0.67383	0.08964	0	0.208491	0.533448	0.51052	0.16366
Novel_004642	XLOC_04933.	transcript	0.369861	0.001925	0.003854	0.573677	0.477392	0.570005	0.539438	0.404375	0.222601
Novel_004643	XLOC_04933.	transcript	0.262478	0	0	0.176921	0.273969	0	0	0	0.216496
Novel_004644	XLOC_04934.	transcript	0.574466	0	0	0	0.4871	0.25583	0	0.371184	0.145145
Novel_004645	XLOC_04934.	transcript	0	0	0.317695	0.401431	0	0	0.519265	0.216282	0.199733
Novel_004646	XLOC_04936.	transcript	0.106982	0	0.135835	0.128154	0.234657	0.098438	0.155089	0.13572	0.137766
Novel_004647	XLOC_04937.	transcript	0.204344	0	0.092587	0.073317	0.148639	0.035162	0.236129	0.069468	0.124133
Novel_004648	XLOC_04940.	transcript	1.97892	0.728792	1.44422	1.729093	1.667204	1.146076	3.149901	2.096925	1.96622
Novel_004649	XLOC_04940.	transcript	0.913015	0.865618	0.856084	2.084226	1.330341	1.657928	1.954474	1.125498	1.893596
Novel_004650	XLOC_04942.	transcript	0.421089	1.036387	0.759882	0.679389	0.840964	0.683267	0.637515	0.812028	1.003016
Novel_004651	XLOC_04943.	transcript	0	0.040119	0	0	0	0	0.004409	0.360104	0.213321
Novel_004652	XLOC_04943.	transcript	0	0.011883	0.104272	0	0.08223	0.087472	0.40246	0	0
Novel_004653	XLOC_04943.	transcript	0.257625	0.01112	0	0.153684	0	0	0.003535	0	0.009371
Novel_004654	XLOC_04943.	transcript	0.045235	0.344922	0.088096	0.071654	0.156614	0.004635	0.072749	0.037077	0.045753
Novel_004655	XLOC_04943.	transcript	0.011263	0.459893	0.035854	0.077755	0.10878	0.019709	0.025672	0.014013	0.010111
Novel_004656	XLOC_04943.	transcript	0.513487	2.116868	0	0.307188	0.207258	0.19515	0.653396	0.144355	0.907943
Novel_004657	XLOC_04943.	transcript	0.083674	0.086741	0.20699	0.018095	0.142012	0.146493	0.198154	0.082935	0.236073
Novel_004658	XLOC_04943.	transcript	0.041705	0	0.121934	0.208829	0.458886	0.130132	0.326155	0.015557	0.061151
Novel_004659	XLOC_04944.	transcript	0.422589	0.114849	0.368098	0.202033	0.445328	0.303386	0.371803	0.365362	0.18186
Novel_004660	XLOC_04944.	transcript	0.859249	0.672648	0.419052	0.538917	0.92414	0	0.263798	0.147691	0.91824
Novel_004661	XLOC_04945.	transcript	1.074321	0.013401	1.953025	0.375688	2.483335	0.717354	2.112522	1.332184	0.961752
Novel_004662	XLOC_04946.	transcript	0	0	0.032748	0	0.111816	0	0.050303	0.063568	0

Novel_004663	XLOC_04946.	transcript	0.040914	0.009386	0.117382	0.028476	0.086832	0	0.068206	0	0.013422
Novel_004664	XLOC_04949.	transcript	0	0	0	0	0	0	0.260573	0	0
Novel_004665	XLOC_04953.	transcript	0.378925	0.145086	1.243656	0.722176	0.93814	0.971114	1.20172	0.716074	0.763535
Novel_004666	XLOC_04954.	transcript	0.033838	1.282134	0.028638	0.017065	0.035538	0.083933	0.051788	0.011671	0.189533
Novel_004667	XLOC_04956.	transcript	0	0.082644	0.36808	0.140764	0.140108	0.351609	0.354137	0.325827	0.384091
Novel_004668	XLOC_04960.	transcript	0.248506	0.066054	0.094883	0	0.189052	0.11374	0.246908	0.085203	0.218323
Novel_004669	XLOC_04960.	transcript	0	0	0	0.340426	0	0	0	0	0
Novel_004670	XLOC_04960.	transcript	0.003878	0	0.031886	0.108695	0.110123	0.216318	0.021781	0.252365	0.034569
Novel_004671	XLOC_04960.	transcript	0	0	0	0	0	0	0	0	0
Novel_004672	XLOC_04962.	transcript	0	0	0	0	0	0	0	0.201995	0
Novel_004673	XLOC_04962.	transcript	0.28508	0.638472	0.490022	0.401986	0.388382	0.543192	0.291763	0.320092	0.221909
Novel_004674	XLOC_04962.	transcript	0.040422	0	0.042735	0.033878	0.041343	0.020159	0.015116	0.150434	0
Novel_004675	XLOC_04962.	transcript	0.106862	0	0.004834	0.001126	0	0	0.133899	0	0.040255
Novel_004676	XLOC_04962.	transcript	0.072234	0	0	0.04343	0	0.084475	0.187313	0	0
Novel_004677	XLOC_04962.	transcript	0.024196	0	0	0	0.023088	0	0.110127	0	0
Novel_004678	XLOC_04962.	transcript	0	0.000816	0	0.020187	0.219991	0.032667	0	0.00252	0.074865
Novel_004679	XLOC_04962.	transcript	0	0.05382	0	0	0.101619	0	0.074591	0.087045	0
Novel_004680	XLOC_04962.	transcript	0	0	0.036554	0.022601	0	0.018977	0	0	0
Novel_004681	XLOC_04962.	transcript	0	0.04536	0	0.112982	0	0	0.070277	0.022409	0
Novel_004682	XLOC_04962.	transcript	0.055577	0	0	0.033144	0	0.086256	0.04773	0	0
Novel_004683	XLOC_04962.	transcript	0.056738	0.055758	0.082085	0.003901	6.00E-06	0.01235	0.045721	0.069447	0.106952
Novel_004684	XLOC_04962.	transcript	0.026029	0.10191	0.016893	0.038121	0	0.000191	0	0	0
Novel_004685	XLOC_04962.	transcript	0.014493	0.148198	0.02785	0.025226	0.017293	0.030364	0.032766	0.096076	0.045195
Novel_004686	XLOC_04963.	transcript	0.056933	0	0.011389	0.043251	0.025106	0.612797	0.146952	0.029493	0
Novel_004687	XLOC_04963.	transcript	0.27415	0.129077	0.265893	0.4182	0.495777	0.103973	0.048272	0.087899	0.272195
Novel_004688	XLOC_04963.	transcript	0	0.002594	0.078975	0	0	0.034328	0.579806	0.309527	0.134244
Novel_004689	XLOC_04963.	transcript	0.550091	0.823954	0.480637	0.432113	0.690582	0.703327	1.059177	0.752857	1.158127
Novel_004690	XLOC_04963.	transcript	0	0.697942	0.034206	0	0	0	0	0.036759	0
Novel_004691	XLOC_04963.	transcript	1.133558	1.584788	0.916931	1.68486	1.307485	1.077631	1.669912	1.394711	1.083579
Novel_004692	XLOC_04965.	transcript	7.884688	24.41198	16.76592	15.75352	18.66152	20.07804	13.34674	17.99951	11.62466
Novel_004693	XLOC_04966.	transcript	3.162638	0.139976	0.531947	0.898319	1.617273	0.469177	2.771426	0.219646	0.374554
Novel_004694	XLOC_04966.	transcript	0.322569	0.098351	0.203683	0.187213	0.335152	0.145541	0.299056	0.282516	0.38373
Novel_004695	XLOC_04966.	transcript	0	0.554797	0.011992	0	0.026612	0.194788	0	0.021219	0.070242
Novel_004696	XLOC_04966.	transcript	0.135252	0.196047	0.260138	0.101712	0.050522	0.238068	0.20493	0.092607	0.135224
Novel_004697	XLOC_04966.	transcript	0.253027	0.351498	0.261896	0.017214	0.122858	0.655938	0.409253	0.362883	0.292425
Novel_004698	XLOC_04966.	transcript	0	0	0	0.368761	0.237614	0	0.301011	0	0
Novel_004699	XLOC_04966.	transcript	0.427771	0.923609	0.532449	0.680123	0.770993	0.629609	0.865375	0.8237	0.489416
Novel_004700	XLOC_04966.	transcript	0	0	0	0	0	0	0	0	0

Novel_004701	XLOC_04967.	transcript	0	0.012883	0	0	0	2.148117	0.112121	0	0.052501
Novel_004702	XLOC_04967.	transcript	0.51498	1.210107	0.927456	1.196077	0.958811	0.700344	0.89438	0.994864	1.39418
Novel_004703	XLOC_04969.	transcript	0.121071	0.110459	0.396851	0.4191	0	0	0.478829	0.149199	0.535549
Novel_004704	XLOC_04969.	transcript	0.152398	0.229278	0.111824	0.278453	0.319065	0.482182	0.134208	0.139502	0.159644
Novel_004705	XLOC_04969.	transcript	0.339715	0.109865	0.161883	0	0	0	0.153288	0.182147	0
Novel_004706	XLOC_04969.	transcript	0.148497	0.199517	0.163222	0	0.161582	0.226955	0.135854	0.126829	0
Novel_004707	XLOC_04969.	transcript	0.166667	0.172419	0.190199	0	0.093606	0.091111	0.16485	0.285082	0
Novel_004708	XLOC_04969.	transcript	0.140481	0.09522	0.116087	0.249499	0	0	0.145244	0.165766	0.204292
Novel_004709	XLOC_04969.	transcript	0.179529	0.215865	0.134115	0.284588	0.092915	0.086192	0.16035	0.099345	0.209102
Novel_004710	XLOC_04969.	transcript	0.177947	0.121628	0.169661	0.282646	0.897025	0.415632	0.147423	0.203273	0.173977
Novel_004711	XLOC_04969.	transcript	0.189389	0.121787	0.14254	0.263009	0.092178	0.145426	0.125769	0.162119	0.128294
Novel_004712	XLOC_04970.	transcript	0	0	0	0	0	0	0	0	0.02876
Novel_004713	XLOC_04970.	transcript	0.192967	0.043357	0.082147	0.174078	0.185883	0.031057	0.05294	0.173994	0.05945
Novel_004714	XLOC_04970.	transcript	0.006481	0.021137	0	0	0.031738	0.096559	0.060615	0	0
Novel_004715	XLOC_04971.	transcript	0.073269	0.007787	0.206119	0	0.021231	0	2.00E-06	0	0
Novel_004716	XLOC_04971.	transcript	0.347257	0.078644	0.18934	0.20068	0.381648	0.143701	0.223691	0.131778	0.097829
Novel_004717	XLOC_04971.	transcript	0	0	0.053692	0	0	0	0	0.185013	0
Novel_004718	XLOC_04971.	transcript	0.130703	0.013722	0.098932	0	0.116421	0.018411	0.236165	0.052119	0
Novel_004719	XLOC_04973.	transcript	0.058941	0.080263	0.027468	0.053302	0.148277	0.131777	0.222408	0.084071	0.195507
Novel_004720	XLOC_04973.	transcript	0	0.064851	0	0	0	0	0	0	0
Novel_004721	XLOC_04973.	transcript	0	0	0	0	0	0	0	0.023549	0
Novel_004722	XLOC_04974.	transcript	0.485765	0.895713	0.536772	0.127568	0.497391	0.324993	0.678019	0.446683	0.635584
Novel_004723	XLOC_04974.	transcript	0	0.239564	0	0.082564	0	0.260874	0	0.152452	0
Novel_004724	XLOC_04974.	transcript	0.317103	0.219793	0.214786	0.126845	0.221842	0	0.381202	0	0.233501
Novel_004725	XLOC_04974.	transcript	0.107182	0.038099	0.02232	0	0	0.210572	0.084048	0.003345	0
Novel_004726	XLOC_04974.	transcript	0.077032	0.016878	0.018302	0.025112	0.302918	0.120313	0.115022	0.003233	0.033731
Novel_004727	XLOC_04974.	transcript	0.129516	0.002544	0.012704	0.128592	0	0.040971	0.063433	0.004728	0.123101
Novel_004728	XLOC_04974.	transcript	0	0	0	0.221769	0.027386	0.112332	0.100771	0.012726	0
Novel_004729	XLOC_04974.	transcript	0	0.211124	0.278052	0.636292	0.485054	0.145897	0.338925	0.66558	0.127138
Novel_004730	XLOC_04977.	transcript	0.003747	0.693762	0.007896	0	0.013012	0	0.002793	0.002138	0
Novel_004731	XLOC_04979.	transcript	0.652329	0.462572	0.567432	0.223276	0.716592	0.519823	0.652775	0	0.609047
Novel_004732	XLOC_04979.	transcript	0	0	0	0	0	0	0	0.526496	0
Novel_004733	XLOC_04981.	transcript	0	0.220929	0	0	0	0.093129	0.031942	0.34986	0.014586
Novel_004734	XLOC_04981.	transcript	0.063658	1.919308	0.204823	0.42603	0.102572	0.356042	0	0.053256	1.64855
Novel_004735	XLOC_04981.	transcript	0.077241	0	0	0	0.229404	0	1.957046	0	0
Novel_004736	XLOC_04981.	transcript	1.001893	0	1.796925	1.969521	1.413015	1.527004	0	1.664961	0
Novel_004737	XLOC_04982.	transcript	0.188668	0	0.057955	0.174262	0.139402	0.411919	0.073584	0.054065	0.065258
Novel_004738	XLOC_04982.	transcript	0	0.042352	0.245294	0	0	0	0	0.086952	0

Novel_004739	XLOC_04982.	transcript	0.105963	0.410112	0.12846	0.086571	0.083112	0.167956	0.229088	0.237435	0.194512
Novel_004740	XLOC_04983.	transcript	0.139446	0.0683	0	0	0.191225	0.31516	0.148616	0	0
Novel_004741	XLOC_04983.	transcript	0	0	0	0	0	0	0	0	0
Novel_004742	XLOC_04983.	transcript	0.006387	0.949816	0.36646	0.421263	0.2555	0.118695	0.764849	0.28221	0.006547
Novel_004743	XLOC_04983.	transcript	0	0	0	0	0.002229	0	0	0	0
Novel_004744	XLOC_04983.	transcript	0.772936	0.085893	0.149674	0.073345	0.255131	0.510636	0.115608	0.361429	0.493711
Novel_004745	XLOC_04983.	transcript	0.15554	0.21791	0.056976	0.109019	0.090236	0.063918	0.147203	0.390796	0.307926
Novel_004746	XLOC_04985.	transcript	0	0.224478	0.045447	0.054773	0.044561	0.023346	0.03253	0	0.025817
Novel_004747	XLOC_04986.	transcript	0.503527	0.121464	0.483788	0.174604	0.259432	0.308544	0.615616	0.220617	0.229941
Novel_004748	XLOC_04987.	transcript	0.064132	0.229037	0.140197	0.067472	0.188092	0.040027	0.095814	0.084082	0.134869
Novel_004749	XLOC_04989.	transcript	0.122945	0	0.501958	0.19524	0.054643	0.101451	0.069594	0.208402	0.752001
Novel_004750	XLOC_04993.	transcript	0.328212	0.128593	0.11019	0.150484	0.265139	0.248732	0.313937	0.309581	0.39851
Novel_004751	XLOC_04993.	transcript	0.015554	0.020435	0.009019	0.00344	0.00961	0.006983	0.007521	0	0.056412
Novel_004752	XLOC_04993.	transcript	0	0	0.217912	0.00537	0.120166	0.121326	0.437252	0.220538	0.113733
Novel_004753	XLOC_04993.	transcript	0	0	0	0.107768	0	0.004055	0	0	0.002075
Novel_004754	XLOC_04993.	transcript	0.969279	0.502916	1.035668	0.743952	1.110477	0.639505	1.098816	0.784251	0.435633
Novel_004755	XLOC_04994.	transcript	0	0	0.036753	0	0	0	0	0	0
Novel_004756	XLOC_04994.	transcript	0.091834	0.45486	0.245329	0.32686	0.533695	0.557592	0.789882	0.650854	0.821902
Novel_004757	XLOC_04994.	transcript	0.853127	0.076187	0.901633	0.572977	0.627063	0.508695	1.003254	0.740486	1.065701
Novel_004758	XLOC_04996.	transcript	0.205062	0.330648	0.288415	0.370665	0.360083	0.358994	0.46297	0.309954	0.668036
Novel_004759	XLOC_04997.	transcript	0	0	0	0	0	0	0	0	0
Novel_004760	XLOC_04997.	transcript	0	0	0	0	0	0	0	0	0
Novel_004761	XLOC_04997.	transcript	0.232145	0	0.186695	0.151058	0.167561	0.090272	0.053129	0.148587	0.03561
Novel_004762	XLOC_04997.	transcript	0.217006	0.035271	0.104844	0.06976	0.158849	0.158722	0.23131	0.158114	0.12564
Novel_004763	XLOC_04998.	transcript	0.093153	0.094578	0	0	0	0.20493	0	0	0
Novel_004764	XLOC_04998.	transcript	0	0	0	0	0	0.001659	0	0.032976	0
Novel_004765	XLOC_04998.	transcript	0.37777	0.430227	0.295689	0.197905	0.284949	0.309449	0.47443	0.366187	0.224673
Novel_004766	XLOC_05010.	transcript	0.082225	1.156976	0.081542	0.116078	0.066497	0.081128	0.148185	0.055019	0.040705
Novel_004767	XLOC_05034.	transcript	0	0.355967	0	0.794007	0	0	0	0	0
Novel_004768	XLOC_05034.	transcript	0	0.613387	0	0	0	0	0.532178	0	0
Novel_004769	XLOC_05034.	transcript	0.809483	1.004207	0.972663	0.857781	0.979487	1.248261	0.972195	0.752284	1.056548
Novel_004770	XLOC_05040.	transcript	0.256056	0.091628	0.215346	0.119965	0.270704	0.054219	0.249192	0.136325	0.2095
Novel_004771	XLOC_05042.	transcript	1.72987	1.668454	1.167686	0.968365	0.910916	0.916673	0.846492	1.438517	2.342372
Novel_004772	XLOC_05042.	transcript	0.094238	0.250375	0.02954	0.026342	0.072195	0.048581	0.86444	0.022494	0.088958
Novel_004773	XLOC_05047.	transcript	0.071984	0.017046	0.062803	0.094776	0.052659	0.176948	0.038326	0.042508	0.054802
Novel_004774	XLOC_05048.	transcript	0.103958	0	0	0	0.135629	0.074755	0.34316	0.047617	0.122874
Novel_004775	XLOC_05048.	transcript	0	0.397853	0	0	0	0	0	0	0.398901
Novel_004776	XLOC_05049.	transcript	0	0	0	0	0.076193	0	0	0	0

Novel_004777	XLOC_05049.	transcript	0.157225	0.181789	0.116616	0	0	0.408286	0.137554	0.030136	0.128125
Novel_004778	XLOC_05049.	transcript	0	0	0	0	0.859458	0	0.200607	0.075125	0
Novel_004779	XLOC_05049.	transcript	0.021802	0	0	0.512436	0	0	0	0	0
Novel_004780	XLOC_05049.	transcript	0.298155	0.30618	0.16369	0.296912	0.001925	0.065386	0	0.25466	0.456054
Novel_004781	XLOC_05057.	transcript	1.212086	0.217426	0.710153	0.563007	0.842007	0.715541	1.352546	0.842917	0.60781
Novel_004782	XLOC_05061.	transcript	0.212503	0.0747	0.022849	0.080166	0.04421	0.033204	0.131706	0.102517	0.048102
Novel_004783	XLOC_05064.	transcript	0	0	0	0	0	0	0	0	1.391981
Novel_004784	XLOC_05064.	transcript	0	0	1.457614	0.681372	0.625935	0	0.715002	1.354193	1.149562
Novel_004785	XLOC_05064.	transcript	1.389695	1.23924	0.648418	0.128539	0.26461	1.335109	0.490964	0.416776	0.447509
Novel_004786	XLOC_05068.	transcript	0.820458	0.79642	1.233199	1.524073	1.254216	0.939243	1.237113	0.647984	1.449662
Novel_004787	XLOC_05068.	transcript	0.404873	0.115316	0.124456	0.126399	0.14197	0.244518	0.393883	0.629056	0.206233
Novel_004788	XLOC_05068.	transcript	0	0.488615	0.037744	0	0	0	0.105219	0.061837	0.043463
Novel_004789	XLOC_05069.	transcript	0.197893	0	0.048347	0.098894	0.076893	0.045053	0.073913	0.096041	0
Novel_004790	XLOC_05069.	transcript	0.014976	0	0	0	0.009198	0	0.007877	0.007141	0
Novel_004791	XLOC_05070.	transcript	0	0	0	0	0	0	0.144488	0	0
Novel_004792	XLOC_05070.	transcript	0.16557	0.166048	0.235982	0.183523	0.125741	0.21257	0.09195	0.377014	0.200167
Novel_004793	XLOC_05071.	transcript	15.78206	19.84401	36.99744	31.23605	37.28421	36.19164	29.21886	20.2736	33.91668
Novel_004794	XLOC_05071.	transcript	0.192796	0.344113	0.137532	0.219551	0.178417	0.421485	0.298152	0.36957	0.073486
Novel_004795	XLOC_05071.	transcript	0	0	0	0.200763	0	0.131207	0.024889	0	0.13323
Novel_004796	XLOC_05071.	transcript	0.068629	0.077867	0.259851	0.008701	0.117525	0.134568	0.143671	0.178183	0
Novel_004797	XLOC_05071.	transcript	0.410808	0.28737	0.366149	0.227192	0.422218	0.340468	0.415527	0.268505	0.324956
Novel_004798	XLOC_05072.	transcript	0.208987	0.001985	0.191432	0.098484	0.535013	0	0.009809	0.027264	0
Novel_004799	XLOC_05072.	transcript	0.205787	0.630343	0.301014	0.436388	0.036069	0.518678	0.831144	0.588912	0.464041
Novel_004800	XLOC_05073.	transcript	0.003626	0.116695	0.377971	0	0	0	0.053602	0.143957	0.106506
Novel_004801	XLOC_05073.	transcript	0.207355	0.530645	0.205163	0.300824	0.351843	0.100565	0.032975	0.015062	0.173591
Novel_004802	XLOC_05073.	transcript	0	0.009727	0	0.028668	0	0.292372	0.06364	0.308766	0
Novel_004803	XLOC_05073.	transcript	0.038583	0	0	0	0.025079	0	0.39592	0.020565	0
Novel_004804	XLOC_05074.	transcript	2.746938	1.338462	0.728943	1.415695	1.75333	1.475148	2.318948	1.538734	1.578693
Novel_004805	XLOC_05074.	transcript	0.046774	0.496141	0.335126	0.205914	0.214956	0.278073	0.335106	0.437987	0.314862
Novel_004806	XLOC_05074.	transcript	1.430259	0.201655	1.71335	1.019702	1.423425	1.335025	2.513037	2.019847	1.357478
Novel_004807	XLOC_05076.	transcript	0.041525	0	0.052321	0	0.077514	0.183087	0	0	0.297713
Novel_004808	XLOC_05076.	transcript	0.161699	0.182671	0.096106	0.091794	0	0.154661	0.298422	0.195607	0.500641
Novel_004809	XLOC_05077.	transcript	0.011382	0.02154	0.425344	0.305283	0	0.014987	0.018779	0.302663	0.526714
Novel_004810	XLOC_05077.	transcript	0.552792	0.133032	1.217329	2.852837	2.033593	1.807901	1.769228	1.327871	3.62251
Novel_004811	XLOC_05088.	transcript	1.02503	1.971025	0.837386	0.735308	0.706976	0.772608	1.31765	0.749442	0.637206
Novel_004812	XLOC_05088.	transcript	0.144013	0.036887	0.16262	0.034485	0.052535	0.049562	0.276599	0.115121	0.060955
Novel_004813	XLOC_05088.	transcript	0	0	0	0	0	0	0	0	0
Novel_004814	XLOC_05089.	transcript	0.115661	0.441379	0	0	0	0	0.046557	0	0

Novel_004815	XLOC_05089.	transcript	0	0	0	0	0	0	0	0	0
Novel_004816	XLOC_05089.	transcript	0.166726	0.03099	0.384057	0.384574	0.655912	0.505053	0.425392	0.534154	0.391735
Novel_004817	XLOC_05094.	transcript	0.220318	0.093825	0.142591	0.132774	0.190017	0.046127	0.191947	0.166648	0.094064
Novel_004818	XLOC_05094.	transcript	0	0	0	0	0	0	0	0	0
Novel_004819	XLOC_05094.	transcript	0.063711	0.210168	0.246984	0.026161	0.285972	0.172405	0.226828	0.095434	0.192835
Novel_004820	XLOC_05094.	transcript	0.45512	0.234695	0.200106	0.36617	0.170078	0.192945	0.41606	0.101217	0
Novel_004821	XLOC_05094.	transcript	0.005968	0	0	0.025307	0.008033	0.010637	0	0.183758	0.018135
Novel_004822	XLOC_05094.	transcript	0.007453	0	0.037969	0.015235	0.062425	0.018021	0.01002	0.100273	0
Novel_004823	XLOC_05094.	transcript	0.156749	0.142008	0.092864	0.199785	0.12648	0.494473	0.133748	0.193109	0.286073
Novel_004824	XLOC_05094.	transcript	0.011886	0.117481	0	0	0.012366	0	0	0	0
Novel_004825	XLOC_05094.	transcript	0.009975	0.195916	0	0.046662	0	0.059995	0.021485	0	0
Novel_004826	XLOC_05094.	transcript	0.000829	0.255597	0.01764	0.016268	0	0.013005	0.032823	0	0.004244
Novel_004827	XLOC_05095.	transcript	2.317327	2.04329	4.051281	5.048362	3.804943	4.805301	3.609666	4.28319	3.851942
Novel_004828	XLOC_05097.	transcript	0	0	0	0	0	0	0.010307	0	0
Novel_004829	XLOC_05097.	transcript	0.145954	0.142045	0.148369	0.135516	0.189287	0.263213	0.131974	0.102491	0.12964
Novel_004830	XLOC_05099.	transcript	0	0	0.000118	0	0.516726	0.427189	0.098816	0.316301	0
Novel_004831	XLOC_05099.	transcript	0.197691	0	0.111211	0.241382	0.206396	0.281006	0.142208	0.029943	0.090707
Novel_004832	XLOC_05099.	transcript	0.368624	0	0.090866	0.279087	0	0	0.223155	0.713702	0.172059
Novel_004833	XLOC_05099.	transcript	0.338437	0	0.412703	0.225664	0.025893	4.60E-05	0.189307	0.001863	0.028529
Novel_004834	XLOC_05099.	transcript	0	0	0	0	0.240455	0	0	0	0
Novel_004835	XLOC_05100.	transcript	0.072539	0.185086	0.183562	0.218228	0.23448	0.153498	0.31248	0.318546	0.143015
Novel_004836	XLOC_05100.	transcript	0	0	0.036146	0.021825	0	0.000149	0.005468	0	0
Novel_004837	XLOC_05100.	transcript	0	0	0	0	0	0.015487	0	0.097076	0.030818
Novel_004838	XLOC_05100.	transcript	0.01403	0.045695	0.018545	0.035929	0.021124	0.017631	0.05508	0.001687	0.010563
Novel_004839	XLOC_05100.	transcript	0.003031	0.015842	0.005034	0	0.018228	1.20E-05	0.020123	4.00E-06	0.032994
Novel_004840	XLOC_05100.	transcript	0.021224	0.04663	0	0.008039	0.02248	0	0.026884	0.156685	0.001526
Novel_004841	XLOC_05100.	transcript	0	1.40E-05	0	0	0	0.046136	0	1.80E-05	0
Novel_004842	XLOC_05100.	transcript	0.101975	0.119497	0	0.083913	0	0.008913	0	0.024078	0
Novel_004843	XLOC_05100.	transcript	0.07325	0	0.021471	0	0.011377	0	0.157959	0.001367	0
Novel_004844	XLOC_05100.	transcript	0.297438	0.473822	0.117091	0.07789	0.128244	0.465393	0.345501	0.071786	0.474256
Novel_004845	XLOC_05100.	transcript	0.040493	0.226424	0.139947	0.164104	0.019884	0.113641	0.11821	0.099205	0.083521
Novel_004846	XLOC_05100.	transcript	0.116841	0.388473	0	0.030626	0.159609	0	0.136751	0.008879	0.07446
Novel_004847	XLOC_05100.	transcript	0.14743	0.158131	0.027267	0.051767	0	0.267889	0	0.028469	0.004046
Novel_004848	XLOC_05100.	transcript	0.042697	0.421505	0	0.037288	0	0	0	0.071175	0.077262
Novel_004849	XLOC_05101.	transcript	0.34077	0.816372	0.634884	0.874489	0.503502	0.73114	0.909757	0.808266	1.461826
Novel_004850	XLOC_05106.	transcript	0	0	0	0	0	0	0	0	0
Novel_004851	XLOC_05106.	transcript	0.053145	0	0.079429	0.026928	0.115744	0.028345	0.235251	0.133452	0.064156
Novel_004852	XLOC_05110.	transcript	0.011189	1.025412	0	0	0.00445	0.010814	0	0.004284	0

Novel_004853	XLOC_05112.	transcript	0.489658	1.453383	0.67018	0.830231	0.78881	0.643912	0.653791	0.550172	0.999126
Novel_004854	XLOC_05113.	transcript	0	0	0	0	0	0	0	0	0
Novel_004855	XLOC_05113.	transcript	0.161917	0.16405	0	0.46406	0.493724	0	0	0.467441	0
Novel_004856	XLOC_05113.	transcript	0.124411	0.347048	0.184733	6.00E-06	0	0.356155	1.80E-05	0	0
Novel_004857	XLOC_05113.	transcript	0	0.074597	0.252826	0.664384	0.560346	0.251525	0.43337	0.60853	0.376269
Novel_004858	XLOC_05115.	transcript	0.520021	0.524734	0.406921	0.215878	0.486127	0.327664	0.660385	0.445718	0.377
Novel_004859	XLOC_05119.	transcript	0	0.060796	0.044101	0.063336	0.030451	0.056307	0	0	0
Novel_004860	XLOC_05119.	transcript	0	0	0	0.154707	0	0.023246	0	0	0
Novel_004861	XLOC_05119.	transcript	0.170687	0.238228	0.070833	0	0.145716	0.048317	0.165423	0.216254	0.179169
Novel_004862	XLOC_05119.	transcript	0	0	0.078045	0	0	0.104123	0	0	0
Novel_004863	XLOC_05119.	transcript	0	0	0	0	0	0	0	0.035317	0
Novel_004864	XLOC_05119.	transcript	0	0	0	0	0.188576	0.053428	0	0.256207	0
Novel_004865	XLOC_05119.	transcript	0.203033	0.078533	0.234064	0.101657	0.032913	0.147538	0.025796	0.001183	0.092459
Novel_004866	XLOC_05120.	transcript	0.374341	0.982712	0.674201	0.560188	0.54818	0.779961	0.688563	0.553489	0.496267
Novel_004867	XLOC_05120.	transcript	0.140927	0	0	0	0	0	0	0.033554	0
Novel_004868	XLOC_05122.	transcript	0.205111	0.046801	0.237576	0.11355	0.108328	0	0	0.058329	0.243331
Novel_004869	XLOC_05122.	transcript	0.231836	0.340522	0	0	0	0	0.478483	0.179329	0
Novel_004870	XLOC_05122.	transcript	0.286495	0.139133	0.401349	0.248984	0.420728	0.245135	0.460136	0.313078	0.221467
Novel_004871	XLOC_05122.	transcript	0.147114	0	0.000576	0.02606	0	0.066373	0	0	0
Novel_004872	XLOC_05124.	transcript	0.354016	0.365475	0.332484	0.159731	0.36279	0.300613	0.413461	0.278743	0.127392
Novel_004873	XLOC_05124.	transcript	0.188502	0.400255	0.35306	0.353307	0.281013	0.347484	0.404511	0.226207	0.560869
Novel_004874	XLOC_05124.	transcript	1.994445	2.499525	3.40044	0	0.569139	6.218974	0.238714	0.246749	4.833136
Novel_004875	XLOC_05124.	transcript	2.881571	0	0	4.308709	1.451007	0	0	0	0
Novel_004876	XLOC_05124.	transcript	0	0	0	0.711883	0	0	4.349773	3.983996	0
Novel_004877	XLOC_05129.	transcript	0.037009	0.229499	0.374381	0.048169	0.147055	0.106838	0.189924	0.186282	0
Novel_004878	XLOC_05129.	transcript	0.363145	0.420368	0.021073	0.018474	0.176705	0.014423	0.437093	0.000212	0.391795
Novel_004879	XLOC_05129.	transcript	0	0.129407	0	0	0.205842	0.407849	0	0.303849	0
Novel_004880	XLOC_05129.	transcript	0	0	0.270107	0	0.026053	0	0	0.178924	0
Novel_004881	XLOC_05129.	transcript	0	0	0.048251	0.433371	0	0	0	0.000834	0
Novel_004882	XLOC_05130.	transcript	0.743194	0.68436	1.332502	1.269629	1.080074	1.298064	2.05546	1.444155	3.740862
Novel_004883	XLOC_05131.	transcript	0.018677	0.383663	0.18837	0.295021	0.383524	0.670921	0.203468	1.306353	0.046976
Novel_004884	XLOC_05131.	transcript	0.886233	2.360623	1.855563	0.799831	1.375957	1.045101	1.532464	0.669955	0.666723
Novel_004885	XLOC_05131.	transcript	0.125691	0	0	0.031603	0	0	0.132703	0.019955	0.050338
Novel_004886	XLOC_05134.	transcript	0.089108	0.350761	0.134433	0.03215	0.033768	0.031058	0.132632	0	0.033538
Novel_004887	XLOC_05134.	transcript	0.32447	0.144051	0.437752	0.164924	0	0.134039	0.297893	0	0.229885
Novel_004888	XLOC_05136.	transcript	0	0	0	0	0	0	0	0	0
Novel_004889	XLOC_05140.	transcript	0	1.86352	0	0	0	0	0	0	1.475478
Novel_004890	XLOC_05140.	transcript	0.094769	0	0.086203	0.065584	0.079642	0.070495	0.141951	0.062665	0.380457

Novel_004891	XLOC_05140.	transcript	0.09521	1.916277	0.0546	0.040913	0.043703	0.076002	0.331885	0.076645	0.746275
Novel_004892	XLOC_05141.	transcript	0.105598	0	0.113214	0.189503	0.256497	0.199964	0.265026	0.261781	0.516181
Novel_004893	XLOC_05147.	transcript	0.356361	0.104331	0.054696	0.260645	0.557052	0.386742	0.54396	0.602364	0.280089
Novel_004894	XLOC_05149.	transcript	0.065018	0.149332	0.132346	0.069956	0.131635	0.100708	0.053533	0.256655	0.070126
Novel_004895	XLOC_05150.	transcript	0.653227	0.596101	0.528189	0.395453	0.477746	0.504694	0.709801	0.674675	0.382548
Novel_004896	XLOC_05153.	transcript	0.181699	0.176362	0.270276	0.154997	0.008429	0.13473	0.363637	0.17295	0
Novel_004897	XLOC_05153.	transcript	0	0	0	0	0	0	0	0	0
Novel_004898	XLOC_05153.	transcript	0.133941	0	0.274665	0.116939	0.055455	0.362222	0.341029	0.174091	0.213941
Novel_004899	XLOC_05153.	transcript	0.200888	0.351991	0.212874	0.149163	0.098951	0.279001	0.434971	0.194812	0.083698
Novel_004900	XLOC_05153.	transcript	0.310069	0	0	0.2943	0.471852	0	0	0.350912	0.23482
Novel_004901	XLOC_05154.	transcript	0	0	0.240944	0	0.102835	0	0	0	0
Novel_004902	XLOC_05154.	transcript	0.417957	0.66228	0	0.256889	0.092569	0.3304	0.738982	0.472413	0.542456
Novel_004903	XLOC_05156.	transcript	0.214311	0.348593	0.362045	0.138602	0.281598	0.393739	0.315991	0.298889	0.302062
Novel_004904	XLOC_05156.	transcript	0.740215	0.283458	0.44838	0.353418	0.507921	0.616164	0.433678	0.524824	0.32813
Novel_004905	XLOC_05158.	transcript	0.488732	0.039196	0.224934	0.243556	0.379398	0.206669	0.240885	0.324621	0.188453
Novel_004906	XLOC_05159.	transcript	0.26853	0.609314	0	0.634116	0	0.429657	0	0	0
Novel_004907	XLOC_05159.	transcript	0	0.09797	0	0	0	0	0	0	0
Novel_004908	XLOC_05159.	transcript	0	0	0.472089	0	0.458545	0	0.550912	0.629129	0.377396
Novel_004909	XLOC_05162.	transcript	0.012941	0	0	0.028955	0	0.00521	0.009565	0.033456	0.005118
Novel_004910	XLOC_05162.	transcript	0	0	0	0	0	0	0	0	0
Novel_004911	XLOC_05162.	transcript	0	0	0	0	0.034155	0	0	0	0
Novel_004912	XLOC_05167.	transcript	0	0	0	0	0	0	0	0	0
Novel_004913	XLOC_05167.	transcript	0.012732	0.025264	0.013461	0.010671	0.07014	0.026738	0	0.094989	0
Novel_004914	XLOC_05171.	transcript	0.776105	0.267264	0.465983	0.380604	0.576317	0.581385	0.759645	0.558194	0.50427
Novel_004915	XLOC_05173.	transcript	0.325832	0.711575	0.2661	0.640528	0.45688	0.4265	0.633557	0.445479	0.506201
Novel_004916	XLOC_05173.	transcript	0	0	0	0	0	0	0	0	0
Novel_004917	XLOC_05173.	transcript	0.641451	0.614109	0.389081	0.136975	0.452378	0.375351	0.666738	0.218925	0.23582
Novel_004918	XLOC_05173.	transcript	0	0	0	0	0	0	0	0	0
Novel_004919	XLOC_05173.	transcript	0	0.009499	0	0.21165	0	0	0.001458	0.279952	0
Novel_004920	XLOC_05173.	transcript	0.529851	0.973189	0.40363	0.426565	0.488746	0.467721	0.68853	0.655453	0.334365
Novel_004921	XLOC_05173.	transcript	0	0	0	0	0	0	0	0	0
Novel_004922	XLOC_05190.	transcript	0.914251	0.712865	0.002025	0.591962	0.567498	0.31709	0.379869	0.322975	0
Novel_004923	XLOC_05190.	transcript	1.15413	0.315625	0.005607	0	0.466941	0	1.066355	0.450347	0
Novel_004924	XLOC_05192.	transcript	0.052636	0	0.553267	0.170498	0.319611	0.373846	0.432553	0.042375	0.236313
Novel_004925	XLOC_05192.	transcript	0.246707	0.11948	0.286544	0.150147	0.172132	0.158319	0.373143	0.215785	0.158984
Novel_004926	XLOC_05192.	transcript	0	0	0	0	0.152578	0	0	0	0
Novel_004927	XLOC_05192.	transcript	0	0	0	0	0	0	0	0	0
Novel_004928	XLOC_05195.	transcript	0.351891	0.500435	0.344273	0.238178	0.375177	0.364566	0.261731	0.264764	0.261446

Novel_004929	XLOC_05196.	transcript	0.371614	0.136115	0.143574	0.116024	0.151439	0.202872	0.392569	0.244895	0.270631
Novel_004930	XLOC_05196.	transcript	0.454051	0.254818	0.031174	0.353366	0.00312	0.15016	0.292173	0.205096	0.211295
Novel_004931	XLOC_05210.	transcript	0.560781	0.746633	0.329143	0.261298	0.389415	0.400305	0.595431	0.28488	0.326618
Novel_004932	XLOC_05213.	transcript	0	0.100723	0.390248	0.077688	0	0.204329	0.104127	0.042045	0
Novel_004933	XLOC_05213.	transcript	0.231876	0	0	0	0.031701	0.122207	0.213675	0.184614	0.035821
Novel_004934	XLOC_05216.	transcript	0.837475	0.062031	0.366701	0.566416	0.680018	0.345239	1.073592	0.589875	0.953039
Novel_004935	XLOC_05217.	transcript	0	0.007002	0	0	0	0.084188	0.003762	0	0
Novel_004936	XLOC_05218.	transcript	0.484181	0.031871	0.021059	0	0	1.906567	0.644772	0	0
Novel_004937	XLOC_05218.	transcript	0	0	0.888047	2.570033	1.477993	0	0	1.402491	0.408483
Novel_004938	XLOC_05218.	transcript	19.87279	2.490062	26.84949	21.1659	21.68909	26.6409	38.09479	24.52824	34.15974
Novel_004939	XLOC_05219.	transcript	0.047558	0.077658	0.052931	0.002318	0.097038	0	0.005552	0.065377	0.175741
Novel_004940	XLOC_05219.	transcript	0.138612	0.434751	0.125361	0.165649	0.034659	0.26005	0.188815	0.045254	0.147581
Novel_004941	XLOC_05219.	transcript	0.263469	0.555427	0.07837	0.001644	0.257283	0.203209	0.233688	0.778834	0.071782
Novel_004942	XLOC_05219.	transcript	0.365913	0.350932	0.295519	0.595392	0.412071	0.406554	0.777519	0.359084	0.554442
Novel_004943	XLOC_05226.	transcript	0.147089	0.174385	0.268938	0.158704	0.27564	0.220923	0.264884	0.132802	0
Novel_004944	XLOC_05226.	transcript	1.285026	0.151379	0.365368	0.263277	0.054186	0.871938	0.941288	0.170141	0.499882
Novel_004945	XLOC_05226.	transcript	0.19571	0	0.388734	0.112202	0	0	0	0.143116	0.147746
Novel_004946	XLOC_05226.	transcript	0	0.059443	0.356387	0	0.063016	0	0.276241	0	0
Novel_004947	XLOC_05226.	transcript	0.259772	0.090492	0.32691	0.107841	0.32209	0.253855	0.378848	0.140432	0.18515
Novel_004948	XLOC_05228.	transcript	0.222213	0.293589	0.236097	0.382028	0.173456	0.533914	0.313693	0.342862	0.200577
Novel_004949	XLOC_05228.	transcript	0.162468	0.150322	0.149216	0.183292	0.305682	0.089345	0.3166	0.29667	0.350336
Novel_004950	XLOC_05228.	transcript	0	0	0	0	0	0.336035	0	0.084804	0.084171
Novel_004951	XLOC_05228.	transcript	0	0.057996	0.044537	0.011647	0	2.155715	0.024457	0.110303	1.646286
Novel_004952	XLOC_05228.	transcript	0	0	0	0	0	0	0	0.12024	0
Novel_004953	XLOC_05228.	transcript	0.145949	0.022554	0.007605	0.01297	0.029836	0	0.266296	0.133433	0.103506
Novel_004954	XLOC_05228.	transcript	0.394001	0.180022	0.507213	0.493245	0.50951	0.355381	0.493095	0.170859	0.327705
Novel_004955	XLOC_05228.	transcript	0.019883	0.011403	0.007001	0.012474	0.017456	0	0	0	0.009663
Novel_004956	XLOC_05228.	transcript	0	0	0	0	0	0	0	0.318992	0
Novel_004957	XLOC_05232.	transcript	0	0	0	0	0	0	0	0	0
Novel_004958	XLOC_05233.	transcript	0.619139	0.466986	0.369666	0.300283	0.426616	0.377802	0.56527	0.646316	0.218767
Novel_004959	XLOC_05233.	transcript	0.09975	0.337453	0.437677	0.378214	0.348558	0.398655	2.102123	2.281655	0.79605
Novel_004960	XLOC_05233.	transcript	1.5163	1.496541	3.038286	3.275365	3.576398	3.427546	1.63099	0.597184	5.220917
Novel_004961	XLOC_05233.	transcript	0	0	0	0	0	0	0	0	0
Novel_004962	XLOC_05236.	transcript	0.213184	4.049762	0.054546	0.14354	0	0.797485	0.04572	0.046503	0.071308
Novel_004963	XLOC_05236.	transcript	0	11.09561	0	0.024394	0	2.261196	0	0.084418	0
Novel_004964	XLOC_05237.	transcript	0.134012	0.118483	0.06436	0	0.092947	0	0	0.085858	0.202835
Novel_004965	XLOC_05237.	transcript	0.440823	0.428593	0.286617	0.41267	0.28966	0.218023	0.311814	0.191498	0.736028
Novel_004966	XLOC_05237.	transcript	0	0.54287	0	0	0	0.120535	0	0	0

Novel_004967	XLOC_05239.	transcript	0.038276	1.024607	0.039256	0.024552	0.068074	0.056035	0.07603	0.024106	0.164634
Novel_004968	XLOC_05239.	transcript	0.031692	1.298585	0.13402	0.053122	0.032414	0.076474	0	0.03629	0
Novel_004969	XLOC_05240.	transcript	0	1.62356	0	0.323403	0.38687	0.238586	0.18844	0.149029	0.166539
Novel_004970	XLOC_05242.	transcript	0	1.13294	0	0	0	0	0.015895	0	0.005053
Novel_004971	XLOC_05242.	transcript	0	0.238683	0.039091	0.046943	0.082881	0	0	0	0.196398
Novel_004972	XLOC_05242.	transcript	0.624616	4.456982	0.310161	0.578727	1.00333	0.284551	0.445448	0.209877	0.72433
Novel_004973	XLOC_05248.	transcript	0.590507	1.482093	0.490473	0	0.408772	0.461219	0.424728	0.33231	1.023256
Novel_004974	XLOC_05248.	transcript	0	0	0	0.533661	0	0	0	0	0
Novel_004975	XLOC_05250.	transcript	0	0.029772	0.037375	0.0232	0.450643	8.10E-05	0.028152	0.000337	0
Novel_004976	XLOC_05250.	transcript	0.58377	0	0.497311	0.585567	0.011999	1.01608	0.259217	0.799304	0.507044
Novel_004977	XLOC_05250.	transcript	0.004345	0	0	0	0.095246	0.099553	0	0.045611	0.072557
Novel_004978	XLOC_05251.	transcript	0.055099	0.13617	0.306329	0.216357	0.170911	0.160272	0.025587	0.232584	0.21344
Novel_004979	XLOC_05251.	transcript	0	0	0	0	0	0	0.491198	0	0
Novel_004980	XLOC_05251.	transcript	0.282957	0.144439	0.256126	0.555991	0.224864	0.305726	0.23539	0.305938	0.196239
Novel_004981	XLOC_05253.	transcript	0.060056	0	0	0	0	0	0.110623	0	0
Novel_004982	XLOC_05253.	transcript	0.05009	0.338201	0.081887	0	0.022803	0.147661	0.030257	0.006242	0
Novel_004983	XLOC_05253.	transcript	0.041743	0.446865	0.045193	0	0.067782	0.079743	0.122235	0	0.09204
Novel_004984	XLOC_05253.	transcript	0	0.581029	0.263265	0	0	0.121625	0.037037	0	0
Novel_004985	XLOC_05256.	transcript	0.122294	0	0	0	0	0	0.001876	0	0.145661
Novel_004986	XLOC_05257.	transcript	0.076734	0.407356	0	0	0	0	0.050399	0.021502	0.066829
Novel_004987	XLOC_05258.	transcript	0.079969	0.280551	0	0	0	0.067315	0.269832	0	0
Novel_004988	XLOC_05258.	transcript	0	0.048862	0	0	0	0	0	0	0
Novel_004989	XLOC_05258.	transcript	0	0.024325	0	0	0	0	0	0	0
Novel_004990	XLOC_05260.	transcript	0.225407	0.47239	0.060163	0.069592	0.076633	0.085848	0.244545	0.094728	0.279988
Novel_004991	XLOC_05260.	transcript	3.151537	6.684854	2.930724	3.05866	3.58742	3.395977	6.075335	0.707503	2.374449
Novel_004992	XLOC_05260.	transcript	2.493519	0	0	0	0	0	0	0	0
Novel_004993	XLOC_05260.	transcript	0.229391	1.531123	2.028903	1.32614	0.64046	0.581016	2.085057	3.702363	6.724737
Novel_004994	XLOC_05263.	transcript	1.289493	0.994545	2.168948	2.121625	2.04036	1.761769	1.6467	1.913728	1.983461
Novel_004995	XLOC_05263.	transcript	0	0	0	0.004177	0	0.034631	0	0	0.315371
Novel_004996	XLOC_05263.	transcript	0	0.091296	0.040945	0.051085	0	0.110904	0.036712	0.057504	0
Novel_004997	XLOC_05263.	transcript	0.172913	0	0.03785	0.038734	0	0	0.137618	0.073837	0
Novel_004998	XLOC_05263.	transcript	0	0.105782	0.060423	0.049816	0	0	0.039488	0.0966	0
Novel_004999	XLOC_05263.	transcript	0	0	0.056944	0.060094	0	0	0.062172	0.108518	0
Novel_005000	XLOC_05263.	transcript	0.091098	0.258086	0.249126	0.134566	0.439685	0.246285	0.253171	0.099742	0
Novel_005001	XLOC_05264.	transcript	0.298093	0.419567	0.387788	0.098463	0.446761	0.430083	0.312712	0.279956	0.287548
Novel_005002	XLOC_05264.	transcript	0.205054	0.283042	0.272937	0.266568	0.165407	0.167268	0.289476	0.150795	0.473924
Novel_005003	XLOC_05264.	transcript	0.692341	0.604467	0.631502	0.387754	0.514364	0.264609	0.463718	1.151335	0.598054
Novel_005004	XLOC_05264.	transcript	0	0	0	0	0	0	0	0	0

Novel_005005	XLOC_05264.	transcript	0.089487	1.038723	0.023572	0.014092	0.12611	0.073684	0.067038	0.054436	0.102953
Novel_005006	XLOC_05265.	transcript	0.977497	1.140239	1.371217	1.227552	1.643616	2.423204	1.640234	1.729718	0.971488
Novel_005007	XLOC_05267.	transcript	1.581607	2.771664	1.365549	1.782266	1.660339	1.496765	2.40344	1.871545	1.58507
Novel_005008	XLOC_05267.	transcript	0	0	0	0	0	0	0	0	0
Novel_005009	XLOC_05270.	transcript	0.596332	0.726865	0.528033	0.277399	0.281914	0.220871	0.37313	0.462894	0.568382
Novel_005010	XLOC_05274.	transcript	0.014091	0.003867	0.07004	0.550744	0.131107	0.099337	0.025983	0.121352	0
Novel_005011	XLOC_05274.	transcript	0	0	0.21657	1.150288	4.60E-05	0	0	0	0
Novel_005012	XLOC_05274.	transcript	0	0	0	0	0.198515	0	0	0	0
Novel_005013	XLOC_05274.	transcript	0	0	0.032984	0.205563	0.150627	0	0	0.224132	0
Novel_005014	XLOC_05274.	transcript	0	0	0	0	0	0	0	0.22267	0
Novel_005015	XLOC_05274.	transcript	0.090929	0	0	0	0	0	0	0.187625	0.015436
Novel_005016	XLOC_05275.	transcript	0.12559	0.16846	0.230385	0.206139	0.362295	0.229788	0.25172	0.311139	0.199362
Novel_005017	XLOC_05275.	transcript	0	0	0	0.057125	0	0	0	0	0
Novel_005018	XLOC_05276.	transcript	0	0.008856	0.072114	0.003052	0.079375	0	0	0	0
Novel_005019	XLOC_05276.	transcript	0.015053	0.203672	0.004321	0	0.003849	0	0.03368	0.008618	0.400607
Novel_005020	XLOC_05277.	transcript	0.156277	0	0	0	0	0	0	0.072867	0.407567
Novel_005021	XLOC_05277.	transcript	0.308364	0.284525	0.380812	0.458935	0.439159	0.427409	0.446033	0.344884	0.793097
Novel_005022	XLOC_05277.	transcript	0	0	0	0	0	0	0	0.102807	0
Novel_005023	XLOC_05277.	transcript	0	0	0	0	0	0	0.368536	0	0
Novel_005024	XLOC_05279.	transcript	0.298551	0.057625	0.111668	0.058076	0.180185	0.028341	0.339757	0.118605	0.036758
Novel_005025	XLOC_05281.	transcript	0	0.576046	0.078547	0.005377	0.08919	0.013293	0.003636	0.041158	0.304002
Novel_005026	XLOC_05281.	transcript	0.336548	0.502607	0.349918	0.514447	0.500337	0.537575	0.814605	0.5093	0.235149
Novel_005027	XLOC_05282.	transcript	0.142767	0	0.128024	0.068103	0.195017	0.157079	0.37696	0.22978	0.063435
Novel_005028	XLOC_05282.	transcript	0.419861	0.302256	0.258447	0.007013	0.124998	0.163973	0.191623	0.060184	0.125645
Novel_005029	XLOC_05282.	transcript	0.119674	0.006651	0.001796	0.07709	0.002006	0.002562	0.002936	0.073176	0.056655
Novel_005030	XLOC_05282.	transcript	0	0	0	0	0	0.025354	0.002904	0.00259	0.030504
Novel_005031	XLOC_05282.	transcript	0	0	0	0	0	0	0	0	0
Novel_005032	XLOC_05282.	transcript	0	0	0	0	0	0	0	0	0
Novel_005033	XLOC_05282.	transcript	0.65131	0.369333	0.682247	0.617686	0.548696	0.729014	0.797686	0.852306	0
Novel_005034	XLOC_05282.	transcript	0	0	0	0	0	0	0	0	0.600895
Novel_005035	XLOC_05283.	transcript	0.232495	0.257962	0.366159	0.429673	0.419551	0.323475	0.387381	0.366857	0.34354
Novel_005036	XLOC_05284.	transcript	0.289671	0.014742	0.188192	0.243897	0.294756	0.24186	0.399533	0.301681	0.540276
Novel_005037	XLOC_05285.	transcript	0.085124	0.015056	0.206938	0.199732	0.102266	0.17379	0.208565	0.223087	0.23464
Novel_005038	XLOC_05286.	transcript	0.309326	0.117229	0.399648	0.383149	0.467222	0.326111	0.769591	0.257361	0.479611
Novel_005039	XLOC_05286.	transcript	0.195588	0	0.304748	0	0.332493	0	0.336118	0.125994	0
Novel_005040	XLOC_05286.	transcript	0.054111	0.337695	0.041003	0.131886	0.085339	0.167875	0.196664	0.099724	0.318603
Novel_005041	XLOC_05287.	transcript	0.509485	0.70634	1.002513	1.09491	0.870149	1.008778	0.960729	0.719968	0.917553
Novel_005042	XLOC_05289.	transcript	0.306512	0.314309	0.175963	0.426237	0.368244	0.267442	0.40956	0.044136	0.379314

Novel_005043	XLOC_05289	transcript	0	0.004844	0.081035	0.015448	0	0	0.116151	0.031022	0.074184
Novel_005044	XLOC_05289	transcript	0.37388	0.175852	0.531893	0.612603	0.525019	0.801646	0.80519	0.639813	0.43689
Novel_005045	XLOC_05289	transcript	0	0	0.722275	0	0.302809	0	0.001296	0.001188	0.274501
Novel_005046	XLOC_05289	transcript	0.994508	0.635819	0.699705	0.486285	0.624803	0.80921	0.985633	0.536716	0.590009
Novel_005047	XLOC_05289	transcript	0.036946	1.588404	0.922509	1.58306	0.046543	0.152602	0.279413	0.826876	0.601205
Novel_005048	XLOC_05289	transcript	0.239612	0.701334	0.411633	0.18111	1.322267	0.117768	0.224719	0.429628	2.118614
Novel_005049	XLOC_05290	transcript	0	0.226206	0	0	0.137463	0	0.11126	0.220089	0
Novel_005050	XLOC_05290	transcript	0.516081	0.269574	0.099649	1.1359	1.086014	0.885342	0.214468	1.078078	1.173908
Novel_005051	XLOC_05290	transcript	0	0.000736	0	0	0	0	0	0	0
Novel_005052	XLOC_05290	transcript	0.637437	0.627707	1.150152	0.750399	0.862571	1.253186	0.827852	0.293845	1.137041
Novel_005053	XLOC_05290	transcript	0.131374	0.446544	0.209471	0.267824	0.24142	0.167953	0.381778	0.24219	0.360636
Novel_005054	XLOC_05290	transcript	0.339965	0.031597	0.284254	0.069439	0.315141	0.885123	0.343692	0.381069	0
Novel_005055	XLOC_05291	transcript	2.223397	0.353055	1.463206	2.096749	1.875277	1.65088	2.578245	2.442073	0.846903
Novel_005056	XLOC_05291	transcript	3.334638	3.306912	2.769584	3.669985	2.870899	2.910313	4.120494	4.730274	2.247886
Novel_005057	XLOC_05291	transcript	0	0	0.034247	0	0	0	0	0.018595	0
Novel_005058	XLOC_05291	transcript	0.114186	0.235746	0.190798	0.262777	0.204815	0.128877	0.002993	0	0.211249
Novel_005059	XLOC_05291	transcript	0	0	0	0	0	0	0.447516	0.354484	0
Novel_005060	XLOC_05292	transcript	0.100012	0	0.047945	0.243405	0.456503	0	0.154271	0.196551	0.416868
Novel_005061	XLOC_05292	transcript	0.085664	0.18308	0	0.091001	0	0.036059	0.265653	0	0
Novel_005062	XLOC_05292	transcript	0.108055	0	0	0.073083	0	0.299738	0.071553	0.10343	0
Novel_005063	XLOC_05292	transcript	0.000363	0	0.599196	0	0	0	0	0	0
Novel_005064	XLOC_05292	transcript	0.018995	0.24267	0.06555	0.014662	0	0	0.036174	0.011193	0.107929
Novel_005065	XLOC_05292	transcript	0.122849	0.191671	0.268214	0.231436	0.217484	0.430184	0.320339	0.327232	0.432257
Novel_005066	XLOC_05292	transcript	0.119627	0	0	0	0.107979	0	0.007052	0.006628	0.005958
Novel_005067	XLOC_05295	transcript	1.246802	2.0199	2.538657	3.660011	2.115151	3.686152	2.502698	1.740916	2.252092
Novel_005068	XLOC_05296	transcript	0.046181	0.10502	0.040363	0	0	0.251519	0	0.131938	0.157345
Novel_005069	XLOC_05296	transcript	0	0.077827	0	0.040849	0.004844	0	0.075356	0	0
Novel_005070	XLOC_05299	transcript	0.154621	0.012193	0.148096	0.204205	0.097906	0.147953	0.195106	0.109839	0.06907
Novel_005071	XLOC_05299	transcript	0.574532	1.097134	0.81522	0.956218	0.843175	1.031159	1.183308	0.82053	1.026416
Novel_005072	XLOC_05299	transcript	0.269702	0.004234	0	0	0	0	0	0	0
Novel_005073	XLOC_05299	transcript	0	0.55211	0	0.003762	0.009183	0	0.013429	0	0
Novel_005074	XLOC_05300	transcript	0.030896	0	0	0.22452	0	0	0	0.094479	0.161946
Novel_005075	XLOC_05300	transcript	0	0.191408	0.123205	0.043613	0.164553	0.043492	0.180364	0.070204	0
Novel_005076	XLOC_05303	transcript	0	0.055879	0	0	0	0	0.153411	0.214602	0
Novel_005077	XLOC_05303	transcript	0.663698	0.776602	0.613338	0.361136	0.356147	0.242426	0.702467	0.393432	0.43383
Novel_005078	XLOC_05303	transcript	0	0.272296	0.000266	0.258023	0.261466	0.363105	0.133735	0.188378	0.156755
Novel_005079	XLOC_05303	transcript	0	0	0.101185	0	0.025028	0	0	0	0
Novel_005080	XLOC_05303	transcript	0.309367	0.881726	0.423287	0.606715	0.610833	1.014167	0.950613	0.940488	0.834187

Novel_005081	XLOC_05305.	transcript	0	0	0	0	0	0	0	0	0
Novel_005082	XLOC_05306.	transcript	0.018702	0	0	0.029747	0	0.012161	0.051951	0	0.012199
Novel_005083	XLOC_05306.	transcript	0.323291	0.498101	0.206477	0.119252	0.282719	0.209928	0.353514	0.454594	0.126537
Novel_005084	XLOC_05306.	transcript	0	0	0.366547	0.534266	0.402766	0.481476	0.599393	0	0.452484
Novel_005085	XLOC_05308.	transcript	0.077414	0.029592	0.077292	0	0	0	0	0.192971	0
Novel_005086	XLOC_05308.	transcript	0.320954	0.666548	0.459239	0.497689	0.681595	0.403028	0.435132	0.388523	0.533548
Novel_005087	XLOC_05308.	transcript	0	0	0	0	0	0	0	0	0
Novel_005088	XLOC_05308.	transcript	0.465107	0.183078	0.443846	0.028029	0.618754	0.613159	0.431206	0.310188	0.120955
Novel_005089	XLOC_05308.	transcript	0	0	0	0.52227	0	0	0	0	0.30294
Novel_005090	XLOC_05308.	transcript	0	0	0	0	0	0	0	0	0
Novel_005091	XLOC_05308.	transcript	0.014119	0.017041	0.017847	0.012486	0.016809	0.010344	0.266454	0.323618	0
Novel_005092	XLOC_05310.	transcript	1.184912	0.168788	1.065069	1.246543	0	0.464968	1.278818	1.436457	0
Novel_005093	XLOC_05310.	transcript	0.009006	1.082751	0.009414	0.206087	1.068966	0.196861	0	0.004689	1.118615
Novel_005094	XLOC_05310.	transcript	0.012359	0.051374	0.282379	0	0	0.114501	0.198422	0.001092	0
Novel_005095	XLOC_05310.	transcript	0.221401	0.21903	0.161582	0.281377	0.234048	0.229009	0.078399	0.277057	0.260977
Novel_005096	XLOC_05311.	transcript	0.396748	1.299926	0.934808	1.827932	1.330725	1.138733	1.161209	1.333995	0.842976
Novel_005097	XLOC_05314.	transcript	0.521966	0.263061	0.374794	0.422248	0.200197	0.185431	1.134897	0.697443	0.482411
Novel_005098	XLOC_05319.	transcript	0.141578	0.166365	0.51407	0.637825	0.163269	0.245273	0.140005	0.155048	0.176285
Novel_005099	XLOC_05319.	transcript	0.504979	0	1.609417	0.611069	0.866409	1.659092	0.634242	0.718188	0.1695
Novel_005100	XLOC_05325.	transcript	0.782783	0.891987	1.368674	2.242522	1.448606	1.825486	1.644626	1.550961	2.307348
Novel_005101	XLOC_05325.	transcript	0.086773	0.732074	0.080466	0.048456	0.17803	0.058021	0.103984	0.144047	0.120472
Novel_005102	XLOC_05325.	transcript	0.077851	1.21017	0.058241	0.02329	0.101259	0.086441	0.215307	0.062624	0.449191
Novel_005103	XLOC_05327.	transcript	0	0.516721	0.641847	0.643909	0.488781	0	0	0.397078	0
Novel_005104	XLOC_05327.	transcript	0	0.171683	0	0.411257	0.372452	0	0.634639	0.31746	0.555109
Novel_005105	XLOC_05327.	transcript	0.138992	0.970011	0.600493	0.256643	0.404041	0.54062	0.386241	0.191406	1.101789
Novel_005106	XLOC_05327.	transcript	0.527179	1.226169	0.109787	0.552823	0.355724	0.774035	0.620027	0.586747	0.007077
Novel_005107	XLOC_05328.	transcript	0.157486	0	0.21481	0.281465	0.247434	0.116595	0.22381	0.190929	0.03334
Novel_005108	XLOC_05328.	transcript	0	0	0.054048	0.270927	0.081075	0	0	0.048952	0
Novel_005109	XLOC_05330.	transcript	0.612716	1.236794	0.627967	0.543305	0.613664	0.80985	0.84692	0.621628	0.830809
Novel_005110	XLOC_05330.	transcript	0.547724	0.37516	0.290533	0.455947	0.696863	0.607411	0.611372	0.512156	0.426004
Novel_005111	XLOC_05331.	transcript	0.139364	0.018738	0.710987	0.13614	0.379552	0	0.099274	0	0.027527
Novel_005112	XLOC_05332.	transcript	0.953435	0.007867	1.142126	0.48682	0.652589	0.620631	1.120256	0.127439	0.814991
Novel_005113	XLOC_05332.	transcript	5.755635	1.024872	4.154791	3.45114	6.852833	5.193515	11.69689	7.07962	21.11547
Novel_005114	XLOC_05332.	transcript	6.479731	0.401345	3.139192	1.219389	5.495405	2.847062	9.883642	5.050111	7.518691
Novel_005115	XLOC_05332.	transcript	0	0	0	2.498381	0	0	0	0	0.020504
Novel_005116	XLOC_05332.	transcript	0	0	3.653476	4.797581	0	0	0	0	0
Novel_005117	XLOC_05333.	transcript	0	0	0	0	0	0	0	0	0
Novel_005118	XLOC_05333.	transcript	0	0.014528	0.057083	0	0.561773	0	0	0	0

Novel_005119	XLOC_05333.	transcript	0	0	0	0	0	0	0	0	0
Novel_005120	XLOC_05333.	transcript	0.395961	0	0.082915	0	0.240673	0	0	0	0
Novel_005121	XLOC_05333.	transcript	0	0.468316	0	0.358294	0	0	0.239547	0	0
Novel_005122	XLOC_05333.	transcript	0.44478	0.010297	0.567149	0.448063	0	0.809726	0.746116	0.830496	0.593911
Novel_005123	XLOC_05336.	transcript	0.166032	0.052318	0.474051	0.353811	0.370772	0.403736	0.55273	0.354366	0.301718
Novel_005124	XLOC_05337.	transcript	0.259168	0.219116	0.208968	0.013532	0.141583	0.660081	0.606519	0.245456	0.035906
Novel_005125	XLOC_05337.	transcript	0.316851	0	0.242577	0.171052	0.176861	0	0	0.411458	0
Novel_005126	XLOC_05337.	transcript	0.271174	0.071156	0.21837	0.289419	0.223797	0	0.421122	0.101551	0.290679
Novel_005127	XLOC_05337.	transcript	0.09007	0.446634	0.145461	0.171818	0.161431	0.219524	0.145329	0.234868	0.112818
Novel_005128	XLOC_05338.	transcript	0.206016	0.059984	0.038636	0.03443	0.00233	0.232846	0.033018	0.011866	0.107513
Novel_005129	XLOC_05338.	transcript	0.261662	0.292365	0.362895	0.202972	0.467306	0	0	0	0
Novel_005130	XLOC_05338.	transcript	0.055829	0.276188	0.336089	0.35089	0.209922	0.545262	0.533246	0.865756	0.758754
Novel_005131	XLOC_05339.	transcript	0.511046	1.789397	1.060609	0.736575	0.737566	0.637081	1.024886	0.803005	1.107573
Novel_005132	XLOC_05339.	transcript	0.54548	0.717614	1.444585	0.519359	0.17906	0.628292	0.952023	1.147608	0.895641
Novel_005133	XLOC_05339.	transcript	0.280009	0.62324	0.180333	0.550674	0.646031	0.38294	0.73124	0.322341	0.554393
Novel_005134	XLOC_05345.	transcript	0	0	0.039193	0.165362	0	0.144083	0.051433	0.059876	0.057162
Novel_005135	XLOC_05349.	transcript	0.038301	0.868992	0.010705	0.005035	0.004142	0.00733	0.073514	0.011502	0.040727
Novel_005136	XLOC_05349.	transcript	0.221057	1.28791	0	0.188326	0.003966	0	0.316702	0.0315	0.446317
Novel_005137	XLOC_05349.	transcript	2.30E-05	0.422564	0.026139	0.004297	0	0.101458	0	0.042232	0.138693
Novel_005138	XLOC_05350.	transcript	0.137884	0.316729	0.407404	0.160292	0.430716	0.42325	0.171773	0.317946	0.187095
Novel_005139	XLOC_05350.	transcript	0.300139	0.693178	0.412201	0.12811	0.387272	0.415774	0.220373	0.354002	0.156879
Novel_005140	XLOC_05350.	transcript	0	0	0	0.273463	0	0	0.226217	0.055158	0.286748
Novel_005141	XLOC_05350.	transcript	0	0	0	0	0	0	0	0	0
Novel_005142	XLOC_05351.	transcript	0.27491	0.056762	0.14417	0.033969	0.198393	0.109994	0.187813	0.14378	0.217226
Novel_005143	XLOC_05351.	transcript	1.068016	4.070937	1.505257	2.417555	1.281554	1.410869	1.778826	1.418688	1.633171
Novel_005144	XLOC_05352.	transcript	0.00827	0.004804	0.015958	0.058348	0.251529	0	0.29269	0.223021	0.013558
Novel_005145	XLOC_05352.	transcript	0.003361	0.151961	0	0	0.223467	0.006259	0.153662	0.165611	0.004517
Novel_005146	XLOC_05352.	transcript	0.52164	0.504887	0.827867	2.23103	1.042898	0.879097	0.495748	0.927794	0.703968
Novel_005147	XLOC_05352.	transcript	0	0.065915	0.394594	0.157475	0.186596	0.733899	0.256959	0.434008	0
Novel_005148	XLOC_05352.	transcript	0	1.216489	0	0	0	0	0	0	0
Novel_005149	XLOC_05352.	transcript	0	0	0.381093	0	0	0.224636	0	0.097991	0
Novel_005150	XLOC_05353.	transcript	0.402897	0.194198	0.174041	0.385059	0.285099	0.073903	0.543461	0.70094	0.011508
Novel_005151	XLOC_05353.	transcript	0.103989	0.113161	0.131549	0.128647	0.334608	0.119087	0.300916	0.034306	0.256101
Novel_005152	XLOC_05353.	transcript	0	0	0	0.245359	0	0	0	0	0
Novel_005153	XLOC_05353.	transcript	0.335859	0.132542	0.670613	0.212097	0.265827	0.539703	0.246557	0.031127	0.307721
Novel_005154	XLOC_05353.	transcript	0	0	0	0	0.984147	0	0	0	0
Novel_005155	XLOC_05353.	transcript	0.07361	2.805456	0.30514	4.943479	1.037046	3.047683	0.197837	1.079199	0.406271
Novel_005156	XLOC_05353.	transcript	0	0	0	0	0	0	0	0	0

Novel_005157	XLOC_05353.	transcript	0.932582	8.019433	3.151516	0.287132	2.699648	0.232168	3.709988	2.946004	4.259514
Novel_005158	XLOC_05353.	transcript	3.459478	0.59059	0.84672	1.773644	0.53746	1.750989	0.581519	0.448356	1.374801
Novel_005159	XLOC_05353.	transcript	0	0.725596	0.64712	0.195995	0	0.615525	0.512723	0	4.40E-05
Novel_005160	XLOC_05355.	transcript	0	0	0	0	0	0	0	0.00956	0
Novel_005161	XLOC_05355.	transcript	0.074928	0.137807	0.123214	0.159425	0.204489	0.135587	0.343132	0.121908	0.10186
Novel_005162	XLOC_05355.	transcript	0	0	0	0	0	0	0	0.111348	0.121456
Novel_005163	XLOC_05355.	transcript	0.026126	0.013832	0	0	0	0.015174	0.106563	0	0
Novel_005164	XLOC_05355.	transcript	0	0.010499	0	0	0	0	0	0	0
Novel_005165	XLOC_05355.	transcript	0	0	0.077765	0.005524	0.327693	0.180885	0	0.030078	0
Novel_005166	XLOC_05355.	transcript	0.176492	0	0.374366	0.001303	0	0.456491	0	0	0
Novel_005167	XLOC_05355.	transcript	0	0	0	0	0	0	0	0	0
Novel_005168	XLOC_05355.	transcript	0	0.218072	0.048257	0.015689	0.388836	0.033436	0.30273	0.575367	0.655799
Novel_005169	XLOC_05355.	transcript	0.087364	0	0	0.2963	0	0	0	0	0
Novel_005170	XLOC_05355.	transcript	0	0	0	0.516294	0	0	0	0	0
Novel_005171	XLOC_05371.	transcript	0.138962	0.007001	0.037716	0.018898	0.097541	0	0.152231	0.032216	0.030244
Novel_005172	XLOC_05371.	transcript	0	0	0	0	0	0.025183	0	0	0
Novel_005173	XLOC_05380.	transcript	0.086255	0.552804	0.103568	0.079946	0.177623	0.205766	0.322048	0.078245	0.421286
Novel_005174	XLOC_05386.	transcript	0.543423	0.195616	0.458631	0.390979	0.456435	0.77862	0.595695	0.527445	0.477458
Novel_005175	XLOC_05386.	transcript	0.378886	0.012368	0.063107	0.118307	0.170233	0.162045	0.21109	0.219434	0.123799
Novel_005176	XLOC_05386.	transcript	0.558287	0.0551	0.659539	0.585597	0.670094	0.527122	1.006674	0.927355	0.557674
Novel_005177	XLOC_05386.	transcript	0	0	0	0	0	0	0	0	0
Novel_005178	XLOC_05388.	transcript	0.506294	0.34173	0.485815	0.283914	0.571607	0.683229	0.773525	0.613849	0.282137
Novel_005179	XLOC_05388.	transcript	0	0	0.216292	0.169993	0.098402	0	0.141357	0.135885	0
Novel_005180	XLOC_05388.	transcript	0.589396	0.339823	0.783829	0.889577	0.505766	1.749415	0.385649	0.614773	0.611618
Novel_005181	XLOC_05390.	transcript	0.044549	0.506243	0.074968	0.071797	0.06649	0.138675	0.02568	0.067989	0.052796
Novel_005182	XLOC_05390.	transcript	0.073263	0.124358	0.028837	0	0.077971	0.019729	0.08362	0.027468	0.083723
Novel_005183	XLOC_05397.	transcript	0.037134	0	0.079374	0.356989	0.038299	0.439597	0.167052	0.286718	0.204338
Novel_005184	XLOC_05397.	transcript	0	0	0	0.439166	0.172969	0.158531	0.288837	0.240656	0.15557
Novel_005185	XLOC_05398.	transcript	0.75479	0.533005	1.075225	0.820599	1.245837	1.147264	1.046551	0.89385	1.129369
Novel_005186	XLOC_05398.	transcript	0	0	0	0	0.466192	0	0	0	0
Novel_005187	XLOC_05412.	transcript	0.384037	0.452457	0.431493	0.465083	0.369859	0.302594	0.462115	0.673334	0.230757
Novel_005188	XLOC_05412.	transcript	0.000948	0.004355	0	0	0	0.001302	0	0	0
Novel_005189	XLOC_05412.	transcript	0.062882	0	0.054212	0.523319	0.284228	0.005571	0.522355	1.049256	0.035812
Novel_005190	XLOC_05412.	transcript	0.76285	0.176714	0.510741	0.206586	0.29477	0.479525	0.391949	0.006144	0.474039
Novel_005191	XLOC_05413.	transcript	0.355139	0.129255	0.230501	0.193842	0.133816	0.279764	0.379033	0.293361	0.247184
Novel_005192	XLOC_05413.	transcript	0	0	0	0	0	0	0.339625	0	0
Novel_005193	XLOC_05413.	transcript	0	0	0.237981	0	0.275177	0	0	0	0
Novel_005194	XLOC_05413.	transcript	0.25089	0	0.045033	0.005335	0.090855	0.033379	0	0.14291	0.037707

Novel_005195	XLOC_05413.	transcript	0	0	0	0	0	0	0	0	0
Novel_005196	XLOC_05413.	transcript	0.003658	0	0	0	0	0	0	0.000609	0
Novel_005197	XLOC_05413.	transcript	0.198551	0.040917	0.020651	0	0.01612	0	0	0.011506	0.919112
Novel_005198	XLOC_05413.	transcript	0.452381	0.583177	0.246577	0.131693	0.207011	1.038562	0.864397	0.205521	0.867961
Novel_005199	XLOC_05420.	transcript	0	0.041842	0	0.120781	0	0	0.201205	0	0
Novel_005200	XLOC_05425.	transcript	0.412184	0.467791	1.161574	1.189343	0.810105	0.366674	0.471652	0.914072	3.095291
Novel_005201	XLOC_05425.	transcript	0.237163	0.192746	0.301166	0.168772	0.291747	0.319566	0.358541	0.245973	0.199404
Novel_005202	XLOC_05425.	transcript	0.286892	0.199078	0	0.105988	0	0	0.005455	0.223696	0
Novel_005203	XLOC_05435.	transcript	2.234684	2.91203	2.082114	1.868394	1.915176	2.43935	2.88276	2.70514	2.798583
Novel_005204	XLOC_05435.	transcript	0.426845	0.215232	0.059417	0.057452	0.040981	0.076099	0.076167	0.051829	0.053602
Novel_005205	XLOC_05435.	transcript	0.654303	0.187094	0.321772	0.19422	0.316414	0.068339	0.271895	0.380541	0.359487
Novel_005206	XLOC_05435.	transcript	0	0	0	0	0	0	0	0.104472	0
Novel_005207	XLOC_05435.	transcript	0	0.21053	0.262186	0.236615	0.189643	0.389075	0.222725	0.139809	0.078067
Novel_005208	XLOC_05440.	transcript	0.335999	0	0.007508	0.258046	0	0	0	0.125893	0
Novel_005209	XLOC_05440.	transcript	0	0.14157	0.665595	0.250471	0.096563	0	0	0.033364	0.260365
Novel_005210	XLOC_05440.	transcript	0.474678	0	0	0.17693	0.435312	0.738227	0.886005	0.921663	0
Novel_005211	XLOC_05444.	transcript	0	0	0	0	0	0	0	0.307353	0
Novel_005212	XLOC_05444.	transcript	0.486886	0	0	0	0	0	0	0.184524	0
Novel_005213	XLOC_05444.	transcript	0.183427	0.709579	0.472676	0.420761	0.422025	0.485308	0.734196	0.190614	0.387935
Novel_005214	XLOC_05444.	transcript	0.018399	0.040965	0.021035	0.044641	0.012618	0.031467	0.058785	0.017729	0.015793
Novel_005215	XLOC_05452.	transcript	0	0	0	0	0	0	0	0	0
Novel_005216	XLOC_05452.	transcript	0.468361	0.500448	0.330177	0.252374	0.411811	0.294734	0.187389	0.348561	0.131439
Novel_005217	XLOC_05452.	transcript	0.333525	0.768745	0.046997	0.331444	0.136529	0.035628	0.41453	0.289314	0.372817
Novel_005218	XLOC_05452.	transcript	0.044519	0.647727	0.22076	0.148263	0.2711	0.117326	0.364869	0.207272	0.213592
Novel_005219	XLOC_05454.	transcript	0	0	0	0	0	0	0	0	0
Novel_005220	XLOC_05462.	transcript	0.266953	0.019115	0.010116	0.156147	0.020015	0.01247	0.079526	0.021589	0.136132
Novel_005221	XLOC_05462.	transcript	0	0.169235	0.269639	0	0.262824	0.323211	0.115756	0.253402	0
Novel_005222	XLOC_05462.	transcript	0	0	0	0	0.003637	0	0.201199	0.085022	0.018265
Novel_005223	XLOC_05468.	transcript	0.582475	0.058059	0.278113	0.392479	0.280116	0.070444	0.575399	0.292203	0.240442
Novel_005224	XLOC_05468.	transcript	0.60618	0.279027	0.233256	0.30763	0.378343	1.050714	0.510652	0.414494	0.680791
Novel_005225	XLOC_05468.	transcript	0	0	0.061259	0.060874	0.179048	0	0	0.123034	0
Novel_005226	XLOC_05468.	transcript	0	0	0.169772	0.149221	0.221963	0	0	0.382207	0
Novel_005227	XLOC_05469.	transcript	0.209675	0.209988	0.145813	0.239772	0.235838	0.159387	0.185033	0.135698	0.155963
Novel_005228	XLOC_05469.	transcript	0	0	0.309221	0	0	0	0	0	0
Novel_005229	XLOC_05474.	transcript	0	0.072042	0	0	6.30E-05	2.90E-05	0	0	0
Novel_005230	XLOC_05474.	transcript	0.808281	0.098531	0.852453	0.692441	0.371063	1.192443	0.687122	1.356818	0.287384
Novel_005231	XLOC_05474.	transcript	0.161787	0	0.23908	0.176136	0.147714	0.286166	0.111375	0.222166	0.172787
Novel_005232	XLOC_05474.	transcript	0	0	0	0	0	0.292608	0	0	0

Novel_005233	XLOC_05478.	transcript	0.032223	0.213918	0.11878	0.157508	0.17028	0.077757	0.136323	0.158153	0.082742
Novel_005234	XLOC_05479.	transcript	0.386693	0.549976	0.428922	0.260291	0.296678	0.314981	0.585605	0.33097	0.316211
Novel_005235	XLOC_05479.	transcript	0.066665	0	0.074259	0.624874	0.013268	0.745809	0.061247	0.378285	0.03296
Novel_005236	XLOC_05480.	transcript	1.548445	0.150373	2.433881	2.352508	2.872409	1.439687	1.82519	1.533903	1.707392
Novel_005237	XLOC_05480.	transcript	0	0	0.060511	0	0	0	0	0	0
Novel_005238	XLOC_05480.	transcript	0	0	0	0	0	0	0	0	0
Novel_005239	XLOC_05483.	transcript	0.277483	0	0.042695	0	0.11464	0.064994	0.09689	0.074118	0.033237
Novel_005240	XLOC_05491.	transcript	0	0	0	0	0	0	0	0	0
Novel_005241	XLOC_05491.	transcript	0.619231	0.149078	2.172177	0.145078	1.944438	0.611653	0.994098	3.086244	0.270818
Novel_005242	XLOC_05491.	transcript	0	0	0	0	0	0	0	0	0
Novel_005243	XLOC_05491.	transcript	1.37157	0	0.412181	3.593283	0.550323	2.338457	1.293889	0.601373	1.59281
Novel_005244	XLOC_05496.	transcript	1.793796	3.732344	2.562254	2.454038	2.718134	3.152633	2.523465	3.480681	2.251106
Novel_005245	XLOC_05496.	transcript	0	0	0	0	0	0	0	0	0
Novel_005246	XLOC_05496.	transcript	0	0	0	0	0	0	0	0	0
Novel_005247	XLOC_05496.	transcript	0.239771	0.080365	0.87398	0.163198	0.222352	0.571943	0.601671	0.073201	0.262782
Novel_005248	XLOC_05496.	transcript	0.242952	0	0.155009	0.20689	0.125982	0.194128	0.010948	0.31881	0.338691
Novel_005249	XLOC_05496.	transcript	0.137927	0	0.090202	0.041379	0.146414	0.152519	0.137206	0.093676	0.158561
Novel_005250	XLOC_05496.	transcript	0.427185	0	0.184779	0.25306	0	0.49779	0.126547	0.240043	0.378993
Novel_005251	XLOC_05496.	transcript	0	0	0.203751	0	0	0	0	0	0
Novel_005252	XLOC_05499.	transcript	0.063968	0	0.03744	0.012204	0.032883	0.272236	0.108564	0	0.045678
Novel_005253	XLOC_05499.	transcript	0.193354	0.154714	0.050889	0.096593	0.195281	0	0.182911	0.381258	0.092307
Novel_005254	XLOC_05508.	transcript	0.047822	0	0.088477	0.066058	0.201513	0.155656	0	0.082141	0.170025
Novel_005255	XLOC_05518.	transcript	0	0.530951	0	0.097145	0	0	0.164864	0.120195	0
Novel_005256	XLOC_05518.	transcript	0.081223	0.609677	0.081763	0.033318	0.095574	0.101476	0.151649	0.039168	0.113484
Novel_005257	XLOC_05518.	transcript	0	0.168465	0	0	0	0	0	0	0
Novel_005258	XLOC_05519.	transcript	0	3.00E-06	0	0	0.015474	0.022227	0.016799	0.008031	0.127366
Novel_005259	XLOC_05519.	transcript	0.174803	0.454777	0.170264	0.37797	0.270402	0.269544	0.306081	0.317577	0.124325
Novel_005260	XLOC_05521.	transcript	0	0.210881	0	0	9.40E-05	0	0.030951	0	0
Novel_005261	XLOC_05526.	transcript	0.044146	0.241438	0.046672	0	0	0	0.0186	0.025276	0.114161
Novel_005262	XLOC_05527.	transcript	3.705772	0	39.85876	31.64865	27.56434	20.18035	21.34573	18.70974	42.84166
Novel_005263	XLOC_05527.	transcript	27.21223	15.0237	19.09249	8.042902	8.831289	27.55985	15.83905	11.58087	15.50721
Novel_005264	XLOC_05527.	transcript	0.059445	0	0.065659	0	0	0.113913	0.219573	0	0.171684
Novel_005265	XLOC_05527.	transcript	0.353772	0.763336	0.490656	0.526648	0.545416	0.581074	0.343253	0.694625	0.261466
Novel_005266	XLOC_05527.	transcript	0.293405	1.02771	0.599323	0.51567	0.435366	0.60899	0.491239	0.483397	0.72733
Novel_005267	XLOC_05530.	transcript	0	0	0	0.601254	0	0	0	0	0.008142
Novel_005268	XLOC_05530.	transcript	0	0	0.777148	0.164298	0.667871	0.718089	0.001792	0.945457	0.648294
Novel_005269	XLOC_05530.	transcript	0.800981	1.322618	0	0	0	0.056593	1.027942	0.047002	0.009729
Novel_005270	XLOC_05530.	transcript	0	0.051503	0.262789	0	0	0.074976	0.139426	0.142317	0.073646

Novel_005271	XLOC_05534.	transcript	0	0.134174	0.304063	0.008588	0	0.289946	0.003279	0.058377	0.088495
Novel_005272	XLOC_05534.	transcript	0.012131	0	0.016275	0	0	0	0	0.090294	0.268502
Novel_005273	XLOC_05534.	transcript	0	0	0	0	0	0	0.389359	0	0
Novel_005274	XLOC_05534.	transcript	0.048627	0	0.002697	0	0.506711	0	0	0.094782	0
Novel_005275	XLOC_05534.	transcript	0.35221	0	0	0	0	0	0	0	0
Novel_005276	XLOC_05534.	transcript	0	0.058948	0.251157	0.040128	0.067312	0	0	0	0
Novel_005277	XLOC_05534.	transcript	0.001328	0	0.001301	0	0	0.325747	0.181915	0.391677	0.266736
Novel_005278	XLOC_05534.	transcript	0.005933	0	0.003746	0.435916	0	0.016383	0	0	0.01201
Novel_005279	XLOC_05538.	transcript	0.247234	1.561456	0.372806	0.224395	0.263143	0.177508	0.420407	0.444767	0.097586
Novel_005280	XLOC_05547.	transcript	0.248489	0.655382	0.381578	0.486235	0.238669	0.364996	0.35289	0.323107	0.422981
Novel_005281	XLOC_05547.	transcript	0.317112	0	0.3421	0.651411	0.35376	0	0.546074	0	0
Novel_005282	XLOC_05547.	transcript	0	0.003855	0	0	0	0	0	0.008074	0.038939
Novel_005283	XLOC_05547.	transcript	0.009074	0.368326	0.039944	0.010435	0.012387	0.274513	0.02299	0.315108	0.617284
Novel_005284	XLOC_05547.	transcript	0	0	0.001601	0.00641	0	0	0.02335	0.006631	0.012459
Novel_005285	XLOC_05547.	transcript	0.335152	0.347167	0.29826	0.218002	0.22849	0.216931	0.301112	0.254417	0.122655
Novel_005286	XLOC_05548.	transcript	0.020535	0.383857	0	0	0.013356	0	0	0	0
Novel_005287	XLOC_05548.	transcript	0.038359	0.371161	0.144903	0.145949	0.143096	0.173403	0.046857	0.062852	0.08208
Novel_005288	XLOC_05549.	transcript	0.264239	0.497815	0.250166	0.296563	0.304458	0.431685	0.34355	0.227094	0.230336
Novel_005289	XLOC_05554.	transcript	0.222219	0.122693	0.352958	0.237611	0.286784	0.270928	0.477439	0.366886	0.236051
Novel_005290	XLOC_05554.	transcript	0	0	0	0	0	0	0	0	0
Novel_005291	XLOC_05556.	transcript	0.218031	0.37592	0.186173	0.225853	0.299383	0.27948	0.504137	0.427714	0.389241
Novel_005292	XLOC_05556.	transcript	0.072644	0	0.059478	0.008669	0.03485	0.080406	0.029653	0.080224	0.39405
Novel_005293	XLOC_05558.	transcript	0.959025	1.349708	0.924796	1.39298	1.003552	0.36638	1.516608	1.273159	1.457426
Novel_005294	XLOC_05567.	transcript	0.045029	0.661927	0.190879	0.090094	0.115132	0.216159	0.31471	0.197357	0.204427
Novel_005295	XLOC_05567.	transcript	0.059374	0.194837	0.037151	0.025361	0.087534	0.023578	0.043428	0.031618	0.03317
Novel_005296	XLOC_05567.	transcript	0.258324	0.001749	0.051029	0.092812	0.100911	0	0.065941	0.149233	0.03949
Novel_005297	XLOC_05567.	transcript	0	0.13839	0.001286	0.008154	0.002789	0.037535	0.005641	0.022442	0.007248
Novel_005298	XLOC_05568.	transcript	0.201344	0.695331	0.20038	0.224833	0.149624	0.262859	0.266982	0.262917	0.207786
Novel_005299	XLOC_05569.	transcript	0.444047	0.086694	1.176898	0.263234	0.010134	0.000185	0.213975	1.085303	1.422841
Novel_005300	XLOC_05569.	transcript	0	0.033645	0	0.399477	0.543614	1.86432	0.311249	0	0
Novel_005301	XLOC_05569.	transcript	0.355259	0.0521	1.169492	0.74675	0.763777	1.937829	0.681158	0.846174	1.108029
Novel_005302	XLOC_05569.	transcript	0.799428	0.019494	2.535918	1.760858	2.036536	5.041463	2.071972	2.255315	3.296866
Novel_005303	XLOC_05573.	transcript	0.18979	0.390576	0	0.029196	0.021527	0.664128	0.168101	0.017389	0.022117
Novel_005304	XLOC_05573.	transcript	0.1894	0.54277	0.340939	0.446834	0.463488	0	0.376722	0.313052	0.345494
Novel_005305	XLOC_05586.	transcript	0.748631	1.01048	0.87883	0.508511	0.490103	0.042026	0.41373	0.952711	0.631849
Novel_005306	XLOC_05586.	transcript	0	0	0	0	0.381077	0.076152	0	0	0.085374
Novel_005307	XLOC_05586.	transcript	0	0.01236	0	0	0.235394	0.022273	0.167276	0.36437	0.062779
Novel_005308	XLOC_05586.	transcript	0.372484	0.310075	0.242407	0.297171	0.298316	0.220983	0.398569	0.064049	0.253737

Novel_005309	XLOC_0559€.	transcript	0	0	0.133357	0.494467	0	0	0.173939	0	0
Novel_005310	XLOC_0559€.	transcript	0	0	0	0.096442	0	0	0.124291	0	0
Novel_005311	XLOC_0559€.	transcript	0	0	0.282809	0.225351	0	0	0.008174	0	0
Novel_005312	XLOC_0559ε.	transcript	0.215904	0.035753	0.136426	0.796943	0.219453	0.240306	0.261147	0.218952	0.106824
Novel_005313	XLOC_0559ε.	transcript	0	0	0	0	0	0	0	0	0
Novel_005314	XLOC_0559ε.	transcript	0.079115	0	0	0	0.052645	0	0	0	0.025417
Novel_005315	XLOC_05601.	transcript	12.40793	7.175021	9.582419	13.34563	11.41257	7.46385	15.21145	15.00454	12.89412
Novel_005316	XLOC_05601.	transcript	0.713052	8.023665	0.44119	1.636118	0.320948	0	0.491711	0.337295	0.395402
Novel_005317	XLOC_05602.	transcript	0.456984	0.505491	0.288583	0.246669	0.331431	0.361515	0.263617	0.241268	0.537827
Novel_005318	XLOC_05607.	transcript	0.269701	0.184811	0.395545	0.240166	0.612378	0.44553	0.386059	0.259162	0.212203
Novel_005319	XLOC_0560ε.	transcript	0.525017	0.687641	0.585312	0.774546	0.326738	0.701841	0.68773	0.722258	0.70023
Novel_005320	XLOC_0560ε.	transcript	0	0	0	0	0.36296	0	0	0	0
Novel_005321	XLOC_0560ς.	transcript	0.00226	0.002187	0	0.043012	0.005369	0	0.018204	0.009316	0.03027
Novel_005322	XLOC_0560ς.	transcript	0	0	0	0	0	0	0	0	0
Novel_005323	XLOC_0560ς.	transcript	0	0	0	0	0	0	0.059151	0	0.029102
Novel_005324	XLOC_0560ς.	transcript	0.205672	0	0.119347	0	0	0	0.140981	0	0.003138
Novel_005325	XLOC_0560ς.	transcript	0	0	0.027176	0	0	0.021636	0	0	0
Novel_005326	XLOC_0560ς.	transcript	0	0.01583	0	0	0.042043	0	0	0	0
Novel_005327	XLOC_0560ς.	transcript	0	0.020593	0	0	0	0.244546	0.027984	0.049989	0
Novel_005328	XLOC_0560ς.	transcript	0.275318	0.413178	0.127205	0.163068	0.113111	0.119126	0.515919	0.204477	0.355649
Novel_005329	XLOC_0560ς.	transcript	0.436025	0.294726	0.100095	0.19915	0	0.029395	0.019789	0.052575	0.359309
Novel_005330	XLOC_0560ς.	transcript	0	0.006653	0.00826	0	0.13748	0.006919	0.120542	0.00517	0.296216
Novel_005331	XLOC_0560ς.	transcript	0.009249	0	0.078431	0.001277	0.178863	0	0.02885	0.047153	0.103182
Novel_005332	XLOC_0560ς.	transcript	0.482328	0.538194	0.357373	0.355901	0.269574	0.368492	0.601224	0.607361	1.265779
Novel_005333	XLOC_0561C.	transcript	0.327422	0.527921	0.256003	0.461527	0.39841	0.284866	0.369048	0.72206	0.303321
Novel_005334	XLOC_0561C.	transcript	0.468049	0.580331	0.729723	0.924434	0.657654	0.806878	0.634723	0.650592	0.380672
Novel_005335	XLOC_0561C.	transcript	0.001715	0.254841	0.002836	0.093865	0	0.003217	0.001951	0.001624	0.028304
Novel_005336	XLOC_0561C.	transcript	0	0	0	0	0.184402	0	0	0	0
Novel_005337	XLOC_0561C.	transcript	0.691497	0	0.883665	3.239113	0.953212	0.977097	1.327913	1.656659	1.219893
Novel_005338	XLOC_0561З.	transcript	0.671741	0.250739	0.396633	0.253063	0.257316	0.225575	0.427997	0.29916	0.283634
Novel_005339	XLOC_0561ε.	transcript	0	0	0	0	0	0	0	0.000757	0
Novel_005340	XLOC_0561ε.	transcript	0	0	0.141539	0.176928	0	0	0	0	0.009178
Novel_005341	XLOC_0561ε.	transcript	0	0	0	0	0.010967	0	0	0	0
Novel_005342	XLOC_0561ε.	transcript	0.185692	0.211656	0.203714	0.190153	0.315639	0.277242	0.005403	0.089167	0.185595
Novel_005343	XLOC_0561ε.	transcript	0	0.241752	0	0	0	0	0.300547	0.200137	0.018635
Novel_005344	XLOC_0562€.	transcript	0.673097	0.250293	1.943448	0.309639	0.154932	0.105562	0.140955	0.765265	1.864257
Novel_005345	XLOC_0562€.	transcript	2.587706	1.511413	1.686675	2.40593	2.289926	2.545029	2.930394	2.579361	1.40484
Novel_005346	XLOC_0563C.	transcript	0	0.307905	0.032878	0.105893	0	0.18551	0.233084	0	0.116172

Novel_005347	XLOC_05630.	transcript	0.085495	0.403071	0.176241	0.079404	0.033642	0.091809	0.213519	0.163474	0.192792
Novel_005348	XLOC_05630.	transcript	0.219757	0	0.193006	0.100031	0.342734	0.142789	0.30834	0.301724	0.114287
Novel_005349	XLOC_05630.	transcript	0.025654	0.015463	0.051237	6.90E-05	0.006858	0.257908	0.016584	0.279431	0.019885
Novel_005350	XLOC_05630.	transcript	0.336362	0.285808	0.376927	0.313133	0.574403	0.269507	0.54809	0.266585	0.031767
Novel_005351	XLOC_05630.	transcript	0	0	0	0	0	0	0	0	0
Novel_005352	XLOC_05631.	transcript	0.700229	0.719698	0.307215	0.439253	0.208137	0.340713	0.572627	0.000993	0.1792
Novel_005353	XLOC_05631.	transcript	0.001262	0	0	0	0.003373	0.000967	0	0.375038	0.000641
Novel_005354	XLOC_05631.	transcript	0	0	0	0.0706	0.119053	0	0.274933	0.42949	0
Novel_005355	XLOC_05631.	transcript	0.157396	0.013731	0.25716	0.330775	0.397273	0.250546	0.489742	0.030442	0.262142
Novel_005356	XLOC_05631.	transcript	0.19546	0.030966	0.1176	0.071288	0.112508	0.095096	0.270687	0.347113	0.056416
Novel_005357	XLOC_05631.	transcript	0.047438	0	0.020088	0.031818	0.124285	0.021924	0.163088	0.041836	0.033609
Novel_005358	XLOC_05631.	transcript	0.256687	0.0139	0.33949	0.715769	0.511176	0.36818	0.794696	0.33295	0.316065
Novel_005359	XLOC_05631.	transcript	0	0	0	0	0	0	0	0	0
Novel_005360	XLOC_05631.	transcript	0	0	0	0	0	0	0	0.001751	0
Novel_005361	XLOC_05633.	transcript	0.144782	0.171554	0	0.116039	0.209738	0.052149	0.411523	0.105085	0.032628
Novel_005362	XLOC_05633.	transcript	0.041617	0.196822	0	0.049999	0.13931	0.295936	0.286583	0.018959	0.573894
Novel_005363	XLOC_05633.	transcript	0	0.142365	0	0.012452	0.083772	0	0.14539	0.016091	0
Novel_005364	XLOC_05633.	transcript	0	0.241799	0.466685	0.046101	0.136416	0	0.135542	0	0.046832
Novel_005365	XLOC_05633.	transcript	0	0.002309	0	0	0.13443	0	0.00591	0.000974	0
Novel_005366	XLOC_05633.	transcript	0.231008	0.196853	0.076488	0.668028	0.099663	0.00306	0.199601	0.171243	0.011721
Novel_005367	XLOC_05633.	transcript	0.266529	0	0.187013	0	0	0.253572	0	0.730486	0.07569
Novel_005368	XLOC_05634.	transcript	1.215401	1.848968	1.795902	1.388586	1.667582	1.622321	1.503069	2.301373	2.124576
Novel_005369	XLOC_05634.	transcript	0.020895	0.525212	0.048019	0.018939	0	0.066595	0	0	0.001544
Novel_005370	XLOC_05634.	transcript	0.05327	0.822212	0	0.012876	0.023715	0	0.12436	0	0
Novel_005371	XLOC_05634.	transcript	0.02714	0.322721	0.014027	0.021676	0	0	0.0829	0.0177	0
Novel_005372	XLOC_05634.	transcript	0.31807	0.030432	0.006195	0	0.027306	0.067037	0.008203	0.127966	0.00222
Novel_005373	XLOC_05634.	transcript	0	1.304549	0.052187	0.075069	0.038651	0	0.193768	0	0.081163
Novel_005374	XLOC_05635.	transcript	0.015188	0.488614	0.080608	0.006365	0.036384	0.200356	0.090293	0	0.07438
Novel_005375	XLOC_05635.	transcript	0	0	0	0.171488	0	0	0	0	0
Novel_005376	XLOC_05635.	transcript	0	0.436579	0	0	0.015515	0	0.001138	0.018439	0.051346
Novel_005377	XLOC_05635.	transcript	0	0	0	0	0	0	0.091783	0.07942	0
Novel_005378	XLOC_05641.	transcript	0.64321	0.459507	1.159203	1.367643	1.001199	1.841423	1.470428	1.147854	1.240129
Novel_005379	XLOC_05648.	transcript	0.046994	0.458524	0.148973	0.148072	0.241064	0.200427	0.355305	0.124156	0.429986
Novel_005380	XLOC_05648.	transcript	0	0	0	0	0	0	0	0.133706	0
Novel_005381	XLOC_05648.	transcript	0.078328	0.026822	0.135975	0.079838	0.13839	0.054463	0.085962	0.015035	0.133362
Novel_005382	XLOC_05649.	transcript	8.827985	0.027654	3.215764	2.374735	4.478001	2.127727	4.143928	3.699961	1.295784
Novel_005383	XLOC_05650.	transcript	0.106011	0.108805	0.172463	0.186154	0.208272	0.231976	0.190789	0.136075	0.255774
Novel_005384	XLOC_05651.	transcript	0.287421	0.193995	0.15244	0	0.098317	0.11598	0.119396	0.055037	0

Novel_005385	XLOC_05651.	transcript	0	0.156843	0.026157	0.326163	0.044189	0.09984	0.045606	0.027613	0
Novel_005386	XLOC_05652.	transcript	0	0	0.142913	0.448948	0	0	0	0.122699	0.183652
Novel_005387	XLOC_05653.	transcript	0.235204	0.00042	0.152008	0.273351	0	0.000914	0.306407	0.291387	0
Novel_005388	XLOC_05653.	transcript	0.245229	0.001934	0.415287	0.29242	0.095414	0.007893	0.322623	0.364054	0.386798
Novel_005389	XLOC_05653.	transcript	0.55795	0.048444	0.137481	0.332173	0.045786	0.138698	0.403862	0.25257	0.535176
Novel_005390	XLOC_05653.	transcript	0.007686	0	0	0.006787	0.002595	0	0.9263	0	0
Novel_005391	XLOC_05653.	transcript	0.254765	0.092361	0.144689	0.364927	0.423335	0.967001	0.293648	0.349184	0
Novel_005392	XLOC_05653.	transcript	0.015079	0	0.028662	0.03373	0.001367	0	0	0.003104	0.040562
Novel_005393	XLOC_05653.	transcript	0	0.017272	0	0	0.015956	0	0	0.300851	0
Novel_005394	XLOC_05653.	transcript	1.657016	0.157551	1.229943	0.786732	0.836609	1.25577	1.45006	1.572345	0.610037
Novel_005395	XLOC_05653.	transcript	0.186317	0	0.076722	0.186409	0.352819	0.011931	0.359646	0.388948	0.106828
Novel_005396	XLOC_05653.	transcript	0	0	0	0	0.130092	0.212011	0	0.029817	0
Novel_005397	XLOC_05653.	transcript	0	0	0	0	0	0.362173	0	0	0
Novel_005398	XLOC_05654.	transcript	0.427735	0.609143	0.5506	0.672937	0.618543	0.619166	0.908728	0.866196	0.737033
Novel_005399	XLOC_05656.	transcript	0	0	0	0	0	0	0	0.190067	0
Novel_005400	XLOC_05657.	transcript	3.318196	6.126787	3.550034	5.099439	4.298997	4.407441	4.697415	5.133825	4.682339
Novel_005401	XLOC_05657.	transcript	0	0	0.025178	0	0	0.249561	0.054055	0	0.021085
Novel_005402	XLOC_05661.	transcript	0.470618	0.366744	0.341544	0.293028	0.326316	0.274167	0.538553	0.409698	0.273243
Novel_005403	XLOC_05662.	transcript	0.144658	0	0	0.189135	0	0	0.212868	0.455098	0
Novel_005404	XLOC_05662.	transcript	0	0.0488	0	0	0.017007	0	0	0	0.008961
Novel_005405	XLOC_05662.	transcript	0	0	0.670743	0.586706	0	0	0.493486	0	0
Novel_005406	XLOC_05662.	transcript	0	0	0	0	0	0	0	0	0.002978
Novel_005407	XLOC_05662.	transcript	0.020925	0.02179	0.087757	0.101293	0.075939	0.017732	0.099088	0.707959	0.084468
Novel_005408	XLOC_05662.	transcript	0	0	0.01923	0.326315	0.046582	0.00985	0.023494	0.216409	0
Novel_005409	XLOC_05662.	transcript	0.967659	1.547291	0.667228	0.122746	0.893716	1.081598	0.610729	0.553386	0.280782
Novel_005410	XLOC_05662.	transcript	0	0	0	0	0	0	0	0	0
Novel_005411	XLOC_05662.	transcript	0.156875	0.107695	0.421598	0.0317	0.252007	0.063777	0.251116	0.176899	0.059387
Novel_005412	XLOC_05671.	transcript	0.666717	0.880338	0.599274	0.555411	0.680975	0.545051	1.011367	0.81771	0.423152
Novel_005413	XLOC_05675.	transcript	0	0	0	0.332742	0	0.029317	0	0	0
Novel_005414	XLOC_05675.	transcript	0.048053	0	0.025443	0.032461	0.067089	0	0.013499	0.016501	0.035356
Novel_005415	XLOC_05682.	transcript	0.53226	0.563995	0.085125	0.196907	0.102256	0.905338	0.296243	0.190046	0.651462
Novel_005416	XLOC_05682.	transcript	0.174183	0	0.863285	0.859974	1.109919	0	0.819507	0.761311	0.475918
Novel_005417	XLOC_05682.	transcript	0.010462	4.223805	3.858904	1.07301	4.873519	6.809469	0	3.914851	1.474615
Novel_005418	XLOC_05682.	transcript	1.701404	0.847426	2.521101	0.349375	0.398071	0.12995	1.593116	1.352439	0.081279
Novel_005419	XLOC_05682.	transcript	1.86857	1.123658	0.04904	0.975444	0.599429	0	0	0.890724	0.209884
Novel_005420	XLOC_05682.	transcript	0	0	0	0	0	0	0	0	0
Novel_005421	XLOC_05682.	transcript	0.671253	1.267995	0.479741	0.098128	0.984951	0.819532	0.001512	0.583302	0.908777
Novel_005422	XLOC_05685.	transcript	0	0	0.217952	0.062944	0	0	0	0.041853	0

Novel_005423	XLOC_05685.	transcript	0	0	0.409326	0	0.040355	0	0.26167	0	0
Novel_005424	XLOC_05685.	transcript	0	0	0	0	0	0	0.198863	0	0
Novel_005425	XLOC_05685.	transcript	0	0	0	0.146349	0	0	0	0.077462	0
Novel_005426	XLOC_05685.	transcript	0.360527	0.305802	0.013084	0.129683	0.396843	0.404247	0.006502	0.436819	0.333599
Novel_005427	XLOC_05688.	transcript	0.120757	0.492171	0.209986	0.361118	0.314437	0.188331	0.141741	0.170818	0.213014
Novel_005428	XLOC_05688.	transcript	0.072697	0	0	0.015851	0.163394	0.230479	0.235627	0.193636	0
Novel_005429	XLOC_05688.	transcript	0.020446	0	0	0	0.068409	0	0.142238	0.132053	0
Novel_005430	XLOC_05688.	transcript	0.465494	0	0	0	0	0	0.485134	0.483621	0
Novel_005431	XLOC_05689.	transcript	1.0933	1.185339	1.962817	3.317773	1.937643	3.080823	1.303248	1.466459	2.530543
Novel_005432	XLOC_05689.	transcript	0.306893	0.996936	1.574791	1.920779	2.001174	1.338812	1.304386	1.173642	2.06662
Novel_005433	XLOC_05694.	transcript	0.260277	0.151755	0.327372	0.255895	0.29038	0.362031	0.442633	0.392044	0.381282
Novel_005434	XLOC_05694.	transcript	0	0	0.044916	0.167946	0	0.242456	0.094898	0	0.05035
Novel_005435	XLOC_05705.	transcript	0.630278	1.382924	0.823617	0.583892	1.269676	1.558941	1.148425	1.148348	0.356013
Novel_005436	XLOC_05705.	transcript	0	0.067005	0.382594	0.744689	0	0	0.491331	0.566909	0.345841
Novel_005437	XLOC_05705.	transcript	0	0.817961	0	0	0	0	0	0	0
Novel_005438	XLOC_05705.	transcript	0.962859	3.01604	1.658491	1.255476	1.458423	1.485427	1.359921	2.004742	0.751725
Novel_005439	XLOC_05705.	transcript	0.474773	1.012043	0.751014	1.069796	0.78517	0.760589	0.882466	1.063256	0.527926
Novel_005440	XLOC_05705.	transcript	0.079055	0	0.017229	0.029818	0.083664	0	0.011767	0.145669	0.015444
Novel_005441	XLOC_05705.	transcript	0.179613	0	0	0	0.279352	0.267309	0	0	0
Novel_005442	XLOC_05705.	transcript	0	0.30543	0.206624	0.104759	0.023555	0.012354	0.43237	0.262549	0.258167
Novel_005443	XLOC_05706.	transcript	0.572244	3.259547	1.081193	0.62673	0.992378	0.767203	0.95766	0.656292	0.816576
Novel_005444	XLOC_05706.	transcript	0.065785	0.152657	0.13876	0.054316	0.108643	0.013039	0.109035	0.076851	0.082272
Novel_005445	XLOC_05707.	transcript	0.838855	1.951897	2.661144	2.146677	2.184668	2.570813	1.86341	1.812167	2.271546
Novel_005446	XLOC_05707.	transcript	0.32701	0	0	0.214856	0	0	0.644916	0.143241	0.480976
Novel_005447	XLOC_05707.	transcript	0.214683	0.551188	0.062479	0.008251	0.170168	0.206423	0.002145	0.131663	0.193594
Novel_005448	XLOC_05707.	transcript	0	0	0.208874	0.095468	0	0	0	0	0
Novel_005449	XLOC_05707.	transcript	0	0	0.244051	0.165434	0	0	0.314784	0	0
Novel_005450	XLOC_05707.	transcript	0.085402	0	0.154446	0	0	0	0.045872	0	0.021489
Novel_005451	XLOC_05708.	transcript	0.143792	0.352395	0.215248	0.390223	0.431972	0.286124	0.317856	0.228708	0.339329
Novel_005452	XLOC_05708.	transcript	0.183452	0.175599	0.139217	0.203325	0.027151	0.063457	0.031832	0.357191	0.312657
Novel_005453	XLOC_05708.	transcript	0	0	0	0.227695	0.205427	0	0.308175	0	0
Novel_005454	XLOC_05709.	transcript	0.512846	0.906282	0.610862	0.567091	0.694925	0.330844	0.500725	0.693033	0.288585
Novel_005455	XLOC_05709.	transcript	0	0	0.282259	0	0	0	0	0	0
Novel_005456	XLOC_05709.	transcript	0	0	0	0	0	0	0	0	0
Novel_005457	XLOC_05709.	transcript	0.078686	0	0	0.212946	0.009289	0.610364	0.624511	0.098203	0.495206
Novel_005458	XLOC_05709.	transcript	0.372916	0.302887	0.77842	0.350358	0.859026	0.501529	0.983693	0.531867	0.37685
Novel_005459	XLOC_05709.	transcript	0.339407	0.012688	0.030301	0.049115	0.046647	0.00992	0.049192	0.572845	0.045376
Novel_005460	XLOC_05709.	transcript	0.019671	0	0.208606	0.277125	0.076148	0	0	0.066438	0.271618

Novel_005461	XLOC_05710.	transcript	0.160307	0.210374	0.263508	0.154321	0.255439	0.226199	0.396147	0.178779	0.218321
Novel_005462	XLOC_05711.	transcript	0	0	0.132805	0	0	0	0	0	0
Novel_005463	XLOC_05711.	transcript	0.161075	0	0.374179	0.290903	0.70123	0.389215	0.087321	0.201372	0.33859
Novel_005464	XLOC_05711.	transcript	0.340338	0	0	0	0	0	0	0	0
Novel_005465	XLOC_05712.	transcript	0.379887	0.526421	0.530876	1.066594	0.984162	0.960149	1.148299	0.915217	0.952545
Novel_005466	XLOC_05715.	transcript	0.02728	0	0.019485	0.236103	0.029934	0.011212	0	0	0.030727
Novel_005467	XLOC_05715.	transcript	0.221334	0.442181	0.285565	0.409562	0.18106	0.448275	0.354127	0.326338	0.413811
Novel_005468	XLOC_05716.	transcript	0	0	0	0.044701	0.068188	0.128273	0.139546	0.030486	0
Novel_005469	XLOC_05718.	transcript	0.107059	0.097744	0.152909	0	0	0.04758	0	0.05555	0.118391
Novel_005470	XLOC_05718.	transcript	0.265012	0.31224	0.066514	0.139546	0.172879	0.209928	0.390757	0.184117	0.431613
Novel_005471	XLOC_05719.	transcript	0	0	0.039684	0.078521	0.042557	0.126751	0	0.039376	0
Novel_005472	XLOC_05719.	transcript	0	0	0	0	0.001209	0	0	0	0
Novel_005473	XLOC_05719.	transcript	0.151534	0	3.20E-05	0.006563	0.007019	0.00011	0.117691	0.102571	0.069839
Novel_005474	XLOC_05720.	transcript	0.03997	0	0	0.138079	0.00406	0.0827	0	0.201628	0.066574
Novel_005475	XLOC_05720.	transcript	0.080043	0.068389	0.420936	0.045502	0.181216	0.214643	0.18266	0	0.02501
Novel_005476	XLOC_05720.	transcript	1.262184	2.557185	1.168706	1.104207	1.162976	0.997158	1.14672	1.122671	0.246923
Novel_005477	XLOC_05721.	transcript	0.583285	0.392366	1.204933	0.727008	1.235961	0.880409	1.018878	0.714264	1.83543
Novel_005478	XLOC_05723.	transcript	0.057778	0.475827	0	0	0	0	0.363706	0	0.166211
Novel_005479	XLOC_05723.	transcript	0	0	0.070625	0.073761	0.161341	0.129033	0	0.127503	0
Novel_005480	XLOC_05724.	transcript	0.150936	0	0.051005	0.118836	0.139835	0.130993	0.49397	0.076065	0.086098
Novel_005481	XLOC_05724.	transcript	0.097693	0.177844	0	0.088655	0	0.062408	0.111052	0.072956	0
Novel_005482	XLOC_05724.	transcript	0.350292	0.093935	0.315316	0.327411	0.473028	0.429606	0.362298	0.301792	0.090161
Novel_005483	XLOC_05724.	transcript	0.002594	0	0	0	0	0	0	0	0.439217
Novel_005484	XLOC_05732.	transcript	0.191984	0.130797	0.090869	0.164365	0.309779	0.245257	0.509514	0.256089	1.765508
Novel_005485	XLOC_05732.	transcript	0	0	0	0	0	0	0.522621	0	0.435443
Novel_005486	XLOC_05732.	transcript	0	0.009901	0	0.02303	0.018079	0.186561	0.021095	0.024679	0.157449
Novel_005487	XLOC_05732.	transcript	0.12081	0.11977	0.021253	0.119184	0.096499	0.029024	0.241628	0.120867	0.286655
Novel_005488	XLOC_05732.	transcript	0.149718	0.099685	0.109928	0.071067	0.051813	0.108147	0.283224	0.062286	0.175124
Novel_005489	XLOC_05732.	transcript	0.18061	0.132535	0.053423	0.210018	0.159138	0.033882	0.175179	0.252662	0.156814
Novel_005490	XLOC_05732.	transcript	0.214955	0.249893	0.087521	0.321845	0.160198	0.061001	0.055273	0.283603	0.069448
Novel_005491	XLOC_05732.	transcript	0.179144	0	0.214152	0.064116	0.173052	0.140996	0	0.053085	0.002674
Novel_005492	XLOC_05732.	transcript	0	0	0	0.14915	0.188988	0.191544	0.005727	0.070861	0.002709
Novel_005493	XLOC_05732.	transcript	0.358858	0	0.194795	0.093988	0.192249	0.14306	0	0.136235	0.002564
Novel_005494	XLOC_05732.	transcript	0	0.016745	0	0.169466	0	0.023167	0	0.014823	0.193034
Novel_005495	XLOC_05732.	transcript	0.205307	0.101557	0.301037	0.177005	0.135808	0.073769	0.479955	0.079195	0.166687
Novel_005496	XLOC_05732.	transcript	0.442548	0	0	0.28251	0.105924	0.106415	0	0.03035	0.162918
Novel_005497	XLOC_05732.	transcript	0	0	0	0.106208	0.099759	0	0	0.029939	0.164981
Novel_005498	XLOC_05732.	transcript	0	0	0.013366	0.192292	0.149118	0	0	0.214644	0.466041

Novel_005499	XLOC_05732.	transcript	0.022181	0.019525	0	0.116487	0.15657	0	0.472116	0.144995	0.000531
Novel_005500	XLOC_05732.	transcript	0.217164	0.227545	0	0.139857	0.434704	0.209524	0.314591	0.195563	0.059289
Novel_005501	XLOC_05732.	transcript	0.017402	0	0.013669	0.318656	0.061166	0.380424	0.240162	0.272753	0
Novel_005502	XLOC_05732.	transcript	0.406232	0.517668	0.268252	0.370068	0.494834	0.5541	0.857657	0.519836	0
Novel_005503	XLOC_05732.	transcript	0	0	0	0	0	0	0	0	0.078382
Novel_005504	XLOC_05732.	transcript	0.229426	0	0.063491	0	0	0	0	0	0
Novel_005505	XLOC_05732.	transcript	0.125578	0.226482	0.207232	0.103233	0.19438	0.18016	0.220454	0.209075	0.330745
Novel_005506	XLOC_05732.	transcript	0.298959	0.110166	0.011099	0.062611	0.033092	0.148	0.233025	0.02287	0
Novel_005507	XLOC_05735.	transcript	0.322789	0.947563	0.561074	0.463524	0.633045	0.516017	0.863597	0.511319	0.4153
Novel_005508	XLOC_05736.	transcript	0.622479	0.847781	0.510936	0.392885	0.517092	0.591028	0.65394	0.447649	0.382152
Novel_005509	XLOC_05736.	transcript	0.684762	1.035885	0.884956	0.616362	1.109661	0.957139	0.896603	1.205425	0.726111
Novel_005510	XLOC_05738.	transcript	0.167416	0.139397	0.479942	0.522169	0.521363	1.192936	1.23822	0.640721	0.287711
Novel_005511	XLOC_05738.	transcript	0	0.440705	0	0.011466	0.914385	0.000507	0.554849	0.283904	1.526108
Novel_005512	XLOC_05738.	transcript	0.556173	0.191052	0.103938	0.119606	0.104382	0	2.898178	0.607855	0.15748
Novel_005513	XLOC_05738.	transcript	0.01078	0	1.040442	0.156439	0.270618	1.049264	0.067714	0.807514	0.006357
Novel_005514	XLOC_05738.	transcript	0.434035	0.184568	0.561339	0.43506	0.003116	1.401552	0.257771	1.043858	0.31428
Novel_005515	XLOC_05747.	transcript	0	0	0.678034	0	0	0	0	0	0
Novel_005516	XLOC_05747.	transcript	0	0	0	0.139019	0	0.263638	0.07082	0.216354	0
Novel_005517	XLOC_05747.	transcript	0	0	0	0.013549	0.030651	0	0	0.150337	0.004035
Novel_005518	XLOC_05747.	transcript	0	0	0	0	0.071965	0.513185	0.456244	0	0
Novel_005519	XLOC_05747.	transcript	0	0	0.020485	0	0.51391	0	0	0.303811	0.033036
Novel_005520	XLOC_05751.	transcript	0.096379	2.659297	0.067204	0.014903	0.112553	0.147457	0.1573	0.093217	0
Novel_005521	XLOC_05751.	transcript	0	0.013514	0.004748	0.006638	0	0	0	0	0.408958
Novel_005522	XLOC_05759.	transcript	0	0	0.050924	0.085702	0	0.002954	0	0	0
Novel_005523	XLOC_05759.	transcript	0.083852	0.071771	0.257298	0.079438	0.125486	0.235148	0.125472	0.364237	0.051697
Novel_005524	XLOC_05760.	transcript	0.052712	0	0.234946	0	0	0	0	0.055478	0.071484
Novel_005525	XLOC_05760.	transcript	0	0	0	0	0.033993	0	0	0	0.138254
Novel_005526	XLOC_05760.	transcript	0.143656	0.241815	0.239028	0.194867	0.145812	0.282955	0.29347	0.393865	0.208957
Novel_005527	XLOC_05760.	transcript	0.197592	0	0	0	0.116874	0	0	0	0
Novel_005528	XLOC_05763.	transcript	0.07199	0.985678	0.035293	0.014016	0.051297	0.020549	0.066348	0.04137	0.108379
Novel_005529	XLOC_05764.	transcript	0.046912	0.811102	0.295806	0.08572	0.182687	0	0.262233	0.167752	0
Novel_005530	XLOC_05764.	transcript	0	0.026009	0	0	0.010418	0.036512	0.022092	0	0.024143
Novel_005531	XLOC_05764.	transcript	0.062807	0.148543	0.03735	0.095352	0.094401	0	0.039122	0.026765	0.08213
Novel_005532	XLOC_05764.	transcript	0	0.569979	0.047441	0	0	0.153909	0.159173	0.04549	0
Novel_005533	XLOC_05764.	transcript	0	0	0	0	0	0	0	0	0
Novel_005534	XLOC_05765.	transcript	0.787586	1.010185	1.677848	1.199695	1.125213	1.671737	0.992687	1.518182	1.384602
Novel_005535	XLOC_05767.	transcript	0.236138	0.042711	0.229689	0.160906	0.160134	0.253211	0.272902	0.231282	0.141702
Novel_005536	XLOC_05772.	transcript	0	0	0	0.059889	0.014612	0.007941	0	0.005185	0.24521

Novel_005537	XLOC_05772.	transcript	0.076567	0.214513	0.476844	0	0.306416	0.28015	0.144793	0.269191	0
Novel_005538	XLOC_05772.	transcript	0.422306	0.476745	0.249737	0.583938	0.275108	0.728576	0.461953	0.611909	0.555629
Novel_005539	XLOC_05775.	transcript	0	0	0	0	0	0	0	0.029659	0
Novel_005540	XLOC_05785.	transcript	0	0	0	0	0	0.532561	0	0	0
Novel_005541	XLOC_05785.	transcript	0.152601	0	0.160682	0.308082	0.075369	0.157927	0.321834	0.372707	0.098249
Novel_005542	XLOC_05787.	transcript	0.922048	0.061757	0.067425	0	0.282086	0.753032	0.036503	0.096099	0.205644
Novel_005543	XLOC_05787.	transcript	0.204159	0	0.162087	0.287297	0.345442	0	0.149124	0.184415	0.193069
Novel_005544	XLOC_05787.	transcript	0.424739	0.387322	0.659488	0.978215	0.58576	1.234928	0.967764	0.672997	0.37008
Novel_005545	XLOC_05787.	transcript	0.064896	0	0.135569	0.064001	0.194881	0	0.07021	0.087027	0.077986
Novel_005546	XLOC_05787.	transcript	0.517103	0.084733	0.514126	0.064854	0.011212	0.233626	0.413776	0.379666	0.343776
Novel_005547	XLOC_05787.	transcript	0	0.073962	0.011329	0.228258	0.105811	0.005709	0.019071	0	0
Novel_005548	XLOC_05787.	transcript	0.214232	0.022506	0	0.08696	0.212108	0.133486	0.14383	0.079002	0.046993
Novel_005549	XLOC_05787.	transcript	0.37532	0.421621	0.414049	0.839996	0.4861	1.032129	0.694159	0.697234	0.752581
Novel_005550	XLOC_05787.	transcript	0.245884	0.479913	0.397597	0.203168	0.375638	0.412019	0.54077	0.401264	0.419568
Novel_005551	XLOC_05787.	transcript	1.27581	2.09674	1.299836	0.910555	1.347906	0.95719	1.471933	0.862535	0.487158
Novel_005552	XLOC_05788.	transcript	0.054457	0.505735	0.074964	0.208805	0.055878	0.213136	0.209377	0.05451	0.057801
Novel_005553	XLOC_05789.	transcript	0	0	0	0.052802	0.046853	0	0	0	0
Novel_005554	XLOC_05789.	transcript	0.045536	0.022745	0	0	0	0.013758	0.046678	0.031624	0.040542
Novel_005555	XLOC_05792.	transcript	0	0	0.070877	0.332226	0.136734	0	0.663277	0.254476	0
Novel_005556	XLOC_05792.	transcript	0	0	0.010916	0.011284	0.021146	0.001183	0	0	0
Novel_005557	XLOC_05792.	transcript	0.65914	0.990837	0.364225	0.341331	0.305781	0.687222	0.651051	0.52909	0.59756
Novel_005558	XLOC_05793.	transcript	0	0	0	0	0.070613	0	0	0	0.140054
Novel_005559	XLOC_05793.	transcript	0.957936	0.210672	0.478397	0.50696	0.391878	0.241637	0.654256	0.651975	0.160764
Novel_005560	XLOC_05793.	transcript	0	0	0	0	0	0	0	0	0
Novel_005561	XLOC_05794.	transcript	0.133309	0.042142	0.361063	0.318954	0.29204	0.303606	0.303801	0.264849	0.090278
Novel_005562	XLOC_05795.	transcript	0	0.050492	0	0	0.017919	0.037523	0.106544	0	0
Novel_005563	XLOC_05795.	transcript	0	0	0.984986	0	0	0	0	0	0
Novel_005564	XLOC_05795.	transcript	0.664188	0.746492	0.248725	0.955395	0.548362	1.185717	0.731171	0.483938	0.585437
Novel_005565	XLOC_05798.	transcript	0.069926	0.019318	0.123211	0	0	0.266602	0.104875	0.189948	0.205704
Novel_005566	XLOC_05798.	transcript	0	0.130535	0	0.25787	0.148157	0	0.109989	0	0
Novel_005567	XLOC_05798.	transcript	0.021103	0	0	0.491133	0.014489	0	0.023179	0.093428	0
Novel_005568	XLOC_05798.	transcript	0.036453	0.007187	0	0.27234	0.114521	0	0.083317	0.126724	0.023737
Novel_005569	XLOC_05799.	transcript	1.071131	0.919151	1.596045	1.559961	1.133003	1.687759	1.177069	1.376517	1.687197
Novel_005570	XLOC_05800.	transcript	0.334945	0.718447	0.691221	0.982595	1.154456	0.643513	0.441096	0.707336	0.768179
Novel_005571	XLOC_05801.	transcript	0.241917	0.637175	0.465585	0.15931	0.351014	0.482363	0.20266	0.244835	0.224334
Novel_005572	XLOC_05801.	transcript	0.2081	0.244839	0.510093	0.390115	0.239131	0.012664	0.449469	0.291617	0.172804
Novel_005573	XLOC_05801.	transcript	0	0	0	0	0.019976	0.75465	0.021304	0	0
Novel_005574	XLOC_05808.	transcript	0.455613	0.807779	0.361591	0.150258	0.307621	0.279558	0.476182	0.234949	0.227591

Novel_005575	XLOC_05812.	transcript	0.844886	0.094845	0.37817	0.236737	0.638437	0.616718	0.598318	1.136241	0.448105
Novel_005576	XLOC_05813.	transcript	0	0	0	0	0	0.017578	0.179949	0	0.431307
Novel_005577	XLOC_05813.	transcript	0	0	0	0	0	1.335778	0	0	0
Novel_005578	XLOC_05813.	transcript	0.852132	0	0	0	0	0	0	0	0
Novel_005579	XLOC_05813.	transcript	0.063134	0.413982	0.374732	0.332227	0.341017	3.08724	0.178843	1.84518	0.017198
Novel_005580	XLOC_05813.	transcript	0	0	0	0	0.140608	0.126637	0	0.137568	0
Novel_005581	XLOC_05813.	transcript	0.1402	0	0.001955	0.084245	0	0.141004	0.186587	0.025849	0.190279
Novel_005582	XLOC_05813.	transcript	0.074381	0	0.394745	0	0	0.117654	0.085507	0	0
Novel_005583	XLOC_05813.	transcript	0.014943	0	0	0	0.42138	0	0	0.201579	0
Novel_005584	XLOC_05814.	transcript	0	0	0	0	0	0	0	0	0
Novel_005585	XLOC_05814.	transcript	0.071991	1.243048	0.069116	0.024329	0.081785	0.061395	0.123202	0.083199	0.06401
Novel_005586	XLOC_05815.	transcript	0	0	0	0.129921	0	0	0	0.567492	0
Novel_005587	XLOC_05815.	transcript	0.821557	0.173523	0.438422	0.608704	0.699886	0.618277	0.577396	0.643148	0.276272
Novel_005588	XLOC_05815.	transcript	0.170082	0	0.351136	0.270363	0	0.166049	0.72072	0	0.133435
Novel_005589	XLOC_05817.	transcript	0.493895	0.193017	0.26081	0.440275	0.322125	0.480543	0.356841	0.378779	0.22947
Novel_005590	XLOC_05817.	transcript	0	0	0	0	0	0	0	0	0.142318
Novel_005591	XLOC_05817.	transcript	0	0	0	0	0	0	0	0	0
Novel_005592	XLOC_05817.	transcript	0.058754	0	0	0.046031	0.08678	0	0	0.009542	0.10224
Novel_005593	XLOC_05817.	transcript	0.337674	0.130362	0.411382	0.127273	0.125643	0	0.117905	0.300028	0.10356
Novel_005594	XLOC_05819.	transcript	0.693212	0.890584	1.25197	0.865494	1.242589	0.882632	1.186208	0.93785	1.530377
Novel_005595	XLOC_05823.	transcript	0.150553	0.150887	0.138876	0.328469	0.171644	0.131438	0.361402	0.152554	0.204095
Novel_005596	XLOC_05823.	transcript	0	0	0	0	0	0	0	0	0.014022
Novel_005597	XLOC_05823.	transcript	0.232326	0.054315	0.019717	0.258512	0.095411	1.029624	0.3518	0.282422	0.131284
Novel_005598	XLOC_05823.	transcript	0.728503	0.327423	0.945354	0.668088	0.968966	0	0.818711	0.945952	0.82014
Novel_005599	XLOC_05859.	transcript	0.201674	0.471175	0.404737	0.603921	0.305294	0.762476	0.327291	0.483629	0.154161
Novel_005600	XLOC_05859.	transcript	0.257613	0.346607	0.314679	0.856198	0.876927	1.293419	0.628546	0.751971	0.229842
Novel_005601	XLOC_05861.	transcript	0.255553	0.07399	0.253242	0.12284	0.307451	0.112262	0.0799	0.12124	0.094606
Novel_005602	XLOC_05867.	transcript	0	0	0	0.002904	0.283625	0	0.012413	0.002648	0.182434
Novel_005603	XLOC_05867.	transcript	0.455904	0.458904	0.169278	0.260274	0.011343	0.36108	0.195191	0.326897	0.247895
Novel_005604	XLOC_05867.	transcript	0.956549	0.369631	0.748558	0.726745	0.90667	0.698954	1.14223	0.868204	0.817637
Novel_005605	XLOC_05867.	transcript	0	0.404984	0	0	0.004235	0	0.139081	0	0.38643
Novel_005606	XLOC_05868.	transcript	0.229563	0.329785	0.305659	0.218833	0.339143	0.284082	0.38735	0.324851	0.265115
Novel_005607	XLOC_05872.	transcript	0	0	0	0	0	0	0.294466	0	0
Novel_005608	XLOC_05872.	transcript	0	0	0.013726	0	0	0.038587	0.009752	0	0.002338
Novel_005609	XLOC_05872.	transcript	0.047395	0	0.020948	0.041046	0	0	0	0.026683	0.055674
Novel_005610	XLOC_05872.	transcript	0.213715	0	0.006128	0.015615	0	0	0	0.234498	0
Novel_005611	XLOC_05872.	transcript	0	0	0.299578	0.183491	0.00736	0.026368	0.003938	0.220615	0
Novel_005612	XLOC_05884.	transcript	0.201767	0	0	0	0	0	0	0	0

Novel_005613	XLOC_05884.	transcript	0	0.247917	0.077901	0.142129	0.310494	0.069276	0.151941	0.013002	0.088666
Novel_005614	XLOC_05885.	transcript	0.446256	0.782353	0.560749	0.812532	0.676892	0.334034	0.694905	0.646062	0.935959
Novel_005615	XLOC_05888.	transcript	0.290057	0.039688	0.207008	0.219298	0.286797	0.243292	0.369806	0.206532	0.107537
Novel_005616	XLOC_05888.	transcript	0	0	0.005212	0	0.231591	0.004522	0	0	0.003413
Novel_005617	XLOC_05888.	transcript	0	0	0	0	0	0	0	0.237937	0
Novel_005618	XLOC_05888.	transcript	0.390745	0.121539	0.399082	0.311439	0.329344	0.382959	0.526547	0.25716	0.492336
Novel_005619	XLOC_05898.	transcript	0.212628	0	0	0	0	0	0	0	0
Novel_005620	XLOC_05902.	transcript	0.172143	0.24051	0.26148	0.462724	0.270585	0.445189	0.291439	0.342706	0.474433
Novel_005621	XLOC_05902.	transcript	0.120583	0	0	0.233696	0.11201	0.262851	0.004274	0	0.15254
Novel_005622	XLOC_05902.	transcript	0.203446	0.262576	0.289437	0	0.140557	0	0.334803	0.326296	0
Novel_005623	XLOC_05902.	transcript	0.003728	0.007607	0.003918	0.013471	0.005182	0.003957	0.005666	0.002752	0.167949
Novel_005624	XLOC_05902.	transcript	0	0	0	0	0	0	0	0	0
Novel_005625	XLOC_05904.	transcript	0.025981	3.557939	0	0.021775	0.088578	0.125284	0.067189	0.085485	0.266719
Novel_005626	XLOC_05907.	transcript	0.384534	0	0	0	0	1.01324	0	0	0
Novel_005627	XLOC_05907.	transcript	0	0.036246	1.331831	1.439437	0.935322	0	0	0.000747	0.676775
Novel_005628	XLOC_05907.	transcript	0	0	0	0	0	0	1.00636	2.026374	0
Novel_005629	XLOC_05908.	transcript	0	0	0	0	0	0.271006	0	0	0
Novel_005630	XLOC_05908.	transcript	0.59536	0.083382	0.101861	0.385666	0	0	0	0	0.29223
Novel_005631	XLOC_05908.	transcript	0	0.006394	0.018159	0.008587	0.034875	0.218968	0.109903	0.363387	0
Novel_005632	XLOC_05908.	transcript	0	0	0	0	0	0.267568	0	0.705719	0
Novel_005633	XLOC_05908.	transcript	0.149415	0	0.330578	0	0.104522	0.009148	0.386302	0.010917	0
Novel_005634	XLOC_05908.	transcript	0	0	0	0	0	0.001129	0.126417	0.022506	0
Novel_005635	XLOC_05909.	transcript	0.497622	0.857529	0.594647	0.420282	0.440133	0.368756	0.684089	0.543706	0.205226
Novel_005636	XLOC_05910.	transcript	0.394115	0.424179	0.293818	0.475505	0.496804	0.382156	0.41558	0.451415	0.259019
Novel_005637	XLOC_05913.	transcript	0.139848	0.081113	0.217319	0.039254	0.052754	0.504105	0.130458	0.134344	0.048011
Novel_005638	XLOC_05913.	transcript	0.020398	0	0.01726	0	0.014323	0	0.030098	0.165785	0.166946
Novel_005639	XLOC_05913.	transcript	0.286735	0.187874	0.008865	0.119166	0.218813	0.176644	0.217828	0	0.078501
Novel_005640	XLOC_05916.	transcript	0.438534	0	0.172639	0.045952	0	0.066152	0.171789	0.158083	0.308646
Novel_005641	XLOC_05916.	transcript	0.046877	0.891406	0.19954	0.141017	0.085616	0.179712	0.111355	0.174118	0.072703
Novel_005642	XLOC_05916.	transcript	0	0.00666	0	0.00094	0	0	0	0	0
Novel_005643	XLOC_05917.	transcript	0	0	0	0	0	0	0.43388	0	0.529099
Novel_005644	XLOC_05917.	transcript	0.157106	0.081588	0.6472	0.008179	0.544019	0.027733	0.37611	0.139922	0.41208
Novel_005645	XLOC_05917.	transcript	0.8377	0.762671	0.218282	0.820654	0.313533	0.642416	0.605147	0.785324	0
Novel_005646	XLOC_05920.	transcript	0	0.386692	0.291915	0	0	0.508357	0.527244	0.184575	0.223318
Novel_005647	XLOC_05920.	transcript	0.200582	0	0	0.276145	0.264911	0	0	0.234979	0.11485
Novel_005648	XLOC_05923.	transcript	0.928399	0.116998	1.302253	1.162297	0.948703	2.887221	1.279905	1.531886	0.966227
Novel_005649	XLOC_05923.	transcript	0	0	0.004171	0.00222	0.005089	0.008023	0	0.004525	0.012923
Novel_005650	XLOC_05923.	transcript	0.895786	0.827391	0.586879	0.757888	0.71268	0.437657	0.536064	0.947703	0.238268

Novel_005651	XLOC_05923.	transcript	0	0.955739	0.857635	0	0.583445	0.493534	0.325621	1.026839	0.373718
Novel_005652	XLOC_05923.	transcript	0	0.084555	0.098567	0.165924	0.087383	0.124919	0.102407	0.054519	0.077014
Novel_005653	XLOC_05923.	transcript	0	0.073766	0.091937	0	0.088033	0.121928	0.096153	0.051623	0.079287
Novel_005654	XLOC_05923.	transcript	0	0.092056	0.088798	0.149006	0.074142	0.115693	0.115347	0.064819	0.085434
Novel_005655	XLOC_05923.	transcript	0	0	0	0	0	0	0	0	0
Novel_005656	XLOC_05923.	transcript	0	0	0	0	0	0	0	0	0
Novel_005657	XLOC_05923.	transcript	0	0.085779	0.08466	0	0.086629	0.18112	0.129893	0.063641	0.087407
Novel_005658	XLOC_05923.	transcript	0.207509	0.076963	0.093033	0	0.090211	0.129253	0.100867	0.05275	0.083013
Novel_005659	XLOC_05923.	transcript	0.195119	0.08374	0.084448	0	0.077108	0.137847	0.121428	0.062858	0.089749
Novel_005660	XLOC_05923.	transcript	0.179662	0.0866	0.093627	0.193659	0.089492	0.119392	0.108356	0.055411	0.078755
Novel_005661	XLOC_05923.	transcript	0.163127	0.093896	0.088397	0.152211	0.075873	0.13934	0.124898	0.065669	0.085751
Novel_005662	XLOC_05923.	transcript	0.207282	0.07631	0.088565	0	0.089687	0.115277	0.103373	0.051994	0.081049
Novel_005663	XLOC_05923.	transcript	0.214595	0.085977	0.087879	0.162701	0.076048	0.125237	0.078983	0.050254	0.078201
Novel_005664	XLOC_05923.	transcript	0	0.082901	0.083406	0.132902	0.066974	0.115162	0.088099	0.056045	0.077904
Novel_005665	XLOC_05923.	transcript	0	0.079653	0.091404	0.189083	0.082231	0.105331	0.075643	0.05244	0.073786
Novel_005666	XLOC_05923.	transcript	0	0.070893	0.076558	0	0.064424	0.116913	0.084972	0.05208	0.081657
Novel_005667	XLOC_05930.	transcript	0.007084	0.050583	0	0	0	0	0	0	0
Novel_005668	XLOC_05930.	transcript	0.068794	0.075145	0.08188	0.158886	0.056555	0.105129	0	0.133444	1.135549
Novel_005669	XLOC_05930.	transcript	0	0.119687	0.697969	0.295307	0.371619	0.409065	0.705631	2.578233	0.581131
Novel_005670	XLOC_05930.	transcript	0	0	0	0	0	0	0.588755	0	0
Novel_005671	XLOC_05930.	transcript	1.036362	0.835214	0.875609	0.367578	0.89053	0.742547	2.410143	0.733986	0.482534
Novel_005672	XLOC_05930.	transcript	2.123149	0.169245	1.443127	1.606751	1.322557	1.455582	0.872859	0.400715	1.619941
Novel_005673	XLOC_05931.	transcript	0.32231	0.066378	0.29156	0.27413	0.196021	0.183844	0.264895	0.301906	0.520529
Novel_005674	XLOC_05931.	transcript	0	0	0	0	0	0	0	0	0
Novel_005675	XLOC_05931.	transcript	0.019886	0	0	0.002242	0	0	0	0.014007	0
Novel_005676	XLOC_05931.	transcript	0	0	0	0	0	0	0	0	0
Novel_005677	XLOC_05935.	transcript	0.220453	0.143573	0.070512	0.091189	0.211333	0.180818	0.1685	0.24633	0.363467
Novel_005678	XLOC_05935.	transcript	0.237169	0.896757	0.588975	0.639215	0.51732	0.788843	0.344643	0.550864	0.697297
Novel_005679	XLOC_05938.	transcript	0	0.071893	0.252901	0	0	0.003379	0	0.199653	0
Novel_005680	XLOC_05938.	transcript	0.375343	0.52774	0	0.451642	0.369261	0.598505	0.474311	0.2529	0.263905
Novel_005681	XLOC_05939.	transcript	0.031246	0.075712	0.109525	0.217937	0.053263	0.211324	0.147715	0.095114	0.123434
Novel_005682	XLOC_05940.	transcript	2.367788	0.833836	0.315487	3.516328	2.249863	3.625117	2.428315	3.653331	0.980475
Novel_005683	XLOC_05940.	transcript	0	0	0.997758	0.006406	0	0.01079	0.005942	0	0.008496
Novel_005684	XLOC_05940.	transcript	0	0.211597	1.34179	0.640923	0.507522	0.720071	0.14695	0.31248	1.333855
Novel_005685	XLOC_05940.	transcript	1.235393	1.444938	0.995451	1.14685	1.082199	1.341035	1.09427	1.384871	0.993029
Novel_005686	XLOC_05941.	transcript	0.042075	0.075435	0.393886	0.739627	0.859203	0.526504	0.387138	0.625865	0.344226
Novel_005687	XLOC_05943.	transcript	0.909944	0.854772	0.409939	0.516933	0.610402	0.744799	0.612896	0.788824	0.548916
Novel_005688	XLOC_05943.	transcript	0.029758	0.189129	0.525642	0.464556	0.268281	0.046496	0.186045	0.069198	0.227823

Novel_005689	XLOC_05953.	transcript	0	0	0.000412	0	0	0	0	0	0.600273
Novel_005690	XLOC_05953.	transcript	0.352593	0.374776	0	0.344717	0.204708	0	0	0	0
Novel_005691	XLOC_05953.	transcript	0.252003	0.333735	0.350589	0.117316	0.206354	0.281133	0.37649	0.350918	0.000228
Novel_005692	XLOC_05953.	transcript	0	0.295763	0.004758	0	0.184308	0.279777	0.373883	0.556579	0.021186
Novel_005693	XLOC_05964.	transcript	0.147888	1.540871	0.169931	0.066042	0.086667	0.193997	0.221489	0.08807	0.183394
Novel_005694	XLOC_05966.	transcript	0.003656	0	0.661435	0	0	0	0.618032	0	0
Novel_005695	XLOC_05966.	transcript	0.129491	0.002738	0.060472	0.020091	0.625033	0.743122	0.070271	0.174858	0.154639
Novel_005696	XLOC_05966.	transcript	0.111672	0.055913	0.414362	0.033236	0.1974	0.152638	0.181706	0.09699	0.417979
Novel_005697	XLOC_05966.	transcript	0.62915	0.540261	0	0.669431	0	0	0.361548	0.750092	0
Novel_005698	XLOC_05982.	transcript	0	0	0	0.009553	0	0	0.004262	0	0
Novel_005699	XLOC_05982.	transcript	0.1419	0	0.086051	0.043476	0.066028	0.084707	0.065736	0.172863	0.098504
Novel_005700	XLOC_05982.	transcript	0	0	0	0.072629	0.043696	0	0.020915	0.087227	0
Novel_005701	XLOC_05982.	transcript	0	0	0	0.002289	0	0	0.068148	0.07449	0
Novel_005702	XLOC_05982.	transcript	0	0	0	0	0	0	0	0	0
Novel_005703	XLOC_05982.	transcript	0	0	0	0	0.03585	0	0	0	0
Novel_005704	XLOC_05982.	transcript	0	0	0	0	0	0	0	0	0
Novel_005705	XLOC_05982.	transcript	0	0.018547	0.060205	0.084456	0	0	0	0.04207	0.024556
Novel_005706	XLOC_05987.	transcript	0.072627	0.320159	0.110444	0.078485	0.092128	0.104883	0.200765	0.121988	0.255004
Novel_005707	XLOC_05990.	transcript	0.041812	0.070243	0.198171	0.035516	0.130027	0	0.253545	0.072788	0.146145
Novel_005708	XLOC_05990.	transcript	0.282138	0.378832	0.131067	0.051475	0.153444	0.167219	0.216866	0.157047	0.272529
Novel_005709	XLOC_05994.	transcript	0.350064	0.589605	0.108261	0.193693	0.225884	0.193063	0.344162	0.523257	0.174059
Novel_005710	XLOC_05994.	transcript	0	0	0	0	0	0	0	0	0
Novel_005711	XLOC_05994.	transcript	0.3913	0	0.340425	0.23104	0.820887	0	0.628552	0.303338	0.347491
Novel_005712	XLOC_05994.	transcript	0.706348	0.401384	0.733558	0.570107	0.318349	0.821216	0.750753	0.561703	0.535456
Novel_005713	XLOC_05994.	transcript	0.4661	0.724859	0.51247	0.708521	0.479943	0.694028	0.637352	0.589089	0.976953
Novel_005714	XLOC_05994.	transcript	0.058587	0.174978	0.001698	0.033711	0.185508	0.017252	0.165136	0.178304	0.084197
Novel_005715	XLOC_05997.	transcript	0.14048	0.289543	0.329818	0.164768	0.353909	0.385703	0.233723	0.289253	0.085329
Novel_005716	XLOC_05997.	transcript	0.043298	0.094126	0.042083	0.040035	0.062711	0.052293	0.156805	0.044762	0.164921
Novel_005717	XLOC_05997.	transcript	0	0	0	0	0	0	0	0.011231	0
Novel_005718	XLOC_05997.	transcript	0.025563	0.105648	0.099553	0.021424	0	0	0.190607	0.039809	0.030295
Novel_005719	XLOC_05998.	transcript	0.621578	0	0.678825	0.6201	0.188613	0	0	0.369115	0
Novel_005720	XLOC_05998.	transcript	0	0.993489	0	0	0	0.796023	0.356895	0.315394	0.324821
Novel_005721	XLOC_05998.	transcript	0	0	0.084111	0.062033	0.20954	0	0	0	0
Novel_005722	XLOC_06000.	transcript	0	0	0.062492	0.215708	0.217507	0.085878	0.274932	0.117719	0
Novel_005723	XLOC_06000.	transcript	0.081354	0	0.213037	0.019318	0.183548	0.046535	0.025132	0.133911	0.114041
Novel_005724	XLOC_06001.	transcript	0.18019	0.091694	0.342945	0.400608	0.314012	0.281372	0.386943	0.233695	0.520909
Novel_005725	XLOC_06001.	transcript	0.024939	0	0	0	0	0	0	0	0
Novel_005726	XLOC_06001.	transcript	0.036283	0.029771	0.038487	0.121131	0	0.217133	0.268375	0.041409	0.128857

Novel_005727	XLOC_06015.	transcript	0.995723	0.877619	0.599575	0.964764	0.680964	0.677698	0.738385	0.879307	0.760784
Novel_005728	XLOC_06017.	transcript	0.039636	0.667692	0.015443	0.015308	0.022447	0.008827	0	0	0
Novel_005729	XLOC_06017.	transcript	0	0	0.058056	0	0	0.271571	0	0	0
Novel_005730	XLOC_06017.	transcript	0	0	0.005133	0.1699	0	0	0	0.246734	0.166697
Novel_005731	XLOC_06017.	transcript	0	0	0	0	0	0	0	0	0.08921
Novel_005732	XLOC_06017.	transcript	0.170317	0.238783	0.233846	0.185352	0.545329	0.143346	0.101589	0.077732	0.122937
Novel_005733	XLOC_06020.	transcript	0.199695	0.5972	0.113042	0.105114	0	0.048949	0.293273	0.09619	0.111974
Novel_005734	XLOC_06020.	transcript	0	0	0	0.037361	0.226752	0	0	0.08111	0
Novel_005735	XLOC_06022.	transcript	0.08482	0	0	0	0	0	0	0.156132	0
Novel_005736	XLOC_06022.	transcript	0.06432	0.066751	0.049324	0.033763	0.03985	0.043812	0.15314	0	0.050113
Novel_005737	XLOC_06022.	transcript	0	0.245556	0.467384	0.380264	0.296209	0	0.601445	0.416961	0
Novel_005738	XLOC_06022.	transcript	0.376095	0	0	0	0	0.035412	0.001792	0	0.012001
Novel_005739	XLOC_06022.	transcript	0	0.065122	0	0	0	0.013281	0.112792	0.163227	0
Novel_005740	XLOC_06022.	transcript	0	0	0.043224	0.011338	0.025198	0	0.020665	0.038556	0.059683
Novel_005741	XLOC_06022.	transcript	0.242945	0.004277	0.146398	0.117148	0.29093	0.450792	0.082167	0.028823	0.452868
Novel_005742	XLOC_06022.	transcript	0.002585	0	0	0	0	0	0	0	0
Novel_005743	XLOC_06023.	transcript	0.181665	0.041076	0.165617	0.092123	0	0	0.224524	0.081504	0.024939
Novel_005744	XLOC_06023.	transcript	0	0	0	0	0	0	0	0.138219	0
Novel_005745	XLOC_06023.	transcript	0.059655	0	0.049785	0.099765	0.111712	0.153758	0.100304	0	0.056069
Novel_005746	XLOC_06023.	transcript	0.018442	0	0	0	0.072776	0	0	0.035167	0.021652
Novel_005747	XLOC_06023.	transcript	0.269825	0.197044	0.155449	0.257899	0.242599	0.343063	0.577137	0.440383	0.067628
Novel_005748	XLOC_06023.	transcript	0	0.038958	0.096485	0.01434	0.146919	0.025182	0.013353	0.067063	0.277063
Novel_005749	XLOC_06024.	transcript	0.343207	0.322464	0.395106	0.286024	0.535426	0.465596	0.385707	0.403564	0.555524
Novel_005750	XLOC_06025.	transcript	0	0	0	0	0	0	0	0	0
Novel_005751	XLOC_06026.	transcript	0.07204	0.700112	0.146794	0.105396	0.196627	0.509974	0.236675	0.418608	0
Novel_005752	XLOC_06026.	transcript	0	0	0	0	0	0	0	0	0
Novel_005753	XLOC_06027.	transcript	0	0	0	0.711583	0	0	0.900475	0	0.626001
Novel_005754	XLOC_06027.	transcript	0	0.065365	0.245051	0.001207	0.35524	0.465692	0	0.624729	0
Novel_005755	XLOC_06027.	transcript	0.517311	0.007423	0.050398	0	0.012064	0.018332	0	0.025731	0
Novel_005756	XLOC_06028.	transcript	0.138988	0	0.078516	0.144469	0.02787	0.092164	0.17106	0.217167	0.064623
Novel_005757	XLOC_06028.	transcript	0.098164	0.009656	0.215462	0.262078	1.046655	0.608844	0.755969	1.568857	0.413151
Novel_005758	XLOC_06033.	transcript	0.225669	0.34054	0.284668	0.2407	0.230256	0.233271	0.254334	0.262813	0.303559
Novel_005759	XLOC_06034.	transcript	0.179383	0.183932	0.245284	0.180638	0.145818	0.335448	0.257243	0.193531	0.258898
Novel_005760	XLOC_06039.	transcript	0	0.155693	0	0	0	0	0	0	0
Novel_005761	XLOC_06060.	transcript	0.216787	0.176092	0.301957	0.242552	0.284749	0.31201	0.427397	0.227343	0.283859
Novel_005762	XLOC_06060.	transcript	0	0	0.016817	0.01729	0	0	0	0	0
Novel_005763	XLOC_06061.	transcript	0.453378	0.063672	0.553676	0.277565	0.255553	0.031413	0.182197	0.194372	0
Novel_005764	XLOC_06061.	transcript	0.061272	0	0.047344	0.012592	0.242886	0.13181	0.186956	0.2137	0.317586

Novel_005765	XLOC_06061.	transcript	0	0	0	0	0	0.591192	0.164854	0.184645	0
Novel_005766	XLOC_06062.	transcript	0.141534	0	0.283889	0.367476	0.176742	0.216418	0.20096	0.288946	0
Novel_005767	XLOC_06062.	transcript	0.063826	0	0.0244	0	0.035574	0.04368	0.088214	0	0.2096
Novel_005768	XLOC_06062.	transcript	0.305004	0.119917	0.040694	0	0.183691	0.050187	0.065954	0	0
Novel_005769	XLOC_06062.	transcript	0	0	0	0	0	0	0.105114	0	0
Novel_005770	XLOC_06062.	transcript	0.574682	0.188886	0.308458	0.08105	0.333051	0.803013	0.594337	0.610023	0.465795
Novel_005771	XLOC_06062.	transcript	0	2.210968	0	0.628273	0.419318	0	0	0.251834	0
Novel_005772	XLOC_0606€.	transcript	4.483723	2.215975	5.254358	6.012288	5.630727	3.776433	8.080978	5.578369	4.713013
Novel_005773	XLOC_0606Ɛ.	transcript	0	0	0	0	0.54959	0.267576	0	0	0
Novel_005774	XLOC_0606Ɛ.	transcript	0.084942	0.932726	0.251224	0	0	0	0.143171	0.401782	0
Novel_005775	XLOC_0606Ɛ.	transcript	0.186285	0.468061	0.015183	0	0.043432	0	0	0.024944	0
Novel_005776	XLOC_0606Ɛ.	transcript	0.013804	0.089853	0.014593	0.017353	0.028236	0.041645	0.038689	0.409272	0.023154
Novel_005777	XLOC_0606Ɛ.	transcript	0.256081	0.02494	0.025569	0.448354	0.268779	0.07763	0.917418	0.039017	0.123438
Novel_005778	XLOC_0606Ɛ.	transcript	0	0.011126	0.199742	0	0	0	0.000133	0	0.488301
Novel_005779	XLOC_0606Ɛ.	transcript	0.046636	0.174305	0	0.009334	0.047075	0.078831	0.138857	0.043096	0.088278
Novel_005780	XLOC_0606Ɛ.	transcript	0.004973	0.38882	0.053039	0.074488	0	0.021184	0.054179	0.074104	0
Novel_005781	XLOC_0606Ɛ.	transcript	0	0	0.063545	0	0	0	0	0	0.042803
Novel_005782	XLOC_0606Ɛ.	transcript	0.351474	0.122166	0.342743	0.278301	0.282847	0.356043	0.410788	0.345275	0.340349
Novel_005783	XLOC_0607C.	transcript	0.163009	0	0.31515	0.213922	0.313022	0.26728	0.376032	0.283134	0.250786
Novel_005784	XLOC_06071.	transcript	0.478836	0.080425	0	0	0	0	0.000936	0	0
Novel_005785	XLOC_06071.	transcript	0	0	0	0	0	0	0	0	0
Novel_005786	XLOC_06071.	transcript	0	0	0	0	0.010738	0	0	0	0
Novel_005787	XLOC_06071.	transcript	0	0	0	0.004773	0	0.025932	0.046317	0	0.080585
Novel_005788	XLOC_06071.	transcript	0.153059	0.741404	0.109101	0.057867	0.12489	0.069709	0.3498	0.196548	0.458623
Novel_005789	XLOC_06071.	transcript	0	0	0	0	0	0	0	0	0
Novel_005790	XLOC_06071.	transcript	0	0.541702	0.099612	0.061131	0.119227	0.136978	0.000494	0.005533	0
Novel_005791	XLOC_06072.	transcript	0.245574	0	0.258057	0.209348	0.50697	0.35382	0.367545	0.381869	0.151893
Novel_005792	XLOC_06072.	transcript	0.070504	0.028176	0.12614	0.081876	0.101728	0.149346	0.47651	0.089795	0.110447
Novel_005793	XLOC_06072.	transcript	0	0.242685	0	0	0.149745	0	0.02701	0	0
Novel_005794	XLOC_06074.	transcript	0	0	0	0.001639	0	0	0.004039	0	0.382724
Novel_005795	XLOC_06074.	transcript	0	0	0	0.298469	0.19927	0	0.498528	0.338935	0
Novel_005796	XLOC_06074.	transcript	0.352685	0.463163	0.592126	0.192784	0.329595	0.640531	0.006845	0	0.149755
Novel_005797	XLOC_06074.	transcript	0.181455	0.089628	0.016149	0.044475	0.055257	0.104545	0.275057	0.061049	1.206601
Novel_005798	XLOC_0607S.	transcript	0	0	0	0	0	0.058208	0.573813	0	0
Novel_005799	XLOC_0608C.	transcript	6.746213	4.9632	8.586484	11.4615	8.576019	10.33219	10.52082	11.86188	6.639603
Novel_005800	XLOC_0608C.	transcript	0.677363	0.160109	1.510241	1.763305	1.020059	1.408642	1.46484	1.651583	1.691582
Novel_005801	XLOC_06081.	transcript	0	0	0	0	0	0	0	0	0
Novel_005802	XLOC_06081.	transcript	0.294418	0.525484	0.629707	0.553998	0.577815	0.334786	0.008084	0.013809	0.889995

Novel_005803	XLOC_06082.	transcript	0.757087	0.723356	0.765647	0.696383	0.845472	1.216697	0.893669	0.881952	0.654075
Novel_005804	XLOC_06082.	transcript	0.018881	0.015496	0.058741	0.007088	0.005864	0.023177	0.006225	0.033474	0.005862
Novel_005805	XLOC_06083.	transcript	0.194473	0.157285	0.380371	0.30235	0.540361	0.291996	0.360108	0.431703	0.475075
Novel_005806	XLOC_06083.	transcript	0.070625	0.164262	0.111147	0.417845	0.341067	0.280352	0.500392	0.209778	0.687414
Novel_005807	XLOC_06085.	transcript	0.084392	0	0	0	0	0.057578	0.093465	0	0.139498
Novel_005808	XLOC_06085.	transcript	0.049321	0.203736	0.187855	0.137543	0.125018	0.224808	0.190115	0.155998	0.093543
Novel_005809	XLOC_06085.	transcript	0	0	0	0	0	0.080686	0	0.001537	0
Novel_005810	XLOC_06085.	transcript	0	0.301804	0	0	0	0.059649	0	0.004794	0
Novel_005811	XLOC_06085.	transcript	0.402422	0.017017	0.404975	0.269916	0.018254	0.43075	0.191507	0.059207	0.470425
Novel_005812	XLOC_06085.	transcript	0	0.430167	0	0.115198	0	0	0	0.004871	0
Novel_005813	XLOC_06085.	transcript	0	0	0.020064	0.639279	0.342037	0.238229	0.008647	0.21463	0.467493
Novel_005814	XLOC_06085.	transcript	0	0	0.043425	0	0	0.147271	0	0.17685	0
Novel_005815	XLOC_06085.	transcript	0	0	0	0	0.465134	0.252329	0	0.244711	0.439787
Novel_005816	XLOC_06085.	transcript	0	0	0.306406	0	0	0.196477	0	0.096575	0
Novel_005817	XLOC_06085.	transcript	0	0.001811	0	0.327764	0.191544	0.415396	0	0.210169	0.323985
Novel_005818	XLOC_06085.	transcript	0	0	0	0	0	0.272137	0	0	0
Novel_005819	XLOC_06085.	transcript	0.302539	0	0.294482	0.33647	0.211575	0.177631	0.087121	0.246922	0.362607
Novel_005820	XLOC_06085.	transcript	0	0	0	0	0.506241	0	0.70729	0.518495	0
Novel_005821	XLOC_06086.	transcript	0	0.17282	0	0.037594	0	0	0.011104	0.0456	0.11979
Novel_005822	XLOC_06086.	transcript	0	0.144203	0.121663	0.060982	0.189491	0.456727	0.128725	0.177867	0
Novel_005823	XLOC_06086.	transcript	0	0	0.041681	0.026698	0.004658	0	0	0	0.343078
Novel_005824	XLOC_06088.	transcript	0.696539	0.500986	0.636561	0.866723	0.631993	0.662892	0.982899	0.861528	0.829328
Novel_005825	XLOC_06088.	transcript	0	0	0.566433	0	0	0	0.316981	0.601255	0
Novel_005826	XLOC_06088.	transcript	0	0	0	0	0.006401	0	0	0	0
Novel_005827	XLOC_06088.	transcript	0.062116	0.265703	0.272935	0.170964	0.101202	0.161461	1.062694	0.109344	1.31222
Novel_005828	XLOC_06088.	transcript	0.522552	0.356769	0.535631	0.518181	0.980474	0.656034	0.40005	0.600295	0.094329
Novel_005829	XLOC_06088.	transcript	0.360369	0.779787	0.447001	0.604029	0.479084	0.456526	0.154303	0.136197	0.043145
Novel_005830	XLOC_06088.	transcript	0	0	0	0	0	0	0.398433	0.381883	0.414581
Novel_005831	XLOC_06088.	transcript	0	0	0	0	0.574591	0	0	0	0
Novel_005832	XLOC_06089.	transcript	0.811563	0.504308	0.725471	0.949068	1.03617	0.899436	1.195617	1.003352	0.6697
Novel_005833	XLOC_06091.	transcript	3.795775	0.039875	4.712615	3.32012	4.104186	3.471187	5.439486	3.666398	6.910706
Novel_005834	XLOC_06093.	transcript	0.091589	0.048921	0.186029	0.248324	0.400874	0.152733	0.069978	0.047723	0.038687
Novel_005835	XLOC_06093.	transcript	0.113117	0.392316	0.317599	0.381194	0.242472	0.597718	0.201287	0.167137	0.540002
Novel_005836	XLOC_06094.	transcript	0	0	0	0	0	0	0	0	0
Novel_005837	XLOC_06094.	transcript	0.425959	0.113197	0.366595	0.294847	0.357489	0.317131	0.361054	0.319077	0.574498
Novel_005838	XLOC_06094.	transcript	0.113749	0.042419	0.033047	0.036829	0.013937	0.03274	0.087512	0.119989	0.069606
Novel_005839	XLOC_06094.	transcript	0.003137	0	0.003714	0	0.00373	0	0.001888	0.074734	0.006345
Novel_005840	XLOC_06094.	transcript	0.005557	0	0	0	0	0	0.277271	0.006449	0

Novel_005841	XLOC_06094.	transcript	0	0.496982	0	0	0	0	0	0	0
Novel_005842	XLOC_06105.	transcript	0.032961	0.699386	0.226301	0.241355	0.278738	0.214011	0.262718	0.275738	0.411275
Novel_005843	XLOC_06105.	transcript	0.212773	0.100368	0	0.04132	0	0	0	0	0.003283
Novel_005844	XLOC_06106.	transcript	0.38214	0.420929	0.434212	0.408047	0.232989	0.47357	0.364759	0.410148	0.265419
Novel_005845	XLOC_06109.	transcript	0.169086	0.133327	0.124702	0.156515	0.104602	0.165631	0.213784	0.123471	0.110331
Novel_005846	XLOC_06112.	transcript	0	0	0	0	0	0	0	0	0
Novel_005847	XLOC_06112.	transcript	0	0	0	0.00359	0.005657	0	0.00594	0.002958	1.019433
Novel_005848	XLOC_06112.	transcript	0.034846	0.041251	0	0	0.117778	0	0.180195	0	0
Novel_005849	XLOC_06116.	transcript	0.272606	0.010715	0.411888	0.128596	0.40719	0.280903	0.802388	0.306294	0.121676
Novel_005850	XLOC_06134.	transcript	0.057604	0	0.337037	0.166787	0.049323	0.099702	0.115789	0.381811	0.208715
Novel_005851	XLOC_06134.	transcript	0	0.23412	0	0	0.308919	0	0	0	0
Novel_005852	XLOC_06134.	transcript	0.259359	0.057737	0.105678	0.220752	0.019715	0.145822	0.21702	0.007606	0.191186
Novel_005853	XLOC_06135.	transcript	3.483909	1.033539	3.840228	3.584882	3.210908	3.310001	3.943484	4.944839	2.98709
Novel_005854	XLOC_06135.	transcript	0	0	0	0	0	0	0.806469	0.002357	0
Novel_005855	XLOC_06137.	transcript	0.059446	1.37923	0.02512	0	0	0	0.056462	0	0
Novel_005856	XLOC_06141.	transcript	0.005675	0	0	0.312332	0	0	0.329156	0.372609	0.343794
Novel_005857	XLOC_06141.	transcript	0.515363	0.915286	0.579279	0	0.53014	0.424852	0.332419	0	0
Novel_005858	XLOC_06141.	transcript	0	0	0	0	0	0	0.014908	0.011413	0
Novel_005859	XLOC_06141.	transcript	0	0	0	0	0	0	0	0	0
Novel_005860	XLOC_06149.	transcript	0.376962	0.029696	0.321959	0.344863	0.368057	0.341274	0.343127	0.445674	0.258547
Novel_005861	XLOC_06149.	transcript	0	0	0	0	0.013094	0.097348	0.288446	0	0
Novel_005862	XLOC_06153.	transcript	0	0	0	0.065961	0	0	0	0.112199	0
Novel_005863	XLOC_06153.	transcript	0.430123	0.604804	0.021681	0.794119	0.502126	0.056504	0.130238	0.36499	0.022201
Novel_005864	XLOC_06153.	transcript	0	0.461953	0.323415	0.196451	0.084598	0.535301	0.481245	0.381774	0.395663
Novel_005865	XLOC_06154.	transcript	0	0	0.002179	0.004311	0	0	0.00223	0.001518	0.002059
Novel_005866	XLOC_06154.	transcript	2.272941	0	1.530285	0.581774	0.627665	1.487087	0.24802	0.719413	0.489319
Novel_005867	XLOC_06154.	transcript	0	0	0	0	0	0	0	0.188779	0
Novel_005868	XLOC_06154.	transcript	0.357879	0	0.362007	0	0	0	0	0.221295	0.667923
Novel_005869	XLOC_06154.	transcript	0	1.044559	0	0.952625	1.075618	0.116063	2.176975	1.050444	0.513538
Novel_005870	XLOC_06155.	transcript	0.066947	0.092999	0.054298	0.011229	0.034259	0.024248	0.05807	0.041629	0.03855
Novel_005871	XLOC_06155.	transcript	0.106475	0.143539	0	0.035767	0.038064	0.032181	0.05983	0.01523	0.078104
Novel_005872	XLOC_06155.	transcript	0	0	0.004463	0	0	0	0	0.001841	0
Novel_005873	XLOC_06156.	transcript	0	0	0	0	0	0	0	0	0
Novel_005874	XLOC_06156.	transcript	1.247475	0.397089	1.210957	0.849625	0.670698	1.250229	0.973937	1.068079	1.312483
Novel_005875	XLOC_06156.	transcript	0.315312	0.488034	0.50859	0.109982	1.221882	0.275223	1.529846	0.46088	0.754409
Novel_005876	XLOC_06158.	transcript	0.229295	0.022617	0.181107	0.192952	0.027523	0.201235	0.118889	0.053151	0.099015
Novel_005877	XLOC_06161.	transcript	3.079211	1.376925	2.232704	1.877733	2.803155	1.290507	3.425022	2.757539	1.308632
Novel_005878	XLOC_06166.	transcript	0.006116	0.001226	0.087155	0	0	0	0.002519	0.003842	0

Novel_005879	XLOC_06166.	transcript	0.07993	0	0.07419	0	0	0	0.00133	0.006008	0
Novel_005880	XLOC_06166.	transcript	0.054902	0.065164	0.049292	0.082176	0.072827	0.119242	0.001351	0.001312	0.183319
Novel_005881	XLOC_06166.	transcript	0.097989	0.141728	0.045124	0	0.037878	0	0.114926	0.021671	0
Novel_005882	XLOC_06166.	transcript	0.059282	0.069027	0.022671	0	0.08504	0	0.335078	0.013259	0
Novel_005883	XLOC_06166.	transcript	0.129656	0	0.107233	0.022709	0.357639	0.14654	0.06683	0.093377	0.160404
Novel_005884	XLOC_06166.	transcript	0.061343	0	0.064056	0	0.199018	0	0	0	0.114673
Novel_005885	XLOC_06166.	transcript	0.107995	0.019727	0.161438	0.049729	0	0.002237	0.027461	0	0
Novel_005886	XLOC_06166.	transcript	0.054551	0	0.083863	0	0.283256	0	0	0	0.150715
Novel_005887	XLOC_06166.	transcript	0	0	0.213903	0	0	0	0.186035	0	0
Novel_005888	XLOC_06166.	transcript	0.007676	0.010092	0.000755	0.00079	0.010461	0.009815	0.013502	0.002271	0.003105
Novel_005889	XLOC_06166.	transcript	0.03962	0.003355	0.000236	0.001402	0.000432	0.001692	0.03939	0.01493	0.001941
Novel_005890	XLOC_06166.	transcript	0.244291	0.353339	0.207499	0.099197	0.043293	0.122966	0.538662	0.228332	0.122704
Novel_005891	XLOC_06166.	transcript	0	0.120216	0.012603	0.004663	0.023167	0	0.020994	0	0
Novel_005892	XLOC_06172.	transcript	0	0.176675	0.060499	0.069789	0	0.050234	0.135764	0	0
Novel_005893	XLOC_06172.	transcript	0.299417	0.081829	0.107838	0.098134	0.116072	0.151785	0.129643	0.241661	0.067978
Novel_005894	XLOC_06172.	transcript	0.000315	0	0	0	0	0	0	0	0
Novel_005895	XLOC_06172.	transcript	0	0	0.220284	0	0.253784	0	0.560176	0.34218	0.452677
Novel_005896	XLOC_06172.	transcript	0	0	0	0	0	0	0	0.212447	0
Novel_005897	XLOC_06172.	transcript	0	0	0	0	0	0	0	0.219236	0
Novel_005898	XLOC_06172.	transcript	0.001923	0	0	0.058784	0	0	0	0	0
Novel_005899	XLOC_06172.	transcript	0.375012	0	0.107265	0.002563	0.153595	0.537402	0.064758	0	0.263517
Novel_005900	XLOC_06172.	transcript	0	0	0	0.330334	0	0	0	0	0
Novel_005901	XLOC_06174.	transcript	0.239742	0.038646	0.125062	0.033819	0	0.07764	0.012639	0.09737	0
Novel_005902	XLOC_06174.	transcript	0	0	0	0.037496	0	0.152727	0	0	0
Novel_005903	XLOC_06174.	transcript	0.19869	0	0	0.084637	0.115531	0.209672	0.158852	0.116491	0
Novel_005904	XLOC_06174.	transcript	0.064083	0	0.054519	0.083369	0.12225	0.13588	0.114522	0.15892	0
Novel_005905	XLOC_06175.	transcript	0.153024	0	0.116054	0.120239	0.118921	0.042325	0.262896	0.016026	0
Novel_005906	XLOC_06175.	transcript	0	0	0	0	0.089944	0.176499	0.290459	0.025683	0
Novel_005907	XLOC_06183.	transcript	0	0.549671	0	0	0	0	0	0	0.013598
Novel_005908	XLOC_06188.	transcript	0.548653	0.66253	0.354556	0	0.384118	0.442215	0.388952	0.369297	0.327243
Novel_005909	XLOC_06188.	transcript	0	0	0	0	0	0	0	0	0
Novel_005910	XLOC_06197.	transcript	0.229967	0.20379	1.06172	0.023124	0.252534	1.571547	0.814594	0.335409	0.124186
Novel_005911	XLOC_06197.	transcript	2.064334	0.347496	1.520922	0.083744	3.457751	1.558063	1.025235	0.250665	1.270443
Novel_005912	XLOC_06197.	transcript	0	0	0	0.114005	0	0	0	0.439282	0
Novel_005913	XLOC_06197.	transcript	0	0	0	1.130822	0	0	0.000246	0	0
Novel_005914	XLOC_06197.	transcript	0	0	0.187067	1.916035	0	0.204477	0.916779	2.372624	0
Novel_005915	XLOC_06197.	transcript	0.612673	0	0.174793	3.094705	0.507465	0.20404	0.913054	3.182373	1.134011
Novel_005916	XLOC_06197.	transcript	0	0	0	0	0	0	0	0	0

Novel_005917	XLOC_06204.	transcript	0	0	0	0	0	0	0	0.124295	0
Novel_005918	XLOC_06205.	transcript	0.087584	0.071031	0.037096	0	0.092173	0.15357	0.121885	0.037749	0.129316
Novel_005919	XLOC_06205.	transcript	0	0	0	0.138179	0	0	0	0.097883	0
Novel_005920	XLOC_06207.	transcript	0	0	0	0	0	0	0	0	0
Novel_005921	XLOC_06207.	transcript	0.039445	0.063667	0.09907	0.069205	0.519525	0.470406	0.061273	0.069055	0.241113
Novel_005922	XLOC_06207.	transcript	0.500483	0.378538	0.515525	0.551763	0.005512	0.212716	0.627517	0.444998	0.277898
Novel_005923	XLOC_06207.	transcript	0.166636	0.000112	0.103175	0.147391	0.125571	0.097542	0.127817	0.153599	0.000151
Novel_005924	XLOC_06208.	transcript	0.119302	0.060748	0.057738	0.143757	0.167892	0.077834	0.160777	0.164588	0.351114
Novel_005925	XLOC_06208.	transcript	0	0	0.044946	0	0	0	0.118926	0.02866	0.075706
Novel_005926	XLOC_06209.	transcript	0.094916	0	0.156332	0.170097	0.07562	0	0.158884	0.412029	0.043665
Novel_005927	XLOC_06209.	transcript	0.208695	0.187966	0.224726	0.211927	0.190011	0.214731	0.271778	0.172916	0.092726
Novel_005928	XLOC_06209.	transcript	0	0.030418	0	0.04932	0	0	0	0	0
Novel_005929	XLOC_06209.	transcript	0.073584	0.052769	0.103754	0.096862	0.085801	0.133727	0.120671	0.096925	0.144932
Novel_005930	XLOC_06209.	transcript	0.391624	0	0.115955	0	0.032572	0.086279	0	0.327354	0
Novel_005931	XLOC_06211.	transcript	1.071041	1.320212	0.974635	1.135056	0.995046	1.341018	1.07112	1.399176	1.179802
Novel_005932	XLOC_06211.	transcript	0.283564	0.031955	0.245165	0.225009	0.249363	0.21147	0.3469	0.123006	0.030565
Novel_005933	XLOC_06212.	transcript	0.786483	0.978492	0.707391	1.043138	0.81667	0.81909	0.736787	1.335905	1.016643
Novel_005934	XLOC_06216.	transcript	0.49736	0.845181	0.447528	0.556734	0.32092	0.25469	0.238489	0.283978	0.675614
Novel_005935	XLOC_06216.	transcript	0.343139	1.155273	0.46528	0.005748	0.343635	0.420156	0.725092	0.374398	0.16085
Novel_005936	XLOC_06228.	transcript	0.019351	0.323715	0	0	0	0	0.040049	0.032599	0.016869
Novel_005937	XLOC_06233.	transcript	0.206984	0	0.102997	0.225156	0.19987	0.19428	0.037345	0.072569	0.315774
Novel_005938	XLOC_06235.	transcript	0.4244	0.749835	0.311071	0.257516	0.358867	0.311001	0.475621	0.297744	0.324087
Novel_005939	XLOC_06239.	transcript	0	0	0	0.088769	0	0	0	0.119945	0
Novel_005940	XLOC_06240.	transcript	0.053917	0.791088	0.047146	0.03946	0.036793	0.190217	0.158921	0.024355	0.108309
Novel_005941	XLOC_06240.	transcript	0	0.938336	0	0	0	0	0.013599	0	0
Novel_005942	XLOC_06240.	transcript	0	0	0	0	0	0	0	0	0
Novel_005943	XLOC_06240.	transcript	0	1.033429	0.006449	0.006916	0	0.017942	0.030835	0	0.016298
Novel_005944	XLOC_06246.	transcript	0.439025	1.704163	1.96441	2.538959	2.351391	2.006118	2.583971	2.583874	1.969271
Novel_005945	XLOC_06246.	transcript	0	0	0	0	0	0	0	0	0
Novel_005946	XLOC_06246.	transcript	0.788641	0	0	0	0	0	0	0	0
Novel_005947	XLOC_06248.	transcript	0.115194	0	0.566692	0.282787	0.267742	0.147856	0.410478	0.063147	0
Novel_005948	XLOC_06248.	transcript	0.368572	0.46552	0.051854	0	0.066728	0.72575	0.001872	1.077611	0.4769
Novel_005949	XLOC_06248.	transcript	0	0	0	0.949492	0	0	0.374318	0	0
Novel_005950	XLOC_06248.	transcript	0.782903	0.569565	0	0.248867	0.043057	0.563444	1.22536	0.313809	0.765991
Novel_005951	XLOC_06248.	transcript	0	0	2.948879	0	0.611216	0	0	0	0
Novel_005952	XLOC_06249.	transcript	0.150904	0.51328	0.349773	0.346101	0.233463	0.406569	0.385337	0.467381	0.202882
Novel_005953	XLOC_06257.	transcript	0.177657	0.413926	0.871632	0.659088	0.456067	0.742137	0.629629	0.995892	0.376373
Novel_005954	XLOC_06257.	transcript	0.121111	0.242835	0.241946	0.204761	0.184636	0.217527	0.202407	0.247009	0.083321

Novel_005955	XLOC_06257.	transcript	0.007603	0.151294	0.005465	0.01629	0	0.020788	0.027625	0.011333	0
Novel_005956	XLOC_06257.	transcript	1.206597	1.196622	0.75973	0.882007	0.680396	1.09342	0.636477	0.06755	1.414233
Novel_005957	XLOC_06258.	transcript	0.027713	0	0	0	0.006816	0.171342	0	0.007519	0
Novel_005958	XLOC_06258.	transcript	0.17421	0.242006	0.120219	0.164195	0.110279	0.089752	0.364419	0.145732	0.165254
Novel_005959	XLOC_06258.	transcript	0	0	0	0	0	0	0	0	0.248195
Novel_005960	XLOC_06262.	transcript	0.55911	0.352549	0.389488	0.22269	0.242994	0.212665	0.494722	0.465689	0.39053
Novel_005961	XLOC_06264.	transcript	0.541879	0.45819	0.64587	0.888398	0.645759	0.578796	0.883655	1.031797	1.745407
Novel_005962	XLOC_06266.	transcript	0.317529	0.182831	0.368983	0.3169	0.337344	0.318305	0.522716	0.340166	0.167134
Novel_005963	XLOC_06266.	transcript	0	0	0.080774	0.031107	0.048554	0.011646	0.360185	0.014489	0
Novel_005964	XLOC_06266.	transcript	0	0	0	0	0	0	0.315802	0	0
Novel_005965	XLOC_06266.	transcript	0	0	0	0	0	0	0.208196	0	0
Novel_005966	XLOC_06266.	transcript	0	0	0	0	0.303035	0.475013	0.331008	0.136305	0.293449
Novel_005967	XLOC_06266.	transcript	0.06962	0.078716	0.246835	0.188335	0	0	0	0.186478	0
Novel_005968	XLOC_06266.	transcript	0.032078	0	0.033914	0.004481	0.021909	0	0	0.010737	0
Novel_005969	XLOC_06266.	transcript	0.008767	0	0.075115	0	0.19954	0.178668	0.055837	0.172397	0.099079
Novel_005970	XLOC_06266.	transcript	0.492065	0.312024	0.672852	0.500834	0.72777	0.778118	0.696875	0.543651	0.445214
Novel_005971	XLOC_06266.	transcript	0	0	0	0	0	0	0.079393	0	0
Novel_005972	XLOC_06266.	transcript	0.001187	0	0.010763	0	0.050162	0.035146	0.292002	0.494641	0
Novel_005973	XLOC_06266.	transcript	0	0.274752	0.027792	0.040519	0.010839	0.015604	0.242804	0.291413	0.084308
Novel_005974	XLOC_06266.	transcript	0	0.224762	0	0.015032	0	0	0.220656	0.149641	0.179384
Novel_005975	XLOC_06266.	transcript	0.28636	0.01058	0.302503	0.329116	0.294843	0.373489	0.004537	0.137435	0.119644
Novel_005976	XLOC_06266.	transcript	0.035344	0.019538	0.024288	0.15197	0.277976	0.054803	0.129593	0.021645	0.061208
Novel_005977	XLOC_06268.	transcript	0	0	0.019702	0	0	0	0.359576	0	0
Novel_005978	XLOC_06268.	transcript	0	0.63364	0.018609	0.495505	0	0.687252	0.381783	1.07927	0.033046
Novel_005979	XLOC_06268.	transcript	0.818408	0.900859	0.836756	0.502459	1.030082	0.059253	0.64824	0.033114	0.555733
Novel_005980	XLOC_06268.	transcript	0	0.383592	0	0	0	0	0	0	0
Novel_005981	XLOC_06268.	transcript	0	0	0	0	0	0	0	0	0.021487
Novel_005982	XLOC_06268.	transcript	0	0	0	0	0.056441	0	0	0	0
Novel_005983	XLOC_06270.	transcript	0.561994	1.265183	0.466117	0.650633	0.83952	0.65256	1.589396	0.718688	1.030337
Novel_005984	XLOC_06270.	transcript	0.376327	0.222703	0.3276	0.321281	0.354223	0.53756	0.515407	0.449712	0.488114
Novel_005985	XLOC_06270.	transcript	0.154587	0.100647	0	0.114823	0	0.124805	0.139941	0	0.081727
Novel_005986	XLOC_06272.	transcript	0	0	0	0	0	0	0	0	0
Novel_005987	XLOC_06272.	transcript	0.416078	0	0.570784	0.401989	0.965478	1.347486	0.362323	0.414819	0.405089
Novel_005988	XLOC_06273.	transcript	0	0	0	0	0.194362	0	0.152467	0	0
Novel_005989	XLOC_06273.	transcript	0.124123	0.068151	0.193824	0.276629	0	0.112622	0	0.15197	0.103412
Novel_005990	XLOC_06273.	transcript	0.057458	0.000677	0.143481	0.031703	0.190481	0.07678	0.144498	0.090548	0.040645
Novel_005991	XLOC_06273.	transcript	0	0	0	0	0	0	0.082454	0	0
Novel_005992	XLOC_06273.	transcript	0	0.094437	0.075813	0	0	0	0	0.125923	0.11463

Novel_005993	XLOC_06273.	transcript	0.033582	0.162544	0.080184	0.050983	0.068049	0.147489	0.063126	0.082738	0.168443
Novel_005994	XLOC_06273.	transcript	0.365214	0	0.157757	0.179334	0.033264	0	0.041132	0.009853	0
Novel_005995	XLOC_06273.	transcript	0.251983	0	0	0.185643	0.050972	0.048491	0.182119	0.011034	0.029082
Novel_005996	XLOC_06273.	transcript	0.043205	0	0	0.19569	0	0	0.08117	0	0.031382
Novel_005997	XLOC_06273.	transcript	0.046533	0.117337	0.143142	0	0.11834	0	0.204028	0.17573	0.092271
Novel_005998	XLOC_06273.	transcript	0.032325	0.041364	0	0	0	0.271797	0	0	0
Novel_005999	XLOC_06278.	transcript	0	0	0	0	0	0	0	0.040364	0
Novel_006000	XLOC_06278.	transcript	0.909008	0.430286	0.467122	0.194604	0.361564	0.527031	0.738322	0.278491	0.365361
Novel_006001	XLOC_06278.	transcript	0	0.648631	0.135714	0.100933	0.284703	0.182054	0.157724	0.279935	0.214699
Novel_006002	XLOC_06278.	transcript	0	0	0	0.773435	0	0	0	0.642997	0
Novel_006003	XLOC_06289.	transcript	0.060436	0.52603	0	0.003294	0.003646	0	0	0.000968	0
Novel_006004	XLOC_06289.	transcript	0	0	0	0	0	0	0	0	0
Novel_006005	XLOC_06289.	transcript	0.039544	0.154862	0.281055	0.320893	0.241357	0.248466	0.498047	0.392302	0.255317
Novel_006006	XLOC_06289.	transcript	0.3286	0	0	0	0	0	0	0	0
Novel_006007	XLOC_06310.	transcript	0.236706	1.000515	0.192649	0.214953	0.170578	0.272733	0.327234	0.259512	0.271448
Novel_006008	XLOC_06318.	transcript	0	0.176798	0.220107	0.099473	0.326058	0	0	0.152988	0.3395
Novel_006009	XLOC_06318.	transcript	0	0	0	0.229406	0	0.22124	0.144317	0.148009	0.137523
Novel_006010	XLOC_06318.	transcript	0.233801	0.044196	0.243147	0.166666	0.253817	0.209215	0.295375	0.26898	0.20317
Novel_006011	XLOC_06318.	transcript	0.055961	0.008822	0.182331	0.154569	0.011447	0.091453	0.119728	0.015523	0
Novel_006012	XLOC_06322.	transcript	0.065791	0.993383	0.191581	0.203517	0.197222	0.154973	0.152569	0.183511	0.156566
Novel_006013	XLOC_06322.	transcript	0.283976	0.123412	0.096846	0	0	0	0.016515	0.013055	0.051733
Novel_006014	XLOC_06322.	transcript	0.046345	0.007189	0.007133	0.032455	0.019375	0.060068	0.054122	0.05496	0.002944
Novel_006015	XLOC_06322.	transcript	0.05643	0	0.019354	0	0.139418	0.016964	0.223118	0.008077	0.127396
Novel_006016	XLOC_06322.	transcript	0.098751	0.420889	0.076055	0	0.081603	0.096275	0	0.108531	0.190632
Novel_006017	XLOC_06323.	transcript	0.529205	0.505768	0.432384	0.212458	0.279324	0.459625	0.543212	0.432405	0.298956
Novel_006018	XLOC_06323.	transcript	0	0	0	0	0.386041	0	0	0	0.015243
Novel_006019	XLOC_06323.	transcript	0.643816	1.175837	0.541817	0.439982	0.57015	0.500348	0.801472	0.771852	0.407185
Novel_006020	XLOC_06324.	transcript	0.05914	0	0.272332	0.017143	0.09565	0.489087	0.144888	0.028649	0
Novel_006021	XLOC_06324.	transcript	0.141449	0.313847	0.134057	0.345898	0.336474	0	0.325934	0.161607	0.454913
Novel_006022	XLOC_06324.	transcript	0.228344	0.141314	0.037653	0.031145	0.005289	0.019492	0.041297	0.196642	0
Novel_006023	XLOC_06328.	transcript	0	0.539937	0.239194	0.042304	0.012025	0.224678	0.416862	0.134952	0.590793
Novel_006024	XLOC_06328.	transcript	0.576895	0.407389	0.386676	0.362715	0.476473	0.383501	0.632999	0.583127	0.293473
Novel_006025	XLOC_06328.	transcript	0.561812	1.833682	0.983869	0.883646	0.848496	1.132153	1.207924	0.834319	1.359292
Novel_006026	XLOC_06330.	transcript	0	0.095965	0	0.140156	0.390505	0.135814	0	0.157578	0
Novel_006027	XLOC_06330.	transcript	0	0	0.227623	0.724822	0.308323	0.181931	0.226257	0.31855	0.218951
Novel_006028	XLOC_06330.	transcript	0	0.115285	0.341365	0.247174	0.342043	0.191717	0.112678	0.217881	0.200915
Novel_006029	XLOC_06330.	transcript	0	0.137701	0	0.256587	0.248161	0.143778	0	0.244882	0
Novel_006030	XLOC_06330.	transcript	0	0	0.252929	0.344353	0.356309	0.176996	0.133308	0.196094	0.221765

Novel_006031	XLOC_06330.	transcript	0.285632	0.094012	0.721736	0.249763	0.441363	0.16878	0.069482	0.251144	0.191975
Novel_006032	XLOC_06330.	transcript	0	0.204157	0.3772	0.650642	0.402432	0.205661	0.564332	0.352001	0.345619
Novel_006033	XLOC_06330.	transcript	0	0.098369	0.261499	0.157097	0.27479	0.158384	0.091044	0.244973	0.154254
Novel_006034	XLOC_06330.	transcript	0	0	0.251479	0.157718	0.259566	0.141017	0.101595	0.203125	0.175931
Novel_006035	XLOC_06330.	transcript	0.320087	0.109579	1.458303	0.440551	1.018611	0.20372	0.095166	0.225687	0.23703
Novel_006036	XLOC_06330.	transcript	0.35323	0.057241	0.298931	0.28688	0.472007	0.106164	0.120937	0.280883	0.086456
Novel_006037	XLOC_06330.	transcript	0	0.313966	0.181586	0.190321	0.174958	0	0	0	0
Novel_006038	XLOC_06330.	transcript	3.785618	4.52117	0.329893	0.714219	0.312748	1.913358	3.602448	3.130444	1.622475
Novel_006039	XLOC_06333.	transcript	0.502231	0.095292	0.708507	0.857322	1.153251	0.6964	1.148591	0.912614	0.324313
Novel_006040	XLOC_06333.	transcript	0.1256	0.004088	0.138535	0.045645	0.332776	0.10116	0.60516	0.516914	0.184789
Novel_006041	XLOC_06333.	transcript	0.609365	0.636598	0.408699	0.54614	0.64535	0.405266	0.397083	0.692063	0.466019
Novel_006042	XLOC_06333.	transcript	0.06682	0.058588	0.088433	0.079251	0.072694	0.054283	0.597976	0.059222	0.131659
Novel_006043	XLOC_06341.	transcript	0.802026	0.399032	0.730843	0.733501	0.736714	0.775845	0.761757	0.832873	0.725161
Novel_006044	XLOC_06341.	transcript	0	0	0	0	0	0	0	0	0
Novel_006045	XLOC_06341.	transcript	0	0	0	0	0	0	0	0	0
Novel_006046	XLOC_06342.	transcript	0	0.917129	0	0	0.342418	0	0.787991	0.493979	0
Novel_006047	XLOC_06342.	transcript	0.256755	0	0.532808	0.202912	0	0.118226	0	0	1.006219
Novel_006048	XLOC_06342.	transcript	0.325546	0.151581	0.044522	0	0	0	0	0.028886	0.057255
Novel_006049	XLOC_06345.	transcript	0.047694	0.072413	0.071758	0.130117	0	0.089562	0.091832	0.000589	0.0727
Novel_006050	XLOC_06345.	transcript	0.261063	0.429308	0.513172	0.546844	0.545864	0.517532	0.419765	0.347338	0.418437
Novel_006051	XLOC_06345.	transcript	0.053866	0	0.098812	0.167668	0.176903	0.157223	0.118147	0.075095	0
Novel_006052	XLOC_06346.	transcript	0.049297	0	0	0	0	0	0.370286	0	0
Novel_006053	XLOC_06346.	transcript	0.520758	0.643089	0.565348	0.503994	0.474414	0.616449	0.139006	0.694794	0.384123
Novel_006054	XLOC_06346.	transcript	0.188207	0.391283	0.180383	0.167134	0.276341	0.169491	0.284904	0.232619	0.22995
Novel_006055	XLOC_06347.	transcript	0	0.012301	0.007237	0.004814	0	0.010774	0.003732	0.005775	0.00268
Novel_006056	XLOC_06347.	transcript	0.593939	0.839867	0.68885	0.402481	0.451118	0.58916	0.674023	0.133941	0.339887
Novel_006057	XLOC_06347.	transcript	0	0	0	0.009166	0.387984	0	0	0.152377	0.287362
Novel_006058	XLOC_06347.	transcript	0	0	0	0	0	0	0.438026	0.158177	0
Novel_006059	XLOC_06347.	transcript	0	0	0	0	0	0	0	0.170694	0
Novel_006060	XLOC_06349.	transcript	1.076646	0.919379	0.543319	0.473242	0.752866	0.584016	0.907018	0.740366	0.573373
Novel_006061	XLOC_06349.	transcript	0	0	0	0	0	0	0	0	0
Novel_006062	XLOC_06350.	transcript	0.385195	0.320802	0.150411	0.130807	0.19996	0.068674	0.427333	0.206492	0.136131
Novel_006063	XLOC_06350.	transcript	0	0.104441	0.151981	0	0.063191	0.14953	0.078418	0.050594	0.087411
Novel_006064	XLOC_06350.	transcript	0	0.42614	0.039511	0.130554	0.106116	0.155761	0.055448	0.069419	0.061377
Novel_006065	XLOC_06350.	transcript	0	0	0	0	0	0	0	0	0
Novel_006066	XLOC_06358.	transcript	0	0	0	0	0	0	0	0	0
Novel_006067	XLOC_06358.	transcript	1.413133	1.99095	2.544905	1.999647	2.226048	2.331994	1.74337	2.597225	1.055383
Novel_006068	XLOC_06362.	transcript	1.595129	1.501511	2.801301	3.782123	2.619922	2.783992	1.892737	3.173525	2.557014

Novel_006069	XLOC_06362.	transcript	0.438021	0.018469	1.512811	1.74496	2.3326	3.080844	1.792933	2.764587	0.82438
Novel_006070	XLOC_06362.	transcript	0	1.798614	1.306646	1.574166	0	0	1.174188	1.623575	0.814952
Novel_006071	XLOC_06362.	transcript	0.083789	0.130015	0.420243	0.167718	0.26244	0	0.005983	0.121176	0.291423
Novel_006072	XLOC_06362.	transcript	0.122155	0.007234	0.190068	0.259721	0.038195	0.323229	0.132379	0.307256	0.020639
Novel_006073	XLOC_06362.	transcript	0	0.294953	0	0.239516	0.019955	0	0.384356	0.101036	0
Novel_006074	XLOC_06365.	transcript	0	0	0.208152	0	0	0	0.060998	0.030668	0
Novel_006075	XLOC_06365.	transcript	0.160304	0.160662	0.245478	0.196404	0.230329	0.236618	0.212913	0.20979	0.190462
Novel_006076	XLOC_06365.	transcript	0	0	0	0	0	0.055134	0.001719	0	0
Novel_006077	XLOC_06365.	transcript	0	0	0.00624	0	0.007019	0	0.051113	0	0.016413
Novel_006078	XLOC_06365.	transcript	0	0	0	0	0.123015	0	0	0	0
Novel_006079	XLOC_06365.	transcript	32.41433	63.9478	44.30047	56.57043	51.68475	75.527	54.04469	64.83112	55.9399
Novel_006080	XLOC_06366.	transcript	0.399981	0.243075	0.148923	0.002064	0.18812	0.089698	0.033979	0.041749	0.028093
Novel_006081	XLOC_06366.	transcript	0.826669	0.865889	1.351734	1.464163	1.062671	1.725375	0.902135	1.636001	1.047871
Novel_006082	XLOC_06366.	transcript	1.583286	1.491132	0.801068	2.450231	2.372925	2.411573	1.801057	0.631085	0.554679
Novel_006083	XLOC_06366.	transcript	0	0	0.473554	0	0	0.545474	0	0.512673	0
Novel_006084	XLOC_06366.	transcript	0	0	0	0	0	0	0.003862	0.499879	0
Novel_006085	XLOC_06366.	transcript	0.075578	0.106526	0.129035	0.067005	0.173671	0.109607	0.177197	0.297495	0.065577
Novel_006086	XLOC_06366.	transcript	0.332889	0	0	0	0	0	0	0	0.343761
Novel_006087	XLOC_06366.	transcript	2.487415	0.701679	2.767066	2.611866	3.625599	1.6726	2.660305	2.320663	1.725121
Novel_006088	XLOC_06366.	transcript	0	0.381675	0	1.172692	0	0.896786	0	0.313138	0
Novel_006089	XLOC_06366.	transcript	0	0	0	0	0	0	0	0.039673	0
Novel_006090	XLOC_06366.	transcript	0.541067	0.207103	0.980477	0	0	0.532645	0.9979	0	0.578798
Novel_006091	XLOC_06367.	transcript	0.140816	0.128978	0.374936	0.086312	0.106721	0.128325	0.156936	0.15433	0
Novel_006092	XLOC_06367.	transcript	0.132131	0	0	0.111806	0.22194	0.24558	0.168396	0.443314	0
Novel_006093	XLOC_06367.	transcript	0.115495	0.466348	0.319226	0.349057	0.372595	0.839928	0.481373	0.244926	0.956718
Novel_006094	XLOC_06367.	transcript	0.000162	0.051818	0.307598	0.043165	0.046501	0.114794	0.010983	0.035378	0.261776
Novel_006095	XLOC_06367.	transcript	0.451911	0	0	0.446405	0.260712	0.239961	0.035072	0.091592	0
Novel_006096	XLOC_06367.	transcript	0.343503	0.224514	0.232928	0.121106	0.335582	0.273226	0.558036	0.330036	0.450944
Novel_006097	XLOC_06367.	transcript	0	0.102098	0.718612	0.663665	0.478291	0.118179	0.23113	0.086674	0
Novel_006098	XLOC_06368.	transcript	0	0.005512	0.005046	0.056206	0.013222	0	0.000111	0.007074	0.006451
Novel_006099	XLOC_06368.	transcript	0.02987	4.463526	0.070235	0.044902	0.040248	0.352359	0.789773	0	0.185088
Novel_006100	XLOC_06368.	transcript	0.726982	2.891718	0.603472	0.425444	0.546194	0.46823	0.367353	0.473295	0.942722
Novel_006101	XLOC_06368.	transcript	0.19212	0.32386	0.401919	0.370746	0.289974	0.277937	0.419826	0.496901	0.418869
Novel_006102	XLOC_06369.	transcript	0.018293	2.60354	0	0.12322	0	0.3783	0	0	0.068164
Novel_006103	XLOC_06369.	transcript	0.428136	0.062274	0.016788	0.30257	0.604982	0.3979	0.014846	0.015911	0.445587
Novel_006104	XLOC_06369.	transcript	0	0	0	0.009438	0	0.012044	0.273797	0	0
Novel_006105	XLOC_06369.	transcript	0.066829	0.383067	0.555635	0	0	0	0.234043	0.289217	0
Novel_006106	XLOC_06369.	transcript	0	0.579188	0.006324	0.097541	0.007589	0.028585	0.390935	0.203881	0.133206

Novel_006107	XLOC_06369.	transcript	0.863663	1.984095	0.767263	0.422056	0.869116	0.653697	1.181233	0.821085	0.576513
Novel_006108	XLOC_06369.	transcript	0.591722	0.851455	0.871226	0.600262	0.647803	0.983576	1.199503	1.10814	0.646789
Novel_006109	XLOC_06369.	transcript	0.709441	2.458411	1.033798	1.220298	1.018705	0.738407	1.252725	1.487082	0.453678
Novel_006110	XLOC_06370.	transcript	0	4.261155	0	0.029323	0.108724	0.054751	0.051668	0.143019	0
Novel_006111	XLOC_06370.	transcript	0.082891	0.39869	0.39759	0.340519	0.088578	0.414078	0.189708	0.183379	0.123954
Novel_006112	XLOC_06370.	transcript	1.526396	0.173528	0.218217	1.156422	0.274188	0.178042	1.59748	0.003776	1.23407
Novel_006113	XLOC_06370.	transcript	0.182015	0	0.804848	0.173296	0.786702	0.696702	0.267812	0.993553	0
Novel_006114	XLOC_06370.	transcript	0.04338	0	1.474187	0.460695	1.614919	1.32019	1.563364	1.743427	0.810994
Novel_006115	XLOC_06370.	transcript	0.118418	0	0	0.123961	0.001195	0	0.21544	0.072465	0.170406
Novel_006116	XLOC_06370.	transcript	0.252878	0.144186	0.782937	0.118506	0.203398	0.445585	0.676011	0.636635	0.34419
Novel_006117	XLOC_06371.	transcript	0.067184	0	0.066441	0.067454	0.046708	0	0.102135	0.144707	0.130339
Novel_006118	XLOC_06372.	transcript	18.95588	19.97568	16.69938	19.23624	17.59801	17.96252	22.83121	24.62004	18.95992
Novel_006119	XLOC_06372.	transcript	0.091629	2.147451	0.176373	0.105556	0.081702	0	0.215236	0.175952	0.585914
Novel_006120	XLOC_06372.	transcript	0.272698	0.218282	0	0.589103	0.700583	1.641259	0	0	1.859079
Novel_006121	XLOC_06372.	transcript	0.580186	0.540294	0.998159	1.516305	1.030663	1.417024	1.417612	1.15481	1.277524
Novel_006122	XLOC_06374.	transcript	0.324242	0	0.166475	0.068695	0.197313	0.468681	0.900053	0.678471	0.215556
Novel_006123	XLOC_06377.	transcript	0.13235	0.347556	0.32519	0.135573	0.195528	0.159093	0.392268	0.281664	0.273792
Novel_006124	XLOC_06378.	transcript	0.283282	0.372403	0.227831	0.369202	0.357702	0.572956	0.54831	0.434253	0.345043
Novel_006125	XLOC_06391.	transcript	0.164508	0.429923	0.057555	0.086124	0.091088	0.065573	0.122932	0.077689	0.048659
Novel_006126	XLOC_06391.	transcript	0.404718	0.309736	0.414103	0.256949	0.366249	0.326823	0.662696	0.39594	0.320693
Novel_006127	XLOC_06396.	transcript	0.767716	1.662503	0.610156	1.041065	0.494629	0.63347	1.235485	0.71935	0.528254
Novel_006128	XLOC_06397.	transcript	0.09702	0.152944	0.110351	0.074874	0.143598	0.087777	0.141336	0.172993	0.064986
Novel_006129	XLOC_06399.	transcript	0.398987	0.411362	0.477203	0.678818	0.67174	0.624635	0.566182	0.561979	0.563614
Novel_006130	XLOC_06399.	transcript	2.836175	5.907125	7.166621	8.218015	5.098589	7.343671	4.599751	5.332342	7.42976
Novel_006131	XLOC_06400.	transcript	0.059594	0.148571	0.07536	0.006254	0.070144	0.044332	0.085676	0.03926	0.079497
Novel_006132	XLOC_06400.	transcript	0.666677	0.832127	0.421512	0.404901	0.585626	0.5995	0.814234	0.565771	0.703038
Novel_006133	XLOC_06400.	transcript	0	1.272045	0.008033	0.276832	0.059207	0	0	0.609742	0.006527
Novel_006134	XLOC_06400.	transcript	0	0.142815	0	0	0.115855	0	0.118679	0.005262	0
Novel_006135	XLOC_06400.	transcript	0	1.445467	0	0	0.147517	0	0	0	0
Novel_006136	XLOC_06401.	transcript	0.205103	1.094228	0.079236	0.11934	0.15609	0.267008	0	0.138007	0.16527
Novel_006137	XLOC_06401.	transcript	0	2.069158	0	0	0	0	0.382908	0	0
Novel_006138	XLOC_06403.	transcript	0.214672	0.147573	0.057201	0.071775	0	0.067816	0.043767	0.077907	0.686858
Novel_006139	XLOC_06403.	transcript	0.719159	1.26888	0.776053	0.86193	1.037993	0.920232	1.224343	1.011815	0.437482
Novel_006140	XLOC_06403.	transcript	0	0	0.115104	0.028751	0	0.028368	0	0.015799	0
Novel_006141	XLOC_06406.	transcript	0.593774	0.932203	0.780322	0.003191	0.534632	0.97803	0.539284	2.032189	0.790923
Novel_006142	XLOC_06406.	transcript	0.477823	0.601085	1.268523	0.012036	0.244016	0.134506	0.15263	0.153226	1.479553
Novel_006143	XLOC_06406.	transcript	0.158134	0.196925	0	1.302389	1.435694	0.998863	0.743172	0.001322	0.696354
Novel_006144	XLOC_06406.	transcript	0	0	0	0.600618	0	0	0	0	0

Novel_006145	XLOC_06406.	transcript	0.129968	0.143485	0	0.165553	0.212122	0.974342	0.068982	0.14639	0.46497
Novel_006146	XLOC_06406.	transcript	0	0	0.497467	1.111986	0.889957	0	0.618685	0.583855	0
Novel_006147	XLOC_06406.	transcript	0.161643	0.526247	0.214149	0.28494	0.264384	0.2458	0.288154	0.285711	0.115638
Novel_006148	XLOC_06406.	transcript	0.038596	0.708982	0	0	0	0	0	0	0
Novel_006149	XLOC_06409.	transcript	0	0	0	0.281366	0	0.0544	0	0	0
Novel_006150	XLOC_06409.	transcript	0.103561	0.024753	0.581064	0.109344	0.335744	0	0.368554	0.112648	0.030093
Novel_006151	XLOC_06409.	transcript	0	0	0.011067	0	0	0	0.003914	0	0
Novel_006152	XLOC_06412.	transcript	0.213517	0.041085	0.133728	0.373096	0.206038	0.166456	0.33704	0.292693	0.118984
Novel_006153	XLOC_06427.	transcript	0.138067	0.289526	0.205294	0.195651	0.261828	0.252171	0.416412	0.251516	0.31608
Novel_006154	XLOC_06430.	transcript	0.236774	0.123694	0.387617	0.20826	0.316507	0.561858	0.225923	0.269539	0.416248
Novel_006155	XLOC_06444.	transcript	0.20617	0.271826	0.275467	0.241251	0.289819	0.334382	0.345182	0.353056	0.112752
Novel_006156	XLOC_06444.	transcript	0	0	0	0	0	0	0	0	0.308313
Novel_006157	XLOC_06453.	transcript	0.357775	0.203145	0.42052	0.278736	0.375461	0.32951	0.441375	0.350018	0.23589
Novel_006158	XLOC_06457.	transcript	0.044513	0.348753	0.055631	0.034375	0.087659	0.10881	0.559423	0.491825	0.017211
Novel_006159	XLOC_06457.	transcript	0.719526	0.172026	0.687978	0.491502	0.42472	0.612042	0.282578	0.360632	0.486778
Novel_006160	XLOC_06457.	transcript	0	0	0	0	0	0	0	0	0
Novel_006161	XLOC_06466.	transcript	0	0	0	0	0	0.194782	1.185314	0.360874	0
Novel_006162	XLOC_06466.	transcript	0	0	0	0	0	0	0	0.73749	0
Novel_006163	XLOC_06466.	transcript	0.990119	0	0	0	0.857942	0.515562	0	0	0
Novel_006164	XLOC_06466.	transcript	0.159351	0.632469	0.77284	0.702454	0.087808	0	0.005916	0	0.565199
Novel_006165	XLOC_06468.	transcript	0	6.917283	0	14.29953	14.93571	22.89943	0	0	29.42739
Novel_006166	XLOC_06469.	transcript	0.067035	0.235127	0.160849	0.105179	0.176234	0.07077	0.065677	0.103358	0.169052
Novel_006167	XLOC_06475.	transcript	0	0	0.651968	0	0	0	0	0	0
Novel_006168	XLOC_06475.	transcript	0	0	0.201601	0	0.005808	0	0.020579	0.025406	0
Novel_006169	XLOC_06475.	transcript	0.469314	0.052294	0.282717	0.695372	0.591761	0.770885	0.558531	0.626154	0.459038
Novel_006170	XLOC_06489.	transcript	0.434185	0.876098	0.147314	0.238169	0.335754	0.202704	0.343647	0.302557	0.253633
Novel_006171	XLOC_06489.	transcript	0	0	0	0	0	0	0.002787	0	0
Novel_006172	XLOC_06489.	transcript	0.003565	0.015181	0.00661	0.003078	0.011235	0.005161	0.002325	0.003461	0.009334
Novel_006173	XLOC_06489.	transcript	1.090463	0.368067	0.668259	0.376937	0.596626	0.691863	0.533557	0.588414	0.633556
Novel_006174	XLOC_06489.	transcript	0.431935	0.045551	0.330681	0.29136	0.137846	0.003677	0.297907	0.228905	0.469594
Novel_006175	XLOC_06490.	transcript	0.072963	0.391384	0	0.098228	0.056841	0.044404	0.055123	0.095388	0.20364
Novel_006176	XLOC_06490.	transcript	0	0	0	0	0	0	0	0	0
Novel_006177	XLOC_06490.	transcript	0.099046	0	0.120546	0.102979	0.010875	0.00587	0.011126	0.061853	0
Novel_006178	XLOC_06490.	transcript	0.018738	0	0.021425	0	0.013522	0.009575	0.01396	0.323634	0
Novel_006179	XLOC_06490.	transcript	0.044687	0	0.00398	0.072993	0.051906	0.106236	0.174495	0.073945	0
Novel_006180	XLOC_06490.	transcript	0.13584	0	0	0.178438	0	0.002868	0	0.128891	0
Novel_006181	XLOC_06490.	transcript	0.031631	0	0	0.106372	0.029257	0.101171	0.299359	0.083483	0
Novel_006182	XLOC_06490.	transcript	0.093326	0	0.296338	0.190629	0.120723	0.237413	0.531063	0.162045	0

Novel_006183	XLOC_06493.	transcript	0.097304	0.049857	0	0.348143	0.093814	0.165168	0.017053	0.103409	0.067523
Novel_006184	XLOC_06493.	transcript	0	0	0	0	0	0	0	0	0
Novel_006185	XLOC_06493.	transcript	0.171714	0	0.290524	0	0.165664	0.072317	0.256059	0.158984	0.15426
Novel_006186	XLOC_06493.	transcript	0.315504	1.345861	0.523016	1.165913	0.810113	0.793993	0.876788	0.167875	0.110895
Novel_006187	XLOC_06493.	transcript	0	0.045769	0.372842	0.000303	0	0	0.027639	0.512237	0.818168
Novel_006188	XLOC_06493.	transcript	0	0	0	0	0	0	0	0	0
Novel_006189	XLOC_06494.	transcript	0.830347	0	1.815565	1.49314	1.539108	1.835852	0.809565	2.142887	0.712152
Novel_006190	XLOC_06494.	transcript	0.300196	0.351181	0.28431	0.489509	0.149649	0.251651	0.360561	0.139557	0.163253
Novel_006191	XLOC_06494.	transcript	0.631597	0.862641	0.437345	0.902547	1.056028	1.11835	0.833919	1.070144	0.66722
Novel_006192	XLOC_06495.	transcript	0.473182	0.532834	0.370038	0.535225	0.582041	0.142666	0.272511	1.034234	0.636526
Novel_006193	XLOC_06495.	transcript	0.179192	0.011859	0.336219	0.083424	0.294413	0.103834	0.221568	0.132716	0.398013
Novel_006194	XLOC_06495.	transcript	0	0.167767	0	0.168894	0	0.101687	0	0	0
Novel_006195	XLOC_06495.	transcript	0.422642	0.288558	1.120407	0.655378	0.633443	0.578229	0.62402	0.72252	0.554928
Novel_006196	XLOC_06495.	transcript	2.108361	2.910707	4.988832	4.905342	4.731241	4.87666	2.684012	3.20997	4.374556
Novel_006197	XLOC_06495.	transcript	0.720583	1.235838	2.034841	1.764589	2.004751	2.000561	1.555729	2.312109	1.008476
Novel_006198	XLOC_06497.	transcript	0	0	0.185088	0	0.177192	0.13786	0.168213	0	0.199312
Novel_006199	XLOC_06497.	transcript	0.683079	0.739478	0.742148	0.3946	0.757824	0.364398	0.546165	1.083735	0.980204
Novel_006200	XLOC_06497.	transcript	0.63288	0.336998	0.282707	0.682373	0.374473	0.596706	0.656186	0.58403	0
Novel_006201	XLOC_06498.	transcript	0.021989	0.034421	0.15424	0.429256	0.235274	0.255888	0.233699	0.344535	0.239688
Novel_006202	XLOC_06498.	transcript	0	0	0	0	0.013473	0	0	0.022794	0
Novel_006203	XLOC_06499.	transcript	1.62731	1.684163	1.927993	3.580171	2.879026	2.242118	2.912205	3.424477	2.284851
Novel_006204	XLOC_06501.	transcript	0	0	0.05138	0.039641	0	0.046309	0.048591	0.008828	0.026928
Novel_006205	XLOC_06501.	transcript	0	0	0	0	0	0	0	0	0
Novel_006206	XLOC_06501.	transcript	0.203451	0.452132	0.029271	0.297812	0.314734	0.1467	0.307986	0.450934	0.198002
Novel_006207	XLOC_06501.	transcript	0	0	0.451098	0.345895	0.189542	0.27763	0.201169	0	0.386553
Novel_006208	XLOC_06502.	transcript	1.09093	1.929334	1.279922	0.203554	0.909776	1.172448	1.076762	1.132125	1.042373
Novel_006209	XLOC_06502.	transcript	0	0	0	0.074476	0	0	0	0	0
Novel_006210	XLOC_06502.	transcript	0	0	0	0.679007	0	0	0.558872	0	0
Novel_006211	XLOC_06502.	transcript	0.299955	0.452737	0.357496	6.80E-05	0.408542	0.023808	0.495917	0.56084	0.220691
Novel_006212	XLOC_06502.	transcript	0.104978	0.11354	0.093145	0.562642	0.096347	0.424363	0.258477	0.084767	0.370018
Novel_006213	XLOC_06502.	transcript	0.043924	0.710538	0.048092	0.029604	0.067427	0.107727	0.178411	0.097433	0.026631
Novel_006214	XLOC_06502.	transcript	0.044875	0.211285	0.106169	0.116292	0.061329	0.010028	0.063984	0.005261	0.178825
Novel_006215	XLOC_06502.	transcript	0	0.004893	0.051717	0	0	0.080676	0.113796	0.022585	0.034109
Novel_006216	XLOC_06503.	transcript	1.070446	0.214373	0.270919	0.60399	0.17178	0.209872	0.438715	0.394585	0.182867
Novel_006217	XLOC_06503.	transcript	0.43063	0.531036	1.403353	1.042315	1.428197	1.557202	1.554216	1.460582	1.278271
Novel_006218	XLOC_06503.	transcript	29.93787	25.49754	41.68035	52.53302	43.41588	42.32579	33.79638	42.40019	36.6852
Novel_006219	XLOC_06504.	transcript	0.163916	0.06088	0.242161	0.366439	0.275516	0.194079	0.03644	0.158135	0.247088
Novel_006220	XLOC_06504.	transcript	0.284512	0.587964	0.484172	0.243704	0.547156	0.623009	0.733792	0.419542	0.427564

Novel_006221	XLOC_06504	transcript	0	0.003055	0.001667	0	0.002641	0	0.00024	0.000673	0
Novel_006222	XLOC_06504	transcript	0.870251	2.278868	1.288526	1.702859	1.60911	1.03197	1.938434	1.730916	1.548053
Novel_006223	XLOC_06505	transcript	0.265519	0.312174	0.541922	0.659736	0.394559	0.863893	0.274397	0.334628	0.473789
Novel_006224	XLOC_06505	transcript	0	0	0	0	0	0	0	0.101096	0
Novel_006225	XLOC_06506	transcript	0	0.134095	0	0.170655	0.069953	0.075368	0.119354	0.057995	0.081056
Novel_006226	XLOC_06506	transcript	1.136988	0.503582	1.501825	1.540374	1.506148	0.641249	2.068063	1.429858	1.536141
Novel_006227	XLOC_06506	transcript	0.20865	0.725586	0.600327	0.767373	0.614016	0.616935	0.488489	0.797191	0.397785
Novel_006228	XLOC_06507	transcript	0.028763	0	0.089893	0	0.029419	0.104112	0.047806	0.032223	0.113931
Novel_006229	XLOC_06507	transcript	0	0	0.008879	0.0065	0.025769	0.030264	0.045334	0.004389	0
Novel_006230	XLOC_06507	transcript	0.1003	0.076201	0.070693	0.111978	0.05514	0.130259	0.207338	0.185072	0.202968
Novel_006231	XLOC_06509	transcript	1.376136	1.187353	1.030835	1.072125	1.16648	1.058782	1.562004	0.717101	0.830914
Novel_006232	XLOC_06509	transcript	0.081438	0.062245	0.121	0.068254	0.055529	0.096183	0.156259	0.130942	0
Novel_006233	XLOC_06509	transcript	0.292096	0.190941	0.031564	0.329305	0	0.133227	0.031067	0.122043	0.159869
Novel_006234	XLOC_06509	transcript	0	0	0.428155	0	0.218792	0.230458	0	0.116514	0.314266
Novel_006235	XLOC_06509	transcript	2.039249	0.893085	1.701206	2.081859	2.246604	1.559855	4.156284	2.135968	3.349173
Novel_006236	XLOC_06510	transcript	1.653702	0.354104	1.748667	2.313416	1.768812	1.762143	1.981354	2.149638	1.353783
Novel_006237	XLOC_06510	transcript	0	0	0	0	0	0	0	0	0
Novel_006238	XLOC_06510	transcript	0.015054	0.004874	0	0.015864	0.031755	0.010365	0	0.048674	0
Novel_006239	XLOC_06510	transcript	0	0	0	0	0	0	1.15648	0	0
Novel_006240	XLOC_06510	transcript	0.07309	0	0.082582	0.205374	0.11876	0.025092	0.058456	0.072807	0.077602
Novel_006241	XLOC_06510	transcript	0	0.013574	0	0	0.298895	0	0	0.118005	0
Novel_006242	XLOC_06511	transcript	0.4747	1.189003	1.941468	0.361632	1.277764	1.748474	0.887095	1.370363	1.104311
Novel_006243	XLOC_06511	transcript	0.114889	0.122943	0.197884	0.770888	0.188154	0.244949	0.232858	0.161434	0.385454
Novel_006244	XLOC_06511	transcript	0.242139	0.095413	0	0.481436	0.071971	0.097403	0	0	0.004931
Novel_006245	XLOC_06512	transcript	0.569023	1.845926	3.622157	3.681132	2.133519	3.349961	1.601226	2.717411	1.679873
Novel_006246	XLOC_06512	transcript	1.346143	1.536772	0.75454	1.024048	1.001634	1.056341	1.866226	1.653589	1.18005
Novel_006247	XLOC_06512	transcript	0.15172	0.0721	0.118095	0.165688	0.163834	0.22201	0.159254	0.14142	0.112028
Novel_006248	XLOC_06514	transcript	2.175735	1.637341	2.596174	2.250524	2.873399	2.229839	2.486569	3.469518	2.198438
Novel_006249	XLOC_06514	transcript	0.220203	1.116542	0.108796	1.265356	0.189479	0.134054	1.544912	0.339177	0.869486
Novel_006250	XLOC_06514	transcript	0.952129	1.54865	1.331182	2.116575	2.09167	1.755593	2.944116	2.374887	1.893325
Novel_006251	XLOC_06516	transcript	0.043965	0.380429	0.024004	0.055185	0.1857	0.061637	0.118752	0.090911	0.313018
Novel_006252	XLOC_06516	transcript	0.443868	0.192641	1.493935	0.591846	0.008195	0.649527	0.250609	0.834832	0.76158
Novel_006253	XLOC_06516	transcript	4.585956	1.937757	4.258989	2.463856	3.458813	2.712857	9.909235	6.361731	2.895905
Novel_006254	XLOC_06516	transcript	1.76935	0.358035	0.600252	0.930527	0.040534	1.935947	0.444277	0.756637	4.216898
Novel_006255	XLOC_06516	transcript	0.38457	0.350535	1.417858	1.578605	0.313996	3.911679	0.773686	2.009935	6.20356
Novel_006256	XLOC_06516	transcript	3.706399	0.433676	1.612103	2.291581	0.905955	0.707032	3.604031	1.552978	11.82031
Novel_006257	XLOC_06516	transcript	0.205951	3.573278	0.989662	0.499288	6.741701	1.333685	0.086764	0.392162	0.768557
Novel_006258	XLOC_06516	transcript	0.774601	0	0.078324	0.522226	0.226499	0.173891	6.705363	0.418493	0.788682

Novel_006259	XLOC_06516.	transcript	0	0	0	0	0	0	4.970467	0	0
Novel_006260	XLOC_06516.	transcript	0.429947	0.681979	0.955096	0.630433	0.934845	0.744456	0.399359	0.57419	0.666226
Novel_006261	XLOC_06516.	transcript	1.077319	0.679557	1.140232	1.228318	1.15265	0.039397	1.505721	1.24785	0.982421
Novel_006262	XLOC_06516.	transcript	0	0	0	0	0.023234	0	0	6.40E-05	0
Novel_006263	XLOC_06518.	transcript	0	0	0	0	0	0.000543	0	0	0
Novel_006264	XLOC_06518.	transcript	0.742602	0.917092	0.972635	0.270693	1.577243	0.515133	0.668502	0.622711	0.001021
Novel_006265	XLOC_06518.	transcript	0	0.698075	0	0.194025	0	0.099412	0.640839	0.286786	0.001434
Novel_006266	XLOC_06518.	transcript	0	0.39461	0.873202	0.001254	1.246129	0.097657	0.688032	1.031556	0.001581
Novel_006267	XLOC_06518.	transcript	0	1.000657	0	0.99919	0	1.53807	0.590945	0.288415	1.190033
Novel_006268	XLOC_06518.	transcript	0.844026	0.275219	0.76889	1.611607	0.672165	1.349037	0.679113	0.563637	0.943799
Novel_006269	XLOC_06518.	transcript	1.057654	0.742636	1.164292	1.340721	0.677469	1.167274	1.394037	0.970411	2.046716
Novel_006270	XLOC_06518.	transcript	0	0.183271	0	0	0	0	0	0	0
Novel_006271	XLOC_06520.	transcript	0.291037	0.236502	0.00422	0.13183	0.311222	0	0.484382	0.2873	0.008014
Novel_006272	XLOC_06520.	transcript	0.20214	0.110831	0.314188	0.099301	0.076459	0.274295	0.116223	0.11008	0.288865
Novel_006273	XLOC_06521.	transcript	0.727473	0.458899	0.557043	0.973845	0.765393	0.970397	1.013835	0.760982	0.865089
Novel_006274	XLOC_06522.	transcript	0.496509	0.572424	0.690406	0.759055	0.498176	0.364405	0.815838	0.42594	0.50141
Novel_006275	XLOC_06522.	transcript	0.046368	0.330647	0.113009	0.079971	0.353735	0.260306	0.177945	0.01681	0.245832
Novel_006276	XLOC_06522.	transcript	0	0	0	0	0	0	0	0	0
Novel_006277	XLOC_06522.	transcript	0	0	0	0	0	0.309949	0	0.258844	0
Novel_006278	XLOC_06522.	transcript	0	0	0	5.90E-05	0	0.083437	0	0.085678	0.073748
Novel_006279	XLOC_06522.	transcript	0.074459	0.128233	0.084198	0.220023	0.103566	0.00538	0.097127	0.068783	0.001049
Novel_006280	XLOC_06523.	transcript	0.069807	0.098242	0.298758	0.179183	0.100758	0.348954	0.069874	0.358995	0.184483
Novel_006281	XLOC_06525.	transcript	0.330576	0.249779	0.187873	0.134799	0.087261	0.21503	0.179401	0.390969	0.312766
Novel_006282	XLOC_06526.	transcript	0.367985	0.445059	0.576688	0.371249	0.457694	0.41284	0.474298	0.437478	0.315417
Novel_006283	XLOC_06526.	transcript	0	0	0	0	0.011208	0	0.012526	0.002824	0
Novel_006284	XLOC_06526.	transcript	0.008688	0	0.024355	0.01448	0.023686	0.061028	0.025943	0.02532	0.007804
Novel_006285	XLOC_06526.	transcript	0.30813	0.07522	0.522324	0.276925	0.314188	0.229761	0.268753	0.352425	0.115126
Novel_006286	XLOC_06526.	transcript	0	0	0	0	0	0	0	0	0
Novel_006287	XLOC_06527.	transcript	0	0.006999	0	0.52951	0	0	0.005636	0.001842	0
Novel_006288	XLOC_06527.	transcript	0.654671	0.000222	0.008012	0	0.23306	1.115202	0.315812	0.001149	0
Novel_006289	XLOC_06527.	transcript	0.677476	2.379139	1.198788	1.148013	1.081245	0.006066	1.466487	1.620929	0.848725
Novel_006290	XLOC_06527.	transcript	0.249783	1.024606	0.488196	0.477946	0.384174	0.436874	0.442465	0.342006	0.257119
Novel_006291	XLOC_06528.	transcript	0	0.074918	0.043615	0.605352	0.453334	0.091969	0.648658	0.083298	0.053954
Novel_006292	XLOC_06528.	transcript	0.044308	0.431907	0.06663	0.092664	0.067077	0.091615	0.038978	0.04868	0.031669
Novel_006293	XLOC_06528.	transcript	0	0.206075	0	0	0	0	0	0	0.047657
Novel_006294	XLOC_06528.	transcript	0.452852	0.465207	0.567024	0.120291	0	0.559409	0	0.325653	0.713474
Novel_006295	XLOC_06528.	transcript	0.201641	0.073235	0.127104	0	0.158124	0.339365	0.048282	0.415263	0.165763
Novel_006296	XLOC_06528.	transcript	0.044579	0.044998	0.045799	0.524462	0.010618	0.026068	0.111177	0.01807	0.106465

Novel_006297	XLOC_06528.	transcript	0.018726	1.0112	0	0	0.546669	0	0.921672	0.552765	0
Novel_006298	XLOC_06529.	transcript	2.965795	2.888592	3.685268	1.998108	2.725666	2.518657	2.949319	3.658979	2.058992
Novel_006299	XLOC_06529.	transcript	0.841188	1.006221	1.400249	2.816589	2.578903	1.072735	2.976409	2.47336	2.884757
Novel_006300	XLOC_06530.	transcript	0.610685	0.373278	0.976868	0.721391	1.181353	1.306869	1.342501	1.368123	1.274052
Novel_006301	XLOC_06530.	transcript	0.88596	0.560711	0.180357	0.818116	0.279902	0	0	0	0
Novel_006302	XLOC_06530.	transcript	0.478331	1.261111	0.328597	0.605208	0.481645	0.392353	0.340424	0.460918	0.53956
Novel_006303	XLOC_06533.	transcript	4.750665	0.801853	3.609993	4.057122	4.304451	3.090931	5.732174	4.711455	3.659035
Novel_006304	XLOC_06533.	transcript	0.320717	0.02802	0.443021	0.439991	0.102464	0.243133	0.170574	0.254693	0.321243
Novel_006305	XLOC_06533.	transcript	0.128749	0.242627	0	0.073722	0.225316	0	0.635365	0.273924	0.088378
Novel_006306	XLOC_06535.	transcript	0.548856	0.322711	0.476188	0.708996	0.707061	0.45576	0.602187	0.555555	0.613475
Novel_006307	XLOC_06535.	transcript	0	0	0	0	0	0	0	0	0
Novel_006308	XLOC_06535.	transcript	0	0	0	0	0	0	0	0.125367	0
Novel_006309	XLOC_06535.	transcript	0	0.562666	0.176465	0.522195	0.264576	0.296086	0.121043	0.366497	0.276435
Novel_006310	XLOC_06535.	transcript	0	0.009057	0.000163	0.081838	0.078312	0	0.024984	0.012513	0.18449
Novel_006311	XLOC_06536.	transcript	0	0.299411	0	0.177251	0	0.265204	0	0.183064	0.011103
Novel_006312	XLOC_06536.	transcript	0.813598	0.370618	0.328371	0.16054	0.13984	0.454377	1.06361	0.222769	0.050118
Novel_006313	XLOC_06536.	transcript	0	0.059416	0	0.255901	0	0	0	0.150093	0.879874
Novel_006314	XLOC_06536.	transcript	0	0.268844	0	0.399696	0.698027	0	0	0.125823	0
Novel_006315	XLOC_06536.	transcript	0.289413	0	0.300223	0.022901	0.272872	0.505668	0.52481	0.032639	0.544611
Novel_006316	XLOC_06536.	transcript	0.587892	0	0.160424	0	0.306008	0.368472	0.68439	0.376296	0.479545
Novel_006317	XLOC_06536.	transcript	0	0	0	0.309349	0	0	0	0.080734	0.076258
Novel_006318	XLOC_06538.	transcript	0	0.10644	0.193948	0.154061	0.057781	0.044357	0.163506	0.042098	0.04357
Novel_006319	XLOC_06538.	transcript	0.336215	0.266228	0.991246	1.00E-05	0.368441	0.945903	0.899649	0.33686	1.05288
Novel_006320	XLOC_06538.	transcript	0	0	0	0	0	0	0	0	0
Novel_006321	XLOC_06538.	transcript	0	0	0	0.024341	0.004254	0.01003	0.006666	0.014023	0.020889
Novel_006322	XLOC_06538.	transcript	0	0	0	0.00196	0	0	0	0	0
Novel_006323	XLOC_06538.	transcript	0	0	0.006977	1.00E-05	0.000205	0	0	3.00E-06	0
Novel_006324	XLOC_06538.	transcript	0.156089	0	0	0	0	0	0	0	0
Novel_006325	XLOC_06538.	transcript	1.045123	0	0.871086	1.801447	1.357322	1.234698	1.449669	1.430132	0.930047
Novel_006326	XLOC_06538.	transcript	0	0	0	0	0.016718	0	0	0	0
Novel_006327	XLOC_06538.	transcript	0.046273	0	0.058949	0.075854	0.03373	0.852268	0.04447	0	0.095807
Novel_006328	XLOC_06538.	transcript	0	0	0	0	0	0	0	0	0.455153
Novel_006329	XLOC_06538.	transcript	0.261315	0	0.310867	0.39161	0.070681	0.093485	0.00762	0.15742	0.267055
Novel_006330	XLOC_06542.	transcript	2.802424	7.102221	1.880758	2.397536	3.131198	2.222228	5.988908	3.900018	4.370368
Novel_006331	XLOC_06542.	transcript	0.311512	1.623465	0.227056	0.142936	0.165195	0.398517	0.244472	0.294981	0.354987
Novel_006332	XLOC_06545.	transcript	0.22017	0.1412	0.064783	0.156451	0.130803	0.183392	0.17606	0.047774	0.144546
Novel_006333	XLOC_06545.	transcript	0.007734	0.291501	0.099304	0.017691	0	0.006383	0.00786	0.015619	0.027521
Novel_006334	XLOC_06545.	transcript	0	0	0	0	0	0	0.007758	0.06818	0

Novel_006335	XLOC_06546.	transcript	0.497231	0.798558	0.545155	0.045506	0.472273	0.09606	0.032664	0.469248	0.248048
Novel_006336	XLOC_06546.	transcript	0	2.111088	0	0	0.437644	0	0.026356	0.551431	1.128081
Novel_006337	XLOC_06546.	transcript	0.012828	0.008926	0.073674	0.36015	0	0.621994	0.410772	0	0
Novel_006338	XLOC_06547.	transcript	1.102939	0.694235	0.995265	0.246482	1.278991	0.97038	0.28604	1.293659	0.797121
Novel_006339	XLOC_06547.	transcript	0.043545	0.190897	0.247466	0.904454	0.164491	0.353798	1.727245	0.21692	0.434067
Novel_006340	XLOC_06548.	transcript	0	0	0	0	0	0	0	0	0.148351
Novel_006341	XLOC_06548.	transcript	0.134356	0.066984	0.237745	0.186497	0.254533	0.202197	0.215295	0.039084	0.191283
Novel_006342	XLOC_06548.	transcript	0.000745	0	0.003126	0.002417	0.025308	0.001937	0.031656	0.007582	0.003838
Novel_006343	XLOC_06548.	transcript	0.004269	0.000296	0.011115	0.020068	0.001217	0.010783	0.002104	0.040548	0.019943
Novel_006344	XLOC_06548.	transcript	0	0.446229	0	0	0	0.010601	0	0.021271	0
Novel_006345	XLOC_06548.	transcript	0	0	0	0	0	0	0	0	0
Novel_006346	XLOC_06549.	transcript	0.318599	0.403967	0.328062	0.404119	0.474167	0.486636	0.527506	0.475527	0.46155
Novel_006347	XLOC_06549.	transcript	0.224027	0.149339	0	0	0	0.193343	0	0.045273	0
Novel_006348	XLOC_06549.	transcript	0.014335	0	0.126668	0.207394	0.100872	0.050841	0.2207	0.156823	0.231326
Novel_006349	XLOC_06550.	transcript	2.126726	4.610843	1.980752	1.524618	3.232672	1.910383	3.050432	3.591598	1.69839
Novel_006350	XLOC_06550.	transcript	0.711551	0.764652	1.129025	1.143714	1.208004	0.818681	1.16294	1.252749	0.923924
Novel_006351	XLOC_06550.	transcript	0	0	0	0.024151	0.051096	0	0	0	0.078642
Novel_006352	XLOC_06550.	transcript	0.563145	0.209373	0.869574	0.961482	1.07475	1.081991	1.422169	0.956708	0.400854
Novel_006353	XLOC_06550.	transcript	0	0	0.002407	0	0.005114	0	0.254572	0.252169	0
Novel_006354	XLOC_06552.	transcript	4.157246	12.24468	10.25357	10.12405	10.43479	8.46236	8.008862	9.577348	7.518998
Novel_006355	XLOC_06552.	transcript	1.326867	0.680783	1.325435	0.449715	1.406999	0.648699	1.695365	0.949279	0.539203
Novel_006356	XLOC_06553.	transcript	8.885645	7.370051	8.404863	6.10237	12.47105	7.014235	17.42594	13.87493	10.09167
Novel_006357	XLOC_06554.	transcript	0.030816	0.024354	0.065032	0.073884	0.048322	0.041245	0.060034	0.098647	0.034825
Novel_006358	XLOC_06555.	transcript	0.187901	0.093069	0	0.068855	0.022111	0.073832	0.074748	0.130096	0.095011
Novel_006359	XLOC_06555.	transcript	0	0.027414	0	0.07166	0	0	0.033439	0	0
Novel_006360	XLOC_06555.	transcript	0.054292	0.008362	0.014349	0.011376	0.018412	0.016376	0	0.019029	0.016086
Novel_006361	XLOC_06555.	transcript	0.126376	0.070786	0.098442	0.01925	0.162905	0.140493	0.065959	0	0.087222
Novel_006362	XLOC_06555.	transcript	0	0	0.164921	0.106246	0	0	0.009919	0	0.026889
Novel_006363	XLOC_06555.	transcript	0	0	0.004029	0	0	5.00E-04	0	0	0
Novel_006364	XLOC_06555.	transcript	0	0.057438	0	0	0	0	0	0	0
Novel_006365	XLOC_06555.	transcript	0	0	0.072879	0.044845	0.162992	0	0.160003	0	0
Novel_006366	XLOC_06563.	transcript	1.481795	0	0.761648	0.433521	0.610608	0.401872	1.086841	0.595223	0.520789
Novel_006367	XLOC_06564.	transcript	0.279372	0.069152	0.052491	0	0.202993	0.35458	0.146981	0.192986	0.04631
Novel_006368	XLOC_06565.	transcript	0.701022	0.389378	0.571894	0.720427	0.759865	0.581864	0.906889	0.510918	0.798302
Novel_006369	XLOC_06566.	transcript	0.418805	0.398049	0.346348	0.571739	0.650936	0.684949	0.709189	0.608058	0.427318
Novel_006370	XLOC_06566.	transcript	0.040334	0.064431	0.201915	0.065316	0.090234	0.18329	0.070314	0.108794	0.101301
Novel_006371	XLOC_06566.	transcript	0.020461	0.556467	0	0	0.371794	0	0.00079	0.514921	0
Novel_006372	XLOC_06566.	transcript	0.282498	0.06396	0.402365	0.4408	0.139305	0.401383	0.731368	0.105969	0.385269

Novel_006373	XLOC_0656€.	transcript	0.188208	0.030525	0.340904	0.278684	0.060435	0.092043	0.106202	0.19107	0.063544
Novel_006374	XLOC_0656€.	transcript	0.00305	0.000508	0.056019	0	0.011598	0.144232	0.264723	0.004841	0
Novel_006375	XLOC_0656€.	transcript	0.001842	0.235958	0.453234	0.043839	0.067275	0.270995	0.387467	0.226974	0.092345
Novel_006376	XLOC_0656€.	transcript	0.754098	0.323916	0.26774	0.337531	0.361385	0.022441	0.24343	0.330199	0.322164
Novel_006377	XLOC_0656€.	transcript	0.676478	0.625636	0.168852	0.661779	0.182038	0	0.181198	0.097117	0.407629
Novel_006378	XLOC_0656€.	transcript	0.483556	0.886649	0.773879	0.125266	0.597171	0.906551	0.747647	0.698443	0.202347
Novel_006379	XLOC_0656€.	transcript	0	0	0.000389	0.224413	2.268203	0.303838	0.387733	0.000492	0.297664
Novel_006380	XLOC_0656€.	transcript	0.000138	0	0	0	0	0	0.281441	0	0
Novel_006381	XLOC_0656€.	transcript	0.335455	0.945386	0.766007	0	0	0	0.324053	1.807886	0
Novel_006382	XLOC_0656€.	transcript	0	0	0	0	0	0	0	0	0
Novel_006383	XLOC_0656€.	transcript	0.060307	0.08025	0.069537	0.006174	0.107637	0	0.108204	0.051787	0
Novel_006384	XLOC_0656€.	transcript	0	0	0	0	0	1.215413	0	0	0
Novel_006385	XLOC_0656€.	transcript	0	0	0	0	0	0.843351	0	0	0
Novel_006386	XLOC_0656€.	transcript	0.113064	0.09506	0	0.077033	0	0.064355	0.160855	0.453496	0.406429
Novel_006387	XLOC_0656€.	transcript	0	0	0	0	0	0	0	0	0
Novel_006388	XLOC_0656€.	transcript	0.078198	1.311195	0	0.013058	0	0.070137	0.19622	0	0.138307
Novel_006389	XLOC_0656€.	transcript	0.100019	0.106165	0.214645	0.053399	0.097922	0.077099	0.0992	0.133599	0.081967
Novel_006390	XLOC_0656€.	transcript	0.040078	0.130767	0.048938	0.023202	0.111613	0.082326	0.060074	0.135106	0.036173
Novel_006391	XLOC_0656€.	transcript	0	0.044375	0	0.028475	0	0	0	0	0
Novel_006392	XLOC_06573.	transcript	0.349655	0.311504	0.028931	0.161111	0.364422	0.32126	0.343457	0.252422	0
Novel_006393	XLOC_06573.	transcript	0.30526	0	0.217384	0	0	0	0	0	0
Novel_006394	XLOC_06573.	transcript	0	0.346481	0.29394	0.491367	0.290916	0.311487	0.458607	0.343767	0.084857
Novel_006395	XLOC_06573.	transcript	0.265008	0.499824	0.4383	0.672834	0.448278	1.012926	0.786369	0.48549	0.644452
Novel_006396	XLOC_06573.	transcript	2.888271	1.67993	0.002964	0.058312	1.961019	0.00246	2.225503	1.939699	0
Novel_006397	XLOC_06573.	transcript	0	0.056044	0	0	0.023042	0	0.10531	0.240152	0
Novel_006398	XLOC_06573.	transcript	0.064921	0.203431	0.220013	0.025777	0.095833	0.224082	0.104567	0.181479	0.339656
Novel_006399	XLOC_06574.	transcript	0.975971	2.005436	0.881765	0.481203	1.051154	0.57659	0.994999	0.828266	0.87737
Novel_006400	XLOC_06574.	transcript	0	0	0	0	0	0	0	0	0
Novel_006401	XLOC_0657€.	transcript	0.130313	0	0.106541	0.196444	0	0	0.214153	0.089533	0.061775
Novel_006402	XLOC_0657€.	transcript	1.272752	1.95113	1.37165	1.715291	1.302977	1.915043	1.401135	1.246024	1.347761
Novel_006403	XLOC_06577.	transcript	1.17931	1.24581	3.172175	4.853959	3.931515	3.797131	2.230867	3.121924	2.115681
Novel_006404	XLOC_06577.	transcript	0	0	0	0	0	0	0	0	0
Novel_006405	XLOC_06577.	transcript	0.675518	0.510661	0.816728	1.021395	0.709335	0.759076	0.992125	0.901865	0.545281
Novel_006406	XLOC_0657€.	transcript	0.225859	0.317989	0.374676	0.412941	0.513885	0.579835	0.501623	0.379042	0.434316
Novel_006407	XLOC_0657€.	transcript	0	0	0	0.078881	0	0	0	0	0
Novel_006408	XLOC_0657€.	transcript	0.059069	1.362589	0.031329	0.086926	0.060617	0.098263	0.110813	0.008483	0.052679
Novel_006409	XLOC_0657€.	transcript	0.317759	0.135887	0.132553	0.067953	0.283981	0.365949	0.044674	0.141101	0.189908
Novel_006410	XLOC_0657€.	transcript	0	0	0	0	0	0	0	0.016068	0

Novel_006411	XLOC_06581.	transcript	0	0	0	0.025567	0	0.01242	0.005522	0.006055	0
Novel_006412	XLOC_06581.	transcript	0	0	0.47359	0	0	0.197552	0.124497	0.299239	0
Novel_006413	XLOC_06581.	transcript	0	0	0.746932	1.214559	0.42652	0.391141	0.355315	0.369125	0.089809
Novel_006414	XLOC_06581.	transcript	0.006384	0	0	1.125071	0.456484	0.158371	0.327466	0.333464	0.056512
Novel_006415	XLOC_06581.	transcript	0	0	0	1.963269	1.045074	0.295025	0.286792	0.130128	0
Novel_006416	XLOC_06581.	transcript	0	1.636588	0.021424	0.048866	0	0.035813	0.037987	0.418003	0.627241
Novel_006417	XLOC_06583.	transcript	0.026255	0.557616	0.013784	0	0.013654	0.040812	0.049921	0.015715	0.047369
Novel_006418	XLOC_06584.	transcript	0.116717	0.483056	0.083462	0.008905	0.054333	0.006409	0.041243	0.037958	0.078193
Novel_006419	XLOC_06584.	transcript	0	0.167478	0	0	0	0	0	0	0
Novel_006420	XLOC_06586.	transcript	0.515267	0.800287	0.348586	0.618092	0.52995	0.308181	0.658284	0.589182	0.376783
Novel_006421	XLOC_06589.	transcript	0.458759	0.572683	0.503031	0	0.000634	0.005603	0.038352	0.015794	0
Novel_006422	XLOC_06589.	transcript	0	0.024892	0	0.633689	0.569615	0.573932	0.540293	0.503344	0.669735
Novel_006423	XLOC_06589.	transcript	0	0	0	0	0	0	0.199583	0	0
Novel_006424	XLOC_06589.	transcript	0	0.579181	0.072615	0.023978	0	0.03814	0.081887	0.014559	0.027149
Novel_006425	XLOC_06589.	transcript	0.130662	0.040661	0	0.165634	0.053717	0	0.167365	0.02594	0
Novel_006426	XLOC_06593.	transcript	0.265011	0.148582	0.469638	0.16223	0.412135	0.22682	0.48851	0.439481	0.465271
Novel_006427	XLOC_06593.	transcript	0.017122	0.418382	0	0.248754	0.180719	0.378862	0.123188	0.027941	0
Novel_006428	XLOC_06593.	transcript	0	0	0.281119	0	0.201568	0.34199	0.434132	0.18479	0.240232
Novel_006429	XLOC_06593.	transcript	0.544459	0.88732	0.070923	0.070415	0.088813	0	0.014958	0.156567	0.553723
Novel_006430	XLOC_06593.	transcript	0	0	0	0	0	0	0	0	0
Novel_006431	XLOC_06593.	transcript	0	0	0.000713	0.077595	0.121586	0.133075	0.016252	0.176796	0
Novel_006432	XLOC_06593.	transcript	0.296781	0.01684	0.088587	0	0	0	0.042318	0	0.097969
Novel_006433	XLOC_06593.	transcript	0	0	0.001525	0.000293	0.773739	0.640298	0.751675	0.014498	0
Novel_006434	XLOC_06593.	transcript	0	0	0.637213	0.822874	0	0	0	0.763214	0
Novel_006435	XLOC_06595.	transcript	0	0.089194	0.041718	0.044948	0.110308	0	0.040244	0.013604	0
Novel_006436	XLOC_06598.	transcript	0.438866	0.80803	0.67787	0.776548	0.724378	0.706094	0.801477	0.693951	0.562694
Novel_006437	XLOC_06598.	transcript	0	0.009822	0	0.245637	0.427083	0.325303	0.397322	0.322764	0.54359
Novel_006438	XLOC_06598.	transcript	0.461732	0	0.324416	0	0	0	0.27905	0.139108	0
Novel_006439	XLOC_06599.	transcript	0	0	0	0	0	0	0	0	0
Novel_006440	XLOC_06599.	transcript	0.350168	0.189426	0.648178	0.534149	0.413991	0.402099	0.624832	0.509658	0.593669
Novel_006441	XLOC_06600.	transcript	0.07662	0.107112	0.080801	0.297598	0.268456	0.376432	0.157208	0.109222	0.201544
Novel_006442	XLOC_06600.	transcript	0.02993	0.073266	0.047212	0.229823	0.176487	0.368811	0.296513	0.043036	0.038895
Novel_006443	XLOC_06600.	transcript	0	0	0.027519	0	0.125773	0.053654	0.054928	0.018184	0.051881
Novel_006444	XLOC_06600.	transcript	0.051244	0.028923	0.211919	0.023018	0.03648	0.041473	0.311788	0.10971	0.308841
Novel_006445	XLOC_06600.	transcript	0	0	0	0	0	0	0	0.300227	0.244543
Novel_006446	XLOC_06600.	transcript	0	0	0	0	0	0	0	0.255906	0
Novel_006447	XLOC_06600.	transcript	0.190657	0.409234	0.292027	0	0.219264	0.279844	0.277514	0.306136	0.062996
Novel_006448	XLOC_06600.	transcript	0	0	0	0	0	0	0	0	0

Novel_006449	XLOC_06600.	transcript	0.008276	0.264414	0.27909	0.122469	0.066404	0.024577	0.184459	0.01418	0
Novel_006450	XLOC_06600.	transcript	0.386123	0.399676	0	0.388071	0	0	0	0	0
Novel_006451	XLOC_06600.	transcript	0.688182	0.123669	0.548374	0.512532	0.667017	0.307103	0.596161	0.422838	0.639072
Novel_006452	XLOC_06603.	transcript	0.524565	0.420487	0.370812	0.431047	0.360006	0.27512	0.632597	0.325804	0.338921
Novel_006453	XLOC_06603.	transcript	0.270112	0.900549	0.379414	0	0	0.484529	0.640251	0.200138	0
Novel_006454	XLOC_06603.	transcript	0.319106	0.825033	0.188085	0.072073	0.295641	0.008416	0.28778	0.4287	0.13686
Novel_006455	XLOC_06603.	transcript	0.002175	0.005839	0	0.035668	0.000397	0	0.004952	0.001138	0
Novel_006456	XLOC_06603.	transcript	0.082996	0.053035	0.010791	0.280974	0.09688	0.002482	0.090677	0.087601	1.92212
Novel_006457	XLOC_06603.	transcript	1.301963	0.832061	1.036983	1.715762	1.632075	1.64143	1.331644	2.101007	0.456462
Novel_006458	XLOC_06603.	transcript	0	0	1.288951	0.736455	0.593619	0.834356	1.361139	0	0
Novel_006459	XLOC_06603.	transcript	0.214863	0.009627	0	0	0	0	1.677791	0	0
Novel_006460	XLOC_06603.	transcript	0	0.364235	0	0	0	0	0	0.074892	0.846267
Novel_006461	XLOC_06603.	transcript	0.442322	2.998125	4.002708	0.555086	3.471905	4.685238	2.612339	5.243945	5.330569
Novel_006462	XLOC_06603.	transcript	0.173482	0.445385	0	0	0.300969	0.810845	0	0.276672	0.526546
Novel_006463	XLOC_06603.	transcript	1.282554	0	0.501678	1.265277	0.384464	0.360824	0.651958	0.480019	0.385054
Novel_006464	XLOC_06603.	transcript	1.157319	0.124186	0.444091	3.513461	0.518123	0.083144	0.188961	0.020608	1.480148
Novel_006465	XLOC_06603.	transcript	0.257822	0.062282	0	0	0.158597	0.037253	0	0.06514	0.357188
Novel_006466	XLOC_06603.	transcript	1.413428	0.166572	0	1.065981	0.834424	0.353265	0.269048	0.592031	0.619638
Novel_006467	XLOC_06604.	transcript	0.026372	0.125532	0.092007	0.037767	0.026973	0	0.090446	0.101758	0.176492
Novel_006468	XLOC_06604.	transcript	0.059183	0.08969	0.148759	0.111801	0.041733	0.08775	0.193625	0.215755	0.438641
Novel_006469	XLOC_06604.	transcript	0	0	0	0	0	0	0	0	0
Novel_006470	XLOC_06604.	transcript	0.281602	0.188967	0.175678	0.292451	0.404797	0.260756	0.271122	0.305603	0
Novel_006471	XLOC_06604.	transcript	0.283519	0.102987	0.199561	0.19878	0.246969	0.18153	0.44714	0.275785	0.237498
Novel_006472	XLOC_06606.	transcript	0	0	0.007887	0	0	0.023758	0	0	0
Novel_006473	XLOC_06606.	transcript	0.143659	0.302004	0.406212	0.045712	0.08943	0	0.067261	0.116091	0.034821
Novel_006474	XLOC_06606.	transcript	0.315258	0.276507	0	0.337956	0.483423	0.283064	0.706211	0.48274	0.32508
Novel_006475	XLOC_06608.	transcript	0.138073	0.278495	0.692169	0	1.324303	0.001148	0	0	0
Novel_006476	XLOC_06608.	transcript	0.918611	0	0.001201	0.352544	0.011382	0.960605	0	0	0.813589
Novel_006477	XLOC_06608.	transcript	0	0	0	0.823713	0	0.779913	0.765967	0	0.523581
Novel_006478	XLOC_06615.	transcript	0.297509	0.198817	0.301567	0.11105	0.244864	0.39647	0.874833	0.748241	0.371638
Novel_006479	XLOC_06615.	transcript	1.013036	0.703868	0.969228	1.029858	1.084861	1.0098	0.614847	0.5959	0.795817
Novel_006480	XLOC_06615.	transcript	0	0	0	0	0	0	0	0	0
Novel_006481	XLOC_06618.	transcript	0.059167	0.003228	0.178881	0	0.149333	0	0	0	0
Novel_006482	XLOC_06618.	transcript	0.102123	0	0.19454	0.118252	0.118701	0.276811	0.102911	0.168287	0.000639
Novel_006483	XLOC_06618.	transcript	0	0.006201	0	0.001358	0.206551	0	0	0.001334	0
Novel_006484	XLOC_06618.	transcript	0	0.396303	0	0	0	0	0	0	0
Novel_006485	XLOC_06618.	transcript	0.210376	0.108363	0	0	0	0.001871	0.002131	0	0.000964
Novel_006486	XLOC_06618.	transcript	0.095767	0	0.26248	0.476025	0.172327	0.469782	0.479613	0.534973	0.741927

Novel_006487	XLOC_0661[illegible]	transcript	0.03	0.005353	0.110079	0	0.047957	0	0.090508	0	0.001323
Novel_006488	XLOC_0661[illegible]	transcript	0	0.007748	0	0.004854	0	1.40E-05	0	0.00304	0.009553
Novel_006489	XLOC_0661[illegible]	transcript	0.219594	0.03304	0.165544	0	0.150434	0.085995	0.064319	0.103007	0.08661
Novel_006490	XLOC_0661[illegible]	transcript	0.239044	0	0	0.128114	0	0	0.64003	0	0
Novel_006491	XLOC_0664[illegible]	transcript	0.408506	1.317351	0.499071	0.354473	0.272866	0.343287	0.54508	0.276015	0.468307
Novel_006492	XLOC_0664[illegible]	transcript	0	0.033244	0	0.004537	0	0	0.026535	0	0
Novel_006493	XLOC_0665[illegible]	transcript	0	0	0	0	0	0	0.483076	0	0
Novel_006494	XLOC_0665[illegible]	transcript	0.520116	0	0.380584	0	0	0	0	0.018066	0
Novel_006495	XLOC_0665[illegible]	transcript	0	0	0	0	0	0	0	0	0.444278
Novel_006496	XLOC_0665[illegible]	transcript	0	0.007995	0	0	0.003682	0.003571	0	0	0
Novel_006497	XLOC_0665[illegible]	transcript	0.019961	0.694851	0.016147	0.44338	0.547904	0.66102	0.903798	0.778769	0
Novel_006498	XLOC_0665[illegible]	transcript	0	0	0	0.004212	0	0	0	0	0.015273
Novel_006499	XLOC_0667[illegible]	transcript	0.38021	0.378546	0.493521	0.356365	0.449806	0.38409	0.306054	0.241332	0.359069
Novel_006500	XLOC_0667[illegible]	transcript	0.122192	0.080581	0.096831	0.120563	0.190895	0.129795	0.168581	0.116208	0.068147
Novel_006501	XLOC_0667[illegible]	transcript	0.152048	0.075435	0.242607	0.237166	0.167507	0.26107	0.194643	0.469364	0.206213
Novel_006502	XLOC_0668[illegible]	transcript	0.542738	0.482578	0.025816	0.212306	0.619538	0.701582	0.713274	0.427746	1.169349
Novel_006503	XLOC_0668[illegible]	transcript	0.025216	0.141101	0.36077	0.130655	0.063855	0.00896	0.017326	0.019373	0.025806
Novel_006504	XLOC_0668[illegible]	transcript	0.603865	1.749386	0.277614	0.631055	0.466247	1.31016	0.425691	0.814021	0.938573
Novel_006505	XLOC_0668[illegible]	transcript	0	1.169844	0.770961	0	0.055229	0	0.866662	0	0
Novel_006506	XLOC_0670[illegible]	transcript	1.296043	0	0.507943	0.350725	0.606958	0.821143	0.610028	0	0.720416
Novel_006507	XLOC_0670[illegible]	transcript	0.084207	0.129018	0.120963	0.006256	0.04731	0.035211	0.065496	0.816494	0.077843
Novel_006508	XLOC_0670[illegible]	transcript	0.012609	0	0.010437	0	0.018548	0.001401	0.005415	0.001572	0.003721
Novel_006509	XLOC_0670[illegible]	transcript	0.327421	0.104812	0.182578	0.270103	0.192585	0.22512	0.281516	0.154567	0.113609
Novel_006510	XLOC_0670[illegible]	transcript	0.09355	0.095799	0.247314	0.024277	0.17649	0.198344	0.206159	0.523672	0.146301
Novel_006511	XLOC_0670[illegible]	transcript	0.248716	0.046728	0.040456	0.127102	0.10636	0.189848	0.390683	0.093347	0.031891
Novel_006512	XLOC_0670[illegible]	transcript	0.263784	0.253714	0.218436	0.213608	0.574416	0.414973	0.457989	0.356124	0.324274
Novel_006513	XLOC_0670[illegible]	transcript	0	0	0	0	0	0	0.004621	0	0
Novel_006514	XLOC_0670[illegible]	transcript	0	0	0.206054	0	0	0	0.166393	0	0
Novel_006515	XLOC_0670[illegible]	transcript	0.045452	0.083541	0.175819	0.026062	0.126424	0.157986	0.126663	0.227733	0.018052
Novel_006516	XLOC_0670[illegible]	transcript	0	0.245567	0	0.051681	0	0.262229	0	0.303427	0
Novel_006517	XLOC_0671[illegible]	transcript	1.527759	0.39572	0.584895	0.666847	0.622722	0.980727	1.951762	0.419944	1.656959
Novel_006518	XLOC_0671[illegible]	transcript	0	0.536908	0.082576	0.046779	0.311483	0.262391	0	0.902128	0.130682
Novel_006519	XLOC_0672[illegible]	transcript	0.438579	0.226081	0.792797	1.186733	1.086104	1.61257	0.792289	1.251262	0.965791
Novel_006520	XLOC_0672[illegible]	transcript	0.01485	0.816471	0	0	0.022428	0	0.009051	0	0
Novel_006521	XLOC_06731.	transcript	0	0.160557	0	0	0	0	0	0.102592	0.26721
Novel_006522	XLOC_06731.	transcript	0.116905	0.216527	0	0.043061	0	0	0.162274	0.092313	0
Novel_006523	XLOC_06731.	transcript	0.105214	0.156085	0.224754	0.044276	0	0.001006	0.10714	0.07765	0.020759
Novel_006524	XLOC_06731.	transcript	0.155617	0.274197	0	0.049796	0.167147	0.162832	0.111656	0.138549	0

Novel_006525	XLOC_06731.	transcript	0.101902	0.240793	0	0.05169	0.038413	0.208571	0.105114	0.13141	0
Novel_006526	XLOC_06732.	transcript	0	0.247351	0	0.000652	0	0	0	0.077228	0
Novel_006527	XLOC_06732.	transcript	0.550626	0.121224	0.456387	0.375066	0.489562	0.450303	0.887367	0.428197	0.321019
Novel_006528	XLOC_06743.	transcript	0.469749	0	0.249092	0.692824	0.61643	0.007105	0.474815	0.500991	0
Novel_006529	XLOC_06743.	transcript	0.652306	0	0	0.621504	0.2994	1.070203	0.3366	0.60777	0.562367
Novel_006530	XLOC_06743.	transcript	0.022727	0.111256	0.284707	0	0.004618	0.013531	0.367535	0	0.006686
Novel_006531	XLOC_06744.	transcript	0.039621	0	0.017957	0	0.114088	0	0.309216	0.023719	0.343026
Novel_006532	XLOC_06791.	transcript	0.440907	0	0.245464	0.293611	0.212612	0.217261	0.552711	0.158863	0.128162
Novel_006533	XLOC_06791.	transcript	0	0	0.058646	0.1613	0.206541	0.054305	0	0.397652	0.032818
Novel_006534	XLOC_06792.	transcript	0	0.039379	0.015364	0	0.075506	0	0	0	0
Novel_006535	XLOC_06794.	transcript	0.012103	0	0.067115	0.03542	0.004423	0.080979	0.014646	0.00525	0.009311
Novel_006536	XLOC_06794.	transcript	0.30744	0.663845	0.043018	0.056171	0.081053	0.063819	0.273949	0.050579	0.296312
Novel_006537	XLOC_06794.	transcript	0	0.352139	0.047296	0	0	0.047709	0.043454	0	0
Novel_006538	XLOC_06796.	transcript	0.368068	0.033949	0	0.297699	0	0	0	0.491752	0.152091
Novel_006539	XLOC_06796.	transcript	0	0	0	0	0	0	0	0	0
Novel_006540	XLOC_06796.	transcript	0.077317	0.024229	0.178983	0.457473	0.259535	0.140902	0.353539	0.194511	0.292849
Novel_006541	XLOC_06797.	transcript	0.198045	0.107204	0.520048	0.338143	0.283562	0.222566	0.273965	0.288436	0.484352
Novel_006542	XLOC_06798.	transcript	0.003132	0.004672	0	0.226243	0.047889	0.04812	0.04924	0.068096	0.055676
Novel_006543	XLOC_06798.	transcript	0.030672	0.035462	0	0.029047	0	0.016942	0.006581	0.008361	0
Novel_006544	XLOC_06798.	transcript	0.162571	0.614394	0.419011	0.328867	0.35413	0.37458	0.411116	0.149927	0.46454
Novel_006545	XLOC_06798.	transcript	0.446896	1.273203	0.34107	0.131483	0.093423	0.3891	1.132291	0.702288	0.514224
Novel_006546	XLOC_06798.	transcript	0.504655	0.079279	0.478946	0.475312	0.531485	0.327002	0.066148	0.096158	0.014192
Novel_006547	XLOC_06804.	transcript	0	0	0	0.020439	0	0.02006	0.163807	0.033375	0.159239
Novel_006548	XLOC_06826.	transcript	0	0	0	0	0	0.012739	0.932076	0	0
Novel_006549	XLOC_06826.	transcript	0.422713	0.605664	0.454971	0.431191	0.260159	0.527815	0.344133	0.24922	0.367536
Novel_006550	XLOC_06826.	transcript	0.302432	0.531852	0.452718	0.515095	0.836656	0.56228	0	0.493078	0.386249
Novel_006551	XLOC_06828.	transcript	0	0.735918	0	0	0	0	0	0	0
Novel_006552	XLOC_06828.	transcript	0	0	0	0.206541	0	0	0	0.167651	0
Novel_006553	XLOC_06837.	transcript	0.160832	0.287161	0.133521	0.208515	0.204113	0.213003	0.173285	0.187359	0.222392
Novel_006554	XLOC_06837.	transcript	0.760537	0.936495	0.426823	0.440139	0.501588	0.517044	0.678254	0.848807	0.776583
Novel_006555	XLOC_06837.	transcript	0.382174	0.961877	1.080166	0	0.030054	0.629167	0.714684	0.038422	0.026571
Novel_006556	XLOC_06837.	transcript	0.611365	0.925816	0.23073	0.174258	0.2058	0.485507	0.224843	0.280384	0.28765
Novel_006557	XLOC_06843.	transcript	0.490885	0	0.036807	0.470101	0.015379	0.364558	0.18491	0.415834	0.087618
Novel_006558	XLOC_06843.	transcript	0.145927	0.719523	0.438099	0.121556	0.484352	0.055051	0.502878	0.124873	0.487242
Novel_006559	XLOC_06845.	transcript	0.26107	1.668501	0.071456	0	0.054013	0.126565	0.297366	0.114804	0.73635
Novel_006560	XLOC_06845.	transcript	0.118716	0	0	0	0	0	0	0	0
Novel_006561	XLOC_06856.	transcript	0.652759	0.266289	0.459505	0.414742	0.458363	0.424242	0.54714	0.300821	0.26929
Novel_006562	XLOC_06856.	transcript	0	0.034438	0.008233	0	0.006287	0.010906	0	0.257466	0

Novel_006563	XLOC_06856.	transcript	0.163295	0.247257	0.069352	0.045662	0.03592	0.17062	0.059377	0.072176	0.131501
Novel_006564	XLOC_06856.	transcript	0.048257	0.177966	0.219465	0.031657	0	0.095238	0.135556	0.018251	0.107919
Novel_006565	XLOC_06859.	transcript	0.082	0.159832	0.071536	0.052507	0.107834	0.116446	0.151467	0.086669	0.033581
Novel_006566	XLOC_06869.	transcript	0	0	0	0	0	0	0.282009	0	0
Novel_006567	XLOC_06871.	transcript	0.220992	0.211066	0.090061	0.28334	0.304444	0.236572	0.360094	0.262993	0.228822
Novel_006568	XLOC_06874.	transcript	1.816206	1.349077	1.191908	1.503443	3.430181	1.743482	1.78569	1.749683	2.877583
Novel_006569	XLOC_06874.	transcript	0.048182	2.520427	1.472678	2.401569	0.011484	1.224924	1.664837	1.68919	0
Novel_006570	XLOC_06874.	transcript	0.012512	0.058916	0	0.041947	0	0.075483	0.020948	0.019725	0.074094
Novel_006571	XLOC_06876.	transcript	0.30558	0	0	0.000385	0.000774	0	0.009185	0.472206	0.000833
Novel_006572	XLOC_06876.	transcript	1.009423	0.561909	0.796221	0.910851	0.950307	0.788694	1.266126	0.818802	0.635392
Novel_006573	XLOC_06876.	transcript	0.20883	0.838381	0.770057	0.554693	0.661123	0.776288	0.717161	0.625676	0.519386
Novel_006574	XLOC_06876.	transcript	0.488938	0	0	0.22356	0	0	0	0.519629	0
Novel_006575	XLOC_06880.	transcript	1.608298	0	1.220082	1.626692	0.28871	1.51358	1.442801	1.437719	2.59354
Novel_006576	XLOC_06884.	transcript	0.506698	0.217169	0	0.162721	0.254892	0.145004	0.573832	0.513212	0.407514
Novel_006577	XLOC_06884.	transcript	0.043105	0.003095	0.01123	0.0037	0.146271	0.02137	0.029805	0.113206	0.039422
Novel_006578	XLOC_06884.	transcript	0.05167	0.413121	0.47563	0.60045	0.417033	0.563728	0.498587	0.505622	0.303935
Novel_006579	XLOC_06884.	transcript	0.001062	0	0.017982	0.173014	0.004778	0.212311	0	0.019333	0.008395
Novel_006580	XLOC_06885.	transcript	3.468477	0	0	0.238485	0.324498	1.454915	0.57812	0.955979	0.763144
Novel_006581	XLOC_06885.	transcript	0	0	0	0	0	0	0	0	0
Novel_006582	XLOC_06885.	transcript	0	0	0	0	0	0	0	0	0
Novel_006583	XLOC_06885.	transcript	6.365536	0	0	0	0.181089	0	0	0	0
Novel_006584	XLOC_06885.	transcript	0	0	0.725095	0.099332	0.001632	0.700226	0.621249	1.364922	0.372292
Novel_006585	XLOC_06885.	transcript	0	0.001746	0	0	0	0	0	0	0
Novel_006586	XLOC_06885.	transcript	0	0	0	0.351951	5.028255	0	0.600641	0	0
Novel_006587	XLOC_06885.	transcript	0.028423	0.053534	0.344114	0.004326	0	0.01462	0	0.013561	0.008693
Novel_006588	XLOC_06889.	transcript	0	0	0	0	0	0.009901	0.007666	0.021941	0.009089
Novel_006589	XLOC_06889.	transcript	0	0	0	0	0	0	0	0	0
Novel_006590	XLOC_06889.	transcript	0	0	0	0	0	0	0	0	0
Novel_006591	XLOC_06889.	transcript	0.176797	0.041907	0.07823	0.061886	0.06553	0.082892	0.09502	0	0.805614
Novel_006592	XLOC_06889.	transcript	0	0	0	0	0	0	0	0	0.070519
Novel_006593	XLOC_06889.	transcript	0	0	0	0	0	0	0	0	0
Novel_006594	XLOC_06890.	transcript	0.168525	0	0.306808	0.23055	0.264475	0.094126	0.226693	0.263854	0.030724
Novel_006595	XLOC_06891.	transcript	1.294032	0.266575	1.613271	1.41459	1.597569	1.67017	1.120817	1.297911	1.529304
Novel_006596	XLOC_06891.	transcript	0.103119	0	0.784251	0.443517	0.061886	0.731062	0.559646	0.711027	1.026167
Novel_006597	XLOC_06891.	transcript	0.006665	0.006161	0	0.000199	0.544083	0.403105	0.308188	0.484504	0
Novel_006598	XLOC_06891.	transcript	0.232813	0.204727	0	0.686064	0	0	0	0	0
Novel_006599	XLOC_06894.	transcript	0.007055	0	0.132094	0.194602	0	0.306934	0.388849	0.002786	0.118771
Novel_006600	XLOC_06894.	transcript	0	0	0.065096	0	0	0	0.073013	0	0.084927

Novel_006601	XLOC_06894	transcript	0.215152	0.279866	0.196569	0.437092	0.152062	0.475443	0.225593	0.161293	0.296986
Novel_006602	XLOC_06899	transcript	0.095484	0.578224	0.090413	0	0	0.090985	0.463459	7.00E-06	0
Novel_006603	XLOC_06899	transcript	0.41175	0.148495	0.320552	0.340574	0.435882	0.210372	0.286118	0.357687	0.313585
Novel_006604	XLOC_06899	transcript	0	0	0	0.12889	0	0	0	0	0
Novel_006605	XLOC_06899	transcript	0	0.287058	0	0	0	0	0.389612	2.50E-05	0
Novel_006606	XLOC_06899	transcript	0.349128	0.19838	0.204485	0.079687	0.162183	0.198238	0.107153	0.149078	0.11901
Novel_006607	XLOC_06914	transcript	0.095869	0.230416	0.074557	0.079384	0.156991	0.040972	0.122019	0.133325	0
Novel_006608	XLOC_06918	transcript	0.413699	0.458241	0.365396	0.32874	0.230718	0.327134	0.530545	0.33949	0.535437
Novel_006609	XLOC_06918	transcript	0.256177	0.063714	0.07221	0.14674	0.196	0.123716	0.216324	0.170711	0.138906
Novel_006610	XLOC_06920	transcript	0	0.122995	0	0.04282	0	0.101897	0.037643	0.144067	0.117889
Novel_006611	XLOC_06921	transcript	1.152941	0.755372	0.777128	0.251965	0.444364	0.453571	0.981172	0.689238	0.713921
Novel_006612	XLOC_06921	transcript	0.00053	0.024768	0.008831	0.317997	0.282601	0.429438	0	0.165188	0
Novel_006613	XLOC_06921	transcript	0.306711	0.017835	0	0.007117	0.465806	0.36673	0.006395	0.131692	0
Novel_006614	XLOC_06921	transcript	0.801175	0.985381	1.179155	1.324337	0.391865	1.287807	0.880782	1.730854	0.880428
Novel_006615	XLOC_06921	transcript	0.476058	0.998947	0.273779	0.251994	0.713178	0	0.625336	0.747106	0.325035
Novel_006616	XLOC_06921	transcript	0.106996	0.130484	0.562446	0.243382	0.077042	0.736015	0.477611	0.049938	0.414601
Novel_006617	XLOC_06921	transcript	1.695712	1.101646	1.477057	1.725584	1.445566	1.343129	2.258883	1.297034	1.046171
Novel_006618	XLOC_06925	transcript	0.284127	0.888684	0.218056	0.135871	0.191365	0.142547	0.654274	0.223799	1.40271
Novel_006619	XLOC_06927	transcript	0.308293	0.040513	0.316167	0.252243	0.495505	0.270553	0.548666	0.273136	0.185151
Novel_006620	XLOC_06932	transcript	0	0.147927	0	0.020767	0	0.009688	0.011388	0.003175	0.009948
Novel_006621	XLOC_06932	transcript	0	1.101623	0	0	0	0	0	0	0
Novel_006622	XLOC_06932	transcript	0	0.167289	0.031184	0	0	0.014312	0	0	0.112588
Novel_006623	XLOC_06932	transcript	0.129284	0.398766	0.209768	0.197001	0.115968	0.137349	0.253978	0.186075	0.152379
Novel_006624	XLOC_06933	transcript	0	0.57073	0	0	0.042708	0	0	0	0
Novel_006625	XLOC_06933	transcript	0.499442	0.141848	1.129708	0.479114	0.857812	1.332061	1.09596	1.151076	0.702041
Novel_006626	XLOC_06933	transcript	0.47173	0	0.31604	0.700536	0.188999	0.287603	0.734044	0.568947	0.234562
Novel_006627	XLOC_06933	transcript	0.739317	1.62597	0.771053	0.618451	0.695908	0.480836	0.825997	0.689092	0.521195
Novel_006628	XLOC_06940	transcript	0.645463	0.772339	0.520917	0.561851	0.557008	0.647314	0.581338	0.688458	0.528224
Novel_006629	XLOC_06940	transcript	0	0.074749	0.057617	0.05717	0.142839	0	0.223188	0.054728	0.13643
Novel_006630	XLOC_06940	transcript	0	0	0.051997	0	0	0	0.115645	0	0
Novel_006631	XLOC_06940	transcript	0.226587	0.09385	0.044763	0.063589	0	0	0.189653	0.055589	0
Novel_006632	XLOC_06940	transcript	0.083543	0.050899	0.0359	0.041716	0	0	0.045489	0.057596	0
Novel_006633	XLOC_06940	transcript	0.072932	0.044025	0.034842	0.036341	0	0	0.045457	0.069901	0
Novel_006634	XLOC_06940	transcript	0.091545	0.039394	0.060431	0.035248	0.081833	0.089146	0.069187	0.062782	0
Novel_006635	XLOC_06940	transcript	0.126115	0	0.06328	0.036215	0.074848	0.121733	0.071311	0.065472	0
Novel_006636	XLOC_06940	transcript	0	0.046483	0.063417	0.037413	0.068581	0	0.074377	0.071544	0
Novel_006637	XLOC_06940	transcript	0.129381	0.046646	0.063488	0.03641	0.073271	0.130269	0.071509	0.066739	0.07898
Novel_006638	XLOC_06940	transcript	0	0.046183	0.062113	0.03673	0.078346	0.128981	0.072732	0.06785	0.074219

Novel_006639	XLOC_0694C .	transcript	0	0.044466	0.061669	0.036498	0.081285	0	0.072237	0.060976	0
Novel_006640	XLOC_0694C .	transcript	0	0.046971	0.063403	0.036006	0.068993	0	0.073846	0.069814	0.07709
Novel_006641	XLOC_0694C .	transcript	0	0.037	0.058922	0.068248	0.18147	0.31662	0.074678	0.129447	0
Novel_006642	XLOC_0694C .	transcript	0.643113	0	0	0	0	0	0	0	0
Novel_006643	XLOC_0694C .	transcript	0	0.029529	0.069614	0.062678	0.072346	0	0.083238	0.076389	0.101483
Novel_006644	XLOC_0694C .	transcript	0.150179	0.022488	0.103113	0.064604	0.086566	0.122178	0.167766	0.091865	4.00E-05
Novel_006645	XLOC_0694C .	transcript	0.187171	0.041314	0.104317	0.064269	0.08052	0.161696	0.171341	0.104652	0.093926
Novel_006646	XLOC_0694C .	transcript	0.011802	0.020503	0.102671	0.066119	0.083933	0	0.170491	0.093114	0.09294
Novel_006647	XLOC_0694C .	transcript	0	0	0	0	0	0	0	0	0
Novel_006648	XLOC_06941 .	transcript	0	0.140757	0	0	0	0	0.282967	0.868814	0
Novel_006649	XLOC_06941 .	transcript	0.607553	0.45586	0.446932	0.54162	0.504706	0.548943	0.462455	0.039019	0.52589
Novel_006650	XLOC_06944 .	transcript	0.210938	0.590351	0.102748	0.118507	0.057497	0.119958	0.230962	0.030736	0.291156
Novel_006651	XLOC_06944 .	transcript	0	0	0	0	0	0	0	0.240117	0
Novel_006652	XLOC_06944 .	transcript	0.480227	0.043538	0.55985	0.270257	0.604261	0.246056	0.804018	0.30607	0.100755
Novel_006653	XLOC_0695C .	transcript	0.459034	0.321866	0.150813	0.333253	0.674369	0.53073	1.07009	0.93362	0.579876
Novel_006654	XLOC_06951 .	transcript	0.864248	1.216712	1.726086	1.482355	1.518457	2.16755	1.474604	1.307803	1.59861
Novel_006655	XLOC_0695E .	transcript	0	3.090739	0	0	0	0	0	0	0
Novel_006656	XLOC_0695E .	transcript	3.528765	6.093605	7.767758	9.348067	9.895172	10.97275	11.24369	11.2713	8.982055
Novel_006657	XLOC_0696C .	transcript	0	0.06252	0.150083	0.104625	0	0	0.165047	0.00087	0
Novel_006658	XLOC_0696C .	transcript	0.053653	0.147684	0.04673	0.064195	0.126508	0.071996	0	0	0.023499
Novel_006659	XLOC_0696C .	transcript	0.286234	0.246721	0.109091	0.113711	0.163372	0.366325	0.310991	0.503768	0.156943
Novel_006660	XLOC_06963 .	transcript	0.12105	0.248507	0.255811	0.298433	0.3103	0.291637	0.258517	0.301987	0.082143
Novel_006661	XLOC_06963 .	transcript	0	0.110252	0.078007	0.015553	0.198188	0.028894	0.345486	0.009251	0.358246
Novel_006662	XLOC_06964 .	transcript	1.844532	1.029365	1.272102	1.297864	1.598602	1.084456	2.240119	2.025706	1.221249
Novel_006663	XLOC_06964 .	transcript	0.150067	0	0	0	0.185087	0.161694	0.166148	0	0.214035
Novel_006664	XLOC_0696E .	transcript	0	0.04524	0	0.247672	0.036919	0	9.450621	0.078651	0.053246
Novel_006665	XLOC_0696E .	transcript	0	0	0	5.580999	0	0	0	12.64622	8.83841
Novel_006666	XLOC_0696E .	transcript	6.818839	0	10.13855	4.433647	6.662276	10.22844	0.004596	0	0
Novel_006667	XLOC_0696E .	transcript	0.139816	4.213517	0	0	1.153619	0	0.04201	0	0.249201
Novel_006668	XLOC_06967 .	transcript	0.013833	0.441767	0.389174	0.450508	0.084161	0.335173	0.214626	0.150325	0.068052
Novel_006669	XLOC_06967 .	transcript	0.661355	0.770054	0.386537	0.30647	0.752539	0.247517	0.703176	0.512323	0.433971
Novel_006670	XLOC_0696E .	transcript	0.528846	0	0	0.096145	7.00E-04	0.045977	0	0	0.021501
Novel_006671	XLOC_0696E .	transcript	0.053838	0.385616	0.278763	0.176726	0.676761	0.014309	0.265509	0.264242	0.012281
Novel_006672	XLOC_0696E .	transcript	0.005455	0.283086	0.180557	0	0	0	0.221754	0.211762	0
Novel_006673	XLOC_0696E .	transcript	0.049209	0.11531	0.193462	0.582963	0.058072	0.142327	0.320315	0.216425	0.000218
Novel_006674	XLOC_0696E .	transcript	0.006181	0	0	0.029052	0	0.034288	0	0	0
Novel_006675	XLOC_0696E .	transcript	0.378879	0.299181	0.187524	0	0.586007	0.818637	0	0	0
Novel_006676	XLOC_0696E .	transcript	0.729911	1.714731	1.625151	2.497531	1.348592	1.47804	2.496389	2.732144	1.664174

Novel_006677	XLOC_06968.	transcript	0	0	0	0	0	0	0	0	0
Novel_006678	XLOC_06968.	transcript	1.840833	1.4375	2.954644	1.975893	2.163248	2.399282	2.560074	2.733765	4.412141
Novel_006679	XLOC_06970.	transcript	1.443393	0.281599	1.755748	2.228563	2.43267	2.419026	2.347855	1.994184	1.547527
Novel_006680	XLOC_06972.	transcript	0.216699	0.652402	0.087704	0.069333	0	0.249656	0.074515	0.379312	0
Novel_006681	XLOC_06972.	transcript	0.572036	0.028855	0.789547	0.781304	0.602617	0.614298	1.25251	0.215931	0.601115
Novel_006682	XLOC_06972.	transcript	0	0	0.068192	0.314557	0.155783	0	0.11676	0.024524	0
Novel_006683	XLOC_06972.	transcript	0	0.974496	0	0	0.214694	0	0	0.395255	0.186541
Novel_006684	XLOC_06972.	transcript	0.632825	1.167787	0.615266	0.106341	0.15188	0.444753	1.009579	0.192436	0.778936
Novel_006685	XLOC_06972.	transcript	0	0.013305	1.796426	1.01745	3.346145	2.43646	1.151	3.518731	2.131624
Novel_006686	XLOC_06972.	transcript	0.842736	2.364875	0.566861	2.934735	1.038824	0.736989	1.051716	0.781755	0.476271
Novel_006687	XLOC_06973.	transcript	0.538424	0	0	0.612892	0.407283	0	0.557137	0.532386	0.217135
Novel_006688	XLOC_06973.	transcript	0	0	0.215384	0	0	0	0	0	0
Novel_006689	XLOC_06973.	transcript	0.086612	0.116151	0.104096	0.190954	0.129666	0	0.168515	0.190803	0.067078
Novel_006690	XLOC_06973.	transcript	0.177935	0.372103	0.143009	0.219789	0.173857	0.376468	0.241102	0.197097	0.313436
Novel_006691	XLOC_06973.	transcript	0.310323	1.032887	1.128312	0.231582	0.167277	1.236	0.216138	0.205797	0.987639
Novel_006692	XLOC_06973.	transcript	0	0.152735	0	0.184583	0.158632	0	0	0.164939	0
Novel_006693	XLOC_06974.	transcript	0	0	0.179482	0	0	0	0.081164	0	0
Novel_006694	XLOC_06974.	transcript	0.003873	0.308797	0.032806	0.017525	0.244993	0.14542	0.069782	0.244903	0.075046
Novel_006695	XLOC_06974.	transcript	0.281041	0.460414	0.318221	0.535673	0.363201	0.367141	0.409668	0.504776	0.353701
Novel_006696	XLOC_06974.	transcript	0.11908	0.103953	0.154805	0.087441	0.32776	0.268102	0.002645	0.469865	0.122092
Novel_006697	XLOC_06974.	transcript	0.322954	0.501196	0.354175	0.450929	0.4715	0.338654	0.631407	0.507872	0.293212
Novel_006698	XLOC_06978.	transcript	0.17801	0.168948	0.075184	0.07098	0.152764	0.007547	0.004621	0.07915	0.008342
Novel_006699	XLOC_06978.	transcript	0.049588	0	0	0.178249	0	0	0.316987	0.014793	0.1385
Novel_006700	XLOC_06978.	transcript	0	0.008175	0.111258	0	0.192627	0.254056	0.00872	0.296314	0
Novel_006701	XLOC_06979.	transcript	0	0	0.2223	0	0	0.092502	0.047826	0	0.031254
Novel_006702	XLOC_06979.	transcript	0	0	0	0	0	0	0	0.048558	0.669544
Novel_006703	XLOC_06979.	transcript	0	0	0.052818	0.104355	0.240255	0.219547	0.075161	0.082627	0.024879
Novel_006704	XLOC_06979.	transcript	0.083487	0.052145	0.013007	0.023283	0.07516	0.040682	0	0.046338	0.113002
Novel_006705	XLOC_06979.	transcript	0.089298	0	0	0	0	0.144101	0.137043	0.011117	0.14723
Novel_006706	XLOC_06983.	transcript	0.165897	0.194096	0.168585	0.032927	0.207298	0	0.212479	0.083491	0.166606
Novel_006707	XLOC_06984.	transcript	0.082621	0.130108	0.085242	0.04897	0.043793	0.062814	0.126588	0.113527	0.169098
Novel_006708	XLOC_06985.	transcript	0.235651	0.471639	0.105413	0.111041	0.068961	0.40621	0.161595	0.186908	0.322909
Novel_006709	XLOC_06985.	transcript	0.456054	0.539196	0.416185	0.060856	0.187749	0.420653	0.467779	0.180657	0.003479
Novel_006710	XLOC_06985.	transcript	0	0	0	0	0.294051	0	0	0	0
Novel_006711	XLOC_06985.	transcript	0.214415	0	0.279561	0.001095	0	0.0996	0.229073	0.071861	0.472645
Novel_006712	XLOC_06985.	transcript	0.183202	0.150308	0.216871	0.370755	0.047888	0.011551	0.215853	0.002554	0.126573
Novel_006713	XLOC_06985.	transcript	0.011837	0.022619	0.075411	0.117786	0.343747	0	0.030311	0	0
Novel_006714	XLOC_06985.	transcript	0	0	0	0.011863	0	0.250874	0	0.288087	0

Novel_006715	XLOC_06985.	transcript	0	0.00331	0	0	0.004825	0.285257	0.006042	0.408555	0
Novel_006716	XLOC_06994.	transcript	0.000628	0	0.00074	0	0.005004	0.007637	0.057715	0.018239	0
Novel_006717	XLOC_06994.	transcript	0.228703	0	0.418865	0	0	0.278446	0.25444	0.328481	0
Novel_006718	XLOC_06994.	transcript	0.026887	0.171545	0	0.156042	0	0	0.504633	0.262845	0
Novel_006719	XLOC_06994.	transcript	0.394572	0.577492	0	0	0	0	0	0	0
Novel_006720	XLOC_06994.	transcript	0	0.005388	0.072854	0.068597	0.134697	0.020589	0.129387	0.167604	0.042599
Novel_006721	XLOC_06994.	transcript	0.540437	0.85365	0.351271	0.408244	0.318604	0.653547	0.705168	0.338814	0.243703
Novel_006722	XLOC_06994.	transcript	0	0.754729	0	0.168723	0.005291	0	0	0.144557	0.095928
Novel_006723	XLOC_06994.	transcript	0.159474	0.105041	0.354133	0.085131	0	0	0	0.079703	0.033329
Novel_006724	XLOC_06994.	transcript	0.035601	0.0188	0.28954	0.025566	0	0	0	0.014111	0.034586
Novel_006725	XLOC_06994.	transcript	0	0	0	0	0	0	0	0	0
Novel_006726	XLOC_06995.	transcript	57.45948	47.56161	75.98325	81.3416	67.56989	87.35046	57.15679	64.54827	89.87168
Novel_006727	XLOC_06996.	transcript	0.176181	0.047072	0.131502	0.020412	0	0.038428	0.137836	0.113136	0.099422
Novel_006728	XLOC_06996.	transcript	0	0	0	0	0.141357	0	0.011207	0	0
Novel_006729	XLOC_06999.	transcript	0.840396	1.511791	0.90488	1.566296	1.145459	1.008454	1.111127	1.399484	0.756957
Novel_006730	XLOC_06999.	transcript	0	0	0	0	0	0	0	0	0.320377
Novel_006731	XLOC_07000.	transcript	0.055459	0.022924	0	0	0.091523	0.106316	0.333237	0	0.127658
Novel_006732	XLOC_07000.	transcript	0.104942	0.053832	0	0.033687	0	0.131377	0.060053	0	0
Novel_006733	XLOC_07000.	transcript	0.125585	0.015801	0.055128	0.008973	0.256626	0	0.004933	0.051601	0.005176
Novel_006734	XLOC_07000.	transcript	0.042165	0.29988	0	0.131009	0.016762	0.134431	0.019629	0.102076	0
Novel_006735	XLOC_07002.	transcript	0.18798	0.409465	0.308315	0.032866	0.35349	0.93957	0.061845	0.09471	0.893418
Novel_006736	XLOC_07002.	transcript	0.390471	0	0.168324	0.130843	0.613774	0.71116	0.435202	0.344764	0
Novel_006737	XLOC_07002.	transcript	1.080892	0.368147	1.076577	0.806328	0.37948	0.510432	0.809135	0.945231	0
Novel_006738	XLOC_07004.	transcript	0	0.234054	0.189394	0	0	0	0	0	0.478534
Novel_006739	XLOC_07004.	transcript	0.114618	0	0.102148	0	0.546473	0.211576	0.020695	0.035668	0
Novel_006740	XLOC_07004.	transcript	0	0	0	0.003958	0	0	0	0	0
Novel_006741	XLOC_07004.	transcript	0.063779	0.409994	0.25873	0.014824	0	0.033463	0.093637	0.208285	0
Novel_006742	XLOC_07004.	transcript	0.198072	0.001053	0	0.017672	0.262422	0.099097	0.463516	0.040723	0.327921
Novel_006743	XLOC_07004.	transcript	0	0	0	0.412665	0	0.217261	0	0.157984	0
Novel_006744	XLOC_07004.	transcript	0	0	0	0	0	0	0	0.310473	0
Novel_006745	XLOC_07004.	transcript	0.161675	0.211242	0.169114	0.187225	0.25931	0.062481	0.435176	0.228734	0.115903
Novel_006746	XLOC_07004.	transcript	0	0.30837	0	0	0.565605	0.005046	0.4766	0.051034	0
Novel_006747	XLOC_07004.	transcript	0.269671	1.102195	0.287229	0.298374	0.029436	0.343257	0.338572	0.366101	0.547635
Novel_006748	XLOC_07004.	transcript	0	0.242816	0	0	0	0.000163	0.031604	0	0.253272
Novel_006749	XLOC_07004.	transcript	0	0.155206	0.323829	0	0.261543	0.333729	0.143756	0.078702	0.460131
Novel_006750	XLOC_07004.	transcript	0.0011	0.443775	0.341186	0.547147	0.409737	0.364751	0.654919	0.414992	0.570593
Novel_006751	XLOC_07004.	transcript	0.408173	0.110914	0.320022	0.953387	0.478828	0.482915	0.95477	0.544856	0.191593
Novel_006752	XLOC_07004.	transcript	0.237837	0.097956	0.137012	0.208821	0.175659	0.025891	0.184943	0.253203	0.032938

Novel_006753	XLOC_07004.	transcript	1.090257	0.194215	0.84387	1.09102	0.962811	0.850635	1.172468	1.153237	1.070011
Novel_006754	XLOC_07004.	transcript	0.371226	1.104115	0	1.026	0.373909	0.709215	1.164878	0.530926	0.751143
Novel_006755	XLOC_07004.	transcript	0.272073	0.79252	0.777788	0.246526	0.534794	0.557793	9.10E-05	0.593528	0.250267
Novel_006756	XLOC_07006.	transcript	0	0.213831	0	0	0	0	0	0	0
Novel_006757	XLOC_07007.	transcript	0.505527	0.696203	0.403675	0.277419	0.329681	0.27739	0.420119	0.271586	0
Novel_006758	XLOC_07007.	transcript	0.131023	0	0	0	0.079045	0	0.17175	0.116834	0.24944
Novel_006759	XLOC_07007.	transcript	0.029779	0.948878	0.015059	0.024542	0.072903	0.031719	0.122346	0.047797	1.120262
Novel_006760	XLOC_07009.	transcript	0.106404	0.585901	0.056059	0.11865	0.087778	0.118504	0.162625	0.177803	0.139125
Novel_006761	XLOC_07009.	transcript	0.16712	0.262073	0.244051	0.253734	0.10649	0.444764	0.183116	0.361827	0.3865
Novel_006762	XLOC_07013.	transcript	0.075736	0.106637	0.082854	0.111326	0.110332	0.081821	0.118361	0.10199	0.116376
Novel_006763	XLOC_07036.	transcript	0.090975	0.073792	0.050112	0.032452	0.044454	0.162226	0.103055	0.070308	0.032207
Novel_006764	XLOC_07042.	transcript	0.056872	0.043316	0.049677	0.038208	0.049937	0.094411	0.046405	0.071631	0.091105
Novel_006765	XLOC_07051.	transcript	0	0.322369	0	0	0	0	0	0	0
Novel_006766	XLOC_07051.	transcript	1.109146	0.353723	0	0	0	0	0	0	0
Novel_006767	XLOC_07051.	transcript	0	0.617433	0	0	0.000197	0	0	0.598887	0
Novel_006768	XLOC_07051.	transcript	0	0	0	0	0	0	0	0	0
Novel_006769	XLOC_07051.	transcript	0.34286	0.073537	0.453394	0.370029	0.365784	0.595037	0.638926	0.073979	0.479005
Novel_006770	XLOC_07051.	transcript	0	0	0	0	0	0	0	0	0
Novel_006771	XLOC_07054.	transcript	0.137571	0.078221	0.145511	0.378747	0.257949	0.331967	0.45464	0.348274	0.595686
Novel_006772	XLOC_07054.	transcript	0	0	0	0	0	0	0	0	0
Novel_006773	XLOC_07055.	transcript	0.456384	0.194306	0.186337	0	0.015883	0.116196	0.319508	0.021409	0.011705
Novel_006774	XLOC_07055.	transcript	0.018214	0.183636	0.091771	0.366175	0.27869	0.043619	0.106386	0.358606	0.484975
Novel_006775	XLOC_07056.	transcript	0.159452	0	0.159256	0	0.00851	0.121128	0.228962	0.04505	0.155552
Novel_006776	XLOC_07056.	transcript	0	0.026332	0.026538	0.06601	0.161855	0	0.046548	0.244382	0
Novel_006777	XLOC_07060.	transcript	1.167946	1.249854	1.399979	1.1363	0.793953	1.099942	1.484026	1.390779	1.153551
Novel_006778	XLOC_07062.	transcript	0	0	0	0	0	0.192274	0.099383	0.188405	0
Novel_006779	XLOC_07062.	transcript	0.031257	0	0.114711	0	0	0.027696	0.195323	0.056877	0
Novel_006780	XLOC_07062.	transcript	0.004313	0	0.047861	0.193831	0.231034	0.052036	0.003226	0.083488	0.182141
Novel_006781	XLOC_07062.	transcript	0.069881	0	0.08219	0	0	0.03606	0.031209	0	0.005218
Novel_006782	XLOC_07063.	transcript	0.025048	0.003867	0.210189	0.007372	0.098533	0.012427	0	0.018292	0
Novel_006783	XLOC_07063.	transcript	0	0	0	0	0	0.002559	0	0	0.000859
Novel_006784	XLOC_07063.	transcript	0.142192	0.435649	0	0.157062	0.007247	0.183375	0.157193	0.083729	0.036511
Novel_006785	XLOC_07063.	transcript	0.084699	0.093664	0.078987	0.128746	0	0.071256	0	0.144645	0.047304
Novel_006786	XLOC_07063.	transcript	0.007508	0.004923	0.221873	0	0.018372	0.002284	0.071573	0.075113	0.23952
Novel_006787	XLOC_07063.	transcript	0.116312	0.085154	0	0.115538	0	0.116377	0.190476	0.216983	0
Novel_006788	XLOC_07063.	transcript	0	0	0	0	0.265238	0.110768	0	0	0
Novel_006789	XLOC_07093.	transcript	0	0.198388	0.030594	0	0.115727	0	0.052953	0.097699	0.272192
Novel_006790	XLOC_07098.	transcript	0.03533	0	0	0	4.60E-05	0.006619	0	0.004974	0

Novel_006791	XLOC_07098.	transcript	0.184899	0.179488	0.165979	0.206659	0.267916	0.21064	0.344921	0.269357	0.14666
Novel_006792	XLOC_07104.	transcript	0.217743	0	0.13536	0	0	0.236106	0	0	0
Novel_006793	XLOC_07104.	transcript	0	0.034503	0	0.204094	0.148418	0	0.277069	0.21257	0.35523
Novel_006794	XLOC_07108.	transcript	0.201917	1.633399	0.435153	0.469974	0.398221	0.418738	0.38106	0.317211	0.335587
Novel_006795	XLOC_07112.	transcript	0.12318	0.038217	0.04698	0.146108	0.09548	0.190704	0.419336	0.072795	0.186618
Novel_006796	XLOC_07114.	transcript	0.264285	0.219539	0.327427	0.25616	0.357363	0.297907	0.509691	0.310294	0.413706
Novel_006797	XLOC_07114.	transcript	0.007384	0	0.002093	0.011868	0	0.003702	0	0.015249	0
Novel_006798	XLOC_07114.	transcript	0.630966	0.891364	0.476835	0.26208	0.564858	0.54751	1.09514	0.52413	0.374971
Novel_006799	XLOC_07114.	transcript	0.639657	0.361241	0.616496	0.280005	0.513647	0.327672	0.315316	0.27915	0.47504
Novel_006800	XLOC_07114.	transcript	0	0	0	0.027764	0.009919	0	0.114618	0.011394	0
Novel_006801	XLOC_07114.	transcript	0.242348	0.081083	0.220529	0.206992	0.308264	0.198433	0.329935	0.345527	0.295023
Novel_006802	XLOC_07115.	transcript	0.015846	0.35308	0.129939	0.042941	0.056355	0.18763	0.286913	0.091889	0.108235
Novel_006803	XLOC_07124.	transcript	0.329724	0.251353	0.798475	0.918553	0.993664	0.767072	0.505476	0.745508	0.514366
Novel_006804	XLOC_07125.	transcript	0.448919	0	0	0.245771	0	0	0.28104	0.438364	0.015346
Novel_006805	XLOC_07125.	transcript	1.954713	1.7819	4.25451	10.7875	5.449095	2.200827	3.527534	6.0638	1.566478
Novel_006806	XLOC_07127.	transcript	0.728938	0.680807	0.738717	0.626592	0.461525	0.634836	0.629283	1.070165	0.608314
Novel_006807	XLOC_07129.	transcript	0	0	0	0	0	0	0	0	0
Novel_006808	XLOC_07129.	transcript	0.901819	0.600669	1.64749	2.58815	1.480868	1.401452	1.428955	2.07987	1.24633
Novel_006809	XLOC_07129.	transcript	0.101202	1.183981	0.026849	0.042492	0.107558	0.126337	0.085677	0.00861	0.189805
Novel_006810	XLOC_07129.	transcript	0	0.938728	0.166356	0.030883	0	0	0.072008	0.126542	0.476924
Novel_006811	XLOC_07129.	transcript	0.066178	0.212927	0.544362	0.466951	0.508994	0.29901	0.492142	0.447487	0.605207
Novel_006812	XLOC_07129.	transcript	0.362879	0.498709	0.00092	0.3993	0.45822	0.366118	0.476171	0.457608	0
Novel_006813	XLOC_07130.	transcript	0.256414	0.009331	0	0	0.160915	0.257301	0	0	0
Novel_006814	XLOC_07130.	transcript	0	0.083687	0.518442	0.886673	0.803871	0.324192	1.030324	0.821339	0.406042
Novel_006815	XLOC_07130.	transcript	0.190248	0	0	0.798597	0.005059	0.00552	1.534976	1.095953	0
Novel_006816	XLOC_07130.	transcript	0.279677	0	0	0	0.104558	0.163152	0.00496	0	0.04067
Novel_006817	XLOC_07130.	transcript	0	0.094053	0.466852	0	0.174121	0.328373	0.026374	0	0.390405
Novel_006818	XLOC_07130.	transcript	0	0	0	0	0	0	0	0	0
Novel_006819	XLOC_07130.	transcript	0	0	0	0	1.644175	0	0	0	0
Novel_006820	XLOC_07130.	transcript	0	0	0.011638	0	0	1.611127	0	0	0
Novel_006821	XLOC_07130.	transcript	0.130202	0.4173	0.351469	0.430574	0.214679	0.222107	0.792509	0.183424	0.455284
Novel_006822	XLOC_07133.	transcript	0.345437	0.535507	0.324637	0.493701	0.504574	0.365677	0.505854	0.494221	0.30168
Novel_006823	XLOC_07134.	transcript	0.14941	0.04523	0.188025	0.07247	0.0733	0.205834	0.269516	0.074435	0.150912
Novel_006824	XLOC_07134.	transcript	0.067551	0.062861	0.1696	0	0	0	0.219671	0.110715	0.135359
Novel_006825	XLOC_07134.	transcript	0.17069	0.293941	0.015	0.079988	0.291147	0.034545	0.104206	0.070336	0.335079
Novel_006826	XLOC_07134.	transcript	0.752047	0.724705	0.470261	0.593547	0.424397	0.596741	0.852962	0.767985	0.506746
Novel_006827	XLOC_07134.	transcript	0.085362	0.024636	0.041262	0.133678	0.059977	0.070752	0.182518	0.094326	0
Novel_006828	XLOC_07135.	transcript	0.137789	0.032635	0.099685	0.059072	0.166839	0.028441	0.238942	0.233038	0.099361

Novel_006829	XLOC_07135.	transcript	0.368146	0.26984	0	0	0.020735	0	0.358211	0.394907	0.542895
Novel_006830	XLOC_07135.	transcript	0.526149	0.342048	0.467433	0.073273	0.206097	0.547459	0.449832	0.385074	0.429461
Novel_006831	XLOC_07136.	transcript	0.050069	0.206093	0.084614	0.176332	0.030947	0.105464	0	0.151734	0.040753
Novel_006832	XLOC_07136.	transcript	0	0	0	0	0.056894	0.03688	0.162733	0.069866	0
Novel_006833	XLOC_07136.	transcript	0.123247	0.345341	0.157523	0.146913	0.26248	0.178582	0.037168	0.045446	0.069525
Novel_006834	XLOC_07136.	transcript	1.493942	0	0	0	0.005288	0	0	0	0
Novel_006835	XLOC_07136.	transcript	0	0	0.721027	0	0	0	0	0	0
Novel_006836	XLOC_07136.	transcript	0.591952	1.139254	2.128134	2.416925	2.113807	2.275087	2.744324	2.364914	2.027572
Novel_006837	XLOC_07138.	transcript	1.687804	2.714947	1.995111	2.369706	1.966944	1.846304	2.180301	2.25772	1.490531
Novel_006838	XLOC_07140.	transcript	0.042567	0.008399	0	0.075756	0	0.062555	0.338143	0.000495	0
Novel_006839	XLOC_07140.	transcript	0.20622	0.189948	0.147698	0.363482	0.372602	0.370729	0.379635	0.15223	0.273866
Novel_006840	XLOC_07140.	transcript	0	0.015707	0.00951	0.036939	0	0	0.006264	0.370887	0.023437
Novel_006841	XLOC_07141.	transcript	0	0.115218	0.003272	0.008029	0	0	0	0	0.017959
Novel_006842	XLOC_07141.	transcript	1.00E-05	0	0	0.071273	0	0.225605	0.077882	0	0.000158
Novel_006843	XLOC_07141.	transcript	0.213912	0.088703	0.077295	0.070293	0.110299	0.084211	0.145994	0.073198	0
Novel_006844	XLOC_07141.	transcript	0.008641	0	0	0.09748	0	0.106378	0.204001	0	0.014657
Novel_006845	XLOC_07141.	transcript	0	0	0	0	0	0.088572	0	0	0.019354
Novel_006846	XLOC_07141.	transcript	0	0.141527	0.002219	0	0.204577	0.014979	0.002648	0.080072	0
Novel_006847	XLOC_07141.	transcript	0.178742	0.1843	0.118792	0.061971	0.139961	0.085553	0.284756	0.046619	0.207654
Novel_006848	XLOC_07141.	transcript	0.057275	0.046503	0.033315	0.033139	0.043967	0.04193	0.038949	0.028515	0.041665
Novel_006849	XLOC_07141.	transcript	0.076342	0.05456	0.048815	0	0.05856	0.058798	0.052065	0.038235	0.072738
Novel_006850	XLOC_07141.	transcript	0.080325	0.063241	0	0.059605	0.053361	0.053587	0	0.047697	0
Novel_006851	XLOC_07141.	transcript	0.063864	0	0.07166	0.051369	0.057127	0.062065	0.053989	0.03739	0.053645
Novel_006852	XLOC_07141.	transcript	0.120684	0.07589	0.075204	0.06798	0.091222	0	0.079953	0.066832	0.111832
Novel_006853	XLOC_07141.	transcript	0.150686	0.068389	0	0.059348	0.107494	0.052242	0	0.046814	0
Novel_006854	XLOC_07141.	transcript	0.007611	0.001559	0.086271	0.091992	0	0	0	0.078266	0.005908
Novel_006855	XLOC_07141.	transcript	0	0.107159	0.079645	0	0.127841	0.081843	0.242537	0.068747	0.001588
Novel_006856	XLOC_07141.	transcript	0	0.002168	0.099751	0.099899	0	0.093842	0	0.071255	0.045158
Novel_006857	XLOC_07141.	transcript	0	0	0.102089	0	0	0.084474	0	0.063163	0
Novel_006858	XLOC_07141.	transcript	0	0	0.08357	0	0	0.091254	0.000298	0.07476	0
Novel_006859	XLOC_07141.	transcript	0.1306	0.013685	0.001174	0.005031	0	0.008696	0.016808	0.007533	6.90E-05
Novel_006860	XLOC_07141.	transcript	0.185125	0	0.110396	0.085254	0.225941	0.121985	0.207615	0.070255	0
Novel_006861	XLOC_07141.	transcript	0.225883	0	0.192526	0.118104	0.246609	0.145213	0.271906	0.095845	0.427933
Novel_006862	XLOC_07141.	transcript	0	0.163084	0.12044	0.123942	0.30207	0.129564	0.313281	0.08009	0
Novel_006863	XLOC_07141.	transcript	0	0.170101	0.14272	0.131127	0.311468	0	0.326474	0.084831	0
Novel_006864	XLOC_07141.	transcript	1.007877	0.865089	0.192281	0.309733	0.388715	0.345127	0.40962	0.614732	0.453095
Novel_006865	XLOC_07141.	transcript	0.108252	0.132045	0.212131	0.117557	0.087953	0.116236	0.321425	0.237289	0.191219
Novel_006866	XLOC_07141.	transcript	0.096388	0.011282	0	0	0	0	0	0.066594	0.013186

Novel_006867	XLOC_07144.	transcript	0.881503	0.955318	1.188429	1.743194	1.417408	1.154047	1.101698	1.411867	1.3622
Novel_006868	XLOC_07145.	transcript	0	0.114249	0.165916	0.189254	0.045239	0.05425	0.084062	0	0.035744
Novel_006869	XLOC_07147.	transcript	0.413915	4.986085	0.251358	0.164216	0.180791	0.191324	0.564778	0.189622	0.904308
Novel_006870	XLOC_07148.	transcript	0	0.293545	0.090589	0	0	0.061546	0	0.028668	0.473217
Novel_006871	XLOC_07149.	transcript	0.520854	0.11667	0.079492	0.090319	0.101826	0.062853	0.069666	0.11274	0.08625
Novel_006872	XLOC_07149.	transcript	0.033825	0	0	0	0.0509	0	0.01515	0	0.30284
Novel_006873	XLOC_07149.	transcript	0	0	0	0	0.023475	0	0.042827	0	0
Novel_006874	XLOC_07149.	transcript	0	0	0.184014	0	0	0	0	0	0
Novel_006875	XLOC_07149.	transcript	0.163528	0.096652	0.368095	0.332217	0.157535	0.139685	0.391138	0.300362	0
Novel_006876	XLOC_07149.	transcript	0.034943	0.263054	0.009021	5.90E-05	0	2.90E-05	0	0.015279	0.133714
Novel_006877	XLOC_07149.	transcript	0.068444	0.268289	0.005438	0	0.213263	0.157441	0.053313	0.026242	0.203368
Novel_006878	XLOC_07149.	transcript	0	0.172896	0	0	0	0.200968	0	0	0
Novel_006879	XLOC_07149.	transcript	0	1.315729	0	0	0.066858	0.056017	0.177799	0	0.442493
Novel_006880	XLOC_07149.	transcript	0.232734	0.011338	0.176071	0.03177	0.054645	0.097831	0.20604	0.15703	0.11952
Novel_006881	XLOC_07149.	transcript	0.005109	0.04018	0.178869	0.094941	0	0.047238	0.044671	0.017963	0
Novel_006882	XLOC_07149.	transcript	0.112941	0	0	0	0.171807	0	0.240523	0.126714	0.073809
Novel_006883	XLOC_07150.	transcript	0.025784	0.090663	0.023024	0.028355	0.042677	0.06123	0.241985	0.034195	0.040029
Novel_006884	XLOC_07150.	transcript	0.620565	0.556755	0.848407	0.552126	0.696373	0.681529	0.809418	0.657191	0.624008
Novel_006885	XLOC_07151.	transcript	0.223986	0.315754	0.66713	1.488915	1.365824	1.375976	0.774653	1.164443	0.32739
Novel_006886	XLOC_07152.	transcript	0.451253	0.750267	0.75843	0.863126	0.691667	0.957511	0.653837	0.776396	0.86176
Novel_006887	XLOC_07152.	transcript	0	0	0.033639	0	0	0	0	0.008289	0.002498
Novel_006888	XLOC_07153.	transcript	0.006862	0.028749	0.022931	0	0.086198	0	0	0	0
Novel_006889	XLOC_07153.	transcript	1.427267	1.94646	1.733849	2.223397	2.01174	1.401516	1.700578	2.425713	1.691548
Novel_006890	XLOC_07154.	transcript	0.026145	2.178388	0.054267	0.038403	0.103752	0.121024	0.039106	0	0.123628
Novel_006891	XLOC_07154.	transcript	0	0.503904	0	0	0	0	0	0	0
Novel_006892	XLOC_07154.	transcript	0.218175	0.396634	0.382303	0.127043	0.204867	0.287127	0.270812	0.209104	0.208354
Novel_006893	XLOC_07155.	transcript	0.256589	0	0	0	0	0	0.29723	0	0
Novel_006894	XLOC_07155.	transcript	0.354631	0.007995	0.262633	0.679529	0.329982	0.87753	0.66907	0.895348	0.526856
Novel_006895	XLOC_07159.	transcript	0.597213	0.180966	0.878681	0.353402	0.524563	0.571956	0.869303	0.523482	0.375396
Novel_006896	XLOC_07161.	transcript	0	0	0	0	2.222451	0	0	0	0.013438
Novel_006897	XLOC_07161.	transcript	1.576717	2.795936	3.84282	3.815075	1.214149	3.686439	4.02191	3.167768	3.147735
Novel_006898	XLOC_07162.	transcript	0	0	0	0	0	0	0	1.60E-05	0
Novel_006899	XLOC_07162.	transcript	0.209244	1.396314	1.219599	1.396152	1.029294	1.14198	1.35179	0.778569	0.586158
Novel_006900	XLOC_07162.	transcript	1.655311	4.00E-05	0	0.027365	0	0	0.334698	0.000854	0.621057
Novel_006901	XLOC_07164.	transcript	0	0	0.320198	0	0	0	0	0	0
Novel_006902	XLOC_07164.	transcript	0.244906	0.292376	0.176757	0.389778	0.416862	0.316095	0.584979	0.498347	0.728526
Novel_006903	XLOC_07165.	transcript	0.181417	0.207944	0.360732	0.45674	0.815123	0.122439	0.268359	0.48387	0.339078
Novel_006904	XLOC_07165.	transcript	0.340249	0.016309	0	0.176811	0	0.022778	0.19451	0.740021	0.001386

Novel_006905	XLOC_07165.	transcript	0.514665	0.825668	0.550761	0.741753	0.673206	0.585034	0.753143	0.582488	0.617867
Novel_006906	XLOC_07165.	transcript	0	0	0	0	0	0	0	0.464699	0
Novel_006907	XLOC_07165.	transcript	0.422984	0.269377	0.315496	0.218497	0.377885	0.432839	0.385162	0	0.293015
Novel_006908	XLOC_07165.	transcript	0.43665	0.501328	0.491957	0.179811	0.580846	0.839264	0.346181	0.371048	0.182491
Novel_006909	XLOC_07165.	transcript	0.134982	0.305939	0.271378	0.358928	0.346016	0.436267	0.363503	0.223353	0.269329
Novel_006910	XLOC_07165.	transcript	0.112861	0	0	0.241903	0.245372	0	0.316293	0.182201	0.225081
Novel_006911	XLOC_07165.	transcript	0.127622	0.276058	0.230733	0.378435	0.338435	0.420355	0.494844	0.230518	0.338476
Novel_006912	XLOC_07165.	transcript	0.189667	0.847822	0.66827	1.128453	0.61591	0.947104	1.320627	0.862796	0.882816
Novel_006913	XLOC_07165.	transcript	1.000413	0.890826	1.213097	0	0	0	0	0.67385	0
Novel_006914	XLOC_07165.	transcript	0.348191	0	0	0.434391	0.535668	0	0.400818	0.513128	0.248511
Novel_006915	XLOC_07165.	transcript	0	0	0	0	0	0	0	0	0
Novel_006916	XLOC_07165.	transcript	0.872744	0.556886	0.808472	1.250249	1.133769	1.26614	1.605033	1.71801	1.097478
Novel_006917	XLOC_07165.	transcript	0.048659	0.202748	0.443559	0.360081	0.227663	0.269887	0.348069	0.395754	0.375598
Novel_006918	XLOC_07167.	transcript	0.349726	0.883034	0.527475	0.810837	0.384659	0.607984	0.519889	0.864012	0.463204
Novel_006919	XLOC_07167.	transcript	0.104118	0.495136	0	0.286228	0.094292	0	0.059406	0.196371	0
Novel_006920	XLOC_07167.	transcript	0	0.136751	0.057744	0.090314	0.105149	0	0.081982	0	0
Novel_006921	XLOC_07167.	transcript	0.140286	0.23101	0.019386	0.178503	0.124082	0.256639	0.197846	0.46618	0.075063
Novel_006922	XLOC_07167.	transcript	0.126143	0.201454	0.854807	1.954326	1.249641	0.604976	0.870307	1.319818	0.322065
Novel_006923	XLOC_07170.	transcript	0	0.23249	0.104907	0.410034	0.416506	0	0.619264	0.250557	0.260679
Novel_006924	XLOC_07170.	transcript	1.015109	0.166088	0.258852	0	0.01511	0.358561	0.232517	0.382459	0.35665
Novel_006925	XLOC_07171.	transcript	0.21365	0.044239	0.199278	0.174818	0.292179	0.335686	0.458827	0.300585	0.328984
Novel_006926	XLOC_07173.	transcript	0	0.389374	0	1.47313	0	0	0.007023	0.593126	0
Novel_006927	XLOC_07173.	transcript	0.120253	0	0.283404	0.29508	0.162528	0.203826	0.111467	0	0.163521
Novel_006928	XLOC_07173.	transcript	0	0	0	0.780234	0.797175	0	0.507626	1.151188	0.288402
Novel_006929	XLOC_07173.	transcript	0.407914	0	0.451584	0.014374	0.375557	0.431909	0.52606	0.214287	0.237669
Novel_006930	XLOC_07173.	transcript	0	0.009413	0	0.029681	0	0.053596	0.020488	0.052895	0.013798
Novel_006931	XLOC_07173.	transcript	0.549872	0.790049	0.459814	0.38785	0.746033	0.633424	0.735411	0.214155	0.744557
Novel_006932	XLOC_07173.	transcript	0.344652	0.155823	0.127761	0.732241	0.318341	0.284092	0.214181	0.338073	0.292594
Novel_006933	XLOC_07173.	transcript	0	0	0	0.255642	0	0	0	0.302203	0
Novel_006934	XLOC_07173.	transcript	0	0	0	0.254125	0	0	0	0.242362	0
Novel_006935	XLOC_07173.	transcript	0.149837	0.047972	0.310439	0.003946	0.04729	0.065827	0.099229	0.217454	0.075703
Novel_006936	XLOC_07173.	transcript	0	0	0	0	0	0	0	0.265216	0
Novel_006937	XLOC_07173.	transcript	0.101146	0.270195	0.125115	0.318536	0.215151	0.245783	0.074414	0.067523	0.18509
Novel_006938	XLOC_07173.	transcript	0	0	0	0.255155	0.018561	0.035189	0	0.230357	0
Novel_006939	XLOC_07173.	transcript	0	0.180931	0	0	0	0	0.050523	0	0.613258
Novel_006940	XLOC_07175.	transcript	0.000633	0	0.569504	0.322496	0	0.020543	0	0	0.534647
Novel_006941	XLOC_07175.	transcript	0	0	0.259607	0.244798	0.146559	0.239676	0	0.227431	0
Novel_006942	XLOC_07175.	transcript	0.00551	0.113825	0.021317	0	0.165341	0.382773	0.192521	0	0

Novel_006943	XLOC_07175.	transcript	0.505723	0	0.196254	0.224182	0.382719	0.191351	0.521643	0.588996	0.132369
Novel_006944	XLOC_07176.	transcript	0	0	0	0	0	0	0	0	0
Novel_006945	XLOC_07176.	transcript	0	0.007576	0.044178	0.360621	0.079044	0.358065	0.107259	0.08694	0.004296
Novel_006946	XLOC_07176.	transcript	0.053262	0.473924	0.139659	0.070589	0.267855	0.155396	0.35926	0.175303	0.129503
Novel_006947	XLOC_07176.	transcript	0	0	0	0	0	0.268152	0	0	0
Novel_006948	XLOC_07176.	transcript	0.029348	0	0	0.004028	0	0	0	0	0
Novel_006949	XLOC_07176.	transcript	0.080878	0.000588	0.04147	0.070125	0.006268	0.086639	0.050862	0.007792	0
Novel_006950	XLOC_07176.	transcript	0.244026	0.005634	0.005032	0.077498	0.010162	0	0	0.223226	0.013327
Novel_006951	XLOC_07176.	transcript	0	0	0.166131	0.047231	0.048003	0.063538	0.229311	0.108906	0
Novel_006952	XLOC_07176.	transcript	0	0.00518	0.008191	0.000627	0.002194	0	0.006564	0.011948	0.109285
Novel_006953	XLOC_07176.	transcript	0	0	0.163487	0.006152	0.009137	0	0.084532	0.06464	0.002276
Novel_006954	XLOC_07176.	transcript	0.184432	0.313159	0.416824	0.804088	0.613038	0.416486	0.711186	0.662603	0.616674
Novel_006955	XLOC_07177.	transcript	0.169721	0.478561	0.540783	0.199253	0.302875	0.385148	0.349341	0.100743	0.343928
Novel_006956	XLOC_07178.	transcript	0.180212	0.119966	0.031824	0.050416	0.138546	0.129841	0.177029	0.118923	0.089187
Novel_006957	XLOC_07181.	transcript	0.084985	0.185856	0.215233	0.132289	0.134114	0.139217	0.177635	0.136018	0.213628
Novel_006958	XLOC_07181.	transcript	0	0	0	0	0	0	0	0	0
Novel_006959	XLOC_07181.	transcript	0.05644	0.181705	0.058086	0.117647	0	0	0.133524	0.029697	0.080869
Novel_006960	XLOC_07181.	transcript	0.611825	0.22511	0.396951	0.393105	0.608403	0.80824	0.610146	0.533401	0.007901
Novel_006961	XLOC_07182.	transcript	0.098768	0.23297	0	0.123354	0.264854	0	0.156975	0	0
Novel_006962	XLOC_07182.	transcript	0.002812	0.013184	0	0.100188	0.070894	0.425889	0.011899	0	0.328052
Novel_006963	XLOC_07182.	transcript	0.130369	0.13984	0.090887	0	0.185202	0	0	0.20077	0
Novel_006964	XLOC_07182.	transcript	0.051556	0.158476	0.112925	0.133214	0	0	0.113265	0.159646	0.036615
Novel_006965	XLOC_07182.	transcript	0.051588	0.261952	0.08291	0.095195	0	0	0.122348	0.043106	0.01252
Novel_006966	XLOC_07182.	transcript	0.062193	0.155968	0.221018	0.119805	0.072905	0.121978	0.108523	0.145992	0.306967
Novel_006967	XLOC_07183.	transcript	0.485942	1.644923	0.732017	0.80948	0.76304	0.977156	0.696322	1.302058	0.787853
Novel_006968	XLOC_07185.	transcript	0	0	0	0.366403	0	0.31316	0.12754	0.057586	0.93802
Novel_006969	XLOC_07185.	transcript	0	0	0	0	0	0	0	0	0
Novel_006970	XLOC_07185.	transcript	0	0	0	0	0	0	0	0	0
Novel_006971	XLOC_07185.	transcript	0.995502	1.027834	0.791256	1.007677	1.28338	1.251281	1.202821	1.212829	0.316045
Novel_006972	XLOC_07185.	transcript	0.365345	0.003049	0.226538	0.16781	0	0.001989	0.001233	0.001869	0.001954
Novel_006973	XLOC_07185.	transcript	0.265388	0.101982	0.379384	0.295915	0.271812	0.393542	0.270962	0.356463	0.271097
Novel_006974	XLOC_07196.	transcript	0.897732	0.182384	0.805911	0.483634	0.932198	0.3942	0.688736	0.776854	0.310175
Novel_006975	XLOC_07198.	transcript	0.100848	0	0	0.248287	0	0	0.242922	0.184305	0.360106
Novel_006976	XLOC_07198.	transcript	0	0.040695	0.024522	0	0	0.130599	0.235602	0	0
Novel_006977	XLOC_07198.	transcript	0.060197	0	0.022018	0.05045	0.021372	0.025212	0.074157	0.152111	0.095755
Novel_006978	XLOC_07200.	transcript	6.042014	5.345634	10.58343	13.37295	12.05605	17.36596	13.84667	15.26284	12.53105
Novel_006979	XLOC_07200.	transcript	2.336374	0.021771	2.794993	3.899115	2.406522	1.479111	2.332881	4.426033	2.922448
Novel_006980	XLOC_07200.	transcript	0.543635	0.925018	0.74065	0.696738	0.527955	0.817336	0.903677	0.745621	0.66987

Novel_006981	XLOC_07201.	transcript	0.992219	0.441991	1.510495	2.061887	1.299923	1.381279	1.864428	1.727025	1.701986
Novel_006982	XLOC_07201.	transcript	0	1.070424	0	0	0	0	0	0	0
Novel_006983	XLOC_07201.	transcript	0	0.013192	0.026281	0.006275	0.032529	0.015906	0.052977	0.011029	0.025566
Novel_006984	XLOC_07205.	transcript	0	0.065141	0.109931	0	0.012645	0	0.014933	0.028479	0
Novel_006985	XLOC_07206.	transcript	0	0.256917	0.081036	0	0	0.139047	0.03697	0	0
Novel_006986	XLOC_07206.	transcript	0.811765	0.318071	0.765908	0.823018	0.918372	0.701701	1.518839	1.068389	0.964808
Novel_006987	XLOC_07207.	transcript	0	2.428129	0	0	0	0	0	0	0
Novel_006988	XLOC_07207.	transcript	0.00121	0.009111	0.702117	0.003007	2.00E-06	0.912175	0.306053	0.23003	0.017367
Novel_006989	XLOC_07207.	transcript	0.638108	0.122627	0.134917	0.508964	0.977097	0.007879	0.268591	0.44248	1.224418
Novel_006990	XLOC_07207.	transcript	0	0	0	0.450879	0.018544	0.030244	0.318234	0.133199	0
Novel_006991	XLOC_07207.	transcript	0.065725	0.049386	0	0	0.00792	0.149499	0.25273	0	0
Novel_006992	XLOC_07207.	transcript	0	0	0	0	0	0	0.27622	0.250569	0.010632
Novel_006993	XLOC_07207.	transcript	0.029226	0	0	0	0	0	0.329141	0.151587	0.022629
Novel_006994	XLOC_07207.	transcript	0	0	0	0	0	0	0.297744	0.415752	0
Novel_006995	XLOC_07207.	transcript	0.378722	0.785278	0.745845	0.546815	0.54568	1.392882	0.118795	0.131335	0.932265
Novel_006996	XLOC_07207.	transcript	0.138696	0.092411	0.103078	0.098902	0.067903	0.159998	0.112096	0.19145	0.133697
Novel_006997	XLOC_07207.	transcript	0.148524	0.167386	0.143871	0.155639	0.114201	0.164616	0.153937	0.125294	0.127793
Novel_006998	XLOC_07207.	transcript	0.696122	0.410984	0.430728	0.817797	0.68052	0.412079	0.656588	0.930744	0.405019
Novel_006999	XLOC_07207.	transcript	0.15053	0.210052	0.156351	0.173092	0.121413	0.176627	0.230704	0.174504	0.133956
Novel_007000	XLOC_07207.	transcript	0.080964	0.073903	0.078258	0.070059	0.06082	0.065884	0.115713	0.088493	0.079266
Novel_007001	XLOC_07207.	transcript	0	0	0	0	0	0	0.269615	0.164562	0
Novel_007002	XLOC_07207.	transcript	0.541702	0.474846	0.705489	0.432318	0.432928	0.563728	0.61479	0.519406	0.69121
Novel_007003	XLOC_07207.	transcript	0	0.089574	0.235381	0.385996	0.304252	0.268316	0.486035	0.430304	0.289918
Novel_007004	XLOC_07207.	transcript	0.090387	1.157768	0.314406	0.598569	0.497844	0.227407	0	0	0.340113
Novel_007005	XLOC_07207.	transcript	1.55225	0.926168	0.875537	1.005218	1.141915	0.815586	0.717662	1.499675	1.039909
Novel_007006	XLOC_07208.	transcript	0.095553	0.15766	0.357956	0.397211	0.244653	0.251266	0.241413	0.286605	0.311937
Novel_007007	XLOC_07208.	transcript	0.294496	0.490299	0.366723	0.463573	0.463125	0.435065	0.471326	0.256952	0.323744
Novel_007008	XLOC_07208.	transcript	0.199144	0.314479	0.227406	0.063523	0.054567	0.143985	0.165991	0.317263	0.183608
Novel_007009	XLOC_07209.	transcript	0.493888	0.336671	0.261752	0.563208	0.524494	0	0.493513	0.530764	0
Novel_007010	XLOC_07209.	transcript	0	0.005294	0.155364	0.335573	0.089509	0.353989	0.183656	0.27746	0.726227
Novel_007011	XLOC_07209.	transcript	0	0	0	0	0	0.352533	0.079082	0	0
Novel_007012	XLOC_07209.	transcript	0	0.400867	0	0	0	0	0.127662	0	0
Novel_007013	XLOC_07209.	transcript	0.069614	1.572492	0.017938	0.033457	0.028556	0.150722	0.105978	0.044098	0.065292
Novel_007014	XLOC_07210.	transcript	0.566701	0.972058	1.066877	1.141192	1.065778	1.186055	1.101478	1.181936	1.277757
Novel_007015	XLOC_07210.	transcript	0.177945	0.231336	0.28106	0.345462	0.390373	0.315773	0.516445	0.429941	0.249051
Novel_007016	XLOC_07211.	transcript	0	0.6608	0.043345	0.020617	0.033212	0	0.049552	0.009374	0
Novel_007017	XLOC_07211.	transcript	0	0	0	0	0	0	0	20.59916	0
Novel_007018	XLOC_07211.	transcript	24.76065	26.0097	39.6874	50.17374	35.57328	52.42757	31.20766	19.01412	38.49327

Novel_007019	XLOC_07212.	transcript	0.16988	0.262473	0.250574	0	0.081949	0	0.139514	0.063149	0.904502
Novel_007020	XLOC_07212.	transcript	0.245367	0.875462	0.118576	0.119224	0.250443	0	0.33687	0.158424	0
Novel_007021	XLOC_07213.	transcript	0.056864	1.235836	0.065729	0.0663	0.070799	0.043328	0.09689	0.103229	0.10361
Novel_007022	XLOC_07213.	transcript	0.369386	0.039267	0	0	0.397328	1.00E-06	0.414251	0	0
Novel_007023	XLOC_07213.	transcript	0.006231	0	0.526856	0.302175	0.240219	0.655927	0.430283	0.678022	0.549254
Novel_007024	XLOC_07213.	transcript	0.011712	0	0.017163	0.004726	0.004815	0.03241	0.025299	0.002397	0.019008
Novel_007025	XLOC_07213.	transcript	0.001329	0.525387	0.000629	0	0.000615	0.00175	0.002897	2.00E-06	0
Novel_007026	XLOC_07213.	transcript	0	0.013017	0	0.110796	0	0	0	0	0
Novel_007027	XLOC_07213.	transcript	2.092757	0.402981	1.685914	1.230438	1.554646	1.255356	6.252748	1.776714	29.43287
Novel_007028	XLOC_07213.	transcript	0.22635	0.335265	0.179233	0.078595	0.271295	0.228478	0.402567	0.118669	0.190932
Novel_007029	XLOC_07214.	transcript	0.175937	0.020392	0.269864	0.092224	0.303765	0.163643	0.317411	0.141018	0.173011
Novel_007030	XLOC_07214.	transcript	0.082447	0	0.632358	0.671534	0.514049	0.269853	0.329386	0.605076	0.375777
Novel_007031	XLOC_07214.	transcript	0	0.049316	0.017692	0.052493	0.024645	0	0.073128	0.008304	0.3109
Novel_007032	XLOC_07214.	transcript	0.18598	0.120478	0.892069	0.634514	0.886432	0.75141	0.973965	0.545944	0.696568
Novel_007033	XLOC_07214.	transcript	0.06814	0.073353	0.136126	0.218361	0.365612	0.060263	0.175984	0.141335	0.116921
Novel_007034	XLOC_07214.	transcript	0.455713	0.151023	0.092245	0.074738	0.235267	0.123133	0.125525	0.045164	0.081945
Novel_007035	XLOC_07215.	transcript	0.555367	0.147432	0	0.119878	0.279845	0.254062	0	0	0.134223
Novel_007036	XLOC_07215.	transcript	0.197472	0.417322	0.213977	0.094221	0.214957	0.276651	0.29043	0.181548	0.180288
Novel_007037	XLOC_07215.	transcript	0	0.259198	0	0	0.325962	0.086402	0.206556	0.256805	0.129351
Novel_007038	XLOC_07215.	transcript	0.481553	0.335571	0.515211	0.49088	0	0.355514	0.497023	0.430005	0.88379
Novel_007039	XLOC_07215.	transcript	0	0.151722	0.334689	0.318037	0	0.26924	0.343273	0.229912	0
Novel_007040	XLOC_07215.	transcript	0.177039	0	0	0	0.658621	0.264024	0	0.028617	0.090046
Novel_007041	XLOC_07215.	transcript	0.957645	2.161168	0	0.146639	0	0	0.508622	2.45897	0
Novel_007042	XLOC_07215.	transcript	1.330129	0.958056	0.144041	0	1.9404	0	0.530403	1.528365	0
Novel_007043	XLOC_07215.	transcript	4.713301	6.518713	9.37901	15.72124	13.91574	16.87538	11.71759	9.347933	9.203766
Novel_007044	XLOC_07216.	transcript	0.164383	0.029735	0.181092	0.277905	0.222072	0.445246	0.282719	0.183485	0
Novel_007045	XLOC_07216.	transcript	0.14768	0.335836	0.00548	0.012508	0.040124	0.007256	0.036235	0.213176	0.27289
Novel_007046	XLOC_07216.	transcript	0	0	0	0.117942	0.002023	0	0	0	0.001857
Novel_007047	XLOC_07216.	transcript	0.085533	0.019817	0.033886	0.031816	0.142308	0.056831	0.183018	0.119858	0.070561
Novel_007048	XLOC_07218.	transcript	1.603887	1.285128	0.953202	1.139819	1.197973	0.552134	1.570706	0.791027	0.603305
Novel_007049	XLOC_07218.	transcript	0.706299	0.839926	0.559537	0.518146	0.836128	1.148296	1.304045	1.05793	0.792367
Novel_007050	XLOC_07219.	transcript	0.276717	0.509925	0	0	0.445919	0.277546	0.377386	0.220802	0.104491
Novel_007051	XLOC_07219.	transcript	0	0.006201	0	0.133715	0	0	0.003341	0	0
Novel_007052	XLOC_07219.	transcript	0.164858	0.287786	0.728996	0.45309	0.1397	0.205737	0.345692	0.50817	0.267135
Novel_007053	XLOC_07219.	transcript	0	0	0	0	0	0	0	0	0.166051
Novel_007054	XLOC_07219.	transcript	0	0	0	0.138575	0	0.213799	0	0	0
Novel_007055	XLOC_07224.	transcript	0.025966	0	0.126698	0.1202	0.273033	0.031433	0.054983	0.095563	0.045923
Novel_007056	XLOC_07225.	transcript	0	0	0	0	0	0	0.090118	0.029844	0.092662

Novel_007057	XLOC_07225.	transcript	0.82673	0.194067	0.553435	0.097056	0.753401	0.614395	1.474444	0.890071	0.633939
Novel_007058	XLOC_07225.	transcript	0	1.182133	0.00183	0.483752	0.004004	0.00528	0.006554	0	0
Novel_007059	XLOC_07227.	transcript	3.006234	1.421509	4.884444	5.068556	5.951474	6.266937	4.781052	5.371885	3.116755
Novel_007060	XLOC_07227.	transcript	0	0	0	0	0.784727	0	0.375238	0.050499	0.084802
Novel_007061	XLOC_07227.	transcript	0.307876	0.056295	0.403428	0.255574	0.644574	0.548819	0.448946	0.384946	0
Novel_007062	XLOC_07227.	transcript	0.275914	0.035189	0.0743	0.109759	0.061663	0.158429	0.218266	0.044348	0.171944
Novel_007063	XLOC_07228.	transcript	0	0.016749	0.344629	0.030071	0.106529	0.014157	0.035349	0	0.018581
Novel_007064	XLOC_07228.	transcript	0.414164	0.000541	0.119457	0.00998	0.172794	0.049537	0.323514	0.052113	0.113466
Novel_007065	XLOC_07228.	transcript	0	0	0	0.133028	0.002977	0.238245	0.181221	0.150488	0
Novel_007066	XLOC_07228.	transcript	0.047262	0.009564	0.010393	0.159129	0.110437	0.042197	0.26239	0.121702	0.070015
Novel_007067	XLOC_07230.	transcript	2.748946	4.105213	2.856551	3.657717	3.592549	3.260051	2.972512	4.177725	2.544213
Novel_007068	XLOC_07230.	transcript	0.377576	0.336756	0.476246	0.430438	0.558884	0.340508	0.583111	0.701622	0.531623
Novel_007069	XLOC_07231.	transcript	0	0	0	0	0	0	0	0	0
Novel_007070	XLOC_07233.	transcript	0.141118	0	0	0.498246	0.518897	0	0.388593	0.15819	0.306172
Novel_007071	XLOC_07233.	transcript	0.03729	0	0.002415	0.00122	0.004598	0	0	0	0.029862
Novel_007072	XLOC_07233.	transcript	0.061423	0.129579	0.309826	0.046045	0.051142	0.370458	0.078775	0.169129	0.058812
Novel_007073	XLOC_07245.	transcript	0.492834	0.351738	0.214235	0.175572	0.158551	0.258677	0.357442	0.361571	0.206658
Novel_007074	XLOC_07245.	transcript	0	0	0.033271	0.080693	0.008342	0	0	0	0
Novel_007075	XLOC_07246.	transcript	0.480539	1.085134	0.670962	0.547133	0.459872	0.863643	0.698871	0.529746	0.532939
Novel_007076	XLOC_07246.	transcript	0.161384	0	0.13652	0.466735	0.229138	0.132878	0.217394	0.442673	0.085071
Novel_007077	XLOC_07247.	transcript	0	0	0	0	0	0	0	0	0
Novel_007078	XLOC_07250.	transcript	1.859294	0.302818	0.234733	0.552367	1.037067	1.914418	0.108712	0.508805	2.181692
Novel_007079	XLOC_07250.	transcript	0	2.941711	1.321483	0.39941	0.62085	0	2.246911	0.303102	3.176014
Novel_007080	XLOC_07252.	transcript	0.324701	0.263536	0.170317	0.15122	0.142902	0.362481	0.316005	0.289447	0.241227
Novel_007081	XLOC_07252.	transcript	0.908915	4.532309	1.254879	1.271813	1.195682	0.905806	1.136991	1.550359	0.800951
Novel_007082	XLOC_07252.	transcript	0.614667	0.469198	0.238857	0.220054	0.232165	0.244265	0.420951	0.292624	0.391694
Novel_007083	XLOC_07255.	transcript	0.054511	0.649907	0.356201	0.100428	0.336626	0.0795	0	0.075611	0
Novel_007084	XLOC_07257.	transcript	0.980998	0.47575	0.953086	1.099475	0.926246	0.638686	1.027664	1.243159	0.81833
Novel_007085	XLOC_07257.	transcript	0.093281	0.012382	0.305832	0.255254	0.132246	0.19961	0.119707	0.215836	0.215105
Novel_007086	XLOC_07257.	transcript	0	0	0.236571	0	0	0	0	0.202595	0
Novel_007087	XLOC_07257.	transcript	0	0	0.503596	0.438089	0.698008	0.288659	0.377825	1.032522	0.377387
Novel_007088	XLOC_07257.	transcript	0.029126	0	0	0.000776	0.250793	0.362039	0.405841	0.148688	0
Novel_007089	XLOC_07257.	transcript	0.297665	0.019255	0.034007	0	0	0	0	0	0.096807
Novel_007090	XLOC_07257.	transcript	0	0	0.038591	0.63727	0.007657	0.02599	0.008558	0.008358	0.008167
Novel_007091	XLOC_07257.	transcript	0	0	0	0	0	0.140632	0.372083	0.139514	0
Novel_007092	XLOC_07257.	transcript	0.327351	0	0.229311	0.208001	0.38104	0.659419	0.269344	0.232522	0.390833
Novel_007093	XLOC_07257.	transcript	0.120755	0	0.347849	0.251104	0	0.117921	0.098726	0.142991	0.229129
Novel_007094	XLOC_07257.	transcript	0.14234	0	0.244001	0.144936	0	0.125273	0	0	0

Novel_007095	XLOC_07257.	transcript	0.104818	0	0.166113	0.13071	0	0.438029	0.033763	0	0.066312
Novel_007096	XLOC_07257.	transcript	0.141196	0	0.18517	0	0.396248	0.312139	0.327805	0.343346	0
Novel_007097	XLOC_07257.	transcript	0.055798	0	0.075903	0.773372	0	0	0	0.655154	0.311013
Novel_007098	XLOC_07259.	transcript	0.225349	0	0.036154	0.123941	0	0	0.037089	0.144105	0.159215
Novel_007099	XLOC_07261.	transcript	0.428849	0.268205	0.290147	0.331338	0.264733	0.250151	0.392672	0.279964	0.167795
Novel_007100	XLOC_07261.	transcript	0	0	0	0	0	0	0	0	0
Novel_007101	XLOC_07262.	transcript	0.071442	0.027009	0.039571	0.090334	0.118754	0.00164	0	0.189021	0.189098
Novel_007102	XLOC_07262.	transcript	0	0	0	0	0.100993	0.0849	0.114527	0	0
Novel_007103	XLOC_07263.	transcript	0.167104	0.187703	0.162033	0.18171	0.272987	0.049488	0.236245	0.214183	0.170399
Novel_007104	XLOC_07263.	transcript	0	0.003436	0	0	0	0	0	0	0
Novel_007105	XLOC_07265.	transcript	0.254064	0.112997	0.180774	0.417852	0.347342	0	0.054466	0.484268	0
Novel_007106	XLOC_07265.	transcript	0	0.121048	0.287891	0.002307	0.008593	0.376862	0.480366	0.011092	0.235248
Novel_007107	XLOC_07265.	transcript	0	0	0	0	0	0.029643	0	0	0.021203
Novel_007108	XLOC_07269.	transcript	0.077257	0.133584	0.140834	0.127065	0.099944	0.2102	0.153457	0.166361	0.030334
Novel_007109	XLOC_07271.	transcript	0.975914	0.216868	1.00581	0.904042	1.603998	0.780861	0.972001	1.452094	0.589403
Novel_007110	XLOC_07271.	transcript	0	0	0	0	0	0	0	0	0
Novel_007111	XLOC_07271.	transcript	0	0	0	0	0	0	0.528949	0.310856	0.422779
Novel_007112	XLOC_07271.	transcript	0	0	0	0	0	0	0	0	1.062941
Novel_007113	XLOC_07273.	transcript	0.66684	0.481703	0.549483	0.63197	0.702179	0.642798	1.017416	0.853986	0.906293
Novel_007114	XLOC_07273.	transcript	0	0	0	0	0	0.050401	0	0	0
Novel_007115	XLOC_07312.	transcript	0.297468	0.121756	0.207046	0.076502	0.262889	0.17182	0.135293	0.117639	0.18682
Novel_007116	XLOC_07313.	transcript	0.329461	0.486248	0.259035	0.27735	0.215486	0.297531	0.38705	0.352426	0.179574
Novel_007117	XLOC_07332.	transcript	0.232079	0.411474	0.298455	0.309499	0.239217	0.348243	0.245063	0.238418	0.238414
Novel_007118	XLOC_07334.	transcript	3.011307	0.134228	2.681474	3.749437	2.685694	5.882386	3.453993	3.256898	1.789883
Novel_007119	XLOC_07334.	transcript	0.09221	0	0	0.313068	0.228493	0.096628	0.256268	0.007836	0.146274
Novel_007120	XLOC_07334.	transcript	0.593594	0.010222	0.354512	0.117415	0.072605	1.022126	0.086499	0.294944	0.381404
Novel_007121	XLOC_07334.	transcript	0.376628	0.042103	0.26758	0.402013	0.229864	0.740186	0.380116	0.000275	0.115359
Novel_007122	XLOC_07334.	transcript	0.01128	0	0.002092	0.003077	0	0	0.002039	0.005585	0.004886
Novel_007123	XLOC_07334.	transcript	0.502463	0	0.320037	0.302887	0.333301	0.598092	0.425473	0.65774	0.396984
Novel_007124	XLOC_07334.	transcript	0.30533	0	0.414405	0.343939	0.298592	0.351206	0.296092	0.14826	0.157907
Novel_007125	XLOC_07334.	transcript	0	0	0.142709	0.139248	0.231875	3.00E-06	0.21909	0	0
Novel_007126	XLOC_07334.	transcript	0.292442	0	0.071763	0.269021	0	0.210457	0.104442	0.282359	0.144065
Novel_007127	XLOC_07334.	transcript	0	0	0.394791	0	0	1.551075	0	0	0
Novel_007128	XLOC_07334.	transcript	0.154669	0	0.094888	0.278177	0.087411	0.529742	0.152426	0.000562	0.113245
Novel_007129	XLOC_07334.	transcript	0	0	0	0	0.502123	0	0	0	0
Novel_007130	XLOC_07334.	transcript	0.029291	0	0.05311	0.025859	0.048399	0.2218	0.118482	0.225189	0
Novel_007131	XLOC_07334.	transcript	0	0	0	0	0.342885	0.113173	0.149957	0	0
Novel_007132	XLOC_07334.	transcript	0.112019	0	0.036152	0.149728	0.04029	0.488418	0.130674	0.076796	0.107647

Novel_007133	XLOC_07334.	transcript	0	0	0.065838	0	0.043592	0.165727	0.017921	0.027322	0.050703
Novel_007134	XLOC_07334.	transcript	0.170726	0	0.086753	0.179432	0.184444	0.357427	0.165867	0.23955	0.137812
Novel_007135	XLOC_07334.	transcript	0.080869	0	0.104789	0.230534	0.172691	0.359852	0.119913	0.133243	0.135078
Novel_007136	XLOC_07334.	transcript	0.186795	0.059605	0.250245	0.239868	0.091555	0.299532	0.024663	0.024387	0.079144
Novel_007137	XLOC_07334.	transcript	0.063859	0	0	0.24897	0.048438	0.318555	0	0	0
Novel_007138	XLOC_07334.	transcript	0	0.02969	0.039283	0	0.014075	0	0.154435	0.041723	0.032713
Novel_007139	XLOC_07334.	transcript	0	0	0.156987	0.297069	0.405073	0	0.023319	0	0.015484
Novel_007140	XLOC_07334.	transcript	0.010435	0.103211	0.014643	0	0	0.120621	0.171299	0.140106	0.049884
Novel_007141	XLOC_07334.	transcript	0	0	0	0	0	0	0.173669	0.126307	0
Novel_007142	XLOC_07334.	transcript	0.124286	0.114582	0.177356	0	0	0.136499	0.030675	0.004021	0.07083
Novel_007143	XLOC_07334.	transcript	0.302828	0.326641	0	0	0	0	0.10807	0	0.194237
Novel_007144	XLOC_07334.	transcript	0	0	0	0	0	0	0.090772	0	0
Novel_007145	XLOC_07338.	transcript	0.159329	0.116481	0.072836	0.103782	0.114943	0.058337	0.051467	0.134626	0.069368
Novel_007146	XLOC_07345.	transcript	0.482882	0.485245	0.391535	0.557426	0.514447	0.465675	0.431921	0.898283	0.515397
Novel_007147	XLOC_07346.	transcript	0.119185	0.378357	0.131238	0.171141	0.212995	0.188157	0.137802	0.107314	0.170853
Novel_007148	XLOC_07346.	transcript	0.241611	0.117392	0.069074	0.196801	0.133001	0.110361	0.437104	0.134269	0.166683
Novel_007149	XLOC_07346.	transcript	0.225925	0.051119	0.053705	0.085711	0.160612	0.073616	0.390048	0.08477	0.089273
Novel_007150	XLOC_07346.	transcript	0	0	0.023917	0.021521	0	0	0	0	0
Novel_007151	XLOC_07346.	transcript	0	0	0	0	0	0	0.000101	0.169102	0
Novel_007152	XLOC_07347.	transcript	0	0	0	0	0.077779	0.091752	0.042656	0.214942	0.045062
Novel_007153	XLOC_07347.	transcript	0.522125	0	0	0.069155	0.027369	0.436724	0	0.779291	0
Novel_007154	XLOC_07347.	transcript	0.609216	0	0.020456	0.013447	0.307929	0.060419	0.185464	0.019546	0.029317
Novel_007155	XLOC_07347.	transcript	0	0.146235	0.69392	0.522824	0	0.107934	0.308847	0	0.376915
Novel_007156	XLOC_07347.	transcript	0	0	0	0	0	0	0	0	0
Novel_007157	XLOC_07347.	transcript	0	0	0	0	0.43966	0	0	0	0
Novel_007158	XLOC_07350.	transcript	0	0.235944	0	0	0	0	0	0	0
Novel_007159	XLOC_07351.	transcript	0	0	0	0	0	0	0	0.040528	0
Novel_007160	XLOC_07351.	transcript	0	0	0	0.01307	0	0.012485	0	0.000508	0.010666
Novel_007161	XLOC_07352.	transcript	5.306723	0.002782	2.836613	1.955075	2.387481	3.142782	4.553213	3.441245	1.247821
Novel_007162	XLOC_07352.	transcript	4.503022	0	1.392841	0.253443	0.30002	0.289551	1.105129	0.998085	0.206468
Novel_007163	XLOC_07371.	transcript	0.229996	0.380241	0.147042	0.275567	0.22054	0.206893	0.179202	0.278982	0.099939
Novel_007164	XLOC_07388.	transcript	0.119994	0	0	0.07897	0.048185	0.358513	0	0.203292	0.111667
Novel_007165	XLOC_07393.	transcript	0	0	0.005817	0.040241	0.007624	0	0	0	0.264157
Novel_007166	XLOC_07393.	transcript	0	0.168798	0.082056	0.053091	0	0	0	0	0
Novel_007167	XLOC_07393.	transcript	0	0.21514	0	0.037681	0	0	0	0	0
Novel_007168	XLOC_07393.	transcript	0.140129	0.198491	0.318928	0.066783	0.328267	0.183377	0.147477	0.08792	0
Novel_007169	XLOC_07393.	transcript	0.271377	0.182316	0	0.038339	0	0.159404	0.380954	0.233083	0
Novel_007170	XLOC_07393.	transcript	0	0	0	0.037652	0	0	0	0	0

Novel_007171	XLOC_07394.	transcript	0.154657	0.33888	0	0.212146	0.082188	0.082824	0.277141	0.161029	0.380774
Novel_007172	XLOC_07397.	transcript	0.298372	0.684401	0.420399	0.294818	0.331541	0.350754	0.428169	0.249808	0.270256
Novel_007173	XLOC_07397.	transcript	0.461703	0.316071	0.424748	0.491718	0.454161	0.565675	0.50856	0.818276	0.547727
Novel_007174	XLOC_07397.	transcript	0	0.022968	0.270135	0.001453	0.556433	0.024569	0.099392	0.122807	0.087457
Novel_007175	XLOC_07397.	transcript	0.249238	0	0.008745	0	0	0.183686	0	0	0
Novel_007176	XLOC_07397.	transcript	0	0	0.173656	0	0	0	0.211887	0	0
Novel_007177	XLOC_07397.	transcript	0.282974	0	0	0	0	0.201914	0	0.172607	0
Novel_007178	XLOC_07397.	transcript	0.064719	0.185576	0.109747	0.111266	0.103715	0.101723	0.309042	0.046941	0.353154
Novel_007179	XLOC_07405.	transcript	0	0.541767	0.019192	0	0	0	0	0.008159	0.047704
Novel_007180	XLOC_07405.	transcript	0.019648	0.046978	0.045163	0	0.012762	0	0	0.012446	0.001406
Novel_007181	XLOC_07405.	transcript	0.249666	0.707975	0.29554	0.173104	0.356849	0.53854	0.394629	0.281557	0.513338
Novel_007182	XLOC_07405.	transcript	0.181142	0.015813	0	0	0	0	0	0.094753	0.003962
Novel_007183	XLOC_07405.	transcript	0.175151	0.530067	0.200699	0.086767	0.103719	0.129871	0.021451	0.087898	0.057337
Novel_007184	XLOC_07405.	transcript	0.168495	0.145655	0	0	0.26601	0.064668	0.157012	0.27795	0.140676
Novel_007185	XLOC_07409.	transcript	0	0.182882	0.104682	0	0.024701	0	0	0.168098	0.000468
Novel_007186	XLOC_07409.	transcript	0.092988	0.277774	0.062396	0.025992	0.188081	0	0.201018	0.010543	0.201355
Novel_007187	XLOC_07410.	transcript	0.126264	0.589831	0.158954	0.052485	0.20539	0.234115	0.168898	0.279937	0.210836
Novel_007188	XLOC_07415.	transcript	0.035147	0.477601	0.027249	0.009786	0.076889	0.028271	0.043811	0.033185	0.069747
Novel_007189	XLOC_07416.	transcript	0	0	0	0	0	0	0	0.49247	0
Novel_007190	XLOC_07416.	transcript	1.385209	0.039564	1.138603	0.783787	1.564736	1.10018	1.091964	0.380134	0.54694
Novel_007191	XLOC_07416.	transcript	0.007034	0.024597	0.024547	0.273856	0.064182	0.052809	0.540579	0.918851	0.010469
Novel_007192	XLOC_07416.	transcript	0	0	0	0.00773	0	0	0	0	0
Novel_007193	XLOC_07416.	transcript	0.013494	0.015389	0.202005	0	0.102988	0.15768	0.938948	0.538216	0
Novel_007194	XLOC_07416.	transcript	0	0	0.000374	0	0	0	0	0.005202	1.057402
Novel_007195	XLOC_07421.	transcript	0.065875	0.024235	0.414688	0.401614	0.564829	0.556375	0.028361	0.40752	0.348975
Novel_007196	XLOC_07421.	transcript	0.002771	0	0	0.003449	0	0	0.042802	0.014527	0.073289
Novel_007197	XLOC_07421.	transcript	0.649718	0.374078	0	0	0	0	0.287127	0.168369	0
Novel_007198	XLOC_07421.	transcript	0	0	0	0	0	0	0.578481	0	0
Novel_007199	XLOC_07422.	transcript	0.187652	1.958682	0.099864	0.102568	0.098342	0.137467	0.277373	0.125016	0.249348
Novel_007200	XLOC_07422.	transcript	0	0.028533	0	0	0	0	0.010826	0	0
Novel_007201	XLOC_07430.	transcript	0.165219	0.179607	0.578852	0.343373	0.280698	0.34634	0.399167	0.382815	0.230987
Novel_007202	XLOC_07430.	transcript	0	0	0	0	0	0	0	0	0
Novel_007203	XLOC_07431.	transcript	0.430556	0.209002	0	0.454795	0.34812	0.4072	0.283973	0.406409	0.222325
Novel_007204	XLOC_07431.	transcript	0	0.211521	0.375648	0	0	0	0	0	0
Novel_007205	XLOC_07434.	transcript	0	0.11872	0.242915	0	0	0	0.029536	0	0
Novel_007206	XLOC_07434.	transcript	0.632232	0.338587	0.433342	0.563953	0.5597	0.745283	0.235145	0.857979	0.56976
Novel_007207	XLOC_07434.	transcript	0	0	0	0	0	0	0.655353	0	0
Novel_007208	XLOC_07434.	transcript	0	0	0.197931	0	0.160594	0	0	0.071763	0

Novel_007209	XLOC_07436.	transcript	0.181713	0	0.185825	0.20014	0.059924	0.042767	0.164246	0.116848	0.294292
Novel_007210	XLOC_07438.	transcript	0.309934	2.756745	0.30604	0.21382	0.277079	0.351776	0.43311	0.261756	0.483626
Novel_007211	XLOC_07439.	transcript	0.117466	0.153132	0.199394	0.158372	0.186054	0.419233	0.215609	0.176856	0.109485
Novel_007212	XLOC_07446.	transcript	0.864602	1.630632	1.44405	1.692914	0.6518	0.975505	1.426445	1.343624	1.168449
Novel_007213	XLOC_07446.	transcript	0.437961	0.061549	0.05504	0.036334	0.739154	0.700779	0.482881	0.514138	0.781847
Novel_007214	XLOC_07446.	transcript	0.199725	0.090653	0.228857	0.186935	0.178741	0.185943	0.447343	0.149331	0.404588
Novel_007215	XLOC_07453.	transcript	0.118455	0	0.075189	0.019869	0	0.028603	0.092685	0.06108	0.056191
Novel_007216	XLOC_07456.	transcript	0.310439	0	0.33852	0.114901	0.18452	0.061872	0.063155	0.144248	0.289704
Novel_007217	XLOC_07458.	transcript	2.293674	0.369639	2.676096	5.347859	4.15035	1.945358	3.22894	2.950264	3.45116
Novel_007218	XLOC_07469.	transcript	0	0	0	0	0.088568	0.052327	0.048654	0.024831	0.102797
Novel_007219	XLOC_07469.	transcript	0.880623	0.002412	0.044362	0.005982	0.010956	0.001755	0.68338	0.585253	0.481672
Novel_007220	XLOC_07469.	transcript	0	0.008334	0	0.018817	0	0	0	0.006646	0
Novel_007221	XLOC_07469.	transcript	0	0	0	0	0	0	0	0	0
Novel_007222	XLOC_07469.	transcript	0.139662	0.632652	0.088345	0.32487	0.076747	0.710631	0.028401	2.00E-06	0
Novel_007223	XLOC_07469.	transcript	0	0	0	0.116009	0.488541	0	0	0.000126	0
Novel_007224	XLOC_07469.	transcript	0.018871	0	0	0.00056	0.00326	0	0.014392	0.02158	0
Novel_007225	XLOC_07469.	transcript	0	0	1.141754	0	0	0	0	0	0.01752
Novel_007226	XLOC_07469.	transcript	0.138695	0.034493	0.034782	0.34123	0.539847	0.111924	0.464606	0.670801	0.409514
Novel_007227	XLOC_07469.	transcript	0.474643	0.560069	0.332443	0.133209	0.206296	0.274823	0.356698	0.395291	0.19777
Novel_007228	XLOC_07475.	transcript	0.180521	0.382467	0.38633	0.379842	0.523107	0.308667	0.463556	0.404451	0.518535
Novel_007229	XLOC_07480.	transcript	0	0.24194	0	0	0	0	0	0	0
Novel_007230	XLOC_07480.	transcript	0	0.050998	0	0	0	0.125285	0	0	0.003907
Novel_007231	XLOC_07480.	transcript	0.031585	0.000991	0.10738	0.001206	0.172495	0.008352	0.011155	0.049164	0.006934
Novel_007232	XLOC_07480.	transcript	0.087713	0.241832	0.011964	0.078424	0	0	0.001483	0.018235	0.0141
Novel_007233	XLOC_07480.	transcript	0.0887	0.075378	0.008091	0.044744	0.018265	0.015267	0.034511	0.045109	0.092925
Novel_007234	XLOC_07480.	transcript	0	0	0.094702	0.010254	0.056409	0	0.139847	0	0.070611
Novel_007235	XLOC_07480.	transcript	0	0	0	0	0	0	0	0	0
Novel_007236	XLOC_07480.	transcript	0.117648	0.132555	0.002392	0.022943	0.004597	0.106262	0.125025	0.010863	0.012079
Novel_007237	XLOC_07480.	transcript	0.002856	0	0	0	0	0	0	0.10501	0
Novel_007238	XLOC_07483.	transcript	0.026851	0.025663	0.005216	0.010211	0.007562	0.266168	0.027217	0.008926	0.046215
Novel_007239	XLOC_07483.	transcript	0.977283	0.320382	0.722704	0.617928	0.723841	0.473592	0.948974	0.681983	0.429147
Novel_007240	XLOC_07483.	transcript	0	0	0	0	0	0	0	0	0.001062
Novel_007241	XLOC_07483.	transcript	0	0	0	0.000316	0	0	0	0	0
Novel_007242	XLOC_07484.	transcript	0.613542	0.233985	1.105998	1.587552	1.042866	0.996716	1.229074	1.180941	0.619029
Novel_007243	XLOC_07500.	transcript	0.365991	0.121934	0.148941	0.051204	0.20099	0.331268	0.102477	0.133881	0.151774
Novel_007244	XLOC_07500.	transcript	0.001203	0	0.028074	0.186772	0	0	0	0	0
Novel_007245	XLOC_07500.	transcript	0.012039	0	0	0.00613	0	0	0.054536	0	0
Novel_007246	XLOC_07500.	transcript	0.067738	0.116934	0.337256	0.196394	0.370515	0.023898	0.735143	0.293455	0.15264

Novel_007247	XLOC_07500.	transcript	0	0	0.032428	0.082735	0	0.124687	0	0	0
Novel_007248	XLOC_07504.	transcript	0.170229	0.195284	0	0.033314	0	0	0	0.043534	0
Novel_007249	XLOC_07505.	transcript	0.157246	0.019666	0.039567	0	0.236528	0	0.116011	0.07281	0
Novel_007250	XLOC_07505.	transcript	0.117107	0.014607	0.071576	0.002147	0.088756	0	0.520563	0.797096	0.307978
Novel_007251	XLOC_07505.	transcript	0.023225	0.027134	0	0.01402	0.013399	0.241296	0.030502	0.006861	0.01708
Novel_007252	XLOC_07505.	transcript	0	0.10237	0.053592	0	0	0.194803	0	0.443367	0.80335
Novel_007253	XLOC_07505.	transcript	0	0	0	0	0	0	0	0	0
Novel_007254	XLOC_07505.	transcript	0.703665	0.771285	0.541321	1.068209	0.703673	0.226067	0.365386	0.168699	0.020304
Novel_007255	XLOC_07505.	transcript	0	0	0	0.049794	0	0	0	0	0
Novel_007256	XLOC_07505.	transcript	0.074232	0	0	0	0	0	0	0	0
Novel_007257	XLOC_07505.	transcript	0.533207	0	0.656041	1.820315	1.320457	1.33861	1.402405	0.368233	0.506223
Novel_007258	XLOC_07505.	transcript	0	0	0	0	0	0	0	0.070129	0.052876
Novel_007259	XLOC_07505.	transcript	0.093765	0.199025	0.170794	0.238641	0.281747	0.075504	0.428311	0.135097	0.154408
Novel_007260	XLOC_07509.	transcript	3.606668	5.698968	3.042838	3.164155	3.668776	3.280075	5.236172	4.168829	3.922348
Novel_007261	XLOC_07509.	transcript	0.117556	0.17599	0.1714	0.068243	0.094277	0.111309	0.142384	0.044079	0.021881
Novel_007262	XLOC_07514.	transcript	0.578395	0.868805	0.306057	0.361473	0.376765	0.381521	0.723737	0.699808	0.371607
Novel_007263	XLOC_07514.	transcript	0	0	0.140524	0.226964	0	0.009876	0.157866	0.103665	0.028472
Novel_007264	XLOC_07514.	transcript	0.012614	0	0	0	0	0	0	0	0
Novel_007265	XLOC_07514.	transcript	0.164983	0.044714	0.015771	0.048739	0.047323	0.058358	0.202662	0.112448	0.044831
Novel_007266	XLOC_07516.	transcript	0.006516	1.213948	0	0	0	0	0	0	0
Novel_007267	XLOC_07519.	transcript	0	0.314427	0	0	0	0	0	0	0
Novel_007268	XLOC_07519.	transcript	0.343668	0.01637	0.32758	0.027332	0.244435	0.070294	0.458753	0.332055	0.178943
Novel_007269	XLOC_07519.	transcript	0	0	0	0.156803	0	0.111647	0	0	0
Novel_007270	XLOC_07520.	transcript	0.134254	0	0	0.077593	0.305226	0.38092	0.290641	0.167731	0.182329
Novel_007271	XLOC_07520.	transcript	0.192698	0	0	0.089361	0	0	0.139085	0	0
Novel_007272	XLOC_07520.	transcript	0.017424	0	0.369967	0.244862	0.08537	0.195113	0.071114	0.30145	0.194736
Novel_007273	XLOC_07521.	transcript	0	0	0.003669	0	0.009592	0	0	0	0.013699
Novel_007274	XLOC_07521.	transcript	0.327994	0.378952	0.304066	0.186634	0.49315	0.09723	0.345927	0.021207	0.339642
Novel_007275	XLOC_07521.	transcript	0.014584	0.278811	0.124118	0.229921	0.132758	0.011975	0.50804	0.015401	0.54349
Novel_007276	XLOC_07521.	transcript	0.050563	0	0.069902	0.105226	0	0	0	0.010261	0.03453
Novel_007277	XLOC_07521.	transcript	0.119849	0.172829	0.276605	0.124215	0.205038	0.122078	0.361423	0.379748	0.190953
Novel_007278	XLOC_07523.	transcript	0.055752	0.323447	0.572119	1.448864	0	0.437276	0	0.540902	0
Novel_007279	XLOC_07525.	transcript	0.45698	0.091278	0.075642	0.264705	0.115194	0.207627	0.831676	0.35631	0.214616
Novel_007280	XLOC_07525.	transcript	0.025641	0.063754	0.162108	0.16089	0.052451	0	0.038354	0	0
Novel_007281	XLOC_07525.	transcript	0	0	0	0.599192	0	0	0	0.473101	0
Novel_007282	XLOC_07525.	transcript	0.687857	1.074725	1.055488	0.547208	0.510628	0.722335	0.941178	0.641567	0.586498
Novel_007283	XLOC_07525.	transcript	0	0	0.004985	0.013227	0.007575	0.056675	0.069858	0	0.006752
Novel_007284	XLOC_07525.	transcript	0.231624	0.143489	0.142319	0.200774	0.212271	0.15263	0.322082	0.261764	0.163097

Novel_007285	XLOC_07525.	transcript	0	0.031944	0.014326	0.010599	0	0	0	0.023922	0.015651
Novel_007286	XLOC_07525.	transcript	0	0	0	0.012647	0	0.067548	0	0	0.009973
Novel_007287	XLOC_07525.	transcript	0.077837	0.023915	0.147224	0.067697	0.080579	0.111429	0.087898	0.087147	0.043213
Novel_007288	XLOC_07527.	transcript	0.125901	0.067312	0.165527	0.188182	0.079959	0	0.199262	0.177977	0.095825
Novel_007289	XLOC_07542.	transcript	0.385539	0	0.019596	0.019173	0	0	0	0	0.020532
Novel_007290	XLOC_07542.	transcript	0.010625	0.10326	0.336056	0.199521	0.28985	0.400461	0.321099	0.403227	0.198651
Novel_007291	XLOC_07545.	transcript	0.074387	0.280685	0.114535	0.024845	0.253465	0	0.22586	0.237919	0.073577
Novel_007292	XLOC_07545.	transcript	0.220413	0.174222	0.267295	0.225409	0.134002	0.395849	0.062255	0.151033	0.131934
Novel_007293	XLOC_07546.	transcript	0.206222	1.032723	0.09635	0.070045	0.33247	0.31971	0.622638	0.185591	1.180648
Novel_007294	XLOC_07548.	transcript	0.205876	1.000517	0.534046	1.202176	0.822522	0.871015	0.585355	0.829079	0.671892
Novel_007295	XLOC_07548.	transcript	0	0	0	0	0	0	0	0	0
Novel_007296	XLOC_07550.	transcript	0.505959	0.553171	0.909923	0.482949	0.538578	0.270831	0.898444	0.514068	0.462936
Novel_007297	XLOC_07555.	transcript	0.012736	1.034141	0.523666	0.051777	0.459383	0.976005	0.151754	0.362039	0.201019
Novel_007298	XLOC_07556.	transcript	0	0	0.2148	0.233984	0	0.175468	0.004172	0.00368	0.336331
Novel_007299	XLOC_07556.	transcript	0.148915	0.101496	0.386985	0.015219	0.244289	0.224162	0.229189	0.035162	0.031936
Novel_007300	XLOC_07556.	transcript	0.097302	0	0	0	0.182611	0	0.508231	0.25432	0
Novel_007301	XLOC_07556.	transcript	0	0.403221	0.213172	0.288146	0	0	0.311873	0.227973	0.029149
Novel_007302	XLOC_07556.	transcript	0.215295	0.531127	0.191141	0.216375	0.469239	0.359287	0.32424	0.452367	0.243815
Novel_007303	XLOC_07560.	transcript	0.25248	0.166356	0.361203	0.497013	0.348324	0.568527	0.375732	0.248709	0.287519
Novel_007304	XLOC_07565.	transcript	0.115015	0.484419	0.029218	0.438945	0	0	0	0.225864	0.215706
Novel_007305	XLOC_07565.	transcript	0	0	0.019625	0	0	0	0.264936	0	0
Novel_007306	XLOC_07565.	transcript	0	0	0	0	0	0	0	0	0
Novel_007307	XLOC_07565.	transcript	0	0	0	0	0	0	0	0	0
Novel_007308	XLOC_07565.	transcript	0	0	0.026668	0.0017	0.069856	0.013798	0	0	0
Novel_007309	XLOC_07565.	transcript	0	0	0	0	0	0.123221	0	0	0
Novel_007310	XLOC_07575.	transcript	0	0	0	0	0	40.42982	204.607	0	89.73505
Novel_007311	XLOC_07575.	transcript	0	0	0	0	0	0	0	0	0
Novel_007312	XLOC_07575.	transcript	0	0	0	0	0	12.13833	0	0	40.97321
Novel_007313	XLOC_07577.	transcript	0.339318	0	0.032289	0.053348	0.285896	0.062354	0.212518	0.232352	0
Novel_007314	XLOC_07579.	transcript	0.116006	0.045382	0.041081	0.150608	0.078999	0.184942	0.029705	0.046078	0.068501
Novel_007315	XLOC_07579.	transcript	0	0	0	0	0	0.01552	0.107792	0.096946	0
Novel_007316	XLOC_07585.	transcript	0.207459	0.006848	0.047242	0.02856	0.034867	0.183362	0.129652	0.032782	0.085759
Novel_007317	XLOC_07585.	transcript	0.038821	0.118658	0.30903	0.182294	0.229282	0.090329	0.365595	0.500347	0.123035
Novel_007318	XLOC_07586.	transcript	0	0.200181	0	0	0	0	0.014487	0	0.010462
Novel_007319	XLOC_07586.	transcript	0	0	0.270349	0	0	0.100946	0	0	0
Novel_007320	XLOC_07586.	transcript	1.698779	1.823344	1.437803	1.045189	1.902359	1.252597	1.660969	1.559477	1.359639
Novel_007321	XLOC_07590.	transcript	0	0.554234	0.090369	0.02388	0	0.13751	0.021238	0	0.063933
Novel_007322	XLOC_07590.	transcript	0.122413	0.201521	0.122467	0.144945	0.236034	0.238398	0.216459	0.181769	0.251857

Novel_007323	XLOC_07590.	transcript	0.218438	0.192992	0.331273	0.239758	0.241411	0.185773	0.293896	0.455304	0.27473
Novel_007324	XLOC_07591.	transcript	0.101606	0.354899	0.132949	0.089753	0.200588	0.149044	0.209193	0.157217	0.221009
Novel_007325	XLOC_07591.	transcript	0.116791	0.059481	0.296603	0.158331	0.2778	0.147082	0.264608	0.256421	0.179092
Novel_007326	XLOC_07592.	transcript	0.082646	0.0959	0.191553	0.25824	0.061772	0.255041	0.255432	0.176805	0.294891
Novel_007327	XLOC_07593.	transcript	0	0	0	0	0.117817	0.090686	0	0.091087	0.088766
Novel_007328	XLOC_07593.	transcript	0	0	0	0	0	0	0	0	0
Novel_007329	XLOC_07593.	transcript	0.260736	0.172582	0.303248	0.122774	0.151748	0.15056	0.46078	0.170647	0.185841
Novel_007330	XLOC_07594.	transcript	0.106781	8.525005	0	0	0.052492	0	0	0	0.243292
Novel_007331	XLOC_07597.	transcript	0	0	0	0.013231	0.194413	0.186306	0.193711	0	0.084656
Novel_007332	XLOC_07597.	transcript	0.020595	0.291023	0.418173	0.208738	0.233552	0.711467	0.166111	0.033557	0.336656
Novel_007333	XLOC_07597.	transcript	0.471264	0.256005	0.438661	0.387731	0.27957	0.644194	0.401114	0.544978	0.190939
Novel_007334	XLOC_07598.	transcript	1.43318	2.015962	1.431891	1.625077	0.946789	1.074198	1.543284	1.304981	0.827651
Novel_007335	XLOC_07598.	transcript	0	0.034789	0.013681	0.031976	0.713577	0	0.009678	0.005035	0.538292
Novel_007336	XLOC_07598.	transcript	0	0	0	0	0	0.160197	0	0	0
Novel_007337	XLOC_07598.	transcript	0.015866	0.035818	0.028436	0.02231	0.017921	0.012383	0.023732	0.361658	0.012535
Novel_007338	XLOC_07598.	transcript	0	0	0	0	0	0	0	0	0
Novel_007339	XLOC_07599.	transcript	0.299108	0.032284	0	0.135836	0.551098	0.102577	0.583858	0.1999	0.150605
Novel_007340	XLOC_07599.	transcript	0	0.499706	1.03162	0.889263	0	0.986692	0	0.790078	0.882179
Novel_007341	XLOC_07600.	transcript	0.194623	0.256078	0.130691	0.204326	0.108543	0.174304	0.08495	0.189005	0.175814
Novel_007342	XLOC_07600.	transcript	0.146979	0	0	0	0	0	0	0.061591	0
Novel_007343	XLOC_07600.	transcript	0.179314	1.348205	0.663257	1.642986	1.125723	1.366897	1.423705	2.195542	1.545695
Novel_007344	XLOC_07600.	transcript	0.241205	0.527623	0	1.053938	0	0	0	0.87713	0
Novel_007345	XLOC_07600.	transcript	0.276103	0	0	0	0	0	0	0	0
Novel_007346	XLOC_07600.	transcript	1.03816	0.739203	1.17657	0	0.897055	1.567332	0.818701	0	0.7924
Novel_007347	XLOC_07600.	transcript	0.140908	1.609797	0.801398	2.114268	1.36894	2.476208	1.586421	1.103104	1.272134
Novel_007348	XLOC_07605.	transcript	0.126566	0.093994	0.106247	0.161928	0.165844	0.183675	0.111868	0.139847	0.020678
Novel_007349	XLOC_07606.	transcript	0.185392	0	0.1345	0	0.139384	0.120027	0.222712	0.158421	0.130792
Novel_007350	XLOC_07606.	transcript	0	0.142395	0	0.115195	0.012412	0	0	0.04251	0
Novel_007351	XLOC_07606.	transcript	0	0	0	0	0	0	0	0	0
Novel_007352	XLOC_07606.	transcript	0.076765	0.25443	0.132627	0.097243	0.120292	0.108941	0.110892	0.136179	0.301097
Novel_007353	XLOC_07606.	transcript	0.135836	0.186501	0.289005	0.159327	0.12879	0.390697	0.528991	0.254416	0.12552
Novel_007354	XLOC_07606.	transcript	0.37855	0.017145	0.019704	0.004703	0.140151	0.015465	0.024661	0.007974	0.006494
Novel_007355	XLOC_07606.	transcript	0	0	0	0	0	0	0	0	0
Novel_007356	XLOC_07606.	transcript	0.262079	0	0	0.26279	0.199586	0	0	0	0.480866
Novel_007357	XLOC_07606.	transcript	0.166686	0.004473	0.301016	0.000395	0.003932	0.062002	0.295552	0.086335	0.09926
Novel_007358	XLOC_07619.	transcript	0.076225	0	0.025671	0.11896	0.057355	0.196599	0.301797	0.39275	0.19704
Novel_007359	XLOC_07622.	transcript	0.047212	0.019564	0.14974	0.452469	0.048288	0.027342	0.119543	0.193362	0.027871
Novel_007360	XLOC_07623.	transcript	0.058299	0.080529	0.205174	0.048861	0.119254	0.19781	0.151684	0.260614	0.06817

Novel_007361	XLOC_07624.	transcript	0.107377	0.170171	0.337505	0.013655	0.397095	0.010258	0.055644	0.056174	0.031408
Novel_007362	XLOC_07624.	transcript	0.004469	0	0	0.003772	0	0	0.039134	0.002436	0.003376
Novel_007363	XLOC_07624.	transcript	0.216892	0.063756	0.546214	0.256836	0.353602	0.891301	0.302972	0.34674	0.373348
Novel_007364	XLOC_07624.	transcript	0.218375	0.092602	0.531108	0.462967	0.521025	0.076321	1.153712	0.494449	0.590617
Novel_007365	XLOC_07629.	transcript	0.20165	0.556897	0.342678	0.252415	0.500437	0.34513	0.404436	0.403145	0.231268
Novel_007366	XLOC_07629.	transcript	0	0.070968	0.063802	0.067159	0	0.175315	0.019977	0.181035	0.063205
Novel_007367	XLOC_07630.	transcript	0	0.085389	0	0	0	0	0.046671	0	0
Novel_007368	XLOC_07630.	transcript	0.418255	0.022589	0.353142	0.25732	0.269492	0.129337	0.022066	0.360046	0.181235
Novel_007369	XLOC_07630.	transcript	0	0.017388	0.001191	0	0.004748	0.148828	0.07786	0	0.020051
Novel_007370	XLOC_07630.	transcript	0.187809	0.220959	0.239483	0.1716	0.213614	0.123194	0.432214	0.250764	0.177761
Novel_007371	XLOC_07630.	transcript	0	0	0	0	0.001294	0.154786	0.324021	0.000451	0
Novel_007372	XLOC_07630.	transcript	0	0	0.006135	0	0	0	0.010619	0.013143	0
Novel_007373	XLOC_07630.	transcript	0.873937	0.088878	0.952188	1.026459	1.005702	1.059197	1.10564	1.020135	1.041258
Novel_007374	XLOC_07631.	transcript	0.12683	0.091831	0.187787	0.103134	0.018315	0.01291	0.075502	0.055895	0.157328
Novel_007375	XLOC_07631.	transcript	0	0	0	0	0	0.266173	0	0.078102	0
Novel_007376	XLOC_07631.	transcript	0.0021	0.48431	0.332352	0	0.350709	0.44494	0	0.286647	0.205936
Novel_007377	XLOC_07631.	transcript	0.875253	0.244508	0	0.469074	0.013656	0	0.647221	0.440311	0.09008
Novel_007378	XLOC_07631.	transcript	0	0	0	0	0	0	0	0.091342	0
Novel_007379	XLOC_07632.	transcript	0.539271	0	0.973648	1.024156	0.963737	0.597019	0.649199	0.714293	0.547838
Novel_007380	XLOC_07632.	transcript	0.537761	0.367242	0.672342	0.82976	0.631365	0.867234	0.330821	0.640762	0.436936
Novel_007381	XLOC_07632.	transcript	0	0	0	0	0	0	0	0	0
Novel_007382	XLOC_07632.	transcript	1.085951	1.321426	1.080513	0.813358	0.84799	0.808789	1.355825	1.205244	0.791046
Novel_007383	XLOC_07636.	transcript	0.35281	0	0.745435	1.091346	0.433827	0.740909	1.072603	1.214503	0
Novel_007384	XLOC_07637.	transcript	3.660382	4.371834	3.561934	2.210826	2.758987	3.701581	3.537795	3.065794	2.362501
Novel_007385	XLOC_07645.	transcript	0	0	0.047964	0	0.544658	0	0	0	0.326773
Novel_007386	XLOC_07645.	transcript	0	0	0.282594	0	0.06873	0	0.030696	0	0
Novel_007387	XLOC_07655.	transcript	0.205893	0.004145	0.2708	0.565197	0.337199	0.39006	0.186858	0.408645	0.110003
Novel_007388	XLOC_07655.	transcript	0.70332	0.218041	0.484029	0.505304	0.585489	0.730126	0.709877	0.784806	0.561938
Novel_007389	XLOC_07655.	transcript	0.104755	0.068386	0.454367	0.396777	0.26227	0.493869	0.280064	0.277705	0.272355
Novel_007390	XLOC_07658.	transcript	0.645665	0.058011	0.789372	0.564682	0.678107	0.8978	0.829002	0.629014	0.63189
Novel_007391	XLOC_07664.	transcript	0.278343	0.008011	0.217807	0.427894	0.334681	0.399494	0.121362	0.302471	0.100796
Novel_007392	XLOC_07664.	transcript	0	0.122419	0.01952	0	0.151073	0.13366	0	0.188167	0
Novel_007393	XLOC_07665.	transcript	0.137224	0.535695	0.865507	0.759319	0.75311	0.67449	0.382059	0.429263	1.05283
Novel_007394	XLOC_07665.	transcript	0.26825	0.010217	0.20889	0.178486	0.238851	0.179298	0.057773	0.078845	0.043339
Novel_007395	XLOC_07665.	transcript	0	0	0.119291	0.137355	0.082398	0.09696	0.176658	0.238493	0.257328
Novel_007396	XLOC_07670.	transcript	0.013473	0.003913	0	0.013209	0	0.003447	0.005188	0	0.009651
Novel_007397	XLOC_07670.	transcript	0.719967	0.982197	0.449492	0.517795	0.497059	0.516217	1.301905	0.580954	0.958537
Novel_007398	XLOC_07680.	transcript	0.749487	1.082498	0.775774	0.705382	1.825695	1.114264	0.439419	0.646598	0.656986

Novel_007399	XLOC_07687.	transcript	0.222709	0.126301	0.247269	0.285212	0.437854	0.327289	0.400499	0.285394	0.203719
Novel_007400	XLOC_07691.	transcript	0.078117	0.042265	0.035367	0.000774	0.023625	0.462514	0.751932	0.046021	0
Novel_007401	XLOC_07691.	transcript	0.8224	0.041077	0.127364	0.036484	0.332534	0.829718	0.052045	0.068365	0.074049
Novel_007402	XLOC_07691.	transcript	0.26996	0.008268	0.241307	0.223966	0.264068	0.177471	0.214473	0.264156	0.298346
Novel_007403	XLOC_07699.	transcript	0.884687	0.184672	0.566672	0.577797	0.898037	0.521907	1.315713	0.706782	0.68087
Novel_007404	XLOC_07699.	transcript	0	0	0	0	0	0	0.001334	0	0
Novel_007405	XLOC_07699.	transcript	0	0	0	0	0	0.019347	0	0.342054	0
Novel_007406	XLOC_07701.	transcript	0.31697	0.069622	0.122559	0.01962	0.145752	0.153024	0.141572	0.158217	0.065562
Novel_007407	XLOC_07703.	transcript	0.034718	0.475313	0.037393	0.014549	0.109072	0.316165	0.136024	0.087098	0.064035
Novel_007408	XLOC_07703.	transcript	0.352239	0.341227	0.287521	0	0.161569	0.487611	0.32942	0.355125	0.177775
Novel_007409	XLOC_07703.	transcript	0	0.28126	0	0	0	0	0.130121	0	0
Novel_007410	XLOC_07703.	transcript	0.216984	0.282462	0.141678	0.104826	0.259747	0.415364	0.1674	0.104354	0.051119
Novel_007411	XLOC_07707.	transcript	0.058679	0.683807	0.082807	0.090263	0.123223	0.176168	0.152079	0.113989	0.181614
Novel_007412	XLOC_07708.	transcript	0.180972	0.491586	0.202284	0.143458	0.134714	0.183428	0.184317	0.324777	0.200617
Novel_007413	XLOC_07708.	transcript	0.06332	0.041844	0.033845	0.310612	0.057509	0.197518	0.020207	0.071349	0
Novel_007414	XLOC_07708.	transcript	0.145814	0.302273	0.145476	0.28871	0.140237	0.199677	0.11302	0.286188	0.242854
Novel_007415	XLOC_07709.	transcript	0.696299	0.928567	0.648565	0.484145	0.715269	0.624511	0.867511	0.754965	0.601838
Novel_007416	XLOC_07710.	transcript	0	0.288348	0	0.006169	0.007529	0	0.010937	0	0
Novel_007417	XLOC_07710.	transcript	0.155917	0.088431	0.098684	0.061208	0.176722	0.06545	0.190179	0.086613	0.169197
Novel_007418	XLOC_07710.	transcript	0.093048	0.164635	0.057577	0.027025	0.112554	0.057137	0.258072	0.057064	0.274289
Novel_007419	XLOC_07712.	transcript	0.010281	0	0.231532	0.20092	0	0.24412	0.222271	0.142024	0
Novel_007420	XLOC_07712.	transcript	0.001764	0	0	0	0	0	0	0.002225	0
Novel_007421	XLOC_07712.	transcript	1.00078	0.774651	0.458009	0.913711	0.578076	0.742615	0.753737	0.981438	1.008789
Novel_007422	XLOC_07712.	transcript	0.007401	0.436289	0.183894	0.002556	0.040594	0.231764	0.330914	0.280007	0.008962
Novel_007423	XLOC_07712.	transcript	0.003204	0.029688	0.002265	0.043489	0.408274	0	0.003248	0.17212	0.672687
Novel_007424	XLOC_07713.	transcript	0.368954	0.047491	0	0.003851	0	0.161481	0.157754	0.181135	0.618706
Novel_007425	XLOC_07713.	transcript	0	0	0	0	0	0	0	0.04757	0
Novel_007426	XLOC_07713.	transcript	0.25578	0	0	0	0.157234	0	0	0	0
Novel_007427	XLOC_07713.	transcript	0.478459	0.223694	0.210582	0.179548	0.587651	0.511788	0.361378	0.436256	0.011702
Novel_007428	XLOC_07713.	transcript	0.511681	1.241442	1.637654	1.374491	1.09081	0.213434	1.232173	0.396932	0.263184
Novel_007429	XLOC_07716.	transcript	0.096375	0.234562	0.220773	0.129058	0.218208	0.26367	0.367203	0.193409	0.259833
Novel_007430	XLOC_07716.	transcript	0.02287	0.815394	0	0	0	0	0.017446	0	0
Novel_007431	XLOC_07716.	transcript	0	0.030357	0	0	0	0	0	0	0
Novel_007432	XLOC_07716.	transcript	0	0.230167	0.013118	0	0.024756	0.108993	0.050818	0.034055	0.023204
Novel_007433	XLOC_07723.	transcript	0	0.141441	0	0	0	0	0	0	0
Novel_007434	XLOC_07723.	transcript	0	0.039888	0.008417	0.00544	0	0	0.284826	0.00551	0.003359
Novel_007435	XLOC_07723.	transcript	0	1.139957	0	0	0	0	0	0	0
Novel_007436	XLOC_07723.	transcript	0	0.97691	0	0	0	0	0	0	0

Novel_007437	XLOC_07723.	transcript	0.037997	0.040248	0.100734	0.091223	0.187139	0.011126	0	0.120067	0.048894
Novel_007438	XLOC_07729.	transcript	0	0	0	0	0.082221	0	0	0.003187	0
Novel_007439	XLOC_07729.	transcript	0.093598	0.113459	0.368694	0.220553	0.205639	0.122376	0.220287	0.326277	0.177969
Novel_007440	XLOC_07730.	transcript	1.086346	0	0.749157	0.006809	0	1.769405	0	0.138034	0
Novel_007441	XLOC_07730.	transcript	0	0	0	1.06585	0	1.00E-04	0.001797	0	0.040191
Novel_007442	XLOC_07730.	transcript	0	0	0	0	0	0	0	0	0
Novel_007443	XLOC_07730.	transcript	0	0	0	0	0	0.255813	0	0	0
Novel_007444	XLOC_07730.	transcript	0.007027	0	0.002865	2.119576	0	0.078555	0.071385	0	0.286689
Novel_007445	XLOC_07730.	transcript	0	4.435456	0	7.30E-05	1.470701	0.027166	0.153671	3.888165	1.540975
Novel_007446	XLOC_07730.	transcript	0	0	0	0	0	0	0	0	0.812778
Novel_007447	XLOC_07735.	transcript	0	0.053345	0.777018	0.038645	0.02851	0.070457	0.074838	0.009812	0.003825
Novel_007448	XLOC_07735.	transcript	0	0	0	0	0	0	0	0.024478	0
Novel_007449	XLOC_07735.	transcript	0.451731	0	0.266777	1.743526	0.508755	0.833529	0.700349	1.184854	0.463161
Novel_007450	XLOC_07735.	transcript	0.461227	0	0	0	0.29257	0	0.56461	0	0
Novel_007451	XLOC_07735.	transcript	0.502282	0	0.253807	0.505111	0.405098	1.504069	0.750496	1.388886	1.133837
Novel_007452	XLOC_07735.	transcript	0	0	0	0.189084	0	0	0	0	0
Novel_007453	XLOC_07735.	transcript	0	0	0	0	0	0.178089	0.350848	0	0
Novel_007454	XLOC_07735.	transcript	0.10279	0.272914	0.071554	0.169242	0.2152	0	0	0.154907	0.087969
Novel_007455	XLOC_07735.	transcript	0	0	0.100494	0	0	0	0	0	0
Novel_007456	XLOC_07735.	transcript	0.012	0.479301	0.353791	0.008219	0	0.060644	0.30788	0.6399	0
Novel_007457	XLOC_07735.	transcript	0.173161	0.434702	0.196744	0.290543	0.420326	0.316761	0.345952	0.393592	0.332178
Novel_007458	XLOC_07735.	transcript	0.135977	0	0.234909	0	0	0	0	0	0
Novel_007459	XLOC_07736.	transcript	0	0	0	0.346969	0	0	0	0	0.420171
Novel_007460	XLOC_07736.	transcript	0.007068	0.340468	0.015721	0.120673	0.083032	0.234247	0.328221	0.014958	0.025525
Novel_007461	XLOC_07736.	transcript	0	0	0	0.006836	0.041536	0.188516	0.238595	0	0
Novel_007462	XLOC_07736.	transcript	0	0	0	0	0	0	0	0	0
Novel_007463	XLOC_07737.	transcript	1.159439	1.965139	1.190037	1.497321	1.34587	1.335492	1.393758	1.307685	1.491696
Novel_007464	XLOC_07737.	transcript	0	0	0	0	0	0	0	0	0
Novel_007465	XLOC_07737.	transcript	0	0	0	0	0	0	0	0	0
Novel_007466	XLOC_07738.	transcript	0.406362	0.572406	1.444415	1.703874	1.418376	1.600666	2.489321	1.242107	1.093529
Novel_007467	XLOC_07738.	transcript	0	0	0.413283	0	0	0	0	0	0
Novel_007468	XLOC_07738.	transcript	1.857759	0	0	0	0	0.005927	0.010753	0	0
Novel_007469	XLOC_07740.	transcript	43.80139	53.47039	56.2463	56.1089	48.88494	58.96623	58.4474	52.73685	64.88665
Novel_007470	XLOC_07741.	transcript	0.78454	0.579053	0.922088	0.929307	0.942542	0.928073	1.251642	1.173258	0.745912
Novel_007471	XLOC_07743.	transcript	0.383402	0	0.452503	0.550871	0.636538	0.262646	0.593942	0.68986	0.149638
Novel_007472	XLOC_07743.	transcript	0.076045	0	0.146604	0.139225	0.184782	0	0.190179	0.168977	0
Novel_007473	XLOC_07743.	transcript	0	0.007109	0.026219	0.120297	0	0	0	0.237262	0
Novel_007474	XLOC_07743.	transcript	0	0	0	0	0	0	0.00023	0	0

Novel_007475	XLOC_07743.	transcript	0	0	0.092487	0.275039	0	0.119579	0.292384	0	0.153139
Novel_007476	XLOC_07743.	transcript	0.054614	0	0.038285	0.049424	0.285311	9.10E-05	0.055538	0.000109	0
Novel_007477	XLOC_07743.	transcript	0.149397	0	0.003934	0.006648	0.030501	0.001376	0.002018	0.093268	0.002866
Novel_007478	XLOC_07745.	transcript	0	0.645274	0.057592	0.085504	0.018842	0.088273	0.102406	0.120886	0.177805
Novel_007479	XLOC_07751.	transcript	0.219022	0	0.091583	0	0	0	0.041145	0.097873	0.051332
Novel_007480	XLOC_07755.	transcript	0.487863	0.897004	0.638667	0.913433	0.843427	0.771227	0.838843	0.698035	0.749642
Novel_007481	XLOC_07756.	transcript	1.908038	0.48954	2.523531	2.251723	2.452488	2.291986	3.050038	2.530212	2.626713
Novel_007482	XLOC_07757.	transcript	0.165551	0.429734	0.106265	0.200244	0.225304	0.125581	0.281259	0.226128	0.020891
Novel_007483	XLOC_07757.	transcript	0.099216	0.786351	0.024342	0.258159	0.087633	0.022801	0.152441	0.17802	0.047126
Novel_007484	XLOC_07757.	transcript	0.08337	0.159326	0.129942	0.36674	0.300614	0.164407	0.12311	0.146621	0.120347
Novel_007485	XLOC_07764.	transcript	0.331082	0.121004	0.153466	0.103633	0.112928	0.09615	0.47562	0.192684	0.230307
Novel_007486	XLOC_07764.	transcript	0	0	0.72326	0.092883	0.12077	0.073749	0.142109	0.023087	0.902906
Novel_007487	XLOC_07764.	transcript	0.033109	0.036569	0.057583	0.022847	0.24091	0	0.006191	0	0.052197
Novel_007488	XLOC_07764.	transcript	0.205311	0	0.005268	0	0	0	0.23435	0.228592	0
Novel_007489	XLOC_07764.	transcript	0	0.272428	0.002285	0	0	0	0.016623	0	0
Novel_007490	XLOC_07764.	transcript	0.660925	0.044353	0.756973	0	0	0.012034	0.215364	0.394726	0.281951
Novel_007491	XLOC_07764.	transcript	0.01587	0.006973	0.005416	0.048756	0	0.013444	0	0.015501	0.299791
Novel_007492	XLOC_07764.	transcript	0.073583	0	0.055594	0.331327	0.025036	0.443766	0.357551	0.117075	0.038639
Novel_007493	XLOC_07764.	transcript	0.002947	0.011379	0.006166	0.145581	0.009444	0.202725	0.004566	0	0
Novel_007494	XLOC_07764.	transcript	0	0.607156	0	0	0	0	0	0	0
Novel_007495	XLOC_07764.	transcript	0	0	0	0.002237	0.504172	0	0.249413	0	0
Novel_007496	XLOC_07767.	transcript	0.096525	0	0.07742	0.493305	0.119001	0.184711	0.184283	0.156874	0
Novel_007497	XLOC_07767.	transcript	0.07829	0	0	3.492056	0.553867	0	0.337427	0.133548	0
Novel_007498	XLOC_07767.	transcript	0.194935	0.024148	0.059051	0.614327	0.203561	0.113787	0.399987	0.14383	0.026137
Novel_007499	XLOC_07767.	transcript	0	0	0	0	0	0	0	0	0.029732
Novel_007500	XLOC_07768.	transcript	0.331369	0.016691	0.010214	0	0.132737	0.005092	0.070386	0.133674	0.104177
Novel_007501	XLOC_07768.	transcript	0.099609	0.143322	0.189828	0.213242	0.044834	0.06662	0.015306	0.089362	0.023776
Novel_007502	XLOC_07768.	transcript	0.046314	0.078994	0	0.138913	0.051757	0	0	0	0
Novel_007503	XLOC_07780.	transcript	0.390141	0.819097	0.786434	0.730558	0.678603	0.662254	0.611965	1.111657	0.515726
Novel_007504	XLOC_07783.	transcript	0.011018	0.00751	0	1.013318	0.445447	0	0	0.792078	0
Novel_007505	XLOC_07783.	transcript	0.350131	0.434066	0.342326	0.04453	0.154408	0.426488	0.587108	0	0.429374
Novel_007506	XLOC_07783.	transcript	0.190121	0.26851	0.200481	0.252868	0.296385	0.194106	0.430678	0.350455	0.120659
Novel_007507	XLOC_07784.	transcript	0	0	0.271782	0	0	0	0	0	0
Novel_007508	XLOC_07784.	transcript	0.385256	0.240813	0.424137	0.439926	0.684908	0.559304	0.747791	0.511848	0.62998
Novel_007509	XLOC_07784.	transcript	0	0	0	0	0	0	0	0	0
Novel_007510	XLOC_07785.	transcript	0.069607	0.500616	0.057492	0	0	0	0	0	0.032224
Novel_007511	XLOC_07785.	transcript	0.004	0	0.528423	0.174053	0.200696	0.12843	0.129773	0.085896	0
Novel_007512	XLOC_07785.	transcript	0	0	0	0	0	1.380283	0	0	0

Novel_007513	XLOC_07785.	transcript	0	0	0	0	0	0	0.371351	0	0
Novel_007514	XLOC_07785.	transcript	1.508467	1.515414	0.173268	0.352016	0.864894	0.252757	1.81943	1.130003	0.192088
Novel_007515	XLOC_07785.	transcript	0.060789	0	0	0.472917	0	0	0.139451	0	0
Novel_007516	XLOC_07785.	transcript	0.002764	0.015739	0.634217	0.43456	0.206827	0.219894	0.615987	0.130177	0.033758
Novel_007517	XLOC_07785.	transcript	0.117697	0.006158	0.960293	1.19555	0.587117	0.246484	0.376476	0.127423	0.047466
Novel_007518	XLOC_07787.	transcript	3.933322	0	0.008869	0.007235	0.014513	0.011716	0.011046	0.003754	0.006061
Novel_007519	XLOC_07787.	transcript	5.528497	5.692194	10.84535	8.431725	13.10217	9.518445	17.27995	15.10735	15.8855
Novel_007520	XLOC_07788.	transcript	0.218109	0.200935	0.204524	0.141064	0.199482	0.190242	0.23391	0.205022	0.116014
Novel_007521	XLOC_07798.	transcript	0.100897	0	0	0.123482	0	0.108256	0.130502	0.086569	0.293144
Novel_007522	XLOC_07798.	transcript	0.107423	0.192685	0.109055	0.15711	0.13832	0.08947	0.200877	0.138304	0.146924
Novel_007523	XLOC_07799.	transcript	0.58586	0.339911	0.323872	0.240846	0.401378	0.338493	0.368504	0.351067	0.199843
Novel_007524	XLOC_07800.	transcript	0	0.164681	0.0952	0.045155	0.084031	0.010197	0.037468	0.006606	0.008613
Novel_007525	XLOC_07800.	transcript	0.347007	0.328825	0.14224	0.308511	0.308466	0.324173	0.356066	0.298672	0.464447
Novel_007526	XLOC_07800.	transcript	0	0	0.29579	0	0.003618	0	0	0.004934	0.042033
Novel_007527	XLOC_07800.	transcript	0	0	0	0	0	0	0	0.004423	0
Novel_007528	XLOC_07800.	transcript	0.661105	1.207523	0.515954	1.054284	0.532525	0.488406	0.89365	1.458171	0.317192
Novel_007529	XLOC_07800.	transcript	0	0	0.204329	0	0.036988	0	0	0	0
Novel_007530	XLOC_07803.	transcript	0	0	0	0	0	0	0	0	0
Novel_007531	XLOC_07803.	transcript	0	0	0	0	0	0	0	0	0
Novel_007532	XLOC_07803.	transcript	0	1.116636	0.104706	0	0.719137	0.70721	0	0	0
Novel_007533	XLOC_07807.	transcript	0.660787	1.593354	0.441188	0.306031	0.442159	0.474224	0.693397	0.425269	0.343821
Novel_007534	XLOC_07809.	transcript	0.028161	0	0	0	0	0.011869	0	0.023364	0.205758
Novel_007535	XLOC_07809.	transcript	0	0	0	0	0	0.38014	0	0	0
Novel_007536	XLOC_07812.	transcript	0.333559	0	0.151229	0.167858	0.062261	0.263267	0.020442	0.179553	0.081313
Novel_007537	XLOC_07815.	transcript	0.381904	0.323186	0.60283	0.618813	0.490218	0.58961	0.52696	0.360788	0.546782
Novel_007538	XLOC_07816.	transcript	0	0.08663	0.087501	0	0	0.11025	0.299334	0	0
Novel_007539	XLOC_07816.	transcript	0.118776	0	0	0.125973	0.063864	0.083143	0	0.190704	0.412111
Novel_007540	XLOC_07816.	transcript	0.02225	0	0	0	0.005689	0.072124	0.021841	0.00637	0.085047
Novel_007541	XLOC_07816.	transcript	0.008271	0.0127	0.004123	0.144971	0.030077	0.004881	0.013038	0.011831	0.008331
Novel_007542	XLOC_07816.	transcript	0.001886	0.007123	0.005549	0.003952	0.006658	0.00809	0.003395	0	0
Novel_007543	XLOC_07816.	transcript	0.045365	0.081345	0.59334	0.231197	0.225054	0	0.042257	0.374628	0.044465
Novel_007544	XLOC_07816.	transcript	0.492657	0.525468	0.134829	0.091961	0.184902	0.315213	0.619597	0.091737	0
Novel_007545	XLOC_07816.	transcript	0.096009	0.096257	0	0.115638	0	0	0.11803	0	0.370941
Novel_007546	XLOC_07816.	transcript	0.257535	0.464943	0.132822	0.052983	0	0.148776	0.474447	0	0.643187
Novel_007547	XLOC_07820.	transcript	0.151898	0	0	0	0.173074	0	0.076145	0.194393	0
Novel_007548	XLOC_07820.	transcript	0.275732	0.067258	0.03983	0.081823	0.10892	0.155549	0.149772	0.11853	0.084698
Novel_007549	XLOC_07820.	transcript	0.258413	0	0.236277	0.118746	0.091472	0	0	0.080661	0
Novel_007550	XLOC_07820.	transcript	0	0	0.136383	0.076751	0.233027	0	0.235561	0.087831	0

Novel_007551	XLOC_07821.	transcript	1.684039	1.875777	0.415381	1.106713	0.848467	0.078199	0.747078	1.007807	0.305191
Novel_007552	XLOC_07821.	transcript	1.250275	0	0.72794	0.066652	0.485743	0.77479	1.074505	0.607028	0.222154
Novel_007553	XLOC_07831.	transcript	0.096078	0.192324	0.067789	0.044992	0.319307	0.024958	0.60912	0.555141	2.093644
Novel_007554	XLOC_07833.	transcript	0.401438	0.007232	0.477397	0.454498	0.37558	0.480676	0.47053	0.413648	0.210693
Novel_007555	XLOC_07833.	transcript	0.014364	0	0.111344	0.017061	0.215649	0.164585	0.011305	0.049184	0.052332
Novel_007556	XLOC_07833.	transcript	0	0.003236	0	0	0	0	0.34471	0	0
Novel_007557	XLOC_07833.	transcript	0.428441	0.020439	0.25428	0.482723	0.41475	0.336928	0.41851	0.395082	0.285327
Novel_007558	XLOC_07833.	transcript	0.034877	0.034345	0.047789	0.074199	0.048563	0.057919	0.123356	0.046003	0.054536
Novel_007559	XLOC_07833.	transcript	0	0	0.146072	0	0.178252	0.154736	0.165094	0.085616	0.110057
Novel_007560	XLOC_07844.	transcript	0.263644	0	0.13032	0.107967	0.106345	0.093926	0.092258	0.105599	0
Novel_007561	XLOC_07846.	transcript	0.432779	0	0.220411	0.211662	0.218406	0.250807	0.312811	0.387599	0.088306
Novel_007562	XLOC_07846.	transcript	0	0	0	0	0	0	0	0	0
Novel_007563	XLOC_07846.	transcript	0	0	0.020494	0	0.018994	0.075792	0	0.396029	0
Novel_007564	XLOC_07846.	transcript	0.601554	0	0.177985	0.21715	0.238455	0.186632	0.112403	0	0.051243
Novel_007565	XLOC_07846.	transcript	0.02147	0	0	0	0	0	0.071155	0	0
Novel_007566	XLOC_07846.	transcript	0	0	0	0.211339	0	0	0	0	0
Novel_007567	XLOC_07846.	transcript	0.389695	0	0.341947	0.031419	0.247172	0.153216	0.281463	0.386539	0.091291
Novel_007568	XLOC_07847.	transcript	0.138239	0	0.479407	0.122578	0.020239	0.065953	0.219243	0.187656	0.353207
Novel_007569	XLOC_07847.	transcript	0.253135	0.902767	0.019423	0	0.538149	0.01182	0.010318	0	0
Novel_007570	XLOC_07848.	transcript	0.556191	0.261234	0.572631	0.478735	0.63036	0.521943	0.7062	0.489255	0.318885
Novel_007571	XLOC_07848.	transcript	0	0.000712	0	0	0	0	0.003522	0	0
Novel_007572	XLOC_07851.	transcript	0.027163	0.012974	0.037502	0.194481	0.070479	0.04688	0.027097	0.021667	0.043718
Novel_007573	XLOC_07851.	transcript	0	0	0	0	0	0	0	0	0
Novel_007574	XLOC_07851.	transcript	0	0	0	0	0	0	0	0.542565	0
Novel_007575	XLOC_07851.	transcript	0.755056	0.272811	0.307316	0.595816	0.730483	0.841861	0.676045	0.443868	0.550983
Novel_007576	XLOC_07851.	transcript	0.160135	0	0.524025	0	0	0	0.355965	0.056929	0
Novel_007577	XLOC_07851.	transcript	0	0	0	0	0	0	0	0	0
Novel_007578	XLOC_07851.	transcript	0.000509	0	0	0	0	0	0	0	0
Novel_007579	XLOC_07851.	transcript	0	0	0	0	0	0	0	0	0
Novel_007580	XLOC_07860.	transcript	0	0	0.011372	0.023798	0.036533	0	0.069318	0.019272	0
Novel_007581	XLOC_07860.	transcript	0.391677	0.014722	0.264458	0.161229	0.243446	0.17288	0.123664	0.246481	0.141673
Novel_007582	XLOC_07860.	transcript	0	0	0	0.025853	0	0	0.340034	0.055759	0
Novel_007583	XLOC_07862.	transcript	0.147608	0.02128	0.13242	0.104826	0.217668	0.071533	0.30089	0.222832	0.040271
Novel_007584	XLOC_07863.	transcript	0	0	0	0.331023	0	0	0.322616	0.273846	0.239679
Novel_007585	XLOC_07863.	transcript	0.238763	0.296582	2.16694	1.605861	1.438205	1.744337	0.784763	1.316934	1.132972
Novel_007586	XLOC_07867.	transcript	0.315942	0.073071	0.128171	0.18566	0.087362	0.299359	0.238712	0.133307	0
Novel_007587	XLOC_07867.	transcript	0.054964	0.030167	0	0.030659	0.081951	0.218538	0.111263	0.141124	0.063836
Novel_007588	XLOC_07867.	transcript	0.14513	0.476481	0.137969	0.12032	0.168801	0.239259	0.182469	0.281472	0.294725

Novel_007589	XLOC_07868.	transcript	0.05504	0.274762	0.224769	0.193204	0.198883	0.217851	0.20225	0.202574	0.105122
Novel_007590	XLOC_07868.	transcript	0.012607	0.165035	0.49491	0	0.379347	0	0.059102	0	0
Novel_007591	XLOC_07868.	transcript	0.387283	0	0	0.157243	0.088571	2.71862	0	0.869034	0
Novel_007592	XLOC_07868.	transcript	0	0	0	0	0	0	0	0	0
Novel_007593	XLOC_07868.	transcript	0.930261	0.438243	0.086218	0.090315	0.087794	0.146453	1.31482	0.976842	1.154801
Novel_007594	XLOC_07869.	transcript	0.985995	1.080602	0.906358	0.862916	0.854092	0.933052	1.197704	0.900749	0.621557
Novel_007595	XLOC_07872.	transcript	0.270443	1.920048	0.214208	0.205566	0.19422	0.245122	0.363988	0.181505	0.228803
Novel_007596	XLOC_07875.	transcript	0.182162	0.302084	0.128605	0.064875	0.14708	0.152892	0.364323	0.124678	0.108996
Novel_007597	XLOC_07878.	transcript	0.027152	0.0578	0.029492	0.05845	0.082742	0.016829	0.144032	0.080072	0.115712
Novel_007598	XLOC_07879.	transcript	0	0	0.107724	0	0	0	0	0.014073	0.133609
Novel_007599	XLOC_07879.	transcript	0.013349	0	0	0	0	0	0	0.087733	0.008851
Novel_007600	XLOC_07879.	transcript	0.25394	0	0	0	0	0	0	0.013288	0
Novel_007601	XLOC_07879.	transcript	0.004561	0.348519	0.149992	0.269109	0.270074	0.238082	0.358006	0.16498	0.012722
Novel_007602	XLOC_07906.	transcript	0.022162	0	0.072947	0	0	0.020499	0	0	0.036462
Novel_007603	XLOC_07906.	transcript	0.345014	0.191441	0.299597	0.561387	0.485344	0.555436	0.576039	0.656316	0.313709
Novel_007604	XLOC_07908.	transcript	0	0	0	0	0	0.39224	0	0	0
Novel_007605	XLOC_07908.	transcript	0.347373	0.515947	0.314521	0.30453	0.321646	0.000212	0.393958	0.410659	0.203471
Novel_007606	XLOC_07909.	transcript	0.118884	0	0.208581	0.283913	0.170274	0.215394	0.123426	0.226532	0.115639
Novel_007607	XLOC_07936.	transcript	0	0	0	0	0.069017	0	0.046856	0	0
Novel_007608	XLOC_07942.	transcript	0.277693	0.07452	0.133147	0.148792	0.257744	0.212877	0.294423	0.170841	0.155758
Novel_007609	XLOC_07949.	transcript	0	0	0.23511	0.161555	0.414862	0	0	0	0
Novel_007610	XLOC_07949.	transcript	0	0	0	0	0	0	0	0	0
Novel_007611	XLOC_07949.	transcript	0.44463	0.360757	0.23511	0.055447	0	0.437984	0.507059	0.257535	0.351957
Novel_007612	XLOC_07951.	transcript	0.052362	0.106942	0.496983	0.02473	0.422004	0.621266	0.235036	0.080405	0.366676
Novel_007613	XLOC_07951.	transcript	0.331821	0	0	0.57497	0.013353	0	0.330141	0.537131	0
Novel_007614	XLOC_07951.	transcript	0	0	0	0	0	0	0	0	0
Novel_007615	XLOC_07953.	transcript	0.384002	0.329078	0.205032	0.117532	0.278223	0.280907	0.426629	0.367506	0.216785
Novel_007616	XLOC_07954.	transcript	0.206088	0.248868	0.10677	0.14502	0.092373	0.155793	0.244289	0.117664	0.108346
Novel_007617	XLOC_07956.	transcript	0.327202	1.538242	0.142351	0.236774	0.334816	0.384765	0.161753	0.406014	0.166418
Novel_007618	XLOC_07958.	transcript	0.12385	0.388392	0.320258	0.147115	0.1639	0.033828	0.143272	0.384972	0.383803
Novel_007619	XLOC_07958.	transcript	0.182513	0.378521	0.164546	0.224031	0.293608	0.288335	0.537267	0.227956	0.101088
Novel_007620	XLOC_07968.	transcript	0.847979	0	0.227176	0.893136	0.901631	1.261344	0.856108	1.508921	0.09758
Novel_007621	XLOC_07985.	transcript	0	0	0	0	0	0	0.15716	0	0
Novel_007622	XLOC_07985.	transcript	0.236011	0.076748	0.134182	0.075268	0.144013	0.156575	0.119946	0.259699	0.078827
Novel_007623	XLOC_07986.	transcript	0.303921	0.17633	0.317642	0.249822	0.292302	0.386928	0.32773	0.410443	0.247915
Novel_007624	XLOC_07986.	transcript	0	0	0	0.182504	0	0	0	0	0
Novel_007625	XLOC_07986.	transcript	0.420868	0.414923	0.214731	0.122806	0.227386	0.227346	0.458252	0.17491	0.145268
Novel_007626	XLOC_07987.	transcript	1.543644	0.82874	1.389097	1.398121	2.049854	1.109032	1.778308	0.77412	2.163127

Novel_007627	XLOC_07987.	transcript	0.110192	0	0	0	0	0	0	0	0
Novel_007628	XLOC_07987.	transcript	0.133284	0.075415	0.224421	0.125723	0.135334	0.139701	0.140574	0.208265	0
Novel_007629	XLOC_07988.	transcript	0	0	0.068112	0	0	0.059583	0	0.028798	0
Novel_007630	XLOC_07994.	transcript	0.704104	0.009585	0.348516	0.002976	0.028838	0.057769	0.409006	0.805733	0.301827
Novel_007631	XLOC_07994.	transcript	0.432555	0.657523	0.511438	0.58143	0.674222	0.630703	0.679576	0.443048	0.301673
Novel_007632	XLOC_07995.	transcript	0.291628	0.206063	0.206659	0.20876	0.245599	0.267835	0.40276	0.266588	0.184144
Novel_007633	XLOC_07997.	transcript	0.269041	0.363822	0.27911	0.399276	0.403545	0.273066	0.360025	0.350901	0.240463
Novel_007634	XLOC_07997.	transcript	0	0	0	0	0	0	0	0	0
Novel_007635	XLOC_08000.	transcript	0.172187	0.158672	0.369769	0.161134	0.199931	0	0.271339	0	0.130196
Novel_007636	XLOC_08000.	transcript	0	0	0	0	0	0	8.90E-05	0.453268	0
Novel_007637	XLOC_08000.	transcript	0.222245	0.068019	0	0	0	0	0	0	0
Novel_007638	XLOC_08000.	transcript	0	0.083658	0	0.076975	0.000162	0	0.000149	0.136754	0.000148
Novel_007639	XLOC_08000.	transcript	0	0.102284	0.069829	0	0	0	0	0	0
Novel_007640	XLOC_08000.	transcript	0.137247	0.027099	0.201111	0.183942	0.366817	0.254757	0.25811	0.213113	0.253848
Novel_007641	XLOC_08002.	transcript	1.689163	5.560585	2.104706	2.233693	2.688165	2.246216	2.161651	2.54009	3.935353
Novel_007642	XLOC_08002.	transcript	0.181931	0	0	0.101545	0	0.159274	0.210371	0.107306	0.465578
Novel_007643	XLOC_08004.	transcript	0.520249	0.214401	0.584805	0.720052	0.792457	0.650011	1.021689	0.729784	0.598187
Novel_007644	XLOC_08007.	transcript	0.141059	0.198306	0.408712	0.122486	0.142748	0.172535	0.251347	0.228867	0.11193
Novel_007645	XLOC_08007.	transcript	0.311197	0.21838	0.157053	0.430224	0.185393	0.220902	0.301808	0.205922	0.320276
Novel_007646	XLOC_08007.	transcript	0.155136	0.660442	0.148808	0.188563	0.179428	0.275083	0.335407	0.333304	0.189196
Novel_007647	XLOC_08015.	transcript	0.726546	0.031033	0.314195	0.290844	0.263422	0.481236	0.602094	0.23392	0.23667
Novel_007648	XLOC_08015.	transcript	0.650558	0.109126	0.426232	0.441471	0.243202	0.542675	0.55482	0.276676	0.189262
Novel_007649	XLOC_08018.	transcript	0.065059	0	0.041568	0.098861	0	0	0.182278	0.169738	0.137386
Novel_007650	XLOC_08018.	transcript	0	0.497705	0.202518	0.090997	0.136971	0.152603	0.099921	0.088166	0.073126
Novel_007651	XLOC_08020.	transcript	0.038866	0	0.095876	0.321829	0.171638	0.186269	0.164542	0.264039	0
Novel_007652	XLOC_08020.	transcript	0	0	0.01156	0	0	0	0	0	5.130561
Novel_007653	XLOC_08020.	transcript	0	0	0	0	0	0	0	2.071848	0
Novel_007654	XLOC_08020.	transcript	0	0	0.096133	0	1.626699	0	0	6.00E-06	0.115928
Novel_007655	XLOC_08020.	transcript	0.475336	0	0	0	0	0	0	0	0
Novel_007656	XLOC_08020.	transcript	0	0	0	0	0	0	0	0.099701	0
Novel_007657	XLOC_08020.	transcript	0.729972	0.138427	0.016476	3.868418	0.457961	5.355309	0	0.878743	1.266736
Novel_007658	XLOC_08020.	transcript	0	0	0	0	0	0	0	0	0
Novel_007659	XLOC_08020.	transcript	0.378633	3.623564	0.006368	0	0.537678	0	4.551628	0.109623	0
Novel_007660	XLOC_08020.	transcript	0	0	0.522163	0	0	0	0	0	0
Novel_007661	XLOC_08020.	transcript	0	0	0	0	0	0	0.413177	0	0
Novel_007662	XLOC_08031.	transcript	0.015625	0.000684	0	0	0	0	0.022313	0.00495	0
Novel_007663	XLOC_08031.	transcript	1.044103	0.755054	0.796531	0.815503	0.848978	1.047293	1.256167	1.216266	0.656765
Novel_007664	XLOC_08031.	transcript	0	0	0	0	0	0	0	0	0

Novel_007665	XLOC_08033.	transcript	0.236074	0.057958	0.025536	0.030399	0.001321	0.100398	0.315081	0.00809	0.010949
Novel_007666	XLOC_08033.	transcript	0	0	0	0	0.144607	0.039425	0.137322	0.472988	0
Novel_007667	XLOC_08033.	transcript	0	0	0	0	0.258643	0.005106	0	0	0
Novel_007668	XLOC_08033.	transcript	0	0	0	0	0	0.002443	0	0	0
Novel_007669	XLOC_08033.	transcript	0.049395	0.009066	0.553815	0.715937	0.152942	0.291343	0.062245	0.085257	0.984739
Novel_007670	XLOC_08033.	transcript	0.417716	0.624679	0.701908	0.774072	0.907943	0.446066	0.867232	1.049537	0.413544
Novel_007671	XLOC_08033.	transcript	0.171391	0.337164	0.209694	0.539842	0.110167	0.294073	0.37478	0.080432	0
Novel_007672	XLOC_08033.	transcript	0.041324	0	0.055196	0.008531	0.004319	0.043679	0.059967	0.031999	0.302671
Novel_007673	XLOC_08033.	transcript	1.882974	0.752115	2.426998	3.560889	2.665208	2.461975	2.719011	3.082119	1.39275
Novel_007674	XLOC_08033.	transcript	0.005173	0	0.004775	0	0	0	0.000759	0.00205	0
Novel_007675	XLOC_08033.	transcript	0.702882	0	0.93012	0.838386	0	0.961795	0.422143	0.013339	0.535092
Novel_007676	XLOC_08033.	transcript	0.0665	1.110283	0	0.179224	0.685833	0	0	0.597933	0.048311
Novel_007677	XLOC_08033.	transcript	0.007753	0	0	0.046895	0.203149	0	0.121441	0.09101	0.072277
Novel_007678	XLOC_08033.	transcript	0.031868	0.050395	0	0	0.150243	0.013019	0.184264	0.143858	0
Novel_007679	XLOC_08033.	transcript	0.010418	0.061133	0.214221	0.262997	0.157493	0.005286	0.174511	0.14489	0.054208
Novel_007680	XLOC_08033.	transcript	0.257994	0.005355	0.076105	0.120441	0.127343	0.232516	0.293302	0.091065	0.101166
Novel_007681	XLOC_08033.	transcript	0.194789	0	0	0.037571	0.100467	0.122997	0.109671	0.085993	0.153481
Novel_007682	XLOC_08034.	transcript	0.426948	0.5784	0.088505	0.45946	0.152483	0.138808	0.043632	0.299811	0.203033
Novel_007683	XLOC_08034.	transcript	0	0.937284	0	0	0.052089	0	0.66257	0	0
Novel_007684	XLOC_08034.	transcript	0	0	0	0	0	0	0	0	0
Novel_007685	XLOC_08036.	transcript	0.143301	0.149707	0.244777	0.194297	0.237564	0.212703	0.300211	0.200685	0.343757
Novel_007686	XLOC_08038.	transcript	0.876481	0.965882	1.28341	0.959518	1.029105	1.224177	1.148497	1.22643	0.814592
Novel_007687	XLOC_08038.	transcript	0.233097	0	0.329409	0.172421	0.417316	0.242321	0.602526	0.761958	0.127873
Novel_007688	XLOC_08038.	transcript	0.013302	0.001295	0.666594	0.186279	0.367997	0.139959	0.334435	0.50441	0.076508
Novel_007689	XLOC_08038.	transcript	0	0	0	0.272449	0	0	0.03461	0	0.159979
Novel_007690	XLOC_08048.	transcript	0.250965	0.1648	0.39843	0.934602	0.406037	0.540301	0.33109	0.442335	0.195502
Novel_007691	XLOC_08048.	transcript	0.135824	0.033242	0.171035	0.467754	0.219168	0.237803	0.174146	0.417801	0.124234
Novel_007692	XLOC_08050.	transcript	0.339638	0.130485	0.399671	0.107633	0.234438	0.192171	0.437863	0.250471	0.257823
Novel_007693	XLOC_08050.	transcript	0.40424	0.019823	0.178924	0.384635	0.432133	0.271127	0.403518	0.108144	0.285681
Novel_007694	XLOC_08050.	transcript	0.382528	0.344059	0.353418	0.237981	0.203184	0.270124	0.362242	0.396011	0.231761
Novel_007695	XLOC_08050.	transcript	0	0	0	0	0	0	0	0	0
Novel_007696	XLOC_08051.	transcript	1.945002	0.736194	2.086326	2.064826	1.915404	2.283143	2.58256	2.416744	3.167333
Novel_007697	XLOC_08056.	transcript	0.079439	0.767099	0.049324	0.090549	0.121836	0.084895	0.174677	0.073104	0.201765
Novel_007698	XLOC_08056.	transcript	0.0268	0.73832	0	0	0	0	0	0.061754	0
Novel_007699	XLOC_08056.	transcript	0	0.16374	0	0.019344	0	0.070548	0.021397	0	0
Novel_007700	XLOC_08056.	transcript	0.192488	0.299337	0.077096	0	0.028465	0.173692	0.346464	0.116171	0.400852
Novel_007701	XLOC_08056.	transcript	0.506057	0.037541	0.432704	0.50013	0.35379	0.460349	0.523982	0.377747	0.310873
Novel_007702	XLOC_08057.	transcript	0.4947	0	0	0	0	0.03743	0	0	0

Novel_007703	XLOC_08057.	transcript	0.113989	0.188238	0.694259	0.349964	0.419571	0.550731	0.508742	0.48464	0.330343
Novel_007704	XLOC_08059.	transcript	0.77399	0.60759	1.134361	0.770254	0.717568	0.976449	1.35932	1.357123	1.234886
Novel_007705	XLOC_08059.	transcript	0.219221	0.002722	0.136461	0	0	0.074428	0.087059	0.106164	0.077894
Novel_007706	XLOC_08059.	transcript	0	0.36695	0	0.161607	0.230529	0	0	0	0
Novel_007707	XLOC_08061.	transcript	0.011865	0.007795	0.018901	0.353738	0.104789	0.598383	0	0.353564	0.19785
Novel_007708	XLOC_08061.	transcript	0.058323	0.038572	0.018929	0.441029	0.032588	0.302745	0.312725	0.221448	0
Novel_007709	XLOC_08061.	transcript	1.231212	0.437058	1.205666	0.567718	1.042176	0.722144	0.831186	0.707654	0.999161
Novel_007710	XLOC_08064.	transcript	0	0.426892	0.565675	0.256667	0.365962	0.276645	0	0	0
Novel_007711	XLOC_08064.	transcript	0.432718	0.016296	0.041259	0.289229	0.133763	0.179036	0.193315	0.115464	0.002702
Novel_007712	XLOC_08064.	transcript	0.29883	0	0	0	0	0	0	0	0
Novel_007713	XLOC_08064.	transcript	0	0.558162	0.341904	0.879448	0.300913	0.383537	0.490772	0.943983	0.813816
Novel_007714	XLOC_08065.	transcript	0.075177	0.267784	0.14518	0.201505	0.296178	0.180171	0.468543	0.241704	0.225455
Novel_007715	XLOC_08065.	transcript	0	0.852915	0.148941	0	0	0.24565	0	0	0
Novel_007716	XLOC_08065.	transcript	0.374264	0.062189	0.228302	0.218612	0.416877	0.05428	0.401524	0.306203	0.329045
Novel_007717	XLOC_08066.	transcript	0.388434	0.164755	0.329569	0.160225	0.279307	0.363737	0.396737	0.254611	0.167159
Novel_007718	XLOC_08068.	transcript	0.320065	0.073049	0.261918	0.172797	0.212035	0	0.358129	0.191381	0
Novel_007719	XLOC_08068.	transcript	0.198126	0.016936	0	0	0	0	0	0	0
Novel_007720	XLOC_08068.	transcript	0	0	0	0	0.17264	0	0	0	0
Novel_007721	XLOC_08068.	transcript	0.144011	0.04491	0.113003	0.136721	0.164008	0	0.221093	0.172352	0
Novel_007722	XLOC_08068.	transcript	0.226407	0	0.12707	0.172068	0.131817	0.138574	0.23282	0.233343	0.082474
Novel_007723	XLOC_08068.	transcript	0.361023	0	0.526273	0.365548	0.196253	0.579027	0.743474	0.318736	0.48807
Novel_007724	XLOC_08068.	transcript	0.225492	0	0.152469	0	0	0	0	0	0
Novel_007725	XLOC_08068.	transcript	0	0	0	0	0	0	0	0	0
Novel_007726	XLOC_08068.	transcript	0.248091	0	0.103564	0	0	0	0	0	0
Novel_007727	XLOC_08071.	transcript	0.405881	0.466698	0.24933	0.54534	0.462412	0.38651	0.660597	0.617224	0.426973
Novel_007728	XLOC_08071.	transcript	0	0	0.15576	0	0	0	0	0	0
Novel_007729	XLOC_08083.	transcript	0.09567	0	0	0	0.009227	0.024867	0	0.412385	0
Novel_007730	XLOC_08083.	transcript	0	0	0	0	0.108278	0.540203	0.101531	0.006159	0
Novel_007731	XLOC_08083.	transcript	0.160299	0.086949	0.514446	0.264426	0.247395	0	0.303595	0.559459	0.476762
Novel_007732	XLOC_08083.	transcript	0	0	0	0	0.001209	0	0.003827	0	0
Novel_007733	XLOC_08083.	transcript	0.689655	0.235932	0	0.451781	0.432605	0	0.777284	0	0
Novel_007734	XLOC_08084.	transcript	0.205219	0.025231	0.077789	0.100577	0.34084	0.429068	0.264475	0.135756	0.157623
Novel_007735	XLOC_08084.	transcript	0.118798	0	0.104215	0.064216	0.000989	0.168334	0.079795	0.057152	0
Novel_007736	XLOC_08084.	transcript	0.280604	0	0.312107	0.419424	0.392344	0.385231	0.275228	0.199538	0.407928
Novel_007737	XLOC_08084.	transcript	0	0.103122	0	0	0.097	0.088432	0.074279	0.048318	0.146324
Novel_007738	XLOC_08084.	transcript	0.288664	0	0.265775	0.364145	0.344741	0.381081	0.285307	0.198178	0.320348
Novel_007739	XLOC_08086.	transcript	0	0	0	0.021287	0	0	0.010571	0	0
Novel_007740	XLOC_08086.	transcript	0.122167	0.266469	0.242278	0.274567	0.21581	0.21641	0.334562	0.31906	0.241244

Novel_007741	XLOC_08090.	transcript	0	0	0	0.090769	0	0	0	0	0
Novel_007742	XLOC_08090.	transcript	0.298949	0.044077	0.257216	0.081727	0.247481	0.24693	0.292712	0.193152	0.149788
Novel_007743	XLOC_08093.	transcript	0	1.217767	0	0.030365	0.048218	0	0.071728	0.083128	0.220872
Novel_007744	XLOC_08093.	transcript	0.142965	0.487834	0.070428	0.006335	0.039706	0.117148	0.173904	0.054353	0.0552
Novel_007745	XLOC_08093.	transcript	0	0.065238	0	0.14394	0	0	0	0	0
Novel_007746	XLOC_08093.	transcript	0	0.087617	0	0.002849	0	0	0	0	0
Novel_007747	XLOC_08094.	transcript	0.375451	2.921002	0.383494	0.351846	0.439192	0.273211	0.499898	0.093273	0.443316
Novel_007748	XLOC_08095.	transcript	1.411978	2.23761	1.382504	1.29454	1.555177	1.202234	1.912762	1.254219	1.429823
Novel_007749	XLOC_08096.	transcript	0	0	0	0	0	0	0	0	0
Novel_007750	XLOC_08096.	transcript	0.382674	0	0	0.009092	0.010028	0	0.003007	0	0
Novel_007751	XLOC_08096.	transcript	0	0.229674	0.361692	0.252434	0.348404	0.426179	0.548836	0.399174	0.295119
Novel_007752	XLOC_08097.	transcript	0.077013	0.128208	0	0.064545	0	0	0.092914	0.123547	0.08217
Novel_007753	XLOC_08097.	transcript	0.103109	0.014281	0	0.014442	0	0.166182	0.159568	0.088791	0.091759
Novel_007754	XLOC_08100.	transcript	0	0	0.500849	0.702751	0.001146	0	0	0	0
Novel_007755	XLOC_08100.	transcript	0	0	0.267783	1.406384	0.656631	0	0.698332	1.810526	0
Novel_007756	XLOC_08100.	transcript	0.597647	0.060815	0.598311	1.068164	0.686605	1.390762	0.674911	0.537737	0.584747
Novel_007757	XLOC_08100.	transcript	0.011356	0.847104	0.005005	0	0.004346	0	0	0	0
Novel_007758	XLOC_08100.	transcript	0	0	0	0.44367	0	0	0.807762	0	0
Novel_007759	XLOC_08100.	transcript	0	0	1.12502	0	0.412856	0.005716	0	0	0
Novel_007760	XLOC_08100.	transcript	0	0	0	0	0	0	0	0	0
Novel_007761	XLOC_08100.	transcript	2.187145	0.195919	1.779771	1.794485	0.746744	2.6711	1.614099	2.108096	1.51773
Novel_007762	XLOC_08100.	transcript	0	0	0	0	0	0	0	0	0
Novel_007763	XLOC_08100.	transcript	0	0	0	0	0.87361	0	0	0	0
Novel_007764	XLOC_08103.	transcript	0	0.038474	0.024539	0.037351	0	0.054975	0.117813	0.172631	0
Novel_007765	XLOC_08106.	transcript	0.177458	0.487816	0.207923	0.191457	0.297356	0.16221	0.255414	0.316889	0.301653
Novel_007766	XLOC_08109.	transcript	0	0.363828	0	0	0	0	0	0	0
Novel_007767	XLOC_08112.	transcript	0.148001	0	0.114142	0.094296	0	0.046646	0.152404	0.043397	0.529015
Novel_007768	XLOC_08112.	transcript	0.093597	0.105705	0.27924	0.138793	0.201713	0.342391	0.398859	0.317238	0.765178
Novel_007769	XLOC_08112.	transcript	0.112303	0	0	0	0.477555	0	0	0	0
Novel_007770	XLOC_08112.	transcript	0.047017	0.030662	0	0.416068	0.04302	0	0	0.148167	0
Novel_007771	XLOC_08112.	transcript	0	0	0	0.335522	0.251575	0.357236	0	0.389648	0
Novel_007772	XLOC_08112.	transcript	0.055044	0.000169	0.123305	0.006322	0.077579	0	0.511855	0.038768	0.775659
Novel_007773	XLOC_08112.	transcript	0.682608	0.284646	0.771507	0.190899	0	0.568741	0.243076	0.323269	0
Novel_007774	XLOC_08113.	transcript	0.924187	0.102943	0.371249	0.48275	0.576874	0.727667	0.619939	0.2693	0.437537
Novel_007775	XLOC_08113.	transcript	0.760187	0	0.006756	0	0.002584	0.012008	0	0.492976	0.511008
Novel_007776	XLOC_08113.	transcript	0	0	0	0	0	0.086209	0.05254	0.289012	0
Novel_007777	XLOC_08113.	transcript	0.095162	0.080564	0.534428	0.283301	0.422089	0.546837	0.361472	0.316551	0.34054
Novel_007778	XLOC_08113.	transcript	0.13661	0.041436	0.026516	0.083873	0.1788	0.067775	0.215656	0.107271	0.059548

Novel_007779	XLOC_08114.	transcript	0.034343	0	0	0	0.0049	0	0	0.14139	0
Novel_007780	XLOC_08114.	transcript	0.270138	0.006554	0.210358	0.367954	0.166703	0.171628	0.198887	0.184158	0.223789
Novel_007781	XLOC_08114.	transcript	0.005068	0	0	0.172403	0.045788	0.289256	0	0.085059	0
Novel_007782	XLOC_08116.	transcript	0.195375	0	0.19141	0.09854	0	0.004385	0.098885	0.227922	0.119955
Novel_007783	XLOC_08116.	transcript	0.129223	0.291165	0.301701	0.395566	0.353776	0.312216	0.297171	0.197711	0.204706
Novel_007784	XLOC_08118.	transcript	1.044858	0.025803	0.915233	0.792753	0.715816	0.768915	0.913287	0.91754	0.822726
Novel_007785	XLOC_08118.	transcript	1.042635	0.924068	0.780671	0.693173	0.797286	0.645481	1.065544	1.067012	0.592766
Novel_007786	XLOC_08119.	transcript	110.9514	0	116.2797	0	0	129.8427	0	0	0
Novel_007787	XLOC_08119.	transcript	0.038751	2.316643	0.015825	0.377412	0.010616	0.024333	4.101388	2.052837	0.023465
Novel_007788	XLOC_08119.	transcript	0	0	0	0	1.973772	0	0	0	0
Novel_007789	XLOC_08119.	transcript	0.02058	282.8445	0.008149	516.4766	235.5383	0.013049	239.7103	370.6854	221.8461
Novel_007790	XLOC_08122.	transcript	0	0	0.497862	0	0.034836	0	0.043495	0.01578	0
Novel_007791	XLOC_08124.	transcript	0.313365	0.467195	0.369448	0.239618	0.360154	0.457852	0.32866	0.179354	0.131095
Novel_007792	XLOC_08124.	transcript	0.018261	1.272438	0.035405	0.020693	0.034254	0.023406	0.017886	0.442932	0
Novel_007793	XLOC_08137.	transcript	0.19131	0.019568	0.173546	0.067321	0.143119	0.220945	0.099013	0.191695	0.258027
Novel_007794	XLOC_08137.	transcript	0.113669	0	0.132009	0.046449	0.018895	0.025421	0.091188	0.065204	0.093372
Novel_007795	XLOC_08139.	transcript	0.671078	0.600592	0.638082	0.612839	0.823791	0.631436	0.687762	0.615443	0.93947
Novel_007796	XLOC_08139.	transcript	0.238242	0.052373	0.255779	0.184356	0.274313	0.218464	0.190838	0.200769	0.176912
Novel_007797	XLOC_08140.	transcript	0	0	0	0	0	0.106641	0	0.148627	0
Novel_007798	XLOC_08140.	transcript	0	0.006352	0	0	0	0	0	0.043012	0.011938
Novel_007799	XLOC_08140.	transcript	0	0	0	0	0	0	0	0.043854	0
Novel_007800	XLOC_08140.	transcript	0.322889	0.765576	0.265807	0.155167	0.334697	0.097999	0.418703	0.044382	0.218373
Novel_007801	XLOC_08140.	transcript	0	0.089849	0	0.270678	0	0.190942	0.127391	0	0
Novel_007802	XLOC_08140.	transcript	0.222648	0.102429	0.264365	0.21706	0.287636	0.101211	0.173198	0.411606	0.280166
Novel_007803	XLOC_08141.	transcript	0.125954	0	0.280604	0	0.056953	0.214006	0.024474	0.033992	0
Novel_007804	XLOC_08141.	transcript	0.012343	0.024322	0.004364	0.19108	0.326173	0.033256	0.252739	0.451652	0.219867
Novel_007805	XLOC_08141.	transcript	0.021371	0	0	0.346899	0	0.007733	0.19843	0.001509	0.009232
Novel_007806	XLOC_08141.	transcript	0.28091	0.024995	0.125891	0.22622	0.23853	0.222447	0.374213	0.28024	0.087724
Novel_007807	XLOC_08143.	transcript	1.527734	1.575308	2.395023	2.650963	1.895179	1.308086	1.230626	2.430912	1.271785
Novel_007808	XLOC_08145.	transcript	0	0	0.033019	0.077878	0.042906	0	0.226943	0.079844	0.053488
Novel_007809	XLOC_08155.	transcript	0.347424	0.134904	0.202956	0.148891	0.145043	0.269501	0.281191	0.198418	0.21633
Novel_007810	XLOC_08156.	transcript	0	0	0.069942	0.135434	0.07989	0.090708	0	0.109076	0.112286
Novel_007811	XLOC_08156.	transcript	0.016236	0	0	0.127464	0.08848	0.030546	0.00473	0.244736	0.067441
Novel_007812	XLOC_08156.	transcript	0	0	0	0	0.415754	0	0.519534	0.756064	0
Novel_007813	XLOC_08156.	transcript	0	0	0	0.205085	0.15673	0.140989	0	0.28496	0.179414
Novel_007814	XLOC_08156.	transcript	0	0	0	0.164803	0.142218	0.155653	0	0.223543	0.180453
Novel_007815	XLOC_08156.	transcript	0	0	0	0	0	0	0	0	0
Novel_007816	XLOC_08156.	transcript	0.36043	0.272997	0.298979	0.265779	0.173925	0.408932	0.289514	0.29713	0.489599

Novel_007817	XLOC_08156.	transcript	0.880808	0	0.425842	0.700393	0.658521	0.22701	0.444874	0.401097	0.199333
Novel_007818	XLOC_08156.	transcript	0.002377	0.001622	0.424622	0.329584	0.148809	0.228411	0.201587	0.339791	0.216494
Novel_007819	XLOC_08156.	transcript	0	0	0	0	0	0	0	0	0
Novel_007820	XLOC_08156.	transcript	0.769553	0.705648	0.741894	0.337596	0.229258	0.755782	0.815129	0.349241	0.208647
Novel_007821	XLOC_08156.	transcript	0	0	0	0	0	0	0	0	0
Novel_007822	XLOC_08159.	transcript	0.082455	0.050264	0.237036	0.022946	0.360015	0.138585	0.431722	0.245729	0.135995
Novel_007823	XLOC_08159.	transcript	0.608914	0	0.008281	0.068009	0.438546	0.154981	0.00822	0.102547	0.031703
Novel_007824	XLOC_08159.	transcript	0	0.030811	0	0.056199	0.054309	0	0	0	0
Novel_007825	XLOC_08159.	transcript	0.036818	0	0.02375	0	0.085931	0.066912	0.014679	0.068878	0.081959
Novel_007826	XLOC_08159.	transcript	0.205168	0	0.129191	0.027648	0.370291	0.387483	0.141995	0.259508	0.825
Novel_007827	XLOC_08160.	transcript	0	0.013589	0.141638	0.071502	0.39785	0.164788	0.240858	0.169498	0.131956
Novel_007828	XLOC_08160.	transcript	0	0	0	0	0	0.216171	0	0.17563	0
Novel_007829	XLOC_08160.	transcript	0.503017	0.02001	0.218901	0.129186	0.576677	0.174454	0.386904	0.213529	0.139654
Novel_007830	XLOC_08167.	transcript	0.211764	0.437946	0.481541	0.369903	0.361742	0.379791	0.47528	0.273769	0.538678
Novel_007831	XLOC_08171.	transcript	0.00243	0.087103	0.06961	0	0.005332	0.250407	0.004789	0.109536	0
Novel_007832	XLOC_08171.	transcript	0.469451	0.414267	0.361167	0.417591	0.495687	0.383067	0.417989	0.428918	0.446955
Novel_007833	XLOC_08171.	transcript	0	0	0	0	0	0	0.034946	0.153545	0
Novel_007834	XLOC_08172.	transcript	0.208176	0.336901	0.265224	0.049101	0.198677	0.133901	0.378644	0.284679	0.147919
Novel_007835	XLOC_08172.	transcript	0.011036	0	0.133043	0.04599	0.056124	0.18626	0.122388	0.083998	0
Novel_007836	XLOC_08172.	transcript	0.154192	0	0.334642	0	0	0.033531	0.059415	0.191116	0.042308
Novel_007837	XLOC_08172.	transcript	0.089258	6.00E-05	0.119273	0	0	0	0.289296	0	0
Novel_007838	XLOC_08172.	transcript	0	0.215954	0	0.449526	0.319048	0.104446	0.066855	0.24652	0.446178
Novel_007839	XLOC_08177.	transcript	0.44215	0.495037	0.577005	0.594175	0.421666	0.539639	0.504977	0.742664	1.045308
Novel_007840	XLOC_08179.	transcript	0.353996	0	0	0	0	0	0	0.476716	0
Novel_007841	XLOC_08179.	transcript	0	0.026639	0.41028	0.732095	0	0.005206	0	0.028083	0.225294
Novel_007842	XLOC_08179.	transcript	0.322366	1.320559	0.02627	0.013559	0.31613	0.886672	1.114435	0.202496	0
Novel_007843	XLOC_08179.	transcript	0.104426	0	0	0	0.000736	0	0	0	0
Novel_007844	XLOC_08179.	transcript	0	0	0	0	0.660085	0	0	0.248294	0
Novel_007845	XLOC_08179.	transcript	0.777937	0.655108	2.319494	3.954345	2.699221	1.986849	1.878642	2.494061	2.072378
Novel_007846	XLOC_08180.	transcript	0	0	0	0	0	0	0	0	0.237131
Novel_007847	XLOC_08180.	transcript	0.226946	0	0.251292	0.154922	0.120649	0.199648	0.109814	0.152222	0
Novel_007848	XLOC_08180.	transcript	0.225131	0	0.212584	0.146167	0.164653	0.167822	0.222931	0.110707	0
Novel_007849	XLOC_08180.	transcript	0.162676	0.10715	0.216	0.145034	0.157806	0.18777	0.243729	0.122154	0.026179
Novel_007850	XLOC_08180.	transcript	0.117839	0	0.248652	0.186387	0.273865	0.309054	0.123428	0.276094	0.269266
Novel_007851	XLOC_08180.	transcript	0.261762	0	0.207922	0.158936	0.110327	0.171548	0.11078	0.130817	0
Novel_007852	XLOC_08180.	transcript	0.114273	0.019597	0.238538	0.16655	0.466026	0.150791	0.667728	0.357942	0.431844
Novel_007853	XLOC_08180.	transcript	0	0	0	0	0	0	0	0	0
Novel_007854	XLOC_08180.	transcript	0.208176	0	0.250966	0.179203	0.279724	0.241028	0.245798	0.364865	0

Novel_007855	XLOC_08180.	transcript	0	0	0	0.004657	0	0	0.002301	0.002981	0
Novel_007856	XLOC_08181.	transcript	12.59178	19.95361	17.27494	24.49388	19.90851	23.2309	20.5424	22.81203	28.9147
Novel_007857	XLOC_08182.	transcript	0	0.863625	0	0	0	0.013237	0	0	0.013002
Novel_007858	XLOC_08186.	transcript	0.032338	0.205418	0.090864	0.746107	0.442687	0.130056	0.140167	0.578532	0.102199
Novel_007859	XLOC_08186.	transcript	0	0.15437	0.071591	0.284281	0.154367	0.028044	0.150253	0.270464	0
Novel_007860	XLOC_08186.	transcript	0	0.059587	0	0.287028	0.21033	0.03973	0.122645	0.185349	0.058538
Novel_007861	XLOC_08187.	transcript	0.282575	0.51178	0.285123	0.211515	0.232536	0	0.384348	0.322541	0.491089
Novel_007862	XLOC_08197.	transcript	0	0.182051	0.428418	0	0.022502	0.024021	0.247839	0.009281	0.015488
Novel_007863	XLOC_08197.	transcript	0	0.392443	0.158381	0	0	0.217608	0.295779	0	0
Novel_007864	XLOC_08199.	transcript	0.058925	0.07168	0.0371	0.020572	0	0.011406	0.159831	0.034327	0.114369
Novel_007865	XLOC_08200.	transcript	0.479372	1.561192	0.270116	0.495377	0.450621	0.4479	0.797996	0.568037	0.305947
Novel_007866	XLOC_08201.	transcript	0.089442	0.098699	0.362861	0.474254	1.1161	0.254083	0.434542	0.331893	0.11445
Novel_007867	XLOC_08201.	transcript	1.580449	0.016082	1.701646	1.840253	4.03147	1.587877	2.663945	0.855674	1.364631
Novel_007868	XLOC_08201.	transcript	10.44364	7.335917	8.804845	15.41564	8.984175	6.120009	16.20203	13.91299	13.359
Novel_007869	XLOC_08202.	transcript	0.099935	0.119337	0.130323	0.052534	0	0.037877	0.259439	0.08987	0.206639
Novel_007870	XLOC_08204.	transcript	0.019254	0	0	0	0	0.084271	0	0.006457	0
Novel_007871	XLOC_08204.	transcript	0	0	0	0	0	0.357991	0	0	0
Novel_007872	XLOC_08205.	transcript	0	0	0	0	0	0.058467	0.209996	0	0
Novel_007873	XLOC_08211.	transcript	0.03794	0	0.128566	0	1.163876	0.751957	0.966908	0.186844	0.402594
Novel_007874	XLOC_08211.	transcript	0.880862	0.192945	1.275938	1.770744	0.182387	0.797479	1.05277	1.6419	1.370374
Novel_007875	XLOC_08215.	transcript	0.025264	0.258153	0.145121	0.137289	0.153963	0.126686	0.171747	0.165828	0.128667
Novel_007876	XLOC_08234.	transcript	0.138313	0.392437	0.13051	0.103491	0.076347	0.37128	0.086919	0.005441	0.05412
Novel_007877	XLOC_08235.	transcript	181.2027	143.7764	180.1436	168.883	158.8334	159.5951	158.339	233.3125	170.3664
Novel_007878	XLOC_08235.	transcript	0.29405	0.048906	0.440955	0.072589	0.176369	0.680402	0.007731	0.001309	0.008149
Novel_007879	XLOC_08235.	transcript	0.274732	0	0.084059	0.099302	0.10271	0.181218	0.000427	0.007295	9.00E-05
Novel_007880	XLOC_08235.	transcript	0.527831	0	0.024153	0.126067	0.050899	0	0.004023	0.019605	0.105815
Novel_007881	XLOC_08235.	transcript	0	0	0	0.060721	0	0	0.003616	0	0.000424
Novel_007882	XLOC_08235.	transcript	0.012333	0.030592	0.239819	0.12464	0.182065	0.002221	0.029406	0.018431	0.016328
Novel_007883	XLOC_08235.	transcript	0.040241	0	0.015266	0.170543	0	0.022737	0.024258	0.045801	0.026086
Novel_007884	XLOC_08235.	transcript	0.366152	0.062748	0.055862	0.049798	0.056947	0.041067	0.02683	0.001142	0.002375
Novel_007885	XLOC_08235.	transcript	0	0	0	0	0	0	0	0.273368	0
Novel_007886	XLOC_08235.	transcript	0.592553	0.193629	0.253709	0.198966	0.291857	0.380768	0.566459	0.226892	0.225993
Novel_007887	XLOC_08235.	transcript	0	0	0.178262	0.097935	0.149741	0.125114	0	0.704855	0
Novel_007888	XLOC_08235.	transcript	0.103499	0	0.114841	0.058378	0.193668	0.107525	0.032807	0.198582	0.040551
Novel_007889	XLOC_08235.	transcript	0.264636	0.212802	0.379973	0.529297	0.155139	0	0.158888	0.571356	0.661442
Novel_007890	XLOC_08244.	transcript	0.308658	0	0.202527	0.055832	0.175941	0.04299	0.041504	0.052278	0.01318
Novel_007891	XLOC_08247.	transcript	1.330533	0.597551	1.183328	1.682964	1.50426	0.64669	0.621523	0.719391	2.914801
Novel_007892	XLOC_08247.	transcript	0.681283	1.835094	1.232667	3.211545	1.951831	0.389908	1.725676	3.055498	2.445522

Novel_007893	XLOC_08247.	transcript	0.769799	0.409159	0.864294	0.565662	1.172676	2.138137	0.889615	0.796758	0
Novel_007894	XLOC_08247.	transcript	0.267767	0.126403	0.481487	0.278977	0.393792	1.32487	0.508879	0.358972	0
Novel_007895	XLOC_08247.	transcript	0	0	0	0	0	0	0	0	0
Novel_007896	XLOC_08247.	transcript	0	0	0	0	0	0	0	0	0
Novel_007897	XLOC_08247.	transcript	0.013651	0	0.042644	0.022882	0	0.595573	0.018149	0	0
Novel_007898	XLOC_08248.	transcript	0.571647	0.900054	0.596232	0.664316	0.49857	0.810355	0.631624	0.805153	0.523471
Novel_007899	XLOC_08249.	transcript	0.269661	0.267009	0.153877	0.141166	0.192463	0.211265	0.345873	0.288257	0.211584
Novel_007900	XLOC_08249.	transcript	0	0	0	0	0	0	0	0.055558	0
Novel_007901	XLOC_08258.	transcript	0	0.012029	0	0.024327	0.007422	0.005837	0.010854	0.00277	0.005733
Novel_007902	XLOC_08258.	transcript	0	0	0	0	0	0	0	0	0
Novel_007903	XLOC_08259.	transcript	0.00516	0.000893	0	0	0	0	0.001717	0	0
Novel_007904	XLOC_08259.	transcript	0.908486	0.578747	0.377874	0.357671	0.332399	0.461655	0.395216	0.5092	0.103144
Novel_007905	XLOC_08259.	transcript	0.061694	0.066	0.142257	0.104749	0	0.093509	0.073062	0.146885	0.169963
Novel_007906	XLOC_08259.	transcript	0	0.104583	0.084744	0.113977	0.211001	0.077804	0.217571	0.016631	0.303292
Novel_007907	XLOC_08259.	transcript	0.06383	0.139234	0.024605	0.082112	0.050741	0.016072	0.195514	0.178826	0
Novel_007908	XLOC_08259.	transcript	0	0	0	0	0	0	0	0	0.002527
Novel_007909	XLOC_08259.	transcript	0	0	0	0.00191	0	0.004691	0	0	0.030412
Novel_007910	XLOC_08269.	transcript	0.095278	0.151161	0.325197	1.465619	0.459779	0.388358	0.265496	0.72028	0.112916
Novel_007911	XLOC_08283.	transcript	0	0	0	0	0	0	0	0	0
Novel_007912	XLOC_08283.	transcript	0.060966	0.101058	0.133086	0.132307	0.158796	0.041992	1.201401	0.279043	0.394049
Novel_007913	XLOC_08283.	transcript	0	0	0	0	0	0	0	0	0.287263
Novel_007914	XLOC_08289.	transcript	0.325639	0.300729	0.32713	0.332459	0.246897	0.26572	0.411131	0.371899	0.254866
Novel_007915	XLOC_08289.	transcript	0.005956	0	0	0.033387	0	0.013096	0	0.023367	0.015646
Novel_007916	XLOC_08291.	transcript	0.337294	0.031561	0.081957	0.074181	0.126018	0.180021	0.448088	0.166134	0.079613
Novel_007917	XLOC_08292.	transcript	0.367335	0.322553	0.495066	0.227847	0.239917	0.569872	0.214215	0.218431	0.173963
Novel_007918	XLOC_08293.	transcript	0.326077	0.009499	0.352775	0.548794	0.3606	0.297148	0.400493	0.446924	0.238895
Novel_007919	XLOC_08293.	transcript	0.040622	0	0.143382	0	0	0.390665	0.200674	0.110815	0
Novel_007920	XLOC_08293.	transcript	0	0	0	0.172023	0.041243	0.070323	0	0	0
Novel_007921	XLOC_08295.	transcript	1.268778	0.995486	0.884619	0.742222	0.546575	0.735446	1.018644	0.664958	0.575536
Novel_007922	XLOC_08296.	transcript	0.106622	0	0	0.001947	0.046024	0	0.434051	0.408405	0.023833
Novel_007923	XLOC_08296.	transcript	1.559273	0.003017	1.030317	0.576489	0.786617	0.432898	1.674934	0.931483	0.542212
Novel_007924	XLOC_08302.	transcript	0.192436	0.259187	0.312246	0.224574	0.243847	0.298042	0.158637	0.125732	0.109826
Novel_007925	XLOC_08303.	transcript	0	0.33026	0.001173	0	0.004176	0	0.002514	0	0
Novel_007926	XLOC_08303.	transcript	0	0	0	0	0	0.030895	0	0	0
Novel_007927	XLOC_08303.	transcript	0	0	0	0	0	0.001871	0	0	0
Novel_007928	XLOC_08303.	transcript	0.459697	0.248196	0.470525	0.494545	0.471565	0.33855	0.662415	0.216738	0.313975
Novel_007929	XLOC_08303.	transcript	0.21014	0	0	0.085107	0	0	0.080736	0.342866	0
Novel_007930	XLOC_08303.	transcript	0.06779	0.007042	0.282888	0.726779	0.006487	0.3888	0.296909	0.399942	0

Novel_007931	XLOC_08303.	transcript	0.075578	0.010232	0	0.09785	0.059264	0.21908	0.080469	0.159777	0.028202
Novel_007932	XLOC_08303.	transcript	0	0	0.059038	0.310828	0.012907	0.005568	0.064336	0.022875	0
Novel_007933	XLOC_08303.	transcript	0.006827	0	0	0.359818	0.163935	0.074726	0.000514	0.096207	0.028424
Novel_007934	XLOC_08303.	transcript	0	0	0	0	0	0	0	0	0
Novel_007935	XLOC_08306.	transcript	0.163001	0.464179	0.222424	0.072863	0.184533	0.245459	0.188161	0.203891	0.044789
Novel_007936	XLOC_08306.	transcript	0.089059	0	0.047077	0	0.196713	0.026863	0.297197	0.063738	0.026387
Novel_007937	XLOC_08307.	transcript	0.31322	0.213705	0.416576	0.305582	0.398492	0.337117	0.642216	0.458565	0.414163
Novel_007938	XLOC_08312.	transcript	0.171532	0	0	0.104483	0	0.296084	0.05846	0	0
Novel_007939	XLOC_08312.	transcript	0.159595	0.131161	0.273044	0.144503	0.2542	0.110736	0.234449	0.35852	0.133685
Novel_007940	XLOC_08315.	transcript	3.311301	0.192784	3.8531	10.09857	5.062619	3.723009	3.58111	6.6297	2.597524
Novel_007941	XLOC_08325.	transcript	0.099043	0.288625	0.278128	0.12134	0.318475	0.293863	0.243096	0.238508	0.181673
Novel_007942	XLOC_08325.	transcript	0.1742	0.147051	0	0.118673	0	0.007132	0.005494	0	0.000292
Novel_007943	XLOC_08326.	transcript	0.40839	0.265072	0.295754	0.09995	0.27358	0.222454	0.4928	0.410931	0.203251
Novel_007944	XLOC_08328.	transcript	0.433092	0.011356	0.396125	0.378794	0.351078	0.49956	0.424992	0.370114	0.514163
Novel_007945	XLOC_08328.	transcript	0	0	0	0	0.002852	0	0.310677	0.004007	0
Novel_007946	XLOC_08328.	transcript	0	0	0.009175	0.000417	0	0	0	0	0
Novel_007947	XLOC_08328.	transcript	0.185602	0.007552	0.079321	0.0668	0.128456	0.439503	0	0.053817	0.124513
Novel_007948	XLOC_08328.	transcript	0.453532	0.031393	0.086453	0.779694	0.495795	0.389705	0.5396	0.2563	0.097346
Novel_007949	XLOC_08328.	transcript	0.255409	0.189359	0.852298	0	0.211039	0.337971	0	0.134135	0
Novel_007950	XLOC_08328.	transcript	0	0	0	0	0.014347	0	0	0.425102	0.182519
Novel_007951	XLOC_08333.	transcript	1.41996	0	0.243823	0.184094	0.154519	0.314456	0.331243	0.222446	0.045076
Novel_007952	XLOC_08334.	transcript	6.621994	3.253304	11.83414	8.355649	12.35259	10.18004	13.97004	12.04187	18.22368
Novel_007953	XLOC_08334.	transcript	0.010439	0	0	0	0	0.019248	0	3.375906	0
Novel_007954	XLOC_08345.	transcript	0.042342	0.826936	0.035653	0	0.028627	0.072562	0.040495	0.003431	0
Novel_007955	XLOC_08360.	transcript	0.087942	0	0.086383	0.1232	0.178906	0.172736	0.180761	0.033879	0
Novel_007956	XLOC_08367.	transcript	0.127419	0.108497	0.122769	0.122628	0.161481	0.136386	0.148087	0.16108	0.182796
Novel_007957	XLOC_08367.	transcript	0.275614	0.185091	0.341455	0.25927	0.176566	0.188936	0.268526	0.396817	0
Novel_007958	XLOC_08368.	transcript	0.264861	0.16443	0.459338	0.240043	0.198005	0.266976	0.310079	0.231204	0.196071
Novel_007959	XLOC_08370.	transcript	0.22328	0	0.003431	0.035538	0.165815	0.172031	0.100963	0.028356	0.064431
Novel_007960	XLOC_08370.	transcript	0.054785	0.193755	0.231788	0.151548	0.22695	0.084121	0.311757	0.30775	0.151357
Novel_007961	XLOC_08371.	transcript	0.130412	0.153714	0.11319	0.205681	0.08875	0.079113	0.30223	0.211364	0.14379
Novel_007962	XLOC_08372.	transcript	0.008925	0.078518	0.072555	0.016628	0.054732	0.0373	0.062153	0	0.127148
Novel_007963	XLOC_08373.	transcript	0	0	0	0	0.036943	0	0	0.206527	0
Novel_007964	XLOC_08373.	transcript	0.395244	0.256701	0.399251	0.344123	0.38675	0.257855	0.334396	0.317505	0.407219
Novel_007965	XLOC_08373.	transcript	0.001416	0.000467	0.039665	0.014325	0.021611	0	0	0	0.010732
Novel_007966	XLOC_08373.	transcript	0	0	0	0	0	0.320503	0.152269	0.154924	0
Novel_007967	XLOC_08373.	transcript	0	0	0	0	0	0	0.12424	0.043759	0
Novel_007968	XLOC_08373.	transcript	0.018394	0.00883	0.043215	0.029544	0.068042	0.004435	0.168236	0.005566	0.17279

Novel_007969	XLOC_08379.	transcript	0.221556	0.04158	0.097581	0.220394	0.025721	0.137092	0.183883	0.134807	0.029803
Novel_007970	XLOC_08379.	transcript	0	0	0	0	0	0	0.264483	0.045631	0
Novel_007971	XLOC_08382.	transcript	0	0.244274	0	0	0	0	0	0	0.026193
Novel_007972	XLOC_08382.	transcript	0.280976	0.226881	0.142163	0.086822	0.085732	0.161244	0.199468	0.132802	0.033506
Novel_007973	XLOC_08382.	transcript	0.020671	0.272268	0.092045	0.225861	0.081806	0.021431	0.09249	0.090131	0
Novel_007974	XLOC_08383.	transcript	0.157304	0.311179	0.08558	0.161684	0.169258	0.185484	0.397539	0.17826	0.238522
Novel_007975	XLOC_08387.	transcript	0.177273	2.021129	0.055323	0.158667	0.079539	0.276725	0.207433	0.146199	0.098244
Novel_007976	XLOC_08394.	transcript	0.054061	1.327627	0.022926	0.025838	0.020137	0.212031	0.220468	0.02229	0.0243
Novel_007977	XLOC_08435.	transcript	0.105962	0.027772	0.158695	0.051615	0.027109	0.202428	0.246475	0.1137	0.092227
Novel_007978	XLOC_08435.	transcript	0.371622	0.023442	0	0.293108	0.101214	0.588832	0.093469	0.018303	0.158496
Novel_007979	XLOC_08435.	transcript	0	0.613557	0	0.288031	0.319338	0	0.658126	0.663065	0
Novel_007980	XLOC_08435.	transcript	0.411347	0	0.570017	0.41816	0.287089	0.181024	0	0	0.456439
Novel_007981	XLOC_08439.	transcript	0	0	0.009228	0	0	0	0	0	0
Novel_007982	XLOC_08439.	transcript	0.182816	0.255029	0.270252	0.214719	0.122622	0.165718	0.115668	0.184098	0.203755
Novel_007983	XLOC_08440.	transcript	0.065871	0.036436	0.038787	0.027558	0.100889	0	0.089017	0.056477	0.058451
Novel_007984	XLOC_08448.	transcript	0	1.128426	0	0	0	0	0	0	0
Novel_007985	XLOC_08449.	transcript	0.452305	0.553216	0.289897	0.21108	0.268738	0.290359	0.421704	0.023609	0.224341
Novel_007986	XLOC_08449.	transcript	0	0	0	0	0	0	0	0.366497	0
Novel_007987	XLOC_08449.	transcript	0.139995	0.094951	0.196398	0.384086	0.300221	0.210067	0.181421	0.229622	0.171571
Novel_007988	XLOC_08453.	transcript	0.32871	0.46081	0.287052	0.337017	0.332229	0.267762	0.65602	0.412903	0.253668
Novel_007989	XLOC_08453.	transcript	0.113562	0.101815	0.018259	0.088231	0.085372	0.071855	0.257658	0.064758	0.060389
Novel_007990	XLOC_08453.	transcript	0	0.444131	0	0.036004	0	0	0	0.039677	0
Novel_007991	XLOC_08453.	transcript	0.11472	0	0.067447	0	0.060395	0.05	0.288493	0.049019	0
Novel_007992	XLOC_08453.	transcript	0.216492	0.165204	0.146691	0.120969	0.11706	0.133387	0.363883	0.206953	0.046259
Novel_007993	XLOC_08453.	transcript	0.160828	0	0.253917	0.009729	0.466109	0.540418	0	0.021432	0
Novel_007994	XLOC_08453.	transcript	0.044846	0	0	0	0	0	0	0	0.61795
Novel_007995	XLOC_08454.	transcript	0.741115	0.660989	0.660013	0.551146	0.546284	0.479601	0.685007	0.570084	0.453245
Novel_007996	XLOC_08454.	transcript	0	0.000599	0	0	0	0	0	0.010869	0
Novel_007997	XLOC_08454.	transcript	0	0	0	0	0	0	0	0	0
Novel_007998	XLOC_08460.	transcript	0.896745	0.17223	0.369156	0.083341	0.612138	0.349531	0.271746	0.181192	0.39573
Novel_007999	XLOC_08465.	transcript	0.086519	1.084461	0.223169	0.652427	0.208336	0.641094	0.311642	0.274503	0.478596
Novel_008000	XLOC_08465.	transcript	0.050996	0	0	0	0	0	0.229865	0.092447	0
Novel_008001	XLOC_08465.	transcript	0.258447	0.012074	0.315787	0	0.262007	0	0	0.115104	0
Novel_008002	XLOC_08465.	transcript	0	0	0	0	0	0	0	0	0
Novel_008003	XLOC_08466.	transcript	0.017414	0	0.065544	0.112741	0.143926	0.392368	0.132717	0	0
Novel_008004	XLOC_08467.	transcript	0	0.016647	0.036843	0.005729	0.007504	0.009258	0.018078	0.116415	0.007198
Novel_008005	XLOC_08467.	transcript	0.496411	0.134594	0.26713	0.473624	0.307791	0.414001	0.38792	0.447767	0.332952
Novel_008006	XLOC_08468.	transcript	0.345962	0.209998	0.115598	0.724279	0.13027	0.468076	0.674166	0.403473	0.499059

Novel_008007	XLOC_08468.	transcript	0.136838	0.108856	0.305683	1.394142	1.244952	0.181169	0.203621	1.181069	0.076082
Novel_008008	XLOC_08468.	transcript	0	0	0	0	0.000597	0	0	0	0
Novel_008009	XLOC_08474.	transcript	0	0.063659	0	0	0.175708	0	0	0	0.559011
Novel_008010	XLOC_08474.	transcript	0	0	0	0	0.013388	0	0.004766	0	0.152763
Novel_008011	XLOC_08474.	transcript	0	0	0	0.14188	0.328669	0	0.311182	0.184367	0
Novel_008012	XLOC_08474.	transcript	0	0.093649	0	0	0	0	0	0.300028	0
Novel_008013	XLOC_08474.	transcript	0	0	0.884128	0.122987	0	0	0	0.142084	0
Novel_008014	XLOC_08474.	transcript	0.098622	0.083638	0	0.199595	0	0.039249	0	0.165038	0
Novel_008015	XLOC_08474.	transcript	0.622031	0.399528	0.025325	0	0.012933	0.618007	0.004247	0.295707	0.003645
Novel_008016	XLOC_08474.	transcript	0	0	0	0.176582	0.480612	0.035984	0.594687	0.171044	0
Novel_008017	XLOC_08474.	transcript	0.054615	0	0	0.001017	0	0	0.007817	0.00068	0.270516
Novel_008018	XLOC_08474.	transcript	0	0	0	0.135784	0	0	0.377626	0	0.148498
Novel_008019	XLOC_08477.	transcript	0.015432	0	0	0.015896	0.003343	0	0.004255	0	0
Novel_008020	XLOC_08477.	transcript	0.405024	0.331675	0.22763	0.301977	0.336909	0.456919	0.392403	0.435457	0.335286
Novel_008021	XLOC_08486.	transcript	0.016931	0	0.097926	0.149363	0.159226	0	0	0.050191	0.012909
Novel_008022	XLOC_08488.	transcript	0.136789	1.183057	0.090384	0.028661	0.052465	0.04126	0.172125	0.0978	0.393796
Novel_008023	XLOC_08500.	transcript	0.044769	0.050116	0.084286	0.065541	0.137367	0.23166	0.167411	0.102444	0.106113
Novel_008024	XLOC_08503.	transcript	0	0.039534	0.061705	0.470549	0.663458	0.480618	0.80751	0.151819	0.566498
Novel_008025	XLOC_08503.	transcript	0	0.369953	0	0.083434	0	0	0	0	0
Novel_008026	XLOC_08503.	transcript	0	0.458146	0	0.219008	0	0.297342	0	0.095634	0.249215
Novel_008027	XLOC_08503.	transcript	0.448379	0	0	0	0.308541	0	0	0	0
Novel_008028	XLOC_08503.	transcript	0	0.13419	0.294203	0.004999	0	0.029505	0.282295	0.158514	0
Novel_008029	XLOC_08503.	transcript	0	0	0	0	0.344176	0	0	0.014749	0
Novel_008030	XLOC_08503.	transcript	0.279406	0.247258	0.091862	0.371888	0	0.746692	0.045225	0.179613	0.293629
Novel_008031	XLOC_08510.	transcript	0.02243	0.447574	0.023634	0.112916	0.067369	0.162372	0.150975	0.205216	0.146201
Novel_008032	XLOC_08511.	transcript	0.396802	0.269502	0.405554	0.24177	0.474692	0.23215	0.183409	0.271416	0.500753
Novel_008033	XLOC_08511.	transcript	0.766207	0.843741	0.602077	0.431639	0.712312	0.665321	0.909991	1.011623	0.661459
Novel_008034	XLOC_08511.	transcript	0	0	0	0	0	0	0.382569	0.178574	0
Novel_008035	XLOC_08511.	transcript	0	0	0.093672	0.201658	0	0	0	0	0
Novel_008036	XLOC_08511.	transcript	0.004489	0.010088	0.217433	0.227459	0	0.354729	0.003785	0.005623	0.00662
Novel_008037	XLOC_08511.	transcript	0	0	0.042121	0	0	0	0	0.181839	0
Novel_008038	XLOC_08511.	transcript	0.015135	0.004676	0	0.033098	0.031717	0.013568	0.080898	0.190696	0.006686
Novel_008039	XLOC_08511.	transcript	0.266218	1.149792	0.161451	0.107967	0	0	0	0.181135	0.311179
Novel_008040	XLOC_08511.	transcript	0.45355	0	0.174727	0	0	0	0	0	0
Novel_008041	XLOC_08512.	transcript	0.25676	0	0.211916	0.098337	0.266768	0	0.062225	0.207547	0.063289
Novel_008042	XLOC_08512.	transcript	0.065236	0.095591	0.313285	0.192879	0.242318	0.131971	0.340705	0.111306	0.03187
Novel_008043	XLOC_08512.	transcript	0.034391	0	0.030034	0.023693	0	0.133838	0.158111	0.053834	0
Novel_008044	XLOC_08512.	transcript	0.225548	0.908155	0.175466	0.287146	0.269088	0.178963	0.376688	0.158044	0.269163

Novel_008045	XLOC_08518.	transcript	0	0	0.002985	0	0	0.01022	0	0.001941	0
Novel_008046	XLOC_08518.	transcript	0	0	0	0	0	0	0	0	0
Novel_008047	XLOC_08518.	transcript	0	0	0	0	0	0	0	0	0
Novel_008048	XLOC_08518.	transcript	0	0	0	0	0	0	0	0.015057	0
Novel_008049	XLOC_08518.	transcript	0	0	0	0	0	0	0	0	0
Novel_008050	XLOC_08518.	transcript	0.007291	0	0.012323	0	0.005966	0	0.004362	0.000622	0.020657
Novel_008051	XLOC_08518.	transcript	0	0	0	0	0	0	0	0	0
Novel_008052	XLOC_08521.	transcript	0	0.334031	0	0	0.022956	0.02708	0.050135	0.032127	0.0266
Novel_008053	XLOC_08522.	transcript	0.058427	0.029603	0.134584	0.094558	0.164815	0.144454	0.191136	0.092496	0.202444
Novel_008054	XLOC_08523.	transcript	0.049544	0.221791	0	0.174935	0	0	0.015064	0.006027	0.311276
Novel_008055	XLOC_08523.	transcript	0.049738	0.542609	0	0.222123	0	0.3816	0.03477	0.141374	0.48192
Novel_008056	XLOC_08523.	transcript	0.119874	0.381819	1.075098	0.308451	0.868693	0	0.089022	0.619141	0
Novel_008057	XLOC_08523.	transcript	0.291405	0.247389	0	0.313869	0.341161	0	1.27175	0.247823	0
Novel_008058	XLOC_08523.	transcript	0.319069	0	0	0	0	0	0	0.157037	0.429208
Novel_008059	XLOC_08523.	transcript	0	0	0	0	0	1.113815	0	0	0
Novel_008060	XLOC_08524.	transcript	0.224822	1.132416	0.187981	0.102412	0.116703	0.296398	0.272315	0.170773	0.216674
Novel_008061	XLOC_08524.	transcript	0	0	0	0	0	0	0	0	0
Novel_008062	XLOC_08527.	transcript	1.844931	1.066773	2.566378	3.491845	2.974325	2.972745	3.450519	3.653998	2.678824
Novel_008063	XLOC_08527.	transcript	0.109317	0.023899	0.505856	0.160469	0.01786	0.248071	0.223272	0.318134	0.085917
Novel_008064	XLOC_08544.	transcript	0.132948	0.53854	0.084533	0.034884	0.106355	0.134143	0.103929	0.132796	0
Novel_008065	XLOC_08544.	transcript	0.042868	0.296058	0.065354	0.017964	0.098818	0.103442	0.28667	0.134418	0.050803
Novel_008066	XLOC_08544.	transcript	0.125691	0.152254	0.062844	0.084274	0.358068	0	0.17371	0	0.210877
Novel_008067	XLOC_08545.	transcript	0.103041	0.256054	0	0.030565	0.131375	0.369837	0.472051	0	0.290455
Novel_008068	XLOC_08545.	transcript	0.151924	0.359803	0.153008	0.277252	0.093461	0	0.126899	0.340963	0.134276
Novel_008069	XLOC_08554.	transcript	0	0.168872	0	0.056971	0.173695	0.183428	0.189729	0.125895	0.082909
Novel_008070	XLOC_08559.	transcript	0.213693	0.059728	0.192245	0.10903	0.250841	0.154788	0.168233	0.185932	0.138987
Novel_008071	XLOC_08560.	transcript	0.369575	0.160615	0.488666	0.48299	0.468891	0.541477	0.444289	0.432179	0.470176
Novel_008072	XLOC_08560.	transcript	0.009723	0	0.006299	0.010688	0	0.046473	0.181906	0	0.015858
Novel_008073	XLOC_08560.	transcript	0.651971	0.522313	0.45444	0.475013	0.460828	0.49793	0.468195	0.667115	0.339247
Novel_008074	XLOC_08561.	transcript	1.185003	0.072575	1.222384	0.714187	0.730021	1.817205	0.796915	0.701354	0.794745
Novel_008075	XLOC_08561.	transcript	0	0	0	0	0.390796	0	0	0	0
Novel_008076	XLOC_08562.	transcript	0.685776	0.043018	0.699214	0.495953	0.545182	1.144062	0.479549	0.46451	0.461692
Novel_008077	XLOC_08562.	transcript	0.638642	0.031123	0.056166	0.27863	0.338701	0.662309	0.410321	0.253598	0.257465
Novel_008078	XLOC_08562.	transcript	0	0	0	0	0	0	0.253952	0	0
Novel_008079	XLOC_08563.	transcript	0.459936	0.021643	0.336055	0.269396	0.245458	0.392336	0.278702	0.256246	0.254507
Novel_008080	XLOC_08563.	transcript	0.70643	0.033675	0.548563	0.333425	0.374758	0.872729	0.361413	0.372534	0.395881
Novel_008081	XLOC_08564.	transcript	1.347852	0	0.101482	0.15179	0	1.336348	0	0.778135	0.594446
Novel_008082	XLOC_08564.	transcript	0	0	0	0.596279	0	0	0	0	0

Novel_008083	XLOC_08564.	transcript	0	0	0.562015	0	0.694912	0	0	0	0
Novel_008084	XLOC_08564.	transcript	0	0.073837	0.545389	0	0.482549	0	0.838888	0	0
Novel_008085	XLOC_08564.	transcript	0.489842	0.022239	0.498013	0.229283	0.28197	0.445421	0.235724	0.241039	0.112109
Novel_008086	XLOC_0856?.	transcript	0.749903	0	0.38944	0	0.237217	0	0.014407	0	0.008951
Novel_008087	XLOC_0856?.	transcript	0	0.360668	0.221676	0.315215	0.505643	0.970206	0.687723	1.020416	0.187098
Novel_008088	XLOC_0856?.	transcript	0.049222	0.550583	0.150312	0.643819	0	0	0	0	0
Novel_008089	XLOC_0856?.	transcript	0	0	0	0	0	0	0	0	0
Novel_008090	XLOC_0856?.	transcript	0	0	0	0	0	0	0	0	0
Novel_008091	XLOC_0857(.	transcript	0.455403	0.373969	0.423914	0.304748	0.339955	0.383965	0.605241	0.422078	0.261118
Novel_008092	XLOC_0857?.	transcript	0.034529	0	0.1191	0.015431	0.053389	0	0.026085	0.159441	0.14467
Novel_008093	XLOC_0857?.	transcript	0	0	0	0	0	0	0	0	0
Novel_008094	XLOC_08574.	transcript	0	0	0.194732	0.139627	0	0	0	0	0
Novel_008095	XLOC_08574.	transcript	0	0	0	0	0	0	0	0	0
Novel_008096	XLOC_08574.	transcript	0	0	0	0.171639	0.001262	0	0	0	0
Novel_008097	XLOC_08574.	transcript	0.128759	0.20904	0.035416	0.085445	0.235121	0.209554	0.251147	0.280299	0.327269
Novel_008098	XLOC_0857?.	transcript	0	0	0	0	0.146567	0	0	0	0.570914
Novel_008099	XLOC_0857?.	transcript	0.727499	1.084165	0.133602	0.286927	0.580892	0.598204	0.030447	0.745284	0.273579
Novel_008100	XLOC_0857?.	transcript	0.029023	0.297987	0.263	0.001175	0.04397	0	0.001261	0	0.040898
Novel_008101	XLOC_0857?.	transcript	0.548585	0.011227	0.707472	0.443865	0.3153	0	0.045582	0	0
Novel_008102	XLOC_0857?.	transcript	0	0	0	0	0	0	0.00226	0	0
Novel_008103	XLOC_0857?.	transcript	0.077834	0.086227	0.226935	0.078206	0.093045	0.651836	0.781591	0.090537	0.040585
Novel_008104	XLOC_0858?.	transcript	0	1.933878	0	0	0.006408	0.006559	0	0.116357	0
Novel_008105	XLOC_0858?.	transcript	0.215967	0.03989	0	0	0	0	0.087529	0	0
Novel_008106	XLOC_0858?.	transcript	0	0	0.005749	0.011698	0.012835	0	0	0.210488	0
Novel_008107	XLOC_0858?.	transcript	0	0	0	0.007197	0.004067	0	0	0.34102	0.003985
Novel_008108	XLOC_0858?.	transcript	0.565135	0.987287	1.065344	1.102507	1.099994	0.272604	1.714006	0.228825	0.630022
Novel_008109	XLOC_0858?.	transcript	0	0	0	0	0	0	0	0.242663	0
Novel_008110	XLOC_0858?.	transcript	0	0	0	3.40E-05	0	0	0	0.2672	0
Novel_008111	XLOC_0858?.	transcript	0.015085	0.057679	0	0	0	0.008419	0.025159	0.120342	0.003864
Novel_008112	XLOC_0858?.	transcript	0	0	0	0.039487	0.038338	0	0.022302	0.162398	0.046868
Novel_008113	XLOC_0858?.	transcript	0	0	0	1.70E-05	0	0	0	0.21156	0
Novel_008114	XLOC_0858?.	transcript	1.175335	0.010961	0.040848	0	0	1.028707	0.019532	0	0
Novel_008115	XLOC_0858?.	transcript	0	0	0	0	0	0	0	0	0
Novel_008116	XLOC_0858?.	transcript	0	0	0	0	0	0	0	0.071727	0
Novel_008117	XLOC_08584.	transcript	0	0	0.904392	0	0	0	0	0	0.577198
Novel_008118	XLOC_08584.	transcript	1.335833	1.109353	0.182452	0.845212	1.12827	0.702053	1.219404	0.672489	0.258038
Novel_008119	XLOC_08584.	transcript	0.133029	0.086577	0.078102	0.124876	0.046765	0.254993	0.021915	0.216489	0.182517
Novel_008120	XLOC_0858(.	transcript	0.193202	0	0.175381	0.050375	0.08774	0	0.324239	0.095848	0.050295

Novel_008121	XLOC_0858€.	transcript	0	0.19779	0.060118	0.116347	0.244526	0.239987	0.145913	0.124466	0.093432
Novel_008122	XLOC_0858€.	transcript	0.365902	0.01277	0.076739	0.012853	0.041781	0.020475	0.0281	0.137779	0
Novel_008123	XLOC_0859C.	transcript	0.338244	0.073848	0.405197	0.210093	0.586134	0.297221	0.650197	0.337545	0.347884
Novel_008124	XLOC_08591.	transcript	0.402922	0	0.772794	1.113614	1.069067	0.988698	0	0.226284	0.738413
Novel_008125	XLOC_08591.	transcript	0.009435	0	0	0.127655	0	0	0.118093	0.265693	0
Novel_008126	XLOC_08591.	transcript	0.040265	0	0.072836	0.048171	0	0.093508	0	0.027969	0
Novel_008127	XLOC_08591.	transcript	0.081238	0.156305	0.412648	0.051132	0.132595	0.294258	0.154294	0.032842	0.084354
Novel_008128	XLOC_08591.	transcript	0.367622	0.019126	0.000587	0	0	0	0	0	0.012813
Novel_008129	XLOC_08591.	transcript	0	0	0.046791	0.070751	0.143303	0.046338	0.132669	0.020032	0.059098
Novel_008130	XLOC_0859€.	transcript	0.211509	0	0.155361	0.253467	0.158829	0.187402	0.260825	0.17387	0.185564
Novel_008131	XLOC_0859€.	transcript	0.206034	0.232294	0.227729	0.131003	0.178661	0	0	0.173391	0
Novel_008132	XLOC_0859€.	transcript	0.515307	0.348943	0	0	0.676221	0.43356	0.35948	0.284589	0.339448
Novel_008133	XLOC_0859€.	transcript	0.112052	0	0.408815	0.322618	0	0	0.684449	0.331009	0
Novel_008134	XLOC_0859€.	transcript	0	0	0	0.122788	0	0	0	0	0.058949
Novel_008135	XLOC_0859€.	transcript	0	0	0	0.039668	0	0	0	0	0.187913
Novel_008136	XLOC_0859€.	transcript	0	0.127346	0.184865	0.044205	0.136422	0.283995	0.195196	0.247973	0.078289
Novel_008137	XLOC_0859€.	transcript	0.678489	0.057898	0.136257	0.079773	0.196477	0.102655	0.38378	0.229852	0.02583
Novel_008138	XLOC_0859€.	transcript	0.117397	1.996324	0.15652	0.082938	0.096374	0.113166	0.167424	0.104572	0.337264
Novel_008139	XLOC_0860C.	transcript	2.452415	2.410552	2.715364	3.58981	2.954	2.584803	3.155715	3.936731	3.260454
Novel_008140	XLOC_08601.	transcript	0.37866	0.087808	0.778359	1.209827	0.901991	0.428703	0.465714	0.360653	0.462107
Novel_008141	XLOC_08604.	transcript	0.183934	0.145976	0.151254	0.078133	0.163522	0.101421	0.217574	0.191594	0.051683
Novel_008142	XLOC_08604.	transcript	0.107385	0.051975	0.156425	0.073442	0.087306	0.191564	0.395007	0.229368	0.219796
Novel_008143	XLOC_08604.	transcript	0.145127	0	0.034388	0.095324	0.116107	0	0.03649	0.040295	0.057824
Novel_008144	XLOC_08612.	transcript	0	0	0	0	0	0	0	0	0
Novel_008145	XLOC_08612.	transcript	0.0871	0.036416	0.074923	0.028955	0.205176	0	0.111898	0.09542	0
Novel_008146	XLOC_0861З.	transcript	0.18407	1.275186	0.150895	0.069654	0.067952	0.255993	0.221851	0.037009	0.174414
Novel_008147	XLOC_0861[illegible].	transcript	0.118242	1.474243	0.605731	0.637701	0.121088	0.152491	0.588004	0.487963	0.155962
Novel_008148	XLOC_0861[illegible].	transcript	0.325919	0.45179	0.28711	0.11282	0.317322	0.492225	0.259712	0.434463	0.173761
Novel_008149	XLOC_0861[illegible].	transcript	0	0	0	0.197996	0	0	0	0	0
Novel_008150	XLOC_0861[illegible].	transcript	0.480904	0.329471	0.291824	0.209619	0.473722	0.435002	0.586584	0.263577	0.540112
Novel_008151	XLOC_0861€.	transcript	1.395452	1.581139	1.666543	2.164507	1.660058	1.709797	1.89886	1.195552	2.705645
Novel_008152	XLOC_08617.	transcript	0.373081	0.322977	0.393846	0.254781	0.312002	0.265766	0.334172	0.315045	0.232548
Novel_008153	XLOC_08617.	transcript	0.296379	0.420303	0.181229	0.161338	0.219757	0.120922	0.165966	0.163038	0.118776
Novel_008154	XLOC_0861€.	transcript	0	0.595395	0.063698	0	0	0	0	0.021278	0
Novel_008155	XLOC_0861€.	transcript	0	0.658242	0	0	0	0	0	0	0.135649
Novel_008156	XLOC_0861€.	transcript	0	1.009729	0.047267	0	0	0	0.033437	0.025513	0
Novel_008157	XLOC_0862C.	transcript	0.149841	0.780366	0.050663	0.036171	0.039171	0.1733	0.174984	0.103414	0.124685
Novel_008158	XLOC_08627.	transcript	0.024811	0.049476	0.144221	0.11298	0.101461	0.074143	0.02771	0.226809	0.044105

Novel_008159	XLOC_08634.	transcript	0.891517	0.28302	0.804244	0.673346	0.833917	0.860503	1.07215	1.030773	1.225581
Novel_008160	XLOC_08634.	transcript	0	0	0.008041	0	0.018266	0	0.014654	0	0.002192
Novel_008161	XLOC_08634.	transcript	0.000406	0	0.000204	0	0.000726	0.000283	0	0.016146	0
Novel_008162	XLOC_08642.	transcript	0.153951	0.117625	0.032583	0.055876	0.077814	0.111881	0.140764	0.048891	0.007697
Novel_008163	XLOC_08646.	transcript	0.302444	0.011089	0.299727	0.244375	0.261904	0.123221	0.143762	0.256608	0.308349
Novel_008164	XLOC_08654.	transcript	0.919821	0.84738	0.332795	0.467097	0.689326	1.244284	0.296435	0.904522	0.327858
Novel_008165	XLOC_08654.	transcript	0	0.12044	0.632187	0	0	0.071006	1.242077	0.264683	0.001876
Novel_008166	XLOC_08655.	transcript	0	0	0	0	0	0.689766	0	0	0
Novel_008167	XLOC_08655.	transcript	0	0	0	0	0	0	0	0	0
Novel_008168	XLOC_08655.	transcript	0	0.016594	0.065889	0	0	0.513654	0.009983	0.010085	0.426665
Novel_008169	XLOC_08655.	transcript	0.050771	0.039611	0.032296	0.07201	0.231173	0	0.119075	0.109037	0
Novel_008170	XLOC_08655.	transcript	0.10315	0.01099	0.020794	0.123675	0.103464	0.000841	0.01585	0.003659	0
Novel_008171	XLOC_08655.	transcript	0.1106	0	0	0	0	0	0.197886	0	0
Novel_008172	XLOC_08655.	transcript	0	0.318996	0.325436	0	0.262955	0	0	0	0
Novel_008173	XLOC_08655.	transcript	0	0.024007	0.001645	0.059561	0	0.20714	0.009232	0.022529	0.008756
Novel_008174	XLOC_08655.	transcript	0.463883	0.308743	0.254807	0.371579	0.001412	0	0.456502	0.618086	0.092854
Novel_008175	XLOC_08656.	transcript	0.10124	0.067126	0.117879	0.220498	0.17603	0.07329	0.151434	0.199785	0.103345
Novel_008176	XLOC_08656.	transcript	0.028929	0.290966	0.463331	0.106359	0.350095	0.127427	0.196486	0.161056	0.364057
Novel_008177	XLOC_08662.	transcript	0	0	0	0	0.009963	0	0	0.00717	0.030964
Novel_008178	XLOC_08662.	transcript	0.521251	0.020448	0.446667	0.436727	0.406344	0.587983	0.511158	0.523325	0.432277
Novel_008179	XLOC_08662.	transcript	0.124553	0	0.098273	0	0	0.100434	0.103088	0	0.052098
Novel_008180	XLOC_08662.	transcript	0.001842	0	0.013487	0.019081	0.08624	0.011482	0.239582	0.059825	0.006245
Novel_008181	XLOC_08669.	transcript	0.120913	0.364335	0.011076	0.249469	0	0	0.058348	0.538227	0
Novel_008182	XLOC_08669.	transcript	0	0	0	0	0	0	0.164698	0	0
Novel_008183	XLOC_08669.	transcript	0	0	0	0	0	0	0.216711	0	0
Novel_008184	XLOC_08669.	transcript	0.39148	0.442569	0.57174	0.232911	0.616222	0.842936	0.119124	0.080526	0.165146
Novel_008185	XLOC_08676.	transcript	0.175508	0.080012	0	0.0847	0	0	0.34333	0.116903	0
Novel_008186	XLOC_08687.	transcript	0.178227	0.024915	0.045627	0.044944	0.033032	0.088744	0.164224	0.062659	0.083977
Novel_008187	XLOC_08712.	transcript	0	0.076092	0.05027	0	0	0.341947	0.16195	0	0.100223
Novel_008188	XLOC_08712.	transcript	0.253994	0.059379	0.167467	0.128206	0.164366	0.083458	0	0.129072	0.048495
Novel_008189	XLOC_08712.	transcript	0.373604	0.36514	0.323803	0.218887	0.191186	0.279161	0.340658	0.339361	0.181634
Novel_008190	XLOC_08712.	transcript	0	0	0	0	0.028183	0	0.014448	0	0
Novel_008191	XLOC_08713.	transcript	0.114688	0.008327	0.090319	0	0.42218	0	0.133703	0.00206	0
Novel_008192	XLOC_08713.	transcript	1.857005	0	1.080452	1.444449	1.196873	1.352313	2.187631	1.165666	0.780953
Novel_008193	XLOC_08714.	transcript	0.184876	0	0.082661	0.106959	0.166336	0.17324	0.214088	0.074461	0.1079
Novel_008194	XLOC_08715.	transcript	0.457907	0	0.257345	0.10212	0.121568	0.164382	0.71862	0.34626	0.128114
Novel_008195	XLOC_08721.	transcript	0.820329	0	0.02782	0.047027	0.166598	0	0.201037	0.187901	0.047254
Novel_008196	XLOC_08721.	transcript	0.285558	0	0.146062	0	0.180435	0.014865	0.074906	0.09416	0.070132

Novel_008197	XLOC_08732.	transcript	0.882939	0.972289	0.67334	0.558399	0.683711	0.627754	0.953039	0.625851	0.36357
Novel_008198	XLOC_08741.	transcript	0	0	0	0	0	0	0	0	0
Novel_008199	XLOC_08741.	transcript	0.165099	0	0.120006	0.279128	0	0	0	0	0
Novel_008200	XLOC_08741.	transcript	0	0	0	0	0.469519	0.210236	0.173583	0.167844	0.181655
Novel_008201	XLOC_08741.	transcript	0	0	0	0	0	0	0	0.181118	0
Novel_008202	XLOC_08741.	transcript	0.065475	0	0.017925	0.379162	0.045646	0	0.008789	0	0.010229
Novel_008203	XLOC_08744.	transcript	0.105589	0	0.088202	0.279574	0.234464	0.078046	0.19978	0.453321	0
Novel_008204	XLOC_08755.	transcript	0.4583	0.146187	0.466947	1.724496	0.763183	0.483963	0.931601	1.105641	0.491452
Novel_008205	XLOC_08756.	transcript	0.166089	0.154915	0.149791	0.049938	0.036137	0.073891	0.193737	0.105517	0.021042
Novel_008206	XLOC_08759.	transcript	0.170745	0.440176	0.08906	0.043124	0.052218	0.059542	0.16104	0.131823	0.308457
Novel_008207	XLOC_08759.	transcript	0.693858	1.646909	1.468786	1.265028	1.420558	1.728204	1.148754	0.910449	2.89914
Novel_008208	XLOC_08759.	transcript	0	0.027292	0.001567	0	0	0	0	0.122562	0
Novel_008209	XLOC_08759.	transcript	0.138234	0.038019	0.356947	0.378944	0.36973	0.18037	0.221256	0.175663	0.139585
Novel_008210	XLOC_08766.	transcript	0.511325	0.320511	0.362028	0.332083	0.412241	0.284199	0.410966	0.297518	0.154008
Novel_008211	XLOC_08766.	transcript	0.00025	0.264859	0.011634	0	0.065366	0	0.295058	0.228491	0.259173
Novel_008212	XLOC_08768.	transcript	0	0	0.146164	0.188792	0.240499	0.12108	0.683598	0.219396	0.133772
Novel_008213	XLOC_08769.	transcript	1.738736	1.653861	2.040048	1.613423	1.435683	2.100086	2.018851	2.175766	1.408962
Novel_008214	XLOC_08770.	transcript	0.073731	0	0.206338	0	0.085382	0.025483	0.07482	0.028299	0
Novel_008215	XLOC_08774.	transcript	0.234273	0	0	0.187002	0.000267	0	0.004473	0.223381	0
Novel_008216	XLOC_08774.	transcript	0.177007	0	0.002272	0.002039	0.19006	0	0.278072	0.001857	0
Novel_008217	XLOC_08774.	transcript	0.118142	0	0.144592	0	0.004477	0.148875	0.02338	0	0.041697
Novel_008218	XLOC_08782.	transcript	0.824666	0.886528	1.566932	1.591717	1.356565	1.510256	1.363038	1.924028	1.393819
Novel_008219	XLOC_08785.	transcript	0	0	0.093737	0.063568	0	0	0.077649	0	0.084937
Novel_008220	XLOC_08785.	transcript	0.211323	0.116527	0.008459	0	0.158298	0	0.14411	0.251317	0.01038
Novel_008221	XLOC_08792.	transcript	0.917185	5.353243	0	0	0	0	0.948032	0.715767	2.437279
Novel_008222	XLOC_08792.	transcript	0.27525	0.112485	0.534421	0.673176	0.593148	0.671208	0.111263	0.263308	0.368975
Novel_008223	XLOC_08792.	transcript	0.337579	0.59859	0.735659	0.458251	0.681633	0.501436	0.4176	0.203229	0
Novel_008224	XLOC_08795.	transcript	0.387922	0.425294	0.258949	0.2872	0.492392	0.282866	0.575762	0.527466	0.211642
Novel_008225	XLOC_08799.	transcript	0.315374	0.126845	0.414898	0.325895	0.148294	0.244076	0.223303	0.237735	0.067157
Novel_008226	XLOC_08799.	transcript	0.197156	0.291302	0	0	0.156486	0.121256	0.378905	0.247125	0.042022
Novel_008227	XLOC_08800.	transcript	0.392104	0.163385	0.806357	1.388685	0.512262	0.629023	0.529048	2.345476	0.00341
Novel_008228	XLOC_08800.	transcript	0.292999	0.036126	0.303865	0.14604	0.370136	0.066443	0.123226	0.07956	0.334844
Novel_008229	XLOC_08800.	transcript	0.127673	0.016377	0.020144	0.03171	0.101735	0.076107	0.181619	0.073208	0
Novel_008230	XLOC_08800.	transcript	0.478442	0.0552	0.217017	0.185658	0.027601	0.255893	0.506015	0.348441	0.048738
Novel_008231	XLOC_08804.	transcript	0.354545	0.271628	0.308892	0.148096	0.269508	0.324473	0.421791	0.218764	0.245045
Novel_008232	XLOC_08810.	transcript	1.322413	1.824496	1.438785	1.689198	1.880238	1.496452	2.08061	1.526513	2.207017
Novel_008233	XLOC_08818.	transcript	0	0	0.04778	0.131956	0.050479	0	0	0	0
Novel_008234	XLOC_08818.	transcript	0.446709	1.973227	0.393821	0.617061	0.743929	0.813654	0.861947	0.643559	1.382002

Novel_008235	XLOC_08818.	transcript	1.278263	1.175561	1.21142	1.118497	0.990341	0.899788	0.579279	1.448717	0.759839
Novel_008236	XLOC_08818.	transcript	0	0	0	0.000547	0	0	0	0	0
Novel_008237	XLOC_08818.	transcript	0	0.650256	0.061557	0.021043	0.016453	0	0	0	0
Novel_008238	XLOC_08818.	transcript	0.060594	0.47823	0	0	0	0.110488	0.077768	0	0.065681
Novel_008239	XLOC_08818.	transcript	0	0.718088	0.026209	0.017936	0	0.169988	0.112016	0	0
Novel_008240	XLOC_08819.	transcript	0.186611	0.362725	0.145329	0.094809	0.136759	0.164885	0.140568	0.093752	0.212386
Novel_008241	XLOC_08819.	transcript	0	0.173551	0	0	0	0	0	0.073342	0.072332
Novel_008242	XLOC_08819.	transcript	0.219509	0.571865	0.117119	0.089806	0.209092	0.195839	0.18933	0.184526	0.125916
Novel_008243	XLOC_08820.	transcript	0.034664	0.062295	0.126759	0.128298	0.102166	0.075253	0.229639	0.139722	0.174146
Novel_008244	XLOC_08821.	transcript	0.177625	0.193849	0.07418	0.213002	0.296567	0.154358	0.353101	0.423673	0.321004
Novel_008245	XLOC_08821.	transcript	0.067803	0.349647	0.13323	0	0	0.50988	0	0	0.181084
Novel_008246	XLOC_08828.	transcript	0.062516	0	0	0	0	0	0	0.000727	0
Novel_008247	XLOC_08828.	transcript	0.286257	0.610259	0.369693	0.575077	0.316488	0.293419	0.553268	0.667401	0.407179
Novel_008248	XLOC_08832.	transcript	0	0	0	0	0	0	0	0.257348	0
Novel_008249	XLOC_08833.	transcript	0	0	0	0.39481	0.379064	0	0	0.347367	0
Novel_008250	XLOC_08833.	transcript	0.452353	0.307278	0.385574	0.313747	0.283885	0.385014	0.521021	0.116532	0.60521
Novel_008251	XLOC_08833.	transcript	0	0	0	0	0	0	0	0	0
Novel_008252	XLOC_08838.	transcript	0.001751	0.47016	0.002329	0	0	0.642914	0	0.434884	0.15737
Novel_008253	XLOC_08838.	transcript	0.460697	0.191914	0.131542	0.076038	0.778794	0.266678	0.779478	0.613867	0.002105
Novel_008254	XLOC_08838.	transcript	0.001403	0.343364	0.001721	0	0	0	0	0.385171	0
Novel_008255	XLOC_08838.	transcript	1.222567	0.83013	0.001088	0	0.560459	1.105844	0.010148	0.590648	0.022866
Novel_008256	XLOC_08838.	transcript	0.094899	0	0.584472	0.889811	0.067825	0	0.290336	0	0.017644
Novel_008257	XLOC_08838.	transcript	0	0	0.140369	0.136821	0	0	0.578396	0	0
Novel_008258	XLOC_08838.	transcript	0	0.00073	0.1741	0.165026	0	0	0.825817	0.000859	0.589267
Novel_008259	XLOC_08838.	transcript	0.178213	0	0	0.240684	0	0.079281	0.113871	0	0.039788
Novel_008260	XLOC_08838.	transcript	0.000326	0	0	0.000405	0.004213	0	0	0	0
Novel_008261	XLOC_08838.	transcript	0.114588	0	0	0	0.158858	0.014841	0	0	0
Novel_008262	XLOC_08838.	transcript	0	0	0.037873	0	0.053209	0.030272	0.039016	0.181534	0
Novel_008263	XLOC_08838.	transcript	0.001252	0.013949	0.011326	0.007204	0.027726	0.014606	0	0.004742	0.012345
Novel_008264	XLOC_08838.	transcript	0.009548	0.047603	0.053973	0.00488	0.075753	0.001959	0.009326	0.036078	0.028816
Novel_008265	XLOC_08838.	transcript	0	0	0	0	0	0.013443	0	0	0
Novel_008266	XLOC_08838.	transcript	0.083967	0	0	0	0	0	0.162759	0	0
Novel_008267	XLOC_08838.	transcript	0	0	0.081168	0.033226	0.003797	0.075185	0	0	0.116138
Novel_008268	XLOC_08867.	transcript	0.000772	0	0	0	0.028055	0	0	0	0
Novel_008269	XLOC_08867.	transcript	0.578629	0.031201	0.402618	0.259858	0.553664	0.204324	0.535977	0.517685	0.311087
Novel_008270	XLOC_08867.	transcript	0	0	0	0	0	0	0.006409	0.192845	0
Novel_008271	XLOC_08880.	transcript	0.522313	0.14536	0	0.000591	0.597587	0.764696	0.000606	0.218871	0.619
Novel_008272	XLOC_08880.	transcript	0	0	0.438891	0.715323	0	0	0.806694	0.636485	0

Novel_008273	XLOC_08880.	transcript	0	1.027189	0	0.00259	0	0	0	0	0
Novel_008274	XLOC_08883.	transcript	0.044709	0.184036	0.160707	0	0	0.350806	0.347358	0.196421	0
Novel_008275	XLOC_08883.	transcript	0.235814	0.213483	0.278736	0.209732	0.336329	0	0.393557	0.236114	0.172566
Novel_008276	XLOC_08883.	transcript	0.036245	0.187027	0.018448	0.029443	0	0.258527	0	0.05877	0
Novel_008277	XLOC_08884.	transcript	0.12871	0.127441	0.203238	0.079175	0.119424	0.060741	0.102924	0.128285	0.079882
Novel_008278	XLOC_08884.	transcript	0.074972	0.077632	0	0	0	0	0	0	0
Novel_008279	XLOC_08885.	transcript	0.064937	0.085749	0.116635	0.077658	0.427894	0.251152	0.062791	0.010315	0.180851
Novel_008280	XLOC_08885.	transcript	0.44062	0.223401	0.156686	0.095307	0.258626	0.329921	0.181032	0.048913	0.032653
Novel_008281	XLOC_08885.	transcript	0.268823	0.164136	0.254579	0.138664	0.146351	0.270456	0.261936	0.210466	0.162435
Novel_008282	XLOC_08886.	transcript	0.015492	0	0.18535	0	0.189085	0.168101	0.069519	0.062001	0.11016
Novel_008283	XLOC_08887.	transcript	0.307187	0.436878	0.454047	0.410855	0.283363	0.453832	0.463817	0.386032	0.366716
Novel_008284	XLOC_08900.	transcript	0.218137	0.297841	0.164897	0.094974	0.094118	0.329157	0.25006	0.201404	0
Novel_008285	XLOC_08900.	transcript	0	0	0.007043	0.032935	0.001404	0.000505	0.004167	1.00E-06	0
Novel_008286	XLOC_08900.	transcript	0.002557	0.002019	0	0.039386	0.193457	0	4.20E-05	0	0
Novel_008287	XLOC_08900.	transcript	0.03328	0	0.001487	0.000761	0.01163	0	0	0.025657	0.02185
Novel_008288	XLOC_08900.	transcript	0.000879	0	0.033705	0.00066	0	0	0	0.013835	0.020995
Novel_008289	XLOC_08900.	transcript	0.042523	0.042198	0.060901	0.014412	0.02241	0.025363	0.035066	0.014446	0.103498
Novel_008290	XLOC_08900.	transcript	0	0.109035	0.028701	0.407449	0	0.465272	0	0.062557	0.022811
Novel_008291	XLOC_08901.	transcript	0.160316	0	0.118762	0.05478	0.102028	0.036434	0.045169	0.070547	0.035708
Novel_008292	XLOC_08904.	transcript	0.299744	0	0.041243	0.118374	0.114177	0.120922	0.69259	0.030925	0.066535
Novel_008293	XLOC_08904.	transcript	0	0.046091	0.189911	0.049667	0	0	0.113028	0.195396	0
Novel_008294	XLOC_08904.	transcript	0	0	0.049362	0.140463	0	0	0.209795	0	0
Novel_008295	XLOC_08904.	transcript	0.156196	0.064992	0.03627	0.042968	0.031949	0.08754	0.319974	0.039159	0.029802
Novel_008296	XLOC_08908.	transcript	0.019501	0	0.003717	0	0	0	0.006789	0.120288	0
Novel_008297	XLOC_08908.	transcript	0	0	0.004701	0.006815	0	0	0	0.114681	0
Novel_008298	XLOC_08908.	transcript	1.868332	0	0	0	0	0	0	0	0
Novel_008299	XLOC_08908.	transcript	0.343591	0.098183	0.929078	1.126045	0.682829	0.730961	1.184393	0.871615	0.542919
Novel_008300	XLOC_08908.	transcript	0.284196	0.020393	0.121543	0.577817	0.145592	0.101489	0.680196	0.41468	0.099317
Novel_008301	XLOC_08908.	transcript	0.193009	0.20258	0.362588	0.818217	0.202753	0.120852	0.544631	0.537915	0.042916
Novel_008302	XLOC_08911.	transcript	0	0.177548	0	0.224268	0	0.096696	0	0.105303	0
Novel_008303	XLOC_08911.	transcript	0	0.269967	0	0.044593	0	0.009215	0	0.085397	0
Novel_008304	XLOC_08911.	transcript	0	0	0.059355	0.243453	0.313198	0.078088	0.001274	0.012411	0.086998
Novel_008305	XLOC_08911.	transcript	0	0.022074	0.001385	0	0.084533	0	0	0.134365	0
Novel_008306	XLOC_08911.	transcript	0	0	0.006129	0	0	0	0	0.115185	0
Novel_008307	XLOC_08911.	transcript	0	0	0.099377	0	0.005174	0	0.104253	0.147194	0
Novel_008308	XLOC_08911.	transcript	0	0	0.015071	0.235582	0.004936	0	0.323694	0.071796	0
Novel_008309	XLOC_08911.	transcript	0.362743	0.045701	0.308067	0.069417	0.163475	0.338981	0.196176	0.091086	0.383853
Novel_008310	XLOC_08912.	transcript	0	0	0.036548	0.506422	0.075779	0.134929	0.045048	0.06025	0.030122

Novel_008311	XLOC_08912.	transcript	0.164278	0	0.09478	0.699547	0.125511	0.055683	0.125068	0.200333	0.191455
Novel_008312	XLOC_08912.	transcript	0	0	0	0.922894	0.20299	0.034532	0.078222	0.221481	0
Novel_008313	XLOC_08912.	transcript	0	0	0	0	0	0.038774	0	0	0
Novel_008314	XLOC_08912.	transcript	0	0	0	0	0	0	0	0	0
Novel_008315	XLOC_08912.	transcript	0	0	0	0	0	0	0	0	0
Novel_008316	XLOC_08920.	transcript	0.159197	0.025475	0.082964	0.455714	0.187279	0.189364	0.120462	0.152757	0.103336
Novel_008317	XLOC_08922.	transcript	4.983275	0.077461	5.95089	4.777864	5.367756	5.12059	6.182544	6.702532	15.10655
Novel_008318	XLOC_08933.	transcript	0.222492	0	0.111874	0.172508	0.132521	0.104571	0.144908	0.172967	0.053036
Novel_008319	XLOC_08933.	transcript	0	0	0	0	0	0	0	0	0
Novel_008320	XLOC_08933.	transcript	0	0	0	0	0	0	0	0	0
Novel_008321	XLOC_08933.	transcript	0	0	0	0	0	0	0.437508	0	0
Novel_008322	XLOC_08933.	transcript	0	0	0.186154	0	0	0	0	0	0.310344
Novel_008323	XLOC_08933.	transcript	0	0	0	0	0	0	0	0	0
Novel_008324	XLOC_08933.	transcript	0	0	0.169092	0	0	0	0	0	0.338647
Novel_008325	XLOC_08933.	transcript	1.315999	0	0.154944	0.291885	0.608184	1.273072	0.888735	0.461474	0.070412
Novel_008326	XLOC_08933.	transcript	0	0	0	0.111099	0.000491	0	0	0.608942	0
Novel_008327	XLOC_08933.	transcript	0.698316	0	0.189558	0	0	0	0.114811	0	0
Novel_008328	XLOC_08933.	transcript	0	0	0.217657	0	0	0	0.359143	0	0
Novel_008329	XLOC_08933.	transcript	0.002322	0	0.165225	0.136071	0.132762	0.14861	0.092805	0.170514	0.024705
Novel_008330	XLOC_08940.	transcript	0	0.548786	0.027947	0.01658	0	0.015947	0.01977	0.111099	0.07032
Novel_008331	XLOC_08942.	transcript	0	0.122495	0.052572	0.216364	0.174559	0.847037	0.316373	0.305203	0.286671
Novel_008332	XLOC_08942.	transcript	0.18714	0	0	0	0	0	0	0	0.017186
Novel_008333	XLOC_08942.	transcript	0.006445	0	0.705618	0	0	0	0	0	0
Novel_008334	XLOC_08942.	transcript	0.054391	0	0	0.032749	0	0.108781	0	0.161404	0
Novel_008335	XLOC_08942.	transcript	0.489018	0.059862	0.180623	0.576986	0.416567	0	0.704407	0.824606	0.333581
Novel_008336	XLOC_08946.	transcript	0.176062	1.745943	0.256249	0.087149	0.06429	0.183136	0.294742	0.12379	0.140032
Novel_008337	XLOC_08949.	transcript	0	0	0	0	0	0	0.144271	0	0
Novel_008338	XLOC_08950.	transcript	0	0	0	0.097721	0.083482	0.070235	0.189865	0.117869	0.033396
Novel_008339	XLOC_08950.	transcript	0.099851	0.149262	0.248053	0.127062	0.351075	0.154427	0.190958	0.341977	0.289147
Novel_008340	XLOC_08950.	transcript	0.061991	0.191664	0.287642	0	0	0.066747	0.160153	0.017608	0.201856
Novel_008341	XLOC_08950.	transcript	0.222148	0.317325	0.221783	0.440281	0.052387	0.464045	0.310831	0.032228	0.145349
Novel_008342	XLOC_08951.	transcript	0	0	0	0	0	0	0	0.890276	0
Novel_008343	XLOC_08951.	transcript	0.832537	2.502617	0.848418	0.771458	0.820347	0.991498	1.163589	0.584876	1.469004
Novel_008344	XLOC_08952.	transcript	0	0	0	0	0	0	0	0	0
Novel_008345	XLOC_08974.	transcript	0.309004	0.218707	0.286678	0.146363	0.162351	0.147376	0.168131	0.312066	0.181607
Novel_008346	XLOC_08974.	transcript	0	0	0	0.037262	0	0	0.055518	0	0.045138
Novel_008347	XLOC_08977.	transcript	0	0	0	0	0	0.010917	0	0	0.008901
Novel_008348	XLOC_08977.	transcript	0.473049	0.539026	0.536273	1.249614	0.51479	0.765704	0.608458	0.629085	0.854517

Novel_008349	XLOC_08983.	transcript	0.597565	0.589061	0.646508	0.759854	0.846375	0.566628	0.659454	0.721149	0.29405
Novel_008350	XLOC_08987.	transcript	0	0	0	0	0.01713	0	0.008816	0	0
Novel_008351	XLOC_08987.	transcript	0.782368	0	0.013839	0.019781	1.260272	0.029344	0.084927	0.022816	0.01381
Novel_008352	XLOC_08987.	transcript	0	0	0	0	0	0	0	0	0.928364
Novel_008353	XLOC_08987.	transcript	0	0	5.50E-05	0	0	0	0	0	0
Novel_008354	XLOC_08987.	transcript	1.520346	0	0.596871	0	0.329396	0.003116	0	0	0
Novel_008355	XLOC_08987.	transcript	0	0.016313	0.53481	1.105332	0.103606	0.473101	0.279385	0.977111	0.226167
Novel_008356	XLOC_08987.	transcript	0.008346	0.691789	0	0	0.004516	0	2.284995	0.485757	0
Novel_008357	XLOC_08987.	transcript	0.050724	0.80979	0.482869	0.515747	0.258209	1.076906	0.501642	0.602688	0.321139
Novel_008358	XLOC_08987.	transcript	0.069068	0.050655	0.00392	0.014642	0.022336	0.042158	0.025382	0.314893	0
Novel_008359	XLOC_08987.	transcript	0.22111	1.458111	0.243178	0.086625	0.334757	0.085414	0.277478	0.212693	0.880304
Novel_008360	XLOC_08997.	transcript	0.223938	0.061339	0.134309	0.040139	0.121194	0.087963	0.061658	0.060664	0.053743
Novel_008361	XLOC_08997.	transcript	0.186988	0.450246	0.05513	0	0.159405	0.079042	0.469192	0.484015	0.219308
Novel_008362	XLOC_08997.	transcript	0	0.014616	0	0	0	0	0	0	0
Novel_008363	XLOC_08997.	transcript	0.08573	0.149568	0.09651	0.017266	0.055112	0.119605	0.086486	0.037032	0
Novel_008364	XLOC_08997.	transcript	0	0	0	0	0.956583	0.66659	0	0	0
Novel_008365	XLOC_08997.	transcript	0.190888	0.367604	0	0.279672	0	0	0.41648	0.044215	0.326208
Novel_008366	XLOC_09017.	transcript	0.368771	0.334244	0.499669	0.320254	0.580899	1.30903	0.402474	0.084349	0.930836
Novel_008367	XLOC_09017.	transcript	0	0.009158	0.486526	0	0	0.060445	0	0.957965	0
Novel_008368	XLOC_09017.	transcript	0	0	0	0	0	0	0	0	0
Novel_008369	XLOC_09017.	transcript	1.12144	0	0.50505	0.945719	0.911341	0.011784	1.669887	0.847551	1.045227
Novel_008370	XLOC_09017.	transcript	0.498633	0.120927	0.540078	0.168511	0.645566	0.500776	0.725198	0.314628	0.448454
Novel_008371	XLOC_09017.	transcript	0	0	0	0.104938	0	0	0	0	0
Novel_008372	XLOC_09017.	transcript	0	0.002783	0	0	0	0	0	0	0
Novel_008373	XLOC_09018.	transcript	0	0.336833	0.531511	0.147849	0	0	0.50143	0.499188	0.397852
Novel_008374	XLOC_09018.	transcript	0.568743	0.281651	0.077007	0.308476	0.573637	0.555826	0.111075	0.003396	0.102415
Novel_008375	XLOC_09018.	transcript	0	0	0	0	0	0	0	0	0
Novel_008376	XLOC_09022.	transcript	0.155153	0.067206	0.002707	0.125038	0	0.144083	0.035726	0.276706	0.045547
Novel_008377	XLOC_09022.	transcript	0	0	0	0	0	0	0.054182	0	0.16962
Novel_008378	XLOC_09022.	transcript	0	0	0	0	0.085287	0	5.90E-05	0	0.114101
Novel_008379	XLOC_09022.	transcript	0.093983	0.0385	0	0.046102	0	0.057676	0	0.046241	0
Novel_008380	XLOC_09022.	transcript	0.114359	0.042586	0	0.06164	0	0.070896	0	0.049711	0
Novel_008381	XLOC_09022.	transcript	0	0.041189	0	0	0	0.070779	0	0.052176	0
Novel_008382	XLOC_09022.	transcript	0.091865	0.024234	0.223349	0.062098	0.179586	0.203287	0	0.022605	0.31893
Novel_008383	XLOC_09022.	transcript	0.383588	0.076504	0.28795	0.103976	0.058824	0.1825	0.319404	0.521581	0.053332
Novel_008384	XLOC_09022.	transcript	0	0	0	0	0	0	0.065546	0.098913	0
Novel_008385	XLOC_09022.	transcript	0.421454	0.046616	0.130932	0.080703	0.114895	0.122415	0	0.052916	0.072099
Novel_008386	XLOC_09022.	transcript	0.054883	0.410916	0	0.05401	0.085207	0.17313	0.13894	0.141537	0

Novel_008387	XLOC_09022.	transcript	0.141659	0.111322	0.415728	0.489982	0.168006	0.258674	0.274369	0.485478	0.310265
Novel_008388	XLOC_09022.	transcript	0	0	0	0.178245	0	0	0	0.253562	0
Novel_008389	XLOC_09022.	transcript	0.078216	0.053057	0.099871	0	0.091157	0.126408	0.096955	0	0.0831
Novel_008390	XLOC_09022.	transcript	0.237487	0.064804	0.011175	0.300774	0.199276	0.244502	0.340735	0.070691	0.760903
Novel_008391	XLOC_09022.	transcript	0	0.037551	0.521309	0	0.183935	0.028706	0.23058	0.866444	0.530606
Novel_008392	XLOC_09024.	transcript	0.849318	0.217132	0.138636	0.672121	0.449576	0.35559	0.265919	0.990574	0.068906
Novel_008393	XLOC_09025.	transcript	0.475211	0.328881	0.760626	0.604886	0.543987	0.31554	0.46314	0.604444	0.530897
Novel_008394	XLOC_09028.	transcript	0.285192	0.26085	0.113097	0.029953	0.181179	0.043119	0.148075	0.020257	0
Novel_008395	XLOC_09028.	transcript	0.510887	0	0.137837	0.053911	0	0.138803	0	0	0.212361
Novel_008396	XLOC_09028.	transcript	0.14411	0.06591	0.069838	0.046087	0.209323	0.121819	0.362204	0.105668	0.038228
Novel_008397	XLOC_09029.	transcript	0	0.704012	0	0	0	0	0	0	0
Novel_008398	XLOC_09029.	transcript	0	0	0.11859	0	0	0.193203	0	0.172857	0
Novel_008399	XLOC_09029.	transcript	0.603373	0.308229	0.419969	0.564925	0.585192	0.232838	1.052808	0.62904	0.712455
Novel_008400	XLOC_09029.	transcript	0	0	9.30E-05	0	0	0.000218	0	2.111163	0.000485
Novel_008401	XLOC_09029.	transcript	1.003915	0.294851	1.518575	0.659916	0.902473	1.256851	1.47029	0	1.223503
Novel_008402	XLOC_09029.	transcript	0	0	0	0	0	0	0	0	0
Novel_008403	XLOC_09029.	transcript	1.274473	0.001532	0.20853	1.079474	1.2656	0.3248	2.594576	0.502469	0.401531
Novel_008404	XLOC_09030.	transcript	0.192034	0	0.023331	0	0	0	0.017031	0.04139	0
Novel_008405	XLOC_09030.	transcript	0.200973	0.0763	0.895989	0.675607	1.075539	0.618875	2.125045	1.463916	0.523922
Novel_008406	XLOC_09030.	transcript	0.306413	0	0.158872	0.078593	0.183155	0	0.113006	0.169484	0.034008
Novel_008407	XLOC_09030.	transcript	2.00E-06	0	0.106942	0.1521	0.510093	0.182085	0.134199	0	0.058818
Novel_008408	XLOC_09030.	transcript	4.848542	0	0.345071	1.439177	0.370081	1.124304	0.789475	0.004121	0.631808
Novel_008409	XLOC_09030.	transcript	0	0	0	0	0	0	0	0.010051	0
Novel_008410	XLOC_09030.	transcript	0.173259	0.05543	0	0	0.207819	0	0	0	0
Novel_008411	XLOC_09030.	transcript	0	0	0	0	0	0	0	0.103425	0
Novel_008412	XLOC_09030.	transcript	0.80214	0.219098	0.62376	0.530062	0.846034	0.424746	0.946621	0.762516	0.517647
Novel_008413	XLOC_09031.	transcript	0.626523	0.138027	0.338398	0.229519	0.170335	0.134293	0.268756	0.39847	0.099015
Novel_008414	XLOC_09031.	transcript	0	0.001711	0	0.018561	0	0	0	0	0
Novel_008415	XLOC_09031.	transcript	0	0.002196	0	0	0	0	0	0	0
Novel_008416	XLOC_09031.	transcript	0	0	0	0	0	0	0.346674	0	0
Novel_008417	XLOC_09031.	transcript	0.313006	0.652567	0.39138	1.016174	0.517773	1.742128	0.007716	0.418814	0.209576
Novel_008418	XLOC_09031.	transcript	0	0.567641	0	0.564713	0	0	0	0	0
Novel_008419	XLOC_09038.	transcript	0.024288	0.980604	0.014063	0.059793	0.013605	0	0	0.060776	0
Novel_008420	XLOC_09038.	transcript	0.370676	0.053811	0.139928	0.16423	0.260514	0.332282	0.319438	0.311316	0.262144
Novel_008421	XLOC_09038.	transcript	0.014697	0.311049	0.088947	0.011507	0.011908	0.016704	0.025835	0.008325	0.008152
Novel_008422	XLOC_09038.	transcript	0	0	0	0	0	0	0	0	0
Novel_008423	XLOC_09038.	transcript	0	0	0	0	0	0	0	0	0
Novel_008424	XLOC_09038.	transcript	0.147833	0.018908	0.150842	0.076482	0.128218	0.102532	0.33695	0.200161	0.157098

Novel_008425	XLOC_09043.	transcript	0.670728	0.636378	0.6176	0.180059	8.90E-05	0	1.156532	0.876909	0.429894
Novel_008426	XLOC_09043.	transcript	0.124432	0.134406	0.141947	0.275703	0.511012	0.741043	0.27203	0.237553	0.251329
Novel_008427	XLOC_09043.	transcript	0	0	0	0.089274	0.019611	0	0	0	0
Novel_008428	XLOC_09043.	transcript	0	0	0	0.130152	0.346651	0	0	0	0
Novel_008429	XLOC_09043.	transcript	0	0	0	0	0	0	0	0	0
Novel_008430	XLOC_09043.	transcript	0	0	0	0	0	0	0	0	0
Novel_008431	XLOC_09047.	transcript	0	0	0.008617	0	0	0	0.000429	0.000417	0
Novel_008432	XLOC_09047.	transcript	0.314956	0.039574	0.086976	0.27688	0.188206	0.104321	0.23545	0.301191	0.11393
Novel_008433	XLOC_09047.	transcript	5.20E-05	0.016965	0	0.076839	0	0.10159	0	1.30E-05	0
Novel_008434	XLOC_09047.	transcript	0	0.082473	0.05815	0	0	0.013474	0	0	0
Novel_008435	XLOC_09047.	transcript	0.398289	0.107507	0.203205	0.298938	0.397608	0.208389	0	0.404789	0.142218
Novel_008436	XLOC_09047.	transcript	0	0.026646	0.015527	0	0.009631	0.061753	0.487884	0	0.014344
Novel_008437	XLOC_09049.	transcript	0.0127	0	0.001862	0.009037	0	0	0.006707	0	0.177903
Novel_008438	XLOC_09049.	transcript	0.773772	0.596994	0.73312	0.774973	0.775989	0.773367	1.171679	0.827139	0.807214
Novel_008439	XLOC_09049.	transcript	0	0	0	0.014322	0	0	0.227885	0	0.366106
Novel_008440	XLOC_09049.	transcript	0	0	0.170524	0.160487	0.130798	0.131806	0.103058	0.100909	0.044891
Novel_008441	XLOC_09050.	transcript	0.165522	0.116503	0.149416	0.213376	0.199672	0.210159	0.347991	0.289509	0.299125
Novel_008442	XLOC_09051.	transcript	0.006704	0	0	0.003959	0.00218	0.027247	0	0	0
Novel_008443	XLOC_09051.	transcript	0	0	0	0	0	0	0	0	0
Novel_008444	XLOC_09051.	transcript	0	4.00E-06	0.171936	0.003109	0	0.002532	0	1.00E-06	0
Novel_008445	XLOC_09051.	transcript	0.018238	0	1.90E-05	0.001756	0	0.069315	0.118446	0.000496	0
Novel_008446	XLOC_09051.	transcript	0	9.80E-05	0.119981	0	0	0	2.00E-06	0.017921	0.003483
Novel_008447	XLOC_09051.	transcript	0	0	0.066458	0	0	0	0	0	0
Novel_008448	XLOC_09051.	transcript	0.000231	0.059299	0.003016	0.015546	0.012872	0.002635	0	0	0
Novel_008449	XLOC_09051.	transcript	0	0.422951	0	0	0	0.201053	0	0	0
Novel_008450	XLOC_09051.	transcript	0	0	0	0	0.002567	0.073133	0	0.013445	0
Novel_008451	XLOC_09051.	transcript	0	0	0	0	0.003105	0.019975	0	0.003947	0
Novel_008452	XLOC_09051.	transcript	0	0	0	4.30E-05	0	0.00164	0	0	0.00839
Novel_008453	XLOC_09051.	transcript	0	0	0	0	0	0	0	0	0.066822
Novel_008454	XLOC_09051.	transcript	0.000628	0	0	0	0.000456	0.037976	0.003375	0.000263	0.00218
Novel_008455	XLOC_09051.	transcript	0.000602	0.000167	0.000179	0.003758	0.009772	0.048837	0.000873	0.000601	0.150714
Novel_008456	XLOC_09051.	transcript	0.448254	0.210028	0.060988	0.213215	0.269865	0	0.241979	0.233178	0.06985
Novel_008457	XLOC_09051.	transcript	0.025593	0.543504	0.116597	0.141752	0.064719	0	0.44702	0.26622	0
Novel_008458	XLOC_09051.	transcript	0	0.249139	0.192037	0	0.065381	0.021226	0.284164	0.262034	0
Novel_008459	XLOC_09051.	transcript	0.489137	0.557524	0.237849	0.339334	0.331198	0.295404	0.438516	0.278377	0.134246
Novel_008460	XLOC_09056.	transcript	0.072324	0.316954	0.015244	0	0.023904	0	0.084611	0.10965	0.019128
Novel_008461	XLOC_09056.	transcript	0.15222	0.226574	0.111797	0.85405	0.076835	0.057213	0.076944	0.137759	0.053407
Novel_008462	XLOC_09056.	transcript	1.198767	1.999324	1.707476	1.346245	1.358383	1.97428	1.552404	1.724129	1.692175

Novel_008463	XLOC_09056.	transcript	0.038066	0.09114	0.324889	0.128685	0.013936	0.295852	0.006874	0	0.606667
Novel_008464	XLOC_09058.	transcript	1.221143	0.466985	0.151446	0.027521	0.161586	0.240544	0.240819	0.769006	0.66204
Novel_008465	XLOC_09058.	transcript	0.327814	0.328884	1.645658	1.671889	1.33803	1.292444	2.733764	0.936739	2.811365
Novel_008466	XLOC_09058.	transcript	0.229116	0	0	0.022999	0	0	0.01417	0	0.098385
Novel_008467	XLOC_09058.	transcript	0.00943	0	0	0.174494	0.174645	0.007616	0.026049	0	0.031465
Novel_008468	XLOC_09058.	transcript	0	0	0	0	0	0.020231	0	0	0.08703
Novel_008469	XLOC_09058.	transcript	0	0.039182	0.207625	0	0.126355	0.019945	0.130161	0.141001	0.281282
Novel_008470	XLOC_09059.	transcript	0	0	0	0.001432	0	0	0	0.013698	0
Novel_008471	XLOC_09059.	transcript	0.085026	0.394354	0.012397	0.450638	0	0	0.703722	0.453199	0
Novel_008472	XLOC_09059.	transcript	0.428821	0.000962	0.467704	0.133862	0.548586	0.452818	0.166316	0.204701	0.51118
Novel_008473	XLOC_09062.	transcript	0.298589	0.077265	0.258935	0.321386	0.212291	0.223367	0.132737	0.48705	0.168211
Novel_008474	XLOC_09062.	transcript	0	0	0	0	0	0	0.406914	0	0.776159
Novel_008475	XLOC_09062.	transcript	0	0	0.71232	0	0	0	0	0	0.481108
Novel_008476	XLOC_09062.	transcript	0.826854	1.238213	0.598936	0.256236	1.651272	1.791694	0.315632	0.584971	0.374712
Novel_008477	XLOC_09062.	transcript	0.716552	0.078357	0.893747	0.500651	0.233418	0.070005	0.65891	0.66573	0.528548
Novel_008478	XLOC_09062.	transcript	0.675701	0.211791	0.475696	0.422115	0.700792	0.593422	0.77188	0.535752	0.405098
Novel_008479	XLOC_09067.	transcript	0	0.210126	0	0.031395	0	0.203588	0.025817	0	0.135321
Novel_008480	XLOC_09073.	transcript	0.246458	0.469124	0.145474	0.053294	0.132706	0.101807	0.131337	0.182232	0.133669
Novel_008481	XLOC_09075.	transcript	0.02414	0.474574	0	0	0	0.021401	0.072945	0	0
Novel_008482	XLOC_09075.	transcript	0.02857	1.651738	0.016563	0.026218	0.032048	0.056456	0.035034	0	0.185488
Novel_008483	XLOC_09079.	transcript	0	0	0	0	0.012259	0	0.016543	0	0
Novel_008484	XLOC_09079.	transcript	0.0754	0	0.205981	0.140948	0.112494	0.090859	0.391332	0.114538	0.127097
Novel_008485	XLOC_09084.	transcript	0.741563	0.494322	0.786204	0.632633	0.921591	1.287686	1.265982	1.143454	1.000946
Novel_008486	XLOC_09087.	transcript	0	0	0.007352	0	0	0.026586	0	0	0
Novel_008487	XLOC_09087.	transcript	1.915029	0.159532	1.47784	1.039495	1.338347	1.958718	3.708443	1.282313	1.321779
Novel_008488	XLOC_09087.	transcript	0.281491	0.100552	0.158707	0.477216	0.224003	0	0.494863	0.333547	0.408977
Novel_008489	XLOC_09090.	transcript	0.025507	0	0	0.058711	0.167061	0.188524	0	0	0
Novel_008490	XLOC_09095.	transcript	0.113526	0.091598	0.217471	0.151361	0.093839	0.191343	0.118587	0.135209	0.1634
Novel_008491	XLOC_09100.	transcript	0.17025	0.082998	0.043744	0.171111	0.121605	0.235056	0.219744	0.360887	0.250842
Novel_008492	XLOC_09100.	transcript	0.360052	0.282395	0.581117	0.550577	0.040992	0.548231	0.318788	0.579348	0.208037
Novel_008493	XLOC_09100.	transcript	0.291052	0.14241	0	0.033299	0.443589	0.023942	0.332227	0.02189	0.213623
Novel_008494	XLOC_09100.	transcript	0.037303	0.336899	0.04829	0.014784	0.023987	0.050079	0.076173	0.015499	0.020845
Novel_008495	XLOC_09100.	transcript	0.156288	0.230295	0.187031	0.306862	0.117803	0.17147	0.142552	0.247252	0.426468
Novel_008496	XLOC_09102.	transcript	0.243312	0.282304	0.313714	0.362388	0.095959	0.388875	0.256288	0.114199	0.32101
Novel_008497	XLOC_09102.	transcript	0	0.30247	0	0	0	0	0	0.148354	0
Novel_008498	XLOC_09112.	transcript	0.321372	1.201281	0.427263	0.268979	0.203448	0.16544	0.43464	0.137289	0.290468
Novel_008499	XLOC_09113.	transcript	0.434149	0.836035	0.462298	0.269772	0.512878	0.52722	0.690948	0.574913	0.496002
Novel_008500	XLOC_09113.	transcript	0.383442	0.410401	0.314709	0.174606	0.38399	0.355706	0.521122	0.239008	0.129618

Novel_008501	XLOC_09113.	transcript	0	0	0	0	0	0	0	0.151394	0
Novel_008502	XLOC_09114.	transcript	0	0.549651	0.123448	0	0	0.199428	0.145567	0	0.121486
Novel_008503	XLOC_09114.	transcript	0.017862	0.107551	0	0.07331	0.045062	0.364306	0	0.055499	0
Novel_008504	XLOC_09114.	transcript	0	0.132761	0	0	0	0	0.054149	0	0
Novel_008505	XLOC_09114.	transcript	0	0	0	0	0	0	0.023524	0	0
Novel_008506	XLOC_09115.	transcript	0.552451	0.584841	0.484494	0.299264	0.500251	0.706129	0.7098	0.482412	0.463742
Novel_008507	XLOC_09115.	transcript	0.229409	0.21983	0.26254	0.10723	0.269692	0.358557	0.335636	0.212053	0.237005
Novel_008508	XLOC_09117.	transcript	4.873463	7.872789	5.968847	6.261623	6.561841	5.657505	5.663229	7.013134	6.195083
Novel_008509	XLOC_09117.	transcript	0.904801	0.646721	0.662091	0.411186	0.944572	0.636511	1.427991	0.761359	0.611422
Novel_008510	XLOC_09118.	transcript	0.063626	0.009079	0	0	0.069034	0	0	0	0
Novel_008511	XLOC_09118.	transcript	0.066744	0.011249	0.027647	0.017026	0.01379	0.035764	0.141009	0.065465	0.374131
Novel_008512	XLOC_09119.	transcript	0.316598	0.3629	0.173727	0.220259	0.199719	0.134812	0.437464	0.152192	0.208312
Novel_008513	XLOC_09120.	transcript	0.052392	0.010855	0.027695	0.026513	0.040189	0	0.014694	0.007499	0.030994
Novel_008514	XLOC_09124.	transcript	0.043068	0.227462	0.057655	0.062382	0.07497	0.098539	0.061	0.092622	0.061587
Novel_008515	XLOC_09124.	transcript	0.093254	0.182106	0.085119	0.05711	0.103457	0.148634	0.109833	0.237549	0.170719
Novel_008516	XLOC_09124.	transcript	0.507984	0.537542	0.351864	0.151224	0.449602	0.502816	0.268369	0.628877	0.135323
Novel_008517	XLOC_09124.	transcript	0.160252	0.274289	0.054032	0.140726	0.187598	0.164748	0.17302	0.0883	0.060342
Novel_008518	XLOC_09124.	transcript	0.114467	0.475611	0.247481	0.45218	0.385267	0	0.312895	0.31181	0.175635
Novel_008519	XLOC_09124.	transcript	1.852293	0.982494	1.795027	2.292342	1.43309	2.098604	1.821576	2.064519	1.331963
Novel_008520	XLOC_09124.	transcript	0	0	0	0.172221	0	0	0	0	0
Novel_008521	XLOC_09124.	transcript	0	0	0	0	0.014065	0	0.010477	0	0
Novel_008522	XLOC_09124.	transcript	0.241573	0.067059	0.13589	0.125223	0.19113	0.183388	0.221096	0.158445	0.150307
Novel_008523	XLOC_09130.	transcript	0.910031	0.223544	1.199098	1.88375	0.430758	1.474694	0	1.6102	1.429996
Novel_008524	XLOC_09130.	transcript	0.041891	0.150206	0.033946	0.265832	0.852051	0.235753	0.406822	0.154306	0.362546
Novel_008525	XLOC_09130.	transcript	0	0	0	0	0	0	0.794809	0	0
Novel_008526	XLOC_09131.	transcript	0	0	0.05985	0.048903	0	0	0	0.354309	0.041156
Novel_008527	XLOC_09139.	transcript	0.060615	0.121056	0.048062	0.025401	0.064891	0.018284	0.025047	0.017353	0
Novel_008528	XLOC_09144.	transcript	3.790016	1.442186	0.889949	0.597161	0	0.651591	0.637733	0.144324	4.506764
Novel_008529	XLOC_09144.	transcript	0	3.00294	2.904245	0.316293	5.018931	6.130141	2.39446	0.771501	0
Novel_008530	XLOC_09144.	transcript	0	0.672857	4.342628	5.06107	0	0.936108	1.177508	5.360728	1.751204
Novel_008531	XLOC_09144.	transcript	0.655605	0.125376	1.997229	1.801321	0.441298	0.764765	0.891076	0.982159	0.371
Novel_008532	XLOC_09146.	transcript	0.233673	0.345911	0.290685	0.227617	0.13077	0.256685	0.454969	0.242983	0.235443
Novel_008533	XLOC_09148.	transcript	0.035749	0.014964	0.086705	0.14891	0	0.043328	0.150743	0	0.274287
Novel_008534	XLOC_09153.	transcript	0.379306	0	0.109615	0.877518	0.166391	0.263907	0.17536	0.360978	0.115811
Novel_008535	XLOC_09153.	transcript	0	0	0	0.132451	0	0	0	0	0.108123
Novel_008536	XLOC_09153.	transcript	0.015117	0	0	0	0	0.218112	0.168664	0	0
Novel_008537	XLOC_09154.	transcript	0	0	0	0	0	0.147254	0.140951	0	0
Novel_008538	XLOC_09154.	transcript	0.243965	0.293671	0.260687	0.392053	0.306554	0.177737	0.339379	0.401663	0.428902

Novel_008539	XLOC_09155.	transcript	0.444003	0.096757	0	0.087153	0	0.224237	0.204026	0.129085	0
Novel_008540	XLOC_09155.	transcript	0	0.345468	0.00041	0.472187	0.48516	0.40719	0.268324	0.260564	0.423608
Novel_008541	XLOC_09155.	transcript	0.067327	0	0.529348	0	0.033699	0	0.232183	0.298496	0.019586
Novel_008542	XLOC_09156.	transcript	0.340891	0.278762	0.418568	0.265783	0.505707	0.388433	0.730114	0.384747	0.453922
Novel_008543	XLOC_09157.	transcript	0.119588	0.315206	0.036963	0.213552	0.164928	0.12241	0.351901	0.31337	0.027117
Novel_008544	XLOC_09157.	transcript	0	0	0	0.249831	0	0	0	0.194894	0
Novel_008545	XLOC_09157.	transcript	0.33527	0.005841	0.377157	0.369453	0.199366	0.331207	0.404525	0.289668	0.292896
Novel_008546	XLOC_09159.	transcript	0.1011	0	0.209306	0.103731	0.002779	0.010401	0.118122	0.088241	0.395623
Novel_008547	XLOC_09159.	transcript	0.62326	1.07723	0.874008	0.931043	1.016121	1.312887	0.429817	0.813227	0.825393
Novel_008548	XLOC_09159.	transcript	0.005015	0.022839	0.009788	0.014813	0.001736	0.592253	0.013247	0.006626	0.012601
Novel_008549	XLOC_09159.	transcript	1.332749	0	0	0	0	0	0.552684	0.624971	0
Novel_008550	XLOC_09159.	transcript	0	0	0	0	0	0	0	0	0
Novel_008551	XLOC_09159.	transcript	0	0.246512	0	0	0.007155	0	0.007202	0	0.03563
Novel_008552	XLOC_09160.	transcript	2.738688	0.101777	1.142214	0.751534	1.840643	0.893622	4.355069	4.273005	0.685491
Novel_008553	XLOC_09165.	transcript	1.174723	0.272835	1.226163	0.71938	0	0	0.758746	1.689601	0.258028
Novel_008554	XLOC_09165.	transcript	0	0.334365	0.083901	0.135248	1.054682	0.738265	0.026057	0.032664	0
Novel_008555	XLOC_09165.	transcript	0	0.941252	0	1.126173	0.580222	0.5241	1.199	0.065223	0.826486
Novel_008556	XLOC_09165.	transcript	0.054664	0.193189	0.202028	0.023278	0.205967	0.108912	0.023646	0.124043	0.149792
Novel_008557	XLOC_09170.	transcript	0.471109	0.599992	0.036055	0.036989	0.217909	0.225913	0.218788	0.107627	0.36836
Novel_008558	XLOC_09170.	transcript	0	0	0	0.002076	0.084538	0.338319	0	0.261983	0
Novel_008559	XLOC_09170.	transcript	0.004979	0	0.635502	0.090655	0.033195	0	0	0.127315	0
Novel_008560	XLOC_09170.	transcript	0	0.182082	0	0.392736	0	0.042098	0.612935	0.267118	0
Novel_008561	XLOC_09170.	transcript	0	0	0	0.46029	0	0	0	0	0
Novel_008562	XLOC_09170.	transcript	0.375212	0	0	0	0	0	0	0	0
Novel_008563	XLOC_09170.	transcript	0	0	0	0.089558	0	0	0	0	0
Novel_008564	XLOC_09170.	transcript	0	0.160464	0.271227	0	0.339762	0.288787	0.307126	0.238361	0.186919
Novel_008565	XLOC_09171.	transcript	0.080391	0	0.080801	0.060599	0.098492	0.132859	0.104394	0.128363	0.047605
Novel_008566	XLOC_09171.	transcript	0	0	0	0	0.016692	0	0	0	0
Novel_008567	XLOC_09171.	transcript	0.135891	0.072881	0.236185	0.151214	0.192257	0.282767	0.235342	0.255737	0.075819
Novel_008568	XLOC_09171.	transcript	0.147414	0	0	0	0	0	0	0	0
Novel_008569	XLOC_09171.	transcript	0.301314	0	0.22639	0	0	0.090222	0.187713	0.042325	0
Novel_008570	XLOC_09171.	transcript	0.070801	0.048912	0.163205	0.031233	0.174971	0.319329	0.025609	0.193837	0
Novel_008571	XLOC_09171.	transcript	0.159461	0.024702	0.017772	0.08348	0.138767	0.20319	0.150095	0.08359	0.02791
Novel_008572	XLOC_09171.	transcript	0.336865	0.300012	0.456585	0.243537	0.497439	0.384367	0.353259	0.485707	0.329283
Novel_008573	XLOC_09171.	transcript	0	0	0	0	0	0.342235	0	0	0
Novel_008574	XLOC_09174.	transcript	1.563568	1.778756	1.149607	1.147505	1.183349	1.296696	1.254823	1.291069	0.987112
Novel_008575	XLOC_09174.	transcript	0.494939	1.019137	0.486985	0.502628	0.648975	0.658629	0.756057	0.837725	0.560586
Novel_008576	XLOC_09174.	transcript	1.546326	0	0.645086	0.632748	0.005181	0	0.650618	0.473375	0.324165

Novel_008577	XLOC_09174.	transcript	0.365011	0.240872	0.184723	0.232198	0.36445	0.354323	0.420028	0.35746	0.194198
Novel_008578	XLOC_09175.	transcript	0.113583	0.117278	0	0.071396	0.140276	0.205561	0.042474	0.075523	0.051331
Novel_008579	XLOC_09175.	transcript	0.010427	0.003632	0.005512	0	0.001706	0	0.005359	0.04351	0
Novel_008580	XLOC_09175.	transcript	0.143662	0.101816	0.232244	0.082911	0.181129	0.079239	0.203109	0.287289	0.111625
Novel_008581	XLOC_09175.	transcript	0	0	0	0	6.20E-05	0	0.146973	0.007233	0
Novel_008582	XLOC_09175.	transcript	0	0	0	0	0.016624	0.080617	0.071028	0	0
Novel_008583	XLOC_09176.	transcript	0.022149	0	0.016332	0	0	0.009185	0.271299	0	0
Novel_008584	XLOC_09176.	transcript	0.26391	0.292474	0.300746	0.36005	0.371503	0.467435	0.166302	0.29948	0.325613
Novel_008585	XLOC_09176.	transcript	0	0.102089	0.020188	0	0	0.000248	0	0	0.043321
Novel_008586	XLOC_09176.	transcript	0	0.002386	0	0	0	0	0	0	0
Novel_008587	XLOC_09176.	transcript	0.000409	0.387375	0.068768	5.10E-05	0.390125	0.19362	0.149761	0.02447	0.464853
Novel_008588	XLOC_09176.	transcript	0.011294	0	0	0	0	0.012483	0.011783	0.017702	0
Novel_008589	XLOC_09176.	transcript	0.007628	0.143219	0	0.088978	0	0	0.158506	0.029326	0
Novel_008590	XLOC_09176.	transcript	0.14091	0	0	0	0.059857	0.00067	0.002674	0	0
Novel_008591	XLOC_09176.	transcript	1.00E-06	0	0	0.105228	0.203017	0.204485	0.178531	0.090766	0.175813
Novel_008592	XLOC_09176.	transcript	0.345317	0	0	0	0	0	0	0	0
Novel_008593	XLOC_09176.	transcript	0.171915	0	1.135948	0	0	0	0	0	0
Novel_008594	XLOC_09176.	transcript	0.405838	0.419612	0	1.035515	0	0.804121	1.054361	1.094884	0
Novel_008595	XLOC_09176.	transcript	0	0	0	0	0	0	0	0	0
Novel_008596	XLOC_09176.	transcript	0	0.002751	0	0.011756	0.258194	0.006359	0.034062	0.00506	0
Novel_008597	XLOC_09176.	transcript	0.062669	0.022835	0	0.083739	0.147084	0.397392	0.197689	0.108973	0.53913
Novel_008598	XLOC_09178.	transcript	0.699672	0.178638	0.829858	0.589957	0.764624	0.629941	1.161297	0.911447	0.377692
Novel_008599	XLOC_09183.	transcript	0.298915	0.899431	0.237429	0.18783	0.365484	0.291924	0.562013	0.298818	0.971314
Novel_008600	XLOC_09183.	transcript	0	0	0	0	0	0	0	0	0
Novel_008601	XLOC_09184.	transcript	0	0.059484	0.019595	0	0.166421	0.37634	0	0.032005	0.413032
Novel_008602	XLOC_09184.	transcript	0.492544	0.692159	0.51662	0.578214	0.475799	0.015863	0.577489	0.629087	0.03442
Novel_008603	XLOC_09185.	transcript	0.005041	0	0	0	0	0	0.429266	0	0
Novel_008604	XLOC_09185.	transcript	0.034353	0	0	0	0	0	0	0	0
Novel_008605	XLOC_09185.	transcript	0	0	0.135342	0	0	0	0	0	0
Novel_008606	XLOC_09185.	transcript	0.329785	0.469441	0.203884	0.44705	0.481085	0.134512	0	0.328737	0.376716
Novel_008607	XLOC_09185.	transcript	0.168518	0.187683	0.039126	0.155008	0.204612	0.100181	0.109093	0.160494	0.132052
Novel_008608	XLOC_09193.	transcript	0.058989	0	0	0.39951	0.154774	0.220435	0.140451	0.204812	0.322947
Novel_008609	XLOC_09194.	transcript	0.097608	0.370854	0.200974	0.15487	0.079909	0.130188	0.125424	0.12909	0.250121
Novel_008610	XLOC_09194.	transcript	0	0	0	0	0	0	0.171339	0	0
Novel_008611	XLOC_09197.	transcript	0.114508	0.011281	0.043255	0.096397	0.008028	0.032362	0.218427	0.020657	0.531823
Novel_008612	XLOC_09197.	transcript	0	0	0	0.02801	0	0.132933	0.097395	0.068865	0.641121
Novel_008613	XLOC_09197.	transcript	0	0	0	0	0	0	0	0	0
Novel_008614	XLOC_09197.	transcript	0.211679	0.039936	0.145333	0.144134	0.184629	0.198622	0.376436	0.109195	1.267648

Novel_008615	XLOC_09202.	transcript	0.139239	0.186975	0.12398	0.035682	0.065437	0.081456	0.232187	0.049787	0
Novel_008616	XLOC_09202.	transcript	0.025803	0.035557	0.071891	0.206173	0.062469	0.136184	0.05771	0.204886	0.072133
Novel_008617	XLOC_09203.	transcript	0.021503	0.017821	0	0	0	0	0	0	0.050458
Novel_008618	XLOC_09204.	transcript	0.467235	0.324167	0.331549	0.207505	0.170882	0.318221	0.570692	0.522752	0.350187
Novel_008619	XLOC_09204.	transcript	0.024458	0	0.021445	0	0	0	0.018636	0	0
Novel_008620	XLOC_09204.	transcript	0.110099	0	0.282049	0.201821	0.099694	0.158598	0.249959	0.150204	0.224004
Novel_008621	XLOC_09205.	transcript	0.520724	1.132706	0.010534	0.130866	0.338041	0.355187	0.404018	0.57651	0.369231
Novel_008622	XLOC_09205.	transcript	0.21018	0.733636	0.422488	0.359687	0.181502	0.156843	0.185809	0.320712	0.283309
Novel_008623	XLOC_09205.	transcript	0	0	0	0	0	0	0.248669	0	0
Novel_008624	XLOC_09205.	transcript	0	0.028092	0	0.091333	0.02606	0.089981	0	0	0
Novel_008625	XLOC_09206.	transcript	0	0	0	0	0.114349	0	0	0	0
Novel_008626	XLOC_09206.	transcript	0.363035	0.06981	0.212609	0.281596	0.23316	0.202511	0.37811	0.277653	0.263969
Novel_008627	XLOC_09207.	transcript	0	0	0	0	0	0	0	0	0
Novel_008628	XLOC_09208.	transcript	0.401944	0.227464	0.739156	0.506392	0.47508	0.192876	0.368549	0.197966	0.440562
Novel_008629	XLOC_09208.	transcript	0	0.078909	0	0	0.019735	0.094841	0.075824	0.053891	0.008777
Novel_008630	XLOC_09208.	transcript	0	0.070998	0.143642	0.1891	0.199415	0.278139	0.301712	0.516207	0.209409
Novel_008631	XLOC_09208.	transcript	0.198789	0	0	0	0	0	0	0	0
Novel_008632	XLOC_09208.	transcript	1.599673	2.44126	1.556588	0.903939	0.969146	1.052569	1.657623	1.580461	0.753619
Novel_008633	XLOC_09209.	transcript	0.367385	0	0.756997	0.620238	0.765744	1.134881	0.357237	0.544896	0.523528
Novel_008634	XLOC_09209.	transcript	0.888598	0.011502	0	0.707473	0.636566	0	0.777024	0.676336	0
Novel_008635	XLOC_09209.	transcript	1.242427	0.168514	0.655663	0.760157	1.163791	1.35483	1.336441	1.096653	0.864921
Novel_008636	XLOC_09210.	transcript	0.198186	0.015658	0.15372	0.079066	0.21713	0.307075	0.395634	0.189298	0.257489
Novel_008637	XLOC_09210.	transcript	0	0	0.03676	0	0	0	0	0	0
Novel_008638	XLOC_09214.	transcript	0.800703	0.108145	0.388643	0.103863	0.346669	0.221258	0.805422	0.392959	0.019193
Novel_008639	XLOC_09214.	transcript	0.277103	0.279858	0	0	1.08895	0	0	0.118702	0
Novel_008640	XLOC_09214.	transcript	0.637745	0.136378	0.20856	0.283484	0.623899	0.565663	0.330565	0.610662	0.644421
Novel_008641	XLOC_09214.	transcript	0.425641	0	0	0	0.206865	0.299728	0	0.20241	0
Novel_008642	XLOC_09214.	transcript	0.453741	0.085279	0.60501	0.076278	0.168074	0.076642	0.230977	0.207862	0.228883
Novel_008643	XLOC_09215.	transcript	0.175403	0.189882	0.245383	0.261649	0.165951	0.150627	0.339598	0.00882	0.301542
Novel_008644	XLOC_09215.	transcript	0.172105	0	0	0	0	0	0	0.479395	0
Novel_008645	XLOC_09215.	transcript	0.367853	0.002566	0	0	0.040991	0	0	0	0
Novel_008646	XLOC_09215.	transcript	0.483686	0.307723	0.124385	0.13252	0.029908	0.080183	0.139668	0.067304	0.231686
Novel_008647	XLOC_09215.	transcript	0.084369	0.282379	0.698553	1.19965	0.813838	0.77945	0.60676	1.21743	0.767438
Novel_008648	XLOC_09215.	transcript	0.513993	0.024631	0.866005	1.218453	0.065306	1.101214	0.216082	0.602904	0.346103
Novel_008649	XLOC_09215.	transcript	0	0	0	0.088606	0.941693	0	0	0	0
Novel_008650	XLOC_09215.	transcript	0	0	0	0	0	0	0	0	0
Novel_008651	XLOC_09215.	transcript	0.007987	0.00768	0.307447	0.03015	0.419965	0.445123	0	0.049097	0.577654
Novel_008652	XLOC_09215.	transcript	0.459803	1.793709	0.090392	0.246523	0	0.190148	0.750349	0.440984	0

Novel_008653	XLOC_09215.	transcript	0	0	0.055773	0	0	0	0	0	0
Novel_008654	XLOC_09216.	transcript	0.250924	0.394278	0.251647	0.226114	0.33323	0.297678	0.343034	0.275524	0.191132
Novel_008655	XLOC_09216.	transcript	0.131923	0.054259	0.064297	0.049584	0	0.175329	0.095559	0.074677	0.053022
Novel_008656	XLOC_09216.	transcript	0.087002	0	0.083051	0	0.051911	0.070454	0.279912	0.067466	0
Novel_008657	XLOC_09218.	transcript	0	0	0.064034	0	0.091951	0	0	0.075026	0.091694
Novel_008658	XLOC_09218.	transcript	0.404594	0.293841	0.281763	0.328384	0.344047	0.309651	0.188973	0.372826	0.323616
Novel_008659	XLOC_09218.	transcript	0	0	0	0	0	0	0.284912	0	0
Novel_008660	XLOC_09220.	transcript	0.24912	0.225806	0.372589	0.177743	0.250222	0.35435	0.330351	0.254213	0.237308
Novel_008661	XLOC_09220.	transcript	0.046672	0.091729	0.21512	0.392921	0.249588	0.090882	0.227912	0.235916	0.044846
Novel_008662	XLOC_09221.	transcript	0.413859	0.512251	0.361317	0.522306	0.434287	0.539295	0.179196	0.606367	0.409362
Novel_008663	XLOC_09221.	transcript	0.448368	0.20281	0.258176	0.272694	0.247084	0.336525	0.74468	0.487155	0.209426
Novel_008664	XLOC_09224.	transcript	2.295292	0.366545	2.61447	2.947069	2.975037	2.097326	3.809817	3.208642	1.494886
Novel_008665	XLOC_09226.	transcript	0.235737	0.438722	0.137685	0.176479	0.191223	0.154412	0.141547	0.280588	0.039696
Novel_008666	XLOC_09226.	transcript	0	0.011019	0	0	0	0	0	0	0
Novel_008667	XLOC_09227.	transcript	0.00654	0.027083	0.037774	0.062595	0.603508	0.586201	0.098756	0.297654	0.064968
Novel_008668	XLOC_09227.	transcript	0.861928	0.59597	0.854012	0.187679	0.092728	0.122055	0	0.152351	0
Novel_008669	XLOC_09227.	transcript	0	1.054626	0	0.426623	0	0	0.96325	0.269326	0.581967
Novel_008670	XLOC_09227.	transcript	0.584076	0.620168	0.950994	1.08565	0.646223	1.005552	0.69159	0.881251	0.81018
Novel_008671	XLOC_09230.	transcript	0.794553	0.916907	0.864971	0.848409	0.814746	0.931686	0.329508	0.899085	0.877874
Novel_008672	XLOC_09230.	transcript	0	0	0	0	0	0	1.047	0	0
Novel_008673	XLOC_09232.	transcript	0.459161	0.487093	0.473732	0.339594	0.340797	0.44731	0.320458	0.391544	0.347691
Novel_008674	XLOC_09232.	transcript	0	0	0	0	0	0	0	0	0
Novel_008675	XLOC_09234.	transcript	0.270667	0.315919	0.248474	0.32652	0.323207	0.211991	0.408426	0.227604	0.493023
Novel_008676	XLOC_09243.	transcript	0	0	0.376453	0	0	0	0	0	0
Novel_008677	XLOC_09243.	transcript	0.801819	0.094225	0.347525	0.550345	0.909572	0.892966	0.700839	0.881878	0.92863
Novel_008678	XLOC_09245.	transcript	0.727926	0.792941	0.724287	0.452127	0.585913	0.554497	1.135072	0.674563	0.328341
Novel_008679	XLOC_09248.	transcript	0	0.291724	0.008735	0	0	0	0	0.27487	0
Novel_008680	XLOC_09248.	transcript	0.361292	0.002921	0.312249	0.314959	0.340447	0.386012	0.511954	0.123938	0.516392
Novel_008681	XLOC_09249.	transcript	0.294213	0.332845	0.153703	0.165281	0.182251	0.149199	0.263779	0.222156	0.103252
Novel_008682	XLOC_09251.	transcript	0.281723	0.707522	0.17778	0.183037	0.307699	0.257098	0.234489	0.278283	0.325112
Novel_008683	XLOC_09251.	transcript	0.069253	0.179093	0	0.011674	0.044126	0.068896	0	0.011908	0.009133
Novel_008684	XLOC_09251.	transcript	0.182157	0.327302	0.164643	0.24731	0.081893	0	0.056194	0.118919	0.020058
Novel_008685	XLOC_09262.	transcript	0.498728	0	1.036932	0.348809	0.431342	0.395746	0.758293	0.44094	0
Novel_008686	XLOC_09263.	transcript	0.074577	0.213357	0.145315	0.3747	0	0.128204	0.417314	0.164343	0.47341
Novel_008687	XLOC_09264.	transcript	3.113271	0.126202	2.078707	1.225332	2.23063	1.819283	3.825557	2.691305	1.904451
Novel_008688	XLOC_09264.	transcript	0	0	0	0	0.005907	0	0.215687	0	0
Novel_008689	XLOC_09264.	transcript	0.604812	0.960592	1.351416	0.828252	1.308544	0.724504	0.8021	0.665255	2.035393
Novel_008690	XLOC_09264.	transcript	0	0	0	0.057022	0	0.061236	0.03446	0	0.416621

Novel_008691	XLOC_09264.	transcript	0.062361	0.019808	0.240758	0.100355	0.376737	0.086797	0.120734	0.205807	0.056838
Novel_008692	XLOC_09267.	transcript	0.522594	0.727222	0.33703	0.334413	0.607051	0.619486	0.63676	0.426633	0.328953
Novel_008693	XLOC_09280.	transcript	3.230007	1.412994	2.517586	2.476145	1.953589	3.454886	2.201894	3.67002	1.252482
Novel_008694	XLOC_09281.	transcript	0.442051	1.322703	0.411675	0.184016	0.253933	0.402189	0.478657	0.404318	0.108335
Novel_008695	XLOC_09282.	transcript	0	0	0.065137	0.061988	0	0.057328	0	0.031451	0
Novel_008696	XLOC_09282.	transcript	1.523804	0.89454	1.332521	1.770316	1.510156	1.009016	1.723755	1.628556	2.160679
Novel_008697	XLOC_09282.	transcript	0	0.084249	0.085139	0.042598	0.069197	0.163529	0.050599	0.0388	0
Novel_008698	XLOC_09282.	transcript	1.993168	1.806146	1.908509	1.266308	1.485289	1.653648	1.488669	1.986791	1.720721
Novel_008699	XLOC_09283.	transcript	1.208702	0	0.693196	0	0.026446	0.071882	0.283466	0.088177	0.012372
Novel_008700	XLOC_09283.	transcript	0	0.491741	0	0.198085	0	0	0	0	0
Novel_008701	XLOC_09283.	transcript	0	0.352817	0	0.665422	0.755295	0.846961	0.901249	0.881028	0.582375
Novel_008702	XLOC_09283.	transcript	0	0	0	0	0	0	0	0	0
Novel_008703	XLOC_09284.	transcript	0.240364	0.234092	0.287666	0.166871	0.432646	0.458194	0.164836	0.479181	0.382206
Novel_008704	XLOC_09284.	transcript	0.631396	0.471239	0.532535	0.482582	0.290351	0.36268	0.640162	0.451232	0.393334
Novel_008705	XLOC_09284.	transcript	0.358145	0.186351	0.31631	0.114126	0.220412	0.407644	0.24601	0.227596	0.269253
Novel_008706	XLOC_09289.	transcript	0.468332	0.302151	0.355832	0.233973	0.204428	0.132619	0.524785	0.302878	0.168284
Novel_008707	XLOC_09289.	transcript	0	0	0	0	0	0	0	0	0
Novel_008708	XLOC_09289.	transcript	0.108984	0.112068	0	0	0	0	0.005103	0	0
Novel_008709	XLOC_09289.	transcript	0	0	0	0	0	0	0	0.032888	0
Novel_008710	XLOC_09290.	transcript	0.069538	0	0	0	0	0	0	0	0
Novel_008711	XLOC_09290.	transcript	0.174836	0	0	0	0	0	0	0	0
Novel_008712	XLOC_09290.	transcript	0.188615	0.206513	0	0.175984	0.326803	0.647761	0.296013	0.315169	0
Novel_008713	XLOC_09290.	transcript	0.207949	0.571652	0.659504	0.200405	0.299821	0.003319	0.388839	0.230732	0.534717
Novel_008714	XLOC_09290.	transcript	0.149164	0.084733	0.038913	0.054072	0.087013	0.075378	0.099173	0.124412	0.033653
Novel_008715	XLOC_09290.	transcript	0	0	0	0.303666	0	0	0	0.004393	0
Novel_008716	XLOC_09290.	transcript	0.376388	0.382645	0.295617	0.235297	0.274592	0.273158	0.441765	0.301525	0.204582
Novel_008717	XLOC_09291.	transcript	0.429624	0.521681	0.32328	0.333738	0.325105	0.322739	0.489003	0.3537	0.362697
Novel_008718	XLOC_09291.	transcript	0.329028	0.066034	0.361424	0.059478	0.52417	0.546089	0.290648	0.941057	0.280778
Novel_008719	XLOC_09291.	transcript	0	0.523084	0	0.830401	0	0	0.325665	0	0.850536
Novel_008720	XLOC_09291.	transcript	0.127128	0	0.148588	0.013697	0	0	0	0.004264	0
Novel_008721	XLOC_09293.	transcript	0.242992	0.353714	0.426082	0.379698	0.438127	0.443498	0.338919	0.610373	0.096559
Novel_008722	XLOC_09294.	transcript	0	0	0	5.481209	0	0	1.167431	1.933779	0
Novel_008723	XLOC_09294.	transcript	0	0	0	0	0	0.7043	0.046439	0	0
Novel_008724	XLOC_09294.	transcript	0.698271	0	1.050172	0.900176	0.478833	0	0.049698	0.216537	0.154706
Novel_008725	XLOC_09294.	transcript	0	0.005109	0	14.21615	4.311319	0	1.332886	5.733703	0
Novel_008726	XLOC_09296.	transcript	0.013385	0	0	0	0	0.060625	0	0	0.090195
Novel_008727	XLOC_09296.	transcript	0.07602	0	0.047334	0.30594	0.075598	0	0.108903	0.130727	0
Novel_008728	XLOC_09296.	transcript	0	0	0	0	0	0.051204	0	0	0.010676

Novel_008729	XLOC_09297.	transcript	0.152453	0.108664	0.206399	0.353459	0.120514	0.264336	0.22725	0.152362	0.045731
Novel_008730	XLOC_09297.	transcript	0.419647	0.119417	0.555879	0.13161	0.366686	0.636271	0.41262	0.346294	0.396391
Novel_008731	XLOC_09297.	transcript	0	0.061954	0.131493	0.185961	0.098717	0.279227	0.154737	0.144966	0.250576
Novel_008732	XLOC_09298.	transcript	0.052304	0.497391	0.243393	0.138535	0.335343	0.286126	0.574091	0.41683	0.054032
Novel_008733	XLOC_09298.	transcript	0	0	0	0	0	0	0	0	0.205885
Novel_008734	XLOC_09298.	transcript	0.274471	0.089542	0.10791	0.162529	0.073352	0.055242	0.086197	0.095894	0.038639
Novel_008735	XLOC_09300.	transcript	0.479331	0.513846	0.630835	0.616487	0.866699	0.995157	0.431021	0.984237	0.44827
Novel_008736	XLOC_09300.	transcript	0	0	0	0	0	0	0.891729	0	0
Novel_008737	XLOC_09300.	transcript	0	0	0.076995	0.000941	0.000594	0	0.024107	0.000314	0
Novel_008738	XLOC_09300.	transcript	0.000956	0.118983	0	0	0.025048	0.003118	0	0.075989	0.00023
Novel_008739	XLOC_09300.	transcript	0.344197	0.279823	0.329102	0.403785	0.326154	0.37794	0.37312	0.426092	0.460906
Novel_008740	XLOC_09300.	transcript	0.043218	0.24099	0	0	0	0	0	0	0.017694
Novel_008741	XLOC_09300.	transcript	0.050227	0.187366	0.249736	0.263247	0.376628	0.388017	0.244371	0.160914	0.466546
Novel_008742	XLOC_09301.	transcript	0.017622	0.041784	0.02751	0.053249	0.029272	0.316057	0.126948	0.211108	0.034946
Novel_008743	XLOC_09301.	transcript	0.183662	0.004609	0.046373	0	0.136754	0.050614	0.04625	0.021485	0.201416
Novel_008744	XLOC_09301.	transcript	0.083925	0.164903	0.157513	0.127309	0.040457	0.065608	0.116992	0.094743	0
Novel_008745	XLOC_09301.	transcript	0	0	0	0.194397	0	0	0	0	0
Novel_008746	XLOC_09306.	transcript	0.077456	0.520725	0.084213	0.151611	0.246758	0.123092	0.113017	0.233157	0.08381
Novel_008747	XLOC_09306.	transcript	0.21949	0.149432	0.155098	0.101952	0.094691	0.16547	0.183706	0.081871	0.143879
Novel_008748	XLOC_09307.	transcript	0.057025	0	0.027997	0.245602	0.095803	0.067478	0.042114	0.079458	0.027056
Novel_008749	XLOC_09307.	transcript	0.262812	0.139301	0.279588	0.199542	0.349874	0.323771	0.414474	0.514065	0.306702
Novel_008750	XLOC_09307.	transcript	0.013878	0	0.076522	0.380287	0.433153	0.036838	0.283626	0.362028	0.016321
Novel_008751	XLOC_09308.	transcript	0.339033	0.330664	0.198985	0.251116	0.286718	0.218409	0.350154	0.414036	0.312589
Novel_008752	XLOC_09309.	transcript	0.275456	0.236763	0.324053	0.111321	0.3135	0.357575	0.289964	0.362572	0.254201
Novel_008753	XLOC_09311.	transcript	0.260391	0.330862	0.020579	0.030697	0.515572	0.015726	0.012894	0.003493	0.465113
Novel_008754	XLOC_09311.	transcript	0.604101	0	0	0	0	0.846693	0	0	0
Novel_008755	XLOC_09311.	transcript	0	0	0	0.509318	0	0	0	0	0
Novel_008756	XLOC_09311.	transcript	0.021507	0.095244	0.534434	0	0.054853	0	0.561173	0	0
Novel_008757	XLOC_09311.	transcript	0	0	0	0	0	0.023142	0.003563	0.012643	0
Novel_008758	XLOC_09311.	transcript	0.046813	0	0	0	0	0	0.023296	0	0
Novel_008759	XLOC_09311.	transcript	0	0	0	0	0	0	0.494865	0.707373	0
Novel_008760	XLOC_09314.	transcript	0.054162	0.022638	0.424526	0.121453	0.318415	0.131489	0.393273	0.244511	0.032826
Novel_008761	XLOC_09316.	transcript	0.657396	1.434628	0.554206	0.684292	0.60694	0.481209	0.616723	0.584797	0.730687
Novel_008762	XLOC_09316.	transcript	0	0.577797	0	0.987974	0.570558	0.173518	0.742345	0.277136	1.190496
Novel_008763	XLOC_09316.	transcript	0.298075	0.415142	0.17956	0.144441	0.183307	0.173381	0.285049	0.204191	0.218925
Novel_008764	XLOC_09317.	transcript	0.815974	0.502258	0.319442	0.870677	1.033825	1.03306	1.385036	0.681977	0.689699
Novel_008765	XLOC_09317.	transcript	0.154555	0.532073	0.353485	0.106281	0.05035	0.100658	0.062629	0.304224	0.147148
Novel_008766	XLOC_09317.	transcript	0	0.93856	0	0	0	0	0	0.341677	0

Novel_008767	XLOC_09318.	transcript	0.661348	0.744514	0.691672	0.625983	0.63456	0.630587	0.973792	0.601505	1.246367
Novel_008768	XLOC_09318.	transcript	0	0	0	0	0.02472	0	0.218877	0.529488	0
Novel_008769	XLOC_09319.	transcript	0.135847	0	0	0.296026	0.222419	0	0.076075	0.147912	0
Novel_008770	XLOC_09319.	transcript	0.070828	0.227451	0.209493	0.081075	0.076128	0.366249	0.137021	0.080812	0.201686
Novel_008771	XLOC_09322.	transcript	0.598577	0.50734	0.155522	0.536383	0.525601	0.110268	0.069513	0.747419	0.133418
Novel_008772	XLOC_09322.	transcript	0.007358	0.269175	0.333703	0	0.007525	0.311879	0.603675	0.001026	0.228508
Novel_008773	XLOC_09322.	transcript	0.352843	0.828722	0.213751	0.294164	0.27367	0.263317	0.421579	0.356125	0.512819
Novel_008774	XLOC_09323.	transcript	0.165919	0.080991	0.11686	0.06183	0.074658	0.132871	0.018371	0.056208	0.087332
Novel_008775	XLOC_09323.	transcript	0.086558	0	0.12126	0.057677	0.024686	0	0.071452	0.131208	0.122247
Novel_008776	XLOC_09325.	transcript	0.479994	0.036167	0.261533	0.170104	0.313006	0.260676	0.453347	0.320681	0.159767
Novel_008777	XLOC_09327.	transcript	0.860116	0.484383	0.350818	0.684947	0.720999	0.881774	1.241754	0.552019	0.725528
Novel_008778	XLOC_09327.	transcript	0	0	0.255804	0	0	0	0	0.176326	0
Novel_008779	XLOC_09327.	transcript	0.264205	0.078184	0.355776	0.120992	0.260621	0.298217	0.13345	0.292101	0.207064
Novel_008780	XLOC_09328.	transcript	0	0	0	0	0	0	0	0.016206	0
Novel_008781	XLOC_09330.	transcript	0.350531	0.79374	0.365193	0.329895	0.483567	0.340746	0.593259	0.282664	0.379243
Novel_008782	XLOC_09333.	transcript	0.285524	0.186165	0.28686	0.117315	0.106314	0.144775	0.252198	0.084864	0.142717
Novel_008783	XLOC_09333.	transcript	0	0.152406	0.181465	0.187482	0.227086	0.00719	0.109005	0.121623	0.014235
Novel_008784	XLOC_09343.	transcript	0.738223	0	0.514336	0.607259	0.700998	0.02805	0.371274	0.645686	0
Novel_008785	XLOC_09343.	transcript	0.000557	0.312132	0	0	0.002983	0.459646	0.30126	0	0.538671
Novel_008786	XLOC_09343.	transcript	0	0	0	0	0	0	0.197206	0	0
Novel_008787	XLOC_09344.	transcript	0.308982	0.449901	0.127613	0.79376	0.49654	0.770732	0.368344	0.60977	0.680653
Novel_008788	XLOC_09350.	transcript	0	0	0	0	0	0	0	0	0
Novel_008789	XLOC_09350.	transcript	0	0	0	0	0	0	0	0	0
Novel_008790	XLOC_09350.	transcript	0.266514	0.151876	0.384499	0.457333	0.412583	0.415436	0.462601	0.447653	0.616178
Novel_008791	XLOC_09354.	transcript	0.045009	0.024261	0.189829	0	0.025134	0	0.031271	0.025071	0
Novel_008792	XLOC_09354.	transcript	0.015571	0	0	0.145354	0	0.091333	0.19345	0	0
Novel_008793	XLOC_09389.	transcript	0.542082	0.879074	0.680795	0.802455	0.405868	0.733434	0.694149	0.611303	0.58657
Novel_008794	XLOC_09390.	transcript	0.355195	0.018895	0.287623	0.3342	0.305127	0.401046	0.565853	0.390399	0.054037
Novel_008795	XLOC_09390.	transcript	1.041109	0.218886	0.491324	0.619368	0.966747	0.326586	0.379633	0.371807	0.441936
Novel_008796	XLOC_09390.	transcript	0.187303	0.13144	0.269632	0.48254	0.252411	0.490479	0.516259	0.24821	0.284729
Novel_008797	XLOC_09390.	transcript	0.349062	0.035008	0.241181	0.079626	0.072091	0.117542	0.128303	0.086021	0.10649
Novel_008798	XLOC_09391.	transcript	0.323821	0.129617	0.031246	0.049375	0.268324	0	0.143126	0.160189	0.08593
Novel_008799	XLOC_09392.	transcript	0.173918	0.035844	0.117154	0.01669	0.033536	0.1518	0.087994	0.020534	0
Novel_008800	XLOC_09392.	transcript	0.026658	0	0.336071	0.187587	0.020169	0.02345	0.095318	0.020783	0.104219
Novel_008801	XLOC_09392.	transcript	0.139653	0.289768	0	0	0.111929	0	0.348937	0.090631	0
Novel_008802	XLOC_09392.	transcript	0.499667	0.036329	0	0	0.39052	0.450212	0.411344	0.249372	0.408345
Novel_008803	XLOC_09392.	transcript	0.014144	0	0.031842	0.005097	0	0.010608	0.345657	0.315533	0.004627
Novel_008804	XLOC_09392.	transcript	0.207653	0.396149	0.264968	0.414956	0.396938	0.293864	0.143257	0.040467	0.348311

Novel_008805	XLOC_09392.	transcript	0	0	0	0	0	0.13065	0	0	0.190161
Novel_008806	XLOC_09392.	transcript	0	0.349661	0	0	0	0.012598	0.277765	0.328575	0
Novel_008807	XLOC_09392.	transcript	0.165817	0	0.003041	0	0	0	0	0	0.005945
Novel_008808	XLOC_09392.	transcript	0	0.074212	0	0.116593	0	0.018576	0.194863	0	0
Novel_008809	XLOC_09392.	transcript	0	0	0	0.091084	0	0	0.001613	0	0
Novel_008810	XLOC_09392.	transcript	0.006323	0.175499	0.342385	0.101619	0.364642	0.328535	0.006342	0.202073	0.135151
Novel_008811	XLOC_09392.	transcript	0.194117	0.01799	0.046381	0.003913	0.035543	0.016899	0.009749	0.014921	0.188243
Novel_008812	XLOC_09392.	transcript	0.179692	0	0.004884	0.04748	0.001309	0	0	0.044095	0.023881
Novel_008813	XLOC_09392.	transcript	0	0	0	0	0	0	0.057505	0	0
Novel_008814	XLOC_09392.	transcript	0.481096	0.04029	0.881183	1.267314	0.776265	0.311255	0.748015	0.81443	1.033783
Novel_008815	XLOC_09392.	transcript	0.001232	0	0	0.004359	0	0.63533	0	0	0
Novel_008816	XLOC_09393.	transcript	0.692406	0.540525	0.743321	0.585081	0.584281	0.65571	0.876821	0.858521	0.545798
Novel_008817	XLOC_09394.	transcript	0	0.077922	0.18336	0.127325	0.218156	0.261627	0.056783	0.174336	0.151223
Novel_008818	XLOC_09395.	transcript	0.354244	0.533339	0.347781	0.305011	0.309179	0.330235	0.581447	0.321296	0.099072
Novel_008819	XLOC_09395.	transcript	0	0	0	0.017185	0	0	0	0	0
Novel_008820	XLOC_09395.	transcript	0.047445	0.613359	0.476091	0.393962	0.417048	0.361527	0.628031	0.498097	0.611052
Novel_008821	XLOC_09395.	transcript	0.369131	0	0	0	0	0	0	0	0
Novel_008822	XLOC_09397.	transcript	0.390311	0.581086	0.406696	0.294116	0.327897	0.412904	0.388109	0.254812	0.24437
Novel_008823	XLOC_09399.	transcript	0.001734	0.101949	0.037768	0	0	0.020254	0.115125	0	0
Novel_008824	XLOC_09399.	transcript	0	0	0.128823	0.305239	0.077568	0.157272	0.129414	0.092387	0.040691
Novel_008825	XLOC_09399.	transcript	0.29925	0	0.188006	0	0.218768	0	0.114023	0	0
Novel_008826	XLOC_09399.	transcript	0.150688	0.271035	0.02832	0	0.174646	0.029831	0.275454	0.214961	0.356662
Novel_008827	XLOC_09400.	transcript	0.319927	0.452798	0.365632	0.639228	0.476448	0.393766	0.39713	0.457829	0.406471
Novel_008828	XLOC_09400.	transcript	0.298492	1.195753	0.663981	0.603492	1.272911	0.805132	0.530673	0.573319	1.488369
Novel_008829	XLOC_09405.	transcript	6.089449	2.032817	6.428495	7.857386	4.359648	9.583369	4.648032	10.47386	15.02667
Novel_008830	XLOC_09405.	transcript	3.795241	0.02826	0.499646	0.355036	2.00239	0.561579	2.589646	0.270245	0.181056
Novel_008831	XLOC_09405.	transcript	2.724655	0.491896	0.819627	0.735619	3.072583	0.911386	2.512473	4.029015	2.710987
Novel_008832	XLOC_09429.	transcript	0.209168	0.057493	0.221646	0.133678	0.149309	0.205699	0.224172	0.23275	0.175194
Novel_008833	XLOC_09429.	transcript	0	0	0	0.016919	0.031289	0	0.00742	0	0
Novel_008834	XLOC_09454.	transcript	0.317129	0.021345	0.291305	0.321057	0.247283	0.908339	0.178129	0.209785	0.095732
Novel_008835	XLOC_09470.	transcript	0.847233	1.362099	1.075823	1.678519	1.477882	1.681919	1.161399	1.734921	1.810059
Novel_008836	XLOC_09470.	transcript	0.173488	0.628464	0.255454	0.227759	0.205338	0.201976	0.552052	0.311172	0.365409
Novel_008837	XLOC_09470.	transcript	0	0	0	0	0	0	0	0	0
Novel_008838	XLOC_09470.	transcript	0.240458	0.428878	0.238523	0.347413	0.26712	0.408467	0.309191	0.25634	0.579157
Novel_008839	XLOC_09470.	transcript	0	0.003544	0.013413	0.16548	0.221953	0.005886	0	0.096896	0
Novel_008840	XLOC_09470.	transcript	0	0	0.151376	0	0	0.001088	0	0.037711	0
Novel_008841	XLOC_09470.	transcript	0.243115	0.264688	0.027322	0.053705	0.322381	0.196079	0.543799	0.073203	0.467661
Novel_008842	XLOC_09470.	transcript	0	0	0.153383	0.248488	0	0.053587	0	0.120525	0

Novel_008843	XLOC_09472.	transcript	113.2333	160.0396	87.72681	104.0482	90.969	86.77247	112.4076	138.5005	105.1736
Novel_008844	XLOC_09475.	transcript	0.563154	0.326763	0.534565	0.778335	0.487318	0.923271	0.754615	0.565226	0.642091
Novel_008845	XLOC_09476.	transcript	0.001213	0.025142	0.129369	0.226428	0.142571	0.107821	0.088718	0.122023	0
Novel_008846	XLOC_09476.	transcript	0.124033	0	0.084881	0.003616	0.062205	0.028853	0.082356	0.059602	0
Novel_008847	XLOC_09476.	transcript	0.069682	0	0.049113	0.116802	0.047513	0.026436	0.117258	0.132097	0.110109
Novel_008848	XLOC_09478.	transcript	0.634613	1.50108	0.083294	0.220839	0.334374	0.218443	0.215318	0.179375	0.589312
Novel_008849	XLOC_09479.	transcript	0.658032	0.541387	0.350708	0.390449	0.423524	0.489407	1.155985	0.775211	0.444957
Novel_008850	XLOC_09482.	transcript	0.624273	0.996755	0.506728	0.448448	0.729359	0.407686	0.894195	0.664458	0.410821
Novel_008851	XLOC_09482.	transcript	0.320464	0.8223	0.88843	0.93077	0.374802	0.689648	0.685427	0.331177	0.226981
Novel_008852	XLOC_09482.	transcript	0	0	0	0	0.047508	0	0	0	0
Novel_008853	XLOC_09482.	transcript	0.295515	0	0	0	0.100937	0	0.565572	0.144245	0
Novel_008854	XLOC_09482.	transcript	0.260543	0.108031	0.48869	0.548048	0.378748	0.43534	0.286302	0.467584	0.303552
Novel_008855	XLOC_09483.	transcript	0.15565	0.636856	0.140247	0.201825	0	0.271017	0.084846	0.077858	0.528115
Novel_008856	XLOC_09483.	transcript	0	0.567277	0	0	0	0	0	0	0
Novel_008857	XLOC_09483.	transcript	0.133041	4.04146	0.069017	0.059963	0.284299	0.432185	0.695964	0.102476	0.194252
Novel_008858	XLOC_09484.	transcript	0.100528	2.821506	0	0	0.068545	0.161719	0.223379	0.06101	0
Novel_008859	XLOC_09484.	transcript	0	0.329152	0.337608	0	0	0	0	0.000463	0
Novel_008860	XLOC_09484.	transcript	0	1.754081	0.038829	0	0	0	0.128976	0	0
Novel_008861	XLOC_09485.	transcript	0.069461	1.965541	0.05194	0.082015	0	0.422187	0.346981	0.071013	0
Novel_008862	XLOC_09485.	transcript	0.14328	0	0	0	0	0	0	0	0
Novel_008863	XLOC_09485.	transcript	0.091115	4.077536	0.273014	0.079996	0.083991	0.382486	0.504199	0.088374	0.181381
Novel_008864	XLOC_09485.	transcript	0.030856	1.043376	0.033389	0	0	0.058517	0.045245	0	0
Novel_008865	XLOC_09486.	transcript	0.292305	1.471814	0.048884	0.069596	0.014053	0	0.160223	0.080596	0.322733
Novel_008866	XLOC_09486.	transcript	0.436973	3.843201	0.271505	0.220302	0.592897	0.615529	0.57739	0.342756	0.353145
Novel_008867	XLOC_09486.	transcript	0.083629	1.144102	0	0	0	0	0.132764	0	0.309188
Novel_008868	XLOC_09486.	transcript	0.2609	0.249077	0	0.063229	0	0.053097	0.049725	0.088261	0
Novel_008869	XLOC_09486.	transcript	0.179505	4.194507	0	0	0	1.882434	0.175124	0.038978	0.058782
Novel_008870	XLOC_09486.	transcript	0.366423	0.321828	0.117469	0.246068	0.11626	0	0.550763	0	0.346031
Novel_008871	XLOC_09486.	transcript	0.142555	0.981985	0.061049	0.055454	0.03394	0.027665	0.151931	0	0.125353
Novel_008872	XLOC_09486.	transcript	0.536125	1.167371	0.365536	0.258288	0	0.103847	0.2842	0.382329	0.030462
Novel_008873	XLOC_09486.	transcript	0.338092	0.582927	0	0.033154	0.249647	0	0.093792	0.405568	0
Novel_008874	XLOC_09486.	transcript	0.396267	0.394429	0	0.136882	0.310653	0.163362	0.135018	0	0.371207
Novel_008875	XLOC_09487.	transcript	0.035123	0.86753	0	0.106611	0	0	0.555072	0	0.125714
Novel_008876	XLOC_09487.	transcript	0.634601	2.101656	0.726378	0.232631	0.348684	0.08177	0.177956	0	0.328952
Novel_008877	XLOC_09487.	transcript	0.008692	1.672825	0	0	0.352255	0.101483	0.188299	0	0.249206
Novel_008878	XLOC_09488.	transcript	1.006054	6.179187	0.080113	0.423732	0.39083	0.046486	0.891555	0.08765	0.362856
Novel_008879	XLOC_09488.	transcript	0	0.438417	0.024539	0	0	0.032672	0.00868	0	0
Novel_008880	XLOC_09488.	transcript	0	0.437235	0.019014	0.04522	0.091973	0	0	0.018466	0.020036

Novel_008881	XLOC_09488.	transcript	0.070286	1.415387	0.016962	0	0.047698	0	0.080676	0.043787	0.167455
Novel_008882	XLOC_09488.	transcript	0.197215	2.251459	0	0	0	0.041007	0.225365	0	0
Novel_008883	XLOC_09488.	transcript	0.092939	0.154688	0.38338	0.227076	0.159883	0	0.228786	0	0.231084
Novel_008884	XLOC_09489.	transcript	0.280517	0.499432	0.316981	0.167225	0	0.194141	0	1.067453	0.903584
Novel_008885	XLOC_09489.	transcript	0.324673	0.632987	0.038664	0	0.263585	0	0.131529	0.359336	0.439356
Novel_008886	XLOC_09489.	transcript	0.101847	1.195662	0	0.060669	0.053147	0	0.326056	0	0.015396
Novel_008887	XLOC_09489.	transcript	0.364084	1.471281	0	0	0.099113	0	0.062368	0	0.592209
Novel_008888	XLOC_09490.	transcript	0.08526	1.86164	0.060092	0	0.375745	0	0.04251	0.064219	0.217786
Novel_008889	XLOC_09490.	transcript	0	0.391512	0.087627	0.035203	0	0	0	0	0.099557
Novel_008890	XLOC_09490.	transcript	0	2.335766	0.293612	0.116511	0.047713	0.219885	0.536827	0.026531	0
Novel_008891	XLOC_09492.	transcript	0.216271	1.009425	0.044924	0.021201	0.088203	0.100784	0.080304	0	0.023406
Novel_008892	XLOC_09492.	transcript	0.07782	0.741413	0.033441	0	0	0.132539	0.044563	0	0
Novel_008893	XLOC_09492.	transcript	0.317334	2.745613	0	0.067105	0.076978	0	0.119764	0	0.09489
Novel_008894	XLOC_09492.	transcript	0	1.088159	0.143879	0.077071	0	0	0	0.083278	0.132856
Novel_008895	XLOC_09492.	transcript	0	0.920181	0.076605	0	0	0.210101	0	0	0
Novel_008896	XLOC_09493.	transcript	0.386532	3.137875	0.669388	0	0.131135	0.155523	0.279958	0.83721	0.511046
Novel_008897	XLOC_09493.	transcript	0	0	0	0.173897	0	0	0	0	0
Novel_008898	XLOC_09493.	transcript	0	0	0	0	0	0	0	0	0
Novel_008899	XLOC_09493.	transcript	0.456643	4.110497	1.366088	0.159945	0	0.18396	0.715373	1.388831	1.012322
Novel_008900	XLOC_09493.	transcript	0	0.779435	0.08186	0	0	0.138707	0.332537	0	0
Novel_008901	XLOC_09493.	transcript	0	2.227206	0	0.246971	0.36182	0.043662	0.132379	0.226505	0.477308
Novel_008902	XLOC_09493.	transcript	0	0.295389	0	0	0	0	0	0	0
Novel_008903	XLOC_09493.	transcript	0.125729	1.608567	0.072178	0.147581	0.233163	0	0.169611	0.154324	0.239729
Novel_008904	XLOC_09493.	transcript	0	0.974139	0	0.012125	0.044511	0	0.109418	0.026153	0.12428
Novel_008905	XLOC_09494.	transcript	0.253089	1.383895	0	0	0	0	0	0.113997	0.077705
Novel_008906	XLOC_09494.	transcript	0	0.384676	0	0	0.114897	0	0.374993	1.710872	0.09985
Novel_008907	XLOC_09494.	transcript	0.033331	0.636316	0.072906	0	0	0	0.017192	0.046064	0.040864
Novel_008908	XLOC_09495.	transcript	0	1.408441	0	0	0.030112	0.068346	0.140747	0.064505	0
Novel_008909	XLOC_09495.	transcript	0	0.523654	0	0	0	0	0	0	0
Novel_008910	XLOC_09495.	transcript	0.457753	1.283762	0	0.266155	0.134586	0	0.597608	0	0.150188
Novel_008911	XLOC_09495.	transcript	0	1.584278	0.065974	0	0	0	0	0.085388	0.103241
Novel_008912	XLOC_09495.	transcript	0.019044	0.664143	0.052466	0	0	0	0.099389	0	0
Novel_008913	XLOC_09495.	transcript	0	1.083363	0	0	0.10318	0.261953	0	0	0
Novel_008914	XLOC_09495.	transcript	0.496378	2.714765	0.088181	0.023302	0.078406	0.128814	0.088858	0	0.1977
Novel_008915	XLOC_09496.	transcript	0.153877	1.182507	0	0	0.084012	0	0.092149	0.152689	0
Novel_008916	XLOC_09497.	transcript	0.186839	4.325528	0.687544	0.392165	0.299037	0.360539	0.425756	0.405928	1.246974
Novel_008917	XLOC_09497.	transcript	0.139288	3.078767	0.027371	0.047658	0.008889	0.135158	0.048681	0.231252	0
Novel_008918	XLOC_09497.	transcript	0	0	0.043547	0	0.069853	0	0.043166	0	0

Novel_008919	XLOC_09497.	transcript	0.607934	2.796691	0.066569	0.013426	0.044998	0.342482	0.424362	0.078818	0.143803
Novel_008920	XLOC_09497.	transcript	0.099181	2.034745	0.312806	0.033684	0.022773	0.024371	0.628566	0	0.047877
Novel_008921	XLOC_09497.	transcript	0.229774	8.934046	0.711876	0	0	0.828731	0.284275	0	0.185674
Novel_008922	XLOC_09497.	transcript	0.193427	4.424451	0.15525	0.072381	0.309586	0.012977	0.612668	0.101216	0.19564
Novel_008923	XLOC_09497.	transcript	0	0.279924	0	0.211482	0	0	0	0	0
Novel_008924	XLOC_09497.	transcript	0.104435	0.39118	0	0.066737	0.066518	0.045329	0.082981	0.004572	0.10277
Novel_008925	XLOC_09497.	transcript	0.042403	14.28017	0.0431	0.030121	0.045548	0.151707	0.25286	0.605488	1.337313
Novel_008926	XLOC_09497.	transcript	4.00E-05	0	0.302878	0	0.31103	0	0.855368	0	0.069085
Novel_008927	XLOC_09497.	transcript	0.002543	8.229069	0.254672	0.268834	0.183075	0.391916	1.723129	0.10169	0.670818
Novel_008928	XLOC_09497.	transcript	1.354228	11.70158	0	0	0	0	0	0	0
Novel_008929	XLOC_09497.	transcript	0	0	0	0	0	0	0	0	0
Novel_008930	XLOC_09497.	transcript	0	1.328077	0.13286	0	0.460669	0	0	0	0
Novel_008931	XLOC_09497.	transcript	0	0	0	0	0	0	0	0	2.005586
Novel_008932	XLOC_09497.	transcript	0.308333	0	0	0	0	0	0	0.211117	0
Novel_008933	XLOC_09497.	transcript	0	5.682496	0	0.443556	0.68271	0	0.733918	0.141548	0
Novel_008934	XLOC_09497.	transcript	1.163868	0	0	0	0	0.708573	0	0.156048	0
Novel_008935	XLOC_09497.	transcript	0	7.502128	0	0	0.450422	0	0	0	0
Novel_008936	XLOC_09497.	transcript	0.082567	1.420634	0.015662	0	0.045874	0.04788	0.003349	0.022401	0.00764
Novel_008937	XLOC_09497.	transcript	0	0	0.353574	0.540679	0	0	0.910888	0.206285	0.907667
Novel_008938	XLOC_09497.	transcript	0	0	0	0	0	0	0.898919	0	0
Novel_008939	XLOC_09497.	transcript	0	0.026238	0	0	0	0	0	0	0
Novel_008940	XLOC_09497.	transcript	0	0	0.139351	0	0	0	0	0	0
Novel_008941	XLOC_09497.	transcript	0	0	0	0	0	0	0.197781	0	0
Novel_008942	XLOC_09497.	transcript	0	0	0	0	0	0	0.349341	0	0
Novel_008943	XLOC_09497.	transcript	0.000592	0.013874	0.002461	0.419726	2.30E-05	0.120963	0.162908	0.400367	0.032224
Novel_008944	XLOC_09497.	transcript	0	0	0	0	0	0	0.359705	0	0
Novel_008945	XLOC_09497.	transcript	0.740791	7.513206	0.100422	0	0.172624	0.760919	0	0	0
Novel_008946	XLOC_09497.	transcript	0.648512	0	0	0	0	0	0	0	0
Novel_008947	XLOC_09497.	transcript	0	0	0	0	0	0	0	0	0
Novel_008948	XLOC_09497.	transcript	0	2.567587	0.323198	0	0.345776	0	0	0	0
Novel_008949	XLOC_09497.	transcript	0	0	0	0	0	0	0	0	0.679146
Novel_008950	XLOC_09497.	transcript	0	0	0	0	0	0	0	0	0
Novel_008951	XLOC_09497.	transcript	0	0.817873	0	0	0	0	0	0	0
Novel_008952	XLOC_09497.	transcript	0	0	0.610717	0	0	0	0	0.256277	0
Novel_008953	XLOC_09497.	transcript	0	0.624901	0	0	0	0	0	0	0
Novel_008954	XLOC_09497.	transcript	0	0	0	0.314898	0	0.234063	0	0	0
Novel_008955	XLOC_09497.	transcript	0	0	0	0	0	0	0	0	0
Novel_008956	XLOC_09497.	transcript	0	0	0	0	0	0	0	0	0

Novel_008957	XLOC_09497.	transcript	0	0.955539	0	0	0	0	0	0	0
Novel_008958	XLOC_09497.	transcript	0.057979	0.069859	0.176817	0.238469	0.073725	0.103714	0.026062	0.217461	0.302098
Novel_008959	XLOC_09499.	transcript	0.947298	3.494412	0.674161	0	0.398763	0.405324	0.808878	1.814374	0.950711
Novel_008960	XLOC_09499.	transcript	0.478978	0.578002	0	0.429808	0	0	0	0.511199	0
Novel_008961	XLOC_09499.	transcript	5.366079	19.56237	7.2833	1.723235	3.196606	2.086653	1.816988	2.697959	3.727612
Novel_008962	XLOC_09499.	transcript	1.983913	7.823277	1.113008	0.678043	0.682061	0.772183	1.582054	1.319459	2.432784
Novel_008963	XLOC_09503.	transcript	0	0	0	0.055551	0.067869	0.005532	0.092739	0.068586	0.034097
Novel_008964	XLOC_09503.	transcript	0	0.549653	0.015412	0	0.003403	0.009218	0	0	0
Novel_008965	XLOC_09503.	transcript	0.006812	0.578801	0.225388	0	0	0	0	0	0
Novel_008966	XLOC_09503.	transcript	1.195378	0.544671	1.489054	1.484344	1.409572	1.017282	1.608205	1.626332	0.873747
Novel_008967	XLOC_09504.	transcript	0.140303	0.051888	0.25101	0.297946	0.511686	0.67756	0.261652	0	0.49601
Novel_008968	XLOC_09504.	transcript	0	0	0.401725	0.257908	0	0	0.407879	0.566341	0
Novel_008969	XLOC_09504.	transcript	0.45763	0.041367	0.50684	0.201038	0	0.257013	0.328652	0.368568	0.148537
Novel_008970	XLOC_09504.	transcript	1.535263	1.172109	2.913302	4.022342	2.905703	3.376365	1.89192	3.131481	2.055343
Novel_008971	XLOC_09504.	transcript	0	0	0	0	0	0	0	0	0
Novel_008972	XLOC_09504.	transcript	0	0	0	0	0	0	0	0	0
Novel_008973	XLOC_09504.	transcript	0.074103	0	0.255037	0.223703	0.044176	0.129947	0.083706	0.106995	0.206057
Novel_008974	XLOC_09504.	transcript	0.004264	0	0.017047	0	0	0	0	0	0
Novel_008975	XLOC_09504.	transcript	0.012976	0.146039	0	0.016104	0.016828	0.005504	0.001248	0.030356	0.001643
Novel_008976	XLOC_09504.	transcript	0	0	0	0	0	0	0	0	0
Novel_008977	XLOC_09504.	transcript	0.424221	0.354746	0.127236	0.415857	0.398109	0.303057	0.672428	0.615301	0.318921
Novel_008978	XLOC_09504.	transcript	0	0	0	0	0	0	0	0	0
Novel_008979	XLOC_09504.	transcript	0	0	0	0.037895	0.025545	0	0.033435	0.023256	0
Novel_008980	XLOC_09504.	transcript	0.032992	0.069087	0.093868	0.116936	0.125583	0.214046	0.263437	0.202133	0.060128
Novel_008981	XLOC_09504.	transcript	1.326179	0.830943	0.926899	0.740149	0.821669	1.01138	1.398429	0.885532	1.043191
Novel_008982	XLOC_09505.	transcript	0	0.58354	0	0	0	0	0	0	0
Novel_008983	XLOC_09506.	transcript	0.953153	0.130817	0.139292	1.486304	1.5827	0.364354	0.61446	0.436872	0.772304
Novel_008984	XLOC_09506.	transcript	0	0	0	0.228833	0.501988	0	0	0	0.558363
Novel_008985	XLOC_09506.	transcript	0.409014	0.010324	0.195823	0	0	0.233392	0.14304	0.225549	0
Novel_008986	XLOC_09506.	transcript	0	0	0.070052	0	0.093227	0.356318	0	0	0
Novel_008987	XLOC_09506.	transcript	0.371119	0	1.151802	0	0	0.335707	0.631947	0.402923	0
Novel_008988	XLOC_09506.	transcript	0	0	0.169445	0	0	0.350604	0.61408	0.559567	0
Novel_008989	XLOC_09507.	transcript	0.119921	0	0.031696	0.023824	0.084395	0.221855	0.044844	0.117295	0.071061
Novel_008990	XLOC_09508.	transcript	0.1354	0.066909	0.159562	0.065977	0.136705	0.094627	0.253811	0.103899	0.051668
Novel_008991	XLOC_09508.	transcript	0	0	0	0	0	0	0	0	0
Novel_008992	XLOC_09508.	transcript	0.385974	0.56541	0.732263	0.465842	0.830519	0.511154	0.884991	0.265264	0.782257
Novel_008993	XLOC_09510.	transcript	4.541594	7.064146	5.612854	6.257733	5.358994	6.517039	4.869835	6.166841	4.475744
Novel_008994	XLOC_09510.	transcript	0	0	0	0.799114	0	0.020303	0	0	1.135926

Novel_008995	XLOC_09510.	transcript	0	0.055825	0.035605	0	0.068892	0	0	0.095706	0
Novel_008996	XLOC_09510.	transcript	0	0	0.154329	0	0	0	0.027744	0	0
Novel_008997	XLOC_09510.	transcript	0.373395	0.446539	0.540681	0.318882	0.351922	0	0.316598	0.595922	0.426936
Novel_008998	XLOC_09510.	transcript	0	0	0	0.066092	0	0.513648	0.200507	0	0.128675
Novel_008999	XLOC_09512.	transcript	0.049616	0.150978	0	0.091728	0.11194	0.169913	0.144426	0.070948	0
Novel_009000	XLOC_09512.	transcript	0	0	0	0	0	0	0	0	0
Novel_009001	XLOC_09515.	transcript	0	0	0.007484	0	0	0	0.196905	0.214316	0.01086
Novel_009002	XLOC_09515.	transcript	0	0	0	0	0	0	0.219635	0.251773	0
Novel_009003	XLOC_09515.	transcript	0.656332	0	0	0.113404	0.082416	0.087609	0.201227	0.179002	0
Novel_009004	XLOC_09515.	transcript	0.097072	0.071768	0.587359	0.379513	0.432842	0.386022	0.213224	0.175117	0.408691
Novel_009005	XLOC_09516.	transcript	0	0	0.056795	0	0	0.182255	0.108008	0.192867	0
Novel_009006	XLOC_09517.	transcript	0.588813	0.627151	0.631872	0.619163	0.44753	0.758684	1.076394	0.74614	0.29804
Novel_009007	XLOC_09517.	transcript	0	0	0	0	0	0	0	0	0
Novel_009008	XLOC_09517.	transcript	0	0	0	0	0	0	0	0	0.222396
Novel_009009	XLOC_09517.	transcript	0	0	0	0	0.497717	0	0	0	0.275271
Novel_009010	XLOC_09517.	transcript	0.21605	0.00896	0.117365	0.004259	0.008717	0.027766	0.200129	0.007807	0.026341
Novel_009011	XLOC_09519.	transcript	0.7127	0	0	0	0	0.013465	0.828681	0.22513	0.178213
Novel_009012	XLOC_09519.	transcript	0	0.042098	0.525568	0.605365	0.571127	0.453831	0.003183	0.026761	0
Novel_009013	XLOC_09519.	transcript	0	0	0	0	0	0	0	0.24689	0.252565
Novel_009014	XLOC_09519.	transcript	0.01598	0	0	0	0	0.008841	0.028727	0	0
Novel_009015	XLOC_09519.	transcript	0	0	0	0	0	0	0	0	0
Novel_009016	XLOC_09520.	transcript	0.518879	0.016888	0.309114	0.519619	0.514596	0.226885	0.796582	0.354378	0.481548
Novel_009017	XLOC_09530.	transcript	0.404189	0.152895	0.311253	0.110721	0.204646	0.137106	0.414047	0.062857	0.138949
Novel_009018	XLOC_09530.	transcript	0	0.077808	0	0	0	0.062149	0	0.215023	0
Novel_009019	XLOC_09530.	transcript	0	0.106424	0	0	0.03756	0	0.156551	0.056541	0
Novel_009020	XLOC_09530.	transcript	0.47226	0.192136	0.416833	0.354745	0.415945	0.227012	0.414062	0.477726	0.25696
Novel_009021	XLOC_09532.	transcript	0.346434	0.129059	0.240164	0.197231	0.326364	0.194674	0.544778	0.337671	0.323351
Novel_009022	XLOC_09533.	transcript	0.785054	0.636613	0.687069	0.72072	0.716904	0.705399	0.808794	0.837809	0.716804
Novel_009023	XLOC_09534.	transcript	0.603786	1.280227	0.832089	0.706228	0.833859	0.938713	1.065054	0.966738	0.887232
Novel_009024	XLOC_09536.	transcript	0.212949	0.27965	0.288777	0.279068	0.297175	0.103457	0.335419	0.407061	0.133231
Novel_009025	XLOC_09536.	transcript	0	0.100722	0.069175	0.074084	0.063379	0.352055	0.149225	0.109673	0.042951
Novel_009026	XLOC_09536.	transcript	0.500388	0.247438	0.328214	0.205384	0.58998	0.101598	0.46203	0.144985	0.390188
Novel_009027	XLOC_09536.	transcript	0.521102	0.768264	0.559392	0.660397	0.763116	0.728747	1.044173	0.659689	0.65097
Novel_009028	XLOC_09538.	transcript	0	0	0	0	0	0	0	0	0
Novel_009029	XLOC_09538.	transcript	0	0.252155	0.015735	0	0.0071	0	0.009446	0.001714	0
Novel_009030	XLOC_09538.	transcript	0.462918	0	0.946636	0.758808	0.772568	0.724356	0.756916	0.916243	0.70498
Novel_009031	XLOC_09539.	transcript	0.322107	0.400529	0.319037	0.332785	0.389309	0.306914	0.591424	0.427278	0.316728
Novel_009032	XLOC_09540.	transcript	1.328281	0.973912	1.702911	1.890375	1.585508	1.912796	1.493715	1.670099	1.675993

Novel_009033	XLOC_09540.	transcript	0	0	0	0	0	0.004192	0	0	0.01074
Novel_009034	XLOC_09541.	transcript	0	0	0	0	0	0.181784	0.659463	0	0
Novel_009035	XLOC_09552.	transcript	0.315309	0.023757	0.227283	0.192031	0.029122	0	0.119308	0.274603	0.123994
Novel_009036	XLOC_09553.	transcript	1.012992	1.411491	0.360006	0.359632	0.619879	0.44701	0.673961	0.524863	0
Novel_009037	XLOC_09553.	transcript	0.740094	0.617107	0.38327	0.28431	0.35089	0.394488	0.367212	0.320698	0.513828
Novel_009038	XLOC_09557.	transcript	0.156229	0.214909	0.219884	0.038138	0	0.186457	0.084657	0.058242	0.255784
Novel_009039	XLOC_09557.	transcript	0.139515	0.147556	0	0.224059	0.562685	0.054759	0.264003	0.531168	0.335967
Novel_009040	XLOC_09557.	transcript	0.00439	0	0	0.292792	0.03714	0	0.027739	0.009734	0
Novel_009041	XLOC_09559.	transcript	0.05873	0.032826	0.115185	0.062876	0.125655	0.099138	0.122403	0.158326	0.219137
Novel_009042	XLOC_09559.	transcript	0.059754	0.371179	0.040332	0.001789	0	0.011348	0	0	0.012283
Novel_009043	XLOC_09559.	transcript	0	0.15493	0.301641	0.324841	0.153768	0.381987	0.555669	0.117108	0.384685
Novel_009044	XLOC_09559.	transcript	0	0	0	0	0	0	0	0.05598	0
Novel_009045	XLOC_09559.	transcript	0.198359	0.25508	0	0	0.070817	0	0	0.05419	0
Novel_009046	XLOC_09559.	transcript	0.170273	0.292815	0	0.030841	0	0.045793	0.015254	0	0
Novel_009047	XLOC_09561.	transcript	0.533624	0.285734	0.602337	0.880304	0.580683	0.682501	0.790028	0.610527	0.555454
Novel_009048	XLOC_09562.	transcript	0.093813	0	0	0.19339	0.062146	0	0.271012	0	0
Novel_009049	XLOC_09564.	transcript	0.533837	0.265613	0.509419	0.642245	0.454591	0.541843	0.514643	0.533051	0.722874
Novel_009050	XLOC_09564.	transcript	0	0	0	0	0	0	0	0.08771	0
Novel_009051	XLOC_09568.	transcript	0.708554	0.883944	0.553827	0.509801	0.676734	0.469172	0.779654	0.86675	0.660399
Novel_009052	XLOC_09568.	transcript	0	0	0	0	0	0	0	0	0
Novel_009053	XLOC_09570.	transcript	0.782277	0.582773	0	1.085788	0.059286	1.152464	1.412468	0	0
Novel_009054	XLOC_09570.	transcript	0.094008	0.014684	1.285292	0.007583	1.379128	0.02449	0.006238	1.543232	1.285648
Novel_009055	XLOC_09572.	transcript	0	0.806927	0.657278	1.000003	0.543428	0.05078	0.013233	0.830845	0.012777
Novel_009056	XLOC_09572.	transcript	0.44376	0.012334	0.013199	0.004176	0.223819	0.633707	0.765626	0.019401	0.436397
Novel_009057	XLOC_09582.	transcript	0.273504	0.746154	0.676633	0.586527	0.83199	0.884044	0.565024	0.981004	0.720331
Novel_009058	XLOC_09596.	transcript	0	0	0	0	0	0.038036	0	0	0
Novel_009059	XLOC_09601.	transcript	0.229282	0.036873	0.120179	0.093247	0.136106	0.219745	0.011091	0.107918	0
Novel_009060	XLOC_09601.	transcript	0.291911	0.481695	0.15388	0.222823	0.235137	0.175851	0.209233	0.255446	0.136738
Novel_009061	XLOC_09601.	transcript	0.193497	0.207112	0.052863	0.28946	0.026772	0.064402	0	0.051699	0.404378
Novel_009062	XLOC_09601.	transcript	0	0.563948	0.453871	0	0.459542	0	0.398165	0.424976	0
Novel_009063	XLOC_09603.	transcript	1.163314	0.060684	0.850745	0.935628	0.602166	2.094797	0.76236	1.096464	1.627593
Novel_009064	XLOC_09603.	transcript	0	0.548671	1.258674	1.108523	0.641269	1.20823	0.35695	0.969283	2.255775
Novel_009065	XLOC_09603.	transcript	0	0	0	0	0	0	0	0	0
Novel_009066	XLOC_09603.	transcript	0	0	0.434246	0	0	0	0	0	0
Novel_009067	XLOC_09604.	transcript	0.191384	0.101926	0.313161	0.227363	0.200881	0.4052	0.201659	0.177491	0.123541
Novel_009068	XLOC_09604.	transcript	0.002177	0.042419	0.015692	0.045232	0.177623	0.157988	0.115823	0	0
Novel_009069	XLOC_09609.	transcript	1.247212	0.850455	1.082401	0.939876	1.166306	1.076371	1.543628	1.633273	0.733353
Novel_009070	XLOC_09615.	transcript	0.365468	0.564348	0.427239	0.447773	0.37739	0.509037	0.466	0.410813	0.393148

Novel_009071	XLOC_09615.	transcript	0	0.04191	0.054336	0.03282	0	0	0.021148	0	0
Novel_009072	XLOC_09618.	transcript	0.245085	0.74536	0.084361	0.078269	0.015698	0.077442	0.026341	0.06132	0.015136
Novel_009073	XLOC_09618.	transcript	0.970642	0.004701	0.977454	0.49974	0.737167	1.1523	1.117347	0.361435	1.503029
Novel_009074	XLOC_09618.	transcript	0	0	0	0	0	0	0	0	0
Novel_009075	XLOC_09624.	transcript	0.543322	0.405265	0.528156	0.519678	0.58959	0.407079	0.770343	0.553668	0.508937
Novel_009076	XLOC_09624.	transcript	0	0.466043	0.014118	0	0	0.028829	0.08004	0.007949	0.006336
Novel_009077	XLOC_09627.	transcript	0.454297	0.006976	0.020298	0.031408	0.379163	0.511949	0.726379	0.004342	0.014009
Novel_009078	XLOC_09627.	transcript	0	0.234835	0.001382	0.005833	0	0	0	0.147983	0
Novel_009079	XLOC_09627.	transcript	0	0	0	0	0	0	0.002167	0	0.227922
Novel_009080	XLOC_09627.	transcript	0.336603	0	0.235954	1.043624	0	0.01707	0.543001	0.036295	1.01366
Novel_009081	XLOC_09627.	transcript	0	0	0.150507	0.012401	0.436854	0.139381	0.630102	0.448423	0
Novel_009082	XLOC_09627.	transcript	0	0.430681	0.528284	0.149575	0.778094	0.712901	0.065612	1.436311	0
Novel_009083	XLOC_09628.	transcript	0	0	0	0	0.004201	0	0.482557	0	0
Novel_009084	XLOC_09628.	transcript	0.131258	0.047238	0.619441	0.744258	0.436917	0.577254	0.036334	0.495771	0.336358
Novel_009085	XLOC_09628.	transcript	0.068595	0.044074	0.170998	0.250463	0.192835	0.317935	0.220627	0.203298	0.0325
Novel_009086	XLOC_09629.	transcript	0	0	0.150397	0	0	0	0	0	0
Novel_009087	XLOC_09629.	transcript	0.234677	0.217844	0.90051	0.503672	0.145723	1.007539	1.063603	0.276869	1.152674
Novel_009088	XLOC_09629.	transcript	0.60044	0	0.157613	0.676888	1.015555	0.108036	0.504814	1.088279	0.052284
Novel_009089	XLOC_09629.	transcript	0.108134	0	0	0.054403	0.108086	0.05373	0.040571	0.03158	0.2113
Novel_009090	XLOC_09632.	transcript	0.260273	0.134413	0.26568	0.379214	0.343446	0.423663	0.463872	0.45602	0.469582
Novel_009091	XLOC_09643.	transcript	0.080076	0	0.169696	0.347929	0.131685	0.178791	0.462553	0.123633	0.047983
Novel_009092	XLOC_09645.	transcript	0.651431	1.71131	0.719803	0.69994	0.635689	0.616683	1.073043	0.941495	0.848037
Novel_009093	XLOC_09645.	transcript	0.226454	0.136782	0.317937	0	0	0	0.551768	0.063208	0.192214
Novel_009094	XLOC_09645.	transcript	0.22278	0	0	0	0.017243	0	0.253677	0.201384	0.120365
Novel_009095	XLOC_09645.	transcript	0.066145	0.201276	0.171098	0.426605	0.477397	0.058068	0.232762	0.079726	0.132464
Novel_009096	XLOC_09645.	transcript	0.090125	0	0.126988	0.063385	0.003951	0.265234	0.09722	0.498383	0.121284
Novel_009097	XLOC_09645.	transcript	0.351623	0.537016	0.43378	0.45778	0.350649	0.362752	0.210915	0.418892	0.337863
Novel_009098	XLOC_09645.	transcript	0.006412	0.068378	0	0	0	0	0.235268	0.005306	0
Novel_009099	XLOC_09647.	transcript	0.037436	0	0	0	0	0	0	0.247311	0
Novel_009100	XLOC_09648.	transcript	0.469139	0.737335	0.672979	0.587727	0.432512	0.802415	0.592883	0.665936	0.475621
Novel_009101	XLOC_09649.	transcript	0.455903	0.741682	0.599366	0.637532	0.588868	0.605921	0.6402	0.641897	0.389286
Novel_009102	XLOC_09649.	transcript	0	0	0	0.170674	0	0	0	0	0
Novel_009103	XLOC_09652.	transcript	0	0	0	0.33894	0	0.190399	0.012747	0	0.189025
Novel_009104	XLOC_09652.	transcript	0.261089	0.221321	0.461398	1.329548	0.716748	0.699236	1.045489	0.566265	0.371592
Novel_009105	XLOC_09652.	transcript	0.0208	0.035855	0.028497	0.338428	0.042454	0.033277	0.019489	0.092326	0.056108
Novel_009106	XLOC_09657.	transcript	0.201256	0.008476	0.191864	0.070911	0.237617	0.145861	0.375478	0.208388	0.04488
Novel_009107	XLOC_09657.	transcript	0.595281	0.774604	0.608267	0.2466	0.662993	0.918161	0.679226	0.626999	0.362216
Novel_009108	XLOC_09657.	transcript	0	0	0	0.761658	0	0	0	0	0.55361

Novel_009109	XLOC_09657	transcript	0	0.017899	0	0.004199	0	0	0	0	0
Novel_009110	XLOC_09657	transcript	0	0	0.490774	0	0.231273	0	0	0	0
Novel_009111	XLOC_09657	transcript	0.053137	0.005625	0	0	0.004848	0	0.067214	0.773119	0.004004
Novel_009112	XLOC_09657	transcript	0.042701	0.181822	0.170984	0	0.228103	0	0.374866	0.012111	0
Novel_009113	XLOC_09658	transcript	0.60281	0.147589	0.446671	0.614866	0.300334	0.742232	0.562203	0.453163	0.507555
Novel_009114	XLOC_09659	transcript	0.111634	0.035945	0.000844	0.100921	0.002636	0.117209	0.151993	0.099112	0.025071
Novel_009115	XLOC_09659	transcript	1.38571	1.095947	0.898637	0.773246	0.711746	1.239054	1.358286	0.916719	0.61844
Novel_009116	XLOC_09660	transcript	0.121486	0.005689	0.247365	0.220143	0.201743	0.110066	0.216564	0.179517	0.330411
Novel_009117	XLOC_09660	transcript	0.039049	0.556348	0.016854	0.001879	0	0	0.017662	0	0.007588
Novel_009118	XLOC_09660	transcript	0.045494	0	0.045483	0.036435	0	0.335708	0.119125	0.244574	0
Novel_009119	XLOC_09660	transcript	0.318406	0.145629	0.245896	0.292327	0.314455	0.277424	0.336017	0.182352	0.265255
Novel_009120	XLOC_09661	transcript	0.728471	0.553033	0.615866	0.730151	0.685146	0.554938	0.545965	0.657734	0.48456
Novel_009121	XLOC_09661	transcript	0	0.044683	0.036011	0.025585	0.229568	0.029816	0.406801	0.198642	0.031656
Novel_009122	XLOC_09662	transcript	0.180697	0.087265	0.044832	0.054804	0.170731	0.103654	0.242367	0.230178	0.05234
Novel_009123	XLOC_09662	transcript	0.150212	0.276804	0.223701	0.164288	0.15805	0.232117	0.259751	0.092723	0.272416
Novel_009124	XLOC_09663	transcript	0.390085	0.326399	0.530658	0.338997	0.363432	0.47531	0.594557	0.414803	0.446555
Novel_009125	XLOC_09664	transcript	0.199261	0.195741	0.077538	0.142529	0.031607	0.279326	0.245814	0.334253	0.291499
Novel_009126	XLOC_09667	transcript	0.040153	0	0.142169	0	0.041343	0.095916	0.211621	0	0
Novel_009127	XLOC_09672	transcript	0.382751	0.328834	0.325797	0.279059	0.35862	0.151093	0.366878	0.407218	0.276596
Novel_009128	XLOC_09675	transcript	0.107545	0	0	0	0	0	0	0	0
Novel_009129	XLOC_09675	transcript	0.006288	0.036481	0.044147	0.01581	0.019294	0.02276	0.093027	0.03053	0.014904
Novel_009130	XLOC_09676	transcript	0.203593	0.205886	0.150031	0.212536	0.246007	0.229918	0.279077	0.311428	0.252498
Novel_009131	XLOC_09677	transcript	0.294609	1.054373	0.657537	0.592794	1.509179	1.440836	1.186527	0.213171	0.686139
Novel_009132	XLOC_09677	transcript	0.604307	0	0.708712	0.958994	0.187146	0.130373	1.075042	1.442889	0.71896
Novel_009133	XLOC_09677	transcript	1.720193	0.739212	2.670185	1.409028	1.817031	1.748201	3.243053	1.562657	5.280254
Novel_009134	XLOC_09677	transcript	0	0	0	1.459728	2.255235	1.314407	2.030013	0.913545	0
Novel_009135	XLOC_09680	transcript	0.294035	0	0.260832	0.343969	0.189415	0.153882	0.002672	0.191012	0.460346
Novel_009136	XLOC_09680	transcript	0.08906	0	0.047199	0.010646	0.05137	0.065228	0.2469	0.216632	0.312685
Novel_009137	XLOC_09685	transcript	0	0.010816	0.049466	0.005684	0.02275	0.013154	0	0.066714	0.055118
Novel_009138	XLOC_09685	transcript	0.171118	0.333505	0.235489	0.186386	0.151939	0.136624	0.358007	0.202559	0.251853
Novel_009139	XLOC_09685	transcript	0.046123	0.02765	0.023789	0.168547	0.115551	0.020167	0.011938	0.012418	0.079693
Novel_009140	XLOC_09685	transcript	0.01879	0.247864	0.201194	0.256082	0.06572	0.225949	0.293849	0.29551	0.129623
Novel_009141	XLOC_09686	transcript	0	0.283105	0	0	0	0	0.013689	0	0
Novel_009142	XLOC_09687	transcript	0	0	0	0	0	0	0	0	0.033825
Novel_009143	XLOC_09687	transcript	0.686648	0.676514	0.66621	0.43798	0.56944	0.712099	0.623193	0.582385	0.488532
Novel_009144	XLOC_09695	transcript	0.196202	0.176448	0.263066	0.298138	0.155682	0.266153	0.151206	0.164943	0
Novel_009145	XLOC_09695	transcript	0	0	0	1.387508	0	0	0	0	0
Novel_009146	XLOC_09695	transcript	1.09453	0.592136	1.409214	0.427645	0.873664	1.520261	1.164257	1.123057	1.784146

Novel_009147	XLOC_09695	transcript	1.073201	1.190941	1.873326	1.75686	2.09983	2.39854	2.697433	2.713854	1.362346
Novel_009148	XLOC_09696	transcript	0	0	0.034067	0.041846	0.020488	0	0.063548	0.028671	0
Novel_009149	XLOC_09697	transcript	0.087803	0.104428	0.129922	0.067257	0.046705	0.013156	0.075921	0.130899	0.039792
Novel_009150	XLOC_09699	transcript	0	0	0	0	0	0	0	0	0
Novel_009151	XLOC_09699	transcript	0.049874	0	0.241746	0.126774	0.18636	0.209882	0.08315	0.207867	0
Novel_009152	XLOC_09699	transcript	0.099468	0	0.215677	0.1818	0.219761	0.160688	0.22411	0.189763	0.243875
Novel_009153	XLOC_09701	transcript	0	0	0	0	0.292249	0	0	0	0
Novel_009154	XLOC_09701	transcript	0.336536	0.120998	0.566531	0.078824	0.280482	0.064247	0.061264	0.103678	0.608624
Novel_009155	XLOC_09702	transcript	0	0	0	0.066987	0	0.315531	0.254139	0.000204	0.084527
Novel_009156	XLOC_09702	transcript	0.177791	0.002388	0.024709	0.001378	0	0	0.334779	0.097159	0.003701
Novel_009157	XLOC_09702	transcript	0.341758	0	0	0	0	0	0	0.030608	0
Novel_009158	XLOC_09702	transcript	0.018564	0	0	0.002295	0	0	0.003219	0.244654	0.0034
Novel_009159	XLOC_09702	transcript	0	0.040615	0.127994	0.066211	0.089193	0	0.1111	0	0.082973
Novel_009160	XLOC_09702	transcript	0	0.365438	0.16818	0.19589	0.313212	0.399531	0.10821	0	0.23515
Novel_009161	XLOC_09705	transcript	0.335794	0.124311	0.292496	0.28203	0.157276	0.262509	0.459326	0.257684	0.156403
Novel_009162	XLOC_09712	transcript	0	0.023548	0	0.029923	0	0	0	0	0.240196
Novel_009163	XLOC_09712	transcript	0.07749	0.045081	0.007879	0.033725	0.031487	0.097518	0.126796	0.050058	0.040609
Novel_009164	XLOC_09712	transcript	0	0	0.191599	0	0	0.19496	0	0	0
Novel_009165	XLOC_09712	transcript	0.105311	0	0.127392	0.084314	1.00E-06	0.211474	0.190471	0.125606	0.109764
Novel_009166	XLOC_09712	transcript	0	0	0	0	0.120429	0	0	0	0
Novel_009167	XLOC_09712	transcript	0.061682	0	0.040511	0	0	0.079374	0.134938	0	0
Novel_009168	XLOC_09712	transcript	0	0	0	0	0	0	0	0	0
Novel_009169	XLOC_09712	transcript	0	0	0	0	0	0	0	0.075587	0
Novel_009170	XLOC_09712	transcript	0	0	0	0	0.013992	0	0	0.065304	0
Novel_009171	XLOC_09712	transcript	0.130772	0	0.031071	0.012833	0.000111	0	0	0.066265	0.004292
Novel_009172	XLOC_09712	transcript	0	0	0	0	0	0	0	0.054622	0
Novel_009173	XLOC_09712	transcript	0.001194	0	0	0	0	0	0.002066	0.044529	0
Novel_009174	XLOC_09712	transcript	0.004963	0.002261	0.009286	0	0.074672	0.017426	0.09135	0.040931	0
Novel_009175	XLOC_09712	transcript	0	0	0	0.065212	0.026676	0	0	0.036426	0.011044
Novel_009176	XLOC_09712	transcript	0	0	0	0	0	0	0	0	0
Novel_009177	XLOC_09712	transcript	0	0	0	0	0	0	0	0	0
Novel_009178	XLOC_09712	transcript	0	0.017813	0	0	0	0	0	0.003308	0
Novel_009179	XLOC_09716	transcript	0.049299	0.038825	0.060208	0.290194	0.205464	0.55795	0.039635	0.101325	0.152344
Novel_009180	XLOC_09716	transcript	0.884589	0.409231	0.551651	0	0.150911	0	0.850057	0.30838	0.257439
Novel_009181	XLOC_09716	transcript	0.005409	0	0	0.072165	0	0	0	0.084765	0.168953
Novel_009182	XLOC_09716	transcript	0.064411	0.055582	0.318772	0.206957	0.138567	0.099029	0.430493	0.055959	0
Novel_009183	XLOC_09722	transcript	0	0.016915	0	0	0	0.049232	0.032247	0.208726	0
Novel_009184	XLOC_09729	transcript	0.039641	0.138027	0	0	0	0	0.070649	0	0

Novel_009185	XLOC_09735.	transcript	0.431449	0.059281	0.224232	0.34572	0.376935	0.416234	0.645802	0.409777	0.298748
Novel_009186	XLOC_09739.	transcript	0	0	0	0.843116	0.357985	1.508604	0.010745	0.666023	0
Novel_009187	XLOC_09739.	transcript	1.078697	0.048068	1.502429	1.175354	1.026397	0.701589	1.938506	1.483413	1.012123
Novel_009188	XLOC_09743.	transcript	0.304815	0.441234	0.409718	0.394836	0.494298	0.499345	0.387485	0.424176	0.413871
Novel_009189	XLOC_09749.	transcript	0	0.437771	0	0	0	0	0	0	0
Novel_009190	XLOC_09749.	transcript	0	0	0	0	0	0	0	1.441998	0
Novel_009191	XLOC_09749.	transcript	1.421936	0.366649	1.21372	1.019125	1.141035	0.933171	1.799752	0.221628	0.979372
Novel_009192	XLOC_09749.	transcript	0.13895	0.305585	0.205767	0.119746	0.163415	0.117991	0.194328	0.194359	0.188022
Novel_009193	XLOC_09750.	transcript	0.185335	0.621408	0.234689	0.164317	0.160125	0.253989	0.36347	0.158104	0.037319
Novel_009194	XLOC_09750.	transcript	0	0	0	0	0	0.085305	0	0.238264	0
Novel_009195	XLOC_09750.	transcript	0	0	0	0	0	0	0	0	0.076081
Novel_009196	XLOC_09750.	transcript	0.281013	0.443105	0.302236	0.125421	0.279051	0.212278	0.547395	0.131693	0
Novel_009197	XLOC_09750.	transcript	0.163781	0.098139	0.102649	0.045852	0.160834	0.211947	0.291365	0.092834	0.057333
Novel_009198	XLOC_09753.	transcript	0.239359	0	0.043719	0.293084	0.176768	0.122759	0.123084	0.122603	0.0698
Novel_009199	XLOC_09753.	transcript	0.038285	0.092957	0.294005	0.08602	0.177792	0.111231	0.260177	0.138604	0.171512
Novel_009200	XLOC_09755.	transcript	0.004994	0	0	0	0.135866	0	0	0	0
Novel_009201	XLOC_09755.	transcript	0	0.084499	0	0.081009	0	0	0	0	0
Novel_009202	XLOC_09755.	transcript	0	0.032001	0	0	0	0	0	0	0
Novel_009203	XLOC_09755.	transcript	0	0	0	0.769056	1.533335	0	0	0	1.41863
Novel_009204	XLOC_09755.	transcript	1.565623	0.208308	1.363357	0.67014	0.580205	1.533847	0.293482	1.773488	0.335482
Novel_009205	XLOC_09755.	transcript	0.057667	0.008876	0.037107	0.03914	0.020131	0.014047	1.274396	0.0724	0
Novel_009206	XLOC_09760.	transcript	0.629908	0.329426	1.278229	0.993582	0.84011	0.585081	0.758101	0.459766	1.186164
Novel_009207	XLOC_09764.	transcript	0	0	0	0.007713	0	0	0.004149	0.0016	0
Novel_009208	XLOC_09764.	transcript	0.379575	0	0	0.317677	0.02554	0.24074	0.402169	0	0
Novel_009209	XLOC_09764.	transcript	0.005756	0.025532	0.232268	0.353534	0.084578	0.070334	0.140356	0.253365	0.109462
Novel_009210	XLOC_09764.	transcript	0.009475	0	0.002448	0.154198	0.669449	0.012326	0.004233	0.00237	0
Novel_009211	XLOC_09764.	transcript	0.352419	0.162179	0.286584	0.595136	0.149629	0.308684	0.350144	0.522896	0.119499
Novel_009212	XLOC_09773.	transcript	0	37.7789	20.18378	13.20729	15.98736	0	24.19282	23.20654	15.48949
Novel_009213	XLOC_09773.	transcript	15.08045	3.084145	4.614359	6.073222	4.513252	12.02963	0.082734	0.080038	5.161776
Novel_009214	XLOC_09777.	transcript	0.010139	0.086309	0.003199	0.631425	0.130871	0.019137	0.01925	0.317487	0.00714
Novel_009215	XLOC_09779.	transcript	0.005921	0.293892	0.482144	0.410545	0.205982	0.51548	0	0.031386	0.083638
Novel_009216	XLOC_09781.	transcript	0	0	0	0.974511	0.372477	0.087312	0.005625	0.157114	0
Novel_009217	XLOC_09781.	transcript	0.117899	0.008899	0.026261	0.00014	0	0.034886	0.339589	0.43573	0.230273
Novel_009218	XLOC_09781.	transcript	0	0	0.055723	0.274944	0.117929	0.043524	0	0.098604	0
Novel_009219	XLOC_09781.	transcript	0	0	0	0.176258	0	0	0	0	0
Novel_009220	XLOC_09782.	transcript	0.321	0.021405	0.337497	0.285748	0.274058	0	0	0	0.143515
Novel_009221	XLOC_09782.	transcript	0	0	0	0	0	0.417775	0.465962	0.342265	0
Novel_009222	XLOC_09789.	transcript	0	0	0.118865	0.058894	0.126613	0.248923	0.055778	0.096202	0

Novel_009223	XLOC_09803.	transcript	0.13947	0.112541	0.047222	0.033548	0.078378	0.122601	0.119517	0.098185	0.103228
Novel_009224	XLOC_09805.	transcript	0.085663	0.084604	0	0.146156	0.150239	0	0.04094	0.174479	0
Novel_009225	XLOC_09807.	transcript	0	0.013382	0	0	0	0	0	0.002981	0
Novel_009226	XLOC_09807.	transcript	0.796358	0.136579	0.699887	0.860036	0.900574	0.975684	1.046278	0.965749	0.501155
Novel_009227	XLOC_09807.	transcript	0	0	0	0	0	0	0	0	0
Novel_009228	XLOC_09807.	transcript	1.467335	0.705871	1.582333	1.580081	1.179367	2.597156	1.23145	2.215431	1.095252
Novel_009229	XLOC_09809.	transcript	0	0.173783	0.178445	0	0.200218	0	0.050427	0	0
Novel_009230	XLOC_09814.	transcript	0.506997	2.103191	0.249441	0.330308	0.389609	0.342985	0.378566	0.373444	0.429972
Novel_009231	XLOC_09816.	transcript	0.056729	0.044876	0.032834	0.023164	0.047308	0.266716	0.020567	0.098215	0.122268
Novel_009232	XLOC_09816.	transcript	0.279597	0.369336	0.18057	0.255171	0.229848	0.186316	0.450109	0.370192	0.232714
Novel_009233	XLOC_09816.	transcript	0.185503	1.238117	0.374509	0.520743	0.499094	1.167484	0.321843	0.566252	0.147784
Novel_009234	XLOC_09817.	transcript	0.751447	0.542446	0.611501	0.355738	0.471579	0.705118	0.705254	0.522397	0.338713
Novel_009235	XLOC_09820.	transcript	0	0	0	0	0.040423	0.239398	0.519801	0.221694	0.194615
Novel_009236	XLOC_09820.	transcript	0.443159	0.449197	0.268414	0.171477	0.172777	0.06265	0	0.002876	0.077154
Novel_009237	XLOC_09820.	transcript	0	0	0	0	0	0.00243	0.00303	0	0
Novel_009238	XLOC_09820.	transcript	0	0	0	0	0	0	0.008972	0.024972	0
Novel_009239	XLOC_09827.	transcript	0.199128	0.062068	0.341615	0.155873	0.23332	0.354382	0.414053	0.302669	0.180962
Novel_009240	XLOC_09827.	transcript	0.001354	0	0.014329	0	0	0	0.000887	0	0
Novel_009241	XLOC_09827.	transcript	0	0	0	0.23765	0	0	0.13235	0.074935	0
Novel_009242	XLOC_09829.	transcript	0.147696	0.007429	0.138765	0.075072	0.078985	0.097043	0.185463	0.078837	0.07397
Novel_009243	XLOC_09830.	transcript	0.881141	0	0.654756	0	0.153286	1.020205	0	0.625045	0
Novel_009244	XLOC_09830.	transcript	0.712309	0	0.741947	0	0.159882	0.89692	0	0.597684	0
Novel_009245	XLOC_09830.	transcript	0.442265	0	0.644612	0.000772	0.135745	0.911296	1.90E-05	0.551163	0.000772
Novel_009246	XLOC_09830.	transcript	1.201492	0	2.210891	5.224923	4.20207	2.402658	8.520733	5.27951	3.832296
Novel_009247	XLOC_09830.	transcript	1.388962	0	0	0	0	0	0	0	0
Novel_009248	XLOC_09830.	transcript	0.776303	0	2.708717	2.163518	4.80392	4.513836	6.185255	8.849903	1.456734
Novel_009249	XLOC_09830.	transcript	1.512842	0.44749	0	0	0	0	0	0	0
Novel_009250	XLOC_09830.	transcript	0.933592	0	0	0	0	0	0	0	0
Novel_009251	XLOC_09830.	transcript	1.193748	0	2.058154	5.131022	4.06435	2.381092	0.052793	0.285177	3.337248
Novel_009252	XLOC_09830.	transcript	0.748338	0	2.847249	2.34441	4.605125	3.809472	0.084736	0.283065	1.766796
Novel_009253	XLOC_09830.	transcript	0.518892	0	0	0	0	0	0	0	0
Novel_009254	XLOC_09830.	transcript	0.69887	0	1.107043	1.089821	1.113054	0.871583	0.519449	1.277625	1.769744
Novel_009255	XLOC_09830.	transcript	0.49097	0	0.727702	0.88599	1.003827	0.857572	0.501688	1.132292	1.894657
Novel_009256	XLOC_09830.	transcript	0.736857	1.522795	0.817474	0.631414	0.809131	0.713376	0.950139	0.652573	0.830232
Novel_009257	XLOC_09831.	transcript	0.175257	0.362344	0.068362	0.189513	0.256919	0.216731	0.230747	0.238587	0.086369
Novel_009258	XLOC_09831.	transcript	0.372671	0.501363	0.317475	0.222352	0.2237	0.345935	0.327695	0.235703	0.417072
Novel_009259	XLOC_09833.	transcript	0.288926	1.203261	0.478899	0.201738	0.453181	0.322338	0.443708	0.476564	0.220181
Novel_009260	XLOC_09833.	transcript	0.458894	0.430179	0.423546	0.483163	0.566997	0.42732	0.5457	0.444451	0.244884

Novel_009261	XLOC_09834.	transcript	0.046717	0.043116	0.014171	0.212003	0.012859	0.043996	0.075803	0.013966	0.028104
Novel_009262	XLOC_09834.	transcript	0.430389	0.626829	0.554915	0.274591	0.491422	0.494188	0.803603	0.47218	0.463143
Novel_009263	XLOC_09834.	transcript	0.457932	0.178357	0.754059	0.393862	0.575184	0.604429	1.073576	0.580426	0.564266
Novel_009264	XLOC_09836.	transcript	0.462851	1.623009	0.613975	0.441988	0.490959	0.47671	0.912606	0.393694	0.785455
Novel_009265	XLOC_09849.	transcript	0.625211	0	0.402561	0.526877	0.483156	0.453233	0.75464	0	0.51369
Novel_009266	XLOC_09849.	transcript	0.02391	0.399237	0.0093	0.018407	0.005721	0	0.026836	0.669553	0
Novel_009267	XLOC_09849.	transcript	0	0.882012	0	0	0	0	0	0	0
Novel_009268	XLOC_09849.	transcript	0.17931	0.04168	0.319071	0.197933	0.089913	0.231321	0.227956	0.207317	0.215268
Novel_009269	XLOC_09850.	transcript	0.110745	0.039154	0.16688	0.106152	0.165833	0.14679	0.295897	0.268113	0.082565
Novel_009270	XLOC_09850.	transcript	0.265107	0.352009	0.246665	0.300693	0.220094	0.358909	0.355845	0.079238	0.282281
Novel_009271	XLOC_09850.	transcript	0.011259	0	0.018362	0	0	0	0.033243	0	0
Novel_009272	XLOC_09850.	transcript	0	0	0	0	0	0	0.008725	0.148722	0
Novel_009273	XLOC_09850.	transcript	0.016583	0	0	0	0	0.050476	0	0.035733	0
Novel_009274	XLOC_09851.	transcript	0	0	0	0	0	0	0	0	0
Novel_009275	XLOC_09851.	transcript	0.023049	0.424241	0.021822	0	0.007037	0.016603	0.020566	0.007879	0.024653
Novel_009276	XLOC_09851.	transcript	0	0	0	0	0	0	0	0	0
Novel_009277	XLOC_09851.	transcript	0.061043	0.486097	0.017608	0	0.028365	0.006661	0.035478	0.031928	0.102127
Novel_009278	XLOC_09862.	transcript	0	0	0	0	0	0	0.348568	0.002455	0
Novel_009279	XLOC_09862.	transcript	0	0	0	0.007221	0	0	0	0	0.000532
Novel_009280	XLOC_09862.	transcript	0.025443	0.018031	0	0.00615	0.000741	0	0.208529	0	0
Novel_009281	XLOC_09862.	transcript	0.361928	0.41872	0.352921	0.316387	0.310148	0.323694	0.05853	0.319304	0.298921
Novel_009282	XLOC_09863.	transcript	0.437909	0.322334	0.317401	0.151095	0.273846	0.203884	0.416486	0.246316	0.117807
Novel_009283	XLOC_09863.	transcript	0	0	0	0	0	0	0	0.01387	0
Novel_009284	XLOC_09863.	transcript	0.10937	0.232955	0	0	0.370938	0.049871	0.233469	0.080067	0
Novel_009285	XLOC_09863.	transcript	0.491052	0.712456	0.360358	0.359688	0.434304	0.415402	0.486462	0.610066	0.364645
Novel_009286	XLOC_09864.	transcript	0	0	0.213808	0.078348	0.33115	0.196478	0.037403	0.136548	0.063508
Novel_009287	XLOC_09864.	transcript	0.530886	0.67607	0.287973	0.613779	0.477307	0.433799	0.760291	0.691156	0.419278
Novel_009288	XLOC_09864.	transcript	0.42504	0.317408	0.380011	0.363807	0.353305	0.312919	0.399648	0.400601	0.543608
Novel_009289	XLOC_09864.	transcript	0	0	0	0	0.186649	0	0	0	0
Novel_009290	XLOC_09865.	transcript	0.199258	0	0	0	0	0.481352	0	0.367126	0
Novel_009291	XLOC_09865.	transcript	0	0.450626	0	0	0	0	0.452608	0	0
Novel_009292	XLOC_09865.	transcript	0.100416	0.137751	0.209951	0.149958	0.389253	0.119426	0.139188	0.129299	0.178183
Novel_009293	XLOC_09865.	transcript	0	0	0	0.212326	0	0	0	0	0
Novel_009294	XLOC_09865.	transcript	0	0	0	0.036285	0	0.335133	0.053965	0	0.21918
Novel_009295	XLOC_09865.	transcript	0.091304	0.396347	0.228237	0.356964	0.402277	0.051519	0.129539	0.222096	0.186947
Novel_009296	XLOC_09866.	transcript	0.005243	0	0.002005	0.003724	0.002999	0.006178	0.000497	0	0
Novel_009297	XLOC_09866.	transcript	0.242202	0.411734	0.274344	0.208234	0.16002	0.273154	0.422137	0.254535	0.163645
Novel_009298	XLOC_09866.	transcript	0.014328	0.01053	0.030256	0.020405	0.037703	0.097682	0.007644	0.021967	0.021127

Novel_009299	XLOC_09866.	transcript	0.288382	0.332158	0.285648	0.272465	0.418995	0	0.450939	0.51612	0.119608
Novel_009300	XLOC_09871.	transcript	0.314868	0.634923	0.26964	0.154754	0.235869	0.134603	0.325392	0.245904	0.264955
Novel_009301	XLOC_09872.	transcript	0.126388	0.78585	0.036295	0.03081	0.020773	0.073513	0.260421	0.046048	0.160966
Novel_009302	XLOC_09873.	transcript	0.273718	0	0.025928	0.023402	0	0.070526	0.061761	0.051675	0.179635
Novel_009303	XLOC_09873.	transcript	0.052528	0.360088	0.295047	0.117612	0.271176	0.123391	0.313757	0.244681	0.264627
Novel_009304	XLOC_09878.	transcript	0	0.109087	0.135167	0.000279	0.002158	0	0.010649	0	0.035
Novel_009305	XLOC_09878.	transcript	0	0.365122	0.004445	0.008662	0.032948	0.013685	0.017132	0	0.018245
Novel_009306	XLOC_09878.	transcript	0.75768	1.638788	0.369518	0.395211	0.483143	0.544558	0.682961	0.430937	0.394283
Novel_009307	XLOC_09882.	transcript	0.035243	0	0.064477	0.176155	0.031773	0.147208	0.023211	0.030251	0.007438
Novel_009308	XLOC_09882.	transcript	0.0161	0	0.401069	0.003851	0.015462	0.508138	0.025102	0.002391	0.003018
Novel_009309	XLOC_09882.	transcript	0	0	0	0.122905	0	0	0	0	0
Novel_009310	XLOC_09882.	transcript	0.005054	0	0.233193	0.132895	0.000632	0.173445	0.024791	0.002009	0.007742
Novel_009311	XLOC_09882.	transcript	0	0	0	0	0	0	0	0	0
Novel_009312	XLOC_09882.	transcript	0	0	0.151482	0.130157	0	0.101895	0	0	0
Novel_009313	XLOC_09882.	transcript	1.251955	0.06785	0	0	0.832609	0	1.184535	0.792673	0.489927
Novel_009314	XLOC_09882.	transcript	0	0.001649	0	0	0	0	0	0.023903	0.001831
Novel_009315	XLOC_09882.	transcript	0	0	0	0.001617	0	0	0	0	0
Novel_009316	XLOC_09882.	transcript	0.259018	0	0.053868	0.077823	0.473294	0.087984	0.211915	0.112334	0.177271
Novel_009317	XLOC_09884.	transcript	0	0	0.118167	0.140516	0	0	0	0.047889	0
Novel_009318	XLOC_09893.	transcript	0	0	0.009926	0	0	0	0	0	0
Novel_009319	XLOC_09893.	transcript	0.035483	0.063619	0	0	0	0.543459	0.419608	0.006624	0
Novel_009320	XLOC_09893.	transcript	0	0	0.01191	0	0	0	0	0	0
Novel_009321	XLOC_09893.	transcript	0	0	0	0	0	0	0	0	0
Novel_009322	XLOC_09893.	transcript	0.392418	0.024652	0.373109	0.273984	0.478797	0.079869	0	0.448217	0.253592
Novel_009323	XLOC_09918.	transcript	0.223734	0	3.515397	2.781205	0	2.500657	4.330426	3.759105	0.385018
Novel_009324	XLOC_09919.	transcript	0.163914	0.117232	0.398534	0.287061	0.469395	0.52861	0.295147	0.44289	0.365028
Novel_009325	XLOC_09920.	transcript	0.308406	0	0.205883	0.1598	0	0.096223	0.237219	0.186661	0
Novel_009326	XLOC_09922.	transcript	0.537677	1.728168	0.886914	0.889909	0.975581	1.028159	1.108661	1.194952	0.665806
Novel_009327	XLOC_09922.	transcript	0.657356	0.169357	0.204664	0.057508	0.297855	0.151345	0.467651	0.236139	0.187342
Novel_009328	XLOC_09926.	transcript	0	0.238822	0	0	0	0.402646	0.143033	0	0
Novel_009329	XLOC_09926.	transcript	0.38023	0.731733	0.080397	0.063097	0	0	0	0.032655	0
Novel_009330	XLOC_09927.	transcript	0.88974	0	0	0	0.273211	0	0	0	0
Novel_009331	XLOC_09927.	transcript	0	0	0	0.034982	0	0.048681	0	0.157407	0.048012
Novel_009332	XLOC_09927.	transcript	0.115257	0.358319	0	0	0	0	0.222506	0	0
Novel_009333	XLOC_09927.	transcript	0.040422	0.679163	0.034473	0	0.019018	0	0.090695	0	0
Novel_009334	XLOC_09927.	transcript	0	0.218216	0	0	0	0	0	0	0
Novel_009335	XLOC_09927.	transcript	0.054939	0.77438	0.060713	0.048131	0	0.066979	0.062707	0	0.340295
Novel_009336	XLOC_09927.	transcript	0.035217	0.52679	0.036794	0.014857	0.046535	0.031867	0.152417	0	0.126048

Novel_009337	XLOC_09928.	transcript	0.144755	1.387824	0	0.249129	0.135725	0	0.260191	0	0.371414
Novel_009338	XLOC_09928.	transcript	0.37578	1.878974	0.207255	0.101397	0.108589	0.327781	0.12859	0	0.134781
Novel_009339	XLOC_09928.	transcript	0.576134	1.939498	0.105212	0.105189	0.014338	0	0.284272	0.112368	0.426181
Novel_009340	XLOC_09928.	transcript	0.330323	1.325783	0.060162	0	0.114169	0	0.088736	0	0.113798
Novel_009341	XLOC_09928.	transcript	0.064824	1.156383	0.116511	0.066242	0.098125	0.156674	0.315824	0	0.270044
Novel_009342	XLOC_09928.	transcript	0.3601	2.401855	0.065468	0.14799	0.060653	0.011925	0.130409	0.039122	0.418246
Novel_009343	XLOC_09928.	transcript	0	0.61917	0	0.099603	0.151879	0.103865	0	0.018689	0.100933
Novel_009344	XLOC_09931.	transcript	1.484161	0.799646	0.792067	0.812817	0.835825	1.907581	0.673891	0.707128	0.701771
Novel_009345	XLOC_09933.	transcript	0.025938	0.04212	0	0.028151	0.004989	0.039329	0.012261	0.025728	0.000593
Novel_009346	XLOC_09933.	transcript	0	0.127607	0	0	0	0	0.024136	0	0.014559
Novel_009347	XLOC_09933.	transcript	0	0.052774	0.019096	0.009111	0.011647	0.014338	0	0	0
Novel_009348	XLOC_09933.	transcript	0.042081	0.798047	0.065254	0.029789	0.090397	0.076238	0.059851	0.036561	0.001322
Novel_009349	XLOC_09937.	transcript	0.344678	0.17115	0.572174	0.184615	0.365016	0.256498	0.635865	0.334179	0.565741
Novel_009350	XLOC_09937.	transcript	0.26631	0.248567	0	0	0.101862	0	0.026365	0	0
Novel_009351	XLOC_09939.	transcript	0.96367	0.06412	1.081093	1.070858	0.893956	0.718711	0.741003	0.779384	0
Novel_009352	XLOC_09939.	transcript	0.374567	0.583142	0.288765	0.740527	0.322992	0.672872	0.60936	0.373375	0.954133
Novel_009353	XLOC_09939.	transcript	0.242652	0.096691	0.167155	0.06384	0.080676	0	0.103657	0.061225	0.067507
Novel_009354	XLOC_09939.	transcript	0.155789	0.216778	0.023496	0.076492	0.133009	0.225598	0.048949	0.040569	0.155151
Novel_009355	XLOC_09939.	transcript	0.365243	0.73323	0.547268	0.642157	0.710201	0.947255	0.976643	0.915768	0.955302
Novel_009356	XLOC_09939.	transcript	0	0.254073	0.009409	0.007401	0.219033	1.368113	0.437381	0.079919	0
Novel_009357	XLOC_09939.	transcript	5.917742	1.130769	6.69558	5.021537	5.779634	3.642379	10.55409	5.635919	25.60148
Novel_009358	XLOC_09939.	transcript	0.663154	0.849443	0.637557	0.444635	0.095912	0.690194	0.08051	0.316396	0.421641
Novel_009359	XLOC_09939.	transcript	0.501067	0.760904	0.557523	0.637378	0.986275	0.518185	1.349552	0.639155	0.74771
Novel_009360	XLOC_09945.	transcript	0	0.029753	0.003764	0	0.000294	0.085665	0	0	0
Novel_009361	XLOC_09945.	transcript	0.174595	0.815991	0.252606	0.033619	0.052509	0.040596	0.392612	0.049215	0.025169
Novel_009362	XLOC_09945.	transcript	0	0	4.00E-05	0	0	0	0	0.000363	0
Novel_009363	XLOC_09945.	transcript	0	0	2.20E-05	0.170207	0.128424	0.070302	0.090547	0.148825	0.374855
Novel_009364	XLOC_09945.	transcript	0.003158	0	0.001738	0	0.001324	0	0	0	0
Novel_009365	XLOC_09945.	transcript	0.042474	0.003026	0.001711	0.00618	0.000186	0.011258	0.147672	0	0.00282
Novel_009366	XLOC_09945.	transcript	0	0	0	0	0.057967	0.063097	0	0	0
Novel_009367	XLOC_09945.	transcript	0.003958	6.10E-05	0.007406	0.000846	0.001482	0	0.00423	0	0
Novel_009368	XLOC_09945.	transcript	0.000489	0.003762	1.00E-06	0.00585	0.002204	0.001249	0.002229	0	0.000228
Novel_009369	XLOC_09945.	transcript	0	0	0	0	0.064982	0	0	0.080386	0
Novel_009370	XLOC_09945.	transcript	0	0	0.001064	0	0.004068	0	0	0.074922	0
Novel_009371	XLOC_09945.	transcript	0	0	0	0	0	0	0	0.073641	0
Novel_009372	XLOC_09945.	transcript	0.002833	0.009425	0	0.035124	0	0	0	0.10059	0.001336
Novel_009373	XLOC_09945.	transcript	0	0.008891	0.10376	0	0	0.002638	0.005565	0	0.035364
Novel_009374	XLOC_09945.	transcript	0.77584	1.788246	0.937671	0.948329	0.993192	0.822562	1.608549	0.99631	1.172279

Novel_009375	XLOC_09946	transcript	0.23792	0.028178	0.039314	0.037994	0.277263	0.164039	0.367828	0.153846	0.084419
Novel_009376	XLOC_09947	transcript	0.379717	0.0286	0.786709	0.044138	0.747541	0.00904	0.370144	0.236275	0.585733
Novel_009377	XLOC_09947	transcript	0	0	0	0.002577	0	0	0.276098	0	0
Novel_009378	XLOC_09947	transcript	0.021699	0	0.126097	0.16717	0.127144	0.083292	0.077944	0.173386	0.04961
Novel_009379	XLOC_09954	transcript	0.369719	0.030891	0.801504	0.637954	0.834353	0.62886	0.756073	0.900557	0.252278
Novel_009380	XLOC_09956	transcript	0.087524	0.059183	0	0.092259	0.170055	0.191724	0.132907	0.440029	0.132475
Novel_009381	XLOC_09957	transcript	1.705766	0.731509	1.913724	1.087486	1.4256	1.603948	1.660303	1.936828	1.366866
Novel_009382	XLOC_09959	transcript	0	0.005558	0	0.453811	0	0	0.25406	0.017279	1.773299
Novel_009383	XLOC_09959	transcript	0.734743	0.591352	1.007008	0.984912	0.818977	0.979325	0.85592	0.721697	1.288174
Novel_009384	XLOC_09960	transcript	0.084012	0.153292	0.184795	0.082606	0.236585	0.203586	0.156689	0.021738	0.210013
Novel_009385	XLOC_09960	transcript	0.008702	0.00324	0.014958	0.006209	0	0	0.033652	0.042544	0.050299
Novel_009386	XLOC_09960	transcript	0.784747	0.473647	0.877652	0.198648	1.178574	0.822545	0.797257	0.645111	0.789471
Novel_009387	XLOC_09960	transcript	3.528756	7.556197	6.413521	6.672509	6.235591	5.9792	7.364269	8.225038	9.207829
Novel_009388	XLOC_09960	transcript	0	0	0	0.063043	0	0	0	0	0
Novel_009389	XLOC_09960	transcript	0	0.001572	0	0	0	0	0	0	0
Novel_009390	XLOC_09960	transcript	0.477873	0.302469	0.306672	0.067116	0.274801	0.38094	0.519905	0.308636	0.227947
Novel_009391	XLOC_09960	transcript	0	0	0	0.017748	0	0	0	0	0
Novel_009392	XLOC_09960	transcript	0.007894	0	0.032408	0	0.128512	0	0.00445	0.007736	0
Novel_009393	XLOC_09960	transcript	0.073349	0	0	0.217715	0	0	0	0.162776	0
Novel_009394	XLOC_09962	transcript	1.63845	0.097895	2.14144	2.223363	3.891669	1.907767	2.489486	2.274024	2.699067
Novel_009395	XLOC_09963	transcript	0	0.1135	0.039124	0	0	0	0.749738	0	0
Novel_009396	XLOC_09963	transcript	0	0	0	0	0	0	0	0	0
Novel_009397	XLOC_09963	transcript	0.982941	0.062345	0.408112	0.454793	0.655008	0.55188	0.038517	0.840656	0.098651
Novel_009398	XLOC_09966	transcript	0	0	0	0	0	0	0	0.170207	0.047841
Novel_009399	XLOC_09966	transcript	0.014721	0.325626	0.001168	0.012413	0	0.520876	0.235301	0.089269	0.229602
Novel_009400	XLOC_09966	transcript	0	0.29496	0.393233	0	0.286405	0	0.19905	0.317449	0
Novel_009401	XLOC_09966	transcript	0	0.4712	0	0.716561	1.50E-05	0	0	0.127573	0
Novel_009402	XLOC_09966	transcript	0	0	0	0	0	0	0	0	0
Novel_009403	XLOC_09966	transcript	0.533423	0.180698	0.429857	0.002861	0.34591	0.168086	0.531244	0.211055	0.573443
Novel_009404	XLOC_09971	transcript	0.079955	0.050315	0.161085	0.146861	0.062796	0.3052	0.134694	0.08744	0.036685
Novel_009405	XLOC_09979	transcript	0.131137	0.085408	0.222624	0.09802	0.140298	0.113052	0.10599	0.150425	0.126206
Novel_009406	XLOC_09983	transcript	0.165515	0.051469	0.083085	0.069473	0.192027	0.110886	0.17574	0.121176	0.061005
Novel_009407	XLOC_09993	transcript	173.6958	192.6537	265.794	240.9877	249.381	259.7056	194.4285	240.0044	245.2543
Novel_009408	XLOC_09993	transcript	0.57991	0.437512	0.238152	0.284265	0.467569	0.380616	0.482453	0.345911	0.282281
Novel_009409	XLOC_09994	transcript	0.011044	0.02157	0	0	0.005369	0.023836	0.017	0.001071	0
Novel_009410	XLOC_09994	transcript	0.306312	0.512716	0.214622	0.01526	0.203345	0.168691	0.262269	0.149828	0.213453
Novel_009411	XLOC_09994	transcript	0	0	0	0	0	0	0	0.013812	0
Novel_009412	XLOC_09994	transcript	0	0.105559	0.055638	0.199602	0.053441	0.089525	0.063025	0.167308	0.164476

Novel_009413	XLOC_0999[illegible]	transcript	0.410777	0.449669	0.33999	0.343933	0.493561	0.450055	0.351517	0.492215	0.388302
Novel_009414	XLOC_0999[illegible]	transcript	0	0	0	0	0	0	0.000555	0.249708	0
Novel_009415	XLOC_0999[illegible]	transcript	0	0.438617	0.01857	0.035916	0	0.015091	0.422213	0.21383	0
Novel_009416	XLOC_0999[illegible]	transcript	0.390075	0	0.267064	0.392752	0.335197	0.267864	0.00459	0	0.219932
Novel_009417	XLOC_0999[illegible]	transcript	0.720941	0.169811	0.784403	1.137373	0.608437	0.380957	1.091954	1.062894	0.914572
Novel_009418	XLOC_0999[illegible]	transcript	0.298715	0.02635	0.054997	0.435822	0.329797	0.654296	0.53552	0.28721	0.092268
Novel_009419	XLOC_0999[illegible]	transcript	0	0	0	0.1423	0.027087	0.175998	0.097251	0.008597	0
Novel_009420	XLOC_0999[illegible]	transcript	0	0	0	0	0.219704	0.302706	0.199935	0.17779	0.57249
Novel_009421	XLOC_0999[illegible]	transcript	0.464201	0.044001	0.012493	0.025519	0.365338	0.174874	0.352221	0.266203	0.171046
Novel_009422	XLOC_0999[illegible]	transcript	0	0	0.293746	0.237678	0	0	0	0	0
Novel_009423	XLOC_0999[illegible]	transcript	0	0.028369	0	0.109943	0.003561	0.939006	0	0.136098	0.004663
Novel_009424	XLOC_0999[illegible]	transcript	0	0	0	0	0	0	0.001812	0	0.001112
Novel_009425	XLOC_0999[illegible]	transcript	1.120809	1.192936	0.863428	0.771758	0.675527	0.035726	1.076713	0.709892	0.698175
Novel_009426	XLOC_0999[illegible]	transcript	0.204864	0	0.249431	0.222485	0.429545	0.236563	0.765659	0.17929	0.577415
Novel_009427	XLOC_0999[illegible]	transcript	0	0	0	0	0	0	0	0.150781	0
Novel_009428	XLOC_0999[illegible]	transcript	0.619711	0	0	0	0	0	0	0.17884	0
Novel_009429	XLOC_0999[illegible]	transcript	0	0.771857	0.003163	0.742099	0.839641	0.975819	0.499664	0.662981	0.76449
Novel_009430	XLOC_0999[illegible]	transcript	0.20733	0	0.233457	0.25272	0	0.117603	0.225282	0.189345	0.29098
Novel_009431	XLOC_1000[illegible]	transcript	0.023279	0	0.019833	0	0	0	0	0	0.527874
Novel_009432	XLOC_1000[illegible]	transcript	0.187627	0.133964	0.245262	0.284191	0.452605	0.254104	0.378195	0.387699	0
Novel_009433	XLOC_1000[illegible]	transcript	0	0	0	0	0	0	0	0	0.145933
Novel_009434	XLOC_1000[illegible]	transcript	0.364851	0.563806	0.543257	0.795585	0.854862	0.512934	0.710495	0.889679	0.486512
Novel_009435	XLOC_1000[illegible]	transcript	0.000204	0.153924	0	0.432512	0	1.000204	0.001624	0.002302	0.300979
Novel_009436	XLOC_1000[illegible]	transcript	1.578131	2.327655	2.368338	1.708732	2.000906	1.851597	2.075157	2.594306	2.365707
Novel_009437	XLOC_1001[illegible]	transcript	0.092021	0.103514	0.075897	0.30644	0.073262	0.271372	0.117084	0.111734	0.084891
Novel_009438	XLOC_1001[illegible]	transcript	0	0	0	0.143305	0	0	0.438264	0	0
Novel_009439	XLOC_1001[illegible]	transcript	0	0.19634	0.056614	0.084393	0.408513	0	0.052444	0	0
Novel_009440	XLOC_1001[illegible]	transcript	0	0	0	0	0.004399	0	0	0	0
Novel_009441	XLOC_1001[illegible]	transcript	0	0.138517	0.02903	0	0	0.049696	0.15156	0.118175	0
Novel_009442	XLOC_1001[illegible]	transcript	0.23861	0.007034	0.171789	0.161323	0.138255	0.103297	0.231325	0.270156	0.12674
Novel_009443	XLOC_1001[illegible]	transcript	0.292199	0.917796	0.244543	0.198063	0.240554	0.26481	0.359451	0.180283	0.18311
Novel_009444	XLOC_1002[illegible]	transcript	0.133835	0.031709	0.012878	0.285059	0.156187	0.088491	0.035627	0.023085	0.039483
Novel_009445	XLOC_1002[illegible]	transcript	0.120116	0.477164	0.300894	0.407178	0.3135	0.340319	0.235222	0.39922	0.255245
Novel_009446	XLOC_1002[illegible]	transcript	0.599337	0.292477	0.464238	0.751262	0.674149	0.731241	1.154748	0.63312	0.500584
Novel_009447	XLOC_10031.	transcript	1.89104	0	3.145363	14.61251	4.723538	1.393107	3.588482	7.823136	1.781232
Novel_009448	XLOC_10031.	transcript	0.751312	0.014234	0.257362	0.016588	0.292096	0.13653	0.603344	0.187353	0.011026
Novel_009449	XLOC_10031.	transcript	0	0	0.071076	0.459889	0	0	0	0	0
Novel_009450	XLOC_10031.	transcript	0	0	0.190166	0	0.085281	0	0.20296	8.10E-05	0.074544

Novel_009451	XLOC_10031.	transcript	0	0	0	0	0.259225	0	0.398083	0.3783	0.416456
Novel_009452	XLOC_10031.	transcript	0.008205	0	0.008375	0.004933	0.0518	0.002081	0.008296	0.003919	0
Novel_009453	XLOC_10033.	transcript	0	0	0	0	0.076352	0	0	0	0
Novel_009454	XLOC_10033.	transcript	0	0	0	0.36005	0.449929	0.144818	0.068283	0.327699	0
Novel_009455	XLOC_10033.	transcript	0.478993	0.415014	0.486524	0.127731	0.005432	0.353629	0.529744	0.269196	0.471476
Novel_009456	XLOC_10033.	transcript	0	0	0.118903	0.081744	0	0.107847	0.089795	0	0
Novel_009457	XLOC_10033.	transcript	0.146804	0	0.017245	0.027342	0.049259	0.098401	0.097594	0	0.096656
Novel_009458	XLOC_10034.	transcript	0.350669	0.40605	0.29357	0.359094	0.332681	0.328715	0.470026	0.490988	0.409073
Novel_009459	XLOC_10035.	transcript	0.226702	0.15779	0.146722	0.270358	0.00533	0.287813	0.280705	0.124255	0.31937
Novel_009460	XLOC_10035.	transcript	0.311485	0.150983	0.190476	0	0.392016	0.1649	0.29187	0.301201	0.017439
Novel_009461	XLOC_10035.	transcript	0.451545	0.639914	0.635222	0.638971	0.659202	1.012362	0.34297	0.137889	0.642589
Novel_009462	XLOC_10036.	transcript	0	0	0	0	0	0	0	0	0
Novel_009463	XLOC_10036.	transcript	0.056382	0.646349	0.088953	0.03273	0.079953	0.123569	0.118312	0.08413	0.013427
Novel_009464	XLOC_10044.	transcript	0.56511	0.404331	0.582368	0.434456	0.396941	0.685423	0.685644	0.565684	0.558973
Novel_009465	XLOC_10048.	transcript	1.664639	0.941095	5.609536	5.895004	3.927996	6.328976	4.133044	3.816163	5.553845
Novel_009466	XLOC_10052.	transcript	0.009519	0.057209	0.011195	0.26036	0.014116	0	0.001619	0.068644	0
Novel_009467	XLOC_10052.	transcript	0.462719	0	0.37656	0.126681	0.410696	0.3111	0.519351	0	0.319511
Novel_009468	XLOC_10053.	transcript	0.343177	0.522351	0.380211	0.364434	0.304664	0.169416	0.801909	0.257735	0.501212
Novel_009469	XLOC_10053.	transcript	1.022136	1.515728	0.51633	0.573947	0.523361	0	0.535291	1.000444	0.506692
Novel_009470	XLOC_10053.	transcript	0	0	0	0	0	0	0	0.18241	0
Novel_009471	XLOC_10053.	transcript	0	0.007831	0.087535	0.107029	0.393223	0.1381	0.279978	0.12987	0.159079
Novel_009472	XLOC_10053.	transcript	0.834877	0	0.540061	0.196364	0.183611	0.415809	0.452751	0.181871	0.337112
Novel_009473	XLOC_10053.	transcript	0	0	0.256319	0	0	0.135588	0.307304	0.224602	0
Novel_009474	XLOC_10053.	transcript	0.960712	0	0.179859	0.012106	0	0.35003	0.270536	0.265135	0
Novel_009475	XLOC_10053.	transcript	0.551738	0	0.619722	0.049321	0.209926	0.129704	0.173593	0.084233	0.263883
Novel_009476	XLOC_10053.	transcript	0.574654	0.033809	0.327292	0.153853	0.256819	0.172266	0.469527	0.262525	0.411633
Novel_009477	XLOC_10069.	transcript	0.395948	0.476755	0.449184	0.213471	0.306294	0.369105	0.460068	0.517193	0.44243
Novel_009478	XLOC_10069.	transcript	0	0.055044	0	0.152373	0	0.133369	0.31941	0.006244	0
Novel_009479	XLOC_10069.	transcript	0	0	0	0	0	0	0	0	0
Novel_009480	XLOC_10070.	transcript	0.215532	0.132706	0.199938	0.329543	0.244949	0.444078	0.408459	0.272946	1.447314
Novel_009481	XLOC_10070.	transcript	0.334889	0.199074	0.238217	0.262471	0.30696	0.31412	0.491712	0.230681	0.226903
Novel_009482	XLOC_10070.	transcript	0.418241	0.289633	0.31909	0.333418	0.2026	0.382181	0.78468	0.648712	0.589836
Novel_009483	XLOC_10072.	transcript	0	0.257414	0	0	0	0	0	0.097419	0
Novel_009484	XLOC_10072.	transcript	0.019622	0	0.010372	0.03289	0.018087	0	0.02935	0.013286	0.103229
Novel_009485	XLOC_10072.	transcript	0	0.030915	0	0	0	0	0	0.008308	0.333431
Novel_009486	XLOC_10074.	transcript	0.367869	0.734752	0.182806	0.498781	0.314548	0.166327	0.320411	0.209679	0.214324
Novel_009487	XLOC_10076.	transcript	0.268185	0.313569	0.336868	0.152786	0.224047	0.242079	0.194199	0.181238	0.664498
Novel_009488	XLOC_10077.	transcript	0	0.468219	0	0	0.01714	0	0	0	0

Novel_009489	XLOC_10077.	transcript	0	0.555877	0	0	0	0	0.043946	0	0
Novel_009490	XLOC_10079.	transcript	2.014421	1.704437	2.164545	2.449017	2.615294	2.378842	2.694155	3.44226	1.531047
Novel_009491	XLOC_10079.	transcript	0	0	0	0.534386	0	0	0	0	0
Novel_009492	XLOC_10083.	transcript	1.719069	2.36037	1.424912	2.003888	2.030461	1.656794	2.926081	2.870629	1.352144
Novel_009493	XLOC_10084.	transcript	0.349999	0	0.317726	0	0.325222	0.001802	0.629196	0	0.347081
Novel_009494	XLOC_10084.	transcript	0	0.342886	0	0.412392	0.052405	0.397411	0.049321	0.496237	0.003087
Novel_009495	XLOC_10084.	transcript	0.016004	0.031998	0	1.049157	0.71167	0.699453	0.258515	0.291285	0.059361
Novel_009496	XLOC_10084.	transcript	0.468502	0.252955	0.577642	0	0.308838	0.380149	0.327484	0.280454	0.476646
Novel_009497	XLOC_10084.	transcript	0	0.040229	0	0	0	0	0.457589	0	0
Novel_009498	XLOC_10085.	transcript	0.137503	0.04643	0.886095	0.469017	0.777494	0.238494	0.342206	0.32846	0.157082
Novel_009499	XLOC_10085.	transcript	0.008291	0	0	0.16422	0.012251	0.00499	0.214803	0.11411	0.411568
Novel_009500	XLOC_10085.	transcript	0.626122	0.205603	0.264781	0.248568	0.299513	0.648543	0.678625	0.512268	0.629114
Novel_009501	XLOC_10086.	transcript	1.599068	2.120865	2.409431	3.764973	2.335827	3.393672	2.060326	2.348211	3.082822
Novel_009502	XLOC_10086.	transcript	0.494114	0.21955	0.84117	1.06936	0.763755	0.464017	1.217599	0.940943	0.913568
Novel_009503	XLOC_10086.	transcript	0.211317	0.347878	0.319903	0.222875	0.319841	0.49701	0.132082	0.109496	0.758032
Novel_009504	XLOC_10087.	transcript	0.117251	0.488119	0.148974	0.169571	0.476716	0.26641	0.269817	0.140081	0.187419
Novel_009505	XLOC_10087.	transcript	0.5664	1.155507	0.542958	0.732043	0.344867	0.585005	0.778132	0.803181	0.737894
Novel_009506	XLOC_10087.	transcript	0	0.134614	0	0	0.285966	0	0	0	0
Novel_009507	XLOC_10090.	transcript	0	0.170706	0	0	0.177934	0.433162	0.038018	0.472538	0
Novel_009508	XLOC_10092.	transcript	0	0.036002	0.299525	0.137801	0.323504	0	0.13917	0	0
Novel_009509	XLOC_10092.	transcript	0.090116	0.253104	0	0	0.662817	0	0	0	0.306244
Novel_009510	XLOC_10092.	transcript	0	0	0	0	0	0	0	0	0
Novel_009511	XLOC_10092.	transcript	0.707538	0.1637	0.536442	0.471792	0	0.404765	0.75499	0.715966	0.131214
Novel_009512	XLOC_10093.	transcript	0.0571	0.054608	0.035513	0.058309	0.050391	0.018331	0.010481	0.005112	0.037366
Novel_009513	XLOC_10093.	transcript	0	0.072039	0	0	0	0	0	0	0
Novel_009514	XLOC_10093.	transcript	0	0	0	0	0	0	0	0.030837	0
Novel_009515	XLOC_10093.	transcript	0.188002	0.372057	0.207496	0.218419	0.37776	0.277323	0.390706	0.373712	0.130472
Novel_009516	XLOC_10093.	transcript	0.031918	0.639923	0	0	0	0	0.108435	0	0
Novel_009517	XLOC_10095.	transcript	0	0.002536	0	0	0	0.036034	0.50923	0.003522	0
Novel_009518	XLOC_10099.	transcript	0.132828	0.77577	0.283265	0.3865	0.15484	0.086124	0.412456	0.180967	0.268557
Novel_009519	XLOC_10100.	transcript	0.123775	0	0	0	0	0	0.043123	0	0
Novel_009520	XLOC_10100.	transcript	0	0	0	0.324909	0	0	0.737623	0	0
Novel_009521	XLOC_10106.	transcript	0.426613	0.034957	0.271961	0.23342	0.403808	0.433214	0.294624	0.245166	0.248806
Novel_009522	XLOC_10111.	transcript	0.18632	0.021232	0.106224	0.099649	0.181362	0.15219	0.228165	0.247592	0.202512
Novel_009523	XLOC_10113.	transcript	0.209223	0.319128	0.343145	0.347006	0.322245	0.360651	0.375201	0.336026	0.391542
Novel_009524	XLOC_10115.	transcript	0.153474	0.04748	0.00208	0	0.160948	0.14951	0.005163	0	0.086158
Novel_009525	XLOC_10115.	transcript	0.54471	1.121445	0.77538	0.599264	0.810905	0.613187	0.846663	0.5485	0.73089
Novel_009526	XLOC_10116.	transcript	0.145092	0.445975	0.562139	0.368447	0.305294	0.505814	0.872707	0.292818	0.45367

Novel_009527	XLOC_10117.	transcript	0.58107	0.684067	0.569663	0.468756	0.610231	0.910504	0.366869	1.054138	0.826133
Novel_009528	XLOC_10117.	transcript	0.162252	0.119815	0.147389	0.298387	0	0	0.259831	0	0.070738
Novel_009529	XLOC_10117.	transcript	0.052929	0.113849	0.106973	0	0.038582	0.164759	0.219657	0.187595	0
Novel_009530	XLOC_10117.	transcript	0	0.191741	0	0.10829	0.196158	0	0.115171	0	0.259472
Novel_009531	XLOC_10118.	transcript	0.804519	0.102679	0	0.036024	0.501225	0.482893	0.135815	0.719387	0
Novel_009532	XLOC_10118.	transcript	0.339761	0.109136	0.337769	0.62326	0.542096	0.505336	0.255866	0.129375	0.113219
Novel_009533	XLOC_10118.	transcript	0	0	0	0	0.05662	0	0	0.262671	0
Novel_009534	XLOC_10119.	transcript	0.454022	0.563753	0.462597	0.649282	0.410592	0.683964	0.626147	0.292108	0.884636
Novel_009535	XLOC_10119.	transcript	0.180072	0.267586	0.04278	0	0	0	0	0.366168	0
Novel_009536	XLOC_10120.	transcript	0.396516	0.685767	0.287717	0.662222	0.468916	0.447493	0.53001	0.762196	0.734723
Novel_009537	XLOC_10121.	transcript	3.488718	1.648309	3.783748	7.461356	4.311118	4.276306	4.369021	5.56042	4.49623
Novel_009538	XLOC_10124.	transcript	0	0	0	0.006174	0	0	0.633065	0	0
Novel_009539	XLOC_10124.	transcript	0.003539	0	0.023648	0.241015	0.120737	0.425804	0	0.014395	0.011133
Novel_009540	XLOC_10124.	transcript	0	0	0	0	0	0.361024	0	0	0
Novel_009541	XLOC_10124.	transcript	0.343956	0.116755	0.480138	0.42566	0.533002	0.349387	0.019826	0.67794	0.448389
Novel_009542	XLOC_10124.	transcript	0	0	0.157502	0.011813	0.305711	0.364432	0.292533	0.238496	0.159242
Novel_009543	XLOC_10124.	transcript	0.029468	0	0.584389	0.204946	0.36787	0.253969	1.18292	0.366633	0
Novel_009544	XLOC_10124.	transcript	0.029686	0	0.062024	0.324859	0.017449	0.200973	0.199671	0.179175	0.155203
Novel_009545	XLOC_10124.	transcript	0.200423	0.320958	0.305324	0	0	0.507121	0	0.117201	0
Novel_009546	XLOC_10124.	transcript	0.778734	0.068956	0.034552	0.115739	0.211389	0.003408	0.086886	0.602848	0.713435
Novel_009547	XLOC_10124.	transcript	1.15828	1.803322	0.312305	0.183046	0.148493	0.17123	0.281341	0.894248	0.581205
Novel_009548	XLOC_10124.	transcript	0	0	0	0	0	0	0	0	0
Novel_009549	XLOC_10124.	transcript	0.223982	0	1.257576	1.430281	1.196661	0.970889	1.643368	0.516279	0
Novel_009550	XLOC_10124.	transcript	0.245931	0.614194	0.27602	0.398166	0.385931	0.389603	0.058655	0.296567	0.336014
Novel_009551	XLOC_10124.	transcript	0	0	0	0	0	0.002215	0.46347	0	0
Novel_009552	XLOC_10125.	transcript	0.079257	0	0.033665	0.006919	0.048853	0	0.203938	0.045225	0.017394
Novel_009553	XLOC_10129.	transcript	0.322441	0.008756	0.261004	0.093739	0.30641	0.143367	0.441039	0.291869	0.034385
Novel_009554	XLOC_10129.	transcript	0.114827	0	0.047404	0.015844	0.073289	0.079443	0.08317	0.093127	0.088897
Novel_009555	XLOC_10129.	transcript	0	0	0	0	0	0	0	0	0
Novel_009556	XLOC_10129.	transcript	0	0.880187	1.121121	0.557249	0	0	0.705183	1.144953	0
Novel_009557	XLOC_10129.	transcript	0	0.348511	0	0.512355	0.113815	0.3704	0.278268	0	0.223487
Novel_009558	XLOC_10129.	transcript	0.00056	0.120597	0.229942	0.083386	0.083931	0.11184	0.275887	0.163258	0.228092
Novel_009559	XLOC_10129.	transcript	0.433245	0.193126	0.232887	0.10426	0.393408	0.120073	0.218894	0.277666	0.36516
Novel_009560	XLOC_10133.	transcript	0	0.166934	0	0	0.054095	0.010521	0	0.297352	0
Novel_009561	XLOC_10133.	transcript	0.02531	0	0.266824	0.164398	0.075917	0.239582	0.166324	0	0.26986
Novel_009562	XLOC_10138.	transcript	0	0.12157	0	0.380768	0	0.244013	0.191699	0.383797	0.27386
Novel_009563	XLOC_10138.	transcript	0.026855	0	0	0.115136	0.037839	0	0.399818	0.401866	0
Novel_009564	XLOC_10138.	transcript	0.015284	0.1295	0.043072	0	0.082669	0.292439	0.182377	0.084452	0

Novel_009565	XLOC_10138.	transcript	0.494334	1.042541	1.001828	0.73598	0.739468	0.326348	0.645857	0.168259	0.303631
Novel_009566	XLOC_10138.	transcript	0.403987	0.67194	0.434788	0.536442	0.438084	0.476405	0.659644	0.664451	0.538925
Novel_009567	XLOC_10138.	transcript	0	0	0	0	0	0.033108	0	0	0
Novel_009568	XLOC_10139.	transcript	0.581148	0.564627	0.520057	0.637014	0.802429	0.508836	0.828647	0.826777	0.522458
Novel_009569	XLOC_10139.	transcript	0.157893	0.114745	0.13248	0.139816	0.201491	0.135961	0.195431	0.153265	0.08705
Novel_009570	XLOC_10141.	transcript	0.437344	0.496393	0.600581	0.385019	0.524393	0.82994	0.473327	0.472422	0.755785
Novel_009571	XLOC_10144.	transcript	0.230662	0.047521	0.511594	0.225176	0.256482	0.267674	0.134526	0.209578	0.189532
Novel_009572	XLOC_10144.	transcript	0.355316	0.086108	0.573554	0.665226	0.63163	0.451591	0.763257	0.69128	0.330735
Novel_009573	XLOC_10147.	transcript	0.587432	0.550878	0.608479	0.820469	0.770388	1.232296	0.7769	0.598582	0.573628
Novel_009574	XLOC_10147.	transcript	0	0	0.2658	0.062866	0.018449	0.124875	0.011398	0.233371	0
Novel_009575	XLOC_10147.	transcript	0.303565	0.578708	0.188859	0.294716	0.32208	0.411155	0.524245	0.297461	0.201685
Novel_009576	XLOC_10147.	transcript	0	0	0	0	0	0	0	0	0
Novel_009577	XLOC_10148.	transcript	0.384087	0	0	0.008257	0	0	0.658245	0	0
Novel_009578	XLOC_10148.	transcript	0.344745	0.025096	0.021274	0.021995	0.022097	0.017822	0.015111	0.543337	0.003108
Novel_009579	XLOC_10148.	transcript	0	0.470495	0.39138	0.532344	0.531085	0.592364	0	0.11248	0.305521
Novel_009580	XLOC_10149.	transcript	0.309184	0	0.450415	0.324569	0	0	0.147377	0.118744	0.33016
Novel_009581	XLOC_10149.	transcript	0	0.286215	0	0.024775	0.377457	0.528781	0.430778	0.329438	0
Novel_009582	XLOC_10149.	transcript	0	0	0	0	0	0	0	0	0
Novel_009583	XLOC_10155.	transcript	0.170855	0.061811	0.085981	0.192352	0.24509	0.0142	0.126471	0.239552	0.180586
Novel_009584	XLOC_10157.	transcript	0.082715	0	0.187705	0.00058	0.065772	0.135156	0.098935	0.027473	0.005111
Novel_009585	XLOC_10157.	transcript	0.212892	0.017678	0.032572	0.047417	0.043784	0.133336	0.13706	0	0
Novel_009586	XLOC_10157.	transcript	0.021134	0	0	0	0	0	0.075999	0	0
Novel_009587	XLOC_10157.	transcript	0	0	0	0	0	0.012553	0.070991	0	0
Novel_009588	XLOC_10157.	transcript	0.061402	0.118491	0.203238	0	0.10703	0	0.319012	0.171399	0.055962
Novel_009589	XLOC_10157.	transcript	0	0.031706	0	0.115827	0	0.735013	0.343573	0.252997	0.189539
Novel_009590	XLOC_10157.	transcript	0.085106	0.02239	0.12832	0.231491	0	0.166118	0.131347	0.072106	0
Novel_009591	XLOC_10157.	transcript	0.001991	0	0.079977	0.039369	0.063042	0.003101	0.001807	0.036107	0.004304
Novel_009592	XLOC_10157.	transcript	1.236569	0	0	0.248679	0.529422	0	0.178507	0.110885	0.161818
Novel_009593	XLOC_10157.	transcript	0	0	0	0	0.00275	0.096028	0	0	0.418309
Novel_009594	XLOC_10157.	transcript	0	0	0	0	0.598559	0	0.715338	0.503942	0.386254
Novel_009595	XLOC_10157.	transcript	0	0	0.777516	0.070332	0.336448	0	0	0.314653	0
Novel_009596	XLOC_10171.	transcript	0.891886	0.556331	1.514327	0.63691	1.266213	1.999723	1.120414	0.726052	1.789064
Novel_009597	XLOC_10178.	transcript	0.015733	0.010652	0.014234	0	0	0.15213	0.859544	0.449707	0.803737
Novel_009598	XLOC_10178.	transcript	0	0.193004	0	0	0	0	0	0	0
Novel_009599	XLOC_10178.	transcript	0.35214	0.410145	0.409575	0.390697	0.530133	0.439988	0.093566	0.190877	0.065972
Novel_009600	XLOC_10178.	transcript	0.318299	0	0.124866	0.146195	0.08288	0	0.176077	0.06174	0
Novel_009601	XLOC_10180.	transcript	0.339889	0.408803	0.449902	0.643848	0.56446	0.601822	0.370622	0.638453	0.493383
Novel_009602	XLOC_10180.	transcript	0	0	0	0	0	0	0	0	0

Novel_009603	XLOC_1018[illegible]	transcript	0.489188	0.56996	0.608576	0.581703	0.433429	0.476758	0.71265	0.535617	0.363154
Novel_009604	XLOC_1019[illegible]	transcript	0.266669	0.978597	0.22729	0.17743	0.247143	0.186915	0.42849	0.237316	0.19007
Novel_009605	XLOC_1019[illegible]	transcript	0.098692	0	0	0	0	0	0	0	0
Novel_009606	XLOC_10197	transcript	20.86531	30.24289	27.43955	22.76978	23.85229	32.14618	27.97633	33.08026	20.99907
Novel_009607	XLOC_1019[illegible]	transcript	0.2697	0.008407	0.743799	0.342052	0.629963	1.039154	0.43052	0.77493	0.752714
Novel_009608	XLOC_1020[illegible]	transcript	0.021222	0	0.011218	0.008893	0.010853	0.012461	0	0	0.012198
Novel_009609	XLOC_1020[illegible]	transcript	0	0	0	0	0	0	0.012915	0.019087	0
Novel_009610	XLOC_1020[illegible]	transcript	0.017089	0	0.115536	1.490395	0.169275	0.202512	0.29794	0.328726	0.117405
Novel_009611	XLOC_1020[illegible]	transcript	0.266249	0	0.0255	0.092622	0.036507	0	0.032366	0.03606	0.025315
Novel_009612	XLOC_1020[illegible]	transcript	0	0	0	0.486827	0.493831	0.088141	0	0.403044	0.037237
Novel_009613	XLOC_1020[illegible]	transcript	0	0	0	0.001834	0.020607	0	0.014514	0	0
Novel_009614	XLOC_1020[illegible]	transcript	0	0	0	0.579271	0.51613	0	0	0.635542	0
Novel_009615	XLOC_1022[illegible]	transcript	0.132782	0	0.099645	0.060631	0.086058	0.022564	0.206643	0.080236	0.006501
Novel_009616	XLOC_1022[illegible]	transcript	0.263918	0	0.152168	0.085289	0.131656	0	0.343176	0	0.046301
Novel_009617	XLOC_1023[illegible]	transcript	0.044322	0.230463	0.05033	0.048778	0.11333	0	0.087475	0.120667	0
Novel_009618	XLOC_1023[illegible]	transcript	0	0	0	0	0.046007	0	0	0	0
Novel_009619	XLOC_1023[illegible]	transcript	0	0	0	0	0	0	0	0	0
Novel_009620	XLOC_1023[illegible]	transcript	0	0	0.158683	0	0	0	0	0	0
Novel_009621	XLOC_1023[illegible]	transcript	0.00031	0	0	0.009306	0	0.000709	0	0.005833	0
Novel_009622	XLOC_1023[illegible]	transcript	0.053433	0.01958	0.025376	0.010632	0.023597	0.040891	0.031895	0.025413	0.004517
Novel_009623	XLOC_1023[illegible]	transcript	0	0	0.001395	0	0.005315	0	0.002467	0	0
Novel_009624	XLOC_1023[illegible]	transcript	0.235206	0.317083	0.191602	0.360728	0.297276	0.423136	0.321189	0.002872	0.349453
Novel_009625	XLOC_1023[illegible]	transcript	0.010764	0.017609	0.00475	0.037509	0	0.026116	0.025564	0.417201	0.032427
Novel_009626	XLOC_1023[illegible]	transcript	0.032352	0.155428	0.0304	0	0.057534	0.028569	0.069865	0.235891	0.040342
Novel_009627	XLOC_1023[illegible]	transcript	0.444457	0.213632	0.349149	0.787835	0.670053	0.474361	0.821956	0.410809	0.603239
Novel_009628	XLOC_1023[illegible]	transcript	0	0	0	0	0	0	0	0	0.132677
Novel_009629	XLOC_1023[illegible]	transcript	0	0	0	0	0	0	0	0.066444	0
Novel_009630	XLOC_1023[illegible]	transcript	0.083719	0.211113	0.113797	0.123324	0.268336	0.119483	0.094887	0.056429	0.056648
Novel_009631	XLOC_1023[illegible]	transcript	0	0	0	0	0	0	0	0	0
Novel_009632	XLOC_1023[illegible]	transcript	0	0	0	0.017535	0	0.012044	0.026427	0.126586	0
Novel_009633	XLOC_1023[illegible]	transcript	0	0.026644	0	0.274956	0.025178	0.017707	0.221966	0.005356	0.054916
Novel_009634	XLOC_1023[illegible]	transcript	0	0	0	0.192065	0	0	0	0	0
Novel_009635	XLOC_1023[illegible]	transcript	0	0	7.405442	0	0.052082	0.065927	0.974467	0	0
Novel_009636	XLOC_1023[illegible]	transcript	0	0	0	0	0	0	0	0.644955	0
Novel_009637	XLOC_1023[illegible]	transcript	0.010117	5.910138	0.311309	0.57659	0.400542	0.699524	1.80E-05	0.387878	2.024678
Novel_009638	XLOC_1023[illegible]	transcript	1.20E-05	0	0	0	0	0	0.936115	0.001441	0
Novel_009639	XLOC_1023[illegible]	transcript	2.800871	0	0	0.710263	0	0	0.52865	1.220204	0
Novel_009640	XLOC_1023[illegible]	transcript	0	0	0	0	0	0	0	0	0

Novel_009641	XLOC_10237.	transcript	0.706079	0.604691	0.410478	0.156417	0.279971	0.454498	0.491939	0.338046	0.334591
Novel_009642	XLOC_10238.	transcript	0.263451	0.323146	0.13505	0.106405	0.106027	0.166076	0.219452	0.144359	0.155995
Novel_009643	XLOC_10247.	transcript	0.204648	0.422429	0.179806	0.143476	0.127155	0.205747	0.225979	0.192928	0.04737
Novel_009644	XLOC_10247.	transcript	0.072072	0	0.006426	0	0	0	0.047204	0.022122	0.04314
Novel_009645	XLOC_10285.	transcript	0	0.847137	0	0	0	0	0	0	0
Novel_009646	XLOC_10285.	transcript	0	0.872808	0	0	0	0	0.000865	0	0
Novel_009647	XLOC_10287.	transcript	0.174655	0.220608	0.285465	0.272082	0.205016	0.320344	0.33523	0.214067	0.189246
Novel_009648	XLOC_10288.	transcript	0.483069	0.012318	0.619036	0.342693	0.615509	0.577222	0.860485	0.64374	0.4742
Novel_009649	XLOC_10288.	transcript	0.137188	0.139013	0.289723	0.16741	0.218938	0.22486	0.120157	0.120141	0.373693
Novel_009650	XLOC_10288.	transcript	0	0	0	0	0	0	0.433862	0	0
Novel_009651	XLOC_10289.	transcript	0.103586	0.005069	0.04433	0.038363	0.048815	0.107903	0.014486	0.014357	0.006172
Novel_009652	XLOC_10289.	transcript	0.294335	0.089099	0.121588	0.314935	0.157072	0.260249	0.401507	0.235422	0.099209
Novel_009653	XLOC_10289.	transcript	1.049535	0.108069	0.842737	1.017319	0.099663	0.570192	0.7113	0.054855	0.665311
Novel_009654	XLOC_10289.	transcript	0	0	0.013931	0	0	0	0	0.083056	0
Novel_009655	XLOC_10289.	transcript	0	0	0	0	0	0	0	0	0
Novel_009656	XLOC_10289.	transcript	0	1.604396	0.104399	0.012839	0.835222	0.538907	0.814769	0.132579	0.00766
Novel_009657	XLOC_10289.	transcript	0	0	0	0	0	0	0	1.227185	0
Novel_009658	XLOC_10289.	transcript	0.814627	0.74337	0.694904	0.86433	0.932341	1.101183	1.142206	1.043176	0.773415
Novel_009659	XLOC_10289.	transcript	0.180035	0	0.368451	0.327378	0.158945	0.471507	0.168951	0.315334	0.091687
Novel_009660	XLOC_10289.	transcript	0.363577	0.436441	0.671365	0.806704	0.73496	0.612121	0.518495	0.4152	0.607264
Novel_009661	XLOC_10289.	transcript	0.432703	0.293078	0.284043	0.157459	0.293591	0.224931	0.516019	0.379244	1.144702
Novel_009662	XLOC_10290.	transcript	0.393997	0.120136	0.171041	0.115576	0.636931	0.284703	0.542674	0.378724	0.232866
Novel_009663	XLOC_10290.	transcript	0	0	0	0.461784	0	0.842962	0.737658	0.719295	0.30224
Novel_009664	XLOC_10290.	transcript	0	0	0	0	0	0	0	0	0
Novel_009665	XLOC_10290.	transcript	0	0	1.264276	0	0	0	0	0	0
Novel_009666	XLOC_10290.	transcript	0	0	0	0	0.000153	0	0.001011	0	0
Novel_009667	XLOC_10292.	transcript	0	0	0	0	0	0.244428	0.097257	0	0
Novel_009668	XLOC_10292.	transcript	0.433768	0	0	0.004548	0.217296	0.257371	0	0.070348	0
Novel_009669	XLOC_10292.	transcript	0.003147	0.057953	0	0	0	0.010998	0	0.356392	0
Novel_009670	XLOC_10292.	transcript	0	0	0.060367	0	0.002574	0.065791	0.076647	0.014522	0.183708
Novel_009671	XLOC_10295.	transcript	0.864127	0.308642	0.589202	0.817239	0.39001	0.83269	0.79785	1.398257	0.14677
Novel_009672	XLOC_10296.	transcript	1.971497	1.165994	1.646523	2.15733	1.785863	1.312024	3.074845	1.394517	1.881832
Novel_009673	XLOC_10296.	transcript	0.051008	0.033419	0.199087	0.034172	0.093871	0.84165	0.146595	0.278285	0.022821
Novel_009674	XLOC_10296.	transcript	0.009443	0.263319	0.029157	0	0.016324	0.012628	0.022402	0.037105	0.284293
Novel_009675	XLOC_10297.	transcript	0.683559	0.176673	0.893654	0.32573	0.56789	0.36486	0.530024	0.522729	0.392751
Novel_009676	XLOC_10297.	transcript	0.435575	0.242958	0.261781	0.329338	0.359929	0.523381	0.626405	0.308435	0.077987
Novel_009677	XLOC_10303.	transcript	0	1.69685	0	0.022191	0	0.060057	0	0	0
Novel_009678	XLOC_10303.	transcript	0	645.2631	0	0	0	94.37936	0	0	0

Novel_009679	XLOC_10303.	transcript	0	0	0	0	0	0	0	0	0
Novel_009680	XLOC_10303.	transcript	10.91061	23.48372	4.578517	1.491336	0	0	0	6.388641	1.180321
Novel_009681	XLOC_10303.	transcript	25.26105	791.102	12.45356	0.841548	14.00738	2.077527	12.95913	0	2.203033
Novel_009682	XLOC_10303.	transcript	7.817708	62.32106	3.485694	1.242192	7.964251	175.7554	5.911958	0.110736	0.525922
Novel_009683	XLOC_10303.	transcript	0	0.741551	0	0	0	0	0	2.964878	0
Novel_009684	XLOC_10303.	transcript	0	316.3733	0	0	0	36.2073	0	0	0
Novel_009685	XLOC_10303.	transcript	0	73.05144	0	0	0	0	0	0	0
Novel_009686	XLOC_10303.	transcript	0.981761	686.1571	0.106755	0.640971	1.634408	1.019276	0.661691	2.68743	0
Novel_009687	XLOC_10303.	transcript	0.005774	0.876134	0	3.983595	0	0	0	10.1456	0
Novel_009688	XLOC_10303.	transcript	0	1.606301	0	0.637506	0	0	1.614464	0.674119	0
Novel_009689	XLOC_10304.	transcript	0.517406	0.621695	0.453805	0.568529	0.365055	0.388579	0.654631	0.306036	0.734358
Novel_009690	XLOC_10305.	transcript	0	0	0.049896	0	0.00812	0.084548	0	0.010751	0
Novel_009691	XLOC_10305.	transcript	0.039715	0	0.169865	0.199159	0.124618	0.111647	0.115231	0.083602	0
Novel_009692	XLOC_10307.	transcript	0.895285	1.030834	1.333261	1.091134	1.050722	1.290656	1.592165	1.228575	1.134346
Novel_009693	XLOC_10307.	transcript	0.00242	0.003799	0.005163	0.001059	0	0	0.00313	0.208144	0
Novel_009694	XLOC_10309.	transcript	0	0.963925	0	0	0.032573	0	0	0	0
Novel_009695	XLOC_10309.	transcript	0.759943	0.025877	0.365575	0.515031	0.486107	0.565304	0.922669	0.644032	0.584055
Novel_009696	XLOC_10310.	transcript	0.958559	0.155265	0.113167	0.137016	0.437245	0.194092	0.516402	0.308496	2.854486
Novel_009697	XLOC_10310.	transcript	0	0.214203	0	0.000979	0.003234	0.006152	0.130375	0	0.003513
Novel_009698	XLOC_10313.	transcript	0.073078	0.078363	1.088337	0.013743	0.841708	0.170751	1.255146	0.059469	0.065389
Novel_009699	XLOC_10313.	transcript	0.013106	0.039685	0	0.073467	0	0.007912	0.059579	0.060273	0.003192
Novel_009700	XLOC_10313.	transcript	0.747396	0.260691	0	0.328473	0	0.473846	0	0.442224	0.576672
Novel_009701	XLOC_10314.	transcript	1.230939	3.440476	1.347992	1.307512	1.010806	1.923811	1.943632	1.333876	3.613492
Novel_009702	XLOC_10314.	transcript	0.367647	1.260162	0.778876	0.273686	0.616559	1.00372	0.357162	0	0.599684
Novel_009703	XLOC_10314.	transcript	0	0.109574	0.063899	0	0	0.072916	0	0.206264	0.102371
Novel_009704	XLOC_10314.	transcript	0.523925	0.564053	0.742274	1.093819	0.880107	1.463263	0.839481	0.515819	1.090425
Novel_009705	XLOC_10314.	transcript	0.068653	0.110201	0.381998	0.352485	0.334784	0.734268	0.78994	0.643107	0.643725
Novel_009706	XLOC_10314.	transcript	0.034035	0.051103	0.045631	0.081674	0.05683	0.182885	0.05971	0.08501	0.035633
Novel_009707	XLOC_10314.	transcript	0.336224	0.305663	0.228494	0.117534	0.138644	0.255778	0.225435	0	0.360452
Novel_009708	XLOC_10316.	transcript	0.370627	0.387239	0.813134	0.591397	0.481154	0.464246	0.629468	0.566128	0.433731
Novel_009709	XLOC_10316.	transcript	0	0	0	0	0	0	0	0	0.003762
Novel_009710	XLOC_10317.	transcript	1.302924	2.095086	1.124326	1.037645	1.151011	1.226317	1.58125	1.700505	1.593557
Novel_009711	XLOC_10318.	transcript	23.09309	85.65213	49.62549	36.53371	36.14295	53.69214	44.26241	45.07341	75.38026
Novel_009712	XLOC_10319.	transcript	0.033212	0.841401	0	0.069339	0.277126	0.266793	0.06287	0.148755	0.122549
Novel_009713	XLOC_10319.	transcript	0	0	0	0.026565	0	0	0.11812	0	0
Novel_009714	XLOC_10327.	transcript	1.618376	1.745115	1.920208	1.512058	1.651076	2.228272	1.255692	1.545373	1.299298
Novel_009715	XLOC_10327.	transcript	0	0.002059	0	0	0	0	0	0	0
Novel_009716	XLOC_10328.	transcript	0.409481	0.002639	0.165752	0.096202	0.333046	0.254052	0.428822	0.442197	0.329488

Novel_009717	XLOC_10328.	transcript	0	0	0.000802	0	0	0	0.000567	0.000868	0
Novel_009718	XLOC_10330.	transcript	0.214184	0.136413	0.259178	0.324894	0.24097	0.250181	0.387106	0.337185	0.248577
Novel_009719	XLOC_10330.	transcript	2.066154	1.937463	2.369801	2.216884	1.643243	2.355509	1.410848	2.259949	2.90542
Novel_009720	XLOC_10332.	transcript	0.251369	0.314171	0.239472	0.288324	0.141706	0.275265	0.28002	0.264462	0.371692
Novel_009721	XLOC_10332.	transcript	0	0	0	0	0	0	0	0	0
Novel_009722	XLOC_10338.	transcript	0.331255	0.432957	0.57036	0.564532	0.465516	0.437408	0.528357	0.533886	0.255927
Novel_009723	XLOC_10340.	transcript	0	0.006016	0	0.013059	0.001575	0	0.045578	0.000223	0.223136
Novel_009724	XLOC_10340.	transcript	0.731301	1.206485	0.588112	0.524779	0.771714	0.526041	0.815277	0.641342	0.626631
Novel_009725	XLOC_10340.	transcript	0.008577	0.045024	0	1.20E-05	0.047807	0	1.10E-05	0.143233	0.140653
Novel_009726	XLOC_10340.	transcript	0	0	0	0	0	0	0	0.133488	0
Novel_009727	XLOC_10344.	transcript	0.030614	0.202981	0.032366	0	0.125038	0	0.181336	0	0.126985
Novel_009728	XLOC_10360.	transcript	0.00893	0.58831	0	0	0	0.005791	0.005343	0.002757	0
Novel_009729	XLOC_10360.	transcript	0	0.777015	0	0	0.071469	0	0	0	0
Novel_009730	XLOC_10361.	transcript	0.135556	0.080029	0.070071	0.044038	0.168373	0.070405	0.34412	0.121999	0.114292
Novel_009731	XLOC_10372.	transcript	0.057373	0	0	0	0.07178	0.00585	0	0	0
Novel_009732	XLOC_10372.	transcript	0.316215	0.34197	0.272146	0.311044	0.248137	0.344753	0.351621	0.282124	0.312118
Novel_009733	XLOC_10372.	transcript	0	0	0	0	0	0	0.005387	0	0
Novel_009734	XLOC_10372.	transcript	0.00257	0	0	0	0	0	0	0	0
Novel_009735	XLOC_10372.	transcript	0.085991	0	0.139882	1.788206	0.113151	0.198842	0.133857	0.085108	0.047507
Novel_009736	XLOC_10372.	transcript	0	0	0.298997	2.47015	0	0.760216	0.107946	0.368704	0
Novel_009737	XLOC_10372.	transcript	0	0	0	0.350696	0	0	0	0	0
Novel_009738	XLOC_10372.	transcript	0.00727	0.012712	0.014418	0.282737	0.041724	0.006163	0.0636	0.077763	0.032764
Novel_009739	XLOC_10374.	transcript	0.646748	0.706421	0.945761	1.146477	0.647321	1.021189	0.901716	0.752654	0.804766
Novel_009740	XLOC_10383.	transcript	0.85152	0.76219	0.97433	0.815469	0.889158	0.91237	1.00816	0.909778	1.166425
Novel_009741	XLOC_10383.	transcript	0.023397	0	0	0	0	0.015377	0.017115	0.023609	0
Novel_009742	XLOC_10384.	transcript	0.134719	0.142031	0.165227	0.022919	0.27303	0.123187	0.265743	0.157981	0.145463
Novel_009743	XLOC_10386.	transcript	0.672149	0.236307	0.645219	0.412004	1.004742	0.508101	0.837777	0.605632	0.710189
Novel_009744	XLOC_10388.	transcript	0.270609	0.528874	0.154845	0.189273	0.163672	0.192969	0.245481	0.141138	0.118512
Novel_009745	XLOC_10388.	transcript	0	0	0	0	0.125819	0	0.018442	0	0.041788
Novel_009746	XLOC_10388.	transcript	0.060044	0.03133	0	0.036339	0.086313	0	0.117882	0.043011	0.065781
Novel_009747	XLOC_10388.	transcript	0.058916	0.041697	0.122223	0.041933	0.082958	0.535358	0.17133	0.054548	0.048832
Novel_009748	XLOC_10388.	transcript	0.060544	0.033688	0	0.041027	0.09305	0	0.113385	0.041238	0.044188
Novel_009749	XLOC_10388.	transcript	0.057634	0.027209	0.08279	0.039654	0.054678	0.046272	0.206568	0.130195	0.076683
Novel_009750	XLOC_10388.	transcript	0.075465	0.029392	0.120217	0.047794	0.184444	0.000147	0	0.082234	0.076481
Novel_009751	XLOC_10388.	transcript	0.203941	0.149001	0.24334	0.179119	0.148943	0	0	0.332135	0.22365
Novel_009752	XLOC_10388.	transcript	0.640437	0.366345	0	0.402413	0.162636	0	0.183974	0.183858	0.465315
Novel_009753	XLOC_10389.	transcript	0.133463	0.474172	0.004801	0.129959	0.109664	0.012773	0	0.10393	0
Novel_009754	XLOC_10389.	transcript	0	0	0.000131	0	0	0	0	0	0

Novel_009755	XLOC_1038[illegible]	transcript	0	0.111782	0.056249	0	0	0.007986	0.150871	0	0.104198
Novel_009756	XLOC_1038[illegible]	transcript	0	1.21744	0	0	0	0.143637	0	0	0
Novel_009757	XLOC_1038[illegible]	transcript	0	0.699638	0	0	0	0	0	0	0
Novel_009758	XLOC_1038[illegible]	transcript	0	0	0.062128	0	0	0	0	0	0
Novel_009759	XLOC_1039[illegible]	transcript	0.031198	0.076346	0	0.226566	0	0.464638	0.2203	0.245779	0.271822
Novel_009760	XLOC_1039[illegible]	transcript	0	0	0.250764	0	0.300671	0	0	0	0
Novel_009761	XLOC_1039[illegible]	transcript	0.731099	0.747725	0.727124	0.870793	0.96171	0.724576	0.934334	0.8964	1.237061
Novel_009762	XLOC_1039[illegible]	transcript	0.132065	0.741805	0.12227	0.055388	0.033797	0.119605	0.176689	0.067744	0.067781
Novel_009763	XLOC_1039[illegible]	transcript	0.870503	1.429123	0.766108	0.912941	0.94492	1.274516	1.015154	1.455006	0.716852
Novel_009764	XLOC_1039[illegible]	transcript	0	0	0	0	0.09423	0	0	0	0
Novel_009765	XLOC_1039[illegible]	transcript	0.423608	0.642079	0.554196	0.716157	0.536834	0.551503	0.693305	0.530805	0.896893
Novel_009766	XLOC_1039[illegible]	transcript	0.153364	0.342487	0.291572	0.362241	0.278098	0.614904	0.09837	0.262321	0.182606
Novel_009767	XLOC_1039[illegible]	transcript	0	0	0	0	0	0	0	0	0.025562
Novel_009768	XLOC_1039[illegible]	transcript	1.408796	0.497605	0	0	0	0	0	0	0
Novel_009769	XLOC_1039[illegible]	transcript	1.042884	0.134134	0	0	0	0	0.6709	0	0
Novel_009770	XLOC_1039[illegible]	transcript	1.758838	2.206183	1.763551	1.558811	1.465841	1.57553	1.861003	2.06429	1.197749
Novel_009771	XLOC_1039[illegible]	transcript	0	0	0	0	0	0	0	0	0
Novel_009772	XLOC_1040[illegible]	transcript	1.000136	1.441567	1.180671	1.586368	1.192673	1.195387	1.528542	1.434336	1.361694
Novel_009773	XLOC_1040[illegible]	transcript	2.370182	1.896491	3.242174	4.446355	3.684061	3.556785	3.072747	3.572765	3.263089
Novel_009774	XLOC_1040[illegible]	transcript	0.130362	0.152291	0.118136	0.33261	0.124172	0.302211	0.169992	0.170696	0.123384
Novel_009775	XLOC_1040[illegible]	transcript	0.389875	0.055791	0.476281	0.27264	0.473006	0.213619	0.195418	0.649061	0.125723
Novel_009776	XLOC_1040[illegible]	transcript	8.083031	0.999157	9.220537	9.028312	9.050898	9.739791	11.39617	8.099896	9.481589
Novel_009777	XLOC_1040[illegible]	transcript	0	0	0.027343	0.015825	0	0	0	0	0
Novel_009778	XLOC_1040[illegible]	transcript	0	0	0	0.187217	0.04456	0.041645	0.011241	0.208454	0
Novel_009779	XLOC_1040[illegible]	transcript	0.517002	0.289754	0.380138	0.574779	0.50872	0.471704	0.503069	0.439774	0.536713
Novel_009780	XLOC_1040[illegible]	transcript	0.020542	0.006813	0.065092	0.065183	0.032425	0.0194	0.018156	0.055958	0.063322
Novel_009781	XLOC_1040[illegible]	transcript	0.518464	0	0	0	0	0.282433	0.104628	0	0.410649
Novel_009782	XLOC_1040[illegible]	transcript	0.001279	0.292737	0	0.441938	0	0.486429	0	0.61671	0.393186
Novel_009783	XLOC_1040[illegible]	transcript	0.167041	0	0	0	0.452578	0.635957	0	0	0
Novel_009784	XLOC_1040[illegible]	transcript	0	1.601188	0.097574	0	0.58	0.667726	0	0.460092	0.496874
Novel_009785	XLOC_1040[illegible]	transcript	0.114367	0.2994	0.253412	0.487969	0.233219	0.175825	0.304239	0.159042	0.267223
Novel_009786	XLOC_1040[illegible]	transcript	0	0.023743	0.016877	0.063488	0	0.037671	0.044148	0	0.008673
Novel_009787	XLOC_1040[illegible]	transcript	0.606184	0.544657	0.545968	0.813218	0.611335	0.782431	0.547044	0.62241	1.319379
Novel_009788	XLOC_1040[illegible]	transcript	1.322816	2.299679	1.359178	1.314829	1.35903	1.108528	1.568461	1.477877	1.661418
Novel_009789	XLOC_1040[illegible]	transcript	0.640464	0.491916	1.00145	0.489734	1.212681	1.076317	1.601859	0.581265	0.580305
Novel_009790	XLOC_1040[illegible]	transcript	0.171321	0	0.108704	0.143585	0.136385	0.082185	0.12677	0.235574	0.045321
Novel_009791	XLOC_1040[illegible]	transcript	0	0.518796	0.331851	0	0	0	0.095482	0	0
Novel_009792	XLOC_1040[illegible]	transcript	0.044549	0.079351	0.057907	0.05797	0.017963	0	0.176356	0.068408	0.091999

Novel_009793	XLOC_10409.	transcript	0	0	0.010874	0.014676	0	0.006379	0.435465	0	0.005466
Novel_009794	XLOC_10409.	transcript	0.72522	0.326829	0.010393	0.463785	0.500394	0.416662	0.220083	0.594913	0.407468
Novel_009795	XLOC_10409.	transcript	0	0	0	0	0	0	0.039821	0.170247	0
Novel_009796	XLOC_10410.	transcript	0.23973	0.477657	0.255102	0.153428	0.160375	0.178285	0.380907	0.271745	0.14775
Novel_009797	XLOC_10411.	transcript	0.197583	0	0.51833	0	0.064816	0	0.47807	0.418323	0
Novel_009798	XLOC_10431.	transcript	0.196622	0.056969	0.09363	0.118299	0.112907	0.05767	0.182826	0.096534	0.134811
Novel_009799	XLOC_10435.	transcript	0.126895	0.028617	0.166229	0.129023	0.070769	0.07412	0.161603	0.155151	0.109655
Novel_009800	XLOC_10436.	transcript	0	0	0.116602	0	0.225609	0	0.213364	0	0
Novel_009801	XLOC_10455.	transcript	0	0	0	0.139955	0	0	0	0.004018	0
Novel_009802	XLOC_10455.	transcript	0.117118	0.024533	0.091143	0.063201	0.102309	0	0.136725	0.179966	0.112885
Novel_009803	XLOC_10455.	transcript	0	0	0.055323	0.054244	0	0.194649	0.143675	0.064155	0.109219
Novel_009804	XLOC_10464.	transcript	0.900483	0.593892	1.188957	1.470483	0.953644	0.912236	0.951963	0.997301	0.656721
Novel_009805	XLOC_10472.	transcript	0	0	0	0	0.731291	0	0	0	0.001241
Novel_009806	XLOC_10472.	transcript	0.695755	0.325632	1.308194	3.43558	1.763759	1.512209	1.546285	2.260176	0.213353
Novel_009807	XLOC_10472.	transcript	0	0	0	0	0	0.300907	0	0.434566	0.235034
Novel_009808	XLOC_10478.	transcript	0.330204	0.09189	0.255531	0.179475	0.216751	0.182583	0.497618	0.298355	0.375717
Novel_009809	XLOC_10478.	transcript	0.552028	0.018853	0.172382	0.72142	0.10752	0.360641	0.437938	0.636398	0.058165
Novel_009810	XLOC_10478.	transcript	0.09746	0	0.145049	0.740571	0.12876	0.065568	0.197413	0.841039	0.113545
Novel_009811	XLOC_10478.	transcript	0.319283	0	0	0.618431	0.732129	0	0	0.866425	0
Novel_009812	XLOC_10478.	transcript	0.298633	0	1.549667	0.727373	0.672291	1.480658	1.058193	0.754496	0.536627
Novel_009813	XLOC_10478.	transcript	0.292729	0	0.428157	0.711468	0.942943	0.48443	0.521878	0.734855	0.662575
Novel_009814	XLOC_10478.	transcript	0.004379	0	0.50007	0.75508	0.42056	0.292414	0.472039	0.544701	0.358656
Novel_009815	XLOC_10478.	transcript	0	0	0	0	0	0.010659	0.632404	0	0
Novel_009816	XLOC_10478.	transcript	0.001934	0	0.186934	0.627458	0.177388	0.138369	0.106586	0.168283	0
Novel_009817	XLOC_10478.	transcript	0.124753	0	0.076822	0.157794	0.113785	0.588237	0.28111	0.197427	0.162917
Novel_009818	XLOC_10478.	transcript	0.136829	0	0.08134	0.46697	0.281536	0.102989	0.040448	0.009429	0.092074
Novel_009819	XLOC_10480.	transcript	0.321003	1.825208	0.399235	0.539405	0.349021	0.675932	0.670325	0.391229	0.832495
Novel_009820	XLOC_10481.	transcript	0	0.038881	0.371811	0.215594	0.335712	0.789027	0.209344	0.187213	0.055597
Novel_009821	XLOC_10484.	transcript	0.200971	0	0.500342	0.142053	0.00843	0.277163	0	0	0
Novel_009822	XLOC_10484.	transcript	0.038556	0.463243	0	0.17192	0.039538	0	0.430973	0.387515	0.434017
Novel_009823	XLOC_10484.	transcript	0.011272	0.764373	0.15446	0.191175	0.132483	0.120947	0.01686	0.15696	0.106689
Novel_009824	XLOC_10485.	transcript	0.028851	0.127375	0.028858	0.066334	0.014754	0.025208	0.055921	0.046383	0
Novel_009825	XLOC_10485.	transcript	0.310372	0.1988	0.253069	0.228494	0.17945	0.296563	0.246767	0.187498	0.03765
Novel_009826	XLOC_10490.	transcript	0.261621	0.093839	0.14435	0.035677	0.178554	0.042199	0.473397	0.269558	0.001436
Novel_009827	XLOC_10490.	transcript	1.878083	0.771481	1.233555	1.6518	1.302231	0.960231	2.07771	2.076103	1.402502
Novel_009828	XLOC_10500.	transcript	0.148649	0.107935	0.210368	0.2231	0.04946	0.129333	0.569065	0.238938	0.050587
Novel_009829	XLOC_10500.	transcript	0.334572	0.337368	0.837111	0.457125	0.888535	0.588128	0.525235	0.612028	0.965356
Novel_009830	XLOC_10500.	transcript	0.005167	0.409061	0.000689	0.006297	0.004138	0.009415	0.012035	0.003606	0.023114

Novel_009831	XLOC_1050(	transcript	0.45412	0.342023	0.498319	1.027278	0.415706	0.771994	0.207147	0.50964	0.527009
Novel_009832	XLOC_1050(	transcript	0.080128	0.084273	0.157373	0.07109	0.151481	0.092345	0.421771	0.153529	0.16601
Novel_009833	XLOC_1050(	transcript	0	0	0	0	0	0	0	0.015225	0
Novel_009834	XLOC_1050(	transcript	0.001617	0.001524	0.052744	0.085585	0.064097	0.001951	0	0	0
Novel_009835	XLOC_1050(	transcript	0	0.002256	0	0	0	0	0	0.232763	0
Novel_009836	XLOC_1050{	transcript	0.47994	0.198298	0.64807	0.007035	0	1.434418	1.358576	1.109085	0
Novel_009837	XLOC_1050{	transcript	0.110249	0.334503	0.024859	0.168946	0.222073	0.031292	0.028835	0.016597	0.289969
Novel_009838	XLOC_1050‘	transcript	0	0.022812	0.008023	0	0.000727	0.157487	0.068326	0	0.053725
Novel_009839	XLOC_1050‘	transcript	0	0	0	0.243156	0	0	0	0.271937	0
Novel_009840	XLOC_1051‹	transcript	0.501603	0.507701	0.786878	0.694236	0.722877	0.660073	0.220772	0.714981	0.535631
Novel_009841	XLOC_1051‹	transcript	0.033919	0	0	0	0	0	0.497931	0	0
Novel_009842	XLOC_1051‹	transcript	0	0.415543	0.468721	0.448376	0.764584	0	0.977813	0.481632	0.679019
Novel_009843	XLOC_1051‹	transcript	0.17557	0.520332	0.314788	0.304595	0.517405	0.464342	0.624267	0.451687	0.167708
Novel_009844	XLOC_1051‹	transcript	0.352031	0	0.163756	0.097215	0	0	0.035649	0.115758	0.157723
Novel_009845	XLOC_1051‹	transcript	0	0	0	0.1475	0	0	0	0	0
Novel_009846	XLOC_1051‹	transcript	0.184345	0.142924	0.137067	0.217963	0.08007	0.097184	0.134422	0.179441	0.067789
Novel_009847	XLOC_1051‹	transcript	0.869282	1.301164	0.410239	0.313953	0.46978	0.402988	1.008768	0.528464	0.304065
Novel_009848	XLOC_1051‹	transcript	0.412917	1.028256	0.615536	1.213854	0.931612	0.827408	0.496747	1.342632	0.672387
Novel_009849	XLOC_1051‹	transcript	0	0	0	0	0	0	0	0	0
Novel_009850	XLOC_1051(	transcript	0.204047	0	0.187999	0.198163	0.300773	0.306819	0.146079	0.23162	0.077683
Novel_009851	XLOC_1051(	transcript	0	0	0	0	0	0	0.291667	0	0
Novel_009852	XLOC_1051‘	transcript	0.130676	0	0.147537	0	0.103219	0.146005	0.092695	0.134516	0.027945
Novel_009853	XLOC_1051‘	transcript	0.935145	0	1.387167	1.357963	2.072864	1.682644	1.667895	1.995754	0.918756
Novel_009854	XLOC_1051‘	transcript	0	0	0	0.915595	0	0	0	0	0
Novel_009855	XLOC_1051‘	transcript	0	0	0	0	0	0	0	0	0
Novel_009856	XLOC_1051‘	transcript	0	0	0.199153	0.238108	0.122437	0.418646	0.257377	0.135817	0
Novel_009857	XLOC_1051‘	transcript	0	0	0	0	0	0	0	0	0
Novel_009858	XLOC_1051‘	transcript	0.348305	1.050816	0.334616	0.366172	0.390406	0.387966	0.417114	0.492054	0.172859
Novel_009859	XLOC_1052(	transcript	0.548255	1.11553	0.509379	0.421197	0.314622	0.521018	0.443721	0.644495	0.359412
Novel_009860	XLOC_1052(	transcript	0	0.086566	0	0	0	0.352639	0.015952	0	0
Novel_009861	XLOC_1052(	transcript	7.56685	26.51729	18.08982	15.5738	13.95234	14.58784	9.151583	14.20293	11.83219
Novel_009862	XLOC_10521	transcript	0	0	0.189806	0	0.006674	0	0.019483	0	0
Novel_009863	XLOC_10521	transcript	0.036752	0.034827	0.025833	0	0	0.191142	0.14865	0.237067	0.036631
Novel_009864	XLOC_10521	transcript	0.471692	0.499434	0.442125	0	0.201911	0.283635	0.11535	0.144645	0.659552
Novel_009865	XLOC_10521	transcript	0.050747	0.050844	0.107162	0.11948	0.300556	0.241544	0.258133	0.194762	0.15905
Novel_009866	XLOC_10521	transcript	0.086071	0.019551	0	0.475705	0.153556	0	0	0.505763	0
Novel_009867	XLOC_10521	transcript	0	0	0	0	0	0	0	0	0
Novel_009868	XLOC_10521	transcript	0	0	0	0.008081	0.143579	0	0	0	0

Novel_009869	XLOC_10525.	transcript	1.387502	0.115882	1.526043	0.405451	0.269914	0.159942	0.473553	0.438098	0.596049
Novel_009870	XLOC_10525.	transcript	0	0	0	0	0	1.620618	0	0	0
Novel_009871	XLOC_10525.	transcript	0	0.354074	0	0.853538	1.15973	0	1.673115	1.294716	2.411461
Novel_009872	XLOC_10525.	transcript	0.038373	0.09541	0	0	0.038927	0.201814	0.057398	0	0.019005
Novel_009873	XLOC_10525.	transcript	2.678671	0.45784	3.705126	3.066557	3.404513	2.921898	3.46931	3.380643	3.597758
Novel_009874	XLOC_10526.	transcript	0.350416	0.362827	0.21905	0.340703	0.294164	0.099596	0.290986	0.433043	0.29145
Novel_009875	XLOC_10526.	transcript	0.225113	0.445642	0	0.153806	0.093848	0.148615	0.102937	0.302036	0.054372
Novel_009876	XLOC_10527.	transcript	0.605515	0.461184	0.638056	0.469886	0.506121	0.699103	0.922544	0.664541	0.670038
Novel_009877	XLOC_10527.	transcript	0.461649	0.50349	0.633416	0.536195	0.626115	0.454435	1.132431	1.217081	0.766209
Novel_009878	XLOC_10528.	transcript	0.134362	0.11507	0.212509	0.195144	0.28279	0.296444	0.434486	0.355999	0.308378
Novel_009879	XLOC_10528.	transcript	0.177762	0.15586	0.337971	0.236156	0.275124	0.240176	0.19807	0.129546	0.106831
Novel_009880	XLOC_10528.	transcript	0.129924	0	0.078554	0.321832	0.124547	0.203453	0.159734	0.342286	0.074578
Novel_009881	XLOC_10528.	transcript	0.573435	0	0.168878	0	0.440479	0.246488	0.448934	0.375063	0.163874
Novel_009882	XLOC_10528.	transcript	0.144581	0	0.200691	0.081471	0.061466	0.090571	0.08116	0.144752	0
Novel_009883	XLOC_10528.	transcript	0.022485	0	0.003472	0	0	0	0.016847	0.015989	0
Novel_009884	XLOC_10528.	transcript	0.555843	1.390265	0.859942	0.716682	0.71906	0.788717	0.766982	0.720106	0.760488
Novel_009885	XLOC_10528.	transcript	0.052001	1.107121	0.02264	0.1437	0.048994	0	0	0	0.076339
Novel_009886	XLOC_10528.	transcript	0	0	0	0	0	0	0	0	0
Novel_009887	XLOC_10528.	transcript	0	0	0	0	0	0	0.190118	0	0
Novel_009888	XLOC_10528.	transcript	0.194644	0.04996	0.339393	0.290084	0.32504	0.173959	0.385015	0.314339	0.365646
Novel_009889	XLOC_10528.	transcript	0.011947	0.385201	0.003522	0.009847	0.04511	0.194265	0.036858	0.011978	0.016907
Novel_009890	XLOC_10529.	transcript	0.290437	0.236275	0.337136	0.202792	0.164035	0.15908	0.349448	0.192069	0.260684
Novel_009891	XLOC_10529.	transcript	0.217138	0.103891	0.472582	0.377297	1.019226	0.112689	0.042977	0.155173	0.736121
Novel_009892	XLOC_10529.	transcript	0.138222	0	0.345576	0.506268	0.038928	0.430818	0.478701	0.705005	0.206102
Novel_009893	XLOC_10531.	transcript	0.03511	0.067599	0.323971	0.125894	0	0.150966	0	0.352134	0
Novel_009894	XLOC_10531.	transcript	0.245025	0.256897	0	0.385532	0.733599	0.251512	0.471675	0.202054	0.644728
Novel_009895	XLOC_10531.	transcript	0.019378	0.016514	0.300771	0	0.028248	0.068173	0.433732	0.324332	0.047701
Novel_009896	XLOC_10531.	transcript	0.325767	1.002876	0.799215	0.604986	1.133547	0.000263	1.125584	0.690919	0.03018
Novel_009897	XLOC_10531.	transcript	0.024132	1.292967	0.598806	0.849801	0.289218	0.037939	1.340731	1.001683	0.041053
Novel_009898	XLOC_10531.	transcript	0.001219	0	0.441569	0.551096	0.367766	1.958444	0.813914	1.137762	1.337366
Novel_009899	XLOC_10531.	transcript	1.946812	0	0	0	0.311229	0	0	0	0
Novel_009900	XLOC_10531.	transcript	0.302741	0.5611	0.03777	0	0	0.004958	0	0.200994	0.001279
Novel_009901	XLOC_10531.	transcript	0	0	0	0.914619	0.464433	0.160138	0.379809	0.280408	0.111566
Novel_009902	XLOC_10534.	transcript	0.433663	0.517939	0.761292	0.210992	0.599023	0.467948	0.424824	0.652691	0.360509
Novel_009903	XLOC_10534.	transcript	0.261966	0	0.179417	0.505154	0.210554	0.353587	0.403549	0	0.300856
Novel_009904	XLOC_10534.	transcript	0.388018	0.31656	0.078399	0.29352	0.207989	0.239955	0.427295	0.27644	0.204418
Novel_009905	XLOC_10535.	transcript	0	0.010294	0.159619	0.003446	0.011233	0.002363	0.01282	0.015936	0.011603
Novel_009906	XLOC_10535.	transcript	0	0.585755	0.028241	0.341159	0.377712	0.438679	0.428371	0.247378	0.306248

Novel_009907	XLOC_10535	transcript	0.225705	0.004708	0.444489	0.24702	0.00028	0.004303	0.000623	0.178162	0.286818
Novel_009908	XLOC_10536	transcript	0	0	0	0	0	0	0	0	0.198877
Novel_009909	XLOC_10536	transcript	0	0	0	0	0	0	0	0	0
Novel_009910	XLOC_10536	transcript	0	0	0	0	0	0	0	0	0
Novel_009911	XLOC_10536	transcript	0	0	0	0	0	0.199974	0	0	0
Novel_009912	XLOC_10536	transcript	0	0	0	0	0.153318	0	0	0	0
Novel_009913	XLOC_10536	transcript	0.371201	0.058396	0.295467	0.215088	0.002726	0.003212	9.50E-05	0.224804	0
Novel_009914	XLOC_10536	transcript	0	0	0	0	0.082376	0.019942	0.028856	0	0.000852
Novel_009915	XLOC_10536	transcript	0	0	0	0	0	0	0	0	0
Novel_009916	XLOC_10536	transcript	0	0	0	0	0.001231	0	0.049582	0	0.048809
Novel_009917	XLOC_10536	transcript	0	0	0	0	0	0	0	0	0
Novel_009918	XLOC_10536	transcript	0.181387	0.016142	0.091227	0.133559	0.125242	0.124847	0.246272	0.085443	0.102874
Novel_009919	XLOC_10540	transcript	0	0	0	0	0	0	0	0	0
Novel_009920	XLOC_10540	transcript	0.029166	0.902798	0.231839	0.16046	0.09745	0.069529	0.474845	0.505692	0.359003
Novel_009921	XLOC_10540	transcript	0	0	0	0	0	0	0	0	0
Novel_009922	XLOC_10540	transcript	0.14014	0	0	0	0.005655	0.008757	0.344107	0	0
Novel_009923	XLOC_10540	transcript	0.0436	0	0	0	0.213519	0.161151	0.15995	0	0
Novel_009924	XLOC_10540	transcript	0	0	0	0	0	0	0	0	0
Novel_009925	XLOC_10543	transcript	0	0	0.144714	0.152963	0.061808	0.063727	0.179501	0.126607	0
Novel_009926	XLOC_10544	transcript	0	0	0	0	0	0	0	0	0
Novel_009927	XLOC_10544	transcript	0.16414	0.043888	0.14678	0.221527	0.191274	0.161797	0.219752	0.340811	0.126655
Novel_009928	XLOC_10546	transcript	0	0	0	0	0	0	0	0	0
Novel_009929	XLOC_10546	transcript	0.011832	0	0	0	0	0.007711	0.054814	0.016253	0
Novel_009930	XLOC_10546	transcript	0.06426	0	0	0.018302	0.014638	3.70E-05	0.032748	0.032841	0.119127
Novel_009931	XLOC_10546	transcript	0.297823	0	0.119388	0.013758	0	0.002437	0.101669	0.224773	0.094802
Novel_009932	XLOC_10546	transcript	0	0	0.005411	0.195422	0	0.371869	0.080798	0	0
Novel_009933	XLOC_10546	transcript	0.044838	0	0.066044	0	0	0	0.06284	0.033089	0.127033
Novel_009934	XLOC_10546	transcript	0.087188	0	0	0	0	0	0.095669	0.174166	0
Novel_009935	XLOC_10549	transcript	0	0	0	0	0.524371	0.007796	0.00195	0	0
Novel_009936	XLOC_10549	transcript	1.135677	1.735652	1.332061	1.767816	1.388743	1.764084	1.642763	1.298746	1.591858
Novel_009937	XLOC_10552	transcript	0.098818	1.098382	0.118259	0.474284	0.143356	0.235356	0.326852	0.412805	0.141323
Novel_009938	XLOC_10553	transcript	0.0812	0.013459	0.196624	0.453045	0.348459	0.179708	0.459553	0.429014	0.096232
Novel_009939	XLOC_10553	transcript	0.524893	0.212205	0.591368	0.362044	0.401657	0.372901	0.071836	0.778064	0.183309
Novel_009940	XLOC_10553	transcript	0.017046	0.22896	0.015321	0.171669	0.173024	0.185408	0.646316	0.021002	0.351208
Novel_009941	XLOC_10553	transcript	0	0.000213	0	0	0	0	0	0	0
Novel_009942	XLOC_10553	transcript	0	0	0	0	0	0	0	0.014277	0
Novel_009943	XLOC_10553	transcript	0.341879	1.348087	0.414668	0.616774	0.276447	0.551094	0.677958	0	0.634197
Novel_009944	XLOC_10553	transcript	0	0.934788	0	0	0.208955	0.007565	0.037868	0.769561	0.070953

Novel_009945	XLOC_10553.	transcript	0	0	0	0	0	0	0.627388	0	0
Novel_009946	XLOC_10554.	transcript	0	0	0	0	0	0	0	0	0
Novel_009947	XLOC_10554.	transcript	0.145205	0.39771	0.682752	0.168786	0.615449	0.655014	0.595437	0.412971	0.523373
Novel_009948	XLOC_10554.	transcript	0.610319	1.529528	0.006987	0.613072	0.00095	0	0.406107	0.449998	0.19453
Novel_009949	XLOC_10555.	transcript	0.187842	0.412886	0.246241	0	0.150294	0	0.603379	0.214162	0.200135
Novel_009950	XLOC_10555.	transcript	0.346982	0.273074	0.292488	0.433742	0.150534	0.358088	0.140249	0.269396	0.325936
Novel_009951	XLOC_10556.	transcript	0.040215	0.06799	0.013941	0.073258	0.208885	0.216212	0.029861	0.01573	0.188907
Novel_009952	XLOC_10556.	transcript	0.262	0.366199	0.290677	0.01039	0.001322	0.02308	0.544053	0.558601	0.012667
Novel_009953	XLOC_10556.	transcript	0.290032	0.254146	0.003905	0	0.011689	0.001523	0	0	0
Novel_009954	XLOC_10556.	transcript	0.275306	0	0.421979	0.042451	0.488119	0.501219	0.487862	0.373129	0.43662
Novel_009955	XLOC_10556.	transcript	0.017142	0	0	0.510763	0	0	0.022154	0.005345	0
Novel_009956	XLOC_10558.	transcript	0	0	0	0.062111	0	0	0.027996	0	0.045118
Novel_009957	XLOC_10559.	transcript	0.239836	0.219071	0.027585	0.152856	0.147361	0.316702	0.836219	0.669765	0.009881
Novel_009958	XLOC_10559.	transcript	0.285198	0	0.193384	0.27806	0.27103	0.221255	0.000817	0	0.579612
Novel_009959	XLOC_10559.	transcript	0.021342	0.007621	0.016643	0.003601	0.119119	0.008194	0	0.047431	0
Novel_009960	XLOC_10559.	transcript	0	0	0.257927	0.095292	0.125641	0	0.051571	0	0.056878
Novel_009961	XLOC_10560.	transcript	1.64993	1.87503	2.478789	2.354592	2.088896	2.129696	2.484824	3.134413	4.684385
Novel_009962	XLOC_10560.	transcript	0	0.00393	0	0	0	0	0.017703	0.045829	0
Novel_009963	XLOC_10561.	transcript	0.174884	0.082002	0.171509	0.233855	0.153036	0.141515	0.276552	0.117624	0.18586
Novel_009964	XLOC_10562.	transcript	0.470722	0.112399	0.622201	0	0.74467	0.681853	0	0	0.670554
Novel_009965	XLOC_10562.	transcript	0	0.193231	0	0.048158	0.019928	0	0	0	0
Novel_009966	XLOC_10562.	transcript	0	0	0	0	0	0	0	0	0
Novel_009967	XLOC_10562.	transcript	0.107672	0.254567	0.310535	0.067297	0.238838	0.334664	0.228232	0.205628	0.08585
Novel_009968	XLOC_10562.	transcript	0	0	0	0.035237	0	0	0.564083	0.47159	0
Novel_009969	XLOC_10562.	transcript	0	0	0	0	0	0	0	0	0
Novel_009970	XLOC_10563.	transcript	0.263972	0.627498	0.542591	0.575811	0.461335	0.509134	0.219519	0.392533	0.254338
Novel_009971	XLOC_10563.	transcript	0	0.030565	0	0.038181	0.009334	0	0.016282	0	0
Novel_009972	XLOC_10563.	transcript	0.324249	0.119966	0.206584	0.154224	0.287511	0.121312	0.241193	0.281227	0.064354
Novel_009973	XLOC_10563.	transcript	0.247167	0.036281	0.106515	0.108395	0.229042	0.213373	0.12955	0.149697	0.104396
Novel_009974	XLOC_10563.	transcript	0	0.003463	0.030841	0	0.028999	0	0	0.002423	0
Novel_009975	XLOC_10563.	transcript	0.056492	0.548468	0.646792	0.94632	0.172453	0.481454	0.380184	0.091412	0.694582
Novel_009976	XLOC_10563.	transcript	0.210147	0.444655	0.206963	0.088267	0.132237	0.05865	0.460534	0.07136	0.445813
Novel_009977	XLOC_10563.	transcript	0.038192	0.037023	0.086435	0.502898	0.0194	0.530715	0.367691	0.018479	0.181765
Novel_009978	XLOC_10563.	transcript	0.306992	0.236837	0.382306	0.032315	0.037002	0	0.116718	0.436694	0.398395
Novel_009979	XLOC_10563.	transcript	0.005502	0.804474	0.079398	0	0.227989	0	0.150273	0	0.051398
Novel_009980	XLOC_10563.	transcript	0	0	0.010315	0	0	0	0.161605	0	0
Novel_009981	XLOC_10563.	transcript	0	0	0	0	0.185444	0.014647	0.096917	0	0
Novel_009982	XLOC_10563.	transcript	0	0	0	0	0	0	0	0	0

Novel_009983	XLOC_10564.	transcript	0.178963	0	0	0	0.664255	0	0	0.62874	0
Novel_009984	XLOC_10564.	transcript	0.336279	0.904039	0.610738	0.883386	0.006296	0.511139	0.012203	0.03904	0.51569
Novel_009985	XLOC_10564.	transcript	0	0.146229	0.083052	0	0	0.277418	1.02407	0	0.026435
Novel_009986	XLOC_10564.	transcript	0.468203	1.009773	0.558544	2.199518	1.167807	0.659704	1.650603	1.435246	0.795945
Novel_009987	XLOC_10568.	transcript	0.766296	1.519329	1.162108	0.760757	1.359324	0.790678	1.120796	1.254312	1.642758
Novel_009988	XLOC_10569.	transcript	6.282981	1.498519	8.115936	7.749806	7.622242	9.621225	6.243025	5.595082	10.95955
Novel_009989	XLOC_10570.	transcript	0	0	0.03197	0.634648	0.222177	0	0	0.134552	0.037135
Novel_009990	XLOC_10570.	transcript	0.219265	0.384918	0.396511	0.072869	0.394174	0.772178	0.036723	0.943124	0
Novel_009991	XLOC_10570.	transcript	0.67157	1.741745	0.759589	2.542015	1.269343	0.602539	2.103626	1.807149	2.114374
Novel_009992	XLOC_10571.	transcript	0.02727	0.169701	0.075948	0.034488	0.114963	0.067701	0.063301	0.070813	0.296205
Novel_009993	XLOC_10571.	transcript	0	0	0	0	0	0	0	0	0
Novel_009994	XLOC_10571.	transcript	0	0	0	0	0	0	0.034028	0.019127	0.081905
Novel_009995	XLOC_10572.	transcript	0	0	0	0	0.006865	0	0	0	0.05106
Novel_009996	XLOC_10573.	transcript	0	0	0	0	5.20E-05	0	0	0	0
Novel_009997	XLOC_10573.	transcript	0.048705	0.040457	0.089911	0.053235	0.055208	0.07278	0.1164	0.139203	0.005236
Novel_009998	XLOC_10573.	transcript	0	0	0.036716	0.016248	0.013278	0	0	0	0
Novel_009999	XLOC_10573.	transcript	0	0	0	0	0	0	0	0	0
Novel_010000	XLOC_10573.	transcript	0	0	0	0	0.046768	0.009648	0.088839	0	0.028695
Novel_010001	XLOC_10573.	transcript	0.013859	0	0.020495	0.096587	0.070716	0	0.000877	0.175168	0
Novel_010002	XLOC_10573.	transcript	0	0	0.118545	0.049152	0.086474	0.117775	0.038142	0	0
Novel_010003	XLOC_10573.	transcript	0.041179	0	0.012006	0.054517	0.162546	0	0.001803	0.002145	0
Novel_010004	XLOC_10573.	transcript	0.292632	0.083756	0.068019	0.065931	0.17361	0.000779	0.001652	0.037484	0.14901
Novel_010005	XLOC_10573.	transcript	0	0	0	0	0	0	0	0	0
Novel_010006	XLOC_10573.	transcript	0.033179	0	0	0	0.075413	0.143091	0.128367	0	0.093624
Novel_010007	XLOC_10573.	transcript	0.02927	0	0	0	0	0	0.754489	0.040256	0
Novel_010008	XLOC_10573.	transcript	0.405118	0.477198	0.379937	0.256397	0.367652	0.565858	0.333886	0.038369	0.252782
Novel_010009	XLOC_10573.	transcript	0	0	0	0	0	0	0	0.378598	0
Novel_010010	XLOC_10573.	transcript	0	0	0	0	0	0	0	0	0
Novel_010011	XLOC_10576.	transcript	0.170222	0.028553	0.239087	0.146436	0.212078	0.266836	0.283286	0.156899	0.224358
Novel_010012	XLOC_10576.	transcript	0.240632	0.313039	0.281427	0.114178	0.358258	0.22397	0.97963	0.231528	0.424291
Novel_010013	XLOC_10576.	transcript	0.091017	0.101024	0.525878	0.411024	0.360928	0.130653	0.378161	0.202547	0.727474
Novel_010014	XLOC_10578.	transcript	0.317977	1.996695	2.680511	3.267719	3.118516	2.21261	2.359836	3.75219	1.838052
Novel_010015	XLOC_10578.	transcript	1.009783	1.368945	0.605752	1.676582	1.247577	0.926673	1.186618	1.377269	1.025115
Novel_010016	XLOC_10578.	transcript	0	0	0	0.053453	0.018747	0.049751	0.007674	0	0
Novel_010017	XLOC_10578.	transcript	0.069364	0.030707	0.403773	0	0.004124	0.024339	0.051553	0.26368	0.177187
Novel_010018	XLOC_10580.	transcript	0.023159	0.000255	0.017551	0.138102	0.021073	0.001214	0.009257	0	0
Novel_010019	XLOC_10580.	transcript	0.002017	0.005542	0.00691	0.065102	0.00228	0	0	6.00E-05	0.001229
Novel_010020	XLOC_10580.	transcript	0.107111	0.002185	0.012541	0	0	0.019539	0	0.115113	0.124514

Novel_010021	XLOC_1058C.	transcript	0.075542	0.031291	5.00E-06	0.087578	0.239469	0.235612	0.2572	0.214704	0.192983
Novel_010022	XLOC_1058З.	transcript	0.061284	0.226194	0.064791	0.140302	0.031236	0	0.17119	0.192049	0.737675
Novel_010023	XLOC_1058З.	transcript	0	0	0	0.169187	0	0	0	0	0
Novel_010024	XLOC_10591.	transcript	0.354804	0.218705	0.718307	0.586754	0.668427	0.52528	0.519763	0.430405	0.600038
Novel_010025	XLOC_10592.	transcript	0.584149	0.609104	0	0.391667	0	0	0	0.679049	0.333029
Novel_010026	XLOC_10592.	transcript	0.364766	0.291131	0.136447	0.075407	0.068974	0.530731	0.343287	0.08105	0.16375
Novel_010027	XLOC_10592.	transcript	0.08916	0.450389	1.020248	0.157625	1.196931	0.165768	1.305644	0.261121	0.168881
Novel_010028	XLOC_10592.	transcript	0.003052	0.576921	0	0	0.002963	0	0.616864	0	0.005697
Novel_010029	XLOC_10592.	transcript	1.275893	0.987963	1.692786	1.04074	2.183434	2.1946	1.018229	1.865878	1.823977
Novel_010030	XLOC_10592.	transcript	0	0.432896	0.900799	0	0.593691	1.570487	0.406039	0.369898	0.660038
Novel_010031	XLOC_10592.	transcript	0.003052	0	0.491149	1.522	0	0	0.054234	0.026645	0
Novel_010032	XLOC_10594.	transcript	0.152814	0.781688	0.078699	0.088302	0.143647	0.185166	0.125991	0.116676	0.271045
Novel_010033	XLOC_10594.	transcript	0.018115	1.415454	0.550728	1.892908	2.177178	1.800593	0.566262	1.967869	2.219596
Novel_010034	XLOC_10594.	transcript	0.028275	0.3036	0.039533	0	0.01474	0	0.326529	0	0.124058
Novel_010035	XLOC_10594.	transcript	0.076213	0	0.60595	0.156398	0.14235	0.234727	0	0.117243	0
Novel_010036	XLOC_10594.	transcript	1.231829	0	0.827146	6.10E-05	0.000184	0	1.376899	0	0
Novel_010037	XLOC_1060C.	transcript	0.016637	0.338107	0.046905	0.019821	0	0.048511	0.051929	0.03026	0.019717
Novel_010038	XLOC_1060C.	transcript	0	0.141572	0	0.041932	0.059273	0	0.013003	0	0
Novel_010039	XLOC_10607.	transcript	0.016122	0.533992	0.168533	0.156121	0.186175	0.143113	0.162535	0.049404	0.024858
Novel_010040	XLOC_10607.	transcript	0.108813	0	0.062149	0.017904	0.059962	0.146443	0.085692	0.33287	0.027867
Novel_010041	XLOC_10607.	transcript	0	0.415683	0	0	0	0	0	0.11699	0
Novel_010042	XLOC_1060Ɛ.	transcript	0	0	0	0	0	0	0.044031	0.246029	0
Novel_010043	XLOC_10612.	transcript	0.178249	0.076075	0	0.038862	0.154922	0.018648	0.026095	0.045682	0.033223
Novel_010044	XLOC_10612.	transcript	0	0.311912	0.100758	0	0	0	0.114665	0	0.108433
Novel_010045	XLOC_10612.	transcript	0.138807	0.694015	0.340583	0.28608	0.351827	0.258139	0.303038	0.315109	0.315837
Novel_010046	XLOC_10614.	transcript	0.026964	0	0	0	0.321971	3.00E-05	0.32266	0.010889	0.009964
Novel_010047	XLOC_10614.	transcript	0.237868	1.844046	0.333821	0.115061	0.152468	0.520807	0.246228	0.235009	1.004149
Novel_010048	XLOC_10614.	transcript	0.184997	0.247295	0.239879	0.336454	0.298142	0.303703	0.43553	0.281288	0.241499
Novel_010049	XLOC_1061€.	transcript	0	0	0	0.264544	0.32411	0.096389	0.319236	0	0
Novel_010050	XLOC_10617.	transcript	0.033355	0.934301	0.020271	0	0	0.022339	0	0.016959	0
Novel_010051	XLOC_10617.	transcript	0	0.900605	0	0	0	0	0.602382	0	0
Novel_010052	XLOC_1062C.	transcript	0	0.047402	0.078187	0.016212	0.148294	0.173556	0.029957	0.090597	0.080009
Novel_010053	XLOC_1062C.	transcript	0.171368	0.034095	0	0.020286	0	0	0	0	0
Novel_010054	XLOC_1062C.	transcript	0	0	0.051974	0.051369	0	0.041478	0.071582	0.051685	0.090399
Novel_010055	XLOC_1062C.	transcript	0	0.041878	0	0.045261	0.079582	0.005071	0.040621	0.001788	0.308072
Novel_010056	XLOC_1062C.	transcript	0	0	0.00491	0	0.053382	0.004783	0	0.208549	0
Novel_010057	XLOC_1062C.	transcript	0.111445	0.023702	0.17443	0.136669	0.146475	0.140436	0.102405	0.011229	0.168349
Novel_010058	XLOC_1062Ƽ.	transcript	0.337772	0.462221	0.840341	0.914078	0.647903	0.687679	0.71357	1.014558	0.595011

Novel_010059	XLOC_10626.	transcript	0	0	0.015392	0.009698	0	0.355758	0.000312	0	0.43712
Novel_010060	XLOC_10626.	transcript	0.033716	0	0.220221	0.374367	0.241008	0.079157	0.144435	0.26169	0
Novel_010061	XLOC_10626.	transcript	0	0.212434	0	0.076632	0.127964	0	0	0.302976	0
Novel_010062	XLOC_10626.	transcript	0.113733	0	0.351581	0.293635	0.15075	0.392754	0.230787	0.159756	0.339349
Novel_010063	XLOC_10627.	transcript	0.26387	0.088809	0.101286	0.184438	0.274342	0.29255	0.250392	0.141552	0.075053
Novel_010064	XLOC_10627.	transcript	36.99582	0	0	4.925427	47.38917	3.040895	38.98023	25.85379	63.35284
Novel_010065	XLOC_10627.	transcript	0.030208	0.111161	0.033548	0.031408	0.089169	0.021159	0.124525	0.150751	0.118774
Novel_010066	XLOC_10627.	transcript	0.254207	0.426293	0.250166	0.377402	0.059802	0.277141	0.426974	0.232766	0.088058
Novel_010067	XLOC_10627.	transcript	0.570802	0.945083	0.441562	0.138146	0.406032	0.494687	0.7881	0.426785	0.522361
Novel_010068	XLOC_10628.	transcript	0	0.163559	0.373537	0	0	0.602771	0.00234	0	0.365107
Novel_010069	XLOC_10628.	transcript	0.209089	0.107276	0.35433	0.537381	0.157426	0.396762	0.832656	0.563379	0.189516
Novel_010070	XLOC_10628.	transcript	0.228895	0.119311	0.342573	0.139611	0	0.458288	0.599752	0.450599	0
Novel_010071	XLOC_10628.	transcript	0.151664	0.094217	0.115402	0.162643	0.124556	0.121871	0.127322	0.172484	0.111439
Novel_010072	XLOC_10628.	transcript	0.284888	0.415393	0.282435	0.337492	0.325724	0.174813	0.284799	0.373625	0.291868
Novel_010073	XLOC_10628.	transcript	0.217305	0.13424	0.149255	0.186746	0.261234	0.131996	0.194087	0.166659	0.372677
Novel_010074	XLOC_10628.	transcript	0.171642	0.105522	0.116274	0.128632	0.239027	0.144037	0.133105	0.160051	0.212761
Novel_010075	XLOC_10628.	transcript	0.177998	0.166786	0.118183	0.134866	0.826474	0.143261	0.137454	0.170641	0.30922
Novel_010076	XLOC_10628.	transcript	0.23344	0.153803	0.155079	0.202631	0.239733	0.166281	0.179845	0.410526	0.294037
Novel_010077	XLOC_10628.	transcript	0	0	0	0	0	0	0	0	0
Novel_010078	XLOC_10628.	transcript	0.212634	0.142117	0.152615	0.181663	0.205595	0.156241	0.16386	0.37774	0.278917
Novel_010079	XLOC_10628.	transcript	0.014301	0.231422	0.190526	0.266967	0.176751	0.342669	0.193184	0	0.412658
Novel_010080	XLOC_10638.	transcript	0	0	0	0.078636	0	0.002516	0.027327	0	0
Novel_010081	XLOC_10638.	transcript	0.046847	0.037048	0.4119	0.060533	0.023957	0.051282	0.092075	0.049511	0.151938
Novel_010082	XLOC_10638.	transcript	0.665752	0.293071	0.489692	0.876323	1.251697	0.898509	0.891083	0.702969	0.53312
Novel_010083	XLOC_10639.	transcript	0.457944	0.259108	0.280119	0.200062	0.236903	0.202172	0.546587	0.478691	0.292161
Novel_010084	XLOC_10641.	transcript	0	0	0.227991	0	0.075788	0	0	0	0
Novel_010085	XLOC_10641.	transcript	1.023722	2.09181	0.686986	1.301546	1.162096	1.099362	1.230703	0.816039	1.452541
Novel_010086	XLOC_10643.	transcript	0.935426	0.690236	0.660613	0.809186	0.571809	0.489609	1.345555	0.702953	0.738635
Novel_010087	XLOC_10643.	transcript	0.336126	0.36941	0.301275	0.060187	0.094291	0.080466	0.049181	0.111608	0.069637
Novel_010088	XLOC_10644.	transcript	0.331872	0.818292	0.217239	0.204708	0.17305	0.229271	0.518456	0.244508	0.208322
Novel_010089	XLOC_10647.	transcript	0.208779	0.005485	0.27697	0.151647	0.02194	0.248601	0.604317	0.234675	0.262204
Novel_010090	XLOC_10647.	transcript	0	0.010898	0	0.068286	0	0.034004	0.055077	0.024858	0.030545
Novel_010091	XLOC_10647.	transcript	0.326346	0.230438	0.278586	0.386639	0.662265	0.437279	0.408764	0.401495	0.412621
Novel_010092	XLOC_10647.	transcript	0.186537	0.366123	0.111674	0.131542	0.137676	0.047457	0.242716	0.26784	0.245549
Novel_010093	XLOC_10647.	transcript	0	0	0.432658	0	0.030702	0	0	0	0
Novel_010094	XLOC_10647.	transcript	0	0	0	0	0	0	0	0	0
Novel_010095	XLOC_10647.	transcript	0.230457	0.119997	0.267917	0.463487	0.310209	0.357626	0.502116	0.605231	0.454896
Novel_010096	XLOC_10654.	transcript	0.087627	0.184209	0.04955	0	0.015979	0	0.023407	0.026285	0

Novel_010097	XLOC_10654.	transcript	0.185733	0	0.490898	0.324214	0.252864	0	0.324119	0.194958	0.072638
Novel_010098	XLOC_10654.	transcript	0.1434	0	0	0	0	0	0.116125	0.205944	0
Novel_010099	XLOC_10658.	transcript	0.25024	0.438199	0.220231	0.085394	0.174898	0.212263	0.208987	0.197355	0.109017
Novel_010100	XLOC_10658.	transcript	0	0.049391	0	0	0.170471	0.006999	0.060327	0	0.022776
Novel_010101	XLOC_10659.	transcript	0.510694	0.768238	0.596326	0.866878	0.890156	0.910238	1.027308	0.86024	0.65344
Novel_010102	XLOC_10660.	transcript	0	0	0.30168	0	0	0	0.448406	0	0
Novel_010103	XLOC_10660.	transcript	0	0.031304	0	0.007693	0	0	0	0.025389	0
Novel_010104	XLOC_10660.	transcript	0.585148	1.131026	0.36327	0.51897	0.450046	0.506514	0.370192	0.502532	0.38916
Novel_010105	XLOC_10660.	transcript	0.007704	0.014363	0.024446	0	0	0	0	0.062413	0
Novel_010106	XLOC_10660.	transcript	0.025997	0.041434	0	0.018767	0.023698	0.024736	0.220981	0.02891	0
Novel_010107	XLOC_10660.	transcript	0	0	0	0	0.005161	0.004611	0.090673	0	0
Novel_010108	XLOC_10660.	transcript	0	0.13274	0	0.019533	0.010783	0.007638	0	0.073101	0
Novel_010109	XLOC_10660.	transcript	0	0.02855	0	0	0	0	0.003037	0.026798	0.008226
Novel_010110	XLOC_10660.	transcript	0	0	0	0.005351	0	0	0	0	0
Novel_010111	XLOC_10660.	transcript	0	0	0.019837	0.015264	0	0	0.015946	0	0
Novel_010112	XLOC_10660.	transcript	1.214548	0.5043	1.647259	1.766897	1.404207	2.14093	1.89037	2.138754	1.11548
Novel_010113	XLOC_10660.	transcript	2.047018	0	0	0	0	0.485108	2.909323	4.109696	0
Novel_010114	XLOC_10660.	transcript	0.720239	0	0	0	0	1.309563	1.228644	0	0
Novel_010115	XLOC_10660.	transcript	0.196088	0	2.062117	0	3.61168	0.319672	0.36468	0	0
Novel_010116	XLOC_10660.	transcript	0.191561	0.055216	0.726478	0.583689	0	0.366835	0.366751	0.202505	1.732854
Novel_010117	XLOC_10660.	transcript	0.194747	0	0	0	0	0.347485	0.369801	0.213823	0
Novel_010118	XLOC_10660.	transcript	0	0	0.011803	0.658285	0.028403	0	0	0	0.073161
Novel_010119	XLOC_10660.	transcript	0.072113	0.207007	0.132904	0.838414	0.196581	0.04483	0.14314	0.132811	0.132041
Novel_010120	XLOC_10660.	transcript	3.083968	0	0	0	0	1.271002	1.169693	1.044702	0
Novel_010121	XLOC_10660.	transcript	0.233932	0	0	0	0	0.382517	0.342739	0.241114	0
Novel_010122	XLOC_10660.	transcript	0	0	1.0947	0	0	0	0	0	0
Novel_010123	XLOC_10660.	transcript	0	0	0	0.83704	0	0	0	0	0
Novel_010124	XLOC_10660.	transcript	0	0	0	0.750847	0	0	0	0	0
Novel_010125	XLOC_10661.	transcript	0.028163	0.366039	0.058339	0.146746	0.098052	0	0.096935	0.165651	0.138838
Novel_010126	XLOC_10661.	transcript	0.147933	0.34613	0.169196	0	0	0.171464	0.174221	0.028898	0.179323
Novel_010127	XLOC_10667.	transcript	0	0	0.232287	0.038504	0.100447	0.055431	0.2433	0.2335	0.054447
Novel_010128	XLOC_10668.	transcript	0.12606	0.453823	0.175927	0.275845	0.284263	0.314566	0.196515	0.337412	0.290109
Novel_010129	XLOC_10668.	transcript	0.81431	1.063011	0.46314	0.546315	0.493721	0.450753	0.706318	0.726125	0.390556
Novel_010130	XLOC_10668.	transcript	0.033044	0.30125	0.154759	0.079759	0	0	0.024713	0.075676	0.227488
Novel_010131	XLOC_10670.	transcript	0	0	0	0.165439	0	0	0	0	0
Novel_010132	XLOC_10670.	transcript	0	0	0	0	0	0	0	0	0
Novel_010133	XLOC_10670.	transcript	0.255588	0.07627	0.34964	0.553112	0.408058	0.304676	0.080541	0.449666	0.261275
Novel_010134	XLOC_10676.	transcript	0.266903	0.144215	0.154947	0.134423	0.343791	0.306685	0.157964	0.244487	0.011416

Novel_010135	XLOC_10676.	transcript	0.271134	0.746391	0.327359	0.157099	0.161089	0.004635	0.439153	0.27702	0.415974
Novel_010136	XLOC_10677.	transcript	0.357641	0.418022	0.803157	0.428991	0.726518	0.715889	0.603589	0.536737	0.433598
Novel_010137	XLOC_10681.	transcript	0.440478	0.011623	0.197383	0.158235	0.494431	0.283371	0.214624	0.33151	0.194245
Novel_010138	XLOC_10696.	transcript	0.073792	0.090517	0.284328	0.156433	0.231003	0.165207	0	0.058822	0
Novel_010139	XLOC_10718.	transcript	0	1.426121	0	0	0	0	0	0	0
Novel_010140	XLOC_10720.	transcript	0	0.104132	0	0	0	0.240941	0	0.195326	0
Novel_010141	XLOC_10720.	transcript	1.056766	0.930362	0.124141	0.08248	0.857475	0.80985	0.699624	0.639924	0.867818
Novel_010142	XLOC_10720.	transcript	0.000473	0.365419	0.663417	0.685127	0.002543	0.00059	0.502649	0.301416	0
Novel_010143	XLOC_10722.	transcript	2.026313	0.88016	1.357825	1.106733	1.455184	1.171236	1.868677	1.617585	1.036044
Novel_010144	XLOC_10730.	transcript	0.219322	0.137825	0.172393	0.227262	0.104795	0.198314	0.2109	0.234951	0.085206
Novel_010145	XLOC_10730.	transcript	0	0	0	0	0	0	0	0	0
Novel_010146	XLOC_10731.	transcript	0	0.040696	0.147084	0.144037	0.150665	0.192686	0.475354	0.066137	0.048494
Novel_010147	XLOC_10735.	transcript	0.039437	0.006537	0.024123	0.019831	0.068543	0.009516	0.005899	0.004516	0.018165
Novel_010148	XLOC_10739.	transcript	0	0	0	0	0	0	0	0	0
Novel_010149	XLOC_10739.	transcript	0.434101	0.435215	0.40193	0.209526	0.191789	0.262652	0.477154	0.319447	0.325946
Novel_010150	XLOC_10740.	transcript	0.74376	0.175793	0.629247	0	0.622734	0.547335	0.249422	0.902212	0.865426
Novel_010151	XLOC_10740.	transcript	0.323715	1.061896	0.329062	0.305035	0.563788	0.26276	0.956125	0.681538	0.440907
Novel_010152	XLOC_10740.	transcript	0	0	0	0.884917	0	0	0	0	0
Novel_010153	XLOC_10745.	transcript	0.435422	0.962448	0.789923	0.78118	0.57803	0.769761	0.351054	0.620269	0.777508
Novel_010154	XLOC_10752.	transcript	0	1.262984	0.042247	0.202381	0.188825	0	0.236924	0.313271	0
Novel_010155	XLOC_10752.	transcript	0.180862	0.024996	0.250719	0.057428	0.048314	0.226497	0.076094	0.013814	0.344649
Novel_010156	XLOC_10752.	transcript	0	0.090427	0	0.007896	0.059623	0.031065	0	0.079839	0.006849
Novel_010157	XLOC_10754.	transcript	0.125992	0.369098	0.318809	0.050216	0.266922	0.135964	0.556394	0.029159	0.097287
Novel_010158	XLOC_10754.	transcript	0.360144	0.272533	0.6984	0.557691	0.56733	0.971712	0.434712	0.497501	0.375866
Novel_010159	XLOC_10755.	transcript	1.037292	0.72041	0.713196	0.745337	0.499118	0.261399	0.964435	0.405447	0.197824
Novel_010160	XLOC_10755.	transcript	1.330793	1.383696	1.160598	0.894707	1.250782	1.774635	1.533828	1.668985	1.3593
Novel_010161	XLOC_10756.	transcript	0	0	0	0	0	0	0	0	0
Novel_010162	XLOC_10756.	transcript	5.154048	5.887592	5.96022	9.811757	8.11107	8.021099	10.02405	7.573988	9.209615
Novel_010163	XLOC_10756.	transcript	0	0	2.093696	0	0.007587	1.26479	0	0.003676	0.440801
Novel_010164	XLOC_10760.	transcript	0.286	0.984454	0.143162	0.280944	0.508055	0.351458	0.240505	0.405745	0.343997
Novel_010165	XLOC_10760.	transcript	0.193652	0	0.053534	0.120498	0.099538	0.170454	0	0.193343	0.155217
Novel_010166	XLOC_10760.	transcript	0.253042	0.407404	0.223724	0.202246	0.159345	0.154913	0.332261	0.260949	0.199547
Novel_010167	XLOC_10760.	transcript	0	0	0	0	0	0	0	0.012556	0
Novel_010168	XLOC_10764.	transcript	0	0	0.199856	0	0	0	0.283078	0	0
Novel_010169	XLOC_10783.	transcript	5.62009	3.564575	4.529321	5.273815	5.969435	4.369061	8.887147	7.551282	4.046679
Novel_010170	XLOC_10785.	transcript	0.744357	0.434983	0.798666	1.019586	0.868684	0.735587	0.875441	0.566011	0.513507
Novel_010171	XLOC_10786.	transcript	0	0	0	0	0	3.315958	0	0	0
Novel_010172	XLOC_10786.	transcript	0	0	0.033018	0	0	19.53166	0	0	0

Novel_010173	XLOC_1078€.	transcript	0	0	0	0	22.53112	0	15.62639	0	0
Novel_010174	XLOC_1078€.	transcript	58.63685	4.687274	2.220994	0.34178	32.29029	21.92471	36.63957	3.011194	6.276109
Novel_010175	XLOC_1078€.	transcript	0	0	0	0	0	0	0	0.003099	0
Novel_010176	XLOC_1078€.	transcript	0	0	0	0	0	0	0	0	0
Novel_010177	XLOC_1078€.	transcript	1.039558	0	0	0	0	0	2.602261	0	0
Novel_010178	XLOC_1078€.	transcript	0	0	0	0.001064	0	0	0	0	0
Novel_010179	XLOC_1078€.	transcript	0	0	0	0	0	0	0	0.008428	0
Novel_010180	XLOC_1078€.	transcript	0	0	0	0	0	0	0	0	0
Novel_010181	XLOC_1078€.	transcript	0	0	0	0	0	0	0	0	0
Novel_010182	XLOC_1078€.	transcript	0	0	0	0	41.78167	0	0	0	0
Novel_010183	XLOC_1078€.	transcript	0	0	0	0	0	0	0	0	0
Novel_010184	XLOC_1078€.	transcript	10.71078	4.781987	0.035244	2.638246	0	12.38642	0	0	13.92743
Novel_010185	XLOC_1078€.	transcript	0	0.291215	0	0	0	0	0	0	0
Novel_010186	XLOC_1078€.	transcript	0	0	0	0.005804	0	0.074174	0	0.001437	0
Novel_010187	XLOC_1078€.	transcript	0	0	6.797405	0.006462	0	0	0	0	0
Novel_010188	XLOC_1078€.	transcript	0	0	2.734801	0	0	0	0	0	1.218416
Novel_010189	XLOC_1078€.	transcript	3.611616	0	0	13.46601	0	0	11.5236	0	0
Novel_010190	XLOC_1078€.	transcript	0	0	0	0	0	0	0	0	0
Novel_010191	XLOC_1078€.	transcript	0	0	0	0	0	0	0	0	0
Novel_010192	XLOC_1078€.	transcript	0	0	0	0.056694	0	0	0	1.704482	0
Novel_010193	XLOC_1078€.	transcript	0	0.942667	0	0	0	0	0	0	0
Novel_010194	XLOC_1078€.	transcript	0	0	0	0	0	0	0	0	0
Novel_010195	XLOC_1078€.	transcript	0	0	0.502825	0	6.199302	0	0	0.488684	0
Novel_010196	XLOC_1078€.	transcript	0	0.026176	0	0	0	0.171377	0	0.718126	0
Novel_010197	XLOC_1078€.	transcript	0	0	0	0.002821	0	0	0	0	0
Novel_010198	XLOC_1078€.	transcript	0	0	0	0.048754	0	2.572887	0	0	0
Novel_010199	XLOC_1078€.	transcript	0.001343	0	0	0	0	0	2.781728	0	0
Novel_010200	XLOC_1079C.	transcript	0.188601	0.325323	0.15786	0.368459	0.232449	0.179991	0.238448	0.213766	0.144599
Novel_010201	XLOC_1081€.	transcript	0	0.071987	0.002013	0.020917	0.018502	0.048434	0.193286	0.024787	0.016967
Novel_010202	XLOC_1081€.	transcript	0.493163	0.693757	0.419478	0.226579	0.292628	0.408683	0.20454	0.323137	0.222711
Novel_010203	XLOC_1081€.	transcript	0.29313	0.191522	0.141339	0.115968	0.081316	0.107854	0.224788	0.094022	0.143891
Novel_010204	XLOC_1081٤.	transcript	0.053265	0.178262	0.159551	0.014881	0.016015	0.08708	0.088902	0.121918	0
Novel_010205	XLOC_1082C.	transcript	0.228895	1.329931	0.12843	0.159597	0.149644	0.112927	0.303917	0.180577	0.239894
Novel_010206	XLOC_1082C.	transcript	0.024791	0	0.1162	0	0.134589	0.084597	0.08826	0.143286	0
Novel_010207	XLOC_10821.	transcript	0	0	0	0	0	0.053002	0	0	0
Novel_010208	XLOC_1082Ξ.	transcript	1.395195	0.250471	0.955182	1.45986	1.200183	1.797307	1.489467	2.290741	0.78791
Novel_010209	XLOC_1082Ξ.	transcript	0.048755	0	0	0	0.324801	0	0.015347	0	0
Novel_010210	XLOC_1082٤.	transcript	0	0	0.115835	0.020338	0.049805	0.029181	0	0.062778	0

Novel_010211	XLOC_10831.	transcript	0.176176	0.293205	0.402889	0.310522	0.218288	0.152183	0.393503	0.328649	0.177456
Novel_010212	XLOC_10831.	transcript	0	0.024193	0.072843	0.058864	0	0	0	0	0.115378
Novel_010213	XLOC_10831.	transcript	0	0.066971	0	0	0	0.077191	0	0	0
Novel_010214	XLOC_10831.	transcript	0.307435	0.290974	0.20123	0.185082	0.277676	0.153292	0.263513	0.235448	0.15775
Novel_010215	XLOC_10831.	transcript	0.012068	0.096067	0.041746	0.035029	0.073406	0.109023	0.072317	0.05277	0.041186
Novel_010216	XLOC_10831.	transcript	0	0.706403	0.192587	0.14263	0.431308	0.909014	0.622485	0.223797	0.414214
Novel_010217	XLOC_10831.	transcript	0.75436	1.521409	0.754059	0.650436	0.577737	0	0.539064	0.712805	0.594538
Novel_010218	XLOC_10831.	transcript	0	0	0	0.488513	0	0	0	0	0
Novel_010219	XLOC_10831.	transcript	0	0	0	0	0.006528	0	0	0	0
Novel_010220	XLOC_10832.	transcript	0	0	0	0	0	0.015988	0	0	0.277376
Novel_010221	XLOC_10832.	transcript	0	0	0	0	0	0	0	0	0
Novel_010222	XLOC_10832.	transcript	0.025498	0.053496	4.680269	4.257897	2.591833	4.055524	0.03318	0.003372	7.863866
Novel_010223	XLOC_10833.	transcript	0.070377	1.050707	0.096202	0.143412	0.07592	0.134635	0.210156	0.061541	0.086112
Novel_010224	XLOC_10836.	transcript	0.6147	1.716835	0.497322	0.379283	0.509782	0.563464	0.611828	0.643036	0.34155
Novel_010225	XLOC_10836.	transcript	0	0	0	0	0.005336	0	0	0.002121	0.051494
Novel_010226	XLOC_10837.	transcript	0.422076	1.032539	0.304381	0.549931	0.785134	0.585557	0.342498	0.513168	0.654637
Novel_010227	XLOC_10838.	transcript	0	0.133063	0	0	0	0	0	0.285961	0
Novel_010228	XLOC_10839.	transcript	0.291955	4.122281	0.070601	0.142138	0.172569	0.184832	0.676077	0.179107	0.218379
Novel_010229	XLOC_10839.	transcript	0.106025	0.212863	0.288728	0.074463	0.219851	0.267365	0.619493	0.115979	0.337664
Novel_010230	XLOC_10839.	transcript	0.234555	0.511239	0.339797	0.085253	0.090066	0.218429	0.300851	0.148372	0.133442
Novel_010231	XLOC_10840.	transcript	0	0.020486	0.012667	0.020072	0.010937	0.639557	0.012894	0.114773	0.018578
Novel_010232	XLOC_10840.	transcript	0.483469	0.599004	0.141009	0.340736	0.192267	0	0.295067	0.593287	0.641929
Novel_010233	XLOC_10841.	transcript	0	0	0.006862	0	0.001081	0.011759	0.035765	0	0
Novel_010234	XLOC_10841.	transcript	0.276163	0.428147	0.375537	0.308736	0.368708	0.349711	0.354452	0.413146	0.254922
Novel_010235	XLOC_10842.	transcript	0.273119	0.415373	0.346621	0.40466	0.324016	0.245446	0.487722	0.477549	0.482635
Novel_010236	XLOC_10845.	transcript	0.689801	0.567789	0.604167	0.716887	0.872001	0.611306	1.130888	0.883998	0.524818
Novel_010237	XLOC_10847.	transcript	0.287044	0.149043	0.493878	0.405337	0.441161	0.397702	0.387264	0.58369	2.438113
Novel_010238	XLOC_10847.	transcript	0.073131	0.017006	0.213104	0.035866	0.04419	0.077845	0.153018	0.051847	0.491178
Novel_010239	XLOC_10847.	transcript	0	0.03701	0.019953	0.034588	0.04126	0.074861	0.161196	0.016427	0.27428
Novel_010240	XLOC_10847.	transcript	0	0.400911	0.090814	0.129999	0.163251	0.125969	0.511567	0.156157	0.565593
Novel_010241	XLOC_10850.	transcript	1.901959	4.689718	3.434339	3.667315	3.152902	3.618178	3.539602	3.754021	2.728737
Novel_010242	XLOC_10855.	transcript	2.991932	2.118325	0.123615	1.268163	0.935262	1.147794	1.853935	2.331319	1.308508
Novel_010243	XLOC_10860.	transcript	0	1.68897	0.638137	0.402631	0	0	0	0.465297	0.422495
Novel_010244	XLOC_10860.	transcript	0.191082	0.83976	0.327398	0.18225	0.155782	0.238066	0.321429	0.23916	0.319712
Novel_010245	XLOC_10867.	transcript	0.475491	1.667133	0.968667	0.890741	1.0524	1.056774	0.832618	1.1508	1.19065
Novel_010246	XLOC_10868.	transcript	0.546455	0.151628	0.323894	0.549286	0.379284	0.429526	0.584395	0.541671	0.322245
Novel_010247	XLOC_10871.	transcript	0.702285	0.593028	0.515101	0.478729	0.482258	0.524884	0.62755	0.692251	0.880322
Novel_010248	XLOC_10888.	transcript	0.238952	0.029819	0.207027	0.131811	0.133356	0.142287	0.237756	0.171496	0.116967

Novel_010249	XLOC_10889.	transcript	0.453638	0.912965	0.321433	0.347938	0.434311	0.454959	0.450502	0.501816	0.488951
Novel_010250	XLOC_10890.	transcript	0.025447	0	0	0	0	0.045247	0.086714	0.045868	0
Novel_010251	XLOC_10894.	transcript	0.150067	0.579703	0.077402	0.046531	0.065206	0.058849	0.205576	0.049412	0.03559
Novel_010252	XLOC_10898.	transcript	0.39118	0	0.91552	0.303568	0.215739	0	0	0	0.630474
Novel_010253	XLOC_10898.	transcript	0	0	0	0	0.516682	0	0	0	0
Novel_010254	XLOC_10898.	transcript	0	0	0	0.225451	0	0.350349	0.721138	0.000985	0
Novel_010255	XLOC_10898.	transcript	0	0	0	0.108034	0	0.53771	0	0	0
Novel_010256	XLOC_10898.	transcript	0.28299	0	0.349203	0.25787	0.234549	0.021345	0	0.005847	0.389261
Novel_010257	XLOC_10898.	transcript	0.144607	0.160547	0.105153	0.01522	0.034202	0.007645	0.291465	0.6299	0.129472
Novel_010258	XLOC_10898.	transcript	0.003438	0	0.006452	0.022995	9.00E-04	0.025221	0.525292	0.110034	0.012594
Novel_010259	XLOC_10899.	transcript	1.058004	0.844519	0.876085	0.698827	0.950494	0.825615	1.14143	1.130534	0.882585
Novel_010260	XLOC_10906.	transcript	0	0.226555	0	0	0.141358	0.069288	0.128849	0.193992	0.068059
Novel_010261	XLOC_10910.	transcript	0	0	0	0	0.334656	0.315451	0.437789	0	0
Novel_010262	XLOC_10912.	transcript	0.571785	0.23777	0.478333	0.053874	0.285447	0.463404	0.663194	0.158919	0.203584
Novel_010263	XLOC_10912.	transcript	0.195272	0.575241	0	0.425996	0	0.446281	0.207299	0.083061	0
Novel_010264	XLOC_10912.	transcript	0.195307	1.457927	0.195701	0.13316	0.179514	0.096502	0.237432	0.160796	0.43235
Novel_010265	XLOC_10917.	transcript	0.011588	0	0.047955	0.102936	0.029569	0.223868	0.224522	0.038966	0.062082
Novel_010266	XLOC_10917.	transcript	1.71982	0.271315	1.566759	1.546805	1.464926	1.285083	1.349493	1.618571	1.924761
Novel_010267	XLOC_10917.	transcript	0.211663	0.231475	0.299452	0.168265	0.31617	0.275538	0.523583	0.677002	0.852119
Novel_010268	XLOC_10917.	transcript	0.264327	0.198905	0.449396	0.065544	0.391043	0.205219	0.532754	0.225586	0.192002
Novel_010269	XLOC_10917.	transcript	0.371505	0.164266	0.537769	0.183459	0.316424	0.260645	0.146621	0.295123	0.654986
Novel_010270	XLOC_10917.	transcript	0.038579	0.116091	0	0.238583	0.03756	0.453134	0.18037	0.275187	0
Novel_010271	XLOC_10917.	transcript	0.28782	0	0	0	0	0	0	0	0
Novel_010272	XLOC_10917.	transcript	0	0.28047	0	0	0	0	0	0	0
Novel_010273	XLOC_10917.	transcript	0.414004	0.217788	0.349437	0.430353	0.347881	0.628807	0.73703	0.443607	0.114369
Novel_010274	XLOC_10917.	transcript	0.212312	0	0.179533	0.140827	0.704588	0	0.416291	0.237138	0
Novel_010275	XLOC_10917.	transcript	0	0.061472	0.247467	0	0.140847	0	0.01662	0.017191	0.031589
Novel_010276	XLOC_10917.	transcript	0.337925	0.329725	0.268974	0.320918	0.138748	0.39541	0.166402	0.253199	0.313825
Novel_010277	XLOC_10917.	transcript	0.20146	0.236748	0.03011	0.064262	0.145989	0.022393	0.151385	0.096627	0.036471
Novel_010278	XLOC_10924.	transcript	0.165073	0.012161	0.291224	0.128271	0.278418	0.13899	0.446029	0.4437	0.678143
Novel_010279	XLOC_10924.	transcript	0	0	0	0	0	0	0	0	0
Novel_010280	XLOC_10934.	transcript	0.262491	0.512327	0.267028	0.332997	0.222026	0.249133	0.367271	0.322118	0.305493
Novel_010281	XLOC_10936.	transcript	0.283134	0.18469	0.000155	0.110122	0.000737	0.192784	0.089722	0.254659	0.149382
Novel_010282	XLOC_10936.	transcript	0.130544	0	0.408052	0.177348	0.370604	0.341107	0.26096	0.314552	0.182523
Novel_010283	XLOC_10937.	transcript	0.061895	0.045207	0.056621	0.124632	0.095013	0.185048	0.103918	0.103556	0.081751
Novel_010284	XLOC_10942.	transcript	3.035773	1.803955	2.888794	3.418543	3.562948	3.801117	4.06944	4.480406	3.892599
Novel_010285	XLOC_10943.	transcript	0.348751	0	0	0.234131	0.465607	0.404502	0.408018	0.029908	0.325377
Novel_010286	XLOC_10943.	transcript	0.093286	0.082958	0.265661	0.103956	0.152783	0.119808	0.295568	0.379576	0.103586

Novel_010287	XLOC_10944.	transcript	0.298907	0.277676	0.343142	0.021176	0.277738	0.235344	0.070349	0.343976	0
Novel_010288	XLOC_10944.	transcript	0	0.328856	0	0.277238	0.011997	0	0.29896	0.021773	0.223493
Novel_010289	XLOC_10946.	transcript	0.611374	0.441756	0.765674	0.726124	0.753527	0.774339	1.274072	0.686073	0.73572
Novel_010290	XLOC_10946.	transcript	0	0	0	0	0	0	0.163745	0.015789	0.021654
Novel_010291	XLOC_10947.	transcript	0.181861	0.129799	0.333854	0.273274	0.296945	0.384059	0.280543	0.352583	0.165824
Novel_010292	XLOC_10947.	transcript	0.328349	0.258991	0.529735	0.359909	0.411458	0.327074	0.590261	0.397478	0.491388
Novel_010293	XLOC_10947.	transcript	0.084797	0.921848	0.131309	0.120745	0.105045	0.085957	0.298913	0.070612	0.151853
Novel_010294	XLOC_10949.	transcript	0.409359	0.029103	0	0	0	0	0.210079	0.01459	0
Novel_010295	XLOC_10949.	transcript	0	0	0	0.0041	0	0	0.169452	0	0
Novel_010296	XLOC_10949.	transcript	0	0	0	0	0	0	0	0	0
Novel_010297	XLOC_10949.	transcript	0.040774	0.554653	0.296989	0.288011	0.286668	0.359077	0.016042	0.45485	0.217834
Novel_010298	XLOC_10949.	transcript	0	0	0	0	0	0.003125	0	0.002657	0
Novel_010299	XLOC_10955.	transcript	0	0	0	0	0.143122	0	0	0.160563	0
Novel_010300	XLOC_10955.	transcript	0.164648	0.216304	0.243009	0.177474	0.04251	0	0.139121	0.122728	0.317938
Novel_010301	XLOC_10955.	transcript	0	0	0	0	0	0.163966	0	0	0
Novel_010302	XLOC_10955.	transcript	0	0	0	0	0	0	0	0	0
Novel_010303	XLOC_10956.	transcript	0	0.085268	0.005379	0.00141	0.17713	0.708834	0.039445	0.00989	0.442111
Novel_010304	XLOC_10956.	transcript	0.500477	0.031583	1.099499	0.409232	0.468497	0	0.561945	0.353816	0.551996
Novel_010305	XLOC_10958.	transcript	0.085117	0.026775	0.048367	0.093098	0.024617	0.087702	0.026617	0.073573	0.029226
Novel_010306	XLOC_10959.	transcript	0.135389	0.04902	0.117752	0.060718	0.122089	0.121604	0.144804	0.695986	0.116123
Novel_010307	XLOC_10959.	transcript	0	0	0	0	0.231591	0	0	0	0
Novel_010308	XLOC_10959.	transcript	0.411241	0.46623	0.718408	0.80527	0.390777	0.578496	0.931634	0	0.741318
Novel_010309	XLOC_10959.	transcript	0.687268	0.976493	0	0	0	0	0	0.272555	0
Novel_010310	XLOC_10959.	transcript	0.298197	0.40766	0.000168	0	0	0	0	0.145782	0
Novel_010311	XLOC_10959.	transcript	0.396273	0.438683	0.858763	0.760215	0.698533	0.908549	0.895041	0	0.782293
Novel_010312	XLOC_10959.	transcript	0.262195	0.363103	0.267256	0.195229	0.118819	0.166954	0.189797	0.282879	0.285184
Novel_010313	XLOC_10960.	transcript	0.006742	0.684274	0	0.034189	0.012093	0.028532	0.077783	0.006324	0.014013
Novel_010314	XLOC_10963.	transcript	0	0	0	0.049971	0	0	0.254019	0	0
Novel_010315	XLOC_10963.	transcript	0.347827	0	0.069083	0.422291	0.191923	0.158336	0.064872	0.122501	0.134066
Novel_010316	XLOC_10963.	transcript	0.0351	0.062073	0.050305	0.373938	0.289777	0.559237	0.248242	0.341499	0.342063
Novel_010317	XLOC_10966.	transcript	0	0	0	0	0	0.068873	0.064059	0.134006	0
Novel_010318	XLOC_10966.	transcript	0.030116	0.03744	0.087557	0.100541	0.061553	0	0	0	0.142764
Novel_010319	XLOC_10969.	transcript	0.000562	0.036818	0.037956	0	0.020986	0.047595	0.188531	0.077231	0.05414
Novel_010320	XLOC_10976.	transcript	0.364706	0.538697	0.488969	0.653926	0.522452	0.56865	0.698767	0.780458	0.299257
Novel_010321	XLOC_10976.	transcript	0	0	0	0	0	0	0	0	0
Novel_010322	XLOC_10977.	transcript	0	0	0	0	0	0	0	0	0
Novel_010323	XLOC_10977.	transcript	0.343324	0.50752	0.310201	0.368167	0.389757	0.501739	0.342948	0.489982	0
Novel_010324	XLOC_10977.	transcript	0	0	0.051923	0	0	0	0	0.052689	0.34121

Novel_010325	XLOC_10977.	transcript	0	0.006542	0	0	0	0	0	0	0
Novel_010326	XLOC_10980.	transcript	0.340309	0.508607	0.298017	0.396859	0.424832	0.475342	0.482291	0.480644	0.295255
Novel_010327	XLOC_10980.	transcript	0	0	0	0	0	0	0.014294	0	0
Novel_010328	XLOC_10984.	transcript	0	0	0	0	0	0	0	0	0
Novel_010329	XLOC_10984.	transcript	0	0	0	0	0	0	0	0	0.143641
Novel_010330	XLOC_10984.	transcript	0	0	0	0	0	0	0	0	0
Novel_010331	XLOC_10984.	transcript	0	0	0	0	0	0	0	0	0
Novel_010332	XLOC_10984.	transcript	0	0	0	0.007985	0	0	0.002986	0	0.001886
Novel_010333	XLOC_10984.	transcript	0	0	0	0	0	0	0.018897	0	0
Novel_010334	XLOC_10984.	transcript	0	0	0	0	0	0	0	0	0
Novel_010335	XLOC_10984.	transcript	0.071713	0	0	0	0	0	0	0	0
Novel_010336	XLOC_10984.	transcript	0	0	0	0	0	0	0	0	0
Novel_010337	XLOC_10984.	transcript	0	0	0	0	0	0	0	0	0
Novel_010338	XLOC_10984.	transcript	0	0	0	0	0	0	0	0	0
Novel_010339	XLOC_10984.	transcript	0	0	0	0	0	0	0	0	0
Novel_010340	XLOC_10984.	transcript	0	0	0	0	0	0	0	0	0
Novel_010341	XLOC_10984.	transcript	0	0	0	0	0	0	0	0	0
Novel_010342	XLOC_10984.	transcript	0	0.010237	0	0.009115	0	0.022059	0.011097	0.003524	0.000113
Novel_010343	XLOC_10995.	transcript	0.49442	0.561531	1.862292	1.15498	0.998253	1.545903	0.954683	1.253433	1.683534
Novel_010344	XLOC_10998.	transcript	0.076024	0.145162	0.129231	0.327145	0.132649	0.148433	0.167198	0.303138	0.122251
Novel_010345	XLOC_11000.	transcript	1.126524	3.612239	1.994968	1.771698	1.318661	2.110032	1.427862	1.959383	1.587564
Novel_010346	XLOC_11003.	transcript	0.036569	0.027148	0.237075	0.275949	0.600887	0.384511	0.284471	0.293302	0.07052
Novel_010347	XLOC_11003.	transcript	0.550349	0.342094	1.096978	0.760577	0.857863	0.568364	1.011554	0.849856	0.940748
Novel_010348	XLOC_11005.	transcript	1.145734	0.938186	1.127779	1.790603	1.14476	1.112804	0.616468	1.390537	1.042949
Novel_010349	XLOC_11005.	transcript	0.150288	1.138003	0.0793	0.03095	0.255615	0.021025	1.146106	0.251513	0.029611
Novel_010350	XLOC_11006.	transcript	6.025452	7.214757	11.66293	9.767623	10.46497	10.73898	5.16361	8.304848	9.270502
Novel_010351	XLOC_11006.	transcript	0	0	0	0	0	0.737217	0	0.573704	0
Novel_010352	XLOC_11006.	transcript	0.253335	0.019943	0.323854	0.306871	0.271226	0	0.412336	0.081068	0.361563
Novel_010353	XLOC_11006.	transcript	0.886667	0	0	0	0	0.006024	0.001427	0.00377	0
Novel_010354	XLOC_11006.	transcript	0.304842	0	0.082263	0.013993	0.296173	0.005061	0.395177	0.014261	0.31425
Novel_010355	XLOC_11006.	transcript	0	0	0	1.098778	0	0	0.904849	1.158016	0
Novel_010356	XLOC_11006.	transcript	0	0	0	0	0.00416	0	0.024025	0	0
Novel_010357	XLOC_11006.	transcript	0.839934	0	1.127971	0.346507	1.019905	0	0.255344	0.248068	0.539509
Novel_010358	XLOC_11006.	transcript	0.696909	0.009653	0	0.329062	0.452125	1.872626	0.248937	0.245235	0.490255
Novel_010359	XLOC_11006.	transcript	0.442071	0.999112	0.732882	0.463195	0.25936	0.141162	0.86869	1.125767	0.311828
Novel_010360	XLOC_11019.	transcript	0.175309	0.136827	0.168718	0.351614	0.202957	0.344862	0.316095	0.211545	0.08029
Novel_010361	XLOC_11025.	transcript	0.431022	0	0.717816	0.505711	0.350799	0.781133	1.163946	0.729901	0
Novel_010362	XLOC_11025.	transcript	0.624083	0	0.008941	0	0.633817	0.138893	0.573253	0.598371	0.661597

Novel_010363	XLOC_11025.	transcript	0.0759	0.002996	0.058852	0.448471	0.087762	0	0.064608	0.026719	0
Novel_010364	XLOC_11025.	transcript	0.04239	0.040506	0.262868	0.138251	0.138089	0.360415	0.441134	0.350263	0.415363
Novel_010365	XLOC_11025.	transcript	0	0.072374	0	0.003649	0.028103	0.013731	0.006629	0.026978	0.012062
Novel_010366	XLOC_11027.	transcript	0	0	0.256142	0	0.487262	0.604394	0.559376	0	0.35366
Novel_010367	XLOC_11027.	transcript	0.317873	0.303286	0	0.507645	0	0	0	0.392624	0
Novel_010368	XLOC_11028.	transcript	0.182298	0.189367	0.140909	0.08135	0.129742	0.091075	0.30773	0.409183	0.222895
Novel_010369	XLOC_11032.	transcript	0.050653	0.012886	0.747611	0.015171	0.171141	0.257144	0.017367	0.196346	0.457729
Novel_010370	XLOC_11032.	transcript	0.009148	0.018437	0	0.023001	0.132207	0	0.011391	0.029996	0.009833
Novel_010371	XLOC_11032.	transcript	0	0	0	0	0	0	0	0	0
Novel_010372	XLOC_11032.	transcript	0.737118	0	0	0.342092	0.005783	0	0.459715	0.101377	0.044722
Novel_010373	XLOC_11032.	transcript	0	0.655179	0.119477	0.979538	0	0.015141	0	0.041631	0.058795
Novel_010374	XLOC_11032.	transcript	0	0	0	0.000605	0	0	0.739857	0	0.005004
Novel_010375	XLOC_11032.	transcript	0.001645	0	0	0.00034	0	0.001155	0	0	0
Novel_010376	XLOC_11032.	transcript	0.242694	0.292097	0.016423	0.215067	0.588266	0.340945	0.204102	0.286742	0.041499
Novel_010377	XLOC_11033.	transcript	0	0.046913	0	1.022375	0	0	0	0	0
Novel_010378	XLOC_11033.	transcript	0.257379	0.019782	0	0	0	0	0	0	0.237921
Novel_010379	XLOC_11033.	transcript	0	0.021125	1.428715	0.630497	1.29916	0.606994	0.566709	0.744417	0
Novel_010380	XLOC_11033.	transcript	0	0.022431	0	0	0	0.644891	0	0	0
Novel_010381	XLOC_11033.	transcript	0.379373	0.043948	0.72024	0	0.878742	1.656592	0.532695	0.597031	0.323597
Novel_010382	XLOC_11034.	transcript	0.552184	0	0.393923	0.325547	0.14212	0.405435	0.349305	0.267586	1.307577
Novel_010383	XLOC_11034.	transcript	0.059502	1.127651	0.098214	0.089632	0.454669	0.109426	0.534307	0.351965	0.651327
Novel_010384	XLOC_11036.	transcript	0.248906	0.234056	0.370138	0.268683	0.231659	0.287039	0.400895	0.19368	0.155002
Novel_010385	XLOC_11037.	transcript	0	0	0	0.091485	0	0	0	0	0
Novel_010386	XLOC_11038.	transcript	0.165085	0.195557	0	0.000535	0	0	0.239834	0.45605	0
Novel_010387	XLOC_11038.	transcript	0.161427	0.195095	0	0.010653	0.002947	0	0.2455	0	0
Novel_010388	XLOC_11038.	transcript	0.162877	0.231619	0	0	0	0	0.406183	0.625077	0
Novel_010389	XLOC_11038.	transcript	0.00267	0	0	0	0	0	8.40E-05	0.018529	0
Novel_010390	XLOC_11038.	transcript	0.166871	0.219527	0.026202	0.936831	0.02253	0	0.100387	0.320655	0.063787
Novel_010391	XLOC_11038.	transcript	0.176438	0.20377	0	0.271998	0.003531	0.386048	0.099866	0	0.012607
Novel_010392	XLOC_11038.	transcript	0.158666	0.265066	0.791709	0	0.692764	0.503506	0.088333	0	0.563398
Novel_010393	XLOC_11040.	transcript	0.418464	0.307943	0.444254	0.579959	0.662542	0.729593	0.416031	0.248549	0.437102
Novel_010394	XLOC_11040.	transcript	0	0	0	0.214275	0	0	0.16955	0	0
Novel_010395	XLOC_11040.	transcript	0.308758	0.000459	0.305621	0.110956	0.057451	0.068672	0.150126	0.378504	0.015063
Novel_010396	XLOC_11040.	transcript	0	0.004861	0.012688	0	0.004399	0	0.254385	0.003694	0
Novel_010397	XLOC_11041.	transcript	0	0	0	0.322677	0.056566	0.151619	0.330251	0	0
Novel_010398	XLOC_11041.	transcript	0	0	0.027578	0	0	0	0.176433	0	0
Novel_010399	XLOC_11042.	transcript	0.155093	0.229331	0.118473	0.207163	0.14122	0.110028	0.225267	0.221798	0.120309
Novel_010400	XLOC_11042.	transcript	0.062982	0.006875	0.144303	0.18207	0.099076	0.009342	0.108205	0.092012	0.00983

Novel_010401	XLOC_11042.	transcript	0	0	0.019363	0	0.009728	0.005171	0.014669	0.146843	0
Novel_010402	XLOC_11042.	transcript	0	0	0	0.019061	0	0	0	0	0
Novel_010403	XLOC_11042.	transcript	0.238696	0.214355	0.137425	0	0.222656	0.197217	0.436043	0.248087	0.178682
Novel_010404	XLOC_11043.	transcript	0	0.06382	0.049857	0.055691	0	0.202614	0.044063	0.180595	0.181703
Novel_010405	XLOC_11043.	transcript	0.002121	0	0.064562	0	0	0	0.186459	0.010275	0
Novel_010406	XLOC_11043.	transcript	0.175685	0	0	0.269585	0.121679	0	0	0.068265	0
Novel_010407	XLOC_11043.	transcript	0.110268	0.222349	0.040754	0.052238	0.085189	0.361633	0	0.11013	0.212298
Novel_010408	XLOC_11043.	transcript	0	0	0.151357	0	0	0	0	0.085333	0
Novel_010409	XLOC_11043.	transcript	0.164514	0	0.067573	0	0.099386	0.135803	0.351257	0.149516	0.057688
Novel_010410	XLOC_11043.	transcript	0.112852	0	0.117083	0	0.632318	0	0	0	0
Novel_010411	XLOC_11043.	transcript	0	0	0.134795	0	0	0	0	0	0
Novel_010412	XLOC_11044.	transcript	0.266328	0.339506	0.429948	0.89595	0.696434	0.84886	0.607133	0.08195	0.726557
Novel_010413	XLOC_11044.	transcript	0	0	0	0	0	0	0.226749	0.716244	0
Novel_010414	XLOC_11044.	transcript	0.028279	0	0	0	0	0	0	0.015714	0
Novel_010415	XLOC_11044.	transcript	0	0	0	0.552404	0	0.200287	0	0.584521	0.603736
Novel_010416	XLOC_11044.	transcript	1.004998	0.766045	1.074665	0.371157	1.087132	0.841039	1.064202	0.706886	0.251412
Novel_010417	XLOC_11044.	transcript	0.206257	0.219322	0.076316	0.399178	0.274035	0.158161	0.283537	0.310431	0
Novel_010418	XLOC_11044.	transcript	0.150382	0	0.139097	0	0	0.107234	0.169053	0.00881	0.208818
Novel_010419	XLOC_11044.	transcript	0.219988	0.082712	0.273732	0.099559	0.083562	0.220175	0.435689	0.291884	0.191656
Novel_010420	XLOC_11047.	transcript	0.247469	0.711446	0.222338	0.533004	0.180226	0.068479	0.496193	0.686006	0.067753
Novel_010421	XLOC_11047.	transcript	0.046469	0.080556	0.175062	0.121835	0.254707	0.153178	0.15747	0	0
Novel_010422	XLOC_11047.	transcript	0.465004	0.379991	0.599407	0.446147	0.677389	0.585937	0.814789	0.148516	0.883345
Novel_010423	XLOC_11047.	transcript	0	0	0	0	0	0	0	0	0
Novel_010424	XLOC_11047.	transcript	0	0	0	0	0	0	0	0.472912	0
Novel_010425	XLOC_11047.	transcript	1.123685	1.172962	1.444131	1.199764	1.406694	0.954616	1.5574	1.341208	1.094056
Novel_010426	XLOC_11048.	transcript	0.123601	0.49616	0.471837	0.089977	0.38537	0.401649	0.456021	0.289754	0.463426
Novel_010427	XLOC_11048.	transcript	0.010888	0	0	0.247602	0.017695	0.131594	0.001323	0.06913	0.099946
Novel_010428	XLOC_11048.	transcript	0.059557	0	0.023985	0.318981	0.010183	0.039934	0.020351	0.008649	0.012038
Novel_010429	XLOC_11050.	transcript	0.017437	0	0.325765	0.106591	0	0	0.487384	0.269471	0.066088
Novel_010430	XLOC_11050.	transcript	0.242993	0.310387	0.289094	0.356586	0.46619	0.342171	0.217401	0.237066	0.419563
Novel_010431	XLOC_11050.	transcript	0	0.163393	0.10042	0	0.0636	0.210686	0.139492	0.11244	0.138193
Novel_010432	XLOC_11050.	transcript	0.073598	0.078141	0.151855	0.437157	0.265853	0.11022	0.092375	0.312255	0.021419
Novel_010433	XLOC_11051.	transcript	0.235429	0.467526	0.547055	0.429257	0.469274	0.581518	0.662554	0.541085	0.414028
Novel_010434	XLOC_11051.	transcript	0.184151	0.093457	0.488122	0.435152	0.47461	0.488135	0.582415	0.521932	0.373644
Novel_010435	XLOC_11051.	transcript	0	0	0	0	0	0	0	0	0.167946
Novel_010436	XLOC_11051.	transcript	0.123225	0.001308	0.25429	0.07487	0.142049	0.089555	0.138997	0.339912	0.198822
Novel_010437	XLOC_11051.	transcript	0.025716	0.078311	0.017281	0.279073	0.169361	0.11498	0.308398	0.017517	0.000616
Novel_010438	XLOC_11051.	transcript	0.309824	0.114372	0.030307	0.328049	0.008092	0.242052	0.237794	0.475409	0

Novel_010439	XLOC_11051.	transcript	0.576615	0.176788	0	0.568858	0.011774	0.27717	0.223384	0.685774	0.05111
Novel_010440	XLOC_11051.	transcript	0.016772	0.276001	0.609936	0	0.811119	0	0.240762	0	0
Novel_010441	XLOC_11051.	transcript	0	0.183293	0	0	0	0.001739	0.259468	0	0
Novel_010442	XLOC_11051.	transcript	0	0.152948	0	0	0	0	0.211224	0	0
Novel_010443	XLOC_11052.	transcript	0	0.149628	0.018683	0.086359	0.092946	0.100125	0.114217	0	0
Novel_010444	XLOC_11052.	transcript	1.092947	1.141863	0.743156	0.576186	0.535095	0.392533	1.129711	0.914877	0.487862
Novel_010445	XLOC_11052.	transcript	0.482027	0	0.021528	0	0.039985	0.251036	0.574427	0.399853	0.166394
Novel_010446	XLOC_11052.	transcript	0	0.18224	0.275979	0	0.378265	0.055318	0.098758	0	0.139329
Novel_010447	XLOC_11052.	transcript	0.00357	0	0	0.309664	0	0	0	0	0
Novel_010448	XLOC_11053.	transcript	0.369471	0.132129	0.258575	0.355301	0.315604	0.255068	0.309279	0.262847	0.256854
Novel_010449	XLOC_11053.	transcript	0.278275	0.426764	0.344092	0.602389	0.44799	0.293488	0.760659	0.568909	0.284725
Novel_010450	XLOC_11053.	transcript	0.199399	0.871862	0.517856	0.483532	0.356922	0.560825	0.643657	0.583045	0.413617
Novel_010451	XLOC_11053.	transcript	0.404558	0.290868	0	0	0	0	0	0	0
Novel_010452	XLOC_11054.	transcript	0	0	0.005572	0.17892	0.121652	0.264518	0.037445	0.031111	0
Novel_010453	XLOC_11054.	transcript	0.111803	0.052109	0.381274	0.055557	0.004799	0.301282	0.125838	0.12433	0.100474
Novel_010454	XLOC_11054.	transcript	0.115059	0.186196	0.180385	0.802647	0.592684	0	0.32587	0.45951	0.220312
Novel_010455	XLOC_11054.	transcript	0.096714	0.259247	0.356672	0.133818	0.168655	0.194911	0.202018	0.223363	0.182935
Novel_010456	XLOC_11054.	transcript	0	0	0	0	0	0	0	0	0
Novel_010457	XLOC_11054.	transcript	0.113516	0.109395	0.202283	0.173031	0.122992	0	1.275454	0	0.318587
Novel_010458	XLOC_11054.	transcript	0	0	0	0	0	0	0	0	0
Novel_010459	XLOC_11054.	transcript	0.764713	1.261581	1.577354	1.762223	0.809575	1.467449	0.890752	1.528631	0.947737
Novel_010460	XLOC_11054.	transcript	0	0	0	0	0	0	0	0	0
Novel_010461	XLOC_11054.	transcript	0.715876	0.106661	2.665513	2.826425	2.819929	3.242767	1.355861	1.484628	2.396752
Novel_010462	XLOC_11057.	transcript	0.22861	0.064287	0.435918	0.242833	0.314699	0.37829	0.276733	0.241756	0.511176
Novel_010463	XLOC_11057.	transcript	0.10728	0	0.027332	0.005411	0	0	0.163781	0.014184	0.031719
Novel_010464	XLOC_11058.	transcript	0.204392	0.024282	0.067948	0	0.186387	0.046237	0.358893	0.027541	0
Novel_010465	XLOC_11058.	transcript	0.395534	0.004062	0.201412	0.050824	0.090526	0.065108	0.47121	0.055081	0.014672
Novel_010466	XLOC_11058.	transcript	0.424715	0	0.241084	0.082823	0.139433	0.082211	0.338276	0.187167	0
Novel_010467	XLOC_11058.	transcript	0	0	0	0	0	0	0.297094	0.106122	0.024556
Novel_010468	XLOC_11058.	transcript	0	0	0	0	0	0	0	0.068162	0.148458
Novel_010469	XLOC_11058.	transcript	0	0	0	0.001179	0	0	0	0.056954	0.117644
Novel_010470	XLOC_11058.	transcript	0	0	0	0	0	0	0	0.050008	0.098223
Novel_010471	XLOC_11058.	transcript	0.003211	0	0.039496	0.072101	0.001142	0.000214	3.20E-05	0.065954	0.135126
Novel_010472	XLOC_11061.	transcript	0.095494	0.107666	0.478578	0.567896	0.540235	0.536994	0.593494	0.212968	0.293285
Novel_010473	XLOC_11062.	transcript	0.144951	0.040287	0.115176	0.175416	0.28528	0.255408	0.294564	0.222359	0.236591
Novel_010474	XLOC_11062.	transcript	0	0.407004	0	0.076826	0	0	0	0	0
Novel_010475	XLOC_11063.	transcript	0.3958	0.244703	0.388515	0.374011	0.415915	0.296857	0.519985	0.262701	0.328166
Novel_010476	XLOC_11063.	transcript	0.149016	0.095191	0.209462	0.169702	0.249579	0.167565	0.172816	0.156893	0.141082

Novel_010477	XLOC_11064.	transcript	0.200113	0.031281	0.245399	0.437713	0.240174	0.295313	0.22328	0.215339	0.283812
Novel_010478	XLOC_11065.	transcript	0	0	0	0	0.524736	0.133309	0	0	0
Novel_010479	XLOC_11065.	transcript	0.62596	0.550807	0.610058	0.253992	0.012565	0.594708	0.673462	0.722269	0.521232
Novel_010480	XLOC_11065.	transcript	0	0	0	0	0	0	0	0	0
Novel_010481	XLOC_11065.	transcript	0	0	0	0.907505	0	0	0	0	0
Novel_010482	XLOC_11065.	transcript	0.134226	0.132963	0.368856	0.785063	0.087335	0.827179	0.282979	0.069235	0.42744
Novel_010483	XLOC_11065.	transcript	0.687274	0.077915	0.423688	0.211257	0.621906	0.238346	0.552427	0.27189	0.26588
Novel_010484	XLOC_11065.	transcript	0	0	0	0	0	0.060334	0.224991	0.352224	0
Novel_010485	XLOC_11066.	transcript	1.326637	0	0	0	0	0	0	0	0
Novel_010486	XLOC_11066.	transcript	0.003247	0	0.002896	0	0.014716	0	0	0.029842	0
Novel_010487	XLOC_11066.	transcript	0.236077	0	3.715668	3.698354	0.152699	2.14471	0.186685	0.262323	1.782264
Novel_010488	XLOC_11066.	transcript	0	0	0	0	0.011274	0	0.020363	0.028619	0
Novel_010489	XLOC_11066.	transcript	2.344914	0.05637	0.070193	0.289613	3.323582	2.196958	3.401092	4.026785	1.6609
Novel_010490	XLOC_11066.	transcript	0.247738	0.247899	0.168311	0.174148	0.21244	0.21913	0.394078	0.273379	0.217482
Novel_010491	XLOC_11066.	transcript	0	0	0.224895	0.266414	0.345989	0.028058	0.026161	0	0.261476
Novel_010492	XLOC_11066.	transcript	0.168594	0.255778	0	0.197715	0	0.316162	0.357425	0.321914	0
Novel_010493	XLOC_11068.	transcript	0.519365	0.089037	0.756315	1.499695	1.00907	0.904415	0.649346	1.098883	0.551194
Novel_010494	XLOC_11068.	transcript	0	0	0	0	0	0	0.739838	0	0.003367
Novel_010495	XLOC_11068.	transcript	0	0	0	0	0	0.068371	0.171796	0	0.196828
Novel_010496	XLOC_11068.	transcript	0.131469	0	0.06698	0.603706	0.035359	0	0.057665	0.101129	0.017809
Novel_010497	XLOC_11068.	transcript	0.116549	0.045124	0.053667	0.058705	0.271474	0	0	0.121984	0
Novel_010498	XLOC_11068.	transcript	0	0.359027	0.462788	0	0.10596	0.35898	0	0	0
Novel_010499	XLOC_11068.	transcript	0.183419	0	0.033586	0.130501	0.034881	0	0	0.435992	0
Novel_010500	XLOC_11068.	transcript	0.161072	0	0	0.00242	0	0.008452	0	0.002605	0.412294
Novel_010501	XLOC_11068.	transcript	0	0	0	0	0	0.177277	0	0	0
Novel_010502	XLOC_11068.	transcript	0	0	0	0	0	0.104414	0.270865	0	0.414468
Novel_010503	XLOC_11068.	transcript	0.1519	0.005564	0	0	0.005536	0	0	0	0.050804
Novel_010504	XLOC_11068.	transcript	0	0.025394	0	0.001069	0.011575	0	0.001174	0.200334	0.002171
Novel_010505	XLOC_11068.	transcript	0.027785	0	0	0	0	0	0.270423	0	0
Novel_010506	XLOC_11068.	transcript	0.138936	0.193408	0.071572	0.066005	0.441459	0.226468	0.053476	0	0.131984
Novel_010507	XLOC_11068.	transcript	0.586661	0.129941	0.648022	0.393519	0.348004	0.5776	0.487689	0.09155	0.48726
Novel_010508	XLOC_11068.	transcript	0.045986	0.125699	0.225954	0.57233	0.272695	0.268311	0.364473	0.316795	0.029848
Novel_010509	XLOC_11071.	transcript	0.953084	0.85222	0.894345	0.883838	1.087665	0.821312	1.60449	0.948479	0.952912
Novel_010510	XLOC_11073.	transcript	0.322219	0.006673	0	7.00E-04	0.092727	0.930177	0.09755	0.370673	0.281833
Novel_010511	XLOC_11073.	transcript	0.528775	0.371356	0.724375	0.175695	0.023045	0.041609	0.39903	0.235718	0.450624
Novel_010512	XLOC_11073.	transcript	0	0.219931	0.162121	0.59601	0.561724	0	0.200984	0.314119	0
Novel_010513	XLOC_11073.	transcript	0.319247	0	0	0	0	0	0	0	0
Novel_010514	XLOC_11074.	transcript	0	0	0	0	0	0	0	0.114035	0.057425

Novel_010515	XLOC_11074.	transcript	0	0	0	0	0	0	0	0.007564	0
Novel_010516	XLOC_11076.	transcript	0.068568	0.526513	0.139201	0.018011	0.212197	0.094697	0.169955	0.108549	0.208608
Novel_010517	XLOC_11079.	transcript	0.171511	0	0	0.273754	0.206261	0	0	0	0
Novel_010518	XLOC_11079.	transcript	0.199419	0.070178	0.455715	0.038634	0.0249	0.645349	0.686706	0.564196	0.627995
Novel_010519	XLOC_11087.	transcript	0.126477	0.139189	0.187812	0	0.242916	0.259201	0.177062	0.014208	0.206143
Novel_010520	XLOC_11087.	transcript	0.169871	0.171024	0.401947	0.610147	0.468385	0.420782	0.307557	0.614361	0.340097
Novel_010521	XLOC_11088.	transcript	0.024717	0.026743	0.286872	0.010905	0.335852	0.052198	0.154545	0.055362	0.030839
Novel_010522	XLOC_11088.	transcript	0.157847	0.043469	0.229195	0.09869	0.302651	0.233536	0.262549	0.328025	0.350986
Novel_010523	XLOC_11091.	transcript	0.055857	0.121689	0.159131	0.092955	0.191266	0.155346	0.039071	0.06205	0.083432
Novel_010524	XLOC_11101.	transcript	0	0	0.080243	0	0	0	0.052269	0	0
Novel_010525	XLOC_11104.	transcript	0	0	0.12032	0.010851	0.311794	0	0	0	0
Novel_010526	XLOC_11104.	transcript	0	0	0	0	0	0	0	0	0
Novel_010527	XLOC_11104.	transcript	0.0138	0.035964	0.007746	0.017264	0	0	0.045275	0.178525	0.154014
Novel_010528	XLOC_11104.	transcript	0.131658	0.01614	0.145531	0.376814	0.077111	0.022002	0.181549	0.033426	0
Novel_010529	XLOC_11104.	transcript	0.149437	0	0.075616	0	0	0	0	0	0
Novel_010530	XLOC_11108.	transcript	0	0.028403	0.107924	0.070565	0	0.269672	0.196685	0.00617	0.065351
Novel_010531	XLOC_11108.	transcript	0.259421	0.273149	0	0	0.254074	0.165692	0	0.1675	0.206385
Novel_010532	XLOC_11108.	transcript	0	0.101116	0.017975	0.017672	0	0	0	0.15076	0.010366
Novel_010533	XLOC_11108.	transcript	0.142436	0.027764	0.261482	0.271063	0.131636	0.099283	0.480979	0.02753	0.112115
Novel_010534	XLOC_11108.	transcript	0	0.020143	0.048201	0	0	0	0	0.087501	0
Novel_010535	XLOC_11108.	transcript	0	0	0	0	0	0	0	0	0
Novel_010536	XLOC_11108.	transcript	0	0.011052	0.025246	0	0.028295	0.304399	0.069888	0.079639	0
Novel_010537	XLOC_11108.	transcript	0	0.051217	0	0.045841	0.145306	0.093364	0.383534	0.088832	0.163632
Novel_010538	XLOC_11110.	transcript	0.916956	1.733919	1.252432	1.485852	1.384878	1.405109	1.590299	1.081243	1.129081
Novel_010539	XLOC_11111.	transcript	0	0	0	0.380579	0	0.093219	0	0.034754	0
Novel_010540	XLOC_11111.	transcript	0.444483	0.072993	0.344606	0.284029	0.395105	0.189179	0.384256	0.299319	0.17406
Novel_010541	XLOC_11111.	transcript	0.570846	0.65515	0.661535	0.654215	0.695983	1.472837	0.733565	0.927254	3.915682
Novel_010542	XLOC_11128.	transcript	0.438687	0	0.000863	0.005026	0.001547	0.020986	0	0.023291	0.001334
Novel_010543	XLOC_11128.	transcript	0.214238	0.042736	0.071809	0.566435	0.768512	0.05159	0.071266	0.081038	0.717852
Novel_010544	XLOC_11128.	transcript	0	0.273798	0.003002	0.241763	0	0	0.004527	0.061152	0.053117
Novel_010545	XLOC_11128.	transcript	0.234288	0.460235	0.621496	0	0	0.715618	0.775667	0.568886	0
Novel_010546	XLOC_11128.	transcript	0.284587	0	0.016873	0.058142	0.0165	0.004236	0.646998	0.224745	0.028245
Novel_010547	XLOC_11130.	transcript	0.574708	0.596594	0	0.90146	0.738002	0.926334	1.337628	1.283841	0.711359
Novel_010548	XLOC_11130.	transcript	0	0	0.015658	0.290252	0.004668	0	0	0	0
Novel_010549	XLOC_11130.	transcript	1.273478	0.183756	0.31556	0.579947	0.885616	0.620848	0.350233	0.890988	0.716303
Novel_010550	XLOC_11130.	transcript	0	0	0	0	0	0	0.436431	0	0
Novel_010551	XLOC_11133.	transcript	0.02764	0	0.014734	0.011681	0.056802	0.008464	0.015722	0.018051	0.024942
Novel_010552	XLOC_11133.	transcript	0	0	0	0	0	0	0	0	0

Novel_010553	XLOC_11138	transcript	0	0.527694	0	0.024008	0	0	0	0.015645	0
Novel_010554	XLOC_11139	transcript	0.130305	0.400126	0.204521	0.289587	0.235457	0.22609	0.304948	0.245406	0.31136
Novel_010555	XLOC_11155	transcript	0.468928	0.335674	0.389036	0.212195	0.358579	0.331351	0.105885	0.117891	0.242322
Novel_010556	XLOC_11155	transcript	0	0	0	0	0	0	0	0.247375	0
Novel_010557	XLOC_11155	transcript	0	0	0	0	0	0	0.40165	0	0
Novel_010558	XLOC_11155	transcript	0.072508	0.290631	0.01393	0.05615	0.485718	0	0	0.011463	0.337932
Novel_010559	XLOC_11155	transcript	0	0.343862	0.077009	0.148784	0	0.102115	0.110248	0.177788	0.166802
Novel_010560	XLOC_11155	transcript	0.294791	0.399992	0.293993	0.415489	0.41991	0.709898	0.382654	0.003202	0
Novel_010561	XLOC_11155	transcript	0	0	0	0	0	0.361833	0.610721	1.292082	0
Novel_010562	XLOC_11156	transcript	0.021176	0.421519	0.030274	0	0.034074	0	0.072393	0.458068	0.173672
Novel_010563	XLOC_11156	transcript	0.223805	0.341525	0.224417	0.438583	0.358484	0.404672	0.289817	0	0.304625
Novel_010564	XLOC_11158	transcript	0.680274	0.670082	0.797888	0.669375	0.659661	0.77051	0.933056	0.78172	0.386476
Novel_010565	XLOC_11158	transcript	0.154645	0.186944	0.422459	0.388135	0.292097	0.494987	0.287413	0	0.241247
Novel_010566	XLOC_11158	transcript	0	0.006964	0	0	0	0	0	0.67514	0
Novel_010567	XLOC_11158	transcript	0.474826	0.172005	0.269071	0.370379	0.071944	0.018465	0.509693	0.187198	0.187407
Novel_010568	XLOC_11158	transcript	0	0.117288	0	0	0.364977	0.282754	0	0.207389	0
Novel_010569	XLOC_11158	transcript	0	0	0	0	0	0	0	0	0
Novel_010570	XLOC_11158	transcript	0	0.148688	0	0	0	0	0	0.171273	0
Novel_010571	XLOC_11161	transcript	1.13691	2.508971	2.323603	2.525077	2.162324	2.405747	2.028626	1.792592	2.101622
Novel_010572	XLOC_11161	transcript	0	0	0	0	0	0	0	0	0
Novel_010573	XLOC_11161	transcript	0.020675	0.284508	0.087142	0.103912	0.138862	0.1372	0.152774	0.21288	0.336915
Novel_010574	XLOC_11163	transcript	1.510639	1.673453	1.789768	2.736093	1.856776	2.31605	2.111181	2.421124	2.067098
Novel_010575	XLOC_11163	transcript	0	0	0	0	0	0	0	0	0
Novel_010576	XLOC_11166	transcript	0.979216	0.848897	1.940922	2.207844	1.452384	1.858407	0.494281	2.260202	0.52674
Novel_010577	XLOC_11167	transcript	0	0.192673	0	0	0.004288	0	0	0.401595	0.161192
Novel_010578	XLOC_11167	transcript	0	0	0	0.359814	0.324926	0.577078	0	0	0.358623
Novel_010579	XLOC_11167	transcript	0.622778	0.303927	0.579352	0.451682	0.480905	0.042919	0.468315	0.46323	0.27613
Novel_010580	XLOC_11167	transcript	0.360454	0.2774	0.260845	0	0	0	0.658587	0.337768	0
Novel_010581	XLOC_11167	transcript	0	0	0	0	0	0	0	0	0
Novel_010582	XLOC_11168	transcript	0.247455	0	0.282814	0.02386	0.10393	0	0.201132	0.14637	0.033626
Novel_010583	XLOC_11169	transcript	0	2.010788	0	0	0.113085	0.166752	0.223129	0.084459	0.272552
Novel_010584	XLOC_11169	transcript	0	0.878648	0	0	0	0	0	0	0
Novel_010585	XLOC_11169	transcript	0.060758	0.067587	0	0.678261	0	0.041754	0.034502	0.015872	0
Novel_010586	XLOC_11170	transcript	1.020719	0.503285	0.03963	0.510354	0.33732	0.07185	0.607129	0.212376	0.60339
Novel_010587	XLOC_11174	transcript	0	0	0	0	0	0	0	0.199393	0
Novel_010588	XLOC_11174	transcript	0.294341	0.217012	0.213489	0.516622	0.327905	0.563355	0.517799	0.982362	0.37349
Novel_010589	XLOC_11177	transcript	0	0	0.135141	0	0.155872	0	0.001724	0	0
Novel_010590	XLOC_11177	transcript	0.455848	0	0.41747	0.667905	0.281344	0.384673	0.163521	0.662518	0.234793

Novel_010591	XLOC_11177.	transcript	0	0	0	0	0	0.192303	0	0	0
Novel_010592	XLOC_11177.	transcript	0	0	0	0	0	0	0	0.181727	0
Novel_010593	XLOC_11193.	transcript	0.076747	0.007951	0.020284	0.056255	0.068618	0.046221	0.057326	0.060254	0
Novel_010594	XLOC_11221.	transcript	0.79159	1.991415	1.564931	0.659382	0.499034	0.626951	1.661471	2.124892	2.251921
Novel_010595	XLOC_11221.	transcript	0.267654	0	0	0	0	0.148798	0	0	0
Novel_010596	XLOC_11221.	transcript	0.286235	0.17162	0	1.685371	1.20461	1.118762	0	0	0
Novel_010597	XLOC_11221.	transcript	0.305286	0	0	0	0	0.963214	0	0	0
Novel_010598	XLOC_11225.	transcript	0.075574	0.351639	0.505157	0.322171	0.292262	0.621138	0.128518	0.377345	0.299539
Novel_010599	XLOC_11231.	transcript	0.123129	0.180352	0.210769	0.212089	0.20596	0.13711	0.094634	0.184607	0.156151
Novel_010600	XLOC_11243.	transcript	0.205963	0.343734	0.131414	0.050572	0	0.130459	0.080392	0.019661	0
Novel_010601	XLOC_11257.	transcript	0	0	0	0	0.015522	0	0.32247	0	0
Novel_010602	XLOC_11257.	transcript	0.315329	0.35929	0.210581	0.180073	0.348466	0.171619	0.372463	0.291121	0.176679
Novel_010603	XLOC_11257.	transcript	0	0.076144	0	0	0	0	0	0	0
Novel_010604	XLOC_11257.	transcript	0.197242	0.245391	0.117693	0.344352	0.22773	0.24301	0.38845	0.263715	0.045642
Novel_010605	XLOC_11257.	transcript	1.360351	1.564766	1.654269	1.259467	1.088646	1.662633	1.545175	1.601772	1.020082
Novel_010606	XLOC_11260.	transcript	0	0.012955	0.0088	0.010009	0.03689	0	0.012543	0.005964	0.060178
Novel_010607	XLOC_11260.	transcript	0	0.421765	0.003544	0.017653	0	0	0.027994	0.080502	0.10804
Novel_010608	XLOC_11260.	transcript	0	0	0.025938	0.041926	0	0	0.130526	0.091149	0
Novel_010609	XLOC_11260.	transcript	0.143293	0.056504	0.146742	0.13286	0.142138	0.140774	0.046434	0.063838	0.111114
Novel_010610	XLOC_11260.	transcript	0.011478	0	0	0.007456	0	0	0	0.033206	0
Novel_010611	XLOC_11260.	transcript	0.867504	0	0	0	0	0	0	0	0
Novel_010612	XLOC_11260.	transcript	0	0	0	0.683854	0.507001	0.942338	0.729861	0.20971	0.160605
Novel_010613	XLOC_11260.	transcript	0.133579	0	0.540326	0.136813	0.189397	0.25643	0.55231	0.164118	0.104307
Novel_010614	XLOC_11260.	transcript	0.023986	0	0	0	0	0	0.240927	0	0
Novel_010615	XLOC_11260.	transcript	0	0	0	0	0	0	0	0	0
Novel_010616	XLOC_11260.	transcript	0	0	0	0.280676	0.36074	0.240629	0.003168	0.2313	0.237723
Novel_010617	XLOC_11260.	transcript	3.157078	0	2.086047	0.516016	1.209421	1.186285	2.73565	0.965446	0.772113
Novel_010618	XLOC_11262.	transcript	0.150966	0	0.098278	0.662697	0	0.014688	1.248045	0.95013	1.342262
Novel_010619	XLOC_11262.	transcript	0.387428	0.209564	0.569565	0.089945	0.654154	0.913941	0.091391	0.11866	0.149244
Novel_010620	XLOC_11262.	transcript	0.418503	0.634153	0.223658	0.287769	0.363836	0.14906	0.559387	0.488642	0.21313
Novel_010621	XLOC_11262.	transcript	0.313673	0.222401	0.092068	0.081546	0.290364	0.616555	0.06162	0.167588	0.194154
Novel_010622	XLOC_11262.	transcript	0.055142	0	0.221036	0	0	0	0.061833	0	0
Novel_010623	XLOC_11262.	transcript	0.215297	0.950696	0	0.299701	0.435209	0.321209	0.454925	0.350781	0.258706
Novel_010624	XLOC_11262.	transcript	0.483941	0.823551	1.006784	0.941908	0.928757	1.162087	1.040621	1.019334	1.192173
Novel_010625	XLOC_11262.	transcript	0	0	0	0	0	0	0	0	0.002285
Novel_010626	XLOC_11263.	transcript	0.000416	0.000537	0.121853	0.000675	0.099907	0.037654	0	0	0.676691
Novel_010627	XLOC_11263.	transcript	1.175936	2.224367	0.794361	0.979684	0.927597	1.133578	1.468728	1.547589	1.00704
Novel_010628	XLOC_11268.	transcript	0.248479	0.534526	0.160861	0.254779	0.263245	0.308667	0.411156	0.230461	0.398922

Novel_010629	XLOC_11269.	transcript	0.420131	2.632704	0.45048	0.460409	0.694194	0.60385	0.556547	0.745624	1.356626
Novel_010630	XLOC_11269.	transcript	0.009728	0.134173	0	0	0	0	0	0	0
Novel_010631	XLOC_11269.	transcript	0.021838	0.620967	0.003402	0.283243	0.025807	0.111537	0.320133	0.009997	0.368289
Novel_010632	XLOC_11269.	transcript	0.394095	0.095588	0.538333	0.236336	0.638706	0.296893	0.490868	0.464926	0.445286
Novel_010633	XLOC_11269.	transcript	0	1.189066	0	0	0	0.187132	0.111388	0	0.035234
Novel_010634	XLOC_11269.	transcript	0	0	0.007974	0	0	0	0.107826	0	0
Novel_010635	XLOC_11271.	transcript	0.661931	2.530231	2.183276	1.722704	1.140577	2.346098	1.85625	1.637009	2.991764
Novel_010636	XLOC_11271.	transcript	0.240571	0.735635	0.614455	0.150628	0.571903	0.601367	0.963132	0.545626	0.895314
Novel_010637	XLOC_11271.	transcript	0.779381	0.015953	0.02789	0.021023	0.019618	0.013992	0	0.144358	0.083217
Novel_010638	XLOC_11271.	transcript	0.088614	0	0.082511	0	0	0	0	0.088027	0
Novel_010639	XLOC_11280.	transcript	0.324329	0.131297	0.525959	0.690507	0.595181	0.586251	0.528726	0.599942	0.197519
Novel_010640	XLOC_11282.	transcript	0	0	0	0	0	0	0.00932	0	0
Novel_010641	XLOC_11283.	transcript	0.067666	0.592755	0.017487	0.131893	0	0.261992	0.107312	0.081678	0.218472
Novel_010642	XLOC_11283.	transcript	0.016976	0.033805	0.519016	0	0.730226	0	0.015891	0	0
Novel_010643	XLOC_11283.	transcript	0.02785	0.365416	0	0	0	0.033601	0.098106	0.03189	0.147197
Novel_010644	XLOC_11283.	transcript	0.127876	0.357341	0.447601	0.362994	0.37238	0.357406	0.312049	0.3678	0.414548
Novel_010645	XLOC_11283.	transcript	0	0	0.032289	0	0.010817	0	0.017644	0.027353	0.025601
Novel_010646	XLOC_11283.	transcript	0	0	0	0	0	0	0	0	0.063073
Novel_010647	XLOC_11284.	transcript	1.681095	2.522312	2.342244	2.001921	1.915104	2.063751	2.363591	1.541324	2.234534
Novel_010648	XLOC_11286.	transcript	0	0	0.062917	0	0	0	0	0	0
Novel_010649	XLOC_11286.	transcript	0.141732	0	0	0	0	0	0.109637	0.282243	0.271538
Novel_010650	XLOC_11286.	transcript	0.150478	0.1874	0.250848	0.241101	0.304832	0.24274	0.324694	0.088828	0.077296
Novel_010651	XLOC_11288.	transcript	0.196151	0	0	0	0	0.00143	0	0	0
Novel_010652	XLOC_11288.	transcript	0.088267	0.060499	0.201518	0.163178	0.173083	0.294416	0.742007	0.484583	0.189839
Novel_010653	XLOC_11288.	transcript	0	0	0	0	0	0.926215	0	0	0
Novel_010654	XLOC_11288.	transcript	3.67202	5.897047	5.569589	4.38154	4.91306	4.304746	4.259527	5.105403	4.064016
Novel_010655	XLOC_11295.	transcript	0.221278	0.328177	0.146437	0.097543	0.233466	0.249741	0.551812	0.213198	0.069011
Novel_010656	XLOC_11295.	transcript	0	0	0	0.408442	0	0	0	0	0.427386
Novel_010657	XLOC_11295.	transcript	0.227126	0.517616	0.05354	0	0.056183	0.002791	0.147317	0.371575	0
Novel_010658	XLOC_11298.	transcript	0	0.398938	0	0	0	0	0	0	0
Novel_010659	XLOC_11298.	transcript	0	0.449881	0	0.003499	0	0	0.053015	0	0
Novel_010660	XLOC_11298.	transcript	0	0	0	0	0	0	0	0.198587	0
Novel_010661	XLOC_11298.	transcript	0.39533	0.007374	0.279563	0.238359	0.528594	0.12707	0.357558	0.121754	0.243249
Novel_010662	XLOC_11298.	transcript	0.019207	0.007326	0.02177	0.019799	0	0	0.011678	0	0
Novel_010663	XLOC_11298.	transcript	0	0	0	0	0	0	0	0	0
Novel_010664	XLOC_11298.	transcript	0.000128	0.091638	0.001065	0	0	0	0.001746	0.156581	0.070471
Novel_010665	XLOC_11298.	transcript	0.00839	0.005707	0.000574	0.008122	0.000746	0.000173	0.026571	0.004259	0.005197
Novel_010666	XLOC_11298.	transcript	0.019408	0.028486	0.064688	0.000349	0.048387	0.087679	0.001891	0.007894	0.005875

Novel_010667	XLOC_1129…	transcript	0.00069	0.002182	0.001044	0.002388	0.105562	0.268789	0.00393	0.004427	0.009642
Novel_010668	XLOC_1129…	transcript	0	0.029226	0	0.046988	0	0.160678	0	0.001101	0
Novel_010669	XLOC_1129…	transcript	0.081819	0.170307	0	0.003726	0	0	0.153608	0.003316	0.00659
Novel_010670	XLOC_1129…	transcript	0.011317	0.00513	0.01429	0.004884	0.31202	0.094574	0.000694	0.002381	0.014397
Novel_010671	XLOC_1129…	transcript	0.377887	0.040673	0	0.086638	0	0	0.142669	0.232836	0.077395
Novel_010672	XLOC_1129…	transcript	0.096536	0.100045	0.186957	0.058195	0.149815	0	0.11362	0.031062	0.103772
Novel_010673	XLOC_1130…	transcript	0	0	0	0	0.236197	0.323186	0.772171	0	0.213879
Novel_010674	XLOC_1130…	transcript	0.000829	0	0.00022	0.039379	0.295418	0.504266	0	0.395688	0.304013
Novel_010675	XLOC_1130…	transcript	0.000316	0	0.037195	0	0.377794	0.10951	0.277726	0.529257	0.385688
Novel_010676	XLOC_1130…	transcript	0.672745	0.066503	0.535014	0.525039	0	0	0	0	0
Novel_010677	XLOC_1130…	transcript	0.156712	0.119356	0	1.126455	0.495129	0	0	0.009882	0.468408
Novel_010678	XLOC_1130…	transcript	1.160996	0.069239	0.779738	1.309703	0.829995	1.270638	1.336771	0.942274	1.176518
Novel_010679	XLOC_1130…	transcript	0	0	0.678874	0.122656	0.052544	0.030141	0	0.159825	0.195822
Novel_010680	XLOC_1130…	transcript	0.00837	0.569284	0	0.286935	0.049454	0	0.118132	0.004468	0.005825
Novel_010681	XLOC_1130…	transcript	0.244166	0.0017	0.100595	0	0	0.080949	0	0.406277	0.233283
Novel_010682	XLOC_1130…	transcript	0.278615	0.000797	0	0.161159	0.042582	0.031366	0.06443	0.417269	0.161399
Novel_010683	XLOC_1130…	transcript	0	0.180657	0.378513	0	0.280109	0.466574	0.561861	0	0.297675
Novel_010684	XLOC_1131…	transcript	0.065138	0.158917	0.031806	0.058854	0.069723	0.135791	0.127845	0.055733	0.049124
Novel_010685	XLOC_1132…	transcript	0.276035	0.171849	0.461427	0.371322	0.531862	0.246328	1.093667	0.279397	1.23848
Novel_010686	XLOC_1132…	transcript	0.342902	0.003794	0.423404	0.342136	0.609488	0.437792	0.549032	0.47026	1.453077
Novel_010687	XLOC_1132…	transcript	0.315974	0.488945	0.749753	1.114069	0.736632	1.046411	0.82076	0.941037	0.725521
Novel_010688	XLOC_1132…	transcript	0.150408	0.348499	0.214637	0.317674	0.333672	0.145993	0.370736	0.457438	0.24194
Novel_010689	XLOC_1132…	transcript	0.131665	0.017961	0.062765	0.010096	0	0	0.263793	0	0
Novel_010690	XLOC_1132…	transcript	0.116584	0.0437	0.109101	0.116605	0.162742	0.056376	0.098922	0.226471	0.027711
Novel_010691	XLOC_1133…	transcript	0.389773	0.549061	0.450794	0.504303	0.586146	0.452523	0.523499	0.6024	0.417872
Novel_010692	XLOC_1133…	transcript	0.036683	0.040537	0.225812	0.233041	0.368934	0.028709	0.18075	0.042029	0.166521
Novel_010693	XLOC_1133…	transcript	0.087103	0.212592	0.144989	0.183633	0.352764	0.087494	0.248053	0.173774	0.102873
Novel_010694	XLOC_1133…	transcript	0.203751	0	0.168709	0.369988	0.271249	0.257745	0.276444	0.157106	0.176968
Novel_010695	XLOC_1133…	transcript	0.179961	0.24997	0.173609	0.803954	0.349162	0.026717	0.442726	0.635749	0.384773
Novel_010696	XLOC_1133…	transcript	0.157602	0	0	0	0	0	0	0	0
Novel_010697	XLOC_1133…	transcript	0.054509	0.08871	0.177887	0.37578	0	0.438166	0	0	0
Novel_010698	XLOC_1133…	transcript	0	0.379189	0	0	0	0	0	0	0
Novel_010699	XLOC_1133…	transcript	0.333081	0	0.382828	0.611819	0.566063	0.345614	0.327918	0.309271	0.190623
Novel_010700	XLOC_1134…	transcript	0.354163	0.58558	1.171409	1.938264	1.865237	1.811262	0.74352	0.959355	1.881159
Novel_010701	XLOC_1134…	transcript	0.103303	0.233898	0.118384	0.0101	0.02627	0.032153	0.49937	0.275538	0.056045
Novel_010702	XLOC_1134…	transcript	0.274627	0	0.147984	0.242035	0.226451	0.29615	0.00396	0.053151	0.265799
Novel_010703	XLOC_1134…	transcript	0	0	0	0.105575	0.210422	0.133245	0	0	0
Novel_010704	XLOC_1134…	transcript	0.140816	0.349006	0.102724	0.074145	0	0.06552	0.297528	0.198847	0.177913

Novel_010705	XLOC_11344.	transcript	0.409241	0	0.275322	0	0	0.010535	0.00225	6.50E-05	0.00169
Novel_010706	XLOC_11344.	transcript	0	0.617743	0.156162	0.461904	0.490076	0.46606	0.758758	0.564944	0.536998
Novel_010707	XLOC_11346.	transcript	0.078735	0.058618	0.008327	0.016349	0.040224	0.037979	0.064579	0.051498	0.018637
Novel_010708	XLOC_11351.	transcript	0.179491	0.22986	0	0.190924	0.12004	0.297731	0.108663	0.10784	0.258321
Novel_010709	XLOC_11351.	transcript	0	0.002465	0.069616	0.005444	0	0.014292	0.27906	0.106629	0.023248
Novel_010710	XLOC_11351.	transcript	0.481722	0.155765	0.423137	0.126659	0.346386	0.026533	0.245534	0.170506	0.03952
Novel_010711	XLOC_11351.	transcript	0	0	0	0.027851	0	0	0	0	0
Novel_010712	XLOC_11351.	transcript	0	0	0	0	0.180572	0	0.097124	0	0
Novel_010713	XLOC_11351.	transcript	0.009594	0	0	0	0.000504	0	0.000232	0	0
Novel_010714	XLOC_11355.	transcript	0.210917	0.278687	0.264523	0.276375	0.224965	0.392813	0.524663	0.195278	0.369874
Novel_010715	XLOC_11358.	transcript	0.013942	0.06441	0.059787	0.243732	0.202918	0.016822	0.076009	0.011974	0.032606
Novel_010716	XLOC_11358.	transcript	0.008786	0.251368	0	0.183063	0.393317	0	0.488605	0.109605	0
Novel_010717	XLOC_11358.	transcript	0.64142	0.330987	0.005562	0	0	0	0.003277	0.116356	0
Novel_010718	XLOC_11358.	transcript	0	0	0	0	0	0.058402	0	0.279636	0
Novel_010719	XLOC_11358.	transcript	0	0	0	0	0	0	0	0	0
Novel_010720	XLOC_11358.	transcript	0.368388	0.813862	0.612142	0.292316	0.44041	0.714516	0.583623	0.286701	0.559016
Novel_010721	XLOC_11360.	transcript	0.147207	0.238717	0.121506	0.204968	0.300951	0.247263	0.305319	0.221033	0.142284
Novel_010722	XLOC_11360.	transcript	0	0.032782	0.341066	0	0.181312	0.015472	0.209391	0.008883	0
Novel_010723	XLOC_11363.	transcript	0.265035	0	0.034201	0.059846	0.061748	0.261711	0.103608	0.082568	0.13224
Novel_010724	XLOC_11369.	transcript	0.494726	0.599805	0.677609	0.754269	0.543987	0.679175	0.558133	0.698232	0.917208
Novel_010725	XLOC_11372.	transcript	0	1.564587	0.023729	0	0	0	0	0	0.093099
Novel_010726	XLOC_11373.	transcript	0.000315	0	0.009459	0	0.349143	0.027088	0.000514	0.52365	0.061547
Novel_010727	XLOC_11373.	transcript	0.590218	0.256039	0.043233	0.515743	0.134235	0.478166	0.062996	0.142069	0.334794
Novel_010728	XLOC_11373.	transcript	0.107239	0.399806	0	0	0	0	0.194151	0	0.017935
Novel_010729	XLOC_11373.	transcript	0	0	0.329036	0	0	0	0	0	0
Novel_010730	XLOC_11373.	transcript	0	0.077796	0	0	0	0	0.791882	0	0
Novel_010731	XLOC_11374.	transcript	0.402698	0.189825	0.392189	0.403549	0.507536	0.407838	0.5654	0.645892	0.44318
Novel_010732	XLOC_11374.	transcript	0.510431	0.157808	0.488903	0.349707	0.5044	0.3838	0.619247	0.451181	0.30045
Novel_010733	XLOC_11375.	transcript	1.50657	4.64507	2.012252	1.60042	1.237993	2.218752	1.162888	1.643222	2.063117
Novel_010734	XLOC_11375.	transcript	0.240265	0.232201	0	0.345032	0.168648	0.165755	0.293908	0	0.048854
Novel_010735	XLOC_11375.	transcript	0.186444	0.062866	0.054081	0.210017	0.190521	0.179167	0.096191	0.064696	0.102835
Novel_010736	XLOC_11375.	transcript	0.108694	0.127609	0.069147	0.022297	0.129883	0.069998	0.275652	0.069651	0.041921
Novel_010737	XLOC_11381.	transcript	0.863187	0	1.086435	1.978025	1.365529	2.012869	1.526461	1.483478	1.926656
Novel_010738	XLOC_11381.	transcript	1.383906	1.938663	0.000939	0.000965	0.581922	0.010034	0.001157	0.016593	0.105297
Novel_010739	XLOC_11389.	transcript	0	0	0.013864	0.086275	0	0.092873	0.083407	0.093962	0
Novel_010740	XLOC_11389.	transcript	0.053266	0.08324	0	0.001566	0.224838	0.000752	0.152383	0.049314	0
Novel_010741	XLOC_11403.	transcript	0.087152	0.048261	0.030781	0	0.067779	0	0.116262	0	0.103516
Novel_010742	XLOC_11405.	transcript	0	1.023512	0	0.632213	0.595319	0.335503	0.103559	0.257463	0.372981

Novel_010743	XLOC_11405.	transcript	0.755353	0.565105	0.66228	0.5301	0.741016	0.751891	0.988845	0.57341	0.246868
Novel_010744	XLOC_11407.	transcript	0.59413	0.716833	0.718272	0.298768	0.572052	0.563056	0.839569	0.339387	2.473341
Novel_010745	XLOC_11409.	transcript	0.588527	0.533886	0.849946	0.774063	0.786343	0.95538	1.407323	0.862073	1.543605
Novel_010746	XLOC_11409.	transcript	0	0	0	0	0	0	0	0	0
Novel_010747	XLOC_11414.	transcript	0.02845	0.23458	0	0.073366	0	0	0.032734	0.050036	0
Novel_010748	XLOC_11417.	transcript	0.038464	0	0.048863	0.051605	0	0.018588	0	0.051032	0
Novel_010749	XLOC_11421.	transcript	0	0	0	0	0	0	0.454338	0.114684	0
Novel_010750	XLOC_11421.	transcript	0.099384	0.011129	0.023996	0.13761	0.109733	0.064423	7.90E-05	0.102146	0.108016
Novel_010751	XLOC_11421.	transcript	0.338118	0.324491	0.42485	0.203907	0.251047	0.242676	0.296989	0.200825	0.126839
Novel_010752	XLOC_11421.	transcript	0.231155	0.406055	0.183245	0.186373	0.17419	0.195949	0.274287	0.192382	0.354471
Novel_010753	XLOC_11421.	transcript	0.238797	0.300346	0.527984	0.477984	0.482731	0.44821	0.547723	0.455392	0.464841
Novel_010754	XLOC_11423.	transcript	0.207852	0.061277	0.14068	0.0656	0.015134	0.021176	0.225535	0.232147	0
Novel_010755	XLOC_11428.	transcript	0.703646	0.22847	0.475183	1.332662	0.50983	0.844947	0.50191	1.156019	0.39373
Novel_010756	XLOC_11428.	transcript	0.040924	0	0	0.301126	0.068697	0	0.071567	0	0.006862
Novel_010757	XLOC_11428.	transcript	0.257885	0	0.239203	0.048048	0.141506	0.102601	0.274832	0.057551	0
Novel_010758	XLOC_11429.	transcript	0.112825	0	0.437079	0.210097	0.319578	0.647058	0.147856	0.119711	0.222852
Novel_010759	XLOC_11429.	transcript	0	0	0.118424	0.082836	0.200493	0.375438	0.258718	0.167031	0
Novel_010760	XLOC_11430.	transcript	0.104702	0	0.151809	0.059183	0.245972	0.222406	0.11135	0.090504	0.035029
Novel_010761	XLOC_11430.	transcript	0.04979	0	0	0.244287	0.035121	0	0.012841	0.127333	0
Novel_010762	XLOC_11430.	transcript	0.294177	0	0.51891	0.153946	0.108924	0.416018	0.537409	0.4166	0.220582
Novel_010763	XLOC_11431.	transcript	0.016874	0	0	0	0	0	0.210347	0.360513	0.054446
Novel_010764	XLOC_11434.	transcript	0.435292	0.031882	0.03126	0	0.365362	0.070817	0	0.542261	0.430718
Novel_010765	XLOC_11434.	transcript	0.422686	0.322803	0.66365	0.631859	0.665862	0.493262	0.539669	0.795737	0.256002
Novel_010766	XLOC_11434.	transcript	0	0	0	0	0	0.221596	0.400099	0	0
Novel_010767	XLOC_11434.	transcript	0.141563	0	0.412721	0.114655	0.208858	0.149575	0.369248	0.055853	0.039941
Novel_010768	XLOC_11438.	transcript	0.373887	0	0.312319	0	0	0	0.060527	0	0
Novel_010769	XLOC_11438.	transcript	0	0	0	0.00614	0	0	0.056759	0.094302	0
Novel_010770	XLOC_11438.	transcript	0	0	0	0.080344	0	0	0.118555	0.056698	0
Novel_010771	XLOC_11438.	transcript	0	0.050482	0	0.22825	0.258288	0.575588	0.14157	0.163176	0.154418
Novel_010772	XLOC_11438.	transcript	0.005108	0.171507	0.301411	0.151989	0	0	0.107663	0.171215	0
Novel_010773	XLOC_11438.	transcript	0.003664	0	0	0	0	0	0	0	0
Novel_010774	XLOC_11438.	transcript	0	0.246607	0	0	0.00258	0	0	0	0
Novel_010775	XLOC_11438.	transcript	0	0	0	0.168883	0.520904	0	0.112937	0	0.499148
Novel_010776	XLOC_11438.	transcript	0	0	0	0	0	0	0	0.150025	0
Novel_010777	XLOC_11438.	transcript	0.03647	0.343946	0.001927	0	0	0.028033	0.128358	0.150999	0
Novel_010778	XLOC_11438.	transcript	0.638626	0.006485	0.141962	0.300265	0.016069	0.334039	0.131973	0.18888	0.335598
Novel_010779	XLOC_11438.	transcript	0.045679	0.602573	0.094533	0.016283	0.018261	0	0.310184	0.097728	0
Novel_010780	XLOC_11438.	transcript	0.319853	0	0.527045	0.005708	0.871549	0.15669	0.358237	0.294621	0

Novel_010781	XLOC_11438.	transcript	0.019511	0.559171	0	0.009396	0	0	0.352818	0.271382	0.00923
Novel_010782	XLOC_11438.	transcript	0	0.15602	0	0.211016	0.003041	0	0	0.083219	0
Novel_010783	XLOC_11439.	transcript	0.664092	0.783982	0.631496	0.539727	0.935611	0.591268	1.061109	0.877739	0.625398
Novel_010784	XLOC_11443.	transcript	0.621188	0.13351	0.498219	0.40881	0.361868	0.616756	0.39446	0.552745	0.305345
Novel_010785	XLOC_11443.	transcript	0.042129	0	0	0.112223	0.007491	0	0.024911	0.013785	0.016853
Novel_010786	XLOC_11444.	transcript	0.187346	0	0.001432	0.488084	0	0.177741	0.121872	0.279581	0
Novel_010787	XLOC_11444.	transcript	0.215685	0	0	0	0	0.207698	0.12583	0.240687	0.077131
Novel_010788	XLOC_11444.	transcript	0	0	0.191264	0	0.038834	0	0.134259	0.260965	0.482576
Novel_010789	XLOC_11444.	transcript	0	0	0	0	0	0	0	0.26038	0
Novel_010790	XLOC_11444.	transcript	0.824753	0.23118	0.900491	0.98747	1.103869	0.886277	1.178209	0.490814	0.437817
Novel_010791	XLOC_11445.	transcript	0	4.349206	0.273531	0.06298	0.162635	0.25471	0.14989	0.056824	0.34207
Novel_010792	XLOC_11445.	transcript	0.540743	0.617825	0.040669	0.147585	0.180949	0.093908	0.55013	0.196291	0.446347
Novel_010793	XLOC_11445.	transcript	18.20922	12.88125	6.377744	6.59127	6.662748	12.32439	11.13086	16.13388	6.678342
Novel_010794	XLOC_11447.	transcript	0.161245	0.132378	0.091315	0.189012	0.286246	0.135148	0.17134	0.213719	0.256077
Novel_010795	XLOC_11447.	transcript	0	0.153925	0	0	0.039846	0	0	0.048381	0
Novel_010796	XLOC_11448.	transcript	0.061747	0.159765	0.100407	0.021785	0.072176	0.063857	0.022404	0.068726	0.110675
Novel_010797	XLOC_11448.	transcript	0	0	0	0	0	0.149997	0	0.065935	0
Novel_010798	XLOC_11454.	transcript	0.198287	0.401519	0.156862	0.076132	0.169473	0.140891	0.220343	0.115604	0.228283
Novel_010799	XLOC_11457.	transcript	0	0	0	0.487253	0	0.179879	0	0.23811	0.147981
Novel_010800	XLOC_11457.	transcript	0	0	0	0	0	0.547055	0	0.573617	0
Novel_010801	XLOC_11457.	transcript	1.061715	1.138468	0.994816	0.304964	1.069048	0.910259	1.290995	0.457852	1.01982
Novel_010802	XLOC_11463.	transcript	0.425037	0.015095	0.179513	0.126619	0.116198	0.215174	0.219493	0.113899	0.19093
Novel_010803	XLOC_11474.	transcript	0	0.035101	0	0.028351	0	0	0	0	0
Novel_010804	XLOC_11474.	transcript	0	0	0	0	0	0	0	0	0
Novel_010805	XLOC_11474.	transcript	0.185932	0.041113	0.134609	0	0.070029	0	0.04226	0	0
Novel_010806	XLOC_11474.	transcript	0.169	0	0.177317	0.091722	0	0.111729	0.052444	0.03578	0
Novel_010807	XLOC_11474.	transcript	0.011349	0	0	0.095524	0	0	0	0.00114	0.021149
Novel_010808	XLOC_11474.	transcript	0	0	0	0	0	0	0.14284	0.176497	0
Novel_010809	XLOC_11474.	transcript	0	0.035686	0.025816	0.087997	0.004915	0.104899	0	0.019057	0.129399
Novel_010810	XLOC_11474.	transcript	0.080977	0.096486	0	0.016978	0	0	0.03002	0.01113	0.02508
Novel_010811	XLOC_11474.	transcript	0.045435	0.058029	0.138076	0.132713	0.159222	0.211988	0.147553	0.068569	0.03707
Novel_010812	XLOC_11474.	transcript	0.129819	0.031921	0.052713	0.017744	0	0.053506	0.150989	0.054822	0.243528
Novel_010813	XLOC_11474.	transcript	0.056512	0.055472	0.026726	0.037606	0.118813	0.043825	0.084852	0.075184	0.079922
Novel_010814	XLOC_11487.	transcript	0	0	0	0.427482	0	0	0	0.127203	0
Novel_010815	XLOC_11492.	transcript	0.771452	0.227562	0	0.212164	0.892875	0	0.018968	0.114321	0
Novel_010816	XLOC_11492.	transcript	0	0.680624	0.004429	0	0	0.023168	0	0.130844	0.009357
Novel_010817	XLOC_11492.	transcript	0	0	0	0.607543	0	0.000389	0	0.123792	0
Novel_010818	XLOC_11492.	transcript	0.377817	0.004251	0.716152	0.005278	0.159669	0.594694	0.817327	0.56391	0.683788

Novel_010819	XLOC_11494.	transcript	0	0.327403	0	0.164591	0.046746	0	0.218216	0.114926	0.133152
Novel_010820	XLOC_11494.	transcript	0.297813	0.741696	0.12	0.05321	0.223699	0.106717	0.08415	0.112775	0
Novel_010821	XLOC_11497.	transcript	0.013778	0	0	0.030413	0	0	0	0	0.010493
Novel_010822	XLOC_11497.	transcript	0	0	0	0	0	0	0	0	0.012672
Novel_010823	XLOC_11497.	transcript	0.107123	0.270772	0.057188	0.072084	0.059431	0.089533	0.144951	1.134061	0.053072
Novel_010824	XLOC_11497.	transcript	1.111574	0.00071	0.431388	0.757694	1.041487	0.114506	0.265283	0.017187	0.780903
Novel_010825	XLOC_11497.	transcript	0	0.02346	0.375165	0.064306	0	0.027962	0.000867	0.055174	0
Novel_010826	XLOC_11497.	transcript	0.028338	1.765943	0	0	0.032016	0.819856	1.149398	0	0
Novel_010827	XLOC_11497.	transcript	0.187529	0.17372	0.275569	0.102604	0.344806	0.141332	0.156019	0.178812	0.244862
Novel_010828	XLOC_11497.	transcript	0	0.004555	0	0	0	0	0.412436	0	0
Novel_010829	XLOC_11497.	transcript	0.749539	1.874596	1.209942	0.389752	1.134869	0.980869	0.448931	1.143393	1.388983
Novel_010830	XLOC_11508.	transcript	0	0.181172	0	0	0	0	0	0	0
Novel_010831	XLOC_11508.	transcript	0	0.432944	0	0.335528	0.024513	0.712106	0.973081	0	0.114242
Novel_010832	XLOC_11508.	transcript	0	0	0	0	0	0	0	0	0
Novel_010833	XLOC_11509.	transcript	0	0.395494	0	0	0.117302	0	0	0	0
Novel_010834	XLOC_11509.	transcript	0.622769	0.074408	0	0	0.900189	0	1.119756	0	3.638507
Novel_010835	XLOC_11510.	transcript	0	0.888406	0	0	0.176754	0.210622	0	0.063864	0.101597
Novel_010836	XLOC_11511.	transcript	0.156787	0	0.39691	0.338669	0.228607	0.058862	0.027503	0.069602	0
Novel_010837	XLOC_11511.	transcript	0.173087	0.743967	0.168265	0.283856	0.2333	0.417165	0.709955	0.570391	0.646559
Novel_010838	XLOC_11511.	transcript	0	0	0	0	0	0	0	0.106238	0
Novel_010839	XLOC_11516.	transcript	0.073465	0.036689	0.163603	0.050212	0.05391	0.1055	0.091129	0.064391	0.038658
Novel_010840	XLOC_11516.	transcript	0	0.013362	0.047032	0	0.007893	0.000334	0	0	0.055993
Novel_010841	XLOC_11516.	transcript	0.176231	0.125879	0.108568	0.061712	0.055113	0.227921	0.151485	0.104304	0.100095
Novel_010842	XLOC_11516.	transcript	0	0.122673	0.013588	0.036943	0.144076	0	0.179277	0.116684	0.060146
Novel_010843	XLOC_11518.	transcript	0.371814	0.346436	0.424183	0.30899	0.40382	0.299169	0.379053	0.741395	0.280635
Novel_010844	XLOC_11518.	transcript	0	0	0	0	0	0	0	0	0
Novel_010845	XLOC_11518.	transcript	0.070514	0.081204	0.027003	0.14356	0.122009	0.033246	0.088439	0.044692	0
Novel_010846	XLOC_11518.	transcript	0.35632	0.285149	0.135702	0.088417	0.086205	0.196465	0.293501	0.349499	0.228374
Novel_010847	XLOC_11521.	transcript	0.209601	0.022767	0.466951	0.447439	0.520113	0.450842	0.259753	0.392104	0.102628
Novel_010848	XLOC_11528.	transcript	0.528268	0.026532	0.279522	0.259012	0.408571	0.212333	0.249715	0.287557	0.176138
Novel_010849	XLOC_11528.	transcript	0	0	0	0	0	0	0.176294	0	0
Novel_010850	XLOC_11532.	transcript	0.050185	0	0.020791	0.161631	0	0.525288	0	0.197698	0.20943
Novel_010851	XLOC_11532.	transcript	0.091807	0.278471	0.246421	0.295837	0.126972	0.107504	0.097015	0.412788	0.34499
Novel_010852	XLOC_11532.	transcript	0.589655	0.452964	0.436087	0.216047	0.503014	0.028299	0.790958	0.277769	0
Novel_010853	XLOC_11532.	transcript	0.012828	0	0.138018	0.108962	0.051111	0.152463	0.095012	0.015196	0.100068
Novel_010854	XLOC_11532.	transcript	0.173131	0	0.011954	0	0.56064	0.044809	0.201615	0.060921	0.024857
Novel_010855	XLOC_11532.	transcript	0	0	0	0	0.204197	0	0	0.091695	0
Novel_010856	XLOC_11532.	transcript	0	0	0.183506	0.162074	0	0	0	0.003631	0

Novel_010857	XLOC_11532.	transcript	0	0	0	0	0	0	0	0	0
Novel_010858	XLOC_11532.	transcript	0	0	0.217237	0.163145	0	0	0.372832	0	0
Novel_010859	XLOC_11532.	transcript	0.536593	0	0.431985	0.133518	0	0.641452	0.247434	0.514988	0.522728
Novel_010860	XLOC_11537.	transcript	0.077499	0.007585	0.220928	0.060317	0.01142	0.043547	0.324333	0.14616	0.201108
Novel_010861	XLOC_11537.	transcript	0	0	0	0	0.103915	0	0.129783	0	0
Novel_010862	XLOC_11544.	transcript	0.534449	1.026354	0.311504	0.374421	0.342463	0.620696	0.632505	0.624785	0.901697
Novel_010863	XLOC_11547.	transcript	0.205975	0.138939	0	0.081718	0	0	0.044242	0	0.036869
Novel_010864	XLOC_11547.	transcript	0.032839	0	0	0.083366	0	0	0	0	0
Novel_010865	XLOC_11548.	transcript	0.433475	0.072033	0.268324	0	0.004915	0.005088	0	0	0.235418
Novel_010866	XLOC_11548.	transcript	0	0	0	0.376677	0.520742	0.311133	0.596932	0.375077	0
Novel_010867	XLOC_11548.	transcript	0	0	0	0	0.004754	0.020091	0.010046	0.008037	0.215209
Novel_010868	XLOC_11550.	transcript	0	0	0	0	0	0	4.642249	0	8.203333
Novel_010869	XLOC_11550.	transcript	10.46352	7.912165	11.17987	10.60793	11.79473	14.0891	11.00654	11.8863	13.62205
Novel_010870	XLOC_11550.	transcript	2.47082	1.581548	3.762331	2.726119	2.884499	3.477377	1.836735	3.008724	0.138585
Novel_010871	XLOC_11551.	transcript	0.006256	0.001937	0.152323	0.015815	0.01093	0.016362	0.004368	0.048679	0.049704
Novel_010872	XLOC_11551.	transcript	0.334584	0.117642	0.129365	0.330277	0.240694	0.47499	0.30495	0.347672	0.24365
Novel_010873	XLOC_11551.	transcript	0.175602	0.017768	0.124313	0.067331	0.142937	0.029955	0.365675	0.092599	0.051999
Novel_010874	XLOC_11554.	transcript	0.285652	0.371252	0.178473	0.203017	0	0.154695	0.136127	0.210591	0.269541
Novel_010875	XLOC_11554.	transcript	0.475296	0.332756	0.194346	0.175737	0.018714	0.17898	0.114561	0	0.003069
Novel_010876	XLOC_11554.	transcript	0.015219	0.423884	0.264385	0.17956	0.439434	0.180523	0.415321	0.383921	0.418972
Novel_010877	XLOC_11554.	transcript	0	0.007903	0.006493	0.010354	0.018758	0	0.001409	0	0.053777
Novel_010878	XLOC_11557.	transcript	0.035343	0.024986	0.19954	0.098059	0.061944	0.223213	0.051055	0.234482	0.064765
Novel_010879	XLOC_11559.	transcript	0.055027	0.29598	0	0.034386	0	0	0.09365	0.011949	0.296798
Novel_010880	XLOC_11565.	transcript	0.177899	0.061168	0.124559	0.025943	0.105653	0.085947	0.1518	0.072929	0.024511
Novel_010881	XLOC_11567.	transcript	0	0.026311	0	0	0	0	0	0	0
Novel_010882	XLOC_11567.	transcript	0.516006	0	0.001069	0	0.373841	0	0.004892	0	0
Novel_010883	XLOC_11567.	transcript	0	0	0	0	0	0	0	0	0
Novel_010884	XLOC_11567.	transcript	0.133614	0.241263	0	0	0.331705	0.130078	0.066756	0	0
Novel_010885	XLOC_11567.	transcript	0	0.243156	0.000299	0.242525	0	0.378708	0.000824	0.620262	0.104841
Novel_010886	XLOC_11567.	transcript	0	0	0.585141	0	0	0.004667	0.821119	0.17894	0
Novel_010887	XLOC_11569.	transcript	0.096545	0.542033	0	0	0	0	0.015379	0	0
Novel_010888	XLOC_11569.	transcript	0.071696	0.752108	0.086627	0.188692	0.07789	0.049431	0.026423	0.013245	0
Novel_010889	XLOC_11569.	transcript	0	0.346622	0.071407	0	0	0	0	0.13119	0.107732
Novel_010890	XLOC_11571.	transcript	0.087325	0.097257	0.079132	0.071457	0.063243	0	0.032064	0.064235	0.044353
Novel_010891	XLOC_11572.	transcript	0	0	0	0	0	0	0	0	0
Novel_010892	XLOC_11572.	transcript	0.325819	0.380896	0.472648	0.417093	0.465993	0.399198	0.573443	0.568499	0.408968
Novel_010893	XLOC_11572.	transcript	0.010697	0	0.015146	0.02796	0	0.017285	0.012681	0.024443	0.064464
Novel_010894	XLOC_11572.	transcript	0	0	0.035351	0	0.005889	0.00186	0.00149	0.007517	0.017847

Novel_010895	XLOC_11572.	transcript	0	0.004872	0.030358	0	0	0.010951	0	0	0.010389
Novel_010896	XLOC_11577.	transcript	0	0	0.083899	0.278377	0.113461	0.234425	0	0.109538	0.13986
Novel_010897	XLOC_11578.	transcript	0.060051	0	0	0	0	0.067301	0	0.067847	0.008856
Novel_010898	XLOC_11579.	transcript	0	0	0	0	0	0	0	0	0.340812
Novel_010899	XLOC_11579.	transcript	0	0.018096	0	0	0	0.022982	0	0	0
Novel_010900	XLOC_11579.	transcript	0.827809	0.409045	0.573112	0.310679	0.593229	0.675296	0.622315	0.612581	0.331203
Novel_010901	XLOC_11579.	transcript	0	0	0	0	0	0	0	0	0.158958
Novel_010902	XLOC_11579.	transcript	0.41636	0.385139	0.311558	0.264069	0.185698	0.195344	0.234593	0.267113	0
Novel_010903	XLOC_11579.	transcript	0.94874	0.483915	0.55171	0.613366	0.497966	0.444863	0.7091	0.796986	0
Novel_010904	XLOC_11579.	transcript	0.138312	0.021338	0.243221	0.154754	0.193341	0.027859	0.169929	0.194737	0.078584
Novel_010905	XLOC_11588.	transcript	0.108363	0.072966	0.052315	0.082296	0.074937	0.123925	0.14158	0.101061	0.21733
Novel_010906	XLOC_11606.	transcript	0.565077	0.399537	0.205279	0.193906	0.356088	0.271597	0.689431	0.414971	0.201043
Novel_010907	XLOC_11606.	transcript	0	0	0	0	0	0	0	0	0
Novel_010908	XLOC_11606.	transcript	0.100357	0.11091	0.064764	0.182666	0.169419	0.086987	0.095547	0.053746	0.104699
Novel_010909	XLOC_11609.	transcript	0	0.033267	0	0	0	0.048421	0.135379	0	0
Novel_010910	XLOC_11609.	transcript	0	0	0	0	0	0	0	0.020106	0
Novel_010911	XLOC_11609.	transcript	0.049559	0.088459	0.047274	0.065223	0.029483	0.029977	0.007244	0.013869	0.06695
Novel_010912	XLOC_11609.	transcript	0	0.605938	0.109698	0.057675	0.268556	0.371776	0	0	0.414416
Novel_010913	XLOC_11609.	transcript	0.309114	0.871424	2.044593	1.603945	1.253188	1.453475	1.639543	0.920305	1.642467
Novel_010914	XLOC_11609.	transcript	0.751248	0	0	0	0	0.56897	0.588452	1.037166	0.038088
Novel_010915	XLOC_11613.	transcript	1.017454	2.616173	1.028562	1.01797	1.014884	1.041183	0.938716	0.996919	1.104846
Novel_010916	XLOC_11614.	transcript	0.152265	0.009206	0.307085	0.334568	0.163464	0.657213	0.193837	0.432725	0.087221
Novel_010917	XLOC_11614.	transcript	0	0	0	0	0	0.040396	0.142125	0	0.097804
Novel_010918	XLOC_11614.	transcript	0.282803	0.222843	0.188523	0.183233	0.198448	0.131768	0.1054	0.369355	0.172049
Novel_010919	XLOC_11615.	transcript	0.20841	0	0.008766	0	0	0	0	0	0
Novel_010920	XLOC_11615.	transcript	0	0.852337	0.301198	0.250747	0.625963	0	0.535543	0.653967	0.782776
Novel_010921	XLOC_11615.	transcript	0.431049	0	0.390328	0.375057	0.022745	0.954202	0	0.000464	0
Novel_010922	XLOC_11615.	transcript	0	0.023442	0	0	0	0	0.252406	0	0
Novel_010923	XLOC_11616.	transcript	0.083467	0	0	0.035546	0	0.032996	0	0	0.196212
Novel_010924	XLOC_11631.	transcript	0.562404	0.692393	0.889587	2.027905	0.887232	1.477888	0.929843	1.133425	0.760488
Novel_010925	XLOC_11632.	transcript	0.066379	0.115719	0.303002	0.244646	0.354003	0.10154	0.186017	0.065604	0.106728
Novel_010926	XLOC_11632.	transcript	0.026859	0.068521	0.137117	0.232054	0.081298	0.151578	0.076017	0.263133	0.199282
Novel_010927	XLOC_11634.	transcript	0.247237	0.093396	0.118416	0.098949	0.13606	0.094797	0.333244	0.132243	0.113533
Novel_010928	XLOC_11634.	transcript	0.206849	0.212229	0.059225	0.361702	0.356005	0.228376	0.09602	0.158936	0.169898
Novel_010929	XLOC_11634.	transcript	0	0	0	0	0.000104	0.034078	0.254737	0	0
Novel_010930	XLOC_11634.	transcript	0.138002	0.024301	0.13758	0.131053	0.241758	0.123167	0.083174	0.25542	0.069497
Novel_010931	XLOC_11634.	transcript	0	0	0	0	0	0	0.001647	0.103842	0
Novel_010932	XLOC_11634.	transcript	0.127776	0.039787	0.18653	0.358821	0.163611	0.192712	0.237582	0.102014	0.141537

Novel_010933	XLOC_11635.	transcript	0.253652	0.174171	0.25455	0.364048	0.228607	0.448874	0.386369	0.248944	0.236229
Novel_010934	XLOC_11635.	transcript	0.127611	0.08104	0.220672	0.186314	0.232866	0.171796	0.192442	0.136168	0.094517
Novel_010935	XLOC_11636.	transcript	0	0.003855	0	0.001547	0.254531	0.009875	0.001782	0.002336	0
Novel_010936	XLOC_11636.	transcript	0.259268	0.288198	0.671378	0.209412	0.458281	0.226621	1.030182	0.542284	0.750701
Novel_010937	XLOC_11636.	transcript	0.013441	0.190725	0.001468	0.009507	0.228014	0.072891	0.156025	0.122373	0.256949
Novel_010938	XLOC_11636.	transcript	0	0.16495	0.397702	0.070212	0.022667	0	0	0	0
Novel_010939	XLOC_11666.	transcript	0.084262	0.210031	0.102473	0.182503	0.179895	0.14381	0.232476	0.255656	0.22084
Novel_010940	XLOC_11667.	transcript	0.181452	0	0.275071	0.23562	0.274823	0.100692	0.467469	0.144088	0.417724
Novel_010941	XLOC_11667.	transcript	0	0.0799	0	0	0	0.129249	0	0.171059	0
Novel_010942	XLOC_11668.	transcript	0.095348	0.218459	0.085817	0.113311	0.047369	0.032265	0.070827	0.080583	0
Novel_010943	XLOC_11670.	transcript	0.719966	0.535914	0.914418	0.963462	0.521667	0.457647	1.395213	1.12557	0.986849
Novel_010944	XLOC_11673.	transcript	0.581991	0.801755	0.598141	0.729603	0.526067	0.88464	0.617188	0.739412	0.565427
Novel_010945	XLOC_11678.	transcript	0.881773	0.477595	1.545902	1.147445	1.237727	1.113115	1.385275	0.980424	1.04036
Novel_010946	XLOC_11678.	transcript	0	0	0	0	0	0	0	0	0
Novel_010947	XLOC_11679.	transcript	0.071946	0.560958	0.028104	0.027663	0.054377	0.008018	0.134563	0.045127	0.145587
Novel_010948	XLOC_11680.	transcript	0.799218	8.547378	0.538804	0.309889	0.510888	0.401076	1.336648	0.415337	2.34008
Novel_010949	XLOC_11680.	transcript	0.293195	0	0.163405	0.097155	0.126513	0.300496	0.152391	0.231571	0.103384
Novel_010950	XLOC_11680.	transcript	0.419948	0	0	0	0	0.050376	0.173947	0.263981	0
Novel_010951	XLOC_11680.	transcript	0.023603	0	0.020995	0.018609	0	0	0	0	0
Novel_010952	XLOC_11680.	transcript	0.370162	0.270986	0.387603	0.370668	0.320731	0.253544	0.509396	0.363295	0.287244
Novel_010953	XLOC_11688.	transcript	0.326697	0.278801	0.347266	0.17791	0.469667	0.429248	0.687986	0.314729	0.211128
Novel_010954	XLOC_11688.	transcript	78.34747	123.1038	86.48508	85.832	78.6433	92.91138	103.8506	93.1639	140.538
Novel_010955	XLOC_11691.	transcript	0.533813	0	0	0	0.41607	0	0.361806	0.40726	0.035119
Novel_010956	XLOC_11691.	transcript	0	0	0	0	0	0	0	0	0
Novel_010957	XLOC_11691.	transcript	0.001737	0.039887	0.329472	0.180524	0	0.113081	0.001778	0.001937	0.102467
Novel_010958	XLOC_11691.	transcript	0.285156	0.453033	0.516335	0.34481	0.192262	0.318909	0.86621	0.463777	0.625841
Novel_010959	XLOC_11691.	transcript	0	0	0	0	0	0	0.286953	0	0
Novel_010960	XLOC_11691.	transcript	0.157858	0.01869	0.856376	0.478266	0.018065	0.359305	0	0.56961	0.685485
Novel_010961	XLOC_11691.	transcript	3.031416	3.351475	4.386187	4.179855	2.964606	3.574555	4.734734	6.541278	6.327587
Novel_010962	XLOC_11692.	transcript	0.573385	0.211562	0.20751	0.164311	0.322448	0.255151	0.725049	0.47141	0.180977
Novel_010963	XLOC_11693.	transcript	0.841245	0.189556	0.659664	0.890323	0.751312	1.119714	0.790211	0.836689	0.734033
Novel_010964	XLOC_11693.	transcript	0.082106	0	0.34601	0.082979	0.366472	0.136726	0.305984	0.167745	0.32929
Novel_010965	XLOC_11693.	transcript	0.305564	0	0.452743	0.410546	0	0	0.53449	0.131466	0.621707
Novel_010966	XLOC_11693.	transcript	0.421368	0	0	0	0.160612	0	0	0.117294	0
Novel_010967	XLOC_11693.	transcript	0.086605	0	0.30522	0.024282	0	0.151709	0	0.240683	0
Novel_010968	XLOC_11694.	transcript	0	0	0.222965	0.20365	0	0.168687	0.228093	0.235152	0.109724
Novel_010969	XLOC_11694.	transcript	0.053548	0.02962	0	0	0.109658	0.02156	0.0049	0	0.018118
Novel_010970	XLOC_11697.	transcript	0	0.134211	0.271332	0	0.035731	0.346492	0.377603	0.003267	0

Novel_010971	XLOC_11697.	transcript	0	0	0	0	0	0	0	0	0
Novel_010972	XLOC_11697.	transcript	0.422002	0.048526	0.009159	0.230284	0.327762	0.158535	0	0.28313	0.376917
Novel_010973	XLOC_11697.	transcript	0	0.128962	0.539263	0	0	0.310274	0.449733	0.245774	0
Novel_010974	XLOC_11697.	transcript	0	0.04386	0	0	0	0	0	0	0
Novel_010975	XLOC_11701.	transcript	0	0	0.003012	0	0	0	0.001244	0	0
Novel_010976	XLOC_11701.	transcript	0.347124	1.02255	0.19183	0.189592	0.222366	0.258435	0.421399	0.170593	0.790452
Novel_010977	XLOC_11707.	transcript	0	0	0	0	0	0.072686	0	0	0
Novel_010978	XLOC_11707.	transcript	0.053465	0.001428	0.011043	0.198146	0.034443	0.080905	0.012056	0	0
Novel_010979	XLOC_11707.	transcript	0.188827	0.26386	0.229845	0.182149	0.260863	0.255936	0.396442	0.474074	0.267388
Novel_010980	XLOC_11708.	transcript	0.307155	0.313302	0.227929	0.169296	0.289481	0.17169	0.219086	0.271999	0.172251
Novel_010981	XLOC_11708.	transcript	0	0.220371	0	0	0.010949	0.395858	0	0.275279	0.028258
Novel_010982	XLOC_11709.	transcript	0.391311	0.292969	0.54491	0.36277	0.282506	0.500717	0.462481	0.421647	0.262519
Novel_010983	XLOC_11709.	transcript	0.455506	1.216012	0.305714	0.285881	0.259764	0.32599	0.401392	0.362099	0.425955
Novel_010984	XLOC_11709.	transcript	0	0.668327	0	0	0	0	0	0	0
Novel_010985	XLOC_11710.	transcript	0.311809	0.102839	0.375812	0.290909	0.151496	0.142779	0.188963	0.166821	0.357587
Novel_010986	XLOC_11710.	transcript	0	0	0	0	0	0.001725	0.569525	0	0.277131
Novel_010987	XLOC_11710.	transcript	0	0.56605	0	0	0	0.634482	0	0.719529	0
Novel_010988	XLOC_11710.	transcript	0.543129	0.264454	0.463036	0.483294	0.537457	0.046523	0.141825	0.12127	0
Novel_010989	XLOC_11715.	transcript	0.112282	0.033188	0.042382	0.120103	0.102424	0.044259	0.216303	0.10096	0.046336
Novel_010990	XLOC_11734.	transcript	1.233301	0.057069	0.81906	0.608593	0.503429	0.585863	2.008667	1.698377	0.525962
Novel_010991	XLOC_11740.	transcript	0.352513	0.480028	0.447749	0.290337	0.515249	0.608338	0.492843	0.435904	0.539263
Novel_010992	XLOC_11740.	transcript	0	0	0	0.47894	0	0	0	0.534612	0
Novel_010993	XLOC_11740.	transcript	1.011662	0	1.013312	0.593257	1.524438	0	0.647651	0.405724	0
Novel_010994	XLOC_11740.	transcript	0	0	0	0	0	0	0	0	0
Novel_010995	XLOC_11740.	transcript	0.004246	0	0	0	0.011144	0	0.001337	0	0
Novel_010996	XLOC_11762.	transcript	0.006953	0.010393	0.130392	0	0.008691	0.012978	0.021613	0.004251	0.013159
Novel_010997	XLOC_11762.	transcript	0.338739	0.272262	0.291952	0.504382	0.226941	0.643696	0.37984	0.366432	0.332049
Novel_010998	XLOC_11762.	transcript	0	0	0	0.012384	0	0	0	0.000437	0
Novel_010999	XLOC_11762.	transcript	0.442495	0	0.195149	0.042035	0.138441	0.420553	0.013009	0.139948	0.012295
Novel_011000	XLOC_11762.	transcript	0.022029	0.133857	0.15733	0.189551	0.224302	0.028631	0.356636	0.094382	0.340124
Novel_011001	XLOC_11762.	transcript	0	0	0	0.226581	0	0	0	0.202172	0
Novel_011002	XLOC_11763.	transcript	0.259845	0.33026	0.173467	0.311752	0.366903	0.283914	0.471141	0.141077	0.301921
Novel_011003	XLOC_11763.	transcript	0	0	0	0	0	0	0	0.293066	0.030519
Novel_011004	XLOC_11763.	transcript	0	0	0.068761	0	0	0	0	0	0
Novel_011005	XLOC_11763.	transcript	0	0	0	0	0	0	0	0	0
Novel_011006	XLOC_11763.	transcript	0	0	0	0	0	0	0	0	0
Novel_011007	XLOC_11764.	transcript	0.787005	0.273529	0.107359	0.211278	0.405411	0.258858	0.663387	0.295216	0.330957
Novel_011008	XLOC_11765.	transcript	0.195683	0	0	0.000288	0	0	0	0.032883	0.283786

Novel_011009	XLOC_11765.	transcript	0	0.255083	0	0	0.226903	0	0.008682	0	0
Novel_011010	XLOC_11765.	transcript	0	0.390148	0.211079	0.153952	0	0.204767	0.263021	0.150275	0
Novel_011011	XLOC_11765.	transcript	0	0	0	0	0	0	0	0.00839	0
Novel_011012	XLOC_11765.	transcript	0	0	0	0	0.005662	0.010035	0.004119	0.027885	0.015075
Novel_011013	XLOC_11765.	transcript	0	0	0	0.010145	0	0	0	0	0
Novel_011014	XLOC_11767.	transcript	0	0.051182	0.410874	0	0.36476	0.405002	0.020993	0	0
Novel_011015	XLOC_11767.	transcript	0	0.582769	0.027552	0	0.042595	0	0.004296	0	0
Novel_011016	XLOC_11767.	transcript	0.070608	1.827803	0.041076	0.017453	0.090138	0.087937	0.21252	0.142918	0.030808
Novel_011017	XLOC_11769.	transcript	0	1.198121	0	0	0	0	0	0	0
Novel_011018	XLOC_11770.	transcript	0.195701	0.543815	0.102452	0.174289	0.182991	0.146276	0.368858	0.327911	0.19335
Novel_011019	XLOC_11771.	transcript	2.524485	0.224375	1.748048	1.938779	1.790272	2.354518	2.861746	2.732344	1.727984
Novel_011020	XLOC_11771.	transcript	0	0	0	0	0	0	0	0	0
Novel_011021	XLOC_11777.	transcript	0.179632	0.372415	0.176581	0.190641	0.227809	0.278706	0.24846	0.314143	0.271725
Novel_011022	XLOC_11777.	transcript	0	0.087878	0	0.104266	0	0	0	0.063944	0
Novel_011023	XLOC_11780.	transcript	1.034444	0.85921	0.968386	0.772212	0.823312	1.01714	0.582028	0.849014	0.630783
Novel_011024	XLOC_11780.	transcript	0	0	0	0	0.179287	0	0.359301	0.23861	0
Novel_011025	XLOC_11780.	transcript	0.052295	0.005824	0.233298	0.038252	0.033616	0.114831	0.06738	0.044109	0.018724
Novel_011026	XLOC_11780.	transcript	0.676046	0.191837	1.304019	2.396313	2.30718	1.068109	1.556491	2.137758	0.914395
Novel_011027	XLOC_11780.	transcript	0	0	0	0	0	0	0	0	0
Novel_011028	XLOC_11782.	transcript	0.213836	0.121235	0.149379	0.189338	0.126776	0.240597	0.329685	0.131223	0.223608
Novel_011029	XLOC_11782.	transcript	0	0	0	0	0	0	0.065926	0.193161	0
Novel_011030	XLOC_11782.	transcript	1.28532	1.383908	1.283317	2.001803	2.253546	1.613652	2.60374	2.043131	1.721459
Novel_011031	XLOC_11782.	transcript	0.47365	0.873086	1.364236	0	0	1.535057	0	0.686934	0.860766
Novel_011032	XLOC_11782.	transcript	0.082442	0.022576	0	0	0	0.128006	0.107847	0	0
Novel_011033	XLOC_11782.	transcript	0.21035	0.143979	0.051416	0.034806	0.145574	0.188103	0.152565	0.09666	0.24505
Novel_011034	XLOC_11782.	transcript	0	0	0	0	0	0	0	0	0
Novel_011035	XLOC_11782.	transcript	0	0	0.141039	0.136378	0.076582	0	0	0.226474	0.138606
Novel_011036	XLOC_11782.	transcript	0	0	0.203734	0.099938	0.112063	0.073404	0	0.149568	0.096616
Novel_011037	XLOC_11783.	transcript	0	0.540012	0.710684	0.754648	0.265171	0.885927	0.391498	0.615633	0.843603
Novel_011038	XLOC_11783.	transcript	0.109105	1.43819	0.016558	0.00878	0.122035	0.071825	0.191268	0.05302	0.165743
Novel_011039	XLOC_11783.	transcript	0.358539	1.940251	0.289219	0.305051	0.450948	0.380098	0.46739	0.278113	0.386747
Novel_011040	XLOC_11783.	transcript	0	0.330452	0.561986	0	0	0	0.930022	0.717541	0
Novel_011041	XLOC_11784.	transcript	0	0.160945	0.057047	0.170825	0	0.053901	0.079949	0.03055	0.165174
Novel_011042	XLOC_11784.	transcript	0	0	0	0	0	0.000333	0.097884	0.099362	0.164345
Novel_011043	XLOC_11784.	transcript	0.075352	0.000471	0.181038	0.198321	0.135749	0.116459	0.047266	0.102055	0.212722
Novel_011044	XLOC_11784.	transcript	0	0.150987	0.073536	0.070325	0	0.078429	0.016329	0.045392	0.085941
Novel_011045	XLOC_11784.	transcript	0	0.114899	0	0	0.062874	0.078846	0	0	0
Novel_011046	XLOC_11786.	transcript	0.135208	0.122903	0	0.376668	0	0.701434	0.040455	0	0

Novel_011047	XLOC_1178€.	transcript	0.059224	0.021046	0	0.141698	0	0.232189	0	0	0
Novel_011048	XLOC_11794.	transcript	0.030825	0.064966	0.013105	0.082802	0.097474	0.041379	0.143395	0.092194	0.029235
Novel_011049	XLOC_11797.	transcript	0.122375	0.040598	0.044255	0.089549	0.081683	0.114835	0.298139	0.115736	0.057078
Novel_011050	XLOC_11799.	transcript	0	0.661898	1.890265	1.377441	1.653435	1.670381	0	0.294433	0
Novel_011051	XLOC_11801.	transcript	0.243666	0.126885	0.121392	0.132	0.148655	0.080439	0.353875	0.12945	0.237533
Novel_011052	XLOC_11803.	transcript	0	0	1.012086	0	0	0	0	0	0
Novel_011053	XLOC_11803.	transcript	0.555807	1.792403	0.301026	0.799483	0.791489	1.148852	1.092999	0.977015	1.538028
Novel_011054	XLOC_11803.	transcript	0.092512	0.024005	0.348619	0.289976	0.005209	0.195561	0.715667	0.08534	0.081362
Novel_011055	XLOC_11803.	transcript	0.032175	0.034609	0.400546	0.031551	0.331671	0	0.090254	0.095693	0.050604
Novel_011056	XLOC_11812.	transcript	0.763762	0	0	0	0	0	0	0.819993	0
Novel_011057	XLOC_11812.	transcript	0.076122	0.136347	0.610163	0.553684	0.682981	0.646446	0.930897	0.194626	0.546438
Novel_011058	XLOC_11812.	transcript	0.004412	0	0	0	0	0.017741	0	0.004781	0.018288
Novel_011059	XLOC_11812.	transcript	0.058162	0	0.263923	0.149363	0	0.267159	0.278756	0.156462	0.21931
Novel_011060	XLOC_11815.	transcript	0.724295	0.195001	0.727131	0.59558	0.807662	0.535525	1.374093	0.609101	0.264504
Novel_011061	XLOC_11820.	transcript	0.555416	2.145597	0.694042	1.091766	0.760822	0.882781	0.713544	1.102262	1.154219
Novel_011062	XLOC_11820.	transcript	0.084718	0.026225	0.190106	0.148542	0.236101	0.130167	0.186582	0.157106	0.184927
Novel_011063	XLOC_11820.	transcript	0.247801	0.396645	0.225622	0.206211	0.201114	0.262076	0.33258	0.342162	0.078215
Novel_011064	XLOC_11820.	transcript	0.099569	0.146818	0.011889	0.13249	0.088456	0.062076	0.058147	0.129666	0.041866
Novel_011065	XLOC_11823.	transcript	0	0	0	0.016134	0	0	0	0	0
Novel_011066	XLOC_11823.	transcript	0.231658	0.1001	0.221618	0.093882	0.189264	0.174833	0.240758	0.190208	0.183267
Novel_011067	XLOC_11824.	transcript	0.783479	0.324699	1.066531	0.669077	0.756044	0.799378	1.482304	1.199236	0.338267
Novel_011068	XLOC_11824.	transcript	0.101139	0	0.027224	0.059905	0.012575	0.388074	0.1732	0.078983	0.06029
Novel_011069	XLOC_11826.	transcript	0.266878	0	0	0	0	0	0	0	0
Novel_011070	XLOC_11826.	transcript	0.015421	0.235146	0.26029	0.377229	0.463599	0.213775	0.31068	0.336501	0.232666
Novel_011071	XLOC_11827.	transcript	0.378094	0.143217	0.249592	0.53113	0.453007	0.386381	0.506646	0.558158	0.408856
Novel_011072	XLOC_11832.	transcript	0	0.289965	0	0	0	0.242231	0	0.050852	0
Novel_011073	XLOC_11832.	transcript	0.491082	0	0	0	0.291569	0.891188	0.492922	0	0
Novel_011074	XLOC_11837.	transcript	0.027711	0.068899	0	0	0	0.116906	0.020724	0	0
Novel_011075	XLOC_11839.	transcript	0.07478	0	0.009066	0.242989	0.072913	0	0.256005	0	0.008179
Novel_011076	XLOC_11839.	transcript	0	0.106772	0.000707	0	0	0.277263	0	0.133465	0
Novel_011077	XLOC_11839.	transcript	0.004769	0	0.159478	0	0	0.263284	0	0.13482	0.089341
Novel_011078	XLOC_11839.	transcript	0.502034	0	0.195985	0	0.180616	0.31531	0.111625	0.148052	0.343549
Novel_011079	XLOC_11839.	transcript	0.814286	0.407125	0.731365	0.595894	0.611355	0.30593	1.092907	0.592953	0.110554
Novel_011080	XLOC_11839.	transcript	2.623187	0	0	1.660598	2.027672	0	2.968801	0	0
Novel_011081	XLOC_11839.	transcript	0.261977	0.059854	0.162752	0.085757	0.153381	0.511352	0.734426	0.387262	0.166795
Novel_011082	XLOC_11839.	transcript	0.741472	0.094796	0.404969	0.383823	0.415248	0	0.496619	0.325533	0.260039
Novel_011083	XLOC_11839.	transcript	0.359681	0.01593	0.045472	0.049644	0.059249	0.043005	0.089976	0.05934	0.029015
Novel_011084	XLOC_11839.	transcript	0.071391	0.002447	0.018677	0.007227	0.006307	0.017072	0.099241	0.016367	0.005294

Novel_011085	XLOC_11841.	transcript	0.098176	0	0.112512	0.22945	0.108304	0.089672	0.207062	0.077653	0
Novel_011086	XLOC_11848.	transcript	0.001028	0	0	0	0	0	0.000949	0.00112	0
Novel_011087	XLOC_11848.	transcript	0.000365	0	0	0	0	0	0	0.000618	0
Novel_011088	XLOC_11848.	transcript	0.004487	0.002799	0.000758	0.004945	0.006089	0.017824	0.00804	0.028377	0
Novel_011089	XLOC_11848.	transcript	0.148698	0.231033	0.53029	0.328418	0.314427	0.50235	0.162544	0.213343	0.620124
Novel_011090	XLOC_11848.	transcript	0.57741	0	0	0.375299	0.431082	0.002222	0.043468	0.786519	0
Novel_011091	XLOC_11848.	transcript	0	0	0.010351	0	0	0.017606	0.313465	0.000458	0.027483
Novel_011092	XLOC_11848.	transcript	0	0	0	0	0	0	0	0	0
Novel_011093	XLOC_11848.	transcript	0	0.005677	0	0	0	0	0.75915	0.006401	0
Novel_011094	XLOC_11848.	transcript	0.151543	0.134621	0.027278	0	0	0	0	0	0
Novel_011095	XLOC_11848.	transcript	0	0.009909	0	0.019047	0	0.01761	0.276423	0.009427	0.016406
Novel_011096	XLOC_11848.	transcript	0	0	0	0	0	0	0	0	0
Novel_011097	XLOC_11848.	transcript	0.001124	0	0	0	0	0.00883	0	0	0
Novel_011098	XLOC_11850.	transcript	0.488844	0.149038	0.546905	0.42104	0.592182	0.318312	0.754027	0.731681	0.645611
Novel_011099	XLOC_11855.	transcript	0	0.349559	0	0	0.149499	0	0.278189	0.113684	0.303079
Novel_011100	XLOC_11862.	transcript	0.013055	0	0.600954	0.022827	0	0.027062	0.016775	0	0.038752
Novel_011101	XLOC_11862.	transcript	0.444633	0	0.139566	0	0.515624	0	0	0	0
Novel_011102	XLOC_11868.	transcript	0.44868	0.34066	0.259181	0.117218	0.581396	0.392182	0.306843	0.154188	0.246195
Novel_011103	XLOC_11870.	transcript	0	0.032554	0.166103	0.235197	0.158062	0.047391	0.14326	0.044977	0.226387
Novel_011104	XLOC_11873.	transcript	0.211018	0.830039	0.272387	0	0.320798	0.225009	0.337476	0.298559	0
Novel_011105	XLOC_11875.	transcript	0.444369	0	0.287737	0.237082	0.348934	0.228702	0.360386	0.240135	0.112968
Novel_011106	XLOC_11875.	transcript	1.041701	0.658087	0.952433	0.673632	0.968415	0.69851	1.519316	0.855549	0.598354
Novel_011107	XLOC_11875.	transcript	0.411186	0.093038	0.274153	0.12141	0.196623	0.253796	0.290564	0.160083	0.224872
Novel_011108	XLOC_11876.	transcript	0.126689	0	0.073572	0.142541	0.025566	0.118174	0.271829	0.194788	0.027515
Novel_011109	XLOC_11876.	transcript	0.078359	0.01896	0.0485	0.165614	0.163784	0.051213	0.11289	0.065604	0.133571
Novel_011110	XLOC_11876.	transcript	0	0	0	0	0	0.169003	0	0	0
Novel_011111	XLOC_11876.	transcript	0.190113	0.039708	0.17626	0.157429	0.174167	0	0.145515	0.206833	0.093517
Novel_011112	XLOC_11885.	transcript	0.242393	0.566645	0.25353	0.231474	0.347544	0.458672	0.55222	0.453665	0.303343
Novel_011113	XLOC_11890.	transcript	0.534146	0.457872	0.594308	0.541098	0.63522	0.455923	0.895451	0.665836	1.291897
Novel_011114	XLOC_11891.	transcript	0.811798	0.383221	0.582856	0.670616	0.631176	0.654693	0.743487	0.516133	0.428775
Novel_011115	XLOC_11895.	transcript	0.227735	0.28424	0	0.457788	0	0	0	0	0
Novel_011116	XLOC_11895.	transcript	0.523281	0.867443	0.529454	0.559394	0.723247	0.751445	0.954051	0.629952	0.676251
Novel_011117	XLOC_11895.	transcript	0	0	0	0	0	0	0	0.577354	0
Novel_011118	XLOC_11896.	transcript	0.259756	0.532179	0.687154	0.651223	0.460132	0.44195	0.448688	0.457737	0.806161
Novel_011119	XLOC_11897.	transcript	0	0.925481	0	0.470928	0.297672	0.534845	0.701464	0.280467	0.497782
Novel_011120	XLOC_11897.	transcript	0.242831	0.202517	0.06696	0	0.437111	0.12865	0.117761	0.479645	0.005805
Novel_011121	XLOC_11898.	transcript	1.528178	0.103118	1.114148	1.816352	1.047828	0.214307	1.351863	0.921546	1.327367
Novel_011122	XLOC_11898.	transcript	0.902457	0.249122	0.902406	0.144718	1.038009	0.265008	1.26093	0.69218	1.12127

Novel_011123	XLOC_11898	transcript	0.050094	0.055188	0.054879	0.088515	0.07073	0.133991	0.086569	0.06308	0.178027
Novel_011124	XLOC_11902	transcript	0.030952	0.032724	0	8.30E-05	0	0	0.01045	0.130902	0
Novel_011125	XLOC_11902	transcript	0	0	0.300352	0.005561	0	0	0	0.32376	0.069996
Novel_011126	XLOC_11902	transcript	0.442594	0	0.228456	0.141533	0.441381	0.202166	0.478586	0.473456	0.352963
Novel_011127	XLOC_11903	transcript	0.341518	0	0.055628	0.070491	0.023686	0.264683	0.104028	0.082201	0.031054
Novel_011128	XLOC_11917	transcript	0.245091	0.113393	0.029742	0.612708	0.014056	0.525899	0.301958	0.206588	0.832316
Novel_011129	XLOC_11917	transcript	0.09258	0.367955	0.966603	0.272611	0.589124	0.014261	0.19683	0.007183	0.015812
Novel_011130	XLOC_11917	transcript	0.010374	0.002674	0	0.00029	0	0	0.007026	0.091526	0
Novel_011131	XLOC_11917	transcript	0.637306	0.890235	0	0	0	0.905922	0.818255	0.510763	0
Novel_011132	XLOC_11918	transcript	0.072589	0.053495	0.082904	0.005833	0.067005	0.079351	0.194514	0.085249	0
Novel_011133	XLOC_11918	transcript	0	0	0	0	0	0	0	0	0
Novel_011134	XLOC_11918	transcript	0.121282	0.038744	0.070408	0.118479	0.043738	0.052184	0.058011	0.100129	0.08793
Novel_011135	XLOC_11918	transcript	0.072664	0.034307	0.07008	0	0.04226	0.043483	0.222167	0.095694	0
Novel_011136	XLOC_11918	transcript	0.121746	0.07044	0.086441	0.118587	0.097946	0.120571	0.075895	0.130447	0.077247
Novel_011137	XLOC_11918	transcript	0.134189	0.029821	0.068618	0.078927	0.043566	0.041557	0.31855	0.068859	0.115728
Novel_011138	XLOC_11918	transcript	0.069279	0.039676	0.065943	0	0.046353	0.096272	0.088969	0.070946	0
Novel_011139	XLOC_11918	transcript	0.094116	0.052164	0.064057	0.122978	0.047425	0.05661	0.041327	0.060587	0.106342
Novel_011140	XLOC_11918	transcript	0.158434	0.081115	0.066281	0.176197	0.05453	0.074471	0.054358	0.098677	0.097913
Novel_011141	XLOC_11918	transcript	0.107387	0.071286	0.082248	0.120778	0.301445	0.201452	2.90E-05	0.338785	0.09682
Novel_011142	XLOC_11918	transcript	0	0.053028	0.108037	0	0	0.107351	0.043143	0	0.154456
Novel_011143	XLOC_11918	transcript	0.059131	0.046713	0.059571	0.051281	0.060106	0.041449	0.043187	0.061761	0.048785
Novel_011144	XLOC_11918	transcript	0.092869	0.080795	0.061186	0.070715	0.06573	0.06453	0.052746	0.085376	0.050604
Novel_011145	XLOC_11918	transcript	0.06646	0.039476	0.06095	0	0.04724	0.044973	0.066972	0.047499	0
Novel_011146	XLOC_11918	transcript	0.13767	0.190159	0.247382	0.020991	0.175959	0	0.102395	0.160533	0
Novel_011147	XLOC_11918	transcript	0	0	0	0	0	0	0	0	0
Novel_011148	XLOC_11920	transcript	0.479876	0.201713	0.310834	0.464327	0.362222	0.342922	0.486546	0.576987	0.316517
Novel_011149	XLOC_11920	transcript	0	0.146344	0.028554	0	0	0	0.159486	0	0
Novel_011150	XLOC_11922	transcript	0.109655	0.412127	0.050717	0.133201	0.192197	0	0.086065	0.141281	0.197592
Novel_011151	XLOC_11922	transcript	0.254341	0.196742	0.073389	0.099971	0.039175	0.60445	0.04746	0.091629	0.166821
Novel_011152	XLOC_11922	transcript	0.315061	0.977102	0.649978	0.615978	0.35954	0.609063	0.599779	0.571572	0.284876
Novel_011153	XLOC_11923	transcript	0.941521	0.266517	0.732054	0.4923	0.638178	0.751249	0.792465	0.484956	0.699127
Novel_011154	XLOC_11923	transcript	0	0.040691	0	0.113608	0	0	0.054392	0	0.324345
Novel_011155	XLOC_11923	transcript	0	0	0	0	0	0	0	0.145476	0
Novel_011156	XLOC_11923	transcript	0.022726	0	0	0	0	0.18328	0	0	0
Novel_011157	XLOC_11926	transcript	0.019351	0	0.382384	0.130507	0.27994	0.252125	0.093708	0.179186	0.185468
Novel_011158	XLOC_11935	transcript	0.097083	0	0	0.777675	0	0.008769	0	0	0
Novel_011159	XLOC_11935	transcript	0	0.070086	0.576514	0	0.711563	0	0.892851	0.08991	0.824898
Novel_011160	XLOC_11935	transcript	0.2976	0	0	0	0	0	0	0.152944	0

Novel_011161	XLOC_11935.	transcript	0.10432	0.330317	0.328895	0.049293	0.382493	0	0	0.511941	0.084301
Novel_011162	XLOC_11936.	transcript	0.34345	0.314521	0.293521	0.216193	0.088076	0.685681	0.357798	0.338131	0.135496
Novel_011163	XLOC_11936.	transcript	0.020886	0	0.195699	0.032318	0.101369	0.018255	0.117321	0.076256	0.029891
Novel_011164	XLOC_11936.	transcript	0	0	0.161995	0.578472	0.678788	0	0.133766	0	0.742506
Novel_011165	XLOC_11936.	transcript	0	0.364901	0.558276	0.515061	0	0.346822	0	0.458333	0.026457
Novel_011166	XLOC_11936.	transcript	0	0	0	0	0.253462	0.074166	0	0	0
Novel_011167	XLOC_11936.	transcript	0.355793	0.057387	0.076051	0.078499	0.02951	0	0.370611	0.049725	0.27648
Novel_011168	XLOC_11936.	transcript	0	0.554178	0.59161	0.689098	0.63263	0.621863	0.663695	0.560751	0.575597
Novel_011169	XLOC_11937.	transcript	0.012399	0	0	0	0	0.015225	0	0.08623	0
Novel_011170	XLOC_11937.	transcript	0.163962	0.958084	0.186973	0.254181	0.167559	0.165008	0.200097	0.226676	0.183798
Novel_011171	XLOC_11938.	transcript	0	0	0	0	0	0	0	0	0
Novel_011172	XLOC_11938.	transcript	0.055967	0.024881	0.04108	0.669082	0.035748	0.046453	0.013512	0.016893	0.052175
Novel_011173	XLOC_11941.	transcript	0.36453	0	0.110215	4.30054	0.185351	0.251563	0.369439	0.819422	34.83706
Novel_011174	XLOC_11941.	transcript	2.137312	3.425706	3.602281	4.092274	4.054774	5.197219	4.08876	4.183959	3.440975
Novel_011175	XLOC_11941.	transcript	0.160953	0.065247	0.358655	0.233559	0.020204	0.311745	0.194711	0.288418	0.004281
Novel_011176	XLOC_11941.	transcript	0.07685	0.098155	0.205214	0.240745	0.040573	0.035997	0.170834	0.303928	0.007188
Novel_011177	XLOC_11941.	transcript	0.02339	0	0.135078	0.012808	0.002123	0.279068	0.27448	0.743894	0.127897
Novel_011178	XLOC_11941.	transcript	0.188987	0.171551	0.310886	0.060134	0.037502	0	0.002116	0.540229	0.003941
Novel_011179	XLOC_11941.	transcript	0	0	0	0	0	0.017433	0	0	0
Novel_011180	XLOC_11941.	transcript	0.079606	0.03974	0.150016	0.238538	0.052595	0.020628	0.138248	0.128692	0.524384
Novel_011181	XLOC_11941.	transcript	1.794872	0.916397	1.75073	0	0.477194	0	0.157143	1.123851	0.512441
Novel_011182	XLOC_11941.	transcript	0	0	0	0.014965	0.52638	2.199261	0.203275	0	0.087102
Novel_011183	XLOC_11941.	transcript	0.114967	0.092281	0.116465	0	0.078845	0	0.076946	0.227357	0
Novel_011184	XLOC_11941.	transcript	0	0	0	2.229782	1.587171	0	1.81013	0	1.102681
Novel_011185	XLOC_11941.	transcript	0.934827	0.785923	0.807261	0.690596	0.682523	0.577516	1.09195	0.829851	0.697085
Novel_011186	XLOC_11942.	transcript	0	0	0.015225	0	0.039354	0	0.185522	0.150921	0
Novel_011187	XLOC_11942.	transcript	0.127308	0	0	0	0	0.016331	0.015458	0.02264	0.021232
Novel_011188	XLOC_11950.	transcript	0.023988	0.029857	0	0.001689	0	0.07987	0.062932	0	0.055365
Novel_011189	XLOC_11950.	transcript	0.02838	0.068656	0.143459	0.109285	0.013864	0.031844	0.073969	0.006435	0.22345
Novel_011190	XLOC_11952.	transcript	0.006278	0.003249	0.026419	0.096604	0	0	0.001707	0	0.571961
Novel_011191	XLOC_11952.	transcript	0	0	0.055729	0.031036	0.072455	0	0.327698	0.187235	0.016146
Novel_011192	XLOC_11952.	transcript	0.0959	0.696853	0	0.027263	0.017636	0.050468	0.388692	0.090433	0.521854
Novel_011193	XLOC_11952.	transcript	0.109078	0.250236	0.116424	0	0.103507	0.021493	0.184048	0	0.391633
Novel_011194	XLOC_11952.	transcript	0.288133	0.369716	0	0	0.132172	0.057756	0.363864	0.017031	1.191709
Novel_011195	XLOC_11953.	transcript	0.17164	0.073896	0.264537	0.356445	0.142897	0.240616	0.669296	0.262895	0.510817
Novel_011196	XLOC_11953.	transcript	0.898292	1.164678	0.72913	0.31475	0.207723	0.762667	0.590751	0.294151	0.753178
Novel_011197	XLOC_11953.	transcript	0	0	0	0	0.431309	0	0	0	0
Novel_011198	XLOC_11953.	transcript	0	0	0	0.277122	0.157418	0	0	0.160894	0.227365

Novel_011199	XLOC_11954.	transcript	1.065837	0.126412	0.07918	1.127865	0.281972	0.234531	0.791971	0.395266	0.140533
Novel_011200	XLOC_11954.	transcript	0.867956	0.087125	0.764817	0.680164	1.043852	0.77266	1.341889	1.485636	0.591828
Novel_011201	XLOC_11954.	transcript	0.510888	1.850276	0.594548	0.507537	0.693913	0.862952	1.171003	0.472803	0.662384
Novel_011202	XLOC_11955.	transcript	1.998535	1.546074	1.649561	1.611588	1.741815	1.697212	2.069945	2.026006	1.315383
Novel_011203	XLOC_11955.	transcript	0.384924	0.393137	0.407677	0.366426	0.322853	0.363183	0.424714	0.28679	0.273253
Novel_011204	XLOC_11955.	transcript	0	0.199394	0.11473	0.179143	0.215555	0.10999	0.108548	0.087057	0.095538
Novel_011205	XLOC_11955.	transcript	0	0	0	0	0	0	0	0.220518	0
Novel_011206	XLOC_11955.	transcript	0	0	0	0	0	0	0	0.097514	0
Novel_011207	XLOC_11956.	transcript	0.800362	0.670103	0.297799	0.916582	0.554365	0.942417	0.228415	0.354036	0.345701
Novel_011208	XLOC_11956.	transcript	0.157805	0.05999	0.672777	0	0.507841	0	0.822748	0.420731	0.650453
Novel_011209	XLOC_11958.	transcript	0	0	0.569261	0	0	0	0	0.005178	0
Novel_011210	XLOC_11958.	transcript	0.104045	0.004619	0.028697	0.462595	0.010498	0.050517	0.682419	0.558216	0
Novel_011211	XLOC_11958.	transcript	0	0	0.078473	0	0	0	0	0	0.596142
Novel_011212	XLOC_11958.	transcript	0.336689	0.620595	0.408852	0.704612	1.201422	1.526064	0.392906	0.916171	0
Novel_011213	XLOC_11958.	transcript	0.751781	1.089965	0.88851	0.552051	1.298023	0.932206	0.555217	0.260555	0.93379
Novel_011214	XLOC_11958.	transcript	0	0	0	0.66965	0	0	0	0	0
Novel_011215	XLOC_11961.	transcript	0.353212	0.785297	0.40508	0.422729	0.421537	0.329056	0.855627	0.356437	0.338831
Novel_011216	XLOC_11961.	transcript	0.413724	0.303906	0.310636	0.572813	0.478307	0.349844	0.246066	0.484877	0.455245
Novel_011217	XLOC_11962.	transcript	0	0	0.04048	0.133139	0.202044	0	0.058859	0.079195	0
Novel_011218	XLOC_11963.	transcript	3.345725	4.333589	6.032145	8.027636	6.264567	13.96706	5.903962	5.539234	11.36441
Novel_011219	XLOC_11964.	transcript	1.09644	2.134185	1.368178	1.879171	1.483524	1.723895	1.758297	2.148959	1.377672
Novel_011220	XLOC_11964.	transcript	0	0	0.005173	0.005229	0.008224	0	0	0.127517	0
Novel_011221	XLOC_11964.	transcript	0.03743	0.144049	0.219791	0.151263	0.192937	0.139691	0.350998	0.297032	0.208049
Novel_011222	XLOC_11964.	transcript	0.084253	0.145574	0.197737	0.166138	0.182947	0.308984	0.32242	0.015873	0.13393
Novel_011223	XLOC_11964.	transcript	0	0	0	0	0	0	0	0.233641	0.032765
Novel_011224	XLOC_11964.	transcript	0.888931	1.501424	1.202394	1.53835	1.261525	1.357318	1.457579	1.235635	1.426994
Novel_011225	XLOC_11965.	transcript	0.29297	0.187365	0.523009	0.384624	0	0	0.607979	0.477108	0.165817
Novel_011226	XLOC_11965.	transcript	0	0	0.058741	0	0.31768	0.049805	0.028746	0.044123	0.050606
Novel_011227	XLOC_11965.	transcript	0	0	0	0	0	0.451889	0	0	0
Novel_011228	XLOC_11965.	transcript	0.098772	0.303771	0.166599	0.259327	0.272497	0.223008	0.406024	0.423093	0.171195
Novel_011229	XLOC_11966.	transcript	0	0.062325	0.22192	0.10815	0.253638	0.213283	0.089938	0.086763	0.100181
Novel_011230	XLOC_11973.	transcript	0.035235	0.830596	0.051115	0.006772	0.008292	0	0.076348	0.004626	0.057203
Novel_011231	XLOC_11974.	transcript	0.73139	0.217575	0.351047	0.265596	0.231451	0.525272	0.552448	0.349413	0.39556
Novel_011232	XLOC_11974.	transcript	0.670538	1.120124	0.726799	1.162783	1.254652	0.851067	1.626882	1.530489	0.445675
Novel_011233	XLOC_11975.	transcript	13.78876	10.1036	15.38043	18.75753	16.30382	13.67107	19.28952	16.22904	17.59401
Novel_011234	XLOC_11975.	transcript	3.983895	3.201036	2.704659	2.782995	3.357075	1.848564	3.359609	4.121752	4.298461
Novel_011235	XLOC_11975.	transcript	3.5133	6.483572	4.470212	3.46115	4.713857	3.260741	5.767096	6.398922	5.030008
Novel_011236	XLOC_11975.	transcript	1.591354	1.850242	3.478997	1.999907	2.995215	2.82701	1.253465	4.062704	2.765396

Novel_011237	XLOC_11976.	transcript	0.261485	0	0	0.047663	0.029529	0.205683	0	0.062348	0.121936
Novel_011238	XLOC_11976.	transcript	0.346247	0.331583	0.291672	0.471597	0.374151	0.407623	0.654206	0.597011	0.520351
Novel_011239	XLOC_11976.	transcript	0	0.00303	0	0	0	0	0	0	0.003056
Novel_011240	XLOC_11976.	transcript	0	0	0.337926	0.091553	0	0.437792	0.10915	0	0
Novel_011241	XLOC_11976.	transcript	0	0.300185	0	0.114059	0.029813	0	0.090903	0.171721	0.026687
Novel_011242	XLOC_11976.	transcript	0	0	0	0	0.247708	0	0.087564	0	0.262592
Novel_011243	XLOC_11976.	transcript	0.348571	0.307851	0	0.120236	0.001539	0	0.087395	0.187054	0.014617
Novel_011244	XLOC_11977.	transcript	0	0	0	0	0	0	0	0.426696	0
Novel_011245	XLOC_11977.	transcript	0.502664	0.420809	0.721585	0.328129	0.898448	0.309622	0.700982	0.652385	0.600726
Novel_011246	XLOC_11977.	transcript	0.15673	0.147807	0.372967	0.201132	0.162412	0.141327	0.177764	0.122048	0.241979
Novel_011247	XLOC_11978.	transcript	0.148037	0.010399	0.29196	0.270888	0.268506	0.178684	0.138435	0.272696	0.196418
Novel_011248	XLOC_11981.	transcript	0	0	0.719489	0.379616	0.548109	0.060666	0.842564	1.010514	0.189994
Novel_011249	XLOC_11981.	transcript	0.99769	0.118985	6.20E-05	0.492852	0.205819	0.491826	4.30E-05	0.173943	0.090262
Novel_011250	XLOC_11984.	transcript	0.015623	0.826199	0.128996	0.168036	0.127139	0.392761	0.303674	0.154567	0.121467
Novel_011251	XLOC_11984.	transcript	0.391137	0.436123	0.433717	0.555313	0.460744	0.349966	0.453311	0.444923	0.239051
Novel_011252	XLOC_11986.	transcript	0.891262	1.560087	0.876349	0.869644	1.038524	0.789164	1.326959	1.241208	0.442485
Novel_011253	XLOC_11988.	transcript	0.062543	0.062409	0.145191	0.617946	0.085084	0.148394	0.166405	0.135398	0.082423
Novel_011254	XLOC_11990.	transcript	0.121717	0.369187	0.300422	0.06998	0.080223	0.110202	0.222009	0.106564	0.19244
Novel_011255	XLOC_11992.	transcript	1.550363	1.414139	0.57424	1.122653	0.987923	1.282457	1.073943	1.089548	1.485645
Novel_011256	XLOC_11992.	transcript	0.874659	1.490584	1.039976	0.842286	0.362909	0.467105	1.27962	1.003942	0.276983
Novel_011257	XLOC_11992.	transcript	0	0.331481	0	0	0.395181	0	0	0	0.309006
Novel_011258	XLOC_11992.	transcript	0.042811	0.047812	0.000828	0.026215	0.02187	0	0.057351	0.001418	0.04459
Novel_011259	XLOC_11992.	transcript	0.000838	0.182594	0.366757	0.004221	0.005763	0	0.06632	0	0
Novel_011260	XLOC_11992.	transcript	0.282978	0.053712	0.702615	0.735841	0.878756	0.725272	0.807695	0.702966	0.725417
Novel_011261	XLOC_11993.	transcript	0.392082	0.388619	0.437706	0.440964	0.558879	0.518801	0.651942	0.445902	0.370895
Novel_011262	XLOC_11998.	transcript	0.249107	0.077116	0.481602	0.164205	0.428423	0.10004	0.222934	0.059398	0.32814
Novel_011263	XLOC_11998.	transcript	0	0.040357	0	0.003217	0	0.014397	0.005705	0.104914	0.254281
Novel_011264	XLOC_11998.	transcript	0	0.002488	0	0	0.047197	0	0.193946	0.01051	0
Novel_011265	XLOC_11998.	transcript	0.026387	0	0	0.222963	0	0.437977	0.248668	0.32063	0.121072
Novel_011266	XLOC_11998.	transcript	0	0	0	0	0	0	0	0	0
Novel_011267	XLOC_11999.	transcript	0.426008	0.694467	0.839685	0.734471	0.789937	0.580818	0.8134	0.679432	0.887971
Novel_011268	XLOC_12001.	transcript	0	0	0	0	0	0	0	0.360573	0.024328
Novel_011269	XLOC_12004.	transcript	0.957192	0.66184	1.808373	0.256569	1.546641	0	1.057516	1.273528	0
Novel_011270	XLOC_12004.	transcript	0	0	0	0	0	2.301708	0	0	0.354031
Novel_011271	XLOC_12006.	transcript	0.10575	0.051963	0.207475	0.317955	0.191203	0.359321	0.105309	0.322417	0.166999
Novel_011272	XLOC_12007.	transcript	0.634763	1.324237	0.859415	0.552092	0.794044	0.816882	1.20188	0.912073	0.508869
Novel_011273	XLOC_12010.	transcript	1.40876	2.568408	1.922116	2.085359	1.502847	1.945177	1.688923	1.694179	3.454347
Novel_011274	XLOC_12010.	transcript	0	0	0	0	0	0	0	0.268896	0

Novel_011275	XLOC_12011.	transcript	0.315336	0.246271	0.17158	0.544567	0.343824	0.430374	0.484176	0.350815	0.270449
Novel_011276	XLOC_12012.	transcript	2.114007	2.818071	1.287873	1.629727	1.604815	1.307743	2.702686	2.424312	1.603187
Novel_011277	XLOC_12012.	transcript	0.546816	1.290552	0.876398	1.056696	0.648014	0.828	0.903067	0.345135	1.590691
Novel_011278	XLOC_12012.	transcript	0	0	0	0	0	0	0	0.454267	0
Novel_011279	XLOC_12013.	transcript	1.247604	0.624753	0.777439	0.745503	0.830925	0.87876	1.006962	0.042577	0.271217
Novel_011280	XLOC_12013.	transcript	0.219054	0.8614	0.525035	0.905425	0.255752	0.733812	0.441595	0.371264	0.848037
Novel_011281	XLOC_12013.	transcript	0	0	0	0	0	0	0	0.471843	0
Novel_011282	XLOC_12013.	transcript	0.066817	0.450081	0.333262	0.575638	0.331942	0.303661	0.431831	1.491195	0.490718
Novel_011283	XLOC_12015.	transcript	3.512441	3.932915	4.141169	4.901752	4.467763	5.428889	5.058803	5.575826	6.177466
Novel_011284	XLOC_12022.	transcript	0.042189	0.644258	0.031944	0.12782	0.354046	0.172719	0.094001	0.075333	0.178413
Novel_011285	XLOC_12022.	transcript	0.488656	0.333411	0.218831	0.29166	0.122774	0.150014	0.151526	0.126598	0.237895
Novel_011286	XLOC_12022.	transcript	0	0	0	0.108834	0	0	0	0	0
Novel_011287	XLOC_12023.	transcript	0.330823	0.21429	0.635128	0.630982	0.05972	0.028601	0.084513	0.020276	0.421878
Novel_011288	XLOC_12023.	transcript	0	0	0	0	0	0	0	0	0
Novel_011289	XLOC_12023.	transcript	0	0	0	0	0.54972	0	0.568765	0	0
Novel_011290	XLOC_12023.	transcript	0	0	0	0	0	0.532058	0.055002	0.623241	0
Novel_011291	XLOC_12023.	transcript	0.009299	0	0.018716	0.011973	0.025008	0.384642	0	0.004264	0
Novel_011292	XLOC_12023.	transcript	0.06148	0	0.002964	0	0	0	0	0	0.874966
Novel_011293	XLOC_12024.	transcript	0.169844	0.561856	0.22043	0.250207	0.209403	0.327719	0.367697	0.275302	0.225535
Novel_011294	XLOC_12025.	transcript	0	0.157334	0.02684	0	0.01977	0.183783	0	0.072677	0.018704
Novel_011295	XLOC_12025.	transcript	0.013431	0.15304	0.022513	0	0	0	0	0.012114	0.204872
Novel_011296	XLOC_12025.	transcript	0.100561	0.288844	0.148816	0	0.045392	0.079964	0.033192	0	0.033691
Novel_011297	XLOC_12030.	transcript	1.489262	1.494136	1.463989	1.84652	1.592893	1.898892	2.682463	1.806748	1.283457
Novel_011298	XLOC_12030.	transcript	0.256809	0.109886	0.263362	0.093386	0.162419	0.303601	0.354037	0.356101	0.561667
Novel_011299	XLOC_12030.	transcript	0.013855	0	0.125671	0	0	0.015623	0.010328	0.086405	0.111994
Novel_011300	XLOC_12030.	transcript	0.063565	0	0.013263	0	0.108453	0.113506	0.006677	0.191554	0.036748
Novel_011301	XLOC_12032.	transcript	0.016497	0.164825	0	0.113167	0.523998	0	0.251801	0.346015	0.328575
Novel_011302	XLOC_12032.	transcript	0.234983	0.147661	0.110001	0.289545	0.157951	0.12238	0.317281	0.321316	0.145167
Novel_011303	XLOC_12032.	transcript	0.525249	0.709957	0.795291	0.657178	0.60375	0.909275	0.976577	0.586748	0.480104
Novel_011304	XLOC_12040.	transcript	0.193522	0	0	0	0	0.064174	0.526552	0	0
Novel_011305	XLOC_12040.	transcript	0.038896	0.214807	0.648518	0.450458	0.542278	0.347914	0	0.708502	1.187577
Novel_011306	XLOC_12040.	transcript	0	0	0	0	0	0	0.704479	0	0
Novel_011307	XLOC_12041.	transcript	0.223349	0.35731	0.451461	0.517826	0.412968	0.427116	0.625277	0.517343	0.541497
Novel_011308	XLOC_12042.	transcript	0.00361	0	0.004113	0	0	0	0.328635	0.005001	0
Novel_011309	XLOC_12042.	transcript	0.559995	0.366577	0.504333	0.109417	0.543082	0.551306	0.449939	0.00015	0.439068
Novel_011310	XLOC_12042.	transcript	0.024318	0	0	0.65487	0.018962	0	0.618026	0	0
Novel_011311	XLOC_12042.	transcript	0.00149	0	0	0	0	0	0.000825	0.940314	0
Novel_011312	XLOC_12050.	transcript	0.030674	0.043135	0.078212	0.166632	0.165183	0.206334	0.129087	0.181646	0.250157

Novel_011313	XLOC_12050.	transcript	0.153301	0.09448	0.120411	0.624546	0.248653	0.022387	0.069161	0.107202	0
Novel_011314	XLOC_12050.	transcript	1.06749	0.972728	0.871918	0.831855	1.127786	1.000815	1.276257	0.908033	1.019753
Novel_011315	XLOC_12051.	transcript	1.400864	0.929756	1.639223	1.746153	1.872859	2.279456	2.404725	1.439175	2.090373
Novel_011316	XLOC_12051.	transcript	0.036934	0.016025	0.150425	0.020982	0.026351	0.002825	0.043059	0.027551	0.039162
Novel_011317	XLOC_12051.	transcript	0.143559	2.216799	0	0.116901	0.161339	0.014314	0.28805	0.293842	0.326746
Novel_011318	XLOC_12051.	transcript	0.007164	0.063525	0.071845	0.108829	0.075339	0.064364	0.066988	0.110589	0.040687
Novel_011319	XLOC_12055.	transcript	0	0	0.053073	0.256859	0.077017	0.348067	0.111507	0.021557	0.500939
Novel_011320	XLOC_12055.	transcript	0	0	0	0	0	0	0	0	0
Novel_011321	XLOC_12055.	transcript	0.727247	0.508988	0.734758	0.718525	0.44905	0.526188	0.887367	0.794614	0.799375
Novel_011322	XLOC_12060.	transcript	0.052571	0.167883	0.125596	0.12343	0.054633	0.203949	0.221304	0.112986	0.228161
Novel_011323	XLOC_12060.	transcript	0.185471	0.272388	0.262213	0.109219	0.098339	0.167021	0.336382	0.280019	0
Novel_011324	XLOC_12060.	transcript	0	0	0.002884	0.000486	0	0.068122	0.001085	0.058135	0.020802
Novel_011325	XLOC_12060.	transcript	0.207912	0.35734	0.007977	0.053114	0.251842	0.033674	0.000976	0.059197	0.020634
Novel_011326	XLOC_12060.	transcript	0	0	0.08412	0.050094	0	0.038262	0.078667	0.020642	0.01033
Novel_011327	XLOC_12061.	transcript	0.105665	0	0.113434	0.082723	0.256347	0.187965	0.055284	0.100118	0
Novel_011328	XLOC_12061.	transcript	0.234894	0.201141	0.333765	0.625027	0.437046	0.277785	0.314569	0.577857	0.296234
Novel_011329	XLOC_12061.	transcript	0	0.190236	0	0	0.005303	0.233244	0.058366	0	0
Novel_011330	XLOC_12061.	transcript	0	0.002285	0	0	0.011065	0	0.009189	0.000895	0
Novel_011331	XLOC_12061.	transcript	0	0	0	0	0	0.195525	0	0	0
Novel_011332	XLOC_12061.	transcript	0.489679	0.397919	0.367007	0	0.234549	0.139369	0.5217	0.510225	0.437966
Novel_011333	XLOC_12062.	transcript	1.13519	0	1.888855	0.309329	1.226772	0.072158	1.135022	1.383693	1.212086
Novel_011334	XLOC_12062.	transcript	0	1.246867	0	0	0.045034	0.10276	0.011801	0.033555	0
Novel_011335	XLOC_12062.	transcript	0	0	0.033306	0.002977	0.004974	0.108719	0.341243	0	0
Novel_011336	XLOC_12062.	transcript	0	0.147936	0	1.324976	0	0.960259	0	0	0
Novel_011337	XLOC_12062.	transcript	0.578981	0.773652	0.594949	0.678617	0.511162	0.480974	1.06638	0.81765	0.367677
Novel_011338	XLOC_12063.	transcript	0.517974	0.008092	0	0	0	0	0	0	0.096806
Novel_011339	XLOC_12063.	transcript	0	0	0.505056	0.367684	0.652198	1.8145	0.989183	0.852431	0.275903
Novel_011340	XLOC_12063.	transcript	0	0	0	0	0	0	0	0	0
Novel_011341	XLOC_12065.	transcript	0.294679	0.5837	0.346291	0.045768	0.130456	0.381038	0.541631	0.564056	0.479041
Novel_011342	XLOC_12065.	transcript	0	0	0	0	0.095896	0	0	0	0
Novel_011343	XLOC_12065.	transcript	0.061021	0.00671	0	0.333569	0	0.034466	0.023118	0.003947	0.0122
Novel_011344	XLOC_12065.	transcript	0	0	0.004021	0.029185	0	0	0	0	0
Novel_011345	XLOC_12065.	transcript	0.061649	0.059703	0.128053	0.010722	0.311436	0.03951	0.1603	0.150556	0.030791
Novel_011346	XLOC_12072.	transcript	0	0.934827	0	0.020838	0	0.045193	0.347367	0	0
Novel_011347	XLOC_12072.	transcript	0	0	0	0	0	0	0.16329	0	0.001224
Novel_011348	XLOC_12072.	transcript	0.290356	0.94124	0.386532	0.303774	0.364485	0.362783	0.408245	0.311678	0.534556
Novel_011349	XLOC_12073.	transcript	0.008596	0.002835	0.140367	0.206032	0.004399	0.002347	0.010206	0.011548	0.139097
Novel_011350	XLOC_12073.	transcript	0.009211	0.02511	0.223734	0.202319	0.002104	0.00373	0.065746	0.24073	0.385187

Novel_011351	XLOC_12073.	transcript	0.741846	0.230207	0.112393	0.13713	0.118835	0.273157	0.296534	0.256106	0
Novel_011352	XLOC_12073.	transcript	0	0.215986	0.047118	0.014663	0.106693	0.232662	0.256469	0.174605	0.013326
Novel_011353	XLOC_12073.	transcript	0.068351	0.502462	0.228362	0.127363	0.540524	0.239605	0.527943	0.22177	0.16327
Novel_011354	XLOC_12075.	transcript	0.073936	0.071729	0.213235	0.062139	0.069187	0.074487	0.164161	0.134333	0.095242
Novel_011355	XLOC_12077.	transcript	0	0.000672	0	0	0.017567	0	0.000606	0	0.00096
Novel_011356	XLOC_12077.	transcript	0.299648	0.428955	0.437153	0.696815	0.313474	0.378476	0.601099	0.695066	0.391411
Novel_011357	XLOC_12080.	transcript	0.160013	0.60148	0.421176	0.461136	0.322646	0.051194	0.477224	0.158324	0.013341
Novel_011358	XLOC_12080.	transcript	0.063515	0.07075	0.17185	0.087567	0.136582	0.492018	0.057232	0.077633	0.293102
Novel_011359	XLOC_12080.	transcript	0.288468	0.150459	0	0.246022	0.1769	0.210793	0.196272	0.217289	0.332234
Novel_011360	XLOC_12080.	transcript	0.224834	0.351959	0.493077	0.466853	0.010711	0.349346	0.572057	0.312719	0.126802
Novel_011361	XLOC_12089.	transcript	0.006322	0.008294	0.0078	0.003046	0.94536	0.051165	0.043881	0.017867	0
Novel_011362	XLOC_12089.	transcript	0.792627	1.033556	0.011695	0	0.005192	0	0.032129	0.131462	0.014952
Novel_011363	XLOC_12089.	transcript	0	0	0	0.010776	0	0	0.008068	0.858936	0
Novel_011364	XLOC_12090.	transcript	0.37183	0.031459	0.502156	0.366935	0.537534	0.405268	0.740038	0.358933	0.283854
Novel_011365	XLOC_12090.	transcript	0.608953	0	0.472327	0.208693	0.537211	0.672673	0.880397	0.579191	0.213192
Novel_011366	XLOC_12092.	transcript	0.040399	0.003075	0.037269	0.023994	0.18402	0.04556	0.4983	0.01784	0.005497
Novel_011367	XLOC_12092.	transcript	0.504147	0.01592	0.55576	0.243327	0.312381	0.443945	0.63778	0.266893	0.247829
Novel_011368	XLOC_12092.	transcript	0.222241	0.008331	0.181143	0.146819	0.134441	0.212754	0.41925	0.119749	0.080651
Novel_011369	XLOC_12092.	transcript	0.040141	0	0.06922	0.026552	0.014189	0	0.042941	0	0
Novel_011370	XLOC_12093.	transcript	0.420471	0	0.264553	0.227636	0.13675	0.273958	0.477198	0.13602	0.126407
Novel_011371	XLOC_12096.	transcript	0.1226	0	0.31183	0.067507	0.158688	0.098271	0.104453	0.090692	0.054285
Novel_011372	XLOC_12097.	transcript	0.239856	0	0.171561	0.216067	0.07265	0.273764	0.388052	0.025923	0.046117
Novel_011373	XLOC_12098.	transcript	0.211521	0.676612	0.168137	0.160703	0.049415	0.081377	0.336711	0.117254	0.12824
Novel_011374	XLOC_12102.	transcript	0.22021	0.103549	0.247429	0.286275	0.301554	0.187581	0.443508	0.356844	0.186694
Novel_011375	XLOC_12102.	transcript	0	0	0	0	0	0	0	0	0
Novel_011376	XLOC_12102.	transcript	0.023086	0	0.009572	0	0	0	0.084715	0	0
Novel_011377	XLOC_12102.	transcript	0	0	0	0	0	0	0.106679	0	0
Novel_011378	XLOC_12102.	transcript	0.098538	0.027814	0.05072	0.03845	0.085639	0	0.003096	0.067187	0.039772
Novel_011379	XLOC_12102.	transcript	0.124955	0.14078	0	0.166431	0	0.208744	0.211642	0.25078	0.271248
Novel_011380	XLOC_12102.	transcript	0.014402	0	0.309739	0.01272	0.231387	0.040677	0.114516	0	0
Novel_011381	XLOC_12102.	transcript	0	0	0	0	0.028172	0	0.300231	0.015919	0
Novel_011382	XLOC_12102.	transcript	0.341457	0.311258	0.337667	0.366868	0.623112	0.399894	0.441377	0.564771	0.03103
Novel_011383	XLOC_12106.	transcript	0.006252	1.272346	0.01706	0.260258	0.234516	0.149364	1.270445	0	0.614119
Novel_011384	XLOC_12106.	transcript	0	0	0	0.384135	0.232501	0.06289	0	0	0.380158
Novel_011385	XLOC_12106.	transcript	0.584065	1.189504	0.848764	0	0.102024	0.71321	0.045083	0.815711	0.019804
Novel_011386	XLOC_12106.	transcript	0	0	0	0	0	0	0	0	0
Novel_011387	XLOC_12108.	transcript	0.072146	0.601511	0.097516	0.111092	0.082678	0.284916	0.145594	0.146358	0.126476
Novel_011388	XLOC_12110.	transcript	0	0.277388	0.415656	0.048039	0.326639	0	0.084766	0.05562	0.193123

Novel_011389	XLOC_12110.	transcript	0.622785	0.204726	0.178355	0.411311	0.389588	0.556769	0.891416	0.528117	0.392487
Novel_011390	XLOC_12110.	transcript	0	0	0	0	0	0	0	0	0
Novel_011391	XLOC_12110.	transcript	0.018201	0.04288	0.012939	0	0.419608	0	0.133206	0	0.202276
Novel_011392	XLOC_12110.	transcript	0	0	0	0	0	0	0.353674	0	0
Novel_011393	XLOC_12110.	transcript	0	0	0.005409	0	0	0	0.213604	0	0
Novel_011394	XLOC_12111.	transcript	0.250024	0.551911	0.162541	0.228173	0.07738	0.389054	0.136059	0.342947	0.134119
Novel_011395	XLOC_12114.	transcript	0.02898	0	0	0	0	0.053802	0	0.124216	0
Novel_011396	XLOC_12114.	transcript	0.245624	0	0.100451	0.111796	0.043173	0.060451	0.072916	0.171552	0.057687
Novel_011397	XLOC_12120.	transcript	0.523617	0	0.067849	0.032917	0.415426	0	0.063997	0.176388	0.025353
Novel_011398	XLOC_12121.	transcript	0.174578	0	0	0	0.185926	0.18786	0	0.144028	0
Novel_011399	XLOC_12122.	transcript	0	0	0.045546	0	0	0	0.180993	0	0
Novel_011400	XLOC_12122.	transcript	0.143058	0.00476	0.206738	0.178677	0.064042	0.087519	0.31147	0.217692	0.149563
Novel_011401	XLOC_12122.	transcript	0	0	0	0	0	0	0	0	0
Novel_011402	XLOC_12123.	transcript	0	0.061335	0.442883	0.475517	0.403744	0.055868	0.857899	0.560861	0.509744
Novel_011403	XLOC_12123.	transcript	0.479941	0.004777	0.07004	0.161011	0.048663	0	0.044974	0.102024	0.01411
Novel_011404	XLOC_12123.	transcript	0.562108	0	0.019499	0	0.019942	0.587846	0.011056	0	0.009294
Novel_011405	XLOC_12124.	transcript	4.50E-05	0.075295	0.01051	0.371688	0	0.477593	0.037388	1.80E-05	0.001637
Novel_011406	XLOC_12124.	transcript	1.373074	0.025277	0	0.048091	0	0	0	0	0
Novel_011407	XLOC_12124.	transcript	0	0.137514	0.281593	0.297608	0.125822	0.128117	0.259076	0.755242	0.077389
Novel_011408	XLOC_12124.	transcript	0.027317	0	0	0.015212	0	0.011715	0.066031	0.050878	0
Novel_011409	XLOC_12133.	transcript	0.632452	0	0.246504	0.173066	0.246214	0.231119	0.351939	0.304701	0.275406
Novel_011410	XLOC_12135.	transcript	0.385931	0.028925	0.173295	0.428342	0.315516	0.137091	0.239362	0.530858	0.027839
Novel_011411	XLOC_12135.	transcript	0.834685	0	0.391204	0.350049	0.522725	0	0.379642	0.238777	0.331716
Novel_011412	XLOC_12135.	transcript	0.552817	0	0.333109	0.157592	0	0.759057	0.509255	0.443089	0.21768
Novel_011413	XLOC_12136.	transcript	0	0	0	0	0	0	0	0	0
Novel_011414	XLOC_12136.	transcript	0.184461	0.378095	0.086399	0.129484	0.17533	0.105208	0.096864	0.33083	0.158423
Novel_011415	XLOC_12139.	transcript	0.003447	0	0	0.024077	0	0.130162	0	0.019234	0.007149
Novel_011416	XLOC_12139.	transcript	0.153566	0	0.209846	0.232931	0.201497	0	0.612194	0.494242	0.116299
Novel_011417	XLOC_12139.	transcript	0.162771	0	0.02175	0.00096	0.006164	0	0.001524	0.00672	0.036528
Novel_011418	XLOC_12139.	transcript	0.020842	0	0.339799	0.133251	0	0	0.007434	0.000565	0.123819
Novel_011419	XLOC_12139.	transcript	0	0	0	0	0	0	0	0.118249	0
Novel_011420	XLOC_12139.	transcript	0.371676	0.028377	0.023736	0.017401	0.043932	0	0.197929	0.211137	0.099296
Novel_011421	XLOC_12141.	transcript	0.019721	0.04637	0	0.062469	0.038149	0.078244	0.247899	0.085707	0
Novel_011422	XLOC_12143.	transcript	0.372479	0.016849	0.367757	0.244396	0.416851	0.320416	0.457135	0.318242	0.216858
Novel_011423	XLOC_12150.	transcript	0.753062	1.047581	0.90806	0.592762	0.710824	0.898131	0.806614	0.451966	0.518524
Novel_011424	XLOC_12151.	transcript	0.141917	0.247628	0.012781	0.25905	0.217347	0.178048	0.048405	0.232379	0.015055
Novel_011425	XLOC_12151.	transcript	0	0	0.162729	0	0	0	0.186438	0	0.200565
Novel_011426	XLOC_12151.	transcript	0.243039	0.040892	0	0.047263	0.263481	0	0	0.048066	0.111852

Novel_011427	XLOC_12151.	transcript	0.181057	0.296944	0.369738	0.430339	0.229763	0.394791	0.373852	0.458697	0.074549
Novel_011428	XLOC_12151.	transcript	0.008837	0	0	0.124873	0.13367	0.07504	0.041155	0.043001	0
Novel_011429	XLOC_12151.	transcript	0	0.073435	0	0.002255	0	0	0	0.159589	0.005442
Novel_011430	XLOC_12151.	transcript	0.132774	0.044369	0.111367	0.059088	0.213403	0.178344	0.199129	0.259637	0.042836
Novel_011431	XLOC_12151.	transcript	0.322573	0.158711	0.001401	0.359976	0.084255	0.56936	0.406122	0.08897	0.510474
Novel_011432	XLOC_12151.	transcript	0	0	0.162459	0	0	0	0	0	0
Novel_011433	XLOC_12151.	transcript	0.157529	0	0	0	0	0.047113	0.037388	0.041649	0.076985
Novel_011434	XLOC_12151.	transcript	0.092111	0.043496	0.056695	0.085005	0.03744	0.042992	0.050252	0.455411	0.022942
Novel_011435	XLOC_12151.	transcript	0.028304	1.159394	0.288905	0.257935	0.052042	0.038919	0.022552	0.016968	0.030495
Novel_011436	XLOC_12151.	transcript	0	0	0	0	0.514552	0	0	0	0
Novel_011437	XLOC_12151.	transcript	0	0	0	0	0	0	0	0	0
Novel_011438	XLOC_12151.	transcript	0	0	0	0	0	0	0	0	0
Novel_011439	XLOC_12151.	transcript	0.696174	0.023318	0	0	0	0	0.012641	0.314315	0.006016
Novel_011440	XLOC_12151.	transcript	0.155277	0.410102	0.415019	0.360167	0.580373	0.256788	0.215956	0.34864	0.18923
Novel_011441	XLOC_12151.	transcript	0	0	0	0	0	0.325639	0	0.000256	0.003637
Novel_011442	XLOC_12151.	transcript	0	0	0.206061	0.010852	0.19015	0.098851	0.499831	0.230131	0.028577
Novel_011443	XLOC_12153.	transcript	0.15419	0.467242	0.426334	0.162395	0.281502	0.075639	0.329557	0.219917	0.280663
Novel_011444	XLOC_12153.	transcript	0	0.587278	0.018437	0.104384	0.012385	0.260311	0	0.003994	0.68298
Novel_011445	XLOC_12154.	transcript	0	0.531086	0	0	0	0	0	0	0
Novel_011446	XLOC_12157.	transcript	1.03128	3.373338	1.365998	4.448894	1.794083	3.059535	2.64012	3.435759	1.60724
Novel_011447	XLOC_12157.	transcript	0.200076	0.292096	0.28578	0.285098	0.361979	0.248038	0.32237	0.276724	0.497531
Novel_011448	XLOC_12168.	transcript	0.282416	0	0.165467	0.218815	0.192732	0.197744	0.131386	0.055329	0
Novel_011449	XLOC_12168.	transcript	0.003984	0.002273	0	0	0.001263	0.003439	0	0.000371	0.00059
Novel_011450	XLOC_12168.	transcript	0.101533	0.244034	0	0.043348	0.09331	0.102797	0.000265	0.078903	0.134783
Novel_011451	XLOC_12168.	transcript	0.031204	0.293593	0.09322	0.00097	0.06968	0.017298	0.101535	0.096662	0.021248
Novel_011452	XLOC_12168.	transcript	0	0.203503	0	0	0.045862	0	0.172438	0.134132	0.072166
Novel_011453	XLOC_12168.	transcript	0.02068	0.248526	0	0	0	0	0.091676	0	0.086924
Novel_011454	XLOC_12168.	transcript	0	0.253724	0.00819	0.0072	0.004641	0.073308	0.163044	0.035483	0.069008
Novel_011455	XLOC_12169.	transcript	0.059907	0	0.096956	0	0.059638	0	0	0	0
Novel_011456	XLOC_12170.	transcript	0.225915	0.241516	0.307934	0.034648	0.057741	0.186222	0.031693	0.206496	0.033481
Novel_011457	XLOC_12172.	transcript	0.009296	0.003852	0	0.108389	0	0	0	0.002661	0.005508
Novel_011458	XLOC_12175.	transcript	0.146528	0.23656	0.285347	0.207579	0.144346	0.272665	0.139526	0.351445	0.139979
Novel_011459	XLOC_12175.	transcript	0.363661	0.414034	0.364575	0.773708	0.475706	0.672574	0.798482	0.509386	0.430266
Novel_011460	XLOC_12175.	transcript	1.232706	0.388601	0.449918	0.779833	1.007666	1.097242	0.299537	0.134225	0.368468
Novel_011461	XLOC_12175.	transcript	0.259627	0	0.285563	0	0	0	0.053684	0.229945	0
Novel_011462	XLOC_12175.	transcript	0.807143	0	0.479289	0	0	0	0.987423	0.000601	0
Novel_011463	XLOC_12175.	transcript	0.12538	0.034778	0.139144	0.151873	0.052032	0.114842	0.072749	0	0.056078
Novel_011464	XLOC_12175.	transcript	0	0	0	0	0	0	0.67449	0.62887	0

Novel_011465	XLOC_12175.	transcript	0	0	0	0	0.207123	0	0	0	0
Novel_011466	XLOC_12175.	transcript	0	0	0	0	0	0	0.010017	0.060762	0
Novel_011467	XLOC_12178.	transcript	0.142526	0.256706	0.076746	0.093689	0.079803	0.058781	0.244655	0.17352	0.197117
Novel_011468	XLOC_12178.	transcript	0.14826	0.142251	0.373272	0.336037	0.199189	0.307389	0.231838	0.239212	0.312352
Novel_011469	XLOC_12179.	transcript	0	0	0.049682	0.327368	0.02488	0.030032	0.013441	0.432115	0
Novel_011470	XLOC_12179.	transcript	0.140471	0.244522	0	0.0156	0.282959	0	5.90E-05	0.267717	0
Novel_011471	XLOC_12179.	transcript	0.152102	0	0.823249	0	0.242813	0	0	0.173308	0
Novel_011472	XLOC_12179.	transcript	0.816291	0	0	0	0.32967	0.745191	0.922767	0.678653	0.840158
Novel_011473	XLOC_12179.	transcript	0.167204	0	0	0.505781	0	0.368628	0.348942	0.373196	0
Novel_011474	XLOC_12179.	transcript	0	0	0	0	0	0	0	0.003856	0
Novel_011475	XLOC_12183.	transcript	1.149568	0.497149	1.572606	1.853579	1.209702	2.236386	1.291287	1.422035	0.799773
Novel_011476	XLOC_12190.	transcript	0.403583	0.31818	0.336573	0.288507	0.191525	0.320747	0.305383	0.299657	0.354809
Novel_011477	XLOC_12190.	transcript	0.113766	0	0.119137	0.08546	0.061668	0.040374	0.083087	0.093444	0.087688
Novel_011478	XLOC_12192.	transcript	0	0.93748	0	0.007689	0	0	0	0	0
Novel_011479	XLOC_12192.	transcript	5.665674	0.134566	3.719026	3.341275	4.019498	3.582478	7.71729	5.068797	5.511791
Novel_011480	XLOC_12203.	transcript	0.423959	0	0	0.411186	0.52732	0.552795	0.054195	0.121783	0.090803
Novel_011481	XLOC_12203.	transcript	0.164897	0.295628	0	0.019136	0	0	0.521779	0.314954	0.222543
Novel_011482	XLOC_12203.	transcript	0.087235	0.222853	0.260645	0.156595	0.136686	0.068191	0.172898	0.012369	0.026439
Novel_011483	XLOC_12203.	transcript	0	0	0.037377	0.001309	0	0	0	0	0
Novel_011484	XLOC_12203.	transcript	0	0.157726	0	0	0	0.665108	0.27389	0.159445	0
Novel_011485	XLOC_12203.	transcript	0.262499	0.201269	0.072762	0.224065	0.125747	0.084784	0.078523	0.402201	0.079675
Novel_011486	XLOC_12206.	transcript	0.540446	0.151442	0.413212	0.397092	0.450388	0.376747	0.856584	0.521697	0.402442
Novel_011487	XLOC_12207.	transcript	0	0.01621	0.129648	0	0	0.076457	0	0	0
Novel_011488	XLOC_12207.	transcript	0.184762	0	0.063655	0.14507	0.127779	0	0.14303	0.087685	0.038744
Novel_011489	XLOC_12207.	transcript	0.085581	0	0	0.017402	0	0	0.080566	0.010889	0
Novel_011490	XLOC_12211.	transcript	0.565093	0.295842	0.414098	0.31392	0.476242	0.26928	0.820969	0.466488	0.832167
Novel_011491	XLOC_12211.	transcript	0.633294	0.193598	0.275598	0.239545	0.065014	0.336894	0.635976	0.354409	0.170975
Novel_011492	XLOC_12211.	transcript	0.672809	0.846977	0.886437	0.951477	0.932756	1.177586	0.789715	0.798225	0.638187
Novel_011493	XLOC_12229.	transcript	1.167289	0.020701	0.609178	0.480249	0.661068	0.860627	1.29899	0.60237	0.138957
Novel_011494	XLOC_12229.	transcript	0.813329	0.068436	0.587468	0.427582	0.628577	0.893841	1.165634	0.475464	0.109606
Novel_011495	XLOC_12229.	transcript	0.877118	0.023527	0.546285	0.406603	0.527327	0.840103	1.029265	0.501003	0.096379
Novel_011496	XLOC_12230.	transcript	0.948419	0.015886	0.487117	0.494854	0.704616	0.906307	1.200616	0.57464	0.138018
Novel_011497	XLOC_12230.	transcript	1.234484	0.047833	0.633879	0.574982	0.719364	1.185324	1.514325	0.65089	0.136953
Novel_011498	XLOC_12231.	transcript	1.833972	0.037174	0.831301	0	1.030079	0	0	0.867621	0.244481
Novel_011499	XLOC_12231.	transcript	0	0	0	0	0	0	0.425797	0	0
Novel_011500	XLOC_12231.	transcript	1.617765	0.058724	0.752154	0.667071	0.915604	1.349894	1.511122	0.802857	0.142716
Novel_011501	XLOC_12231.	transcript	0.510545	0	0	0	0	0	0	0	0
Novel_011502	XLOC_12231.	transcript	0.904996	0.002015	0.598934	0.463003	0.688867	1.036772	0.607806	0.526701	0.125486

Novel_011503	XLOC_12234.	transcript	0	0	0.470008	0.277396	0.277721	0.182811	0	0.17415	0.255805
Novel_011504	XLOC_12234.	transcript	0.433937	0.545503	0.378255	0.292977	0.367938	0.655349	0.715808	0.578903	0.305207
Novel_011505	XLOC_12241.	transcript	0.114826	0.216775	0.105201	0.060654	0.246666	0.292727	0.251544	0.167946	0.12865
Novel_011506	XLOC_12246.	transcript	0.40263	0.401389	0.338256	0.252291	0.338959	0.314546	0.517315	0.428788	0.486146
Novel_011507	XLOC_12247.	transcript	0.376525	0.794284	0.287201	0.244051	0.437645	0.179193	0.704449	0.258765	1.269267
Novel_011508	XLOC_12247.	transcript	0.298517	0.951825	0.270906	0	0.215879	0.884075	0.411547	0.725019	0
Novel_011509	XLOC_12247.	transcript	0.879654	1.340288	1.11065	1.123968	0.899653	0.192714	1.568848	0.672049	0.986727
Novel_011510	XLOC_12247.	transcript	0.4621	0	0	0	0	0	0	0.56129	0
Novel_011511	XLOC_12248.	transcript	0	0	0	0	0	0	0.911555	0	0
Novel_011512	XLOC_12248.	transcript	0	0	0	0	0	0.633092	0	0.433161	0
Novel_011513	XLOC_12248.	transcript	1.503577	1.492018	1.779932	2.060494	1.36888	1.334381	0.243661	1.222088	1.528912
Novel_011514	XLOC_12248.	transcript	0	0	0	0	0	0	0.370573	0	0
Novel_011515	XLOC_12248.	transcript	0.344859	0.805765	0.34081	0.618196	0.282154	0.300463	0.151279	0.949909	0.280207
Novel_011516	XLOC_12249.	transcript	0.539346	0.157594	0	0.634205	0.011045	0.300493	0	0.03083	0.009305
Novel_011517	XLOC_12249.	transcript	0	0.248282	0.192413	0.000662	0.575939	0.365949	0.153535	0.1869	0.115752
Novel_011518	XLOC_12249.	transcript	0	0	0	0	0	0	0	0	0.276527
Novel_011519	XLOC_12249.	transcript	0	0	0	0	0	0	0.271257	0	0
Novel_011520	XLOC_12249.	transcript	0	0.067479	0.169196	0.001622	0.056719	0	0.247869	0.502525	0
Novel_011521	XLOC_12249.	transcript	0.011178	0.001795	0.260412	0.042733	0.000507	0.019282	0.144819	0.188553	0.17445
Novel_011522	XLOC_12251.	transcript	0.031935	0	0.033762	0.05353	0.139331	0.03853	0.170053	0.201126	0
Novel_011523	XLOC_12261.	transcript	0.13784	0	0.102805	0.041197	0.121301	0.07353	0.327712	0.196865	0.033615
Novel_011524	XLOC_12261.	transcript	0.641834	0.153422	0.197773	0.106759	0.234684	0.234135	0.334985	0.355742	0.195917
Novel_011525	XLOC_12262.	transcript	0.153509	0.073224	0.273911	0.092559	0.125749	0.026649	0.317167	0.100368	0.052353
Novel_011526	XLOC_12267.	transcript	0.155887	0.258838	0.150645	0.284341	0.408531	0.048537	0.152224	0.110771	0.258285
Novel_011527	XLOC_12267.	transcript	0.058059	0.047371	0	0	0.012023	0.389111	0.174527	0.153958	0.104559
Novel_011528	XLOC_12274.	transcript	0.215918	0.334113	0.270246	0.182845	0.077249	0.167186	0.250857	0.185629	0.201995
Novel_011529	XLOC_12275.	transcript	0	0	0.68799	0.23682	0.306728	0.48532	0.535101	1.04628	0.36933
Novel_011530	XLOC_12275.	transcript	0.580122	0	0.133378	0.117178	0.183884	0.15541	0.117917	0.152526	0.320999
Novel_011531	XLOC_12275.	transcript	0	0	0.097327	0.159134	0.096328	0.362885	0.085013	0.087811	0.139178
Novel_011532	XLOC_12275.	transcript	0	0	0.119925	0.131065	0.17921	0.179978	0.126986	0.151485	0
Novel_011533	XLOC_12275.	transcript	0.078316	0	0	0	0.15732	0.311252	0.113487	0.097304	0
Novel_011534	XLOC_12275.	transcript	0.000682	0.011112	0.383531	0.135073	0.223717	0.344138	0.180954	0.44246	0.279629
Novel_011535	XLOC_12275.	transcript	7.50E-05	0	0.334357	0.142033	0.214473	0.379148	0.207077	0.426411	0
Novel_011536	XLOC_12280.	transcript	0.238489	0	0.150152	0.092333	0.244587	0.122551	0.354263	0.154995	0.060075
Novel_011537	XLOC_12281.	transcript	0	0	0	0.196147	0	0	0	0	0
Novel_011538	XLOC_12281.	transcript	0.28942	0.067469	0.427128	0.119742	0.351685	0.253101	0.423657	0.36761	0.099113
Novel_011539	XLOC_12283.	transcript	0.034723	0.180532	0.153217	0.250938	0.141435	0.266121	0.191245	0.337115	0.019975
Novel_011540	XLOC_12283.	transcript	0	0	0	0	0.12712	0	0.142839	0	0

Novel_011541	XLOC_12283.	transcript	0.237824	0.077043	0.089906	0	0	0	0.168354	0	0.289518
Novel_011542	XLOC_12283.	transcript	0.259155	0.514665	0	0.203776	0.114328	0.41279	0.660206	0.687215	0.279834
Novel_011543	XLOC_12283.	transcript	0	0	0	0	0	0	0	0	0
Novel_011544	XLOC_12283.	transcript	0.783331	0.186105	0.492992	0.491736	0.458905	0.543112	0.575904	0.578894	0.328973
Novel_011545	XLOC_12284.	transcript	0	0	0	0	0.591873	0.073108	0	0	0
Novel_011546	XLOC_12284.	transcript	1.668378	0	0	0.836988	0.560006	1.288956	2.512885	0.710168	2.393277
Novel_011547	XLOC_12284.	transcript	0.330843	0.781682	1.217737	0.870274	0.547647	0.806955	0.313261	1.039414	0.175526
Novel_011548	XLOC_12284.	transcript	1.175729	1.02085	1.343884	0.859273	0.819805	0	0.478053	1.30785	0
Novel_011549	XLOC_12285.	transcript	0.29496	0	0.001893	0.209768	0.400918	0	0	0.372585	0.08792
Novel_011550	XLOC_12285.	transcript	0.380188	0.139856	0.344678	0.073345	0.087621	0.256959	0.646273	0.082436	0.191492
Novel_011551	XLOC_12285.	transcript	0.002024	0	0	0	0	0	0	0.006867	0.00573
Novel_011552	XLOC_12285.	transcript	0.1572	0	0.055895	0	0.041407	0	0	0	0
Novel_011553	XLOC_12288.	transcript	0	0	0	0	0	0	0	0	0
Novel_011554	XLOC_12288.	transcript	0.389004	0.238695	0.310749	0.263882	0.033026	0.061845	0.167639	0.32871	0.302341
Novel_011555	XLOC_12288.	transcript	0.326991	0.943818	0.280702	0.319136	0.663844	0.729079	0.642377	0.567286	0.37657
Novel_011556	XLOC_12294.	transcript	0.347506	0.227799	0.450908	0.390967	0.687062	0.426835	0.389071	0.161607	0.443299
Novel_011557	XLOC_12294.	transcript	0.235614	0.119985	0.20848	0.113779	0.182778	0.155336	0.246712	0.579406	0.115586
Novel_011558	XLOC_12301.	transcript	0	0.495357	0	0.010443	0	0	0.022372	0	0
Novel_011559	XLOC_12301.	transcript	0.091479	0.080044	0.088492	0.031158	0.104675	0.105156	0.29564	0.051321	0.062018
Novel_011560	XLOC_12301.	transcript	0	0.095014	0	0.050081	0.134372	0	0.112831	0	0.242938
Novel_011561	XLOC_12302.	transcript	0.076348	0.016291	0	0	0.137445	0.294294	1.273851	0.179691	0.105976
Novel_011562	XLOC_12302.	transcript	0.267889	0.385494	0.011489	0.170822	0	0	0.076228	0.0278	0
Novel_011563	XLOC_12302.	transcript	0.521552	0.079227	1.111964	1.243825	1.002904	0.758313	0.025192	1.118151	0.564629
Novel_011564	XLOC_12302.	transcript	0.063098	0.056816	0.758119	0	0.180927	0.042695	0.174581	0.103852	0
Novel_011565	XLOC_12302.	transcript	0	0.108858	0	0	0	0	0	0.106268	0.369958
Novel_011566	XLOC_12302.	transcript	0.173318	0.226281	0.058223	0.470004	0.157282	0.775196	0.363427	0.364304	0.437514
Novel_011567	XLOC_12303.	transcript	0.223437	0	0.159519	0	0	0.044484	0.084635	0.129584	0.044705
Novel_011568	XLOC_12303.	transcript	0	0	0	0	0.019705	0	0	0.013564	0
Novel_011569	XLOC_12304.	transcript	0.009727	0.008088	0.102694	0.085537	0.067972	0.105431	0	0.050523	0.131619
Novel_011570	XLOC_12304.	transcript	0	0	0.119642	0	0	0	0	0	0
Novel_011571	XLOC_12311.	transcript	0	0	0	0	0	0	0	0	0
Novel_011572	XLOC_12311.	transcript	0	0	0	0.064869	0	0	0.236143	0	0
Novel_011573	XLOC_12311.	transcript	0.38898	0.060793	0.317869	0.201374	0.27456	0.303261	0	0.173087	0.200521
Novel_011574	XLOC_12313.	transcript	0	0	0	0	0	0	0	0	0
Novel_011575	XLOC_12313.	transcript	0	0	0	0	0.254601	0	0	0.414576	0
Novel_011576	XLOC_12313.	transcript	0	0	0	0	0	0	0	0	0
Novel_011577	XLOC_12313.	transcript	0.21968	0.441109	0.329114	0.503724	0.331676	0.317917	0.363492	0.225779	0.651217
Novel_011578	XLOC_12313.	transcript	0	0	0.145862	0.091827	0.009827	0.14284	0.344364	0.086424	0

Novel_011579	XLOC_12316.	transcript	0.208393	0.001937	0	0.000488	0	0	0	0.004225	0
Novel_011580	XLOC_12316.	transcript	0.050744	0.398456	0.610387	0.859255	0.836279	0.618598	0.478372	0.505317	0.254639
Novel_011581	XLOC_12316.	transcript	0	0	0	0	0	0	0.023368	0	0
Novel_011582	XLOC_12317.	transcript	0	0.034012	0.174696	0.08505	0.000169	0.012439	0.017331	0.090444	0
Novel_011583	XLOC_12317.	transcript	0.133772	0	0	0	0.459715	0.073375	0.170288	0	0
Novel_011584	XLOC_12317.	transcript	0.800601	0.163907	0.880592	0.846927	0.641569	1.074138	1.253824	1.020421	1.1083
Novel_011585	XLOC_12323.	transcript	0.248105	0	0.180331	0.366492	0.179534	0.074836	0.178674	0.168627	0.330299
Novel_011586	XLOC_12330.	transcript	0.272263	0	0.615139	0	0	0	0	0.105286	0.228941
Novel_011587	XLOC_12330.	transcript	0.038693	0.332021	0.072326	0	0.136369	0.044603	0.081746	0.021166	0
Novel_011588	XLOC_12336.	transcript	0.1016	0	0.298431	0.036991	0	0.802164	0.184155	0.13791	0.029792
Novel_011589	XLOC_12336.	transcript	0.800373	0.915487	0.675912	0.417548	0.60462	0.669786	0.695103	0.594108	0.706446
Novel_011590	XLOC_12337.	transcript	0.021046	0.94325	0	0.072689	0.085522	0.017491	0.079539	0.009793	0.034361
Novel_011591	XLOC_12337.	transcript	0	0	0	0	0	0	0	0	0
Novel_011592	XLOC_12337.	transcript	0.306644	0	0.080381	0.173636	0.088765	0.267157	0.149558	0.132902	0.017118
Novel_011593	XLOC_12337.	transcript	0.014676	0	0.01182	0.031213	0.017446	0.439539	0.218828	0.066514	0.122814
Novel_011594	XLOC_12337.	transcript	0	0.035573	0.691956	0	0.051957	0	0	0	0
Novel_011595	XLOC_12337.	transcript	0	0	0	0.008202	0	0.202207	0.084301	0.050693	0
Novel_011596	XLOC_12337.	transcript	0.022191	0.005848	0.018932	0.008753	0.016468	0.038225	0.094411	0.053423	0.003002
Novel_011597	XLOC_12337.	transcript	0	0	0	0	0.053275	0	0	0.186184	0
Novel_011598	XLOC_12337.	transcript	0.017581	0	0	0	0.007583	0	0.011988	0.05347	0
Novel_011599	XLOC_12337.	transcript	0.011121	0.28206	0.046186	0.112637	0.251002	0.065409	0.031576	0.009809	0.220547
Novel_011600	XLOC_12337.	transcript	0	0	0	0.188943	0	0	0.254479	0.241758	0
Novel_011601	XLOC_12337.	transcript	0.051476	0.043808	0.015052	0.032552	0.028276	0.030109	0.099922	0.092484	0.022701
Novel_011602	XLOC_12337.	transcript	1.499014	0.491173	0	0	0.224244	0	0	0	0
Novel_011603	XLOC_12337.	transcript	0.313638	0.060771	0.12569	0.236904	0.287963	0.296945	0.338221	0.301555	0.230103
Novel_011604	XLOC_12338.	transcript	0.042996	0	0.037988	0.025609	0.02837	0.07745	0	0.026545	0.057562
Novel_011605	XLOC_12338.	transcript	0.017117	0.209692	0.000422	0.00566	0.037296	0.001551	0.084958	0	0.024138
Novel_011606	XLOC_12338.	transcript	0	0	0	0	0	0	0.064978	0.013464	0
Novel_011607	XLOC_12338.	transcript	0.024618	0	0	0	0	0	0	0	0.052755
Novel_011608	XLOC_12339.	transcript	0.123026	0.029918	0.194848	0.092923	0.203608	0.164083	0.328255	0.155549	0.170923
Novel_011609	XLOC_12339.	transcript	0	0	0	0	0	0	0	0	0.130355
Novel_011610	XLOC_12340.	transcript	0.33884	0	0.00137	0.019497	0.036626	0.032281	0.453939	0.109753	0
Novel_011611	XLOC_12340.	transcript	0.592537	0.325336	0.727516	0.630729	0.539116	0.741102	0.622727	0.305867	0.641203
Novel_011612	XLOC_12342.	transcript	2.753675	6.599047	3.727066	5.068392	3.57648	4.583296	4.71287	3.183374	2.981278
Novel_011613	XLOC_12342.	transcript	1.215029	1.037704	3.390785	4.080761	4.598361	3.391747	0.515016	3.585093	4.119634
Novel_011614	XLOC_12345.	transcript	0	0.508593	0.160062	0.261647	0.18582	0.42161	0.333143	0.310675	0.353593
Novel_011615	XLOC_12349.	transcript	0.045317	0.166114	0.306199	0.124373	0.352268	0.245862	0.290656	0.162686	0.164809
Novel_011616	XLOC_12350.	transcript	0.419736	0.278948	0.351837	0	0.224977	0	0.416546	0	0

Novel_011617	XLOC_12350.	transcript	0.005811	0	0	0.090922	0	0.240435	0.003258	0.37714	0.280571
Novel_011618	XLOC_12350.	transcript	0	0.002254	0.217524	0.293449	0.245242	0	0	0	0
Novel_011619	XLOC_12351.	transcript	0	0.022276	0	0.017524	0	0.008116	0	0.007244	0.010077
Novel_011620	XLOC_12351.	transcript	0.533168	0.556688	0.509517	0.321594	0.500117	0.456144	0.721338	0.487844	0.293219
Novel_011621	XLOC_12351.	transcript	0.03188	0	0.263761	0	0.345194	0	0.060819	0.221099	0.49224
Novel_011622	XLOC_12351.	transcript	0.296534	0.107142	0.03128	0.015798	0	0.08885	0.121674	0	0.296244
Novel_011623	XLOC_12352.	transcript	0.056943	0.071007	0.224259	0	0.03416	0.246753	0.055403	0.167608	0.132857
Novel_011624	XLOC_12352.	transcript	0.212292	0.33212	0	0	0.547805	0	0.30849	0.15165	0
Novel_011625	XLOC_12352.	transcript	0.12056	0.291332	0.125569	0.630628	0	0.087366	0.298803	0.138856	0.354158
Novel_011626	XLOC_12354.	transcript	0	0.744045	1.106074	0.480835	0	0.854975	0.719526	0.403595	0
Novel_011627	XLOC_12354.	transcript	0.314797	0.1976	0	0	0	0.00414	1.205643	0.416421	0
Novel_011628	XLOC_12354.	transcript	0	0	0	0.302421	1.014219	0	0	0	0
Novel_011629	XLOC_12354.	transcript	0.205341	1.419124	0.539375	0.484062	0.675561	0.614923	0.750646	0.840252	0.044845
Novel_011630	XLOC_12354.	transcript	0.919256	0.368728	0.196223	0.343125	0.272374	0.224225	0.278562	0.352155	0.568003
Novel_011631	XLOC_12354.	transcript	0	0.217922	0	0.198558	0.201609	0.357778	0.133781	0.266243	0
Novel_011632	XLOC_12358.	transcript	0	0	0.12206	0.042988	0	0	0	0	0
Novel_011633	XLOC_12358.	transcript	0	0	0.021814	0.00027	0.033027	0.050602	0.036163	0.007611	0
Novel_011634	XLOC_12359.	transcript	0.942724	0.412081	1.538582	0.832922	1.20119	0.550147	0.669403	1.141117	0.920588
Novel_011635	XLOC_12361.	transcript	1.45677	2.429218	2.755042	3.095664	3.090501	3.870985	2.162014	2.222791	2.50034
Novel_011636	XLOC_12361.	transcript	0.23339	0.197403	0.011769	0.13795	0	0.21234	0	0.012748	0.365521
Novel_011637	XLOC_12361.	transcript	0.045055	0	0	0	0.433356	0	0.514624	0	0
Novel_011638	XLOC_12361.	transcript	0	0.355646	0.402612	0.19195	0.161918	0.099952	0.062445	0.528841	0
Novel_011639	XLOC_12364.	transcript	0.502133	1.102743	0.46889	0.550414	0.53195	0.619887	0.565776	0.51924	0.369591
Novel_011640	XLOC_12368.	transcript	0	1.010329	0.112976	0.040745	0.010187	0	0.021677	0.059031	0.140415
Novel_011641	XLOC_12368.	transcript	0.219578	0.112576	0.449309	0.730992	0.555848	1.001016	0.490134	0.432813	0.277192
Novel_011642	XLOC_12377.	transcript	0.092386	0	0	0.092996	0	0.372024	0.140651	0.127566	0.2802
Novel_011643	XLOC_12377.	transcript	0.015417	0.324284	0	0.140576	0	0	0.681503	0	0.036369
Novel_011644	XLOC_12377.	transcript	0.469806	0.086419	1.000167	0.487849	0.974716	0.636626	0.583424	0.056062	0.224206
Novel_011645	XLOC_12377.	transcript	0.580285	0.108791	0.718388	1.309873	0.653748	0.939697	0.682121	1.865252	1.370202
Novel_011646	XLOC_12380.	transcript	0.020514	0.608134	0.108367	0.044946	0.104558	0.107226	0.198717	0.086967	0.192112
Novel_011647	XLOC_12380.	transcript	0.218167	0.69875	0	0.024128	0	0	0	0	0.016455
Novel_011648	XLOC_12385.	transcript	0.375149	0.150335	0.134345	0.261542	0.032967	0.213338	0.691949	0.277514	0.334117
Novel_011649	XLOC_12386.	transcript	0.062005	0.111312	0	0	0	0	0	0	0.15917
Novel_011650	XLOC_12386.	transcript	0	0	0	0.676026	0	0	0.007267	0.683172	0
Novel_011651	XLOC_12386.	transcript	1.539738	2.96827	0.699327	1.457253	1.213843	0.94595	2.189659	0.511678	1.726947
Novel_011652	XLOC_12386.	transcript	0.823395	0	1.423842	1.1475	1.534772	1.93916	1.095626	2.529258	1.571336
Novel_011653	XLOC_12386.	transcript	0.281066	0	1.298038	0.403744	0.293254	1.085554	0.66381	0.757508	0
Novel_011654	XLOC_12386.	transcript	0	0	0.200393	0.079519	0.272449	0	0.082878	0.090639	0.337707

Novel_011655	XLOC_12390.	transcript	0.635683	0.129524	0.557317	0.640475	0.605513	0.640683	0.872861	0.606063	0.308969
Novel_011656	XLOC_12391.	transcript	0	0.016675	0	0	0	0	0.033852	0.053973	0
Novel_011657	XLOC_12391.	transcript	0	0	0	0	0	0	0	0	0
Novel_011658	XLOC_12391.	transcript	0	0	0	0	0	0	0.005111	0	0
Novel_011659	XLOC_12391.	transcript	0.331704	0.291068	0.552822	0.476377	0.427546	0.396646	0.463714	0.476533	0.998692
Novel_011660	XLOC_12396.	transcript	0.389418	0	0.259842	0	0	0	0.542935	0.207803	0.146306
Novel_011661	XLOC_12396.	transcript	0.607296	0.001937	0.617697	0.761309	0.617571	1.478717	0.300642	0.281132	0.060832
Novel_011662	XLOC_12396.	transcript	0.27951	1.501659	0.372805	0.703987	0.449828	0	0.65324	1.002364	0.854053
Novel_011663	XLOC_12396.	transcript	0.38334	0.498082	0.361497	0.402397	0.4178	0.39377	0.495915	0.558429	0.307985
Novel_011664	XLOC_12397.	transcript	0.319863	0	0.099281	0.046104	0.066218	0.141718	0.308197	0.317776	0.047756
Novel_011665	XLOC_12397.	transcript	0	0.037337	0.114168	0.076818	0.23721	0.027177	0	0.039126	0.037995
Novel_011666	XLOC_12398.	transcript	0.347933	0	0.264408	0	0.3236	0	0.064544	0	0.086405
Novel_011667	XLOC_12398.	transcript	0.432161	0	0.596264	0.957873	0.518161	0.271929	0.308262	0.935116	0.622596
Novel_011668	XLOC_12400.	transcript	0	0	0.022716	0	0	0	0.37527	0	0
Novel_011669	XLOC_12401.	transcript	2.167442	0.983234	3.282637	2.41381	2.583528	2.812288	1.872304	2.657797	3.133231
Novel_011670	XLOC_12402.	transcript	0	0.017053	0	0.424634	0.03314	0	0	0	0
Novel_011671	XLOC_12403.	transcript	0	0.000103	0	0	0.067011	0.000627	0.117166	0.079688	0
Novel_011672	XLOC_12403.	transcript	0.283704	1.790639	0.045282	0.092127	0.479522	0.757472	0.185267	1.090056	0.118434
Novel_011673	XLOC_12403.	transcript	0	0.171497	0.28993	0.415952	0	0.248075	0	0	0.176371
Novel_011674	XLOC_12403.	transcript	0.0245	0.20929	0.475147	0.197692	0	0	0	0	0.035234
Novel_011675	XLOC_12403.	transcript	0	0.398927	0.005268	0	0	0	0	0.002717	0
Novel_011676	XLOC_12403.	transcript	0	0	0	0	0	0	0	0	0
Novel_011677	XLOC_12404.	transcript	0.073157	0	0.103448	0.139069	0.035971	0.031377	0.130163	0.053316	0.164182
Novel_011678	XLOC_12404.	transcript	0.195937	1.652761	0.213956	0.039578	0.112591	0.075873	0.346703	0.089704	0.707526
Novel_011679	XLOC_12404.	transcript	0.302184	0.370701	0.453402	0.536403	0.651814	0.505616	0.641259	0.589594	0.404117
Novel_011680	XLOC_12404.	transcript	0	0.022419	0	0.000268	0	0.000937	0	0	0
Novel_011681	XLOC_12404.	transcript	0.323332	0.389205	0.309792	0.339608	0.271452	0.37105	0.459337	0.318209	0.354646
Novel_011682	XLOC_12405.	transcript	0.2371	0	0.020562	0.054231	0.003436	0.011439	0.038535	0	0.013403
Novel_011683	XLOC_12405.	transcript	0	0.309432	0.002286	0	0.000573	0.008093	0	0.015573	0
Novel_011684	XLOC_12405.	transcript	0.128519	0.220759	0.35968	0.276588	0.338573	0.388539	0.538328	0.381642	0.349368
Novel_011685	XLOC_12405.	transcript	0.019513	0.001582	0	0	0	0	0.014969	0	0.04286
Novel_011686	XLOC_12408.	transcript	0.050467	0.2458	0.133297	0.042297	0.040421	0.043302	0.2459	0.140042	0.139153
Novel_011687	XLOC_12408.	transcript	0	0	0.090874	0.062259	0.153778	0	0	0.135339	0.050934
Novel_011688	XLOC_12408.	transcript	0.077585	0.022739	0.011308	0.201945	0.024486	0	0.002491	0.002411	0.030158
Novel_011689	XLOC_12408.	transcript	0.518939	1.750675	0.991811	1.001598	1.116706	1.041974	1.325803	1.358336	0.971753
Novel_011690	XLOC_12408.	transcript	0.12564	1.175579	0	0.273407	0	0.359099	0	0.244572	0.326941
Novel_011691	XLOC_12409.	transcript	0	1.166172	1.912071	0	0	0	0	0	0
Novel_011692	XLOC_12409.	transcript	0	2.530491	0.579324	39.8966	0.876182	0	1.106907	0	1.967311

Novel_011693	XLOC_12409.	transcript	18.00964	0	0.211281	5.799557	0.06924	55.86854	0	26.98534	0
Novel_011694	XLOC_12409.	transcript	0	0.000605	0	0	0.953553	0	0	0	0
Novel_011695	XLOC_12412.	transcript	0.165804	0.302996	0.331753	0.104953	0.247438	0.191804	0.396936	0.234803	0.341414
Novel_011696	XLOC_12412.	transcript	0.435601	0.855539	0.481664	0.357317	0.352986	0.40165	0.29431	0.197112	0.438743
Novel_011697	XLOC_12413.	transcript	5.882561	7.335383	8.949492	10.17206	9.103248	9.415362	8.249926	8.086098	9.48275
Novel_011698	XLOC_12413.	transcript	0.230642	0.181021	0.351123	0.106737	0.190621	0.342076	0.352982	0.260042	0.395905
Novel_011699	XLOC_12413.	transcript	0	0.145307	0	0.153962	0.146743	0.031181	0.000197	0	0.400983
Novel_011700	XLOC_12414.	transcript	0.593	0.330552	0.254418	0.433587	0.142028	0.385642	0.351238	0.719391	0
Novel_011701	XLOC_12414.	transcript	0.123391	0.145928	0.354752	0.171975	0.349103	0.197827	0.206751	0	0.086248
Novel_011702	XLOC_12414.	transcript	0.70013	0.994492	0.967612	1.304014	1.147807	1.103644	1.617519	1.381337	2.138985
Novel_011703	XLOC_12415.	transcript	0	0	0.00111	0	0	0	0	0	0
Novel_011704	XLOC_12415.	transcript	0.130201	0	0.049538	0.110677	0.070521	0.186298	0.034589	0.175497	0.039213
Novel_011705	XLOC_12415.	transcript	0.129828	0.049208	0.125121	0.045932	0.022974	0	0.192049	0.084178	0
Novel_011706	XLOC_12415.	transcript	0.196612	0.119358	0.157672	0.088546	0	0.111237	0.118911	0.114322	0.107282
Novel_011707	XLOC_12417.	transcript	0.379083	0.085902	0.06442	0.065274	0.027565	0.148935	0.028245	0.072526	0.296741
Novel_011708	XLOC_12417.	transcript	0.056729	0.652693	0	0.18796	0	0	0	0	0.024083
Novel_011709	XLOC_12417.	transcript	0	0	0.229278	0	0.33092	0.19377	0.382412	0.410776	0
Novel_011710	XLOC_12418.	transcript	0	0.054759	0.083291	0.228043	0.133509	0	0.056803	0	0.051335
Novel_011711	XLOC_12418.	transcript	0.029801	0	0	0	0.029037	0	0.0085	0	0
Novel_011712	XLOC_12418.	transcript	0.595883	0.435731	0.041028	0.44235	0.521948	0.848779	0.935959	0.610066	0.687951
Novel_011713	XLOC_12418.	transcript	0	0	0.610814	0	0	0	0	0	0
Novel_011714	XLOC_12419.	transcript	0	0	0	0	0	0	1.53354	0	0
Novel_011715	XLOC_12419.	transcript	0	0	0	0	0	0	0	3.807794	0
Novel_011716	XLOC_12419.	transcript	0	0	0	0	0	0	0	4.09647	0
Novel_011717	XLOC_12419.	transcript	0.766768	0	0.484296	0.291332	0.298239	0.706189	0.557499	0.188739	0.501696
Novel_011718	XLOC_12419.	transcript	0	0.438184	0	0	4.126029	0	1.343928	0	0.003607
Novel_011719	XLOC_12420.	transcript	0.046115	0.067282	0.026814	0.033372	0.147015	0.113086	0	0.063352	0.079305
Novel_011720	XLOC_12420.	transcript	0.139115	0.028527	0.221009	0.127139	0.128534	0.026247	0.367807	0.387231	0.215123
Novel_011721	XLOC_12420.	transcript	0.290002	0.01412	0.420916	0.547184	0.371492	0.378967	0.698614	0.194741	0.192191
Novel_011722	XLOC_12420.	transcript	0	0	0	0	0	0	0	0	0
Novel_011723	XLOC_12420.	transcript	0	0	0	0	0	0	0.077727	0.039669	0
Novel_011724	XLOC_12420.	transcript	0.288449	0.5253	0.400782	0.500934	0.463968	0.507405	0.75142	0.410176	0.351118
Novel_011725	XLOC_12421.	transcript	0.138239	0.203345	0.29206	0.460588	0.388051	0.419939	0.421508	0.398464	0.391878
Novel_011726	XLOC_12421.	transcript	1.156294	0	0	0	0	0	0.353984	0	0
Novel_011727	XLOC_12421.	transcript	0.49254	0	1.453011	1.139001	1.475037	0	1.086715	1.144395	0
Novel_011728	XLOC_12421.	transcript	0.000665	0.556198	0.495941	0.310401	0.28027	4.487871	0.147787	0.291583	2.821707
Novel_011729	XLOC_12422.	transcript	0.067248	0.22554	0.07709	0.32208	0.612291	0.513143	0	0	0.289214
Novel_011730	XLOC_12422.	transcript	0.272801	0.259315	0.524629	0.446252	0	0.23946	0.645854	0.681146	0.319834

Novel_011731	XLOC_12422.	transcript	0.002405	0	0.023838	0.298633	0	0.006595	0.00493	0.009517	0.006345
Novel_011732	XLOC_12425.	transcript	0.213894	1.358213	0.197913	0.093522	0.194177	0.153614	0.275566	0.137075	0.148885
Novel_011733	XLOC_12425.	transcript	1.847896	4.067979	1.383091	3.150812	2.163493	2.257274	2.209578	1.776326	3.136623
Novel_011734	XLOC_12426.	transcript	0.945216	0.995511	1.037336	1.20699	1.39279	1.019138	1.7197	1.470079	0.347372
Novel_011735	XLOC_12426.	transcript	0.455224	0.527964	0.399606	0.500368	0.315666	0.607949	0.168976	0.205298	0.44733
Novel_011736	XLOC_12427.	transcript	0.082935	0.026733	0.137182	0.027715	0.151498	0.235545	0.233599	0.121293	0.255995
Novel_011737	XLOC_12427.	transcript	0.088427	0	0	0.02951	0	0	0	0	0
Novel_011738	XLOC_12430.	transcript	0.430447	0.65037	0.646982	0.693944	0.831924	0.618713	0.952497	0.838375	0.46443
Novel_011739	XLOC_12430.	transcript	0.028336	0.368465	0.171743	0.189435	0.123378	0.110956	0.095419	0.141356	0.091622
Novel_011740	XLOC_12430.	transcript	0.312825	0.642333	0.536772	0.495787	0.406431	0.474416	0.820641	0.551972	0.340123
Novel_011741	XLOC_12431.	transcript	0.078102	0.607353	0.041232	0.032855	0.057211	0.095568	0	0.01103	0.155388
Novel_011742	XLOC_12433.	transcript	0.282563	0.583875	0.264688	0.369067	0.235759	0.341535	0.510469	0.514302	0.271686
Novel_011743	XLOC_12433.	transcript	0.384543	0.000642	0	0.47318	0	0	0.416967	0	0
Novel_011744	XLOC_12433.	transcript	0	0.356676	0.627201	0.019261	0.517849	0.578458	0	0.645945	0.388199
Novel_011745	XLOC_12433.	transcript	0.100259	0.28734	0	0	0.098802	0	0.087568	0	0.056322
Novel_011746	XLOC_12434.	transcript	0.233035	1.712406	0.199752	0.171938	0.289487	0.257228	0.392494	0.179209	0.290981
Novel_011747	XLOC_12434.	transcript	0.669926	0.388135	0.438232	0.558914	0.472649	0.463561	0.395081	0.606653	0.286597
Novel_011748	XLOC_12434.	transcript	0.45937	0.508635	0.388786	0.479179	0.358218	0.491079	0.536564	0.571616	0.273976
Novel_011749	XLOC_12444.	transcript	0.257148	0.100716	0.291086	0.222184	0.083915	0.324652	0.276429	0.259311	0.170204
Novel_011750	XLOC_12444.	transcript	0.285483	0.200306	0.49663	0.287649	0.194622	0.363045	0.377626	0.243698	0.255532
Novel_011751	XLOC_12449.	transcript	1.382971	0.396117	1.134115	0.173224	0.773077	0.606049	0.477929	0.637327	0
Novel_011752	XLOC_12449.	transcript	0	0.187808	0.217985	0.245765	0.31068	0.279857	0.177021	0.218947	0
Novel_011753	XLOC_12449.	transcript	0.272694	0.173789	0.106848	0.24031	0.135667	0.171461	0.115986	0	0.36738
Novel_011754	XLOC_12449.	transcript	0	0.181099	0	0.004421	0.003557	0.313916	0.184115	0.287961	0
Novel_011755	XLOC_12449.	transcript	0.038061	0.257743	0.377047	0.328293	0.345843	0.505422	0.450713	0.002368	0.104261
Novel_011756	XLOC_12449.	transcript	0.577004	0.847658	0.255252	0.80834	0.422241	0.354077	0.666644	0.943766	0.73668
Novel_011757	XLOC_12449.	transcript	0.336731	0.021474	0	0.06429	0	0.070352	0.087431	0.236391	0
Novel_011758	XLOC_12449.	transcript	0.413184	0.001215	0.076683	0.31284	0.105158	0.203201	0.26165	0.200084	0.420761
Novel_011759	XLOC_12449.	transcript	0.487613	0.748534	0.503185	0.281661	0.470697	0.474746	0.260451	0.233297	0.306308
Novel_011760	XLOC_12449.	transcript	0.299213	0.011763	0.184107	0.226461	0.453487	0.291534	0.002218	0.006301	0.210033
Novel_011761	XLOC_12449.	transcript	0	0.674613	0	0	0	0	0.428811	0.452851	0
Novel_011762	XLOC_12450.	transcript	0	0	0.447408	0	0	0	0	0	0.077023
Novel_011763	XLOC_12450.	transcript	0.163534	1.438603	0.305409	0.455282	0.557105	0.54455	0.419813	0.549265	0.180923
Novel_011764	XLOC_12455.	transcript	2.702133	4.268958	2.675354	3.452741	3.510267	1.458011	3.293189	3.629791	3.678874
Novel_011765	XLOC_12455.	transcript	0	0	0	0	0	1.405545	0	0	0
Novel_011766	XLOC_12455.	transcript	0	0.05851	0.02737	0.020755	0.115875	0.134256	0.028662	0.033456	0.072394
Novel_011767	XLOC_12455.	transcript	0	0.454126	0	0	0	0	0	0	0
Novel_011768	XLOC_12455.	transcript	0.403678	1.789145	0.319448	0.206013	0.29515	0.281022	0.562923	0.478377	0.384428

Novel_011769	XLOC_1245‹	transcript	0	0	0	0	0	0	0	0	0
Novel_011770	XLOC_1245‹	transcript	0	0	0	0	0	0	0.029941	0	0
Novel_011771	XLOC_1245‹	transcript	0	0	0	0	0	0	0	0	0.06309
Novel_011772	XLOC_1245‹	transcript	0	0	0	0	0	0	0.210549	0	0.008089
Novel_011773	XLOC_1245‹	transcript	0	0	0	0	0	0	0	0	0
Novel_011774	XLOC_1245‹	transcript	0.927545	0.342274	0.486213	0.437204	0.382033	0.605111	0.714714	0.448844	1.169137
Novel_011775	XLOC_1245‹	transcript	0.227835	0	0.722814	0.318581	0.496254	0.595171	0.126243	0.365743	0.538841
Novel_011776	XLOC_1245‹	transcript	0.012023	0.783059	0.036924	0.0343	0.75008	0.009241	0.043079	0.050247	0
Novel_011777	XLOC_1245‹	transcript	0.969353	0.248026	1.107894	1.486699	0.405997	1.349698	1.240654	1.316388	1.341992
Novel_011778	XLOC_1246‹	transcript	0.30491	5.47286	1.963373	2.157302	2.120353	2.731743	0.816156	3.608239	1.185132
Novel_011779	XLOC_1246‹	transcript	0.873146	0.515688	0.976327	0.98524	1.720059	0.837213	1.298554	1.216079	1.0449
Novel_011780	XLOC_1246‹	transcript	0.160536	0.103197	0	0.06899	0.051866	0.12038	0.206623	0.09953	0
Novel_011781	XLOC_1246‹	transcript	0.488305	0.429217	0.50083	0.499794	0.771081	0.575736	0.535593	0.639335	0.163275
Novel_011782	XLOC_1246‹	transcript	0	0	0	0	0	0	0.608021	0	0.31176
Novel_011783	XLOC_1246‹	transcript	0.104353	0.372816	0.381012	0.234586	0.282146	0.138076	0.068158	0.361587	0.282651
Novel_011784	XLOC_1246‹	transcript	0.064626	0.041594	0.114668	0.267898	0.21488	0.084764	0.224529	0.184087	0.065393
Novel_011785	XLOC_1247‹	transcript	0.571203	0.896568	0	0.350896	0.033167	0.192178	0	0.303202	0
Novel_011786	XLOC_1247‹	transcript	0.000146	0	0.224126	0.107766	0.526999	0.185596	0.70067	0	0.066552
Novel_011787	XLOC_1247‹	transcript	0.11196	0.433787	0.314876	0.08341	0	0.159145	0.299619	0.436989	0.254474
Novel_011788	XLOC_1247‹	transcript	0	0	0	0	0	0	0.361457	0	0
Novel_011789	XLOC_1248‹	transcript	0	0	0.018563	0.006076	0	0	0	0	0
Novel_011790	XLOC_1248‹	transcript	0	0.245887	0	0	0	0	0	0	0
Novel_011791	XLOC_1248‹	transcript	0.176036	0.057774	0.151835	0.230416	0.255116	0.281227	0.280875	0.260211	0.309765
Novel_011792	XLOC_1248‹	transcript	0	0.007834	0	0.016271	0	0	0	0	0
Novel_011793	XLOC_1248‹	transcript	0	0	0	0	0	0	0	0	0
Novel_011794	XLOC_1248‹	transcript	0	0	0	0	0	0	0	0	0
Novel_011795	XLOC_1248‹	transcript	0	0	0	0.007314	0	0	0.005246	0	0
Novel_011796	XLOC_1248‹	transcript	0	0	0.011738	0	0.01538	0	0	0.032876	0.046053
Novel_011797	XLOC_1248‹	transcript	0.010584	0	0	0	0	0.021638	0	0	0
Novel_011798	XLOC_1248‹	transcript	0	0	0	0	0	0	0	0	0
Novel_011799	XLOC_1248‹	transcript	0	0	0	0	0	0	0	0	0
Novel_011800	XLOC_1248‹	transcript	0.008775	0	0	0.014512	0	0.021103	0	0.050238	0.015598
Novel_011801	XLOC_1248‹	transcript	0.09808	0	0	0	0	0	0	0	0
Novel_011802	XLOC_1248‹	transcript	0	0.34924	0	0.01463	0.0232	0	0.139145	0	0
Novel_011803	XLOC_1248‹	transcript	0.021623	0	0.026119	0.014984	0.01051	0.033016	0	0.003533	0
Novel_011804	XLOC_1248‹	transcript	0.10946	0.009892	0.020458	0.097774	0.156848	0.019898	0.022039	0.084668	0
Novel_011805	XLOC_1248‹	transcript	0.048555	0.312309	0.20252	0.077797	0.116541	0.20779	0.270784	0.037166	0.165348
Novel_011806	XLOC_1249‹	transcript	0	0	0.313064	0	0	0	0	0	0

Novel_011807	XLOC_12491.	transcript	0.346662	0.590531	0.396029	0.551301	0.387688	0.606696	1.237756	1.103425	0.505793
Novel_011808	XLOC_12497.	transcript	0.027796	1.500818	0.27333	0.03453	0.444707	0.066718	0.437731	0.174404	0
Novel_011809	XLOC_12497.	transcript	0.210198	0.012818	0	0.3536	0	0.217546	0	0.158673	0.358835
Novel_011810	XLOC_12498.	transcript	0.032089	0.319138	0.225825	0.198642	0.136479	0.120536	0.239829	0.133333	0.175948
Novel_011811	XLOC_12508.	transcript	0.245598	0.064954	0.1009	0.435632	0.149861	0.081238	0.517667	0.080363	0.037771
Novel_011812	XLOC_12508.	transcript	0.115992	0	0	0.089894	0.210963	0.229978	0	0.139364	0.041721
Novel_011813	XLOC_12510.	transcript	0	0.109389	0.159153	0.26696	0.106544	0.064432	0.140145	0.219717	0.49236
Novel_011814	XLOC_12510.	transcript	0	0	0	0	0	0	0	0	0
Novel_011815	XLOC_12510.	transcript	0.137166	0	0.018031	0	0	0.041328	0.059231	0.071005	0.002331
Novel_011816	XLOC_12510.	transcript	0	0.081608	0.149026	0.179726	0.173419	0.195517	0.259353	0.092533	0.273119
Novel_011817	XLOC_12510.	transcript	0	0	0	0	0	0	0	0.042231	0
Novel_011818	XLOC_12536.	transcript	0.159405	0	0.313011	0.267793	0	0	0.203742	0.09171	0.116623
Novel_011819	XLOC_12536.	transcript	0.100543	0.011291	0.041173	0.304167	0.120433	0.016436	0.223692	0.243414	0
Novel_011820	XLOC_12536.	transcript	0.102053	0	0	0.370719	0	0	0.073126	0	0
Novel_011821	XLOC_12536.	transcript	0	0	0.378055	0	0	0	0	0	0
Novel_011822	XLOC_12536.	transcript	0	0	0	0	0	0	0	0	0
Novel_011823	XLOC_12536.	transcript	0.178235	0.145122	0	0.691944	0.511388	0.222778	0.55246	0.46712	0.239477
Novel_011824	XLOC_12538.	transcript	0.625974	0.322524	0.45122	0.425199	0.475242	0.642771	1.01843	0.518753	0.332952
Novel_011825	XLOC_12567.	transcript	0.398376	0.01226	0.277239	0	0.319163	0.037728	0.434423	0.009778	0
Novel_011826	XLOC_12567.	transcript	0	0.429328	0	0.402902	0	0.372605	0	0.488587	0.24057
Novel_011827	XLOC_12567.	transcript	0	0	0	0.01831	0	0.019265	0.023788	0.144045	0.097228
Novel_011828	XLOC_12568.	transcript	0.301161	0.200529	0.274964	0.26405	0.26778	0.357498	0.373247	0.286282	0.358484
Novel_011829	XLOC_12568.	transcript	0	0	0	0	0	0	0	0	0
Novel_011830	XLOC_12572.	transcript	0.212181	0.049659	0.212589	0.257092	0.172914	0.124325	0.257861	0.210555	0.264472
Novel_011831	XLOC_12578.	transcript	0.505105	0.104534	0.149338	1.626679	0.195195	0.112512	0.478329	0.382358	0.648894
Novel_011832	XLOC_12578.	transcript	2.575029	0.931824	2.507897	1.750562	2.802128	2.371035	3.244132	3.325208	5.029026
Novel_011833	XLOC_12579.	transcript	0.05648	0.037817	0.047372	0.024245	0.073648	0.045568	0.389157	0.349318	0.022131
Novel_011834	XLOC_12579.	transcript	0	0	0	2.30E-05	0.76221	0	0.785925	0.57082	0
Novel_011835	XLOC_12579.	transcript	0.754963	0.457555	0.690299	0.510859	0.004421	0.699613	0.010225	0.094226	0.609919
Novel_011836	XLOC_12579.	transcript	0	0	0	0.448097	0.011664	0	0	0	0.003251
Novel_011837	XLOC_12579.	transcript	0.012074	0	0.273528	0.282485	0.253663	0	0	0.490889	0
Novel_011838	XLOC_12581.	transcript	0.517143	0	0	0.017685	0	0	0.041099	0.268707	0
Novel_011839	XLOC_12581.	transcript	0	0	0	0.391131	0.029139	0.01828	0.017016	0.282779	0.026759
Novel_011840	XLOC_12581.	transcript	0	0.276849	0	0.135663	0.007859	0	0	0.120971	0
Novel_011841	XLOC_12581.	transcript	0	0	0	0.263706	0	0	0	0.149238	0
Novel_011842	XLOC_12581.	transcript	0	0.083346	0.209262	0.303855	0	0	0	0.093349	0.030056
Novel_011843	XLOC_12581.	transcript	0.222387	0.142721	0	0.187443	0.243345	0.09116	0.528209	0.240325	0.095937
Novel_011844	XLOC_12581.	transcript	0.015715	0.028294	0.068853	0.155685	0.007968	0.067939	0.008663	0.028661	0

Novel_011845	XLOC_12581.	transcript	0.068242	0	0	0	0	0.314367	0.223637	0	0.06642
Novel_011846	XLOC_12581.	transcript	0.134505	0.387645	0.839631	0	0.301915	0.20817	0.191417	0	0.434687
Novel_011847	XLOC_12581.	transcript	0.158362	0.422029	0.207991	0	0.229163	0.13608	0.232937	0	0.435479
Novel_011848	XLOC_12581.	transcript	0.21503	0.406509	0.209227	0.323724	0.251473	0.392827	0.358208	0.498641	0.764436
Novel_011849	XLOC_12581.	transcript	0.122016	1.007644	0.085105	0.148727	0.200679	0.535564	0.507613	0.787208	0.211731
Novel_011850	XLOC_12587.	transcript	0.389853	0.176791	0.187589	0.285412	0.278127	0.243406	0.317531	0.337951	0.259034
Novel_011851	XLOC_12597.	transcript	0.524223	0.789997	0.433124	0.595807	0.529598	0.670141	0.757293	0.37442	0.278137
Novel_011852	XLOC_12597.	transcript	0.039795	0.036662	0.440922	0.025014	0	0.432191	0.016246	0.005696	0.02459
Novel_011853	XLOC_12600.	transcript	0.99238	0.440083	0.007824	2.065956	0.040861	1.570636	1.725175	1.932719	0.68372
Novel_011854	XLOC_12600.	transcript	0.091228	0.150246	0.079776	0.047381	0.131909	0.066591	0.157734	0.14088	0.061976
Novel_011855	XLOC_12602.	transcript	0.121209	0	0.140459	0.202946	0.08933	0.053587	0.112113	0.139759	0.019153
Novel_011856	XLOC_12603.	transcript	9.660085	7.056847	15.7156	12.8002	13.7589	20.37944	10.00795	9.76203	15.13796
Novel_011857	XLOC_12605.	transcript	0.560729	0.170206	0.558574	0.759157	0.471022	0.608072	0.450544	0.722278	0.621494
Novel_011858	XLOC_12605.	transcript	0	0	0	0	0	0	0.238678	0	0
Novel_011859	XLOC_12605.	transcript	0	0	0	0	0.088477	0	0	0	0
Novel_011860	XLOC_12606.	transcript	0.221996	0.01942	0.135535	0.13813	0.143339	0.056448	0.20764	0.109381	0.153014
Novel_011861	XLOC_12608.	transcript	0.526201	1.232085	1.685286	1.547321	1.624441	1.933119	1.600345	1.725182	1.436496
Novel_011862	XLOC_12608.	transcript	1.447112	0.518092	0	0	0	0	0	0	0
Novel_011863	XLOC_12613.	transcript	0.445497	0.852852	0.494157	0.779132	0.692022	0.665193	0.689222	0.601688	0.235162
Novel_011864	XLOC_12613.	transcript	3.320456	7.137775	3.8107	4.38908	4.996786	4.258408	6.09846	7.276913	5.864698
Novel_011865	XLOC_12614.	transcript	0.198587	0.271527	0.089462	0.334056	0.146227	1.394239	0.36977	0.262095	0.887252
Novel_011866	XLOC_12614.	transcript	0	0	0	0.847565	0	0	0	0	0
Novel_011867	XLOC_12614.	transcript	0.051844	1.332536	0.060744	0	0.608493	0.024804	0.184434	0.460229	0.030494
Novel_011868	XLOC_12614.	transcript	0	1.403226	0	0.024389	0	0.006912	0.005444	0	0
Novel_011869	XLOC_12620.	transcript	0.171219	0.255968	0.226355	0.223294	0.092514	0	0.232723	0.177938	0.367767
Novel_011870	XLOC_12620.	transcript	0.033687	0.373906	0	0	0.097309	0.090483	0.031989	0.026271	0
Novel_011871	XLOC_12621.	transcript	0	0	0.001475	0.002444	0	0.010557	0	0	0.335452
Novel_011872	XLOC_12621.	transcript	0.012932	0	0.001962	0	0.002244	0.005088	0.005188	0	0.358379
Novel_011873	XLOC_12621.	transcript	0.942487	0.945491	0.830237	0.868581	0.744135	0.945455	0	0.519933	0.066099
Novel_011874	XLOC_12621.	transcript	0.018467	0	0.006038	0.056245	0	0.009589	0.266876	0.281115	0.295387
Novel_011875	XLOC_12621.	transcript	0.000442	0	0	0.000258	0.003028	0.000293	1.134487	0.31116	0
Novel_011876	XLOC_12621.	transcript	0	0.253201	0	0	0	0	0	0	0
Novel_011877	XLOC_12622.	transcript	0.029753	0.258649	0.171849	0.104336	0.13463	0.144098	0.14154	0.170859	0.182973
Novel_011878	XLOC_12623.	transcript	0.036162	0.030071	0	0	0	0	0	0	0
Novel_011879	XLOC_12625.	transcript	0.356388	0.298469	0.40197	0.470749	0.486947	0.406871	0.469697	0.553205	0.484494
Novel_011880	XLOC_12625.	transcript	0	0.25623	0	0.290016	0	0	0	0	0
Novel_011881	XLOC_12627.	transcript	0.702698	0.285696	0.49222	0.687916	0.768873	0.842285	0.584567	0.878683	0.630153
Novel_011882	XLOC_12627.	transcript	0.178376	0.135359	0	0	0	0	0	0.249507	0.228655

Novel_011883	XLOC_12629.	transcript	0	0	0	0	0	0	0	0.242156	0
Novel_011884	XLOC_12629.	transcript	0.157403	0.125169	0.266258	0.82004	0.642779	0.499432	0.801549	0.196934	0.113066
Novel_011885	XLOC_12629.	transcript	0.142717	0.059684	0.176983	0.078683	0.17304	0.061056	0.031463	0.072468	0.154521
Novel_011886	XLOC_12629.	transcript	0.066623	0	0.104705	0.156867	0	0.186411	0.197459	0.135843	0
Novel_011887	XLOC_12629.	transcript	0.102867	0	0.014679	0.200445	0.063205	0.260832	0.220468	0.157128	0.07516
Novel_011888	XLOC_12637.	transcript	0.22054	0.233533	0.277405	0.024136	0.171923	0.138989	0.294774	0.193875	0.168006
Novel_011889	XLOC_12639.	transcript	0.067703	0.045366	0.053183	0.084558	0.055751	0.054938	0.062558	0.050055	0.039334
Novel_011890	XLOC_12646.	transcript	0.167659	0	0.255411	0.181199	0.322742	0.894732	0.339326	0.439607	0.116926
Novel_011891	XLOC_12650.	transcript	0.724822	0.644769	0.445516	0.571964	0.290356	0.513367	0.390753	0.711166	0.355362
Novel_011892	XLOC_12650.	transcript	0.009484	0	0.056795	0	0.241246	0.028491	0.35637	0	0.007279
Novel_011893	XLOC_12650.	transcript	0	0	0	0	0	0	0	0	0
Novel_011894	XLOC_12650.	transcript	0.075482	0.046343	0.046506	0.205663	0.040366	0.015205	0.234354	0.15177	0.127417
Novel_011895	XLOC_12654.	transcript	0	0.780991	0.828415	0.649524	0.469458	0.040353	0.902385	0.065614	0.010151
Novel_011896	XLOC_12654.	transcript	1.065924	0.608139	0.236619	0.344838	0.602705	0.941403	0.398598	1.147281	0.608322
Novel_011897	XLOC_12670.	transcript	0.824662	2.074035	0.555828	0.481369	0.892501	0.823421	1.433908	0.767682	5.361227
Novel_011898	XLOC_12670.	transcript	0	0	0.081968	0	0	0	0.022472	0	0.05213
Novel_011899	XLOC_12674.	transcript	0.843006	0.550833	1.012017	1.294671	1.078916	1.166937	1.184618	1.181522	0.918626
Novel_011900	XLOC_12680.	transcript	0	0.052279	0.06635	0.026613	0	0	0.140057	0	0.037883
Novel_011901	XLOC_12691.	transcript	0.132007	0.033135	0.159482	0.213081	0.099792	0.421175	0.138016	0.346326	0.070982
Novel_011902	XLOC_12697.	transcript	0.185568	0	0.540608	0.466993	0.575907	0.623518	0.229725	0.337073	0.25521
Novel_011903	XLOC_12698.	transcript	0.811571	0	0.51651	0.978743	0.534828	0.561472	0.861857	0.633471	0.391444
Novel_011904	XLOC_12699.	transcript	0.116669	0	0	0.079271	0.298271	0	0	0.279555	0
Novel_011905	XLOC_12702.	transcript	0.459524	0.244988	0.634302	0.588908	0.515864	0.586116	0.561159	0.590679	0.527245
Novel_011906	XLOC_12707.	transcript	0	0	0.604364	0	0	0.312567	0	0	0
Novel_011907	XLOC_12707.	transcript	0.135033	0	0.131852	0	0	0	0.872328	0	0
Novel_011908	XLOC_12713.	transcript	0	0	0	0	0.00117	0	0	0	0.001791
Novel_011909	XLOC_12713.	transcript	0.483805	0.170504	1.000659	0.631145	0.885895	0.669137	0.634372	1.270347	1.255844
Novel_011910	XLOC_12713.	transcript	1.054285	0.007106	0.237795	0.582579	0.389195	0.340613	0.478765	0.779822	0.444005
Novel_011911	XLOC_12713.	transcript	0	0	0	0	0	0	0.640039	0	0
Novel_011912	XLOC_12715.	transcript	0.374517	0.194217	0.26106	0.220188	0.276824	0.165895	0.336465	0.358338	0.174625
Novel_011913	XLOC_12715.	transcript	0	0	0.373675	0	0.196422	0	0.123518	0	0
Novel_011914	XLOC_12715.	transcript	0.200805	0.450518	0.119788	0.119056	0.20728	0.189926	0.242839	0.1238	0.128748
Novel_011915	XLOC_12715.	transcript	0.185042	0.383706	0	0	0.255027	0	0	0.210036	0
Novel_011916	XLOC_12722.	transcript	102.8518	13.79218	27.36585	47.71782	35.76873	37.55546	25.7622	63.61102	32.4931
Novel_011917	XLOC_12727.	transcript	0.101688	0.221592	0.055269	0.053076	0.106013	0.150061	0.169396	0.053117	0.155262
Novel_011918	XLOC_12727.	transcript	0	0.033942	0	0	0.382293	0	0.043574	0.046818	0
Novel_011919	XLOC_12738.	transcript	1.264254	1.2161	1.48618	1.470328	1.555096	1.162022	1.96256	1.805035	1.583145
Novel_011920	XLOC_12738.	transcript	0.361259	0.275285	0	0.452709	0.280644	0.510957	0.260523	0.238489	0

Novel_011921	XLOC_12741.	transcript	0	0	0	0	0	0	0	0.178905	0
Novel_011922	XLOC_12741.	transcript	0.004049	0.032857	0	0.309228	0	0.188748	0	0.002781	0.252766
Novel_011923	XLOC_12741.	transcript	0.24668	0	0.105646	0	0.286382	0	0.200258	0.132162	0
Novel_011924	XLOC_12741.	transcript	0.176405	0	0.264618	0.435085	0.31426	0.2116	0.339831	0.226167	0
Novel_011925	XLOC_12741.	transcript	0.107968	0	0.043716	0	0	0	0.056802	0.015614	0
Novel_011926	XLOC_12741.	transcript	0.405965	0.019908	0.396453	0.30717	0.413881	0.291111	0.656181	0.494209	0.140152
Novel_011927	XLOC_12743.	transcript	0.133023	0.000301	0.031988	0.012796	0	0.09079	0.338933	0	0
Novel_011928	XLOC_12743.	transcript	0.12013	0.855328	0.12201	0.106562	0.143126	0.042045	0.00861	0.262579	0.171617
Novel_011929	XLOC_12744.	transcript	0.041867	0.375503	0	0.052424	0	0.037734	0.090289	0.080147	0
Novel_011930	XLOC_12747.	transcript	0	0	0	0	0.016908	0	0	0	0
Novel_011931	XLOC_12747.	transcript	1.210349	0.571226	1.118042	0.744803	1.11171	1.354797	0.75253	0.942218	2.439248
Novel_011932	XLOC_12747.	transcript	0.488435	0.327885	0.412656	0.335949	0.386792	0.647742	0.284454	0.302984	0.805901
Novel_011933	XLOC_12747.	transcript	3.482485	2.421896	3.339417	3.717055	2.737744	4.45412	4.653239	3.336208	6.06395
Novel_011934	XLOC_12750.	transcript	0.628031	0.072434	1.042069	1.75495	0.699094	0.87702	0.815206	1.272567	1.382326
Novel_011935	XLOC_12750.	transcript	0	0	0	0	0	0	0	0	0
Novel_011936	XLOC_12750.	transcript	0	0	0	0	0.355429	0	0	0	0
Novel_011937	XLOC_12753.	transcript	0.001391	0.070449	0.091301	0.018443	0.059526	0.040476	0.025403	0.083719	0.08603
Novel_011938	XLOC_12753.	transcript	0.221556	0.056287	0.248363	0.432572	0.349386	0.294665	0.34441	0.105667	0.33483
Novel_011939	XLOC_12753.	transcript	0	0.07825	0.25103	0.266411	0.170855	0.127393	0.088343	0.145495	0.39511
Novel_011940	XLOC_12753.	transcript	0.09314	0.063591	0.084007	0.16072	0.095615	0.146183	0.100881	0.123868	0.455793
Novel_011941	XLOC_12755.	transcript	0.095094	0.03505	0.152004	0.137349	0.103418	0.15786	0.333952	0.095511	0.126766
Novel_011942	XLOC_12755.	transcript	0.203526	0.002863	0.07441	0.007209	0.021278	0.097447	0.01917	0.029321	0.015363
Novel_011943	XLOC_12757.	transcript	0.296337	0.396793	0.509596	0.458142	0.652576	0.762397	0.609836	0.643805	0.313511
Novel_011944	XLOC_12757.	transcript	0.096143	0.640037	0.146221	0	0	0.067234	0	0.052136	0.429011
Novel_011945	XLOC_12771.	transcript	0.116219	0.027171	0.051989	0.136099	0	0.118795	0.196353	0.140713	0.079466
Novel_011946	XLOC_12774.	transcript	0.349482	0.555352	0.405607	0.339175	0.372027	0.333233	0.446389	0.478967	0.640188
Novel_011947	XLOC_12774.	transcript	0.61232	1.720899	0.736011	0.547157	0.864599	1.076893	0.907521	1.140376	0.441787
Novel_011948	XLOC_12774.	transcript	0	0	0	0	0	0	0	0	0
Novel_011949	XLOC_12776.	transcript	0.664923	0.149573	0.796835	0.443	0.732555	0.687241	0.870553	0.886359	0.564549
Novel_011950	XLOC_12776.	transcript	0	0	0	0	0	0	0	0	0
Novel_011951	XLOC_12776.	transcript	0	0	0	0.16351	0	0	0.155258	0	0
Novel_011952	XLOC_12779.	transcript	0	0	0.320802	0.023448	0.11986	0	0	0.042005	0.154707
Novel_011953	XLOC_12779.	transcript	0	0	0	0	0	0	0.222947	0	0
Novel_011954	XLOC_12780.	transcript	0	1.106735	1.011759	1.719852	1.199903	0	0.656425	0.81264	0.478317
Novel_011955	XLOC_12780.	transcript	0	0	0	0	0	0	0.561225	0	0
Novel_011956	XLOC_12780.	transcript	0.575853	0	0.118401	0.069617	0.101073	0.68938	0.369375	0	0.314141
Novel_011957	XLOC_12781.	transcript	0.770692	0.698762	0.490056	0.472615	0.515052	0.439312	0.943138	0.972425	0.459937
Novel_011958	XLOC_12781.	transcript	0.355231	1.391356	0.478533	0.45687	0.382609	0.580421	0.447622	0.515	0.26615

Novel_011959	XLOC_12782.	transcript	2.113646	0.156384	0.33743	1.03698	1.186347	1.08194	2.441097	1.512277	1.757301
Novel_011960	XLOC_12782.	transcript	0	0	0	0.001321	0	0	0.000429	0	0
Novel_011961	XLOC_12782.	transcript	0	0	0.437519	0.09863	0.34749	0.215319	0.122679	0.138565	0.075697
Novel_011962	XLOC_12785.	transcript	0.08231	0.034109	0	0	0	0.066206	0.096923	0.069014	0
Novel_011963	XLOC_12787.	transcript	0.521027	0.728799	0	0.439735	0.42571	0.561771	0.665006	0.279955	0.506901
Novel_011964	XLOC_12787.	transcript	0.113375	0.167033	0.239167	0.030684	0.02596	0.069077	0.03029	0.214208	0.070515
Novel_011965	XLOC_12787.	transcript	0	0.467615	0	0	0	0	0	0	0
Novel_011966	XLOC_12789.	transcript	0.089185	0.143033	0.091227	0.096427	0.411471	0.30239	0.198029	0.112315	0.102631
Novel_011967	XLOC_12789.	transcript	0	0.085376	0.17927	0.005317	0.25574	0.341332	0.039415	0.117515	0.406255
Novel_011968	XLOC_12789.	transcript	0	0	0	0	0.010633	0	0	0	0
Novel_011969	XLOC_12789.	transcript	0	0.467095	0.40481	0.42287	0.66442	0.492	0.900513	0.405252	0.84367
Novel_011970	XLOC_12790.	transcript	0.100957	0.555622	0.055496	0.009521	0.058031	0.023066	0.052802	0.006113	0.035902
Novel_011971	XLOC_12790.	transcript	0	0.15268	0	0	0	0	0	0.035177	0
Novel_011972	XLOC_12790.	transcript	0	0.223391	0	0	0	0	0.000915	0	0
Novel_011973	XLOC_12797.	transcript	0.120974	0.144224	0.318553	0.285692	0.454043	0.254021	0.23996	0.39689	0.209876
Novel_011974	XLOC_12797.	transcript	0	0	0	0	0	0	0	0	0
Novel_011975	XLOC_12803.	transcript	0.051627	0.135368	1.222103	1.091738	1.463837	1.679852	0	0.17007	1.269005
Novel_011976	XLOC_12808.	transcript	0.0357	0.103163	0.037742	0	0.200824	0.086146	0.118992	0.020172	0.037063
Novel_011977	XLOC_12810.	transcript	0.283965	0.330134	0.484883	0.313345	0.205443	0.685744	0.001578	0.457729	0.241115
Novel_011978	XLOC_12810.	transcript	0	0.7967	0	0.216846	0.099794	0.169907	0.102232	0.096259	0.679791
Novel_011979	XLOC_12810.	transcript	0.058513	0.742915	0	0.011111	0.077292	0	0.001622	0	0.177947
Novel_011980	XLOC_12810.	transcript	0.112631	0.192067	0.092261	0	0.215138	0.017729	0.333227	0.163738	0.251349
Novel_011981	XLOC_12810.	transcript	0	0.477608	0.262865	0.172138	0	0.002262	0.285956	0.090191	0.028619
Novel_011982	XLOC_12810.	transcript	0	0.25251	0.036118	0.086742	0.064447	0.163306	0.113513	0.037453	0.159341
Novel_011983	XLOC_12810.	transcript	0.096946	0.179547	0.108103	0	0.149114	0.403132	0.138267	0.160705	0
Novel_011984	XLOC_12810.	transcript	0.020359	0	0	0	0	0	0.028819	0.070592	0
Novel_011985	XLOC_12810.	transcript	0.313417	0.466787	0.14066	0.191232	0.249228	0.303484	0.289515	0.231831	0.637955
Novel_011986	XLOC_12810.	transcript	0.016631	0.07977	0.025165	0.009918	0.030736	0.031285	0.125469	0.083503	0.037954
Novel_011987	XLOC_12810.	transcript	0	0.228299	0.593644	0.119389	0.310738	0.117446	0.106296	0.153067	0.175342
Novel_011988	XLOC_12816.	transcript	0.165289	0	0.1399	0.12781	0.342001	0.108781	0.299715	0.178203	0.189845
Novel_011989	XLOC_12816.	transcript	0.298935	0	0.188861	0.296297	0.262496	0.115246	0.208068	0.095133	0.097369
Novel_011990	XLOC_12820.	transcript	0.098414	1.232823	0.068852	0.037471	0.015297	0	0.140755	0.047562	0.079173
Novel_011991	XLOC_12822.	transcript	1.755899	0.856725	2.225267	0.953225	1.880686	1.31714	2.536636	1.807174	4.062558
Novel_011992	XLOC_12823.	transcript	0.162127	0.001634	0.219923	0.140842	0.17944	0.248826	0.147524	0.332365	0.253768
Novel_011993	XLOC_12823.	transcript	0.080552	0.164388	0.022891	0.019873	0.035414	0.04539	0.034931	0.028736	0.023311
Novel_011994	XLOC_12823.	transcript	0.000597	0.15361	0	0.013518	0.000364	0.057412	0.003587	0.06375	0.051348
Novel_011995	XLOC_12823.	transcript	0.183425	0.269588	0.133884	0.085387	0.224816	0.114162	0.205597	0.085566	0
Novel_011996	XLOC_12823.	transcript	0.335509	0	0.23791	0.312604	0.083635	0	0.312687	0.040904	0.101049

Novel_011997	XLOC_12823.	transcript	0.297944	0.189288	0.260213	0.23443	0.188908	0.12461	0.21221	0.184777	0.020479
Novel_011998	XLOC_12826.	transcript	0	0.049718	0.152844	0	0	0.161945	0.282467	0.128301	0.09789
Novel_011999	XLOC_12826.	transcript	0.323948	0	0.037191	0.22408	0.22777	0	0	0	0
Novel_012000	XLOC_12826.	transcript	0.428055	0.340905	0.079441	0.376627	0.397826	0.292529	0.590124	0.421364	0.194631
Novel_012001	XLOC_12831.	transcript	0.198718	0.124389	0.036248	0.177929	0.072357	0.056803	0.047943	0.144311	0.100625
Novel_012002	XLOC_12831.	transcript	0	0	0	0	0	0	0.108495	0	0
Novel_012003	XLOC_12832.	transcript	0.572945	0.309649	0.547881	0.099875	0.169796	0.55737	0.777114	0.544607	0.435248
Novel_012004	XLOC_12836.	transcript	0	0.038208	0.024369	0	0	0.027718	0.034479	0	0.027318
Novel_012005	XLOC_12837.	transcript	0.181553	0.434955	0.253526	0.205816	0.177517	0.26166	0.276976	0.271379	0.21612
Novel_012006	XLOC_12837.	transcript	0	0.013233	0	0.008175	0	0	0.013886	0	0.010427
Novel_012007	XLOC_12837.	transcript	0	0	0	0	0.237146	0	0	0.003046	0
Novel_012008	XLOC_12837.	transcript	0	0	0.013647	0	0	0	0	0	0
Novel_012009	XLOC_12837.	transcript	0	0.029929	0	0	0	0.097287	0	0.110258	0.009426
Novel_012010	XLOC_12837.	transcript	0.249576	0.05749	0.079654	0.123118	0.16355	0.182023	0.31391	0.217532	0.03726
Novel_012011	XLOC_12837.	transcript	0.124314	0.065792	0.381423	0.152309	0.058939	0.043368	0.521446	0.209846	0.057476
Novel_012012	XLOC_12838.	transcript	0	0.404406	0	0	0	0	0	0	0
Novel_012013	XLOC_12838.	transcript	0.01836	0	0	0.067211	0.046785	0.883041	0	0	0
Novel_012014	XLOC_12838.	transcript	0.965805	0.34486	0.757859	0.098871	0.088787	0.025502	1.595838	0.26625	0.680411
Novel_012015	XLOC_12838.	transcript	0	0.016445	0	0.013102	0	0.016145	0.036544	0.294184	0
Novel_012016	XLOC_12840.	transcript	0.224987	1.201852	0.153039	0.186018	0.179104	0.116295	0.234713	0.18315	0.171889
Novel_012017	XLOC_12842.	transcript	0.05039	0	1.331837	0	0.617775	0	0.282649	0.028851	0.238876
Novel_012018	XLOC_12847.	transcript	0.16541	0.094407	0.492924	0.414987	0.452198	0.461281	0.493456	0.405362	0.563714
Novel_012019	XLOC_12847.	transcript	0.239139	0.017355	0.24942	0.114828	0.378224	0.350325	0.522811	0.276707	0.325156
Novel_012020	XLOC_12851.	transcript	0.253953	0.343328	0.208953	0.26093	0.501686	0.331922	0.523481	0.33553	0.322955
Novel_012021	XLOC_12851.	transcript	0	0	0.134303	0.230937	0.045207	0.041297	0.051796	0.009417	0.224503
Novel_012022	XLOC_12851.	transcript	0.075135	0.160872	0.243799	0.064054	0.196063	0.195997	0.453497	0.102989	0.046751
Novel_012023	XLOC_12851.	transcript	0.366882	0.026471	0.163962	0.110051	0.271608	0.091749	0.148378	0.304236	0.076636
Novel_012024	XLOC_12851.	transcript	0.260361	0.426188	0.135331	0.232216	0.246157	0.418407	0.541493	0.211849	0.375182
Novel_012025	XLOC_12851.	transcript	0	0	0.12775	0	0	0	0	0	0
Novel_012026	XLOC_12851.	transcript	0	0	0	0	0	0	0	0	0
Novel_012027	XLOC_12851.	transcript	0.188203	0.033934	0.002247	0.007383	0.001436	0.056999	0.181362	0.151512	0.025105
Novel_012028	XLOC_12851.	transcript	0.09752	0.004896	0.00152	0.000642	0.001819	0	0.126912	0.071423	0.00122
Novel_012029	XLOC_12851.	transcript	0.164701	0.109333	0.125878	0.185901	0.126863	0.13705	0.33448	0.16041	0.077553
Novel_012030	XLOC_12851.	transcript	0.090076	0.231625	0.357868	0	0.333159	0	0	0.053904	0
Novel_012031	XLOC_12851.	transcript	0	0	0.111181	0.213153	0	0	0.466312	0	0
Novel_012032	XLOC_12853.	transcript	1.488165	1.187591	1.686745	1.648193	1.887168	1.488744	2.576918	1.859821	1.371525
Novel_012033	XLOC_12853.	transcript	0.020064	0.486364	0.073197	0.015116	0.041416	0.043627	0.089413	0.057562	0.048372
Novel_012034	XLOC_12853.	transcript	0.068337	0.196454	0.086062	0.111138	0.310677	0.180781	0.522687	0.217205	0.237065

Novel_012035	XLOC_12853.	transcript	0	0	0	0	0	0	1.120097	0	0
Novel_012036	XLOC_12853.	transcript	0.16065	0.921809	0.8286	0.183762	0.151899	0.226637	0.000461	0.200309	0.823399
Novel_012037	XLOC_12853.	transcript	0.598287	0.120641	0.061234	0.803494	0.949072	0.685272	0.092877	0.629049	0.132634
Novel_012038	XLOC_12853.	transcript	0.610734	0	0.433024	0.37631	0.089513	0.390102	0.835408	0.441579	0
Novel_012039	XLOC_12855.	transcript	0	0.407074	0.864621	0	0	0	0	0	0.008296
Novel_012040	XLOC_12855.	transcript	0.153353	0	0	0.151569	0.056336	0	0.246735	0.27586	0
Novel_012041	XLOC_12855.	transcript	0.03591	0.428494	0.071387	0.193674	0.073599	0.000304	0.181301	0.300174	0.730255
Novel_012042	XLOC_12855.	transcript	0.573522	0.774442	0.613155	0.732167	0.65967	0.82467	0.909229	0.750821	0.49235
Novel_012043	XLOC_12855.	transcript	0.140605	0.062807	0.206119	0.386565	0.205715	0	0.188762	0.085672	0.1477
Novel_012044	XLOC_12855.	transcript	0	0.159345	0	0	0	0	0	0.009425	0
Novel_012045	XLOC_12855.	transcript	0.223704	0.486318	0.23313	0.282708	0.39971	0.177595	0.417241	0.427799	0.280978
Novel_012046	XLOC_12855.	transcript	0.035401	0.063014	0.049594	0.010236	0.036069	0.035398	0.0754	0.191339	0.021391
Novel_012047	XLOC_12855.	transcript	0.326876	0.084907	0.62053	0	0.049004	0.757561	0.806011	0.160342	0.811921
Novel_012048	XLOC_12855.	transcript	0	0	0	0	0	0	0	0	0
Novel_012049	XLOC_12856.	transcript	0.03069	0.351017	0.555496	0.073165	0.329291	0	0.484338	0.713214	0.410776
Novel_012050	XLOC_12856.	transcript	0.672699	0.468169	0.715797	0.596208	0.978405	0.46015	0.513333	0.198479	0.377376
Novel_012051	XLOC_12856.	transcript	0.105586	0.110347	0	0.894132	0	0.037374	0	1.144589	0
Novel_012052	XLOC_12856.	transcript	0	0	0.072761	0	0.031707	0	0.114986	0	0.055941
Novel_012053	XLOC_12856.	transcript	0.121507	0.200312	0.202396	0.473628	0.203115	0.354278	0.182652	0.093341	0.218486
Novel_012054	XLOC_12856.	transcript	0.12928	0.215609	0.471982	0.250823	0.182925	0.205894	0.533184	0.449486	0.118674
Novel_012055	XLOC_12856.	transcript	0.09857	0.353295	0.340495	0.102057	0.44288	0.129243	0.138594	0.099777	0.351517
Novel_012056	XLOC_12856.	transcript	0	0	0	0	0	0	0	0.000444	0
Novel_012057	XLOC_12856.	transcript	0.103955	0.035135	0	0.0507	0.076243	0.157459	0.10348	0.107279	0.093221
Novel_012058	XLOC_12856.	transcript	0.119227	0.006288	0	0.130525	0.000163	0.187401	0.093944	0.220879	0.201372
Novel_012059	XLOC_12856.	transcript	0	0	0	0	0	0	0	0	0
Novel_012060	XLOC_12856.	transcript	0.281559	0	0	0.275475	0	0	0.253324	0.283041	0.165999
Novel_012061	XLOC_12856.	transcript	0	0.002535	0.009969	0	0.003685	0.000719	0	0	0
Novel_012062	XLOC_12856.	transcript	0.13889	0.024631	0	0.198626	0	0	0.159801	0.169855	0.045007
Novel_012063	XLOC_12858.	transcript	0	0	0	0.033802	0.257929	0	0.024762	0.205838	0.019423
Novel_012064	XLOC_12864.	transcript	1.448952	1.813129	2.487939	2.008876	1.321956	2.161446	1.249015	1.378649	1.454424
Novel_012065	XLOC_12867.	transcript	0.29844	0.609085	0	0	0	0	0.106809	0.220654	0.053934
Novel_012066	XLOC_12867.	transcript	0.090867	0.254073	0.205264	0.383108	0.246421	0.206473	0.149682	0.198786	0.080897
Novel_012067	XLOC_12867.	transcript	0.51261	0.092408	0.182367	0	0.243926	0.127256	0.534234	0.402252	0.49863
Novel_012068	XLOC_12867.	transcript	0	0.027764	0	0	0	0.0757	0.155164	0.211256	0
Novel_012069	XLOC_12867.	transcript	0	0	0	0	0	0.038429	0	0	0.175008
Novel_012070	XLOC_12867.	transcript	0	0	0	0	0	0.11098	0.097739	0	0.429855
Novel_012071	XLOC_12877.	transcript	0	0.881629	0.856326	1.15334	0.037852	1.164009	0.737214	1.182908	1.036216
Novel_012072	XLOC_12878.	transcript	0.076996	0.132228	0.094426	0.166026	0.022275	0.150657	0.15466	0	0.044902

Novel_012073	XLOC_12880.	transcript	0.100033	0.04694	0.124849	0.28926	0.167133	0.12022	0.295203	0.165352	0.110657
Novel_012074	XLOC_12880.	transcript	0	0.287066	0.328093	0.319611	0.048929	0.965098	0	0	0
Novel_012075	XLOC_12880.	transcript	0	0	0	0	0	0	0	0.267873	0
Novel_012076	XLOC_12880.	transcript	0	0	0.032128	0.063933	0.01765	0.042542	0	0.062716	0
Novel_012077	XLOC_12880.	transcript	0	0.039071	0.01639	0	0	0	0.039776	1.403099	0
Novel_012078	XLOC_12880.	transcript	0	0	0	0	0	0	0	0.346336	0.594867
Novel_012079	XLOC_12880.	transcript	0.187054	0.001284	0.292373	0.042374	0.676172	0.001547	0.227604	0.171802	0.010707
Novel_012080	XLOC_12880.	transcript	1.137682	0	0.205255	0.014984	0.247535	0	0.087396	0	0.047011
Novel_012081	XLOC_12880.	transcript	0.015986	0	0	0	0	0	0	0.159277	0.001138
Novel_012082	XLOC_12880.	transcript	0	1.568474	0.828399	1.544976	1.225434	1.990122	1.824225	0.216655	1.174642
Novel_012083	XLOC_12881.	transcript	0.46941	1.38284	0.521135	0.256239	0.315844	0	0.391506	0.068278	0.311394
Novel_012084	XLOC_12881.	transcript	0	1.850574	0	0	0	0.678645	0	0.785614	0
Novel_012085	XLOC_12881.	transcript	0.38098	0	0	0	0	0	0	0	0
Novel_012086	XLOC_12883.	transcript	0	0.243325	0.019523	0	0	0	0.365607	0	0.001434
Novel_012087	XLOC_12883.	transcript	0	0.000439	0.095526	0.014658	0.527836	0.006118	0.002734	0.037644	0.16512
Novel_012088	XLOC_12883.	transcript	0.077073	0.0042	0.019218	0.006901	0.005323	0.018725	0.001471	0.063205	0.003491
Novel_012089	XLOC_12883.	transcript	0	0	0	0.039621	0	0	0	0	0
Novel_012090	XLOC_12883.	transcript	0.000843	0	0.008503	0	0.051638	0	0	6.80E-05	0
Novel_012091	XLOC_12883.	transcript	0.216838	0.007213	0	0	0.006235	0	0	0.01877	0.003087
Novel_012092	XLOC_12883.	transcript	0	0	0.047924	0.06519	0.018606	0.259443	0.002876	0.0227	0
Novel_012093	XLOC_12883.	transcript	0.179753	0.1834	0.417937	0.314678	0.109399	0.057286	0	0.208272	0.082843
Novel_012094	XLOC_12883.	transcript	0	0	0	0	0	0	0	0.350485	0.006443
Novel_012095	XLOC_12888.	transcript	0	0.021108	0.409676	0.663065	0.108887	0.587248	0.982447	0.449181	0.608227
Novel_012096	XLOC_12888.	transcript	0	0.102211	0.162497	0.370706	0	0	0.086047	0.122733	0
Novel_012097	XLOC_12888.	transcript	0.801729	0.122509	0.158966	0.053218	0.514576	0.45673	0.168969	0.124179	0.44091
Novel_012098	XLOC_12890.	transcript	2.10648	0	0.700878	0.382832	0.7809	0.736525	0.533645	1.036129	0.089622
Novel_012099	XLOC_12893.	transcript	0.299397	0.479152	0.717616	0.587873	0.413456	0.588005	0.520857	0.471766	0.525327
Novel_012100	XLOC_12900.	transcript	0.158643	0.063376	0.117297	0.419064	0.071493	0.308205	0.459477	0.489165	0.297168
Novel_012101	XLOC_12914.	transcript	0.407246	0.099882	0.269278	0	0.012204	0	0	0.016786	0.015956
Novel_012102	XLOC_12914.	transcript	0.26318	0	0	0.278921	0.019773	0.198738	0.478845	0.070846	0.019067
Novel_012103	XLOC_12914.	transcript	0	0	0	0.006314	0	0	0	0	0
Novel_012104	XLOC_12914.	transcript	0.505424	0.243052	0.940395	0.541413	0.727052	0.548342	0.41988	0.670263	0.776832
Novel_012105	XLOC_12915.	transcript	0	0.099532	0	0.059614	0	0.067743	0	0.061315	0.034578
Novel_012106	XLOC_12915.	transcript	0.331558	0	0.295316	0.147742	0.188999	0.319703	0.163229	0.303077	0.121737
Novel_012107	XLOC_12917.	transcript	0.158654	0.000838	0.058147	0.007108	0.050191	0	0.063311	0.110686	0.206119
Novel_012108	XLOC_12917.	transcript	0.043399	0.052531	0	0.116006	0	0.049329	0.171186	0.115084	0
Novel_012109	XLOC_12917.	transcript	0.041141	0.070954	0.038247	0.03076	0.170154	0.11996	0.096475	0.055953	0.006882
Novel_012110	XLOC_12917.	transcript	0.148395	0.009581	0.000449	0.000343	0.109392	0.000702	0.014965	0.000307	0.118388

Novel_012111	XLOC_12917.	transcript	0.075422	0.038131	0.136329	0.173591	0.050686	0.151388	0.085115	0.173367	0.126274
Novel_012112	XLOC_12917.	transcript	0.040054	0.037496	0.22496	0.096284	0.114161	0.110146	0.063295	0.06384	0.004253
Novel_012113	XLOC_12918.	transcript	0.533985	0.029327	0.248553	0.35615	0.281039	0.270943	0.40149	0.525467	0.457644
Novel_012114	XLOC_12928.	transcript	0	0	0.352138	0	0.102201	0	0	0	0.394742
Novel_012115	XLOC_12928.	transcript	0	0	0	0	0	0	0	0	0
Novel_012116	XLOC_12928.	transcript	0.3603	0.243488	0.379192	0.311561	0.473917	0.364048	0.259083	0.34504	0.470356
Novel_012117	XLOC_12928.	transcript	0	0	0.030222	0	0	0	0	0	0
Novel_012118	XLOC_12942.	transcript	0.051779	0	0	0.079911	0.084042	0	0	0.085518	0.059005
Novel_012119	XLOC_12943.	transcript	0.366072	0	0.098937	0.158117	0.17029	0.149778	0.215386	0.170786	0.695882
Novel_012120	XLOC_12943.	transcript	0.576919	0.098321	0.972035	0.289242	0.574505	0.459242	0.779977	0.838464	0.150432
Novel_012121	XLOC_12943.	transcript	0.137685	0.018019	0.144891	0.147969	0.483132	0.111253	0.721385	0.34311	0.036574
Novel_012122	XLOC_12943.	transcript	0	0	1.743408	0	0	0	0.002229	0.591595	0
Novel_012123	XLOC_12943.	transcript	0.751444	0	0.613265	0.396759	0.781906	1.057076	0.991342	0.236445	0.165797
Novel_012124	XLOC_12943.	transcript	0.10195	0	0.1558	0.014331	0.136851	0.184789	0.298535	0.199231	0.162363
Novel_012125	XLOC_12943.	transcript	0	0	0	0.317416	0	0.235573	0	0.015272	0.454806
Novel_012126	XLOC_12943.	transcript	0.546866	0.209458	0.189625	1.313217	0.613616	0	0.894084	0.780799	0.283286
Novel_012127	XLOC_12946.	transcript	0	0	0	0	0	0	0.714098	0.296989	0
Novel_012128	XLOC_12946.	transcript	3.063761	2.3543	2.441449	2.996323	3.091074	2.28635	3.186007	3.428068	2.808585
Novel_012129	XLOC_12946.	transcript	0	0	0	0	0	0	0	0.025741	0
Novel_012130	XLOC_12947.	transcript	0.008213	0	0	0	0	0.376182	0.700925	0.006758	0
Novel_012131	XLOC_12947.	transcript	1.600761	2.064905	0.949323	0.68081	0	0	0	0	0
Novel_012132	XLOC_12947.	transcript	0	1.25201	0	0	1.40E-05	0.355822	0.739957	6.30E-05	0.805606
Novel_012133	XLOC_12947.	transcript	0	0	0	0.394484	0	0.214026	0.000924	0.013259	0
Novel_012134	XLOC_12947.	transcript	0.053924	0.002452	0.059213	0.000638	0.466275	0	0.049362	1.386287	0.122151
Novel_012135	XLOC_12947.	transcript	0.029023	0	0	0.299972	0.414363	0.15724	0	0.131139	0
Novel_012136	XLOC_12949.	transcript	0	0	0.005485	0	0	0	0	0	0
Novel_012137	XLOC_12949.	transcript	0	0	0	0	0	0	0	0	0
Novel_012138	XLOC_12949.	transcript	0.598104	0.440425	0.618731	0.359897	0.326277	0.747537	0.873593	0.50285	0.422684
Novel_012139	XLOC_12949.	transcript	0.24347	0.002691	0.245714	0.154137	0.338615	0.00511	0.175406	0.239609	0.008323
Novel_012140	XLOC_12949.	transcript	0.095049	0	0	0.18752	0.125963	0	0	0.093515	0
Novel_012141	XLOC_12949.	transcript	0.143549	0	0	0.077608	0	0	0	0	0
Novel_012142	XLOC_12949.	transcript	0.194108	0	0.127039	0.102502	0.087862	0	0.180895	0.068262	0
Novel_012143	XLOC_12956.	transcript	0.098817	0	0.26128	0.187154	0.342009	0.430176	0.513548	0.155979	0.089679
Novel_012144	XLOC_12956.	transcript	0.288652	0	0.160776	0.146476	0.249464	0	0.37949	0.251457	0.143504
Novel_012145	XLOC_12957.	transcript	0	0	0	0	0.457353	0	0.585761	0.427088	0.464197
Novel_012146	XLOC_12957.	transcript	0.429984	0.638178	0.345573	0.424055	0.116654	0.438741	0.276545	0.078398	0.116321
Novel_012147	XLOC_12957.	transcript	0.629561	0	0.467265	0.222351	0.385919	0.368504	1.391715	0.727567	0
Novel_012148	XLOC_12957.	transcript	0.676164	0.607279	0.171877	0.536369	0.355311	0.470251	0.043476	0.003938	0.463591

Novel_012149	XLOC_12957.	transcript	0.212772	0	0.021088	0.049322	0	0	0.001266	0.465569	0
Novel_012150	XLOC_12958.	transcript	0.161652	0.226682	0.22801	0.071266	0.19049	0.144639	0.100628	0.370866	0.126844
Novel_012151	XLOC_12958.	transcript	0	0	0.229692	0.016048	0.071568	0	0.050362	0.02973	0.292895
Novel_012152	XLOC_12958.	transcript	0.026612	0	0	0.020904	0	0	0	0.006286	0
Novel_012153	XLOC_12960.	transcript	0	0	0	0	0	0	0	0.135469	0
Novel_012154	XLOC_12960.	transcript	0.475512	0.411447	0.398934	0.170894	0.382592	0.302395	0.35448	0.282049	0.326723
Novel_012155	XLOC_12961.	transcript	0.417823	1.281644	0.414576	0.228081	0.493311	0.495523	0.513908	0.355285	0.199631
Novel_012156	XLOC_12969.	transcript	0	1.851259	0	0	0	0	0	0	0
Novel_012157	XLOC_12969.	transcript	0	0.394986	0	0	0	0	0	0	0
Novel_012158	XLOC_12975.	transcript	0.46736	0	0.428096	0.266734	0.514513	0.276962	0.749324	0.496464	0.382204
Novel_012159	XLOC_12975.	transcript	0.288635	0.142341	0.237991	0.218669	0.399645	0.223418	0.21704	0.321408	0.13048
Novel_012160	XLOC_12987.	transcript	0.002215	0	0	0	0	0	0.134092	0	0
Novel_012161	XLOC_12987.	transcript	0.45294	0.272993	0.608155	0.16974	0.26285	0.609867	0.662677	0.66472	0.375967
Novel_012162	XLOC_12987.	transcript	0.246537	0.218538	0.115872	0.037096	0.405571	0.100335	0.225098	0.131987	0.102623
Novel_012163	XLOC_12987.	transcript	0.07874	0.162361	0.159233	0.169376	0.153631	0.212408	0.084884	0.20394	0.086838
Novel_012164	XLOC_12987.	transcript	0.074814	0.14629	0	0.122763	0.152086	0	0.077974	0.102343	0
Novel_012165	XLOC_12991.	transcript	0.614867	0.712905	0.29871	0.22591	0.338641	0.431986	0.621468	0.359618	0.275011
Novel_012166	XLOC_12996.	transcript	0.151593	0.029461	0.302936	0.151331	0.136217	0.096377	0.178537	0.148084	0.051748
Novel_012167	XLOC_13023.	transcript	0.254708	1.205915	0.15346	0.457376	0.507608	0	0.329823	0.446061	0.113143
Novel_012168	XLOC_13023.	transcript	0	1.603518	0.166787	0.357206	0.093258	0.314313	0.313375	0.197218	0
Novel_012169	XLOC_13023.	transcript	0.017857	0	0	0	0	0	0.092666	0	0.005292
Novel_012170	XLOC_13023.	transcript	0.435165	0.13589	0.326661	0.282047	0.274668	0.367617	0.425626	0.339841	0.332611
Novel_012171	XLOC_13025.	transcript	1.420347	0.70356	0.735383	0.665916	1.311422	0.961868	1.443074	1.235456	0.66513
Novel_012172	XLOC_13025.	transcript	0	0	0.471387	0	0	0	0	0	0
Novel_012173	XLOC_13032.	transcript	0.406451	0.126553	0.108616	0.174842	0.221084	0.137401	0.335774	0.27834	0.168162
Novel_012174	XLOC_13032.	transcript	0	0	0.004761	0	0.023026	0.012626	0.043298	0	0.01561
Novel_012175	XLOC_13032.	transcript	0	0	0	0	0	0	0	0	0
Novel_012176	XLOC_13037.	transcript	0.164112	0.172162	0	0.067845	0	0.193356	0	0	0.024871
Novel_012177	XLOC_13037.	transcript	0	0.325495	0	0.128721	0.152306	0	0.250412	0.262542	0
Novel_012178	XLOC_13042.	transcript	0.090823	0.031694	0.131395	0.041879	0.040135	0.103582	0.153272	0.099436	0.270979
Novel_012179	XLOC_13042.	transcript	0	0	0	0	0	0	0	0	0
Novel_012180	XLOC_13045.	transcript	0.21961	0	0.23962	0	0.231684	0	0.016353	0.001168	0
Novel_012181	XLOC_13045.	transcript	0	0.407347	0	0.232335	0	0.32738	0.226109	0.236775	0.358942
Novel_012182	XLOC_13046.	transcript	0.376428	0.150192	0	0.060958	0.001726	0.112981	0.084502	0.152876	0.202928
Novel_012183	XLOC_13046.	transcript	0.177321	0.213572	0	0.076008	0.27584	0.306646	0.459763	0.098288	0.089571
Novel_012184	XLOC_13046.	transcript	0.163866	0.134221	0	0.306562	0.118499	0.321862	0.110551	0.228292	0.100267
Novel_012185	XLOC_13046.	transcript	0	0.528268	0	0.059848	0	0	0	0	0.111053
Novel_012186	XLOC_13046.	transcript	0.366701	0	0	0.053565	0	0	0.055039	0.037423	0.124825

Novel_012187	XLOC_13046	transcript	0	0.075615	0.059247	0.12447	0	0.070203	0	0	0
Novel_012188	XLOC_13046	transcript	0.147501	0	0	0.145129	0.154099	0	0	0	0.099232
Novel_012189	XLOC_13046	transcript	0.12836	0.151235	0	0.288056	0	0.049448	0.082479	0.09162	0.16297
Novel_012190	XLOC_13046	transcript	0	0	0	0	0	0.009974	0	0	0
Novel_012191	XLOC_13046	transcript	0.108282	0.096181	0	0.053957	0	0.183814	0.1944	0.13909	0.121998
Novel_012192	XLOC_13046	transcript	0.089729	0	0	0.064676	0	0	0	0	0.16123
Novel_012193	XLOC_13046	transcript	0.103007	0	0	0.0473	0	0	0	0	0.12237
Novel_012194	XLOC_13046	transcript	0	0	0	0	0	0	0	0	0
Novel_012195	XLOC_13046	transcript	0	0	0.837328	0.104142	0.215964	0.050797	0.204197	0.193825	0.080918
Novel_012196	XLOC_13046	transcript	0	0	0.31852	0	0.265362	0	0.384486	0.414283	0
Novel_012197	XLOC_13046	transcript	0	0	0.134718	0.057064	0.10407	0.187501	0.320728	0.256255	0.170107
Novel_012198	XLOC_13046	transcript	0.078549	0.002497	0.338681	0.046698	0.334237	0.239192	0.127039	0.336855	0.053305
Novel_012199	XLOC_13046	transcript	0	0	0	0.115149	0.327048	0	0	0	0.107024
Novel_012200	XLOC_13046	transcript	0	0	0	0	0.373974	0	0	0	0
Novel_012201	XLOC_13047	transcript	0.41544	0.626995	0.445822	0.856302	0.630709	0.436198	0.810426	0.819035	0.327952
Novel_012202	XLOC_13050	transcript	0.222759	0	0.020099	0.187064	0.124835	0.157201	0.149294	0.096477	0.30199
Novel_012203	XLOC_13051	transcript	0.183012	0	0.192031	0	0.111217	0	0.019356	0.061071	0
Novel_012204	XLOC_13051	transcript	0.070833	0	0.024944	0	0.205322	0	0.194754	0.121516	0
Novel_012205	XLOC_13055	transcript	0.240384	1.131026	0.385626	0.538435	0.395034	0.382182	0.392253	0.43832	0.283537
Novel_012206	XLOC_13056	transcript	0.023482	0.327866	0.273236	0.090748	0.067353	0.315465	0.022607	0.263291	0.384319
Novel_012207	XLOC_13056	transcript	0	0.461445	0.045025	0.072221	0.040357	0	0.015057	0.210632	0
Novel_012208	XLOC_13056	transcript	0.310264	0.004643	0.060296	0.237964	0.388334	0.014045	0.70133	0.011844	0.013891
Novel_012209	XLOC_13058	transcript	0.829425	1.782252	1.275057	1.487473	4.780229	1.58683	0.929516	0.816263	3.553298
Novel_012210	XLOC_13060	transcript	0	0	0	0	0	0.350053	0	0	0
Novel_012211	XLOC_13060	transcript	0.378897	0.624679	1.499675	1.102594	1.076302	1.089563	0.297089	1.241459	0.463767
Novel_012212	XLOC_13060	transcript	0.474426	0.617079	0.788892	0.943449	0.46026	0.437529	0.176584	0.937243	0.75725
Novel_012213	XLOC_13062	transcript	0.540174	0.858082	0.372903	0.35832	0.443531	0.463321	0.627922	0.507784	0.573355
Novel_012214	XLOC_13074	transcript	0	0	2.411407	0	0.010301	0	0.186393	0.1815	0.576856
Novel_012215	XLOC_13074	transcript	0.36795	0	0.044006	0.90005	0.499939	1.890158	0.582832	0.934504	0.492994
Novel_012216	XLOC_13074	transcript	0.023312	0.007771	0	0.013395	0.245058	0.006054	0.007013	0.158065	0
Novel_012217	XLOC_13074	transcript	0.025262	0	4.749588	0.017296	0.005066	0.991689	0.015178	0.472395	1.044698
Novel_012218	XLOC_13074	transcript	1.586375	1.212091	2.194011	0	0.505964	0.938431	0.176537	0.502701	0
Novel_012219	XLOC_13074	transcript	5.739018	0.781702	2.054539	5.576603	1.367509	5.942925	2.340491	6.966624	4.296061
Novel_012220	XLOC_13074	transcript	0	0	0	0	0	0	0	0	0
Novel_012221	XLOC_13074	transcript	0.679926	0.710896	0.388095	0.746823	0.433905	0.322053	0.808452	1.049669	0.359355
Novel_012222	XLOC_13076	transcript	0.368443	0.642317	0.416102	0.314544	0.270115	0.403602	0.460333	0.371898	0.478346
Novel_012223	XLOC_13076	transcript	0	0.45673	0	0	0	0	0	0	0
Novel_012224	XLOC_13081	transcript	0	0	0	0.00361	0	0	0	0.003351	0

Novel_012225	XLOC_13081.	transcript	0.008655	0.012679	0.025472	0.015988	0.033348	0.057056	0.541016	0.003422	0
Novel_012226	XLOC_13081.	transcript	0.814476	1.058942	0.788655	0.767013	0.578674	0.524143	0.78538	0.93314	0.678728
Novel_012227	XLOC_13089.	transcript	0	0	0.134153	9.60E-05	0.086132	0	0.113982	0.102544	0
Novel_012228	XLOC_13089.	transcript	0.701417	0	1.694934	0.669768	0.669237	2.007746	1.208536	0.419855	1.336202
Novel_012229	XLOC_13090.	transcript	0.024691	0	0.260688	0.337445	0.324624	0.476645	0.479935	0.12723	0.158111
Novel_012230	XLOC_13090.	transcript	91.46629	191.5614	186.1682	160.2274	155.0988	255.6885	154.5289	152.9522	258.6602
Novel_012231	XLOC_13091.	transcript	0.232263	0.31189	0.368521	0.391272	0.361321	0.424596	0.294921	0.435778	0.411487
Novel_012232	XLOC_13097.	transcript	0.321144	0.629937	0.315742	0	0.287693	0.369138	0.096113	0.431456	0.396952
Novel_012233	XLOC_13097.	transcript	0.017499	0.033013	0	0.38172	0	0	0.381959	0.033683	0.012939
Novel_012234	XLOC_13097.	transcript	0.297829	1.31357	0.72358	0.547323	0.316211	0.457919	0.289023	0.523803	0.344201
Novel_012235	XLOC_13097.	transcript	1.384053	3.586455	1.116582	1.422318	1.14995	1.475305	1.628692	1.834905	1.607882
Novel_012236	XLOC_13097.	transcript	0.609157	0.136395	0.505027	0.158762	0.21205	0.150818	0.246735	0.231047	0.234959
Novel_012237	XLOC_13097.	transcript	0.056271	0.109758	0.152158	0.126267	0.167457	0	0	0.130773	0.227756
Novel_012238	XLOC_13097.	transcript	0.116794	0.566388	0	0.319122	0.271731	0.54663	0.674259	0.173066	0.530119
Novel_012239	XLOC_13097.	transcript	0	0.407152	0	0.145438	0.189257	0.042989	0	0.15162	0.254626
Novel_012240	XLOC_13098.	transcript	0	0	0	0	0	0.003326	0	0.001539	0
Novel_012241	XLOC_13098.	transcript	1.136348	0.615658	0.591792	0.790749	1.023565	0.722858	1.366358	1.264415	1.032646
Novel_012242	XLOC_13098.	transcript	0	0	0.417489	0	0	0	0	0	0
Novel_012243	XLOC_13098.	transcript	0	1.345592	0	0	0	0	0	0	0
Novel_012244	XLOC_13099.	transcript	0.002242	0	0	0	0	0	0	0	0
Novel_012245	XLOC_13099.	transcript	0.311323	0.009641	0.022464	0.182859	0.017656	0.182367	0.406425	0.157874	0.016231
Novel_012246	XLOC_13099.	transcript	0.286278	0.098	0.068467	0.392373	0	0.082565	0.428752	0.089818	0.011437
Novel_012247	XLOC_13099.	transcript	0.13613	0.206631	0.313573	0.115893	0.33632	0.68292	0.015451	0.403321	0.228891
Novel_012248	XLOC_13099.	transcript	0	0	0	0	0	0	0	0	0
Novel_012249	XLOC_13099.	transcript	0	0.68659	0	0	0	0	0	0	0
Novel_012250	XLOC_13099.	transcript	0	0	0.527707	0.140654	0	0	0.3616	0.164433	0.743441
Novel_012251	XLOC_13099.	transcript	0.073263	0.046619	0.049512	0.025975	0.237729	0.006589	0.120523	0.051687	0.133121
Novel_012252	XLOC_13099.	transcript	0.542867	0	0	0.096478	0.422898	0.185759	0.305441	0.152211	0
Novel_012253	XLOC_13102.	transcript	0.639612	0.077773	0.321786	0	0.440231	0.215191	0.716916	0.436099	0
Novel_012254	XLOC_13102.	transcript	0	0	0	0.264461	0	0.005679	0.003814	0	0.307544
Novel_012255	XLOC_13102.	transcript	0.122407	0	0.164285	0.0739	0.153644	0.079275	0.286197	0.140725	0.100274
Novel_012256	XLOC_13102.	transcript	0.183147	0.033844	0.310782	0.278253	0.247914	0.227655	0.582294	0.294133	0.190648
Novel_012257	XLOC_13112.	transcript	0.28667	0.735015	0.313451	0.412871	0.354776	0.400787	0.379365	0.411291	0.353017
Novel_012258	XLOC_13112.	transcript	0.208094	0.223934	0.153716	0.205753	0.158841	0.127292	0.139936	0.136905	0.147722
Novel_012259	XLOC_13112.	transcript	0.202413	0.322625	0.093815	0.199127	0.187186	0.18974	0.361255	0.184567	0.158272
Novel_012260	XLOC_13113.	transcript	0	0	0	0.126416	0.01576	0	0	0	0
Novel_012261	XLOC_13113.	transcript	0.5534	0.195023	0.365803	0.255477	0.359609	0.473886	0.4636	0.324488	0.276412
Novel_012262	XLOC_13113.	transcript	0.128384	0.053093	0.151307	0.230768	0.180896	0.303798	0.213529	0.216294	0.251685

Novel_012263	XLOC_13119.	transcript	0.383425	0.200856	0.318669	0.22575	0.30194	0.327559	0.446261	0.295756	0.260767
Novel_012264	XLOC_13121.	transcript	0.020402	0.020486	0	0.095791	0.065214	0.091954	0.016437	0	0
Novel_012265	XLOC_13121.	transcript	0	0	0	0	0	0	0	0.053238	0
Novel_012266	XLOC_13121.	transcript	0.495042	0.385775	0.468159	0.300356	0.481136	0.461013	0.329124	0.46906	0.232725
Novel_012267	XLOC_13123.	transcript	0	0	0	0	0.237209	0	0	0	0
Novel_012268	XLOC_13123.	transcript	0	0.054499	0	0.003911	0	0	0	0	0
Novel_012269	XLOC_13123.	transcript	0	0.127236	0.030536	0.017167	0.20222	0.258911	0	0.000195	0.223161
Novel_012270	XLOC_13123.	transcript	0.308246	0.170179	0.199546	0.105293	0	0	0.437973	0.404891	0.350754
Novel_012271	XLOC_13123.	transcript	0.258428	0.147642	0.015986	0.253758	0.027128	0.048472	0	0	0
Novel_012272	XLOC_13128.	transcript	1.132302	0.813934	0.823759	0.599337	0.660637	0.814033	0.893615	0.688123	0.469549
Novel_012273	XLOC_13128.	transcript	0.39016	0.468589	0.345346	0.321111	0.41547	0.519546	0.514965	0.408916	0.470203
Novel_012274	XLOC_13129.	transcript	0.32035	0.433197	0.261919	0.306825	0.1701	0.221107	0.314674	0.264636	0.237372
Novel_012275	XLOC_13130.	transcript	0.337888	0.389753	0.829711	1.554341	0.786521	0.981626	0.489341	1.043314	0.343491
Novel_012276	XLOC_13138.	transcript	0	0	0	0.02736	0	0.033865	0.024578	0.930024	0
Novel_012277	XLOC_13138.	transcript	0	0	0	0	0	0	0	0	0
Novel_012278	XLOC_13138.	transcript	0	0	0.014268	0.006198	0	0	0.012772	0	0.400482
Novel_012279	XLOC_13138.	transcript	0	0.084754	0.111591	0.137538	0.304875	0.206237	0.36174	0.113358	0.692551
Novel_012280	XLOC_13138.	transcript	0.019679	0.00647	0.184942	0	0	0	0.01843	0	0.067443
Novel_012281	XLOC_13138.	transcript	0	0	0	0.054418	0.022433	0.096768	0	0.142074	0.005435
Novel_012282	XLOC_13142.	transcript	0.079211	0	0.094769	0.107215	0.060762	0.142399	0.02962	0.067121	0.232183
Novel_012283	XLOC_13157.	transcript	0.953563	1.359549	1.254401	1.252445	0.953914	0.883049	1.306498	1.725885	1.143283
Novel_012284	XLOC_13158.	transcript	0.058939	0.568814	0.067954	0	0	0.096373	0.132978	0.02334	0
Novel_012285	XLOC_13158.	transcript	0	0	0.01679	0.00077	0.307773	0.02956	0.012824	0.152836	0
Novel_012286	XLOC_13158.	transcript	0.215375	0.480671	0	0	0	0	0.667315	0.204222	0
Novel_012287	XLOC_13158.	transcript	0.379021	0.134733	0.285188	0.51154	0.068012	0.283534	0	0.269227	0.419988
Novel_012288	XLOC_13159.	transcript	0.275925	0.005989	0.121716	0	0.117338	0.049461	0.084707	0.270823	0.062737
Novel_012289	XLOC_13160.	transcript	0	0	0.271968	0	0	0	0	0.162909	0
Novel_012290	XLOC_13160.	transcript	0.141119	0.084656	0.133359	0.298757	0.163988	0	0.155044	0.108487	0.522156
Novel_012291	XLOC_13160.	transcript	0.079674	0	0	0	0	0	0.156054	0.243598	0
Novel_012292	XLOC_13160.	transcript	0	0	0	0	0	0	0	0	0
Novel_012293	XLOC_13174.	transcript	0.320847	1.641877	0.25585	0.143552	0.201104	0.30002	0.405483	0.231457	0.199623
Novel_012294	XLOC_13175.	transcript	0	0	0	0	0	0	0	0	0
Novel_012295	XLOC_13175.	transcript	0.120738	0	0.114596	0.041369	0.084595	0.011911	0.340081	0.11977	0.023438
Novel_012296	XLOC_13180.	transcript	0.020492	0.006479	0.077664	0	0.018979	0.044777	0.314081	0.013301	0.213428
Novel_012297	XLOC_13180.	transcript	0	0	0	0.159801	0	0	0.188654	0	0.084437
Novel_012298	XLOC_13182.	transcript	0	0.005134	0	0	0.044876	0	0.00355	0	0
Novel_012299	XLOC_13182.	transcript	0	0	0	0	0	0	0	0	0
Novel_012300	XLOC_13183.	transcript	0.40634	0.412622	0.599801	0.26349	0.687602	0.431422	0.916558	0.502961	0.530111

Novel_012301	XLOC_13183.	transcript	0	0	0	0	0	0	0	0	0.003024
Novel_012302	XLOC_13183.	transcript	0.001334	0.246756	0.133421	0.095849	0	0.015666	0.007902	0.11529	0.012018
Novel_012303	XLOC_13183.	transcript	0.289485	0	0	0	0.109182	0.077054	0.214733	0.09753	0.1255
Novel_012304	XLOC_13183.	transcript	0	0	0	0	0	0	0	0	0
Novel_012305	XLOC_13183.	transcript	0.176527	0	0.203721	0.110066	0	0.224966	0	0	0.199443
Novel_012306	XLOC_13183.	transcript	0.080958	0.371081	0.011748	0.000137	0.219941	2.70E-05	0.692408	0.198736	0.273495
Novel_012307	XLOC_13183.	transcript	0.090813	0.145586	0	5.60E-05	0.197259	4.60E-05	0.137435	0.020731	0
Novel_012308	XLOC_13183.	transcript	0	0	0.112288	0.00994	0	0	0.104185	0	0.056683
Novel_012309	XLOC_13183.	transcript	0	0	0	0.178826	0	0.108881	0	0	0
Novel_012310	XLOC_13183.	transcript	0	0	0.171812	0.131478	0.220038	0.085726	0	0.284066	0
Novel_012311	XLOC_13183.	transcript	0.052533	0.150856	0.139128	0.118524	0.323145	0.206827	0.008877	0.214455	0.017063
Novel_012312	XLOC_13183.	transcript	0	0	0	0.103426	0.199028	0.149322	0	0.186686	0
Novel_012313	XLOC_13183.	transcript	0.020019	0.040154	0.053073	0.039296	0.01928	0.046816	0.215019	0.047538	0.134455
Novel_012314	XLOC_13183.	transcript	0.27615	0.15186	0.286676	0.239022	0.241841	0.145301	0.250006	0.184933	0
Novel_012315	XLOC_13184.	transcript	0.111134	0.056666	0.26766	0.07387	0.112146	0.07436	0.081162	0.096561	0
Novel_012316	XLOC_13184.	transcript	0.263831	1.965465	0.285194	0.278893	0.177819	0.462747	0.508501	0.283169	1.276429
Novel_012317	XLOC_13185.	transcript	1.045326	0.873624	2.030928	2.081314	1.718696	1.814264	2.202294	1.65222	1.698301
Novel_012318	XLOC_13185.	transcript	0	0	0.42631	0.439951	0	0	0.431844	0.341486	0
Novel_012319	XLOC_13185.	transcript	0.45072	0.308689	0.441494	0.34637	0.412246	0.374559	0.589527	0.452807	0.519998
Novel_012320	XLOC_13187.	transcript	0	0	0	0	0	0	1.194317	0	1.260086
Novel_012321	XLOC_13187.	transcript	0	0	0	0	0	0	0	0	0
Novel_012322	XLOC_13187.	transcript	0	0.648748	0	0	0	0	0	0	0
Novel_012323	XLOC_13187.	transcript	0.915963	0.876523	1.096521	1.34761	1.57888	0.907046	0.862013	1.84241	0.771711
Novel_012324	XLOC_13188.	transcript	0.933858	0.532541	0.910042	0.569224	0.604586	0.64437	1.094055	0.565894	0.381711
Novel_012325	XLOC_13189.	transcript	0	0	0	0	0	0	0.475529	0.177588	0
Novel_012326	XLOC_13189.	transcript	0	0	0.410935	0	0	0	0	0	0
Novel_012327	XLOC_13189.	transcript	0.432676	0.28597	0.250915	0.491737	0.698703	0.567961	0.629984	0.394835	0.343816
Novel_012328	XLOC_13189.	transcript	0.343675	0.211192	0.53624	1.353126	0.334741	0.732766	0	0.779946	0.473584
Novel_012329	XLOC_13190.	transcript	0	0.063614	0.34873	0	0.332363	0	0	0.112507	0
Novel_012330	XLOC_13190.	transcript	0.269617	0.159004	0.304264	0.014585	0.062816	0.102733	0.263262	0.175596	0.066097
Novel_012331	XLOC_13190.	transcript	0	0.203977	0	0.244116	0	0.209249	0	0.132114	0.150241
Novel_012332	XLOC_13190.	transcript	0	0.049582	0	0.061825	0	0.137312	0	0.07465	0.22524
Novel_012333	XLOC_13191.	transcript	10.85504	8.31827	8.7061	12.67781	11.44008	15.53062	9.010136	11.40126	12.1424
Novel_012334	XLOC_13191.	transcript	0.453488	0	0	0.186021	0.251946	0	0.319734	0.364901	0.215712
Novel_012335	XLOC_13191.	transcript	0	0	0.157468	0.130054	0	0	0.041133	0	0.155259
Novel_012336	XLOC_13191.	transcript	0.269712	0.087803	0	0.173713	0.122288	0.274247	0.117816	0.075459	0.107509
Novel_012337	XLOC_13191.	transcript	0	0.519527	0.002122	0	0.144352	0.157405	0.110707	0.087766	0
Novel_012338	XLOC_13191.	transcript	0.011598	0	0.368492	6.20E-05	0.163468	0.009409	0.132751	0.087619	0.003202

Novel_012339	XLOC_13192.	transcript	0.175772	0.215631	0.341344	0.169945	0.361919	0.199455	0.453067	0.320692	0.225344
Novel_012340	XLOC_13192.	transcript	0.743016	0.417305	0.777097	0.531243	0.615413	0.711252	0.694682	0.816004	0.856542
Novel_012341	XLOC_13193.	transcript	0.089723	0	0	0.153	0.096677	0	0.221909	0.111908	0
Novel_012342	XLOC_13193.	transcript	0	0	0	0	0	0	0.004348	0.075749	0
Novel_012343	XLOC_13193.	transcript	0.084464	0.043496	0.084344	0	0	0.094191	0.168627	0.126328	0.020366
Novel_012344	XLOC_13193.	transcript	0.087215	0.314915	0	0.047807	0.141644	0.062372	0	0.081787	0.09512
Novel_012345	XLOC_13194.	transcript	0.104936	0.06812	0.244336	0.17759	0.147085	0.299453	0	0.175126	0.008281
Novel_012346	XLOC_13194.	transcript	0	0	0.003288	0	0	0	0	0.094286	0
Novel_012347	XLOC_13194.	transcript	0.309924	0	0.072586	0.054657	0.156242	0.133918	0.362548	0.118385	0.253235
Novel_012348	XLOC_13195.	transcript	0	0	0	0	0	0	0	0	0
Novel_012349	XLOC_13195.	transcript	0	0	0	0	0	0	0	0	0
Novel_012350	XLOC_13195.	transcript	0.315282	0	0	0	0	0	0.588614	0	0
Novel_012351	XLOC_13195.	transcript	0.685705	0.563114	0.670664	0.345852	0.622296	0.561638	0.737965	1.209061	0.411497
Novel_012352	XLOC_13195.	transcript	0	0	0	0	0	0	0	0	0
Novel_012353	XLOC_13195.	transcript	1.068761	1.491419	1.038748	1.26264	1.034654	0.925046	0.564416	0.589866	1.139519
Novel_012354	XLOC_13199.	transcript	0.082016	0	0.021701	0.297069	0.209198	0.103557	0.085611	0.346067	0.055013
Novel_012355	XLOC_13202.	transcript	0.530523	0.066156	0.326484	0.361045	0.355673	0.428966	0.62448	0.450865	0.360102
Novel_012356	XLOC_13202.	transcript	0.150341	0.258816	0.158037	0.272978	0.351278	0.201914	0.408636	0.328841	0.166823
Novel_012357	XLOC_13202.	transcript	0	0	0	0	0	0	0	0	0
Novel_012358	XLOC_13202.	transcript	0	0.053129	0	0	0.116901	0	0	0.024602	0
Novel_012359	XLOC_13203.	transcript	0	0.148541	0	0	0	0	0	0	0
Novel_012360	XLOC_13203.	transcript	0	0.227959	0.266636	0	0	0.026285	0	0	0
Novel_012361	XLOC_13203.	transcript	0	0.206859	0.19581	0	0	0	0	0	0
Novel_012362	XLOC_13204.	transcript	0.048913	0	0.002493	0	0.196911	0	0	0	0
Novel_012363	XLOC_13204.	transcript	0.177237	0	0.113824	0	0	0	0	0.116899	0
Novel_012364	XLOC_13204.	transcript	0	0	0.101013	0	0	0	0	0	0
Novel_012365	XLOC_13204.	transcript	0.894762	0	0.438589	0.294683	0.150475	0.101931	0	0.120429	0
Novel_012366	XLOC_13204.	transcript	0	0.442435	0	0.590069	0.611405	0.746086	1.880358	0.148114	0.880161
Novel_012367	XLOC_13206.	transcript	0.078064	0.084638	0	0.003538	0.263187	0.123168	0.071021	0.685586	0.152113
Novel_012368	XLOC_13206.	transcript	0.838892	0.111261	0.589323	0.374837	1.152957	0.062902	0.97254	2.702545	0.095181
Novel_012369	XLOC_13206.	transcript	0.495783	0.065731	0.60269	0.419045	0	0.320466	0.304804	0.55468	0.479188
Novel_012370	XLOC_13206.	transcript	2.075681	0.074927	1.176352	1.264439	1.769241	0.500943	1.470001	2.03384	0.451489
Novel_012371	XLOC_13206.	transcript	0.156278	0.068471	0.146332	0.399779	0.370888	0	0.182776	0.079284	0.213834
Novel_012372	XLOC_13206.	transcript	0	0	0	0	0.341056	0	0	0.111626	0.337187
Novel_012373	XLOC_13211.	transcript	0	0	0	0.068619	0	0.26138	0.260792	0	0.101948
Novel_012374	XLOC_13211.	transcript	0.31283	0	0.902975	0.777379	0.173696	0	0.838793	0.122051	0.202487
Novel_012375	XLOC_13217.	transcript	0.024931	0.273903	0.026358	0.020895	0.02339	0.048128	0.046615	0.071371	0
Novel_012376	XLOC_13219.	transcript	0.260876	0.845927	0.099294	0.053507	0.238858	0.058253	0.197634	0.147265	0.136148

Novel_012377	XLOC_13219.	transcript	0.208369	0.267375	0	0.040109	0.076969	0.028369	0.192943	0.011925	0.301554
Novel_012378	XLOC_13219.	transcript	0.146846	0.152202	0	0.03545	0	0	0.013273	0	0.236549
Novel_012379	XLOC_13221.	transcript	0.473538	1.139983	0.414405	0.261544	0.223786	0.081168	0.365761	0.205306	1.240953
Novel_012380	XLOC_13221.	transcript	0	2.052976	0	0	0	0	0	0	0
Novel_012381	XLOC_13222.	transcript	0.87528	0.654213	0.872927	0.953918	0.870042	0.829505	0.961068	0.995092	0.4919
Novel_012382	XLOC_13224.	transcript	0.119143	0	0.013688	0.071271	0	0	0	0.072748	0
Novel_012383	XLOC_13224.	transcript	0.111217	0	0.401117	0.193251	0	0	0.256263	0.155756	0
Novel_012384	XLOC_13224.	transcript	0.140278	0	0	0	0	0	0.054108	0	0
Novel_012385	XLOC_13224.	transcript	0.078622	0	0	0.271373	0	0	0.067834	0.272453	0
Novel_012386	XLOC_13226.	transcript	7.396698	8.177923	6.786146	6.766095	6.207357	6.157763	5.94504	6.157942	6.812861
Novel_012387	XLOC_13228.	transcript	0.139253	0.031585	0.791235	0.393268	0.638537	0.954899	0.422313	0.548605	0.624406
Novel_012388	XLOC_13229.	transcript	0.023358	0.623349	0	0	0.071192	0	0.106519	0.109277	0
Novel_012389	XLOC_13230.	transcript	0.05101	0.348967	0.005054	8.40E-05	0.044517	0.0015	0.022517	0.027774	0.018795
Novel_012390	XLOC_13230.	transcript	0	0	0.012054	0.011707	0	0.000455	0.144199	0	0
Novel_012391	XLOC_13230.	transcript	0.266579	0.190057	0.451592	0.579558	0.508762	0.418739	0.599845	0.60124	0.394003
Novel_012392	XLOC_13234.	transcript	0.539622	0	0.090394	0.056743	0.285979	0	0.378069	0.213253	0.496635
Novel_012393	XLOC_13235.	transcript	3.945256	7.151315	12.39314	12.86318	12.60364	14.04789	11.05063	8.131564	13.84943
Novel_012394	XLOC_13236.	transcript	0.102714	0.311047	0.355576	0.276869	0.34534	0.304215	0.273575	0.27114	0.279025
Novel_012395	XLOC_13236.	transcript	0.541877	0.584146	0.580346	0.130497	0.011173	0.008249	0.606271	0.382018	0.775684
Novel_012396	XLOC_13236.	transcript	0	0	0	0	0	0	0.567496	0	0
Novel_012397	XLOC_13236.	transcript	1.040421	0.655623	1.222754	1.032412	1.42613	1.129854	2.339493	1.178562	2.147294
Novel_012398	XLOC_13236.	transcript	0	0	0	0.373958	0	0	0	0	0
Novel_012399	XLOC_13236.	transcript	0.34432	0.354961	0.328639	0.243919	0.343356	0.398084	0.44364	0.358534	0.273216
Novel_012400	XLOC_13237.	transcript	0	0	0	0.014616	0.017836	0	0.024102	0	0.067929
Novel_012401	XLOC_13239.	transcript	0	0	0	0	0	0	0	0.020684	0
Novel_012402	XLOC_13240.	transcript	0	0	0.20084	0.141167	0.127961	0.251456	0.032803	0.155959	0.11227
Novel_012403	XLOC_13240.	transcript	0.069451	0.014746	0.015487	0.045085	0.461081	0	0.101464	0.202891	0
Novel_012404	XLOC_13248.	transcript	0.31285	1.500243	0.366707	0.339177	0.298045	0.292071	0.466501	0.340732	1.257684
Novel_012405	XLOC_13249.	transcript	0.020835	0.210235	9.00E-05	0	0.076311	0.018464	0.017475	0.005404	0.051107
Novel_012406	XLOC_13249.	transcript	0.052221	0.248583	0.038671	0.07537	0.016879	0.029019	0.019694	0.035671	0.04966
Novel_012407	XLOC_13252.	transcript	0.089765	0.051035	0.231222	0.120072	0.274496	0.178919	0.325089	0.145352	0.048781
Novel_012408	XLOC_13252.	transcript	0.157949	0.22292	0.297075	0.215572	0.801574	0.215287	0.228242	0.09004	1.445928
Novel_012409	XLOC_13252.	transcript	0	0	0.005344	0	0	0	0.00378	0	0
Novel_012410	XLOC_13252.	transcript	0	0	0	0	0.138549	0	0	0.070331	0
Novel_012411	XLOC_13252.	transcript	0.135237	0.077384	0.230005	0.875054	0.141121	1.141794	1.289518	0.225736	0.441591
Novel_012412	XLOC_13252.	transcript	0.038659	0.067456	0.017216	0.141921	0.216013	0	0	0.072602	0
Novel_012413	XLOC_13252.	transcript	0.175164	0.540387	0.260618	0.77124	0.12703	0	0	0.686439	0
Novel_012414	XLOC_13253.	transcript	0.349652	0.53755	0.271336	0.118209	0.244691	0.194269	0.647358	0.032465	0.608552

Novel_012415	XLOC_13254.	transcript	0.32685	0.348651	0	0.30522	0.504999	0.543851	0.692234	0.488501	0.30275
Novel_012416	XLOC_13254.	transcript	0	0	0	0	0	0	0.011384	0.049704	0
Novel_012417	XLOC_13255.	transcript	0.565733	0.588252	0.905332	0.696266	0.57158	0.86649	0.959219	0.702088	0.387236
Novel_012418	XLOC_13255.	transcript	0.1828	0.177731	0	0.164764	0	0	0	0.079959	0.262911
Novel_012419	XLOC_13255.	transcript	0	0	0	0	0	0	0	0	0
Novel_012420	XLOC_13255.	transcript	0.097211	0	0.028919	0	0	0	0.597928	0	0
Novel_012421	XLOC_13255.	transcript	0	0.26025	0.016391	0	1.826529	1.716015	2.60607	0	3.263454
Novel_012422	XLOC_13255.	transcript	0.132457	0.199552	0.090495	0.317343	0.041041	0.094575	0.21128	0.05658	0
Novel_012423	XLOC_13255.	transcript	0.766378	2.711421	1.072754	0.481899	1.127576	1.459539	1.750744	1.059075	0.966458
Novel_012424	XLOC_13255.	transcript	0.055578	1.393267	0	0.579061	0.047231	0	0	0.300039	0.496811
Novel_012425	XLOC_13255.	transcript	0	0.046244	0.008193	0	0	0.025789	0	0	0
Novel_012426	XLOC_13255.	transcript	0	0	0	0	0.017412	0	0	0	0
Novel_012427	XLOC_13258.	transcript	0.518943	0.092211	1.056581	1.252437	1.356873	1.300591	0.735377	1.297022	0.721253
Novel_012428	XLOC_13258.	transcript	0.192455	0	0.243429	0.08261	0.135223	0.196649	0.352506	0.233899	0.104534
Novel_012429	XLOC_13258.	transcript	0.000634	0.227911	0	0	0.220898	0.222133	0.317616	0.181419	0.129042
Novel_012430	XLOC_13258.	transcript	0.277672	0.042372	0.477823	0.850882	0.276279	0.298479	0.419511	0.379394	0.233423
Novel_012431	XLOC_13258.	transcript	0.230976	0	0	0	0	0	0	0	0.11051
Novel_012432	XLOC_13258.	transcript	0	0	0	0	0	0	0.086967	0	0.018601
Novel_012433	XLOC_13258.	transcript	0	0	0	0	0	0	0	0	0.065539
Novel_012434	XLOC_13258.	transcript	0.120583	0.006963	0.026983	0.009662	0.318793	0.049449	0.028974	0.055569	0.054528
Novel_012435	XLOC_13258.	transcript	0	0	0	0	0	0	1.00E-06	0.068291	0
Novel_012436	XLOC_13264.	transcript	0.08588	0.060864	0.174847	0.195645	0	0.081745	0.296747	0.047555	0.062576
Novel_012437	XLOC_13264.	transcript	0.201445	0.132153	0.145332	0.32836	0.173233	0.218339	0.407221	0.111525	0
Novel_012438	XLOC_13264.	transcript	0.188244	0.159525	0.11264	0	0.267292	0.050869	0.208483	0.283477	0.244924
Novel_012439	XLOC_13267.	transcript	0.313902	0.301933	0.180523	0.182783	0.335637	0.327654	0.45785	0.213862	0.300186
Novel_012440	XLOC_13267.	transcript	0	0	0	0	0	0	0.010972	0	0
Novel_012441	XLOC_13268.	transcript	0.510985	0.358142	0.055641	0.670609	0.461141	1.528203	0	0.313819	0.309033
Novel_012442	XLOC_13268.	transcript	0.565668	0.84804	1.836953	1.799999	1.605036	1.573922	1.560977	0.721565	0.828773
Novel_012443	XLOC_13268.	transcript	0.223695	0.619089	1.077982	0.69531	0.491535	0.316805	0.325969	0.196009	0.485001
Novel_012444	XLOC_13268.	transcript	0.376225	0.190953	0.0261	0.490709	0.067476	0.345723	0.386133	0.401357	0.112588
Novel_012445	XLOC_13268.	transcript	0	0	0	0	0.780659	0.270081	0.703439	1.426908	0
Novel_012446	XLOC_13270.	transcript	0.795745	0	0	0	0.495428	0	0	0.015412	0
Novel_012447	XLOC_13270.	transcript	0.372016	0.485104	1.070046	1.863341	1.450156	1.094823	1.03095	1.487547	0.992925
Novel_012448	XLOC_13270.	transcript	0	0	0.342981	0	0	0	1.028158	0.565406	0.025175
Novel_012449	XLOC_13273.	transcript	1.426963	0.029338	0.518654	0.242285	1.04334	0.386697	0.169825	0.186326	0.479136
Novel_012450	XLOC_13273.	transcript	0	0.634704	0.328155	0.100058	0	0	0.00705	0	0.062619
Novel_012451	XLOC_13273.	transcript	0.180785	0.004373	0.188819	0	0	0	0	0.147694	0
Novel_012452	XLOC_13273.	transcript	0.00408	0	0.442442	0.111944	0	0	0.003192	0	0.001209

Novel_012453	XLOC_13273.	transcript	0.39135	0.366469	0.075545	0.407711	0.301887	0.356176	0.485434	0.446181	0.518207
Novel_012454	XLOC_13275.	transcript	0	0	0	0.154586	0	0	0.381046	0	0
Novel_012455	XLOC_13275.	transcript	0.217384	0	0	0.1353	0	0.092851	0.344391	0	0
Novel_012456	XLOC_13275.	transcript	0.676465	0.748855	0.571072	0.133135	0.38846	0.53476	0	0.503179	0.431504
Novel_012457	XLOC_13275.	transcript	0	0.209301	0	0.116156	0.493883	0	0	0.416224	0.003713
Novel_012458	XLOC_13275.	transcript	0.033992	0.175755	0.033968	0.065213	0.087041	0.123841	0.245846	0.124489	0.150012
Novel_012459	XLOC_13275.	transcript	0.177637	0.291731	0.169142	0.094453	0.316561	0.180238	0.432776	0.133301	0
Novel_012460	XLOC_13299.	transcript	0.11135	0.109457	0.25477	0.380836	0.1148	0.46623	0.087806	0.236799	0.248456
Novel_012461	XLOC_13301.	transcript	0	0.097082	0	0	0	0	0.069975	0	0.033578
Novel_012462	XLOC_13301.	transcript	0.206552	0.279027	0.295914	0.244044	0.226942	0.294167	0.282282	0.233499	0.164265
Novel_012463	XLOC_13302.	transcript	0.252334	0.268909	0.459202	0.342293	0.300463	0.156604	0.566582	0.500214	0.434958
Novel_012464	XLOC_13306.	transcript	0.830408	4.494554	0.547263	0.415273	0.62674	0.432055	1.242382	0.836581	2.365086
Novel_012465	XLOC_13307.	transcript	0	0	0	0	0	0.421326	0	0	0
Novel_012466	XLOC_13308.	transcript	0.164197	0.219227	0.159791	0.21694	0.244884	0.306487	0.282242	0.266435	0.231207
Novel_012467	XLOC_13309.	transcript	0.012604	0	0.311868	0.039545	0.193241	0	0.211252	0.021845	0.021478
Novel_012468	XLOC_13309.	transcript	2.547523	1.779974	2.65371	2.465423	2.712283	2.30347	3.047045	2.650669	3.502281
Novel_012469	XLOC_13310.	transcript	0	0	0	0	0.040026	0.058305	0	0.1871	0.101321
Novel_012470	XLOC_13311.	transcript	0.757747	0.66052	1.12006	1.064861	1.159113	0.840946	1.054686	1.303594	1.038348
Novel_012471	XLOC_13311.	transcript	0.97152	0.690856	0.851099	0.733997	0.556267	0.866625	1.04776	0.975485	0.669402
Novel_012472	XLOC_13311.	transcript	0	0	0	0	0	0	0	0	0
Novel_012473	XLOC_13315.	transcript	0	0	0	0.032199	0.136849	0.296209	0.013924	0	0.040355
Novel_012474	XLOC_13315.	transcript	1.110406	0.317526	0.312924	0.320028	0.509416	0.726139	0.035919	0.995985	0.243182
Novel_012475	XLOC_13315.	transcript	0	0.049088	0.078951	0	0	0	0.279608	0	0
Novel_012476	XLOC_13315.	transcript	0.071426	0.081816	0.193953	0.025108	0.179795	0	0.009101	0.176525	0.032667
Novel_012477	XLOC_13315.	transcript	0.040983	0.106912	0.060955	0	0.017341	0	0	0	0.000324
Novel_012478	XLOC_13315.	transcript	0	0.00156	0.001578	0.067227	0.174124	0	0.578597	0.012717	0.406783
Novel_012479	XLOC_13315.	transcript	0	0	0	0.042216	0.148838	0	0.203231	0	0.196648
Novel_012480	XLOC_13315.	transcript	0	0	0	0	0	0	0	0	0
Novel_012481	XLOC_13315.	transcript	0	0	0	0.421013	0	0	0	0.001853	0
Novel_012482	XLOC_13315.	transcript	0.000277	0	0	0	0	0.314574	0.00018	0	0
Novel_012483	XLOC_13316.	transcript	0.423219	0.388789	0.524212	0.503039	0.478667	0.481564	0.652142	0.658316	0.301055
Novel_012484	XLOC_13316.	transcript	0	0	0	0	0	0.0745	0	0.142733	0
Novel_012485	XLOC_13324.	transcript	0	0	0	1.014948	0	0	0	0	0
Novel_012486	XLOC_13324.	transcript	3.832757	0.406414	4.468368	0.00808	4.495151	1.766354	3.052557	7.387682	3.401632
Novel_012487	XLOC_13324.	transcript	0	0	0	0.004849	0	0.007039	0.025096	0.007476	0
Novel_012488	XLOC_13324.	transcript	0.098031	0.423196	0.139798	2.108771	0.217712	1.852946	0.349633	0.106785	0.263926
Novel_012489	XLOC_13328.	transcript	0.113149	0.511206	0.553463	0.465607	0.209975	0.622298	0.248861	0.266383	0.364889
Novel_012490	XLOC_13330.	transcript	0.271274	0.268727	0.462886	0.436415	0.393833	0.336422	0.168103	0.375749	0.281816

Novel_012491	XLOC_13346.	transcript	0	0	0.097276	0.113103	0.160211	0	0	0.263404	0
Novel_012492	XLOC_13347.	transcript	2.05173	0.506946	3.933111	8.507429	3.660727	5.35753	2.791785	4.50879	2.774172
Novel_012493	XLOC_13349.	transcript	0.100741	0.172323	0.149446	0.891141	0.41167	0.056078	0.417385	0.329637	0.057018
Novel_012494	XLOC_13349.	transcript	0.489194	0.054076	0.2709	0.313726	0.403013	0.293533	0.5901	0.478334	0.269005
Novel_012495	XLOC_13351.	transcript	0.034075	0.041079	0.004175	0	0.029512	0	0.118465	0.005205	0
Novel_012496	XLOC_13351.	transcript	0	0	0	0	0	0	0	0.182414	0
Novel_012497	XLOC_13351.	transcript	0.26482	0.230094	0.191258	0.220905	0.222583	0.205408	0.277094	0.205803	0.055566
Novel_012498	XLOC_13351.	transcript	0	0.009037	0.003406	0	0	0.005337	0	0	0
Novel_012499	XLOC_13351.	transcript	0	0.094824	0	0	0	0	0	0	0
Novel_012500	XLOC_13353.	transcript	0	0	0.023828	0.039398	0	0	0.045001	0.024277	0
Novel_012501	XLOC_13357.	transcript	0.329161	0.435204	0.310788	0.314064	0.294763	0.362477	0.410267	0.260057	0.222487
Novel_012502	XLOC_13357.	transcript	0	0	0	0	0	0	0	0	0
Novel_012503	XLOC_13361.	transcript	57.22016	57.93675	116.5257	96.45778	96.08541	126.7522	78.77958	75.34847	112.6746
Novel_012504	XLOC_13362.	transcript	0	0	0	0	0	0	0	0.09054	0
Novel_012505	XLOC_13362.	transcript	0	0.565414	0	0.198952	0.347135	0.070365	0.470393	0.194642	1.322818
Novel_012506	XLOC_13362.	transcript	0	0	0	0.107035	0.03386	0.1847	0	0.105593	0.120686
Novel_012507	XLOC_13362.	transcript	0.852752	0.544093	0.314113	0	0	0	0.537497	0.235445	0
Novel_012508	XLOC_13362.	transcript	0.211703	0.278013	0.359842	0	0	0	0.133433	0.081605	0
Novel_012509	XLOC_13364.	transcript	0.404215	0.456404	0.287961	0.253091	0.188299	0.294119	0.407903	0.370287	0.198384
Novel_012510	XLOC_13366.	transcript	0.131061	1.061938	0.687894	0.687662	0.284434	1.394065	0.586443	0.487012	0.134649
Novel_012511	XLOC_13367.	transcript	0.073298	0.058668	0.073151	0.020489	0.27662	0.116604	0.178109	0.090908	0.014486
Novel_012512	XLOC_13368.	transcript	0	0	0.014239	0	0	0.01625	0.013383	0.015423	0.031745
Novel_012513	XLOC_13369.	transcript	0.40898	0.573311	0.31313	0.20803	0.329382	0.298229	0.551138	0.147409	0.329649
Novel_012514	XLOC_13369.	transcript	0	0.001832	0	0	0.000922	0	0	0	0
Novel_012515	XLOC_13373.	transcript	0.647731	0.581331	0.773914	1.789868	1.248856	0.679819	0.504044	1.594869	0.633458
Novel_012516	XLOC_13373.	transcript	0.181018	0.068205	0.310856	0.148491	0.145857	0.468659	0.756831	0.10429	0.340305
Novel_012517	XLOC_13373.	transcript	0	0	0	0	0	0	0	0	0
Novel_012518	XLOC_13374.	transcript	0.094027	0.143657	0.052334	0.091089	0.062821	0.141808	0.13232	0.163763	0.11073
Novel_012519	XLOC_13374.	transcript	0.080879	0.40717	0	0	0	0.20458	0.101701	0.225063	0
Novel_012520	XLOC_13375.	transcript	0	0.291316	0	0.027932	0.041243	0.072979	0	0.02709	0.023895
Novel_012521	XLOC_13375.	transcript	0.59126	0.527202	0.255167	0.534951	0.624366	0.514621	0.742727	0.647664	0.462123
Novel_012522	XLOC_13375.	transcript	0	0.610702	0	0	0	0	0.008292	0.006883	0
Novel_012523	XLOC_13375.	transcript	0	0	0.830318	0	0	0	0	0	0
Novel_012524	XLOC_13377.	transcript	0.167303	0	0	0	0	0	0	0	0.102307
Novel_012525	XLOC_13377.	transcript	0.357154	0.634497	0.407121	0.293736	0.543994	0	0.730572	0.48438	0.282098
Novel_012526	XLOC_13381.	transcript	0.394315	0.592291	0.243384	0.388143	0.306477	0.26814	0.407957	0.425483	0.27887
Novel_012527	XLOC_13388.	transcript	0	0.59285	0.038402	0.039492	0.028026	0.059612	0.079376	0.021645	0
Novel_012528	XLOC_13389.	transcript	0.155793	0.811496	0.020069	0.017657	0.034482	0.067942	0.345262	0.024148	0.051669

Novel_012529	XLOC_13389.	transcript	0.358631	0.938175	0.300819	0.190302	0.280653	0.179406	0.382122	0.239446	0.316921
Novel_012530	XLOC_13394.	transcript	0.102903	0.031403	0.181502	0.227373	0.294329	0.138461	0.24097	0.193533	0.244991
Novel_012531	XLOC_13394.	transcript	0.312306	0	0	0.1651	0.193477	0.296476	0	0.337367	0.226145
Novel_012532	XLOC_13394.	transcript	0	0.013755	0.317014	0.158767	0.178709	0	0.483685	0	0.009834
Novel_012533	XLOC_13395.	transcript	0.861085	0.233365	1.056464	1.146351	1.118728	0.988407	0.76115	1.29635	0.550111
Novel_012534	XLOC_13395.	transcript	0	0	2.70E-05	0	0	0	6.00E-06	0.001713	0.753968
Novel_012535	XLOC_13395.	transcript	0	0	0	0	0	0	0.810387	0	0
Novel_012536	XLOC_13395.	transcript	0	0	0	0	0	0	0	0	0
Novel_012537	XLOC_13395.	transcript	0.346409	0	0.102362	0.10017	0.112861	0.253528	0.047908	0.206425	0.028269
Novel_012538	XLOC_13397.	transcript	0	0	0	0.147469	0.139098	0	0	0.29091	0.20959
Novel_012539	XLOC_13397.	transcript	0.40511	0.016175	0.179456	0.113741	0.121857	0.111147	0	0.2173	0.241574
Novel_012540	XLOC_13397.	transcript	0.313623	0	0	0	0	0.169879	0	0.074454	0.179926
Novel_012541	XLOC_13397.	transcript	0.219206	0.660349	0.00671	0.059939	0.004672	0	0.139715	0.095274	0.187946
Novel_012542	XLOC_13397.	transcript	0	0.324757	0.00321	0	0	0	0.029703	0	0
Novel_012543	XLOC_13397.	transcript	0	0.005218	0	0.305302	0.198119	0	0	0.091847	0.326193
Novel_012544	XLOC_13397.	transcript	0.047466	0.419431	0.276021	0.007523	3.90E-05	0.177328	0	0.083665	0.361805
Novel_012545	XLOC_13397.	transcript	0.035547	0.024589	0.021272	0	0.038375	0.009242	0.003516	0.007396	0.009083
Novel_012546	XLOC_13411.	transcript	0	0	0.02129	0	0	0	0	0.065914	0
Novel_012547	XLOC_13411.	transcript	0.254775	0.062916	0.275384	0.403613	0.067056	0.378391	0.041373	0.256271	0.277689
Novel_012548	XLOC_13411.	transcript	0.004561	0	0	0	0.519168	0	0.674785	0	0
Novel_012549	XLOC_13411.	transcript	0.222768	0	0	0	0	0	0	0	0.144906
Novel_012550	XLOC_13411.	transcript	0.040158	0.461529	0.704382	0.287998	0.471145	0.660707	0.895532	0.208327	0.437179
Novel_012551	XLOC_13411.	transcript	0	0.138801	0	0	0.230139	0.083756	0	0	0.108366
Novel_012552	XLOC_13411.	transcript	0.000873	0.224696	0	0.000465	0.000384	0	0	0	0
Novel_012553	XLOC_13411.	transcript	0	0.000609	0	0.281571	0	0.001795	0	0.493041	0.018741
Novel_012554	XLOC_13411.	transcript	0	0.000582	0	0.075607	0.249696	0.00446	0	0.000179	0
Novel_012555	XLOC_13412.	transcript	0	0	0.468604	0	0.009414	0	0.167124	0	0
Novel_012556	XLOC_13412.	transcript	0.310257	0.226376	0.132688	0.307792	0.394316	0.171228	0.390081	0.50879	0.288826
Novel_012557	XLOC_13412.	transcript	0	0.198037	0	0.210644	0	0	0.140095	0	0
Novel_012558	XLOC_13412.	transcript	0	0	1.418929	0.482779	0	0	0	0.963224	0.450595
Novel_012559	XLOC_13412.	transcript	1.478239	0.261491	0	1.575584	1.344849	1.276074	2.300242	1.291036	0.993934
Novel_012560	XLOC_13412.	transcript	0.85376	1.468883	0	0.098001	0.299565	0.332623	0.332076	0	0
Novel_012561	XLOC_13414.	transcript	0	0.01032	0.86415	0	0	0.138232	0.124319	0	0
Novel_012562	XLOC_13414.	transcript	1.015447	0	0	0	0.016483	0	0	0	0
Novel_012563	XLOC_13414.	transcript	0	0.015657	0.056742	0.011497	0.111668	0.142263	0.001013	0.005201	0.038805
Novel_012564	XLOC_13414.	transcript	0	0	0	0	0	0	0	0	0
Novel_012565	XLOC_13414.	transcript	0	0	0	0.009955	0	0	0	0	0.014377
Novel_012566	XLOC_13414.	transcript	0.287034	0.254143	0.35686	0.689107	0.637024	0.511809	0.922781	0.983633	0.681613

Novel_012567	XLOC_13414.	transcript	0	0.020485	0	0	0	0	0	0	0
Novel_012568	XLOC_13414.	transcript	0	0	0	0	0	1.876146	0.357508	0	0
Novel_012569	XLOC_13417.	transcript	0.356393	0.171544	0.156853	0.285619	0.200195	0.404199	0.202732	0.205659	0.25363
Novel_012570	XLOC_13417.	transcript	0.158133	0.191967	0.254066	0.156001	0.237737	0.028273	0.165608	0.116571	0.103758
Novel_012571	XLOC_13417.	transcript	0	0	0.140205	0.010161	0	0.020172	0.288291	0	0.166576
Novel_012572	XLOC_13417.	transcript	6.503072	7.592868	6.278585	7.306818	6.418637	4.758311	8.80336	9.170528	6.700078
Novel_012573	XLOC_13417.	transcript	1.525969	3.75163	2.907913	2.981162	2.625121	4.558877	2.024121	2.651593	3.591887
Novel_012574	XLOC_13417.	transcript	0.233676	0	0.283721	0.16668	0.177939	0.346625	0.351468	0	0.424517
Novel_012575	XLOC_13417.	transcript	0.25725	0	0.207678	0.096244	0.058593	0.283639	0.16302	0	0
Novel_012576	XLOC_13417.	transcript	0	0	0	0	0.173839	0	0	0.168584	0
Novel_012577	XLOC_13417.	transcript	0.150636	0.169049	0.058815	0	0.109267	0	0	0.287657	0.122228
Novel_012578	XLOC_13421.	transcript	0.299838	0.129618	0.213626	0.618117	0.46396	0.183084	0.317361	0.319209	0.138842
Novel_012579	XLOC_13429.	transcript	0	0.203062	0	0	0.035396	0.182605	0.309942	0	0.001015
Novel_012580	XLOC_13429.	transcript	0.018983	0.118123	0	0.446478	0.066079	0.539329	0	0.752259	0.033999
Novel_012581	XLOC_13429.	transcript	0	0.34026	0	0	0.078236	0.311111	0.275455	0	0
Novel_012582	XLOC_13429.	transcript	1.299519	0	0	0.361162	1.129991	0	0.003683	0.001666	1.016593
Novel_012583	XLOC_13429.	transcript	0	0	0	0.384498	0	0	0	0	0
Novel_012584	XLOC_13429.	transcript	0	0.118968	0	0	0.036095	0.13658	0.087724	0	0
Novel_012585	XLOC_13429.	transcript	0	0.166191	0	0	0.079833	0.430751	0.304986	0	0
Novel_012586	XLOC_13429.	transcript	0	0.185079	0	0	0.005092	0	0.23877	0	0
Novel_012587	XLOC_13429.	transcript	0.066635	0.860802	1.172354	0.335818	0.056356	0	0.195492	1.182423	0.109022
Novel_012588	XLOC_13432.	transcript	0	1.06513	0	0	0	0	0	0	0
Novel_012589	XLOC_13436.	transcript	0	0	0	0.001298	0	0.002356	0	0	0
Novel_012590	XLOC_13436.	transcript	0	0	0	0	0.012544	0	0	0	0
Novel_012591	XLOC_13436.	transcript	0	0	0	0	0	0	0	0.245915	0
Novel_012592	XLOC_13436.	transcript	0.007755	0	0	0.014692	0.237954	0.193498	0.133749	0.0192	0
Novel_012593	XLOC_13436.	transcript	0	0	0.048025	0	0	0	0.037827	0.000504	0
Novel_012594	XLOC_13436.	transcript	0.176842	0.017865	0.092512	0.115523	0.019636	0.160964	0.353404	0.14705	0.149441
Novel_012595	XLOC_13436.	transcript	0.161692	0	0.058887	0	0.002561	0	0.000109	0	0
Novel_012596	XLOC_13436.	transcript	0	0	0.107918	0	0	0.016337	0.107032	0	0
Novel_012597	XLOC_13438.	transcript	0.408507	0.245225	0.341148	0.353545	0.282744	0.25657	0.297751	0.447085	0.302599
Novel_012598	XLOC_13438.	transcript	0	0	0	0	0	0	0	0	0
Novel_012599	XLOC_13439.	transcript	0.789635	1.457241	0.633349	0.432146	0.809035	0.527866	0.789409	0.399074	0.448194
Novel_012600	XLOC_13441.	transcript	0	0.002723	0.005559	0	0	0	0	0	0.009756
Novel_012601	XLOC_13441.	transcript	0.050922	0.138808	0.076362	0.614601	0.320974	0.051786	0.311802	0.341642	0.05194
Novel_012602	XLOC_13444.	transcript	0.034141	0.227537	0.014943	0.05081	0.062124	0.048571	0.075714	0.080024	0.097928
Novel_012603	XLOC_13444.	transcript	0	0	0.089412	0	0	0	0	0	0
Novel_012604	XLOC_13454.	transcript	0.397381	0.727935	0.465215	0.510529	0.649195	0.4276	0.250891	0.253809	0.302298

Novel_012605	XLOC_13454.	transcript	0.120225	1.927244	0.15875	0.204199	0.111849	0.123041	0.688396	0.605675	0.432621
Novel_012606	XLOC_13454.	transcript	0.435725	0.352748	0.662615	0.575253	0.722274	0.353848	0.357835	0.647962	0.562359
Novel_012607	XLOC_13454.	transcript	0	0	0	0.075636	0	0.141975	0.086159	0	0
Novel_012608	XLOC_13455.	transcript	0.162068	0	0.051222	0	0.2217	0.000233	0.014316	0	0
Novel_012609	XLOC_13455.	transcript	0	0	0	0	0.13621	0	0.336585	0	0
Novel_012610	XLOC_13455.	transcript	0.303954	0.182494	0	0.423939	0.092559	0.390154	0	0.274736	0
Novel_012611	XLOC_13455.	transcript	0.326089	0.230364	0.396625	0.237353	0.187501	0.254785	0.294302	0.728696	0.508107
Novel_012612	XLOC_13455.	transcript	0.020044	0	0.005396	0.020096	0.024058	0.017477	0.014922	0.016194	0.007694
Novel_012613	XLOC_13456.	transcript	0.863986	1.954275	2.470821	2.808295	2.132885	1.394572	2.424689	2.070722	2.692842
Novel_012614	XLOC_13459.	transcript	0.206683	0.725729	0.245802	0.348212	0.240691	0.183584	0.402485	0.266608	0.440756
Novel_012615	XLOC_13460.	transcript	0.362389	0.543318	0.301317	0.495007	0.381654	0.396778	0.438529	0.391677	0.397482
Novel_012616	XLOC_13460.	transcript	0.091221	0.282752	0.160474	0.319999	0.322226	0.141485	0.260879	0.212779	0.414555
Novel_012617	XLOC_13460.	transcript	0.548068	0.41454	0.45669	0.497267	0.811847	0.73406	0.371717	0.759744	0.564017
Novel_012618	XLOC_13460.	transcript	0.065747	0.747483	0.13016	0.09916	0.044937	0.016211	0.454522	0.040514	0
Novel_012619	XLOC_13463.	transcript	0.579279	0.079192	0.043064	0.714555	0.680094	0.949648	1.329895	0.646128	0.291068
Novel_012620	XLOC_13463.	transcript	0.004075	0.00798	0.774386	0.003416	0.184415	0.27071	0.001924	0.090466	0.202137
Novel_012621	XLOC_13463.	transcript	2.999049	3.562987	2.671314	3.879107	2.674217	3.46742	3.890363	3.598708	2.641038
Novel_012622	XLOC_13464.	transcript	0.104655	0.286163	0.232013	0.147159	0.221498	0.182742	0.18106	0.240797	0.097059
Novel_012623	XLOC_13464.	transcript	0.558491	0.572955	0.397806	0	0.483845	0.43343	0.000439	0.312119	0.433409
Novel_012624	XLOC_13464.	transcript	0	0.649495	0.020799	0.419198	0.038515	0.020515	0.771722	0.291142	0.039431
Novel_012625	XLOC_13464.	transcript	0.828977	1.286401	1.665813	2.373468	1.55444	2.825982	1.087856	1.021461	2.336949
Novel_012626	XLOC_13464.	transcript	0.112467	0.491981	0	0.134085	0.097966	0	0	0.102934	0
Novel_012627	XLOC_13464.	transcript	0	0.186033	0.012579	0	0.018476	0	0.429868	0	0
Novel_012628	XLOC_13464.	transcript	0	0.0096	0	0.21681	0.036421	0.037652	0.052678	0.156432	0.009484
Novel_012629	XLOC_13464.	transcript	0.327632	0	0	0	0	0	0	0	0
Novel_012630	XLOC_13467.	transcript	0.22979	1.045946	0.199333	0.142194	0.21789	0.173723	0.406647	0.218992	0.628895
Novel_012631	XLOC_13467.	transcript	0	0.214181	0	0	0	0	0	0	0
Novel_012632	XLOC_13468.	transcript	0	0	0.234297	0.137093	0.29096	0.040082	0.616698	0.183387	0.112021
Novel_012633	XLOC_13468.	transcript	0.071502	0	0.019021	0.013048	0.011552	0	0.308052	0.122938	0
Novel_012634	XLOC_13468.	transcript	0.257118	0.167611	0.356507	0.216898	0.212015	0.279327	0.326783	0.230308	0.209176
Novel_012635	XLOC_13468.	transcript	0.170553	0.197856	0.051046	0.367517	0.093051	0.152785	0.018686	0.179156	0.174224
Novel_012636	XLOC_13469.	transcript	1.795113	1.519877	1.798777	1.847367	1.529503	1.785645	2.047681	2.178303	1.610793
Novel_012637	XLOC_13480.	transcript	0.176253	0.006864	0.080672	0.041645	0.080058	0.154374	0.221873	0.071188	0
Novel_012638	XLOC_13480.	transcript	0	0	0.239091	0.048732	0.085189	0.101483	0.175595	0.169099	0
Novel_012639	XLOC_13489.	transcript	0.670371	0.174142	0.92855	0.763175	0.682167	0.803163	0.684771	0.566707	0.865846
Novel_012640	XLOC_13492.	transcript	0.011685	0	0.013864	0.059894	0.072744	0.039916	0.012566	0.020027	0
Novel_012641	XLOC_13492.	transcript	0	0	0.214348	0	0	0.629269	0.591301	0.541103	0.913508
Novel_012642	XLOC_13492.	transcript	1.867017	0.009273	0.656741	1.501088	2.025718	0.957758	1.794254	0.915172	2.190983

Novel_012643	XLOC_13492.	transcript	0	0	0	0	0	0	0	0	0
Novel_012644	XLOC_13492.	transcript	0	0	2.282574	0	0	0.40434	0.603728	2.351679	0.891113
Novel_012645	XLOC_13495.	transcript	0.540962	0.279511	0.282278	0.487459	0.309455	0.695305	0.660671	0.415661	0.497933
Novel_012646	XLOC_13496.	transcript	0.213773	0.324933	0.126815	0.13191	0.283342	0.239772	0.286782	0.306387	0.223424
Novel_012647	XLOC_13496.	transcript	0.032701	0.173712	0.040362	0.010978	0.090064	0.022971	0.175209	0	0
Novel_012648	XLOC_13496.	transcript	0.111473	0	0.436553	0.168455	0.534599	0.299069	0.150099	0.128201	0.316605
Novel_012649	XLOC_13508.	transcript	0	0.038568	0	0	0	0	0	0.016753	0
Novel_012650	XLOC_13509.	transcript	0.205514	0	0	0.347053	0.364303	0.170579	0	0.004224	0
Novel_012651	XLOC_13509.	transcript	0.069586	0.172325	0.20336	0.026105	0.11496	0.22458	0.385626	0.374912	0.428788
Novel_012652	XLOC_13513.	transcript	0.269569	0.299072	0.235527	0.261405	0.292913	0.274126	0.404861	0.389569	0.161442
Novel_012653	XLOC_13514.	transcript	0.27554	0.150898	0.139267	0.238141	0.26068	0.31678	0.166757	0.208985	0.197949
Novel_012654	XLOC_13514.	transcript	2.601321	0.989936	3.148985	2.947519	2.903506	3.373633	2.922868	2.93786	0.791226
Novel_012655	XLOC_13514.	transcript	0.025245	0.071961	0.023819	0.165712	0	0.129383	0.153087	0.005475	0.691308
Novel_012656	XLOC_13514.	transcript	1.309015	0.680608	0.648827	0.610797	0.574268	0.93456	1.273246	0.950923	0
Novel_012657	XLOC_13514.	transcript	0.247657	0.269546	0.38194	0.278323	0.37625	0.264597	0.54749	0.510465	0.44725
Novel_012658	XLOC_13514.	transcript	0.256901	0.091366	0.031941	0	0	0.200388	0.049594	0.05888	0
Novel_012659	XLOC_13514.	transcript	0	0	0	0.288827	0	0	0	0	0
Novel_012660	XLOC_13517.	transcript	0.593113	0.650815	0.409914	0.341725	0.384529	0.554643	0.440953	0.363426	0.376147
Novel_012661	XLOC_13518.	transcript	1.602626	1.774511	2.498193	3.509329	1.869516	3.5586	0.375693	0.254896	0.368533
Novel_012662	XLOC_13518.	transcript	0	0	1.270532	0	2.368976	0	4.256447	0	0
Novel_012663	XLOC_13520.	transcript	0.374192	0.218449	0.385626	0.378959	0.40091	0.228724	0.265555	0.47442	0.284449
Novel_012664	XLOC_13522.	transcript	2.578342	2.693828	3.611267	2.887211	3.947191	3.448037	4.844365	3.89188	3.294069
Novel_012665	XLOC_13522.	transcript	2.456355	0.71369	0.926754	0.623076	0.761561	1.671243	0.907979	1.598876	0.558484
Novel_012666	XLOC_13522.	transcript	0.472868	1.069707	0	0	0	0.401915	0	0.297019	0
Novel_012667	XLOC_13522.	transcript	0.126316	0.349554	0.561713	0.307157	0.385644	0.152692	0.39797	0.360038	0.133094
Novel_012668	XLOC_13524.	transcript	1.314111	2.103412	1.047681	1.040966	1.165548	0.903702	1.776762	1.464734	1.465056
Novel_012669	XLOC_13526.	transcript	0.444897	9.40E-05	0.445653	0.344867	0.339597	0.251791	0.240305	0.175116	0
Novel_012670	XLOC_13526.	transcript	0	0.472733	0	0	0.078938	0	0.14175	0.121582	0.25764
Novel_012671	XLOC_13526.	transcript	0	0.923598	0	0.110705	0.04007	0.363463	0.370703	0.396054	0.397484
Novel_012672	XLOC_13527.	transcript	0	0.049988	0	0.009175	0	0	0	0.176995	0.043644
Novel_012673	XLOC_13527.	transcript	0.188352	0.111773	0.118551	0.25755	0.268268	0.162323	0.170925	0	0.154573
Novel_012674	XLOC_13527.	transcript	0.15803	0.043477	0	0.591463	0.153336	0.258653	0.201535	0.053781	0.113294
Novel_012675	XLOC_13527.	transcript	0	0	0	0.157316	0	0	0	0	0
Novel_012676	XLOC_13527.	transcript	0	0	0	0	0	0	0	0.113498	0
Novel_012677	XLOC_13527.	transcript	0.178012	0.206441	0.267888	0.085099	0.273419	0.206798	0.437573	0.279672	0.104365
Novel_012678	XLOC_13546.	transcript	0	0	0	0	0	0	0	0	0
Novel_012679	XLOC_13546.	transcript	0	0.005437	0	0	0	0	0	0.000329	0
Novel_012680	XLOC_13546.	transcript	0	0	0	0	0	0	0.09379	0	0

Novel_012681	XLOC_1354€.	transcript	0	0	0.002369	0.01937	0	0.146663	0.035817	0.000247	0
Novel_012682	XLOC_1354€.	transcript	0	0	0	0	0	0	0.06503	0	0
Novel_012683	XLOC_1354€.	transcript	0	0	0	0	0.001648	0	0.027711	0.030539	0
Novel_012684	XLOC_1354€.	transcript	0	0.012249	0	0	0	0	0.110987	0	0
Novel_012685	XLOC_1354€.	transcript	0	0	0.009769	0.003295	0	0.019288	0.051649	0	0
Novel_012686	XLOC_1354€.	transcript	0.004304	0.000613	0	0	0	0	0	0	0
Novel_012687	XLOC_1354€.	transcript	0	0	0	0	0	0	0.032034	0	0
Novel_012688	XLOC_1354€.	transcript	0.093558	0.016216	0.000768	0	0.138951	0.00224	0.088101	0	0.062117
Novel_012689	XLOC_1354€.	transcript	0	0.006029	0	0	0	0	0.092685	0	0
Novel_012690	XLOC_1354€.	transcript	0	0	0.370027	0	0.12922	0.005792	0.157359	0	0
Novel_012691	XLOC_1354€.	transcript	0	0	0.081059	0	0	0	0	0.060359	0
Novel_012692	XLOC_1354€.	transcript	0	0.001074	0	0	0	0.066377	0.050967	1.90E-05	0.006396
Novel_012693	XLOC_1354€.	transcript	0	0	0	0	0	0	0.069832	0	0
Novel_012694	XLOC_1354€.	transcript	0	0	0	0	0	0	0	0	0
Novel_012695	XLOC_1354€.	transcript	0.004282	0	0	0.020252	0.00184	0.001591	0	0.002199	0.010723
Novel_012696	XLOC_1354€.	transcript	0	0	0.003589	0	0.000371	0	0.13882	1.00E-06	0
Novel_012697	XLOC_1354€.	transcript	0.010496	0	0.010278	0.249061	0.008416	0.004902	0.006033	0.033443	0.002195
Novel_012698	XLOC_1354€.	transcript	0.01792	0	0	0.006181	0.00962	0	0.14894	0.25897	0.000192
Novel_012699	XLOC_1354€.	transcript	0	0	0	0	0	0	0.003045	0	0.005372
Novel_012700	XLOC_1354€.	transcript	0.062651	0	0	0.340778	0.153464	0	0.494692	0	0
Novel_012701	XLOC_1354€.	transcript	0.003841	0	0.268788	0.121221	0	0	0.031736	0.032314	0
Novel_012702	XLOC_1354€.	transcript	0	0	0.196897	0	0.007001	0	0.041221	0.049983	0
Novel_012703	XLOC_1354€.	transcript	0.505215	0	0	0	0	0	0.05358	0	0.184797
Novel_012704	XLOC_1354€.	transcript	0	0	0	0	0	0	0.059785	0	0.354726
Novel_012705	XLOC_1354€.	transcript	0.006164	0	0	0	0	0	0	0	0
Novel_012706	XLOC_1354€.	transcript	0.005054	0	0	0.006128	0.00102	0.471283	0.000138	0.000355	0.698397
Novel_012707	XLOC_1354€.	transcript	0	0	0	0	0	0	0.000389	0.006485	0
Novel_012708	XLOC_1354€.	transcript	0.217962	0	0	0.22074	0.234725	0	0.002985	0.161228	0
Novel_012709	XLOC_1354€.	transcript	0	0	0	0	0	0.275704	0.224351	0	0
Novel_012710	XLOC_1354€.	transcript	0	0	0.87676	0	0.244215	0	0.134337	0.787433	0
Novel_012711	XLOC_1355.	transcript	0.056607	0.646963	0.006567	0	0.155712	0.278782	0.161325	0.501827	0.238008
Novel_012712	XLOC_1355.	transcript	0.064057	0.660846	0.005292	0.002726	0.183893	0.518401	0.179229	0.322889	0.331678
Novel_012713	XLOC_1355.	transcript	0.368011	0.677013	0.275381	0	0.122721	0.180653	0.182783	0.227511	0.21844
Novel_012714	XLOC_1355.	transcript	0.14288	0.995496	0.229273	0.185074	0.209507	0.498471	0	0.249677	0.238839
Novel_012715	XLOC_1355.	transcript	0.453318	0.625761	0.272323	0.209445	0.129919	0.202288	0.171673	0.242194	0.391657
Novel_012716	XLOC_1355.	transcript	0	0.634142	0	0	0	0	0	0	0
Novel_012717	XLOC_1355.	transcript	0.365123	0.503076	0.196923	0.174028	0.116918	0.14029	0.208829	0.17496	0.133901
Novel_012718	XLOC_1355.	transcript	0.372167	0.551573	0.20385	0	0.135863	0.139061	0.186437	0.297619	0.12851

Novel_012719	XLOC_13555.	transcript	0.473972	0.545175	0.222211	0.189297	0.146049	0.14184	0.224055	0.23642	0.135283
Novel_012720	XLOC_13555.	transcript	0	0.031927	0	0	0	0	0	0	0
Novel_012721	XLOC_13555.	transcript	0.262917	0.607408	0.389769	0.175234	0.152814	0.352545	0.172357	0.209824	0.176671
Novel_012722	XLOC_13555.	transcript	0.291887	0.515648	0.191873	0.182924	0.134945	0.139895	0.20397	0.161698	0.130212
Novel_012723	XLOC_13555.	transcript	0	0	0	0	0.150798	0.32715	0	0	0
Novel_012724	XLOC_13555.	transcript	0.004201	0.962363	0.411202	0	0.006207	0.003321	0.184856	0.223078	0.002385
Novel_012725	XLOC_13555.	transcript	0	0.740203	0	0	0	0	0	0	0
Novel_012726	XLOC_13555.	transcript	0	0.960055	0	0	0	0	0.189645	0	0
Novel_012727	XLOC_13555.	transcript	0.184452	0.740762	0.24701	0	0.699826	0.320723	0	0.23028	0.2799
Novel_012728	XLOC_13555.	transcript	0.177603	0.57904	0.298216	0.196689	0.133729	0.200017	0.164805	0.168247	0.32128
Novel_012729	XLOC_13557.	transcript	0.138439	0.081475	0.291035	0.606589	0.667456	0.32052	0	0.429077	0.213109
Novel_012730	XLOC_13557.	transcript	0.434345	0.68577	0.157274	0.332343	0.129472	0.438708	0	0.514121	0
Novel_012731	XLOC_13565.	transcript	0.040677	0.705665	0.297652	0.284798	0	0.291194	0	0.349272	0.598304
Novel_012732	XLOC_13565.	transcript	0	0.034334	0	0	0.103713	0	0	0.27007	0.012403
Novel_012733	XLOC_13565.	transcript	0	0.051156	0.113857	0.155601	0.024828	0.037443	0	0.005117	0.013049
Novel_012734	XLOC_13565.	transcript	0.087792	0.243801	0.146764	0	0	0.026768	0.104085	0	0.002144
Novel_012735	XLOC_13565.	transcript	0.361831	0.113286	0.150849	0	0	0.023449	0.304995	0	0
Novel_012736	XLOC_13565.	transcript	0.004898	0.005913	0	0.140942	0.326645	0.145618	0.004874	0.278852	0.167765
Novel_012737	XLOC_13566.	transcript	0.129538	0.061266	0.140472	0.031331	0.048133	0.29636	0.043797	0.06279	0.058142
Novel_012738	XLOC_13566.	transcript	0	0.030733	0.126734	0.132458	0	0	0.111181	0	0.085117
Novel_012739	XLOC_13567.	transcript	0.417197	0.388016	0	0.307451	0.206326	0.29131	0.191161	0.251505	0.304921
Novel_012740	XLOC_13569.	transcript	0.05676	0	0	0.241069	0.305345	0	0.328345	0.231875	0
Novel_012741	XLOC_13569.	transcript	0	0.006871	0	0	0	0.012977	0.031978	0.034566	0
Novel_012742	XLOC_13569.	transcript	0.065865	0.412667	0.138564	0.099062	0.109454	0.094841	0.278926	0.112371	0.070414
Novel_012743	XLOC_13569.	transcript	0	0	0.029584	0.019367	0.029144	0.168914	0	0	0
Novel_012744	XLOC_13569.	transcript	0	0	0	0	0	0	0	0	0
Novel_012745	XLOC_13569.	transcript	0.175393	0.180324	0.076664	0.132564	0.095873	0.131947	0.431382	0.203167	0.03703
Novel_012746	XLOC_13571.	transcript	0.186516	0.032292	0.075566	0	0.039876	0	0.197045	0.047422	0
Novel_012747	XLOC_13573.	transcript	0	0.495226	0.877348	0.924368	0	1.155636	0	0	1.825527
Novel_012748	XLOC_13573.	transcript	1.622057	1.30779	1.814575	2.101062	2.342313	2.343646	2.049695	2.424796	1.562553
Novel_012749	XLOC_13575.	transcript	0	0.038335	0.024781	0	0.247005	0.297088	0.078653	0	0
Novel_012750	XLOC_13577.	transcript	0	0	0	0	0	0	0.000575	0	0
Novel_012751	XLOC_13577.	transcript	0.024433	0.01225	0.024253	0.02027	0.020812	0.019104	0.039482	0.180872	0.01912
Novel_012752	XLOC_13577.	transcript	0.116807	0.012593	0.027602	0.327101	0.356249	0.457713	0.547341	0.000334	0.172023
Novel_012753	XLOC_13577.	transcript	0.129753	0.239124	0.355105	0.002762	0	0	0	0	0.003762
Novel_012754	XLOC_13577.	transcript	0.01011	0	0	0	0	0	0	0.17517	0
Novel_012755	XLOC_13577.	transcript	0.333259	0.37308	0.479666	0.520634	0.496203	0.390304	0.315519	0.37899	0.34955
Novel_012756	XLOC_13588.	transcript	0.289354	0	0.033857	0.064732	0.096878	0	0	0.085859	0

Novel_012757	XLOC_13592.	transcript	1.194059	0.140584	1.768886	0.87151	1.712274	1.78332	1.733701	1.474787	1.118639
Novel_012758	XLOC_13594.	transcript	1.526547	2.203819	1.962303	1.784616	2.281981	2.216416	3.040759	1.945808	4.203971
Novel_012759	XLOC_13594.	transcript	0.066549	0.147247	0.172596	0.150078	0.044359	0.18599	0.126468	0.318792	0.157055
Novel_012760	XLOC_13594.	transcript	0.219292	0.291223	0.38121	0.417931	0.657407	0.616702	0.492111	0.479727	0.494764
Novel_012761	XLOC_13595.	transcript	0	0	0	0	0	0.233786	0	0	0.004132
Novel_012762	XLOC_13595.	transcript	0	0	0	0	0.148655	0.318468	0	0.039013	0.537483
Novel_012763	XLOC_13595.	transcript	0.196536	0.100832	0.366469	0.287615	0.292401	0.16059	0.122726	0.130351	0.154692
Novel_012764	XLOC_13595.	transcript	0	0	0.107855	0.046557	0.408207	0	1.043547	0.223253	0
Novel_012765	XLOC_13595.	transcript	0	0	0	0	0.143622	0.570109	0	0.123369	0.421434
Novel_012766	XLOC_13595.	transcript	0.03497	0.0166	0.016158	0	0.031253	0.02022	0.386462	0.125754	0
Novel_012767	XLOC_13595.	transcript	0.46329	0	0.196069	0.117763	0.094519	0	0	0.151755	0
Novel_012768	XLOC_13595.	transcript	0	0	0	0	0	0	0	0	0
Novel_012769	XLOC_13595.	transcript	0	0	0.030575	0	0.122257	0	0.345019	0.160225	0
Novel_012770	XLOC_13595.	transcript	0.701557	0.271505	0.408716	0.588332	0.279299	0.309368	0.514592	0.427938	0.472963
Novel_012771	XLOC_13595.	transcript	0.701201	0.019601	0	0.10819	0.089162	0.704332	0.663579	0.470128	0
Novel_012772	XLOC_13595.	transcript	0.066128	0	0.186075	0.437681	0.414647	0.573326	0.695624	0.01867	0.326813
Novel_012773	XLOC_13595.	transcript	0.172931	0.883722	0	0	0.489193	0.060462	0.475009	0.027813	0.222024
Novel_012774	XLOC_13595.	transcript	0	0.079671	0.883196	0.908368	0	0	0	0.590803	1.035312
Novel_012775	XLOC_13595.	transcript	0.152605	0.039284	0.064729	0.041916	0.112743	0	0.060096	0.473693	0.749617
Novel_012776	XLOC_13596.	transcript	0	1.050745	0	0	0	0	0	0	0
Novel_012777	XLOC_13596.	transcript	0.065225	0.982727	0	0	0	0	0	0	0
Novel_012778	XLOC_13596.	transcript	0	2.344811	0	0	0	0	0	0	0
Novel_012779	XLOC_13596.	transcript	0	0.81504	0	0	0	0	0	0	0
Novel_012780	XLOC_13596.	transcript	1.669201	1.163108	1.185539	2.259011	2.240233	3.032771	2.429815	3.10389	2.461872
Novel_012781	XLOC_13596.	transcript	0.368982	0.701969	1.033222	0.703561	0.987136	0	0.807851	0.775195	0.380533
Novel_012782	XLOC_13596.	transcript	1.07299	0.214876	0.735723	1.202356	1.764646	1.18735	1.763583	1.785245	2.094581
Novel_012783	XLOC_13596.	transcript	0.20227	0	0	0.141809	0	0.17048	0.146573	0.081045	0
Novel_012784	XLOC_13596.	transcript	0.364566	0.038651	0.744305	1.114162	0.540193	0.948642	0.712572	0.625006	0.222515
Novel_012785	XLOC_13596.	transcript	0	0	1.650215	0	0	0.002657	0	0	0
Novel_012786	XLOC_13596.	transcript	0.169536	0.149899	0.035176	0.020483	0.449551	0.163278	0.148861	0.187349	0.263696
Novel_012787	XLOC_13610.	transcript	0.042279	0.285538	0.020954	0.009215	0.064972	0.043687	0.095019	0.122987	0
Novel_012788	XLOC_13610.	transcript	0	0.098816	0	0	0	0.025953	0.135765	0.000178	0.160675
Novel_012789	XLOC_13610.	transcript	0.38178	0.183892	0	0.179055	0.197889	0.219868	0.19104	0.155764	0
Novel_012790	XLOC_13611.	transcript	0	0	0.183345	0	0	0	0	0	0
Novel_012791	XLOC_13611.	transcript	0.08364	0.549275	0.330425	0.413607	1.073505	0.525087	0.739159	0.415951	0.330019
Novel_012792	XLOC_13611.	transcript	0.56375	0.355479	0.083245	0	0	0	0	0.219239	0.14358
Novel_012793	XLOC_13611.	transcript	0.310497	0.242495	0	0	0	0	0	0.252151	0
Novel_012794	XLOC_13611.	transcript	0	0.260807	0	0	0	0.028795	0	0	0.138394

Novel_012795	XLOC_13611.	transcript	0	0.201485	0	0	0	0	0.360778	0	0
Novel_012796	XLOC_13611.	transcript	1.047138	1.464514	0.63354	0.749728	0.492961	0.814536	1.535126	0.71045	0.091268
Novel_012797	XLOC_13613.	transcript	0.069537	0	0.151107	0.141203	0.310799	0.13983	0.431591	0.450152	0.233344
Novel_012798	XLOC_13613.	transcript	0	0.000833	0	0	0	0	0.010479	4.40E-05	0
Novel_012799	XLOC_13613.	transcript	0	0	0	0.199816	0	0	0.080763	0.001529	0
Novel_012800	XLOC_13613.	transcript	0.012914	0.020563	0.078863	0.031473	0	0.305264	0.001884	0.021927	0.020153
Novel_012801	XLOC_13613.	transcript	0.000948	0.519471	0.158811	0.006596	0	0.101241	0.403189	0.434629	0.019952
Novel_012802	XLOC_13613.	transcript	0.014598	0	0.040533	0.100752	0.14057	0.071285	0.000743	0.02039	0.06808
Novel_012803	XLOC_13613.	transcript	0	0	0	0	0	0	0	0	0
Novel_012804	XLOC_13613.	transcript	0.139166	0.008646	0.009822	0.284319	0.256908	0.106707	0.201279	0.063387	0.048889
Novel_012805	XLOC_13613.	transcript	0	0.000972	0	0	0	0	0.006644	0.001273	0
Novel_012806	XLOC_13613.	transcript	0.032001	0.00726	0	0	0	0.049136	0.000827	0.019911	0
Novel_012807	XLOC_13613.	transcript	0.153933	0.131912	0.027363	0.037508	0.24953	0.015538	0.102315	0.072722	0.139008
Novel_012808	XLOC_13613.	transcript	0.153573	0.080451	0.357818	0.154015	0.124939	0.041497	0.002366	0.02434	0.309977
Novel_012809	XLOC_13613.	transcript	6.457975	6.657845	7.104825	8.608386	7.441869	8.0584	7.206382	9.331112	8.101427
Novel_012810	XLOC_13614.	transcript	0.403872	0	0.184853	0.008289	0.007636	0.40732	0	0.231701	0
Novel_012811	XLOC_13614.	transcript	0	0	0	0.091869	0.117874	0.477645	0.228959	0.104038	0
Novel_012812	XLOC_13614.	transcript	0.178775	0.097564	0.226243	0.35643	0.321348	0.273863	0.15468	0.216088	0.089774
Novel_012813	XLOC_13615.	transcript	0	0.260945	0	0.455657	0.167595	0.260745	0.000938	0.428527	0.182922
Novel_012814	XLOC_13615.	transcript	0.175683	0.049504	0.137435	0.095594	0.240942	0.126342	0.159091	0.181413	0.091332
Novel_012815	XLOC_13615.	transcript	0	0	0.024349	0	0	0	0	0	0
Novel_012816	XLOC_13615.	transcript	0	0.010423	1.014959	0.002699	0	0	0	0.003814	0.312562
Novel_012817	XLOC_13615.	transcript	0.717806	0.473653	0.174589	0.278946	0.683757	0.012429	1.0128	0.460786	0
Novel_012818	XLOC_13615.	transcript	0	0.698005	0	0.872468	0.469668	0.120068	0	0.890006	0
Novel_012819	XLOC_13615.	transcript	0	0	0	0	0.001813	1.301176	0	0.000305	0.378231
Novel_012820	XLOC_13615.	transcript	0.132734	0	0	0.293914	0	0	0	0.3378	0
Novel_012821	XLOC_13616.	transcript	0.577269	0.440508	0.535434	0.711966	0.743604	0.514881	1.021036	0.008538	0.610085
Novel_012822	XLOC_13616.	transcript	0.328314	0.220278	0.215899	0.250223	0.318539	0.404713	0.426409	1.021424	0.081646
Novel_012823	XLOC_13616.	transcript	0.163971	0.148428	0.19783	0.124753	0.214354	0.180617	0.232346	0.171105	0.230517
Novel_012824	XLOC_13616.	transcript	0.034395	0.236921	0.310003	0.630441	0.00048	0.667853	0.417703	0.123799	4.40E-05
Novel_012825	XLOC_13616.	transcript	0	0.054726	0.562024	0	0.664274	0.168238	0.797053	0.343599	0.667901
Novel_012826	XLOC_13616.	transcript	0.909579	0.372694	0.549619	0	0.661567	0.161958	0.343976	0.16942	0.718844
Novel_012827	XLOC_13616.	transcript	0	0	0	0	0	0.105633	0	0.147815	0
Novel_012828	XLOC_13616.	transcript	0	0.004056	0.009027	0.824439	0.019229	0.185344	0.012063	0.416303	0.01114
Novel_012829	XLOC_13616.	transcript	0	0	0	0	0	0.179178	0	0.202957	0
Novel_012830	XLOC_13618.	transcript	0.27678	0.210823	0.525242	0.186026	0.776529	0	0	0.420919	0
Novel_012831	XLOC_13618.	transcript	0.003498	0.25728	0.000325	0.188871	0	0.142392	0.004122	0.263716	0.320708
Novel_012832	XLOC_13618.	transcript	0	0.26252	0	0.307299	0	0	0	0.247785	0.316059

Novel_012833	XLOC_1361[illegible]	transcript	0	0	0	0	0.319266	0.258528	0.45726	0	0.706992
Novel_012834	XLOC_1361[illegible]	transcript	0.105633	0	0.055889	0	0.137379	0	0.033751	0	0
Novel_012835	XLOC_1361[illegible]	transcript	0.118677	0.039648	0.066705	0.119511	0.01548	0.554384	0.436179	0.236354	0.090676
Novel_012836	XLOC_1361[illegible]	transcript	0	0.153723	0	0.339322	0	0.898419	0	0.138245	0.209456
Novel_012837	XLOC_1361[illegible]	transcript	0	1.292586	0.993918	0.647851	0.704016	1.240363	0.458353	0.109652	0.049155
Novel_012838	XLOC_1361[illegible]	transcript	0.019831	0.523469	0.21757	0.603639	0.106209	0.096278	0.392228	0.144209	0.120349
Novel_012839	XLOC_1361[illegible]	transcript	0.580674	0.193087	0.27504	0.479621	0.283511	0.295778	0.692147	0.840466	0.658835
Novel_012840	XLOC_1361[illegible]	transcript	0.079376	0.275948	0.329794	0.24612	0.482083	0.651402	0.047453	0.118796	0.531266
Novel_012841	XLOC_1362[illegible]	transcript	0.191944	0.021145	0.194636	0.237211	0.203042	0.341114	0.40655	0.206857	0.156087
Novel_012842	XLOC_1362[illegible]	transcript	0.122667	0.016833	0.226456	0.104282	0.249939	0.137111	0.318275	0.28631	0.022826
Novel_012843	XLOC_1363[illegible]	transcript	0.227224	0.02174	0.080422	0.263127	0.134469	0.137776	0.217209	0.165558	0.071065
Novel_012844	XLOC_1364[illegible]	transcript	0	0	0	0.012414	0	0	0.057572	0	0.00896
Novel_012845	XLOC_1364[illegible]	transcript	0.325156	0	0.212705	0.431426	0.286759	0.541151	0.171803	0.32088	0.677219
Novel_012846	XLOC_1365[illegible]	transcript	0	0	0	0	0.437173	0	0.519531	0.385958	0
Novel_012847	XLOC_1365[illegible]	transcript	0.600567	0.76572	0.46267	0.592546	0.106429	0.650534	0.141499	0.121529	0.718368
Novel_012848	XLOC_1365[illegible]	transcript	0	0.049395	0	0	0.041572	0	0.110027	0	0
Novel_012849	XLOC_1365[illegible]	transcript	0.940499	0.78449	0.870194	1.15647	1.267575	1.358613	1.439781	1.395682	1.202015
Novel_012850	XLOC_1365[illegible]	transcript	0	0.513142	0	1.093276	0.466049	0.853086	0	0.268371	0.226178
Novel_012851	XLOC_1365[illegible]	transcript	0.222957	0.06017	0.279662	0.134332	0.233269	0.043818	0.448698	0.28475	1.245343
Novel_012852	XLOC_1365[illegible]	transcript	0	0	0	0	0	0	0	0	0
Novel_012853	XLOC_1365[illegible]	transcript	0.168427	0.429711	0.224975	0.180193	0.221853	0.229115	0.333115	0.231571	0.260825
Novel_012854	XLOC_1365[illegible]	transcript	0	0	0	0	0.025358	0	0	0	0
Novel_012855	XLOC_1365[illegible]	transcript	0	0	0.036925	0.130179	0	0	0.009953	0	0
Novel_012856	XLOC_1365[illegible]	transcript	0.535981	0	0.347079	0.202783	0.26894	0.249608	0.373769	0.201044	0.485248
Novel_012857	XLOC_1365[illegible]	transcript	0	0	0.046473	0.175773	0.519671	1.10091	0.173783	0.222543	0.534061
Novel_012858	XLOC_1365[illegible]	transcript	0	0	0	0.104795	0.499936	0	0	0.569036	0.432879
Novel_012859	XLOC_1365[illegible]	transcript	0.756076	1.552452	1.167152	0.519296	0.351166	0.09578	0.820834	0.364199	0.240231
Novel_012860	XLOC_1365[illegible]	transcript	0.038089	0.034022	0.167948	8.60E-05	0.209227	0	0.086177	3.40E-05	0
Novel_012861	XLOC_1365[illegible]	transcript	0.430086	0.456904	0.194486	0.486495	0.201181	0.448529	0.338597	0.493616	0.339056
Novel_012862	XLOC_1365[illegible]	transcript	0.038115	0	0.156705	0.035494	0.021657	0.131441	0.110677	0.109112	0
Novel_012863	XLOC_1366[illegible]	transcript	0.172822	0.011153	0.107687	0.170397	0.2351	0.098317	0.198218	0.220953	0.088767
Novel_012864	XLOC_1367[illegible]	transcript	0.863711	0.609613	1.109293	0.812999	0.745367	0.802593	0.789265	1.072222	0.617022
Novel_012865	XLOC_1368[illegible]	transcript	0	0	0	0	0	0	0	0.988047	0
Novel_012866	XLOC_1368[illegible]	transcript	0.359287	0.086854	0.476192	1.323176	0.569155	0.827443	0.496966	0.246074	0.403741
Novel_012867	XLOC_1369[illegible]	transcript	0.05095	0.606778	0.099164	0.092678	0.077003	0.033513	0.176959	0.07787	0.030326
Novel_012868	XLOC_1369[illegible]	transcript	0.091857	0.353687	0.203078	0.331229	0.350127	0.26382	0.439543	0.293785	0.333633
Novel_012869	XLOC_1369[illegible]	transcript	0.173745	0	0.073302	0	0.001259	0.057682	0.099641	0	0.090846
Novel_012870	XLOC_1370[illegible]	transcript	0	0	0	0.246057	0.013174	0	0.013947	0	0

Novel_012871	XLOC_13703.	transcript	0.340754	0.011466	0.118306	0.368564	0.208431	0.221413	0.323549	0.271831	0.078484
Novel_012872	XLOC_13703.	transcript	0	0	0	0	0	0.120282	0.002642	0.116232	0
Novel_012873	XLOC_13704.	transcript	0	0.267559	0.196897	0	0.062903	0.049734	0.098449	0.096927	0.072928
Novel_012874	XLOC_13705.	transcript	0.037768	0.633179	0.168122	0.065776	0.098414	0.065379	0.110225	0.084324	0.273618
Novel_012875	XLOC_13705.	transcript	0.063457	0	0	0.001097	0	0.008007	0.084247	0	0
Novel_012876	XLOC_13705.	transcript	0.054386	0	0	0.02573	0	0.043945	0.02766	0	0.005592
Novel_012877	XLOC_13705.	transcript	0.032916	0	0	0	0	0	0.040294	0	0
Novel_012878	XLOC_13709.	transcript	0.433788	0.44275	0.336065	0.227549	0.27915	0.420723	0.443775	0.309308	0.346409
Novel_012879	XLOC_13710.	transcript	0.226425	0.559187	0.191268	0.047719	0.305826	0.132184	0.292071	0.309229	0.168468
Novel_012880	XLOC_13710.	transcript	0.142035	0.859333	0.145104	0.188494	0.282821	0.162016	0.362254	0.188213	1.045829
Novel_012881	XLOC_13712.	transcript	0.1915	0.396981	0.434263	0.107028	0.41524	0.303438	0.410731	0.376793	0.542555
Novel_012882	XLOC_13712.	transcript	0.722057	0.467653	0.662896	0.936384	0.751185	0.852576	0.824488	0.765214	0.871377
Novel_012883	XLOC_13712.	transcript	0	0	0	0	0	0	0	0	0
Novel_012884	XLOC_13713.	transcript	0	0	0	0	0	0	0	0	0
Novel_012885	XLOC_13713.	transcript	0	0	0	0	0	0	0	0	0.178336
Novel_012886	XLOC_13713.	transcript	0	0	0	0	0	0	0	0	0
Novel_012887	XLOC_13713.	transcript	0	0	0	0	0	0	0	0	0
Novel_012888	XLOC_13713.	transcript	0	0	0	0	0	0	0	0	0
Novel_012889	XLOC_13713.	transcript	0	0	0	5.392259	0	0	0	0	0
Novel_012890	XLOC_13713.	transcript	1.00E-06	0	0	0.000797	0	3.089607	0	0	0
Novel_012891	XLOC_13713.	transcript	1.649325	0.374587	1.559546	0	1.82903	0	0	1.14552	0
Novel_012892	XLOC_13713.	transcript	0	0	0	0	0	0	0	0	0
Novel_012893	XLOC_13713.	transcript	0	0	0	0	0	0	0	0	0
Novel_012894	XLOC_13713.	transcript	0	0	0	0	0	0	0	0	0
Novel_012895	XLOC_13713.	transcript	0	3.676569	0	0	0	0	0	0	0
Novel_012896	XLOC_13713.	transcript	0	0	0	0	0	0	0	0	0
Novel_012897	XLOC_13713.	transcript	0.934674	0	4.625905	0	5.677217	0.395202	3.988255	3.586538	2.418731
Novel_012898	XLOC_13713.	transcript	0	0	0	0	0	0	0	0	0.424009
Novel_012899	XLOC_13713.	transcript	0	0	0	0	0	0	0	0	0
Novel_012900	XLOC_13714.	transcript	0	0.169916	0	0.130894	0.044756	0.020577	0.143146	0	0.8295
Novel_012901	XLOC_13714.	transcript	0.714305	0.729589	0.322595	0.085223	0.416911	0.536671	0.558648	0.394837	0.051266
Novel_012902	XLOC_13714.	transcript	0	0	0	0	0	0	0	0	0
Novel_012903	XLOC_13714.	transcript	0.657349	0	0.682287	0.981608	0.784373	0.341639	0.666815	0.764397	0
Novel_012904	XLOC_13715.	transcript	0.44438	0.594761	0.386652	0.571743	0.519584	0.400887	0.862531	0.672213	0.492003
Novel_012905	XLOC_13717.	transcript	0.083147	0.156306	0.336134	0.15665	0.483999	0.293682	0.238019	0.259128	0.304658
Novel_012906	XLOC_13717.	transcript	0	0	0	0	0	0	0	0	0
Novel_012907	XLOC_13718.	transcript	0	0.304128	0	0.268256	0	0.026311	0	0.132704	0.098121
Novel_012908	XLOC_13718.	transcript	0.084476	0	0.245641	0.087237	0.217792	0.139658	0.294107	0.126396	0.087246

Novel_012909	XLOC_13729.	transcript	0	0.337098	0.013418	0	0	0	0	0.095924	0
Novel_012910	XLOC_13729.	transcript	0.091997	0.372406	0.085271	0	0.021705	0.074493	0	0.013207	0
Novel_012911	XLOC_13730.	transcript	0.974158	0.019929	0	0.024728	0.006501	0	0.821795	0.014616	0
Novel_012912	XLOC_13730.	transcript	0	0.017005	0	0.014486	0	0.022393	0.162399	0.007495	0
Novel_012913	XLOC_13730.	transcript	0.043239	0.026896	0.247761	0.064034	0.117777	0	0	0.125955	0
Novel_012914	XLOC_13730.	transcript	0	0.046883	0	0	0	0	0	0.000374	0
Novel_012915	XLOC_13730.	transcript	0.366663	0.665949	0.165116	0.362453	0.338093	0.397595	0.212723	0.319367	0.079435
Novel_012916	XLOC_13730.	transcript	0.552451	0.431176	0.048451	0.142652	0.155919	0.440231	0.355293	0.149033	0.311937
Novel_012917	XLOC_13730.	transcript	0.131826	0.190476	0.06485	0.10282	0	0	0.068814	0.122805	0.13991
Novel_012918	XLOC_13734.	transcript	0.150924	0.387149	0.232121	0.196683	0.1371	0.276532	0.321722	0.174177	0.047832
Novel_012919	XLOC_13738.	transcript	0.080371	0.103254	0.336936	0.126193	0.22086	0.081687	0.149058	0.187918	0
Novel_012920	XLOC_13738.	transcript	0.517592	0.222819	0.115751	0.148935	0.208747	0.375362	0.170658	0.128276	0.261112
Novel_012921	XLOC_13738.	transcript	0.274956	0.006147	0.297844	0.284849	0.275451	0.351956	0.302385	0.463	0.163267
Novel_012922	XLOC_13738.	transcript	0.089115	0	0.087817	0.147287	0.175657	0	0.100661	0.162486	0.030821
Novel_012923	XLOC_13739.	transcript	0.007734	0	0	0.208154	0	0.008756	0.003279	0.004849	0.022291
Novel_012924	XLOC_13739.	transcript	0.255389	0	0	0.074286	0	0.1144	0.150181	0	0
Novel_012925	XLOC_13739.	transcript	0.12768	0.064893	0.429194	0.185832	0.487361	0.5385	0.551617	0.13394	0.37228
Novel_012926	XLOC_13739.	transcript	0	0	0	0	0	0	0	0.255488	0
Novel_012927	XLOC_13739.	transcript	0.123006	0.01392	0.096173	0.125248	0.126365	0.169063	0.221952	0.183913	0.193424
Novel_012928	XLOC_13741.	transcript	0.740937	0.832626	1.068552	1.98498	1.356717	1.478795	1.757882	1.72639	1.862952
Novel_012929	XLOC_13741.	transcript	0.500004	0.17274	0.573701	0.748656	0.491152	0.808594	0.749313	0.485454	0.704274
Novel_012930	XLOC_13745.	transcript	0.31151	0.174497	0.538148	0.583416	0.329388	0.13042	0.621458	0.373046	0.695765
Novel_012931	XLOC_13748.	transcript	0.969009	1.399549	1.153743	1.384403	1.022123	1.345074	1.182225	1.095535	1.341115
Novel_012932	XLOC_13748.	transcript	1.693017	1.869944	1.62145	0.938027	1.743413	1.349384	1.579858	1.075669	1.196279
Novel_012933	XLOC_13748.	transcript	0	0	0	0	0	0	0	0.1706	0
Novel_012934	XLOC_13748.	transcript	0	0.048239	0.009272	0	0.030309	0.08953	0.012762	0.148019	0
Novel_012935	XLOC_13748.	transcript	0.385483	0.500738	0.56622	0.337722	0.45548	0.561761	0.520856	0.458384	0.348891
Novel_012936	XLOC_13748.	transcript	0.028176	0	0	0.064076	0	0.913631	0	0.022954	0.071663
Novel_012937	XLOC_13748.	transcript	2.996878	8.385839	2.242247	1.975275	2.403582	1.284841	3.754475	3.519216	2.071795
Novel_012938	XLOC_13750.	transcript	0.028517	0.262125	0.090346	0.119343	0.23849	0.160437	0.190279	0.100892	0.17544
Novel_012939	XLOC_13750.	transcript	0.160392	0.073823	0	0	0.020373	0	0.049137	0	0.115891
Novel_012940	XLOC_13751.	transcript	0.529649	0.812115	0.615456	0.600109	0.509603	0.717416	0.607503	0.538027	0.404684
Novel_012941	XLOC_13753.	transcript	0	0	0.008423	0	0	0	0.482836	0	0.007678
Novel_012942	XLOC_13753.	transcript	0	0.214837	0.259244	0	0	0	0.514385	0.66524	0.392313
Novel_012943	XLOC_13753.	transcript	0.147249	0.220487	0.22147	0.389357	0.042504	0.432934	0.018203	0	0.104426
Novel_012944	XLOC_13753.	transcript	0.446894	0	0	0	0.568805	0	0	0	0
Novel_012945	XLOC_13753.	transcript	0	0.18545	0	0	0	0	0	0	0
Novel_012946	XLOC_13754.	transcript	0	0.145067	0	0	0	0.157831	0	0.110562	0

Novel_012947	XLOC_13754.	transcript	0.126888	0.202177	0	0.172812	0.06489	0.031002	0.094898	0.077069	0.278826
Novel_012948	XLOC_13755.	transcript	0.012075	0	0	0.048862	0	0	0	0.004373	0
Novel_012949	XLOC_13755.	transcript	0.041763	0	0	0	0	0	0	0	0
Novel_012950	XLOC_13755.	transcript	0.256923	0	0.021946	0	0.001939	0.018144	0.257783	0.358023	0.007945
Novel_012951	XLOC_13755.	transcript	0.462547	0	0	0	0	0	0.55212	0	0
Novel_012952	XLOC_13755.	transcript	0.176015	0.09659	0.561926	0.422714	0.429264	0.539612	0	0.461821	0.286665
Novel_012953	XLOC_13755.	transcript	0	0	0	0	0	0	0	0	0
Novel_012954	XLOC_13759.	transcript	0.400961	0.397646	0.280362	0.176318	0.474481	0.243638	0.387278	0.576008	0.391503
Novel_012955	XLOC_13759.	transcript	0.039461	0	0	0.01557	0	0.10845	0.021883	0.012027	0.027566
Novel_012956	XLOC_13759.	transcript	0	0	0	0	0	0	0	0	0
Novel_012957	XLOC_13760.	transcript	0.381225	0.571693	0.372021	0.603929	0.33742	0.385266	0.372335	0.509985	0.319093
Novel_012958	XLOC_13760.	transcript	0.015492	0	0.049136	0.078654	0.016235	0.024958	0.068986	0.012146	0
Novel_012959	XLOC_13761.	transcript	0.012498	0	0.063516	0.088425	0.06155	0.06039	0.032171	0.016732	0.132366
Novel_012960	XLOC_13761.	transcript	6.900164	2.320429	6.163517	8.181685	5.435277	4.416592	7.844676	6.114439	8.037
Novel_012961	XLOC_13768.	transcript	1.429826	0	0	0	0.425756	0	0.875621	1.543385	0
Novel_012962	XLOC_13768.	transcript	1.068883	1.831302	1.929943	2.086924	1.600542	2.224051	2.249506	1.533546	1.652657
Novel_012963	XLOC_13772.	transcript	0	0	0	0.04529	0	0.321521	0.242994	0	0
Novel_012964	XLOC_13772.	transcript	0.27798	0.165906	0	0.176972	0.077323	0	0.02816	0.077861	0.438154
Novel_012965	XLOC_13773.	transcript	0	0	0	0	0.07985	0.029862	0.211453	0.034674	0.095695
Novel_012966	XLOC_13773.	transcript	0.044117	0.10969	0	0	0	0	0.233724	0	0
Novel_012967	XLOC_13775.	transcript	0.123818	0	0	0.005525	0	0	0.017868	0.045138	0.149439
Novel_012968	XLOC_13775.	transcript	8.126998	17.66126	13.02319	11.31116	9.046963	11.78466	7.775705	14.65021	8.84928
Novel_012969	XLOC_13777.	transcript	0.244054	0	0	0.106291	0	0.138111	0	0.130452	0.02861
Novel_012970	XLOC_13777.	transcript	0	0.030778	0.002316	0.002012	0	0.041927	0.36014	0.003833	0.039518
Novel_012971	XLOC_13787.	transcript	0.065261	0	0.212473	0.107313	0.016445	0.154542	0.202877	0.006519	0
Novel_012972	XLOC_13787.	transcript	0	0.024758	0.183535	0.37296	0.23017	0	0	0.209218	0.08835
Novel_012973	XLOC_13790.	transcript	0.178158	0.723781	0.367791	0.48322	0.344918	0.469567	0.282004	0.479354	0.622013
Novel_012974	XLOC_13791.	transcript	0.327237	0.014419	0.593568	0.584272	0.895265	0.331192	0.405388	0.070259	0.111023
Novel_012975	XLOC_13791.	transcript	0	0	0	0	0	0	0	0.451258	0
Novel_012976	XLOC_13791.	transcript	0	0	0	0	0	0.031941	0	0	0
Novel_012977	XLOC_13791.	transcript	0	0.839914	0.360462	0.91142	0.750487	0.722987	0.772845	0.500572	0
Novel_012978	XLOC_13791.	transcript	0.080836	1.212287	1.212867	0.801939	0.86953	0.764322	1.120333	0.635618	0.877608
Novel_012979	XLOC_13791.	transcript	0.727	0.193924	0.008495	0.093032	0.191181	0	0.012638	0.2114	0.314816
Novel_012980	XLOC_13794.	transcript	0.498181	0.558729	0.772114	0.566091	0.883139	0.743461	0.932679	0.846838	0.320997
Novel_012981	XLOC_13794.	transcript	0.347751	0	0	0	0	0	0	0	0
Novel_012982	XLOC_13794.	transcript	0	0.332909	0.039183	0.060855	0.256031	0.239429	0.156063	0.154175	0.075528
Novel_012983	XLOC_13794.	transcript	0	0	0.295637	0.234996	0.24705	0	0.223307	0.155957	0.325907
Novel_012984	XLOC_13795.	transcript	0	0	0	0	0	0	0	0	0

Novel_012985	XLOC_13795.	transcript	0.234628	0.405964	0.246652	0.237634	0.236045	0.307554	0	0.278824	0.254102
Novel_012986	XLOC_13795.	transcript	0.080421	0.109675	0.07386	0.078367	0.129599	0.161294	0.703929	0.143047	0.102873
Novel_012987	XLOC_13795.	transcript	0.41848	0.792097	0.299094	0.560384	0.19146	0.351822	0.271081	0.40891	0.079719
Novel_012988	XLOC_13795.	transcript	0.340804	0.166984	0.158378	0.192622	0.401358	0.179376	0	0.113643	0.326038
Novel_012989	XLOC_13795.	transcript	0.309148	0.295426	0.249684	0.307202	0.559055	0.523951	0.086978	0.313882	0.358119
Novel_012990	XLOC_13795.	transcript	0.155154	0.14185	0.216124	0.083047	0.182495	0.208545	0.440989	0.209836	0.146299
Novel_012991	XLOC_13795.	transcript	0.888275	0.105749	0.594446	0.492819	0.552992	0.455332	0.830457	0.496569	0.419517
Novel_012992	XLOC_13797.	transcript	0.420894	0.072161	0.234073	0.115102	0.300893	0.327713	0.166584	0.442703	0.00415
Novel_012993	XLOC_13798.	transcript	0.045327	0.106054	0	0.069293	0.204327	0.355688	0	0.204618	0
Novel_012994	XLOC_13799.	transcript	0.431745	0.093415	0.383223	0.337286	0.287443	0.240674	0.458286	0.410829	0.156698
Novel_012995	XLOC_13799.	transcript	0	0	0	0	0.287905	0	0	0	0
Novel_012996	XLOC_13799.	transcript	0.516841	0.040665	0.531245	0.301324	0.666476	0.393612	0.648247	0.630277	0.305831
Novel_012997	XLOC_13803.	transcript	0.249715	0.482345	0.106013	0.140939	0.242089	0.260407	0.276685	0.340624	0.376159
Novel_012998	XLOC_13803.	transcript	0.402911	0.343975	0.708914	0.691871	0.610399	0.586965	0.905013	0.762338	0.831235
Novel_012999	XLOC_13803.	transcript	0	0.090121	0	0.042495	0	0.166168	0	0.133598	0
Novel_013000	XLOC_13803.	transcript	28.34933	0.40779	46.35569	77.33951	36.62666	64.18293	27.65211	41.6409	203.8209
Novel_013001	XLOC_13803.	transcript	0	0	0	0	0	0	0.010127	0	0.026387
Novel_013002	XLOC_13804.	transcript	0	0	0	0	0	2.668821	0	0	0
Novel_013003	XLOC_13804.	transcript	0.003503	0.001805	0.053161	0.363046	0.731996	0.007218	0.000767	0.002864	0
Novel_013004	XLOC_13804.	transcript	0.872219	0.78207	0.841451	0.681882	0.502631	0.311844	0.920066	0.976999	1.90495
Novel_013005	XLOC_13813.	transcript	0.104861	0.085572	0.08282	0.013837	0.058984	0.036903	0.094371	0	0.120466
Novel_013006	XLOC_13813.	transcript	0.0129	0	0	0	0	0	0	0.034645	0
Novel_013007	XLOC_13813.	transcript	0	0	0.129462	0	0	0	0.019385	0.029213	0
Novel_013008	XLOC_13816.	transcript	0.310046	0.190513	0.215657	0.212541	0.329909	0.206404	0.395045	0.034271	0.232257
Novel_013009	XLOC_13816.	transcript	0	0	0.346477	0	0	0	0	0.234067	0
Novel_013010	XLOC_13818.	transcript	0.297608	0.478604	0.298926	0.421808	0.292627	0.303718	0.253961	0.305991	0.445681
Novel_013011	XLOC_13828.	transcript	0.414874	0.524874	0.025969	0.212315	0.313116	0.315223	0.392548	0.1988	0.207201
Novel_013012	XLOC_13830.	transcript	0.148697	0.129752	0.052401	0	0	0	0.074139	0.052791	0
Novel_013013	XLOC_13835.	transcript	1.03993	0.027986	0.526542	0.347008	0.059107	1.60E-05	0.408316	0.626104	0.004339
Novel_013014	XLOC_13835.	transcript	0	0	0	0	0.72898	0.551566	0.305939	0	0
Novel_013015	XLOC_13835.	transcript	0.77777	0.023973	0.547099	0.439857	0.52913	0.552917	0.436794	0.396474	0.293933
Novel_013016	XLOC_13835.	transcript	0	0	0.019008	0	0	0	0.504495	0	0.093393
Novel_013017	XLOC_13838.	transcript	2.897277	0.806709	2.739534	3.262312	3.110064	2.514953	3.335206	3.823186	1.840938
Novel_013018	XLOC_13839.	transcript	0.164035	0.065383	0.023507	0.007454	0.05458	0.010731	0.122547	0.050854	0.803095
Novel_013019	XLOC_13843.	transcript	0.015785	0	0	0.196587	0	0	0.019669	0	0
Novel_013020	XLOC_13843.	transcript	0.014294	0	0.164856	0.143257	0.385374	0.372739	0.145483	0.029265	0
Novel_013021	XLOC_13844.	transcript	0.375708	0.382943	0.294443	0.307664	0.260512	0.471781	0.422279	0.45477	0.270745
Novel_013022	XLOC_13846.	transcript	0	0	2.967978	0	12.03599	0	1.81091	0	11.99111

Novel_013023	XLOC_13846.	transcript	0.019416	1.313031	0	0.014512	0	0.106745	0	0	0
Novel_013024	XLOC_13846.	transcript	0	0	0	0	0	0	0	0.451513	0
Novel_013025	XLOC_13846.	transcript	0.000354	0	0	0	0	0	0	0	0
Novel_013026	XLOC_13846.	transcript	0	0	0	0	0	0	0	0	0
Novel_013027	XLOC_13846.	transcript	5.89681	0	0.096693	0.010595	2.38274	0	3.020719	4.70E-05	0.108389
Novel_013028	XLOC_13846.	transcript	0	0.537146	0	0	0	0	0	0	0
Novel_013029	XLOC_13846.	transcript	2.577338	0	0	2.18777	0	0	7.20E-05	0.006358	0
Novel_013030	XLOC_13846.	transcript	0	0	2.564346	1.536874	0	8.443112	0	0	1.933222
Novel_013031	XLOC_13846.	transcript	0	0	0	0	0	0	0	13.38561	0
Novel_013032	XLOC_13846.	transcript	0	0	2.895579	0	0	0	0	1.353096	0
Novel_013033	XLOC_13846.	transcript	0	0	0	0	0	0	0	0	0
Novel_013034	XLOC_13846.	transcript	0.000439	4.00E-06	0	0	0	0.006027	0	0	0
Novel_013035	XLOC_13846.	transcript	0.00017	0	0.012058	0.13523	0	0.000206	8.90E-05	0.009264	0.000103
Novel_013036	XLOC_13846.	transcript	0	0.008579	0	0	0	0	0	0.048964	0.001794
Novel_013037	XLOC_13848.	transcript	0.128471	0.070362	0.231759	0.303215	0.290948	0.425463	0.332171	0.217121	0.183433
Novel_013038	XLOC_13848.	transcript	0.286884	0	0.252628	0.164602	0.323659	0.221838	0.156904	0.083331	0.396693
Novel_013039	XLOC_13848.	transcript	0.309201	0	0	0	0	0	0.000166	0	0
Novel_013040	XLOC_13848.	transcript	0.010486	0.401033	0.344956	0.598077	0.498008	0.353215	1.278986	0.72122	0.355607
Novel_013041	XLOC_13848.	transcript	0	0	0	0	0	0.087807	0.016288	0.184219	0
Novel_013042	XLOC_13849.	transcript	0.159856	0.312649	0.26418	0.160406	0.385037	0.200731	0.328141	0.267502	0.146162
Novel_013043	XLOC_13855.	transcript	0.455159	0.813098	0.168862	0.46955	0.443557	0.474684	0.331516	0.526052	0.513299
Novel_013044	XLOC_13859.	transcript	0.349717	0.085084	0.4312	0.512301	0.424346	0.49037	0.472516	0.630108	0.33174
Novel_013045	XLOC_13865.	transcript	0	0	0.293782	0.493938	0	0	0.023051	0.008685	0
Novel_013046	XLOC_13865.	transcript	0.015332	0	0.00339	0.004205	0	0.006633	0.015603	0.004717	0
Novel_013047	XLOC_13865.	transcript	0.144886	0.01157	0.126489	0.297212	0.193905	0.188373	0.234387	0.236584	0.35776
Novel_013048	XLOC_13865.	transcript	0	0	0	0	0.162146	0	0	0	0
Novel_013049	XLOC_13865.	transcript	0.019674	0.000631	0.140376	0.05099	0.024578	0.00689	0.002581	0.015256	0.009393
Novel_013050	XLOC_13865.	transcript	0.407508	0.098754	0.257106	0.207797	0.198933	0.336731	0.659579	0.507707	0.226331
Novel_013051	XLOC_13866.	transcript	0.132416	1.48309	0.322226	0.21197	0.181826	0.327655	0.126929	0.209019	0.309806
Novel_013052	XLOC_13866.	transcript	0.370459	4.228994	0.354479	0.325586	0.222326	0.307909	0.339961	0.290454	0.258671
Novel_013053	XLOC_13867.	transcript	0.508152	0.544751	0.450951	0.402041	0.2626	0.539099	0.548032	0.398012	0.224693
Novel_013054	XLOC_13867.	transcript	0.11659	0.225059	0.191222	0.202339	0.159504	0.244166	0.152206	0.121321	0.297889
Novel_013055	XLOC_13870.	transcript	0.362498	0.110605	0.221446	0.327175	0.180708	0.362077	0.369372	0.305439	0.253418
Novel_013056	XLOC_13871.	transcript	0.261558	0.135824	0.304538	0.344884	0.336231	0.322483	0.145322	0.362345	0.337297
Novel_013057	XLOC_13871.	transcript	0	0	0	0	0	0	0.181669	0	0
Novel_013058	XLOC_13871.	transcript	0.263467	0.026077	0	0	0	0	0	0	0
Novel_013059	XLOC_13878.	transcript	0	0.250562	0.070899	0	0	0	0	0.098707	0
Novel_013060	XLOC_13878.	transcript	0.383052	0	0.029297	0.047857	0.372932	0.271644	0.406154	0.034358	0.186783

Novel_013061	XLOC_13878.	transcript	0	0.002808	0.02056	0	0	0	0	0	0
Novel_013062	XLOC_13878.	transcript	0	0.684351	0.426771	0.354748	0	0	0	0.434189	0
Novel_013063	XLOC_13885.	transcript	0	64.87801	0	0	26.1233	0	55.87108	89.96905	0
Novel_013064	XLOC_13885.	transcript	45.14504	6.516344	53.78833	46.3027	32.20203	45.94524	4.206254	6.940139	79.53973
Novel_013065	XLOC_13887.	transcript	0.049836	0	0.041138	0.080966	0.107355	0	0.164313	0.306297	0.089423
Novel_013066	XLOC_13888.	transcript	0.027804	0.403386	0.017505	0.040906	0.445445	0.222369	0.066991	0.188331	0.000618
Novel_013067	XLOC_13888.	transcript	0.028854	0.067435	0	0.037011	0	0	0.056159	0	0.163182
Novel_013068	XLOC_13889.	transcript	0	0.006858	0	0	0	0	0	0.444172	0
Novel_013069	XLOC_13889.	transcript	0.879265	0.497648	0.351054	0.729475	0.502409	0.318241	1.044236	0.357742	0.319316
Novel_013070	XLOC_13894.	transcript	0.096843	0	0	0.067318	0.317489	0	0.290198	0.11372	0
Novel_013071	XLOC_13894.	transcript	0.156157	0.172828	0	0.05631	0.243999	0	0.18952	0.109609	0
Novel_013072	XLOC_13894.	transcript	0.109121	0.063844	0.066714	0.174821	0	0.065233	0	0	0
Novel_013073	XLOC_13894.	transcript	0.050743	0.264867	0	0.4714	0.002666	0.053843	0.482577	0.028929	0
Novel_013074	XLOC_13894.	transcript	0.234776	0	0	1.081897	0.435334	0.818158	0.428586	0.968491	0
Novel_013075	XLOC_13895.	transcript	0	0.601571	0	0	0	0	0	0	0
Novel_013076	XLOC_13895.	transcript	0	0	0	0.088771	0.236473	0.013881	0.12897	0	0.111842
Novel_013077	XLOC_13895.	transcript	0	0	0	0.028509	0	0	0	0.042925	0
Novel_013078	XLOC_13895.	transcript	0.027527	0.079373	0.154764	0	0	0.129324	0.128043	0.124057	0.083931
Novel_013079	XLOC_13896.	transcript	0	0	0.204745	0.06485	0.118146	0	0.237943	0.207752	0.029004
Novel_013080	XLOC_13896.	transcript	0.105113	0.105497	0.018565	0.001616	0.112525	0	0.017051	0.0534	0.046522
Novel_013081	XLOC_13896.	transcript	0.085085	0.344232	0.108313	0.074491	0.203267	0.181112	0.337256	0.036163	0.062976
Novel_013082	XLOC_13897.	transcript	0.14306	0.54477	0.312757	0.179753	0.104093	0.265594	0.253975	0.136756	0.16588
Novel_013083	XLOC_13898.	transcript	0.095994	0.086682	0.173932	0.252446	0.201268	0.242571	0.246428	0.079364	0.059746
Novel_013084	XLOC_13900.	transcript	0.476977	1.058894	0.586387	1.038346	0.552682	0.863246	0.661609	1.189819	0.945879
Novel_013085	XLOC_13900.	transcript	0.478888	0.367197	0.2299	0.163581	0	0.167245	0.447995	0	0
Novel_013086	XLOC_13900.	transcript	0	0.304101	0.262782	0.304254	0.625457	0.252003	0.384703	0.23093	0
Novel_013087	XLOC_13901.	transcript	0	0	0.163183	0.127809	0.19619	0.206474	0.074803	0.20799	0.100177
Novel_013088	XLOC_13901.	transcript	0.197766	0.168092	0.374817	0.525145	0.264457	0.368194	0.438211	0.227702	0.125968
Novel_013089	XLOC_13901.	transcript	0	0	0	0	0	0	0	0	0
Novel_013090	XLOC_13901.	transcript	0.139935	0.144744	0.040637	0.060724	0.031356	0.066907	0.091358	0.131738	0.117068
Novel_013091	XLOC_13901.	transcript	0.412679	0.155877	0.172608	0.075199	0.02438	0.076086	0.104912	0.146584	0.290165
Novel_013092	XLOC_13903.	transcript	0	0	0	0	0	0	0.025058	0	0.274372
Novel_013093	XLOC_13903.	transcript	0.189278	0.03688	0.246299	0.256899	0.288401	0.281962	0.397737	0.043858	0.013407
Novel_013094	XLOC_13904.	transcript	0.565917	0.387825	1.795341	1.093912	1.598861	1.443673	0.889181	0.547316	0.585534
Novel_013095	XLOC_13904.	transcript	1.408433	0.710373	0.531188	0.853921	0.554364	0.800153	1.696081	1.490305	0.423027
Novel_013096	XLOC_13904.	transcript	0	0	0	0	0	0	0	0	0.584165
Novel_013097	XLOC_13904.	transcript	0.186197	0.431002	0.57067	0.646649	0.586679	0.485068	0.307927	0.490251	0.153225
Novel_013098	XLOC_13904.	transcript	0	0	0	0.199614	0	0	0.131656	0	0.04192

Novel_013099	XLOC_13904	transcript	0	0.087888	0	0.404641	0.127689	0	0.022735	0.152454	0.158268
Novel_013100	XLOC_13904	transcript	0.016244	0.09596	0	0.018277	0.001418	0	0.003032	0.007071	0
Novel_013101	XLOC_13904	transcript	0.108573	0.362005	0.108477	0.110432	0.109113	0.102315	0.094629	0.090667	0.037865
Novel_013102	XLOC_13905	transcript	0.265809	0.096754	0.387332	0.328562	0.17276	0.141148	0.370928	0.252129	0.411669
Novel_013103	XLOC_13906	transcript	0.194601	0.768239	0.497357	0.650724	0.187483	0.671721	0.419051	0.225528	0
Novel_013104	XLOC_13906	transcript	0.347341	0.096403	0.145031	0.156606	0.20155	0.166716	0.320882	0.126587	0.230302
Novel_013105	XLOC_13907	transcript	0	0	0	0	0	0	0	0	0
Novel_013106	XLOC_13907	transcript	0	0.023029	0	0	0.020984	0	0.079871	0	0
Novel_013107	XLOC_13909	transcript	0	0.937871	0	0	0	0	0	0	0
Novel_013108	XLOC_13923	transcript	0.7449	0.095518	0.496068	0.048476	0.094593	0.151534	0.548112	0.460839	0.628093
Novel_013109	XLOC_13923	transcript	1.376908	0.265513	1.029556	1.159512	0.050268	0.025156	0.71193	0.816449	0.027507
Novel_013110	XLOC_13923	transcript	0.6533	0.180599	0.279585	0.087339	0.531893	0.476335	0.359594	0.307147	0.405863
Novel_013111	XLOC_13923	transcript	0.029402	0	0.006228	0	0	0	0	0	0
Novel_013112	XLOC_13923	transcript	0.006172	0.495531	0	0	0.003153	0	0	0	0.003677
Novel_013113	XLOC_13926	transcript	0	0.524161	0	0	0	0	0	0	0
Novel_013114	XLOC_13940	transcript	0.012838	0.068686	0.085823	0.018829	0.131397	0.083152	0.253334	0.109765	0.35706
Novel_013115	XLOC_13946	transcript	0	0.367468	0.563713	0.522056	0.416463	0	0.023851	0.261533	0
Novel_013116	XLOC_13954	transcript	1.120347	1.39565	0.900905	0.750383	1.046777	1.009872	1.402749	1.122045	0.852932
Novel_013117	XLOC_13963	transcript	0.509652	0.273828	0.525407	0.281382	0.375756	0.35325	0.442633	0.54424	0.794087
Novel_013118	XLOC_13966	transcript	0	0	0	0	0	0	0	0.030374	0.106368
Novel_013119	XLOC_13968	transcript	0.557734	0.020768	0.561294	0.609958	0.644247	0.561497	0.742539	0.862336	0.490128
Novel_013120	XLOC_13971	transcript	0.541439	0.865518	0.859196	0.566453	0.508991	0.476705	0.706107	0.312479	0.698528
Novel_013121	XLOC_13971	transcript	0	0	0	0	0	0	0	0	0.000199
Novel_013122	XLOC_13971	transcript	0.289678	0	0	0.010671	0.092275	0	0.155307	0.604745	0
Novel_013123	XLOC_13981	transcript	0.858406	2.497937	1.042086	1.005574	1.08101	1.246565	1.122476	1.021969	0.983538
Novel_013124	XLOC_13982	transcript	0	0.143705	0.866905	0.682123	0	0.194247	1.235334	1.263108	0.549803
Novel_013125	XLOC_13982	transcript	1.160241	0	0.002293	0.003657	1.122909	1.190553	0.003632	0.005227	0.00267
Novel_013126	XLOC_13982	transcript	0	0	0.749242	1.563264	0	0	0.325622	0.813051	1.633485
Novel_013127	XLOC_13984	transcript	0.369849	2.606593	0.900214	0.944396	0.850297	1.263499	1.058691	1.108433	1.118316
Novel_013128	XLOC_13984	transcript	0	0.377391	0	0	0.25705	0	0.454631	0	0
Novel_013129	XLOC_13986	transcript	1.604761	0.740348	0.837331	1.945863	1.948876	1.850852	2.155409	1.014447	1.637571
Novel_013130	XLOC_13986	transcript	0	0	1.109313	0	0	0	0	0	0
Novel_013131	XLOC_13986	transcript	0	1.472285	0	0	0	0	0	1.807359	0
Novel_013132	XLOC_13986	transcript	0.218096	0.21496	0.159731	0.155152	0.203907	0.106689	0.191965	0.210151	0.11812
Novel_013133	XLOC_13987	transcript	1.418828	4.532578	2.010222	1.625754	1.821044	1.716106	2.97134	2.710355	2.051997
Novel_013134	XLOC_13987	transcript	0.347916	0.183956	0.028546	0.520766	0.428227	0.387693	0.059755	0.151123	0.035065
Novel_013135	XLOC_13988	transcript	0.667844	1.167323	0.775809	0.528864	0.692758	0.623699	0.878767	0.566947	0.400638
Novel_013136	XLOC_13988	transcript	0.010598	0.050152	0.016768	0	0	0.051145	0.026866	0	0.487231

Novel_013137	XLOC_13989.	transcript	0	0	0	0	0	0	0.005478	0.434847	0
Novel_013138	XLOC_13989.	transcript	0.536633	0.140735	0.528311	0.539328	0.578358	0.513363	0.677118	0.0219	0.398129
Novel_013139	XLOC_13989.	transcript	0.037191	0.010348	0.022044	0.011592	0.024149	0.027358	0.0371	0.210361	0
Novel_013140	XLOC_13989.	transcript	0	0	0	0	0	0.021156	0	0	0
Novel_013141	XLOC_13991.	transcript	0.447075	0.774484	0.374286	0.547327	0.549605	0.593222	0.676717	0.871199	0.274334
Novel_013142	XLOC_13992.	transcript	0	0	0.157387	0.041326	0.191388	0.139763	0.232759	0.05612	0.042241
Novel_013143	XLOC_13992.	transcript	0.121321	0	0.046485	0.063411	0.07761	0.086229	0.23765	0.113412	0.063491
Novel_013144	XLOC_13992.	transcript	0.019205	0	0.132761	0.065034	0.048809	0.054811	0.242531	0.165233	0
Novel_013145	XLOC_13992.	transcript	0.083925	0.023685	0.060427	0.023952	0.17122	0.025631	0.183812	0.102103	0.016934
Novel_013146	XLOC_13993.	transcript	0.080535	0.000329	0.347291	0.084311	0.016589	0.089381	0.248106	0.004508	0.01998
Novel_013147	XLOC_13993.	transcript	0	0	0	0.136123	0	0	0	0	0
Novel_013148	XLOC_13993.	transcript	0.105604	0.055428	0	0.013729	0.129244	0.080624	0.162727	0.088107	0.090994
Novel_013149	XLOC_13993.	transcript	0.028345	0.123907	0	0.129373	0.365534	0.091107	0	0.291926	0.073101
Novel_013150	XLOC_13996.	transcript	0.277012	0.137033	0	0	0	0	0.062277	0.127136	0
Novel_013151	XLOC_13998.	transcript	0.264225	1.592056	0.167667	0.20316	0.209076	0.244053	0.279229	0.285396	0.15592
Novel_013152	XLOC_14001.	transcript	0.438954	0.50742	0.209376	0.145739	0.018148	0.39934	0.009161	0.041673	0.33513
Novel_013153	XLOC_14001.	transcript	0	0.318541	0.483691	0	0	0	0.01121	0	0
Novel_013154	XLOC_14001.	transcript	0	0	0	0	0	0	0.368171	0.062073	0
Novel_013155	XLOC_14019.	transcript	0.115391	0.160368	0.080469	0.13264	0.123057	0.061894	0.089885	0.085045	0.045635
Novel_013156	XLOC_14019.	transcript	0	0	0	0	0.034685	0	0	0	0
Novel_013157	XLOC_14020.	transcript	0	0.026578	0	0	0	0	0.007915	0.00612	0
Novel_013158	XLOC_14020.	transcript	0	0	0	0	0	0	0.006274	0	0
Novel_013159	XLOC_14020.	transcript	0	0	0	0	0	0	0	0	0
Novel_013160	XLOC_14021.	transcript	0.273215	0.140631	1.171349	0.053626	0.943079	1.035279	1.213607	0.686888	1.024642
Novel_013161	XLOC_14021.	transcript	0	0	0	0	0	0	0	0	0
Novel_013162	XLOC_14021.	transcript	0	0	0	0	0	0	0	0	0
Novel_013163	XLOC_14021.	transcript	0	0.658205	0	0	0	0	0	0.315857	0
Novel_013164	XLOC_14021.	transcript	0.900928	0.010478	0	1.095805	0.002331	0	0.055873	0.122406	0
Novel_013165	XLOC_14030.	transcript	0.242719	0.596667	0.35913	0.168299	0.197504	0.485204	0.386861	0.373794	0.249438
Novel_013166	XLOC_14030.	transcript	1.056985	0.595803	0.98452	0.537141	0.805739	0.794822	0.407906	0.984926	0.328645
Novel_013167	XLOC_14030.	transcript	6.014254	3.071961	7.766266	12.30311	7.285969	5.75156	8.075616	11.14281	7.103029
Novel_013168	XLOC_14035.	transcript	0.050493	0	0.155173	0	0.084635	0.094093	0.477726	0.097397	0.805683
Novel_013169	XLOC_14047.	transcript	0.009737	0	0.003915	0	0	0	0	0	0.004607
Novel_013170	XLOC_14047.	transcript	0	0	0	0	0	0	0.030904	0	0
Novel_013171	XLOC_14047.	transcript	0	0	0	0	0	0	0	0	0
Novel_013172	XLOC_14047.	transcript	0.38053	0.476127	0.313779	0.341049	0.402042	0.358753	0.477862	0.444406	0.202096
Novel_013173	XLOC_14047.	transcript	0	0	0	0	0	0	0	0	0
Novel_013174	XLOC_14048.	transcript	0.856835	2.920815	0.483078	0.368019	0.52662	0.640392	1.200888	0.713523	1.900662

Novel_013175	XLOC_14049.	transcript	0.283244	0.293453	0.020702	0.516505	0	0.139929	0.675013	0.621924	0.085689
Novel_013176	XLOC_14049.	transcript	0	0	0	0	0	0	0.227688	0.115036	0
Novel_013177	XLOC_14049.	transcript	0.516833	0.62875	0.458003	0.453399	0.573143	0.471957	0.675777	0.545846	0.663093
Novel_013178	XLOC_14050.	transcript	0.529409	0.527795	1.263214	1.146083	0.956014	0.950417	0.97842	0.750607	1.137796
Novel_013179	XLOC_14050.	transcript	0.398542	0	0.008743	0	0.018055	0	0	0	0.332081
Novel_013180	XLOC_14051.	transcript	0.120024	0.145015	0.170397	0.137529	0.117818	0.112468	0.280172	0.233376	0.092476
Novel_013181	XLOC_14051.	transcript	0.181068	0.190874	0.201087	0.177566	0.186286	0.200579	0.453699	0.21705	0.459208
Novel_013182	XLOC_14053.	transcript	1.046487	1.377266	1.39157	0.965127	1.488107	0.849544	1.628165	1.138347	1.004157
Novel_013183	XLOC_14053.	transcript	0.340447	0.004703	0.342155	0.223037	0.469521	0.276855	0	0.029864	0.241016
Novel_013184	XLOC_14053.	transcript	0.332297	0.211729	0	0.162134	0	0.41987	0.729406	0.289195	0
Novel_013185	XLOC_14054.	transcript	0	0	0	0	0	0	0	0	0
Novel_013186	XLOC_14055.	transcript	1.181146	1.03742	1.00885	0.953595	0.839455	1.148836	1.010838	0.799601	0.543539
Novel_013187	XLOC_14057.	transcript	0	0.008769	0.018171	0.04925	0.472973	0.013032	0	0	0.027175
Novel_013188	XLOC_14057.	transcript	0.570514	0.622388	0.823377	0.94036	0.670108	0.967054	1.153964	1.001387	1.266606
Novel_013189	XLOC_14057.	transcript	0	0	0	0	0	0	0	0	0
Novel_013190	XLOC_14058.	transcript	0.593162	0.549719	0.752475	0.540472	0.734501	0.746367	1.029539	0.963866	0.502479
Novel_013191	XLOC_14058.	transcript	0	0.009293	0	0.014497	0	0.030212	0	0	0.020623
Novel_013192	XLOC_14058.	transcript	0	0	0	0	0.016239	0	0.067684	0	0
Novel_013193	XLOC_14060.	transcript	0	0.977784	0	0.020156	0.033534	0	0.034234	0.040436	0.097284
Novel_013194	XLOC_14060.	transcript	0.052707	0.029022	0.031732	0.266226	0	0.123766	0.013918	0.127348	0.024459
Novel_013195	XLOC_14063.	transcript	1.206166	1.087468	2.488007	2.111861	1.930066	1.870975	1.942669	2.375727	2.391956
Novel_013196	XLOC_14064.	transcript	0.364051	0.162321	0	0	0.504572	0.085856	0.495427	0.650764	0.242542
Novel_013197	XLOC_14064.	transcript	0.16989	0.341306	0.415574	0.562483	0.302362	0.316524	0.510287	0.176731	0.349402
Novel_013198	XLOC_14065.	transcript	0.03146	0	0.040854	0.04557	0.084356	0.018979	0.229261	0.076672	0.078855
Novel_013199	XLOC_14075.	transcript	0.121258	0.131838	0.132815	0.146718	0.158613	0.18341	0.261754	0.295253	0.041154
Novel_013200	XLOC_14075.	transcript	0	0	0	0.018095	0	0	0.299467	0	0
Novel_013201	XLOC_14075.	transcript	0.262042	0.214854	0.150971	0.105186	0.174682	0.286169	0.258936	0.19524	0.165345
Novel_013202	XLOC_14076.	transcript	0.359214	0.117316	0.294139	0.43487	0.464457	0.257842	0.823154	0.366406	0.358441
Novel_013203	XLOC_14076.	transcript	0.012277	0.017227	0.107666	0.057168	0	0.036834	0.044306	0.058553	0.062668
Novel_013204	XLOC_14076.	transcript	0.600046	0.480487	0.688137	0.417615	0.72157	0.610209	0.837831	0.515806	0.396434
Novel_013205	XLOC_14086.	transcript	0.145232	0.209989	0.080045	0.112562	0.191883	0.088156	0.228336	0.190387	0.182663
Novel_013206	XLOC_14087.	transcript	1.279474	1.101965	1.62295	1.503064	1.522931	1.625903	1.539891	1.274294	1.958629
Novel_013207	XLOC_14090.	transcript	0.135302	0.065907	0.315112	0.051546	0.36439	0.040259	0.138121	0.104052	0.169189
Novel_013208	XLOC_14092.	transcript	0	0	0	0.099921	0	0.053254	0.1098	0	0
Novel_013209	XLOC_14092.	transcript	0.128562	0	0	0	0	0	0.221632	0.004162	0
Novel_013210	XLOC_14092.	transcript	0	0.177393	0.232469	0.030001	0.24821	0.159365	0	0.230655	0.143173
Novel_013211	XLOC_14092.	transcript	0.048615	0.740848	0.962315	0.997348	0.249102	1.091052	0.070239	1.149959	1.144678
Novel_013212	XLOC_14092.	transcript	0.248778	0.114531	0.278993	0.296891	0.283485	0.187105	0.348827	0.432791	0.204352

Novel_013213	XLOC_14093.	transcript	0.07804	0.37679	0.237918	0.04358	0.032947	0.439367	0.261021	0.280565	0.279005
Novel_013214	XLOC_14094.	transcript	0.404135	0.158467	0.092993	0.410219	0.393102	0.325259	0.461287	0.234371	0.108385
Novel_013215	XLOC_14094.	transcript	1.163349	0.294917	0.435601	0.383023	0.127057	0.147751	1.542464	0.867457	0.734703
Novel_013216	XLOC_14094.	transcript	0	0.94458	0.445441	0.317653	0	0	0	0	0
Novel_013217	XLOC_14095.	transcript	0.454389	0.685642	0.441189	0.478066	0.614513	0.63835	0.658201	0.43239	0.381782
Novel_013218	XLOC_14095.	transcript	1.045781	0.364507	1.446505	1.016005	0.822149	0.774125	0.738519	0.773357	0.655074
Novel_013219	XLOC_14095.	transcript	0.000122	9.40E-05	0.065264	0.046467	0.532775	0.056641	0.062913	0.013635	0.068136
Novel_013220	XLOC_14095.	transcript	0.010568	0.013091	0.000282	0	0.170433	0.975203	0.001677	1.022066	0
Novel_013221	XLOC_14096.	transcript	0.208426	0.566287	0.415649	0.289122	0	0.229949	0.693622	0.167075	0.19065
Novel_013222	XLOC_14096.	transcript	0.181507	0.011808	0.272855	0.272826	0.523842	0.466501	0.01872	0.416525	0.28307
Novel_013223	XLOC_14097.	transcript	0.138065	0.168237	0.252568	0.054551	0.271733	0.113498	0.211617	0.135817	0.216221
Novel_013224	XLOC_14098.	transcript	0	0	0	0	0	0	0	0	0
Novel_013225	XLOC_14100.	transcript	0.477037	1.027925	0.640603	1.619238	1.61308	1.594933	1.124512	1.134975	1.28331
Novel_013226	XLOC_14100.	transcript	0.228356	0.161486	0.247151	0.211948	0.360099	0.266027	0.235376	0.214848	0.253114
Novel_013227	XLOC_14100.	transcript	0.136743	0.112047	0.233074	0.253724	0.13816	0.213377	0.338781	0.088533	0.194565
Novel_013228	XLOC_14100.	transcript	0	0.087519	0.016138	0.128355	0.245871	0	0.049387	0	0.134343
Novel_013229	XLOC_14102.	transcript	0.429756	0.523897	0.408054	0.301167	0.360758	0.450527	0.70466	0.410431	0.355501
Novel_013230	XLOC_14102.	transcript	2.685725	1.496889	1.804567	1.813785	0.185938	2.648723	1.43867	1.719809	1.330224
Novel_013231	XLOC_14102.	transcript	0.089957	0.058756	0.074866	0.009936	0.023847	0.036956	0.091895	0.068753	0
Novel_013232	XLOC_14102.	transcript	0.51406	0.163808	0.381946	0.53085	0.329601	0.782027	0.389126	0.781533	0.478
Novel_013233	XLOC_14102.	transcript	0	0	0	0	0	0	0.327564	0	0
Novel_013234	XLOC_14103.	transcript	0.368474	0.992095	0.184817	0.446226	0.417485	0.187716	0.225607	0.385139	0.502528
Novel_013235	XLOC_14103.	transcript	0.075319	0	0	0.106205	0	0.219701	0.245876	0.248025	0
Novel_013236	XLOC_14103.	transcript	0	0	0	0	0	0.004116	0	0	0
Novel_013237	XLOC_14103.	transcript	0	0	0.122354	0	0	0.061542	0.067377	0	0
Novel_013238	XLOC_14103.	transcript	0.022714	0.00055	0.174205	0.001014	0	0.001447	0	0.002967	0
Novel_013239	XLOC_14104.	transcript	0.13234	0.308549	0.136991	0.176593	0.156319	0.13968	0.234167	0.115239	0.107355
Novel_013240	XLOC_14104.	transcript	0.024898	0	0.002337	0	0.026577	0.040542	0.024068	0.206547	0
Novel_013241	XLOC_14104.	transcript	0	0	0	0	0	0	0	0	0
Novel_013242	XLOC_14104.	transcript	0.014603	0	0	0	0.03255	0.010606	0	0.021457	0.016057
Novel_013243	XLOC_14105.	transcript	0.611214	1.368335	0.658565	0.538704	0.726221	0.47021	1.190176	0.74901	1.082928
Novel_013244	XLOC_14105.	transcript	0.05405	0.099847	0.055939	0.098403	0	0.078504	0.080472	0.071247	0
Novel_013245	XLOC_14106.	transcript	0.82318	0.193691	0.836621	0.765561	0.740397	0.706392	1.414151	0.847034	0.626209
Novel_013246	XLOC_14109.	transcript	0.500454	0.665619	0.237187	0.527337	0.534827	0.527386	0.843634	0.680316	0.486865
Novel_013247	XLOC_14109.	transcript	0	0	0.374254	0	0	0	0	0	0
Novel_013248	XLOC_14109.	transcript	0.064676	0.114715	0.016043	0.076307	0.058095	0.113193	0.106785	0.090261	0.019118
Novel_013249	XLOC_14109.	transcript	0.020061	0	0	0.23475	0	0	0.442261	0.044038	0
Novel_013250	XLOC_14109.	transcript	0.026077	0.126815	0.316993	0	0.227951	0.217966	0	0.177922	0.277739

Novel_013251	XLOC_14109.	transcript	0.160556	0.116093	0.004858	0.045232	0.189223	0.103774	0.033378	0.108734	0
Novel_013252	XLOC_14113.	transcript	0	0.055168	0	0.023276	0.080622	0.044329	0.03127	0.048079	0.007683
Novel_013253	XLOC_14113.	transcript	0.520569	0.075179	0.327374	0.502732	0.445832	0.70908	0.045593	0.038392	0.496637
Novel_013254	XLOC_14113.	transcript	0	0	0	0.005282	0.007175	0.031576	0	0	0
Novel_013255	XLOC_14113.	transcript	0	1.027819	0.3742	0	0	0	0.885156	0.845345	0
Novel_013256	XLOC_14113.	transcript	0.020389	0.019405	0	0	0	0	0.006611	0.001029	0.040969
Novel_013257	XLOC_14117.	transcript	0.356107	0.008308	0.036286	0.362344	0.394708	0.26859	0.220498	0.238478	0.452922
Novel_013258	XLOC_14117.	transcript	0.013625	0.178944	0.387459	0.001584	0	0.073437	0.004577	0.006555	0
Novel_013259	XLOC_14117.	transcript	0	1.159875	0	0	0	0	0.253767	0.535628	0
Novel_013260	XLOC_14124.	transcript	0.200509	0.117308	0	0.097445	0	0.105269	0.105827	0.01667	0.086263
Novel_013261	XLOC_14128.	transcript	2.041605	3.132974	1.31501	0.604622	1.150148	1.474334	1.686398	1.009011	0.604595
Novel_013262	XLOC_14129.	transcript	0	0	0	0	0.051563	0	0	0	0.011612
Novel_013263	XLOC_14129.	transcript	0	0.017812	0.017386	0.016954	0.022712	0	0.006618	0.009963	0
Novel_013264	XLOC_14129.	transcript	0	0.094116	0.171901	0.444279	0.14217	0	0.070655	0.041365	0
Novel_013265	XLOC_14129.	transcript	0.099898	0.119252	0.068784	0	0.124093	0.119179	0.194076	0.224124	0.093652
Novel_013266	XLOC_14131.	transcript	0.322574	0.5486	0.357424	0.047547	0.257221	0.436143	0.446479	0.13366	0.380287
Novel_013267	XLOC_14131.	transcript	0	0	0	0.547629	0	0	0	0.475747	0
Novel_013268	XLOC_14131.	transcript	0	0	0	0	0	0	0	0	0
Novel_013269	XLOC_14132.	transcript	0	0	0	0.400942	0.447693	0	0.565328	0.223342	0.926147
Novel_013270	XLOC_14132.	transcript	0.233278	0.148567	0.487677	0	0	0.537497	0	0	0
Novel_013271	XLOC_14141.	transcript	0.303491	0.169738	0.276136	0	0.262722	0.418373	0.384363	0.23476	0
Novel_013272	XLOC_14141.	transcript	0	0.104207	0	0.122526	0	0	0	0.006339	0.292373
Novel_013273	XLOC_14145.	transcript	0.306342	0.730769	0.311364	0.20761	0.182432	0.402415	0.282185	0.218229	0.202387
Novel_013274	XLOC_14146.	transcript	0.086002	0.03948	0	0.020617	0	0.224458	0.082093	0.070376	0
Novel_013275	XLOC_14151.	transcript	0.430885	0.067683	0.246975	1.270273	0	0.43012	0	0	0.291234
Novel_013276	XLOC_14151.	transcript	0	0	0.209943	0.524542	0.64868	0	0.562276	0.898777	0
Novel_013277	XLOC_14155.	transcript	0	0	0.191052	0	0	0	0	0	0
Novel_013278	XLOC_14155.	transcript	0	0.443232	0	0	0	0	0	0	0
Novel_013279	XLOC_14155.	transcript	0	0.677143	0.277032	0	0.23491	0	0	0	0
Novel_013280	XLOC_14155.	transcript	0.373899	0	0	0.733896	0.136724	0.551747	0.461292	0.578466	0.380046
Novel_013281	XLOC_14155.	transcript	0.781985	0.599859	0.968192	1.560723	0.920108	1.036796	1.143819	1.143185	0.792052
Novel_013282	XLOC_14156.	transcript	1.231328	1.597721	0.824388	1.447288	1.355781	1.31246	1.035584	0.864686	1.405236
Novel_013283	XLOC_14156.	transcript	5.384438	1.705649	5.727571	5.472342	5.432065	8.37621	5.611382	7.599438	6.282112
Novel_013284	XLOC_14156.	transcript	0.081256	0	0.03711	0.049511	0.123562	0	0.085217	0.042006	0.590261
Novel_013285	XLOC_14171.	transcript	0	0.0796	0.011826	0.171992	0.061837	0.212664	0.159943	0.085312	0.32871
Novel_013286	XLOC_14182.	transcript	0.107434	0	0	9.70E-05	0.006547	0.000649	0.058546	0	0.038035
Novel_013287	XLOC_14182.	transcript	0	0.004401	0.362285	0.182216	0.017482	0.023139	0.000469	0.01745	0
Novel_013288	XLOC_14182.	transcript	0	0	0	0	0	0	0	0.001481	0.005179

Novel_013289	XLOC_14182…	transcript	0	0	0	0	0.059996	0	0.37925	0.033383	0
Novel_013290	XLOC_14182…	transcript	0.007203	0.030542	0.18553	0.126709	0.021336	0.031027	0.003705	0	0.005783
Novel_013291	XLOC_14182…	transcript	0	0.001511	0	0	0.013512	0	0.002013	0.007742	0
Novel_013292	XLOC_14182…	transcript	0.025921	0	0.464994	0.21876	0.011835	0	0.004846	0	0
Novel_013293	XLOC_14188…	transcript	0	0.039244	0	0.109958	0	0	0.100849	0.138522	0
Novel_013294	XLOC_14189…	transcript	0.008606	0	0.026017	0	0.292487	0.045724	0	0	0
Novel_013295	XLOC_14189…	transcript	0.121787	0.888073	0.295143	0.414581	0.070883	0.333472	0.252242	0.382766	0.067375
Novel_013296	XLOC_14192…	transcript	0	0.028286	0.073547	0.039043	0	0.224823	0.079436	0.118157	0.110417
Novel_013297	XLOC_14198…	transcript	0.08126	0	0.083741	0	0.156965	0	0	0	0.107206
Novel_013298	XLOC_14198…	transcript	0.390063	0.268933	0.208615	0.005112	0.229598	0.428481	0.362711	0.301674	0.112146
Novel_013299	XLOC_14198…	transcript	0	0	0.112741	0.336779	0	0	0	0.057675	0
Novel_013300	XLOC_14198…	transcript	0.145482	0	0.118554	0.005022	0.02552	0.002839	0.163596	0.064131	0.218129
Novel_013301	XLOC_14198…	transcript	0.350149	0.279021	0.315452	0.151204	0.354011	0.390197	0.307355	0.27233	0.183302
Novel_013302	XLOC_14198…	transcript	0.004041	0	0.062562	0	0	0	0.0389	0.084154	0
Novel_013303	XLOC_14199…	transcript	0.281703	1.706551	1.57545	2.080219	1.135132	1.22672	1.197865	1.495422	0.788703
Novel_013304	XLOC_14201…	transcript	0.0461	0.092948	0.267193	0.142205	0.14011	0.157522	0.232932	0.34809	0.098189
Novel_013305	XLOC_14202…	transcript	0.436539	0.074865	0.431954	0.112993	0.763795	0.504029	0.82158	0.408116	0.636042
Novel_013306	XLOC_14203…	transcript	3.23988	1.037115	4.631297	4.639962	4.599885	5.506407	4.702903	5.193322	5.927229
Novel_013307	XLOC_14208…	transcript	0.163952	0.757345	0.057949	0.068909	0.018687	0.065806	0.094149	0.030872	0
Novel_013308	XLOC_14208…	transcript	0.331936	0.405384	0.168406	0.117641	0.125592	0.131042	0.183154	0.123297	0.061475
Novel_013309	XLOC_14208…	transcript	0	0	0	0.007896	0	0	0	0	0.002283
Novel_013310	XLOC_14208…	transcript	0.164465	0.029257	0.063562	0.080711	0.091886	0	0.131273	0.03761	0.039588
Novel_013311	XLOC_14208…	transcript	0	0.018331	0.012607	0.018532	0.019068	0.000136	0.008717	0.004902	0
Novel_013312	XLOC_14208…	transcript	0	0	0.098413	0.052641	0	0.11658	0	0	0.172186
Novel_013313	XLOC_14208…	transcript	0.275167	0	0	0	0.368021	0	0.103062	0	0.287942
Novel_013314	XLOC_14208…	transcript	0.057837	0.022853	0.035769	0	0.053361	0.042709	0.076869	0.039275	0.004894
Novel_013315	XLOC_14208…	transcript	0	0	0	0	0	0	0	0.069021	0.002308
Novel_013316	XLOC_14208…	transcript	0.018706	0.126719	0.133839	0.22912	0.014577	0.034048	0	0.086401	0.01067
Novel_013317	XLOC_14208…	transcript	0.020203	0.057529	0.072461	0.015819	0.009595	0.071372	0	0.056637	0.000318
Novel_013318	XLOC_14208…	transcript	0.056285	0.026067	0	0	0.015317	0.031277	0.322902	0.031557	0
Novel_013319	XLOC_14208…	transcript	0.003285	0.108266	0.493833	0.146774	0.035565	0.103121	0	0.109521	0.009071
Novel_013320	XLOC_14208…	transcript	0	0	0.511998	0.83703	0.388494	0.178107	0.487569	0.471212	0.28457
Novel_013321	XLOC_14208…	transcript	0.004871	0	0.098082	0	0.006648	0.054254	0	0.002524	0
Novel_013322	XLOC_14208…	transcript	0.214315	0	0	0	0.237445	0	0.137668	0.445737	0
Novel_013323	XLOC_14208…	transcript	0	0	0.049003	0.05128	0.055737	0.111748	0.185937	0.059059	0.029706
Novel_013324	XLOC_14208…	transcript	0.174666	0	0.091069	0.061892	0.062025	0.06606	0.119043	0.046077	0.016693
Novel_013325	XLOC_14208…	transcript	0	0	0	0	0	0	0	0.099663	0
Novel_013326	XLOC_14209…	transcript	0.263525	0.094664	0.16252	0.195391	0.313	0.270468	0.161743	0.253572	0.152199

Novel_013327	XLOC_14212.	transcript	1.125391	0.092573	0.028319	0.532497	0.16864	0.77356	0.31239	0.008487	0.548577
Novel_013328	XLOC_14212.	transcript	0	0	0	0.289757	0	0	0.221857	0.199793	0
Novel_013329	XLOC_14212.	transcript	0.01903	0.862039	0.73212	0	0.918257	0	0.480821	0.763246	0
Novel_013330	XLOC_14212.	transcript	0.07373	0	0.260211	0.150876	0	0.293661	0.048774	0	0.320037
Novel_013331	XLOC_14212.	transcript	0.401643	0.824371	0.268889	0.296479	0.18951	0.254848	0.403993	0.32138	0.255656
Novel_013332	XLOC_14212.	transcript	0.33878	0.122677	0.080432	0.174818	0.110645	0.138982	0.32993	0.189211	0.09945
Novel_013333	XLOC_14212.	transcript	0	0	0.05187	0.028918	0.089214	0	0	0.013656	0.040311
Novel_013334	XLOC_14213.	transcript	1.525922	0.320876	2.565007	2.510929	2.815729	2.116595	2.727077	2.535888	1.069502
Novel_013335	XLOC_14215.	transcript	0.125218	0	0.090563	0.104422	0.127218	0	0	0.138727	0
Novel_013336	XLOC_14215.	transcript	0.44379	0	0.17313	0.079854	0.715914	0.126155	0.452709	0.146737	0
Novel_013337	XLOC_14222.	transcript	0.050254	0	0	0.015861	0.036889	0.138798	0.025318	0.061235	0.031759
Novel_013338	XLOC_14223.	transcript	0	0	0.48559	0	0	0	0	0	0.070363
Novel_013339	XLOC_14223.	transcript	0.635528	0.214752	0.363578	0.166177	0.294366	0.451909	0.39405	0.077525	0.618267
Novel_013340	XLOC_14223.	transcript	0.111275	0	0	0.147647	0.224993	0	1.460657	0	0
Novel_013341	XLOC_14224.	transcript	0.02424	0.492494	0.90058	0.977839	0.622346	0.516042	0.64239	0.717858	0.521868
Novel_013342	XLOC_14224.	transcript	0.776405	0.744636	0.55592	1.172356	1.110562	1.428837	0.7237	0.882379	1.823291
Novel_013343	XLOC_14226.	transcript	0.079618	0.874916	0.262412	0.231343	0.269283	0.237414	0.173749	0.265779	0.073442
Novel_013344	XLOC_14226.	transcript	0	0.132351	0	0	0	0.102392	0.032691	0.039865	0
Novel_013345	XLOC_14226.	transcript	0.044965	0.710089	0.248227	0.19226	0.132221	0.258149	0.134321	0.233054	0.043298
Novel_013346	XLOC_14226.	transcript	0.024034	0	0	0	0.043591	0	0.024748	0	0
Novel_013347	XLOC_14226.	transcript	0.001527	0.021921	0.011942	0	0	0	0.029289	0	0
Novel_013348	XLOC_14226.	transcript	0.189651	0.281812	0.333232	0.315541	0	0.240104	0.41237	0.263718	0.226674
Novel_013349	XLOC_14226.	transcript	0	0	0	0	0.287337	0	0	0	0
Novel_013350	XLOC_14226.	transcript	0.296809	0.241626	0.106257	0.13104	0.236995	0.220694	0.450821	0.281378	0.160848
Novel_013351	XLOC_14226.	transcript	0.242701	0.281127	0.090507	0.048649	0.066897	0.140792	0.181647	0.316559	0.054397
Novel_013352	XLOC_14226.	transcript	0.049323	0.060154	0.275485	0.051694	0.067239	0.115044	0.180159	0.06556	0.018552
Novel_013353	XLOC_14229.	transcript	0.001517	0.356905	0	0	0.002033	0	0.001492	0	0
Novel_013354	XLOC_14229.	transcript	0.418372	0.392743	0.262227	0.280195	0.280995	0.279625	0.460263	0.322008	0.179737
Novel_013355	XLOC_14229.	transcript	0	0	0	0	0	0	0	0	0.112358
Novel_013356	XLOC_14229.	transcript	0	0	0	0	0.014069	0	0	0	0
Novel_013357	XLOC_14240.	transcript	0	1.125767	0.317825	0	0	0.856642	0.471218	0	0.711895
Novel_013358	XLOC_14240.	transcript	0	0	0	0	0	0	0.87366	0	0
Novel_013359	XLOC_14240.	transcript	0	0.019681	0.113609	0	0	0.010028	0	0.281786	0
Novel_013360	XLOC_14240.	transcript	0.233371	0	0.113576	0.459956	0.486507	0.111395	0.106193	0.16158	0.165002
Novel_013361	XLOC_14240.	transcript	0	0	0	0	0	0	0	0.05522	0
Novel_013362	XLOC_14244.	transcript	0	0.561608	0	0	0	0	0	0	0
Novel_013363	XLOC_14245.	transcript	0.146689	0	0.25514	0.266124	0.202726	0.038459	0	0.000527	0
Novel_013364	XLOC_14245.	transcript	0.37859	0	0	0.011127	0	0	0	0.28518	0

Novel_013365	XLOC_14245.	transcript	0	0.073289	0	0.287033	0	0.27488	1.093628	0.287643	0.259979
Novel_013366	XLOC_14245.	transcript	0.622	0	0.418692	0	0.548578	0.01387	0	0.167281	0
Novel_013367	XLOC_14247.	transcript	0.928671	0.555082	1.242916	2.650052	1.782604	1.80242	1.251855	2.576757	1.088355
Novel_013368	XLOC_14247.	transcript	0.477069	0.27845	1.07861	0.529922	0.956416	0.901471	0.888751	0.591556	1.118304
Novel_013369	XLOC_14249.	transcript	0.212481	0.083583	0.306334	0.150533	0.391866	0.166354	0.564525	0.411553	0.317592
Novel_013370	XLOC_14250.	transcript	0.34055	0.004869	0.128737	8.80E-05	0.16307	0.067951	0.066907	0.162392	0.00136
Novel_013371	XLOC_14250.	transcript	0	0	0	0.662246	0	0	0	0.232247	0
Novel_013372	XLOC_14250.	transcript	0.005861	0.146333	0.04056	0.239646	0.124094	0	0.303126	0.152596	0.059378
Novel_013373	XLOC_14250.	transcript	0	0.009313	0	0	0	0	0	0.060514	0
Novel_013374	XLOC_14250.	transcript	0	0	0.355786	0	0.55022	0.536372	0.605319	0.453296	0.647945
Novel_013375	XLOC_14250.	transcript	0.247956	0.012745	0.009953	0.033043	0.044487	0.141392	0.014954	0.015797	0.047651
Novel_013376	XLOC_14250.	transcript	0	0	0.721798	0.073381	0	0	0.002719	0	0
Novel_013377	XLOC_14253.	transcript	0.086256	0.023789	0.610046	0.229568	0.320004	0.215594	0.221685	0.212347	0.190685
Novel_013378	XLOC_14253.	transcript	0.708393	0	0	0.392115	0.650006	0.647153	0.266298	0.487264	0.284146
Novel_013379	XLOC_14253.	transcript	0.504661	0	0	0	0	0.408933	0	0	0
Novel_013380	XLOC_14253.	transcript	0.44228	0.135824	1.454837	0.605731	0.519426	0.447966	0.989735	0.730791	0.3732
Novel_013381	XLOC_14253.	transcript	0.162323	0.612321	0.280478	0.180129	0.240944	0.24104	0.244922	0.277101	0.203111
Novel_013382	XLOC_14256.	transcript	0.014918	0.014191	0.111671	0.033473	0.025265	0	0	0.030253	0.012194
Novel_013383	XLOC_14256.	transcript	0.067408	0	0.176741	0.113894	0.061943	0.114811	0.045758	0.14	0.242209
Novel_013384	XLOC_14256.	transcript	0.064472	0	0	0	0	0	0	0	0
Novel_013385	XLOC_14256.	transcript	0.06279	0.128515	0.036353	0.017746	0.013966	0	0.120451	0.012521	0.018282
Novel_013386	XLOC_14256.	transcript	0	0.09697	0	0.439439	0	0.337333	0.080129	0.372399	0
Novel_013387	XLOC_14256.	transcript	0	0.161708	0.086225	0	0.250748	0.191162	0.422855	0.299435	0.353293
Novel_013388	XLOC_14262.	transcript	0	0	0	0.082705	2.017395	0	0.011074	0.223479	0
Novel_013389	XLOC_14262.	transcript	0.82337	0	0	0	0	0	0	0	0
Novel_013390	XLOC_14262.	transcript	0	0	0	0	0	0	0	0	0
Novel_013391	XLOC_14262.	transcript	0	0.131818	0	0.001067	0.003433	0	0.191866	0.035055	0.934229
Novel_013392	XLOC_14262.	transcript	0	0	0	0	0	0	0	0	0
Novel_013393	XLOC_14262.	transcript	0	0	0	0	0	0	0.046458	0.00078	0
Novel_013394	XLOC_14262.	transcript	0	0	0	1.557855	0	0	0	1.042323	0
Novel_013395	XLOC_14262.	transcript	6.10E-05	0	1.554799	0.201605	0.246036	1.371044	0.446039	1.066392	0
Novel_013396	XLOC_14262.	transcript	0	0	0	0.002267	0	0	2.664181	0	0
Novel_013397	XLOC_14262.	transcript	0	0	0	0	0	0	0	0	0
Novel_013398	XLOC_14262.	transcript	0.179165	0	0	0	0	0	0	0	0
Novel_013399	XLOC_14264.	transcript	0.211299	0.078577	0.031425	0.146074	0.048029	0.040426	0.127906	0.159743	0.045124
Novel_013400	XLOC_14264.	transcript	0	0	0	0.422076	0	0	0.008597	0.023637	0
Novel_013401	XLOC_14265.	transcript	0.103409	0.377698	0.557971	0.602957	0.537187	0.620967	0.3905	0.808265	0.439778
Novel_013402	XLOC_14265.	transcript	0	0.02796	0	0.016054	0.07014	0.002542	0	0	0.367967

Novel_013403	XLOC_14265.	transcript	0.009894	0.868289	1.369734	0.074074	0.020498	0.030824	0.007323	0	0.019357
Novel_013404	XLOC_14265.	transcript	0	0.47807	0	0.480618	0	0	0.872595	2.118689	0
Novel_013405	XLOC_14265.	transcript	2.266431	0	0	0.545618	0	0	0	0	0
Novel_013406	XLOC_14265.	transcript	0.020542	0	0.152098	0	0.345385	0.521329	0.770248	0	0.388747
Novel_013407	XLOC_14265.	transcript	0.022936	0.680038	0	0.32278	1.031106	0.871819	0.429745	0	0.391156
Novel_013408	XLOC_14265.	transcript	0	0	0	0	0	0	0	0	0
Novel_013409	XLOC_14265.	transcript	0	0	0	0.134226	0	0.000411	0.411297	0.012847	0
Novel_013410	XLOC_14267.	transcript	0.383768	0.376381	0.289532	0.132826	0.230071	0.427168	0.290297	0.200252	0.240708
Novel_013411	XLOC_14267.	transcript	0	0	0	0.055703	0.115346	0	0.168738	0.227444	0
Novel_013412	XLOC_14269.	transcript	0.236647	0	0.062547	0	0	0	0.040046	0	0
Novel_013413	XLOC_14270.	transcript	0	2.00E-04	0.060758	0.100772	0.123566	0	0	0.212368	0
Novel_013414	XLOC_14270.	transcript	0	0	0.103289	0.70745	0.215091	0.447197	0.544404	0.180247	0.326048
Novel_013415	XLOC_14270.	transcript	0.158153	0	0.249274	0.760391	0.554505	0	0.125921	0.588747	0.114765
Novel_013416	XLOC_14270.	transcript	0	0.117483	0.197725	0.044996	0.011955	0.18102	0.02258	0.008124	0
Novel_013417	XLOC_14274.	transcript	0.004223	0.131954	0.004823	0.008102	0.008504	0.016532	0.010717	0.05988	0.023462
Novel_013418	XLOC_14278.	transcript	0	0.280064	0.1343	0.072509	0	0	0.128335	0.015131	0
Novel_013419	XLOC_14279.	transcript	0	0.118497	0.081921	0.126024	0	0	0.485784	0.394771	0.28438
Novel_013420	XLOC_14279.	transcript	0	0.265193	0.163624	0.054074	0	0	0.143206	0.073782	0.010418
Novel_013421	XLOC_14279.	transcript	0	0.013737	0	0.022371	0.125603	0.014774	0.030789	0	0.063107
Novel_013422	XLOC_14279.	transcript	0	0	0.009963	0	0.191969	0	0	0	0
Novel_013423	XLOC_14279.	transcript	0.267237	0.479845	0.066444	0.058257	0.049865	0.216378	0.166966	0.082656	0.055174
Novel_013424	XLOC_14283.	transcript	0	0	0	0.150194	0	0.162874	0	0.151954	0
Novel_013425	XLOC_14283.	transcript	0.111299	0.343197	0.07447	0	0.261453	0	0.235136	0	0.128005
Novel_013426	XLOC_14283.	transcript	0.035346	0.052813	0	0.001384	0.085959	0.160338	0.020683	0.105476	0.165938
Novel_013427	XLOC_14284.	transcript	0.434537	0.230779	0.343193	0.072261	0.047818	0.191208	0.160345	0.123206	0.120137
Novel_013428	XLOC_14284.	transcript	0.26942	0.329114	0	0.12537	0.183687	0.137327	0.156954	0.172279	0.213798
Novel_013429	XLOC_14284.	transcript	0	0	0	0.066156	0.155674	0.097044	0.158989	0	0.171519
Novel_013430	XLOC_14284.	transcript	0.087564	0.197253	0.24158	0.068323	0.109313	0.160654	0.112181	0.166816	0.19986
Novel_013431	XLOC_14285.	transcript	0.008419	2.320934	0	0	0	0	0	0	0
Novel_013432	XLOC_14285.	transcript	0	2.201236	0	0	0	0	0	0	0
Novel_013433	XLOC_14287.	transcript	0	0	0	0.024956	0	0	1.54332	0.041137	0
Novel_013434	XLOC_14287.	transcript	0.801636	0.299262	0.663438	1.133966	0.782157	0.706723	0.827062	1.554465	0.251555
Novel_013435	XLOC_14287.	transcript	0.231421	0.397149	0.228484	1.123765	0.851578	0	0.237284	0.309382	0.05026
Novel_013436	XLOC_14293.	transcript	0.308257	0.311005	0.194472	0.431413	0.465666	0.332579	0.262461	0.337631	0.691757
Novel_013437	XLOC_14293.	transcript	0.525082	0.948312	0.84733	1.069124	0.672898	1.024882	1.025689	1.052143	0.835267
Novel_013438	XLOC_14293.	transcript	0.182805	0.710518	0.42649	0.684237	0.298241	0.496499	0.862475	0.452536	0.253887
Novel_013439	XLOC_14293.	transcript	0.46271	0.558355	0.462085	0.398327	0.928299	0.456416	0.494323	1.038585	0.396174
Novel_013440	XLOC_14301.	transcript	0	0	0.088569	0.012926	0.266156	0.403352	0	0	0

Novel_013441	XLOC_14301.	transcript	0.001968	0	0.133358	0	0.114992	0.107908	0	0	0
Novel_013442	XLOC_14301.	transcript	0	0.00184	0.233144	0.26181	0.11977	0.095514	0.001419	0	0
Novel_013443	XLOC_14301.	transcript	0.077654	0.001839	0.087065	0.090268	0.107428	0.086701	0.315692	0.402171	0.208596
Novel_013444	XLOC_14301.	transcript	0	0	0.150451	0.048686	0.124436	0.108035	0.180672	0.424997	0.457023
Novel_013445	XLOC_14301.	transcript	0.570668	0.216809	0.188782	0.328067	0.126477	0.105068	0.280758	0.140237	0.108234
Novel_013446	XLOC_14301.	transcript	0	0.09216	0.084712	0.232466	0.099759	0.10218	0.1714	0.122051	0.113139
Novel_013447	XLOC_14301.	transcript	1.733682	0	0	0	0.124922	0	0.264573	0.197432	0
Novel_013448	XLOC_14301.	transcript	1.653912	0.73813	4.263762	3.207332	0.632958	0.94704	0.215504	5.342512	1.655971
Novel_013449	XLOC_14324.	transcript	0.73772	0.548745	0.708967	0.762667	0.941826	0.427698	0.374084	1.601887	0.571774
Novel_013450	XLOC_14325.	transcript	0	0	0	0	0	0.156992	0	0	0
Novel_013451	XLOC_14325.	transcript	0	0	0	0	0	0	0.089198	0.02386	0
Novel_013452	XLOC_14325.	transcript	0.337192	0.05251	0.215016	0.001425	0.180982	0	0.381004	0.175131	0
Novel_013453	XLOC_14325.	transcript	0.001201	0.043082	0.002773	0.020645	0.002875	0.010986	0.002202	0.002241	0.026051
Novel_013454	XLOC_14325.	transcript	0.227977	0.089256	0.166561	0.246624	0.232365	0.324954	0.272096	0.115166	0.124059
Novel_013455	XLOC_14327.	transcript	0.342001	0.080217	0.076133	0.059175	0.125318	0.065843	0.252026	0.115631	0.01533
Novel_013456	XLOC_14327.	transcript	0	0	0.09743	0	0.009048	0.039091	0.02444	0	0.182957
Novel_013457	XLOC_14327.	transcript	0	0	0	0	0	0	0.122157	0	0
Novel_013458	XLOC_14327.	transcript	0.187108	0	0.025751	0.286828	0.046996	0.040214	0.187094	0.074478	0.016299
Novel_013459	XLOC_14336.	transcript	0	0	0	0.016055	0.01668	0.042934	0.037117	0	0
Novel_013460	XLOC_14336.	transcript	0.15644	0.054916	0.314081	0.17894	0.321173	0.393723	0.316787	0.473159	0.217306
Novel_013461	XLOC_14336.	transcript	0	0	0	0.399308	0	0	0	0	0
Novel_013462	XLOC_14336.	transcript	0.352306	0	0	0	0	0	0	0	0
Novel_013463	XLOC_14336.	transcript	3.794162	0.688336	2.812469	3.470017	3.967808	3.588916	4.198811	1.976144	3.260019
Novel_013464	XLOC_14336.	transcript	0.016813	0	0.185213	0	0	0	0.031499	0.166517	0
Novel_013465	XLOC_14337.	transcript	0.238574	0.029236	0.263576	0.180799	0.293045	0.125951	0.100183	0.038157	0.180759
Novel_013466	XLOC_14337.	transcript	0	0	0	0	0.152992	0	0.203933	0	0
Novel_013467	XLOC_14337.	transcript	0.779565	0	0	0	0	0	0	0	0
Novel_013468	XLOC_14337.	transcript	0.460107	0.371925	1.345777	1.560078	1.067507	1.143432	1.176931	1.700727	1.140757
Novel_013469	XLOC_14337.	transcript	0	0	0.026779	0.011109	0.021248	0	0.016436	0.012813	0
Novel_013470	XLOC_14345.	transcript	0	0.016361	0.079393	0	0.03281	0.065303	0.255009	0.079584	0.202208
Novel_013471	XLOC_14345.	transcript	0.351751	0.293832	0.316446	0.182504	0.188298	0.006615	0.289218	0.111286	0.011382
Novel_013472	XLOC_14345.	transcript	0.282048	0.200101	0.262844	0.545364	0.445571	0.396519	0.253489	0.441797	0.568539
Novel_013473	XLOC_14345.	transcript	0.031038	0.128578	0.102926	0.178319	0.052965	0.058584	0.135554	0.143737	0.020174
Novel_013474	XLOC_14345.	transcript	0.295761	0	0.100604	0	0.105856	0.006448	0.332061	0.049801	0.294994
Novel_013475	XLOC_14348.	transcript	0	0	0	0.175219	0.115947	0.163995	0.082801	0.420424	0.202519
Novel_013476	XLOC_14348.	transcript	0.707937	0.801159	1.028259	1.281489	0.669492	1.219948	1.069475	0.903891	0.908739
Novel_013477	XLOC_14348.	transcript	0	0	0	0	0.389505	0	0	0.004607	0
Novel_013478	XLOC_14350.	transcript	1.438432	0.591622	0.937833	1.42895	0.815794	1.777574	0.684178	0.804783	1.570127

Novel_013479	XLOC_14350.	transcript	1.693231	3.010439	1.994998	2.817697	2.000229	3.797069	1.29232	0.105109	2.414358
Novel_013480	XLOC_14350.	transcript	0	0	0	0	0	0	0	0	0
Novel_013481	XLOC_14350.	transcript	0	0	0	0	0	0	0	1.90748	0
Novel_013482	XLOC_14352.	transcript	0.069246	0.062174	0	0.020384	0	0	0.281281	0.25509	0.111087
Novel_013483	XLOC_14353.	transcript	0.098073	0.46557	0.159887	0.06978	0.014219	0.127201	0.183247	0.022603	0.006861
Novel_013484	XLOC_14353.	transcript	0	0.094255	0	0	0	0	0	0	0
Novel_013485	XLOC_14353.	transcript	0	0	0.002531	0	0	0	0	0	0.008641
Novel_013486	XLOC_14353.	transcript	0	0	0	0	0	0	0	0.116224	0
Novel_013487	XLOC_14353.	transcript	0	0	0	0	0	0	0	0.14854	0
Novel_013488	XLOC_14353.	transcript	0	0.009681	0	0	0	0.009394	0.009302	0	0.002271
Novel_013489	XLOC_14353.	transcript	0.251447	0.233983	0.175944	0.117779	0	0.15422	0.132363	0	0
Novel_013490	XLOC_14353.	transcript	0	0	0	0.0368	0.331689	0	0	0	0.16214
Novel_013491	XLOC_14357.	transcript	0	0.63434	0.154818	0.030071	0.231163	0	0.008234	0.544956	0
Novel_013492	XLOC_14357.	transcript	0	0	0	0.473057	0	0	0	0	0
Novel_013493	XLOC_14357.	transcript	0.679993	0.471473	0.638021	0.002804	0.392777	0.624392	0.077655	0.169836	0.691789
Novel_013494	XLOC_14357.	transcript	0	0	0	0.258585	0	0	0	0	0
Novel_013495	XLOC_14357.	transcript	0	0	0	0	0	0	0.849728	0	0
Novel_013496	XLOC_14360.	transcript	0	0.028121	0.905072	0.014303	0	0.127078	0.419179	0	0
Novel_013497	XLOC_14360.	transcript	0.141082	0.017094	0.122981	0.349426	0.268562	0.14949	0.197057	0.214476	0.109249
Novel_013498	XLOC_14361.	transcript	0	0	0.068569	0	0	0	0	0	0
Novel_013499	XLOC_14361.	transcript	0	0.025006	0.017135	0	0.054487	0	0	0	0
Novel_013500	XLOC_14361.	transcript	0.168059	0.07804	0.057248	0.175549	0.178261	0.49488	0.270064	0.133403	0.125492
Novel_013501	XLOC_14361.	transcript	0.186991	0	0	0	0.253392	0.028922	0	0.272043	0
Novel_013502	XLOC_14361.	transcript	0	0.25983	0.319297	0.345454	0.111706	0	0.19821	0	0.555374
Novel_013503	XLOC_14366.	transcript	0	0.661552	0.540684	0.004721	0	0.004763	0	0	0.074954
Novel_013504	XLOC_14366.	transcript	0.581571	0	0	0.186836	0.475765	0.523507	0.148091	0.165541	0.236655
Novel_013505	XLOC_14366.	transcript	0	0	0.013904	0.234806	0.130341	0	0.507408	0.561891	0
Novel_013506	XLOC_14366.	transcript	0	0.235563	0	0	0	0	0.018477	0.035372	0
Novel_013507	XLOC_14367.	transcript	0.033217	0.090176	0.449375	0.219131	0.391596	0	0	0.256256	0
Novel_013508	XLOC_14367.	transcript	0.633272	0.256612	0.625362	0.462961	0.405382	0.311558	0.364527	0.551873	0.526751
Novel_013509	XLOC_14367.	transcript	0.30589	0.002457	0.18712	0.171246	0.372643	0.357243	0.104858	0.256466	0.113729
Novel_013510	XLOC_14367.	transcript	0	0	0.737734	0	0.011436	0	0.324039	0	0
Novel_013511	XLOC_14370.	transcript	0	0	0.407271	0	0	0	0	0	0.559014
Novel_013512	XLOC_14370.	transcript	0.749542	1.030267	1.086918	1.261523	1.162282	1.27027	1.654762	0.892491	0.927822
Novel_013513	XLOC_14370.	transcript	1.369694	1.950275	1.662168	1.732601	1.42675	1.598997	1.680373	1.961514	1.032856
Novel_013514	XLOC_14371.	transcript	0.150185	0.225566	0.118407	0.147962	0.106332	0.21712	0.332323	0.193919	0.271678
Novel_013515	XLOC_14374.	transcript	0.615634	0.64802	0.476108	0.405797	0.527606	0.42219	0.72386	0.518735	0.510342
Novel_013516	XLOC_14377.	transcript	0.205774	0.259181	0.327948	0.229679	0.27001	0.226394	0.239235	0.329561	0.156411

Novel_013517	XLOC_14377.	transcript	2.32556	3.089671	2.588208	3.627013	2.451091	3.211039	2.578268	3.685297	2.634935
Novel_013518	XLOC_14378.	transcript	0.101244	0	0.159401	0.082578	0.044543	0.152273	0.300606	0.219317	0.128491
Novel_013519	XLOC_14385.	transcript	0.302151	0.467701	0.46564	0.304956	0.413287	0.229141	0.498236	0.411592	0.290741
Novel_013520	XLOC_14390.	transcript	0	0.002507	0	0	0	0	0.093203	0	0.001732
Novel_013521	XLOC_14390.	transcript	0.028752	0.1854	0	0	0	0.059589	0.042668	0	0
Novel_013522	XLOC_14390.	transcript	0	0.089198	0	0	0	0	0.043848	0	0.186764
Novel_013523	XLOC_14390.	transcript	0	0.322305	0.352867	0.296297	0.479087	0	0.198199	0	0.13993
Novel_013524	XLOC_14390.	transcript	0.399614	0.093906	0	0	0	0.391278	0.079337	0.345697	0
Novel_013525	XLOC_14390.	transcript	0.112546	0.271686	0.193156	0.132275	0	0.035753	0.203563	0.045911	0.355056
Novel_013526	XLOC_14390.	transcript	0.164895	0.223012	0.109359	0.022622	0.230818	0	0.011705	0	0
Novel_013527	XLOC_14395.	transcript	0.058878	0.153063	0.064152	0.095195	0.071098	0.05595	0.089228	0.043076	0.051415
Novel_013528	XLOC_14397.	transcript	0.043589	0.197262	0.866953	0.052484	1.000459	0.07793	0.043295	0.103613	0.783372
Novel_013529	XLOC_14397.	transcript	0.017984	0.890421	0.14314	0.403432	0.125318	0.474577	0.810657	0.759181	0.04582
Novel_013530	XLOC_14397.	transcript	0.515155	0	0	0	0	0	0	0	0
Novel_013531	XLOC_14397.	transcript	0.675851	0.587452	0	0.354575	0	0	0.906887	0.596964	0
Novel_013532	XLOC_14397.	transcript	0.170232	0.267364	0.001927	0.365825	0	0	0	0	0
Novel_013533	XLOC_14398.	transcript	0.081703	0.258134	0	0.138393	0.144101	0.077362	0.03206	0.19442	0.07785
Novel_013534	XLOC_14399.	transcript	0	0.313433	0	0	0	0	0	0.028932	0
Novel_013535	XLOC_14399.	transcript	0.06685	0.213715	0	0.794654	0.316754	0.739822	0.15764	0.001839	0.479729
Novel_013536	XLOC_14399.	transcript	0.401129	0.727831	0.709931	0.133788	0.597941	0.127942	0.616125	0.831852	0.133992
Novel_013537	XLOC_14399.	transcript	0.083822	0.379481	0.05226	0.087067	0.026209	0.109673	0.097118	0.040892	0.043091
Novel_013538	XLOC_14407.	transcript	0.134583	0	1.00E-05	0	9.10E-05	0	0	0	0
Novel_013539	XLOC_14407.	transcript	1.217697	3.093764	1.574623	1.901062	1.823712	1.474851	1.640431	2.049773	1.303873
Novel_013540	XLOC_14407.	transcript	0.155464	0.596844	0.701077	0.564725	0.155798	0.761355	0.319584	1.195834	0.472169
Novel_013541	XLOC_14413.	transcript	0.258861	0.693036	0.221735	0.334741	0.456837	0.534149	0.515598	0.313527	0.240062
Novel_013542	XLOC_14414.	transcript	0.051882	0.257708	0.1097	0	0	0	0.160332	0.066539	0.009018
Novel_013543	XLOC_14414.	transcript	0.40651	0.697585	0.36078	0.486129	0.502366	0.502343	0.50613	0.716948	0.499611
Novel_013544	XLOC_14414.	transcript	0.007516	0.014367	0.220429	0.00939	0.289922	0.0744	0.021713	0.249699	0.237738
Novel_013545	XLOC_14414.	transcript	0.551794	1.190184	0.340504	0.49117	0.287679	0.541594	0.642519	0.57079	0.374786
Novel_013546	XLOC_14416.	transcript	0.363055	0.351694	0.703891	0.781804	0.462243	0.662611	0.576704	0.467179	0.859296
Novel_013547	XLOC_14418.	transcript	0.213623	0.516154	0.642491	0.285432	0.446695	0.408308	0.695745	0.557843	0.702658
Novel_013548	XLOC_14419.	transcript	0	0	0.139151	0.219841	0.41255	0.246338	0.098423	0.143262	0.53062
Novel_013549	XLOC_14419.	transcript	0	0.157632	0.900957	0.431046	0.053277	0.517775	0.093336	0	0
Novel_013550	XLOC_14421.	transcript	0.175442	0.05405	0.146978	0.152412	0.172549	0.161147	0.276516	0.173357	0.13877
Novel_013551	XLOC_14422.	transcript	0	0.042746	0	0	0	0	0.038832	0	0.658944
Novel_013552	XLOC_14423.	transcript	0	0	0.529847	6.066753	0.060576	0.023722	3.93095	5.682851	1.123738
Novel_013553	XLOC_14423.	transcript	10.15188	11.70513	12.86392	8.197695	12.65756	13.65559	5.999042	7.957249	16.22621
Novel_013554	XLOC_14425.	transcript	0.669726	0.340496	0.475685	0	0.585093	0.396338	0.751197	0.213276	0.835247

Novel_013555	XLOC_14425.	transcript	0.070198	0.045733	0.054514	0.307328	0.094893	0	0.073135	0.172943	0.098956
Novel_013556	XLOC_14425.	transcript	0.196514	0.136735	0.38835	0.095962	0	0	0.8493	0.325376	0.444898
Novel_013557	XLOC_14425.	transcript	0.65147	0.336729	0.616668	0.374135	0.645929	0.738799	0.872154	0.638099	0.440508
Novel_013558	XLOC_14425.	transcript	0.475941	0.258516	0.493351	0.269438	0.259619	0.214307	0.337927	0.512171	0.132889
Novel_013559	XLOC_14425.	transcript	0.939631	0.00577	0.208252	0.119414	0.194862	0.005679	0.212635	0.010951	0.146835
Novel_013560	XLOC_14425.	transcript	0.031729	0.388307	0.074922	0.254317	0.289484	0.505036	0.276598	0.446728	0.441654
Novel_013561	XLOC_14425.	transcript	0	0	0.201269	0.122353	0.232479	0	0.237765	0	0.420341
Novel_013562	XLOC_14425.	transcript	0.117316	0.218153	0.370831	0.192714	0.115127	0.056473	0.25576	0.167132	0.153577
Novel_013563	XLOC_14425.	transcript	0.02683	0.171942	0.134374	0.172823	0.433472	0.494572	0.420184	0.269936	0.236824
Novel_013564	XLOC_14428.	transcript	0	0.762841	0.014752	0.023195	0	0	0.015654	0	0
Novel_013565	XLOC_14430.	transcript	0.655634	0.845882	1.233766	0.99609	1.260304	1.110936	1.480537	1.661	0.79793
Novel_013566	XLOC_14431.	transcript	0.355235	0.251326	0.162493	0.164979	0.191175	0.37664	0.146835	0.230441	0.123019
Novel_013567	XLOC_14431.	transcript	0.068272	0	0	0	0	0	0.167967	0	0
Novel_013568	XLOC_14431.	transcript	0.067652	0.036403	0.031785	0.043637	0.006173	0.049711	0.028905	0.048895	0.197368
Novel_013569	XLOC_14431.	transcript	0	0	0	0	0	0	0.194185	0	0
Novel_013570	XLOC_14431.	transcript	0.029181	0.024803	0.145562	0.104223	0.39275	0.085605	0	0.080025	0
Novel_013571	XLOC_14431.	transcript	0.175155	0.011765	0	0.119748	0.037092	0.338908	0.299926	0.112572	0.092727
Novel_013572	XLOC_14431.	transcript	0.001764	0.029438	0	0	0	0	0	0	0
Novel_013573	XLOC_14431.	transcript	0.330765	0	0	0	0	0	0.007613	0	0.361565
Novel_013574	XLOC_14431.	transcript	0.213905	0	0.371897	0.232613	0.272863	0.156084	0.409907	0.36841	0.153271
Novel_013575	XLOC_14432.	transcript	6.283157	4.640512	7.131515	7.120181	7.347354	11.91139	7.228695	7.729994	9.60056
Novel_013576	XLOC_14438.	transcript	0	0	0.679277	0	0	0	0	0	0
Novel_013577	XLOC_14438.	transcript	0	0	0	0	0	0	0	0	0
Novel_013578	XLOC_14440.	transcript	0.353831	0.513545	0.310703	0.167993	0.207484	0.194541	0.548711	0.305549	0.251798
Novel_013579	XLOC_14441.	transcript	0.465127	0.782404	0.505336	0.512981	0.753408	0.552166	0.863811	0.587356	0.591348
Novel_013580	XLOC_14441.	transcript	0.366027	0.704579	0.280692	0.041836	0.117768	0.36246	0.142888	0.158849	0.222022
Novel_013581	XLOC_14441.	transcript	0	0.061874	0.041923	0.028826	0.107041	0	0.230508	0.159569	0.128694
Novel_013582	XLOC_14441.	transcript	0	0	0.066304	0.006868	0.018073	0	0	0	0.077419
Novel_013583	XLOC_14443.	transcript	0	0	0	0	0	0.048186	0	0	0
Novel_013584	XLOC_14443.	transcript	0	0	0.02878	0	0.051807	0.020567	0.012749	0	0
Novel_013585	XLOC_14443.	transcript	0	0	0.025866	0	0.017536	0	0	0	0
Novel_013586	XLOC_14446.	transcript	0	0.229736	0.440319	0.550672	0.217731	0	0.507116	0.322386	0.076132
Novel_013587	XLOC_14446.	transcript	0.395192	0.115092	0.171157	0.120932	0.295414	0.539229	0.240485	0.170844	0.320305
Novel_013588	XLOC_14447.	transcript	0.541953	0.617811	1.0401	0.516846	0.567587	0.557529	0.806533	0.670216	0.617664
Novel_013589	XLOC_14448.	transcript	0.253582	0.62481	0.359843	0.127859	0	0	0.076064	0	0
Novel_013590	XLOC_14449.	transcript	0	0	0	0	0	0	0	0	0
Novel_013591	XLOC_14449.	transcript	0.939796	1.430942	1.216998	1.278274	1.393378	1.435139	1.649905	2.036118	1.57848
Novel_013592	XLOC_14449.	transcript	0.278953	0.429583	0.544146	0.597011	0.392198	0.445473	0.44024	0.60859	0.747385

Novel_013593	XLOC_14449.	transcript	0.141772	0.258229	0.340529	0.453932	0.234476	0.378847	0.288436	0.280602	0.393405
Novel_013594	XLOC_14449.	transcript	0	0	0	0	0	0	1.91883	0	0
Novel_013595	XLOC_14450.	transcript	0.376995	0.382868	0.244566	0.387271	0.366491	0.329774	0.524262	0.188569	0.270647
Novel_013596	XLOC_14450.	transcript	0	0.007075	0.273376	0.513491	0.391625	0	0.100524	0.506546	0.291846
Novel_013597	XLOC_14451.	transcript	0.776237	1.067145	0.848085	0.749105	0.990792	0.887693	1.151104	1.05123	0.665991
Novel_013598	XLOC_14451.	transcript	0.313369	0.25217	0.042171	0.530524	0.016067	0.239074	0.068551	0.019723	0.014874
Novel_013599	XLOC_14451.	transcript	0.26033	0.159873	0.194406	0.163552	0.28529	0	0.136039	0.228151	0.172381
Novel_013600	XLOC_14451.	transcript	0.238093	0.098501	0.356471	0.203584	0.171645	1.125476	0.61077	0.206317	0.039166
Novel_013601	XLOC_14451.	transcript	0.239753	0	0.244854	0.141425	0.23684	0	0.161328	0.14323	0.241779
Novel_013602	XLOC_14451.	transcript	0	0.00968	0	0.028594	0	0.041422	0.046987	0.070307	0.106887
Novel_013603	XLOC_14451.	transcript	0	0	0	0	0.324431	1.473557	1.187614	0.850591	0.092979
Novel_013604	XLOC_14451.	transcript	0	0	0	0.311856	0	0	0	0	0
Novel_013605	XLOC_14451.	transcript	0.246023	0.367065	2.320972	0.254912	0.719013	0.269013	0.514055	0.313803	0
Novel_013606	XLOC_14451.	transcript	0.226323	0.361668	0	0.349872	0.274816	0	0.571045	0.601003	1.900083
Novel_013607	XLOC_14451.	transcript	0	0	0	0	0.393998	0.580066	0.804826	0.469043	0
Novel_013608	XLOC_14451.	transcript	0.17661	0	0.252676	0.318349	0.241008	0.166291	0.439974	0.288856	0
Novel_013609	XLOC_14451.	transcript	0.212792	0	0.259126	0.271522	0.33983	0.168165	0.444363	0.299723	1.521041
Novel_013610	XLOC_14451.	transcript	0	0	0	0	0.187983	0.229229	0.530649	0.30275	0
Novel_013611	XLOC_14451.	transcript	0.318138	0.156639	0.040005	0.245409	0.218884	0.22712	0.522158	0.310774	1.267243
Novel_013612	XLOC_14451.	transcript	0.693054	0	0.429262	0.428204	0	0	0.859919	0	0
Novel_013613	XLOC_14451.	transcript	1.928457	0.85674	0.433369	0.322422	1.203442	0.034594	0.032456	0.490679	2.015979
Novel_013614	XLOC_14451.	transcript	0	0	0	0	0	0	0	0	0
Novel_013615	XLOC_14451.	transcript	1.470208	0.904703	1.565164	1.26786	0.903219	0.378712	0.855978	1.846707	0.579084
Novel_013616	XLOC_14451.	transcript	0.95176	1.419542	1.653096	2.577941	1.695987	2.120242	1.68288	1.112605	1.645658
Novel_013617	XLOC_14452.	transcript	0.049504	0.124517	0.011378	0.316295	0.013711	0.199991	0.519856	0.460159	0.221661
Novel_013618	XLOC_14452.	transcript	0.168797	0.131126	0.226169	0.195237	0.112673	0.436477	0.711991	0.384814	0.355221
Novel_013619	XLOC_14452.	transcript	0.690641	0.286915	0.702422	0.243867	0.965797	0.101011	0.24794	0.307384	0.123196
Novel_013620	XLOC_14453.	transcript	2.432875	2.160084	2.417935	3.192012	2.426208	2.68063	3.646371	2.633281	2.167358
Novel_013621	XLOC_14453.	transcript	0.240475	1.334666	0.168777	0.114758	0.019847	0.018606	0.113732	0.205479	0.192182
Novel_013622	XLOC_14455.	transcript	0.116455	0.055786	0.189637	0.050032	0.120158	0.207546	0.081563	0.095364	0.231033
Novel_013623	XLOC_14456.	transcript	0.457292	0.028133	0.874822	0.612642	0.02586	0.482002	0.650116	0.014196	0.000122
Novel_013624	XLOC_14456.	transcript	0.409374	0.236114	0.25081	0.055972	0.714028	0.225866	0.263651	0.888178	0.610677
Novel_013625	XLOC_14456.	transcript	0	0.999831	0.0432	0.011668	0.005215	0.012305	0.041219	0	0
Novel_013626	XLOC_14457.	transcript	0.102428	0	0.027814	0.082969	0.064867	0	0.037632	0.117656	0.146047
Novel_013627	XLOC_14500.	transcript	0.098109	0.027104	0.257758	0.237268	0.408124	0.078783	0.24332	0.264633	0.105613
Novel_013628	XLOC_14502.	transcript	0	0.65582	0	0	0.086329	0.992216	0	0	0
Novel_013629	XLOC_14502.	transcript	0.624947	0.119869	0.022179	1.069771	0.929425	0.591596	1.457942	0.224768	2.458337
Novel_013630	XLOC_14502.	transcript	0	0.002061	0.389082	0.129493	0.184574	0.004702	0	2.406775	0

Novel_013631	XLOC_14502.	transcript	0	0	0.749837	0	0	0	0	0	0
Novel_013632	XLOC_14502.	transcript	0	0	0	0	0	0	0	0	0
Novel_013633	XLOC_14502.	transcript	0.22739	0.052947	0	0	0	0	0.085276	0	0
Novel_013634	XLOC_14502.	transcript	0.017137	0.031553	0.029139	0.033711	0.375087	0	0.18449	0.019432	0.807737
Novel_013635	XLOC_14502.	transcript	0.772606	0.441432	1.454193	0.673588	0.325431	0.633305	0	0.010359	0
Novel_013636	XLOC_14503.	transcript	0	0.148365	0	0.061053	0.068961	0.085691	0.113951	0.154417	0.151947
Novel_013637	XLOC_14504.	transcript	0.39644	0.481786	0.459988	0.278309	0.290459	0.336431	0.392469	0.301191	0.308338
Novel_013638	XLOC_14505.	transcript	0.410788	0.608737	0.335323	0.136068	0.278108	0.307136	0.353216	0.160604	0.19631
Novel_013639	XLOC_14505.	transcript	0.003266	0.17527	0	0.000265	0.268381	0.175238	0.183303	0.010343	0.171085
Novel_013640	XLOC_14505.	transcript	0	0	0.074063	0.110786	0	0.072612	0.269254	0.210043	0
Novel_013641	XLOC_14505.	transcript	0.346245	0.251951	0.189251	0.110707	0	0.072697	0	0	0
Novel_013642	XLOC_14506.	transcript	0	0	0	0	0	0	0	0	0
Novel_013643	XLOC_14506.	transcript	0.532407	0.733959	0.257052	0.393809	0.380345	0.493648	0.330224	0.37111	0.297477
Novel_013644	XLOC_14506.	transcript	0.055798	0.495759	0.025701	0.020663	0.06988	0.073638	0.225362	0.034807	0.060132
Novel_013645	XLOC_14506.	transcript	0.577686	0.191388	0.375953	0.257996	0.425802	0.472087	0.461939	0.568635	0.274149
Novel_013646	XLOC_14506.	transcript	0.385504	0.039805	0	0.399987	0.20661	0.069469	0.323457	0.203189	0
Novel_013647	XLOC_14509.	transcript	0.186788	0	0.120086	0.190714	0.145267	0.034319	0.202095	0.17051	0.005336
Novel_013648	XLOC_14514.	transcript	0	0	0.20379	0.204396	0.105546	0.148072	0.060999	0	0.182541
Novel_013649	XLOC_14514.	transcript	0.147552	0.029056	0	0.049047	0.010847	0.036461	0.180283	0.194573	0.021775
Novel_013650	XLOC_14514.	transcript	0.035359	0.747834	0.029305	0.015094	0	0.077817	0.069667	0.059321	0.107051
Novel_013651	XLOC_14517.	transcript	0.399939	0.285756	0.438789	0.546565	0.419946	0.444325	0.38628	0.435323	0.238261
Novel_013652	XLOC_14518.	transcript	0.159671	0.160811	0.218686	1.188652	0.382648	0.457414	0.255907	0.588821	0.615526
Novel_013653	XLOC_14518.	transcript	0.286228	0	0.051301	0.198547	0.056992	0.09225	0.192987	0.201578	0.074293
Novel_013654	XLOC_14519.	transcript	0.462579	0.405479	0.451369	0.35259	0.31878	0.425201	0.509748	0.280907	0.318458
Novel_013655	XLOC_14519.	transcript	0.186117	0.447391	0.092086	0.129586	0.017817	0.043366	0.273798	0.104761	0.531657
Novel_013656	XLOC_14519.	transcript	0.304452	0.740229	0	0.376368	0.402886	0.667599	0	0	0.165373
Novel_013657	XLOC_14519.	transcript	0.271812	0.605824	0.490961	0.191174	0.140488	0.058987	0.273083	0.617282	0.231962
Novel_013658	XLOC_14519.	transcript	0.354473	0	0.133451	0	0	0	0.540872	0.248128	0
Novel_013659	XLOC_14519.	transcript	1.235329	0.910357	0.900125	0.361762	0.718507	0.389308	1.185655	0.752675	1.227151
Novel_013660	XLOC_14519.	transcript	0	0	0	0	0	0	0	0	0
Novel_013661	XLOC_14519.	transcript	0	0.120811	1.142242	1.462742	0.993939	1.264934	0.826221	1.274283	5.049704
Novel_013662	XLOC_14522.	transcript	0.026481	0	0.07618	0.010721	0	0	0.202122	0	0
Novel_013663	XLOC_14522.	transcript	0.251943	0.295253	0.220033	0.425824	0.300581	0.602229	0.3114	0.597837	0.557368
Novel_013664	XLOC_14530.	transcript	0	0	0	0	0	0	0	0	0
Novel_013665	XLOC_14530.	transcript	1.027237	2.068401	1.569178	1.969573	1.563436	1.726031	1.668016	1.453869	0.309781
Novel_013666	XLOC_14530.	transcript	0.907634	0.62477	0.60375	0.307676	0.387318	0.435324	0.303774	1.148264	1.374547
Novel_013667	XLOC_14531.	transcript	1.559844	0.781933	1.590802	2.107649	1.645837	1.673336	2.142854	2.198869	1.576676
Novel_013668	XLOC_14532.	transcript	0.278611	0.400919	0.249184	0.317872	0.420124	0.23747	0.511163	0.388247	0.505998

Novel_013669	XLOC_14533.	transcript	0.138235	0	0.059545	0.021738	0.026233	0.020909	0.106494	0.052091	0.10738
Novel_013670	XLOC_14534.	transcript	0.168719	0.201494	0.28217	0.180356	0.189823	0.080215	0.49605	0.296525	0.612273
Novel_013671	XLOC_14535.	transcript	0.237576	0	0.185529	0	0.048598	0	0	0.044616	0
Novel_013672	XLOC_14537.	transcript	0.912377	0.713901	0.57429	0.730169	0.795397	0.850394	0.675498	0.925268	0
Novel_013673	XLOC_14537.	transcript	0	0	0	0	0	0	0	0	0
Novel_013674	XLOC_14537.	transcript	0.024444	0.034092	0.135079	0.081949	0.226586	0.101259	0.231356	0.081446	0.65386
Novel_013675	XLOC_14537.	transcript	0	0.004323	0.222794	0.019068	0.00405	0.034599	0.012584	0	0.088233
Novel_013676	XLOC_14538.	transcript	0.009351	0	0.002101	0.001666	0.001443	0.011856	0	0.011058	0.005312
Novel_013677	XLOC_14538.	transcript	0.618105	0.049843	0.328337	0.519724	0.377008	0.506487	0.603568	0.619234	0.36399
Novel_013678	XLOC_14538.	transcript	0.019058	0.001423	0	0.015677	0	0.25814	0	0.047746	0.006838
Novel_013679	XLOC_14538.	transcript	0.100553	0.350818	0.032358	0.232234	0.003758	0.109028	0.025584	0	0.099765
Novel_013680	XLOC_14538.	transcript	0	0	0	0	0	0	0.019916	0	0.379253
Novel_013681	XLOC_14538.	transcript	0	0	0	0	0	0	0	0	0
Novel_013682	XLOC_14538.	transcript	0.221471	0	0	0	0.370556	0	0.178741	0.233268	0
Novel_013683	XLOC_14538.	transcript	0.301703	0.112601	0.442545	0.099671	0.057809	0	0.21012	0.09427	0.085231
Novel_013684	XLOC_14538.	transcript	0	0.161058	0	0	0	0	0	0	0
Novel_013685	XLOC_14546.	transcript	0	0.137359	0.237972	0	0	0.175317	0.159796	0	0.086306
Novel_013686	XLOC_14546.	transcript	0.255754	0.160383	0.155157	0.344969	0.212764	0.362663	0.368021	0.216405	0.099085
Novel_013687	XLOC_14546.	transcript	0.213824	0	0.13075	0.162614	0.210391	0.173579	0	0.124654	0.193072
Novel_013688	XLOC_14547.	transcript	0.859904	0.543367	0.503054	0.58344	0.694318	1.19131	1.103905	0.965736	0.481773
Novel_013689	XLOC_14548.	transcript	0	0	0.244019	0	0.072638	0	0	0	0.298231
Novel_013690	XLOC_14553.	transcript	0.119997	0.759894	0.003543	0.335562	0.11846	0	0.147322	0	0.293995
Novel_013691	XLOC_14553.	transcript	0.12893	0.041039	0.271205	0.036392	0.318214	0.326743	0.264468	0.300921	0.120199
Novel_013692	XLOC_14554.	transcript	0.24587	0.253276	0.195232	0.011541	0.027787	0.065493	0.015381	0.180712	0
Novel_013693	XLOC_14554.	transcript	0	0	0	0	0	0	0	0	0
Novel_013694	XLOC_14554.	transcript	0.22288	1.372171	0.60321	0.857727	0.7879	0.960628	0.65473	0.704558	0.72447
Novel_013695	XLOC_14555.	transcript	0.536046	0.496465	0.534869	0.372765	0.448153	0.487699	0.814692	0.518193	0.375256
Novel_013696	XLOC_14556.	transcript	0.577941	0.130364	0.003201	0	0.169296	0.005298	0.355666	0.418037	0.157097
Novel_013697	XLOC_14556.	transcript	0.081788	0	0.428606	0.24741	0.141124	0.56366	0.029287	0.042941	0
Novel_013698	XLOC_14562.	transcript	0.198183	0	0.21183	0.159711	0	0.261304	0.32418	0.397052	0
Novel_013699	XLOC_14562.	transcript	0.0229	0	0.118258	0.112907	0.366663	0.114812	0.07468	0.180593	0.073177
Novel_013700	XLOC_14564.	transcript	0.238563	0.313554	0.466287	0.398881	0.40971	0.253779	0.473868	0.594895	0.291714
Novel_013701	XLOC_14568.	transcript	0	0	0	0	0	0	0.006408	0	0
Novel_013702	XLOC_14568.	transcript	0.140998	0.346687	0.131857	0	0.128574	0.185302	0.472176	0.162267	0.247915
Novel_013703	XLOC_14569.	transcript	0	0.126055	0	0	0	0	0	0	0.402027
Novel_013704	XLOC_14570.	transcript	0.039753	0.243754	0	0	0.054991	0.164029	0	0	0
Novel_013705	XLOC_14572.	transcript	0	0	0	0	0	0	0	0.066213	0
Novel_013706	XLOC_14572.	transcript	0	0	0	0	0	0	0	0	0

Novel_013707	XLOC_14572.	transcript	0	0	0	0	0	0	0	0	0
Novel_013708	XLOC_14572.	transcript	0.308054	0.178916	0.221694	0.205204	0.267516	0.138527	0.242671	0.136577	0.175736
Novel_013709	XLOC_14572.	transcript	0.052259	0.111294	0.175409	0.113035	0.224759	0.07898	0.136064	0.204311	0.042581
Novel_013710	XLOC_14572.	transcript	0.039759	0	0.051483	0.10516	0.055564	0.089078	0.245493	0.280715	0.036555
Novel_013711	XLOC_14573.	transcript	0	0	0	0	0	0.127699	0.368176	0.511605	0.119024
Novel_013712	XLOC_14573.	transcript	1.035983	1.153646	0.745963	1.012717	0.893547	0.809105	1.052352	0.544328	0.560925
Novel_013713	XLOC_14575.	transcript	0	8.087164	0.001028	0	0	0	0.334248	0	0
Novel_013714	XLOC_14575.	transcript	1.031898	7.46585	0.676959	0.737253	0.790947	0.912733	1.733411	0.645453	1.082002
Novel_013715	XLOC_14582.	transcript	0	0	0	0.006352	0	0	0.009039	0	0
Novel_013716	XLOC_14582.	transcript	0	0	0	0	0	0	0	0	0
Novel_013717	XLOC_14582.	transcript	0	0	0	0	0	0	0	0	0
Novel_013718	XLOC_14582.	transcript	0.017096	0	0	0.183888	0.04211	0.039746	0.096986	0.093745	0.030534
Novel_013719	XLOC_14582.	transcript	0	0	0	0	0	0	0	0	0
Novel_013720	XLOC_14584.	transcript	0.008476	0	0.021398	0.143176	0.042412	0.010057	0.012872	0.051829	0
Novel_013721	XLOC_14584.	transcript	0.132744	0.115763	0.426871	2.181327	0.668716	0.448105	0.348014	0.956094	0.179434
Novel_013722	XLOC_14584.	transcript	0.025516	0.072729	0.12574	0.217078	0.114443	0.10733	0.087133	0.066049	0.020373
Novel_013723	XLOC_14589.	transcript	0.062767	0.056578	0.134209	0.03507	0.077611	0.11351	0.234649	0.081177	0.148689
Novel_013724	XLOC_14595.	transcript	0.413469	0.47317	0.772891	0.518802	1.484458	0.819802	1.24734	0.793432	1.896948
Novel_013725	XLOC_14596.	transcript	0	0.043204	0	0	0	0.052234	0.052447	0	0.625444
Novel_013726	XLOC_14596.	transcript	0.271483	0	0.866103	0.862319	0.223731	0.648308	0.232639	0.220455	0
Novel_013727	XLOC_14596.	transcript	0.10519	0.036438	0.659851	0.070649	0.083977	0.085312	0.093519	0.151917	0.538591
Novel_013728	XLOC_14596.	transcript	0.310947	0.308595	0.111129	0.350968	0.180152	0.100527	0.175039	0.311226	0
Novel_013729	XLOC_14596.	transcript	2.00E-06	0.007316	1.60E-05	0.002921	0.137567	0.097928	0.119233	0.005261	0.134502
Novel_013730	XLOC_14596.	transcript	0	0	0.129245	0	0	0.094339	0	0	0
Novel_013731	XLOC_14596.	transcript	0.256902	0.26619	0	0	0.169476	0.092532	0.134484	0	0.001601
Novel_013732	XLOC_14596.	transcript	0.108341	0.067378	0.003718	0.03947	0.064161	0.032032	0.088858	0.034226	1.81348
Novel_013733	XLOC_14596.	transcript	0.90451	0.541805	0.783697	0.88841	0.955332	0.827724	1.188253	1.028122	1.022084
Novel_013734	XLOC_14597.	transcript	0.004917	0.322196	0	0	0.228241	0	0.02079	0.14943	0.05041
Novel_013735	XLOC_14597.	transcript	0.047543	0.523326	0	0.07078	0.001187	0	0.096148	5.80E-05	0.09732
Novel_013736	XLOC_14597.	transcript	0.018899	0.060075	0.11404	0.120782	0.029934	0.164174	0.124938	0.037629	0.078583
Novel_013737	XLOC_14597.	transcript	5.00E-05	7.30E-05	0	0.001718	0	0	0	0.000239	0.033231
Novel_013738	XLOC_14597.	transcript	0.002021	0.012349	0.04448	0.027946	0.009713	0	0.008361	0	0
Novel_013739	XLOC_14597.	transcript	0.746593	0.032083	0.781982	0.495905	0.743747	0.741652	0.46697	0.740446	0.659599
Novel_013740	XLOC_14599.	transcript	0	0	0.006076	0.00527	0.042789	0	0.286704	0	0.005741
Novel_013741	XLOC_14599.	transcript	0.674165	0.078489	0.078898	0.069575	0.30514	0.015434	0.613921	0.092767	0.65472
Novel_013742	XLOC_14599.	transcript	0	0.797276	0	0	0	0	0	0	0
Novel_013743	XLOC_14599.	transcript	0.065968	0.00613	0	0.734678	0	0.019234	0.02377	1.128249	0
Novel_013744	XLOC_14599.	transcript	0.228719	0.021879	0.369553	0.070714	0.54576	0.16291	1.777933	0.15529	0.132943

Novel_013745	XLOC_1459…	transcript	0.85508	0.006019	1.067946	0.424839	1.063375	1.263746	0.313148	0.48313	0.900594
Novel_013746	XLOC_1459…	transcript	0	0.340163	0	0.356944	0	0	0	0	0
Novel_013747	XLOC_1459…	transcript	0.087817	0.19761	0.337266	0.884799	0.342314	0.984252	0.384032	0.62639	0.723753
Novel_013748	XLOC_1460…	transcript	1.986811	1.462167	2.90055	2.956132	3.597756	3.211908	3.253248	2.69898	2.173627
Novel_013749	XLOC_1460…	transcript	0.01982	0.007559	0.02606	0.069728	0.043427	0.053287	0.606006	0.01549	0.025501
Novel_013750	XLOC_1460…	transcript	0.586088	0.47255	0.883625	0.775016	0.774114	0.788945	0.738308	0.654248	0.610266
Novel_013751	XLOC_1460…	transcript	0.57787	0.415856	0.368521	0.200631	0.491391	0.74433	0.469304	0.189537	0.179911
Novel_013752	XLOC_1460…	transcript	0.070954	0.028788	0	0.065745	0.122045	0	0.02	0	0
Novel_013753	XLOC_1460…	transcript	0.098637	0	0.312006	0.032636	0	0	0.052651	0	0
Novel_013754	XLOC_1460…	transcript	0.111854	0	0.124532	0.049629	0.162576	0.261847	0	0.055627	0
Novel_013755	XLOC_1460…	transcript	0	0	0	0.299062	0	0	0.415254	0	0
Novel_013756	XLOC_1460…	transcript	0.103081	0.318958	0.271644	0.233037	0.320039	0.436472	0	0.376684	0.244098
Novel_013757	XLOC_1460…	transcript	0.440659	1.795177	0.752373	0.677244	0.693651	2.816232	0.490771	1.394533	1.094883
Novel_013758	XLOC_1460…	transcript	0.009641	0	0.022959	0	0	0	0	0.04072	0.025396
Novel_013759	XLOC_1460…	transcript	0.078688	0.023288	0.024902	0.057099	0.029847	0	0.076776	0.045977	0.047255
Novel_013760	XLOC_1460…	transcript	0	0	0	0	0	0	0.013145	0	0.121407
Novel_013761	XLOC_1460…	transcript	0	0	0	0.011625	0	0	0	0.00031	0.024642
Novel_013762	XLOC_1460…	transcript	0	0.115541	0	0	0	0	0	0	0
Novel_013763	XLOC_1460…	transcript	0	0	0	0	0	0	0	0	0
Novel_013764	XLOC_1460…	transcript	0.007435	0.005683	0	0.376528	0.463063	0	0.438264	0.181963	0.690641
Novel_013765	XLOC_1460…	transcript	0	0	0	0	0	0	0	0	0
Novel_013766	XLOC_1461…	transcript	4.942788	76.98596	0.156216	1.196159	0.71078	0.602889	4.954779	1.352525	1.028603
Novel_013767	XLOC_1461…	transcript	0.383212	64.76373	0	0.459931	0.626823	1.585031	0.657818	1.025902	0.039206
Novel_013768	XLOC_1461…	transcript	0.142899	47.31173	0	0	0	0	0	0	0
Novel_013769	XLOC_1461…	transcript	0	0.069186	0	0.255962	0.212443	0	0.337756	0.363635	0
Novel_013770	XLOC_1461…	transcript	0	0.099199	0	0.008136	0	0	0	0	0.048353
Novel_013771	XLOC_1461…	transcript	0.165287	0.164994	0.158563	0.137643	0.15133	0.160039	0.167258	0.127018	0.110679
Novel_013772	XLOC_1461…	transcript	0	0	0	0	0	0	0	0	0
Novel_013773	XLOC_1461…	transcript	0	0	0	0	0	0.000159	0.001375	0.012002	0
Novel_013774	XLOC_1461…	transcript	0	0	0	0.022492	0	0	0	0	0
Novel_013775	XLOC_1461…	transcript	0.43342	0.726936	0.381172	0.184512	0.399003	0.408717	0.269516	0.278552	0.46123
Novel_013776	XLOC_1461…	transcript	0	0	0.009851	0.018383	0.045906	0	0	0	0.045981
Novel_013777	XLOC_1461…	transcript	0	0	0	0.382357	0.023069	0.055552	0.407544	0.323016	0
Novel_013778	XLOC_1461…	transcript	0.206816	0.430962	0.180275	0.536402	0.330007	0.228872	0.182224	0.314846	0.044263
Novel_013779	XLOC_1461…	transcript	0.262	0.003381	0.669252	0.635633	0.769873	0.078668	0.438887	0.452913	0.46043
Novel_013780	XLOC_1461…	transcript	0.111457	0.169076	0.229512	0.125826	0.047802	0.604718	0.115341	0.334396	0.040536
Novel_013781	XLOC_1461…	transcript	0.206336	0	0.065778	0.256419	0.063775	0.210989	0.007879	0	0
Novel_013782	XLOC_1461…	transcript	0.989495	0.26102	0.792719	1.171556	1.261389	1.21244	1.293639	0.84728	1.203412

Novel_013783	XLOC_14617.	transcript	0.528444	0	0.572036	0.35117	0.198798	0.310313	1.009503	0.606254	0
Novel_013784	XLOC_14617.	transcript	0.158759	0.03638	0.077609	0.07632	0.090983	0.050222	0.196822	0.137299	0.054004
Novel_013785	XLOC_14620.	transcript	0.14898	0.135413	0.283601	0.380662	0.212629	0.152418	0.272886	0.19827	0.562353
Novel_013786	XLOC_14621.	transcript	1.132424	0.350282	0.500487	0.80261	0.472177	0.490104	1.328817	0.694365	2.226879
Novel_013787	XLOC_14621.	transcript	0.576644	1.175421	0.827159	0.383914	0.963466	0.618777	0.51994	0.909558	0.709968
Novel_013788	XLOC_14621.	transcript	0.023871	0.368913	0.115575	0.297273	0.375732	0.07333	0.280926	0.191128	0.039962
Novel_013789	XLOC_14621.	transcript	0	0.012408	0	0	0.126617	0	0.009014	0.028266	0
Novel_013790	XLOC_14625.	transcript	0	0.15211	0.008181	0.162891	0.052568	0	0.119966	0.164849	0.009619
Novel_013791	XLOC_14625.	transcript	0.238862	0.205458	0.169585	0.233107	0.1978	0.182221	0.195043	0.25516	0.04185
Novel_013792	XLOC_14625.	transcript	0	0	0.190975	0.196878	0.207555	0.173639	0.187754	0.159667	0.017644
Novel_013793	XLOC_14625.	transcript	0.265886	0.246156	0.192875	0.19542	0.222577	0.315794	0.177515	0.264193	0.035453
Novel_013794	XLOC_14625.	transcript	0.217454	0.175497	0.134758	0.156846	0.157038	0.150384	0.460476	0.209633	0
Novel_013795	XLOC_14625.	transcript	0	0	0.193549	0.130342	0.171973	0.130381	0.298348	0.146037	0
Novel_013796	XLOC_14625.	transcript	0.234002	0.166341	0.203343	0.13233	0.18014	0.282172	0.217377	0.180873	0.023266
Novel_013797	XLOC_14625.	transcript	0	0	0	0	0	0	0	0	0.82137
Novel_013798	XLOC_14626.	transcript	0.757577	0.616428	1.295435	1.468032	0.531917	1.027035	1.474689	1.441821	1.193753
Novel_013799	XLOC_14626.	transcript	0	0	0	0	0.912063	0	0	0	0
Novel_013800	XLOC_14626.	transcript	0.007966	0	0.030429	0	0.008397	0.415066	0.233457	0.047885	0
Novel_013801	XLOC_14627.	transcript	0.116321	0	0.104007	0.023244	0.043075	0.016957	0.047299	0.080386	0.299804
Novel_013802	XLOC_14628.	transcript	0.140819	0.021116	0.416585	1.092984	0.035206	0.043658	0.092179	0.066611	0.031652
Novel_013803	XLOC_14629.	transcript	0.282001	0.077684	0.398541	0.294474	0.292375	0.286397	0.327151	0.377266	0.208818
Novel_013804	XLOC_14635.	transcript	0.053362	0	0.226135	0.156722	0.346843	0.194663	0.282928	0.128111	0.19733
Novel_013805	XLOC_14638.	transcript	0.202423	0	0.141838	0.080885	0.164758	0.068379	0.214014	0.090835	0.04992
Novel_013806	XLOC_14639.	transcript	0	0	0.060853	0.054383	0.095634	0.046299	0.064815	0.021751	0
Novel_013807	XLOC_14643.	transcript	0.25696	0.281556	0.102974	0	0.100126	0.533913	0	0.139378	0.105508
Novel_013808	XLOC_14643.	transcript	0.248448	0.873779	0.070759	0.040845	0.344113	0.772617	0.092521	0.172304	0.211482
Novel_013809	XLOC_14644.	transcript	0.038264	0	0.131048	0.029769	0.07495	0	0.105734	0.078858	0.063537
Novel_013810	XLOC_14644.	transcript	0	0	0	0	0	0	0	0	0
Novel_013811	XLOC_14644.	transcript	0.034381	0	0.026336	0	0	0	0.050136	0	0
Novel_013812	XLOC_14644.	transcript	0.735878	0.314598	0.697765	0.393215	0.451572	0.437743	0.859708	0.559721	0.283351
Novel_013813	XLOC_14644.	transcript	0	0	0	0	0	0	0	0.067952	0
Novel_013814	XLOC_14644.	transcript	0	0.208771	0	0	0.20288	0	0	0	0.344192
Novel_013815	XLOC_14645.	transcript	0.324409	0.179106	0.603428	0.402778	0.458924	0.202389	0.501107	0.453239	0.692426
Novel_013816	XLOC_14645.	transcript	0.103235	0.023927	0.257365	0.35209	0.167942	0.230414	0.307182	0.30638	0.093219
Novel_013817	XLOC_14648.	transcript	0.153891	0.318805	0.094789	0.102078	0.039338	0.137051	0.152041	0.12836	0.174833
Novel_013818	XLOC_14649.	transcript	0	0	0	0	0	0	0	0.002651	0
Novel_013819	XLOC_14649.	transcript	0.331385	0.152368	0.659171	0.923737	0.744407	0.579475	0.706124	0.915608	0.899151
Novel_013820	XLOC_14649.	transcript	0.052814	0.575348	0.028042	0.087766	0.026799	0.09006	0.163219	0.041813	0.067373

Novel_013821	XLOC_14650.	transcript	1.738359	1.199624	1.105025	1.706605	1.02992	1.322672	1.173232	0.071856	2.036414
Novel_013822	XLOC_14650.	transcript	0	0	0	0	0	0	0	1.005264	0
Novel_013823	XLOC_14650.	transcript	0	0	0.540395	0	0.504697	0	0.612028	0.749343	0
Novel_013824	XLOC_14650.	transcript	0.133913	0.311173	0.195209	0.307292	0.302246	0.276053	0.427532	0.238285	0.21408
Novel_013825	XLOC_14650.	transcript	0.015144	0	0.036961	0	0	0	0.013955	0	0
Novel_013826	XLOC_14650.	transcript	0.182068	0.002608	0.133365	0.125742	0.111848	0.152539	0.176886	0.231776	0.226009
Novel_013827	XLOC_14652.	transcript	0.287232	0.137882	0.336662	0.273845	0.246081	0.018658	0.239596	0.548357	0.04404
Novel_013828	XLOC_14652.	transcript	0.417892	0.04186	0.202113	0.039145	0.242933	0.223137	0.124059	0.260745	0.019997
Novel_013829	XLOC_14653.	transcript	0.62251	0.191352	0.563323	0.692706	0.425532	0.783036	0.459763	0.215322	0.422705
Novel_013830	XLOC_14654.	transcript	0.585438	1.28671	0.541739	0.46443	0.654308	0.843226	0.524688	0.796378	0.657314
Novel_013831	XLOC_14654.	transcript	0.316163	0.014423	0	0	0.006706	0	0.605972	0.328804	0
Novel_013832	XLOC_14654.	transcript	0.12096	0.474884	0.321683	0.420564	0.3782	0.348499	0.234633	0.10377	0.442302
Novel_013833	XLOC_14654.	transcript	0	0.334173	0	0	0	0	0	0	0
Novel_013834	XLOC_14655.	transcript	0.251982	0.658003	1.091142	1.146305	1.201571	1.035904	1.05885	0.887204	1.068246
Novel_013835	XLOC_14656.	transcript	0.384838	0.368365	0.541527	0.633112	0.716741	0.582827	0.68841	0.578962	0.456498
Novel_013836	XLOC_14656.	transcript	0.399	0.884003	0.886175	1.230021	1.088084	0.912327	0.937861	1.007482	0.796372
Novel_013837	XLOC_14656.	transcript	0	0	0	0	0	0	0	0	0
Novel_013838	XLOC_14656.	transcript	0.118275	0	0.122808	0.142621	0.054991	0.176664	0.112524	0	0.004122
Novel_013839	XLOC_14656.	transcript	1.029769	0.681835	0.87992	0.842053	0.880012	0.894643	1.564309	1.344017	0.975307
Novel_013840	XLOC_14656.	transcript	0.19631	0.782946	0.291819	0.540404	0.498462	0.453032	0.524533	0.275425	0.094839
Novel_013841	XLOC_14657.	transcript	3.4183	3.568779	7.769394	10.00726	8.075143	6.405103	5.889507	5.373437	5.488404
Novel_013842	XLOC_14657.	transcript	2.356973	5.146404	2.334921	3.42815	2.142457	2.952159	1.451365	1.276299	4.950137
Novel_013843	XLOC_14659.	transcript	0	0.294593	0	0	0.01672	0	0	0.032039	0
Novel_013844	XLOC_14659.	transcript	0.092686	0.296437	0.13805	0.08536	0.171302	0.15602	0.200646	0.088578	0.066489
Novel_013845	XLOC_14660.	transcript	0	0.192739	0	0.087777	0	0	0.157708	0	0.071346
Novel_013846	XLOC_14660.	transcript	0.130225	0.084819	0.228332	0	0.095319	0	0	0.12269	0
Novel_013847	XLOC_14660.	transcript	0	0.003952	0.001371	0.003297	0.004605	0	0	0	0.00116
Novel_013848	XLOC_14660.	transcript	0.39319	0.418052	0.312215	0.34624	0.396502	0.433222	0.700951	0.46852	0.318757
Novel_013849	XLOC_14661.	transcript	76.45184	72.20818	120.9467	120.3857	119.6862	154.8832	107.3715	121.3548	166.4996
Novel_013850	XLOC_14664.	transcript	0.021131	0.314808	0	0	0.083107	0	0.294445	0.01077	0
Novel_013851	XLOC_14664.	transcript	0.348364	0	0.319099	0.104115	0.106265	0.1285	0.156412	0.194867	0.34405
Novel_013852	XLOC_14664.	transcript	0	0	0	0	0	0	0	0	0
Novel_013853	XLOC_14664.	transcript	0.316639	0.305366	0.031974	0.674384	0.387356	0.252211	0.362926	0.162505	0.137614
Novel_013854	XLOC_14664.	transcript	0	0.177579	0.478781	0	0.099547	0	0.160432	0.148108	0.186205
Novel_013855	XLOC_14664.	transcript	0.082056	0.199916	0.041131	0.035274	0.103713	0.07791	0.093131	0.109166	0
Novel_013856	XLOC_14664.	transcript	0.000165	0.024657	0	0.03036	0	0.040967	0	0.20665	0
Novel_013857	XLOC_14664.	transcript	0.043952	0.26681	0.009336	0.02515	0.124122	0.070762	0.112597	0.042184	0.002066
Novel_013858	XLOC_14664.	transcript	0.374694	0.541433	0.409587	0.262327	0.301959	0.218458	0.548657	0.200475	0.572855

Novel_013859	XLOC_14665	transcript	0.124395	0.472065	0.14556	0.101388	0.134642	0.143749	0.230276	0.119464	0.321552
Novel_013860	XLOC_14666	transcript	0	0	0	0	0	0	0.00086	0	0
Novel_013861	XLOC_14666	transcript	0.02681	0.592088	0	0.574278	0.254072	0.043375	0.648491	0.690175	0
Novel_013862	XLOC_14666	transcript	0.307156	0.047195	0.392877	0	0.238815	0.57015	0.048883	0.009884	0.282512
Novel_013863	XLOC_14666	transcript	0.741275	0.221121	0.165594	0.155544	0.124396	0.268601	0.167142	0.463576	0.405775
Novel_013864	XLOC_14677	transcript	0.010094	0	0	0.054296	0.219609	0.037985	0.37107	0.045035	0.135479
Novel_013865	XLOC_14677	transcript	0.289205	0	0.277868	0.189907	0.409213	0.334284	0.505751	0.537493	0.126655
Novel_013866	XLOC_14678	transcript	0.712226	0.607756	0	0.703721	0.866896	0.034893	0	0.910319	0.861824
Novel_013867	XLOC_14678	transcript	0.039463	0	0.002639	0	0.035683	0	0.034507	0	0
Novel_013868	XLOC_14678	transcript	0	0	0.734738	0	0	0.734016	1.020997	0	0
Novel_013869	XLOC_14680	transcript	0	0.012585	0.468907	0.117639	0.267869	0.532999	0.079526	0.038028	0.005433
Novel_013870	XLOC_14680	transcript	0.381512	0.404942	0.482836	0.143615	0	0.008405	0.395864	0.24761	0.785175
Novel_013871	XLOC_14680	transcript	0.55232	0.745289	0.487879	0.988544	0.168216	1.246565	1.379349	0.574291	0.564283
Novel_013872	XLOC_14680	transcript	0.012191	0.011126	0.019025	0	0.839979	0	0.071739	0.214686	0.319382
Novel_013873	XLOC_14681	transcript	0.14821	0.454912	0.698914	0.516939	0.957619	1.023091	0.780981	0.689302	0.91144
Novel_013874	XLOC_14681	transcript	0.603333	0	0.272807	0.569851	0.217858	0.219892	0.723443	0.403816	0
Novel_013875	XLOC_14681	transcript	0.137866	0.011361	0.124849	0.099804	0.057766	0.0341	0.126368	0.053926	0
Novel_013876	XLOC_14681	transcript	0.234581	0.01854	0.136259	0.056417	0.1848	0.096757	0.146628	0.230393	0.144483
Novel_013877	XLOC_14681	transcript	0	0	0.064892	0.063945	0.014294	0	0	0.000343	0
Novel_013878	XLOC_14682	transcript	0.257413	0.205249	0.329865	0.309216	0.354123	0.283652	0.258926	0.39947	0
Novel_013879	XLOC_14682	transcript	0.066279	0.021293	0.029365	0.03116	0.008252	0.085488	0.065953	0.042906	0.274873
Novel_013880	XLOC_14682	transcript	0	0.041287	0.181993	0.004638	0.010818	0.010286	0.172832	0.016949	0
Novel_013881	XLOC_14682	transcript	1.139424	2.100568	0.810401	0.847287	0.888111	0.709424	1.237375	1.219381	0.706141
Novel_013882	XLOC_14682	transcript	0	0	0	0	0.098565	0	0	0	0
Novel_013883	XLOC_14687	transcript	0.010503	0.432279	0.159732	0.262055	0.281896	0.148278	0.141102	0.411974	0.018198
Novel_013884	XLOC_14687	transcript	0	0.003629	0.008244	0.032331	0.042648	0	0.015657	0.08091	0.129357
Novel_013885	XLOC_14688	transcript	0	0	0	0.019526	0	0	0	0	0
Novel_013886	XLOC_14688	transcript	0.427807	0.498191	0.315653	0.297462	0.301825	0.460756	0.908732	0.330617	0.317194
Novel_013887	XLOC_14689	transcript	0.384822	0.452068	0.080075	0.14273	0.244889	7.90E-05	0.148352	0.112207	0.311584
Novel_013888	XLOC_14689	transcript	0	0.12907	0	0.202374	0.243324	0	0	0	0.074496
Novel_013889	XLOC_14689	transcript	0.24355	0	0	0.169062	0.376287	0	0.000279	0	0
Novel_013890	XLOC_14689	transcript	0.735131	0	0	0	0	1.195496	0	0.612131	0
Novel_013891	XLOC_14689	transcript	0	0	0	0.084602	0.000793	0	0	0	0.397947
Novel_013892	XLOC_14689	transcript	2.371238	0	2.191026	0	0	0	0	1.600282	0
Novel_013893	XLOC_14689	transcript	1.316855	0	2.060891	0	0	0	0	0.998022	0
Novel_013894	XLOC_14689	transcript	0	0.074204	0	0.054831	0.074149	1.191829	4.079987	0	0.040116
Novel_013895	XLOC_14689	transcript	0	0	0	0	0	0.307495	1.314964	0	0
Novel_013896	XLOC_14690	transcript	1.28813	0.978717	0.747832	0.65936	0.819706	0.962753	1.240824	0.814006	0.599577

Novel_013897	XLOC_14692.	transcript	0.17742	0.632482	0.105702	0.067115	0.157779	0.16144	0.284524	0.144566	0.13859
Novel_013898	XLOC_14692.	transcript	0.487792	0.257478	0.074483	0.092847	0.501618	0.303609	0.602429	0.103726	0.048809
Novel_013899	XLOC_14692.	transcript	0.035153	0.013282	0.397028	0.20463	0.037061	0	0.092018	0.427342	0.335155
Novel_013900	XLOC_14693.	transcript	0	0	0	1.134927	0	0	0.830747	0.779165	0
Novel_013901	XLOC_14693.	transcript	0.625439	1.003039	1.101951	0.714077	1.380248	1.229961	0.612345	0.612712	1.212085
Novel_013902	XLOC_14694.	transcript	0.569307	0.102744	0.455446	0.38915	0.472702	0.360276	0.481164	0.751256	0.332258
Novel_013903	XLOC_14695.	transcript	0.346626	0	0.388388	0.595249	0.466484	0.546653	0.36059	0.351782	0.516538
Novel_013904	XLOC_14695.	transcript	0.303172	0	0	0.355846	0.253158	0.245125	0.284247	0.635958	0.444077
Novel_013905	XLOC_14695.	transcript	0.028166	0.205883	0.078779	0.048601	0.044705	0.221545	0.04005	0.053254	0.01038
Novel_013906	XLOC_14695.	transcript	0.138519	0.109191	0.417268	0.277455	0.324432	0.134628	0.586522	0.013509	0.23731
Novel_013907	XLOC_14695.	transcript	0.325296	0	0	0.186651	0.2217	0.112966	0.231329	0	0.114141
Novel_013908	XLOC_14695.	transcript	0	0	0	0.23615	0	0.131343	0.300604	0	0
Novel_013909	XLOC_14695.	transcript	0.5587	0.337385	0.116969	0	0.048423	0.164801	0	0.136217	0.104453
Novel_013910	XLOC_14695.	transcript	0.040884	0	0.681295	1.000903	0.994704	0.794457	0.356316	1.128253	0.557373
Novel_013911	XLOC_14696.	transcript	0.566685	1.509881	0.409527	0.225801	0.381898	0.373983	0.690301	0.36808	0.340495
Novel_013912	XLOC_14698.	transcript	0.010516	0.02215	0.053715	0.002418	0.105413	0.257253	0.292229	0	0.006003
Novel_013913	XLOC_14698.	transcript	0	0.014089	0	0	0	0	0	0.227536	0
Novel_013914	XLOC_14698.	transcript	0.043838	0.008359	0.100174	0.107281	0.045902	0.109493	0.138302	0.015868	0
Novel_013915	XLOC_14698.	transcript	0.034437	0.001498	0	0.024443	0	0.111706	0.331072	0	0.097816
Novel_013916	XLOC_14698.	transcript	0.370574	0.481162	0.433777	0.457912	0.440443	0.02002	3.80E-05	0.544975	0.18345
Novel_013917	XLOC_14699.	transcript	0.207575	0.313766	0.341683	0.369776	0.339218	0.472128	0.384232	0.259934	0.300316
Novel_013918	XLOC_14700.	transcript	0.03825	0	0.101643	0.133874	0.050909	0.241106	0.081092	0.170643	0.115874
Novel_013919	XLOC_14700.	transcript	0.338581	0.479232	0.146173	0.200329	0.129112	0.193074	0.202163	0.264737	0.284106
Novel_013920	XLOC_14700.	transcript	0.671933	1.228873	0.672961	0.583455	0.641466	0.648544	0.908826	0.716906	0.574968
Novel_013921	XLOC_14700.	transcript	0	0	0	0	0	0.022839	0	0	0
Novel_013922	XLOC_14700.	transcript	0.027333	0.062235	0	0.004203	0.013966	0	0.038503	0.020673	0.031249
Novel_013923	XLOC_14702.	transcript	0	0	0	0.197715	0.047084	0.184345	0	0.145604	0
Novel_013924	XLOC_14705.	transcript	0	0.011366	0.01627	0.017916	0.012111	0.000936	0	0.248571	0.002959
Novel_013925	XLOC_14705.	transcript	0.291749	0.120115	0.043248	0.036896	0.261416	0.320226	0.689054	0	0.228327
Novel_013926	XLOC_14705.	transcript	0	0	0	0	0	0	0	0	0
Novel_013927	XLOC_14705.	transcript	0	0.255446	0.517031	0.574518	0.403034	0.264225	0.346346	0.327228	0.433073
Novel_013928	XLOC_14705.	transcript	0	0.011565	0.014835	0.017709	0	0.054693	0.04266	0	0
Novel_013929	XLOC_14705.	transcript	1.29033	0.667567	2.772475	2.701871	2.723084	5.082287	2.278851	1.788022	2.633841
Novel_013930	XLOC_14709.	transcript	0.395271	0.3215	0.362565	1.148506	0.991659	0.572832	0.50762	0.791085	0.181543
Novel_013931	XLOC_14709.	transcript	0	0	0.052383	0.294542	0	0.277828	0.368739	0.448546	0.320902
Novel_013932	XLOC_14709.	transcript	0	0	0	0.313358	0.003775	0.002506	0	0.108548	0
Novel_013933	XLOC_14709.	transcript	0.547399	0.614562	0.406007	0.451111	0.485328	0.414613	1.095577	0.745616	0.557017
Novel_013934	XLOC_14709.	transcript	0.314088	0.329167	0.333209	0.549266	0.368705	0.443141	0.617425	0.379462	0.742221

Novel_013935	XLOC_14709.	transcript	2.655173	2.006197	1.013553	0.737575	1.239963	0.697442	1.579013	1.811907	1.242492
Novel_013936	XLOC_14709.	transcript	0.163876	0.436646	0.168161	0.354525	0.307968	0.352822	0.644778	0.289073	0.628846
Novel_013937	XLOC_14709.	transcript	0.523251	1.483927	0.755957	1.419517	0.218531	0.846317	0.425512	2.145371	1.462987
Novel_013938	XLOC_14709.	transcript	0	0	0.342254	0.563031	0	0	0	0	0.18638
Novel_013939	XLOC_14709.	transcript	2.77582	1.973655	1.710062	1.220839	2.013418	1.359114	2.122021	1.785178	1.943131
Novel_013940	XLOC_14709.	transcript	1.137611	0	1.23064	0	0.338513	0	1.098876	1.668762	1.266235
Novel_013941	XLOC_14709.	transcript	0	0	0	0.418638	0	0	2.932212	0	0.432603
Novel_013942	XLOC_14709.	transcript	0.284412	0.457335	0.464557	0.663049	0.615373	0.742294	0.808534	0.494946	0.839762
Novel_013943	XLOC_14709.	transcript	0.245311	0.33339	0.215484	0.190953	0.271699	0.193632	0.399551	0.417742	0.302026
Novel_013944	XLOC_14709.	transcript	2.648255	2.378942	2.639513	1.846224	2.624629	2.230692	3.956782	3.505299	2.693642
Novel_013945	XLOC_14710.	transcript	0.126313	0.171028	0.166573	0.130503	0.072758	0.215046	0.189492	0.052387	0.154297
Novel_013946	XLOC_14710.	transcript	0	0.020386	0	0	0.103365	0	0	0	0
Novel_013947	XLOC_14710.	transcript	0	0.02237	0.059786	0	0.070933	0	0.19845	0.04236	0.026357
Novel_013948	XLOC_14710.	transcript	0	0	0	0	0.123111	0.081441	0.159516	0.193246	0
Novel_013949	XLOC_14720.	transcript	0.486998	0.71603	0.519622	0.485486	0.708039	0.288591	0.778827	0.231276	0.562797
Novel_013950	XLOC_14720.	transcript	0.047553	1.06164	0.108392	0.197911	0.076467	0.446646	0.297315	0.436069	0.446342
Novel_013951	XLOC_14723.	transcript	0.519885	0.261425	0.454539	0.354986	0.769268	0.680461	0.741813	0.757401	0.510506
Novel_013952	XLOC_14723.	transcript	0.173255	0.160682	0.32107	0.255494	0.019806	0.033852	0.012944	0.001719	0.008144
Novel_013953	XLOC_14729.	transcript	0.031256	0.811024	0.050067	0.094199	0.403422	0.076184	0.188897	0.101769	0.017247
Novel_013954	XLOC_14730.	transcript	0	0.621757	0	0.000945	0.00313	0	0.414844	0.229573	0
Novel_013955	XLOC_14730.	transcript	0.150648	0	0	0	0	0.055321	0	0.329745	0
Novel_013956	XLOC_14730.	transcript	0	0.041491	0	0	0	0	0.180647	0	0
Novel_013957	XLOC_14730.	transcript	0.025831	4.00E-06	0.009028	0.013712	0.023592	0	0.188673	0	0
Novel_013958	XLOC_14730.	transcript	0.35917	0.010984	0.398142	0.416281	0.540219	0.344079	0	0.018253	0.455284
Novel_013959	XLOC_14730.	transcript	0.461755	0.21909	0.559082	0.25782	0.056179	0.005681	0.374889	0.616457	0.319386
Novel_013960	XLOC_14730.	transcript	0	0.605525	0	0.400452	0.537407	0.57291	0.44767	0.038467	0.19373
Novel_013961	XLOC_14731.	transcript	0.012335	0.014671	0.730631	0.008509	0.053354	0.50223	0.927987	0.012482	0.400979
Novel_013962	XLOC_14731.	transcript	0	0	0	0	0	0	0	0	0
Novel_013963	XLOC_14731.	transcript	0.624965	0.209556	0.002513	0.385442	0.42928	0.042938	0.009269	0.449143	0.012597
Novel_013964	XLOC_14733.	transcript	0.079197	0.101154	0.1887	0.223763	0.08913	0.230891	0.172057	0.221034	0.104
Novel_013965	XLOC_14735.	transcript	1.137501	1.441535	0.966861	1.098611	0.916758	1.496796	0.4929	1.867125	0.191016
Novel_013966	XLOC_14739.	transcript	0.485608	0.015644	0.459729	0.442536	0.477316	0.558828	0.613352	0.48453	0.488529
Novel_013967	XLOC_14739.	transcript	0.32271	0.15398	0.182413	0	0.011795	0	0	0.307289	0.211125
Novel_013968	XLOC_14742.	transcript	1.830243	0.065107	1.331407	1.658072	2.022329	1.910574	1.556061	2.674371	0.423559
Novel_013969	XLOC_14774.	transcript	0.497937	0.146626	0.460153	0.526598	0.49463	0.548698	0.671255	0.722688	0.296732
Novel_013970	XLOC_14785.	transcript	0.297523	0.265035	0.43018	0.4543	0.574044	0.444341	0.487844	0.408613	0.553784
Novel_013971	XLOC_14808.	transcript	0.463072	0.00166	0.224186	0.182531	0.003347	0.029171	0.051041	0.042761	0.268572
Novel_013972	XLOC_14808.	transcript	0	0	0.045067	0	0.042322	0	0	0.066267	0

Novel_013973	XLOC_14808.	transcript	1.085175	0	1.052359	0	0.384344	0.337894	0.330063	0.117313	0.184507
Novel_013974	XLOC_14808.	transcript	0	0	0	0	0	0	0	0	0
Novel_013975	XLOC_14808.	transcript	0.185643	0	0.323638	0.01786	0.032549	0.030768	0.064509	0.10891	0.187479
Novel_013976	XLOC_14813.	transcript	0.795803	0.726442	1.173098	1.179526	0.07816	0.842648	0.880691	0.600591	0.646635
Novel_013977	XLOC_14822.	transcript	0.525067	0	0.486578	0.389574	0.20615	0.206595	0.309428	0.68977	0.24901
Novel_013978	XLOC_14822.	transcript	0.139931	0	0	0	0.15181	0.145833	0.1734	0	0
Novel_013979	XLOC_14822.	transcript	0.222467	0.037733	0.187348	0.123702	0.106588	7.60E-05	0.119003	0.000753	0.100563
Novel_013980	XLOC_14827.	transcript	0.163937	0	0.134523	0.058135	0.018593	0.021356	0.235256	0.057084	0.085122
Novel_013981	XLOC_14827.	transcript	0	0	0	0	0	0.059338	0	0	0
Novel_013982	XLOC_14852.	transcript	0	0	0	0	0	0	0	0	0
Novel_013983	XLOC_14852.	transcript	0	0	0	0	0.178132	0	0	0	0
Novel_013984	XLOC_14852.	transcript	0.034341	0.041527	0.046614	0.077538	0.060112	0.062019	0.035632	0.143528	0.033458
Novel_013985	XLOC_14852.	transcript	0.01644	0.051588	0.060122	0.065924	0.091934	0.069526	0.053259	0.180211	0.030843
Novel_013986	XLOC_14852.	transcript	0.038258	0.082341	0.069302	0.099039	0.071728	0.098627	0.07572	0.151703	0.037545
Novel_013987	XLOC_14852.	transcript	0.056534	0.026587	0.252191	0.050181	0.080099	0.019598	0.053929	0.048588	0.043755
Novel_013988	XLOC_14852.	transcript	0.161036	0.03742	0.099044	0.096995	0.261325	0.05301	0.09274	0.092151	0.067771
Novel_013989	XLOC_14852.	transcript	0.069584	0.04174	0.078681	0.216682	0.06334	0.05134	0.094536	0.135157	0.105326
Novel_013990	XLOC_14852.	transcript	0.104426	0.051707	0	0	0	0.100616	0	0.233166	0.106304
Novel_013991	XLOC_14852.	transcript	0.069578	0.032103	0.046061	0.165688	0.06519	0.02112	0.335597	0.099884	0.088393
Novel_013992	XLOC_14852.	transcript	0.057029	0.036579	0.041944	0.183418	0.036574	0.038962	0.055208	0.085658	0.137408
Novel_013993	XLOC_14852.	transcript	2.574304	2.312669	1.69084	1.376353	0.008022	0.308794	2.479018	0.122499	1.509709
Novel_013994	XLOC_14852.	transcript	0.00144	0	0	0.001051	0	0.212142	0	0	0.000639
Novel_013995	XLOC_14852.	transcript	0.001238	0.020722	0	0	0	0.232268	0	0.000947	0.000489
Novel_013996	XLOC_14852.	transcript	0.161596	0	0.189712	0	0.296738	0.070963	0.193781	0.209558	0
Novel_013997	XLOC_14855.	transcript	0.125461	0.532082	1.094154	1.214814	0.652473	1.155002	0.221253	0.431031	0.85852
Novel_013998	XLOC_14855.	transcript	0.513709	0.19403	0.344844	0.270022	0.470097	0.397517	0.718327	0.453808	0.455068
Novel_013999	XLOC_14855.	transcript	0.119729	0	0	0.209792	0	0	0.146187	0	0.167575
Novel_014000	XLOC_14855.	transcript	0.205367	0.079637	0.419131	0.101343	0.292008	0.257513	0.491463	0.365163	0.497986
Novel_014001	XLOC_14858.	transcript	0.249494	0.121504	0.252442	0.459721	0.443361	0.345868	0.717544	0.479386	0.397884
Novel_014002	XLOC_14861.	transcript	0.212566	0.048827	0.079884	0.056028	0.133957	0.080589	0.054411	0.153236	0.079226
Novel_014003	XLOC_14862.	transcript	0	0.41537	0	0	0	0.066206	0.037492	0	0
Novel_014004	XLOC_14862.	transcript	0	0.461633	0	0	0	0	0	0	0
Novel_014005	XLOC_14862.	transcript	0.406649	0.490392	0.250508	0.309966	0.304582	0.328151	0.577115	0.446178	0.652977
Novel_014006	XLOC_14862.	transcript	0.128312	0.1293	0.121638	0.155065	0.122715	0.118665	0.194873	0.147121	0.335155
Novel_014007	XLOC_14866.	transcript	0	0	0.022041	0	0	0	0.090488	0	0
Novel_014008	XLOC_14866.	transcript	0.302852	0.096641	0	0.01084	0	0.509933	0.046544	0	0.017294
Novel_014009	XLOC_14866.	transcript	0.058726	1.857962	0.332424	0.250013	0.415934	0	0.198256	0.173676	0.366463
Novel_014010	XLOC_14866.	transcript	0.16585	0.109607	0	0.01821	0	0	0.063977	0.241336	0.014572

Novel_014011	XLOC_14873.	transcript	0.122599	0.303432	0.329927	0.267687	0.219712	0.32573	0.053216	0.245756	0.363789
Novel_014012	XLOC_14873.	transcript	0	0	0	0	0.121426	0	0.258559	0	0
Novel_014013	XLOC_14878.	transcript	0.150904	0.136615	0.197962	0.183541	0.146692	0.1538	0.205591	0.282069	0.197204
Novel_014014	XLOC_14881.	transcript	0	0	0	0	0	0	0	0	0.231992
Novel_014015	XLOC_14883.	transcript	0.357673	0.41989	0.273521	0.275977	0.222009	0.308363	0.523586	0.360013	0.208906
Novel_014016	XLOC_14888.	transcript	0.157214	0.188899	0.145326	0.169593	0.181598	0.125536	0.176355	0.141705	0.135809
Novel_014017	XLOC_14888.	transcript	0.271436	0.663873	0.324327	0.277132	0.489624	0.275539	0.675762	0.276034	0.228436
Novel_014018	XLOC_14888.	transcript	0.072456	0.62408	0.216835	0.050778	0.225065	0.160067	0.209133	0.101708	0.036838
Novel_014019	XLOC_14892.	transcript	0.20555	0.257301	0.16202	0.112467	0.175754	0.187679	0.204665	0.340911	0.173163
Novel_014020	XLOC_14892.	transcript	0	0	0.298554	0.468158	0.160342	0	0	0.544735	0
Novel_014021	XLOC_14892.	transcript	0	0	0.021388	0	0	0	0	0	0
Novel_014022	XLOC_14896.	transcript	0	0	0	0.267025	0	0	0	0	0
Novel_014023	XLOC_14896.	transcript	0	0	0	0	0	0	0	0	0
Novel_014024	XLOC_14896.	transcript	0	0	0	0	0	0	0.475411	0	0
Novel_014025	XLOC_14896.	transcript	0.174846	0.418177	0	0.059692	0.168812	0	0.19235	0	0
Novel_014026	XLOC_14896.	transcript	1.713401	0.483543	1.200053	1.189207	1.004755	1.18044	1.031959	1.064886	0.810937
Novel_014027	XLOC_14896.	transcript	0	0	0	0	0	0	0	0.573607	0
Novel_014028	XLOC_14896.	transcript	0	0.069196	0.173019	0.013272	0.528073	0.108273	0.068719	0.232923	0.118835
Novel_014029	XLOC_14896.	transcript	0.020726	0.603966	0.072378	0.128979	0.046441	0.054332	0.153673	0.117461	0.13721
Novel_014030	XLOC_14896.	transcript	0	0	0	0	0	0	0	0	0
Novel_014031	XLOC_14902.	transcript	0.190635	0	0.029181	0.131239	0.110343	0.01545	0.087437	0.049768	0
Novel_014032	XLOC_14902.	transcript	0.062365	0	0	0	0	0.024333	0.044356	0.10144	0
Novel_014033	XLOC_14902.	transcript	0.102191	0	0	0	0	0	0	0.037328	0
Novel_014034	XLOC_14902.	transcript	0	0	0.253596	0.006997	0.05734	0	0.195225	0.078126	0
Novel_014035	XLOC_14903.	transcript	0	0	0.355221	0	0	0.306111	0	0.335426	0
Novel_014036	XLOC_14903.	transcript	0.463223	0.022898	0.311771	0.24024	0.030496	0.07625	0.356381	0.043191	0.170368
Novel_014037	XLOC_14903.	transcript	0	0	0.000644	0.002057	0	0	0	0.001794	0
Novel_014038	XLOC_14903.	transcript	0.008891	0	0.2371	0.055323	0.002853	0.009503	0	0	0.23582
Novel_014039	XLOC_14903.	transcript	0	0	0	0	0	0	0	0	0
Novel_014040	XLOC_14903.	transcript	0	0	0	0	0	0	0	0	0
Novel_014041	XLOC_14903.	transcript	0.005966	0.006417	0.000703	0.000948	0	0	0.276294	0.069404	0.227227
Novel_014042	XLOC_14903.	transcript	0	0	0.129058	0.401552	0.109441	0	0.436268	0.102395	0
Novel_014043	XLOC_14903.	transcript	0	0	0.086491	0	0.002252	0.122397	0.436616	0	0.061489
Novel_014044	XLOC_14903.	transcript	0.172497	0.004527	0	0.055337	0.082329	0.019962	0.012894	0.069129	0
Novel_014045	XLOC_14903.	transcript	0.960483	0.073306	0.189925	0.546324	0.571581	0.022002	1.547577	0.323061	0.159631
Novel_014046	XLOC_14905.	transcript	0.192526	0.007431	0.203094	0.194587	0.225555	0.118343	0.501524	0.094165	0.082465
Novel_014047	XLOC_14912.	transcript	0.272625	0	0.109934	0.164602	0.163523	0.064243	0.569974	0.121549	0.104329
Novel_014048	XLOC_14919.	transcript	0.861144	1.220375	1.070413	1.165034	0.872739	1.068153	1.340126	1.410311	0.766194

Novel_014049	XLOC_14919.	transcript	0.075435	0.01651	0.063183	0.263742	0.020375	0.31246	0.065862	0	0.118045
Novel_014050	XLOC_14922.	transcript	0	0.037054	0	0.068618	0	0	0	0.010751	0.228721
Novel_014051	XLOC_14922.	transcript	0.321905	0.312393	0.218138	0.16979	0.189868	0.266729	0.438879	0.461396	0.228083
Novel_014052	XLOC_14927.	transcript	0.31483	0.197674	0.70254	0.205722	0.433362	0.338506	0.219851	0.258412	0.408636
Novel_014053	XLOC_14928.	transcript	0.089996	10.44029	0.194626	0.171522	0.249632	0.237186	0.285834	0.20356	0.174526
Novel_014054	XLOC_14929.	transcript	6.690144	919.8538	20.17149	4.187272	8.564948	21.58249	24.47118	23.33942	34.50951
Novel_014055	XLOC_14929.	transcript	7.544352	3195.886	12.21725	7.00243	20.72349	11.88183	12.60577	0.839431	11.58151
Novel_014056	XLOC_14929.	transcript	16.60942	1444.316	12.35492	2.142186	5.761748	28.5997	21.56783	12.1047	12.17174
Novel_014057	XLOC_14929.	transcript	0	1389.245	1.176737	7.578853	0	3.644754	0	1.009625	0
Novel_014058	XLOC_14929.	transcript	26.15434	1595.941	0	0	25.49571	13.79288	38.45228	13.02836	12.61984
Novel_014059	XLOC_14930.	transcript	0.900839	91.60357	0.915982	1.042093	1.584511	3.577731	0.155129	1.662627	0.728964
Novel_014060	XLOC_14930.	transcript	0.085405	18.70579	0.090292	0.447845	0.217213	0	0.063874	0.09633	0
Novel_014061	XLOC_14932.	transcript	0.187439	0.457319	0.257109	0.281517	0.191684	0.19692	0.22683	0.136825	0.216184
Novel_014062	XLOC_14932.	transcript	0.122546	0.111414	0	0	0.040224	0.004873	0.22054	0.179764	0
Novel_014063	XLOC_14932.	transcript	0.369557	0.87538	0.965758	0.357952	0.790025	0.579539	0.828023	0.532865	0.549466
Novel_014064	XLOC_14932.	transcript	0.172525	0.149557	0.173506	0.091881	0.159699	0.244038	0.333056	0.240208	0.247199
Novel_014065	XLOC_14932.	transcript	0.207307	0.478878	0.512872	0.365976	0.326602	0.414479	0.244765	0.295668	0.005867
Novel_014066	XLOC_14932.	transcript	0.39132	0.108459	0.391242	9.60E-05	0.592213	0.077494	0.898659	0.514956	0.716056
Novel_014067	XLOC_14932.	transcript	0	0	9.20E-05	0	0	0	0	0	0
Novel_014068	XLOC_14932.	transcript	0.05202	0.018632	0	0.039734	0.05263	0.192554	0.011112	0.042299	0.036499
Novel_014069	XLOC_14932.	transcript	0.050376	0.066987	0.012461	0.22006	0	0.001318	0.084857	0.034289	0.056548
Novel_014070	XLOC_14933.	transcript	0.255918	0.013122	0.73471	0.153942	0	0	0.178477	0	0.319444
Novel_014071	XLOC_14933.	transcript	0.532922	0.662335	0.242509	0.233015	0.674707	0.568024	0.744842	0.419342	0.898472
Novel_014072	XLOC_14934.	transcript	0.004467	0	0	0	0	0.012865	0	0.000245	0
Novel_014073	XLOC_14934.	transcript	0.002469	0	0	0	0.098465	0	0	0.007043	0.007726
Novel_014074	XLOC_14934.	transcript	0.519995	0.309209	0.316006	0.480542	0.335438	0.316236	0.662418	0.50467	0.40628
Novel_014075	XLOC_14935.	transcript	0	0	0	0	0.756156	0	0	0	0
Novel_014076	XLOC_14935.	transcript	1.217843	1.304148	1.557495	1.543383	1.715628	1.816419	1.86703	1.969767	1.597389
Novel_014077	XLOC_14935.	transcript	0.316349	0.532383	0.399272	0.699786	0	0.839645	0.380118	0.208001	0.406051
Novel_014078	XLOC_14935.	transcript	0	0	0	0.131471	0	0	0.035376	0	0
Novel_014079	XLOC_14937.	transcript	0.322936	0.030909	0.457182	0.446133	0.035605	0	0.329237	0.398759	0
Novel_014080	XLOC_14937.	transcript	0.113476	0.692043	0.290069	0.387355	0.62641	0.408108	0.502074	0.286913	0.72545
Novel_014081	XLOC_14937.	transcript	0	0.036832	0.011785	0.027934	0.039771	0.018367	0.041518	0.036025	0.038513
Novel_014082	XLOC_14941.	transcript	0	0.094035	0.510494	0.076482	0.058489	0.006973	0.168687	0.078372	0.006849
Novel_014083	XLOC_14941.	transcript	0	0	0.135524	0	0.02513	0.273201	0	0	0
Novel_014084	XLOC_14941.	transcript	0.545178	0.429746	0.204429	0.498739	0.391998	0.155412	0.372532	0.363175	0.266079
Novel_014085	XLOC_14941.	transcript	0	0	0	0	0	0	0	0	0
Novel_014086	XLOC_14941.	transcript	0	0	0	0	0.422598	0	0	0.001662	0

Novel_014087	XLOC_14941.	transcript	0.113584	0.107859	1.396698	0.717461	0.522952	1.080689	1.020312	0.422753	0.885882
Novel_014088	XLOC_14942.	transcript	0.020158	0.029907	0.27628	0.573201	0.027518	0	0.012146	0.019329	0
Novel_014089	XLOC_14942.	transcript	0.054371	0	0.149404	0.5565	0	0.0682	0.034424	0.062522	0
Novel_014090	XLOC_14942.	transcript	1.002876	0.135365	0.218849	0.065277	0.054609	0.788716	1.168889	0.936204	0.449742
Novel_014091	XLOC_14942.	transcript	0.010648	0	0.139552	0	0.120635	0	0.005967	0	0.097382
Novel_014092	XLOC_14942.	transcript	0	0	0.282855	0	0.219145	0	0	0.011585	0
Novel_014093	XLOC_14942.	transcript	0.236756	1.012399	0.469756	0.859451	0.625704	0.482768	0.641887	0.53383	0.851304
Novel_014094	XLOC_14945.	transcript	0.349019	0.131502	0.297167	0.106427	0.312941	0.323862	0.412616	0.242943	0.17063
Novel_014095	XLOC_14945.	transcript	1.22074	4.688766	3.153718	2.329557	1.715976	1.52597	1.040983	1.454666	2.308673
Novel_014096	XLOC_14946.	transcript	0.022117	0.062323	0.037396	0.059592	0.027145	0.100501	0.149344	0.07045	0
Novel_014097	XLOC_14946.	transcript	0.476603	0	0	0	0	0	0.241403	0.25407	0.00974
Novel_014098	XLOC_14946.	transcript	0.019376	0.169965	0.358398	0.32811	0.319173	0.297834	0.27108	0.261017	0.213633
Novel_014099	XLOC_14946.	transcript	0.002211	0	0.052632	0.210374	0.134564	0.038912	0.297047	0.025782	0.111971
Novel_014100	XLOC_14946.	transcript	0.02912	0.373557	0.305628	0.402817	0.437604	0.237524	0.399282	3.20E-05	0.242314
Novel_014101	XLOC_14947.	transcript	0.341903	0	0.62993	0.395752	0.487299	0.518908	0.983889	0.46545	0.355781
Novel_014102	XLOC_14947.	transcript	0.013992	0.014476	0.020052	0.022873	0.324441	0.020479	0.021029	0.012407	0.005886
Novel_014103	XLOC_14949.	transcript	0.589964	0	0	0.219718	0	0	0.404234	0.410825	0.231744
Novel_014104	XLOC_14952.	transcript	0	0	0.037109	0.061403	0.030294	0	0.033462	0.011762	0.183834
Novel_014105	XLOC_14952.	transcript	0.103206	0	0	0.081446	0	0.050599	0	0	0
Novel_014106	XLOC_14952.	transcript	0.103566	0	0.20899	0.040434	0.085337	0.058973	0.096745	0.102223	0.01382
Novel_014107	XLOC_14952.	transcript	0	0	0	0.039879	0	0.043414	0.074308	0	0
Novel_014108	XLOC_14952.	transcript	0	0	0	0	0.05372	0	0.072209	0	0.010105
Novel_014109	XLOC_14952.	transcript	0	0	0.028239	0.135085	0	0.099029	0.091389	0.017379	0
Novel_014110	XLOC_14952.	transcript	0	0	0	0	0	0	0	0	0
Novel_014111	XLOC_14952.	transcript	0.008796	0.003995	0.044251	0.00194	0	0.002004	0.014799	0.003745	0
Novel_014112	XLOC_14952.	transcript	0	0	0.21579	0.051252	0	0.076303	0.073177	0.086816	0
Novel_014113	XLOC_14957.	transcript	0.077672	0.126104	0.012168	0.027556	0.031945	0.055545	0.07833	0.028167	0.01364
Novel_014114	XLOC_14958.	transcript	0.132443	0.52591	0.369298	0.224373	0.412378	0.550752	0.227612	0.408347	1.728983
Novel_014115	XLOC_14958.	transcript	0.458758	0.478329	0.402211	0.477275	0.426862	0.358854	0.611717	0.390873	0.533437
Novel_014116	XLOC_14958.	transcript	0	0	0	0	0	0	0	0	0
Novel_014117	XLOC_14963.	transcript	0.424197	0.117389	0.25247	0.0947	0.272812	0.183288	0.444894	0.330862	0.075032
Novel_014118	XLOC_14965.	transcript	0.326291	0.023138	0.305305	0.215203	0	0.326662	0.481134	0.227606	0.146982
Novel_014119	XLOC_14968.	transcript	0.350004	1.25914	0.313058	0.30567	0.404066	0.357032	0.450635	0.400458	0.208341
Novel_014120	XLOC_14970.	transcript	0.506067	0.896574	0.521337	0.788333	0.659277	0.850089	0.827912	1.072077	0.562483
Novel_014121	XLOC_14970.	transcript	0.247644	0	0.163633	0	0.150705	0.071709	0.153185	0.017723	0
Novel_014122	XLOC_14971.	transcript	0.132091	0	0.139393	0.064546	0.283435	0.199626	0.116654	0.146354	0.07107
Novel_014123	XLOC_14971.	transcript	0.165381	0.202932	0.284672	0.360044	0.293657	0.212724	0.282226	0.415404	0.1506
Novel_014124	XLOC_14971.	transcript	0.041795	0.044239	0	0.012852	0.003146	0.079144	0.106088	0.009637	0.007416

Novel_014125	XLOC_14972.	transcript	0.042678	0.114917	0	0	0	0.175685	0	0	0.031783
Novel_014126	XLOC_14972.	transcript	0.085035	0.150509	0.448202	0.602078	0.356097	0.264403	0.3468	0.514435	0.207149
Novel_014127	XLOC_14972.	transcript	0.017362	0.053799	0	0.442949	0.290334	0.354552	0.288746	0.381373	0.075094
Novel_014128	XLOC_14973.	transcript	0.144191	0.154463	0.24509	0.199667	0.049159	0.207243	0.186442	0.055037	0.107467
Novel_014129	XLOC_14973.	transcript	0.372914	1.225995	0.411281	0.565382	0.446894	0.536926	0.914951	0.58554	0.62527
Novel_014130	XLOC_14973.	transcript	0.85348	0.403714	0.709208	0.360848	0.729208	0.709815	0.643297	0.837321	0.337656
Novel_014131	XLOC_14974.	transcript	0.368154	0.353415	0.233281	0.477653	0.522432	0.492022	0.648571	0.554735	0.37391
Novel_014132	XLOC_14975.	transcript	0.594545	0.62401	0.568023	0.513758	0.663561	0.90662	0.569658	0.48492	0.445036
Novel_014133	XLOC_14976.	transcript	0.362587	0.447176	0.403157	0.486273	0.422252	0.614797	0.597466	0.728262	0.571955
Novel_014134	XLOC_14976.	transcript	0	0	0	0.072239	0	0.183805	0.003964	0.054461	0.136642
Novel_014135	XLOC_14976.	transcript	0	0	0.045614	0.123585	0.029419	0.015848	0.075449	0.032937	0.047724
Novel_014136	XLOC_14977.	transcript	0	0.225788	0	0	0	0	0.006214	0	0
Novel_014137	XLOC_14977.	transcript	0.263171	0.239819	0.355788	0.435403	0.345839	0.491669	0.669453	0.392875	0.481421
Novel_014138	XLOC_14977.	transcript	0.646075	1.282393	1.526198	1.897052	1.259648	1.967521	1.325592	1.620241	1.183111
Novel_014139	XLOC_14977.	transcript	143.3779	178.1733	179.7007	206.6175	169.0181	210.128	166.71	186.8519	201.3327
Novel_014140	XLOC_14977.	transcript	0.410405	0.788092	0.375162	0.543462	0.581452	0.477936	0.481865	0.503109	0.568105
Novel_014141	XLOC_14979.	transcript	0	0.091102	0.015841	0.041619	0.489839	0.008998	0.048445	0	0.094094
Novel_014142	XLOC_14979.	transcript	0.782254	0.586224	0.599498	0.680538	0.178028	0.734977	0.901343	0.819927	0.888748
Novel_014143	XLOC_14980.	transcript	0	0.164265	0.415352	0.22904	0.65433	0	0.292211	0.081598	0.181647
Novel_014144	XLOC_14980.	transcript	0.072565	0.096687	0.32861	0.13886	0	0.296797	0	0.14009	0.267663
Novel_014145	XLOC_14980.	transcript	0.565346	0.676206	0	0.529548	0.073317	0.375418	0.399084	0.738842	0
Novel_014146	XLOC_14981.	transcript	0.331898	2.013595	0.580852	0.446029	0.554987	0.76357	1.047339	1.497938	0.641299
Novel_014147	XLOC_14981.	transcript	0	0.013056	0	0.099074	0	0.157548	0	0	0
Novel_014148	XLOC_14981.	transcript	0.161504	0.232702	0.178855	0.153734	0.30571	0.076436	0.280988	0.363413	0.352542
Novel_014149	XLOC_14981.	transcript	0	0	0.054568	0	0	0.002519	0	0.002187	
Novel_014150	XLOC_14981.	transcript	0	0	0.066305	0	0.472368	0	0	0.019568	0
Novel_014151	XLOC_14981.	transcript	0.334379	0.260382	0.251201	0.49336	0.673982	0.360741	0.586954	0.59627	0.41372
Novel_014152	XLOC_14981.	transcript	0.18962	0.432073	0.24735	0.247757	0.214251	0.255711	0.36044	0.199765	0.664073
Novel_014153	XLOC_14982.	transcript	1.316899	1.426786	1.412292	1.604092	1.60796	1.342429	2.264437	1.595694	4.106133
Novel_014154	XLOC_14990.	transcript	0	0.339436	0	0	0	0	0	0	0.063091
Novel_014155	XLOC_15004.	transcript	0.495577	0	0.2209	0.326665	0.057054	0.02009	0.390642	0.045395	0.191671
Novel_014156	XLOC_15004.	transcript	0.117053	0	0.156063	0	0.219897	0.170548	0.042365	0.161126	0
Novel_014157	XLOC_15009.	transcript	0	0	0.034358	0	0	0.041939	0.037164	0.035802	0
Novel_014158	XLOC_15028.	transcript	0.001858	0	0.010078	0.012441	0.128145	0.012911	0.005264	0.007408	0.019841
Novel_014159	XLOC_15028.	transcript	0.312478	0.372263	0.442122	0.497152	0.294795	0.516481	0.501303	0.472191	0.325379
Novel_014160	XLOC_15028.	transcript	0.180336	0.164357	0.219252	0.074788	0.218137	0.021758	0.203701	0.0413	0.128375
Novel_014161	XLOC_15029.	transcript	0	0	0.128005	0	0	0	0	0	0
Novel_014162	XLOC_15029.	transcript	0.473231	0.04887	0.206839	0.243345	0	0	0.128051	0.27192	0.115788

Novel_014163	XLOC_15029.	transcript	0.042259	0.354522	0.277012	0.502066	0.45082	0.521147	0.464171	0.417142	0.262067
Novel_014164	XLOC_15029.	transcript	0.118542	0.11884	0.048483	0.062987	0.093639	0.045024	0.171881	0.052723	0.040657
Novel_014165	XLOC_15029.	transcript	0.030989	0.0395	0.015423	0.005585	0.00584	0.063702	0.111604	0.060219	0.05755
Novel_014166	XLOC_15029.	transcript	0.086682	0	0	0	0.065669	0.082842	0.05876	0.045596	0.01885
Novel_014167	XLOC_15029.	transcript	0.213256	0.040309	0.033257	0.035003	0.103697	0.126336	0.06799	0.108949	0.000248
Novel_014168	XLOC_15029.	transcript	0.095328	0.046782	0.105889	0	0.121752	0.048212	0.101945	0.061475	0
Novel_014169	XLOC_15029.	transcript	0.115976	0.043396	0	0.13841	0	0.010505	0.104319	0.057449	0.116114
Novel_014170	XLOC_15029.	transcript	0.007539	0.03341	0.007524	0	0	0	0.083867	0.139722	0.003435
Novel_014171	XLOC_15029.	transcript	0	0	0	0.002621	0	0.291296	0	0	0.198893
Novel_014172	XLOC_15029.	transcript	0.236299	0.205885	0.326744	0.259	0.361232	0.03107	0.001491	0.145262	0.009415
Novel_014173	XLOC_15029.	transcript	0	0	0	0	0	0	0.271088	0	0
Novel_014174	XLOC_15029.	transcript	0.122231	0.00701	0.035871	0.019714	0.010882	0.007054	0.136105	0.006989	0.001331
Novel_014175	XLOC_15029.	transcript	0.010331	0	0	0	0	0	0.079178	0.109245	0
Novel_014176	XLOC_15030.	transcript	0	0	0.297603	0.123988	0.225484	0.045006	0.123638	0.1578	0.143891
Novel_014177	XLOC_15030.	transcript	0.1652	0.03334	0.14185	0.287527	0.102352	0.338565	0.15272	0.23562	0.306488
Novel_014178	XLOC_15031.	transcript	0	0	0.007464	0	0	0	0	0	0
Novel_014179	XLOC_15031.	transcript	1.302423	2.976837	2.849463	1.64016	0.343683	3.159389	0.471309	0.563838	1.021114
Novel_014180	XLOC_15031.	transcript	0.314942	0.316101	0.012088	0.069915	0.242324	0	0.63195	0.003613	0
Novel_014181	XLOC_15031.	transcript	0.351182	0.622299	0.42518	0.553182	0.302818	0.444674	0.636892	0.221014	0.325946
Novel_014182	XLOC_15032.	transcript	0.634537	0.666477	0.525178	0.683986	0.588527	0.608642	0.560221	0.548511	0.778755
Novel_014183	XLOC_15033.	transcript	0.49055	0.126055	0.371733	0.295101	0.393965	0.477086	0.534032	0.339891	0.255347
Novel_014184	XLOC_15034.	transcript	0.004098	0.054682	0.022679	0.012286	0.01712	0.021537	0.025675	0.01938	0.009208
Novel_014185	XLOC_15034.	transcript	0.1994	0.399419	0.330205	0.255015	0.408861	0.344332	0.796541	0.332105	0.247342
Novel_014186	XLOC_15034.	transcript	0.382919	0	2.00E-06	0	0	0	1.00E-05	1.30E-05	0
Novel_014187	XLOC_15034.	transcript	0	0	0	0	0	0	0	0	0
Novel_014188	XLOC_15034.	transcript	0	0.58589	0.087612	0.102136	0.115903	0.166987	0.173727	0.19694	0.121126
Novel_014189	XLOC_15034.	transcript	0.412725	0.798039	0.426367	0.554201	0.922048	0.564031	0.48745	0.556166	0.587178
Novel_014190	XLOC_15035.	transcript	0.184856	0.20478	0.217061	0.111119	0.222265	0.078542	0.314	0.128625	0.18991
Novel_014191	XLOC_15036.	transcript	0.243267	0.068285	0.380553	0.332984	0.619552	0.659136	0.354655	0.26654	0.399058
Novel_014192	XLOC_15036.	transcript	0.12101	0.190607	0.370489	0.233291	0.463287	0.248979	0.466771	0.234525	0.175388
Novel_014193	XLOC_15036.	transcript	0.09911	0.338087	0.179393	0.177949	0.363828	0.238679	0.31833	0.409156	0.282226
Novel_014194	XLOC_15036.	transcript	1.050894	1.643617	1.480196	1.500068	1.394464	1.533029	1.806898	1.983538	1.93584
Novel_014195	XLOC_15037.	transcript	5.90E-05	0	0.084229	0.078666	0	0.36118	0.08565	0	0.036671
Novel_014196	XLOC_15037.	transcript	0.287004	1.159816	0.316855	0.248559	0.39543	0.104375	0.463401	0.537946	0.21926
Novel_014197	XLOC_15038.	transcript	0.320385	0.763174	0.472647	0.438381	0.432911	0.522472	0.557161	0.468622	0.602356
Novel_014198	XLOC_15039.	transcript	0.548596	0.976816	0.677167	0.732673	0.395175	0.792193	0.505205	0.968863	0.727036
Novel_014199	XLOC_15040.	transcript	0.200652	0.115475	0.157856	0.124777	0	0.077179	0.21036	0.527747	0.045013
Novel_014200	XLOC_15040.	transcript	0.248105	2.241242	0.765287	0.385808	0.621155	0.67024	0.733315	0.876776	0.514332

Novel_014201	XLOC_15040.	transcript	0.626336	0.989843	0.20988	0.377185	0.220916	0.581223	0.579832	0.40454	0.592609
Novel_014202	XLOC_15041.	transcript	0.411788	0.30654	0.226465	0.169326	0.46602	0.416294	0.478969	0.537961	0.218425
Novel_014203	XLOC_15041.	transcript	0	0.209602	0.065995	0.195771	0.079827	0.234379	0.023092	0.099095	0.046636
Novel_014204	XLOC_15041.	transcript	0.601267	0.607993	0.752246	0.740112	0.898365	0.605767	0.860087	0.862412	0.708479
Novel_014205	XLOC_15041.	transcript	0.164881	0.209691	0.195357	0.123432	0.233586	0.087179	0.282532	0.272176	0.198942
Novel_014206	XLOC_15041.	transcript	0.011607	0	0.017655	0.006096	0.031759	0.055046	0.061593	0	0.051267
Novel_014207	XLOC_15041.	transcript	0.203724	0.222081	0.173796	0	0	0	0.132693	0	0
Novel_014208	XLOC_15041.	transcript	0.292403	0.02069	0.170856	0	0.039024	0.277598	0.242884	0.118211	0.166852
Novel_014209	XLOC_15041.	transcript	0	0	0.036074	0	0.002493	0.03961	0	0	0
Novel_014210	XLOC_15041.	transcript	0.117291	0.40318	0.10924	0.243231	0.140837	0.125023	0.055508	0.18772	0.126716
Novel_014211	XLOC_15041.	transcript	0.188462	0.188462	0.235643	0.122202	0.22051	0.227377	0.348959	0.232594	0.250621
Novel_014212	XLOC_15041.	transcript	0.083925	0	0.124111	0.160824	0.058114	0.102599	0.08282	0.081284	0
Novel_014213	XLOC_15042.	transcript	0	0	0	0.030958	0	0	0	0	0
Novel_014214	XLOC_15042.	transcript	0.192967	0.223546	0.438182	0.200677	0.389004	0.280752	0.449025	0.207674	0.384425
Novel_014215	XLOC_15042.	transcript	2.627845	8.014694	4.594395	6.046315	5.08393	4.220298	5.160199	5.518749	4.525424
Novel_014216	XLOC_15043.	transcript	0.064073	0.136938	0.361697	0.06656	0.457785	0.379567	0.238572	0.1153	0.709002
Novel_014217	XLOC_15043.	transcript	0.537766	0.421875	0.351472	0.453925	0.204262	0.264401	0.368216	0.446685	0.138268
Novel_014218	XLOC_15043.	transcript	0	0	0	0	0.256563	0.109978	0.160847	0.21073	0
Novel_014219	XLOC_15043.	transcript	0.154683	0.590941	0.348572	0.375651	0.368009	0.224644	0.35039	0.278558	0.314096
Novel_014220	XLOC_15043.	transcript	0.321911	0.463517	0.296152	0.198374	0.247204	0.601148	0.201771	0.277228	0.28512
Novel_014221	XLOC_15043.	transcript	0.076672	0.004104	0.092242	0.094063	0.061816	0.030091	0.06275	0.103527	0.103304
Novel_014222	XLOC_15044.	transcript	0	0	0.8233	0	0	0	0	0	0
Novel_014223	XLOC_15044.	transcript	0	0.825897	0.134942	1.431412	0.944086	1.577408	1.002167	1.292694	0.313483
Novel_014224	XLOC_15044.	transcript	0	0.163061	0.366482	0.368959	0	0.149779	0.013831	0.180408	0.542362
Novel_014225	XLOC_15044.	transcript	0.029641	0	0	0	4.30E-05	0	0	0	0
Novel_014226	XLOC_15044.	transcript	0.234606	0.127834	0.006227	0.134111	0.411433	0.221005	0.184257	0.346914	0.186819
Novel_014227	XLOC_15044.	transcript	1.010998	0.072923	0.029222	0.095014	0.273247	0.170781	0.612462	0.063607	0.123161
Novel_014228	XLOC_15048.	transcript	0.080228	0.266413	0.212187	0.464628	0.399431	0.222258	0.292463	0.194711	0.11885
Novel_014229	XLOC_15049.	transcript	0	0.12899	0.191871	0.146621	0.154454	0.088932	0.702285	0.015568	0.109795
Novel_014230	XLOC_15049.	transcript	0.221775	0.484638	0.378846	0.48155	0.495333	0.569315	0.312554	0.485969	0.231412
Novel_014231	XLOC_15050.	transcript	0	0	0.052873	0	0	0	0.028006	0.014955	0.088906
Novel_014232	XLOC_15055.	transcript	0.743091	0.050581	0.639717	0.81197	0.5668	0.515548	1.252894	0.687867	0.209707
Novel_014233	XLOC_15064.	transcript	0.246244	0.404141	0.388872	0.852991	0.069709	0.429053	0.253512	0.003047	0.495587
Novel_014234	XLOC_15064.	transcript	0.108172	0.060479	0.110566	0.151919	0.147156	0.135774	0.297989	0.165873	0.064107
Novel_014235	XLOC_15064.	transcript	0	0	0	0	0	0	0.008972	0	0
Novel_014236	XLOC_15064.	transcript	0	0	0	0	0	0	0	0	0
Novel_014237	XLOC_15066.	transcript	0.326463	0.421214	0.261006	1.749593	0.06234	0.235606	0.168739	0.4095	0.224346
Novel_014238	XLOC_15067.	transcript	0.019956	0.539462	0	0.035672	0	0	0	0.438421	0.34459

Novel_014239	XLOC_15067.	transcript	0.359131	0	0.248067	0.32018	0.320455	0.402514	0.616031	0	0
Novel_014240	XLOC_15067.	transcript	0.014415	0	0.196878	0.044909	0.167148	0.033267	0.122011	0.116992	0.065355
Novel_014241	XLOC_15068.	transcript	0.190116	0.125776	0.300194	0.332738	0.301504	0.444288	0.336124	0.384824	0.429629
Novel_014242	XLOC_15068.	transcript	0.212525	0.443063	0.446162	0.465215	0.415652	0.367553	0.600642	0.317923	0.522999
Novel_014243	XLOC_15069.	transcript	0.155805	0.264674	0.095898	0.122702	0.185209	0.170227	0.3122	0.227387	0.143552
Novel_014244	XLOC_15069.	transcript	0.102622	0.315247	0.207946	0.221258	0.235208	0.288615	0.254519	0.311719	0.262236
Novel_014245	XLOC_15069.	transcript	0.044469	0.124936	0.054445	0.022486	0.022557	0.094731	0.055678	0.056003	0.082431
Novel_014246	XLOC_15069.	transcript	0.072671	0.307562	0.308893	0.335057	0.337785	0.254219	0.315818	0.29619	0.083581
Novel_014247	XLOC_15070.	transcript	0	0	0.737891	0	0.856652	0.708349	0.60313	0	0
Novel_014248	XLOC_15070.	transcript	0	0	0	0	0	0	0.682836	0	0
Novel_014249	XLOC_15070.	transcript	0.273752	0.214442	0.329452	0.146177	0.09322	0.214231	0.043049	0.380302	0.24076
Novel_014250	XLOC_15070.	transcript	0	0	0.030423	0	0	0	0	0	0
Novel_014251	XLOC_15070.	transcript	0	0	0	0	0	0	0	0	0
Novel_014252	XLOC_15070.	transcript	0	0.110683	0.017929	0.127433	0.025486	0	0.107076	0.035331	0
Novel_014253	XLOC_15070.	transcript	0	0	0	0	0.301908	0	0.203827	0	0
Novel_014254	XLOC_15071.	transcript	0.716666	0.986333	0.887419	1.282045	0.807062	0.50695	1.546354	1.319683	0.681746
Novel_014255	XLOC_15071.	transcript	0.247584	1.637788	1.149896	0.363973	1.20002	0.523521	0.226104	1.70728	0.702921
Novel_014256	XLOC_15072.	transcript	2.521782	1.118174	2.612282	1.824036	1.999983	2.765489	2.41484	2.433169	2.128276
Novel_014257	XLOC_15074.	transcript	0	0.102108	0.02695	0.017159	0.123488	0.101293	0.160311	0.090702	0.013731
Novel_014258	XLOC_15074.	transcript	0.000215	0	0.373495	0.074206	0.094932	0.128257	0.287648	0.167218	0
Novel_014259	XLOC_15074.	transcript	0.333237	0	0.122586	0.4917	0.19572	0	0.437315	0.520446	0.160334
Novel_014260	XLOC_15074.	transcript	0.399044	0.28222	0	0.353732	0.235222	0.424705	0.356864	0.332651	0.113594
Novel_014261	XLOC_15075.	transcript	1.087565	0.688277	1.137998	0.942508	1.026705	1.018797	1.095871	0.863184	0.504826
Novel_014262	XLOC_15079.	transcript	0.303928	0	0.046576	0.127543	0.143568	0.260525	0.188153	0.277311	0.034185
Novel_014263	XLOC_15082.	transcript	0.115393	0.010021	0.063781	0.067948	0.057218	0.097226	0.105423	0.060125	0.119352
Novel_014264	XLOC_15083.	transcript	0	0	0	0	0	0	0	0	0.125239
Novel_014265	XLOC_15084.	transcript	0	0.427225	0	0	0	0	0	0	0
Novel_014266	XLOC_15090.	transcript	0.593659	0.141118	0.671854	0.538347	0.638895	0.50253	0.61424	0.183405	0.488939
Novel_014267	XLOC_15090.	transcript	0	0.000286	0	0	0	0	0.239987	0.404004	0
Novel_014268	XLOC_15090.	transcript	0.022828	0	0	0.110591	0	0	0	0.053624	0
Novel_014269	XLOC_15090.	transcript	0	0	0	0	0	0	0.045865	0.222697	0
Novel_014270	XLOC_15090.	transcript	0	0	0.092538	0.005634	0.070611	0	0	0.018525	0.017407
Novel_014271	XLOC_15090.	transcript	0.131901	0.19406	0	0	0	0.113036	0.017029	0	0
Novel_014272	XLOC_15090.	transcript	0	0.339714	0.012302	0.022494	0	0.033146	0.012081	0	0
Novel_014273	XLOC_15090.	transcript	0	0.690155	0	0	0	0	0	0	0
Novel_014274	XLOC_15095.	transcript	0.581303	0	0.251411	0.499744	0	0.226359	0.394056	0.170942	0
Novel_014275	XLOC_15095.	transcript	0.260422	0.173388	0.150671	0.052669	0.136737	0.144952	0.119249	0.066409	0.108242
Novel_014276	XLOC_15095.	transcript	0.265831	0.237583	0.092312	0.245207	0.108538	0.204818	0.203435	0.238176	0.136745

Novel_014277	XLOC_15095.		transcript	0.083746	0.197892	0.035553	0.037835	0.068365	0.079869	0.121261	0.208012	0.062535
Novel_014278	XLOC_15095.		transcript	0	0	0	0	0	0	0.007312	0	0
Novel_014279	XLOC_15095.		transcript	0	0	0	0	0	0	0	0	0
Novel_014280	XLOC_15095.		transcript	0	0	0.014854	0	0	0	0	0	0
Novel_014281	XLOC_15098.		transcript	0.044039	0.036864	0	0	0	0	0.145656	0.063598	0
Novel_014282	XLOC_15098.		transcript	0.201685	0.454563	0.235458	0.304951	0.430218	0.383746	0.225698	0.387163	0.249348
Novel_014283	XLOC_15098.		transcript	0	0	0	0	0	0	0	0	0
Novel_014284	XLOC_15098.		transcript	0	0	0.007432	0.138006	0	0	0.13957	0	0.013091
Novel_014285	XLOC_15098.		transcript	0.010034	0.204232	0	4.00E-06	0.082855	0.09266	0.059992	3.00E-06	0.000137
Novel_014286	XLOC_15098.		transcript	0	0	0	0.002108	0.134294	0.098636	0.092125	0.174215	0.14312
Novel_014287	XLOC_15098.		transcript	0.147786	0.054961	0.042644	0.011311	0	0.018125	0.009699	0	0.059216
Novel_014288	XLOC_15098.		transcript	0.371308	0.216929	0.439205	0.250238	0.220102	0.146592	0.133363	0.105191	0.341565
Novel_014289	XLOC_15098.		transcript	0	0	0.018458	0	0	0	0.08715	0	0
Novel_014290	XLOC_15098.		transcript	0	0	0	0	0	0	0	0	0
Novel_014291	XLOC_15111.		transcript	0.01175	0	0.043756	0.035426	0	0.006415	0	0	0
Novel_014292	XLOC_15111.		transcript	0.174811	0	0.166658	0.186315	0.249321	0.005818	0.143394	1.00172	0
Novel_014293	XLOC_15111.		transcript	0.164218	0	0	0.528016	0	0	0	0	0.274279
Novel_014294	XLOC_15111.		transcript	0	0	0	0.304926	0	0.189317	0	0	0
Novel_014295	XLOC_15111.		transcript	0.190173	0.004205	0.174032	0.199878	0.054405	0.167432	0.197327	0.175015	0.198251
Novel_014296	XLOC_15111.		transcript	0.243916	0.023807	0.108669	0.191409	0.010581	0.030771	0.005894	0.003894	0.034742
Novel_014297	XLOC_15111.		transcript	0	0	0	0	0	0.376828	0	0	0
Novel_014298	XLOC_15111.		transcript	0.028815	0	0.227472	0.047495	0.481167	0.005135	0.147948	0.06763	0.289749
Novel_014299	XLOC_15111.		transcript	0	0	0	0	0	0.026086	0.186179	0.160717	0
Novel_014300	XLOC_15114.		transcript	0.101244	0	0.033118	0.343129	0.075774	0.044279	0.121142	0.285129	0.10863
Novel_014301	XLOC_15127.		transcript	0.296857	0.005851	0.088498	0.10692	0.213296	0.118483	0.307872	0.055045	0.079617
Novel_014302	XLOC_15127.		transcript	0	0.242326	0	0	0	0	0	0	0
Novel_014303	XLOC_15127.		transcript	0.004756	0.132866	0.89931	0.021501	0.037917	1.069332	0.010608	0.618561	0
Novel_014304	XLOC_15127.		transcript	0.441076	0.528812	0	0.419447	0.423319	0.037323	0.429304	0.030295	0.343009
Novel_014305	XLOC_15127.		transcript	0.010932	0	0.006001	0.254206	0	0.01504	0	0	0.061806
Novel_014306	XLOC_15127.		transcript	0	0	0.419532	0	0	0	0	0	0
Novel_014307	XLOC_15127.		transcript	0	0	0	0	0	0	0	0	0
Novel_014308	XLOC_15127.		transcript	0	0.552889	0.080906	0.139985	0	0.211871	0.254364	0.310757	0
Novel_014309	XLOC_15130.		transcript	0.436497	0.647922	0.411853	0.397865	0.598773	0.293301	0.455485	0.394357	0.473597
Novel_014310	XLOC_15139.		transcript	0.239678	0	0.312352	0	0.224485	0	0	0	0
Novel_014311	XLOC_15139.		transcript	0.171565	0	0	0.069621	0.019929	0.107359	0.136457	0.08053	0.026009
Novel_014312	XLOC_15139.		transcript	0.022574	0	0.00128	0.015954	0	0	0	0	0
Novel_014313	XLOC_15139.		transcript	0.219536	0	0	0	0	0	0	0	0
Novel_014314	XLOC_15139.		transcript	1.719869	0	0.042099	0.288742	0.670159	0.038794	0.040689	0.094944	0

Novel_014315	XLOC_15139.	transcript	0	0	0.774958	0.194083	0	0.385177	0.46477	0.690795	0.104088
Novel_014316	XLOC_15139.	transcript	1.505973	0	0.417791	0.14948	0.282001	0.158607	0.270062	0.326495	0.030296
Novel_014317	XLOC_15139.	transcript	0	0	0	0	0	0.019445	0	0.009292	0
Novel_014318	XLOC_15178.	transcript	0.367479	0.346408	0.277198	0.380785	0.361414	0.243027	0.367423	0.387036	0.256621
Novel_014319	XLOC_15178.	transcript	0	0	0	0.009382	0.316121	0	0.523451	0.278265	0
Novel_014320	XLOC_15178.	transcript	0	0.007436	0	0	0.138141	0	0.212308	0.175965	0
Novel_014321	XLOC_15178.	transcript	0.269478	0.053517	0.106451	0.112422	0.133257	0.059396	0.263011	0	0.06972
Novel_014322	XLOC_15178.	transcript	0	0.068742	0	0.031727	0.002882	0	0.025561	0.009432	0.094414
Novel_014323	XLOC_15178.	transcript	0.884256	0.002425	0.465083	0.700454	0.649466	0.3155	0.563555	0.387256	0.122215
Novel_014324	XLOC_15178.	transcript	0.319302	0.460737	0.23929	0.035843	0.207415	0.295828	0.165021	0.174068	0.234294
Novel_014325	XLOC_15178.	transcript	0	0.000425	0	0	0	0	0	0	0
Novel_014326	XLOC_15178.	transcript	0	0	0	0	0	0	0	0	0.220151
Novel_014327	XLOC_15180.	transcript	0.170301	0.141159	0.238534	0.015949	0.039452	0	0.421766	0.058494	0.147885
Novel_014328	XLOC_15180.	transcript	0.099067	0.067396	0.064584	0.147955	0.162068	0.106618	0.164572	0	0.117535
Novel_014329	XLOC_15182.	transcript	0.161927	0.217133	0.380843	0.358645	0.220229	0.173743	0.222636	0.204345	0.317587
Novel_014330	XLOC_15185.	transcript	0.284562	0.106161	0	0.057553	0.104011	0	0.270627	0.174761	0
Novel_014331	XLOC_15185.	transcript	0	0.099531	0	0	0	0	0	0	0
Novel_014332	XLOC_15185.	transcript	0	0	0.032647	0.000971	0	0	0	0	0.011389
Novel_014333	XLOC_15185.	transcript	0.005664	0.010671	0.124714	0.047553	0.095956	0.167823	0.011247	0.032073	0.102842
Novel_014334	XLOC_15188.	transcript	0.249694	0.109327	0.047899	0.075923	0.099639	0.090019	0.100387	0.093037	0.035886
Novel_014335	XLOC_15193.	transcript	0.002751	0.155812	0	0.735567	0	0.463519	0.004199	0	0.358614
Novel_014336	XLOC_15193.	transcript	0.681514	0.071572	0.743747	0.151566	0.769581	0.300771	0.979836	0.849321	0.314618
Novel_014337	XLOC_15194.	transcript	1.058362	1.659131	1.023728	1.40112	1.300795	1.198341	1.641899	1.759002	1.17041
Novel_014338	XLOC_15194.	transcript	0	0	0	0	0	0	0	0	0.63313
Novel_014339	XLOC_15211.	transcript	0.079684	0.046749	0.492132	0.058504	0.360223	0.355233	0.165935	0.172054	0
Novel_014340	XLOC_15211.	transcript	0.639578	0.243255	0.536015	0.509602	0.291348	0.299734	0.802919	0.69896	0.441979
Novel_014341	XLOC_15212.	transcript	0	0.027532	0.035179	0.031932	0.026757	0	0	0.190032	0
Novel_014342	XLOC_15213.	transcript	0.185432	0.112794	0.294173	0.444212	0.2617	0.575668	0.317829	0.527208	0.184858
Novel_014343	XLOC_15218.	transcript	0.197479	0.096129	0.063536	0.201985	0.409169	0.211246	0.29368	0.224998	0.079803
Novel_014344	XLOC_15218.	transcript	1.95534	1.192196	0.972964	1.377529	1.529331	1.354277	2.126561	1.843258	0.947345
Novel_014345	XLOC_15220.	transcript	0	0	0	0	0	0	0	0.03302	0
Novel_014346	XLOC_15220.	transcript	0.163969	0	0.176146	0.132156	0.334559	0.752732	0.177051	0.090345	0.137614
Novel_014347	XLOC_15220.	transcript	0	0.106117	0	0	0	0	0	0	0
Novel_014348	XLOC_15220.	transcript	0	0	0	0	0	0	0	0	0
Novel_014349	XLOC_15220.	transcript	2.024718	0.114212	1.17934	1.069269	1.254396	0.656465	2.863456	1.77769	1.112168
Novel_014350	XLOC_15220.	transcript	0.017986	0	0.256975	0.007261	0.212139	0.037241	0.006623	0	0.007177
Novel_014351	XLOC_15223.	transcript	0.002314	0	0	0.080449	0.002147	0	0	0.176491	0
Novel_014352	XLOC_15223.	transcript	0.161713	0.059569	0.260597	0.161337	0.167868	0.193758	0.370776	0.142225	0.264257

Novel_014353	XLOC_15229.	transcript	0.358062	0	0	0	0	0	0.275452	0.298813	0
Novel_014354	XLOC_15229.	transcript	0	0	0	0	0	0	0.094315	0.092307	0
Novel_014355	XLOC_15229.	transcript	0.195518	0.236374	0.211013	0.352292	3.20E-05	0.208216	0.161054	0.202061	0.265579
Novel_014356	XLOC_15229.	transcript	0.014484	0	0.310947	0.100436	0	0.069702	0.168564	0.1512	0.054256
Novel_014357	XLOC_15229.	transcript	0	0.100383	0	0.050756	0	0	0	0.05201	0
Novel_014358	XLOC_15229.	transcript	0	0.123145	0.139239	0.080364	0.071963	0.021241	0.089049	0.098208	0.083455
Novel_014359	XLOC_15230.	transcript	0.22474	0	0.107478	0	0.091164	0.002217	0.073604	0.12693	0.062685
Novel_014360	XLOC_15230.	transcript	0.430917	0	0.376519	0.12504	0.725819	0.504534	0.118947	1.16314	0.361468
Novel_014361	XLOC_15230.	transcript	0.445442	0	0.498116	0.396664	0.412853	0.814857	0.677958	0.328263	0.463326
Novel_014362	XLOC_15230.	transcript	0.450451	0.060799	0.705848	0.882436	0.489308	0.740323	1.170904	0.498157	0.661889
Novel_014363	XLOC_15230.	transcript	0.005065	0.11114	0.003498	0.001836	0	0.003019	0.005161	0.01828	0
Novel_014364	XLOC_15230.	transcript	0	0	0.067438	0.102996	0	0	0	0	0
Novel_014365	XLOC_15232.	transcript	0.053438	1.219719	0.050137	0.029079	0.023658	0	0	0	0.068532
Novel_014366	XLOC_15232.	transcript	0.345813	0.787798	0.53266	0.556314	0.557266	0.7106	0.816285	0.484527	0.460498
Novel_014367	XLOC_15232.	transcript	0.117924	0.388851	5.20E-05	0	0.1493	0	0	0	0.139795
Novel_014368	XLOC_15232.	transcript	0	0.011365	0.170504	0.128473	0	0.087735	0.214715	0.148277	0
Novel_014369	XLOC_15232.	transcript	0.024419	0	0.196882	0	0.097239	0.029463	0	0.113155	0
Novel_014370	XLOC_15237.	transcript	0.559562	0.590153	0.567499	0.578787	0.562624	0.481597	0.373766	0.729335	0.537648
Novel_014371	XLOC_15238.	transcript	0	0	0	0.04269	0	0	0	0.211492	0.050637
Novel_014372	XLOC_15239.	transcript	0	0	0	3.03069	0	0	0	0	0
Novel_014373	XLOC_15239.	transcript	0.021133	2.584847	5.399196	3.291952	5.328294	7.714181	4.701022	4.353048	6.964306
Novel_014374	XLOC_15239.	transcript	2.034533	0	0	0	0	0	0	0	0
Novel_014375	XLOC_15239.	transcript	1.520272	0	0	0	0	0	0	0	0
Novel_014376	XLOC_15239.	transcript	1.236162	0.001435	0.002496	0.000194	0.002051	0	0.001375	0.001008	0
Novel_014377	XLOC_15239.	transcript	0	0	0	0	0	0	0	0	0
Novel_014378	XLOC_15239.	transcript	0	0	0	0.518541	0.023845	0	0.040336	0.627316	0.515639
Novel_014379	XLOC_15240.	transcript	0	0.137583	0	0	0	0	0.002674	0	0
Novel_014380	XLOC_15240.	transcript	0	0.008744	0	0	0.344415	0.001737	0	0	0.243769
Novel_014381	XLOC_15240.	transcript	0.617278	0.722662	0.425356	0.582936	0.309965	0.641513	0.485429	0.521152	0.030589
Novel_014382	XLOC_15240.	transcript	0.040134	0.056742	0	0	0	0	0	0	0
Novel_014383	XLOC_15240.	transcript	0	0	0.007386	0	0	0	0	0	0
Novel_014384	XLOC_15240.	transcript	0	0	0.028628	0.312735	0.164414	0.04443	0.032285	0	0.256656
Novel_014385	XLOC_15240.	transcript	0.069354	0	0.035693	0.108406	0.12497	0.130636	0.112596	0.22776	0.006475
Novel_014386	XLOC_15240.	transcript	0.005872	0.043977	0.612861	0.523482	0.326626	0.469305	0.060362	0.208952	0.13931
Novel_014387	XLOC_15240.	transcript	1.435328	0.089638	1.713848	1.621267	1.651248	1.822026	1.724955	1.956189	1.044822
Novel_014388	XLOC_15241.	transcript	0.033262	0.072643	0.017196	0.016557	0.015828	0.163746	0.027088	0.106436	0
Novel_014389	XLOC_15243.	transcript	0	0	0	0	0	0	0	0	0
Novel_014390	XLOC_15243.	transcript	0.000421	0.00849	0.008216	0	0.016437	0	0	0.004941	0

Novel_014391	XLOC_15243.	transcript	0.293169	0.222515	0.220165	0.317136	0.158191	0.199179	0.246263	0.242474	0.139673
Novel_014392	XLOC_15250.	transcript	0.031567	0.031692	0.132778	0.236391	0.24318	0.133345	0.045357	0.014321	0.137372
Novel_014393	XLOC_15250.	transcript	0.022142	0.052457	0.049934	0.098297	0.0972	0.078029	0.020087	0.004371	0.235364
Novel_014394	XLOC_15250.	transcript	0.039138	0.08306	0.068648	0	0	0.01329	0	0.111983	0
Novel_014395	XLOC_15250.	transcript	0.029383	0.048899	0.061674	0.237631	0.112551	0.119518	0.024511	0.006704	0.094956
Novel_014396	XLOC_15250.	transcript	0	0	0.112617	0	0	0	0	0.010264	0.08297
Novel_014397	XLOC_15250.	transcript	0	0.016475	0	0	0	0	0.001328	0	0.075811
Novel_014398	XLOC_15250.	transcript	0.037101	0.075356	0.086375	0	0	0	0.026897	0	0
Novel_014399	XLOC_15250.	transcript	0.020766	0.014075	0.044621	0.04852	0.065692	0.019298	0.041403	0.006433	0.056799
Novel_014400	XLOC_15250.	transcript	0	0	0.044583	0	0	0	0	0.021693	0.058485
Novel_014401	XLOC_15250.	transcript	0.003542	0.017158	0.135112	0	0.094612	0.163255	0.001105	0.000979	0.075933
Novel_014402	XLOC_15250.	transcript	0.002619	0.005827	0.080602	0	0.124023	0.042992	0.001184	0.001319	0.047229
Novel_014403	XLOC_15250.	transcript	0.100256	0.321747	0.023843	0.024832	0	0.017465	0.091468	0.050879	0.010024
Novel_014404	XLOC_15250.	transcript	0.005121	0.006742	0.173739	0	0.175398	0	0.001814	0.012949	0.083784
Novel_014405	XLOC_15250.	transcript	0.006218	0.013381	0.133232	0	0.199059	0.246171	0.002248	0.006577	0.113792
Novel_014406	XLOC_15250.	transcript	0	0	0	0	0	0	0	0	0
Novel_014407	XLOC_15250.	transcript	0	0	0	0	0	0	0	0	0
Novel_014408	XLOC_15250.	transcript	0.023115	0.031859	0	0	0	0	0.031482	0	0
Novel_014409	XLOC_15250.	transcript	0.253698	0.629801	0.044652	0.006128	0.011884	0.011216	0.315042	0.244726	0.018675
Novel_014410	XLOC_15250.	transcript	0.06668	0.274902	0.09351	0.040981	0.00161	0.021978	0.220451	0.122883	0.113413
Novel_014411	XLOC_15250.	transcript	0.015132	0.045585	0.008653	0	0.024929	0.030847	0.030497	0.032977	0.005781
Novel_014412	XLOC_15250.	transcript	0.124474	0.160943	0.122103	0.403711	0.114555	0.120444	0.247122	0.110831	0.132593
Novel_014413	XLOC_15250.	transcript	0.04501	0.019524	0.017422	0.040276	0.01456	0.017953	0.071165	0.099353	0.022707
Novel_014414	XLOC_15250.	transcript	0.815574	0.446391	0.566105	0.663368	0.354025	0.425621	0.741582	0.528642	0.351408
Novel_014415	XLOC_15250.	transcript	0.062437	0.052023	0.093713	0.095011	0.006417	0.076121	0.093162	0.05376	0.041126
Novel_014416	XLOC_15257.	transcript	1.713345	0.193189	1.587312	1.226234	1.23282	0.419072	0.12397	1.414438	1.418591
Novel_014417	XLOC_15257.	transcript	0	0	0	0	0	0	0	0	0
Novel_014418	XLOC_15257.	transcript	0	2.180825	0.00079	0.51439	0.576372	1.457041	1.786821	0.966995	0
Novel_014419	XLOC_15258.	transcript	0.037404	0	0.004765	0	0.155908	0.002548	0	0.078311	0
Novel_014420	XLOC_15258.	transcript	0.185494	0.200869	0.172835	0.311648	0.273291	0.340946	0.368073	0.25755	0.188997
Novel_014421	XLOC_15258.	transcript	0.344647	0.578121	0.388968	0.405428	0.443882	0.332315	0.610221	0.455917	0.189791
Novel_014422	XLOC_15258.	transcript	0	0	0	0	0	0	0	0	0.205807
Novel_014423	XLOC_15258.	transcript	0.002494	0.001052	0	0.089713	0	0	0.002517	0.147696	0
Novel_014424	XLOC_15258.	transcript	0.008124	0.000876	0.206303	0	0	0	3.20E-05	0.050372	0
Novel_014425	XLOC_15258.	transcript	0.002421	0.001089	0	0.071656	0.006388	0	0.001879	0.157869	0
Novel_014426	XLOC_15258.	transcript	0.002519	0.001006	0	0.121926	0	0.28828	0.001466	0.088219	0
Novel_014427	XLOC_15258.	transcript	0.704413	1.095979	0.108255	0.085869	0.502289	0	0.177438	0.19057	0.218693
Novel_014428	XLOC_15258.	transcript	0.438426	0.46989	0.125227	0.189096	0.174671	0.215334	0.086795	0.138363	0.104067

Novel_014429	XLOC_15258.	transcript	0.128495	0.165262	0.111868	0.042417	0.20498	0.258889	0.159758	0.072141	0.239506
Novel_014430	XLOC_15258.	transcript	0.169081	0.104506	0.255077	2.60E-05	1.20E-05	0.014381	0.44104	0.082669	0.063197
Novel_014431	XLOC_15258.	transcript	0.15591	0.118106	0	0.099887	1.00E-06	0.094203	0.269397	0.05159	0
Novel_014432	XLOC_15258.	transcript	0.135374	0.101246	0.107148	0.006004	0.06192	0	0.197436	0.064492	0.058557
Novel_014433	XLOC_15258.	transcript	0	0	0	0	1.00E-06	0	0	0	0
Novel_014434	XLOC_15258.	transcript	0	0.15585	0.065272	0.075928	0.032108	0.070741	0.250493	0.120895	0
Novel_014435	XLOC_15259.	transcript	0.227064	0	0	0	0	0.029116	0.030749	0.024683	0
Novel_014436	XLOC_15259.	transcript	0.093925	0.321868	0.618651	0.212695	0.486872	0.401446	0.304722	0.404115	0.162466
Novel_014437	XLOC_15260.	transcript	0.007515	0.003758	0	0.006669	0	0.024204	0	0.013731	0
Novel_014438	XLOC_15260.	transcript	0.217534	0.212986	0.340689	0.463901	0.407628	0.341456	0.398067	0.405259	0.254407
Novel_014439	XLOC_15260.	transcript	2.223387	1.406229	2.008582	3.167825	1.795912	3.216625	3.891067	2.562535	2.056499
Novel_014440	XLOC_15260.	transcript	0.036283	0.462096	0.057539	0.17977	0.237195	0.076596	0.135408	0.114187	0.107357
Novel_014441	XLOC_15261.	transcript	0.151282	0.631701	0.103017	0.302403	0.392378	0.292759	0.36691	0.225573	0.253228
Novel_014442	XLOC_15261.	transcript	0	0	0	0	0	0.964596	0	0	0
Novel_014443	XLOC_15262.	transcript	0.245302	0.224311	0.287247	0.630373	0.011593	0.691318	0.267296	0.363767	0.465093
Novel_014444	XLOC_15262.	transcript	0.030475	0.00536	0.353591	0.061165	0.680432	0.059237	0.58617	0.494972	0.043833
Novel_014445	XLOC_15263.	transcript	1.325065	0.858786	1.581825	1.352769	1.480311	1.68792	1.373904	1.524246	1.438627
Novel_014446	XLOC_15263.	transcript	0	0.000567	0	0	0	0	0	0.659658	0
Novel_014447	XLOC_15263.	transcript	0.019343	0	0	0	0	0	0	0	0
Novel_014448	XLOC_15263.	transcript	0	0	0	0	0	0	0	0	0.439996
Novel_014449	XLOC_15263.	transcript	0.489838	0.675786	0.004946	0.588275	1.112835	1.302282	0.215116	0.647373	0.582382
Novel_014450	XLOC_15263.	transcript	0.288216	0.377636	0.583909	0.501534	0.528366	0.729804	0.049803	0.464871	0.328553
Novel_014451	XLOC_15263.	transcript	1.406997	0.758008	1.765813	1.183283	0.735574	0	1.97847	0.619063	0.510045
Novel_014452	XLOC_15263.	transcript	0	0.151518	0.275608	0	0	0.970486	0.362512	1.070745	0
Novel_014453	XLOC_15263.	transcript	0	0	0	0	0	0	0	0	0
Novel_014454	XLOC_15263.	transcript	0	0	0	0	0	0.238839	0	0	0
Novel_014455	XLOC_15263.	transcript	0	0.394552	0	0	0	0	0	0	0
Novel_014456	XLOC_15264.	transcript	0	0	0	0	0.116726	0	0	0	0.200138
Novel_014457	XLOC_15264.	transcript	0.038911	0.032249	0.095988	0	0.006809	0.093604	0.033952	0.01295	0.209515
Novel_014458	XLOC_15264.	transcript	0.272104	0.140674	0.277887	0.336919	0.298634	0.287891	0.402598	0.465284	0.275313
Novel_014459	XLOC_15265.	transcript	8.317031	6.931942	9.379057	9.963848	10.1291	10.76599	9.708156	9.873869	9.028995
Novel_014460	XLOC_15271.	transcript	0.888605	0.916175	0.68348	0.58514	0.858074	0.683051	1.14046	0.692042	1.411355
Novel_014461	XLOC_15271.	transcript	0	0	0	0	0	0	0	0.008849	0
Novel_014462	XLOC_15275.	transcript	0.179007	0	0.37879	0	0.228527	0.298053	0.649287	0.24183	0.461473
Novel_014463	XLOC_15280.	transcript	0.089967	0.038986	0.255556	0.114094	0.224705	0.03847	0.259871	0.152942	0.179546
Novel_014464	XLOC_15280.	transcript	0	0.097963	0	0.152718	0	0	0	0	0
Novel_014465	XLOC_15281.	transcript	0.56932	0.144083	0.236494	0.229541	0.28314	0.201697	0.323093	0.174448	0.423529
Novel_014466	XLOC_15281.	transcript	0	0.122277	0	0	0	0.181035	0	0	0

Novel_014467	XLOC_15288.	transcript	0.420025	0.735874	1.549727	1.985788	1.8241	0.379412	1.768657	1.647284	1.243742
Novel_014468	XLOC_15288.	transcript	1.010262	0	0	0	0	1.218616	0	0	0
Novel_014469	XLOC_15289.	transcript	0	0	0	0.011626	0.041473	0	0.131934	0.013673	0.19514
Novel_014470	XLOC_15289.	transcript	0.169583	0.449172	0.38201	0.312218	0.264859	0.275486	0.288978	0	0.074937
Novel_014471	XLOC_15289.	transcript	0.011995	0.044038	0.054196	0.014882	0	0.077184	0	0.364315	0.088077
Novel_014472	XLOC_15290.	transcript	0.50019	0.594718	0.468406	0.135968	0.427464	0	0.570016	0.212178	0.350114
Novel_014473	XLOC_15290.	transcript	0	0	0	0	0	0	0	0	0
Novel_014474	XLOC_15290.	transcript	0.032655	0.110668	0.045019	0.229935	0.184275	0.19052	0.009434	0.090274	0.007838
Novel_014475	XLOC_15290.	transcript	0	0.006747	0	0	0	0.447975	0	0.004859	0
Novel_014476	XLOC_15291.	transcript	0.662485	3.056092	0.589373	0.754886	0.907847	1.060421	1.361129	0.810281	2.183657
Novel_014477	XLOC_15295.	transcript	0.588343	0.372244	0.299228	0.193407	0.371867	0.594531	0.563847	0.385773	0.198499
Novel_014478	XLOC_15296.	transcript	0.07452	3.104328	0.416002	0.446953	0.337881	0	0.291341	0	0.519555
Novel_014479	XLOC_15303.	transcript	0.275784	0.128166	0.231111	0.283589	0.22638	0.207252	0.246582	0.103125	0.150949
Novel_014480	XLOC_15303.	transcript	0	0	0	0	0	0	0	0	0
Novel_014481	XLOC_15308.	transcript	0	0	0	0	0	0	0	0	0
Novel_014482	XLOC_15308.	transcript	0	0	0	0	0	0	0.267413	0	0
Novel_014483	XLOC_15308.	transcript	0	0	0.014087	0.057123	0	0	0.408555	0	0
Novel_014484	XLOC_15308.	transcript	0.334147	0.657158	0.5351	0.569056	0.690151	0.09727	0.622753	0.398205	0.421147
Novel_014485	XLOC_15308.	transcript	0.749979	0.300544	0.634471	0.809195	0.717503	1.301351	0.486741	0.738279	0.870674
Novel_014486	XLOC_15311.	transcript	0.184024	0.562282	0.209659	0.377139	0.247374	0.496586	0.490687	0.487545	0.248691
Novel_014487	XLOC_15311.	transcript	0.304713	1.039075	0.615646	0.700108	0.682459	0.847412	0.774592	0.733694	0.391211
Novel_014488	XLOC_15312.	transcript	0.750193	0.053025	0.866543	0.410161	1.012648	0.956424	0.843848	0.477012	0.62016
Novel_014489	XLOC_15313.	transcript	0.333388	0.033925	0.432335	0.299975	0.271402	0.365161	0.504068	0.189458	0.361578
Novel_014490	XLOC_15314.	transcript	0.369118	0.478287	1.094379	1.324978	1.053328	1.255378	0.580505	0.975347	0.911042
Novel_014491	XLOC_15315.	transcript	0.167689	0.560018	0.130446	0.091785	0.173214	0.092556	0.321438	0.168709	0.221382
Novel_014492	XLOC_15316.	transcript	0	0.066544	0.021239	0.15114	0.041094	0.240283	0	0.060803	0.117692
Novel_014493	XLOC_15319.	transcript	0	0.737722	0	0.475451	0.537241	0.015101	0.876733	0.381575	0.070586
Novel_014494	XLOC_15319.	transcript	0.411545	0	0.469463	0	0.002226	0.636744	0	0.382756	0.444308
Novel_014495	XLOC_15320.	transcript	0.204142	0.06484	0.356279	0	0	0.008903	0.171482	0.145469	0.184849
Novel_014496	XLOC_15326.	transcript	0.321984	0.435198	0.412159	0.435834	0.247055	0.357677	0.192775	0.308666	0.351753
Novel_014497	XLOC_15326.	transcript	0	0	0	0	0	0	0.178981	0	0
Novel_014498	XLOC_15328.	transcript	0.58038	0.031865	1.160107	0.637496	1.06792	0.498519	1.757388	0.71975	0.734684
Novel_014499	XLOC_15329.	transcript	0.08526	0.161385	0.034452	0.113924	0	0.137598	0.150624	0.066402	0.75523
Novel_014500	XLOC_15329.	transcript	0	0	0	0	0	0	0	0	0
Novel_014501	XLOC_15329.	transcript	0.051502	1.469767	0	0.033697	0	0.070894	0.300361	0	0.095253
Novel_014502	XLOC_15329.	transcript	0	0.267596	0	0.008752	0.18663	0.014591	0	0	0.041319
Novel_014503	XLOC_15329.	transcript	0	0	0	0	0	0	0	0	0
Novel_014504	XLOC_15329.	transcript	0	0	0	0	0	0	0	0	0.048798

Novel_014505	XLOC_15329.	transcript	0.033983	0	0.106542	0	0	0.131691	0	0.248436	0.931492
Novel_014506	XLOC_15329.	transcript	0.278262	0.285124	0	0.117069	0.113777	0.245487	0.117216	0.201451	0
Novel_014507	XLOC_15329.	transcript	0.22612	1.013707	0	0.455715	0.556682	0.237151	0.478043	0	0.013777
Novel_014508	XLOC_15329.	transcript	0.072665	0.188889	0.898863	0.061826	0.078066	0.052127	0.085145	0	0
Novel_014509	XLOC_15329.	transcript	0	0	0	0	0	0	0	0.162877	0
Novel_014510	XLOC_15330.	transcript	0.318397	0.349173	0.425291	0.649747	0.495456	0.375964	0.516639	0.369888	0.38534
Novel_014511	XLOC_15330.	transcript	0.045471	0.27999	0.143671	0.012263	0	0.032622	0	0.239957	0.353723
Novel_014512	XLOC_15335.	transcript	0.065964	0.375709	0.512083	0.571476	0.089866	0.099238	0.233538	0.010805	0.502674
Novel_014513	XLOC_15335.	transcript	0.009784	0	0.260701	0	0	0.447364	0.738518	0.714762	0.039192
Novel_014514	XLOC_15335.	transcript	0.711677	0.179961	0	0.077358	0.712372	0	0	0.173871	0
Novel_014515	XLOC_15336.	transcript	0.281528	0.125999	0.469006	0.336587	0.298268	0.193214	0.187168	0.289662	0.270747
Novel_014516	XLOC_15339.	transcript	0	0	0.010553	0.308269	0	0.05014	0	0.080839	0.056796
Novel_014517	XLOC_15341.	transcript	0.076227	0.057656	0.083603	0.099371	0.072566	0.152276	0.047314	0.081498	0.113149
Novel_014518	XLOC_15345.	transcript	2.203121	0.796331	2.892748	2.265268	2.056793	4.750607	2.181337	2.557847	5.327011
Novel_014519	XLOC_15345.	transcript	0	0.013995	0	0	0	0.248715	0	0.207364	0
Novel_014520	XLOC_15350.	transcript	0.126071	0.00641	0.038806	0.018463	0	0	0.035942	0.042017	0.053534
Novel_014521	XLOC_15350.	transcript	0	0	0	0	0.061572	0.03963	0.014149	0.07919	0
Novel_014522	XLOC_15350.	transcript	0	0.020124	0.005362	0.042898	0	0	0	0.040304	0.327867
Novel_014523	XLOC_15350.	transcript	0.149255	0.351815	0.227333	0.104477	0.117403	0.225036	0.305834	0.008012	0.145283
Novel_014524	XLOC_15350.	transcript	0	0.009724	0.019511	0	0.108668	0.089138	0	0	0
Novel_014525	XLOC_15357.	transcript	1.767117	2.053077	2.008964	1.367309	1.455204	1.356285	1.76774	2.081624	0.983197
Novel_014526	XLOC_15358.	transcript	0.270491	0	0.252561	0.126549	0	0	0	0.520077	0
Novel_014527	XLOC_15358.	transcript	0	6.50E-05	0.017616	0.032065	0.698719	0.592669	0.03216	0.36935	0.314681
Novel_014528	XLOC_15358.	transcript	0	0.460328	0	0.490639	0	0	0.679177	0	0
Novel_014529	XLOC_15361.	transcript	0	0	0.042367	0.047605	0	0	0.044007	0.038846	0.376927
Novel_014530	XLOC_15361.	transcript	0.00551	0	0.128479	0	0.477494	0	0.08861	0.114049	0.264191
Novel_014531	XLOC_15361.	transcript	0.030151	0	0.226714	0.267374	0	0.006897	0.423227	0.891211	0.184005
Novel_014532	XLOC_15361.	transcript	0.607341	0.243347	0	0.024094	0.178769	0.462212	0	0	0.000612
Novel_014533	XLOC_15361.	transcript	0	0	0.534192	0.273652	0	0	0.54786	0	0
Novel_014534	XLOC_15363.	transcript	0	0	0	0	0	0.40544	0	0	0
Novel_014535	XLOC_15363.	transcript	0.161923	0.065112	0.026269	0.241733	0.460264	0	0.040506	0.072339	0.333982
Novel_014536	XLOC_15365.	transcript	0.74062	0.986917	0.439642	0.667257	0.640322	0.647368	0.69398	0.638956	0.409678
Novel_014537	XLOC_15365.	transcript	0	0.185218	0.101866	0	0	0	0.243237	0.165812	0
Novel_014538	XLOC_15365.	transcript	0.068687	0.104283	0.07611	0	0.076824	0.055854	0.276672	0.115775	0.23221
Novel_014539	XLOC_15365.	transcript	0.070439	0	0.038065	0.000944	0	0.023076	0.096763	0	0.026935
Novel_014540	XLOC_15365.	transcript	0	0.112277	0.05455	0.285469	0.069526	0.055399	0.027343	0.066992	0.185639
Novel_014541	XLOC_15365.	transcript	0.608835	0.00694	0	0.983754	0.685147	0.661782	0.075041	0.630071	0.049616
Novel_014542	XLOC_15375.	transcript	0.090239	0.001981	0.06553	0.144833	0.127103	0.100905	0.181559	0.166966	0.087812

Novel_014543	XLOC_15375.	transcript	0.001963	0	0	0	0.88434	0.696549	0	0	0.002481
Novel_014544	XLOC_15375.	transcript	0.006488	0	0.02061	1.006207	0.003409	0	0.006964	0.015356	0.008871
Novel_014545	XLOC_15375.	transcript	0.001839	0	0	0.006615	0	0	0.003134	0.372893	0
Novel_014546	XLOC_15375.	transcript	0.078211	0	0.025909	0	0.047666	0	0	0.580609	0.057938
Novel_014547	XLOC_15375.	transcript	1.467645	0	0	0.901121	0.555426	0	0.091638	0	0.008274
Novel_014548	XLOC_15375.	transcript	0	0	0.006814	0.649066	0.007518	0	0	0.001087	0
Novel_014549	XLOC_15375.	transcript	0.122454	0.42426	1.954622	0.736771	0.000554	0	0.019469	0.000692	0.012982
Novel_014550	XLOC_15375.	transcript	0.000446	0	0	0.001337	0.00048	0.003226	0.00121	0.766887	0
Novel_014551	XLOC_15375.	transcript	0.006291	0	0.005859	0.005705	0.059204	0.032115	0.191315	0	0.025577
Novel_014552	XLOC_15375.	transcript	0.057293	0	0.305857	0.801211	0.001414	0	0	0.534594	0
Novel_014553	XLOC_15375.	transcript	0	0	0	0	0	0	0	0.752588	0
Novel_014554	XLOC_15375.	transcript	1.622079	0.004768	0.099551	0.946909	1.416859	1.363385	3.139307	0.046758	4.252062
Novel_014555	XLOC_15388.	transcript	0	0	0	0.026275	0.066229	0.025537	0.028661	0.073821	0
Novel_014556	XLOC_15397.	transcript	0.111999	0.017313	0.061248	0.175315	0.10451	0.024562	0.2361	0.065482	0.255557
Novel_014557	XLOC_15401.	transcript	0.336445	0	0.029641	0.046996	0.086028	0	0.122227	0	0.033227
Novel_014558	XLOC_15405.	transcript	0.327773	0.878008	0.312561	0.257226	0.621432	0.317929	0.443964	0.35546	0.577442
Novel_014559	XLOC_15405.	transcript	0.274117	0	0.309342	0.770347	0	0.517872	0.563439	0.430522	0.535017
Novel_014560	XLOC_15405.	transcript	0.257971	0.179021	0.54994	0.449165	0.158904	0.271531	0.30167	0.282306	0.519217
Novel_014561	XLOC_15406.	transcript	0.504397	0.527497	0.55704	0.616845	0.656874	0.775684	0.797652	0.585965	0.607683
Novel_014562	XLOC_15407.	transcript	2.073148	0.347256	2.595291	1.223162	1.907561	1.815957	2.395273	1.833243	1.308855
Novel_014563	XLOC_15408.	transcript	0.44321	0.227051	0	0	0.012039	0	0.036361	0.081334	0.007604
Novel_014564	XLOC_15408.	transcript	0	0	0.354277	0.016025	0.014962	0.014863	0.01573	0	0.012581
Novel_014565	XLOC_15408.	transcript	0.006179	0	0.374656	0.058091	0	0.03122	0.017039	0	0
Novel_014566	XLOC_15408.	transcript	0	0	0.244504	0	0.047643	0.029435	0.150006	0.66753	0.317415
Novel_014567	XLOC_15408.	transcript	0.299342	0.028876	0	0.040581	0	0	0.003544	0.011087	0
Novel_014568	XLOC_15408.	transcript	0	0	0	0	0.477259	0	0	0.328977	0
Novel_014569	XLOC_15408.	transcript	0	0	0	1.048047	0	0.790343	0.787646	0	0.679663
Novel_014570	XLOC_15409.	transcript	0.062988	0.327913	0.326878	0.167721	0.209077	0.346046	0.177192	0.183065	0.157357
Novel_014571	XLOC_15409.	transcript	0.080936	0.012322	0	0.027281	0.069463	0	0.096959	0.03615	0.110738
Novel_014572	XLOC_15410.	transcript	0.27684	0	0.999793	0.608194	0.413726	0.445355	0.981124	0.520384	0.231375
Novel_014573	XLOC_15410.	transcript	4.039557	5.708074	2.997407	5.571716	4.275591	4.572878	5.014063	6.028816	6.615067
Novel_014574	XLOC_15410.	transcript	0	0.362702	0.764153	0.606864	0.649998	0	0.706118	0.60718	0.866684
Novel_014575	XLOC_15411.	transcript	0.369851	0.305389	0.280807	0.551639	0.40437	0.359195	0.523065	0.414592	0.492935
Novel_014576	XLOC_15411.	transcript	0.018993	0.025169	0.090198	0.007971	0.086825	0.05713	0.012234	0.07876	0
Novel_014577	XLOC_15411.	transcript	0	0	0	0	0.505148	0	0	0.85666	0
Novel_014578	XLOC_15411.	transcript	0	0.254013	0	0.021441	0.057064	0.03562	0	0.062716	0.043797
Novel_014579	XLOC_15416.	transcript	0	0	1.144879	0	0	0.005452	0	0.005027	0
Novel_014580	XLOC_15416.	transcript	2.372123	1.247923	1.303844	1.721117	2.174665	2.509182	2.366236	1.432722	2.149946

Novel_014581	XLOC_15419.	transcript	0	0	0.191406	0.171542	0.094576	0.090049	0.103481	0.143687	0.171127
Novel_014582	XLOC_15424.	transcript	0.323033	0.694407	0.720275	0.634966	0.777683	0.960525	0.673314	0.884743	0.664629
Novel_014583	XLOC_15424.	transcript	0.19379	0.285586	0.116261	0.035739	0.403428	0.22168	0.362593	0.204949	0.034611
Novel_014584	XLOC_15424.	transcript	0.229016	0.663004	0.597186	0.955492	0.474539	0.615326	0.393207	0.285603	0.42802
Novel_014585	XLOC_15444.	transcript	0.993837	0.869813	0.842898	0.69393	0.708045	0.802008	1.237107	1.056423	0.710509
Novel_014586	XLOC_15444.	transcript	0.111416	1.867861	0.10451	0.115812	0.135343	0.113387	0.363595	0.108126	0.124846
Novel_014587	XLOC_15445.	transcript	0.591334	0.456123	0.318984	0.526139	0.223619	0.43921	0.599045	0.421894	0.291969
Novel_014588	XLOC_15445.	transcript	0	0	0	0	0	0	0	0	0
Novel_014589	XLOC_15445.	transcript	0	0.071902	0.213598	0.108627	0.103387	0	0.090403	0.130423	0.265839
Novel_014590	XLOC_15447.	transcript	0	0	0.519436	0.389583	0.263568	0	0	0	0
Novel_014591	XLOC_15447.	transcript	0.555938	0.011022	0.423335	0	0	0	0	0	0
Novel_014592	XLOC_15447.	transcript	0	0	0	0	0	0	0	0	0.130697
Novel_014593	XLOC_15447.	transcript	0	0	0	0.075255	0.518531	0.705083	1.350685	0.53066	0.145809
Novel_014594	XLOC_15448.	transcript	0.24202	0.052806	0.314057	0.413873	0.911212	0.828723	0.768561	0.585086	0.025919
Novel_014595	XLOC_15448.	transcript	0	0	0	0	0	0	0	0	0
Novel_014596	XLOC_15448.	transcript	0.029381	0	0	0.4257	0	0	0	0.157746	0
Novel_014597	XLOC_15456.	transcript	0.0693	0.019851	0.047939	0.073438	0.110004	0.31343	0.292425	0.101718	0.261166
Novel_014598	XLOC_15456.	transcript	0	0	0	0	0.002936	0	0.571549	0	0
Novel_014599	XLOC_15461.	transcript	0.201389	0.287778	0.013677	0.214137	0.1716	0.217675	0	0.254626	0.212526
Novel_014600	XLOC_15461.	transcript	0.245157	0.126065	0.183184	0.104272	0.149175	0.130486	0.363818	0.087826	3.00E-06
Novel_014601	XLOC_15461.	transcript	0	0.23299	0	0.200897	0.15961	0	0	0.137993	0
Novel_014602	XLOC_15461.	transcript	0	0.073828	0.224457	0.065033	0.076024	0.251555	0.269969	0.073363	0
Novel_014603	XLOC_15461.	transcript	0	0.106861	0	0.097961	0.132921	0.144274	0.168437	0.198156	0.007964
Novel_014604	XLOC_15464.	transcript	0.297304	1.188443	0.87762	0.458695	0.579201	0.95163	0.978232	0.529452	3.49316
Novel_014605	XLOC_15465.	transcript	1.331094	0.926003	0.755576	1.010796	0.788602	0.866534	1.774155	0.987599	2.035725
Novel_014606	XLOC_15465.	transcript	0	0.014679	0.09076	0.029184	0.010273	0	0.037061	0.018278	0.071425
Novel_014607	XLOC_15469.	transcript	0	0	0.464673	0.258379	0.165374	0.146312	0.104198	0.043973	0.383245
Novel_014608	XLOC_15479.	transcript	0.930781	2.561443	1.004662	0.689	0.745078	0.656316	1.763862	1.353579	1.447637
Novel_014609	XLOC_15480.	transcript	0.069552	0.525954	0.08707	0.073866	0.159213	0.09838	0.226887	0.156564	0
Novel_014610	XLOC_15480.	transcript	0.093623	0.142479	0.110025	0	0.005002	0	0	0	0
Novel_014611	XLOC_15480.	transcript	3.276216	6.580878	2.133055	1.121424	2.57625	1.727386	5.984714	1.826969	32.08968
Novel_014612	XLOC_15481.	transcript	0.535925	0.28921	0.747842	0.617116	0.778063	0.931212	1.323906	1.134536	1.076026
Novel_014613	XLOC_15481.	transcript	0.137545	0.072972	0.368557	0.162334	0.477002	0.464865	0.645554	0.351726	0.225916
Novel_014614	XLOC_15481.	transcript	0	0.002209	0	0.291837	0	0	0	0	0.231008
Novel_014615	XLOC_15484.	transcript	0.187364	0	0	0	0	0	0.043412	0	0
Novel_014616	XLOC_15486.	transcript	8.927772	4.520635	6.427433	5.291477	6.197977	6.636539	5.913702	5.480383	4.39664
Novel_014617	XLOC_15489.	transcript	0.171954	0.171649	0.125733	0.0082	0.173624	0.18844	0.334462	0.288481	0.178564
Novel_014618	XLOC_15489.	transcript	0	0.11656	0	0	0	0.291487	0.181762	0.161237	0

Novel_014619	XLOC_15489.	transcript	0.199213	0.079898	0.147431	0.021669	0.112157	0	0	0.082449	0.052273
Novel_014620	XLOC_15489.	transcript	0.094193	0.125998	0.145538	0.183383	0.161713	0	0	0.082833	0.087156
Novel_014621	XLOC_15489.	transcript	0.201131	0.15497	0.165715	0.399067	0.113916	0.137757	0.177367	0.086113	0.055263
Novel_014622	XLOC_15489.	transcript	0.520221	0	0	0.636886	0.1645	0.095357	0	0.033003	0.22752
Novel_014623	XLOC_15489.	transcript	0.244287	0.151733	0.310252	0.063177	0.563643	0.055238	0.14369	0.170123	0.053618
Novel_014624	XLOC_15489.	transcript	0.285484	0.344158	0.511836	0.207355	0.096068	0.253424	0.412361	0.493159	0.260347
Novel_014625	XLOC_15491.	transcript	0	0	0.000245	0.884774	0	0.001286	0	0.007384	1.229174
Novel_014626	XLOC_15491.	transcript	0	0	0	0	0	0	0	0	0
Novel_014627	XLOC_15491.	transcript	0.661665	0.058901	0.232436	0.303924	0.552932	0.268402	0.302124	0.550914	0.284416
Novel_014628	XLOC_15491.	transcript	0	0.34086	0.339256	0.002687	0.177276	1.257351	1.110549	0.509138	0.021515
Novel_014629	XLOC_15491.	transcript	0	0.026235	0.457085	0	0	0.044097	0.497442	0.220353	0
Novel_014630	XLOC_15491.	transcript	0	0.01817	0	0	0	0.029431	0	0	0
Novel_014631	XLOC_15491.	transcript	0	0	0.270512	0.212571	0	0	0	0	0
Novel_014632	XLOC_15491.	transcript	0	0	0	0	0	0	0	0.170673	0
Novel_014633	XLOC_15491.	transcript	0	0	0	0	0	0	0	0	0
Novel_014634	XLOC_15491.	transcript	0	0	0	0	0	0	0	0	0
Novel_014635	XLOC_15491.	transcript	0	0	0	0	0	1.00E-06	0	0	0
Novel_014636	XLOC_15493.	transcript	2.141344	0.690743	1.80484	2.688013	1.833314	2.078027	1.5759	2.463701	1.510612
Novel_014637	XLOC_15493.	transcript	1.332688	2.120054	1.168323	1.168625	1.331396	1.494259	0.969601	1.740352	1.076652
Novel_014638	XLOC_15493.	transcript	0.683744	1.475191	2.253508	1.184793	0.898491	0.772538	0.66737	1.183764	0.045232
Novel_014639	XLOC_15493.	transcript	2.198101	2.448166	0.754987	2.085011	1.904345	1.97712	4.269841	1.879858	2.373622
Novel_014640	XLOC_15494.	transcript	0.296017	0.464033	0.264421	0.126214	0.285149	0.314557	0.407744	0.301078	0.26259
Novel_014641	XLOC_15494.	transcript	0	0	0	0	0	0	0	0	0
Novel_014642	XLOC_15497.	transcript	0.633315	0.131341	0.988295	0.511724	0.734667	0.42752	0.711338	0.626223	0.569307
Novel_014643	XLOC_15497.	transcript	0.090617	0.328237	0.338873	0.00416	0.16992	0.300668	0.122279	0.010622	0.38736
Novel_014644	XLOC_15497.	transcript	0.152453	0	0	0	0.11835	0	0.160222	0.114656	0
Novel_014645	XLOC_15497.	transcript	0.085778	0.024863	0.028405	0.346038	0.12758	0.049822	0.287365	0.23829	0.009494
Novel_014646	XLOC_15498.	transcript	0	0.000504	0	0	0	0	0.001352	0	0
Novel_014647	XLOC_15498.	transcript	0.010114	0.005729	0.014319	0.005733	0.016836	0.002956	0.671367	0.026763	0.002539
Novel_014648	XLOC_15498.	transcript	0.866031	1.201786	1.450602	1.438304	1.355599	1.614774	0.84564	1.33683	1.738738
Novel_014649	XLOC_15498.	transcript	0.04506	0.141275	0.058972	0.008075	0.15484	0.167997	0.095727	0.124853	0.136825
Novel_014650	XLOC_15498.	transcript	0.104563	0	0.072535	0.19771	0.003964	0.052412	0.094894	0.065242	0
Novel_014651	XLOC_15500.	transcript	0.122626	0.05081	0.485322	0.388891	0.328014	0.666586	0.122535	0.393249	0.235491
Novel_014652	XLOC_15500.	transcript	0.128334	0.038832	0	0.000124	0.015964	0.00899	0.114415	0.000791	0.131253
Novel_014653	XLOC_15500.	transcript	0.450586	0	0.534331	0.448332	0.616392	0	0.941157	0.487634	0.444277
Novel_014654	XLOC_15500.	transcript	0.016847	0.025057	0.360633	0.127608	0	0.554318	0.009963	0.013251	0.392153
Novel_014655	XLOC_15500.	transcript	0.397961	0.227289	0	0.15671	0.310253	0.193733	0.415579	0.264809	0.350067
Novel_014656	XLOC_15500.	transcript	0	0.403793	0.33162	0.37213	0.458095	0.090783	0.360826	0.203188	0

Novel_014657	XLOC_15500.	transcript	0	0.203203	0	0	0	0	0	0.164426	0
Novel_014658	XLOC_15501.	transcript	0.380932	0.177746	0.416942	0.240655	0.341054	0.314117	0.327531	0.234903	0.127619
Novel_014659	XLOC_15501.	transcript	0.133078	0.048138	0.170194	0.155111	0.104511	0.321819	0.13934	0.162042	0.122213
Novel_014660	XLOC_15501.	transcript	0.286807	0.3076	0.434565	0	0.059065	0.089201	0	0.353881	0.05255
Novel_014661	XLOC_15501.	transcript	0	0	0	0	0	0	0	0	0
Novel_014662	XLOC_15501.	transcript	0	0	0	0.107934	0	0	0.026943	0.002643	0.022402
Novel_014663	XLOC_15502.	transcript	0.036249	0	0.131574	0.138221	0.025313	0.003128	0.028548	0.047504	0.03259
Novel_014664	XLOC_15505.	transcript	0.26438	0.434109	0.319565	0.363944	0.123522	0.128854	0.039472	0.469586	0.109932
Novel_014665	XLOC_15505.	transcript	0.086424	0.367071	0.219026	0.175639	0.380175	0.301147	0.575118	0.019717	0.698971
Novel_014666	XLOC_15505.	transcript	0.543873	0	0.03867	0.037885	0	0.124435	0.110035	0.131029	0.271246
Novel_014667	XLOC_15505.	transcript	0	0.001486	0.112966	0.160595	0	0.00339	0.166343	0.087221	0
Novel_014668	XLOC_15506.	transcript	0	0	0.091464	0	0	0.078461	0	0.099067	0
Novel_014669	XLOC_15508.	transcript	0.040339	0.308962	0	0.000766	0	0.828099	0	0.002137	0
Novel_014670	XLOC_15508.	transcript	0	0	0.794183	0.044037	0.921378	0.0341	0	0.906723	0.918864
Novel_014671	XLOC_15508.	transcript	0.032658	0	0	0	0	0	0.419121	0	0
Novel_014672	XLOC_15508.	transcript	0.614885	0.047747	0.040912	0.916512	0.027557	0	0.689096	0.005209	0.017311
Novel_014673	XLOC_15508.	transcript	0.26031	0.046583	0.020494	0	0.342468	0.024145	0.572829	0.196406	0.396544
Novel_014674	XLOC_15508.	transcript	0	0	0.069848	0.390792	0	0	0	0.085481	0.016559
Novel_014675	XLOC_15508.	transcript	0	0	0	0	0	0	0	0	0
Novel_014676	XLOC_15508.	transcript	0.022872	0.342999	0.448172	0.087697	0.176287	0.486302	0.138529	0.219031	0.1504
Novel_014677	XLOC_15509.	transcript	0.155048	0.236788	0.18642	0.310198	0.305742	0.35464	0.371974	0.140629	0.307416
Novel_014678	XLOC_15509.	transcript	0.070099	0.173304	0.077093	0.004256	0.011468	0.005444	0.044074	0.124073	0
Novel_014679	XLOC_15509.	transcript	0.350502	0.608837	0.519651	0.44773	0.475911	0.454056	0.565845	0.594793	0.36882
Novel_014680	XLOC_15510.	transcript	0.625995	0.847326	0.734261	0.417774	0.430017	0.787664	0.699537	0.375611	0.677585
Novel_014681	XLOC_15510.	transcript	0.020897	1.2657	0.076316	0.01911	0.018345	0.0012	0	0.019772	0.068829
Novel_014682	XLOC_15510.	transcript	0	1.452539	0	0	0	0	0.628453	0	0
Novel_014683	XLOC_15510.	transcript	0	0	0	0.432165	0.384722	0	0.000553	0.418518	0.000302
Novel_014684	XLOC_15510.	transcript	0.072766	0.256039	0.363367	0.063192	0.077256	0.227655	0.441457	0.206501	0.597174
Novel_014685	XLOC_15514.	transcript	0.153292	0.168616	0	0.092759	0	0.410283	0.064531	0.112485	0.117945
Novel_014686	XLOC_15515.	transcript	0.858442	0.322805	1.257297	1.249109	1.507178	1.15623	1.886563	1.593928	0.996392
Novel_014687	XLOC_15515.	transcript	0	0	0	0	0	0.346629	0	0	0
Novel_014688	XLOC_15515.	transcript	0	0	0	0	0	0	0	0	0
Novel_014689	XLOC_15515.	transcript	0.306322	0.13108	0.413871	0.53484	0.272343	0.336086	0.741184	0.258196	0.450552
Novel_014690	XLOC_15515.	transcript	0	0	0.105128	0.108725	0.090729	0.132861	0	0.096028	0
Novel_014691	XLOC_15516.	transcript	0.135391	0.169868	0.084147	0.208429	0.145413	0.183559	0.248559	0.228035	0.171147
Novel_014692	XLOC_15516.	transcript	0	0.02496	0	0	0.052168	0	0.084775	0.084936	0.016889
Novel_014693	XLOC_15516.	transcript	0	0.106997	0	0.00653	0	0	0.055023	0.002003	0.006817
Novel_014694	XLOC_15517.	transcript	0.37076	0.520051	0.250661	0.108744	0.435226	0.190211	0.69046	0.297168	0.130454

Novel_014695	XLOC_15518.	transcript	0	0	0.440163	0.01581	0	0.363753	0.340296	0.505853	0.034493
Novel_014696	XLOC_15518.	transcript	0.390303	0.105116	0	0.249156	0.302382	0	0.247607	0.002758	0.185601
Novel_014697	XLOC_15524.	transcript	0	0	0	0.319453	0.002397	0.468511	0	0.251455	0
Novel_014698	XLOC_15524.	transcript	0	0	0	0.013121	0.099365	0.002774	0	0	0
Novel_014699	XLOC_15524.	transcript	0.034096	0.03577	0.010799	0.112821	0.106828	0.099911	0.134201	0.114314	0.051236
Novel_014700	XLOC_15524.	transcript	0	0.01093	0	0	0	0	0	0	0
Novel_014701	XLOC_15524.	transcript	0	0.018628	0.033156	0	0	0	0	0	0.006691
Novel_014702	XLOC_15525.	transcript	0.363488	0.314031	0.185071	0.338538	0.447525	0.199529	0.215166	0.308448	0.173373
Novel_014703	XLOC_15525.	transcript	0	0	0.190877	0.382427	0.052897	0.193362	0.211695	0.217167	0
Novel_014704	XLOC_15526.	transcript	0.071662	0.025656	0.133424	0.180058	0.256246	0.147198	0.149905	0.237051	0.173036
Novel_014705	XLOC_15526.	transcript	0	0.022658	0	0	0	0	0	0	0
Novel_014706	XLOC_15526.	transcript	0	0	0	0	0	0.411412	0.021518	0	0
Novel_014707	XLOC_15526.	transcript	0.269696	0.366154	0.185491	0.095521	0.536591	1.00E-06	0.406087	0.22564	0.397423
Novel_014708	XLOC_15526.	transcript	0	0.001794	0.296915	0.472889	0	0	0	0	0.644245
Novel_014709	XLOC_15527.	transcript	0.270286	0.370343	0.39989	0.436018	0.511373	0.312233	0.35754	0.353009	0.307335
Novel_014710	XLOC_15527.	transcript	0.207783	0.100343	0.369509	0.401679	0.490702	0.364266	0.710276	0.332508	0.442468
Novel_014711	XLOC_15527.	transcript	0.293299	0.262313	0.392063	0.394694	0.430797	0.525531	0.495843	0.397501	0.384252
Novel_014712	XLOC_15527.	transcript	0.656586	0.093884	0.750569	0.425454	0.603369	0.612932	0.435948	0.425014	0.216388
Novel_014713	XLOC_15527.	transcript	0.085289	0.012349	0.123508	0.121071	0.170128	0.130326	0.107646	0.170143	0.02504
Novel_014714	XLOC_15527.	transcript	0.433653	0.445581	0.107471	0.448831	0.299933	0.589566	0.258338	0.27882	0.430137
Novel_014715	XLOC_15527.	transcript	0	0.633505	0	0	0.197248	0	0.351145	0	0
Novel_014716	XLOC_15527.	transcript	0.055111	0.051395	0.536465	0.091458	0.119769	0.09932	0.364407	0.503048	0.256522
Novel_014717	XLOC_15528.	transcript	0	0	0	0	0	0.9132	0	0.873326	0
Novel_014718	XLOC_15528.	transcript	0.393657	0.583507	0.441507	0.604297	0.572625	0.028645	0.673247	0.076914	0.463497
Novel_014719	XLOC_15529.	transcript	0.148481	0.148057	0.217562	0.163141	0.110196	0.183695	0.249912	0.143002	0.089619
Novel_014720	XLOC_15531.	transcript	0	0.036703	0.009626	0.032201	0.05767	0.012948	0.111229	0.009973	0.057439
Novel_014721	XLOC_15531.	transcript	0.060923	0.011738	0.03067	0	0	0.04077	0.001531	0	0.032842
Novel_014722	XLOC_15531.	transcript	0.033719	0.021971	0.072436	0.06114	0.069842	0.060074	0.041838	0.052929	0.029404
Novel_014723	XLOC_15531.	transcript	0	0.119489	0.534004	0.102632	0.074969	0.249209	0.653717	0.072437	0.163209
Novel_014724	XLOC_15531.	transcript	0.968213	0	0.607959	1.007859	1.212832	0.523472	0.677078	1.149651	0.514648
Novel_014725	XLOC_15532.	transcript	0.318721	0.16024	0.304332	0.220448	0.404425	0.374354	0.432446	0.214718	0.214718
Novel_014726	XLOC_15532.	transcript	0.023835	0.99365	0.066698	0.066578	0.057428	0.024061	0.082963	0.064753	0.027813
Novel_014727	XLOC_15534.	transcript	0.21459	0.240316	0.284637	0.338913	0.3443	0.321399	0.467365	0.323423	0.263074
Novel_014728	XLOC_15534.	transcript	0	0	0	0.066989	0	0	0	0	0
Novel_014729	XLOC_15536.	transcript	0.342517	0.078416	0.300105	0	0.029398	0.01323	0.37974	0.004302	0.122714
Novel_014730	XLOC_15536.	transcript	0	0	0	0	0.021035	0	0	0	0
Novel_014731	XLOC_15536.	transcript	0.020289	0	0	0.143953	0.076221	0.27156	0.009999	0.026312	0.210573
Novel_014732	XLOC_15536.	transcript	0.888721	0.343928	0.679796	1.019368	0.803623	1.006508	1.12657	1.031359	0.856871

Novel_014733	XLOC_15539.	transcript	0	0.010287	0.099131	0.185992	0.111655	0	0	0	0.000102
Novel_014734	XLOC_15540.	transcript	0.255832	0.050121	0.023684	0.114423	0.173728	0.072201	0.387426	0.248077	0.034398
Novel_014735	XLOC_15540.	transcript	0.487758	0	0.338396	0.145798	0.231786	0.099468	0.459483	0.275	0.360682
Novel_014736	XLOC_15540.	transcript	0.15024	0.089983	0	0.074051	0.048789	0.020827	0.060205	0	0.01339
Novel_014737	XLOC_15540.	transcript	1.042582	0.89049	1.254282	1.905234	1.19011	1.553264	1.569451	1.661209	1.807542
Novel_014738	XLOC_15543.	transcript	0.779865	0.267225	1.21421	1.04266	0.673915	0.450008	1.525249	0.574485	0.552719
Novel_014739	XLOC_15543.	transcript	0.05472	0.059029	0.65126	0.047898	0.076885	0.463795	0.467756	0.518211	0
Novel_014740	XLOC_15543.	transcript	0.165819	0.139907	0.19277	0.663287	0.49626	0.172617	0.43163	0.098903	0.388582
Novel_014741	XLOC_15544.	transcript	0.328307	0.297613	0.313977	0.272935	0.328277	0.20017	0.360094	0.301956	0.277069
Novel_014742	XLOC_15544.	transcript	0	0	0	0	0	0	0	0	0
Novel_014743	XLOC_15544.	transcript	0	0	0	0	0	0	0	0	0
Novel_014744	XLOC_15545.	transcript	0	0	0.971204	1.274996	0	0.336702	0	0.504513	0
Novel_014745	XLOC_15545.	transcript	0.011901	0	0.401304	0	1.161511	0.359898	0.043041	0.347574	1.528853
Novel_014746	XLOC_15545.	transcript	0.019317	0.135546	0.386941	0.146828	0.316088	0.428101	0.186485	0.085023	0.311727
Novel_014747	XLOC_15545.	transcript	0.201281	0.356561	0.380825	0.467921	0.553882	0.390766	0.583538	0.458433	0.443786
Novel_014748	XLOC_15545.	transcript	0.033224	0	0.062777	0.057285	0.268654	0.233208	0.154127	0.044247	0
Novel_014749	XLOC_15547.	transcript	0.345156	2.146925	0.446505	0.005976	0.036124	1.061552	0.03717	0.845884	0.516407
Novel_014750	XLOC_15547.	transcript	0.069418	0	0	0.497741	0.207686	0	0	0.19006	0.407707
Novel_014751	XLOC_15548.	transcript	3.783343	3.960799	4.796684	4.491323	3.96371	5.188221	5.321482	4.070814	4.589178
Novel_014752	XLOC_15548.	transcript	0.469097	0.471331	0.12947	0.227953	0.222572	0.330787	0.3126	0.191636	0.278313
Novel_014753	XLOC_15548.	transcript	0.087	0.075483	0.009984	0.084388	0.037709	0.077174	0.012692	0.034886	0.022883
Novel_014754	XLOC_15548.	transcript	0.211187	0.069325	0.069737	0.042579	0	0.174911	0.229958	0.076625	0.027427
Novel_014755	XLOC_15548.	transcript	0.304382	0.409096	0.552957	0.239315	0	0.197171	0.350089	0.198089	0.036455
Novel_014756	XLOC_15548.	transcript	0.461914	0.266225	0.314215	0.500489	0	0.247757	0.278416	0.183837	0.833702
Novel_014757	XLOC_15548.	transcript	0.444362	1.428366	0.176228	0.465445	1.04089	0.34355	0.508083	1.043682	0.817255
Novel_014758	XLOC_15549.	transcript	0.250385	0.071628	0.549177	0	0	0.325253	0	0.067079	0.006582
Novel_014759	XLOC_15549.	transcript	0.08851	0.011618	0.094106	0.350514	0.538318	0.239732	0.409641	0.029356	0.321338
Novel_014760	XLOC_15549.	transcript	0	0.104144	0	0	0	0	0	0	0
Novel_014761	XLOC_15550.	transcript	1.093828	1.897507	1.064782	0.993435	1.266164	1.105179	1.322867	1.207681	1.030976
Novel_014762	XLOC_15550.	transcript	0	0.216385	0	0	0	0	0	0.131561	0
Novel_014763	XLOC_15550.	transcript	0.406689	0.0887	0.138081	0.179095	0.072975	0.1638	0.241223	0.200395	0.147443
Novel_014764	XLOC_15551.	transcript	0.006849	0	0.222304	0.005119	0.049315	0.027066	0.0073	0.183058	0.052302
Novel_014765	XLOC_15551.	transcript	0	0.129039	0	0	0.030254	0	0	0	0
Novel_014766	XLOC_15551.	transcript	0	0	0.007274	0.072741	0	0.003139	0.000951	0	0.086097
Novel_014767	XLOC_15553.	transcript	0	0.276787	0.161353	1.086509	0.236944	0.146457	0.293644	0.205179	0.13091
Novel_014768	XLOC_15553.	transcript	0.437918	0	0.002953	0.095613	0.028121	0	0.059743	0	0
Novel_014769	XLOC_15554.	transcript	0.20942	0	0	0.089259	0.043645	0.144451	0.079704	0.063206	0
Novel_014770	XLOC_15555.	transcript	0.352778	0.178906	0	0	0.043719	0	0.391177	0.12296	0

Novel_014771	XLOC_15555.	transcript	0.003237	0	0.881495	0.551554	0.758268	0.505159	0.331245	0.69484	0.665416
Novel_014772	XLOC_15556.	transcript	0	0	0.187181	0	0.062683	0	0	0	0.052632
Novel_014773	XLOC_15557.	transcript	0	0	0.15382	0.108392	0.029643	0.131849	0.340132	0	0.060526
Novel_014774	XLOC_15557.	transcript	0	0	0	0	0	0	0	0	0
Novel_014775	XLOC_15557.	transcript	0	0	0	0	0	0	0	0.170067	0
Novel_014776	XLOC_15557.	transcript	0	0	0	0	0	0	0	0.012417	0
Novel_014777	XLOC_15557.	transcript	0	0.32477	0	0	0	0.236395	0.048845	0.057386	0.154801
Novel_014778	XLOC_15557.	transcript	1.350868	0.714956	1.883666	2.11407	1.814735	0.837949	2.643363	1.196294	1.639182
Novel_014779	XLOC_15557.	transcript	0	1.218511	0.200135	0.294803	0.115334	1.674043	0.241492	1.400618	0.286717
Novel_014780	XLOC_15557.	transcript	0.210583	0.75764	0.093648	0.106441	0.133356	0.206978	0.440927	0.115867	0.921002
Novel_014781	XLOC_15557.	transcript	0.01828	1.641623	0.012022	0.054389	0.026614	0.149068	0.084925	0.127011	0.122987
Novel_014782	XLOC_15558.	transcript	0	0.16462	0	0.076832	0.101738	0	0.013712	0	0.44263
Novel_014783	XLOC_15559.	transcript	1.090968	0.358103	1.552924	0.313943	1.3657	1.348144	1.886881	1.096388	1.082433
Novel_014784	XLOC_15559.	transcript	0	0	0	0	0.002435	0	0	0	0
Novel_014785	XLOC_15559.	transcript	0.298533	0.2335	0.194952	0.234504	0.253381	0.223678	0.226123	0.179162	0.205626
Novel_014786	XLOC_15560.	transcript	0.105322	0.015124	0.189115	0.074282	0.070211	0	0	0.247233	0
Novel_014787	XLOC_15575.	transcript	0	0	0	0	0.197418	0	0	0	0
Novel_014788	XLOC_15575.	transcript	0.225451	0.042358	0.015715	0.017651	0.028297	0.209034	0.029823	0.064818	0.12226
Novel_014789	XLOC_15575.	transcript	0	0.101926	0	0.181228	0	0	0.24991	0.124561	0
Novel_014790	XLOC_15579.	transcript	0	0	0	0.050605	0	0.073039	0.180476	0.087696	0
Novel_014791	XLOC_15579.	transcript	0.028851	0	0.182906	0.025792	0.165871	0.06129	0	0.131965	0
Novel_014792	XLOC_15580.	transcript	0.529047	0.353047	0.487201	0.363629	0.311405	0.351514	0.767666	0.367953	0.194485
Novel_014793	XLOC_15580.	transcript	0	0	0	0	0	0	0	0	0
Novel_014794	XLOC_15581.	transcript	0	0	0	0	0	0	0	0.018949	0.041728
Novel_014795	XLOC_15581.	transcript	0	0.661354	0	0.010587	0	0.074204	0.009479	0	0.124747
Novel_014796	XLOC_15582.	transcript	0.103824	0.086704	0.109579	0	0.095689	0	0.403356	0.161636	0.03123
Novel_014797	XLOC_15583.	transcript	0	0.429173	0	0	0	0	0	0	0
Novel_014798	XLOC_15587.	transcript	0.061141	0.032421	0.512808	0.0096	0.375996	0.182479	0.126471	0.221243	0.013652
Novel_014799	XLOC_15587.	transcript	0.428732	0	0.005957	0	0.251644	0.242837	0	0.29146	0.012798
Novel_014800	XLOC_15587.	transcript	0	0	0	0	0	0	0	0	0
Novel_014801	XLOC_15587.	transcript	0	1.429435	0.447942	3.586972	0.139581	0.381721	2.549816	0.038849	2.643271
Novel_014802	XLOC_15587.	transcript	0.020001	0.023949	0.061053	0.114587	0.202183	0.003426	0.10849	0.016884	0.002645
Novel_014803	XLOC_15587.	transcript	0	0	0.573934	0	0.253602	0.299769	0	0.369328	0
Novel_014804	XLOC_15587.	transcript	0.161601	0.149803	0.030301	0.214093	0.028943	0.065717	0.069704	0.022507	0.072876
Novel_014805	XLOC_15587.	transcript	0.005195	0	0.074171	0	0	0	0	0.014857	0
Novel_014806	XLOC_15587.	transcript	0.087674	0.292019	0.107607	0.233572	0.009203	0.475119	0.192357	0.025882	0.342223
Novel_014807	XLOC_15587.	transcript	0.302037	0.142276	1.08915	0.805495	0.51104	0.016883	0.360974	0.297041	0.63498
Novel_014808	XLOC_15587.	transcript	0	0	0	0	0.111955	0.353118	0	0.204791	0

Novel_014809	XLOC_15587.	transcript	0	0	0	0.132372	0.21546	0.062687	0.438305	0.242334	0
Novel_014810	XLOC_15587.	transcript	0	0	0	0	0	0	0	0	0
Novel_014811	XLOC_15587.	transcript	0	0.054398	0	0.214821	0.128198	0.09954	0	0.125766	0.603779
Novel_014812	XLOC_15589.	transcript	0	0	0	0	0.148685	0	0	0	0
Novel_014813	XLOC_15589.	transcript	0.06571	0.049537	0.161809	0.050004	0.122453	0	0.133637	0.076715	0.03533
Novel_014814	XLOC_15589.	transcript	0.023534	0	0	0.040236	0	0	0	0.009115	0
Novel_014815	XLOC_15589.	transcript	0	0	0	0	0	0	0	0.00423	0
Novel_014816	XLOC_15589.	transcript	0	0	0	0.009561	0	0.007725	0	0	0
Novel_014817	XLOC_15589.	transcript	0	0.031789	0.088789	0.01405	0.048515	0.155653	0.051219	0.000657	0
Novel_014818	XLOC_15589.	transcript	0	0	0.457673	0.393376	0.024803	0.242616	0.063803	0.305094	0.142093
Novel_014819	XLOC_15589.	transcript	0.07907	0.096646	0	0.097543	0.01386	0	0.089246	0	0.048019
Novel_014820	XLOC_15589.	transcript	0	0	0	0	0.424803	0	0.243373	0	0.225818
Novel_014821	XLOC_15590.	transcript	0.394439	0.20847	0.766492	0.804018	0.561426	0.655682	0.863712	0.62076	0.591201
Novel_014822	XLOC_15595.	transcript	0.528794	0.418077	0.493359	0.486004	0.573954	0.632877	0.622202	0.627777	0.331308
Novel_014823	XLOC_15595.	transcript	0.152397	0.135427	0	0.159046	0.166082	0	0	0	0.17311
Novel_014824	XLOC_15595.	transcript	0.129317	0.024821	0.30367	0.042569	0.120656	0.311958	0.172264	0.289073	0.056104
Novel_014825	XLOC_15598.	transcript	0.617601	0.626033	1.535794	0.200891	1.199487	1.47293	1.321964	1.082238	0.496561
Novel_014826	XLOC_15598.	transcript	0	0	0	1.177091	0	0	0	0	0
Novel_014827	XLOC_15598.	transcript	0.111168	0	0	0.020306	0	0	0	0	1.483924
Novel_014828	XLOC_15598.	transcript	0	0	0	0	0.286641	0.048252	0	0.021518	0
Novel_014829	XLOC_15598.	transcript	0.534421	0.350903	0.240713	0	0.03157	0	0.04341	0	0.249019
Novel_014830	XLOC_15598.	transcript	0.023611	0	0.011493	0.278934	0	0.118614	0.059445	0.443862	0
Novel_014831	XLOC_15600.	transcript	0.113086	0	0.053647	0.011481	0.06409	0.000118	0.070047	0.205456	0.113877
Novel_014832	XLOC_15600.	transcript	0	0	0	0	0.043398	0	0	0	0
Novel_014833	XLOC_15600.	transcript	0	0.003701	0	0	0.036793	0	0.31874	0.109217	0.034445
Novel_014834	XLOC_15600.	transcript	0	0.101882	0.121091	0.242553	0.113669	0.215841	0	0	0.028085
Novel_014835	XLOC_15600.	transcript	0	0	0	0	0	0	0	0	0
Novel_014836	XLOC_15600.	transcript	0.448161	0.243466	0.618388	0.610895	0.361649	0.508102	0.495578	0.391053	0.354941
Novel_014837	XLOC_15600.	transcript	0.023409	0.14856	0.355245	0	0.009428	0.127885	0.324681	0.09943	0.023204
Novel_014838	XLOC_15600.	transcript	0.029243	0	0	0.01106	0.008528	0	0.026946	0.035435	0
Novel_014839	XLOC_15600.	transcript	0.022195	0	0	0.431174	0	0.008332	0	0.087218	0.016654
Novel_014840	XLOC_15600.	transcript	0.010299	0	0.085522	0	0.248824	0	0.271932	0.458709	0
Novel_014841	XLOC_15600.	transcript	0.763727	0	0.03888	0.0402	0.230833	0.048913	0.213377	0.010145	0.318971
Novel_014842	XLOC_15600.	transcript	0	0	0	0.276953	0.563669	0.561359	0.012246	0	0.019932
Novel_014843	XLOC_15600.	transcript	0.035641	0.023732	0.030853	0	0.048011	0.051778	0.002077	0.123696	0.073945
Novel_014844	XLOC_15600.	transcript	0.020216	0.459752	0.225959	0.013247	0.278267	0.244898	0.246403	0.039152	0.409676
Novel_014845	XLOC_15600.	transcript	0.474355	0	0.467671	0.509442	0	0.352901	0.236559	0.188559	0
Novel_014846	XLOC_15600.	transcript	0	0	0	0	0	0	0.255814	0.243434	0

Novel_014847	XLOC_15600.	transcript	0.235928	0.203575	0.112587	0.023974	0.463113	0.260412	0.556143	0.403358	0.21849
Novel_014848	XLOC_15600.	transcript	0.099715	0.003075	0.527927	0.424215	0.081885	0.201553	0.109753	0.0435	0.119001
Novel_014849	XLOC_15600.	transcript	0.459686	0	0.466962	0.44149	0.38216	0.745511	0.992695	0.129267	0.538168
Novel_014850	XLOC_15601.	transcript	0.064991	0	0	0	0.182798	0.042685	0.145819	0.018605	1.074147
Novel_014851	XLOC_15602.	transcript	0	0	0	0.365126	0	0	0	0.195744	0.979836
Novel_014852	XLOC_15602.	transcript	0.965204	0.018365	0.889062	0.367903	1.215893	0.850672	1.338591	0.653219	0
Novel_014853	XLOC_15602.	transcript	0	0	0	0	0	0	0	0	0
Novel_014854	XLOC_15608.	transcript	0.1208	0.006327	0.012085	0.00761	0	0.000786	0.049613	0.136765	0.122933
Novel_014855	XLOC_15608.	transcript	0.00672	0.104916	0.233225	0.208306	0.21346	0.226718	0.200191	0	0.005215
Novel_014856	XLOC_15608.	transcript	0.259751	0.113269	0.240583	0.229669	0.243826	0.279374	0.272365	0.307394	0.081223
Novel_014857	XLOC_15609.	transcript	0.06652	0.0589	0.222869	0.37582	0	0	0	0	0.082238
Novel_014858	XLOC_15609.	transcript	0	0	0	0	0.128669	0.097047	0	0	0.034288
Novel_014859	XLOC_15609.	transcript	0.093468	0.081103	0.103926	0.155141	0	0.011184	0.149881	0.11762	0.056678
Novel_014860	XLOC_15609.	transcript	0.288044	0.30869	0.113185	0.278633	0.545428	0.216006	0.515867	0.229249	0.154312
Novel_014861	XLOC_15609.	transcript	0.23984	0	0.245148	0	0.002222	0.117748	0	0.000703	0.223888
Novel_014862	XLOC_15609.	transcript	0	0	0.39048	0.380108	0	0.300883	0	0.08392	0.011097
Novel_014863	XLOC_15609.	transcript	0.249482	0.272748	0.032427	0.017814	0.213795	0.076208	0.291182	0.284523	0.256198
Novel_014864	XLOC_15610.	transcript	0.270665	0.128044	0.204452	0.126164	0.334342	0.226737	0.376468	0.182542	0.148876
Novel_014865	XLOC_15611.	transcript	0.173127	0.09317	0	0	0.084267	0.254078	0	0.203484	0.14708
Novel_014866	XLOC_15611.	transcript	0.192516	0.116328	0.392005	0.214289	0.277771	0.236903	0.352879	0.15114	0.096905
Novel_014867	XLOC_15611.	transcript	0	0	0	0	0	0	0.002157	0.17121	0
Novel_014868	XLOC_15612.	transcript	0.463214	0.173247	0.255501	0.181085	0.261664	0.297188	0.505364	0.332078	0.129809
Novel_014869	XLOC_15613.	transcript	0.062345	0	0	0	0	0	0	0	0
Novel_014870	XLOC_15613.	transcript	0.271533	0	0.133199	0	0	0.148205	0.246558	0.472885	0.114847
Novel_014871	XLOC_15613.	transcript	0.000159	0.206122	0.003989	0.01126	0.511037	0.001539	0	0.008832	0.019369
Novel_014872	XLOC_15613.	transcript	0	0	0	0	0	0	0	0	0
Novel_014873	XLOC_15613.	transcript	0.222239	0.135537	0.086615	0.276356	0	0	0.138912	0.099381	0
Novel_014874	XLOC_15613.	transcript	0.631675	0.621021	0.257315	0.354312	0.457773	0.383264	0.692802	0.389559	0.367032
Novel_014875	XLOC_15616.	transcript	0.429236	0.256857	0.44722	0.338831	0.443935	0.457083	0.413415	0.427176	0.282599
Novel_014876	XLOC_15616.	transcript	0.270288	0.440567	0.414192	0.571314	0.383968	0.4434	0.49331	0.560762	0.501671
Novel_014877	XLOC_15617.	transcript	0.032074	0.081626	0.063895	0.128455	0.130427	0.067835	0.152253	0.082347	0.107177
Novel_014878	XLOC_15617.	transcript	0.003075	0.391546	0.037788	0.155662	0.133538	0.165337	0.242045	0.185226	0.025396
Novel_014879	XLOC_15617.	transcript	0.013034	0	0	0	0	0	0	0	0
Novel_014880	XLOC_15618.	transcript	6.351174	4.141119	6.05472	7.667241	6.681493	6.776991	7.602201	9.173896	6.041375
Novel_014881	XLOC_15618.	transcript	0.917825	21.66026	0.890994	0.516247	1.039216	1.181708	3.023481	0.726019	0.778954
Novel_014882	XLOC_15618.	transcript	0.172245	0.527494	0.400751	0.338978	0.066641	0.21856	0.180453	0.27125	0.142357
Novel_014883	XLOC_15628.	transcript	0	0.9614	0	0.00829	0	0.014378	0.026025	0.006555	0
Novel_014884	XLOC_15632.	transcript	0.122651	0.15226	0	0.110821	0.320392	0.619157	0.084653	0.117144	0.081831

Novel_014885	XLOC_15632.	transcript	0.718081	0.198643	0.960702	0.832144	0.855013	0.762523	1.201984	0.746282	0.505471
Novel_014886	XLOC_15633.	transcript	0.003992	0.002448	0.051691	0.497547	0.002143	0.256676	0.010473	0	0.001346
Novel_014887	XLOC_15633.	transcript	0.943618	1.563401	1.097572	0.539975	1.21244	0.825685	1.242111	1.344629	1.339383
Novel_014888	XLOC_15633.	transcript	0.843233	1.191614	0.959667	1.026332	0.781975	0.943978	1.363216	1.008348	1.141447
Novel_014889	XLOC_15639.	transcript	0.100365	0.37219	0.193124	0.461726	0.210597	0.524905	0.568505	0.153882	0.154331
Novel_014890	XLOC_15641.	transcript	0.02575	0	0.051861	0.095858	0.177209	0	0.240699	0.210614	0.052134
Novel_014891	XLOC_15658.	transcript	0.141928	0.003481	0.029357	0.046344	0.07632	0.116853	0.090376	0.065029	0
Novel_014892	XLOC_15658.	transcript	0.022127	0.003433	0.034938	0	0.040091	0.007333	0.005393	0.043787	0.270103
Novel_014893	XLOC_15658.	transcript	0.058714	0.145813	0.087954	0.033115	0.072226	0.049956	0.069538	0.091117	0.071704
Novel_014894	XLOC_15658.	transcript	0.119028	0.017657	0	0.026845	0.082301	0.096143	0.266572	0.039669	0.02255
Novel_014895	XLOC_15658.	transcript	0	0.076053	0	0	0	0	0	0	0
Novel_014896	XLOC_15658.	transcript	0	0.606385	0.203782	0.149373	0.232681	0.079874	0.336532	0	0.237753
Novel_014897	XLOC_15666.	transcript	0.061478	0	0	0.001483	0	0.030849	0	0.17939	0
Novel_014898	XLOC_15666.	transcript	0	0	0	0	0.319653	0	0	0	0.360566
Novel_014899	XLOC_15666.	transcript	0.309595	0	0.36982	0.369153	0.010945	0.214894	0.306304	0.298115	2.140011
Novel_014900	XLOC_15666.	transcript	0	0	0.130802	0	0.011318	0	0	0	0.388504
Novel_014901	XLOC_15666.	transcript	0.605787	0	0.284533	0.26796	0.271307	0.23181	0.394763	0.981457	2.689547
Novel_014902	XLOC_15666.	transcript	0	0	0	0	0	0	0	0	0.40454
Novel_014903	XLOC_15666.	transcript	0.191524	0	0.046121	0.054949	0	0.026318	0.032627	0.182723	0.360938
Novel_014904	XLOC_15667.	transcript	0	0	0	0	0	0	0	0	0
Novel_014905	XLOC_15675.	transcript	0	0.280965	0	0	0	0.16844	0	0	5.10E-05
Novel_014906	XLOC_15675.	transcript	0.286833	0.140207	0.147959	0.077073	0.141508	0.134682	0.258266	0.162149	0.112271
Novel_014907	XLOC_15693.	transcript	0.247526	0.492293	0.260863	0.345861	0.311981	0.268034	0.415339	0.181379	0.273712
Novel_014908	XLOC_15695.	transcript	0.102077	2.053441	0.106153	0.037445	0.07075	0.144463	0.168861	0.086576	0.138976
Novel_014909	XLOC_15695.	transcript	0.026778	2.650091	0.031329	0.008734	0.005905	0.023191	0.057575	0.041221	0.008012
Novel_014910	XLOC_15696.	transcript	0	0.784181	0	0.01876	0.045332	0.165458	0	0.008016	0.1126
Novel_014911	XLOC_15698.	transcript	0.028187	0.00867	0.278026	0.011804	0.005621	0	0.007011	0	0
Novel_014912	XLOC_15698.	transcript	0.176443	0.09682	0.051512	0.083711	0.245695	0.085596	0.293395	0.197436	0.13631
Novel_014913	XLOC_15698.	transcript	1.688165	1.337886	3.881823	4.023207	3.78163	3.834779	3.059465	2.741621	4.558922
Novel_014914	XLOC_15699.	transcript	0.388688	0.644421	0.39443	0.642688	0.706269	0.490044	0.449186	0.635486	0.678192
Novel_014915	XLOC_15699.	transcript	0	0	0	0	0	0	0	0	0
Novel_014916	XLOC_15700.	transcript	0.193652	0	0.101683	0.040575	0.290494	0	0.325868	0.052481	0.222616
Novel_014917	XLOC_15700.	transcript	0.491003	0.010724	1.128806	1.155446	1.082102	0.980857	0.935581	1.110791	1.360803
Novel_014918	XLOC_15700.	transcript	0.367592	0	0	0.311578	0	0.481153	0.218042	0	0.353309
Novel_014919	XLOC_15700.	transcript	0.251519	0	0.599594	0.788647	0.911646	0.451073	0.260491	0.761392	0.618632
Novel_014920	XLOC_15700.	transcript	0	0.288265	0	0.304661	0.424086	0.327133	0	0.539548	0.243625
Novel_014921	XLOC_15702.	transcript	1.755964	0.076662	1.839788	1.880803	1.904042	0.956034	3.165426	2.38301	1.655267
Novel_014922	XLOC_15704.	transcript	0.159397	0.014004	0.252674	0.123555	0.105827	0.072935	0.075809	0.202701	0.062039

Novel_014923	XLOC_15705.	transcript	0.10023	0.220167	0.201741	0.253304	0.397856	0.216695	0.219078	0.065612	0
Novel_014924	XLOC_15705.	transcript	0.091238	0.280817	0.130609	0.260327	0.188083	0.189368	0.485601	0.409828	0.203577
Novel_014925	XLOC_15713.	transcript	0.027967	0.155747	0.063118	0.035589	0.057272	0.088904	0.146553	0.082879	0.059154
Novel_014926	XLOC_15734.	transcript	0.334429	0.228674	0.184093	0.320914	0.215195	0.311321	0.370904	0.210755	0.189652
Novel_014927	XLOC_15737.	transcript	0.105092	0	0.374003	0	0	0.211828	0	0	0.061806
Novel_014928	XLOC_15737.	transcript	0.085318	0.009229	0.089032	0.002552	0.007412	0.179533	0.00811	0	0.067524
Novel_014929	XLOC_15737.	transcript	0.175805	0.136477	0.101816	0.012606	0.12471	0.072599	0.547427	0.087365	0.067707
Novel_014930	XLOC_15737.	transcript	0.089687	0.290292	0.09969	0.245914	0	0.160554	0.114342	0.111177	0.19921
Novel_014931	XLOC_15737.	transcript	0.098188	0.129503	0.120399	0.000381	0.000548	0.067318	0.106217	0.09116	0.070195
Novel_014932	XLOC_15737.	transcript	0.159794	0.232585	0.082159	0.172138	0.3736	0.17546	0.100999	0.237143	0.062242
Novel_014933	XLOC_15737.	transcript	0	0	0	0	0	0	0.002914	0.089958	0
Novel_014934	XLOC_15737.	transcript	0.178178	0.035561	0.331747	0.345474	0.089335	0.334506	0.405932	0.068145	0.53234
Novel_014935	XLOC_15737.	transcript	0.135063	0	0.496101	0.143337	0.354427	0	0.311474	0.309319	0
Novel_014936	XLOC_15738.	transcript	0.16356	0	0.036384	0.014562	0.026773	0.066664	0.434031	0.033202	0.458778
Novel_014937	XLOC_15739.	transcript	0.318181	0.570868	0.429555	0.388013	0.686508	0.714438	0.818739	0.42647	0.582699
Novel_014938	XLOC_15742.	transcript	0.014642	0.011949	0.082737	0	0.046277	0.041395	0.002956	0.028625	0.037262
Novel_014939	XLOC_15742.	transcript	0.403735	0	0	0	0	0	0.264343	0	0.022177
Novel_014940	XLOC_15742.	transcript	0	0.26641	0	0.222125	0	0	0.39977	0	0
Novel_014941	XLOC_15742.	transcript	0.160284	0.302959	0.395486	0.31148	0.30766	0.372291	0.145558	0.547608	0.257886
Novel_014942	XLOC_15743.	transcript	0.230303	0.177141	0.166525	0.09849	0.180581	0.261082	0.419029	0.210718	0.242797
Novel_014943	XLOC_15743.	transcript	0	0	0	0	0	0.291115	0	0.702315	0.862396
Novel_014944	XLOC_15743.	transcript	0.069592	0	0.050771	1.56743	0.518482	0.311693	0.008634	0.346723	0.605642
Novel_014945	XLOC_15743.	transcript	0.061466	0.527296	0.468892	0.266393	0.284502	0.260646	0.395502	0.274343	0
Novel_014946	XLOC_15743.	transcript	0	0.129903	0	0.136267	0.098321	0	0.306363	0.208361	0
Novel_014947	XLOC_15743.	transcript	0	0	0	0	0	0.28788	0.349198	0.126845	0
Novel_014948	XLOC_15743.	transcript	0.579207	0.03151	0.094762	0.006172	0.032182	0	0.005998	0	0
Novel_014949	XLOC_15744.	transcript	0.126975	0.283287	0.125804	0.107901	0.225638	0.09022	0.173542	0.272317	0.115128
Novel_014950	XLOC_15745.	transcript	0	0	0	0	0	0	0	0	0
Novel_014951	XLOC_15745.	transcript	1.19609	0.807217	1.249492	0.634038	1.320548	1.531172	1.691066	2.067392	1.462854
Novel_014952	XLOC_15745.	transcript	0.248057	0.533863	0.393347	0.406791	0.767694	0.407819	0.346806	0.258603	0.654986
Novel_014953	XLOC_15745.	transcript	0.014466	0.045797	0.026904	0.932653	0.050946	0	0.066519	0.055893	0.055575
Novel_014954	XLOC_15745.	transcript	0.008826	0	0.023559	0.112888	0.003981	0.005761	0.252166	0	0.002801
Novel_014955	XLOC_15745.	transcript	0.285277	0.316935	0.194237	0.31342	0.432508	0.301195	0.391256	0.302619	0.403941
Novel_014956	XLOC_15745.	transcript	0.103685	0.17953	0.209384	0.091271	0.116815	0.336102	0.185241	0.248858	0.191229
Novel_014957	XLOC_15748.	transcript	0	0.004764	0	0.152528	0.013456	0.12874	0	0	0.045019
Novel_014958	XLOC_15756.	transcript	0.229962	0.317734	0.219544	0.113217	0.259364	0.071326	0.269895	0.217223	0.056427
Novel_014959	XLOC_15757.	transcript	0.047446	0	0.250053	0.062285	0.260539	0	0.280798	0.008112	0
Novel_014960	XLOC_15757.	transcript	0.063718	0.021797	0	0.016296	0	0.084351	0.04419	0.100733	0.233552

Novel_014961	XLOC_15757.	transcript	0.048011	0.300378	0.077543	0.039652	0.121486	0.108841	0.235856	0.126998	0.06739
Novel_014962	XLOC_15760.	transcript	1.804127	0.79949	3.450691	2.089005	2.452127	3.403399	2.904894	2.652593	4.707745
Novel_014963	XLOC_15760.	transcript	0.519085	0.635756	0.505086	0.614695	0.583139	0.482053	0.778249	0.73494	0.552091
Novel_014964	XLOC_15761.	transcript	0.873753	0.449023	0.88805	0.706776	0.895112	0.918585	0.838974	0.766606	0.838291
Novel_014965	XLOC_15762.	transcript	0.249679	0.184858	0.140806	0.019872	0.140948	0.053105	0.23973	0.159494	0.07049
Novel_014966	XLOC_15763.	transcript	0.045942	0.034583	0.34933	0.071658	0.085626	0.195652	0.169643	0.098139	0.194827
Novel_014967	XLOC_15763.	transcript	0	0.186106	0.021981	0.060059	0	0	0.093247	0	0
Novel_014968	XLOC_15769.	transcript	0	0.401432	0.240369	0	0.055187	0	0.253352	0.381475	0
Novel_014969	XLOC_15769.	transcript	0	0.324524	0	0.013455	0	0.003209	0	0.149775	0
Novel_014970	XLOC_15769.	transcript	0.050524	0.037229	0	0.086868	0.208002	0.186326	0.087448	0.093897	0.089796
Novel_014971	XLOC_15769.	transcript	0.438433	0.028065	0.350837	0.201019	0.589896	0.742413	0	0.000588	0.191383
Novel_014972	XLOC_15769.	transcript	0.064545	0.005622	0	0.663533	0.123971	0.077958	0.037452	0.287052	0
Novel_014973	XLOC_15769.	transcript	0.604421	0	0.133928	0	0	0	0.537049	0.136835	0.364946
Novel_014974	XLOC_15769.	transcript	0.623629	0.61212	0	0.164066	0.355726	0.165316	0.461003	0.403564	0.047565
Novel_014975	XLOC_15769.	transcript	0.739424	0.572404	0.72909	0.761567	0.458028	0.623619	0.859375	0.802121	0.807158
Novel_014976	XLOC_15776.	transcript	6.818226	1.38387	6.999576	6.995015	6.521031	5.972638	11.04962	8.642382	25.90699
Novel_014977	XLOC_15776.	transcript	0.250396	0.613436	0	0	0	0	0	0.061018	0
Novel_014978	XLOC_15776.	transcript	0.058271	0	0	0	0	0	0	0.032352	0
Novel_014979	XLOC_15776.	transcript	0.224025	0.624048	0.245351	0.058296	0.253816	0.195848	0.166757	0.110489	0.38438
Novel_014980	XLOC_15777.	transcript	2.508778	3.137843	2.353816	4.327485	1.712697	3.68843	3.813002	3.838873	3.225376
Novel_014981	XLOC_15779.	transcript	0.454581	1.19294	0.631413	0.615566	0.642561	0.671472	0.590027	0.808758	0.509805
Novel_014982	XLOC_15780.	transcript	0.117996	1.693119	0.096983	0.081808	0.061171	0.214081	0.287584	0.053106	0.185599
Novel_014983	XLOC_15781.	transcript	0.109491	0.917646	0.025347	0.492997	0.261634	0.16146	1.283616	0.901429	0.189194
Novel_014984	XLOC_15781.	transcript	0.305715	0.350702	0.098463	0.409721	0.404941	0.245452	0.50717	0.44338	0.45722
Novel_014985	XLOC_15782.	transcript	0	0	0.063866	0	0	0	0	0	0
Novel_014986	XLOC_15782.	transcript	0.078563	0.43519	0.160828	0.142503	0.137927	0.103133	0.201969	0.173293	0.181537
Novel_014987	XLOC_15783.	transcript	0	0.421016	0	0	0	0	0	0	0
Novel_014988	XLOC_15783.	transcript	0	0.401274	0	0	0	0.012885	0.254149	0	0.013831
Novel_014989	XLOC_15783.	transcript	0.120003	0.426087	0.070597	0.098407	0.044847	0.170815	0.247012	0.049895	0.021918
Novel_014990	XLOC_15783.	transcript	0	1.623272	0	0	0	0	0	0	0.168329
Novel_014991	XLOC_15783.	transcript	0.116245	0.108604	0.258706	0.251679	0.489541	0.089208	0.183821	0.278809	0.261522
Novel_014992	XLOC_15783.	transcript	0.042294	0	0.051053	0.209379	0	0.367045	0.374939	0.269099	0.022171
Novel_014993	XLOC_15784.	transcript	0.320416	0.449161	0.396361	0.084831	0.243093	0.199836	0.193704	0.283829	0.215206
Novel_014994	XLOC_15785.	transcript	0.156988	0.10037	0.291418	0.859088	0.457279	0.320599	0.419981	0.457972	0.162244
Novel_014995	XLOC_15786.	transcript	0	0.136165	0.235813	0.213378	0.101488	0.179989	0.034828	0.123425	0.105349
Novel_014996	XLOC_15787.	transcript	0	0	0	0.028745	0	0.064336	0	0	0
Novel_014997	XLOC_15787.	transcript	0	0.08799	0.233722	0	0	0	0	0.042755	0
Novel_014998	XLOC_15787.	transcript	0.035153	0.067187	0.026279	0.3004	0.240854	0.286452	0.13574	0.057475	0.219499

Novel_014999	XLOC_15787.	transcript	0.399541	0	0.099996	0.49747	0	0.026348	0.499816	0.301477	0.206951
Novel_015000	XLOC_15812.	transcript	0	1.334168	0.763556	0.961149	1.057988	0.58593	1.548077	1.28683	0.513933
Novel_015001	XLOC_15812.	transcript	0	0	0	0	6.70E-05	0	0	0	0
Novel_015002	XLOC_15812.	transcript	0.652007	0.09143	0.06365	0.126584	0.076938	0.120871	0.175929	0.161811	0.107791
Novel_015003	XLOC_15814.	transcript	0	0.279512	0.020355	0.29186	0.364472	0.150644	0.190359	0	0.102184
Novel_015004	XLOC_15814.	transcript	0	0	0	0.073057	0.125405	0	0	0	0
Novel_015005	XLOC_15814.	transcript	0.528215	0	0.254261	0	0	0	0	0.284081	0
Novel_015006	XLOC_15815.	transcript	0	0	0	0	0	0	0	0	0
Novel_015007	XLOC_15815.	transcript	0.096307	0.025513	0.046525	0	0.107332	0	0.099372	0	0.414998
Novel_015008	XLOC_15815.	transcript	0.228309	0	0	0.549609	0.358453	1.068854	0.322634	0.652889	0.411858
Novel_015009	XLOC_15815.	transcript	0.317553	2.187077	0.99965	0.584438	0.292358	0	0.704908	0.806228	1.285054
Novel_015010	XLOC_15815.	transcript	0.726903	0.027873	0.373454	0.331231	0.380869	0.186382	0.720579	0.528596	0.29983
Novel_015011	XLOC_15816.	transcript	0.041789	0	0	0.063205	0	0	0.086063	0.115916	0
Novel_015012	XLOC_15816.	transcript	0.011301	0.20142	0.142251	0	0.059007	0.023203	0.131742	0	0.032552
Novel_015013	XLOC_15817.	transcript	0.412397	0.569907	0.553714	0.553618	0.598326	0	0.505764	0.130226	0.542847
Novel_015014	XLOC_15818.	transcript	0.208465	0.447914	0.278089	0.115514	0.291543	0.160419	0.421445	0.181655	0.291059
Novel_015015	XLOC_15818.	transcript	0.122085	0.055729	0.186961	0.070313	0.130328	0.19161	0.249321	0.143868	0.079352
Novel_015016	XLOC_15818.	transcript	2.471478	1.926884	2.697016	2.332332	2.549359	2.389924	3.006258	2.091694	2.136084
Novel_015017	XLOC_15819.	transcript	0	1.268199	0	0.006264	0	0.888244	0.039797	1.286143	0.01362
Novel_015018	XLOC_15819.	transcript	0	0	0	0	0	0	0	0	0
Novel_015019	XLOC_15819.	transcript	1.404736	1.052095	0.806224	1.949489	2.161413	1.105781	1.75789	0.675018	1.561766
Novel_015020	XLOC_15819.	transcript	1.515317	0.007088	0.03284	0.311654	0	0.009187	0.976264	1.438393	0.209765
Novel_015021	XLOC_15819.	transcript	0	0	1.62753	0	0	0	0	0	0.00362
Novel_015022	XLOC_15819.	transcript	0	0.092465	0.159156	0.025947	0	0	0	0	0.01814
Novel_015023	XLOC_15819.	transcript	0.662596	0	0.653606	0.580522	1.024268	0.586371	0.857904	1.023934	0
Novel_015024	XLOC_15819.	transcript	0.17494	1.053098	0	0.20391	0.019276	0.137716	0.620085	0.048783	0.751198
Novel_015025	XLOC_15819.	transcript	0.194919	0.337104	0.171701	0.1881	0.182849	0.144216	0.232668	0.218159	0.127189
Novel_015026	XLOC_15820.	transcript	0.166802	0.011556	0.095451	0.110874	8.40E-05	0.164169	0.116229	0.001835	0.004347
Novel_015027	XLOC_15820.	transcript	0.13757	0.117932	0.117231	0.198188	0.296699	0.304768	0.306567	0.165771	0.240138
Novel_015028	XLOC_15820.	transcript	0.037985	0.444718	0.084915	0.075245	0.068425	0.149526	0.20762	0.114179	0
Novel_015029	XLOC_15821.	transcript	0.083551	0.015135	0.059157	0.154454	0.037354	0	0.339094	0.119713	0.104455
Novel_015030	XLOC_15821.	transcript	0.063191	0.246185	0.126597	0.11182	0.10634	0.112053	0.169282	0.101839	0.257994
Novel_015031	XLOC_15821.	transcript	0.071799	0	0.066691	0.113649	0.150526	0	0	0.101903	0
Novel_015032	XLOC_15821.	transcript	0	0	0	0	0	0.355625	0.000837	0	0.076656
Novel_015033	XLOC_15821.	transcript	0	0	0	0	0	0.047647	0	0	0
Novel_015034	XLOC_15822.	transcript	0.326472	0	0.372407	0.607422	0.354602	0.22467	0.652172	0.284868	0
Novel_015035	XLOC_15822.	transcript	0.527592	0.005369	0.631545	0.433587	0.730936	0.79683	0.610157	0.792456	0.754018
Novel_015036	XLOC_15822.	transcript	0	0.320483	0	0	0.191534	0.193353	0.35818	0.137952	0.238701

Novel_015037	XLOC_15822.	transcript	0.197734	0.04014	0.295291	0.254754	0.009385	0.072272	0.04818	0.082105	0.01526
Novel_015038	XLOC_15823.	transcript	0.76784	0.370661	0.435866	0.723106	0.396104	0.592289	0.791865	0.912082	0.577972
Novel_015039	XLOC_15823.	transcript	0.000985	0.002566	0.108064	0.019283	0.007034	0.077264	0.020967	0.005555	0
Novel_015040	XLOC_15823.	transcript	1.375326	1.342309	1.519059	2.224773	1.863455	1.364836	2.194169	1.836821	2.029439
Novel_015041	XLOC_15823.	transcript	0.261762	0.206519	0.408519	0.301015	0.230022	0.208041	0.322747	0.392334	0.462178
Novel_015042	XLOC_15824.	transcript	0	0.02188	0	0.003262	0.003244	0	0.021537	0.011333	0.130224
Novel_015043	XLOC_15824.	transcript	0.438136	0.303806	0.266742	0.264342	0.275452	0.262031	0.360696	0.380531	0.068691
Novel_015044	XLOC_15824.	transcript	0	0	0.003678	0	0.003728	0.036047	0	0	0
Novel_015045	XLOC_15825.	transcript	0.137071	0.406418	0.273035	0.252079	0.233302	0.258674	0.36678	0.325827	0.410277
Novel_015046	XLOC_15825.	transcript	0	0	0	0	0.360209	0	0.001948	0	0
Novel_015047	XLOC_15825.	transcript	0	0.03074	0.030941	0	0.000984	0.050645	0	0.024672	0.02912
Novel_015048	XLOC_15828.	transcript	0	0	0	0	0.273171	0	0.009234	0	0
Novel_015049	XLOC_15828.	transcript	0.027763	0	0	0.000107	0	0.005684	0.170939	0.003419	0
Novel_015050	XLOC_15828.	transcript	0	0	0	0.29001	0	0	0.12007	0	0.011197
Novel_015051	XLOC_15828.	transcript	0	0.133591	0	0	0.004672	0	0	0	0
Novel_015052	XLOC_15828.	transcript	0.359033	0.064213	0.325434	0.024949	0.042454	0.257403	0	0.281296	0.209887
Novel_015053	XLOC_15828.	transcript	0	0	0	0.025196	0.062255	0	0.133399	0.072219	0
Novel_015054	XLOC_15828.	transcript	0	0	0	0.063101	0	0	0.425479	0	0
Novel_015055	XLOC_15834.	transcript	0.062335	0.021493	0.139619	0.112541	0.09352	0.093559	0.193629	0.063614	0.177356
Novel_015056	XLOC_15834.	transcript	0.100295	0.071512	0	0.098457	0.000641	0.205827	0.021133	0.001902	0
Novel_015057	XLOC_15834.	transcript	0.037448	0	0	0	0.277776	0	0	0.122252	0.384604
Novel_015058	XLOC_15834.	transcript	0.521319	0	0.310248	0	0	0	0.437981	0.216944	0
Novel_015059	XLOC_15834.	transcript	0.466496	0.830208	0.474978	0.540485	0.433013	0.420986	0	0	0.431024
Novel_015060	XLOC_15834.	transcript	0	0	0	0.006403	0	0	0.753086	0.241104	0
Novel_015061	XLOC_15834.	transcript	0	0	0	0	0	0	0	0.414099	0
Novel_015062	XLOC_15835.	transcript	0	0	0	0.08933	0.163522	0.064299	0.159164	0.091538	0
Novel_015063	XLOC_15836.	transcript	0.281271	0.298572	0.208005	0.180696	0.229971	0.195985	0.220346	0.279136	0.181285
Novel_015064	XLOC_15836.	transcript	0.449078	0.045299	0.377427	0.187939	0.168431	0.066528	0.07724	0.02626	0.023929
Novel_015065	XLOC_15837.	transcript	0.39252	0.344552	0.470893	0.356581	0.380154	0.469382	0.726845	0.479688	0.40509
Novel_015066	XLOC_15837.	transcript	0.004205	0.008967	0.005298	0.215611	0	0	0.006178	0	0
Novel_015067	XLOC_15838.	transcript	0	0	0.724967	0	0	0	0	0	0
Novel_015068	XLOC_15838.	transcript	0.04239	0.062626	0.0652	0.045261	0.069785	0.155403	0.156327	0.424307	0.030529
Novel_015069	XLOC_15838.	transcript	0.158064	0.165265	0.063248	0.033868	0	0.125438	0.164951	0.34572	0
Novel_015070	XLOC_15838.	transcript	0	0	0	0	0	0	0	0	0
Novel_015071	XLOC_15838.	transcript	0.00071	0.069494	0.355238	0.373409	0	0.102833	0.655523	0.344488	0.274258
Novel_015072	XLOC_15838.	transcript	0.973696	0.934076	0.874244	1.001465	1.67151	1.402775	1.179363	1.307144	1.321874
Novel_015073	XLOC_15838.	transcript	0	0	0	0	0	0	0	0	0
Novel_015074	XLOC_15842.	transcript	0	0	0	0	0	0	0.417	0.017144	0

Novel_015075	XLOC_15842.	transcript	0.293985	0	0.474664	0.727173	0.484989	0.612261	0.14687	0.789376	0.23863
Novel_015076	XLOC_15848.	transcript	0.176038	0	0.029278	0	0.276822	0.027571	0	0.034805	0.046797
Novel_015077	XLOC_15848.	transcript	0.339826	0	0	0.104111	0.234463	0	0.158485	0	0
Novel_015078	XLOC_15848.	transcript	0.031805	0	0.018184	0	0.021747	0.015803	0.034255	0.017565	0
Novel_015079	XLOC_15848.	transcript	0.384248	0	0	0.061643	0.228644	0.005116	0.42019	0.002228	0.026163
Novel_015080	XLOC_15848.	transcript	0.36685	0	0	0	0.262615	0	0	0.116696	0
Novel_015081	XLOC_15848.	transcript	0.18583	0	0	0.182134	0	0	0.00747	0.031092	0.020121
Novel_015082	XLOC_15848.	transcript	0.470063	0	0	0	0	0	0.195076	0	0
Novel_015083	XLOC_15848.	transcript	0.04472	0	0.015812	0.012493	0.409069	0.07058	0.047925	0	0
Novel_015084	XLOC_15848.	transcript	0.125033	0	0.143233	0	0.131322	0.00444	0.177143	0.066109	0
Novel_015085	XLOC_15848.	transcript	0.085908	0	0.087112	0	0.171318	0	0.358912	0	0
Novel_015086	XLOC_15861.	transcript	0.273963	0.372943	0.210287	0.320285	0.275096	0.309132	0.555178	0.374762	0.335168
Novel_015087	XLOC_15861.	transcript	0	0	0	0	0	0	0	0.001323	0
Novel_015088	XLOC_15862.	transcript	0	0.287118	0.282196	0.104773	0	0.080872	0.16737	0.126935	0.146706
Novel_015089	XLOC_15862.	transcript	0.467042	0.320261	0.48986	0.379377	0.644635	0.597353	0.268936	0.725335	0.365609
Novel_015090	XLOC_15862.	transcript	0.230595	0.166442	0.160674	0.137497	0.182299	0.155484	0.223257	0.190304	0.244675
Novel_015091	XLOC_15862.	transcript	0.123314	0.253724	0.035278	0.212509	0.179715	0.084802	0.314363	0.349744	0.295172
Novel_015092	XLOC_15862.	transcript	0.085132	0.101887	0.174658	0	0.089799	0.156658	0.148309	0	0
Novel_015093	XLOC_15864.	transcript	0	0	0.717928	0.662167	0	0	0.013884	0.167142	0
Novel_015094	XLOC_15864.	transcript	0.523153	0	1.001989	2.83895	1.695129	1.836475	0.916123	2.087007	0.518038
Novel_015095	XLOC_15866.	transcript	0	0	0	0	0	0	0	0.129421	0
Novel_015096	XLOC_15866.	transcript	0	0.020632	0	0	0	0.158913	0	0.148547	0
Novel_015097	XLOC_15866.	transcript	0.330891	0	0.126459	0.23557	0.193083	0.237407	0.127461	0.273803	0.19232
Novel_015098	XLOC_15866.	transcript	0.091461	0.023593	0	0	0.152886	0	0.173836	0	0
Novel_015099	XLOC_15866.	transcript	0	0	0.153631	0.002966	0.015026	0.016631	0	0.065556	0
Novel_015100	XLOC_15866.	transcript	0	0	0.000958	0.03541	0.095878	0	0	0	0
Novel_015101	XLOC_15866.	transcript	0	0	0	0.02306	0.033028	0	0.037151	0.054056	0
Novel_015102	XLOC_15866.	transcript	0	0	0	0	0.048586	0	0	0.039569	0
Novel_015103	XLOC_15866.	transcript	0	0	0.063133	0	0	0.143788	0	0.03396	0
Novel_015104	XLOC_15866.	transcript	0.52709	0	0.084184	0.03142	0.047849	0	0.140538	0.033367	0.099527
Novel_015105	XLOC_15866.	transcript	0	0	0.08925	0.03045	0.046764	0	0.135527	0.03378	0.049775
Novel_015106	XLOC_15866.	transcript	0	0	0	0.027889	0.031835	0	0.059231	0.065259	0
Novel_015107	XLOC_15866.	transcript	0	0	0	0	0	0	0	0	0
Novel_015108	XLOC_15866.	transcript	0	0	0.001275	0.035142	0	0.036507	0	0.076847	0
Novel_015109	XLOC_15866.	transcript	0	0.062865	0.042373	0.066154	0.054935	0.02217	0.144502	0.039045	0.046251
Novel_015110	XLOC_15866.	transcript	0.069436	0.002519	0.060383	0.079722	0.055686	0.134236	0.149276	0.03847	0.042022
Novel_015111	XLOC_15866.	transcript	0	0	0.059507	0	0	0.085709	0	0.035913	0
Novel_015112	XLOC_15866.	transcript	0.225679	0.263738	0.455146	0.059802	0.276466	0.284109	0.232417	0.150342	0.249748

Novel_015113	XLOC_15871.	transcript	0.016059	0	0.141433	0.00487	0	0	0	0.689648	0
Novel_015114	XLOC_15871.	transcript	0.620771	0.518346	0.800471	0.23906	0.742225	0	0.889949	0.241718	0.664366
Novel_015115	XLOC_15871.	transcript	0.271505	0.140643	0.674648	0.229638	0.665136	0.447827	0.370629	0.359314	0.627239
Novel_015116	XLOC_15871.	transcript	0	0.331836	0	0.379015	0	0.450448	0	0	0
Novel_015117	XLOC_15871.	transcript	0.19137	0	0.079135	0.019587	0.004753	0	0.467551	0.208865	0
Novel_015118	XLOC_15872.	transcript	0.76997	0	0	0.371197	0	0	0	0	0
Novel_015119	XLOC_15872.	transcript	0.091713	0.53093	0.121049	0.493212	0.024005	0.69553	0.177369	0.038161	0.618681
Novel_015120	XLOC_15872.	transcript	0	0	0.759058	0.003571	0.549832	0	0.992225	0.797086	0
Novel_015121	XLOC_15872.	transcript	0.028211	0.000301	0	0	0.236954	0.015638	0.170171	0.003324	0.225843
Novel_015122	XLOC_15872.	transcript	1.808702	1.88724	2.366424	2.837069	2.105274	2.127009	2.574718	3.126337	2.543043
Novel_015123	XLOC_15872.	transcript	0.064744	0	0.035697	0.022276	0.054551	0	0.136754	0.137061	0
Novel_015124	XLOC_15872.	transcript	0.623791	1.422321	1.150231	1.182401	1.063577	1.167488	0.851424	1.111451	0.963069
Novel_015125	XLOC_15873.	transcript	0	0.010141	0.105692	0.077179	0.170723	0.20509	0.206518	0.029017	0.021582
Novel_015126	XLOC_15873.	transcript	0.340856	0.140778	0.649209	0.790788	0.638094	0.951661	0.559067	0.511188	0.449959
Novel_015127	XLOC_15873.	transcript	0	0	0	0	0	0	0	0	0.282949
Novel_015128	XLOC_15875.	transcript	0.006853	0.009312	0.008432	0.005024	0	0.001625	0.008451	0.001732	0.189396
Novel_015129	XLOC_15875.	transcript	0.366818	0.496475	0.445617	0.692795	0.641983	0.475419	0.867822	0.69487	0.423744
Novel_015130	XLOC_15875.	transcript	0	0	0	0.381135	0	0	0	0	0
Novel_015131	XLOC_15876.	transcript	0.099003	0.027766	0	0	0	0	0.606592	0.151888	0.010067
Novel_015132	XLOC_15876.	transcript	1.596913	0	2.276109	1.038675	1.504486	1.45668	2.052127	0.393474	2.074157
Novel_015133	XLOC_15876.	transcript	0	0.057706	0.294676	0	0	0	0.008097	0.00625	0
Novel_015134	XLOC_15876.	transcript	1.169824	1.493763	1.412883	1.490458	1.622587	1.827237	2.153907	1.486466	2.196788
Novel_015135	XLOC_15877.	transcript	0	1.341055	0	0.008132	0	0.045402	0.041418	0.02609	0.062628
Novel_015136	XLOC_15877.	transcript	0.056094	0.646299	0	0	0.038461	0.001152	0.002085	0	0
Novel_015137	XLOC_15877.	transcript	0	0.370009	0	0	0	0	0	0	0
Novel_015138	XLOC_15878.	transcript	0.075758	0	0	0	0.362564	0	0.083036	0.057211	0
Novel_015139	XLOC_15880.	transcript	1.8735	0	1.121682	0.260201	0.800107	0.46854	0	0.601453	0.207857
Novel_015140	XLOC_15880.	transcript	0	0.536581	0	0	0	0.523721	0	0.471283	0
Novel_015141	XLOC_15880.	transcript	0.790092	0.951466	0.894374	0.380814	0.684898	0.632302	1.117057	1.020383	0.407926
Novel_015142	XLOC_15880.	transcript	0.457642	0.574321	0.620771	1.245433	1.214452	0.418627	1.679153	0.330423	1.121173
Novel_015143	XLOC_15881.	transcript	0.407166	0	0	0.001996	0	0	8.90E-05	0	0
Novel_015144	XLOC_15882.	transcript	0.439569	0.160589	0.414492	0.185958	0.416273	0.398399	0.616808	0.380358	0.425488
Novel_015145	XLOC_15882.	transcript	0.014299	0.025138	0.028004	0.063323	0.052012	0.061583	0.030314	0.357221	0.027029
Novel_015146	XLOC_15882.	transcript	0.243843	0.360797	0.210115	0.345491	0.358051	0.290104	0.527938	0.009183	0.264227
Novel_015147	XLOC_15883.	transcript	0	0	0	0	0	0	0	0	0
Novel_015148	XLOC_15883.	transcript	0	0	0.031152	0	0	0	0.003124	0	0
Novel_015149	XLOC_15883.	transcript	0	0	0.67626	0	0.644963	0.000672	0.484071	0	0
Novel_015150	XLOC_15883.	transcript	0.722381	0.209924	0	0.540954	0.041571	0.535553	0.751358	0.818066	0.803361

Novel_015151	XLOC_15884.	transcript	0.076629	0.044493	0.050407	0.271959	0.105599	0.0684	0.283721	0.149995	0.041457
Novel_015152	XLOC_15884.	transcript	0.075945	0.329258	0.232498	0.071532	0.103669	0.053875	0.158525	0.200227	0.129279
Novel_015153	XLOC_15885.	transcript	2.753211	2.547319	2.69339	2.277195	3.005435	2.493621	3.078785	3.216627	2.625706
Novel_015154	XLOC_15887.	transcript	0.164988	0.306186	0.170364	0.17593	0.134171	0.165939	0.108505	0.142678	0.203321
Novel_015155	XLOC_15887.	transcript	0	0.008285	0	0	0.009519	0	0	0	0
Novel_015156	XLOC_15887.	transcript	0.069665	0.058697	0.069591	0.059862	0.036526	0.1621	0.290504	0.054344	0.237049
Novel_015157	XLOC_15887.	transcript	0.09105	0.075859	0.181968	0.109784	0.227754	0.123336	0.132908	0.103125	0
Novel_015158	XLOC_15887.	transcript	0.182367	0	0.123332	0.109904	0.091141	0.104879	0.067227	0.068541	0.195359
Novel_015159	XLOC_15890.	transcript	0.149064	0.217546	0.343796	0.141363	0.320116	0.126707	0.202271	0.300427	0.233097
Novel_015160	XLOC_15890.	transcript	0	0.080174	0	0.136263	0.021895	0.108234	0	0.059613	0.106613
Novel_015161	XLOC_15890.	transcript	0.049076	0	0.239769	0.101671	0.243961	0.189085	0.044838	0.147325	0
Novel_015162	XLOC_15893.	transcript	0.416617	0.581534	0.239686	0.345204	0.550174	0.449373	0.603212	0.47281	0.391465
Novel_015163	XLOC_15893.	transcript	0	0.269992	0.080195	0.037792	0.38433	0.361854	0.219581	0.009661	0.142506
Novel_015164	XLOC_15895.	transcript	0.039785	0.229277	0.082646	0.025681	0.094021	0.026496	0.091668	0.176669	0
Novel_015165	XLOC_15895.	transcript	3.645006	6.036686	4.141472	5.15868	5.236796	7.207354	3.585503	4.486977	6.232531
Novel_015166	XLOC_15896.	transcript	0.247794	0.573249	0.630165	0.237065	0.11737	0.687463	0.51965	0.533596	0.557943
Novel_015167	XLOC_15898.	transcript	0.100624	0.050936	0.062454	0.060908	0	0.245311	0.570956	0.099475	0.135066
Novel_015168	XLOC_15898.	transcript	0.268613	0.210946	0.797469	0.626522	0.800319	0.66196	0.308771	0.569854	0.562239
Novel_015169	XLOC_15899.	transcript	0.414399	0	0	0	0.30007	0.521353	0.766281	0	0.397884
Novel_015170	XLOC_15899.	transcript	0.270855	0.148105	0.031959	0.237523	0.364684	0.249075	0.242795	0.718003	0
Novel_015171	XLOC_15899.	transcript	0.103555	0.234649	0.591394	0.47513	0.181672	0.180848	0.251548	0.243458	0.163139
Novel_015172	XLOC_15900.	transcript	0.097653	0.119138	0.157817	0.177048	0.135749	0.190154	0.260275	0.123725	0.10481
Novel_015173	XLOC_15900.	transcript	0.125056	0	0.094073	0	0.122985	0.04304	0.244911	0.049455	0.071253
Novel_015174	XLOC_15900.	transcript	0.819191	0.262471	0.670164	0.116651	0.955491	0.764447	0.200256	0.143951	0.701234
Novel_015175	XLOC_15900.	transcript	0	0.748236	0	0.731888	0	0	1.148902	1.077105	0
Novel_015176	XLOC_15905.	transcript	0.69043	0.395139	0.460458	0.505516	0.550803	0.482626	0.716571	0.500455	0.33826
Novel_015177	XLOC_15905.	transcript	1.002588	0.281119	0.095468	0.552485	1.351201	0.983489	1.601382	0.909628	1.043501
Novel_015178	XLOC_15905.	transcript	0	1.087489	1.139893	0.611225	0	0	0.30613	0.330468	1.927005
Novel_015179	XLOC_15906.	transcript	0	0	0.038227	0.314152	0.014236	0.142186	0.180646	0.153465	0.05647
Novel_015180	XLOC_15906.	transcript	0.146057	0.019411	0.148755	0	0.160351	0.05491	0.039177	0.005969	0
Novel_015181	XLOC_15906.	transcript	0	0.445415	0.057727	0.003374	0.088418	0	0.033462	0.001531	0
Novel_015182	XLOC_15906.	transcript	0.110839	0.781304	0.239542	0.533597	0.419066	0.344779	0.740026	0.536186	0.545184
Novel_015183	XLOC_15906.	transcript	0.228733	0.050754	2.208543	1.547529	3.426445	0.0825	3.590691	2.374964	1.054714
Novel_015184	XLOC_15906.	transcript	3.376513	0	0.928775	1.524871	0.246062	2.736725	1.02362	0.219982	1.021191
Novel_015185	XLOC_15906.	transcript	0	0.545232	0.044012	0	0.005594	0	0.102756	0.040229	0
Novel_015186	XLOC_15906.	transcript	0	0	0	0	0	0	0	0	0
Novel_015187	XLOC_15907.	transcript	0.512158	0.673331	0.677987	0.483799	0.524919	0.569587	0.622072	0.415312	0.329992
Novel_015188	XLOC_15907.	transcript	1.359565	1.562524	2.39433	2.771743	2.164652	2.494141	1.885051	2.015794	2.111272

Novel_015189	XLOC_15907.	transcript	0	0	0.328511	0	0	0.059988	0.057248	0	0
Novel_015190	XLOC_15909.	transcript	0.074769	0.062244	0.10163	0.07741	0.160995	0.158784	0.267925	0.2139	1.19822
Novel_015191	XLOC_15909.	transcript	0.037217	0.058184	0.418229	0.881635	0.011648	1.225212	0.041295	0.079444	0.018337
Novel_015192	XLOC_15909.	transcript	0.957223	0.261747	0.845756	0.669835	1.203148	0.1952	1.057068	0.866618	1.505852
Novel_015193	XLOC_15909.	transcript	0	0	0.411819	0	0	0	0.796806	0	0
Novel_015194	XLOC_15909.	transcript	0.565261	0.123071	0.132511	1.409878	0.171595	0.158243	0.192305	0.138122	0.118399
Novel_015195	XLOC_15909.	transcript	2.94179	4.048131	4.102235	3.922379	5.398374	4.809738	6.079007	4.549545	4.665113
Novel_015196	XLOC_15909.	transcript	1.16078	1.322451	1.541544	1.161844	0.96774	0.863201	1.874296	2.170074	1.643284
Novel_015197	XLOC_15909.	transcript	0.323864	0.753891	0.440271	0.581107	0.368827	0.443911	0.68351	0.409215	0.402927
Novel_015198	XLOC_15915.	transcript	0.223746	0	0.090628	0.016341	0.043157	0.038919	0.089852	0.100465	0
Novel_015199	XLOC_15915.	transcript	0.027706	0.230193	0.119451	0.184971	0.25945	0.083794	0.025186	0	0.201853
Novel_015200	XLOC_15916.	transcript	0	0	0.380925	0	0.989708	0	0.678955	0.461264	0
Novel_015201	XLOC_15916.	transcript	0.015879	0	0	0	0.228666	0.011926	0.198472	0.601925	0.033815
Novel_015202	XLOC_15916.	transcript	0.567495	0.130214	0.412267	0.117281	0	0.188298	0.203354	0	0.427513
Novel_015203	XLOC_15916.	transcript	0	0	0	0	0	0	0	0	0
Novel_015204	XLOC_15916.	transcript	0.420652	0	0.076423	0.162832	0.346784	0.616387	0.010625	0	0
Novel_015205	XLOC_15916.	transcript	0	2.50E-05	0.311949	0	0	0	2.90E-05	0.30107	0.21898
Novel_015206	XLOC_15916.	transcript	0.336179	0.412965	1.278439	1.021251	0.706756	0.466217	0.984728	0.677962	0.973828
Novel_015207	XLOC_15916.	transcript	0	0.219943	0	0.285658	0	0.166177	0.283546	0.16062	0.002323
Novel_015208	XLOC_15916.	transcript	0	0	0	0	0	0.157949	0.099006	0	0
Novel_015209	XLOC_15917.	transcript	0	0.81811	0.025059	0.051709	0.057205	0.062769	0.065812	0.012975	0.080734
Novel_015210	XLOC_15917.	transcript	0.103914	0.014987	0.29262	0.354176	0.248444	0.176375	0.347678	0.263312	0.225051
Novel_015211	XLOC_15918.	transcript	6.880296	7.550107	11.24912	13.36243	11.3833	14.31155	9.712609	12.74028	15.03062
Novel_015212	XLOC_15918.	transcript	0	10.67241	0	0	0	0	0	0	3.22028
Novel_015213	XLOC_15918.	transcript	33.49148	0	0	0	0	0	0	0	1.491915
Novel_015214	XLOC_15918.	transcript	13.85135	12.69676	0	0	0	0	0	0	0.20952
Novel_015215	XLOC_15918.	transcript	3.120847	0	0	0	0	0	0	0	0
Novel_015216	XLOC_15918.	transcript	10.75667	0	23.16701	29.17237	30.95884	35.02422	66.05074	31.14685	23.60229
Novel_015217	XLOC_15921.	transcript	0.091028	1.469947	0	0	0	0	0	0	0
Novel_015218	XLOC_15922.	transcript	0.168763	0.099897	0.348725	0.318079	0.335811	0.39783	0.348884	0.297476	0.286691
Novel_015219	XLOC_15922.	transcript	0.038258	0	0	0	0.203985	0	0	0.194988	0
Novel_015220	XLOC_15922.	transcript	0	0	0	0	0.053676	0	0.059273	0.022515	0
Novel_015221	XLOC_15922.	transcript	0.218853	0.010065	0.149705	0.02615	0.013191	0.155644	0.171974	0.002839	0.119404
Novel_015222	XLOC_15922.	transcript	0.116812	0	0	0	0.501532	0.095469	0.022354	0.387658	0
Novel_015223	XLOC_15922.	transcript	0	0	0	0	0	0	0.279992	0	0
Novel_015224	XLOC_15922.	transcript	0.005645	0.024918	0.286952	0.118318	0.006109	0.003222	0.103501	0.001253	0.150016
Novel_015225	XLOC_15922.	transcript	0	0	0	0	0	0	0.01723	0	0
Novel_015226	XLOC_15924.	transcript	0.691962	0.971053	0.902332	0.710015	1.584101	0.792538	1.622822	0.823354	0.790894

Novel_015227	XLOC_1592[illegible]	transcript	0.107289	0.03065	0.11912	0.045595	0.088369	0.069848	0.154415	0.0831	0.040211
Novel_015228	XLOC_1592[illegible]	transcript	0	0	0	0	0.337848	0.002587	0.001016	0	0.002857
Novel_015229	XLOC_1592[illegible]	transcript	0	0.193242	0	0	0	0.202466	0	0.270835	0
Novel_015230	XLOC_1592[illegible]	transcript	0	0	0	0	0	0.088183	0.278123	0.077546	0
Novel_015231	XLOC_1592[illegible]	transcript	0.43873	0.226505	0.455575	0.312349	0.102262	0.280665	0.206319	0.152046	0.399042
Novel_015232	XLOC_1592[illegible]	transcript	0.20591	0	0.090547	0	0.004389	0	0.054015	0.074046	0.096234
Novel_015233	XLOC_1592[illegible]	transcript	0.595511	0.406028	0.524706	0.560642	0.57462	0.528452	0.408839	0.38319	0.365672
Novel_015234	XLOC_1592[illegible]	transcript	0.364552	0.425009	0.390894	0.581571	0.684266	0.677729	0.333338	0.510578	0.503648
Novel_015235	XLOC_1592[illegible]	transcript	0.004253	0	0.318663	0	0	0	0.171354	0	0
Novel_015236	XLOC_1593[illegible]	transcript	8.099748	6.178041	9.604722	5.832978	9.484129	10.13467	4.576376	4.873951	7.325685
Novel_015237	XLOC_1593[illegible]	transcript	0.183033	0.041254	0.975143	1.218573	0.191309	1.049849	0.485939	0.970332	0.380785
Novel_015238	XLOC_1593[illegible]	transcript	0.672757	0.179234	1.330484	1.243605	0.200915	0.662634	2.699559	0.636671	1.10591
Novel_015239	XLOC_1593[illegible]	transcript	0	0	0	0	0.247695	0	0	0.001265	0
Novel_015240	XLOC_1593[illegible]	transcript	0	0	0	0	2.355309	0	0.053267	0.076175	0
Novel_015241	XLOC_1593[illegible]	transcript	0.005512	0	0.005271	0.021774	1.796707	0.003254	0	1.90261	0.004566
Novel_015242	XLOC_1593[illegible]	transcript	1.042255	0.424347	1.47254	1.55458	1.608449	1.144825	1.638507	1.244876	1.585457
Novel_015243	XLOC_1593[illegible]	transcript	1.01253	2.465749	2.332535	2.717355	2.529764	2.602279	1.388868	2.552944	1.830357
Novel_015244	XLOC_1593[illegible]	transcript	0	1.48054	0.010518	1.419358	0.791637	0.401999	1.174207	0.265713	0.44148
Novel_015245	XLOC_1593[illegible]	transcript	34.66581	30.08662	46.92333	53.1989	57.6619	49.06406	64.42188	48.41037	48.10918
Novel_015246	XLOC_1593[illegible]	transcript	0.003284	0.116744	0.122667	0.32309	0	0	0	0.291407	0.275826
Novel_015247	XLOC_1593[illegible]	transcript	0.120151	0.111338	0.12451	0.03118	0.029568	0.232065	0	0	0
Novel_015248	XLOC_1593[illegible]	transcript	0	0	0	0.00137	0	0	0.343707	0	0
Novel_015249	XLOC_1593[illegible]	transcript	0	1.709378	0.786661	0.600872	0.865867	0.688599	0.765785	0.321199	0.665797
Novel_015250	XLOC_1593[illegible]	transcript	0.317632	0.386314	0.03959	0.252368	0.104917	0.446005	0.246468	0.395178	0.438483
Novel_015251	XLOC_1593[illegible]	transcript	0.405618	0.423152	0.631416	0.158905	0.709332	0.36795	0.553182	0.336935	0.129572
Novel_015252	XLOC_1593[illegible]	transcript	0.260985	0.22131	0.261015	0	0.180146	0.198052	0.314994	0	0.251614
Novel_015253	XLOC_1593[illegible]	transcript	1.491811	1.348587	1.916387	1.529706	1.666798	1.321836	1.704845	2.055224	1.720497
Novel_015254	XLOC_1593[illegible]	transcript	0	0	0	0.478938	0	0	0	0.247409	0
Novel_015255	XLOC_1593[illegible]	transcript	0.159101	0.146957	0.379286	0.354672	0.437688	0.191408	0.772448	0.394758	0.515021
Novel_015256	XLOC_1593[illegible]	transcript	0.122755	0	0.004412	0	0.047187	0.115438	6.00E-05	0.061492	0.039796
Novel_015257	XLOC_1593[illegible]	transcript	0.17926	0	0.548279	1.584131	0.635418	0.226498	0.459823	0.385229	0.114958
Novel_015258	XLOC_1593[illegible]	transcript	0.717514	3.359602	1.62699	0.982371	1.113716	1.218974	1.249133	1.326454	1.361255
Novel_015259	XLOC_1594[illegible]	transcript	0	0.062939	0.320946	0.265203	0.093012	0.128082	0.315781	0	0.245428
Novel_015260	XLOC_1594[illegible]	transcript	0.423368	0.581644	0.030268	0.085803	0.23774	0.272402	0.352299	0.320614	0.045394
Novel_015261	XLOC_1594[illegible]	transcript	0.467021	0.183717	1.005343	0.079185	0.842941	0	0.383812	0.38514	0.537182
Novel_015262	XLOC_1594[illegible]	transcript	0	0.923257	0.174342	1.067961	0.096495	0.727818	0.467794	0.594436	0.411214
Novel_015263	XLOC_1594[illegible]	transcript	0.426747	0.018801	0.594478	0.157576	0.382823	0.427826	0.895067	0.617277	0.216923
Novel_015264	XLOC_1594[illegible]	transcript	0.100235	0.013999	0.099656	0.143523	0.090019	0.036116	0.064031	0.087916	0.06819

Novel_015265	XLOC_1594[illegible]	transcript	1.030735	1.275549	1.681007	1.863508	2.73923	1.683843	2.442762	1.671994	1.53555
Novel_015266	XLOC_15947	transcript	0	0	0.001117	0	0	0	0	0.143211	0
Novel_015267	XLOC_15947	transcript	0.005627	0	0	0	0	0.02516	0.012827	0	0.155283
Novel_015268	XLOC_15947	transcript	0.010835	0	0	0	0	0	0.009111	0	0.06596
Novel_015269	XLOC_15947	transcript	0.456458	0.27047	0.492555	0.545838	0.575427	0.442009	0.688117	0.533938	0
Novel_015270	XLOC_15947	transcript	0	0	0	0	0	0	0	0	0.125783
Novel_015271	XLOC_1594[illegible]	transcript	0	0	0	1.828993	0	0	0	0.381054	0
Novel_015272	XLOC_1594[illegible]	transcript	0	0	0.258309	0	0	0	0	0	0
Novel_015273	XLOC_1594[illegible]	transcript	1.169912	2.086088	1.62777	0.872104	1.539335	1.882494	1.60221	1.772627	1.252828
Novel_015274	XLOC_1595[illegible]	transcript	0	0.003906	0	0	0	0	0	0.004224	0
Novel_015275	XLOC_1595[illegible]	transcript	0	0	0	0.122811	0	0.012761	0	0	0
Novel_015276	XLOC_1595[illegible]	transcript	3.526135	0.217562	0.317009	0.09984	0.048305	0.344668	0.153807	0.149249	0.027447
Novel_015277	XLOC_1595[illegible]	transcript	0	2.049112	0	0.138324	0.151607	0	0.185851	0.029379	0.099617
Novel_015278	XLOC_1595[illegible]	transcript	0	0.038417	0	0.010671	0	0.077728	0.110492	0.033757	0
Novel_015279	XLOC_1595[illegible]	transcript	0.634551	0	0	0.234414	0.336378	0	0	0.319946	1.089028
Novel_015280	XLOC_1595[illegible]	transcript	0.114835	0.067655	0.011864	0.005287	0.110152	0.404319	0.196197	0.148997	0.034139
Novel_015281	XLOC_1595[illegible]	transcript	0	0	0	0	0	0	0	0	0
Novel_015282	XLOC_1595[illegible]	transcript	0.302969	0.095427	0.254544	0.709929	0.307586	0.238067	0	0.398153	0
Novel_015283	XLOC_1595[illegible]	transcript	0	0	0	0	0.165985	0	0.32657	0	0
Novel_015284	XLOC_1595[illegible]	transcript	0.119311	0.253925	0.018459	0.028782	0.017603	0	0.128735	0.041442	0.414043
Novel_015285	XLOC_1595[illegible]	transcript	0	0.13096	0	0.068114	0.068428	0	0.191533	0.050998	0.116395
Novel_015286	XLOC_1595[illegible]	transcript	0	0.102237	0.081896	0.061833	0.007129	0	0.037509	0.01583	0.14957
Novel_015287	XLOC_1595[illegible]	transcript	0.35127	0.163243	0.409085	0.318195	0.153334	0.217799	0.552994	0.268669	0.349098
Novel_015288	XLOC_15951	transcript	0.116617	0.01745	0.091702	0.021401	0.007185	0.106398	0.038071	0.088125	0
Novel_015289	XLOC_15951	transcript	0	0.281209	0.123072	0.117021	0.115364	0.018753	0.102686	0.097888	0.039367
Novel_015290	XLOC_15952	transcript	2.019606	0	0.924301	0	0.671507	0.930921	0.283003	0.425398	1.41703
Novel_015291	XLOC_15952	transcript	4.123431	5.930008	8.097399	8.287771	7.722907	7.059594	7.740197	8.820249	6.060599
Novel_015292	XLOC_15952	transcript	2.80794	3.594371	6.591557	8.56733	7.428806	8.724305	6.998687	3.809045	4.992884
Novel_015293	XLOC_1595[illegible]	transcript	0.00019	0	0	0	0	0.275837	0	0	0
Novel_015294	XLOC_1595[illegible]	transcript	0.235574	0.044211	0.227952	0.288455	0.18759	0.01269	0.333138	0.188182	0.132426
Novel_015295	XLOC_1595[illegible]	transcript	0.729281	0.084316	0.53732	0.726031	0.728142	0.462137	0	0.305486	0.411979
Novel_015296	XLOC_15954	transcript	0.27958	0.561061	0.591061	0.638729	0.64174	0.793624	0.528694	0.391547	0.573563
Novel_015297	XLOC_1595[illegible]	transcript	0.453444	0.006825	0.492963	1.013912	0	0.726113	1.659242	0.837294	1.03029
Novel_015298	XLOC_1595[illegible]	transcript	0.710947	0.959588	0.872979	0.836437	1.140903	0.998593	0.324839	0.723484	0.443621
Novel_015299	XLOC_15957	transcript	0.560159	0.257602	0.795061	0.592058	0.927799	0.538048	0.633712	0.518886	0.349247
Novel_015300	XLOC_15957	transcript	0.558795	0.27331	0.348069	0.129939	0.294459	0.195158	0.488437	0.247066	0.226713
Novel_015301	XLOC_15957	transcript	0.214383	0.288789	0.360452	0.184931	0.285247	0.181818	0.272326	0.284287	0.261924
Novel_015302	XLOC_15957	transcript	0.156356	0	0.106875	0.135303	0.159882	0	0.205426	0.178581	0.271722

Novel_015303	XLOC_15957.	transcript	0	0.122807	0.003971	0.008569	0.132992	0	0	0	0
Novel_015304	XLOC_15957.	transcript	0.147814	0.204847	0.165717	0.017323	0	0.182804	0.160858	0.084035	0.04475
Novel_015305	XLOC_15957.	transcript	0.280169	0.027405	0.117376	0.146034	0.231318	0.308148	0.234529	0.125528	0
Novel_015306	XLOC_15958.	transcript	4.952826	1.753873	4.844925	2.607222	2.562451	3.181355	6.225173	5.724107	0.713334
Novel_015307	XLOC_15958.	transcript	0.346227	1.779751	0.246622	1.644507	2.213531	0.313197	0.257967	0.253463	3.222119
Novel_015308	XLOC_15958.	transcript	0.274243	0.569494	0.223599	0.84879	0	0.624916	0.191354	0.400287	0
Novel_015309	XLOC_15958.	transcript	1.042321	5.79809	1.014953	5.237638	5.334304	1.758269	1.250077	1.898747	2.810627
Novel_015310	XLOC_15958.	transcript	0.395839	0.172929	0.46522	0.733182	0	0.258619	0.612029	0.332737	0
Novel_015311	XLOC_15958.	transcript	0.000911	0	0.007655	0.006168	0.014202	0.470876	0	0.005613	0
Novel_015312	XLOC_15958.	transcript	0.85073	0.221189	0.44955	0.27981	0.439544	0.27649	0.81167	0.758556	0
Novel_015313	XLOC_15958.	transcript	0	0	0.23422	0.795261	0	0	0.242789	0	0
Novel_015314	XLOC_15958.	transcript	0	0	0.154337	0.40799	0	0	0.115104	0	0
Novel_015315	XLOC_15958.	transcript	0.016141	0	0.079942	0.022203	0.085499	0	0.095566	0.147303	0.640191
Novel_015316	XLOC_15958.	transcript	0.13606	0.035346	0.194579	0.289125	0.302177	0.394609	0.251219	0.311012	0.184573
Novel_015317	XLOC_15958.	transcript	0	0.002975	0	0	0	0	0	0	0
Novel_015318	XLOC_15958.	transcript	0	0.150849	0	0	0	0	0.095106	0.156315	0
Novel_015319	XLOC_15959.	transcript	0	0	0.0809	0.235877	0	0.120172	0	0	0.125103
Novel_015320	XLOC_15959.	transcript	0.282528	0.298325	0.07792	0	0.380415	0.128257	0.027461	0.702876	0.411679
Novel_015321	XLOC_15960.	transcript	0.448766	0.504382	0.422066	0	0	0.102977	0.214915	0	0.247051
Novel_015322	XLOC_15960.	transcript	0.325781	0.50434	0	0.41777	0.306697	0.090266	0.301138	0.128073	0
Novel_015323	XLOC_15961.	transcript	3.894401	1.670206	4.102266	3.180621	3.888665	3.718099	4.792869	4.030802	2.794362
Novel_015324	XLOC_15961.	transcript	0.049079	0.121145	0	0.164382	0.186683	0.256151	0.274064	0.215437	0.249174
Novel_015325	XLOC_15961.	transcript	0.073101	0	0.296935	0	0.178128	0.180786	0.137101	0	0
Novel_015326	XLOC_15962.	transcript	0.556794	0.625392	0.804512	0.679615	0.912742	0.886215	1.125704	0.879016	0.675731
Novel_015327	XLOC_15962.	transcript	0	0	0	0	0.015789	0	0.158375	0.058097	0
Novel_015328	XLOC_15963.	transcript	0.089722	0.270543	0.043178	0.562381	0.786025	0.035288	0.447899	0.435293	0.782692
Novel_015329	XLOC_15963.	transcript	0.653316	0.254361	0.893965	0.597489	0.300621	0.966621	0.65689	0.682561	0.15972
Novel_015330	XLOC_15963.	transcript	5.327241	1.78338	7.216269	6.877116	7.256019	8.339126	7.857642	7.10618	7.998841
Novel_015331	XLOC_15963.	transcript	1.068944	0.765201	0.963946	0.868993	0.993978	0.93707	0.863257	0.887379	1.272575
Novel_015332	XLOC_15965.	transcript	0.019427	0.120179	0.16786	0.055637	0.175827	0.013319	0.134335	0.032138	0.053218
Novel_015333	XLOC_15965.	transcript	0	0	0	0	0	0	0	0	0
Novel_015334	XLOC_15965.	transcript	3.087432	3.523088	2.853161	2.638403	2.777577	2.761612	3.850511	3.211728	3.391623
Novel_015335	XLOC_15966.	transcript	0.319648	1.035191	0.548105	0.663772	0.758593	0.781442	0.606198	0.820821	0.45642
Novel_015336	XLOC_15966.	transcript	0	0.107987	0	0.227366	0	0	0.083689	0.005021	0.009102
Novel_015337	XLOC_15966.	transcript	0	0.398211	1.798221	1.037242	0.752893	0.670743	0.218148	0.761744	0.731905
Novel_015338	XLOC_15966.	transcript	2.406814	1.236494	2.249055	4.469785	4.189871	4.415924	4.11204	3.538126	3.0824
Novel_015339	XLOC_15967.	transcript	0.250363	0.054438	0.132341	0.493339	0.265698	0.270187	0.334311	0.078782	0.351493
Novel_015340	XLOC_15967.	transcript	0.193402	0	0.205882	0	0.514252	0.340979	0.561933	0.547704	0

Novel_015341	XLOC_15972.	transcript	0.127532	0	0	0.060039	0.061484	0.39574	0.083293	0.057528	0.115232
Novel_015342	XLOC_15972.	transcript	0	0	0	0	0.047172	0	0	0	0.138157
Novel_015343	XLOC_15972.	transcript	0.081741	0	0	0.000685	0.672786	0.013268	0	0.002178	0.212811
Novel_015344	XLOC_15972.	transcript	0	0	0	0.001981	0	0	0	0	0
Novel_015345	XLOC_15972.	transcript	0	0.002458	0.006772	0	0	0	0.004425	0	0
Novel_015346	XLOC_15972.	transcript	0	0	0	0	0	0	0	0.058724	0
Novel_015347	XLOC_15972.	transcript	0	0.000172	0.388314	0.436935	0.001435	0.589014	0.514621	0.06235	0.296281
Novel_015348	XLOC_15972.	transcript	0.861492	1.178677	0.173078	0	0.070938	4.00E-04	0.763321	0	0
Novel_015349	XLOC_15975.	transcript	0.166705	0.138927	0.176277	0.14454	0.189878	0.184112	0.245751	0.211436	0.139298
Novel_015350	XLOC_15975.	transcript	0.015029	0.060816	0.18581	0.155301	0.201369	0.14946	0.102642	0.130357	0
Novel_015351	XLOC_15975.	transcript	0.01098	0.291549	0.101768	0.070534	0	0.115186	0.16263	0.080435	0.028802
Novel_015352	XLOC_15975.	transcript	0.314816	0	0	0.025884	0.048316	0.049853	0	0.070303	0.17095
Novel_015353	XLOC_15976.	transcript	0	0	0	0	0.037202	0.014506	0	0	0
Novel_015354	XLOC_15976.	transcript	0.151453	0.179032	0.177323	0.1683	0.109579	0	0.025342	0.159382	0.185382
Novel_015355	XLOC_15976.	transcript	0	0	0.058905	0	0	0.172133	0.12086	0.008484	0
Novel_015356	XLOC_15976.	transcript	0	0	0	0	0	0	0	0	0.44686
Novel_015357	XLOC_15976.	transcript	0	0.284501	0	0.207436	0	0	0	0	0
Novel_015358	XLOC_15976.	transcript	2.310561	1.423997	2.114723	2.24909	2.450193	2.436093	2.658578	2.453083	2.128633
Novel_015359	XLOC_15983.	transcript	0.290948	0.110742	0.205087	0.146227	0.05118	0.313808	0.018238	0.227843	0.246663
Novel_015360	XLOC_15983.	transcript	0	0.018815	0	0	0	0	0.245864	0	0
Novel_015361	XLOC_15984.	transcript	0.522106	0	0.024591	0	0.268111	0.056127	0.080021	0.063479	0.040981
Novel_015362	XLOC_15993.	transcript	3.807009	3.905072	2.738516	3.285433	2.772547	3.064002	2.860767	2.608253	2.339099
Novel_015363	XLOC_15993.	transcript	1.375941	0.984265	1.315546	0.440033	1.037058	0	1.415543	1.267914	0.865362
Novel_015364	XLOC_15997.	transcript	0	0	0	0.327861	0.011852	0	0	0.081366	0.391256
Novel_015365	XLOC_15997.	transcript	0.028728	0.146281	0.0455	0.271777	0.108727	0.152639	0.189338	0.49375	0.043472
Novel_015366	XLOC_15999.	transcript	0.03425	0.1484	0	0	0	0.074061	0.188803	0.071655	0.071193
Novel_015367	XLOC_16001.	transcript	0.447882	0.212049	0.454301	0.605684	0.755193	0.699731	0.486438	0.393336	0.234159
Novel_015368	XLOC_16001.	transcript	0	0.01477	0	0.253005	0	0	0	0	0
Novel_015369	XLOC_16003.	transcript	1.86276	1.825408	1.776522	1.549125	1.851842	1.974736	1.88937	1.753167	1.517787
Novel_015370	XLOC_16003.	transcript	0.084422	0	0.371406	0.133962	0.349046	0.274846	0.193653	0	0
Novel_015371	XLOC_16003.	transcript	0.567539	0.615897	0.700645	0.267385	0.501786	0.40894	0.485973	0.599946	0.338996
Novel_015372	XLOC_16003.	transcript	0	0	0	0	0.132028	0	0.003917	0	0.313248
Novel_015373	XLOC_16003.	transcript	0	0	0	0.401579	0.148759	0	0.262202	0	0.341383
Novel_015374	XLOC_16003.	transcript	0	0	0	0	0.215172	0	0.140999	0.179222	0
Novel_015375	XLOC_16003.	transcript	0.338054	0.037163	0.436562	0.009684	0.003397	0.415108	0.038589	0.143851	0.01007
Novel_015376	XLOC_16005.	transcript	0.307694	0	0.064981	0.024299	0.065963	0.140392	0.589946	0.149899	0.338546
Novel_015377	XLOC_16006.	transcript	0.597424	1.855836	1.442317	2.163122	1.814432	1.695379	1.210497	1.843797	0.819186
Novel_015378	XLOC_16007.	transcript	0	0	0	0	0.278362	0	0	0	0

Novel_015379	XLOC_16007.	transcript	0.278629	0.063272	0.206921	0.187666	0.089918	0.149077	0.339848	0.175561	0.13733
Novel_015380	XLOC_16008.	transcript	1.171829	0.026339	3.032933	9.406132	6.097332	4.05588	3.093489	8.561807	1.132265
Novel_015381	XLOC_16008.	transcript	0	0	0.231199	0.224986	0.254079	0.167905	0.059638	0.142629	0
Novel_015382	XLOC_16008.	transcript	0.11547	0.003613	0	0.520548	0	0.300095	0.133069	0.435874	0.171537
Novel_015383	XLOC_16008.	transcript	0	0	0.152874	0	0.410764	0.166337	0.425943	0	0
Novel_015384	XLOC_16008.	transcript	0.021581	0	0	0	0.19232	0	0.040134	0.036986	0
Novel_015385	XLOC_16008.	transcript	0.127848	0.039393	0.167573	0.172812	0.04504	0.130554	0.222597	0.051861	0.037594
Novel_015386	XLOC_16009.	transcript	0.083149	0.030225	0.46579	0.063865	0.047762	0	0.103825	0.01392	0.149253
Novel_015387	XLOC_16010.	transcript	0.079889	0.519046	0	0.039541	0	0	0.361092	0.027012	0.135867
Novel_015388	XLOC_16010.	transcript	0.524368	3.333471	0.063764	0.079393	0.369445	0.526873	0.474866	0.192073	0.108618
Novel_015389	XLOC_16011.	transcript	0	0	0	0	0.299559	0	0.21923	0.101737	0
Novel_015390	XLOC_16013.	transcript	0.013681	0.029706	0.114216	0.152198	0.111425	0.016939	0.217392	0.19371	0.03187
Novel_015391	XLOC_16013.	transcript	0	0.554849	0.845248	0.687804	0.742409	0.874077	0.974694	0.339736	0.177563
Novel_015392	XLOC_16013.	transcript	1.039133	0.34398	0.51986	0.428262	0.668749	0.491499	1.051162	0.797515	0.649536
Novel_015393	XLOC_16017.	transcript	0.120599	0.078573	0.259526	0.11822	0.032115	0.245566	0.285346	0.009047	0.070108
Novel_015394	XLOC_16017.	transcript	0.796584	0.494208	1.445327	0.577874	1.048228	1.381735	1.470921	1.020578	0.865529
Novel_015395	XLOC_16018.	transcript	0.60818	0.268381	0.334065	0.658871	0.388579	0.404439	0.509074	0.580868	0.51263
Novel_015396	XLOC_16018.	transcript	0	0	0	0	0	0	0	0	0
Novel_015397	XLOC_16018.	transcript	0.249342	0.177649	0.217403	0.088752	0.206876	0.217369	0.282901	0.285746	0.105153
Novel_015398	XLOC_16019.	transcript	0.357694	0.311172	0.159215	0.163828	0.316556	0.270633	0.385496	0.363256	0.241506
Novel_015399	XLOC_16021.	transcript	0	0	0.052636	0	0	0.06007	0.408664	0.028031	0.054318
Novel_015400	XLOC_16023.	transcript	0	0	0	0	0	0	0	0	0
Novel_015401	XLOC_16023.	transcript	0.225335	0	0	0	0	0	0.267768	0	0
Novel_015402	XLOC_16023.	transcript	0.793248	0.779953	1.048177	0.85849	0.651938	0.816055	0.15845	0.628763	0.76255
Novel_015403	XLOC_16023.	transcript	0	0	0	0	0	0	0.007362	0.329607	0
Novel_015404	XLOC_16023.	transcript	0	0	0	0	0	0	0.238077	0	0
Novel_015405	XLOC_16023.	transcript	0.443443	0.502626	0.514396	0.337908	0.396563	0.495422	0.463378	0.393871	0.31
Novel_015406	XLOC_16023.	transcript	0	0	0	0.251067	0	0	0	0	0
Novel_015407	XLOC_16024.	transcript	0.12431	0.105697	0.208022	0.075166	0.207864	0.163373	0.21362	0.100973	0.244018
Novel_015408	XLOC_16024.	transcript	0.057445	0	0.065637	0	0.146261	0.028946	0.142044	0.057749	0.16107
Novel_015409	XLOC_16024.	transcript	0.103611	0.166761	0.086113	0.324296	0.186669	0	0.111605	0.131657	0.220425
Novel_015410	XLOC_16026.	transcript	0.429654	0.373797	0.567446	0.686613	0.369771	0.31149	0.30044	0.454587	0.266783
Novel_015411	XLOC_16026.	transcript	0.430072	0.144195	0.355417	0.222317	0.068578	0.070054	0.263799	0.090314	0.183911
Novel_015412	XLOC_16026.	transcript	0.138701	0.188012	0.075576	0.220311	0.383196	0.281563	0	0.308524	0.029305
Novel_015413	XLOC_16027.	transcript	0	0.174538	0	0.248065	0.095581	0.046796	0.03	0.024192	0.004413
Novel_015414	XLOC_16027.	transcript	0.291276	0.001645	0.261257	0	0.172916	0.304556	0.297557	0.16751	0.124825
Novel_015415	XLOC_16028.	transcript	0.526386	0.375895	0.193444	0.468525	0.304344	0.375166	0.472662	0.504088	0.323524
Novel_015416	XLOC_16028.	transcript	1.206918	0.897457	0.890449	0.772913	0.822003	0.832097	0.889427	0.863347	0.616256

Novel_015417	XLOC_16028.	transcript	0.339388	0.150177	0.109354	0.138609	0.201329	0.168749	0.23201	0.257624	0.109439
Novel_015418	XLOC_16029.	transcript	0.13902	0	0.189815	0.049658	0.190752	0.07559	0.159997	0.008728	0
Novel_015419	XLOC_16029.	transcript	0.020858	0.244981	0	0.360052	0	0.228841	0.02522	0.320698	0.138267
Novel_015420	XLOC_16029.	transcript	0	0	0	0	0	0	0	0.1148	0
Novel_015421	XLOC_16030.	transcript	0.277753	0.410476	0.200992	0.219391	0.197965	0.135581	0.26614	0.23776	0.227184
Novel_015422	XLOC_16030.	transcript	0	0	0	0	0	0	0.001644	0	0
Novel_015423	XLOC_16030.	transcript	0.046057	0.046205	0	0.06742	0.006041	0	0	0.055898	0
Novel_015424	XLOC_16030.	transcript	0.128365	0.121575	0.158037	0.171176	0.047618	0	0.164092	0.174403	0.039998
Novel_015425	XLOC_16031.	transcript	0	0	0	0	0	0	0	0	0
Novel_015426	XLOC_16031.	transcript	0.442029	0.292113	0.623304	0.516653	0.386023	0.769208	0.714248	0.482738	0.470036
Novel_015427	XLOC_16032.	transcript	0.164556	0.344058	0.208424	0.262099	0.168854	0.406582	0.444572	0.205003	0.240677
Novel_015428	XLOC_16032.	transcript	0.123172	0.13207	0.236993	0.390584	0.371052	0.376254	0.339203	0.317698	0.202135
Novel_015429	XLOC_16033.	transcript	0	0	0	0.214907	2.00E-06	0.038197	0	0.159172	0.000167
Novel_015430	XLOC_16033.	transcript	0.071847	0.129153	0.296999	0	0.134288	0.137254	0.095225	0	0.103457
Novel_015431	XLOC_16033.	transcript	0.174565	0.176599	0.011659	0.193436	0.15926	0.17326	0.196654	0.136533	0.108678
Novel_015432	XLOC_16033.	transcript	0	0.049163	0.065385	0.029769	0.041065	0	0.037533	0.059929	0.066839
Novel_015433	XLOC_16038.	transcript	1.284071	1.427701	1.853652	1.972168	0.31758	2.788379	1.021916	1.159311	1.540058
Novel_015434	XLOC_16038.	transcript	0	0	0	0	1.948862	0	0	0	0
Novel_015435	XLOC_16042.	transcript	0.483485	1.297892	0.654713	1.050849	0.998144	1.142343	0.749469	1.505569	2.195857
Novel_015436	XLOC_16042.	transcript	0	0	0.400121	0	0	0	0	0	0
Novel_015437	XLOC_16046.	transcript	0.01548	0	0.273451	0.208289	0.188842	0.018554	0.151339	0.051635	0.018224
Novel_015438	XLOC_16046.	transcript	0	0	0	0	0.214708	0	0	0	0
Novel_015439	XLOC_16051.	transcript	0.022736	0.129516	0	0	0	0.027431	0.070728	0.009021	0
Novel_015440	XLOC_16054.	transcript	0.168818	0.396934	0.262008	0.382337	0.407274	0.25328	0.692242	0.517136	0.53282
Novel_015441	XLOC_16054.	transcript	0.232387	0	0	0	0	0	0.025498	0.009191	0
Novel_015442	XLOC_16056.	transcript	0	0	0	0.146326	0	0	0	0	0
Novel_015443	XLOC_16056.	transcript	0.476272	0.519015	0.515269	0.253911	0.556176	0.579331	0.173628	0.391858	0.356101
Novel_015444	XLOC_16056.	transcript	0	0	0	0	0	0	0.254979	0	0
Novel_015445	XLOC_16058.	transcript	0.478302	0.68638	0.468755	1.282969	1.054981	1.347619	0.942975	0.983389	1.219257
Novel_015446	XLOC_16058.	transcript	0.780948	0.105797	1.801708	1.64971	1.186834	2.065795	1.818028	1.926191	1.042405
Novel_015447	XLOC_16059.	transcript	0.784081	0.262646	0.513867	0.689906	0.512098	0.205067	0.999166	0.839882	0.51798
Novel_015448	XLOC_16059.	transcript	0.148065	0	0	0	0	0.216994	0	0.164827	0.09267
Novel_015449	XLOC_16059.	transcript	0.272603	0.205229	0.114159	0.240917	0.219615	0.462398	0.764364	0.390929	0.27095
Novel_015450	XLOC_16059.	transcript	0	0.357541	0	0	0	0	0	0	0
Novel_015451	XLOC_16059.	transcript	0	0.508877	0.830773	0.467049	0.517974	0.645664	0.935588	0	0
Novel_015452	XLOC_16059.	transcript	0	0.294882	0.566133	0.086997	0.005463	0.030565	0.327841	0.554836	0.036718
Novel_015453	XLOC_16059.	transcript	0	0.322061	0.315972	0.086417	0.154	0.142188	0.306632	0.30461	0.256528
Novel_015454	XLOC_16059.	transcript	0.330261	0.479974	0.248755	0.311725	0	0.425958	0.448795	0.382354	0.268038

Novel_015455	XLOC_16059.	transcript	0.021132	0	0	0.002212	0	0.001751	0	0	0.029066
Novel_015456	XLOC_16059.	transcript	0.195633	0.425438	0.378417	0.246427	0.464426	0.131263	0	0.256168	0.241081
Novel_015457	XLOC_16064.	transcript	0.027291	1.337618	0	0	0	0	0	0	0.008952
Novel_015458	XLOC_16064.	transcript	0	0	0	0	0	0	0	0.140782	0.03851
Novel_015459	XLOC_16064.	transcript	8.00E-04	0	0.011456	0	0	0	0	0	0
Novel_015460	XLOC_16064.	transcript	0	0	0	0	0	0	0.094253	0	0.008856
Novel_015461	XLOC_16064.	transcript	0.165381	0	0.000937	0.21855	0	0	0	0	0.165406
Novel_015462	XLOC_16064.	transcript	0.013113	0.046473	0.150859	0.013038	0.068293	0.126244	0	0.147734	0.03665
Novel_015463	XLOC_16064.	transcript	0	0.004532	0	0.213282	0.072307	0	0.069204	0	0
Novel_015464	XLOC_16064.	transcript	0.131809	0	0.109074	0	0	0.000218	0	0.158154	0
Novel_015465	XLOC_16064.	transcript	0.045916	0	0.039675	0.004428	0.120156	0.222211	0.042985	0.005557	0.012871
Novel_015466	XLOC_16064.	transcript	0.030108	0	0	0	0	0.03323	0	0.26905	0
Novel_015467	XLOC_16064.	transcript	0.017371	0	0.27076	0.025623	0.055827	0.000811	0.189935	0.049274	0
Novel_015468	XLOC_16064.	transcript	0	0	0.002691	0	0	0	0.394819	0	0
Novel_015469	XLOC_16065.	transcript	0	0.507868	1.586165	0.763382	0.963946	1.285737	1.636672	0.82267	0.921553
Novel_015470	XLOC_16065.	transcript	0	0	0	0	0	0	0	0	0
Novel_015471	XLOC_16065.	transcript	0.016392	0.19752	0.008303	0	0.008508	0	0.054259	0	0.007886
Novel_015472	XLOC_16065.	transcript	0.069857	0	0	0	0	0	0	0	0
Novel_015473	XLOC_16065.	transcript	0	0.166276	0	0	0	0	0	0	0
Novel_015474	XLOC_16065.	transcript	0.389204	1.447005	0.625888	0.482548	0.641789	0.687681	0.77882	0.689527	0.550512
Novel_015475	XLOC_16066.	transcript	0.215815	0.420384	0.372693	0.229997	0.373006	0.330709	0.14366	0.282241	0.361002
Novel_015476	XLOC_16066.	transcript	0.022439	0.044893	0.065448	0.040068	0.120285	0.014219	0.374858	0.042421	0.137511
Novel_015477	XLOC_16066.	transcript	1.552116	1.433068	2.71655	3.063987	3.169463	2.324652	2.708681	4.908247	1.343084
Novel_015478	XLOC_16066.	transcript	0.585245	0.107762	0.831212	0.70521	0.484428	1.01117	0.824715	1.988941	0.240509
Novel_015479	XLOC_16066.	transcript	0.085347	0	0.425971	0.328099	0.10107	0.150599	0.047737	0.554395	0.33374
Novel_015480	XLOC_16066.	transcript	0.634596	0.030602	2.043221	2.431165	0.701278	0.477109	0.688657	3.553049	0
Novel_015481	XLOC_16068.	transcript	0.335926	0.302738	0.263238	0.133704	0.13373	0.249646	0.480341	0.442317	0.270178
Novel_015482	XLOC_16068.	transcript	0.230431	0	0	0	0.170625	0.320029	0.135816	0.420828	0.117918
Novel_015483	XLOC_16068.	transcript	0	0	0	0	0.038111	0	0.332811	0	0
Novel_015484	XLOC_16068.	transcript	0	0.101591	0.206771	0	0.238389	0.152622	0.191504	0.155084	0.072767
Novel_015485	XLOC_16073.	transcript	0	0	0.296129	0	0.641361	0	0	0	0.192003
Novel_015486	XLOC_16073.	transcript	0.318626	0.378001	0.2322	0.30067	0.036989	0.4162	0.451621	0.033641	0.403866
Novel_015487	XLOC_16073.	transcript	0.14305	0.017587	0.038097	0.016879	0.095059	0.081049	0.208388	0.281043	0.106704
Novel_015488	XLOC_16073.	transcript	0	0	0	0	0	0	0	0	0
Novel_015489	XLOC_16076.	transcript	0.421393	0.029787	0	0.076096	0	0.070587	0	0	0.113475
Novel_015490	XLOC_16076.	transcript	0	0	0	0	0	0	0.001459	0	0
Novel_015491	XLOC_16076.	transcript	0	0	0	0	0	0.000456	0	0	0
Novel_015492	XLOC_16076.	transcript	0.088004	0.158795	0.04756	0.256174	0.276696	0.482948	0.606871	0.137136	0.389426

Novel_015493	XLOC_16076.	transcript	0	0	0.269549	0	0.363368	0	0.334724	0.235857	0
Novel_015494	XLOC_16077.	transcript	0.260048	0.6054	0.335352	0.362502	0.265161	0.394748	0.454015	0.368883	0.424071
Novel_015495	XLOC_16078.	transcript	0	0	0	0	0	0	0.035809	0	0
Novel_015496	XLOC_16078.	transcript	0.08917	0	0.058812	0.357558	0.065319	0.086768	0.434916	0.109143	0.018095
Novel_015497	XLOC_16078.	transcript	0.415088	0	0.445466	0	0.437671	0.602998	0.260959	0.567601	0.340696
Novel_015498	XLOC_16079.	transcript	0.164785	0.073661	0.179844	0.245608	0.329755	0.50715	0.133397	0.269424	0.1638
Novel_015499	XLOC_16079.	transcript	0	0	0	0.037482	0	0.136705	0.225778	0.086503	0.03434
Novel_015500	XLOC_16081.	transcript	0.757283	0	0.215478	0.270899	0.284122	0	0.464177	0.675805	0
Novel_015501	XLOC_16081.	transcript	0.058594	0.045151	0.079789	0.065013	0.190074	0	0.352345	0.154748	0
Novel_015502	XLOC_16081.	transcript	0.011767	0	0.013583	0	0.003259	0	0.017774	0.004344	0
Novel_015503	XLOC_16081.	transcript	0.006147	0	0.009024	0	0	0.238447	0.021143	0.00319	0.305456
Novel_015504	XLOC_16081.	transcript	0.468343	0.368589	0.542618	0.659589	0.447375	0.68325	0.615696	0.602602	0.441735
Novel_015505	XLOC_16082.	transcript	0.623856	0.657317	0.240571	0.412556	0.549058	0.357557	0.799966	0.73067	0.467244
Novel_015506	XLOC_16082.	transcript	0	0.525142	0.22024	0	0	0	0	0.062293	0
Novel_015507	XLOC_16082.	transcript	0.352183	0.527164	0.492569	0.469831	0.344554	0.339137	0.529716	0.431559	0.52047
Novel_015508	XLOC_16082.	transcript	0	0	0	0	0	0	0	0	0
Novel_015509	XLOC_16083.	transcript	0	0.059053	0.049711	0.050649	0	0.107897	0.141548	0	0.025025
Novel_015510	XLOC_16083.	transcript	0.087575	0.016155	0.000513	0.001462	0	0	0.19209	0	0.071667
Novel_015511	XLOC_16083.	transcript	0.212642	0.033784	0.183296	0.147965	0.122545	0.100288	0.31965	0.089845	0.135739
Novel_015512	XLOC_16083.	transcript	0.00415	0	0.00026	0.007674	0	0	0.049845	0.261502	0.002459
Novel_015513	XLOC_16083.	transcript	0	0	0	0	0	0	0.187367	0.198417	0
Novel_015514	XLOC_16083.	transcript	0	0.655823	0	0.072414	0.086603	0	0.063112	0	0
Novel_015515	XLOC_16084.	transcript	1.375718	0.199281	0.351275	0.507399	0.479917	0.552129	0.53165	0.638848	0.364639
Novel_015516	XLOC_16085.	transcript	0.031462	0.020737	1.021787	0.005132	0.021339	0.039831	0.016405	0	0.021319
Novel_015517	XLOC_16085.	transcript	0	0	0	0	0	0	0.139902	1.285882	0
Novel_015518	XLOC_16085.	transcript	0.922887	0.827278	0.007756	1.168364	1.140978	0.864412	1.150997	0.157984	1.17024
Novel_015519	XLOC_16085.	transcript	0.26533	0.293306	0.356263	0.445741	0.399081	0.42755	0.573736	0.14203	0.210193
Novel_015520	XLOC_16086.	transcript	0.020775	0.218023	0.214024	0.12612	0.210321	0.217872	0.307575	0.335422	0.266893
Novel_015521	XLOC_16086.	transcript	0.13167	0	0	0.019025	0	0	0	0.041654	0
Novel_015522	XLOC_16096.	transcript	0.067086	0.482053	0.088655	0.126753	0.102864	0.126071	0.098004	0.100334	0.170286
Novel_015523	XLOC_16096.	transcript	0	0	0	0	0	0	0.257499	0	0
Novel_015524	XLOC_16096.	transcript	0	0	0	0	0	0	0.162232	0	0
Novel_015525	XLOC_16096.	transcript	0	0	0	0	0	0	0.516223	0	0
Novel_015526	XLOC_16096.	transcript	0.043999	0.133945	0.013033	0.126348	0	0	0.315094	0.068132	0.131712
Novel_015527	XLOC_16096.	transcript	0.074877	0	0	0	0.10379	0.118494	0.047182	0	0
Novel_015528	XLOC_16096.	transcript	0.149407	0.147541	0.094771	0	0	0	0	0	0
Novel_015529	XLOC_16096.	transcript	0.274822	0.206035	0.105934	0.058983	0.126464	0.082209	0.080747	0.164913	0.069586
Novel_015530	XLOC_16096.	transcript	0.17866	0.48524	0.116728	0.079973	0.080398	0.115484	0.063937	0.113772	0.093691

Novel_015531	XLOC_1609€.	transcript	0.174431	0.324785	0.151713	0.04967	0.101324	0.127016	0.060338	0.094852	0.065932
Novel_015532	XLOC_1609€.	transcript	0.294091	0.222813	0.096877	0.062947	0.112043	0.064853	0.057806	0.128511	0.088829
Novel_015533	XLOC_1609€.	transcript	0	0	0.155852	0	0	0	0	0	0
Novel_015534	XLOC_1609€.	transcript	0	0	0.105129	0	0	0	0	0	0
Novel_015535	XLOC_1609€.	transcript	0.267321	0.24791	0.086977	0.053267	0.111911	0.036972	0.082729	0.082698	0.091223
Novel_015536	XLOC_1609€.	transcript	0.272954	0.218733	0.096649	0.03528	0.138705	0.081854	0.056888	0.163396	0.068562
Novel_015537	XLOC_1609€.	transcript	0.160986	0.296623	0.13723	0.715192	0.465022	1.191448	0.033989	1.142596	0.229197
Novel_015538	XLOC_1609€.	transcript	0.106582	0.401737	0.080162	0	0	0	0	0	0
Novel_015539	XLOC_1609€.	transcript	0	0	0	0	0	0	0	0	0
Novel_015540	XLOC_1609̄7.	transcript	0.492099	0.488878	0.482975	0.722889	0.492433	0.547307	0.549025	0.731939	0.112223
Novel_015541	XLOC_16097.	transcript	0	0.001142	0	0.021406	0	0	0.061504	0.022709	0
Novel_015542	XLOC_16097.	transcript	0	0	0	0	0.018404	0	0	0	1.409942
Novel_015543	XLOC_1609₈.	transcript	0	0.504738	0.277352	0	0.265164	0.177824	0.039378	0.366065	0
Novel_015544	XLOC_1609₈.	transcript	0.488484	0.137982	0	0.194786	0	0	0.743444	0	0.529357
Novel_015545	XLOC_1609₈.	transcript	0	0	0	0	0	0	0	0	0
Novel_015546	XLOC_1609₈.	transcript	0	0	0	0.620459	0	0	0	0.228228	1.190568
Novel_015547	XLOC_1609ɛ.	transcript	0.106738	0.152269	0.11751	0.107983	0.080575	0.079064	0.086566	0.1135	0.105831
Novel_015548	XLOC_1609ɛ.	transcript	0.214498	0.215353	0.071577	0.165242	0	0.023556	0	0.002144	0
Novel_015549	XLOC_1609ɛ.	transcript	0.645947	1.735541	0.379752	0.260581	0.351938	0.392646	0.44147	0.259441	0.192023
Novel_015550	XLOC_1609ɛ.	transcript	0.05858	0.20642	0	0.093235	0.113361	0	0.289773	0.224063	0.199561
Novel_015551	XLOC_1609ɛ.	transcript	0.440156	0.621934	0.572927	0.289062	0.400513	0.550699	0.534279	0.470626	0.431002
Novel_015552	XLOC_1610Ƽ.	transcript	0.064037	0.029279	0.265308	0.172272	0.369623	0.277962	0.194671	0.07794	0.288144
Novel_015553	XLOC_16107.	transcript	0.686148	0.180721	0.453334	0.542956	0.456511	0.595304	0.73652	0.690299	0.392488
Novel_015554	XLOC_16107.	transcript	0.091967	0.190041	0.152791	0.134042	0.424296	0.169524	0.019588	0.646889	0
Novel_015555	XLOC_16107.	transcript	0.962501	0.031911	0.273139	0.420228	0.21305	0.316283	0.718862	0.142394	0.485218
Novel_015556	XLOC_1610ɛ.	transcript	0.07715	0.586604	0.033768	0	0.088164	0.013536	0.079182	0.041359	0
Novel_015557	XLOC_16112.	transcript	0.043374	0.102043	0.189941	0.382544	0.406097	0.22539	0.286977	0.452708	0.038795
Novel_015558	XLOC_16112.	transcript	0	0	0	0.039776	0	0	0.026211	0	0
Novel_015559	XLOC_16112.	transcript	0.028374	0	0.056257	0.094981	0.128185	0.103995	0.069399	0.413822	0
Novel_015560	XLOC_16114.	transcript	0.604412	1.692757	0.643195	0.659902	0.757016	0.603499	1.071163	0.808045	1.017559
Novel_015561	XLOC_1611Ƽ.	transcript	0.898736	0.542417	1.088216	1.462026	1.201459	1.125891	1.193259	1.174741	0.727248
Novel_015562	XLOC_1611Ƽ.	transcript	0.546003	0.562508	0.001938	0.49634	0.42085	0.458068	0.573368	0.456928	0.41359
Novel_015563	XLOC_1611Ƽ.	transcript	0	0	0	0	0	0	0	0	0.264493
Novel_015564	XLOC_1611Ƽ.	transcript	0.000794	0.607725	0.376707	0.019205	0.045489	0	0.060981	0.066733	0
Novel_015565	XLOC_1611Ƽ.	transcript	0	0.048293	0.067797	0.095114	0.074166	0.294711	0.222039	0.171183	0.161992
Novel_015566	XLOC_1611Ƽ.	transcript	0	0.596734	0	0	0	1.37741	1.15846	0.851916	0
Novel_015567	XLOC_1611€.	transcript	0.300438	0.334721	0.15792	0.292188	0.339355	0.213891	0.338544	0.247498	0.101879
Novel_015568	XLOC_1611€.	transcript	0.106992	0.071495	0	0.109538	0.183596	0.091112	0.127988	0.093373	0.079634

Novel_015569	XLOC_16117.	transcript	0.495974	0.757598	0.420066	0.45773	0.568298	0.640269	1.021571	0.737521	0.461349
Novel_015570	XLOC_16118.	transcript	0.911739	0.445664	1.01295	1.025247	1.022343	0.61464	1.506314	1.299085	0.685704
Novel_015571	XLOC_16119.	transcript	0.227013	0	0.195041	0.178288	0.174059	0.026218	0.480564	0.017962	0.291655
Novel_015572	XLOC_16119.	transcript	0.302464	1.039696	0.209464	0.198037	0.262267	0.221623	0.41772	0.293974	0.310699
Novel_015573	XLOC_16119.	transcript	0	0	0	0	0	0	0.080139	0	0
Novel_015574	XLOC_16119.	transcript	0	0	0	0	0	0.007849	0	0	0
Novel_015575	XLOC_16120.	transcript	0.275628	0.402607	0.194909	0.112309	0.245361	0.149637	0.356116	0.250475	0.160656
Novel_015576	XLOC_16120.	transcript	0.306879	0.219046	0.099511	0.053672	0.179607	0.131524	0.231781	0.099036	0.101082
Novel_015577	XLOC_16124.	transcript	6.480905	7.645263	8.312912	11.10073	9.184093	10.30776	8.032614	13.09833	8.285347
Novel_015578	XLOC_16125.	transcript	0.20262	0.064098	0.453554	0	0.208644	0.293922	0.173128	0	0
Novel_015579	XLOC_16125.	transcript	0.044241	0.028411	1.138792	0.140956	0.06121	0.086457	0.041335	0.084675	0
Novel_015580	XLOC_16125.	transcript	0.157319	0.398699	0	0.495659	0.355728	0.294479	0.257849	0.366229	0.760109
Novel_015581	XLOC_16125.	transcript	0.289479	0.141401	0.019411	0.548203	0.318366	0.431204	0.305203	0.419206	0.234858
Novel_015582	XLOC_16125.	transcript	1.985361	0.804735	1.525703	1.329494	1.380203	1.344463	3.173396	1.926166	1.73762
Novel_015583	XLOC_16125.	transcript	0.36429	0.620584	0.885561	0.738578	1.077572	0.941359	0.423488	0.916576	0.824069
Novel_015584	XLOC_16125.	transcript	0.338933	0.636362	0.411474	0.912317	0.63832	0.390846	0.459155	0.802876	1.646764
Novel_015585	XLOC_16125.	transcript	0	0	0	0	0	0	0	0	0
Novel_015586	XLOC_16125.	transcript	0.02304	0.062408	0.037165	0.052293	0.041397	0.041874	0.509009	0.037238	0.07721
Novel_015587	XLOC_16126.	transcript	0	0.034134	0.178322	0.132404	0	0.056891	0.380047	0.112515	0.069575
Novel_015588	XLOC_16126.	transcript	0.217758	0.321145	0.013713	0	0.200276	0.25522	0.000935	0.186416	0.201974
Novel_015589	XLOC_16126.	transcript	0.514638	0	0	0.276963	0.770332	1.336675	1.574616	0.483332	0.006774
Novel_015590	XLOC_16126.	transcript	0.947538	1.492272	1.404766	1.327191	0.844997	0.04427	0.473233	0.9326	1.260993
Novel_015591	XLOC_16126.	transcript	0	0	0	0	0	0	0	1.521598	0
Novel_015592	XLOC_16127.	transcript	0.252902	0.05429	0.148605	0.317415	0.196169	0.174152	0.417702	0.267447	0.122108
Novel_015593	XLOC_16128.	transcript	0.208627	0.366483	0.285054	0.288521	0.320968	0.379811	0.436954	0.226621	0.230572
Novel_015594	XLOC_16128.	transcript	0.040455	0.20958	0.121487	0.132229	0.129336	0.138647	0.216346	0.216085	0.232697
Novel_015595	XLOC_16128.	transcript	0	0	0	0.012558	0	0	0	0	0
Novel_015596	XLOC_16128.	transcript	0.173779	0.112651	0.208723	0.110637	0.168879	0.105317	0.252445	0.150478	0.089562
Novel_015597	XLOC_16130.	transcript	0	0	0	0	0	0.008734	0	0	0
Novel_015598	XLOC_16130.	transcript	0.316275	0.526878	0.525958	0.655822	0.465979	0.548603	0.318932	0.505859	0.582104
Novel_015599	XLOC_16130.	transcript	0	0.020904	0	0.006905	0.004199	0.062963	0.268021	0.269307	0
Novel_015600	XLOC_16130.	transcript	0	0	0	0.145535	0	0	0.28581	0	0
Novel_015601	XLOC_16132.	transcript	0.095186	0.032362	0.088145	0	0	0.100594	0.272233	0.05967	0
Novel_015602	XLOC_16132.	transcript	0	0.185746	0	0	0.212204	0	0	8.40E-05	0.039021
Novel_015603	XLOC_16132.	transcript	0.126063	0.063002	0.1536	0.354481	0.076813	0.384348	0.672175	0.438212	0.305764
Novel_015604	XLOC_16132.	transcript	0.234629	0.496781	0.290733	0	0.406482	0	0	0	0
Novel_015605	XLOC_16132.	transcript	0.033153	0.354802	0.051597	0	0	0	0	0	0
Novel_015606	XLOC_16133.	transcript	0.022038	0.013702	0.441601	0.343554	0.476319	0	0.846748	0.534088	0

Novel_015607	XLOC_16133.	transcript	0.489193	0.646438	0	0.097362	0	0.406897	0	0	0.287316
Novel_015608	XLOC_16133.	transcript	0.143762	0.109584	0.002326	0.254965	0.032037	0	0.294686	0.114336	0.121757
Novel_015609	XLOC_16133.	transcript	0.159599	0.085431	0.090142	0.244846	0.150069	0.109848	0.159849	0.250107	0.134716
Novel_015610	XLOC_16133.	transcript	0	0.02511	0	0.002413	0	0	0	0	0
Novel_015611	XLOC_16134.	transcript	0.307158	3.684591	0.202349	0.187094	0.249134	0.262412	0.375837	0.142195	0.699449
Novel_015612	XLOC_16134.	transcript	0	0.539413	0.002009	0.013336	0.072363	0.122424	0.026271	0.002991	0.522614
Novel_015613	XLOC_16135.	transcript	0.201333	0.479369	0.105797	0.16699	0.108588	0.197731	0.388462	0.246929	0.205641
Novel_015614	XLOC_16135.	transcript	0.361595	0.074596	0.428895	0.299774	0.38248	0.049938	0.419932	0.527244	0.030431
Novel_015615	XLOC_16135.	transcript	0.091106	0.449835	0	0.104078	0	0.44885	0.02898	0.089452	0.450964
Novel_015616	XLOC_16135.	transcript	0.010205	0.07723	0.015318	0	0.168634	0.030338	0.278748	0	0
Novel_015617	XLOC_16135.	transcript	0.502606	0.322761	0.402853	0.334894	0.305732	0.420433	0.770458	0.363839	0.610345
Novel_015618	XLOC_16136.	transcript	0.177563	0.618256	0.319255	0.451519	0.547959	0.535954	0.642726	0.696463	0.332467
Novel_015619	XLOC_16136.	transcript	0.177054	0.02787	0.303131	0.157268	0.292445	0.186041	0.247561	0.056702	0.028317
Novel_015620	XLOC_16137.	transcript	0.623881	0.069712	0.309978	0.170852	0.196146	0.149367	0.691622	0.200779	0.186164
Novel_015621	XLOC_16137.	transcript	0.019364	0	0.234625	0.017263	0.134647	0.076284	0.143992	0.107689	0
Novel_015622	XLOC_16137.	transcript	0.150164	0.274794	0.297682	0.114212	0.242706	0.08667	0.306369	0.116525	0.181266
Novel_015623	XLOC_16138.	transcript	0	0.044303	0.125473	0	0	0	0.205509	0	0.196815
Novel_015624	XLOC_16138.	transcript	0.160979	0.007277	0	0.100902	0.059872	0	0	0.108143	0
Novel_015625	XLOC_16139.	transcript	0.148423	0.150461	0.144001	0.093821	0.093797	0.21296	0.195175	0.085011	0.071418
Novel_015626	XLOC_16139.	transcript	0	0	0.031755	0	0	0	0.030236	0.012219	0
Novel_015627	XLOC_16141.	transcript	0.350433	0.756306	0.987392	0.525904	0.44728	0.677814	0.693522	0.63576	0.700449
Novel_015628	XLOC_16142.	transcript	0.749412	1.074892	0.372035	0.833461	0.643106	0.320557	0.336709	0.497145	1.092205
Novel_015629	XLOC_16142.	transcript	0.163752	0.507086	0.996651	0.179841	0.022833	0.813063	1.278683	0.558251	0.048163
Novel_015630	XLOC_16144.	transcript	0.155524	0.029673	0.37731	0.166142	0.130417	0.152958	0.420776	0.356237	0.074293
Novel_015631	XLOC_16144.	transcript	0.158875	0.012068	0.077258	0.341527	0.195733	0.114662	0	0	0.305554
Novel_015632	XLOC_16148.	transcript	0.085143	0.67527	0.155909	0.106991	0.278899	0.312533	0.275386	0.414363	0.914069
Novel_015633	XLOC_16148.	transcript	0.053658	0.032668	0.187731	0.065262	0.323721	0.008093	0.087884	0.150396	0
Novel_015634	XLOC_16148.	transcript	0.353234	0.065751	0.007844	0.346136	0.089279	0.27509	0.39407	0.338766	0.228886
Novel_015635	XLOC_16148.	transcript	0	0	0.351387	0	0	0	0.153724	0.015342	0
Novel_015636	XLOC_16148.	transcript	1.135423	1.213244	2.425178	2.465665	1.788784	1.72338	1.863913	2.599794	2.246648
Novel_015637	XLOC_16148.	transcript	0.429423	0.851275	0.404827	0.315108	0.547644	0	0.379331	0.284671	0.402924
Novel_015638	XLOC_16149.	transcript	0.045324	0.029392	0.349303	0.035893	0.106565	0.072222	0.078536	0.048388	0.126476
Novel_015639	XLOC_16149.	transcript	0.534859	0.062901	0	0.030891	0.053626	0.085813	0.030107	0	0.020344
Novel_015640	XLOC_16149.	transcript	0.220069	0.684649	0.533551	0.912546	0.975697	0.848261	1.338184	0.704896	0.553534
Novel_015641	XLOC_16149.	transcript	0	0.038808	0.49698	0	0	0	0	0.366348	0.039716
Novel_015642	XLOC_16150.	transcript	0.930762	0.597481	1.124975	0.672845	0.986373	0.891432	1.508177	0.984071	0.692203
Novel_015643	XLOC_16152.	transcript	0.453874	0.837985	0.659169	0.598337	0.605407	0.460066	0.674744	0.596606	0.514079
Novel_015644	XLOC_16152.	transcript	0.209826	0.553517	0.342194	0.342067	0.471228	0.402951	0.626454	0.529295	0.372561

Novel_015645	XLOC_16155.	transcript	0.073593	0.08539	0.102622	0.127169	0.07527	0.095279	0.109797	0.183345	0.012642
Novel_015646	XLOC_16157.	transcript	0.120507	0.552822	0.22928	0.050499	0.31339	0.145395	0.101645	0.240933	0.084813
Novel_015647	XLOC_16157.	transcript	0	0.597589	0	0	0	0	0	0	0
Novel_015648	XLOC_16157.	transcript	0.056582	0.039795	0	0.015807	0	0.029086	0.073849	0.028377	0.088732
Novel_015649	XLOC_16158.	transcript	0	0.115272	0	0	0	0.049329	0.04149	0	0
Novel_015650	XLOC_16158.	transcript	0.750024	0	0	0	0	0	0	0	0
Novel_015651	XLOC_16158.	transcript	0	0.869464	1.229336	0	0	0	0.644411	0	1.101492
Novel_015652	XLOC_16158.	transcript	0.862686	0	0	1.753818	1.647492	1.435996	0	2.222451	0
Novel_015653	XLOC_16158.	transcript	0	0	0.051617	0	0	0	1.499468	0	0.019898
Novel_015654	XLOC_16159.	transcript	0.022536	1.2676	0.088352	0.027471	0.024216	0.087501	0.126318	0.039225	0.239276
Novel_015655	XLOC_16161.	transcript	0	0.195741	0.017954	0.286317	0	0.004919	0	0.078211	0
Novel_015656	XLOC_16161.	transcript	0	0.370577	1.036136	0.698882	0	1.174547	0	0.868764	0
[truncated: 553,870 more chars]
